# Reliable Target Prediction of Bioactive Molecules Based on Chemical Similarity Without Employing Statistical Methods

**DOI:** 10.3389/fphar.2019.00835

**Published:** 2019-07-26

**Authors:** Abed Forouzesh, Sadegh Samadi Foroushani, Fatemeh Forouzesh, Eskandar Zand

**Affiliations:** ^1^Iranian Research Institute of Plant Protection, Agricultural Research Education and Extension Organization (AREEO), Tehran, Iran; ^2^Department of Medicine, Tehran Medical Branch, Islamic Azad University, Tehran, Iran

**Keywords:** pharmacophore, structural similarity (SSIM), mechanism of action (MOA), minimum structure, target identification

## Abstract

The prediction of biological targets of bioactive molecules from machine-readable materials can be routinely performed by computational target prediction tools (CTPTs). However, the prediction of biological targets of bioactive molecules from non-digital materials (e.g., printed or handwritten documents) has not been possible due to the complex nature of bioactive molecules and impossibility of employing computations. Improving the target prediction accuracy is the most important challenge for computational target prediction. A minimum structure is identified for each group of neighbor molecules in the proposed method. Each group of neighbor molecules represents a distinct structural class of molecules with the same function in relation to the target. The minimum structure is employed as a query to search for molecules that perfectly satisfy the minimum structure of what is guessed crucial for the targeted activity. The proposed method is based on chemical similarity, but only molecules that perfectly satisfy the minimum structure are considered. Structurally related bioactive molecules found with the same minimum structure were considered as neighbor molecules of the query molecule. The known target of the neighbor molecule is used as a reference for predicting the target of the neighbor molecule with an unknown target. A lot of information is needed to identify the minimum structure, because it is necessary to know which part(s) of the bioactive molecule determines the precise target or targets responsible for the observed phenotype. Therefore, the predicted target based on the minimum structure without employing the statistical significance is considered as a reliable prediction. Since only molecules that perfectly (and not partly) satisfy the minimum structure are considered, the minimum structure can be used without similarity calculations in non-digital materials and with similarity calculations (perfect similarity) in machine-readable materials. Nine tools (PASS online, PPB, SEA, TargetHunter, PharmMapper, ChemProt, HitPick, SuperPred, and SPiDER), which can be used for computational target prediction, are compared with the proposed method for 550 target predictions. The proposed method, SEA, PPB, and PASS online, showed the best quality and quantity for the accurate predictions.

## Introduction

Bioactive molecules such as drugs and pesticides are produced in large numbers by many commercial and academic groups around the world (Orchard et al., 2011). Most bioactive molecules perform their actions by interacting with proteins or other macromolecules (Gfeller et al., 2013). However, for a significant fraction of bioactive molecules, targets remain unknown (Gfeller et al., 2013). Moreover, even for well-studied molecules, our knowledge of their targets is far from complete (Gfeller et al., 2013).

Target-identification and mechanism-of-action studies have important roles in bioactive molecule probe and drug and pesticide discovery (Schenone et al., 2013). Experimental and computational approaches are used to predict biological targets that interact with bioactive molecules. Experimental approaches are usually more costly and slower than computational approaches. Computational approaches make predictions based on models with several approximations (Schomburg and Rarey, 2014). The common drawbacks of these models are that the real predictability beyond training space cannot always be guaranteed (Cheng et al., 2011). Computational approaches can facilitate the study of biological targets of bioactive molecules and assist the discovery of on-target and off-target effects and understand the mechanism of action of bioactive molecules, thereby playing a crucial role in many scientific projects (Wang et al., 2013a). With the ever-increasing public availability of chemical structures, bioactivity data, and receptor structures (Bento et al., 2014), it is possible to construct reliable target prediction models (Wang et al., 2016) for CTPTs in machine-readable materials using chemical structure similarity searching (Keiser et al., 2009), data mining/machine learning (Nidhi et al., 2006), panel docking (Li et al., 2006), and bioactivity spectra-based algorithms (Cheng et al., 2011). The target of these CTPTs may be a protein, cell line, whole organism, or biological activity. However, bioactivity data have not been increased in all areas. For example, databases are rich in human targets and molecules that modulate these targets, but contain limited information when it comes to bacterial targets (Koutsoukas et al., 2011).

Generally, the available computational target prediction approaches fall into two major categories of target-based methods (also called structure-based or receptor-based) and ligand-based methods (Liu et al., 2014b). Ligand-based methods incorporate chemical structures to predict targets (Schenone et al., 2013). Hence, the chemical similarity criteria for bioactive molecules play key roles in ligand-based modeling (Wang et al., 2016). Target-based methods rely on three-dimensional (3D) receptor structures to predict receptor–bioactive molecule interactions (Haupt and Schroeder, 2011). While ligand-based methods are fast, target-based methods take considerably more computational resources for a docking run against hundreds, or even thousands, of targets while still not achieving reliable results (Koutsoukas et al., 2011).

Databases that can be used for the ligand-based target prediction have grown tremendously in size in the past (Koutsoukas et al., 2011) but are still far from perfect. The screening data in databases are less rigorous than those in peer-reviewed articles and contain many false positives (Baker, 2006). Deposited data are not curated, and hence, mistakes in structures, units, and other characteristics can and do occur (Baker, 2006). Worse, since structural similarity does not guarantee similar bioactivity, chemical structures without other data are not always useful (Baker, 2006). In addition, chemical structures in some journals are not provided as machine-readable descriptions, which can be deposited in databases.

The prediction of biological targets of bioactive molecules from machine-readable materials can be routinely performed by CTPTs. However, the prediction of biological targets of bioactive molecules from non-digital materials (e.g., printed or handwritten documents) has not been possible due to the complex nature of bioactive molecules and impossibility of employing computations. Despite many advances over the last decades, computational target prediction is still a very challenging task, as reflected by the low experimental target validation success rate (Liu et al., 2014b). The removal of false positives reduces the risk of yielding predictions that could incorrectly affect the downstream experiments for drug and pesticide discovery (Wang et al., 2013a).

An attempt to improve the target prediction success rate led to the creation of an innovative method based on chemical similarity. The proposed method is significantly different from the available methods based on chemical similarity. The proposed method not only refers to the application of chemical similarity without employing statistical methods to use in both formats of non-digital and machine-readable materials but also helps in improving the target prediction success rate. The proposed method has several distinctive features compared to the available computational target prediction methods. First, the prediction is performed without employing statistical methods. Second, it is highly accurate. Third, it can be used appropriately without similarity calculations in non-digital materials and with similarity calculations (perfect similarity) in machine-readable materials. Fourth, it enables us to gain a deeper understanding (more informative) of the relationship between the chemical structure and the target. Fifth, little knowledge regarding high-performance computing techniques or algorithms does not prevent its implementation.

## Methods

The proposed method steps for target prediction of bioactive molecules from chemical structures include i) query molecule, ii) similarity searching, iii) data collection, iv) minimum structure identification, and v) target prediction. The proposed method process of target prediction from chemical structures can be found in a hypothetical example with a simple expression in **Figure S1**.

It is well known that drugs and pesticides interact with multiple targets rather than with a single target (called the off-target effect) (Wang et al., 2016), and this fact can be beneficial (Solomon and Lee, 2009) or harmful (known as side effect or toxicity) (Henney, 2000). For instance, a recent study on a set of 802 drugs and drug interactions data assembled from seven different databases has shown that known drugs have on average six molecular targets on which they exhibit activity (Mestres et al., 2009). Also, because one target may have thousands of structurally diverse ligands, one unique model may not recover all features, and the prediction performance may not be satisfying (Wang et al., 2013a). Hence, a minimum structure is identified for each group of neighbor molecules in the proposed method. Each group of neighbor molecules represents a distinct structural class of molecules with the same function in relation to the target. The proposed method is not applicable in cases where no neighbor bioactive molecules for a target exist, since in these situations no training on the minimum structure-based information is possible.

### Query Molecule

A bioactive molecule such as a drug or a pesticide is used as a query molecule. The query molecule may have a known or an unknown target. The query molecule with a known target can be used as a reference to predict the target of neighbor molecules with an unknown target.

### Similarity Searching

The query molecule is used to search for structurally related bioactive molecules with similar chemical scaffold (molecules are compared to each other as a whole) and similar substructures (the specified substructures in molecules are compared to each other). However, it must be born in mind that structurally related analogs may bind in a slightly or considerably different manner (Poulos and Howard, 1987). The main shortcoming of most ligand-based methods is that it results in insufficient extrapolation in practice since only molecules are compared to each other as a whole (Koutsoukas et al., 2011). It should also be pointed out that our proposed method can predict unseen interactions between bioactive molecules and potential targets in other methods. KEGG (Kanehisa et al., 2017), PubChem (Kim et al., 2019), DrugBank (Wishart et al., 2018), and ChEMBL (Gaulton et al., 2017) databases provide common names and chemical structures for large numbers of bioactive molecules and, in some cases, their targets. All four databases support structure similarity searches.

### Data Collection

The target information is collected for all structurally related bioactive molecules. If the target of the query molecule is not known, information on the structure–activity relationship (the consistent correlation of structural features or groups with the biological activity of molecules in a given biological assay; Bleicher et al., 2003) and the pharmacophore (the spatial orientation of various functional groups or features necessary for activity at a biomolecular target; Bleicher et al., 2003) will be collected for all structurally related bioactive molecules. If the target of the query molecule is known, information on the structure–activity relationship and the pharmacophore will be collected only for structurally related bioactive molecules with the same target as the query molecule. Information on the target, the structure–activity relationship, and the pharmacophore are obtained from databases with the annotated target (e.g., KEGG, PubChem, DrugBank, and ChEMBL databases), scientific literature, and pharmacophoric descriptors (including hydrogen bonds as well as hydrophobic and electrostatic interaction sites; Wolber et al., 2006). The proposed method criteria for allocation of target–bioactive molecule interactions are not limited to cell-based and/or *in vivo* evidence, and binding data are not necessary to find out interactions.

### Minimum Structure Identification

A minimum structure does not represent a real molecule or a real association of functional groups, but is a part of a molecular structure that is necessary to ensure the target prediction of bioactive molecules. The minimum structure describes the presence or absence of chemical features in the molecule. It can be employed for distinguishing bioactive molecules based on their targets without similarity calculations in non-digital materials and with similarity calculations (perfect similarity) in machine-readable materials. Ligand-based approaches employ statistical methods to link structural features to biological activities (Huang et al., 2010), whereas the minimum structure involves specific structural features of a ligand required for interacting with its target without employing statistical methods. In the proposed method, unlike ligand-based approaches, it is necessary to know which part(s) of the bioactive molecule determines the precise target or targets responsible for the observed phenotype. Ligand-based approaches suffer from the problem of activity cliff, which is defined as pairs of structurally similar molecules with large differences in potency (Maggiora, 2006; Hu et al., 2013). The minimum structure is identified using data collection about structurally related bioactive molecules. The minimum structure consists of the core with or without the peripheral part. Here, the peripheral part is shown as the comment. The core plays an essential role in a bioactive molecule. Furthermore, modifying at some key position on the peripheral part can make a big change in the target or the activity of a bioactive molecule. Thus, the peripheral part can be useful for distinguishing bioactive molecules based on their targets. Since the minimum structure depends on structurally related bioactive molecules and information about them, when they become available, the minimum structure can be updated to further refine it.

### Target Prediction

The minimum structure is employed as a query to search for molecules that perfectly satisfy the minimum structure of what is guessed crucial for the targeted activity. The proposed method is based on chemical similarity, but only molecules that perfectly satisfy the minimum structure are considered. Structurally related bioactive molecules found with the same minimum structure were considered as neighbor molecules of the query molecule. The known target of the neighbor molecule is used as a reference for predicting the target of the neighbor molecule with an unknown target. A lot of information is needed to identify the minimum structure, because it is necessary to know which part(s) of the bioactive molecule determines the precise target or targets responsible for the observed phenotype. Therefore, the predicted target based on the minimum structure without employing the statistical significance is considered as a reliable prediction. Since only molecules which perfectly (and not partly) satisfy the minimum structure are considered, the minimum structure can be used without similarity calculations in non-digital materials and with similarity calculations (perfect similarity) in machine-readable materials. Without doubt, the assessment of molecule similarity for activity cliff definition and analysis is the most difficult task, for several reasons: first, the quantification of molecule similarity is strongly dependent on chosen molecular representations (descriptors), and second, there are no generally accepted similarity measures (Hu et al., 2013).

## Results

We evaluate our predictive performance by applying it to 550 drugs and pesticides with a known or an unknown target and comparing results to those from the CTPTs. These drugs and pesticides include fungicides and bactericides that may be used for medical, veterinary, and agricultural applications. Common name, CAS Registry Number, InChIKey, InChI, SMILES, primary target, chemical structure, and pharmacophore for 550 bioactive molecules of the present study have been shown in **Table S1**.

Phenotypic effects of bioactive molecules result from interactions of bioactive molecules with protein targets, i.e., primary targets for which they were designed for as well as off-targets (Schomburg and Rarey, 2014). It is well known that the majority of bioactive molecules have more than one target (Gfeller et al., 2013), and predictable targets may vary in the CTPTs. Therefore, in this study, only the prediction of primary targets of bioactive molecules was considered, because primary targets should be identified before other targets.

Here, the primary target information on 381 bioactive molecules was collected from scientific literature (**Table S2**) and databases (**Table S3**). The databases include KEGG (Kanehisa et al., 2017), PubChem (Kim et al., 2019), DrugBank (Wishart et al., 2018), and ChEMBL (Gaulton et al., 2017). Of these 381 bioactive molecules, scientific literature and databases contain the primary target information on 372 (97.6%) and 160 (42%) bioactive molecules, respectively. Here, a part of primary targets of bioactive molecules has been extracted from databases. However, the primary target information has not been collected from annotated targets in bioactivity assays and target predictions of databases, because in those parts, primary targets are not distinguished from other targets. Three hundred eighty-one bioactive molecules with known primary targets were used for the identification of eight minimum structures. Then, these eight minimum structures were employed to predict primary targets of 169 bioactive molecules with unknown primary targets. Finally, predictions made by the proposed method (**Tables 1**–**4**) were compared with those 381 bioactive molecules with known primary targets and 169 bioactive molecules with unknown primary targets in nine CTPTs (**Table S5**). The nine CTPTs include PASS online (Lagunin et al., 2000), PPB (Awale and Reymond, 2017), SEA (Keiser et al., 2007), TargetHunter (Wang et al., 2013a), PharmMapper (Wang et al., 2017), ChemProt (Kringelum et al., 2016), HitPick (Liu et al., 2013), SuperPred (Nickel et al., 2014), and SPiDER (Reker et al., 2014).

**Table 1:** The bioactive molecules with primary target of bacterial type IIA topoisomerase (DNA gyrase and topoisomerase IV) inhibition predicted by the minimum structure.

Minimum structure	Bioactive molecules
4(1*H*)-Pyridinone or 4-Pyridone 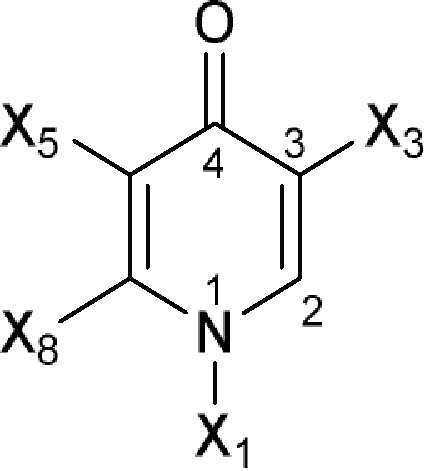	A 57132, A 57241, A 57274 (A 62917), A 60919 (PD 118106), A 61867 (BRN 4276829), A 62251 (A 57531; PD 137954), A 62255, A 62824, A 65326, ACH 702, Acorafloxacin (avarofloxacin), ADDNC (A 65485), Alalevonadifloxacin, Alatrofloxacin, Amifloxacin, Antofloxacin, AT 4929, Balofloxacin, BAY Y-3118 free base, Besifloxacin, Binfloxacin, BMY 40062, BMY 40397, BMY 42230, BMY 43261, BMY 43748, BMY 45243, BMY 45706, BRN 4913428 (PD 131199), Cadrofloxacin, Cetefloxacin, Chinfloxacin, CI 990 (PD 131112), Ciprofloxacin, Clinafloxacin, CP 100964, CP 104830, CP 105532 (PD 125275), CP 115953, CP 115955, CP 135803, CP 67015, CP 67804, CP 74667, CP 92121, CP 99433, Danofloxacin, DC 159a free base, Delafloxacin, Desfluorociprofloxacin (SQ 4004), Difloxacin, DJ 6783, DK 507k, DN 9494, Droxacin, DS 8587 free base, DU 6611, DU 6668, DV 7751a (DV 7751), DW 8186, DX 619, E 3604, E 3846, E 4441, E 4474, E 4480, E 4497, E 4501, E 4502, E 4527, E 4528, E 4534, E 4535, E 4695, Ecenofloxacin, EN 272, Enoxacin, Enrofloxacin, Esafloxacin, FA 103, Fandofloxacin, Finafloxacin, Fleroxacin, Flumequine, Garenoxacin, Gatifloxacin, Gemifloxacin, Grepafloxacin, Ibafloxacin, Irloxacin, K 12, KB 5246, KPI 10 free base (WQ 3810), Lascufloxacin, Levofloxacin, Levonadifloxacin, Levonadifloxacin arginine (WCK 771), Lomefloxacin, Marbofloxacin, Merafloxacin, Metioxate, MF 5101, MF 5103, MF 5112 free base, MF 5126, MF 5137, MF 5143, MF 5168, Miloxacin, Moxifloxacin, Nadifloxacin, Nalidixic acid, Nemonoxacin, Norfloxacin, NSFQ 104, NSFQ 105, Ofloxacin, Olamufloxacin, Orbifloxacin, Oxolinic acid, Ozenoxacin, Pazufloxacin, PD 111834, PD 112388, PD 114111, PD 115311, PD 116507, PD 117596, PD 118362, PD 119344, PD 129626, PD 131628, PD 135042 (AM 1147), PD 135144 (BMY 33315), PD 137156, PD 138312, PD 140248, PD 163449, PD 164488, Pefloxacin, Pipemidic acid, Piromidic acid, Piroxacin, Pradofloxacin, Premafloxacin, Prulifloxacin, PubChem CID-11531032, PubChem CID-11566845, PubChem CID-11610627, PubChem CID-11696318, PubChem CID-11844920, PubChem CID-11996799, PubChem CID-11996800, PubChem CID-11997263, PubChem CID-25022869, PubChem CID-44408626, PubChem CID-44408894, PubChem CID-44408896, PubChem CID-44408994, PubChem CID-44409001, PubChem CID-44409010, PubChem CID-53236573, PubChem CID-53236796, PubChem CID-122195336, PubChem CID-122195337, QA 241 free base, RO 13-5478, RO 14-9578, Rosoxacin, Rufloxacin, S 25932, S 31076, Sarafloxacin, Sitafloxacin, Sparfloxacin, T 14097, Temafloxacin, Tioxacin, Tosufloxacin, Trovafloxacin, Ulifloxacin, Vebufloxacin (benofloxacin), VG 6/1, WCK 1152 free base, WIN 57273, WIN 57294, WIN 58161, WQ 2743, WQ 2756, WQ 2908, WQ 2942, WQ 3330, Y 688, Zabofloxacin
Comments: X_1_ ≠ H (weak inhibitory activity) X_3_ = -COO; isothiazol-3(2*H*)-one (**a**) fused to the core X_8_ = aromatic ring Active ingredient ≠ non-fused aromatic ring or chain attached to an aromatic ring at X_8_ (here X_8_ means six-membered aromatic ring fused to the core at X_5_) in position 6; the core fused to more than two (if X_3_ = -COO) or three (if X_3_ = isothiazol-3(2*H*)-one fused to the core) aromatic rings **a** 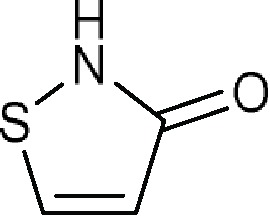	

**Table 2:** The bioactive molecules with primary target of small ribosomal subunit inhibition predicted by the minimum structure.

Minimum structure	Bioactive molecules
2,4(or 5)-Diaminocyclohexan-1-ol or 2,4(or 5)-Diaminocyclohexanol 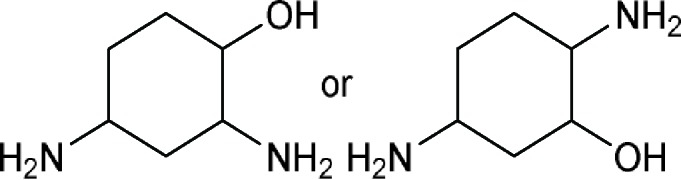	1-Epidactimicin, A 396I (SS 56D), Ambistrin (streptoduocin), Amikacin, Apramycin (nebramycin II), Aprosamine, Arbekacin, Astromicin (fortimicin A), Astromicin B (fortimicin B), Bekanamycin (kanamycin B; nebramycin V), Betamicin (gentamicin B), Butikacin, Butirosin A, Butirosin B, Dactimicin, Destomycin A, Destomycin B, Dibekacin, Dihydrostreptomycin, Etimicin, Fortimicin AE, Fortimicin AH, Fortimicin AI, Fortimicin AK, Fortimicin AL, Fortimicin AM, Fortimicin AN, Fortimicin AO, Fortimicin AP, Fortimicin AQ, Fortimicin AS, Fortimicin C, Fortimicin D, Fortimicin E (fortimicin KH), Fortimicin KE, Fortimicin KF, Fortimicin KG, Fortimicin KL_1_, Fortimicin KR, Framycetin (neomycin B), Geneticin (gentamicin G-418), Gentamicin A, Gentamicin A_1_, Gentamicin A_2_, Gentamicin A_3_, Gentamicin A_4_, Gentamicin B_1_, Gentamicin C_1_, Gentamicin C_1a_, Gentamicin C_2_, Gentamicin C_2a_, Gentamicin X_2_, Hybrimycin A_1_, Hybrimycin A_2_, Hybrimycin B_1_, Hybrimycin B_2_, Hybrimycin C_1_, Hybrimycin C_2_, Hybrimycin D, Hygromycin B (A 396II), Isepamicin, Istamycin A (sannamycin A), Istamycin A_0_ (sannamycin B), Istamycin A_1_, Istamycin A_2_, Istamycin A_3_, Istamycin AO, Istamycin AP (sannamycin E), Istamycin B, Istamycin B_0_, Istamycin B_1_, Istamycin B_3_, Istamycin C, Istamycin C_0_, Istamycin C_1_, Istamycin KL_1_, Istamycin X_0_ (sannamycin G), Istamycin Y_0_ (sannamycin H), Kanamycin (kanamycin A), Kanamycin C, Kanamycin D, Kanamycin X, Lividamine (nebramycin IX), Lividomycin, Lividomycin B (3’-deoxyparomomycin I), Mannosylparomomycin, Micronomicin (gentamicin C_2b_), Neamine (neomycin A; nebramycin X), Nebramine (nebramycin VIII), Nebramycin III, Nebramycin IV, Nebramycin V’, Nebramycin XI, Nebramycin XII, Nebramycin XIII, Neomycin C, Neomycin F (paromomycin II), Netilmicin, NK 1001, Oxyapramycin (nebramycin VII), Paromamine (neomycin D), Paromomycin (paromomycin I; neomycin E), Pentisomicin, Plazomicin, Propikacin, Pyrankacin, Ribostamycin, Saccharocin (KA 5685), Sannamycin C, Sannamycin F, Sannamycin J, Sannamycin K, Sannamycin KR, Sannamycin L, Seldomycin, Seldomycin 1 (seldomycin factor 1), Seldomycin 2 (seldomycin factor 2), Seldomycin 3 (seldomycin factor 3), Seldomycin 5 (seldomycin factor 5), Sisomicin, Sisomicin B, Sisomicin D, Spectinomycin, Sporaricin A, Sporaricin B, Sporaricin C, Sporaricin D, Sporaricin E, SS 56A, SS 56B, SS 56C, Streptomycin, Streptoniazid (streptonicozid), Tobramycin (nebramycin VI), Trospectomycin, Verdamicin, Verdamicin C_2_, Vertilmicin
Comment: The active ingredient comprises a ring attached to the core directly or indirectly	
(4*S*,12a*S*)-4-(Dimethylamino)-3,10,12,12a-tetrahydroxy-1,11-dioxo-1,4,4a,5,5a,6,11,12a-octahydrotetracene-2-carboxamide or (4a*RS*,5a*RS*)-Sancycline 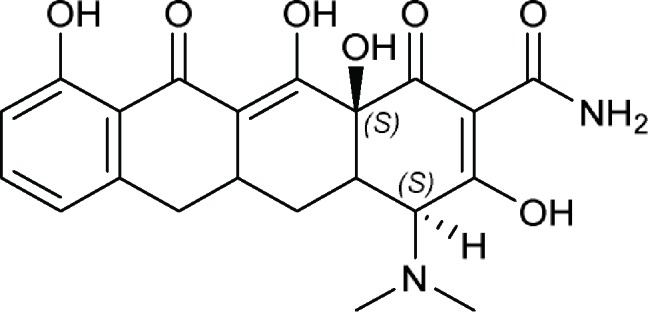	7-Iodosancycline, Amicycline, Apicycline, Bromotetracycline (bromtetracycline), Chlortetracycline (chlorotetracycline), Clomocycline, Demeclocycline, Demecycline, DMG-DMDOT (DMG-DM DOT), DMG-MINO, Doxycycline, Eravacycline, Etamocycline, Glycocycline, Guamecycline, Lymecycline, Meclocycline, Meglucycline, Metacycline (methacycline), Minocycline, Morphocycline, Nitrocycline, Omadacycline, Oxytetracycline, Pecocycline, Penimepicycline, Penimocycline, Pipacycline, Rolitetracycline, Sancycline, Sarecycline, Tetracycline, Tigecycline, TP 271

**Table 3:** The bioactive molecules with primary target of large ribosomal subunit inhibition predicted by the minimum structure.

Minimum structure	Bioactive molecules
4-Aminopyrimidin-2(1*H*)-one or Cytosine 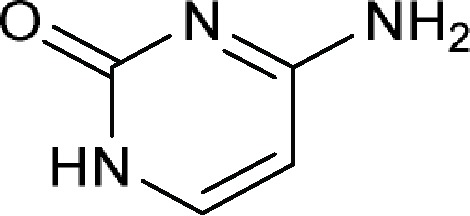	Amicetin (allomycin), Antelmycin (anthelmycin), Arginomycin, Bagougeramine A, Bagougeramine B, Bamicetin, Blasticidin H, Blasticidin S, Cytimidine, Cytomycin (saitomycin), Cytosamine, Cytosaminomycin A, Cytosaminomycin B, Cytosaminomycin C, Cytosaminomycin D, Gougerotin, Mildiomycin, Mildiomycin B, Mildiomycin C, Mildiomycin D, Mildiomycin M, Norplicacetin, Oxamicetin, Oxyplicacetin (cytosaminomycin E), Plicacetin (amicetin B), Rodaplutin, SCH 36605, SF 2457
Comment: The active ingredient contains at least one of the following components without a phosphorus group including 5-amino-5,6-dihydro-2*H*-pyran-2-yl (**a**), 5-aminotetrahydro-2*H*-pyran-2-yl (**b**) or 4-formamidobenzoyl (**c**)	
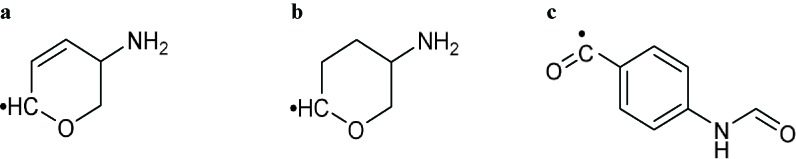	
2,6-Dioxo-4-piperidinyl or 3-Glutarimidyl 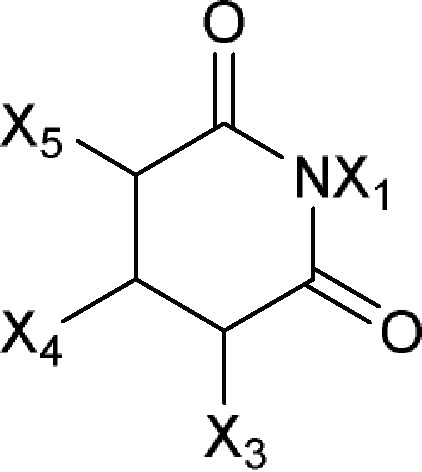	9-Methylstreptimidone (S 632A_2_), Acetoxycycloheximide (streptovitacin E-73), Actiketal, Actiphenol (actinophenol), Cycloheximide, Epiderstatin, Inactone, Isocycloheximide, Isomigrastatin, Lactimidomycin, Naramycin B, Neoisocycloheximide, S 632A_3_, Streptimidone (S 632A_1_), Streptovitacin A, Streptovitacin B, Streptovitacin C_2_
Comments: X_1_ = X_3_ = X_5_ = H X_4_ ≠ H; straight-chain longer than 12-membered chain; components without -OH or -CO Active ingredient = if there is a macrocyclic lactone ring, this ring will have at least one of the following components as part of the ring including (1*R*)-1-methyl-3-hydroxybutan-1-yl formate (**a**), (*R*)-1-methylbut-3-en-1-yl formate (**b**), (1*R*)-1-methyl-3-aminobutan-1-yl formate (**c**) or (1*R*)-1-methyl-3-oxobutan-1-yl formate (**d**)	
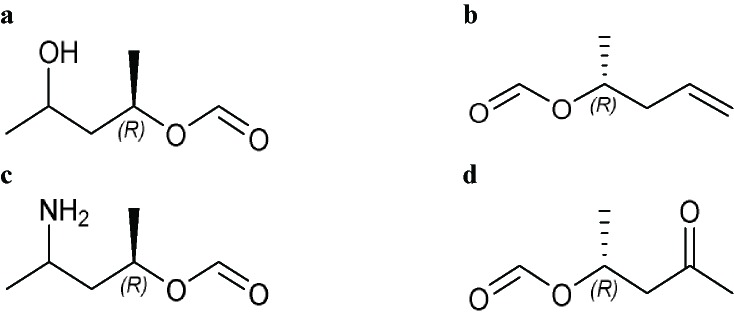	
(1*R*)-Propan-1-ol or (1*R*)-Propanol 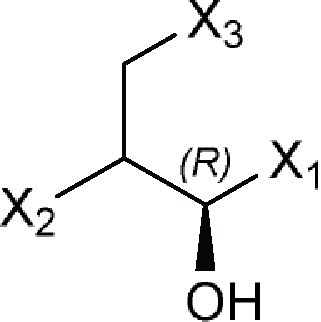	Azidamfenicol, Bromamphenicol (bromoamphenicol), Cetofenicol (cetophenicol), Chloramphenicol, Florfenicol, Monoiodoamphenicol, Racefenicol (racephenicol), Tevenel, Thiamphenicol, WIN 5094-2
Comments: (1*R*)-Propanol ≠ cyclic bonds X_1_ = aromatic ring X_2_ = -NCO; -NSO_2_ X_3_ = O; F; Cl; Br; I	

**Table 4:** The bioactive molecules with primary target of sterol 14α-demethylase inhibition predicted by the minimum structure.

Minimum structure	Bioactive molecules
1*H*-Imidazol-1-yl or Imidazol-1-yl 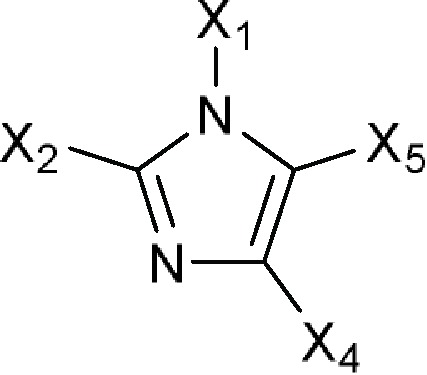 Comments: X_1_ = hydrophobic group (directly or indirectly) X_1_ ≠ methylbenzonitrile (**a**); methylphenylacetonitrile (**b**) X_2_ = X_4_ = X_5_ = H Active ingredient = if there is a fused aromatic ring, the molecule will have an aromatic ring with a halogen in position 2 or 4 Active ingredient ≠ carbanilate (**c**); an imidazol-1-yl at one end of the molecule and a carboxylate (-COO) at the other end; single (*S*)-enantiomer in the molecule with one atom stereocenter	1-Dodecylimidazole (N-dodecylimidazole), AFK 108, Aliconazole, Arasertaconazole, Azalanstat, BAY C-9263, BAY D-9603, Becliconazole, Bifonazole, Brolaconazole, Butoconazole, Cisconazole, Climbazole, Clotrimazole, Croconazole, Democonazole, Dichlorophenyl imidazoldioxolan (elubiol), Doconazole, Eberconazole, Econazole, Fenapanil, Fenticonazole, Flutrimazole, Imazalil (enilconazole), Isoconazole, Ketaminazole, Ketoconazole, Lanoconazole, Lombazole, Luliconazole, MH 0685, Miconazole, Neticonazole, OK 8705, OK 8801, Omoconazole, Orconazole, Oxiconazole, Oxpoconazole, Parconazole, Pefurazoate, PR 967-234, Prochloraz, R 31000, Sertaconazole, SM 4470, SSF 105, Sulconazole, Tioconazole, Triflumizole, UK 38667, Valconazole, Zinoconazole, Zoficonazole
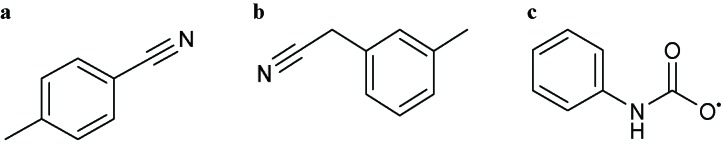	
1*H*-1,2,4-Triazol-1-yl or 1,2,4-Triazol-1-yl 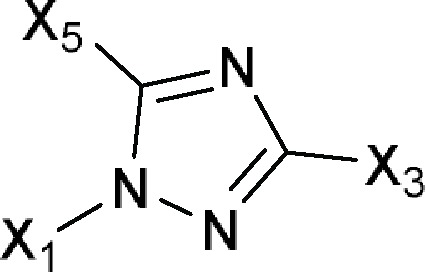 Comments: X_1_ = hydrophobic group (directly or indirectly) X_1_ ≠ methylbenzonitrile (**a**); methylphenylacetonitrile (**b**) X_3_ = X_5_ = H Active ingredient = if there is a fused aromatic ring, the molecule will have an aromatic ring with a halogen in position 2 or 4 Active ingredient ≠ carbanilate (**c**); single (*S*)-enantiomer in the molecule with one atom stereocenter	Albaconazole, Alteconazole, Azaconazole, BAS 110, BAS 111, BAS 45406F, Bitertanol, Bromuconazole, Cyproconazole, D 0870, Diclobutrazol, Difenoconazole, Diniconazole, Efinaconazole, Embeconazole, Epoxiconazole, Etaconazole, Fenbuconazole, Fluconazole, Fluotrimazole, Fluquinconazole, Flusilazole, Flutriafol, Fosfluconazole, Furconazole, Genaconazole (SCH 39304), Hexaconazole, ICI 153066, ICI 195739, Imibenconazole, Ipconazole, Ipfentrifluconazole, Isavuconazole, Itraconazole, LAB 158241F, LAB 170250F, Mefentrifluconazole, Metconazole, Myclobutanil, Penconazole, Posaconazole, PP 969, Pramiconazole, Propiconazole, Quinconazole, Ravuconazole, Saperconazole, SCH 42427, SCH 51048, SDZ 89-485, Simeconazole, SSF 109 (huanjunzuo), SSY 726, SYN 2506, SYN 2836, SYN 2869, SYN 2903, SYN 2921, T 8581, TAK 187, TAK 456, Tebuconazole, Terconazole (triaconazole), Tetraconazole, Triadimefon, Triadimenol, Triticonazole, UK 47265, UK 51486, Uniconazole, UR 9746, UR 9751, Vibunazole (BAY N-7133), Voriconazole, YH 1715R
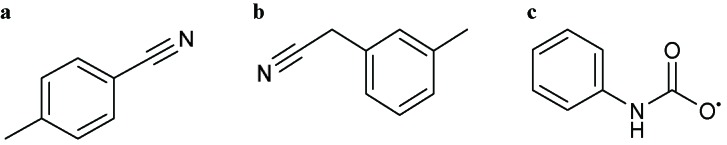	

The whole list of predicted targets with any statistical significance, including high confidence targets (e.g., low *p*-value, low target rank, high probability of being active, or low probability of being inactive) and low confidence targets (e.g., high *p*-value, high target rank, low probability of being active, or high probability of being inactive), was considered to obtain the maximum prediction potential in the CTPTs. Also, since the CTPTs are not able to distinguish between a primary target and other targets, the primary target found in the target list predicted by the CTPT was considered as an accurate prediction. Predictive results of the proposed method and the CTPTs are presented in eight groups.

### Validation

Results of bioactivity assays and target predictions from ChEMBL database were applied to 169 bioactive molecules with unknown primary targets to assess the prediction accuracy of our proposed method. ChEMBL_24 contains 1,828,820 distinct bioactive molecules, 12,091 targets, and 15,207,914 bioactivity entries from 69,861 publications. Each bioactivity data of ChEMBL database was applied only if it had an activity value of IC_50_, EC_50_, or *K* *_i_*. Activity values were classified as potent (value ≤ 1 μM), moderate (1 μM < value ≤ 10 μM), weak (10 μM < value ≤ 30 μM), and inactive (value > 30 μM). The following formula was used to convert the activity value from μg/mL to μM (or μM/L).

$$\begin{array}{l} \text{Activity value (}\mu \text{M)}=[\frac{1}{\text{molecular weight (g/mol)}}]\\ \;\times [\text{acivity value (}\mu \text{g/mL)}]\times 1000 \end{array}$$

Of 169 bioactive molecules, 111 bioactive molecules were not found in ChEMBL database. Also, the target data for 51 predictions were not found in ChEMBL database. Therefore, the target data were available only for seven predictions. Of these seven predictions, two predictions were reported as potent activity, three predictions were reported as moderate activity, and two predictions were reported as inactive. Activity values of PubChem CID-122195336 (DNA gyrase; IC_50_ = 0.17 µM; Itoh et al., 2015) and PubChem CID-122195337 (DNA gyrase; IC_50_ = 0.41 µM; Itoh et al., 2015) were reported as potent activity; binfloxacin (topoisomerase IV subunit A; IC_50_ = 10 µM; Gaulton et al., 2017), ecenofloxacin (topoisomerase IV subunit A; IC_50_ = 10 µM; Gaulton et al., 2017), and irloxacin (topoisomerase IV subunit A; IC_50_ = 10 µM; Gaulton et al., 2017) were reported as moderate activity; and PD 118362 (DNA gyrase; IC_50_ = 66.75 µM; Domagala et al., 1988) and PD 111834 (DNA gyrase; IC_50_ = 201 µM; Domagala et al., 1986) were reported as inactive.

Fifty-one molecules without target data and 111 molecules not found for predictions of the proposed method in ChEMBL database were used to search for molecules with a structural similarity of 85% and more in ChEMBL database. Results of bioactivity assays and target predictions of structural similarity molecules from ChEMBL database were applied to evaluate new target predictions of the proposed method in 51 molecules without target data and 111 molecules not found in ChEMBL database. Activity values of structural similarity molecules in ChEMBL database found with predicted targets of the proposed method were reported 56.45% as moderate activity (613 activities), 22% as potent activity (239 activities), 11.33% as weak activity (123 activities), and 10.22% as inactive (111 activities) (**Table S4**).

Recently, authors applied the proposed method to predict 22 targets and 15 mechanisms of action against more than 100 herbicides, two of which were validated *in vivo* and *in vitro* to be potent. The utility of the proposed method was documented by predicting and confirming the mechanism of action and target of tiafenacil and ipfencarbazone. The proposed method is well suited to provide insights into mechanisms of action and targets of bioactive molecules. For example, tiafenacil herbicide mechanism of action was predicted as protoporphyrinogen oxidase (PPG oxidase or protox) inhibition based on the proposed method (Forouzesh et al., 2015). Later, Park et al. (2018) proved tiafenacil mechanism of action as an inhibitor of PPG oxidase or protox with an IC_50_ of 22–28 nM through biochemical and physiological experiments. For example, the target and mechanism of action of ipfencarbazone herbicide were predicted as very long chain fatty acid (VLCFA) synthesis inhibition (K_3_ group) and mitosis inhibition (15 group), respectively, on the basis of the proposed method (Forouzesh et al., 2015). Ipfencarbazone inhibited the incorporation of [2-^14^C] malonyl-CoA into stearoyl-CoA (C18:0) and arachidoyl-CoA (C20:0) in rice and late watergrass microsomes at a low concentration (IC_50_ less than 1 µM), similar to cafenstrole, a known VLCFA synthesis inhibitor (Kasahara et al., 2019). Therefore, the target of ipfencarbazone was considered to be VLCFA synthesis inhibition (Kasahara et al., 2019).

### 4-Pyridone Group From DNA Gyrase and Topoisomerase IV Inhibitors

The primary target information on 135 bioactive molecules was collected from scientific literature (**Table S2**) and databases (**Table S3**). Of these 135 bioactive molecules, scientific literature and databases contain the primary target information on 134 (99.3%) and 48 (35.6%) bioactive molecules, respectively.

Based on the proposed method, the primary target can be identified for all 135 bioactive molecules with a known primary target. In addition, the primary target can be predicted for 59 bioactive molecules with an unknown primary target by the use of the proposed method. Generally, 4-pyridone group contains 194 bioactive molecules with a predictable primary target on the basis of the proposed method (**Table 1**).

PASS online, SEA, PPB, ChemProt, TargetHunter, PharmMapper, and SuperPred applied to 135 bioactive molecules with a known primary target led to make accurate predictions on 135 (100%), 131 (97%), 126 (93.3%), 112 (83%), 51 (37.8%), 36 (26.7%), and 1 (0.7%) bioactive molecules, respectively (**Table S5**). Also, PASS online, PPB, SEA, ChemProt, PharmMapper, TargetHunter, and SuperPred applied to 59 bioactive molecules with an unknown primary target led to make accurate predictions on 58 (98.3%), 57 (96.6%), 54 (91.5%), 47 (79.7%), 15 (25.4%), 12 (20.3%), and 0 (0%) bioactive molecules, respectively (**Table S5**). Since the primary target information on the 4-pyridone group is not available in HitPick and SPiDER, these tools are not able to predict the primary target of bioactive molecules of this group.

For example, six molecules of **Figure S2** were used to predict the DNA gyrase and topoisomerase IV inhibition by the proposed method and the CTPTs. Only molecule 3 in **Figure S2** is a DNA gyrase and topoisomerase IV inhibitor, based on the proposed method. Among the CTPTs, PASS online, PPB, ChemProt, and TargetHunter predicted molecule 3 as a DNA gyrase and topoisomerase IV inhibitor. Georgopapadakou et al. (1987) proved the target of molecule 3 as a DNA gyrase inhibitor by applying molecule 3 in *Escherichia coli* and *Staphylococcus aureus*.

PPB predicted molecule 6 as a DNA gyrase inhibitor, whereas molecule 6 is a highly selective HIV-1 integrase inhibitor with a potent antiviral activity against both B and non-B subtypes of HIV-1 (Matsuzaki et al., 2006).

PPB, ChemProt, and TargetHunter predicted molecule 2 as a DNA gyrase inhibitor, whereas molecule 2 (particularly as its potassium salt) is a plant growth regulator that is used as a chemical hybridization agent for commercial hybrid seed production (Kim et al., 2019).

PPB predicted molecule 5 as a DNA gyrase inhibitor, whereas preliminary clinical studies suggest that molecule 5 has marked diuretic and natriuretic activities (Van Dijk et al., 1976).

PASS online, SEA, and TargetHunter predicted molecule 1 as a DNA gyrase and topoisomerase IV inhibitor, whereas molecule 1 is nontoxic according to Brooks and Hurley (2010) and Balasubramanian et al. (2011).

The target data of molecules 1, 2, 4, 5, and 6 and molecules with a structural similarity of 85% and more to molecules 1, 2, 4, 5, and 6 for the DNA gyrase and topoisomerase IV inhibition were not found in ChEMBL database, but based on the obtained information, it is unlikely that the target of these molecules is DNA gyrase and topoisomerase IV inhibition. Employing the minimum structure in 4-pyridone group for identifying a bioactive molecule with the target of DNA gyrase and topoisomerase IV inhibition has been shown in **Figure S2**.

### 2,4(or 5)-Diaminocyclohexanol Group From Small Ribosomal Subunit Inhibitors

The primary target information on 64 bioactive molecules was collected from scientific literature **(Table S2**) and databases (**Table S3**). Of these 64 bioactive molecules, scientific literature and databases contain the primary target information on 63 (98.4%) and 26 (40.6%) bioactive molecules, respectively.

Based on the proposed method, the primary target can be identified for all 64 bioactive molecules with a known primary target. In addition, the primary target can be predicted for 74 bioactive molecules with an unknown primary target by the use of the proposed method. Generally, 2,4(or 5)-diaminocyclohexanol group contains 138 bioactive molecules with a predictable primary target on the basis of the proposed method (**Table 2**).

PASS online, PharmMapper, TargetHunter, and ChemProt applied to 64 bioactive molecules with a known primary target led to make accurate predictions on 64 (100%), 51 (79.7%), 41 (64.1%), and 16 (25%) bioactive molecules, respectively (**Table S5**). Also, PASS online, PharmMapper, TargetHunter, and ChemProt applied to 74 bioactive molecules with an unknown primary target led to make accurate predictions on 74 (100%), 49 (66.2%), 46 (62.2%), and 10 (13.5%) bioactive molecules, respectively (**Table S5**). Since the primary target information on the 2,4(or 5)-diaminocyclohexanol group is not available in PPB, SEA, HitPick, SPiDER, and SuperPred, these tools are not able to predict the primary target of bioactive molecules of this group.

For example, six molecules of **Figure S3** were used to predict the small ribosomal subunit inhibition by the proposed method and the CTPTs. Only molecule 2 in **Figure S3** is a small ribosomal subunit inhibitor, based on the proposed method. Among the CTPTs, PASS online and PharmMapper predicted molecule 2 as a small ribosomal subunit inhibitor. Molecule 2 is a 30S ribosomal subunit (small ribosomal subunit) inhibitor, as shown by experiments on reconstituted 70S ribosomes containing subunits from sensitive and from resistant ribosomes (Davies et al., 1965).

PASS online and PharmMapper predicted molecule 4 and molecule 6 as small ribosomal subunit inhibitors, whereas molecule 4 and molecule 6 are trehalase inhibitors (Xu et al., 2009).

PASS online and PharmMapper predicted molecule 5 as a small ribosomal subunit inhibitor, whereas molecule 5 is used as the substrate of 3-ketovalidoxylamine A C–N lyase (Zhang et al., 2007). One of the three key enzymes in production of valienamine is 3-ketovalidoxylamine A C–N lyase, which is a potent glucosidase inhibitor from validamycin A (Zhang et al., 2007).

PASS online predicted molecule 3 as a small ribosomal subunit inhibitor, whereas molecule 3 has roles as metabolite, antimutagen, and antioxidant (Ammar et al., 2007).

PASS online predicted molecule 1 as a small ribosomal subunit inhibitor, whereas molecule 1 binds to large ribosomal subunit and inhibits its peptidyl transferase activity (Kaminishi et al., 2015).

The target data of molecules 1, 3, 4, 5, and 6 and molecules with a structural similarity of 85% and more to molecules 1, 3, 4, 5, and 6 for the small ribosomal subunit inhibition were not found in ChEMBL database, but based on the obtained information, it is unlikely that the target of these molecules is small ribosomal subunit inhibition. Employing the minimum structure in 2,4(or 5)-diaminocyclohexanol group for identifying a bioactive molecule with the target of small ribosomal subunit inhibition has been shown in **Figure S3**.

### (4*aRS*,5*aRS*)-Sancycline Group From Small Ribosomal Subunit Inhibitors

The primary target information on 27 bioactive molecules was collected from scientific literature (**Table S2**) and databases (**Table S2**). Of these 27 bioactive molecules, scientific literature and databases contain the primary target information on 26 (96.3%) and 15 (55.6%) bioactive molecules, respectively.

Based on the proposed method, the primary target can be identified for all 27 bioactive molecules with a known primary target. In addition, the primary target can be predicted for seven bioactive molecules with an unknown primary target by the use of the proposed method. Generally, (4a*RS*,5a*RS*)-Sancycline group contains 34 bioactive molecules with a predictable primary target on the basis of the proposed method (**Table 2**).

PASS online and PharmMapper applied to 27 bioactive molecules with a known primary target led to make accurate predictions on 27 (100%) and 20 (74.1%) bioactive molecules, respectively (**Table S5**). Also, PASS online and PharmMapper applied to seven bioactive molecules with an unknown primary target led to make accurate predictions on 7 (100%) and 3 (42.9%) bioactive molecules, respectively (**Table S5**). Since the primary target information on the (4a*RS*,5a*RS*)-Sancycline group is not available in PPB, SEA, TargetHunter, ChemProt, HitPick, SPiDER, and SuperPred, these tools are not able to predict the primary target of bioactive molecules of this group.

For example, six molecules of **Figure S4** were used to predict the small ribosomal subunit inhibition by the proposed method and the CTPTs. Only molecule 1 in **Figure S4** is a small ribosomal subunit inhibitor, based on the proposed method. Among the CTPTs, PASS online and PharmMapper predicted molecule 1 as a small ribosomal subunit inhibitor. The target of molecule 1 was assessed using both cell-based and *in vitro* assays and confirmed target of 30S ribosomal subunit (Grossman et al., 2012).

PASS online predicted molecule 2 as a small ribosomal subunit inhibitor, whereas molecule 2 is a neuropeptide substance P binding inhibitor (Wong et al., 1993).

PASS online predicted molecule 3 and molecule 4 as small ribosomal subunit inhibitors, whereas the most prominent characteristic of chemically modified tetracycline analogs (e.g., molecule 3 and molecule 4) is their loss of antibacterial activity, accompanied by retention (or even enhancement) of their efficacy as inhibitors of mammal-derived matrix metalloproteinases (Liu et al., 2002).

PASS online predicted molecule 5 as a small ribosomal subunit inhibitor, whereas based on the studies carried out using blocked mutants, the first stable oxytetracycline intermediate is likely the fully aromatized tetracyclic compound called molecule 5, which then undergoes further processing and tailoring reactions of the fully formed tetracycline backbone (McCormick et al., 1965).

PASS online predicted molecule 6 as a small ribosomal subunit inhibitor, whereas molecule 6 is a neuropeptide Y receptor antagonist (Kodukula et al., 1995). Due to a lack of key pharmacophores of tetracycline antibiotics, molecule 6 showed no antimicrobial activity against *Staphylococcus aureus*, *Bacillus subtilis*, *Micrococcus luteus*, *Escherichia coli*, and *Saccharomyces cerevisiae* at a concentration of 100 µg/mL (Kodukula et al., 1995).

The target data of molecules 2, 3, 4, 5, and 6 and molecules with a structural similarity of 85% and more to molecules 2, 3, 4, 5, and 6 for the small ribosomal subunit inhibition were not found in ChEMBL database, but based on the obtained information, it is unlikely that the target of these molecules is small ribosomal subunit inhibition. Employing the minimum structure in (4a*RS*,5a*RS*)-Sancycline group for identifying a bioactive molecule with the target of small ribosomal subunit inhibition has been shown in **Figure S4**.

### Cytosine Group From Large Ribosomal Subunit Inhibitors

The primary target information on 25 bioactive molecules was collected from scientific literature (**Table S2**) and databases (**Table S3**). Of these 25 bioactive molecules, scientific literature and databases contain the primary target information on 25 (100%) and 1 (4%) bioactive molecules, respectively.

Based on the proposed method, the primary target can be identified for all 25 bioactive molecules with a known primary target. In addition, the primary target can be predicted for three bioactive molecules with an unknown primary target by the use of the proposed method. Generally, cytosine group contains 28 bioactive molecules with a predictable primary target on the basis of the proposed method (**Table 3**).

PASS online, PharmMapper, and ChemProt applied to 25 bioactive molecules with a known primary target led to make accurate predictions on 25 (100%), 14 (56%), and 0 (0%) bioactive molecules, respectively (**Table S5**). Also, PASS online, PharmMapper, and ChemProt applied to three bioactive molecules with an unknown primary target led to make accurate predictions on 3 (100%), 0 (0%), and 0 (0%) bioactive molecules, respectively (**Table S5**). Since the primary target information on the cytosine group is not available in PPB, SEA, TargetHunter, HitPick, SPiDER, and SuperPred, these tools are not able to predict the primary target of bioactive molecules of this group.

For example, six molecules of **Figure S5** were used to predict the large ribosomal subunit inhibition by the proposed method and the CTPTs. Only molecule 2 in **Figure S5** is a large ribosomal subunit inhibitor, based on the proposed method. Among the CTPTs, PASS online and PharmMapper predicted molecule 2 as a large ribosomal subunit inhibitor. The target of molecule 2 was investigated and results suggest that the molecule blocks the peptidyl transferase center (large ribosomal subunit) (Feduchi et al., 1985).

PASS online predicted molecule 1 as a large ribosomal subunit inhibitor, whereas molecule 1 is a pyrimidine analogue that has an activity against fungal species by interfering with purine and pyrimidine uptake and deaminating to 5-fluorouracil and then converting to 5-fluorodeoxyuridylic acid monophosphate, a noncompetitive inhibitor of thymidylate synthetase that interferes with DNA synthesis (McManus, 2015).

PharmMapper predicted molecule 3 as a large ribosomal subunit inhibitor, whereas it has been shown that molecule 3 works by activating transient receptor potential vanilloid (TRPV) channels in insect chordotonal organs (Nesterov et al., 2015).

PASS online predicted molecule 4 as a large ribosomal subunit inhibitor, whereas molecule 4 is a dihydropteroate synthase inhibitor (Wang et al., 2013b).

PASS online and ChemProt predicted molecule 6 as a large ribosomal subunit inhibitor, whereas molecule 6 is an RNA-directed DNA polymerase inhibitor (Tipples et al., 1996).

The target data of molecules 1, 3, 4, 5, and 6 and molecules with a structural similarity of 85% and more to molecules 1, 3, 4, 5, and 6 for the large ribosomal subunit inhibition were not found in ChEMBL database, but based on the obtained information, it is unlikely that the target of these molecules is large ribosomal subunit inhibition. Employing the minimum structure in cytosine group for identifying a bioactive molecule with the target of large ribosomal subunit inhibition has been shown in **Figure S5**.

### 3-Glutarimdyl Group From Large Ribosomal Subunit Inhibitors

The primary target information on 15 bioactive molecules was collected from scientific literature (**Table S2**). Based on the proposed method, the primary target can be identified for all 15 bioactive molecules with a known primary target. In addition, the primary target can be predicted for two bioactive molecules with an unknown primary target by the use of the proposed method. Generally, 3-glutarimidyl group contains 17 bioactive molecules with a predictable primary target on the basis of the proposed method (**Table 3**).

SEA, PASS online, PPB, TargetHunter, PharmMapper, ChemProt, and SPiDER applied to 15 bioactive molecules with a known primary target led to make accurate predictions on 13 (86.7%), 12 (80%), 10 (66.7%), 10 (66.7%), 10 (66.7%), 7 (46.7%), and 2 (13.3%) bioactive molecules, respectively (**Table S5**). Also, SEA, PASS online, PPB, TargetHunter, PharmMapper, ChemProt, and SPiDER applied to two bioactive molecules with an unknown primary target led to make accurate predictions on 2 (100%), 2 (100%), 2 (100%), 2 (100%), 0 (0%), 0 (0%), and 0 (0%) bioactive molecules, respectively (**Table S5**). Since the primary target information on the 3-glutarimidyl group is not available in HitPick and SuperPred, these tools are not able to predict the primary target of bioactive molecules of this group.

For example, six molecules of **Figure S6** were used to predict the large ribosomal subunit inhibition by the proposed method and the CTPTs. Only molecule 6 in **Figure S6** is a large ribosomal subunit inhibitor, based on the proposed method. Among the CTPTs, SEA, PASS online, PPB, TargetHunter, and ChemProt predicted molecule 6 as a large ribosomal subunit inhibitor. Rao and Grollman (1967) showed molecule 6 as an inhibitor of 60S ribosomal subunit (large ribosomal subunit) in resistant and sensitive strains of *Saccharomyces*.

PASS online predicted molecule 5 as a large ribosomal subunit inhibitor, whereas molecule 5 binds at a distinct binding site associated with a Cl^−^ ionopore at the GABA_A_ receptor, increasing the duration of time for which the Cl^−^ ionopore is open (Wishart et al., 2018).

PharmMapper predicted molecule 1 as a large ribosomal subunit inhibitor, whereas studies using purified mammalian DNA topoisomerase II suggest that molecule 1 and its structural analogs (e.g., mitonafide) represent a new class of intercalative topoisomerase II-active antitumor drugs (Hsiang et al., 1989).

PharmMapper predicted molecule 2 and molecule 4 as large ribosomal subunit inhibitors, whereas molecule 2 and the immunomodulatory drug, molecule 4, are therapeutically active in hematological malignancies (Lopez-Girona et al., 2012). The ubiquitously expressed E3 ligase protein cereblon has been identified as the primary teratogenic target of molecule 2 and molecule 4 (Lopez-Girona et al., 2012).

PharmMapper predicted molecule 3 as a large ribosomal subunit inhibitor, whereas molecule 3 is a tetralone-fused spiro-glutarimide derivative mainly known for sedative and hypnotic activity (Mondal et al., 2018).

The target data of molecules 1, 2, 3, 4, and 5 and molecules with a structural similarity of 85% and more to molecules 1, 2, 3, 4, and 5 for the large ribosomal subunit inhibition were not found in ChEMBL database, but based on the obtained information, it is unlikely that the target of these molecules is large ribosomal subunit inhibition. Employing the minimum structure in 3-glutarimidyl group for identifying a bioactive molecule with the target of large ribosomal subunit inhibition has been shown in **Figure S6**.

### (1*R*)-Propanol Group From Large Ribosomal Subunit Inhibitors

The primary target information on 10 bioactive molecules was collected from scientific literature (**Table S2**) and databases (**Table S3**). Of these 10 bioactive molecules, scientific literature and databases contain the primary target information on 9 (90%) and 6 (60%) bioactive molecules, respectively.

Based on the proposed method, the primary target can be identified for all 10 bioactive molecules with a known primary target (**Table 3**). PASS online, PharmMapper, and SEA applied to 10 bioactive molecules with a known primary target led to make accurate predictions on 10 (100%), 9 (90%), and 0 (0%) bioactive molecules, respectively (**Table S5**). Since the primary target information on the (1*R*)-propanol group is not available in PPB, ChemProt, TargetHunter, HitPick, SPiDER, and SuperPred, these tools are not able to predict the primary target of bioactive molecules of this group.

For example, six molecules of **Figure S7** were used to predict the large ribosomal subunit inhibition by the proposed method and the CTPTs. Only molecule 4 in **Figure S7** is a large ribosomal subunit inhibitor, based on the proposed method. Among the CTPTs, PASS online and PharmMapper predicted molecule 4 as a large ribosomal subunit inhibitor. Thiamphenicol and molecule 4 were shown to be as chloramphenicol in inhibiting peptidyl transferase activity specifically on 70S ribosomes (Cannon et al., 1990).

PASS online and SEA predicted molecule 1 as a large ribosomal subunit inhibitor, whereas molecule 1 (an amino acid) is an important intermediate in many syntheses (Mishra et al., 2003). Amino acids are extensively used in the synthesis of several products used in chemical, pharmaceutical, food, and health industries (Liu et al., 2014a).

PASS online predicted molecule 2 as a large ribosomal subunit inhibitor, whereas molecule 2 is an acetamide derivative of safingol. Safingol is a lysosphingolipid protein kinase C inhibitor that competitively interacts at the regulatory phorbol binding domain of protein kinase C (Sachs et al., 1995).

PASS online and PharmMapper predicted molecule 3 as a large ribosomal subunit inhibitor, whereas molecule 3 is a commonly used intense artificial sweetener, being approximately 200 times sweeter than sucrose (Sathyapalan et al., 2015). The interaction of sugars (or any sweet tasting ligand) with the T1R2+T1R3 sweet receptor (taste receptor type 1 members 2 and 3) sets into motion a biochemical chain of events that impacts on the activity of the TRPM5 cation channel (transient receptor potential cation channel subfamily M member 5), which is critical for further propagation of the taste signal (Zhang et al., 2003).

PASS online and PharmMapper predicted molecule 5 as a large ribosomal subunit inhibitor, whereas findings by Heneka et al. (2010) suggest that norepinephrine or the norepinephrine precursor molecule 5 acts through an adrenergic receptor in nearby microglia to stimulate their migration toward and phagocytic clearance of Aβ aggregates.

PASS online and PharmMapper predicted molecule 6 as a large ribosomal subunit inhibitor, whereas molecule 6 (*p*-chlorophenyl-α-glycerol ether) has been recommended as an antibacterial and antifungal agent of pharmaceutical interest (Hartley, 1947). The glycerol ethers (e.g., molecule 6) are non-irritant but possess the weakest antimicrobial action (Berger et al., 1953). The antimicrobial activity increased with the number of substituents on the benzene nucleus and, to a certain extent, was a function of the position of substitution (Berger et al., 1953).

The target data of molecules 1, 2, 3, 5, and 6 and molecules with a structural similarity of 85% and more to molecules 1, 2, 3, 5, and 6 for the large ribosomal subunit inhibition were not found in ChEMBL database, but based on the obtained information, it is unlikely that the target of these molecules is large ribosomal subunit inhibition. Employing the minimum structure in (1*R*)-propanol group for identifying a bioactive molecule with the target of large ribosomal subunit inhibition has been shown in **Figure S7**.

### Imidazol-1-yl Group From Sterol 14α-Demethylase Inhibitors

The primary target information on 42 bioactive molecules was collected from scientific literature (**Table S2**) and databases (**Table S3**). Of these 42 bioactive molecules, scientific literature and databases contain the primary target information on 38 (70.4%) and 30 (55.6%) bioactive molecules, respectively.

Based on the proposed method, the primary target can be identified for all 42 bioactive molecules with a known primary target. In addition, the primary target can be predicted for 12 bioactive molecules with an unknown primary target by the use of the proposed method. Generally, imidazol-1-yl group contains 54 bioactive molecules with a predictable primary target on the basis of the proposed method (**Table 4**).

PASS online, SEA, PPB, TargetHunter, ChemProt, SuperPred, and HitPick applied to 42 bioactive molecules with a known primary target led to make accurate predictions on 42 (100%), 37 (88.1%), 26 (62%), 22 (52.4%), 18 (42.9%), 17 (40.5%), and 16 (38.1%) bioactive molecules, respectively (**Table S5**). Also, PASS online, SEA, PPB, TargetHunter, HitPick, SuperPred, and ChemProt applied to 12 bioactive molecules with an unknown primary target led to make accurate predictions on 12 (100%), 11 (91.7%), 11 (91.7%), 8 (66.7%), 4 (33.3%), 3 (25%), and 1 (8.3%) bioactive molecules, respectively (**Table S5**). Since the primary target information on the imidazol-1-yl group is not available in PharmMapper and SPiDER, these tools are not able to predict the primary target of bioactive molecules of this group.

For example, six molecules of **Figure S8** were used to predict the sterol 14α-demethylase inhibition by the proposed method and the CTPTs. Only molecule 5 in **Figure S8** is a sterol 14α-demethylase inhibitor, based on the proposed method. Among the CTPTs, PASS online, SEA, and PPB predicted molecule 5 as a sterol 14α-demethylase inhibitor. Molecule 5 is a novel topical imidazole with a target similar to that of other azole antifungals, namely, lanosterol 14α-demethylase inhibition (Torres-Rodríguez et al., 1999).

PASS online, SEA, and PPB predicted molecule 1 as a sterol 14α-demethylase inhibitor, whereas molecule 1 is an imidazole derivative that is devoid of antifungal activity (Vanden Bossche, 1992). Molecule 1 is known to inhibit several cytochrome P-450 enzymes, including retinoic acid 4-hydroxylase and aromatase (Bruynseels et al., 1990; De Coster et al., 1992).

PASS online predicted molecule 2 as a sterol 14α-demethylase inhibitor, whereas molecule 2 is used in agriculture in seed treatment, only in mixture with other fungicides, to control a range of fungal diseases (European Food Safety Authority, 2011). Molecule 2 is a contact and non-systemic fungicide; target organisms are killed on contact with the fungicide, although the mode of action is not known (European Food Safety Authority, 2011).

PASS online, SEA, PPB, SuperPred, and HitPick predicted molecule 3 as a sterol 14α-demethylase inhibitor. Molecule 3 (an anticancer drug) is a racemate comprising equimolar amounts of *cis*-isomer and *trans*-isomer. Molecule 3 isomers represent a class of chemical compounds that are unique in that in mammalian cells only the *cis*-isomer inhibits tubulin polymerization, whereas the *trans*-isomer does not (Geuens et al., 1985). Structurally, molecule 3 is related to a β-tubulin inhibitor called diethofencarb due to the carbanilate (phenylcarbamate) moiety.

PASS online predicted molecule 4 as a sterol 14α-demethylase inhibitor, whereas it is now clear that farnesyl pyrophosphate synthase is a major site of action of the nitrogen-containing bisphosphonates (e.g., molecule 4) (Van Beek et al., 1999).

PASS online and SEA predicted molecule 6 as a sterol 14α-demethylase inhibitor, whereas it has been suggested that molecule 6 is a selective thromboxane synthase inhibitor (Iizuka et al., 1981). OKY (development code) is a group of selective thromboxane A_2_ biosynthesis inhibitors that have been collaboratively developed by the two companies (Kitamura et al., 1984). As a result of screening of OKY derivatives synthesized by the two companies, molecule 6 was selected (Kitamura et al., 1984).

The target data of molecules 1, 2, 3, 4, and 6 and molecules with a structural similarity of 85% and more to molecules 1, 2, 3, 4, and 6 for the sterol 14α-demethylase inhibition were not found in ChEMBL database, but based on the obtained information, it is unlikely that the target of these molecules is sterol 14α-demethylase inhibition. Employing the minimum structure in imidazol-1-yl group for identifying a bioactive molecule with the target of sterol 14α-demethylase inhibition has been shown in **Figure S8**.

### 1,2,4-Triazol-1yl Group From Sterol 14α-Demethylase Inhibitors

The primary target information on 63 bioactive molecules was collected from scientific literature (**Table S2**) and databases (**Table S3**). Of these 63 bioactive molecules, scientific literature and databases contain the primary target information on 62 (98.4%) and 34 (54%) bioactive molecules, respectively.

Based on the proposed method, the primary target can be identified for all 63 bioactive molecules with a known primary target. In addition, the primary target can be predicted for 12 bioactive molecules with an unknown primary target by the use of the proposed method. Generally, 1,2,4-triazol-1-yl group contains 75 bioactive molecules with a predictable primary target on the basis of the proposed method (**Table 4**).

PASS online, SEA, PPB, TargetHunter, ChemProt, SuperPred, and HitPick applied to 63 bioactive molecules with a known primary target led to make accurate predictions on 63 (100%), 62 (98.4%), 57 (90.5%), 47 (74.6%), 32 (50.8%), 24 (38.1%), and 13 (20.6%) bioactive molecules, respectively (**Table S5**). Also, PASS online, SEA, PPB, TargetHunter, ChemProt, HitPick, and SuperPred applied to 12 bioactive molecules with an unknown primary target led to make accurate predictions on 12 (100%), 12 (100%), 11 (91.7%), 7 (58.3%), 3 (25%), 2 (16.7%), and 2 (16.7%) bioactive molecules, respectively (**Table S5**). Since the primary target information on the 1,2,4-triazol-1-yl group is not available in PharmMapper and SPiDER, these tools are not able to predict the primary target of bioactive molecules of this group.

For example, six molecules of **Figure S9** were used to predict the sterol 14α-demethylase inhibition by the proposed method and the CTPTs. Only molecule 2 in **Figure S9** is a sterol 14α-demethylase inhibitor, based on the proposed method. Among the CTPTs, PASS online predicted molecule 2 as a sterol 14α-demethylase inhibitor. Molecule 2 is known to bind and inhibit fungal sterol 14α-demethylase, a cytochrome P450 enzyme found in plants, animals, fungi, and *Mycobacteria* (Kapteyn et al., 1994).

Molecule 3 in PASS online, SEA, PPB, and ChemProt, as well as molecule 5 in PASS online and SEA are predicted as sterol 14α-demethylase inhibitors, whereas molecule 3 and molecule 5 are non-steroidal molecules that potently inhibit aromatase *in vitro* and *in vivo* (Wouters et al., 1989a; Wouters et al., 1989b; Bhatnagar et al., 1990). Also, molecule 5, in contrast to the azole antifungal agents, is devoid of effects on the P450-dependent ergosterol and cholesterol synthesis (Vanden Bossche et al., 1990).

PASS online and SEA predicted molecule 4 as a sterol 14α-demethylase inhibitor, whereas molecule 4 is a potent, highly selective 5-HT_1B/1D_ receptor agonist with rapid onset of action for acute treatment of migraine (Street et al., 1995).

PASS online and PPB predicted molecule 6 as a sterol 14α-demethylase inhibitor, whereas molecule 6 is an organotin miticide whose target is to disrupt oxidative phosphorylation by inhibition of the mitochondrial ATP synthase (Linnett and Beechey, 1979).

The target data of molecules 1, 3, 4, 5, and 6 and molecules with a structural similarity of 85% and more to molecules 1, 3, 4, 5, and 6 for the sterol 14α-demethylase inhibition were not found in ChEMBL database, but based on the obtained information, it is unlikely that the target of these molecules is sterol 14α-demethylase inhibition. Employing the minimum structure in 1,2,4-triazol-1-yl group for identifying a bioactive molecule with the target of sterol 14α-demethylase inhibition has been shown in **Figure S9**.

## Discussion and Conclusion

Altogether, PASS online, SEA, PPB, ChemProt, TargetHunter, PharmMapper, SuperPred, HitPick, and SPiDER applied to 381 bioactive molecules with known primary targets led to make accurate predictions on 378 (99.2%), 243 (63.8%), 219 (57.5%), 185 (48.6%), 171 (44.9%), 140 (36.7%), 42 (11%), 29 (7.6%), and 2 (0.5%) bioactive molecules, respectively (**Table S5**). Also, PASS online, PPB, SEA, TargetHunter, PharmMapper, ChemProt, HitPick, SuperPred, and SPiDER applied to 169 bioactive molecules with unknown primary targets led to make accurate predictions on 168 (99.4%), 81 (47.9%), 79 (46.7%), 75 (44.4%), 67 (39.6%), 61 (36.1%), 6 (3.6%), 5 (3%), and 0 (0%) bioactive molecules, respectively (**Table S5**).

If groups that do not have the primary target information or the neighbor molecule in the CTPTs are removed from results of the CTPTs, then we will have predictions of the CTPTs as follows: a) Prediction results of PASS online, SEA, PPB, ChemProt, TargetHunter, PharmMapper, HitPick, SuperPred, and SPiDER on bioactive molecules with known primary targets led to make accurate predictions on 99.2%, 91.7%, 85.9%, 53.8%, 53.6%, 50.7%, 27.6%, 17.5%, and 13.3% bioactive molecules, respectively. b) Prediction results of PASS online, PPB, SEA, TargetHunter, PharmMapper, ChemProt, HitPick, SuperPred, and SPiDER on bioactive molecules with unknown primary targets led to make accurate predictions on 99.4%, 95.3%, 92.9%, 47.2%, 46.2%, 37.7%, 25%, 6%, and 0% bioactive molecules, respectively. Among the CTPTs, PASS online, SEA, and PPB had the most accurate predictions. Unlike the other six CTPTs, the accuracies of predictions on bioactive molecules with unknown primary targets in PPB, SEA, and PASS online were respectively 9.4%, 1.2%, and 0.2% higher than those on bioactive molecules with known primary targets. This means that PPB, SEA, and PASS online can predict unseen interactions between bioactive molecules and potential targets better than other six CTPTs.

It should be noted that results presented for the CTPTs include all targets of the prediction list with any statistical significance. The approach of each CTPT compared to other CTPTs to provide prediction results may vary greatly in terms of the number of targets on the prediction list and the use of statistical methods. For example, PASS online and SEA, unlike each other, contain a large number and a small number of targets on the prediction list, respectively. In the present study, results of accurate predictions of some CTPTs, which use a large number of targets on the prediction list, may change with consideration of the statistical significance. For example, in the present study, all of accurate predictions of SEA have high chances of the statistical significance, but approximately 41% of accurate predictions of PASS online are unlikely to exhibit the activity in experiment (if Pa < 0.5, the molecule is unlikely to exhibit the activity in experiment).

The CTPTs have made significant advances and improvements, but are still far from perfect. For example, molecule 3 and molecule 4 in **Figure S4** are considered as effective inhibitors of mammal-derived matrix metalloproteinases (Liu et al., 2002), whereas activity probabilities of molecule 3 and molecule 4 for matrix metalloproteinase are predicted to be 0.065 and 0.04, respectively (if Pa < 0.5, the molecule is unlikely to exhibit the activity in experiment) by PASS online. Also, according to Liu et al. (2002), molecule 3 and molecule 4 in **Figure S4** have lost their antibacterial activity, whereas activity probabilities of molecule 3 and molecule 4 for antibacterial activity are predicted to be 0.573 and 0.512, respectively (if 0.5 < Pa < 0.7, the molecule is likely to exhibit the activity in experiment) by PASS online. For example, molecule 2 in **Figure S4** is an inhibitor of neuropeptide substance P binding (Wong et al., 1993), whereas the activity probability of molecule 2 for neurokinin 1 and neurokinin is predicted to be 0.089 and 0.071, respectively, by PASS online. For example, molecule 1 in **Figure S7**, which is an important intermediate for the synthesis of several products used in the chemical industry (Mishra et al., 2003; Liu et al., 2014a), is predicted as a 60S ribosomal protein L19-A by SEA, whereas molecule 4 in **Figure S7**, which is a peptidyl transferase activity inhibitor (Cannon et al., 1990), is not predicted as a ribosomal activity inhibitor by SEA. For example, molecule 5 in **Figure S8** is a novel topical imidazole with a target similar to that of other azole antifungals, namely, lanosterol 14α-demethylase inhibition (Torres-Rodríguez et al., 1999), whereas molecule 5 is predicted for cytochrome P450 51 and lanosterol 14-alpha demethylase with rankings 85 and 134, respectively, by PPB. For example, molecule 3 in **Figure S8**, which is a tubulin inhibitor (Geuens et al., 1985), is predicted as a microtubule-associated protein tau with *p*-value >0.01 by PPB, whereas molecule 3 is predicted as a cytochrome P450 51 with *p*-value ≤0.01 by PPB. For example, molecule 3 in **Figure S8** is a racemate comprising equimolar amounts of *cis*-isomer and *trans*-isomer. Molecule 3 isomers represent a class of chemical compounds that are unique in that in mammalian cells only the *cis*-isomer inhibits tubulin polymerization, whereas the *trans*-isomer does not (Geuens et al., 1985). The CTPTs applied to the *cis*-isomer and the *trans*-isomer of molecule 3 showed the same predicted targets (also the relevant statistical significance) for these two isomers. The available information on stereochemistries from suppliers is usually ambiguous (configuration of the stereocenter is not resolved) and also molecules in the CTPTs are often processed as non-stereo; hence, large differences in the potency of stereoisomers of a molecule are not distinguished in the CTPTs.

The high accuracy of the proposed method in target prediction can be attributed to several causes. First, information on the target and the structure–activity relationship is mainly collected from peer-reviewed articles. A wealth of information on the activity of bioactive molecules exists in the literature, and access to this information can enable many types of analysis and making the right decision (Gaulton et al., 2012). Second, information obtained from various sources including scientific literature, databases, and pharmacophoric descriptors is checked for mistakes. Third, while information on the target, the chemical structure, the structure–activity relationship, and the pharmacophore is valid in its own right, the confidence in the observed outcome is significantly increased by a multi-validation method. Fourth, the nature of the proposed method is based on minimal mistakes because it is necessary to know which part(s) of the bioactive molecule determines the precise target or targets responsible for the observed phenotype in the proposed method.

The proposed method was applied to 550 target predictions, of which 169 are new predictions. Results of bioactivity assays and target predictions from ChEMBL database were available for seven predictions of the proposed method, which confirmed five targets predicted by the proposed method, two of which were validated *in vitro* to be potent with affinities less than 1 µM. Recently, the proposed method has been applied to predict mechanisms of action and targets in herbicides, two of which were confirmed by *in vivo* and *in vitro* experiments with an IC_50_ of less than 1 µM. If the reliable prediction of bioactive molecule targets from non-digital materials is not the most important achievement in this field, it is undoubtedly one of the most important achievements. The proposed method can be a prelude to future studies and facilitate solving complex scientific puzzles about the behavior of bioactive molecules.

## Data Availability

The raw data supporting the conclusions of this manuscript will be made available by the authors, without undue reservation, to any qualified researcher.

## Author Contributions

Conceptualization: AF and SF. Methodology: AF and SF. Validation: AF, SF, FF, and EZ. Investigation: SF, AF, FF, and EZ. Resources: SF, AF, FF and EZ. Data Curation: SF, AF, FF, and EZ. Writing - Original Draft Preparation: AF, FF, SF, and EZ. Writing - Review & Editing: AF, FF, SF, and EZ.

## Conflict of Interest Statement

The authors declare that the research was conducted in the absence of any commercial or financial relationships that could be construed as a potential conflict of interest.

## Abbreviation

CTPT, computational target prediction tool.
