# Supplementary material for: Reliable Target Prediction of Bioactive Molecules Based on Chemical Similarity Without Employing Statistical Methods
Source: Front Pharmacol. 2019 Jul 26;10:835. doi: 10.3389/fphar.2019.00835 (PMC6676798; doi:10.3389/fphar.2019.00835)
Supplement: Supplementary file 1 [file DataSheet_1.pdf]

Figure S1. The process of target prediction from chemical structures based on the proposed method in a hypothetical example.

Figure S2. The identification of a bioactive molecule with the target of DNA gyrase and topoisomerase IV inhibition by the minimum structure in 4-pyridone group.

Figure S3. The identification of a bioactive molecule with the target of small ribosomal subunit inhibition by the minimum structure in 2,4(or 5)-diaminocyclohexanol group.

Figure S4. The identification of a bioactive molecule with the target of small ribosomal subunit inhibition by the minimum structure in (4a*RS*,5a*RS*)-Sancycline group.

Figure S5. The identification of a bioactive molecule with the target of large ribosomal subunit inhibition by the minimum structure in cytosine group.

Figure S6. The identification of a bioactive molecule with the target of large ribosomal subunit inhibition by the minimum structure in 3-glutarimidyl group.

Figure S7. The identification of a bioactive molecule with the target of large ribosomal subunit inhibition by the minimum structure in (1*R*)-propanol group.

Figure S8. The identification of a bioactive molecule with the target of sterol 14α-demethylase inhibition by the minimum structure in imidazol-1-yl group.

Figure S9. The identification of a bioactive molecule with the target of sterol 14α-demethylase inhibition by the minimum structure in 1,2,4-triazol-1-yl group.

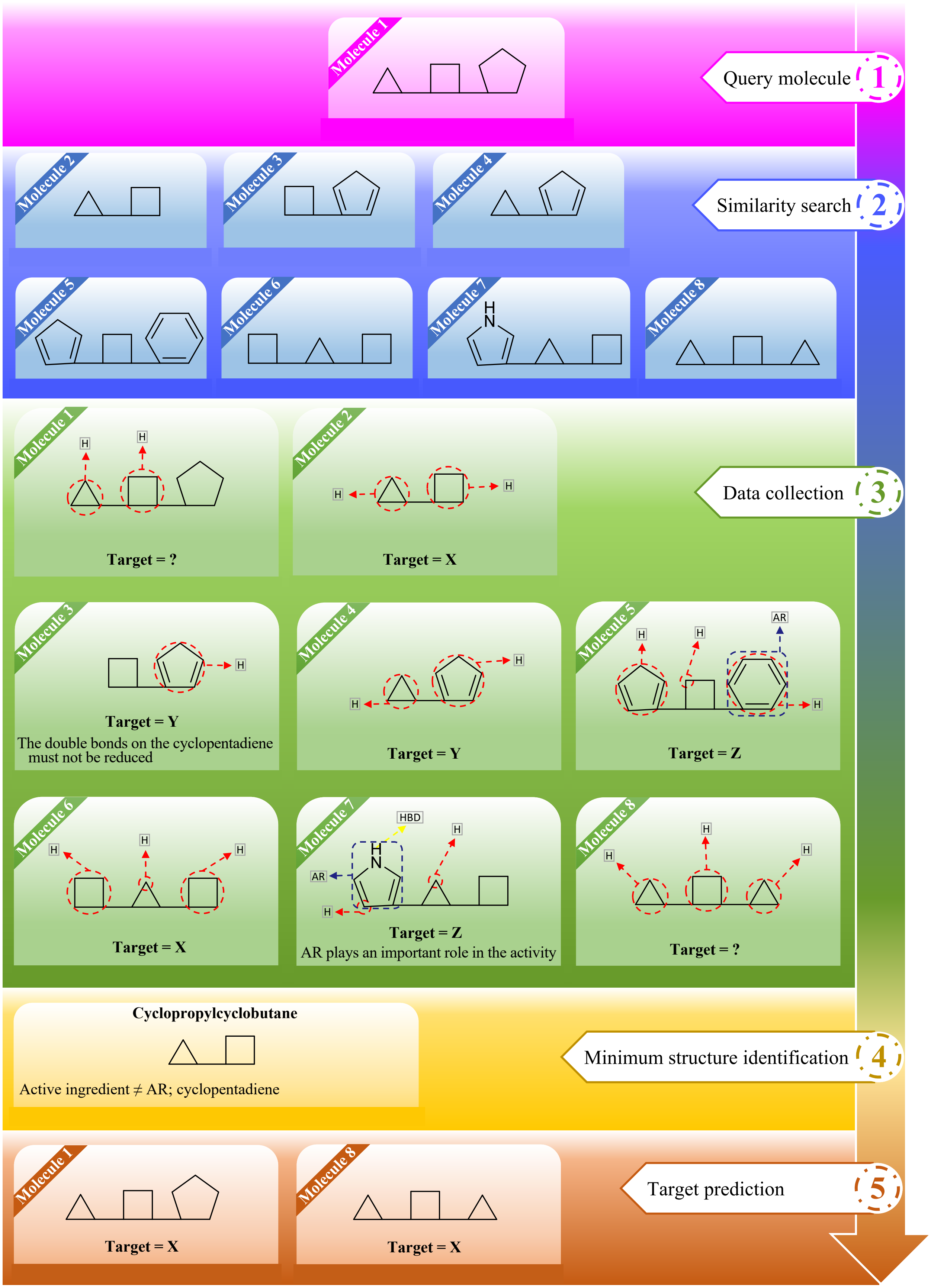

Molecule 1
Query molecule
1
Molecule 2
Molecule 3
Molecule 4
Similarity search
2
Molecule 5
Molecule 6
Molecule 7
Molecule 8
Target = ?
Target = X
Data collection
3
Target = Y
The double bonds on the cyclopentadiene must not be reduced
Target = Y
Target = Z
Target = X
Target = Z
AR plays an important role in the activity
Target = ?
Cyclopropylcyclobutane
Active ingredient ≠ AR; cyclopentadiene
Minimum structure identification
4
Target = X
Target = X
Target prediction
5

Figure S1. The process of target prediction from chemical structures based on the proposed method in a hypothetical example. Molecule 1 is used as the query molecule. Seven structurally related molecules to molecule 1 are found by similarity searching. Since the target of the query molecule (molecule 1) is unknown, information on the target, structure-activity relationship and pharmacophore are collected for the eight structurally related molecules. Then we consider the following assumptions:

Molecules 3 and 4 have the target of Y. Molecule 3 consists of a square attached to a pentagon with two double bonds, and molecule 4 consists of a triangle attached to a pentagon with two double bonds. Based on information on the structure-activity relationship of molecule 3, the double bonds on the pentagon with two double bonds (cyclopentadiene) must not be reduced. Therefore, the core of these two molecules is the common and important part, named the pentagon with two double bonds (cyclopentadiene). The core of molecules 3 and 4 in the presence of square and triangle is attributed to the pentagon with two double bonds. As a result, the pentagon with two double bonds has priority over the square and the triangle in expressing the target of the molecule.

Molecules 5 and 7 have the target of Z. Molecule 5 consists of a square attached to a six-membered aromatic ring (benzene) and a pentagon with two double bonds (cyclopentadiene), and molecule 7 consists of a triangle attached to a five-membered aromatic ring (pyrrole) and a square. Based on information on the structure-activity relationship of molecule 7, the aromatic ring plays an important role in the activity. Therefore, the core of these two molecules is the common and important part, named the aromatic ring (the aromatic ring in molecules 5 and 7 is characterized by LigandScout with AR). There is the pentagon with two double bonds in molecules 3, 4 and 5, but the core of molecule 5 is attributed to the aromatic ring. As a result, the aromatic ring has

priority over the pentagon with two double bonds (cyclopentadiene) in expressing the target of the molecule.

Molecules 2 and 6 have the target of X. Molecule 2 consists of a triangle attached to a square, and molecule 6 consists of a triangle attached to two squares. Because there is no molecule with the target of X having the square or the triangle alone, so the core of these two molecules is the common part, named the triangle attached to the square. The triangle attached to the square is also present in molecule 7, but the core of this molecule is attributed to the aromatic ring. As a result, the aromatic ring has priority over the triangle attached to the square in expressing the target of the molecule.

Molecules 1 and 8 have an unknown target. Molecule 1 consists of a square attached to a triangle and a pentagon, and molecule 8 consists of a square attached to two triangles. There is also a triangle attached to a square (or a square attached to a triangle) in molecules 2, 6 and 7. In addition, the results showed that the aromatic ring and possibly the pentagon with two double bonds (cyclopentadiene) have priority over the triangle attached to the square in expressing the target of the molecule, but molecules 1 and 8 don’t contain any of the aromatic ring or the pentagon with two double bonds. Therefore, the core of molecules 1 and 8 consists of the triangle attached to the square (cyclopropylcyclobutane) and the peripheral part does not consist the aromatic ring and the pentagon with two double bonds (cyclopentadiene). As a result, the target of molecules 1 and 8 is identical to the target of their neighbor molecules, named molecules 2 and 6, and the target of the two molecules is predicted as X.

Abbreviations: AR, aromatic ring; H, hydrophobic interaction; HBD, hydrogen bond donor.

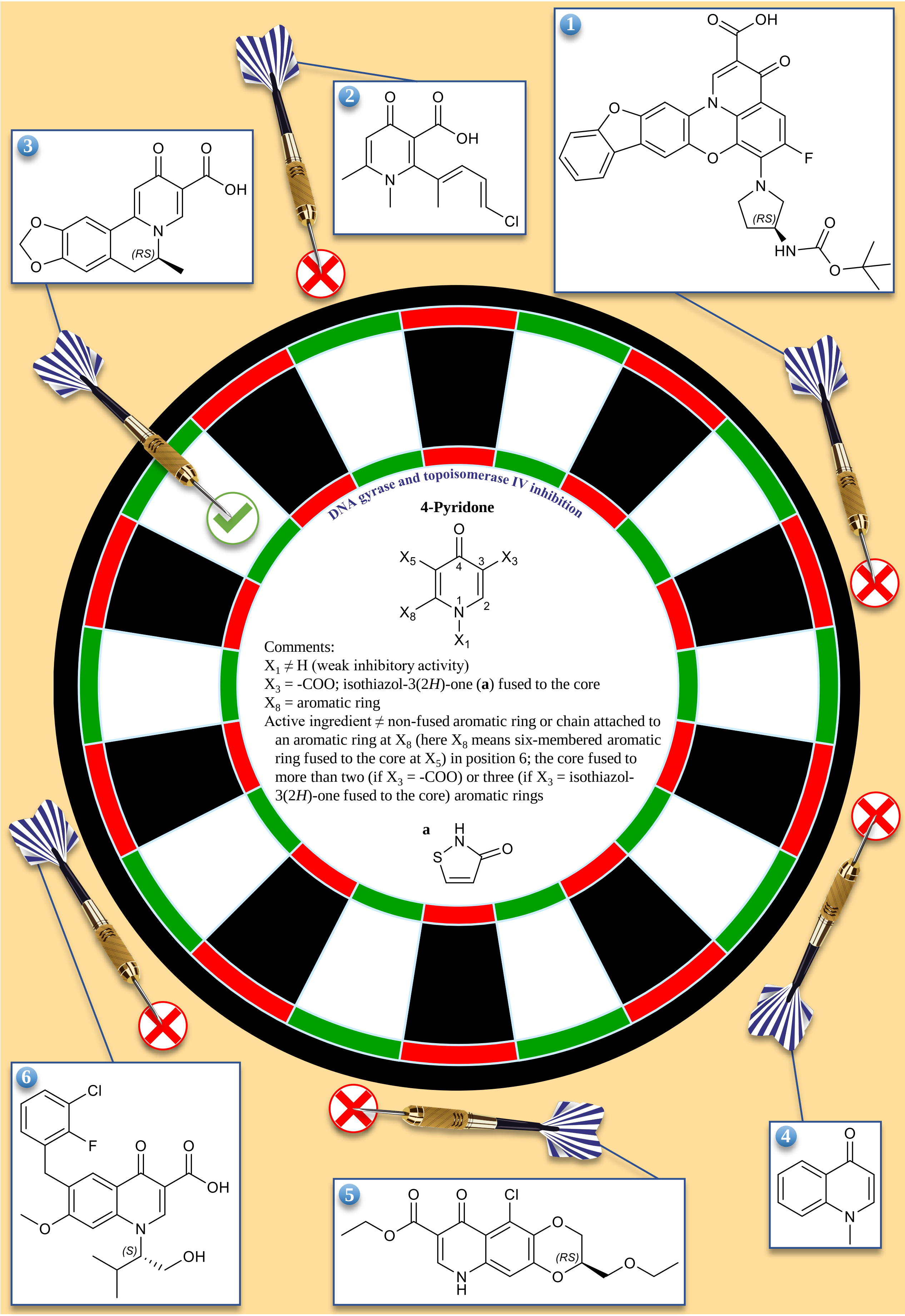
DNA gyrase and topoisomerase IV inhibition
4-Pyridone
Comments:
X1 ≠ H (weak inhibitory activity)
X3 = -COO; isothiazol-3(2H)-one (a) fused to the core
X8 = aromatic ring
Active ingredient ≠ non-fused aromatic ring or chain attached to an aromatic ring at X8 (here X8 means six-membered aromatic ring fused to the core at X5) in position 6; the core fused to more than two (if X3 = -COO) or three (if X3 = isothiazol-3(2H)-one fused to the core) aromatic rings
a
1
2
3
4
5
6

Figure S2. The identification of a bioactive molecule with the target of DNA gyrase and topoisomerase IV inhibition by the minimum structure in 4-pyridone group. Molecule 1 contains five aromatic rings fused to the core (4-pyridone), molecule 2 contains one methyl at X_8, molecule 4 doesn't contain -COO or isothiazol-3(2*H*)-one fused to the core at X_3, molecule 5 contains hydrogen at X_1 and molecule 6 contains one methyl attached to the aromatic ring (phenyl) at X_8 (here X_8 means six-membered aromatic ring fused to the core at X_5) in position 6. Therefore, the target of molecules 1, 2, 4, 5 and 6 is not DNA gyrase and topoisomerase IV inhibition.

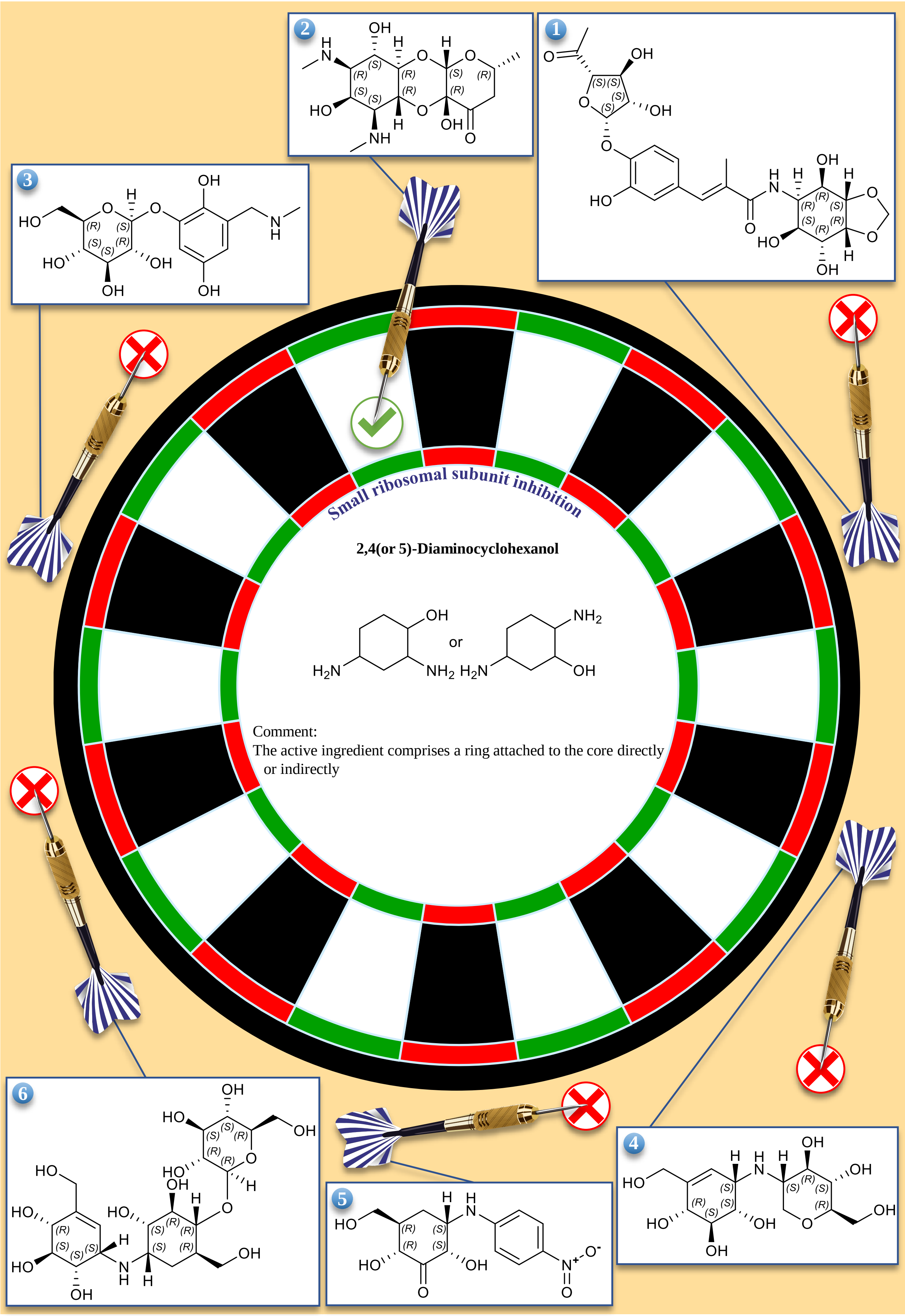
Small ribosomal subunit inhibition
2,4(or 5)-Diaminocyclohexanol
or
Comment:
The active ingredient comprises a ring attached to the core directly or indirectly
1
2
3
4
5
6

Figure S3. The identification of a bioactive molecule with the target of small ribosomal subunit inhibition by the minimum structure in 2,4(or 5)-diaminocyclohexanol group. Molecules 1, 3, 4, 5 and 6 don't contain 2,4(or 5)-diaminocyclohexanol (the core). Therefore, the target of molecules 1, 3, 4, 5 and 6 is not small ribosomal subunit inhibition.

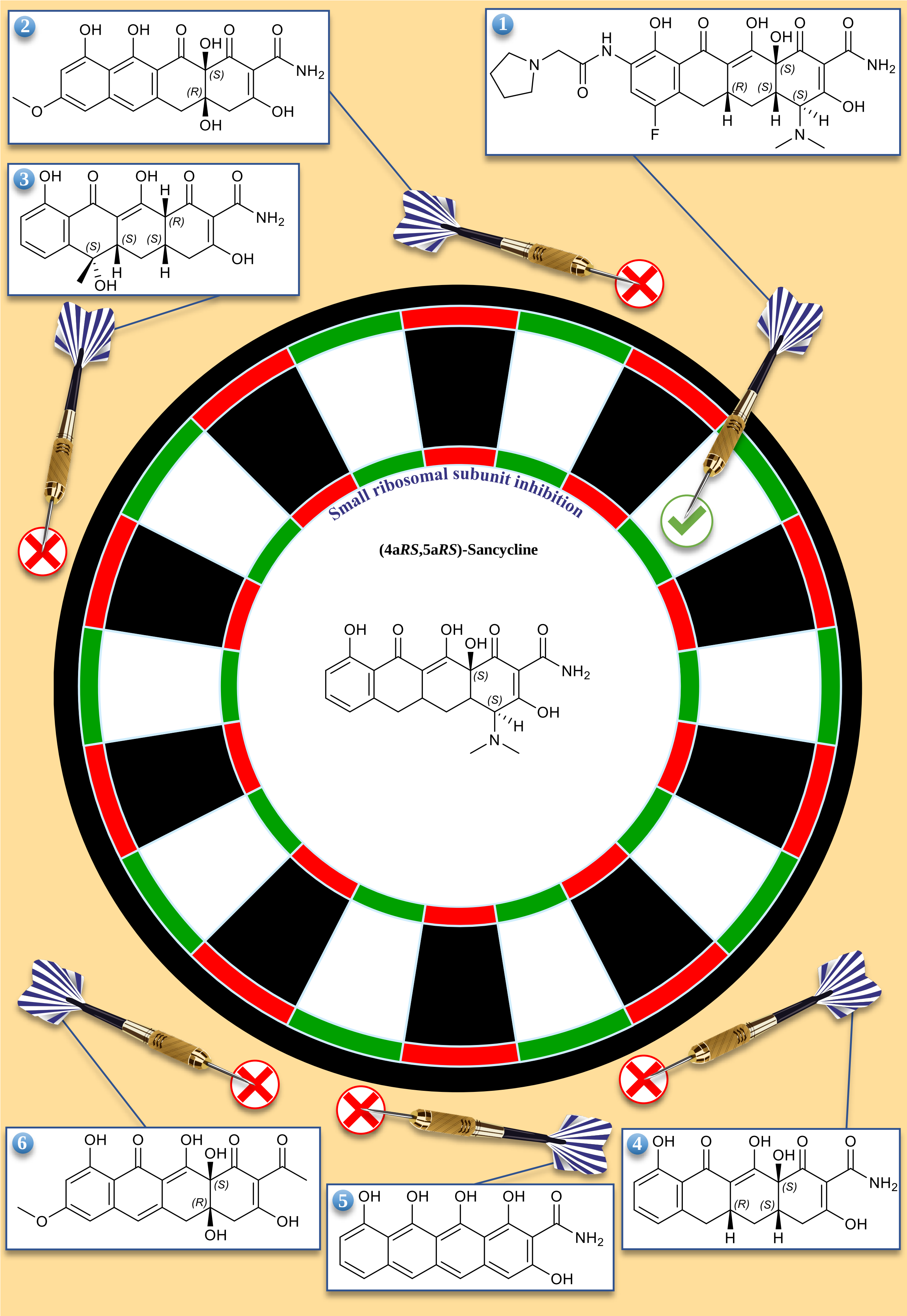
Small ribosomal subunit inhibition
(4aRS,5aRS)-Sancycline
1
2
3
4
5
6

Figure S4. The identification of a bioactive molecule with the target of small ribosomal subunit inhibition by the minimum structure in (4a*RS*,5a*RS*)-Sancycline group. Molecules 2, 3, 4, 5 and 6 don't contain (4a*RS*,5a*RS*)-Sancycline (the core). Therefore, the target of molecules 2, 3, 4, 5 and 6 is not small ribosomal subunit inhibition.

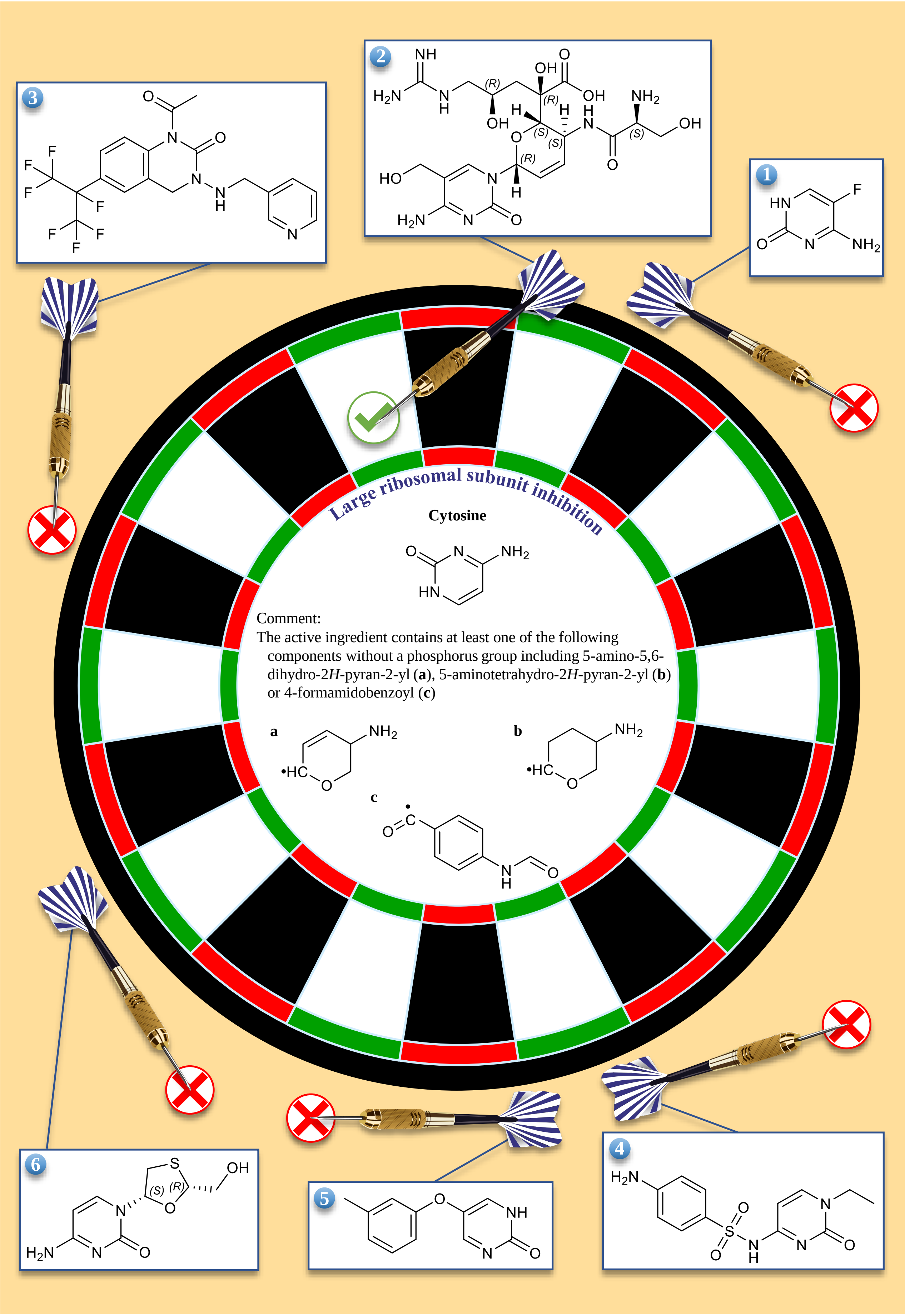
Large ribosomal subunit inhibition
Cytosine
Comment:
The active ingredient contains at least one of the following components without a phosphorus group including 5-amino-5,6-dihydro-2H-pyran-2-yl (a), 5-aminotetrahydro-2H-pyran-2-yl (b) or 4-formamidobenzoyl (c)
a
b
c
1
2
3
4
5
6

Figure S5. The identification of a bioactive molecule with the target of large ribosomal subunit inhibition by the minimum structure in cytosine group. Molecules 3 and 5 don't contain cytosine (the core) and molecules 1, 4 and 6 don't contain at least one of the following components including 5-amino-5,6-dihydro-2*H*-pyran-2-yl, 5-aminotetrahydro-2*H*-pyran-2-yl or 4-formamidobenzoyl. Therefore, the target of molecules 1, 3, 4, 5 and 6 is not large ribosomal subunit inhibition.

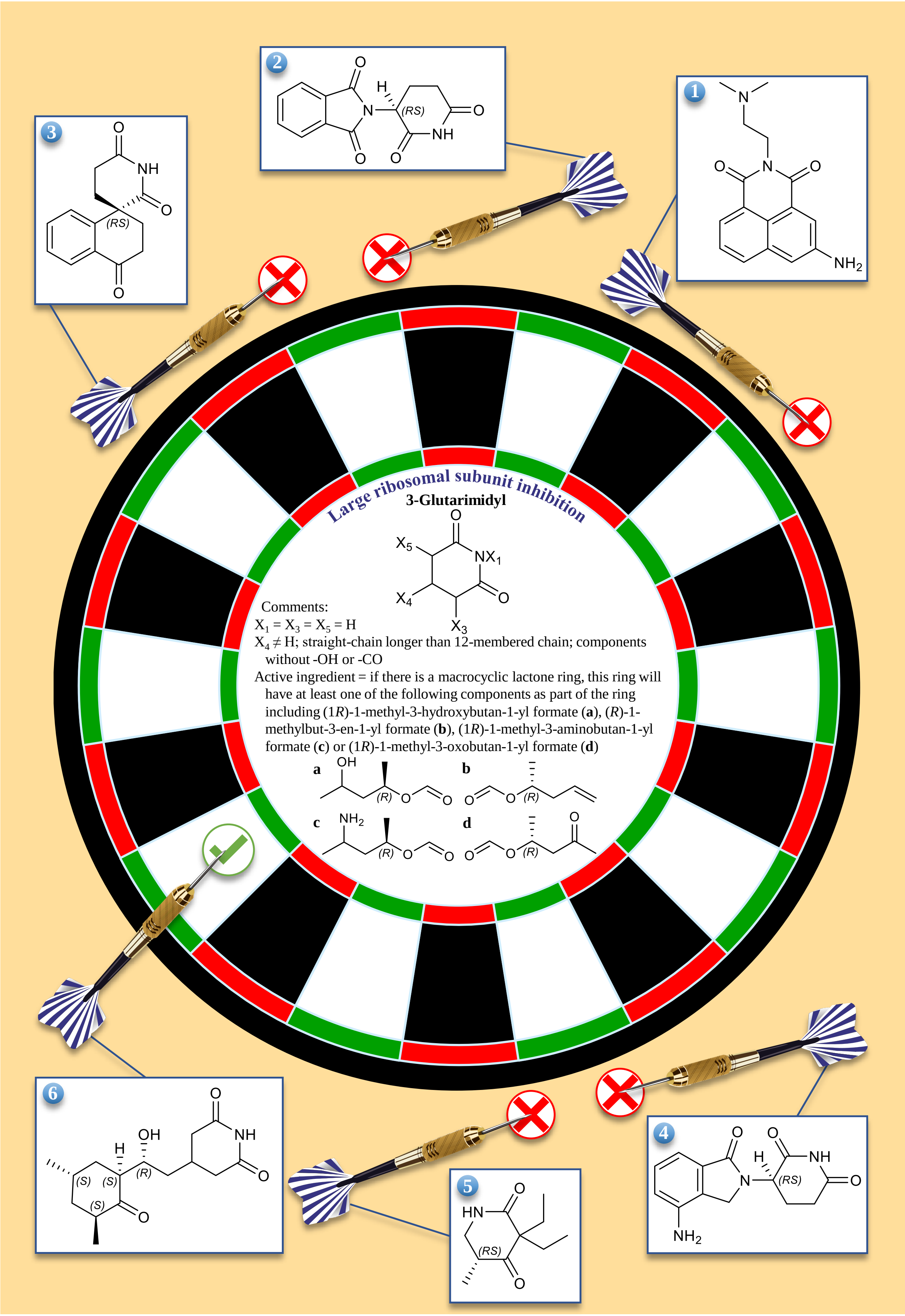

Figure S6 | Page 1 of 2

Figure S6. The identification of a bioactive molecule with the target of large ribosomal subunit inhibition by the minimum structure in 3-glutarimidyl group. Molecules 2, 3, 4 and 5 don't contain 3-glutarimidyl (the core) and molecule 1 contains components other than hydrogen at X_1, X_3 and X_5. Furthermore, molecule 1 contains components without -OH or -CO at X_4. Therefore, the target of molecules 1, 2, 3, 4 and 5 is not large ribosomal subunit inhibition.

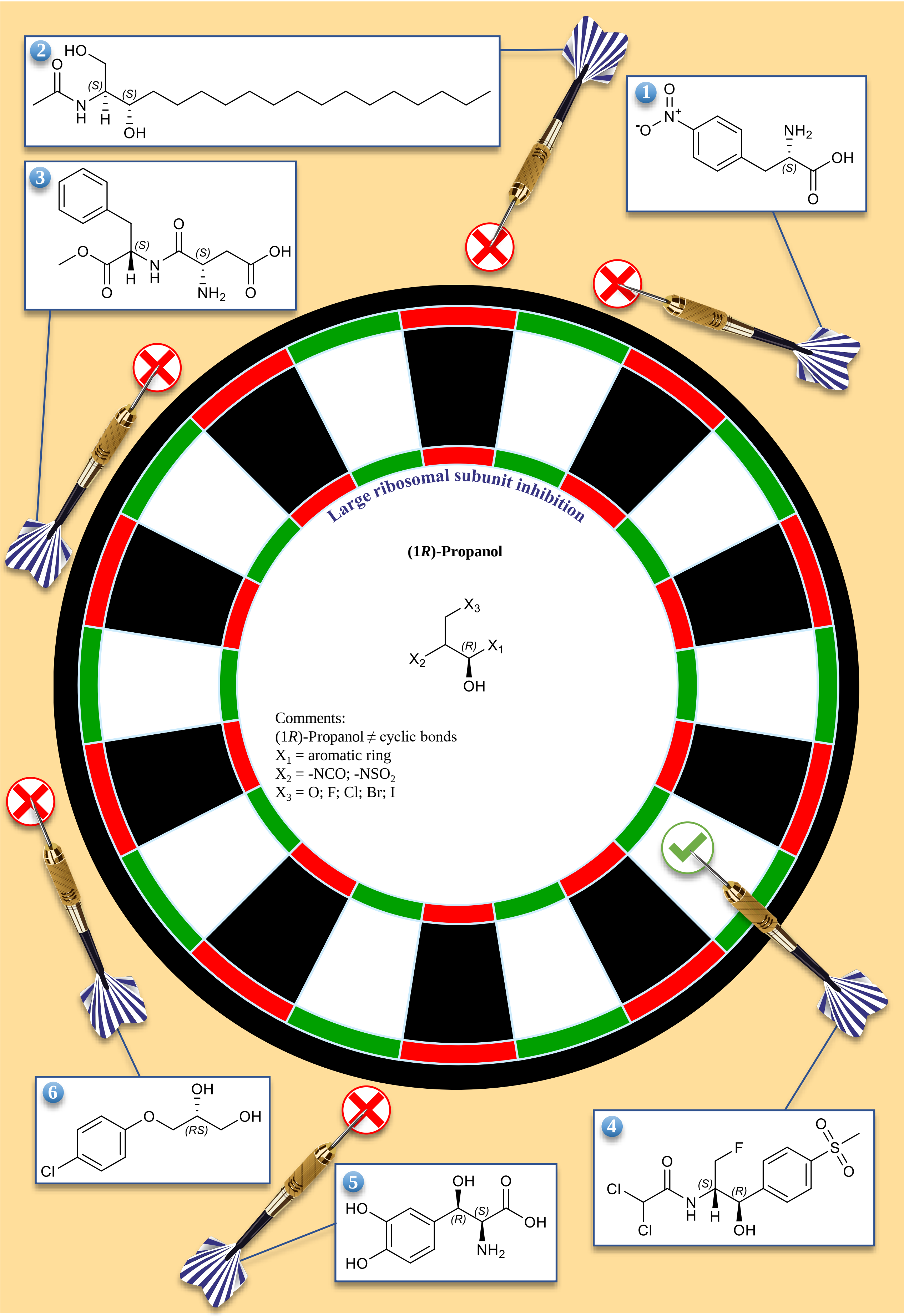
Large ribosomal subunit inhibition
(1R)-Propanol
Comments:
(1R)-Propanol ≠ cyclic bonds
X1 = aromatic ring
X2 = -NCO; -NSO2
X3 = O; F; Cl; Br; I
1
2
3
4
5
6

Figure S7. The identification of a bioactive molecule with the target of large ribosomal subunit inhibition by the minimum structure in (1*R*)-propanol group. Molecules 1, 2, 3 and 6 don't contain (1*R*)-propanol (the core) and molecule 5 doesn't contain -NCO or $-NSO_2$ at X_2. Therefore, the target of molecules 1, 2, 3, 5 and 6 is not large ribosomal subunit inhibition.

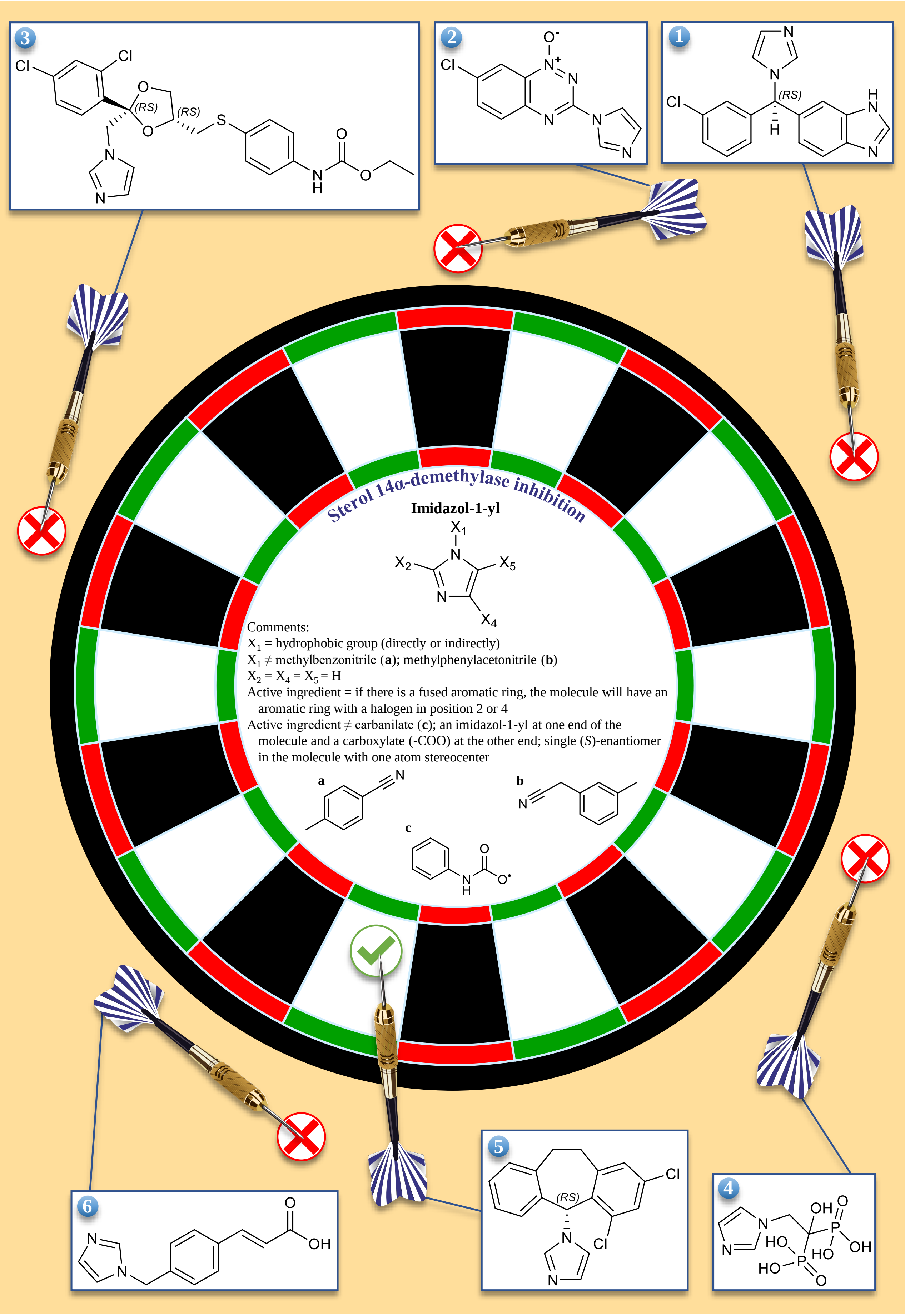
Sterol 14α-demethylase inhibition
Imidazol-1-yl
Comments:
X1 = hydrophobic group (directly or indirectly)
X1 ≠ methylbenzonitrile (a); methylphenylacetonitrile (b)
X2 = X4 = X5 = H
Active ingredient = if there is a fused aromatic ring, the molecule will have an aromatic ring with a halogen in position 2 or 4
Active ingredient ≠ carbanilate (c); an imidazol-1-yl at one end of the molecule and a carboxylate (-COO) at the other end; single (S)-enantiomer in the molecule with one atom stereocenter
a
b
c
1
2
3
4
5
6

Figure S8. The identification of a bioactive molecule with the target of sterol 14α-demethylase inhibition by the minimum structure in imidazol-1-yl group. Molecules 1 and 2 contain a fused aromatic ring but these molecules don't have an aromatic ring with a halogen in position 2 or 4, molecule 3 has a carbanilate, molecule 4 doesn't contain a hydrophobic group (directly or indirectly) at X_1 and molecule 6 contains an imidazol-1-yl at one end of the molecule and a carboxylate (-COO) at the other end. Therefore, the target of molecules 1, 2, 3, 4 and 6 is not sterol 14α-demethylase inhibition.

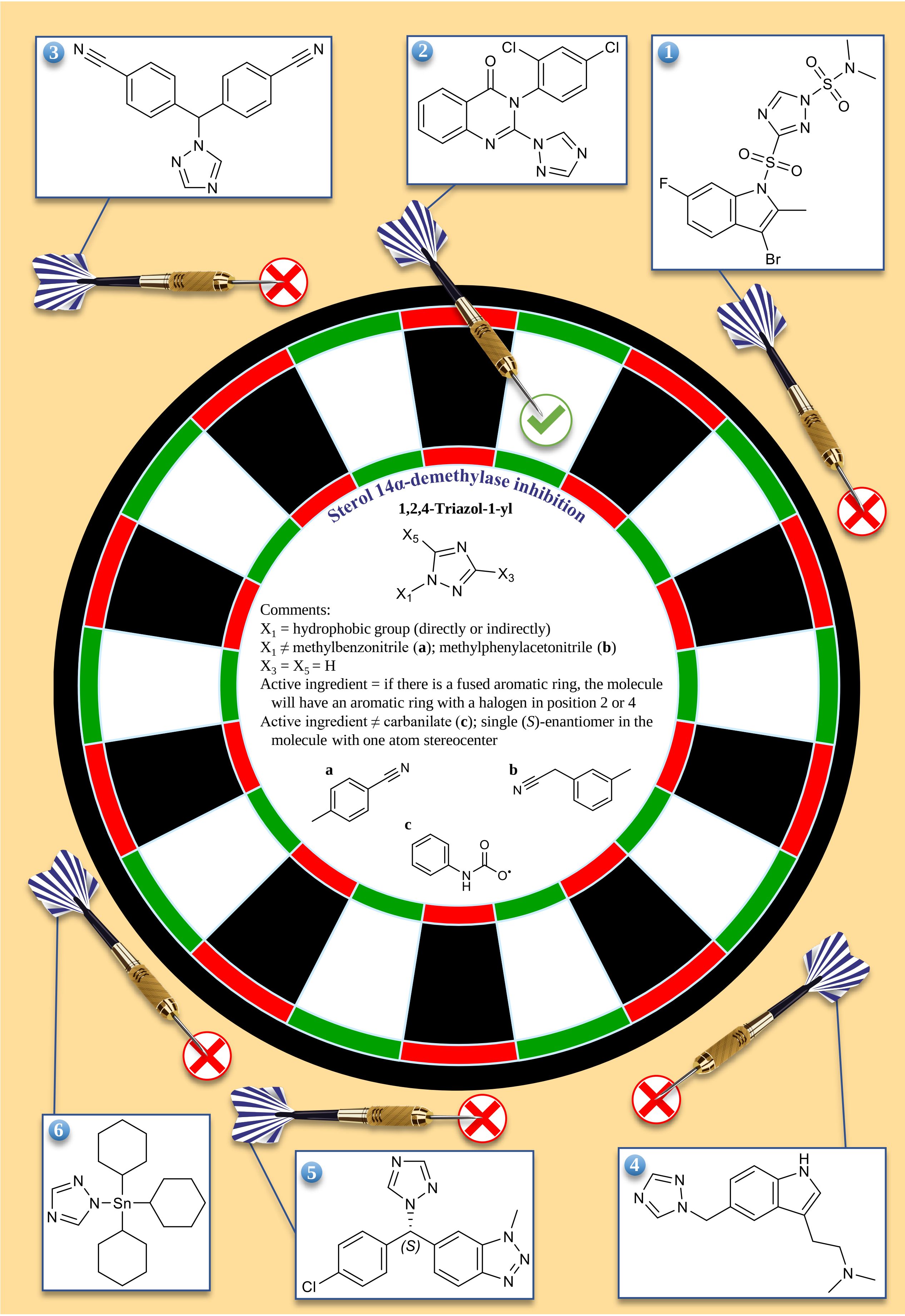
Sterol 14α-demethylase inhibition
1,2,4-Triazol-1-yl
Comments:
X1 = hydrophobic group (directly or indirectly)
X1 ≠ methylbenzonitrile (a); methylphenylacetonitrile (b)
X3 = X5 = H
Active ingredient = if there is a fused aromatic ring, the molecule will have an aromatic ring with a halogen in position 2 or 4
Active ingredient ≠ carbanilate (c); single (S)-enantiomer in the molecule with one atom stereocenter
a
b
c
1
2
3
4
5
6
(S)

Figure S9. The identification of a bioactive molecule with the target of sterol 14α-demethylase inhibition by the minimum structure in 1,2,4-triazol-1-yl group. Molecule 1 contains components other than hydrogen at X_3, molecule 3 has a methylbenzonitrile at X_1, molecule 4 contains a fused aromatic ring but this molecule doesn’t have an aromatic ring with a halogen in position 2 or 4, molecule 5 contains one atom stereocenter and single (*S*)-enantiomer and molecules 1 and 6 don’t contain a hydrophobic group (directly or indirectly) at X_1. Therefore, the target of molecules 1, 3, 4, 5 and 6 is not sterol 14α-demethylase inhibition.
