# Supplementary material for: Reliable Target Prediction of Bioactive Molecules Based on Chemical Similarity Without Employing Statistical Methods
Source: Front Pharmacol. 2019 Jul 26;10:835. doi: 10.3389/fphar.2019.00835 (PMC6676798; doi:10.3389/fphar.2019.00835)
Supplement: Supplementary file 2 [file DataSheet_2.pdf]

Table S1. Common name, CAS Registry Number, InChIKey, InChI, SMILES, primary target, structure and pharmacophore for bioactive molecules of the present study

Table S2. Primary targets published by scientific literature for bioactive molecules of the present study

Table S3. Primary targets annotated by PubChem, ChEMBL, KEGG and DrugBank databases for bioactive molecules of the present study

Table S4. Activity values of the molecules in ChEMBL database with a structural similarity of 85% and more to molecules in new predictions found with predicted targets of the proposed method

Table S5. Primary targets predicted by the computational target prediction tools for bioactive molecules of the present study

Table S1. Common name, CAS Registry Number, InChIKey, InChI, SMILES, primary target, structure and pharmacophore for bioactive molecules of the present study

Common name	CAS Reg. No.	InChIKey	InChI	SMILES	Primary target	2D Structure	Pharmacophore[1]
A 57132	100490-21-9	ROMLGDPRVIVYSV-UHFFFAOYSA-N	InChI=1S/C19H16F2N4O3/c20-11-1-3-12(4-2-11)25-10-14(19(27)28)16(26)13-9-15(21)18(23-17(13)25)24-7-5-22-6-8-24/h1-4,9-10,22H,5-8H2,(H,27,28)	FC1=CC=C(C=C1)N2C=C(C(C3=CC(F)=C(N=C32)N4CCNCC4)=O)C(O)=O	DNA gyrase and topoisomerase IV inhibitor	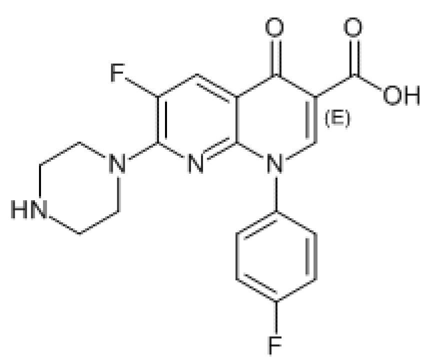	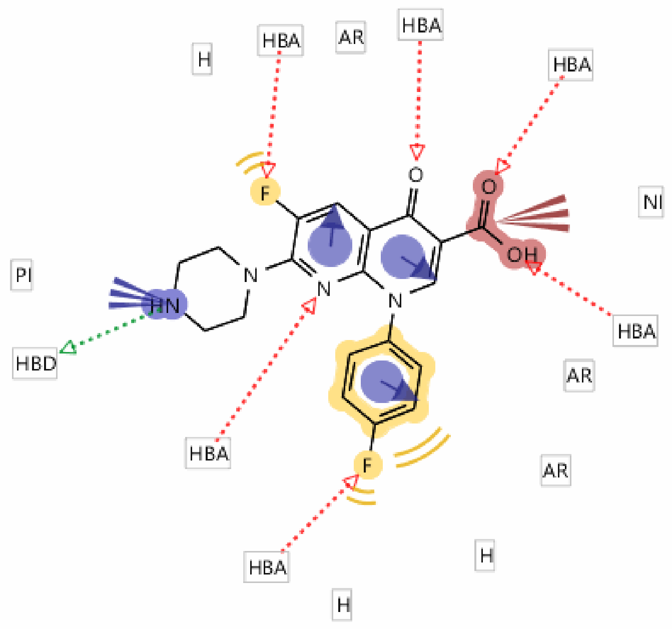
A 57241	98106-49-1	DVIWICJJGSWWRN-IZIBOJBPSA-N	InChI=1S/2C20H17F2N3O3/c2*21-11-1-3-13(4-2-11)25-10-15(20(27)28)19(26)14-7-16(22)18(8-17(14)25)24-6-5-12(23)9-24/h2*1-4,7-8,10,12H,5-6,9,23H2,(H,27,28)/t2*12-/m10/s1	O=C1C(C(O)=O)=CN(C2=C1C=C(C(N3CC[C@@H](C3)N)=C2)F)C4=CC=C(C=C4)F.O=C5C(C(O)=O)=CN(C6=C5C=C(C(N7CC[C@H](C7)N)=C6)F)C8=CC=C(C=C8)F	DNA gyrase and topoisomerase IV inhibitor	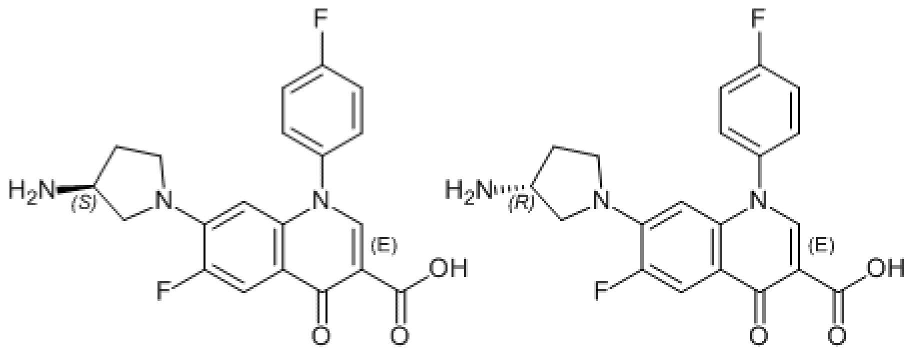	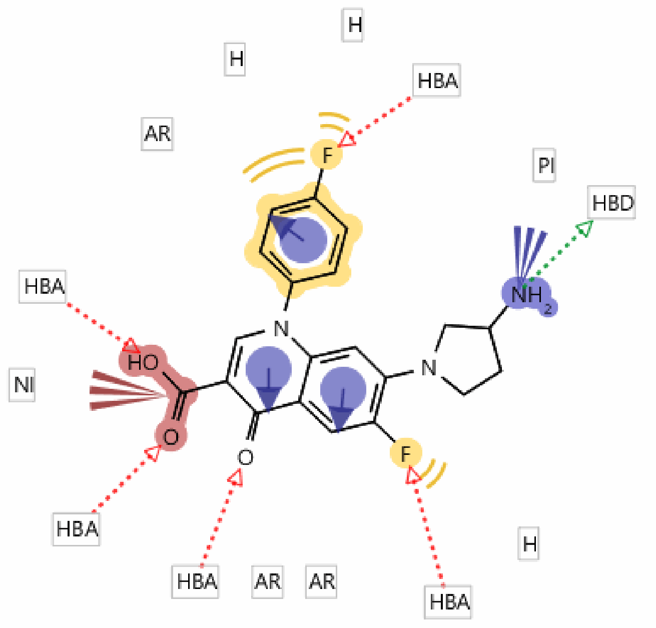
A 57274 (A 62917)	100490-19-5	CVAQZAKLVQJAQT-UHFFFAOYSA-N	InChI=1S/C19H15F3N4O3/c20-10-1-2-15(13(21)7-10)26-9-12(19(28)29)16(27)11-8-14(22)18(24-17(11)26)25-5-3-23-4-6-25/h1-2,7-9,23H,3-6H2,(H,28,29)	FC1=CC(F)=C(N2C=C(C(O)=O)C(C3=CC(F)=C(N4CCNCC4)N=C32)=O)C=C1	DNA gyrase and topoisomerase IV inhibitor	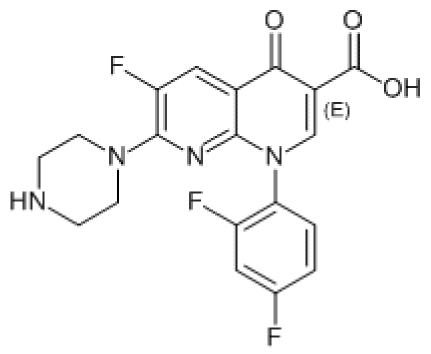	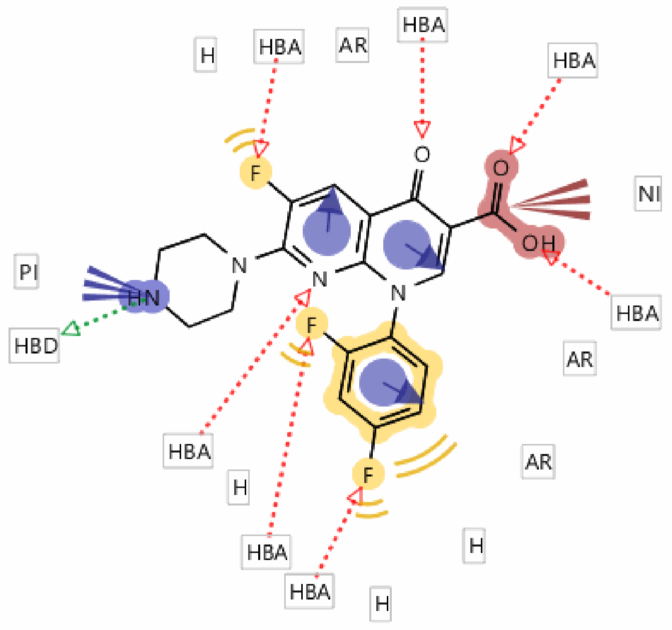
A 60919 (PD 118106)	99735-41-8	SKYHBWSFRMRRRT-UHFFFAOYSA-N	InChI=1S/C16H17FN4O3/c17-12-7-10-13(22)11(16(23)24)8-21(9-1-2-9)14(10)19-15(12)20-5-3-18-4-6-20/h7-9,18H,1-6H2,(H,23,24)	O=C1C2=CC(F)=C(N=C2N(C3CC3)C=C1C(O)=O)N4CCNCC4	DNA gyrase and topoisomerase IV inhibitor	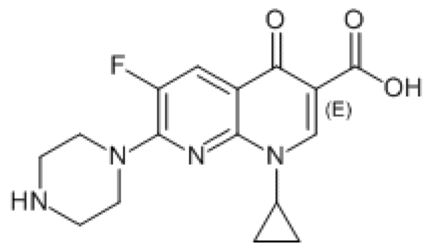	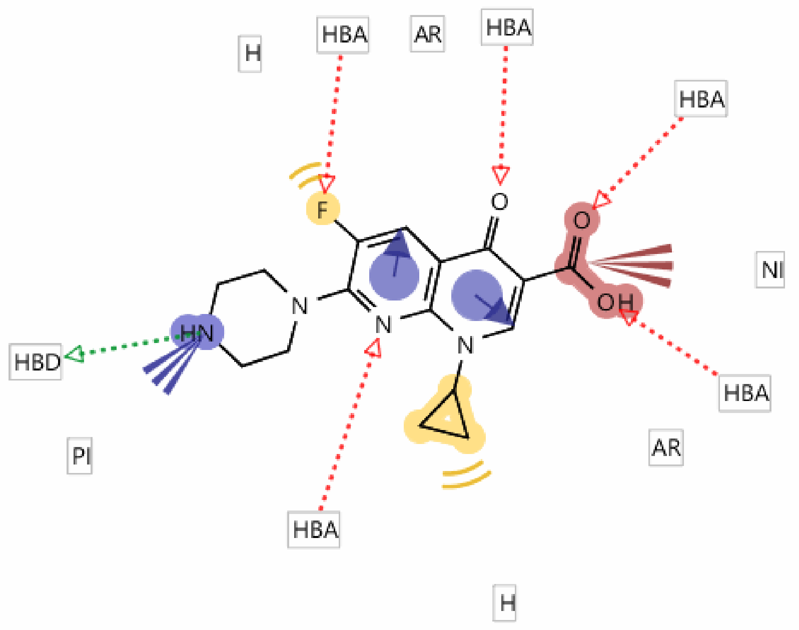
A 61867 (BRN 4276829)	102856-07-5	YPNGGDMKKVRWBI-FGYXOPSTSA-N	InChI=1S/2C20H16F3N3O3/c2*21-10-1-2-16(14(22)5-10)26-9-13(20(28)29)19(27)12-6-15(23)18(7-17(12)26)25-4-3-11(24)8-25/h2*1-2,5-7,9,11H,3-4,8,24H2,(H,28,29)/t2*11-/m10/s1	O=C1C(C(O)=O)=CN(C2=CC=C(F)C=C2F)C3=C1C=C(F)C(N4CC[C@@H](N)C4)=C3.O=C5C(C(O)=O)=CN(C6=CC=C(F)C=C6F)C7=C5C=C(F)C(N8CC[C@H](N)C8)=C7	DNA gyrase and topoisomerase IV inhibitor	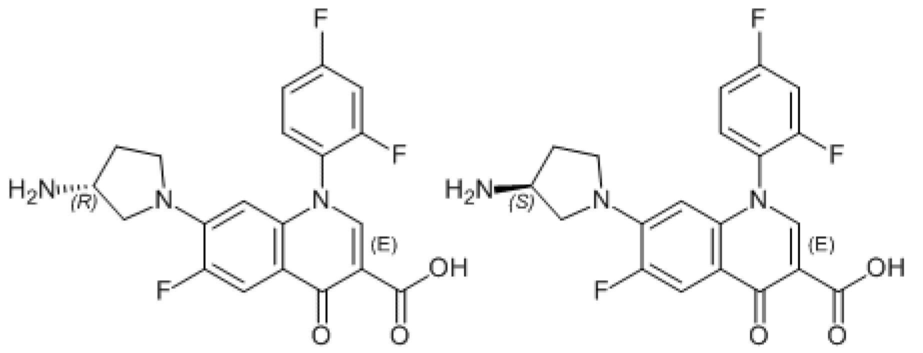	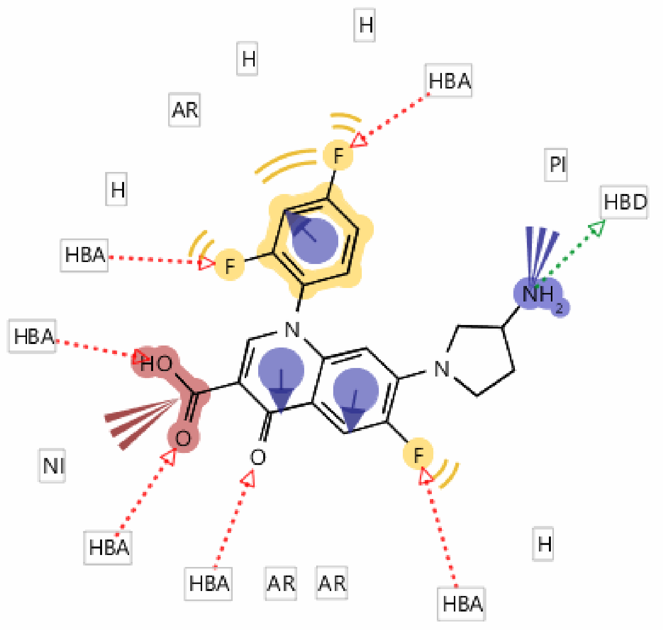

Common name	CAS Reg. No.	InChIKey	InChI	SMILES	Primary target	2D Structure	Pharmacophore[1]
A 62251 (A 57531; PD 137954)	108138-27-8	AAMFCDRGHVWGAW-UHFFFAOYSA-N	InChI=1S/C20H16F3N3O3/c21-11-1-2-16(14(22)7-11)26-10-13(20(28)29)19(27)12-8-15(23)18(9-17(12)26)25-5-3-24-4-6-25/h1-2,7-10,24H,3-6H2,(H,28,29)	O=C1C(C(O)=O)=CN(C2=C(F)C=C(F)C=C2)C3=CC(N4CCNCC4)=C(F)C=C31	DNA gyrase and topoisomerase IV inhibitor	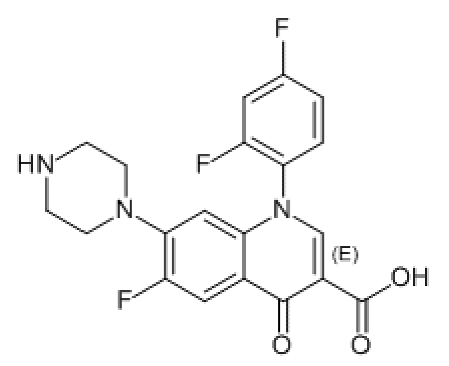	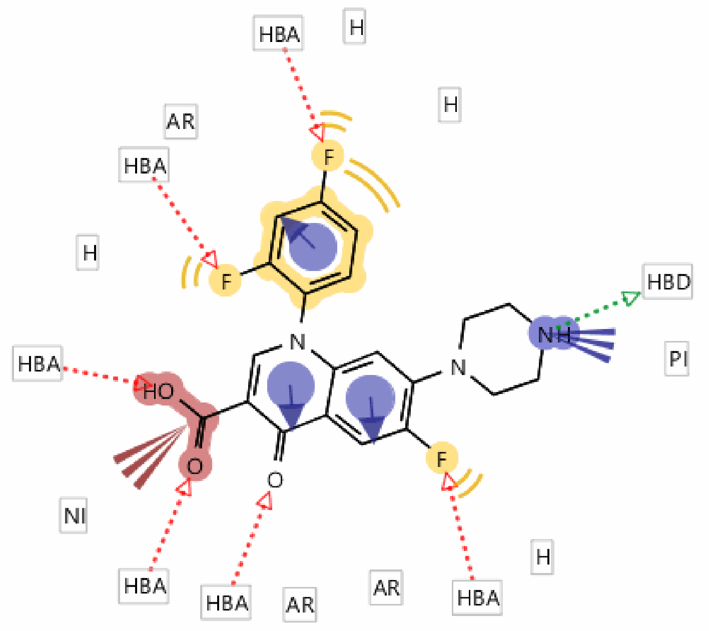
A 62255	119530-21-1	OIIYFHOOSMWVAK-IZIBOJBPSA-N	InChI=1S/2C21H19F2N3O3/c2*1-12-10-25(7-6-24-12)19-9-18-15(8-17(19)23)20(27)16(21(28)29)11-26(18)14-4-2-13(22)3-5-14/h2*2-5,8-9,11-12,24H,6-7,10H2,1H3,(H,28,29)/t2*12-/m10/s1	O=C1C(C(O)=O)=CN(C2=C1C=C(C(N3CCN[C@H](C)C3)=C2)F)C4=CC=C(C=C4)F.O=C5C(C(O)=O)=CN(C6=C5C=C(C(N7CCN[C@@H](C)C7)=C6)F)C8=CC=C(C=C8)F	DNA gyrase and topoisomerase IV inhibitor	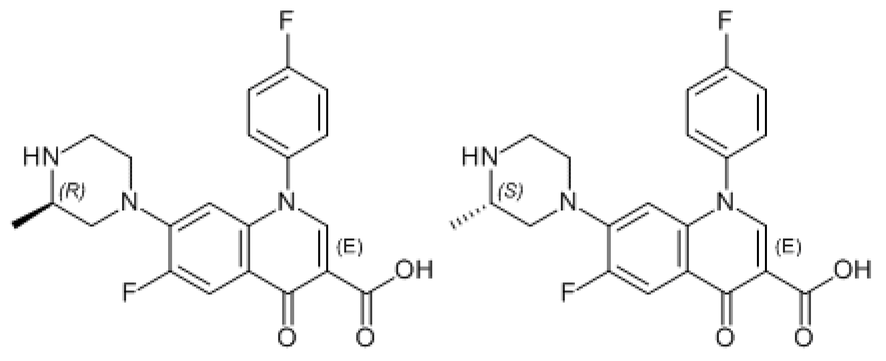	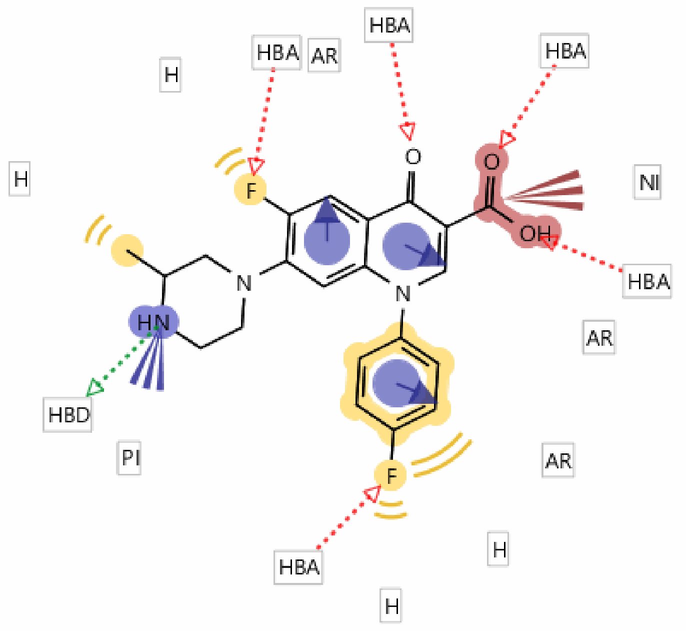
A 62824	111279-87-9	BEZDZMQEVWAVTH-UHFFFAOYSA-N	InChI=1S/C17H17FN4O2S/c18-11-7-10-12(8-13(11)21-5-3-19-4-6-21)22(9-1-2-9)17-14(15(10)23)16(24)20-25-17/h7-9,19H,1-6H2,(H,20,24)	O=C(C1=C2SNC1=O)C3=CC(F)=C(N4CCNCC4)C=C3N2C5CC5	DNA gyrase and topoisomerase IV inhibitor	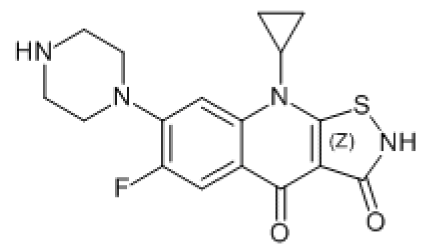	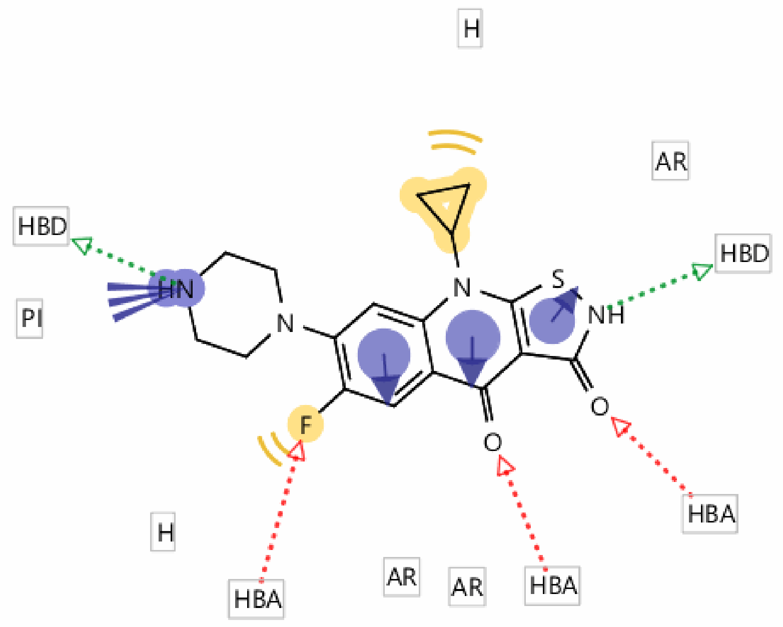
A 65326		CQSCBORSOIETFL-JQWIXIFHSA-N	InChI=1S/C21H18F3N3O3/c1-10-4-12(25)8-26(10)19-7-18-13(6-16(19)24)20(28)14(21(29)30)9-27(18)17-3-2-11(22)5-15(17)23/h2-3,5-7,9-10,12H,4,8,25H2,1H3,(H,29,30)/t10-,12-/m0/s1	C[C@H]1C[C@H](N)CN1C2=C(F)C=C(C(C(C(O)=O)=CN3C4=C(F)C=C(F)C=C4)=O)C3=C2	DNA gyrase and topoisomerase IV inhibitor	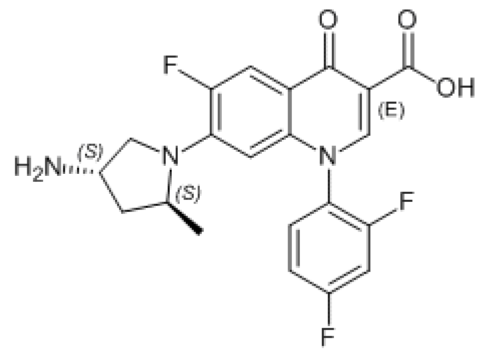	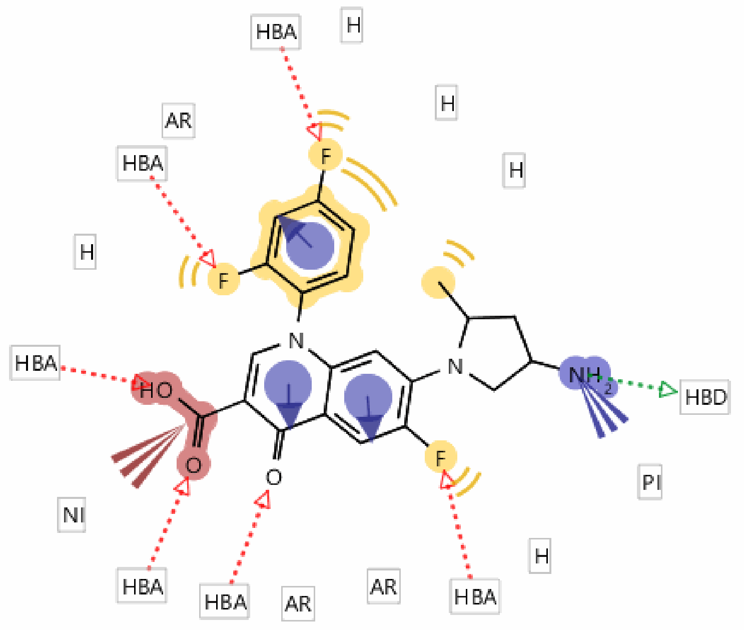
ACH 702	922491-46-1	CECJUHKZSOSOJJ-SNVBAGLBSA-N	InChI=1S/C21H25FN4O3S/c1-21(2,23)10-6-7-25(9-10)16-13(22)8-12-15(18(16)29-3)26(11-4-5-11)20-14(17(12)27)19(28)24-30-20/h8,10-11H,4-7,9,23H2,1-3H3,(H,24,28)/t10-/m1/s1	CC(N)(C)[C@@H]1CCN(C1)C2=C(C=C3C(N(C4=C(C3=O)C(NS4)=O)C5CC5)=C2OC)F	DNA gyrase and topoisomerase IV inhibitor	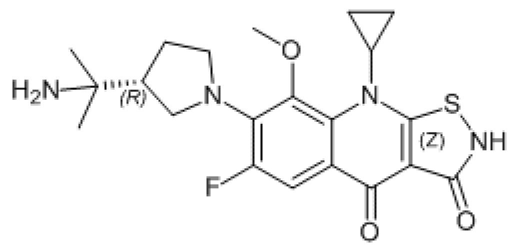	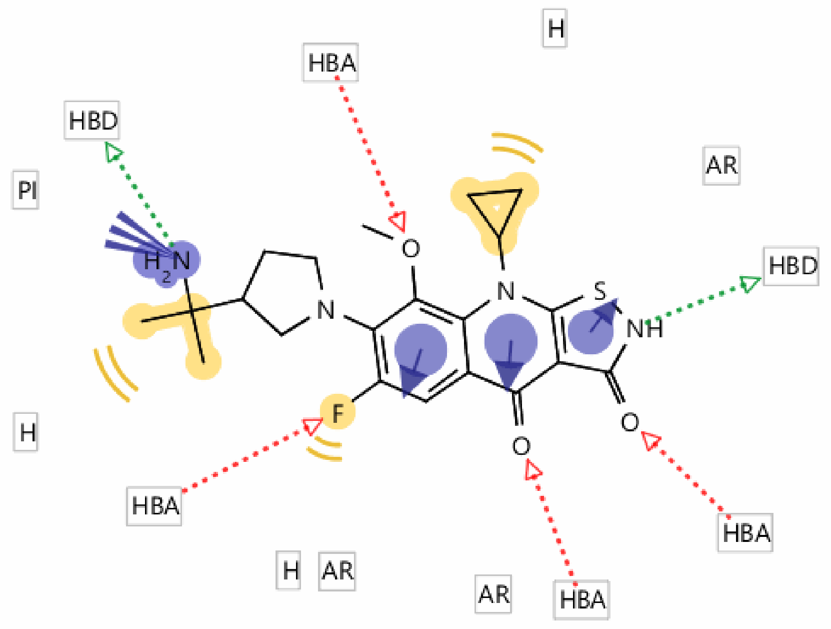
Acorafloxacin (avarofloxacin)	878592-87-1	VMKVDAAFMQKZJS-LFIBNONCSA-N	InChI=1S/C21H23F2N3O4/c1-30-20-17-13(19(27)14(21(28)29)10-26(17)12-4-5-12)7-15(22)18(20)25-6-2-3-11(9-25)16(23)8-24/h7,10,12H,2-6,8-9,24H2,1H3,(H,28,29)/b16-11+	COC1=C2C(C(C(C(O)=O)=CN2C3CC3)=O)=CC(F)=C1N4CCC/C(C4)=C(F)/CN	DNA gyrase and topoisomerase IV inhibitor	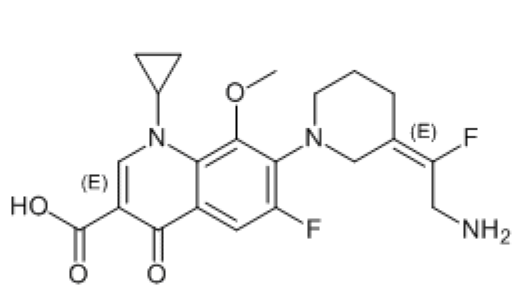	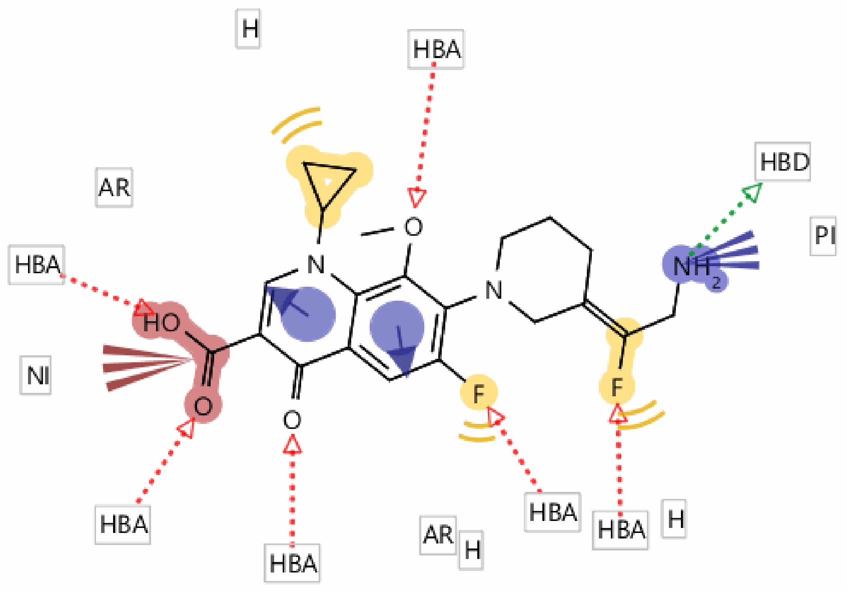

Common name	CAS Reg. No.	InChIKey	InChI	SMILES	Primary target	2D Structure	Pharmacophore[1]
ADDNC (A 65485)	114676-84-5	IHZWSSPWZKJJMD-ONGXEEELSA-N	InChI=1S/C20H17F3N4O3/c1-9-4-11(24)7-26(9)19-15(23)6-12-17(28)13(20(29)30)8-27(18(12)25-19)16-3-2-10(21)5-14(16)22/h2-3,5-6,8-9,11H,4,7,24H2,1H3,(H,29,30)/t9-,11-/m0/s1	C[C@H]1C[C@H](N)CN1C2=C(F)C=C(C3=N2)C(C(C(O)=O)=CN3C4=C(F)C=C(F)C=C4)=O	DNA gyrase and topoisomerase IV inhibitor	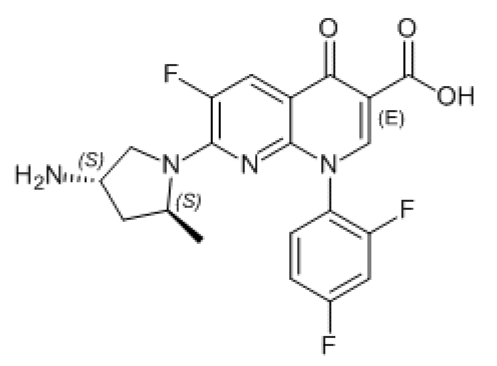	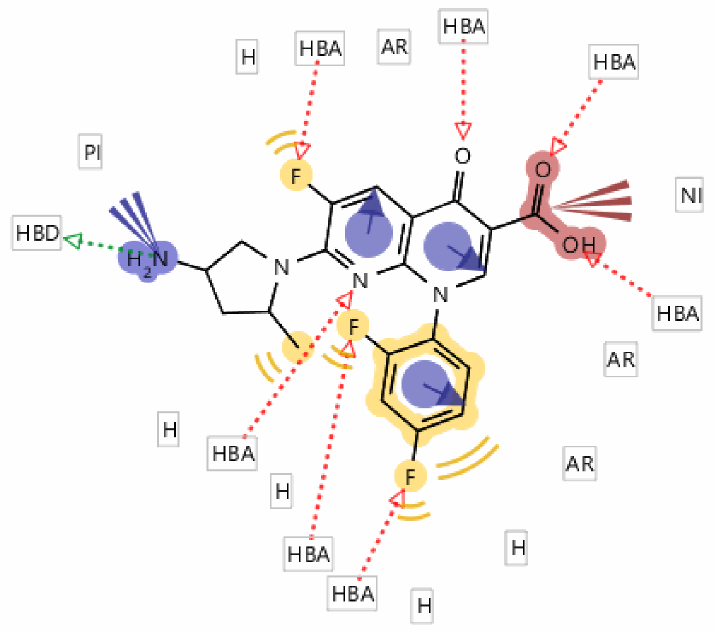
Alalevonadifloxacin	706809-20-3	OUXXDXXQNWKOIF-RYUDHWBXSA-N	InChI=1S/C22H26FN3O5/c1-11-3-4-14-18-15(20(27)16(21(28)29)10-26(11)18)9-17(23)19(14)25-7-5-13(6-8-25)31-22(30)12(2)24/h9-13H,3-8,24H2,1-2H3,(H,28,29)/t11-,12-/m0/s1	C[C@H]1CCC2=C3C(C(C(C(O)=O)=CN13)=O)=CC(F)=C2N4CCC(CC4)OC([C@H](C)N)=O	DNA gyrase and topoisomerase IV inhibitor	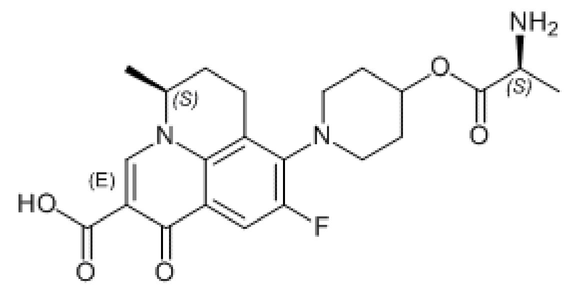	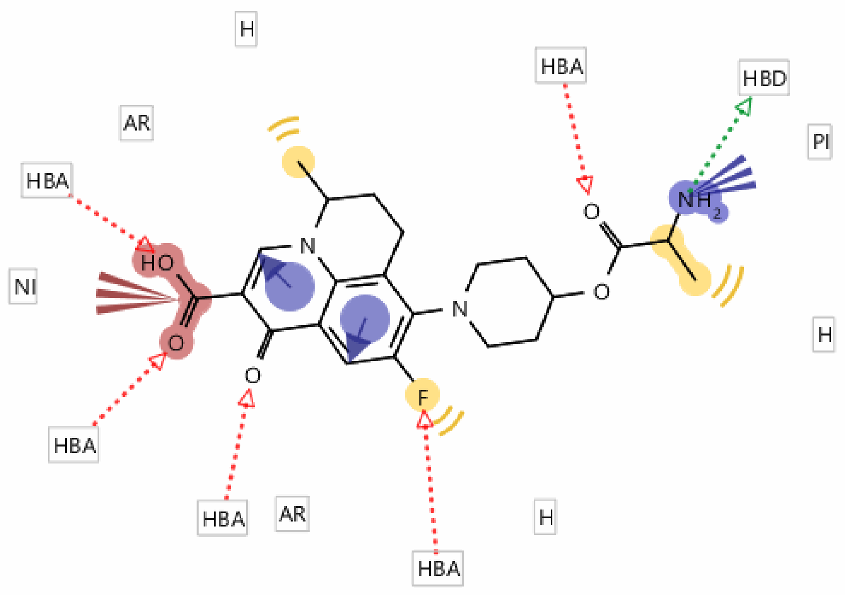
Alatrofloxacin	146961-76-4	UUZPPAMZDFLUHD-VUJLHGSVSA-N	InChI=1S/C26H25F3N6O5/c1-10(30)24(37)31-11(2)25(38)32-20-14-7-34(8-15(14)20)23-18(29)6-13-21(36)16(26(39)40)9-35(22(13)33-23)19-4-3-12(27)5-17(19)28/h3-6,9-11,14-15,20H,7-8,30H2,1-2H3,(H,31,37)(H,32,38)(H,39,40)/t10-,11-,14-,15+,20+/m0/s1	C[C@H](N)C(N[C@H](C(N[C@@H]1[C@@]2([H])CN(C3=C(F)C=C(C4=N3)C(C(C(O)=O)=CN4C5=C(F)C=C(F)C=C5)=O)C[C@]21[H])=O)C)=O	DNA gyrase and topoisomerase IV inhibitor	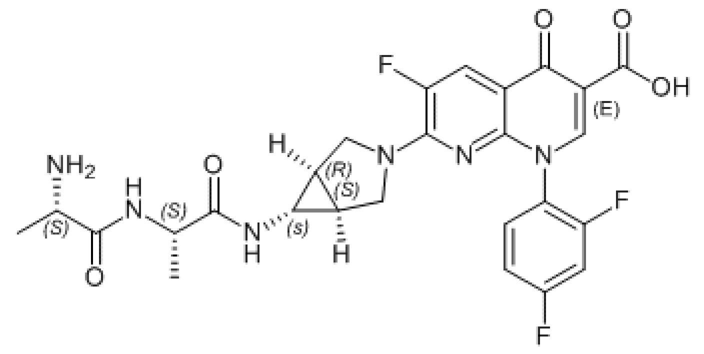	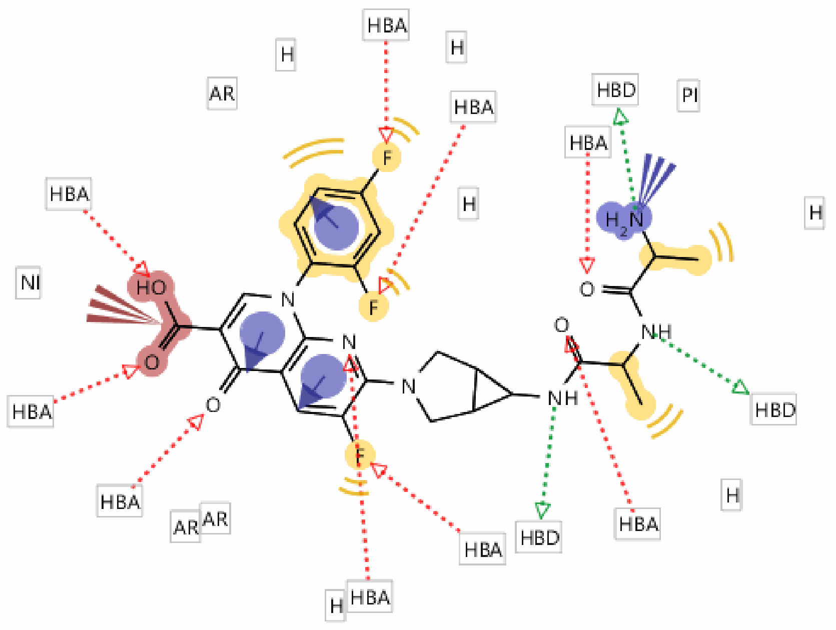
Amifloxacin	86393-37-5	RUXPNBWPIRDVTH-UHFFFAOYSA-N	InChI=1S/C16H19FN4O3/c1-18-21-9-11(16(23)24)15(22)10-7-12(17)14(8-13(10)21)20-5-3-19(2)4-6-20/h7-9,18H,3-6H2,1-2H3,(H,23,24)	CNN1C=C(C(C2=CC(F)=C(C=C21)N3CCN(C)CC3)=O)C(O)=O	DNA gyrase and topoisomerase IV inhibitor	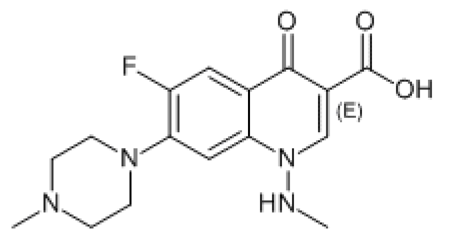	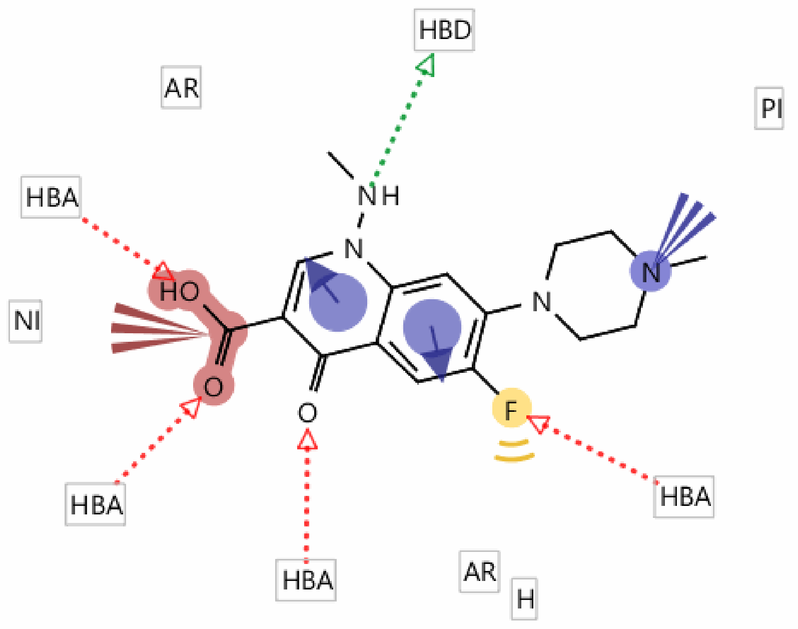
Antofloxacin	119354-43-7	MHFCVNDHFSOKMK-VIFPVBQESA-N	InChI=1S/C18H21FN4O4/c1-9-8-27-17-14-11(16(24)10(18(25)26)7-23(9)14)13(20)12(19)15(17)22-5-3-21(2)4-6-22/h7,9H,3-6,8,20H2,1-2H3,(H,25,26)/t9-/m0/s1	C[C@H]1COC2=C3C(C(C(C(O)=O)=CN13)=O)=C(C(F)=C2N4CCN(C)CC4)N	DNA gyrase and topoisomerase IV inhibitor	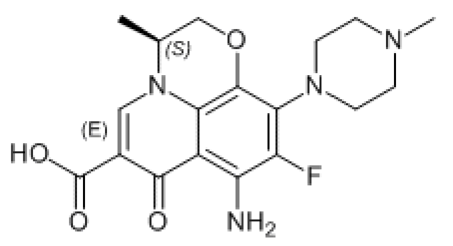	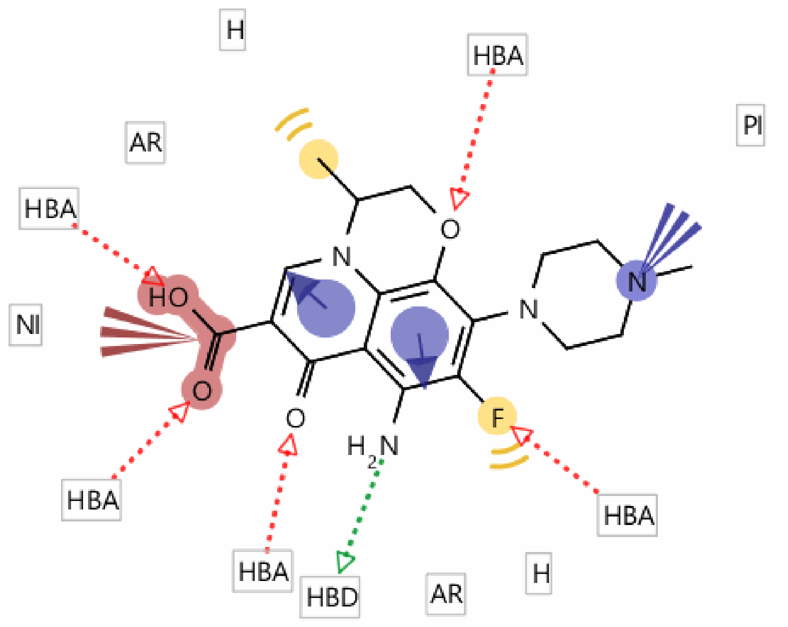
AT 4929		CMFPAJWVRYDUFP-AOOOYVTPSA-N	InChI=1S/C19H21F2N3O3/c1-9-6-23(7-10(2)22-9)14-5-13-15(17(21)16(14)20)18(25)12(19(26)27)8-24(13)11-3-4-11/h5,8-11,22H,3-4,6-7H2,1-2H3,(H,26,27)/t9-,10+	C[C@@H]1CN(C2=C(F)C(F)=C3C(N(C4CC4)C=C(C(O)=O)C3=O)=C2)C[C@H](C)N1	DNA gyrase and topoisomerase IV inhibitor	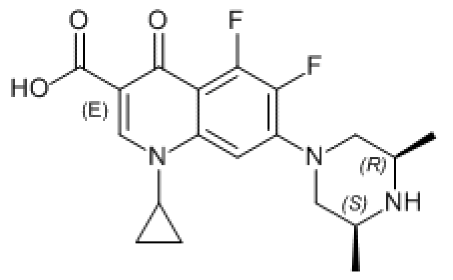	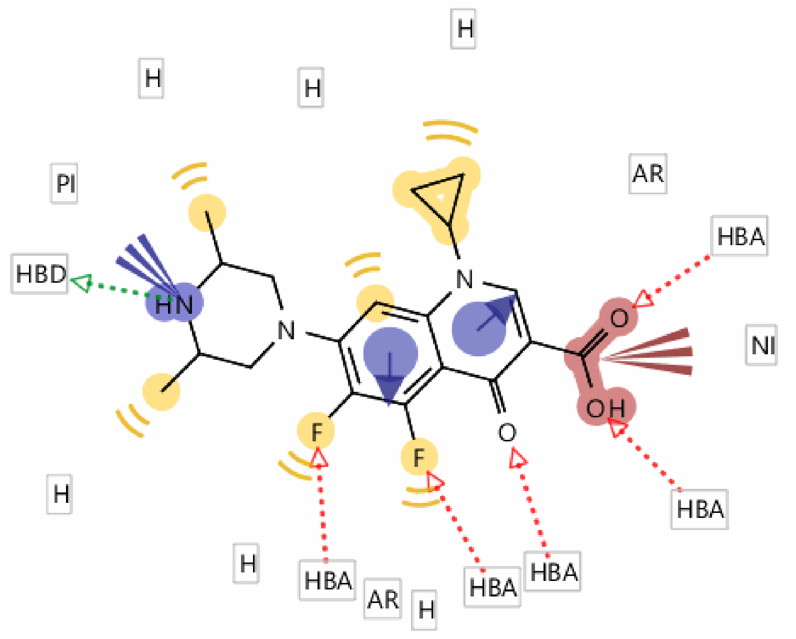

Common name	CAS Reg. No.	InChIKey	InChI	SMILES	Primary target	2D Structure	Pharmacophore[1]
Balofloxacin	127294-70-6	HXMCIKMQHQEIPI-FGYXOPSTSA-N	InChI=1S/2C20H24FN3O4/c2*1-22-11-4-3-7-23(9-11)17-15(21)8-13-16(19(17)28-2)24(12-5-6-12)10-14(18(13)25)20(26)27/h2*8,10-12,22H,3-7,9H2,1-2H3,(H,26,27)/t2*11-/m10/s1	CN[C@@H]1CCCN(C2=C(F)C=C(C(C(C(O)=O)=CN3C4CC4)=O)C3=C2OC)C1.CN[C@H]5CCCN(C6=C(F)C=C(C(C(C(O)=O)=CN7C8CC8)=O)C7=C6OC)C5	DNA gyrase and topoisomerase IV inhibitor	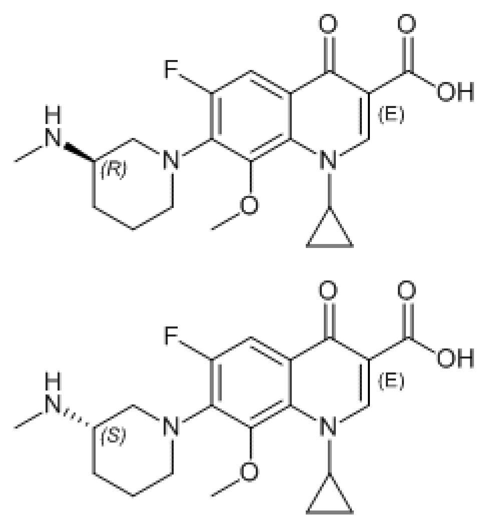	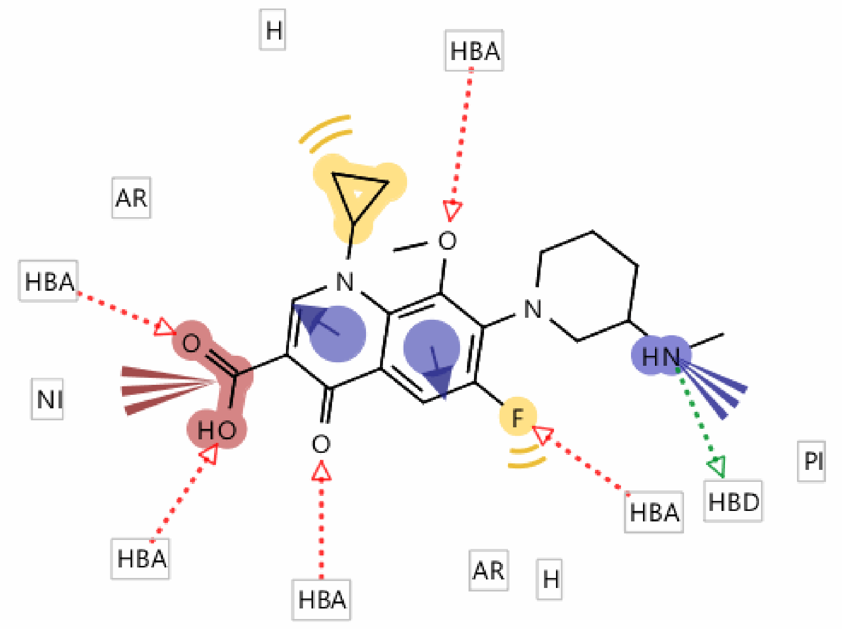
BAY Y-3118 free base	151213-16-0	VRXORHRXNRJZCQ-ZUZCIYMTSA-N	InChI=1S/C20H21ClFN3O3/c21-16-17-12(19(26)13(20(27)28)8-25(17)11-3-4-11)6-14(22)18(16)24-7-10-2-1-5-23-15(10)9-24/h6,8,10-11,15,23H,1-5,7,9H2,(H,27,28)/t10-,15+/m0/s1	ClC1=C2C(C(C(C(O)=O)=CN2C3CC3)=O)=CC(F)=C1N4C[C@]5([H])CCCN[C@]5([H])C4	DNA gyrase and topoisomerase IV inhibitor	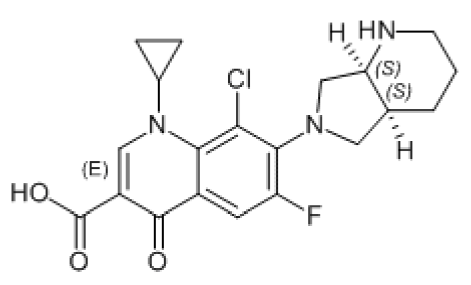	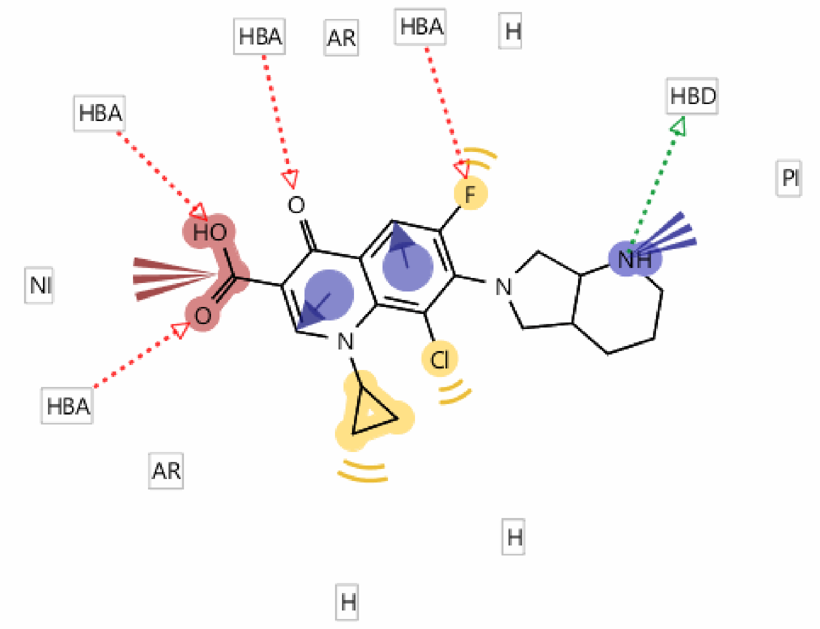
Besifloxacin	141388-76-3	QFFGVLORLPOAEC-SNVBAGLBSA-N	InChI=1S/C19H21ClFN3O3/c20-15-16-12(18(25)13(19(26)27)9-24(16)11-4-5-11)7-14(21)17(15)23-6-2-1-3-10(22)8-23/h7,9-11H,1-6,8,22H2,(H,26,27)/t10-/m1/s1	ClC1=C2C(C(C(C(O)=O)=CN2C3CC3)=O)=CC(F)=C1N4CCCC[C@@H](N)C4	DNA gyrase and topoisomerase IV inhibitor	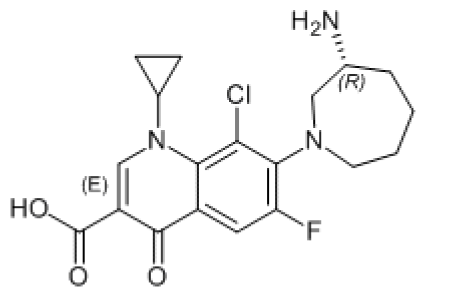	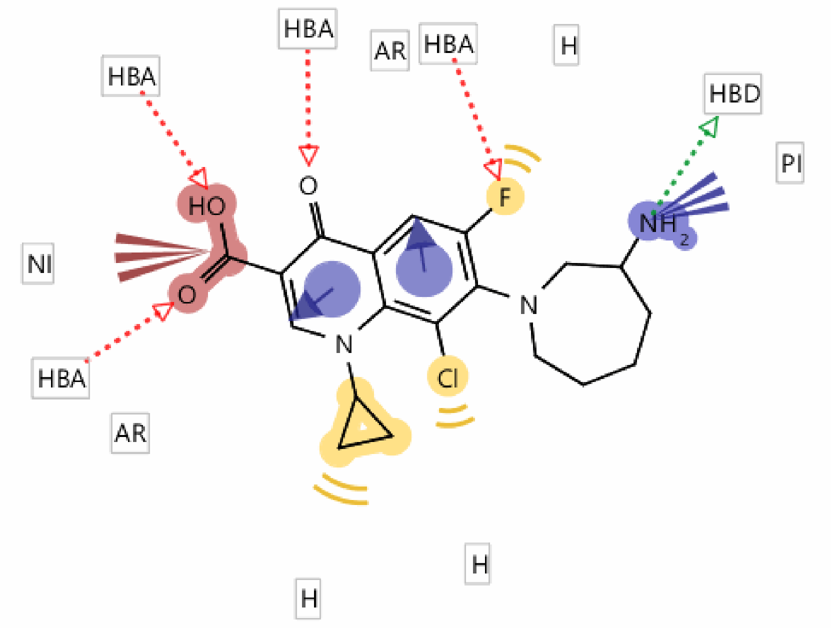
Binfloxacin	108437-28-1	UBGCCYGMLOBJOJ-UHFFFAOYSA-N	InChI=1S/C19H22FN3O3/c1-2-22-11-14(19(25)26)18(24)13-9-15(20)17(10-16(13)22)23-8-7-21-5-3-12(23)4-6-21/h9-12H,2-8H2,1H3,(H,25,26)	CCN1C=C(C(C2=CC(F)=C(C=C21)N3CCN4CCC3CC4)=O)C(O)=O	DNA gyrase and topoisomerase IV inhibitor	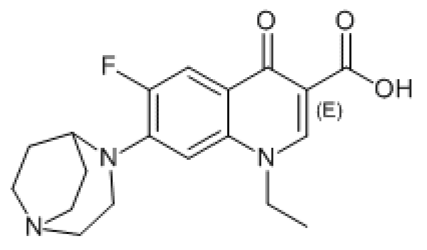	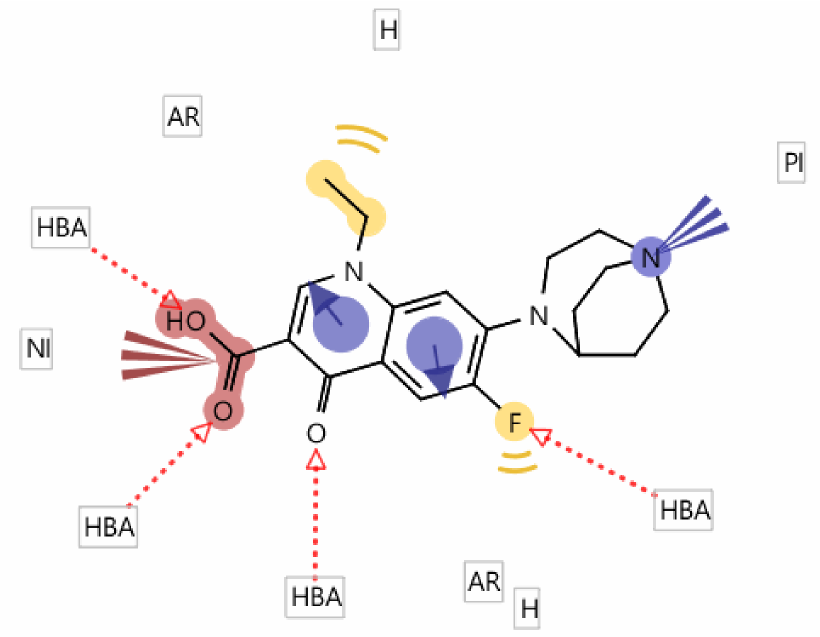
BMY 40062	116143-32-9	KNHLHFDFFDCJJR-NXEZZACHSA-N	InChI=1S/C18H21FN4O3/c1-18(2,3)23-8-12(17(25)26)14(24)11-5-13(19)16(21-15(11)23)22-7-9-4-10(22)6-20-9/h5,8-10,20H,4,6-7H2,1-3H3,(H,25,26)/t9-,10-/m1/s1	CC(N1C=C(C(C2=CC(F)=C(N=C21)N3C[C@H]4C[C@@H]3CN4)=O)C(O)=O)(C)C	DNA gyrase and topoisomerase IV inhibitor	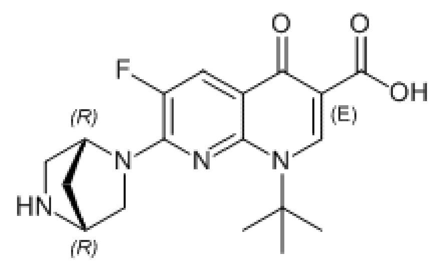	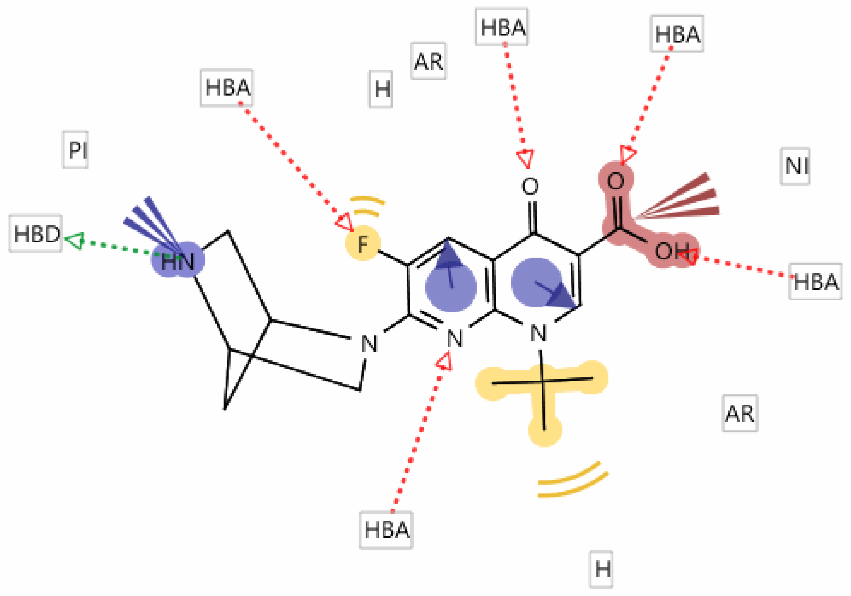
BMY 40397		TZPGWYMANNLLGM-HUUCEWRRSA-N	InChI=1S/C31H32F2N6O6/c1-30(2,3)38-12-18(28(42)43)22(40)16-8-20(32)26(34-24(16)38)36-10-15-7-14(36)11-37(15)27-21(33)9-17-23(41)19(29(44)45)13-39(25(17)35-27)31(4,5)6/h8-9,12-15H,7,10-11H2,1-6H3,(H,42,43)(H,44,45)/t14-,15-/m1/s1	CC(C)(C)N1C=C(C(O)=O)C(C2=CC(F)=C(N3C[C@H]4C[C@@H]3CN4C5=C(F)C=C6C(C(C(O)=O)=CN(C(C)(C)C)C6=N5)=O)N=C21)=O	DNA gyrase and topoisomerase IV inhibitor	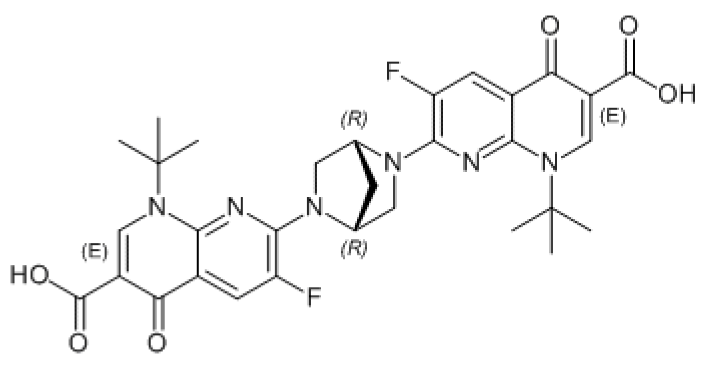	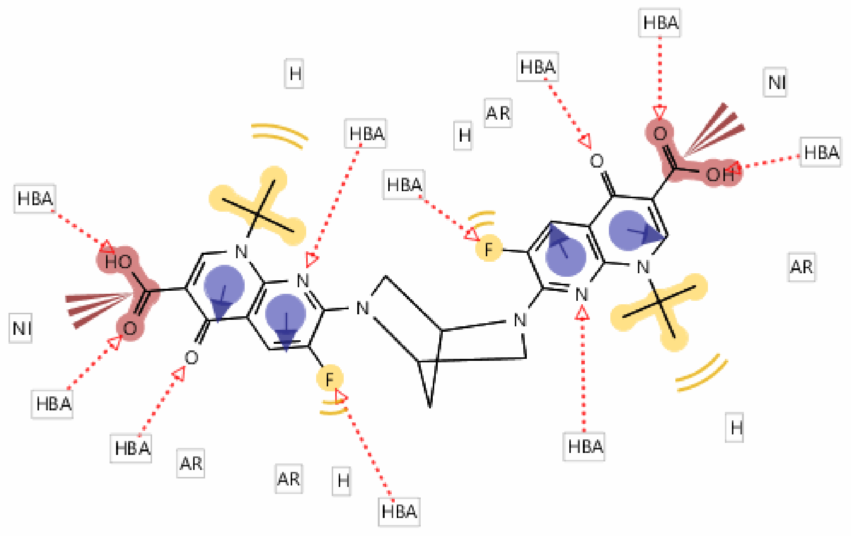

Common name	CAS Reg. No.	InChIKey	InChI	SMILES	Primary target	2D Structure	Pharmacophore[1]
BMY 42230		MERNMZCMIBTAPW-UHFFFAOYSA-N	InChI=1S/C17H20F2N4O3/c1-17(2,9-18)23-8-11(16(25)26)13(24)10-7-12(19)15(21-14(10)23)22-5-3-20-4-6-22/h7-8,20H,3-6,9H2,1-2H3,(H,25,26)	CC(N1C=C(C(C2=CC(F)=C(N=C21)N3CCNCC3)=O)C(O)=O)(C)CF	DNA gyrase and topoisomerase IV inhibitor	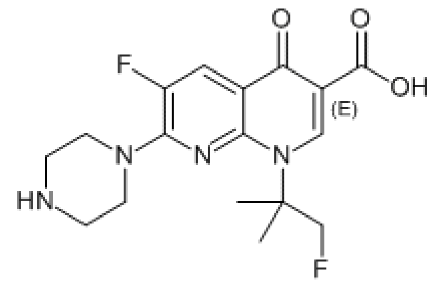	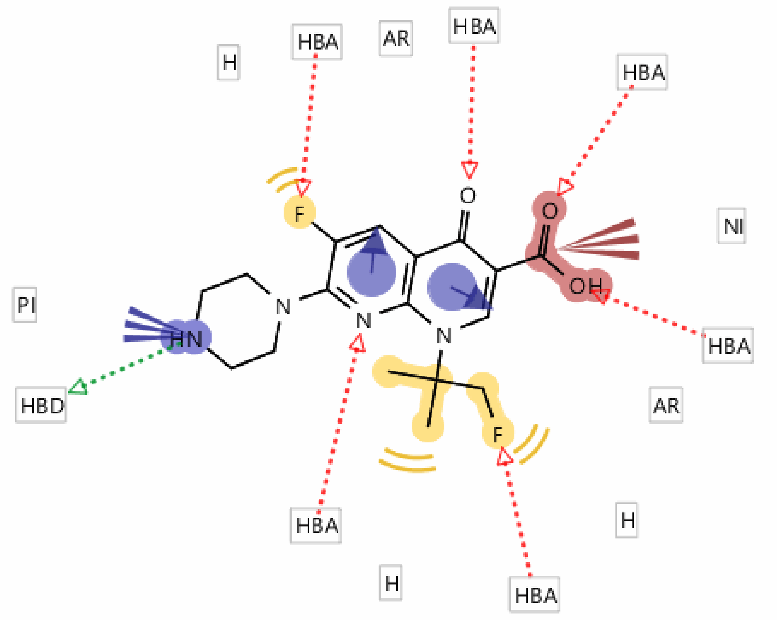
BMY 43261		SHTKKXFERYDICJ-UHFFFAOYSA-N	InChI=1S/C17H18F4N4O3/c18-7-17(8-19,9-20)25-6-11(16(27)28)13(26)10-5-12(21)15(23-14(10)25)24-3-1-22-2-4-24/h5-6,22H,1-4,7-9H2,(H,27,28)	FCC(N1C=C(C(C2=CC(F)=C(N=C21)N3CCNCC3)=O)C(O)=O)(CF)CF	DNA gyrase and topoisomerase IV inhibitor	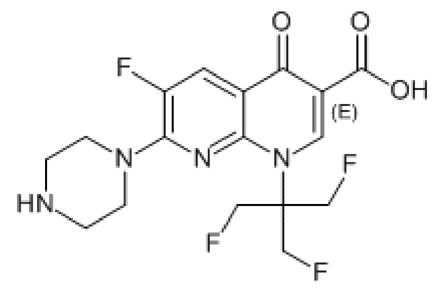	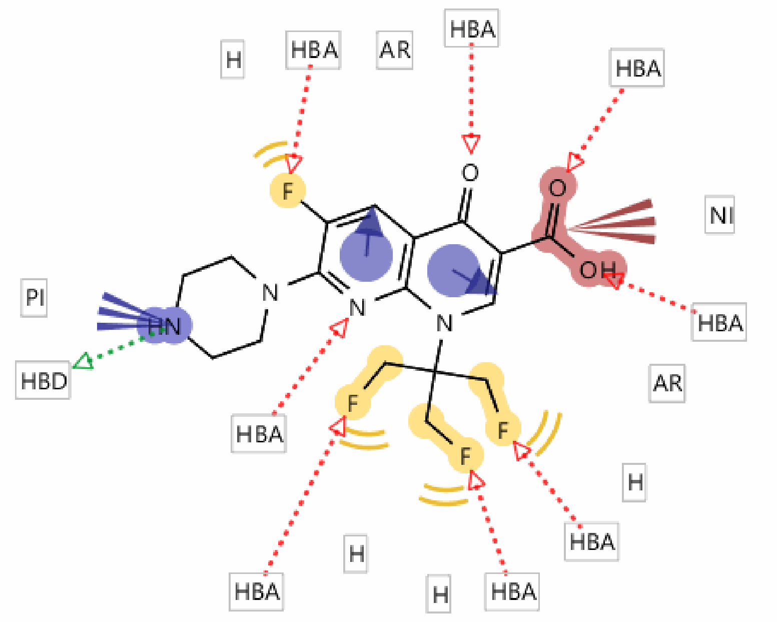
BMY 43748		SKYPQEVVXGJKIJ-NSHDSACASA-N	InChI=1S/C20H17F3N4O3/c1-9-15-17(28)12(20(29)30)8-27(14-3-2-10(21)6-13(14)22)18(15)25-19(16(9)23)26-5-4-11(24)7-26/h2-3,6,8,11H,4-5,7,24H2,1H3,(H,29,30)/t11-/m0/s1	CC1=C(C2=NC(N3CC[C@H](N)C3)=C1F)C(C(C(O)=O)=CN2C4=C(F)C=C(F)C=C4)=O	DNA gyrase and topoisomerase IV inhibitor	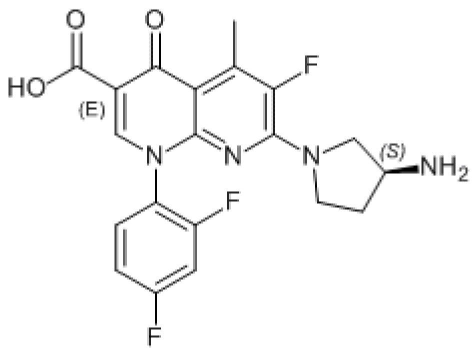	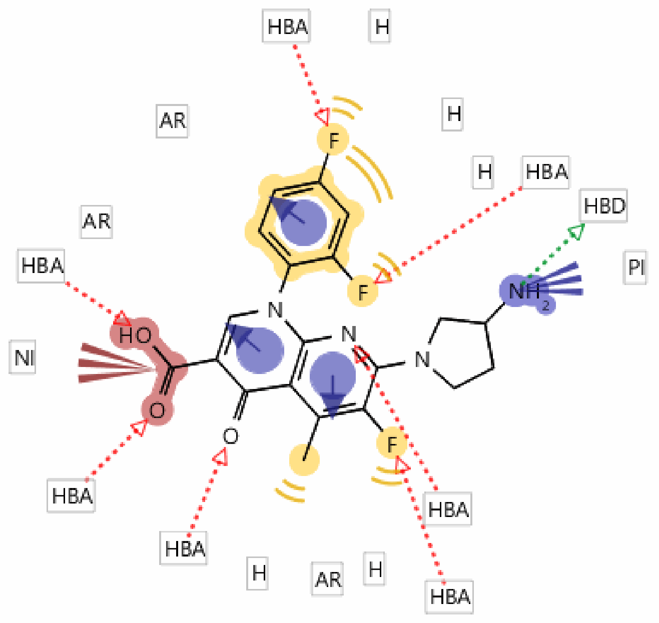
BMY 45243	147740-98-5	NAPVPKCCULWZNJ-QMMMGPOBSA-N	InChI=1S/C17H22FN5O3/c1-17(2,3)23-7-9(16(25)26)13(24)10-12(20)11(18)15(21-14(10)23)22-5-4-8(19)6-22/h7-8H,4-6,19H2,1-3H3,(H2,20,21)(H,25,26)/t8-/m0/s1	CC(N1C=C(C(C2=C(C(F)=C(N=C21)N3CC[C@@H](C3)N)N)=O)C(O)=O)(C)C	DNA gyrase and topoisomerase IV inhibitor	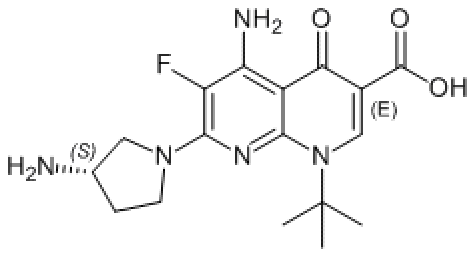	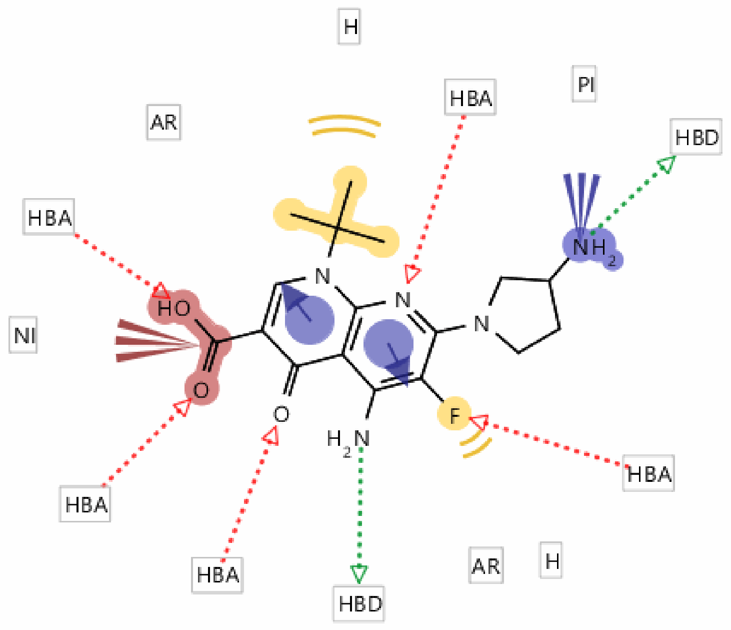
BMY 45706		NPNPCYDYHQBFMR-CHWSQXEVSA-N	InChI=1S/C21H25FN4O4/c1-11(27)7-24-8-13-5-12(24)9-25(13)19-16(22)6-14-17(28)15(20(29)30)10-26(18(14)23-19)21(2,3)4/h6,10,12-13H,5,7-9H2,1-4H3,(H,29,30)/t12-,13-/m1/s1	CC(CN1C[C@H]2C[C@@H]1CN2C3=C(C=C4C(C(C(O)=O)=CN(C4=N3)C(C)(C)C)=O)F)=O	DNA gyrase and topoisomerase IV inhibitor	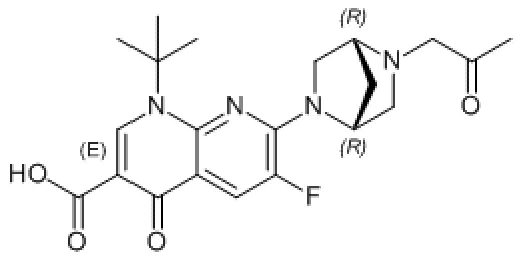	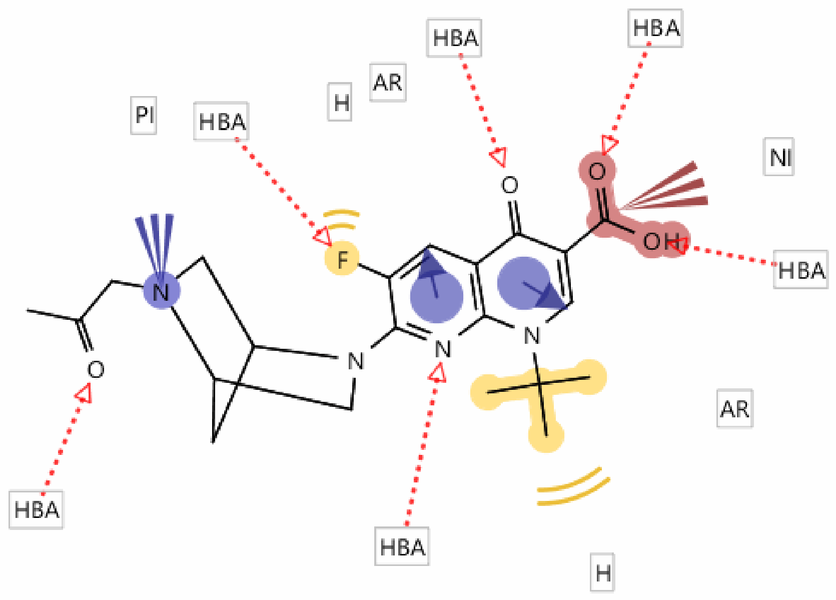
BRN 4913428 (PD 131199)	118829-10-0	WTYZUTHYOYVLAX-RMTNWKGQSA-N	InChI=1S/2C18H17F4N3O3/c2*19-12-5-10-14(25(9-1-2-9)7-11(16(10)26)17(27)28)13(18(20,21)22)15(12)24-4-3-8(23)6-24/h2*5,7-9H,1-4,6,23H2,(H,27,28)/t2*8-/m10/s1	O=C1C2=CC(F)=C(C(C(F)(F)F)=C2N(C3CC3)C=C1C(O)=O)N4CC[C@@H](C4)N.O=C5C6=CC(F)=C(C(C(F)(F)F)=C6N(C7CC7)C=C5C(O)=O)N8CC[C@H](C8)N	DNA gyrase and topoisomerase IV inhibitor	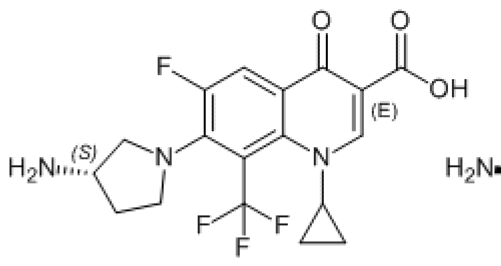 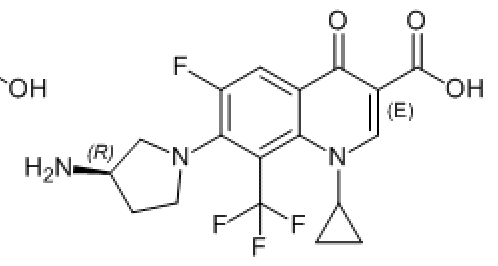	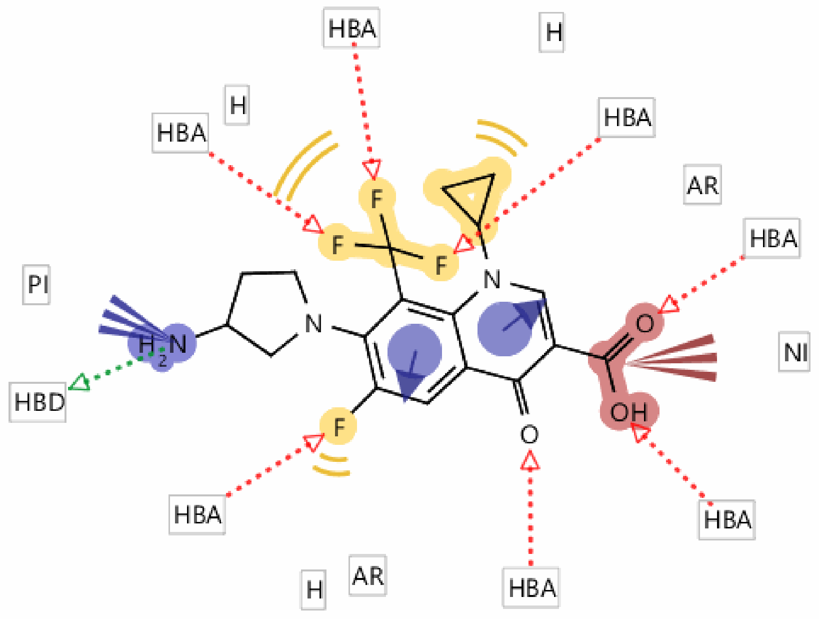

Common name	CAS Reg. No.	InChIKey	InChI	SMILES	Primary target	2D Structure	Pharmacophore[1]
Cadrofloxacin	153808-85-6	QBDBUKJBJJWZMG-VIFPVBQESA-N	InChI=1S/C19H20F3N3O4/c1-9-7-24(5-4-23-9)15-13(20)6-11-14(17(15)29-19(21)22)25(10-2-3-10)8-12(16(11)26)18(27)28/h6,8-10,19,23H,2-5,7H2,1H3,(H,27,28)/t9-/m0/s1	C[C@H]1CN(C2=C(F)C=C(C(C(C(O)=O)=CN3C4CC4)=O)C3=C2OC(F)F)CCN1	DNA gyrase and topoisomerase IV inhibitor	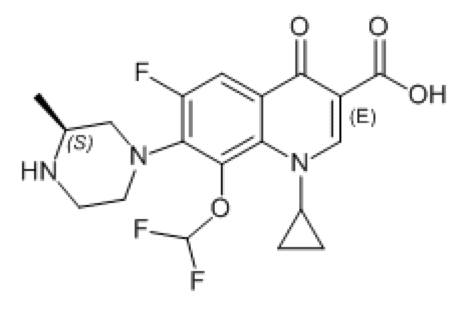	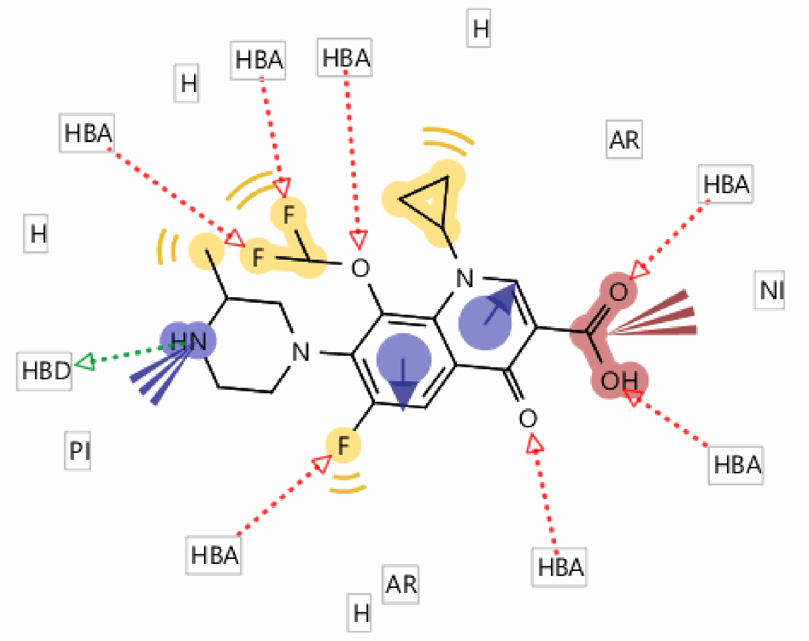
Cetefloxacin	141725-88-4	LTDOHCZUKCZDEQ-BJOHPYRUSA-N	InChI=1S/C20H16F3N3O3/c1-9-15(24)8-25(9)18-6-17-11(5-14(18)23)19(27)12(20(28)29)7-26(17)16-3-2-10(21)4-13(16)22/h2-7,9,15H,8,24H2,1H3,(H,28,29)/t9-,15+/m0/s1	C[C@H]1[C@@H](CN1C2=C(C=C(C3=C2)C(C(C(O)=O)=CN3C4=C(C=C(C=C4)F)F)=O)F)N	DNA gyrase and topoisomerase IV inhibitor	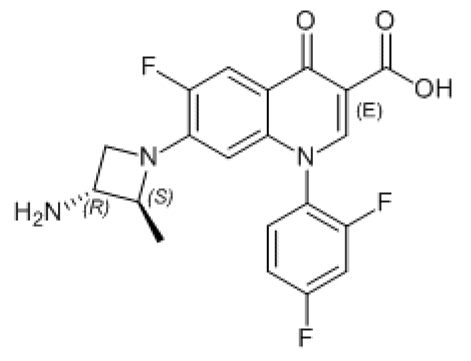	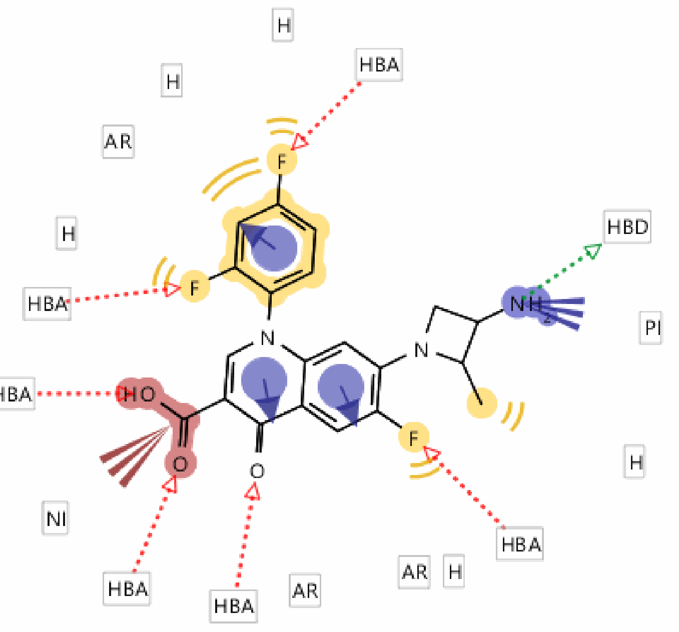
Chinfloxacin		KNLARSZRTBDTIA-ZUZCIYMTSA-N	InChI=1S/C21H22F3N3O4/c22-14-6-12-16(27(11-3-4-11)8-13(18(12)28)20(29)30)19(31-21(23)24)17(14)26-7-10-2-1-5-25-15(10)9-26/h6,8,10-11,15,21,25H,1-5,7,9H2,(H,29,30)/t10-,15+/m0/s1	FC1=C(N2C[C@]3([H])CCCN[C@]3([H])C2)C(OC(F)F)=C4C(C(C(C(O)=O)=CN4C5CC5)=O)=C1	DNA gyrase and topoisomerase IV inhibitor	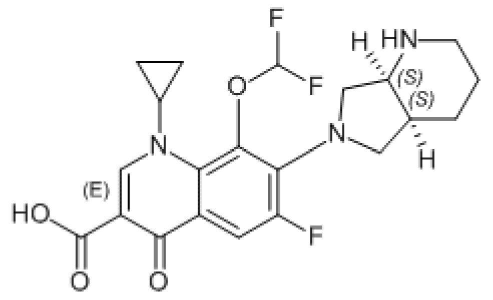	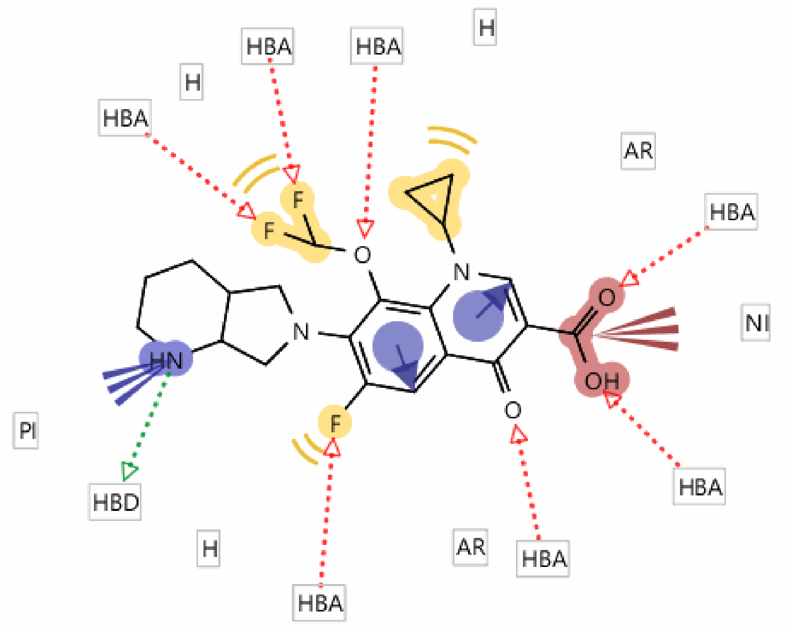
CI 990 (PD 131112)		ZVZRDFTXNAZBOX-UWVGGRQHSA-N	InChI=1S/C19H22FN5O4/c1-9(21)18(27)22-10-4-5-24(7-10)17-14(20)6-12-15(26)13(19(28)29)8-25(11-2-3-11)16(12)23-17/h6,8-11H,2-5,7,21H2,1H3,(H,22,27)(H,28,29)/t9-,10-/m0/s1	C[C@@H](C(N[C@H]1CCN(C1)C2=C(C=C(C(C(C(O)=O)=CN3C4CC4)=O)C3=N2)F)=O)N	DNA gyrase and topoisomerase IV inhibitor	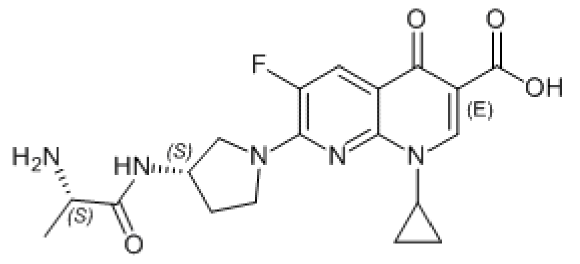	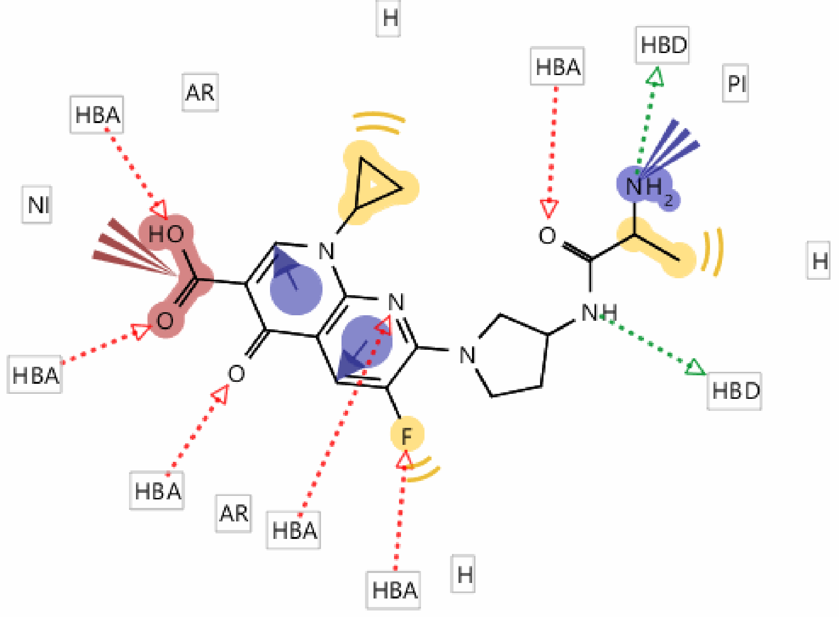
Ciprofloxacin	85721-33-1	MYSWGUAQZAJSOK-UHFFFAOYSA-N	InChI=1S/C17H18FN3O3/c18-13-7-11-14(8-15(13)20-5-3-19-4-6-20)21(10-1-2-10)9-12(16(11)22)17(23)24/h7-10,19H,1-6H2,(H,23,24)	FC1=C(C=C2C(C(C(C(O)=O)=CN2C3CC3)=O)=C1)N4CCNCC4	DNA gyrase and topoisomerase IV inhibitor	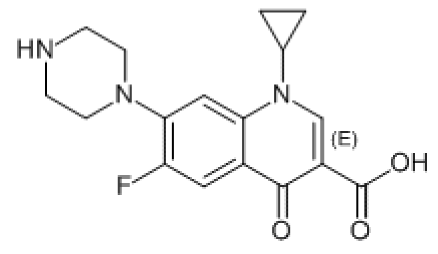	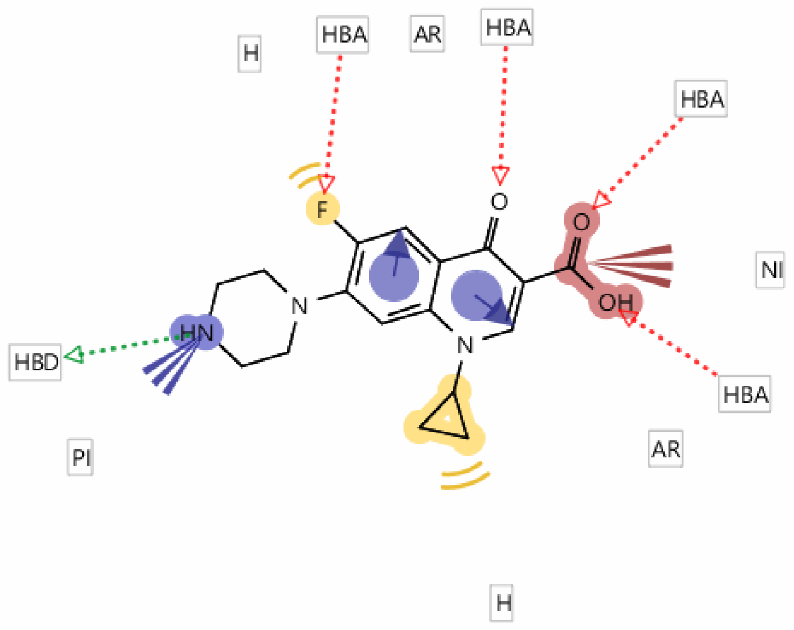
Clinafloxacin	105956-97-6	JTWNENGOYQRQAZ-RMTNWKGQSA-N	InChI=1S/2C17H17ClFN3O3/c2*18-13-14-10(5-12(19)15(13)21-4-3-8(20)6-21)16(23)11(17(24)25)7-22(14)9-1-2-9/h2*5,7-9H,1-4,6,20H2,(H,24,25)/t2*8-/m10/s1	ClC1=C2C(C(C(C(O)=O)=CN2C3CC3)=O)=CC(F)=C1N4CC[C@@H](N)C4.ClC5=C6C(C(C(C(O)=O)=CN6C7CC7)=O)=CC(F)=C5N8CC[C@H](N)C8	DNA gyrase and topoisomerase IV inhibitor	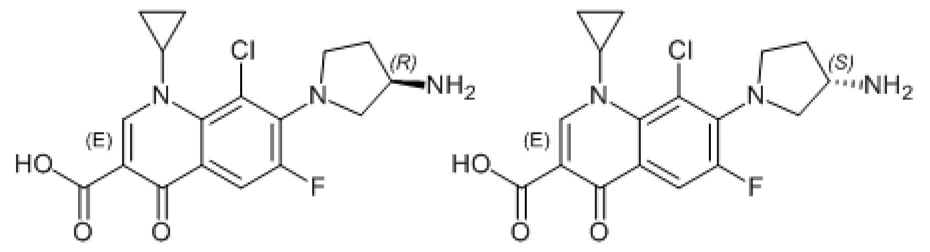	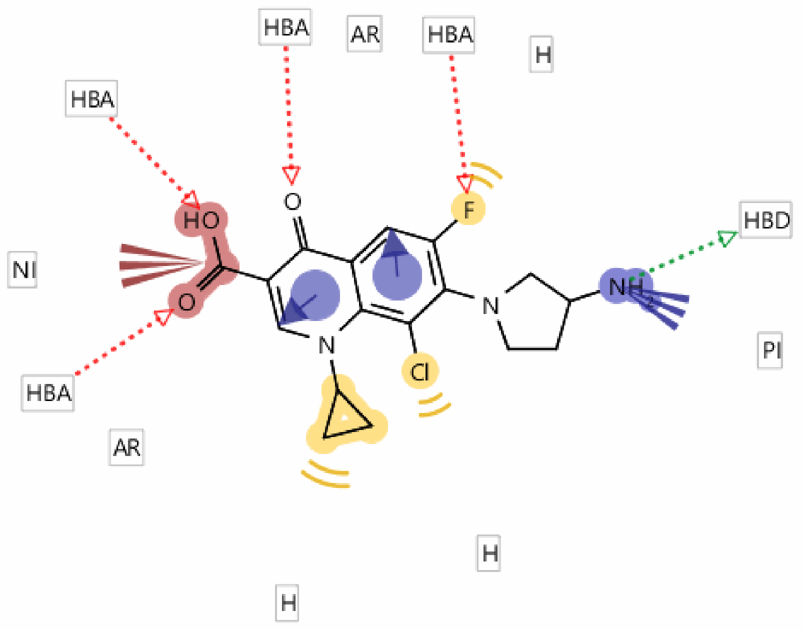

Common name	CAS Reg. No.	InChIKey	InChI	SMILES	Primary target	2D Structure	Pharmacophore[1]
CP 100964		XBFLDWQAWQYCBO-NSHDSACASA-N	InChI=1S/C22H15FN2O4/c1-11-10-29-21-18(13-4-5-17-12(7-13)3-2-6-24-17)16(23)8-14-19(21)25(11)9-15(20(14)26)22(27)28/h2-9,11H,10H2,1H3,(H,27,28)/t11-/m0/s1	C[C@H]1COC2=C3C(C(C(C(O)=O)=CN13)=O)=CC(F)=C2C4=CC=C5C(C=CC=N5)=C4	DNA gyrase and topoisomerase IV inhibitor	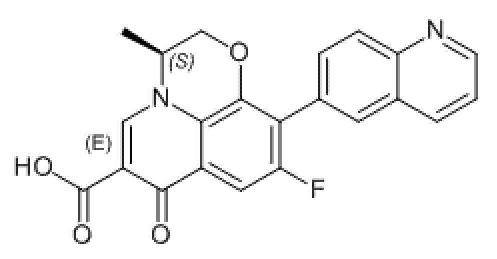	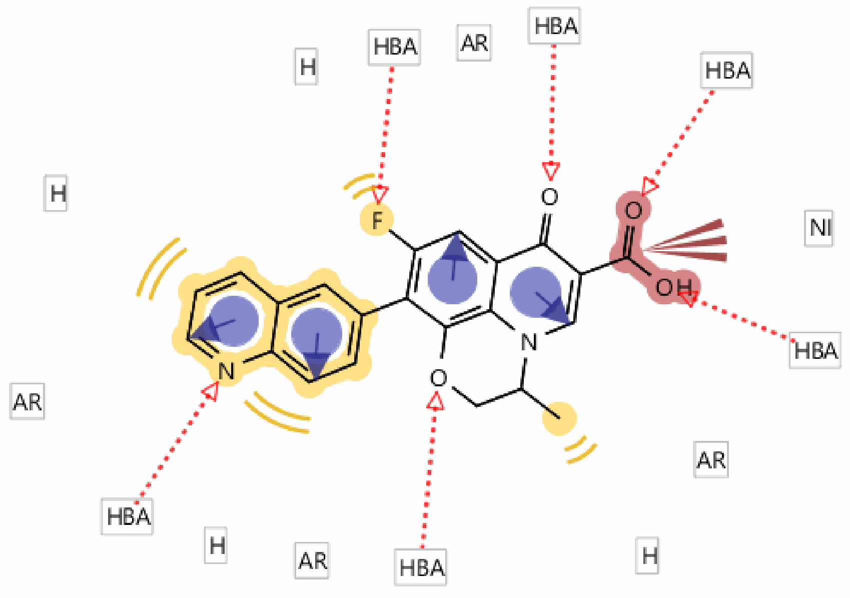
CP 104830	110236-78-7	LMOANZHDQSNEII-UHFFFAOYSA-N	InChI=1S/C17H18F2N4O3/c18-11-13(20)10-14(12(19)15(11)22-5-3-21-4-6-22)23(8-1-2-8)7-9(16(10)24)17(25)26/h7-8,21H,1-6,20H2,(H,25,26)	O=C(C1=C2C(F)=C(N3CCNCC3)C(F)=C1N)C(C(O)=O)=CN2C4CC4	DNA gyrase and topoisomerase IV inhibitor	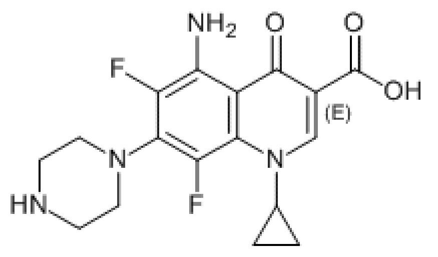	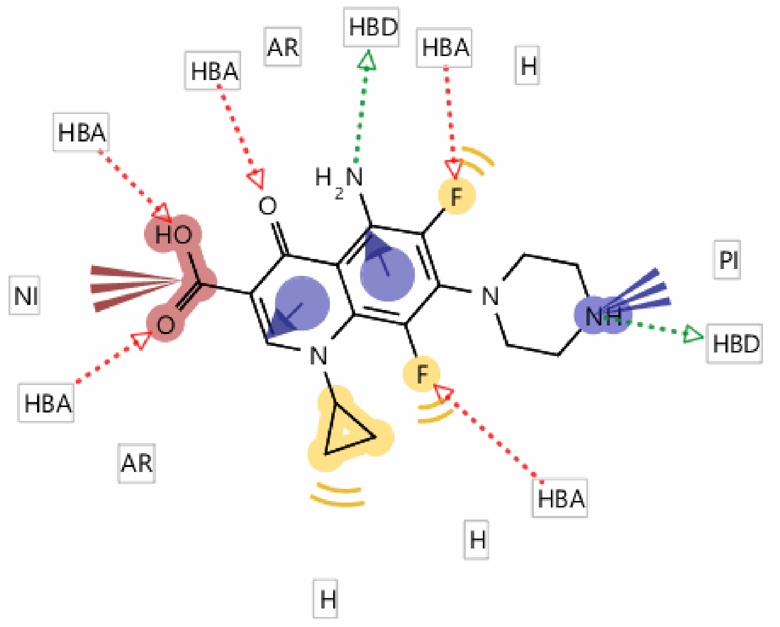
CP 105532 (PD 125275)	103460-89-5	MSUMUQMBYGRNHT-IGXPJQGBSA-N	InChI=1S/2C18H19F2N3O3/c2*1-9-7-22(5-4-21-9)16-13(19)6-11-15(14(16)20)23(10-2-3-10)8-12(17(11)24)18(25)26/h2*6,8-10,21H,2-5,7H2,1H3,(H,25,26)/t2*9-/m10/s1	C[C@@H]1CN(CCN1)C2=C(C=C3C(N(C=C(C3=O)C(O)=O)C4CC4)=C2F)F.C[C@H]5CN(CCN5)C6=C(C=C7C(N(C=C(C7=O)C(O)=O)C8CC8)=C6F)F	DNA gyrase and topoisomerase IV inhibitor	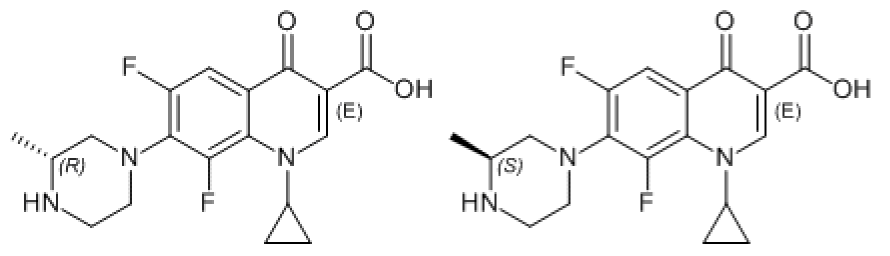	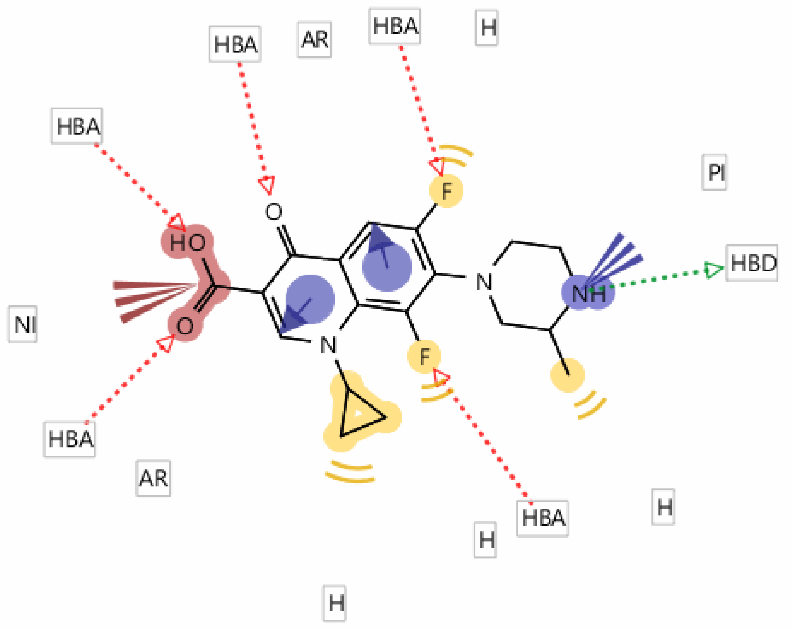
CP 115953	136440-70-5	ISQTYKAFNUVODK-UHFFFAOYSA-N	InChI=1S/C19H13F2NO4/c20-14-7-12-17(16(21)15(14)9-1-5-11(23)6-2-9)22(10-3-4-10)8-13(18(12)24)19(25)26/h1-2,5-8,10,23H,3-4H2,(H,25,26)	FC1=C(C(F)=C2C(C(C(C(O)=O)=CN2C3CC3)=O)=C1)C4=CC=C(O)C=C4	DNA gyrase and topoisomerase IV inhibitor	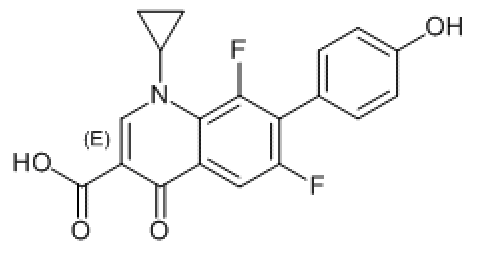	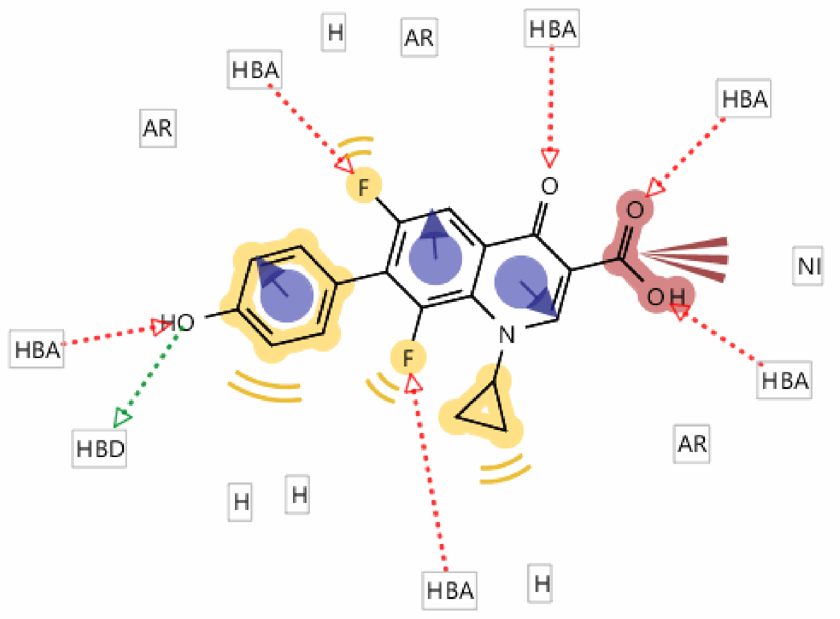
CP 115955	141874-19-3	DISCUNPKVHOSCE-UHFFFAOYSA-N	InChI=1S/C19H14FNO4/c20-16-7-14-17(8-13(16)10-1-5-12(22)6-2-10)21(11-3-4-11)9-15(18(14)23)19(24)25/h1-2,5-9,11,22H,3-4H2,(H,24,25)	FC1=CC2=C(C=C1C3=CC=C(C=C3)O)N(C4CC4)C=C(C(O)=O)C2=O	DNA gyrase and topoisomerase IV inhibitor	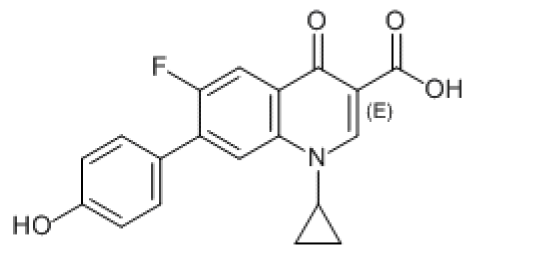	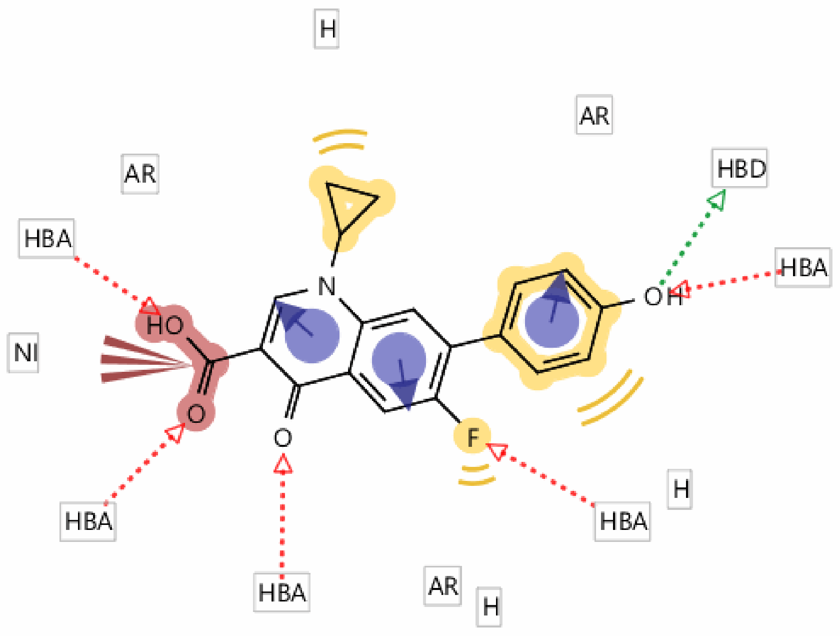
CP 135803	110871-85-7	UJUUIEXGSSKCSB-RMTNWKGQSA-N	InChI=1S/2C18H20F2N4O3/c2*1-8-6-23(5-4-22-8)16-12(19)14(21)11-15(13(16)20)24(9-2-3-9)7-10(17(11)25)18(26)27/h2*7-9,22H,2-6,21H2,1H3,(H,26,27)/t2*8-/m10/s1	C[C@@H]1CN(C2=C(F)C3=C(C(C(C(O)=O)=CN3C4CC4)=O)C(N)=C2F)CCN1.C[C@H]5CN(C6=C(F)C7=C(C(C(C(O)=O)=CN7C8CC8)=O)C(N)=C6F)CCN5	DNA gyrase and topoisomerase IV inhibitor	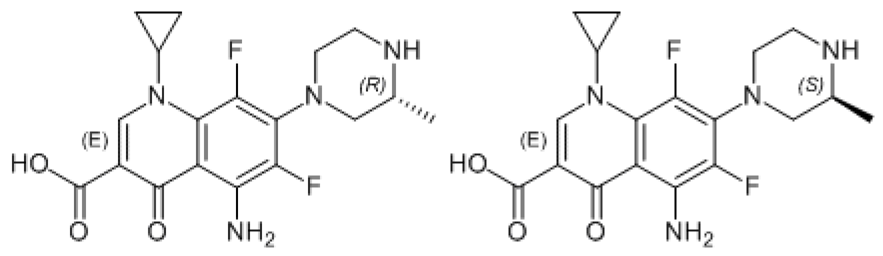	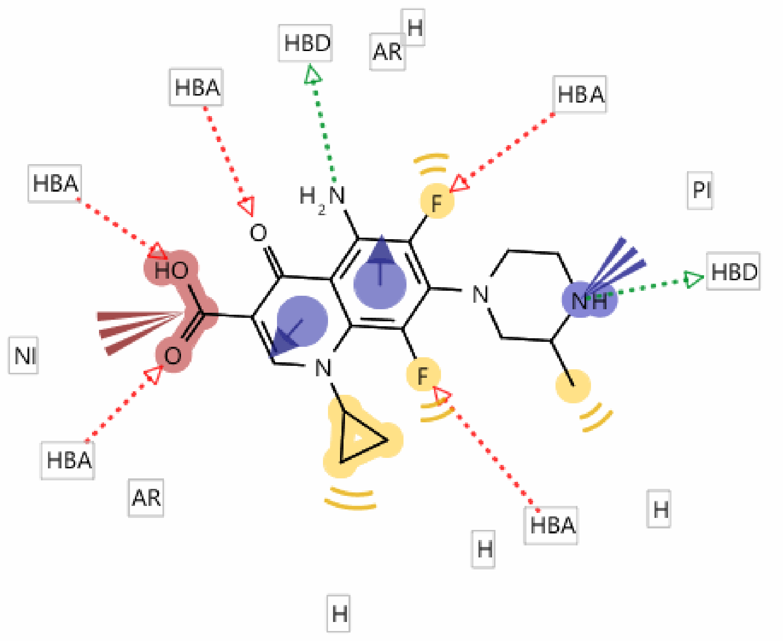

Common name	CAS Reg. No.	InChIKey	InChI	SMILES	Primary target	2D Structure	Pharmacophore[1]
CP 67015	100325-51-7	AVTJRMCCKOBMQP-UHFFFAOYSA-N	InChI=1S/C17H12F2N2O3/c1-2-21-8-11(17(23)24)16(22)10-7-12(18)13(14(19)15(10)21)9-3-5-20-6-4-9/h3-8H,2H2,1H3,(H,23,24)	CCN1C=C(C(C2=CC(F)=C(C3=CC=NC=C3)C(F)=C21)=O)C(O)=O	DNA gyrase and topoisomerase IV inhibitor	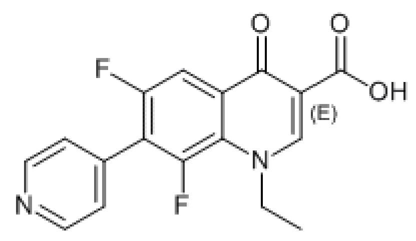	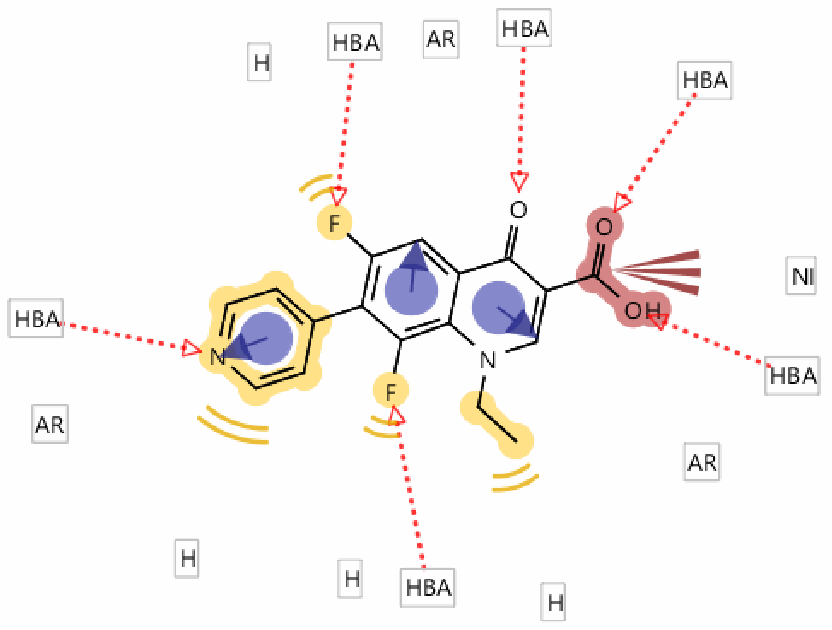
CP 67804	103978-08-1	MYERGISJXHBTKE-UHFFFAOYSA-N	InChI=1S/C18H13F2NO4/c1-2-21-8-12(18(24)25)17(23)11-7-13(19)14(15(20)16(11)21)9-3-5-10(22)6-4-9/h3-8,22H,2H2,1H3,(H,24,25)	CCN1C=C(C(C2=CC(F)=C(C3=CC=C(C=C3)O)C(F)=C21)=O)C(O)=O	DNA gyrase and topoisomerase IV inhibitor	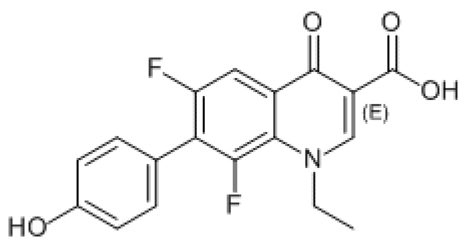	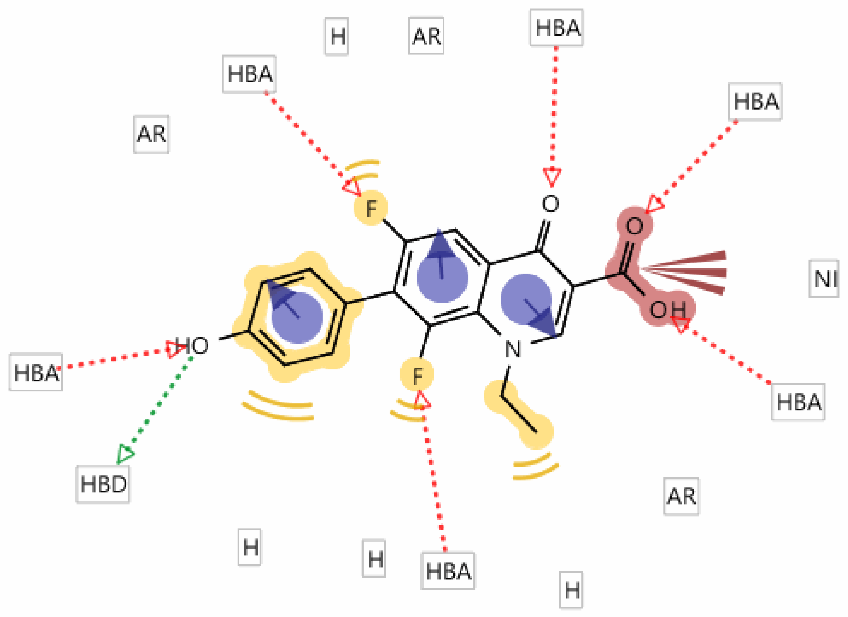
CP 74667	108461-05-8	ALRZZABRVIYCNY-BETUJISGSA-N	InChI=1S/C20H22FN3O3/c1-22-12-4-5-13(22)9-23(8-12)18-7-17-14(6-16(18)21)19(25)15(20(26)27)10-24(17)11-2-3-11/h6-7,10-13H,2-5,8-9H2,1H3,(H,26,27)/t12-,13+	CN1[C@H]2CC[C@@H]1CN(C2)C3=C(C=C(C4=C3)C(C(C(O)=O)=CN4C5CC5)=O)F	DNA gyrase and topoisomerase IV inhibitor	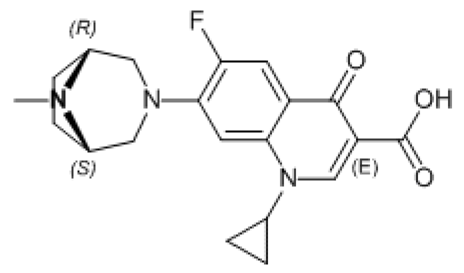	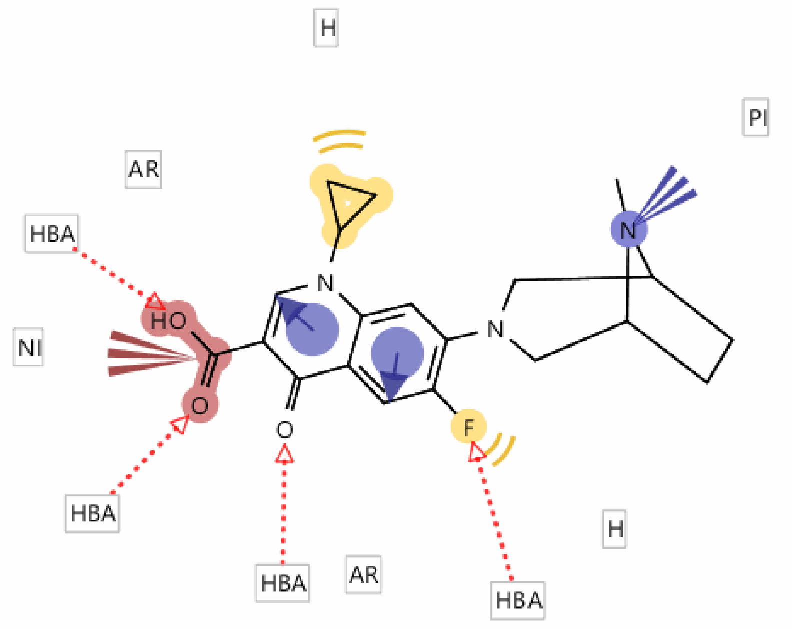
CP 92121		OGHURWZVYMSQSB-VIFPVBQESA-N	InChI=1S/C18H13FN2O4/c1-9-8-25-17-14(10-2-4-20-5-3-10)13(19)6-11-15(17)21(9)7-12(16(11)22)18(23)24/h2-7,9H,8H2,1H3,(H,23,24)/t9-/m0/s1	C[C@H]1COC2=C3C(C(C(C(O)=O)=CN13)=O)=CC(F)=C2C4=CC=NC=C4	DNA gyrase and topoisomerase IV inhibitor	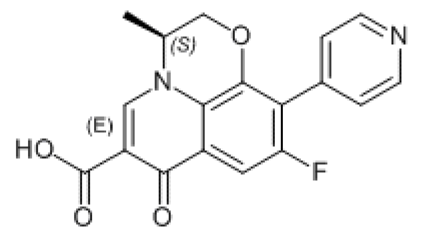	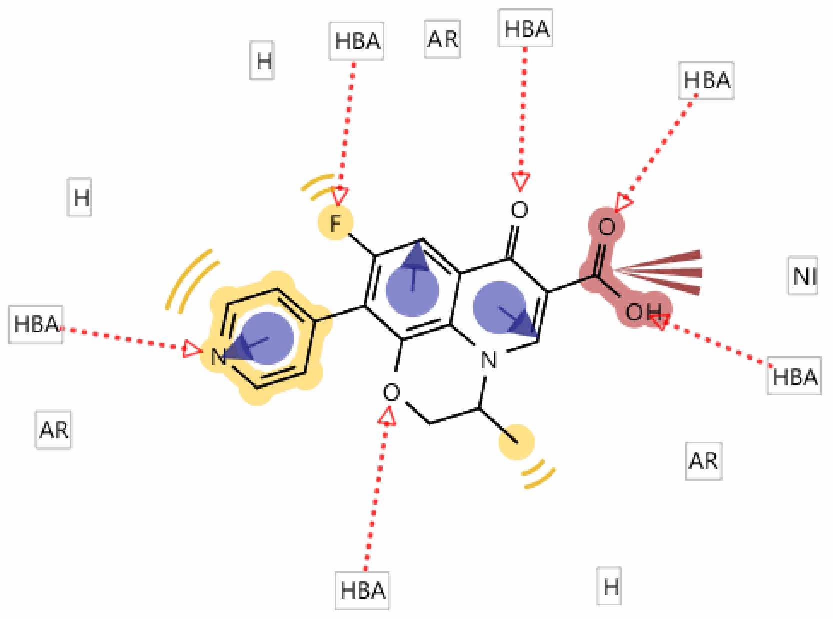
CP 99433	147059-71-0	HPQJHUNCWWNOJL-IWIIMEHWSA-N	InChI=1S/C17H17FN4O3/c18-12-3-8-14(23)11(17(24)25)6-22(7-1-2-7)15(8)20-16(12)21-4-9-10(5-21)13(9)19/h3,6-7,9-10,13H,1-2,4-5,19H2,(H,24,25)/t9-,10+,13+	FC1=C(N=C2C(C(C(C(O)=O)=CN2C3CC3)=O)=C1)N4C[C@@]([C@H]5N)([H])[C@]5(C4)[H]	DNA gyrase and topoisomerase IV inhibitor	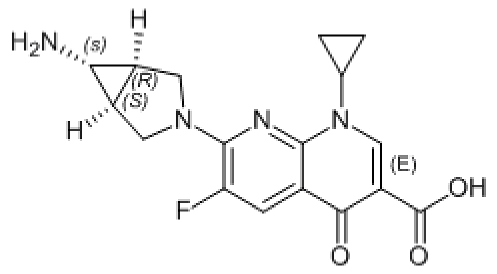	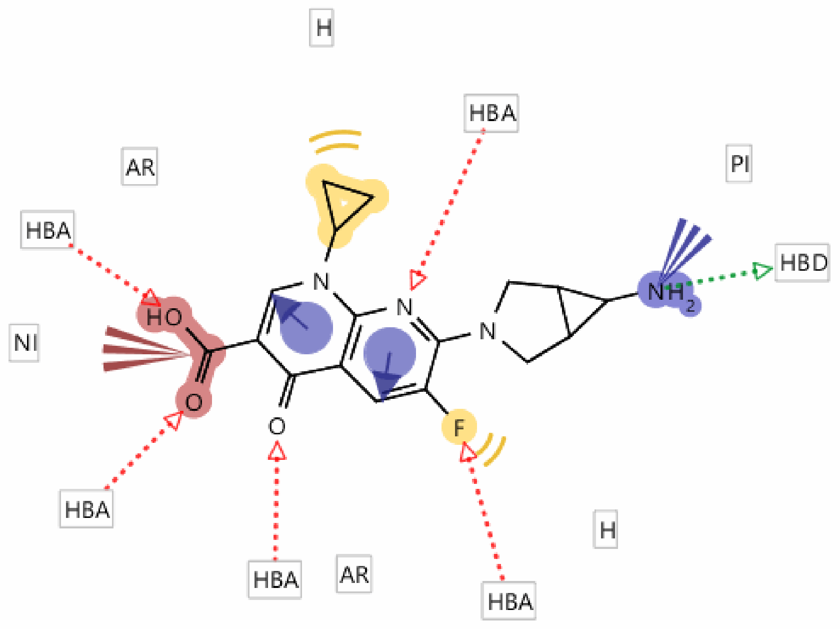
Danofloxacin	112398-08-0	QMLVECGLEOSESV-RYUDHWBXSA-N	InChI=1S/C19H20FN3O3/c1-21-7-12-4-11(21)8-22(12)17-6-16-13(5-15(17)20)18(24)14(19(25)26)9-23(16)10-2-3-10/h5-6,9-12H,2-4,7-8H2,1H3,(H,25,26)/t11-,12-/m0/s1	CN1C[C@@H]2C[C@H]1CN2C3=C(C=C(C4=C3)C(C(C(O)=O)=CN4C5CC5)=O)F	DNA gyrase and topoisomerase IV inhibitor	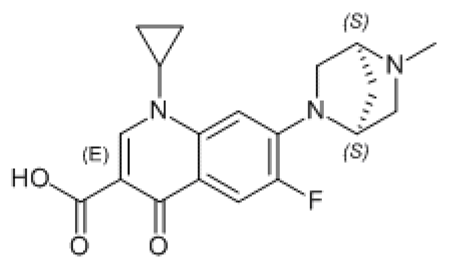	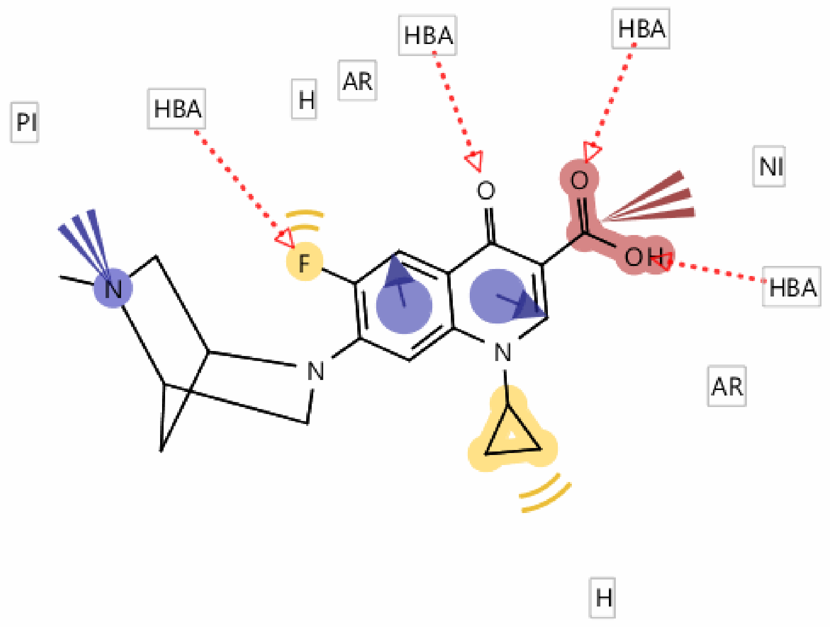

Common name	CAS Reg. No.	InChIKey	InChI	SMILES	Primary target	2D Structure	Pharmacophore[1]
DC 159a free base		GBLYBAYNQGZGCK-KBARZSOFSA-N	InChI=1S/C21H23F2N3O4/c1-20(24)8-25(9-21(20)3-4-21)16-13(23)5-10-15(18(16)30-2)26(14-6-12(14)22)7-11(17(10)27)19(28)29/h5,7,12,14H,3-4,6,8-9,24H2,1-2H3,(H,28,29)/t12-,14+,20+/m0/s1	C[C@@]1(N)CN(C2=C(F)C=C(C(C(C(O)=O)=CN3[C@@H]4C[C@@H]4F)=O)C3=C2OC)CC15CC5	DNA gyrase and topoisomerase IV inhibitor	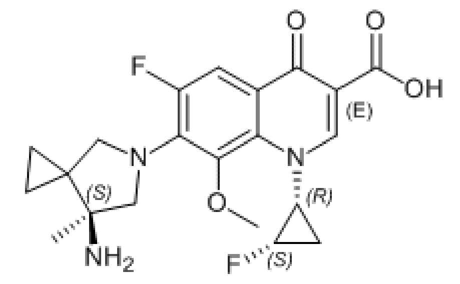	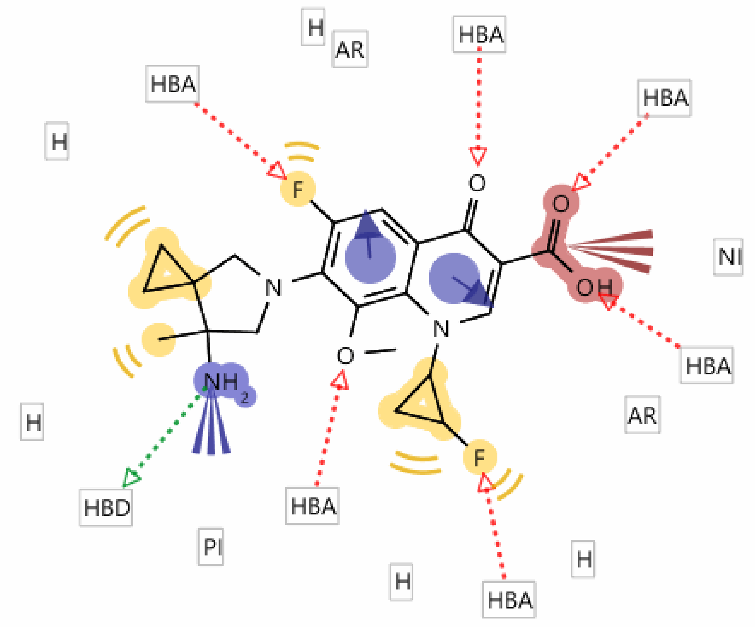
Delafloxacin	189279-58-1	DYDCPNMLZGFQTM-UHFFFAOYSA-N	InChI=1S/C18H12ClF3N4O4/c19-12-13-7(1-9(20)14(12)25-3-6(27)4-25)15(28)8(18(29)30)5-26(13)17-11(22)2-10(21)16(23)24-17/h1-2,5-6,27H,3-4H2,(H2,23,24)(H,29,30)	ClC1=C(N(C2=NC(N)=C(F)C=C2F)C=C(C(O)=O)C3=O)C3=CC(F)=C1N4CC(O)C4	DNA gyrase and topoisomerase IV inhibitor	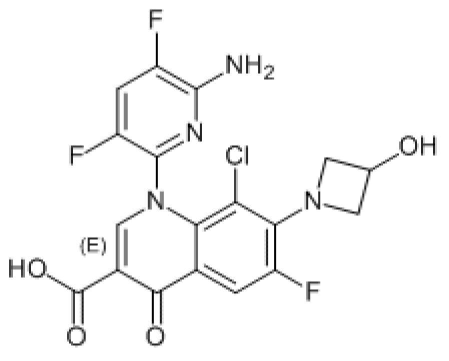	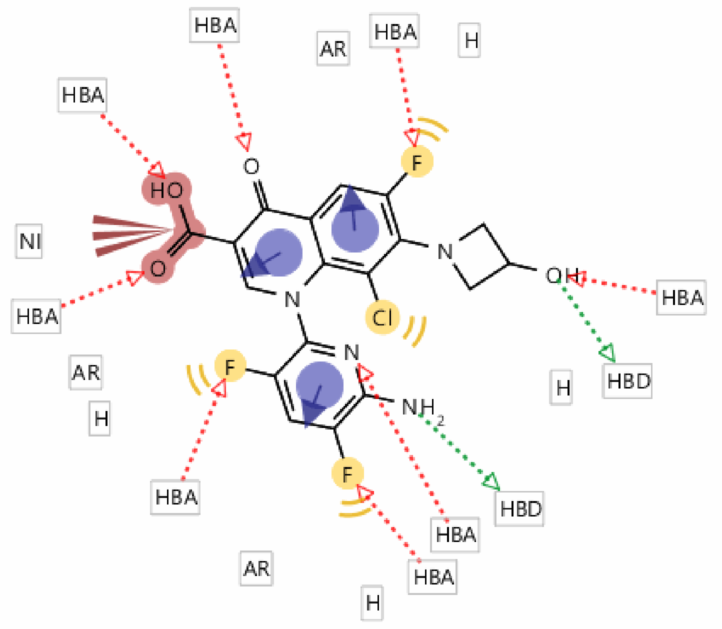
Desfluorociprofloxacin (SQ 4004)	93107-11-0	IWQJEAWVHIBCDP-UHFFFAOYSA-N	InChI=1S/C17H19N3O3/c21-16-13-4-3-12(19-7-5-18-6-8-19)9-15(13)20(11-1-2-11)10-14(16)17(22)23/h3-4,9-11,18H,1-2,5-8H2,(H,22,23)	O=C(C(C(O)=O)=CN1C2CC2)C3=C1C=C(N4CCNCC4)C=C3	DNA gyrase and topoisomerase IV inhibitor	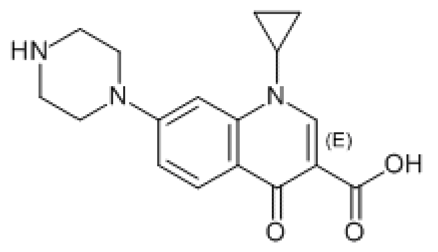	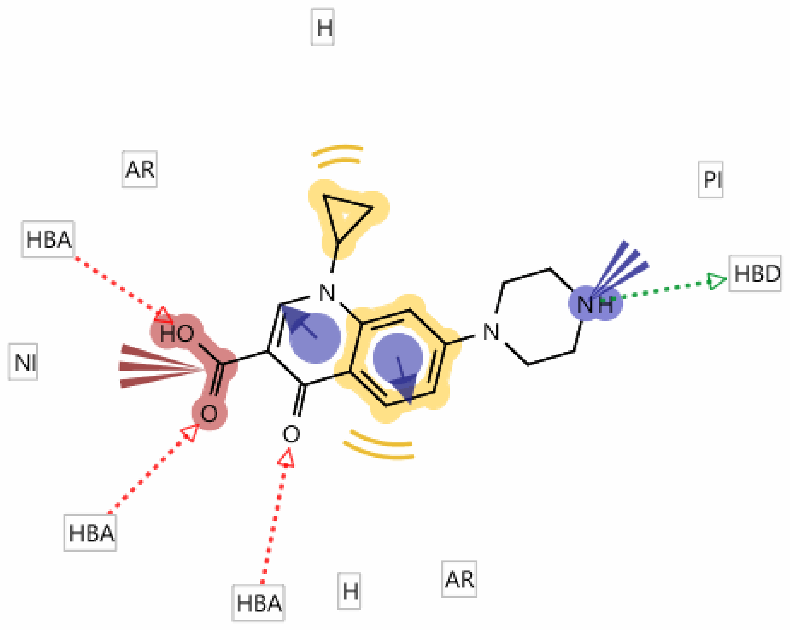
Difloxacin	98106-17-3	NOCJXYPHIIZEHN-UHFFFAOYSA-N	InChI=1S/C21H19F2N3O3/c1-24-6-8-25(9-7-24)19-11-18-15(10-17(19)23)20(27)16(21(28)29)12-26(18)14-4-2-13(22)3-5-14/h2-5,10-12H,6-9H2,1H3,(H,28,29)	CN1CCN(CC1)C2=C(C=C(C3=C2)C(C(C(O)=O)=CN3C4=CC=C(C=C4)F)=O)F	DNA gyrase and topoisomerase IV inhibitor	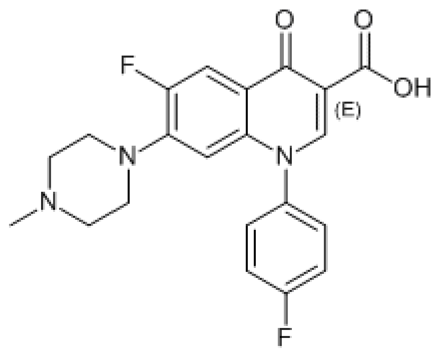	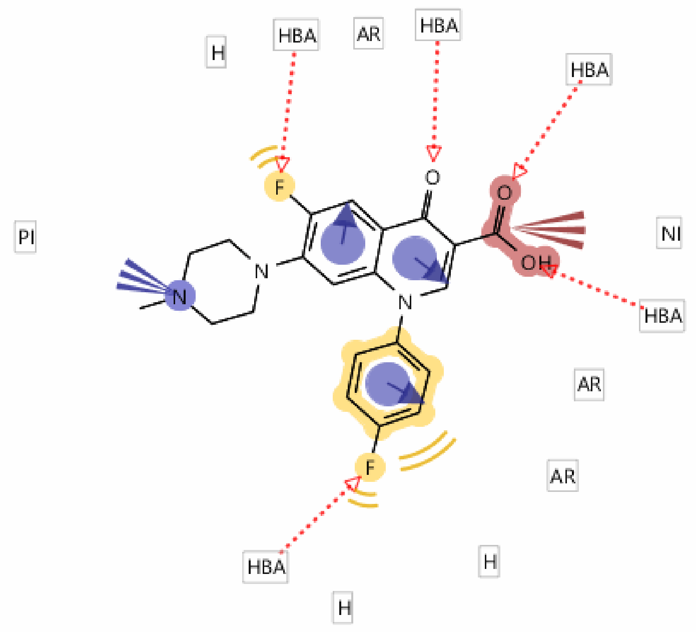
DJ 6783	73101-87-8	IRRYFLAZTLGICF-UHFFFAOYSA-N	InChI=1S/C13H10N2O4/c1-2-15-6-8(13(17)18)11(16)7-5-10-9(3-4-19-10)14-12(7)15/h3-6H,2H2,1H3,(H,17,18)	CCN1C=C(C(C2=CC3=C(C=CO3)N=C21)=O)C(O)=O	DNA gyrase and topoisomerase IV inhibitor	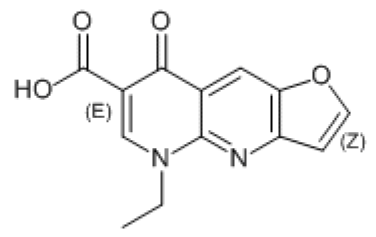	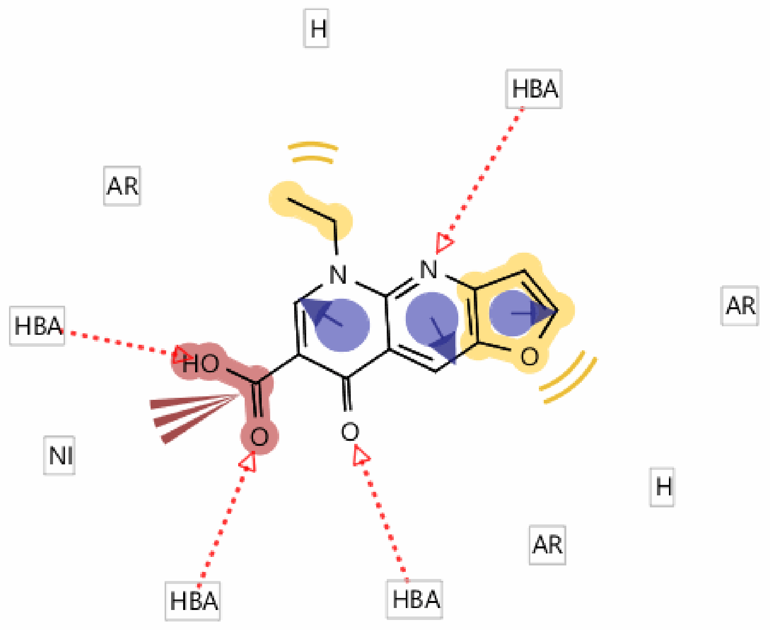
DK 507k		JYRHGXXQSIPDDP-IACUBPJLSA-N	InChI=1S/C20H21F2N3O4/c1-29-18-15-9(17(26)10(19(27)28)6-25(15)13-5-11(13)21)4-12(22)16(18)24-7-14(23)20(8-24)2-3-20/h4,6,11,13-14H,2-3,5,7-8,23H2,1H3,(H,27,28)/t11-,13+,14+/m0/s1	COC1=C2C(C(C(C(O)=O)=CN2[C@@H]3C[C@@H]3F)=O)=CC(F)=C1N4C[C@@H](N)C5(C4)CC5	DNA gyrase and topoisomerase IV inhibitor	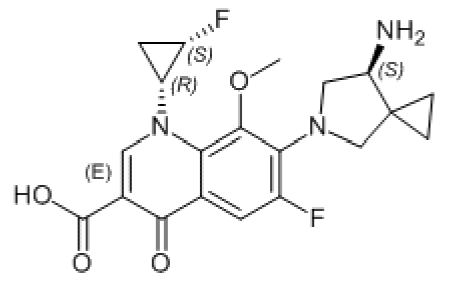	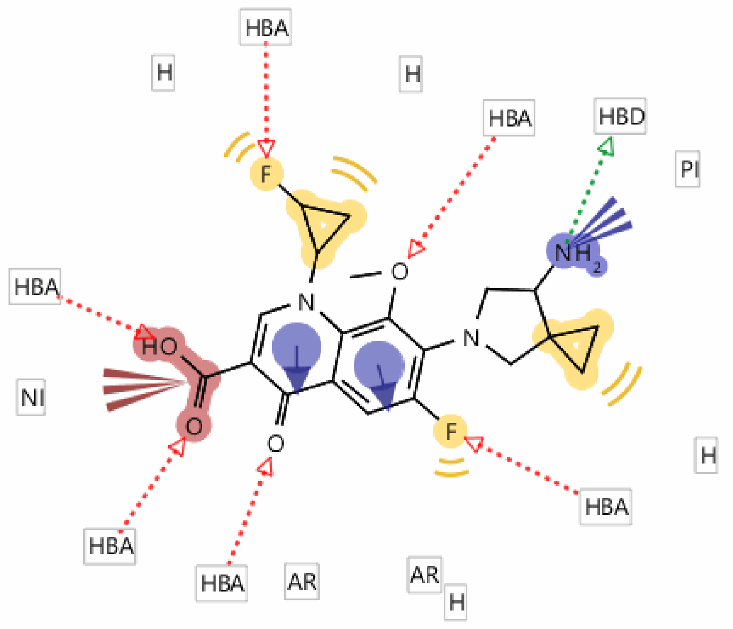

Common name	CAS Reg. No.	InChIKey	InChI	SMILES	Primary target	2D Structure	Pharmacophore[1]
DN 9494		PBPGWYQXPMJMIG-UHFFFAOYSA-N	InChI=1S/C18H18FN3O4/c1-10-9-26-17-14-11(16(23)12(18(24)25)8-22(10)14)7-13(19)15(17)21-5-3-20(2)4-6-21/h7-8H,1,3-6,9H2,2H3,(H,24,25)	C=C1COC2=C3C(C(C(C(O)=O)=CN13)=O)=CC(F)=C2N4CCN(CC4)C	DNA gyrase and topoisomerase IV inhibitor	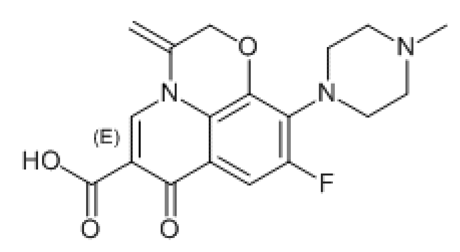	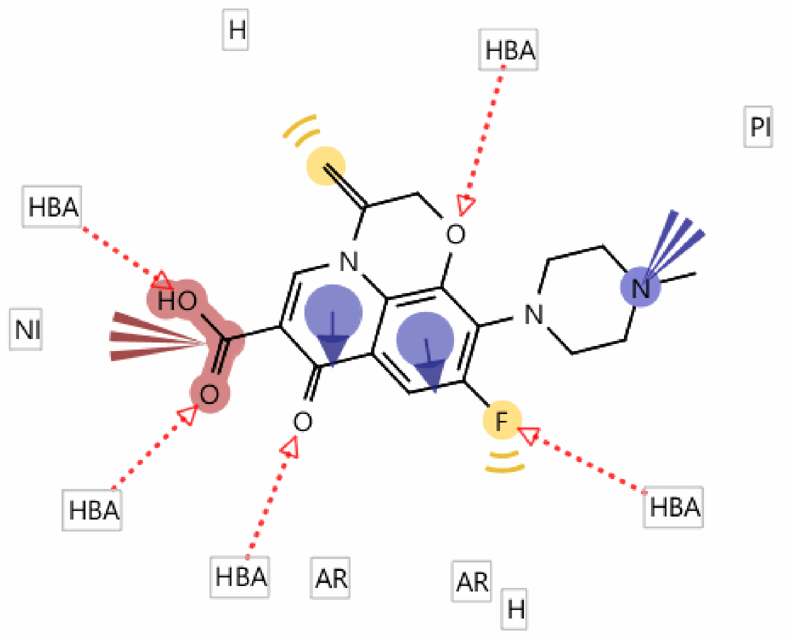
Droxacin	35067-47-1	IIRVYWCKYUQJCL-UHFFFAOYSA-N	InChI=1S/C14H13NO4/c1-2-15-7-10(14(17)18)13(16)9-6-12-8(3-4-19-12)5-11(9)15/h5-7H,2-4H2,1H3,(H,17,18)	CCN1C=C(C(O)=O)C(C2=CC3=C(C=C21)CCO3)=O	DNA gyrase and topoisomerase IV inhibitor	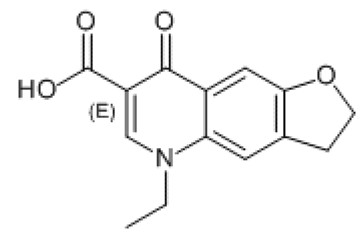	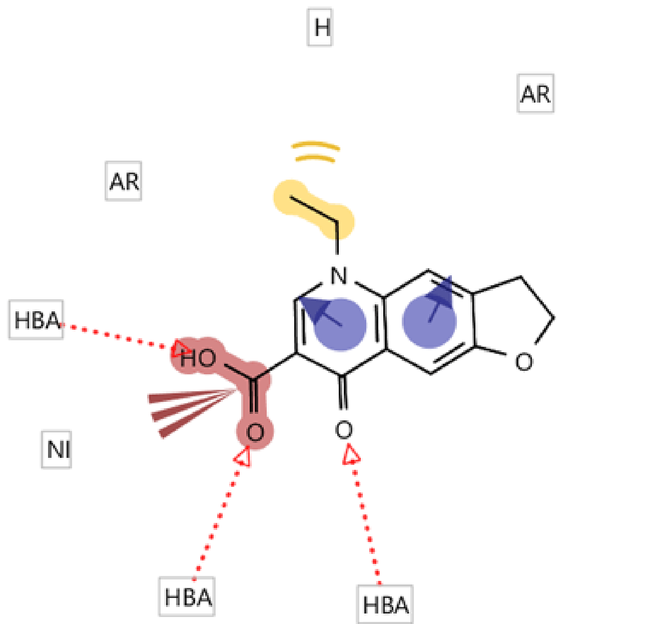
DS 8587 free base	1037366-13-4	JMXNWTLVUUGTDR-UTQQSMSJSA-N	InChI=1S/C21H22F3N3O3/c1-10-16-11(18(28)12(19(29)30)7-27(16)15-6-13(15)22)5-14(23)17(10)26-8-20(24)3-2-4-21(20,25)9-26/h5,7,13,15H,2-4,6,8-9,25H2,1H3,(H,29,30)/t13-,15+,20-,21+/m0/s1	CC1=C2C(C(C(C(O)=O)=CN2[C@@H]3C[C@@H]3F)=O)=CC(F)=C1N4C[C@]5(CCC[C@]5(C4)N)F	DNA gyrase and topoisomerase IV inhibitor	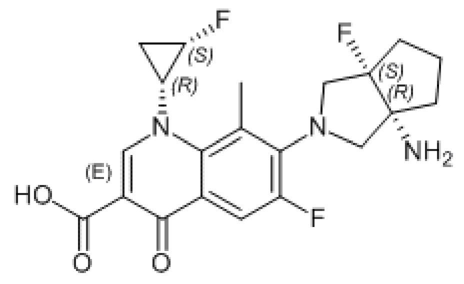	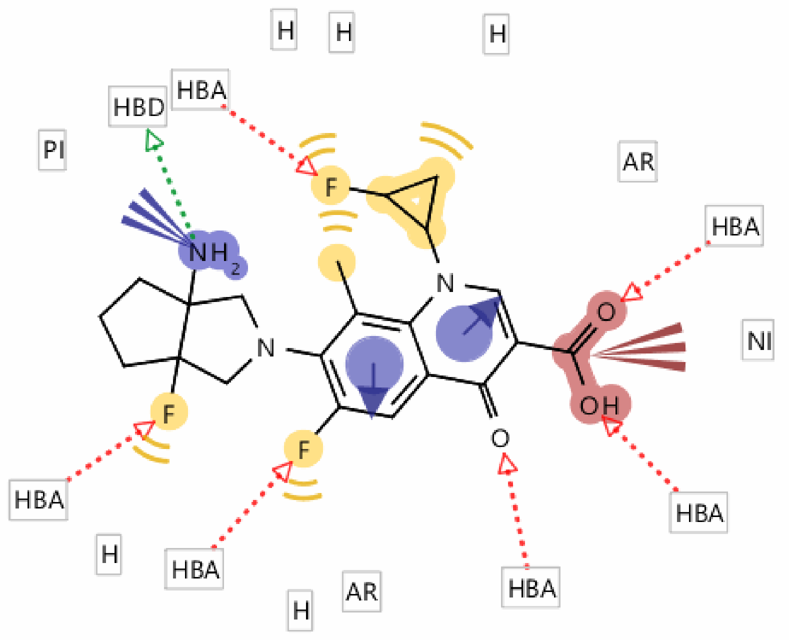
DU 6611		RHXWDMANWNEVNN-VIRWGQHYSA-N	InChI=1S/C17H16ClF2N3O3/c18-13-14-8(3-11(20)15(13)22-2-1-7(21)5-22)16(24)9(17(25)26)6-23(14)12-4-10(12)19/h3,6-7,10,12H,1-2,4-5,21H2,(H,25,26)/t7-,10-,12+/m0/s1	N[C@@H]1CN(C2=C(F)C=C3C(N([C@@H]4C[C@@H]4F)C=C(C(O)=O)C3=O)=C2Cl)CC1	DNA gyrase and topoisomerase IV inhibitor	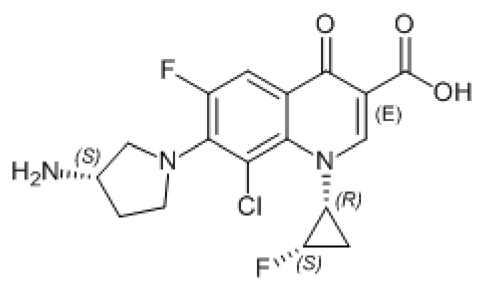	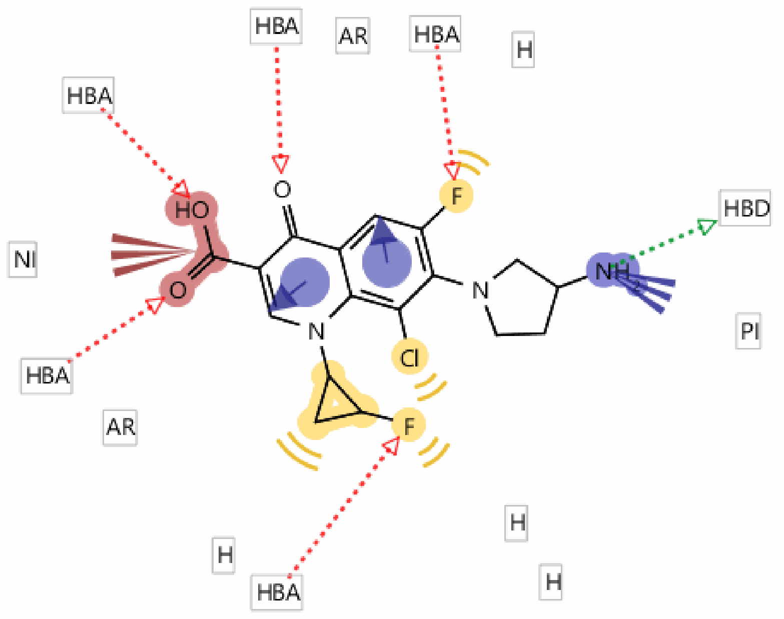
DU 6668		JPKXWJYRVJVZOY-CYBMUJFWSA-N	InChI=1S/C19H19ClFN3O3/c20-14-15-10(17(25)11(18(26)27)6-24(15)9-1-2-9)5-12(21)16(14)23-7-13(22)19(8-23)3-4-19/h5-6,9,13H,1-4,7-8,22H2,(H,26,27)/t13-/m1/s1	ClC1=C2C(C(C(C(O)=O)=CN2C3CC3)=O)=CC(F)=C1N4C[C@H](C5(CC5)C4)N	DNA gyrase and topoisomerase IV inhibitor	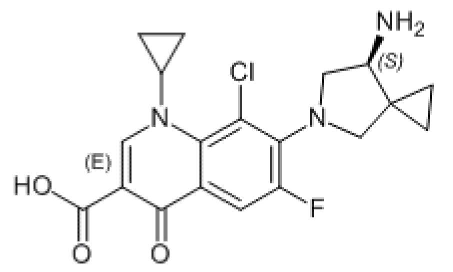	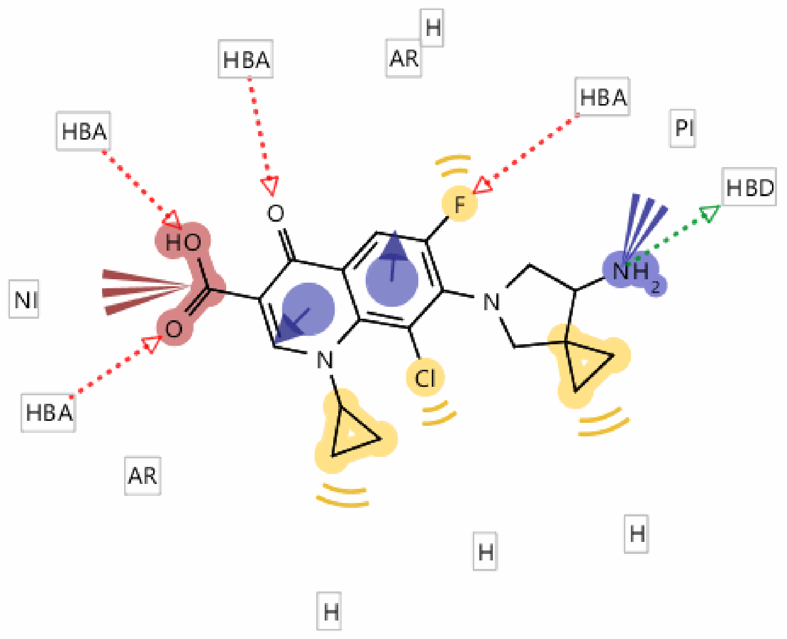
DV 7751a (DV 7751)	151390-79-3	UGGPRXAHUOKTKV-IINYFYTJSA-N	InChI=1S/C20H22FN3O4/c1-10-8-28-18-15-11(17(25)12(19(26)27)6-24(10)15)5-13(21)16(18)23-7-14(22)20(9-23)3-2-4-20/h5-6,10,14H,2-4,7-9,22H2,1H3,(H,26,27)/t10-,14+/m0/s1	C[C@H]1COC2=C3N1C=C(C(O)=O)C(C3=CC(F)=C2N4C[C@@H](N)C5(CCC5)C4)=O	DNA gyrase and topoisomerase IV inhibitor	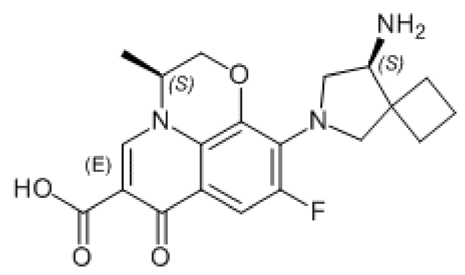	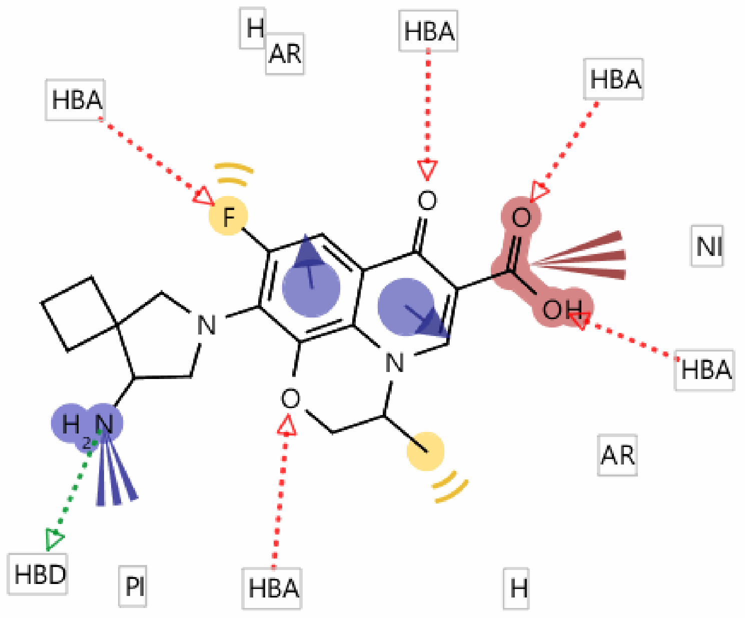

Common name	CAS Reg. No.	InChIKey	InChI	SMILES	Primary target	2D Structure	Pharmacophore[1]
DW 8186		CQGFCCLFQFQTTI-APHBMKBZSA-N	InChI=1S/C19H19F2N3O3/c20-11-3-9-13(5-14(11)23-7-16(22)19(8-23)1-2-19)24(15-4-12(15)21)6-10(17(9)25)18(26)27/h3,5-6,12,15-16H,1-2,4,7-8,22H2,(H,26,27)/t12-,15+,16+/m0/s1	N[C@@H]1CN(C2=C(F)C=C3C(N([C@@H]4C[C@@H]4F)C=C(C(O)=O)C3=O)=C2)CC51CC5	DNA gyrase and topoisomerase IV inhibitor	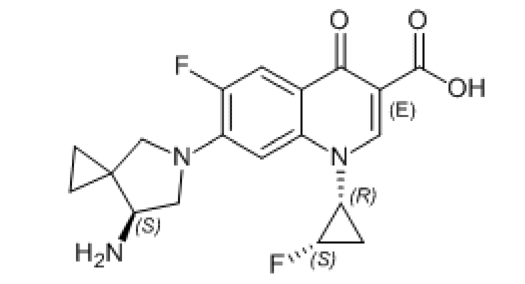	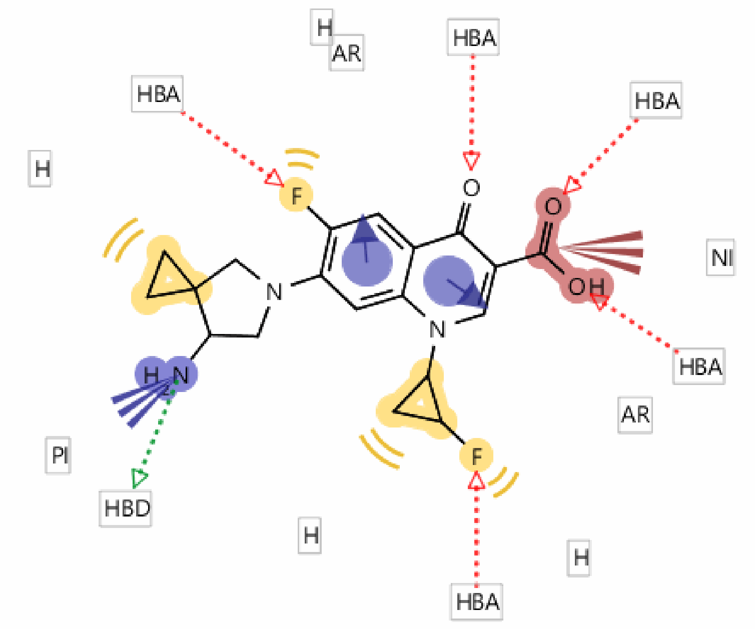
DX 619	431058-65-0	ZLICIITZJTYKAQ-DIOULYMOSA-N	InChI=1S/C21H24FN3O4/c1-29-19-15(24-7-4-11(9-24)21(23)5-6-21)3-2-12-17(19)25(16-8-14(16)22)10-13(18(12)26)20(27)28/h2-3,10-11,14,16H,4-9,23H2,1H3,(H,27,28)/t11-,14+,16-/m1/s1	COC1=C(C=CC2=C1N(C=C(C2=O)C(O)=O)[C@@H]3C[C@@H]3F)N4CC[C@H](C4)C5(CC5)N	DNA gyrase and topoisomerase IV inhibitor	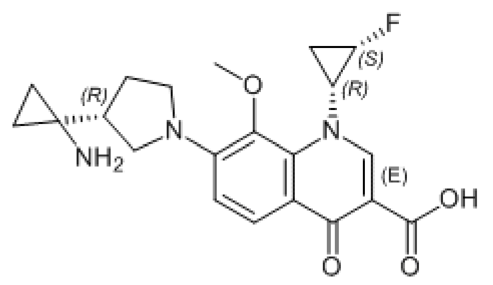	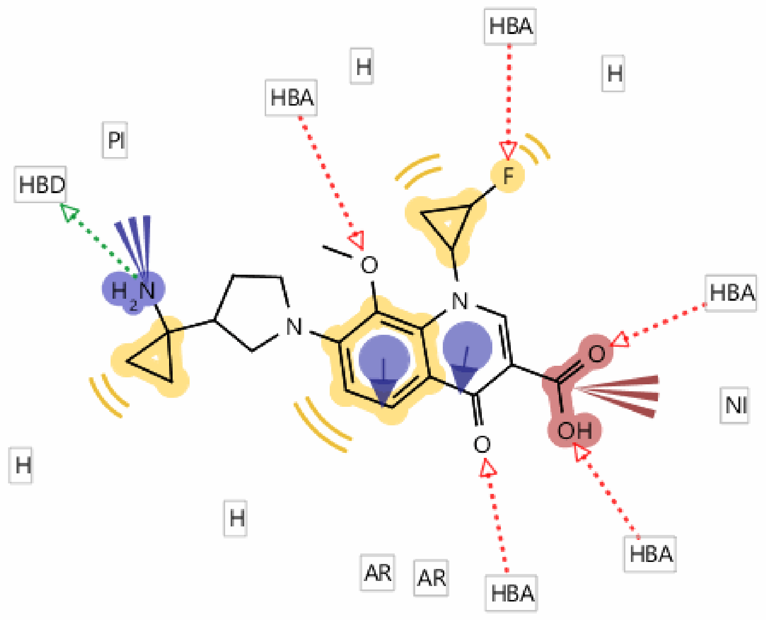
E 3604	106221-35-6	ZCGCCNYXTFRUSZ-UHFFFAOYSA-N	InChI=1S/C16H12F2N2O3/c17-3-6-20-9-11(16(22)23)15(21)10-7-12(18)14(8-13(10)20)19-4-1-2-5-19/h1-2,4-5,7-9H,3,6H2,(H,22,23)	FCCN1C=C(C(C2=CC(F)=C(C=C21)N3C=CC=C3)=O)C(O)=O	DNA gyrase and topoisomerase IV inhibitor	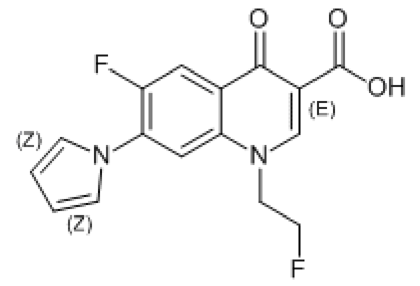	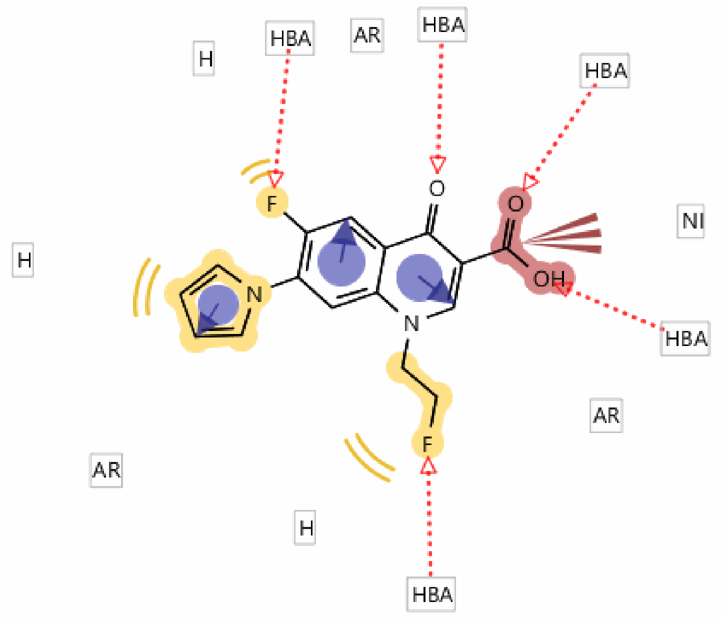
E 3846	106891-93-4	ISJRMOARTUIEPA-UHFFFAOYSA-N	InChI=1S/C17H13FN2O3/c18-13-7-11-14(8-15(13)19-5-1-2-6-19)20(10-3-4-10)9-12(16(11)21)17(22)23/h1-2,5-10H,3-4H2,(H,22,23)	FC1=C(C=C2C(C(C(C(O)=O)=CN2C3CC3)=O)=C1)N4C=CC=C4	DNA gyrase and topoisomerase IV inhibitor	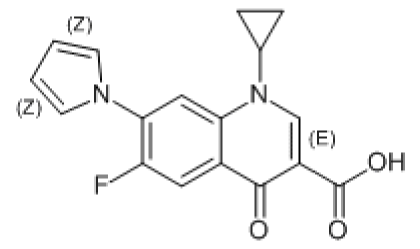	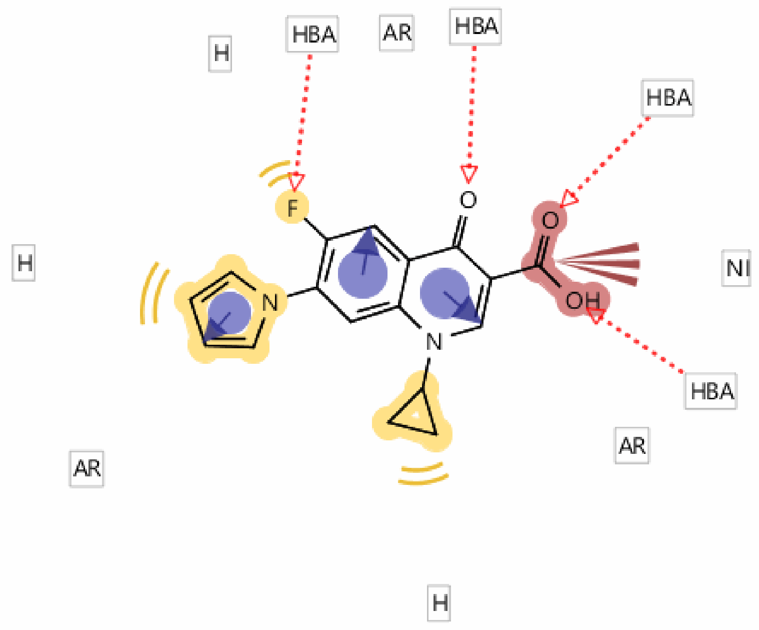
E 4441	124668-12-8	FEMZVDSBIXDODB-UHFFFAOYSA-N	InChI=1S/C17H17F2N3O3/c1-17(20)6-21(7-17)14-11(18)4-9-13(12(14)19)22(8-2-3-8)5-10(15(9)23)16(24)25/h4-5,8H,2-3,6-7,20H2,1H3,(H,24,25)	CC1(CN(C1)C2=C(C=C(C3=C2F)C(C(C(O)=O)=CN3C4CC4)=O)F)N	DNA gyrase and topoisomerase IV inhibitor	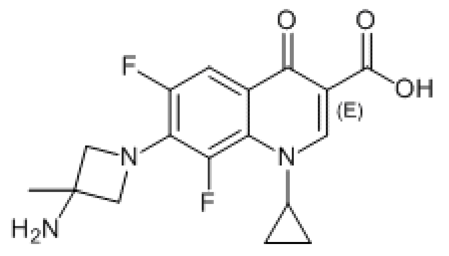	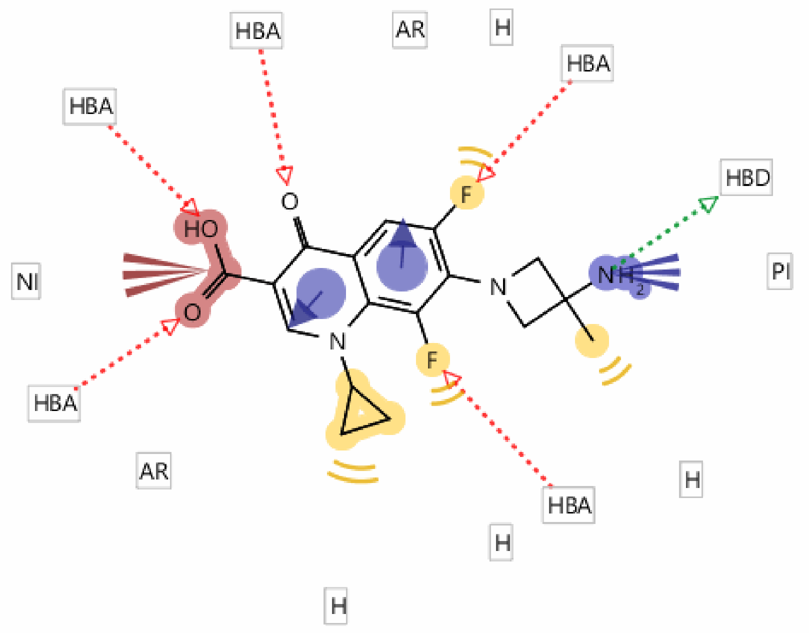
E 4474		IKZBNCORGKYEDZ-UHFFFAOYSA-N	InChI=1S/C16H15F2N3O3/c17-11-3-9-13(12(18)14(11)20-4-7(19)5-20)21(8-1-2-8)6-10(15(9)22)16(23)24/h3,6-8H,1-2,4-5,19H2,(H,23,24)	FC1=C(C(F)=C2C(C(C(C(O)=O)=CN2C3CC3)=O)=C1)N4CC(C4)N	DNA gyrase and topoisomerase IV inhibitor	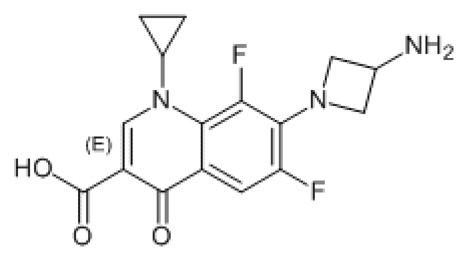	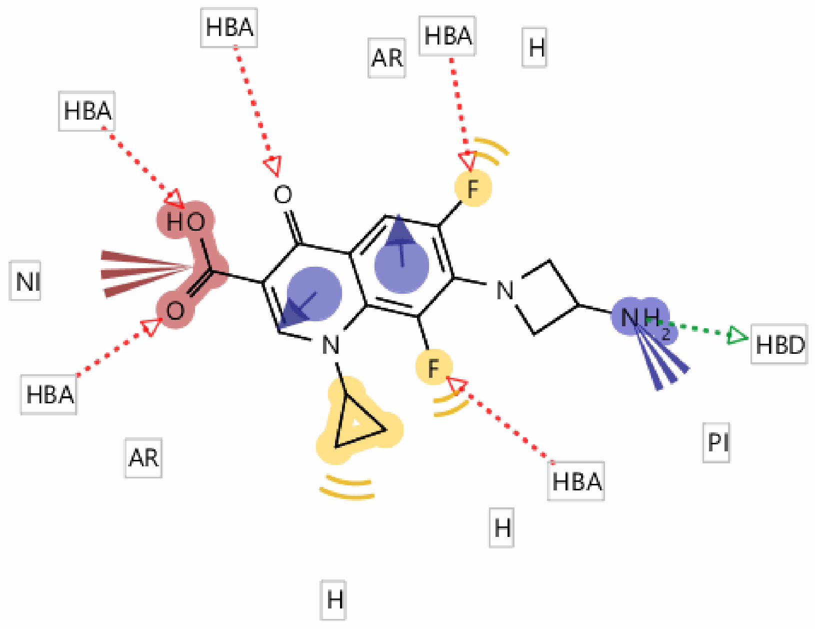

Common name	CAS Reg. No.	InChIKey	InChI	SMILES	Primary target	2D Structure	Pharmacophore[1]
E 4480		UAYYWDWWESZIPA-UHFFFAOYSA-N	InChI=1S/C17H18FN3O3/c1-17(19)7-20(8-17)14-5-13-10(4-12(14)18)15(22)11(16(23)24)6-21(13)9-2-3-9/h4-6,9H,2-3,7-8,19H2,1H3,(H,23,24)	CC1(CN(C1)C2=C(C=C(C3=C2)C(C(C(O)=O)=CN3C4CC4)=O)F)N	DNA gyrase and topoisomerase IV inhibitor	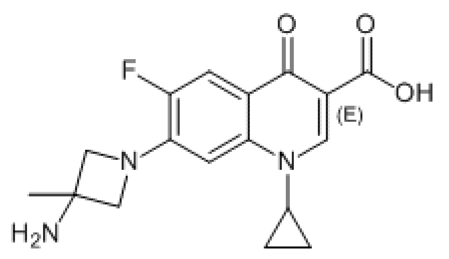	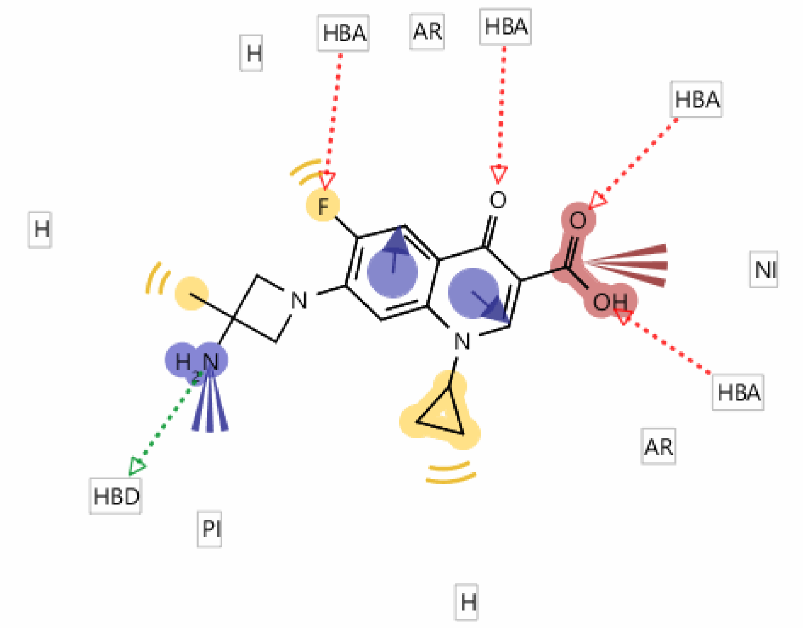
E 4497	124668-23-1	DJLDMUYZDOLNGQ-QMMMGPOBSA-N	InChI=1S/C17H18FN3O4/c1-8-5-25-15-12-9(14(22)10(16(23)24)4-21(8)12)3-11(18)13(15)20-6-17(2,19)7-20/h3-4,8H,5-7,19H2,1-2H3,(H,23,24)/t8-/m0/s1	C[C@H]1COC2=C3C(C(C(C(O)=O)=CN13)=O)=CC(F)=C2N4CC(N)(C)C4	DNA gyrase and topoisomerase IV inhibitor	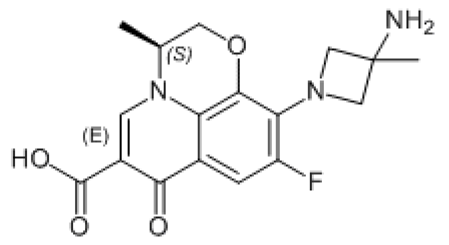	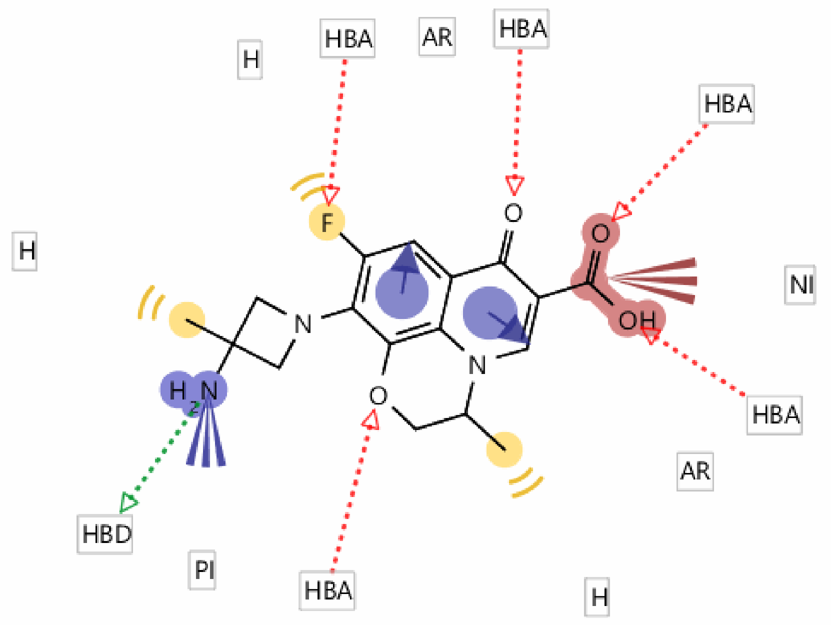
E 4501		QSNYNWOQOIJCFV-UHFFFAOYSA-N	InChI=1S/C17H17F2N3O3/c1-20-8-5-21(6-8)15-12(18)4-10-14(13(15)19)22(9-2-3-9)7-11(16(10)23)17(24)25/h4,7-9,20H,2-3,5-6H2,1H3,(H,24,25)	CNC1CN(C2=C(F)C=C(C(C(C(O)=O)=CN3C4CC4)=O)C3=C2F)C1	DNA gyrase and topoisomerase IV inhibitor	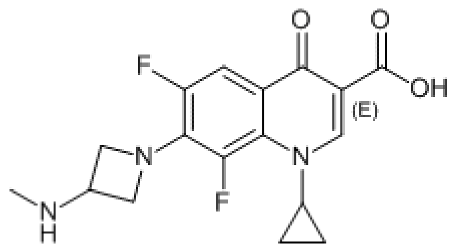	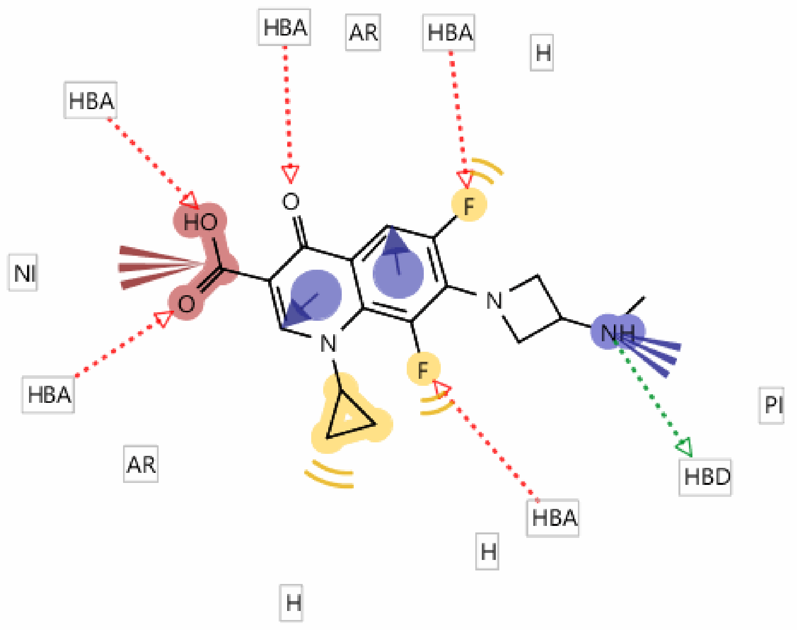
E 4502		XDLPWPNVUPKKSN-UHFFFAOYSA-N	InChI=1S/C17H18FN3O3/c1-19-9-6-20(7-9)15-5-14-11(4-13(15)18)16(22)12(17(23)24)8-21(14)10-2-3-10/h4-5,8-10,19H,2-3,6-7H2,1H3,(H,23,24)	CNC1CN(C1)C2=C(C=C(C3=C2)C(C(C(O)=O)=CN3C4CC4)=O)F	DNA gyrase and topoisomerase IV inhibitor	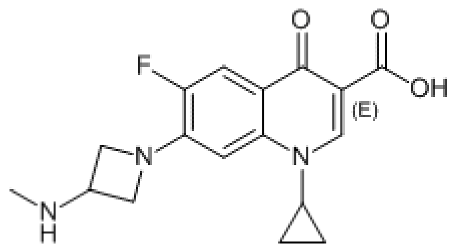	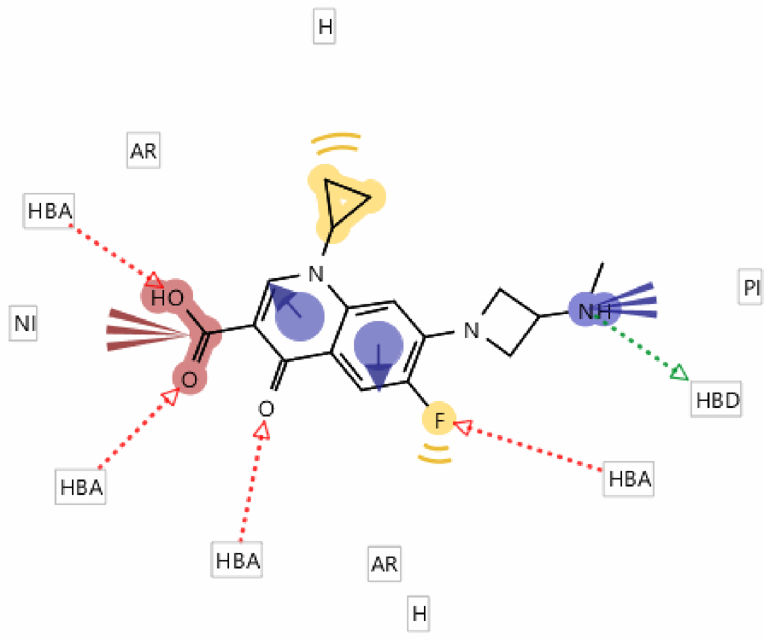
E 4527		UXCMKYWTPWHUIC-UHFFFAOYSA-N	InChI=1S/C18H19F2N3O3/c1-18(21-2)7-22(8-18)15-12(19)5-10-14(13(15)20)23(9-3-4-9)6-11(16(10)24)17(25)26/h5-6,9,21H,3-4,7-8H2,1-2H3,(H,25,26)	CC1(CN(C1)C2=C(C=C3C(N(C=C(C3=O)C(O)=O)C4CC4)=C2F)F)NC	DNA gyrase and topoisomerase IV inhibitor	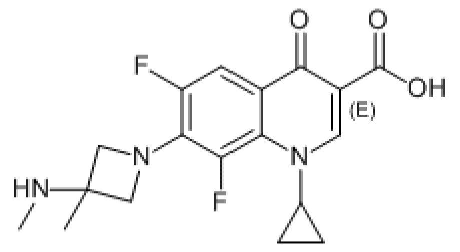	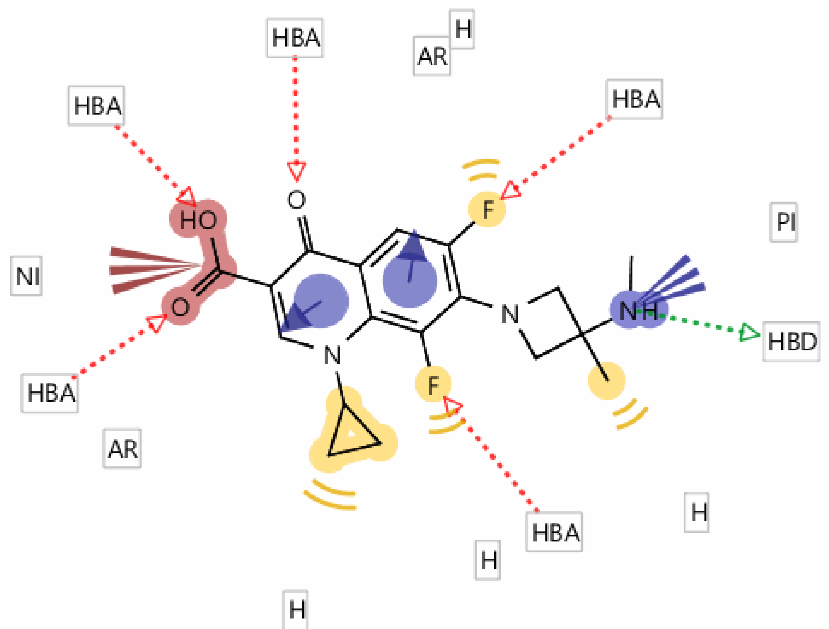
E 4528		NYJWUOYWVKEHPC-UHFFFAOYSA-N	InChI=1S/C18H20FN3O3/c1-18(20-2)8-21(9-18)15-6-14-11(5-13(15)19)16(23)12(17(24)25)7-22(14)10-3-4-10/h5-7,10,20H,3-4,8-9H2,1-2H3,(H,24,25)	CC1(NC)CN(C2=C(F)C=C3C(N(C4CC4)C=C(C(O)=O)C3=O)=C2)C1	DNA gyrase and topoisomerase IV inhibitor	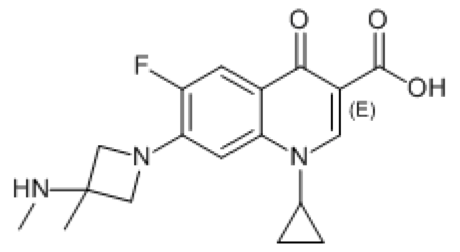	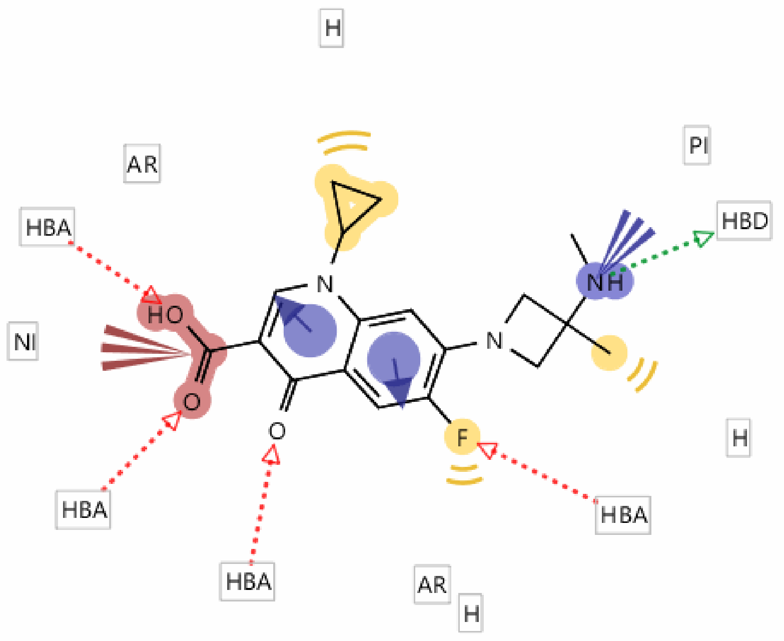

Common name	CAS Reg. No.	InChIKey	InChI	SMILES	Primary target	2D Structure	Pharmacophore[1]
E 4534		ORHUFIOIUIPUTP-JVXZTZIISA-N	InChI=1S/C17H17F2N3O3/c1-7-12(20)6-21(7)15-11(18)4-9-14(13(15)19)22(8-2-3-8)5-10(16(9)23)17(24)25/h4-5,7-8,12H,2-3,6,20H2,1H3,(H,24,25)/t7-,12+/m0/s1	C[C@H]1[C@H](N)CN1C2=C(F)C=C(C(C(C(O)=O)=CN3C4CC4)=O)C3=C2F	DNA gyrase and topoisomerase IV inhibitor	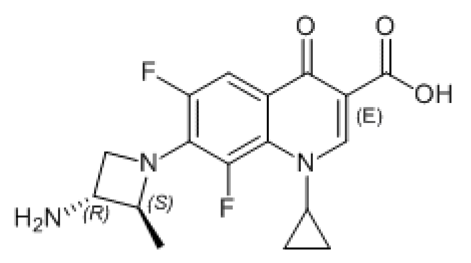	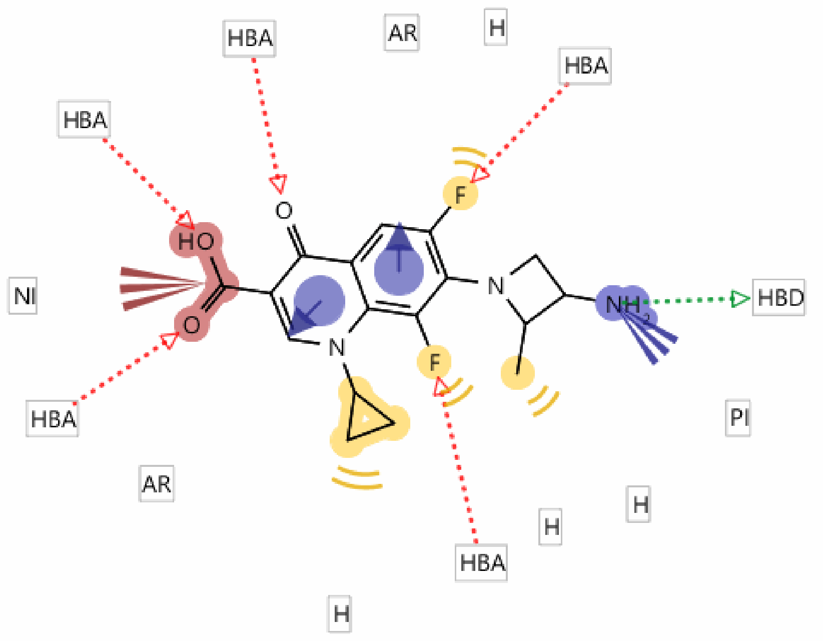
E 4535		BYUOTYDQGYSLFD-ISVAXAHUSA-N	InChI=1S/C17H18FN3O3/c1-8-13(19)7-20(8)15-5-14-10(4-12(15)18)16(22)11(17(23)24)6-21(14)9-2-3-9/h4-6,8-9,13H,2-3,7,19H2,1H3,(H,23,24)/t8-,13+/m0/s1	C[C@H]1[C@H](N)CN1C2=C(F)C=C3C(N(C4CC4)C=C(C(O)=O)C3=O)=C2	DNA gyrase and topoisomerase IV inhibitor	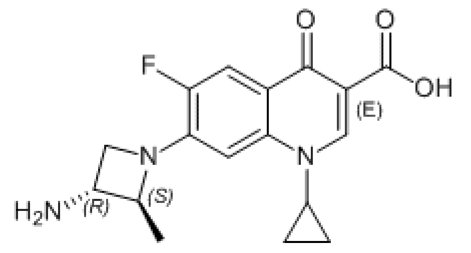	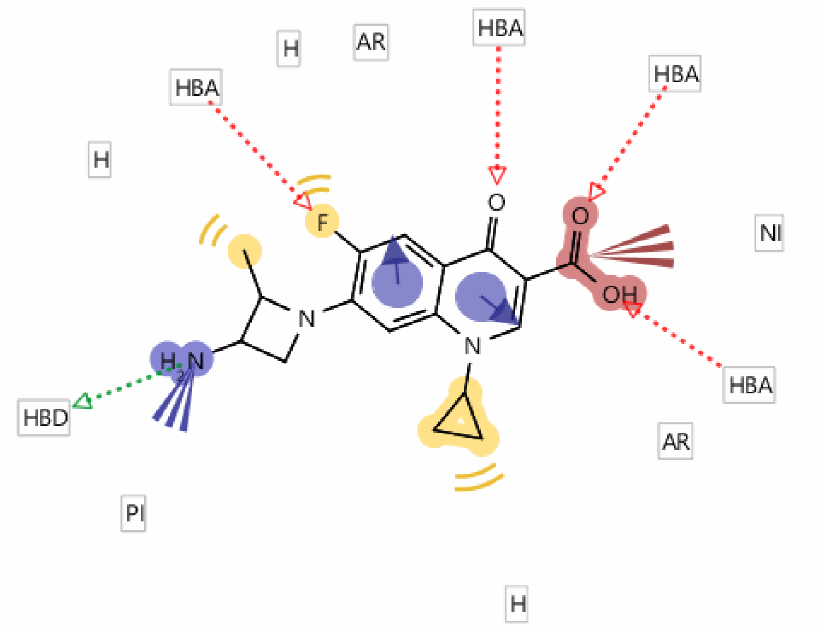
E 4695	132832-30-5	AUJKWLGOXRSXRX-JVXZTZIISA-N	InChI=1S/C16H17FN4O3/c1-7-12(18)6-20(7)15-11(17)4-9-13(22)10(16(23)24)5-21(8-2-3-8)14(9)19-15/h4-5,7-8,12H,2-3,6,18H2,1H3,(H,23,24)/t7-,12+/m0/s1	C[C@H]1[C@H](N)CN1C2=C(F)C=C(C3=N2)C(C(C(O)=O)=CN3C4CC4)=O	DNA gyrase and topoisomerase IV inhibitor	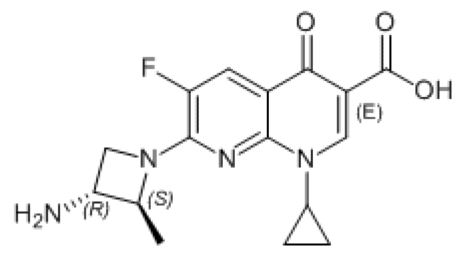	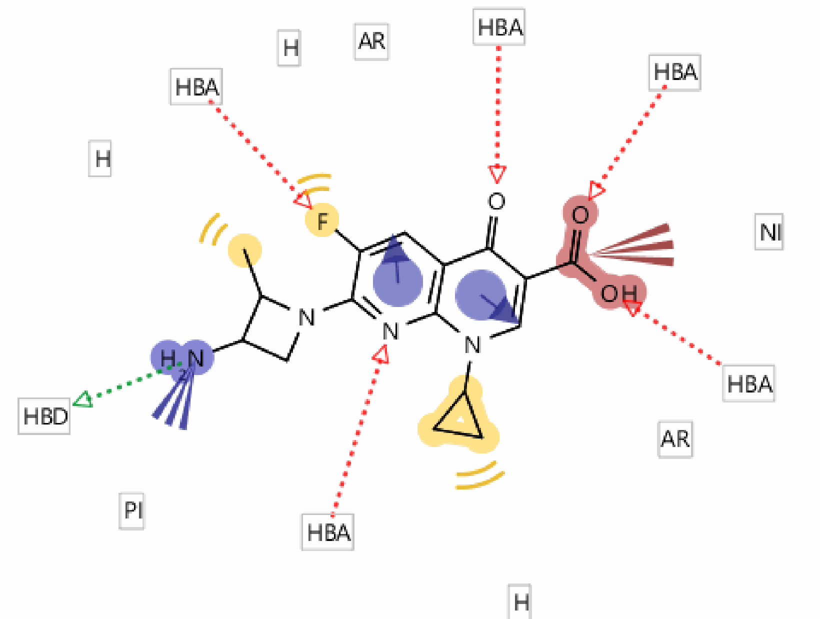
Ecenofloxacin	162301-05-5	WNVIWAUAXKEKKF-PJFSTRORSA-N	InChI=1S/C19H21FN4O3/c1-19-5-14(21)12(19)7-23(8-19)17-13(20)4-10-15(25)11(18(26)27)6-24(9-2-3-9)16(10)22-17/h4,6,9,12,14H,2-3,5,7-8,21H2,1H3,(H,26,27)/t12-,14-,19-/m0/s1	C[C@@]12C[C@@H]([C@@]1(CN(C2)C3=C(C=C(C(C(C(O)=O)=CN4C5CC5)=O)C4=N3)F)[H])N	DNA gyrase and topoisomerase IV inhibitor	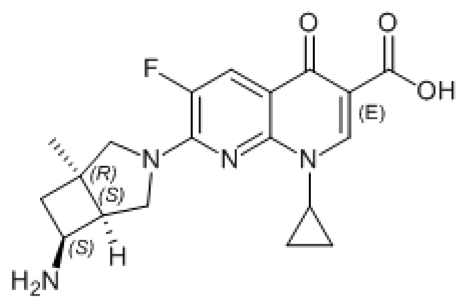	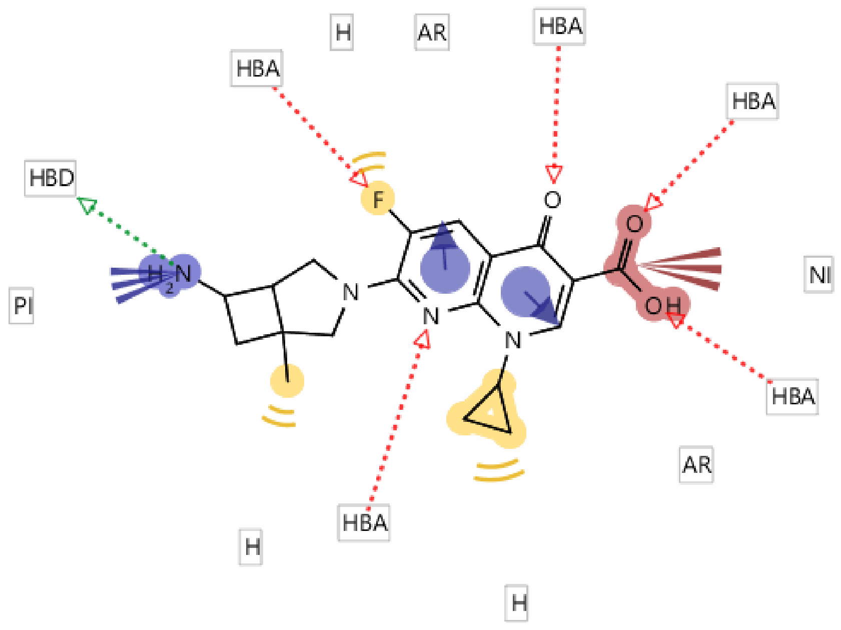
EN 272	64621-47-2	JYJKFMPCGBLLHN-UHFFFAOYSA-N	InChI=1S/C12H7F2NO5/c13-12(14)15-3-6(11(17)18)10(16)5-1-8-9(2-7(5)15)20-4-19-8/h1-3,12H,4H2,(H,17,18)	FC(F)N1C=C(C(C2=CC3=C(C=C21)OCO3)=O)C(O)=O	DNA gyrase and topoisomerase IV inhibitor	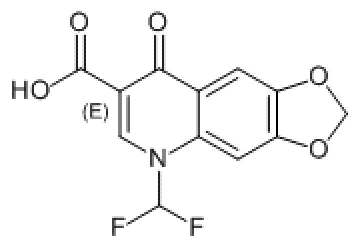	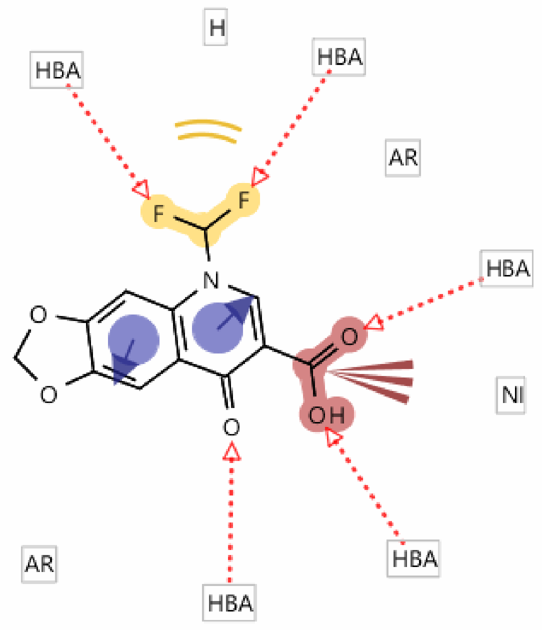
Enoxacin	74011-58-8	IDYZIJYBMGIQMJ-UHFFFAOYSA-N	InChI=1S/C15H17FN4O3/c1-2-19-8-10(15(22)23)12(21)9-7-11(16)14(18-13(9)19)20-5-3-17-4-6-20/h7-8,17H,2-6H2,1H3,(H,22,23)	CCN1C=C(C(C2=CC(F)=C(N=C21)N3CCNCC3)=O)C(O)=O	DNA gyrase and topoisomerase IV inhibitor	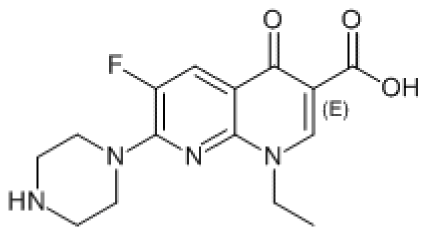	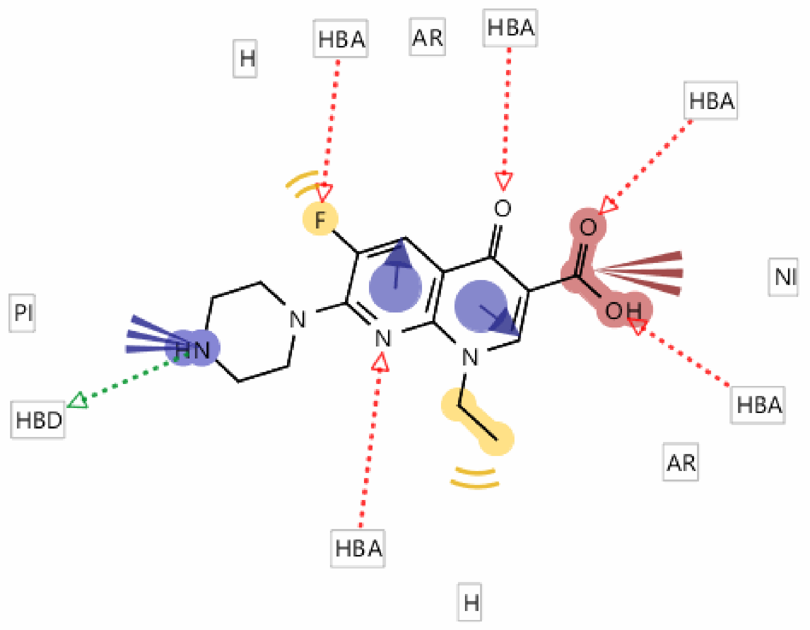

Common name	CAS Reg. No.	InChIKey	InChI	SMILES	Primary target	2D Structure	Pharmacophore[1]
Enrofloxacin	93106-60-6	SPFYMRJSYKOXGV-UHFFFAOYSA-N	InChI=1S/C19H22FN3O3/c1-2-21-5-7-22(8-6-21)17-10-16-13(9-15(17)20)18(24)14(19(25)26)11-23(16)12-3-4-12/h9-12H,2-8H2,1H3,(H,25,26)	CCN1CCN(C2=C(F)C=C(C(C(C(O)=O)=CN3C4CC4)=O)C3=C2)CC1	DNA gyrase and topoisomerase IV inhibitor	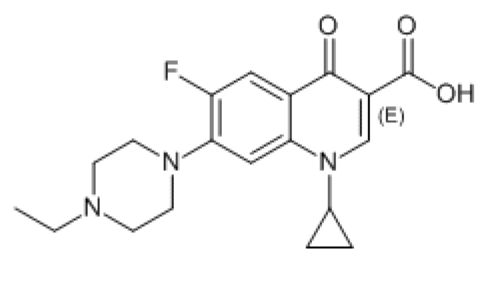	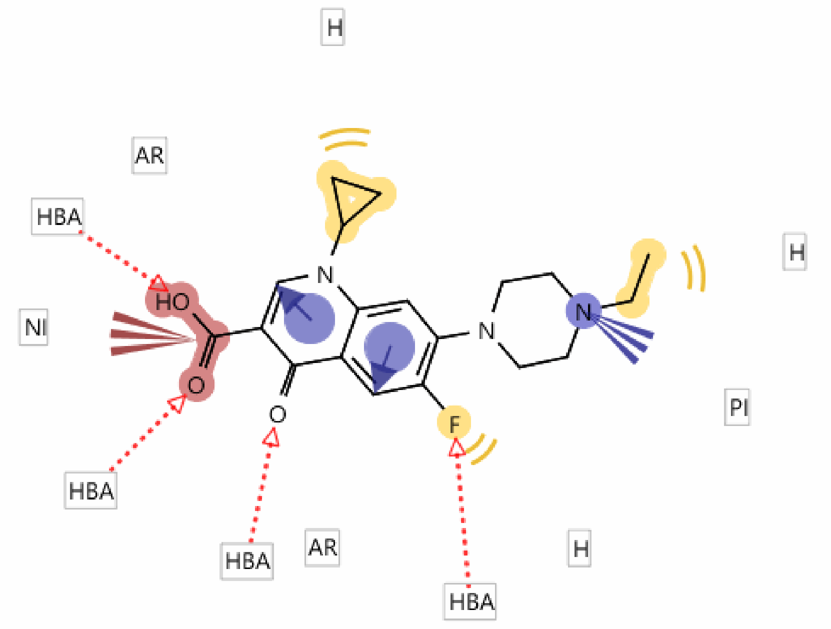
Esafloxacin	79286-77-4	INACKBWBZLPNAP-RMTNWKGQSA-N	InChI=1S/2C15H17FN4O3/c2*1-2-19-7-10(15(22)23)12(21)9-5-11(16)14(18-13(9)19)20-4-3-8(17)6-20/h2*5,7-8H,2-4,6,17H2,1H3,(H,22,23)/t2*8-/m10/s1	CCN1C=C(C(C2=CC(F)=C(N=C21)N3CC[C@H](C3)N)=O)C(O)=O.CCN4C=C(C(C5=CC(F)=C(N=C54)N6CC[C@@H](C6)N)=O)C(O)=O	DNA gyrase and topoisomerase IV inhibitor	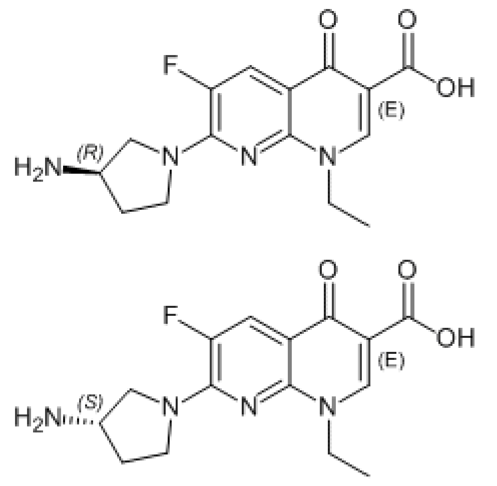	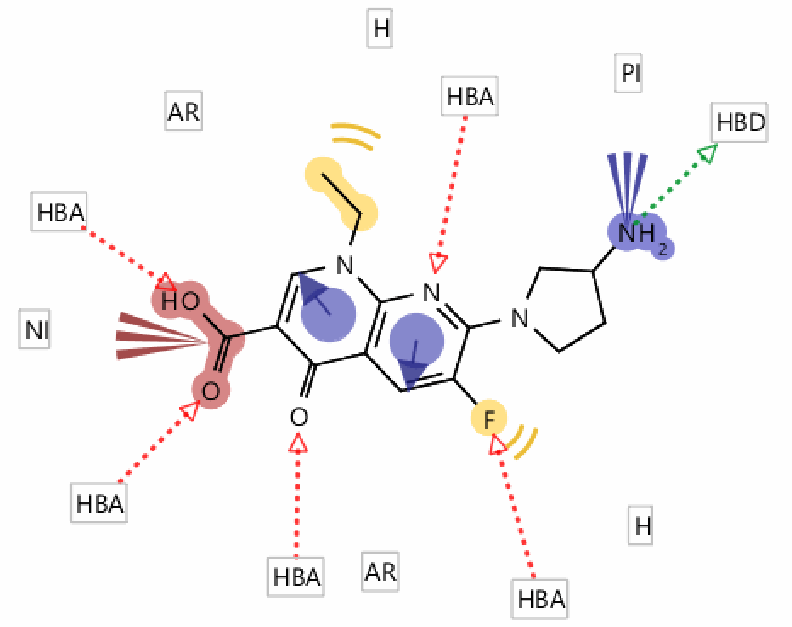
FA 103		CPQWMBJYDCTDKL-UHFFFAOYSA-N	InChI=1S/C18H18F2N4O4/c19-12-14(21)11-15(13(20)16(12)23-5-3-10(25)22-4-6-23)24(8-1-2-8)7-9(17(11)26)18(27)28/h7-8H,1-6,21H2,(H,22,25)(H,27,28)	FC1=C(C(F)=C2C(C(C(C(O)=O)=CN2C3CC3)=O)=C1N)N(CC4)CCNC4=O	DNA gyrase and topoisomerase IV inhibitor	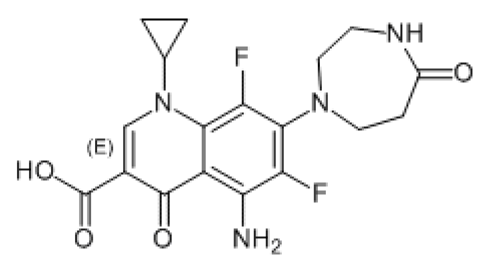	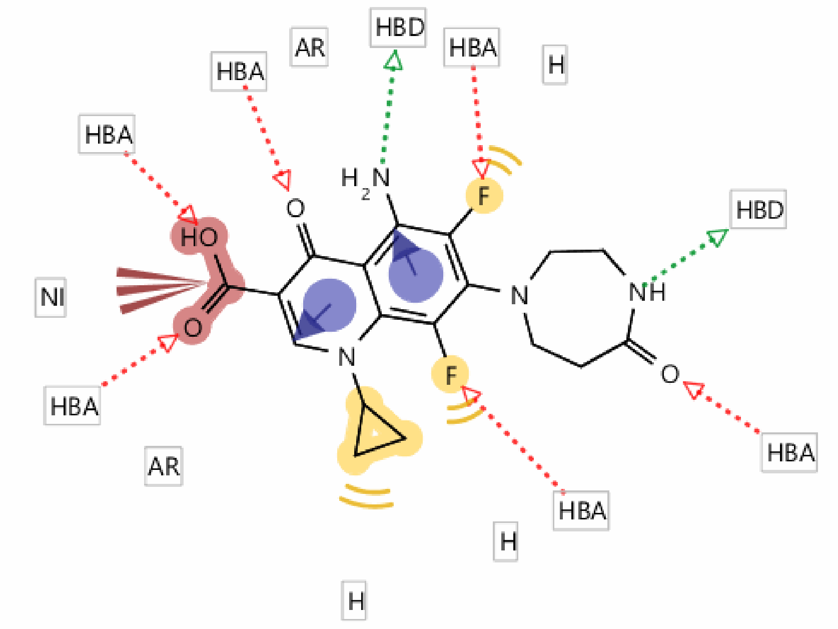
Fandofloxacin	164150-99-6	AWCAUUQZTXYMPB-UHFFFAOYSA-N	InChI=1S/C20H18F2N4O3/c1-24-4-6-25(7-5-24)17-9-16-13(8-15(17)22)19(27)14(20(28)29)11-26(16)18-3-2-12(21)10-23-18/h2-3,8-11H,4-7H2,1H3,(H,28,29)	CN1CCN(C2=C(F)C=C(C(C(C(O)=O)=CN3C4=NC=C(F)C=C4)=O)C3=C2)CC1	DNA gyrase and topoisomerase IV inhibitor	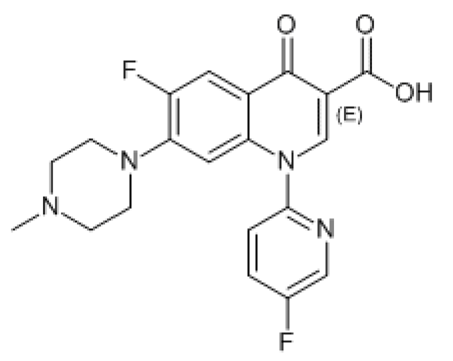	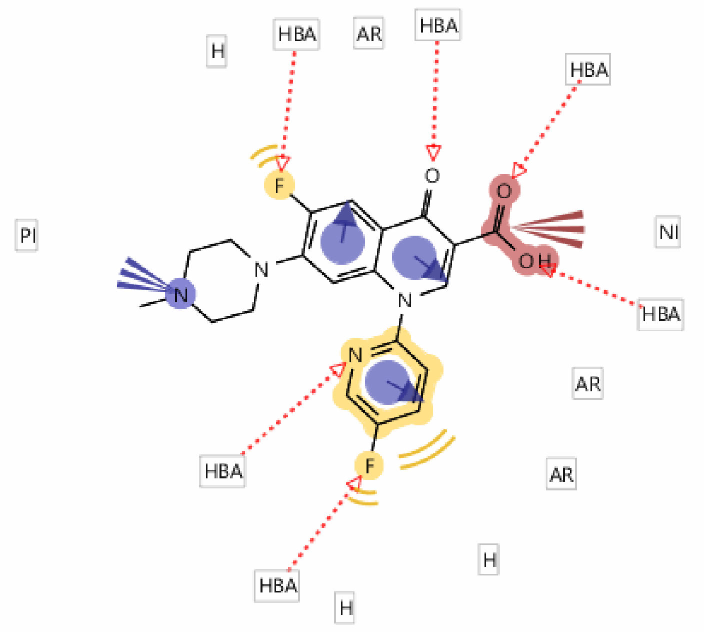
Finafloxacin	209342-40-5	FYMHQCNFKNMJAV-HOTGVXAUSA-N	InChI=1S/C20H19FN4O4/c21-14-5-11-17(25(10-1-2-10)7-13(19(11)26)20(27)28)12(6-22)18(14)24-8-15-16(9-24)29-4-3-23-15/h5,7,10,15-16,23H,1-4,8-9H2,(H,27,28)/t15-,16-/m0/s1	FC1=C(N2C[C@@]3(NCCO[C@@]3([H])C2)[H])C(C#N)=C4C(C(C(C(O)=O)=CN4C5CC5)=O)=C1	DNA gyrase and topoisomerase IV inhibitor	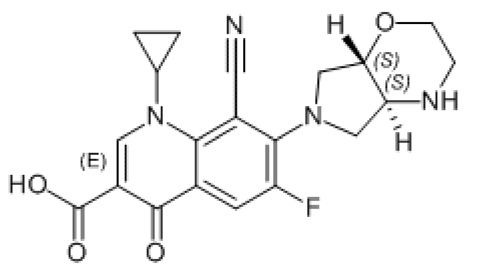	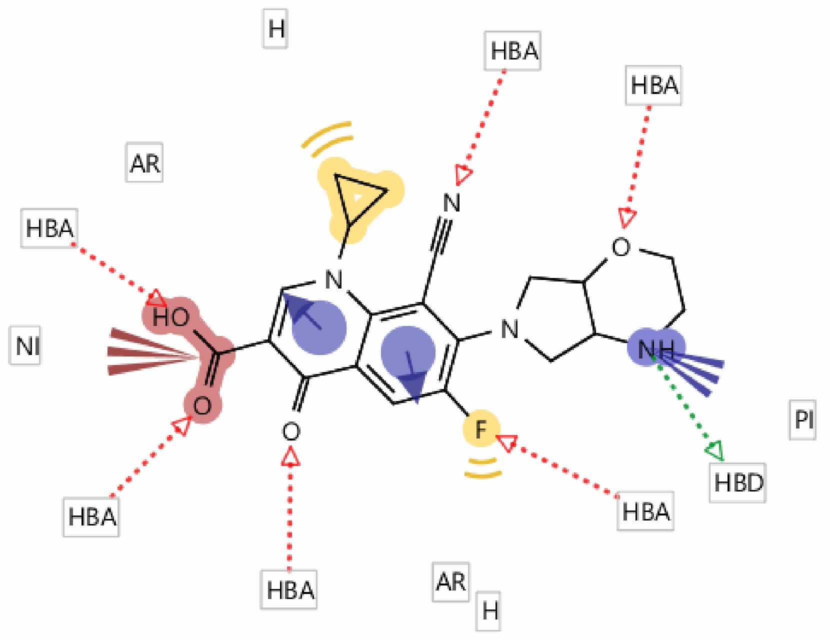
Fleroxacin	79660-72-3	XBJBPGROQZJDOJ-UHFFFAOYSA-N	InChI=1S/C17H18F3N3O3/c1-21-4-6-22(7-5-21)15-12(19)8-10-14(13(15)20)23(3-2-18)9-11(16(10)24)17(25)26/h8-9H,2-7H2,1H3,(H,25,26)	CN1CCN(C2=C(F)C=C(C(C(C(O)=O)=CN3CCF)=O)C3=C2F)CC1	DNA gyrase and topoisomerase IV inhibitor	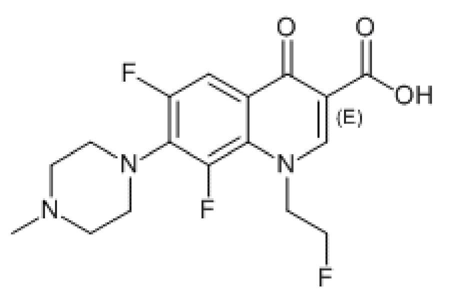	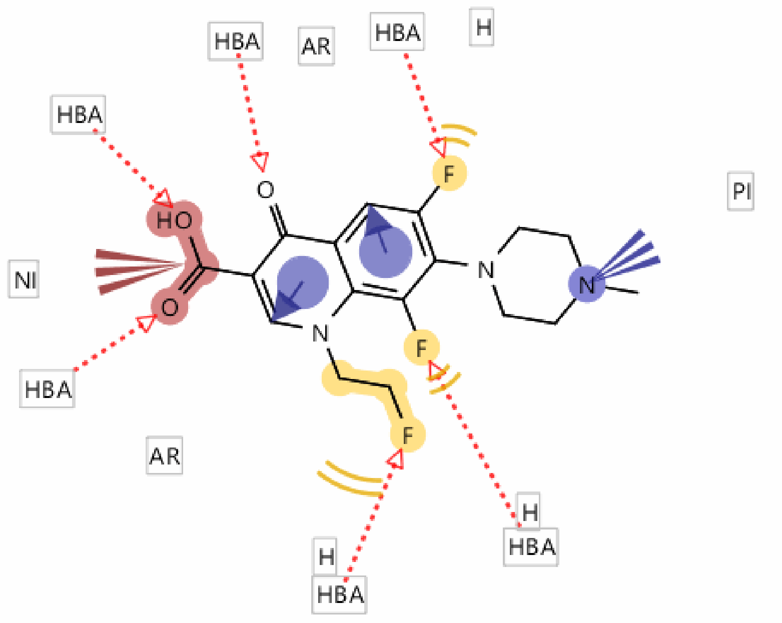

Common name	CAS Reg. No.	InChIKey	InChI	SMILES	Primary target	2D Structure	Pharmacophore[1]
Flumequine	42835-25-6	DUIUYJJSXDLYCM-UNLCMTKJSA-N	InChI=1S/2C14H12FNO3/c2*1-7-2-3-8-4-9(15)5-10-12(8)16(7)6-11(13(10)17)14(18)19/h2*4-7H,2-3H2,1H3,(H,18,19)/t2*7-/m10/s1	C[C@@H]1CCC2=CC(F)=CC3=C2N1C=C(C3=O)C(O)=O.C[C@H]4CCC5=CC(F)=CC6=C5N4C=C(C6=O)C(O)=O	DNA gyrase and topoisomerase IV inhibitor	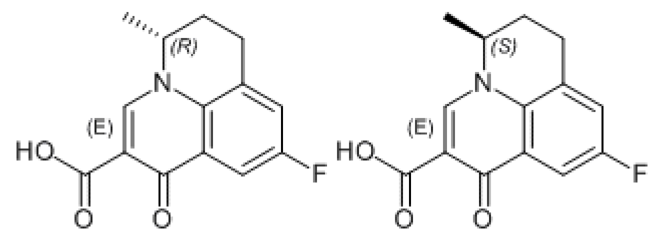	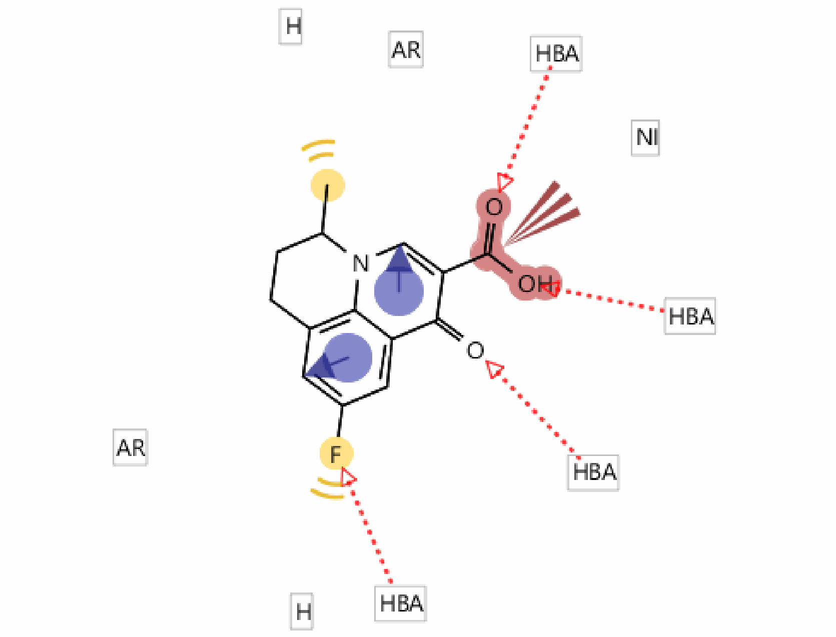
Garenoxacin	194804-75-6	NJDRXTDGYFKORP-LLVKDONJSA-N	InChI=1S/C23H20F2N2O4/c1-11-15-5-2-12(8-13(15)9-26-11)16-6-7-17-19(21(16)31-23(24)25)27(14-3-4-14)10-18(20(17)28)22(29)30/h2,5-8,10-11,14,23,26H,3-4,9H2,1H3,(H,29,30)/t11-/m1/s1	C[C@@H]1C2=C(CN1)C=C(C3=C(OC(F)F)C4=C(C(C(C(O)=O)=CN4C5CC5)=O)C=C3)C=C2	DNA gyrase and topoisomerase IV inhibitor	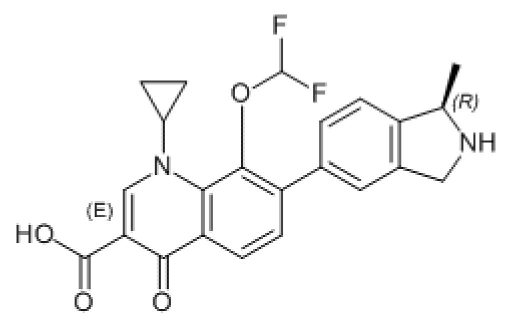	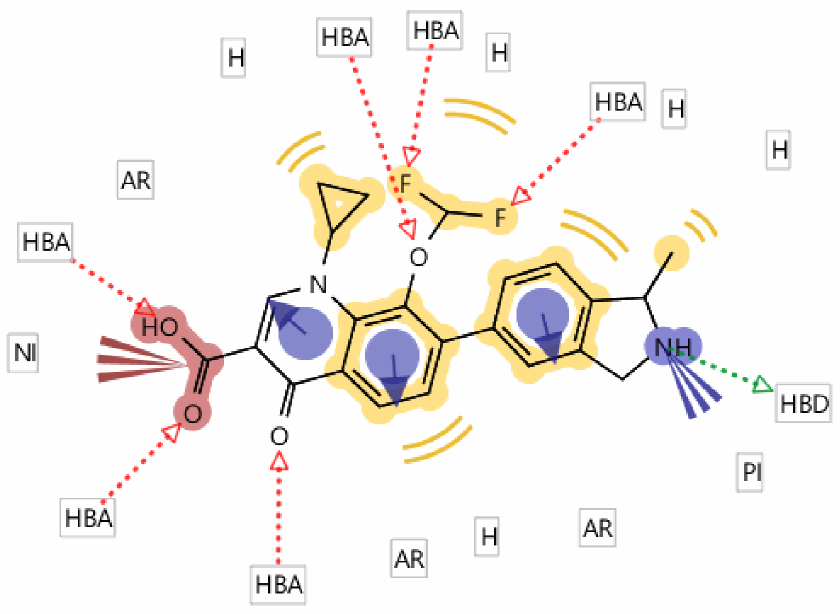
Gatifloxacin	112811-59-3	LCHNOAXZYULHND-FTYBWHBYSA-N	InChI=1S/2C19H22FN3O4/c2*1-10-8-22(6-5-21-10)16-14(20)7-12-15(18(16)27-2)23(11-3-4-11)9-13(17(12)24)19(25)26/h2*7,9-11,21H,3-6,8H2,1-2H3,(H,25,26)/t2*10-/m10/s1	C[C@@H]1CN(CCN1)C2=C(C=C(C3=C2OC)C(C(C(O)=O)=CN3C4CC4)=O)F.C[C@H]5CN(CCN5)C6=C(C=C(C7=C6OC)C(C(C(O)=O)=CN7C8CC8)=O)F	DNA gyrase and topoisomerase IV inhibitor	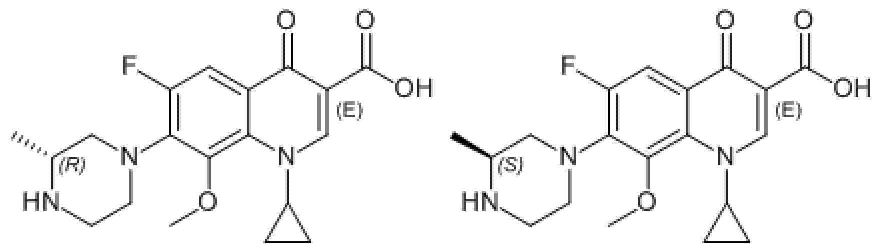	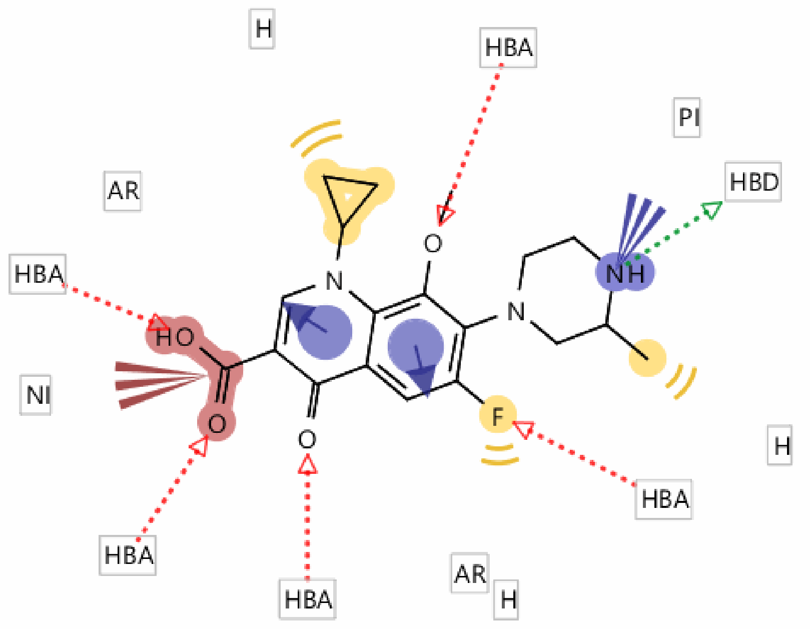
Gemifloxacin	175463-14-6	AWFVHALVCKCSEK-IDSNOWMPSA-N	InChI=1S/2C18H20FN5O4/c2*1-28-22-14-8-23(6-9(14)5-20)17-13(19)4-11-15(25)12(18(26)27)7-24(10-2-3-10)16(11)21-17/h2*4,7,9-10H,2-3,5-6,8,20H2,1H3,(H,26,27)/b2*22-14+/t2*9-/m10/s1	CO/N=C1CN(C[C@H]\1CN)C2=C(C=C(C(C(C(O)=O)=CN3C4CC4)=O)C3=N2)F.CO/N=C5CN(C[C@@H]\5CN)C6=C(C=C(C(C(C(O)=O)=CN7C8CC8)=O)C7=N6)F	DNA gyrase and topoisomerase IV inhibitor	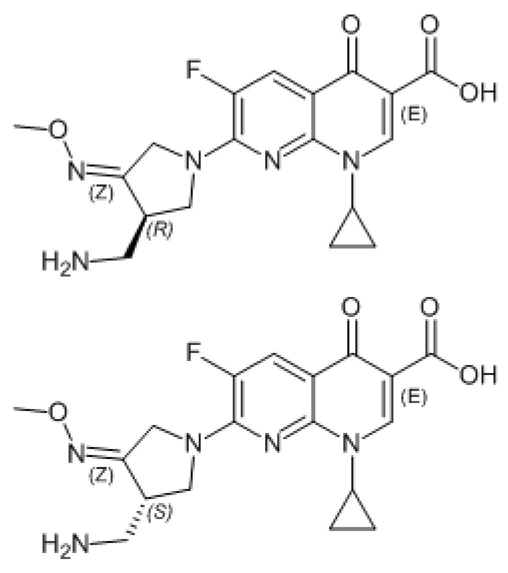	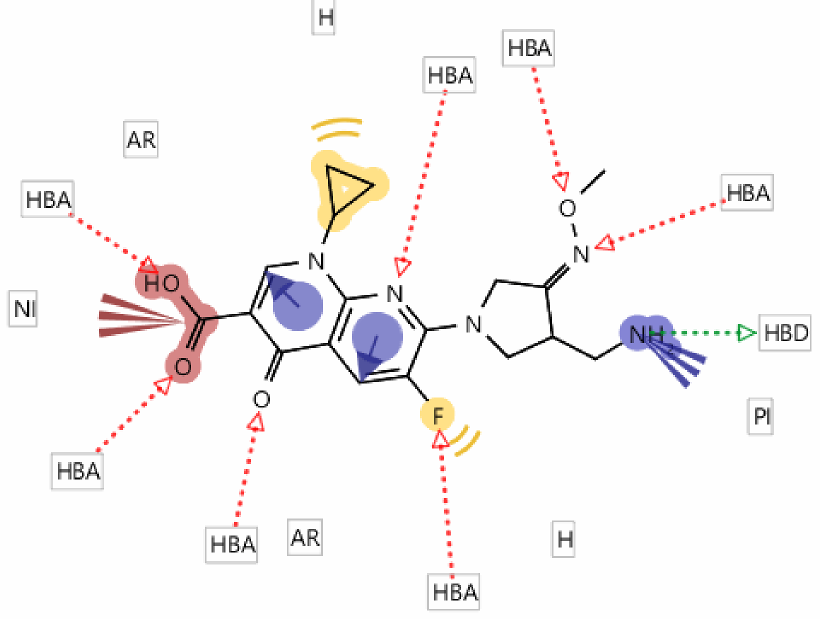
Grepafloxacin	119914-60-2	YKLZHTPFCBHELM-FTYBWHBYSA-N	InChI=1S/2C19H22FN3O3/c2*1-10-8-22(6-5-21-10)15-7-14-16(11(2)17(15)20)18(24)13(19(25)26)9-23(14)12-3-4-12/h2*7,9-10,12,21H,3-6,8H2,1-2H3,(H,25,26)/t2*10-/m10/s1	C[C@@H]1CN(CCN1)C2=C(C(C)=C(C3=C2)C(C(C(O)=O)=CN3C4CC4)=O)F.C[C@H]5CN(CCN5)C6=C(C(C)=C(C7=C6)C(C(C(O)=O)=CN7C8CC8)=O)F	DNA gyrase and topoisomerase IV inhibitor	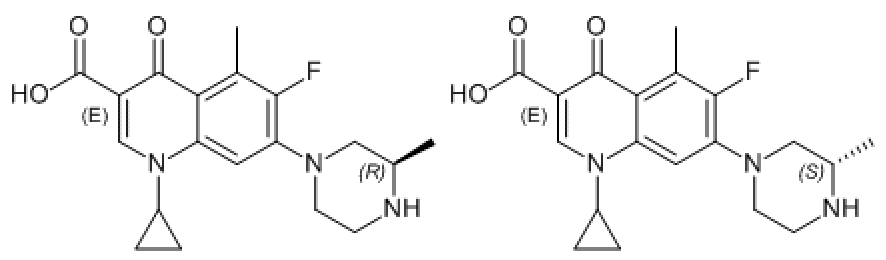	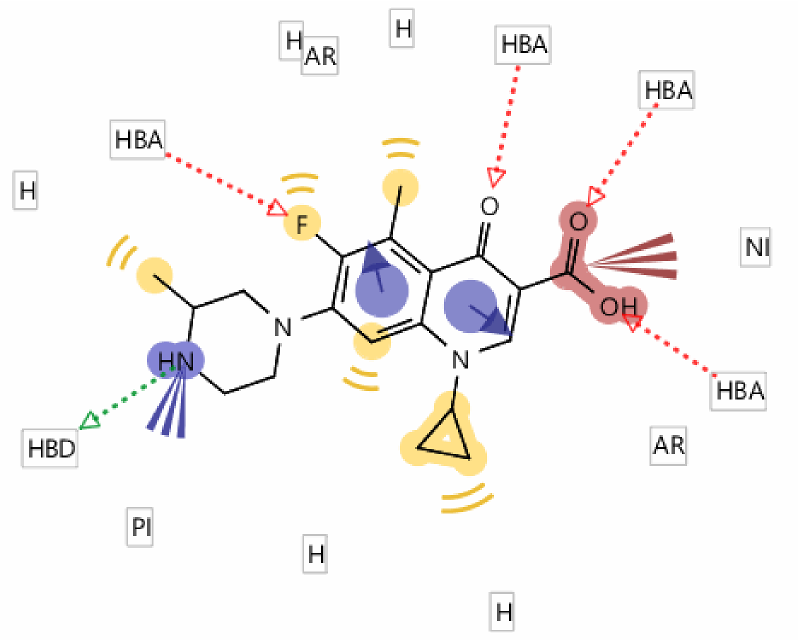
Ibafloxacin	91618-36-9	AEOLDEPXDCCJPC-UNLCMTKJSA-N	InChI=1S/2C15H14FNO3/c2*1-7-3-4-9-8(2)12(16)5-10-13(9)17(7)6-11(14(10)18)15(19)20/h2*5-7H,3-4H2,1-2H3,(H,19,20)/t2*7-/m10/s1	C[C@@H]1CCC2=C(C)C(F)=CC3=C2N1C=C(C3=O)C(O)=O.C[C@H]4CCC5=C(C)C(F)=CC6=C5N4C=C(C6=O)C(O)=O	DNA gyrase and topoisomerase IV inhibitor	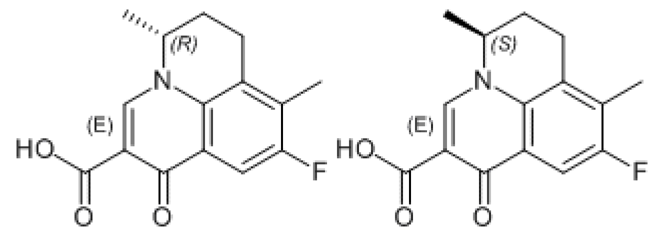	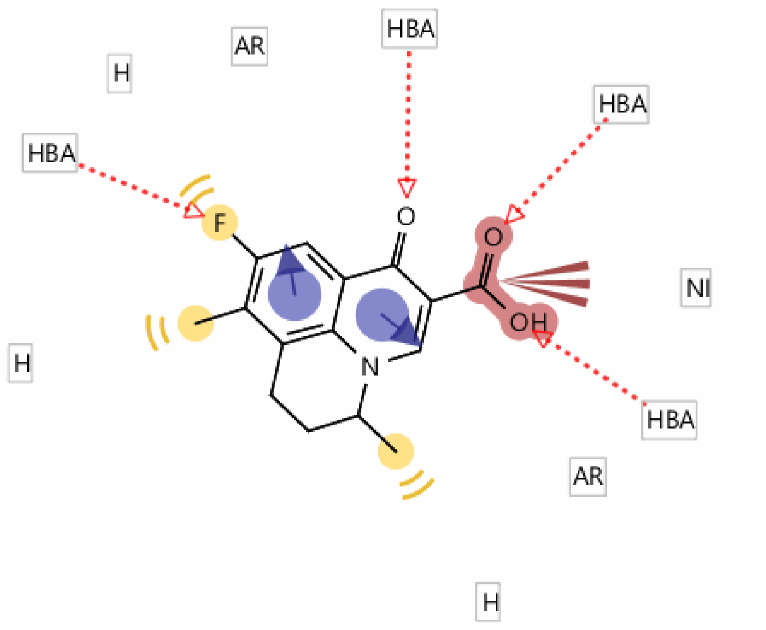

Common name	CAS Reg. No.	InChIKey	InChI	SMILES	Primary target	2D Structure	Pharmacophore[1]
Irloxacin	91524-15-1	RZLHGQLYNZQZQQ-UHFFFAOYSA-N	InChI=1S/C16H13FN2O3/c1-2-18-9-11(16(21)22)15(20)10-7-12(17)14(8-13(10)18)19-5-3-4-6-19/h3-9H,2H2,1H3,(H,21,22)	CCN1C=C(C(O)=O)C(C2=CC(F)=C(N3C=CC=C3)C=C21)=O	DNA gyrase and topoisomerase IV inhibitor	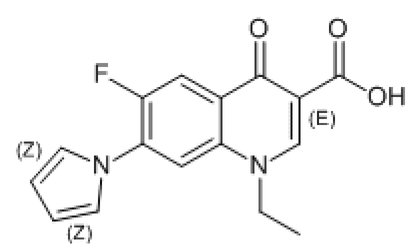	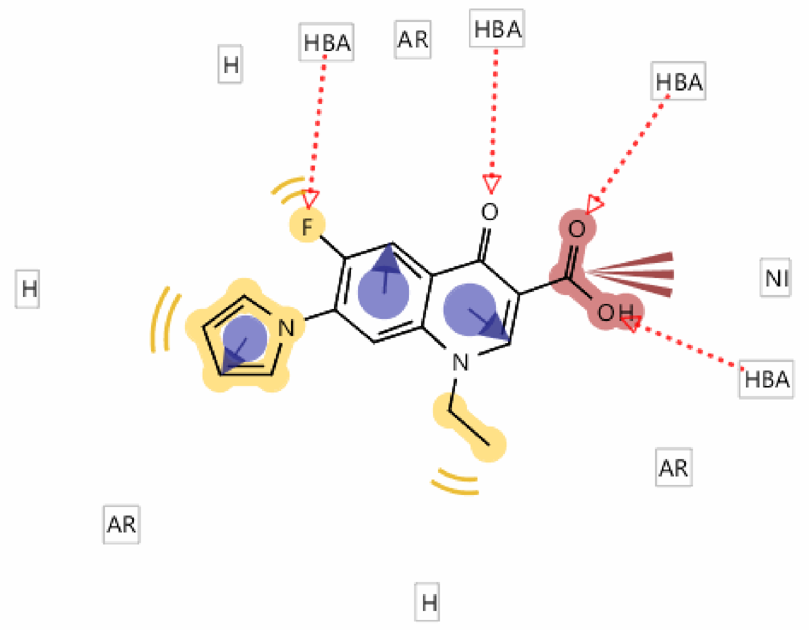
K 12	153468-00-9	DEISLDFBMJLVTC-UHFFFAOYSA-N	InChI=1S/C24H24F3N3O5/c1-3-28-13-15(23(32)33)21(31)14-12-16(25)20(22(19(14)28)35-24(26)27)30-10-8-29(9-11-30)17-6-4-5-7-18(17)34-2/h4-7,12-13,24H,3,8-11H2,1-2H3,(H,32,33)	CCN1C=C(C(C2=CC(F)=C(C(OC(F)F)=C21)N3CCN(CC3)C4=CC=CC=C4OC)=O)C(O)=O	DNA gyrase and topoisomerase IV inhibitor	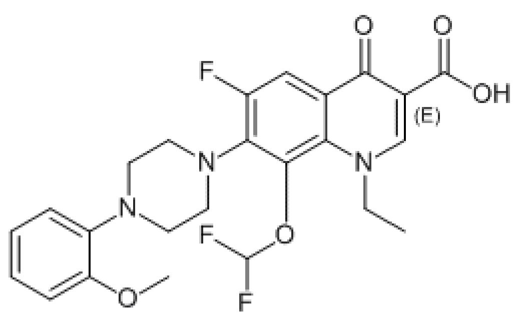	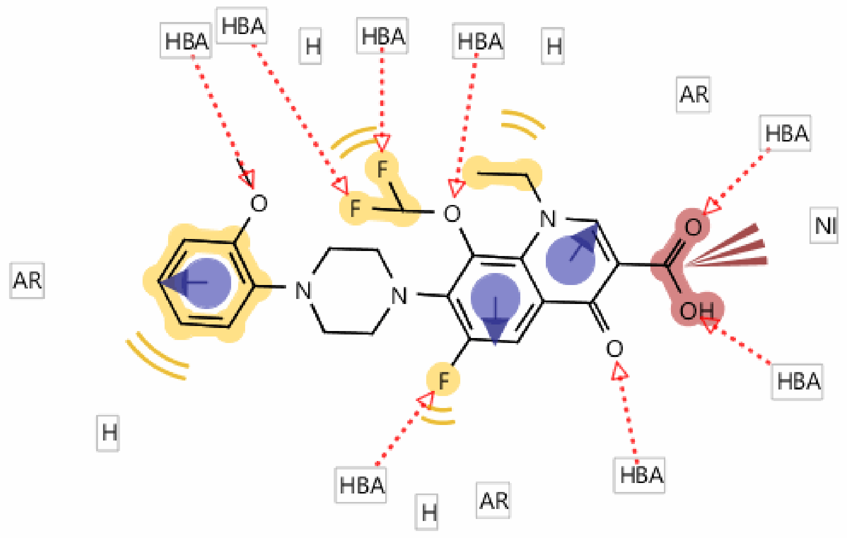
KB 5246	119474-55-4	YNPLQSCXSPGFPU-UHFFFAOYSA-N	InChI=1S/C18H16FN3O4S.ClH/c1-20-2-4-21(5-3-20)14-11(19)6-10-13-16(14)26-7-9-8-27-17(22(9)13)12(15(10)23)18(24)25;/h6,8H,2-5,7H2,1H3,(H,24,25);1H	CN1CCN(CC1)C2=C(C=C3C4=C2OCC(N45)=CSC5=C(C3=O)C(O)=O)F.Cl	DNA gyrase and topoisomerase IV inhibitor	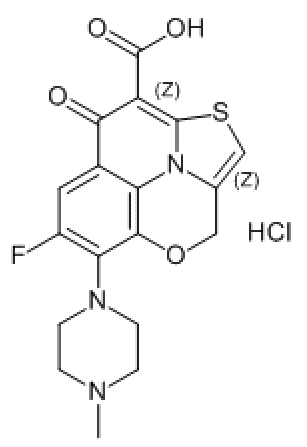	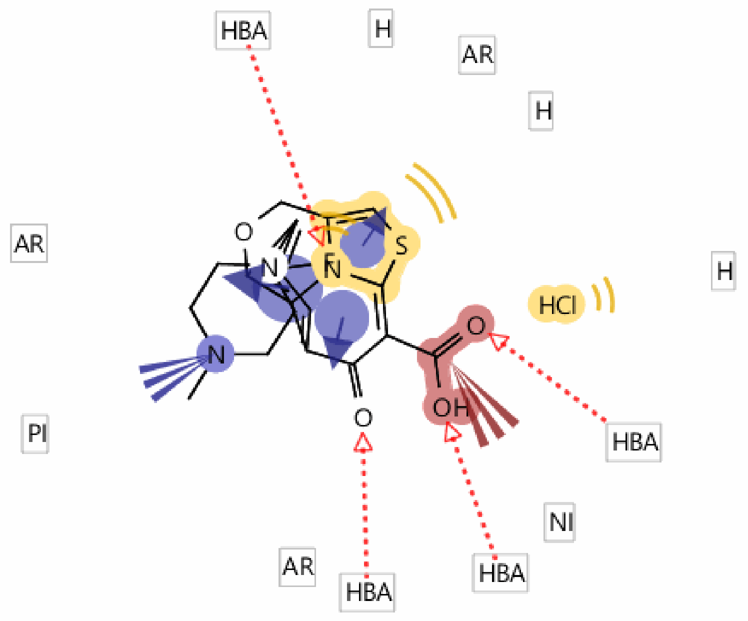
KPI 10 free base (WQ 3810)	888032-58-4	MJMVUQNNSOBCGF-UHFFFAOYSA-N	InChI=1S/C22H22F3N5O3/c1-9(2)27-11-6-29(7-11)18-10(3)17-12(4-14(18)23)19(31)13(22(32)33)8-30(17)21-16(25)5-15(24)20(26)28-21/h4-5,8-9,11,27H,6-7H2,1-3H3,(H2,26,28)(H,32,33)	CC(C)NC1CN(C1)C2=C(C=C(C3=C2C)C(C(C(O)=O)=CN3C4=C(C=C(C(N)=N4)F)F)=O)F	DNA gyrase and topoisomerase IV inhibitor	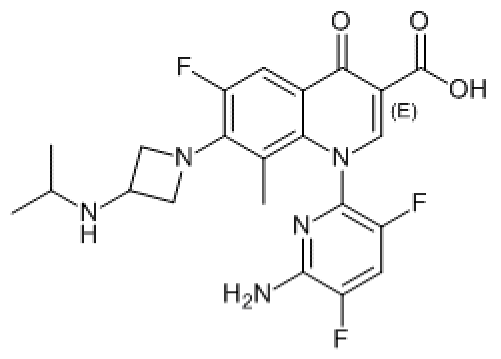	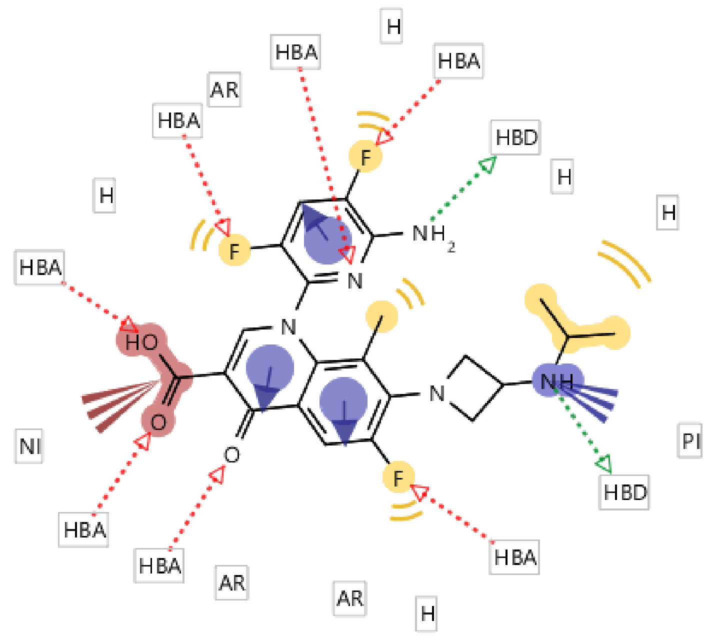
Lascufloxacin	848416-07-9	ZFIOCUITTUUVPV-MEDUHNTESA-N	InChI=1S/C21H24F3N3O4/c1-31-20-17-13(19(28)14(21(29)30)9-26(17)5-4-22)6-15(23)18(20)27-8-11(16(24)10-27)7-25-12-2-3-12/h6,9,11-12,16,25H,2-5,7-8,10H2,1H3,(H,29,30)/t11-,16+/m0/s1	COC1=C2C(C(C(C(O)=O)=CN2CCF)=O)=CC(F)=C1N(C[C@@H]3CNC4CC4)C[C@H]3F	DNA gyrase and topoisomerase IV inhibitor	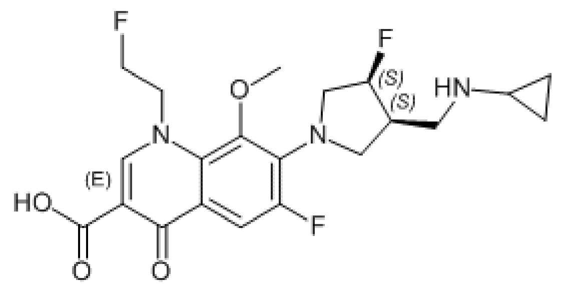	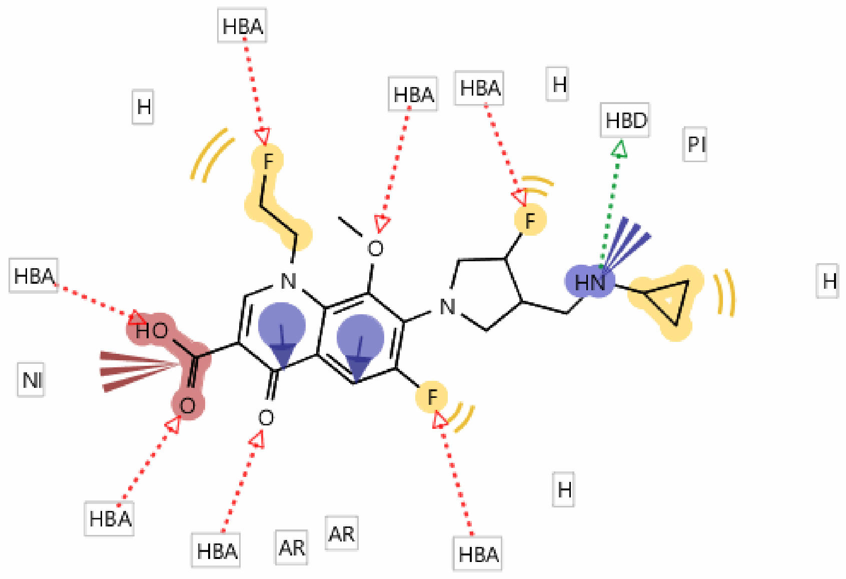
Levofloxacin	100986-85-4	GSDSWSVVBLHKDQ-JTQLQIEISA-N	InChI=1S/C18H20FN3O4/c1-10-9-26-17-14-11(16(23)12(18(24)25)8-22(10)14)7-13(19)15(17)21-5-3-20(2)4-6-21/h7-8,10H,3-6,9H2,1-2H3,(H,24,25)/t10-/m0/s1	C[C@H]1COC2=C3C(C(C(C(O)=O)=CN13)=O)=CC(F)=C2N4CCN(C)CC4	DNA gyrase and topoisomerase IV inhibitor	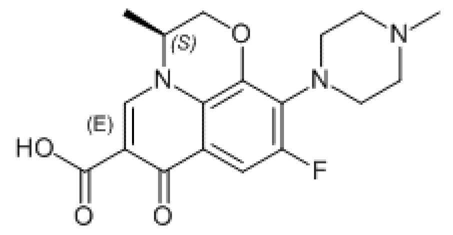	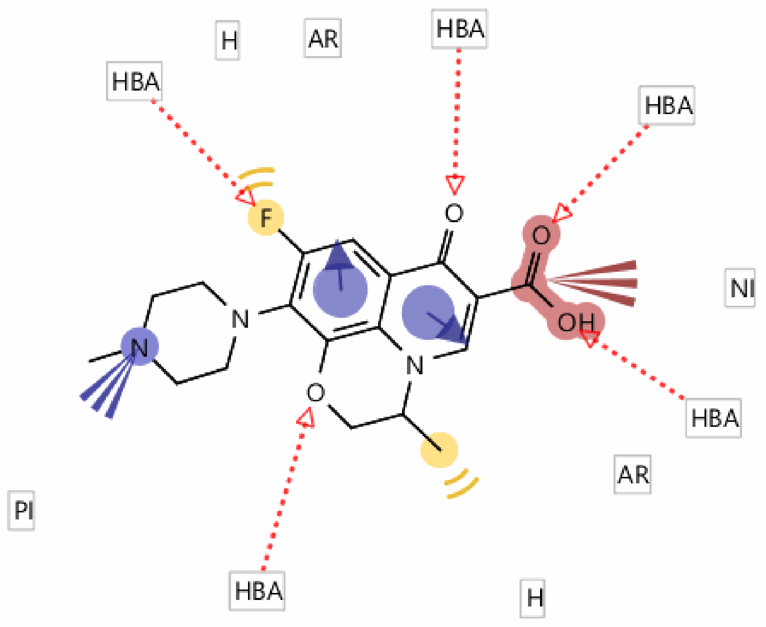

Common name	CAS Reg. No.	InChIKey	InChI	SMILES	Primary target	2D Structure	Pharmacophore[1]
Levonadifloxacin	154357-42-3	JYJTVFIEFKZWCJ-JTQLQIEISA-N	InChI=1S/C19H21FN2O4/c1-10-2-3-12-16-13(18(24)14(19(25)26)9-22(10)16)8-15(20)17(12)21-6-4-11(23)5-7-21/h8-11,23H,2-7H2,1H3,(H,25,26)/t10-/m0/s1	C[C@H]1CCC2=C3C(C(C(C(O)=O)=CN13)=O)=CC(F)=C2N4CCC(CC4)O	DNA gyrase and topoisomerase IV inhibitor	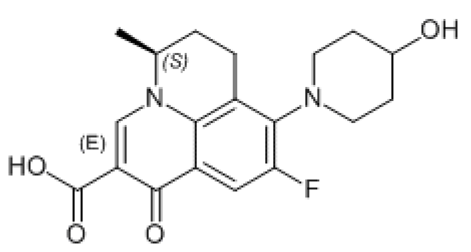	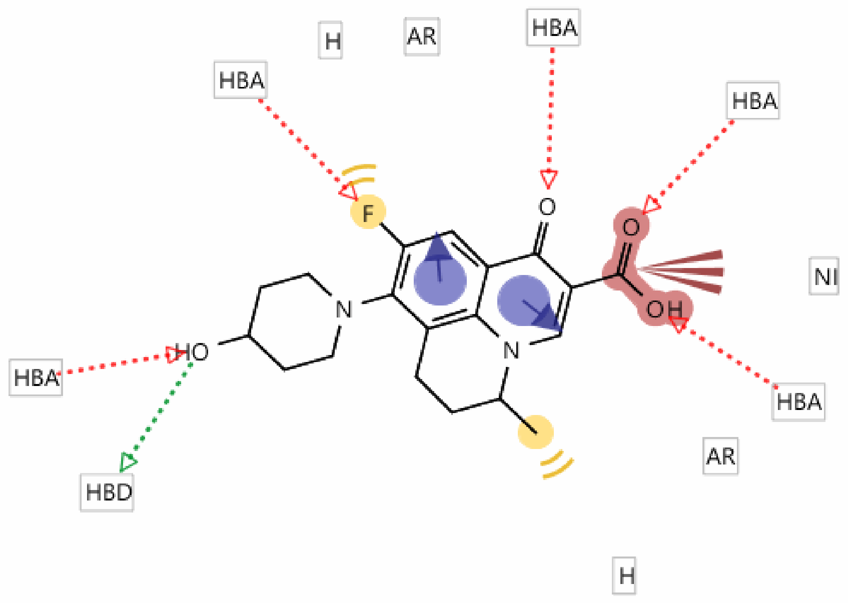
Levonadifloxacin arginine (WCK 771)	306748-89-0	WIFOPRFVQIVWTB-YBVIJTQESA-N	InChI=1S/C19H21FN2O4.C6H14N4O2/c1-10-2-3-12-16-13(18(24)14(19(25)26)9-22(10)16)8-15(20)17(12)21-6-4-11(23)5-7-21;7-4(5(11)12)2-1-3-10-6(8)9/h8-11,23H,2-7H2,1H3,(H,25,26);4H,1-3,7H2,(H,11,12)(H4,8,9,10)/t10-;4-/m00/s1	C[C@H]1CCC2=C3C(C(C(C(O)=O)=CN13)=O)=CC(F)=C2N4CCC(CC4)O.N[C@@H](CCCNC(N)=N)C(O)=O	DNA gyrase and topoisomerase IV inhibitor	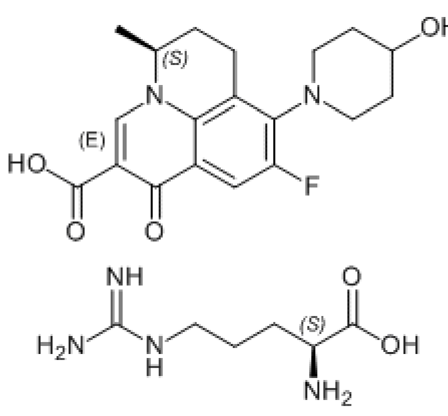	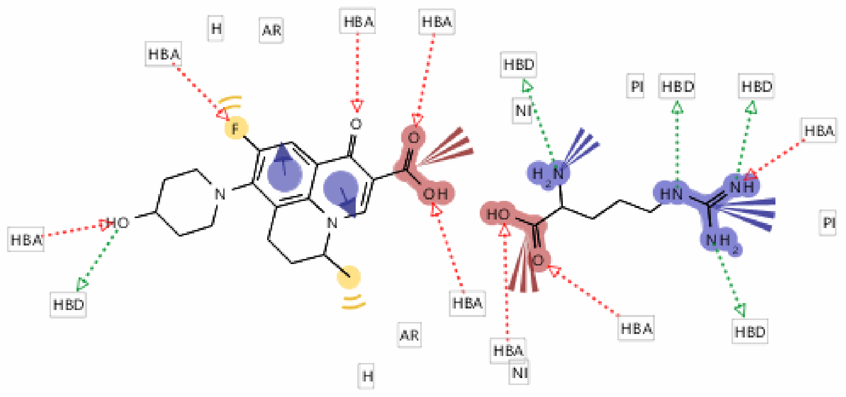
Lomefloxacin	98079-51-7	PQAYUIDIWWNEFR-IGXPJQGBSA-N	InChI=1S/2C17H19F2N3O3/c2*1-3-21-8-11(17(24)25)16(23)10-6-12(18)15(13(19)14(10)21)22-5-4-20-9(2)7-22/h2*6,8-9,20H,3-5,7H2,1-2H3,(H,24,25)/t2*9-/m10/s1	CCN1C=C(C(O)=O)C(C2=CC(F)=C(N3CCN[C@@H](C3)C)C(F)=C21)=O.CCN4C=C(C(O)=O)C(C5=CC(F)=C(N6CCN[C@H](C6)C)C(F)=C54)=O	DNA gyrase and topoisomerase IV inhibitor	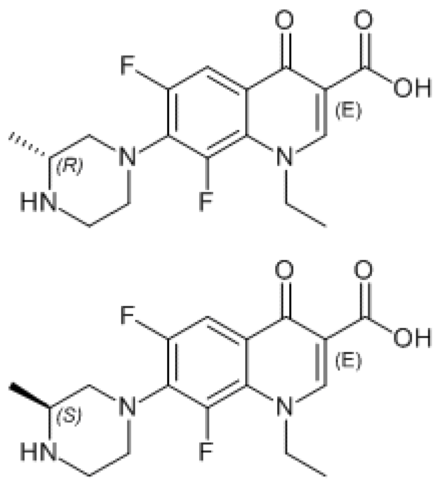	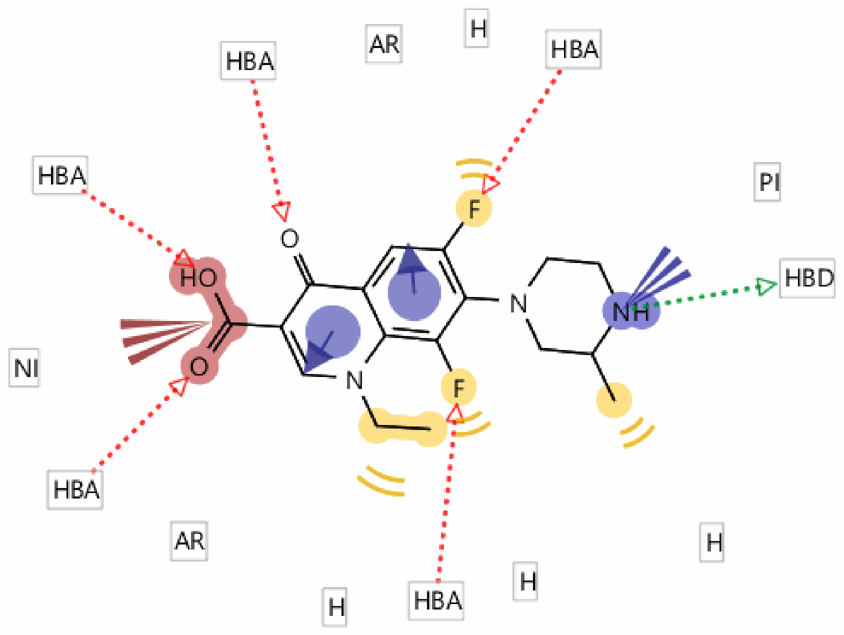
Marbofloxacin	115550-35-1	BPFYOAJNDMUVBL-UHFFFAOYSA-N	InChI=1S/C17H19FN4O4/c1-19-3-5-21(6-4-19)14-12(18)7-10-13-16(14)26-9-20(2)22(13)8-11(15(10)23)17(24)25/h7-8H,3-6,9H2,1-2H3,(H,24,25)	CN1CCN(C2=C(F)C=C(C(C(C(O)=O)=CN34)=O)C3=C2OCN4C)CC1	DNA gyrase and topoisomerase IV inhibitor	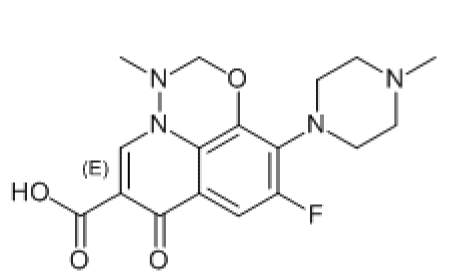	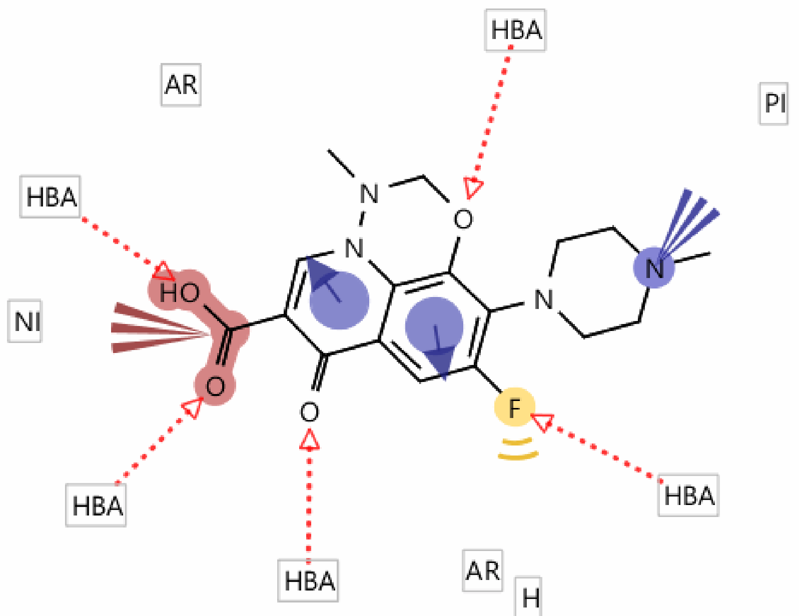
Merafloxacin	110013-21-3	IDEPESJEIWLDQG-FGYXOPSTSA-N	InChI=1S/2C19H23F2N3O3/c2*1-3-22-8-11-5-6-24(9-11)17-14(20)7-12-16(15(17)21)23(4-2)10-13(18(12)25)19(26)27/h2*7,10-11,22H,3-6,8-9H2,1-2H3,(H,26,27)/t2*11-/m10/s1	CCNC[C@H]1CCN(C1)C2=C(C=C(C3=C2F)C(C(C(O)=O)=CN3CC)=O)F.CCNC[C@@H]4CCN(C4)C5=C(C=C(C6=C5F)C(C(C(O)=O)=CN6CC)=O)F	DNA gyrase and topoisomerase IV inhibitor	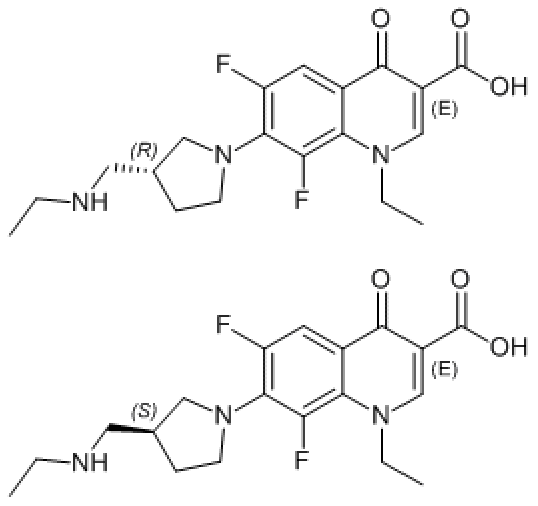	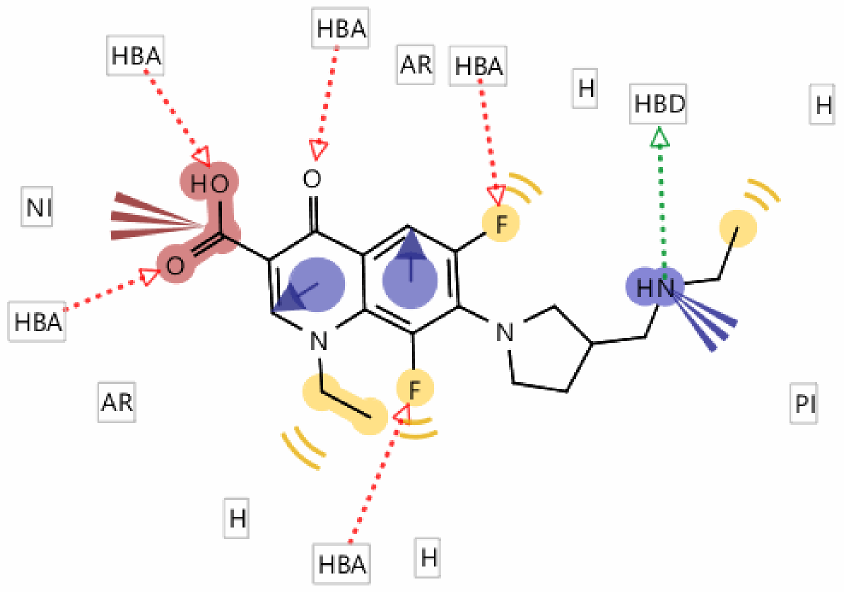
Metioxate	42110-58-7	IXQKKLAOJKWQCM-UHFFFAOYSA-N	InChI=1S/C22H27N3O4S/c1-4-25-13-15(21(27)29-12-11-24-9-7-14(2)8-10-24)19(26)18-16(25)5-6-17-20(18)30-22(28)23(17)3/h5-6,13-14H,4,7-12H2,1-3H3	CCN(C1=C2C(S3)=C(N(C3=O)C)C=C1)C=C(C(OCCN4CCC(CC4)C)=O)C2=O	DNA gyrase and topoisomerase IV inhibitor	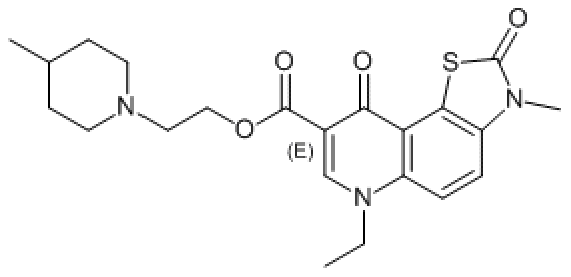	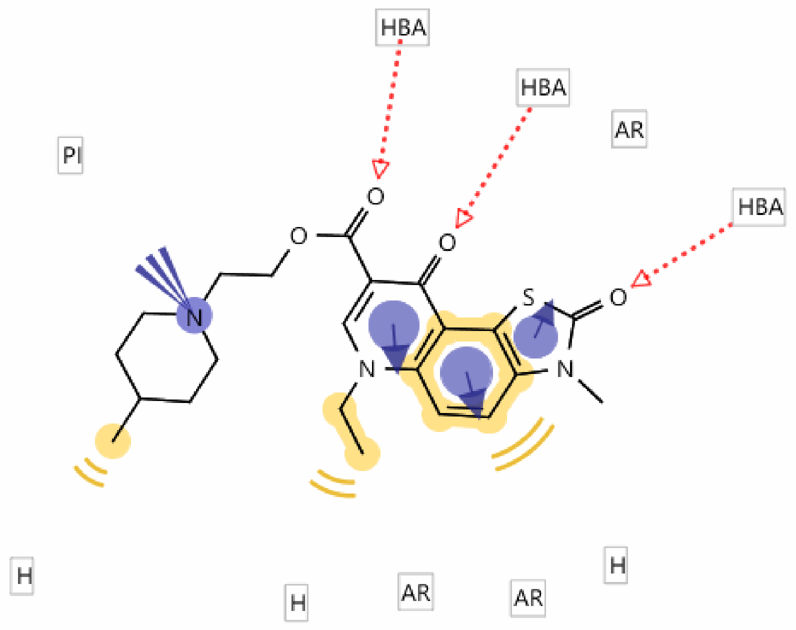

Common name	CAS Reg. No.	InChIKey	InChI	SMILES	Primary target	2D Structure	Pharmacophore[1]
MF 5101		OLAGJLWUNWVRMJ-UHFFFAOYSA-N	InChI=1S/C18H22N4O3/c1-20-4-6-21(7-5-20)16-9-15-12(8-14(16)19)17(23)13(18(24)25)10-22(15)11-2-3-11/h8-11H,2-7,19H2,1H3,(H,24,25)	CN1CCN(C2=C(N)C=C(C(C(C(O)=O)=CN3C4CC4)=O)C3=C2)CC1	DNA gyrase and topoisomerase IV inhibitor	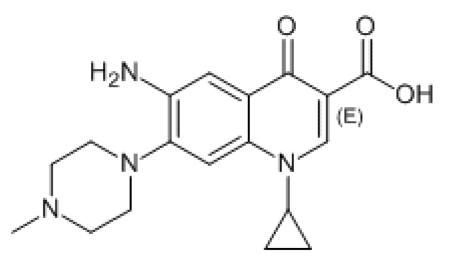	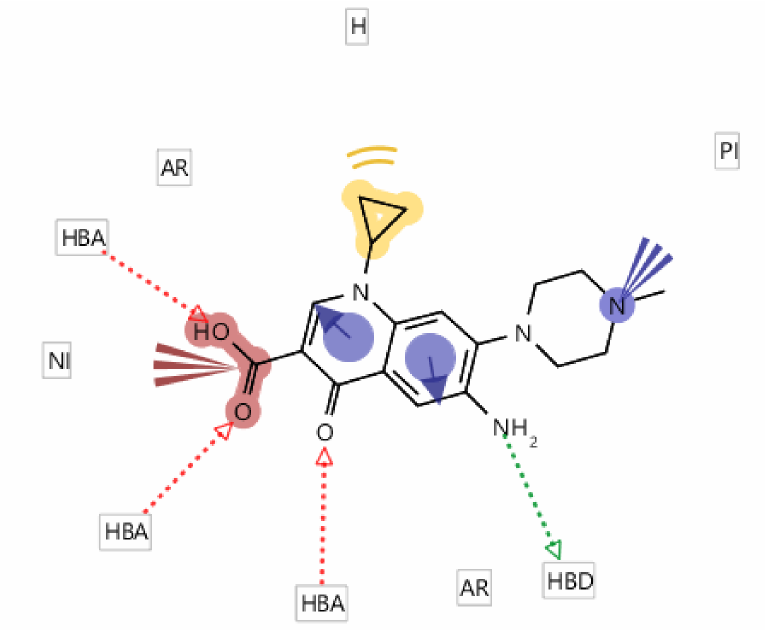
MF 5103		PMFKJVDGMKVQDN-UHFFFAOYSA-N	InChI=1S/C18H21FN4O3/c1-21-4-6-22(7-5-21)16-13(20)8-11-15(14(16)19)23(10-2-3-10)9-12(17(11)24)18(25)26/h8-10H,2-7,20H2,1H3,(H,25,26)	CN1CCN(C2=C(N)C=C(C(C(C(O)=O)=CN3C4CC4)=O)C3=C2F)CC1	DNA gyrase and topoisomerase IV inhibitor	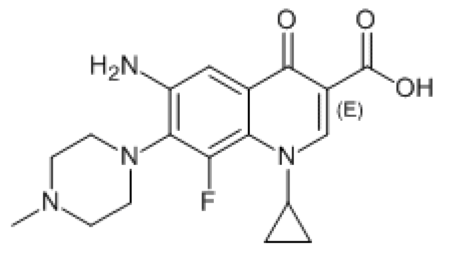	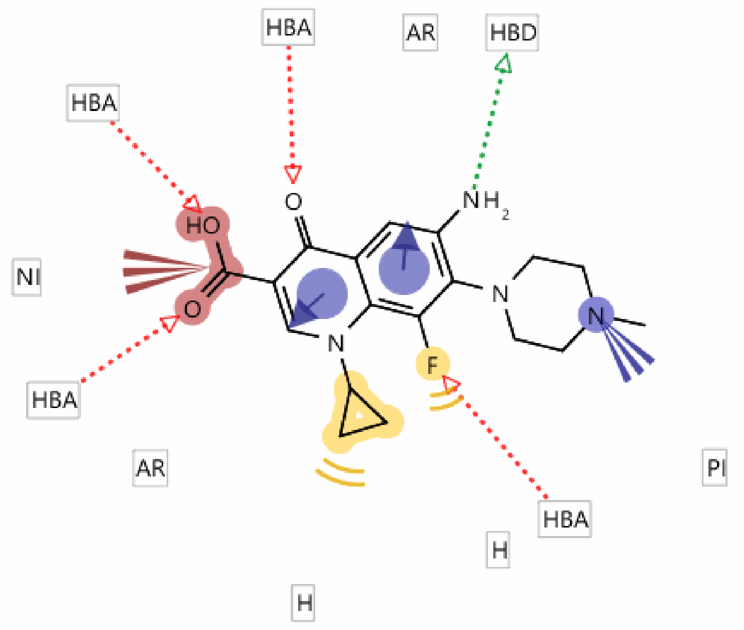
MF 5112 free base		SWTDLXUESQTGOR-UHFFFAOYSA-N	InChI=1S/C17H21N5O3/c1-20-4-6-21(7-5-20)16-13(18)8-11-14(23)12(17(24)25)9-22(10-2-3-10)15(11)19-16/h8-10H,2-7,18H2,1H3,(H,24,25)	CN1CCN(CC1)C2=C(C=C(C(C(C(O)=O)=CN3C4CC4)=O)C3=N2)N	DNA gyrase and topoisomerase IV inhibitor	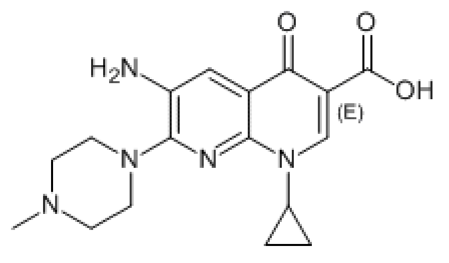	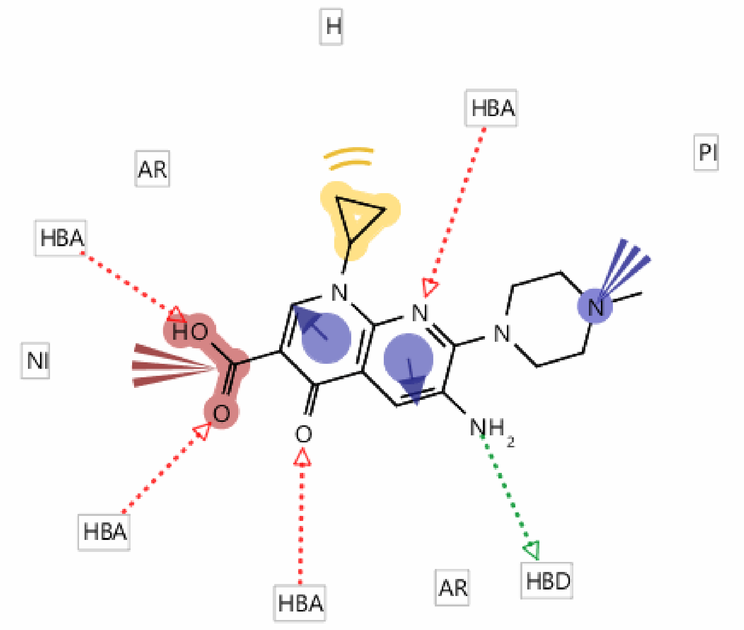
MF 5126	148927-12-2	CHVILWZHRHTRGR-UHFFFAOYSA-N	InChI=1S/C17H18FN3O3S/c18-13-14-10(7-12(19)15(13)20-3-5-25-6-4-20)16(22)11(17(23)24)8-21(14)9-1-2-9/h7-9H,1-6,19H2,(H,23,24)	FC1=C2C(C(C(C(O)=O)=CN2C3CC3)=O)=CC(N)=C1N4CCSCC4	DNA gyrase and topoisomerase IV inhibitor	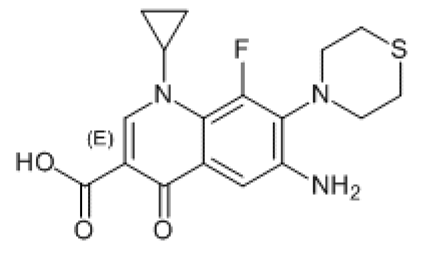	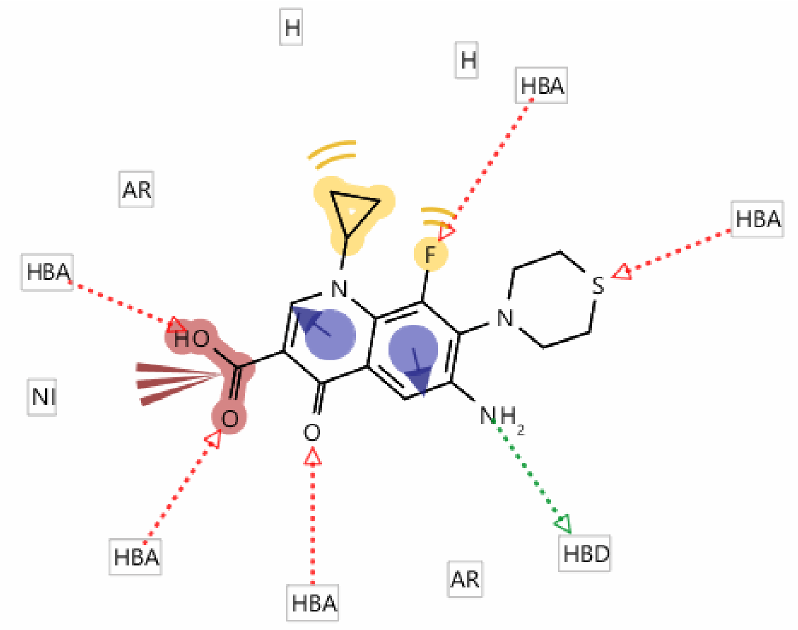
MF 5137		LLVLMYRWABCVEU-UHFFFAOYSA-N	InChI=1S/C23H23N3O3/c1-13-20-17(22(27)18(23(28)29)12-26(20)16-6-7-16)10-19(24)21(13)25-9-8-14-4-2-3-5-15(14)11-25/h2-5,10,12,16H,6-9,11,24H2,1H3,(H,28,29)	CC1=C2C(C(C(C(O)=O)=CN2C3CC3)=O)=CC(N)=C1N4CCC5=CC=CC=C5C4	DNA gyrase and topoisomerase IV inhibitor	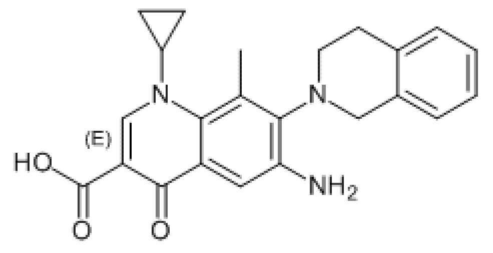	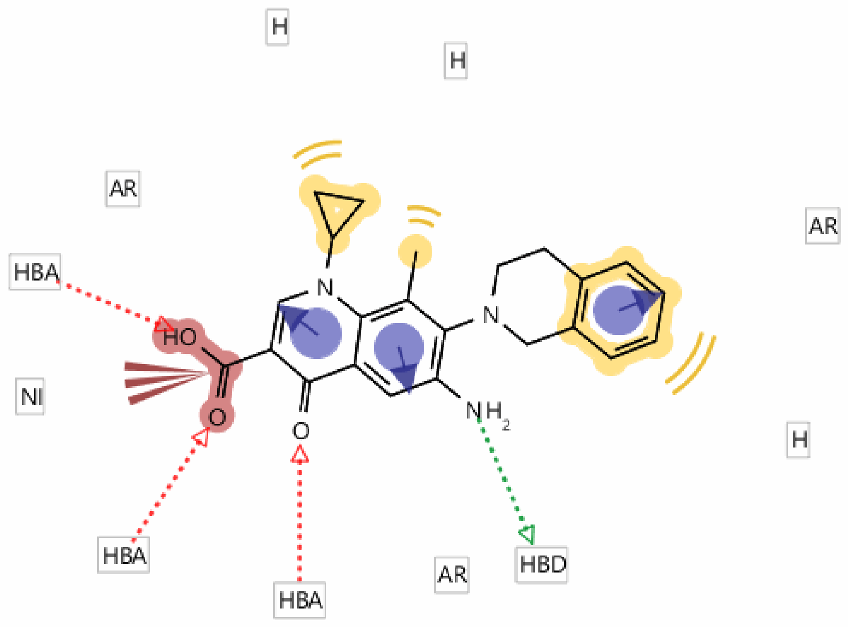
MF 5143		PJOOZALYLNTBAW-UHFFFAOYSA-N	InChI=1S/C19H23N3O3/c1-11-16-13(9-15(20)17(11)21-7-3-2-4-8-21)18(23)14(19(24)25)10-22(16)12-5-6-12/h9-10,12H,2-8,20H2,1H3,(H,24,25)	CC1=C2C(C(C(C(O)=O)=CN2C3CC3)=O)=CC(N)=C1N4CCCCC4	DNA gyrase and topoisomerase IV inhibitor	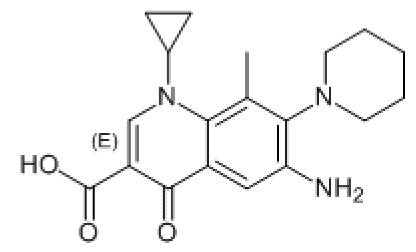	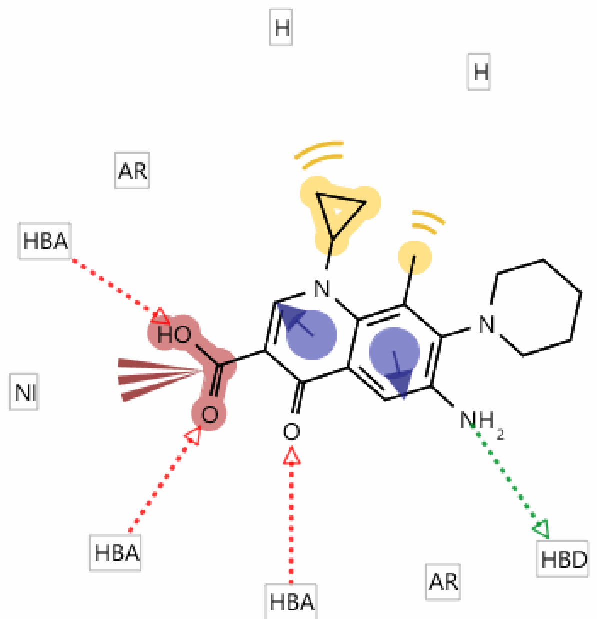

Common name	CAS Reg. No.	InChIKey	InChI	SMILES	Primary target	2D Structure	Pharmacophore[1]
MF 5168	93106-68-4	QUJXLLBTRBWHJW-UHFFFAOYSA-N	InChI=1S/C18H20N4O5/c1-19-4-6-20(7-5-19)15-9-14-12(8-16(15)22(26)27)17(23)13(18(24)25)10-21(14)11-2-3-11/h8-11H,2-7H2,1H3,(H,24,25)	CN1CCN(C2=C([N+]([O-])=O)C=C3C(N(C4CC4)C=C(C(O)=O)C3=O)=C2)CC1	DNA gyrase and topoisomerase IV inhibitor	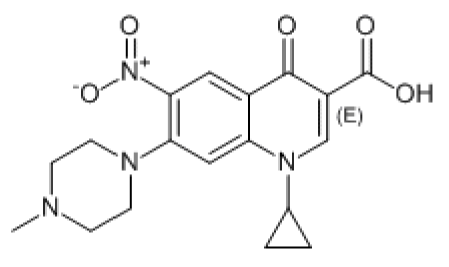	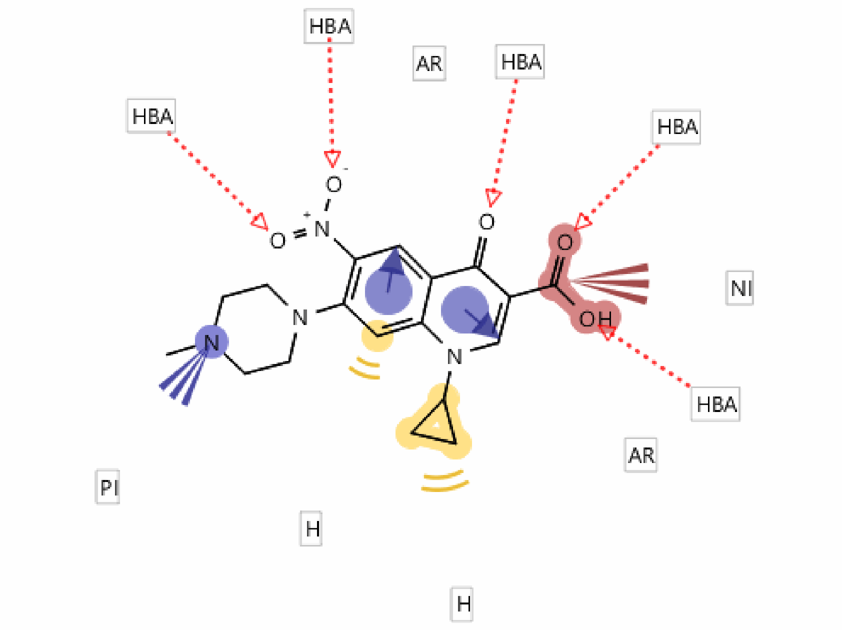
Miloxacin	37065-29-5	ABQYZRZVRIPTPI-UHFFFAOYSA-N	InChI=1S/C12H9NO6/c1-17-13-4-7(12(15)16)11(14)6-2-9-10(3-8(6)13)19-5-18-9/h2-4H,5H2,1H3,(H,15,16)	CON1C=C(C(C2=CC3=C(C=C21)OCO3)=O)C(O)=O	DNA gyrase and topoisomerase IV inhibitor	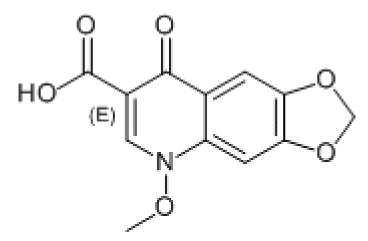	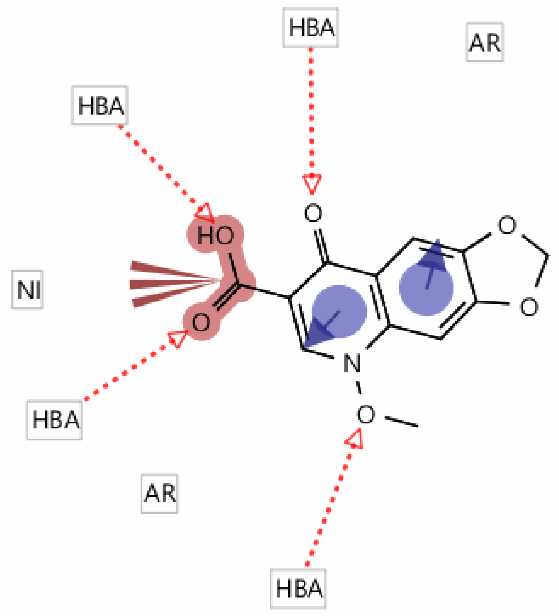
Moxifloxacin	151096-09-2	FABPRXSRWADJSP-MEDUHNTESA-N	InChI=1S/C21H24FN3O4/c1-29-20-17-13(19(26)14(21(27)28)9-25(17)12-4-5-12)7-15(22)18(20)24-8-11-3-2-6-23-16(11)10-24/h7,9,11-12,16,23H,2-6,8,10H2,1H3,(H,27,28)/t11-,16+/m0/s1	COC1=C2C(C(C(C(O)=O)=CN2C3CC3)=O)=CC(F)=C1N4C[C@]5([H])CCCN[C@]5([H])C4	DNA gyrase and topoisomerase IV inhibitor	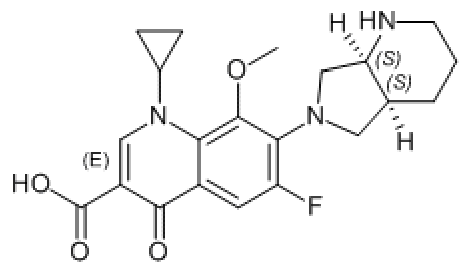	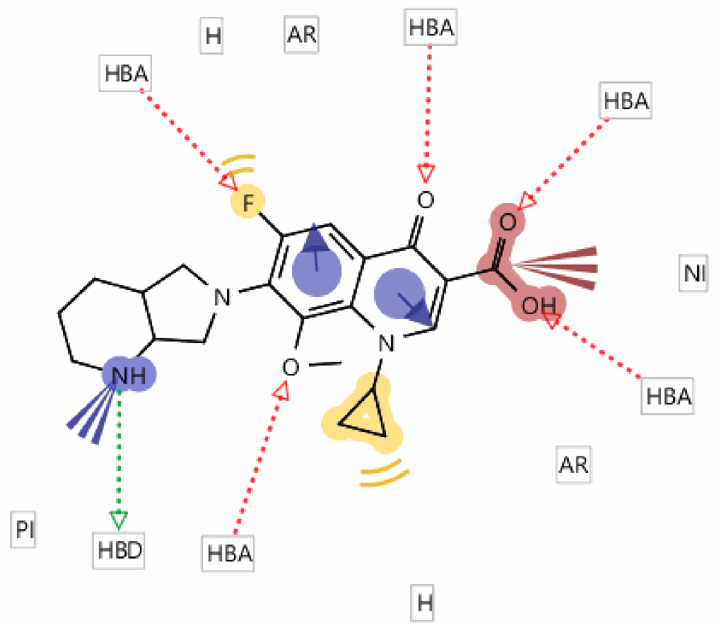
Nadifloxacin	124858-35-1	MYCSIOHFFGBUFS-FTYBWHBYSA-N	InChI=1S/2C19H21FN2O4/c2*1-10-2-3-12-16-13(18(24)14(19(25)26)9-22(10)16)8-15(20)17(12)21-6-4-11(23)5-7-21/h2*8-11,23H,2-7H2,1H3,(H,25,26)/t2*10-/m10/s1	C[C@@H]1CCC2=C3C(C(C(C(O)=O)=CN13)=O)=CC(F)=C2N4CCC(CC4)O.C[C@H]5CCC6=C7C(C(C(C(O)=O)=CN57)=O)=CC(F)=C6N8CCC(CC8)O	DNA gyrase and topoisomerase IV inhibitor	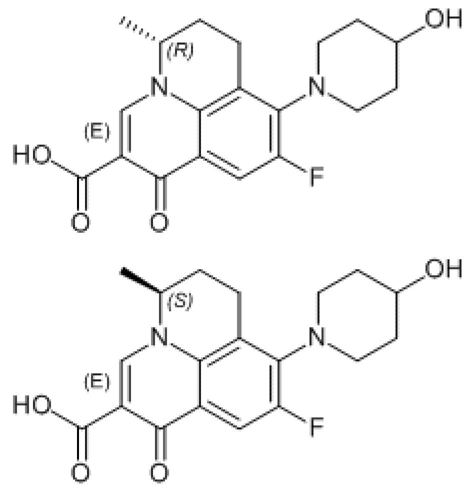	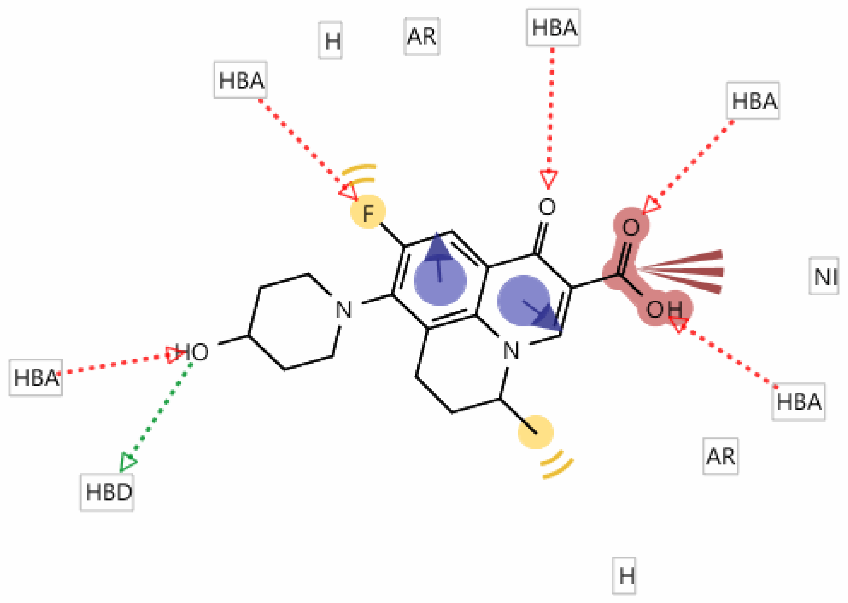
Nalidixic acid	389-08-2	MHWLWQUZZRMNGJ-UHFFFAOYSA-N	InChI=1S/C12H12N2O3/c1-3-14-6-9(12(16)17)10(15)8-5-4-7(2)13-11(8)14/h4-6H,3H2,1-2H3,(H,16,17)	CCN1C=C(C(C2=C1N=C(C)C=C2)=O)C(O)=O	DNA gyrase and topoisomerase IV inhibitor	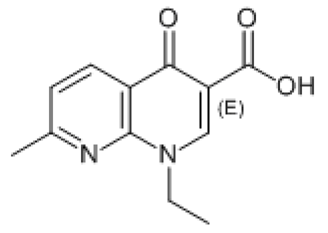	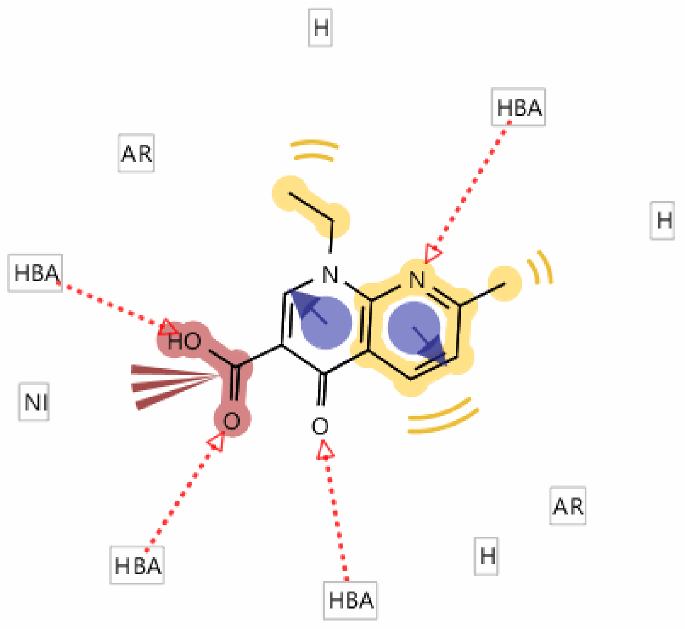
Nemonoxacin	378746-64-6	AVPQPGFLVZTJOR-RYUDHWBXSA-N	InChI=1S/C20H25N3O4/c1-11-7-12(21)9-22(8-11)16-6-5-14-17(19(16)27-2)23(13-3-4-13)10-15(18(14)24)20(25)26/h5-6,10-13H,3-4,7-9,21H2,1-2H3,(H,25,26)/t11-,12-/m0/s1	C[C@H]1C[C@@H](CN(C1)C2=C(C3=C(C=C2)C(C(C(O)=O)=CN3C4CC4)=O)OC)N	DNA gyrase and topoisomerase IV inhibitor	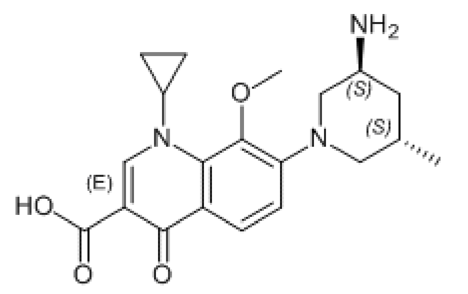	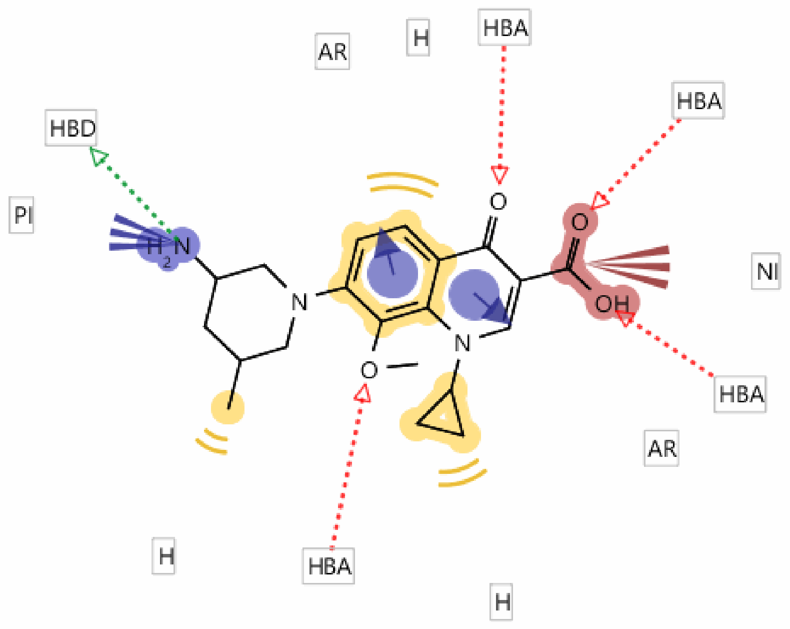

Common name	CAS Reg. No.	InChIKey	InChI	SMILES	Primary target	2D Structure	Pharmacophore[1]
Norfloxacin	70458-96-7	OGJPXUAPXNRGGI-UHFFFAOYSA-N	InChI=1S/C16H18FN3O3/c1-2-19-9-11(16(22)23)15(21)10-7-12(17)14(8-13(10)19)20-5-3-18-4-6-20/h7-9,18H,2-6H2,1H3,(H,22,23)	CCN1C=C(C(O)=O)C(C2=CC(F)=C(N3CCNCC3)C=C21)=O	DNA gyrase and topoisomerase IV inhibitor	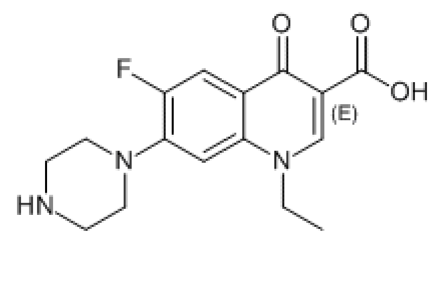	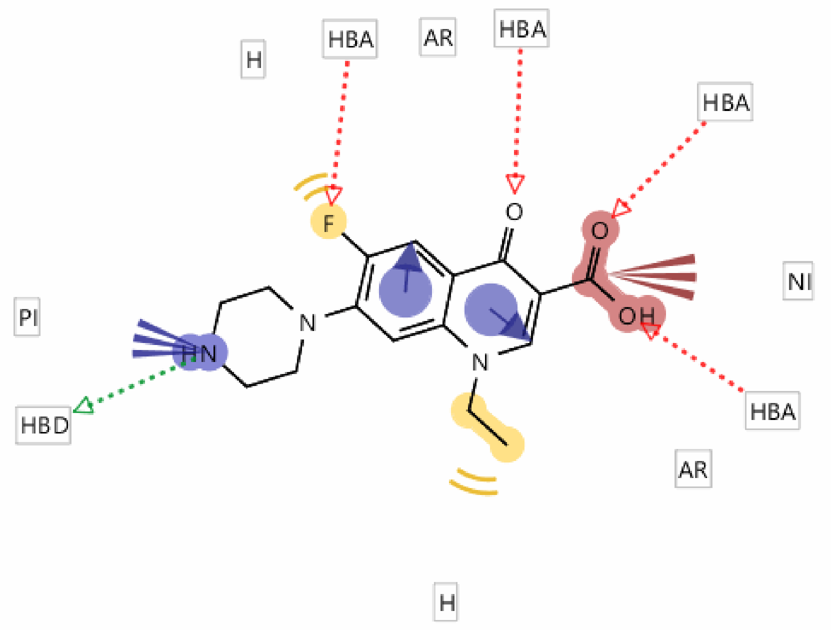
NSFQ 104	154269-13-3	HHYYBLLPAATIIQ-UHFFFAOYSA-N	InChI=1S/C22H23FN4O5S/c1-2-25-13-17(22(29)30)21(28)16-11-18(23)20(12-19(16)25)26-7-9-27(10-8-26)33(31,32)15-5-3-14(24)4-6-15/h3-6,11-13H,2,7-10,24H2,1H3,(H,29,30)	CCN1C=C(C(O)=O)C(C2=CC(F)=C(N3CCN(S(C4=CC=C(N)C=C4)(=O)=O)CC3)C=C21)=O	DNA gyrase and topoisomerase IV inhibitor	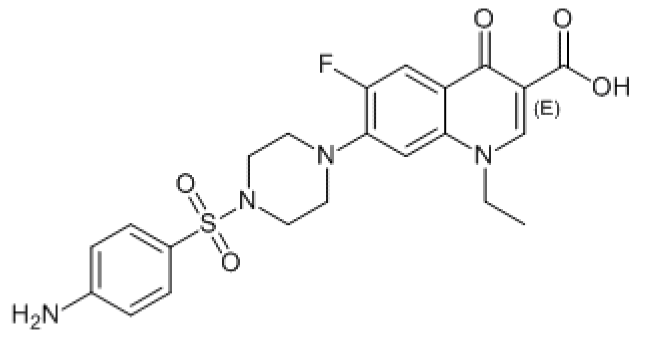	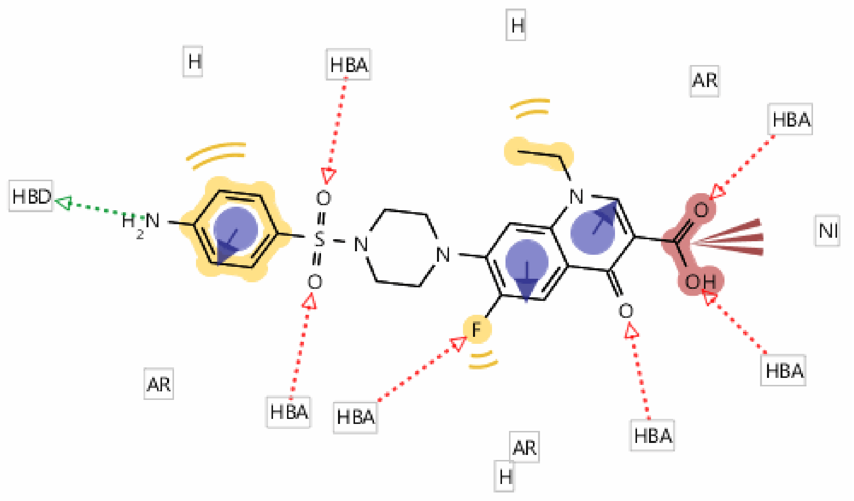
NSFQ 105	154269-12-2	LHQCNYXFOFNLNV-UHFFFAOYSA-N	InChI=1S/C23H23FN4O5S/c24-19-11-17-20(28(15-3-4-15)13-18(22(17)29)23(30)31)12-21(19)26-7-9-27(10-8-26)34(32,33)16-5-1-14(25)2-6-16/h1-2,5-6,11-13,15H,3-4,7-10,25H2,(H,30,31)	FC1=C(C=C2C(C(C(C(O)=O)=CN2C3CC3)=O)=C1)N4CCN(CC4)S(C5=CC=C(C=C5)N)(=O)=O	DNA gyrase and topoisomerase IV inhibitor	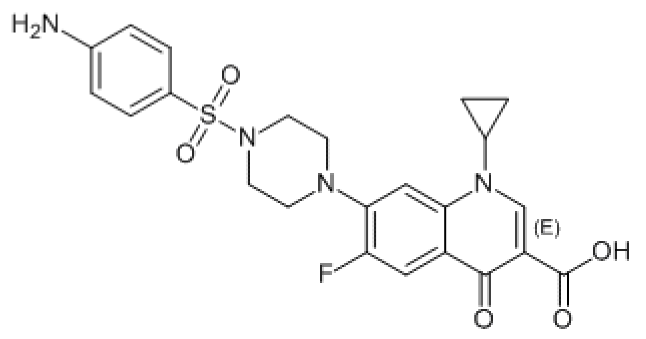	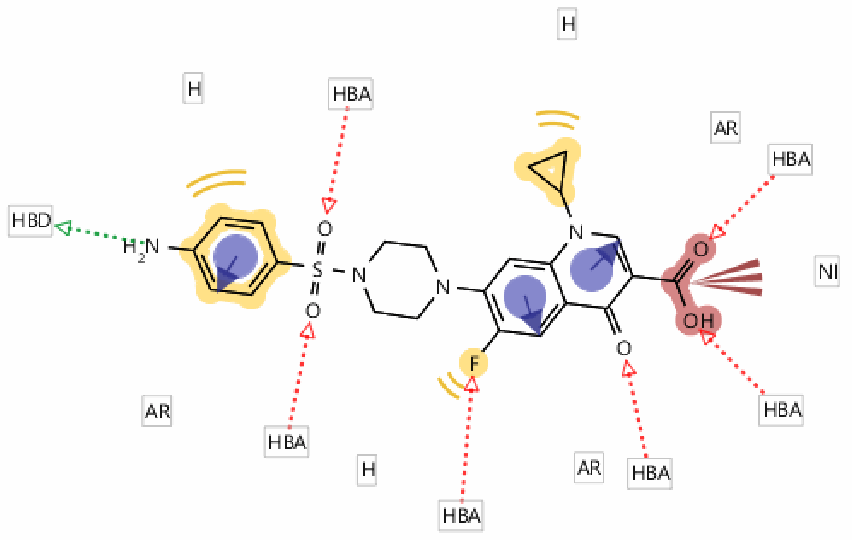
Ofloxacin	82419-36-1	BADKREAVONENKJ-FTYBWHBYSA-N	InChI=1S/2C18H20FN3O4/c2*1-10-9-26-17-14-11(16(23)12(18(24)25)8-22(10)14)7-13(19)15(17)21-5-3-20(2)4-6-21/h2*7-8,10H,3-6,9H2,1-2H3,(H,24,25)/t2*10-/m10/s1	C[C@@H]1COC2=C3C(C(C(C(O)=O)=CN13)=O)=CC(F)=C2N4CCN(C)CC4.C[C@H]5COC6=C7C(C(C(C(O)=O)=CN57)=O)=CC(F)=C6N8CCN(C)CC8	DNA gyrase and topoisomerase IV inhibitor	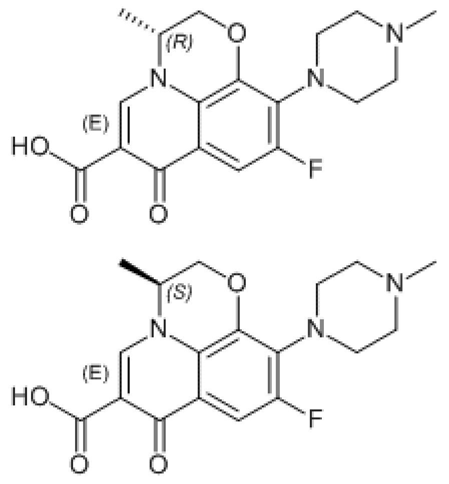	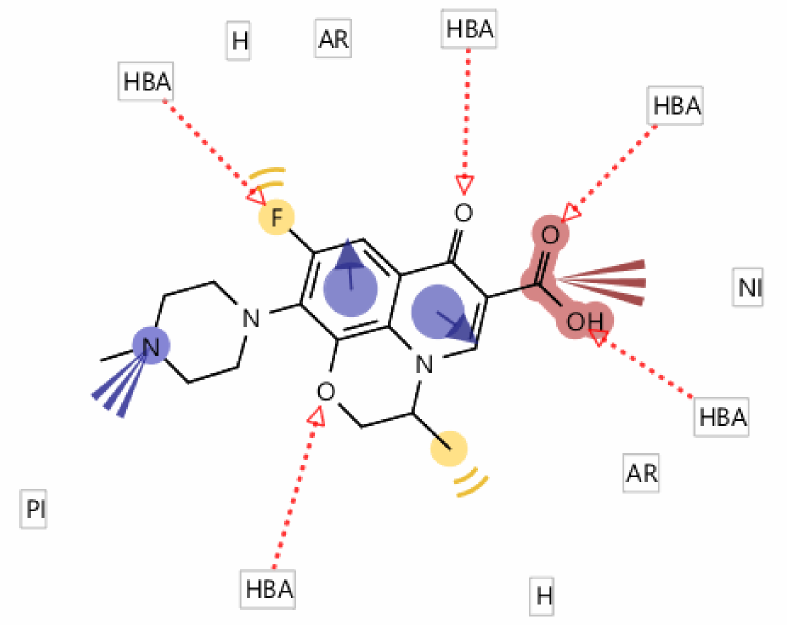
Olamufloxacin	167887-97-0	LEILBPMISZFZQK-GFCCVEGCSA-N	InChI=1S/C20H23FN4O3/c1-9-16-13(18(26)11(19(27)28)6-25(16)10-2-3-10)15(23)14(21)17(9)24-7-12(22)20(8-24)4-5-20/h6,10,12H,2-5,7-8,22-23H2,1H3,(H,27,28)/t12-/m1/s1	CC1=C2C(C(C(C(O)=O)=CN2C3CC3)=O)=C(C(F)=C1N4C[C@H](C5(CC5)C4)N)N	DNA gyrase and topoisomerase IV inhibitor	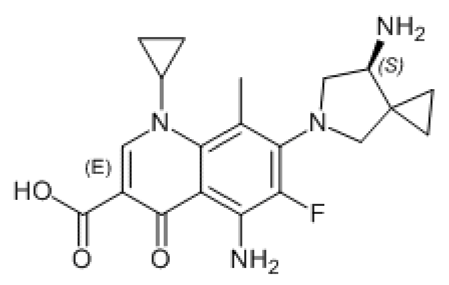	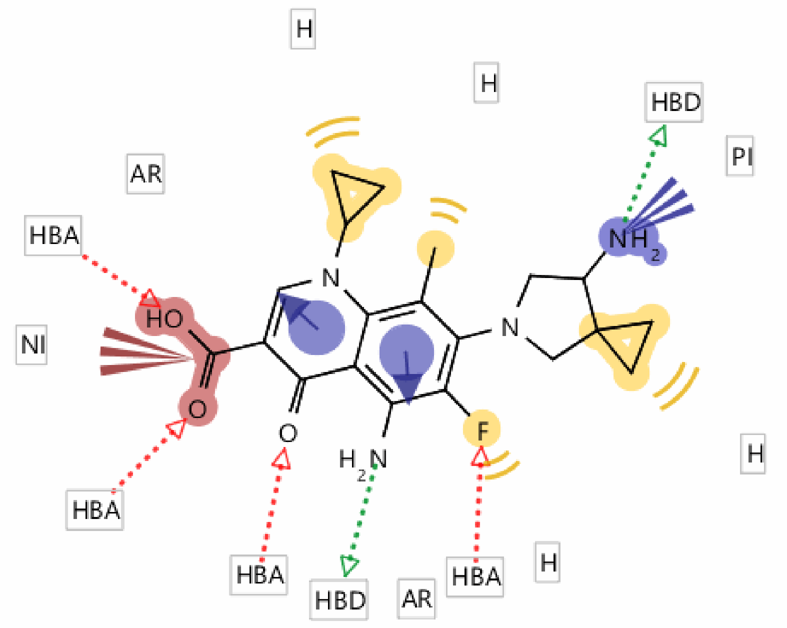
Orbifloxacin	113617-63-3	QIPQASLPWJVQMH-DTORHVGOSA-N	InChI=1S/C19H20F3N3O3/c1-8-5-24(6-9(2)23-8)17-14(21)13(20)12-16(15(17)22)25(10-3-4-10)7-11(18(12)26)19(27)28/h7-10,23H,3-6H2,1-2H3,(H,27,28)/t8-,9+	C[C@@H]1N[C@H](C)CN(C2=C(C(F)=C(C3=C2F)C(C(C(O)=O)=CN3C4CC4)=O)F)C1	DNA gyrase and topoisomerase IV inhibitor	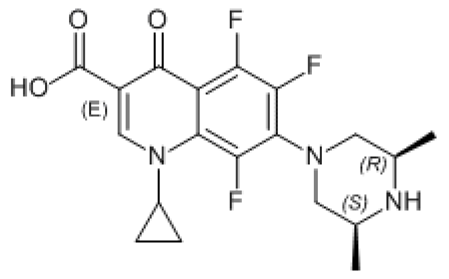	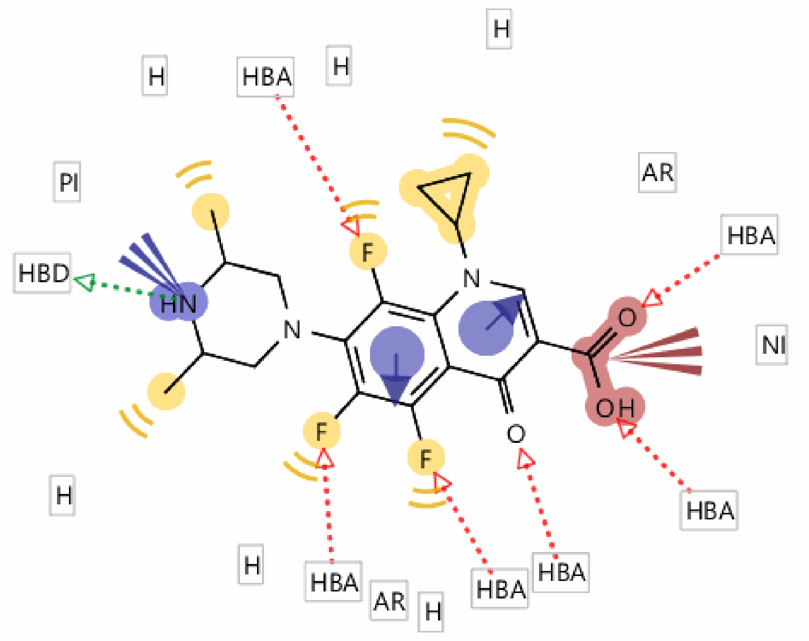

Common name	CAS Reg. No.	InChIKey	InChI	SMILES	Primary target	2D Structure	Pharmacophore[1]
Oxolinic acid	14698-29-4	KYGZCKSPAKDVKC-UHFFFAOYSA-N	InChI=1S/C13H11NO5/c1-2-14-5-8(13(16)17)12(15)7-3-10-11(4-9(7)14)19-6-18-10/h3-5H,2,6H2,1H3,(H,16,17)	CCN1C=C(C(O)=O)C(C2=CC(OCO3)=C3C=C21)=O	DNA gyrase and topoisomerase IV inhibitor	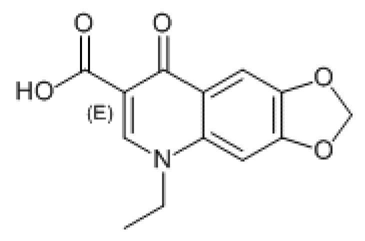	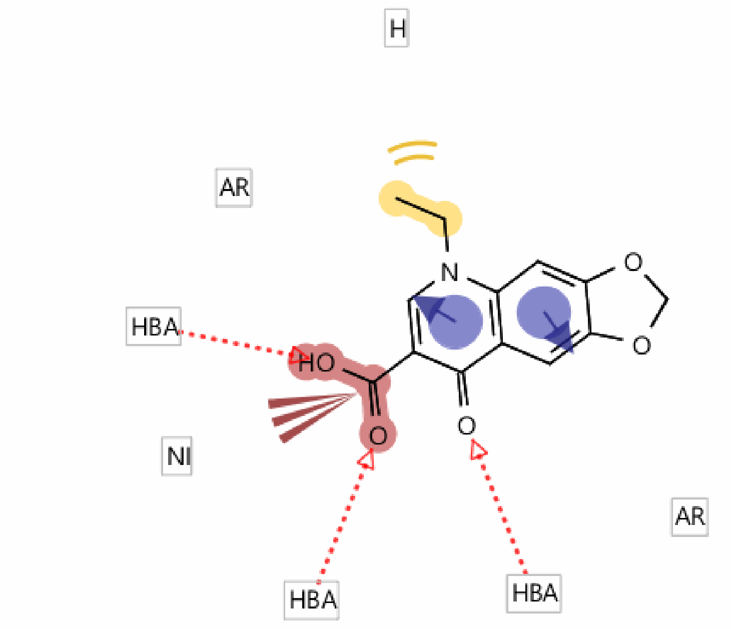
Ozenoxacin	245765-41-7	XPIJWUTXQAGSLK-UHFFFAOYSA-N	InChI=1S/C21H21N3O3/c1-11-8-13(9-23-20(11)22-3)15-6-7-16-18(12(15)2)24(14-4-5-14)10-17(19(16)25)21(26)27/h6-10,14H,4-5H2,1-3H3,(H,22,23)(H,26,27)	CC1=CC(C(C=CC2=C3N(C4CC4)C=C(C(O)=O)C2=O)=C3C)=CN=C1NC	DNA gyrase and topoisomerase IV inhibitor	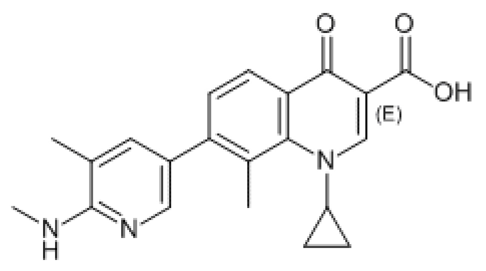	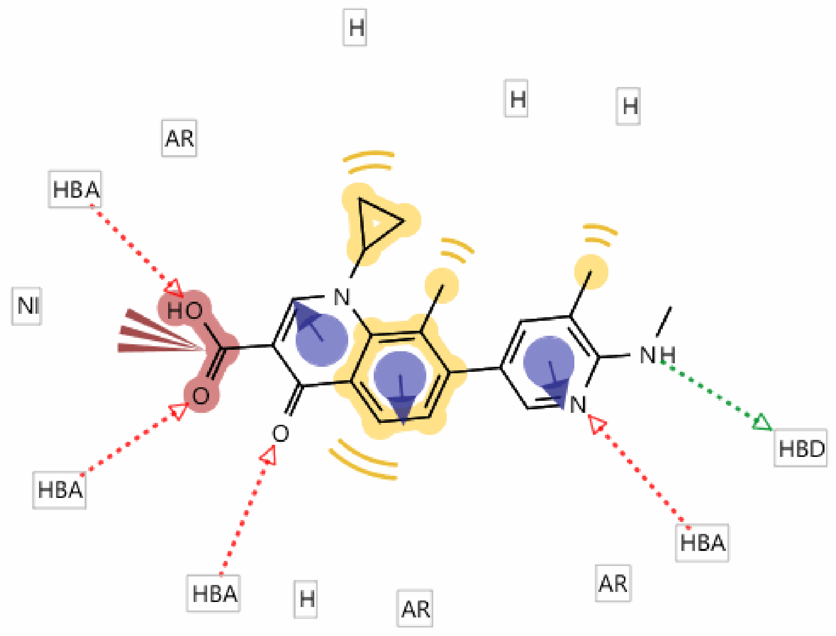
Pazufloxacin	127045-41-4	XAGMUUZPGZWTRP-ZETCQYMHSA-N	InChI=1S/C16H15FN2O4/c1-7-6-23-14-11(16(18)2-3-16)10(17)4-8-12(14)19(7)5-9(13(8)20)15(21)22/h4-5,7H,2-3,6,18H2,1H3,(H,21,22)/t7-/m0/s1	C[C@H]1COC2=C3C(C(C(C(O)=O)=CN13)=O)=CC(F)=C2C4(CC4)N	DNA gyrase and topoisomerase IV inhibitor	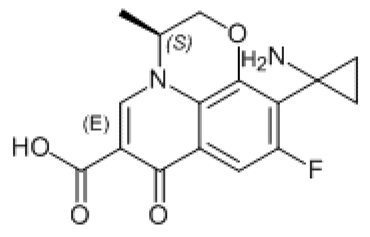	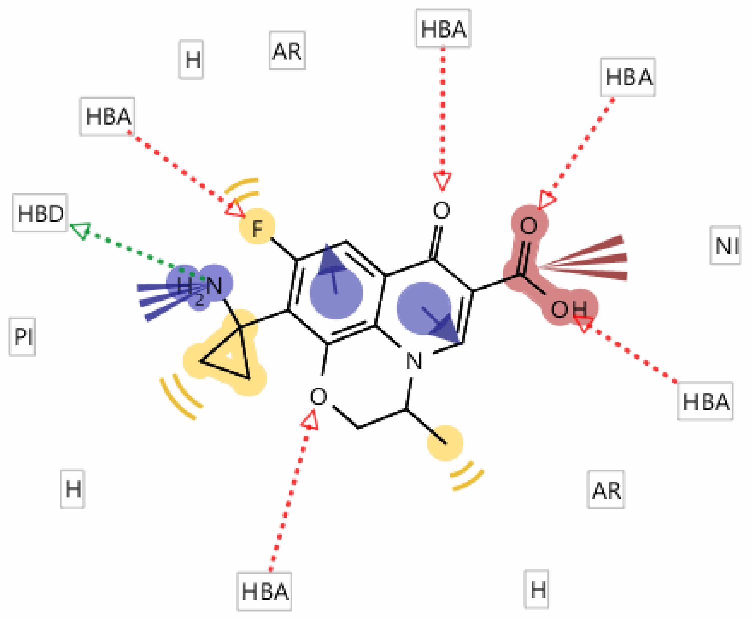
PD 111834		CCPFENJVIWXKFU-UHFFFAOYSA-N	InChI=1S/C14H14FN3O3S/c1-2-17-6-9(14(20)21)11(19)8-5-10(15)13(16-12(8)17)18-3-4-22-7-18/h5-6H,2-4,7H2,1H3,(H,20,21)	CCN1C=C(C(C2=CC(F)=C(N=C21)N3CCSC3)=O)C(O)=O	DNA gyrase and topoisomerase IV inhibitor	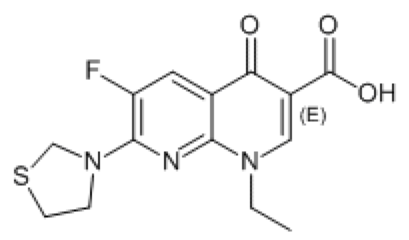	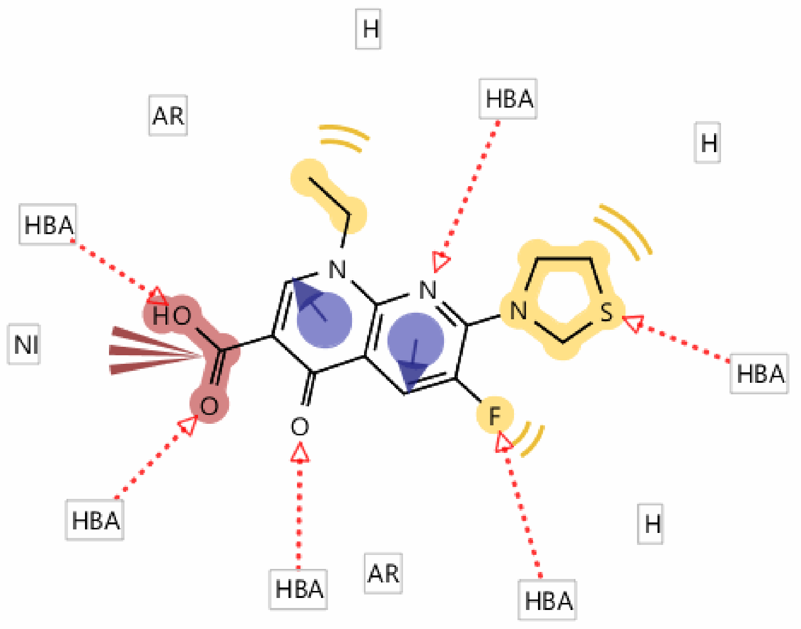
PD 112388		IWWJUVSVSYUGKY-UHFFFAOYSA-N	InChI=1S/C14H15FN2O3S/c1-2-17-7-9(14(19)20)13(18)8-5-10(15)12(6-11(8)17)21-4-3-16/h5-7H,2-4,16H2,1H3,(H,19,20)	CCN1C=C(C(O)=O)C(C2=CC(F)=C(SCCN)C=C21)=O	DNA gyrase and topoisomerase IV inhibitor	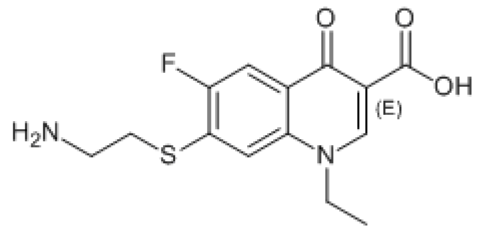	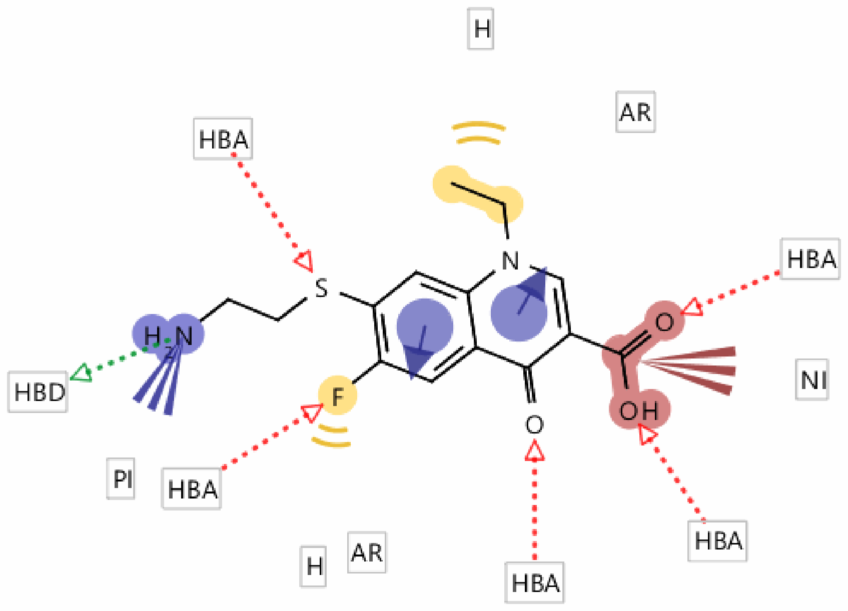
PD 114111		XMXSCUUDJMDYLC-UHFFFAOYSA-N	InChI=1S/C16H17FN2O3S/c1-2-18-9-11(16(21)22)15(20)10-7-12(17)14(8-13(10)18)19-3-5-23-6-4-19/h7-9H,2-6H2,1H3,(H,21,22)	CCN1C=C(C(O)=O)C(C2=CC(F)=C(N3CCSCC3)C=C21)=O	DNA gyrase and topoisomerase IV inhibitor	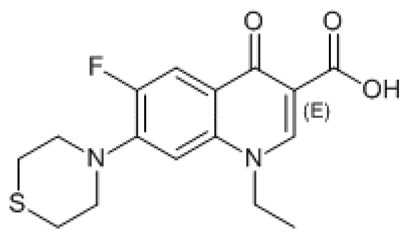	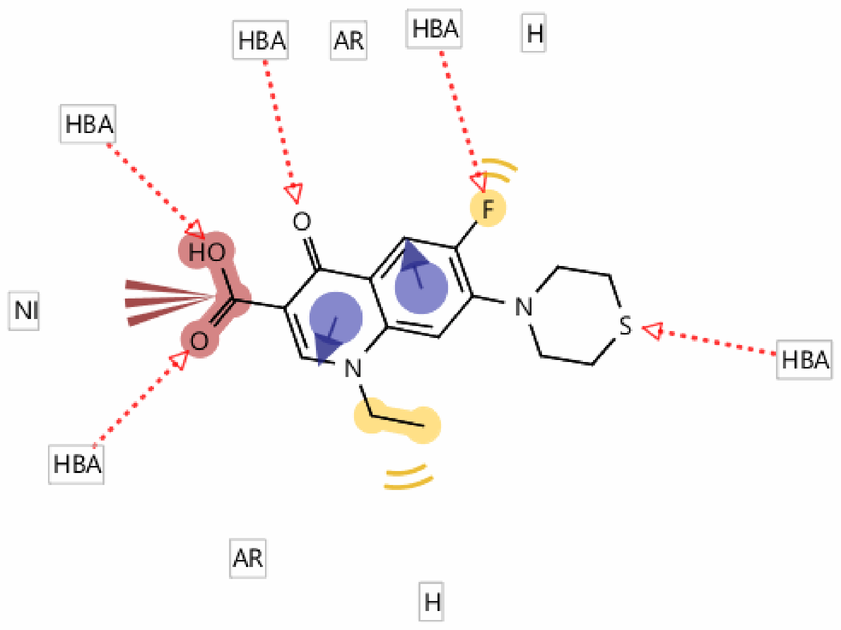

Common name	CAS Reg. No.	InChIKey	InChI	SMILES	Primary target	2D Structure	Pharmacophore[1]
PD 115311		SUVNNPSGSOXWOF-UHFFFAOYSA-N	InChI=1S/C16H16F2N2O4S/c1-2-19-8-10(16(22)23)15(21)9-7-11(17)14(12(18)13(9)19)20-3-5-25(24)6-4-20/h7-8H,2-6H2,1H3,(H,22,23)	O=C1C2=CC(F)=C(N3CC[S+]([O-])CC3)C(F)=C2N(CC)C=C1C(O)=O	DNA gyrase and topoisomerase IV inhibitor	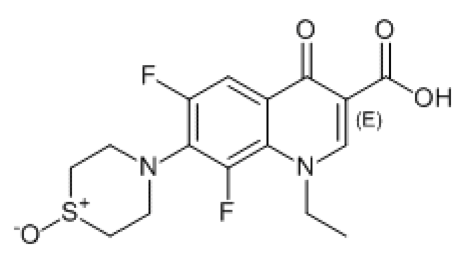	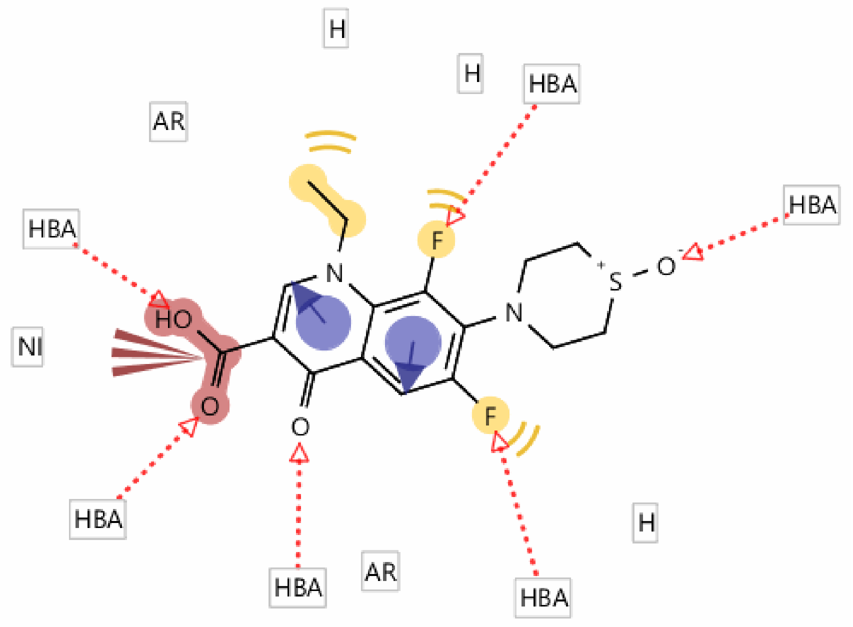
PD 116507		MOSUGPZHRCUMJE-UHFFFAOYSA-N	InChI=1S/C14H15FN4O3/c1-2-18-6-9(14(21)22)11(20)8-3-10(15)13(17-12(8)18)19-4-7(16)5-19/h3,6-7H,2,4-5,16H2,1H3,(H,21,22)	CCN1C=C(C(O)=O)C(C2=CC(F)=C(N3CC(N)C3)N=C21)=O	DNA gyrase and topoisomerase IV inhibitor	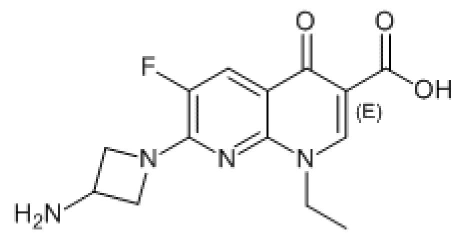	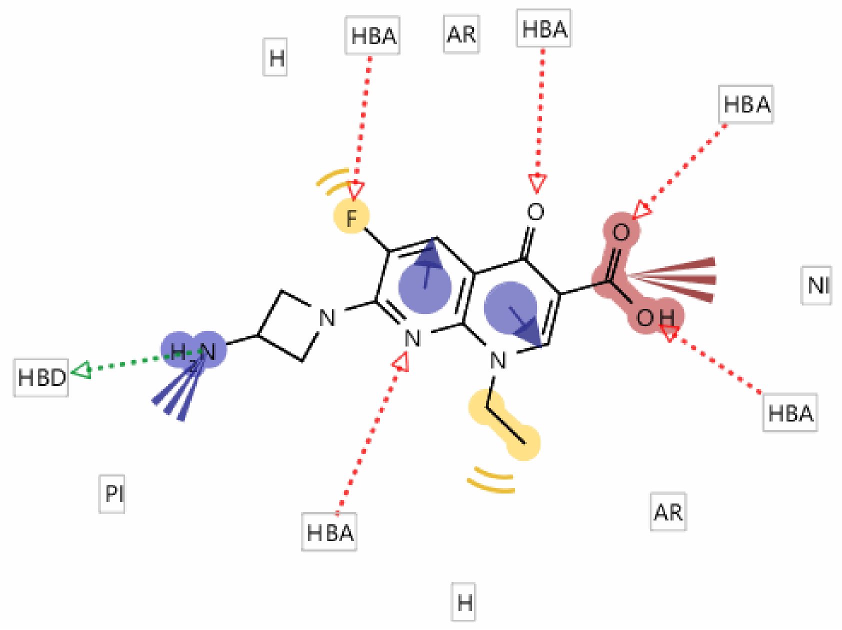
PD 117596	99734-98-2	SVQIBSLOCFSNNB-RMTNWKGQSA-N	InChI=1S/2C17H17F2N3O3/c2*18-12-5-10-14(13(19)15(12)21-4-3-8(20)6-21)22(9-1-2-9)7-11(16(10)23)17(24)25/h2*5,7-9H,1-4,6,20H2,(H,24,25)/t2*8-/m10/s1	FC1=C(C(F)=C2C(C(C(C(O)=O)=CN2C3CC3)=O)=C1)N4CC[C@H](C4)N.FC5=C(C(F)=C6C(C(C(C(O)=O)=CN6C7CC7)=O)=C5)N8CC[C@@H](C8)N	DNA gyrase and topoisomerase IV inhibitor	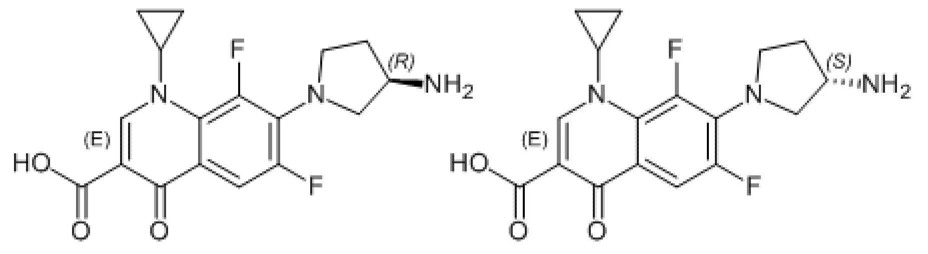	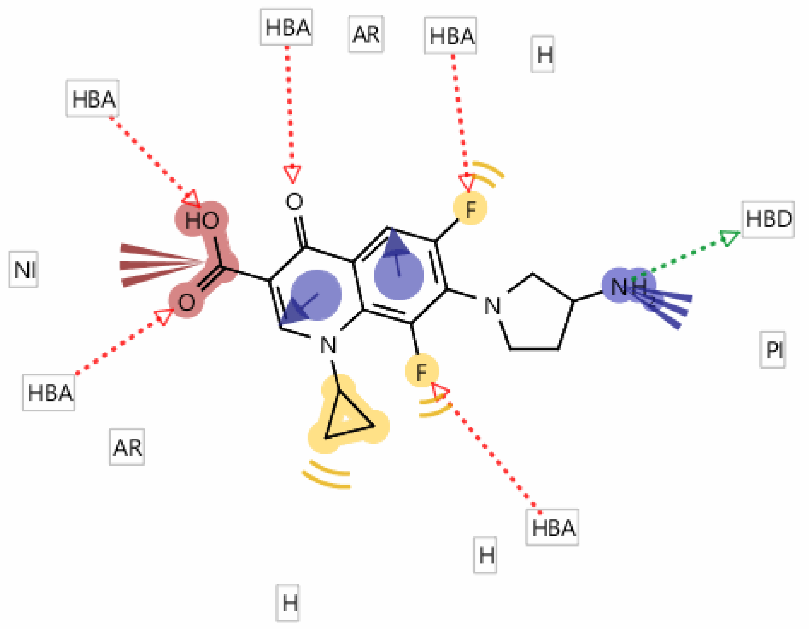
PD 118362		QMHKGLGDXMFABJ-JACLSRQLSA-N	InChI=1S/2C22H27F2N3O3/c2*1-2-25-10-13-7-8-26(11-13)20-17(23)9-15-19(18(20)24)27(14-5-3-4-6-14)12-16(21(15)28)22(29)30/h2*9,12-14,25H,2-8,10-11H2,1H3,(H,29,30)/t2*13-/m10/s1	CCNC[C@@H]1CCN(C1)C2=C(C=C3C(N(C=C(C3=O)C(O)=O)C4CCCC4)=C2F)F.CCNC[C@H]5CCN(C5)C6=C(C=C7C(N(C=C(C7=O)C(O)=O)C8CCCC8)=C6F)F	DNA gyrase and topoisomerase IV inhibitor	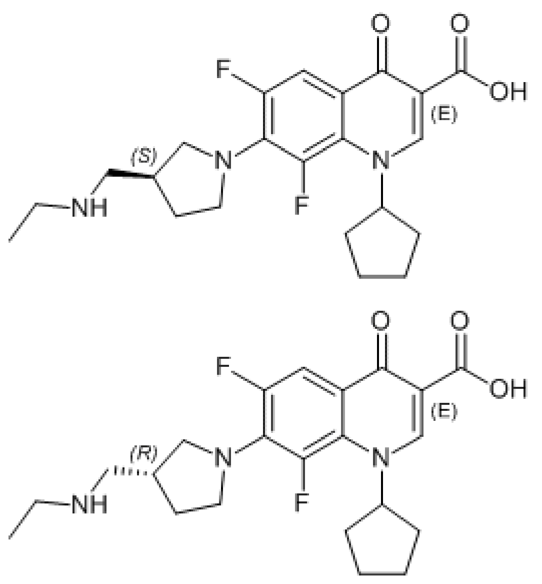	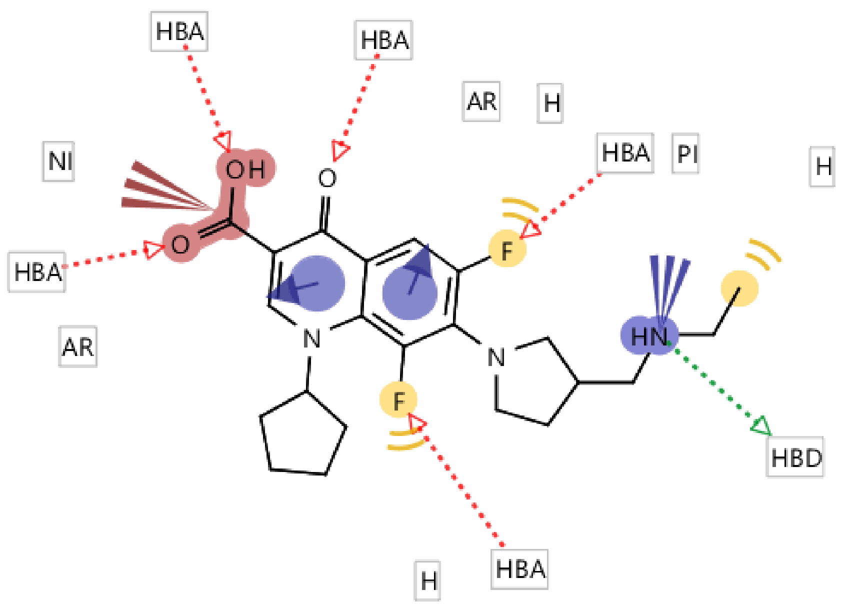
PD 119344		VCUHWVJKWOLLCR-JACLSRQLSA-N	InChI=1S/2C21H25F2N3O3/c2*1-2-24-8-13-5-6-25(10-13)19-16(22)7-14-18(17(19)23)26(9-12-3-4-12)11-15(20(14)27)21(28)29/h2*7,11-13,24H,2-6,8-10H2,1H3,(H,28,29)/t2*13-/m10/s1	CCNC[C@H]1CCN(C1)C2=C(C=C3C(N(C=C(C3=O)C(O)=O)CC4CC4)=C2F)F.CCNC[C@@H]5CCN(C5)C6=C(C=C7C(N(C=C(C7=O)C(O)=O)CC8CC8)=C6F)F	DNA gyrase and topoisomerase IV inhibitor	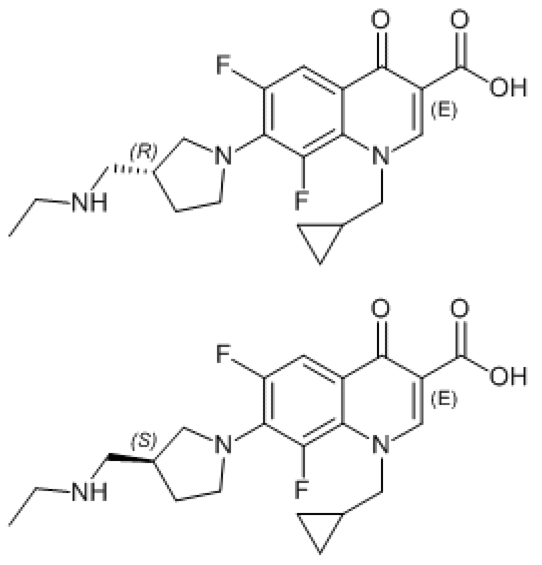	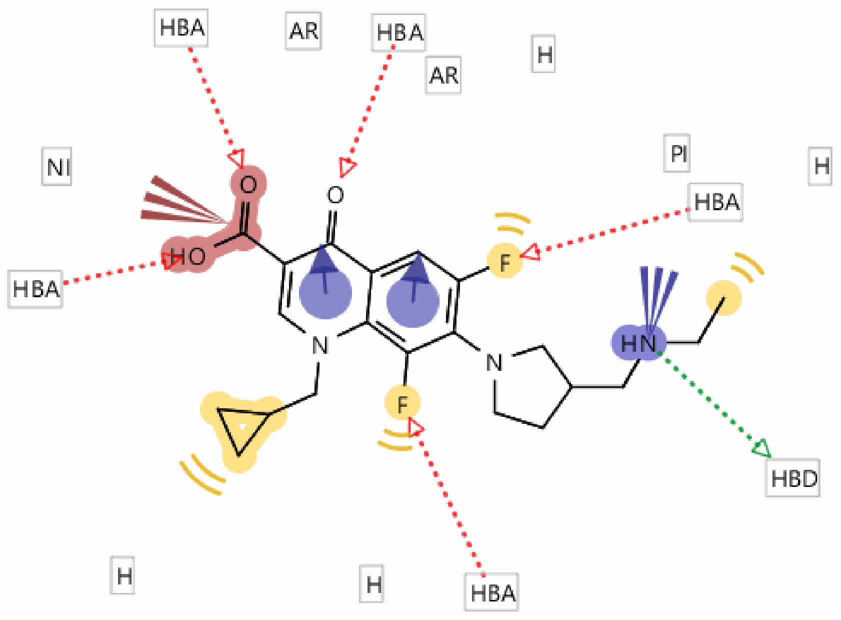
PD 129626		BDALLOYHJRARDE-FGYXOPSTSA-N	InChI=1S/2C20H23FN4O5/c2*1-2-22-8-11-5-6-23(9-11)17-15(21)7-13-16(18(17)25(29)30)24(12-3-4-12)10-14(19(13)26)20(27)28/h2*7,10-12,22H,2-6,8-9H2,1H3,(H,27,28)/t2*11-/m10/s1	CCNC[C@H]1CCN(C1)C2=C(C=C3C(N(C=C(C3=O)C(O)=O)C4CC4)=C2[N+]([O-])=O)F.CCNC[C@@H]5CCN(C5)C6=C(C=C7C(N(C=C(C7=O)C(O)=O)C8CC8)=C6[N+]([O-])=O)F	DNA gyrase and topoisomerase IV inhibitor	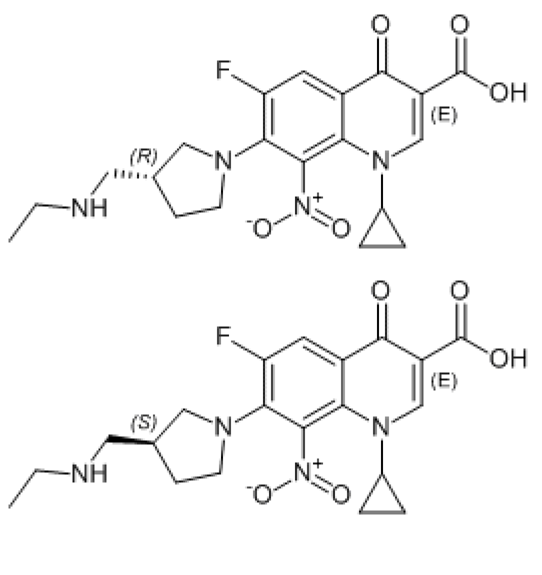	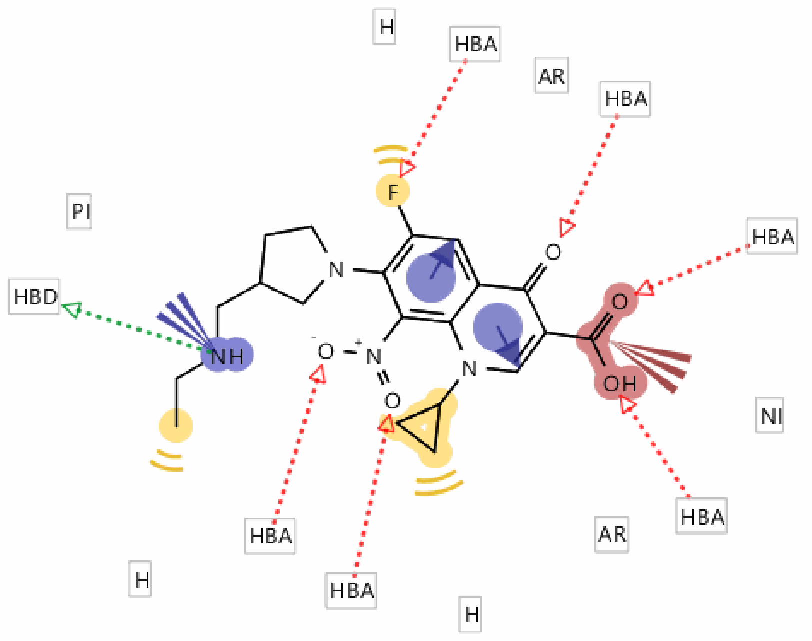

Common name	CAS Reg. No.	InChIKey	InChI	SMILES	Primary target	2D Structure	Pharmacophore[1]
PD 131628	127967-03-7	MUKSDTOOLRNSIO-QMMMGPOBSA-N	InChI=1S/C16H17FN4O3/c17-12-5-10-13(22)11(16(23)24)7-21(9-1-2-9)14(10)19-15(12)20-4-3-8(18)6-20/h5,7-9H,1-4,6,18H2,(H,23,24)/t8-/m0/s1	FC1=C(N2CC[C@H](N)C2)N=C3C(C(C(C(O)=O)=CN3C4CC4)=O)=C1	DNA gyrase and topoisomerase IV inhibitor	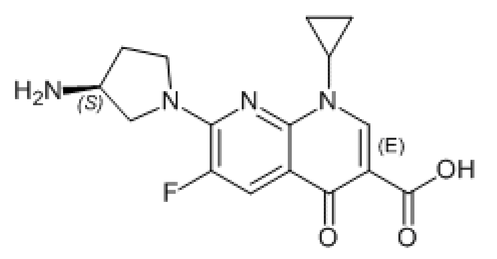	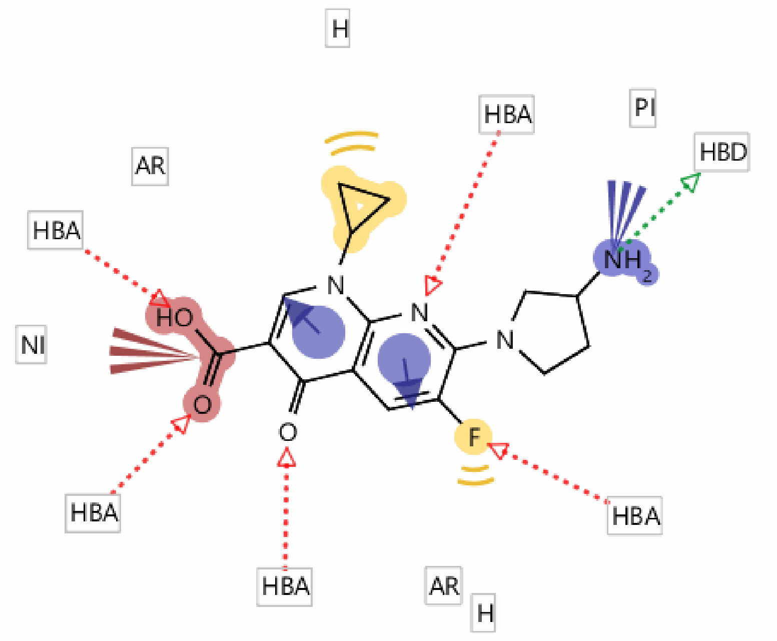
PD 135042 (AM 1147)	112811-57-1	XJCSNIFKGXSDGN-UHFFFAOYSA-N	InChI=1S/C18H20FN3O4/c1-26-17-14-11(8-13(19)15(17)21-6-4-20-5-7-21)16(23)12(18(24)25)9-22(14)10-2-3-10/h8-10,20H,2-7H2,1H3,(H,24,25)	COC1=C2C(C(C(C(O)=O)=CN2C3CC3)=O)=CC(F)=C1N4CCNCC4	DNA gyrase and topoisomerase IV inhibitor	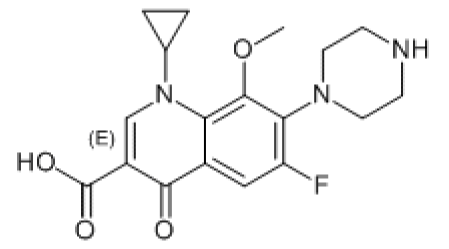	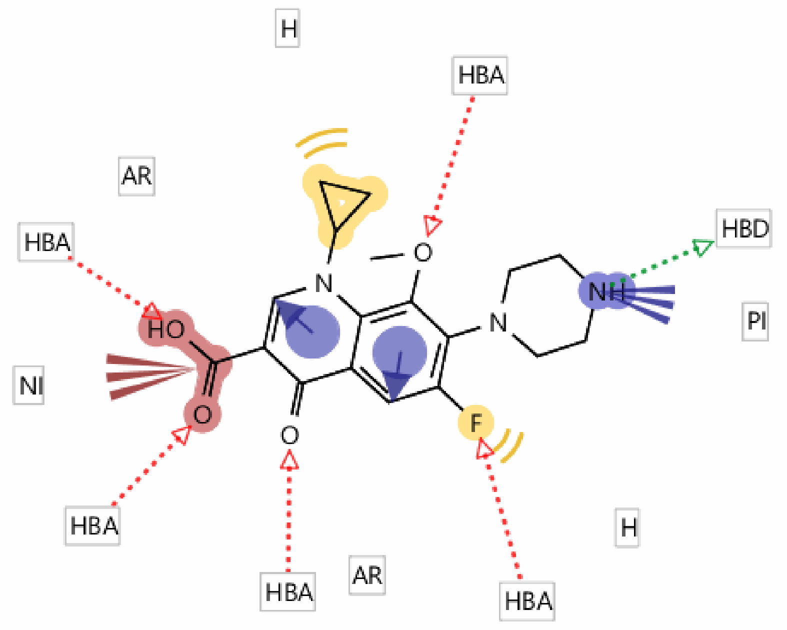
PD 135144 (BMY 33315)	116162-91-5	DEYKINRXCLHCDG-UHFFFAOYSA-N	InChI=1S/C17H21FN4O3/c1-17(2,3)22-9-11(16(24)25)13(23)10-8-12(18)15(20-14(10)22)21-6-4-19-5-7-21/h8-9,19H,4-7H2,1-3H3,(H,24,25)	CC(N1C=C(C(C2=CC(F)=C(N=C21)N3CCNCC3)=O)C(O)=O)(C)C	DNA gyrase and topoisomerase IV inhibitor	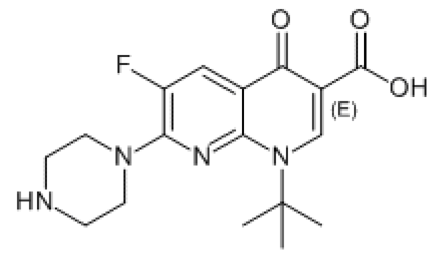	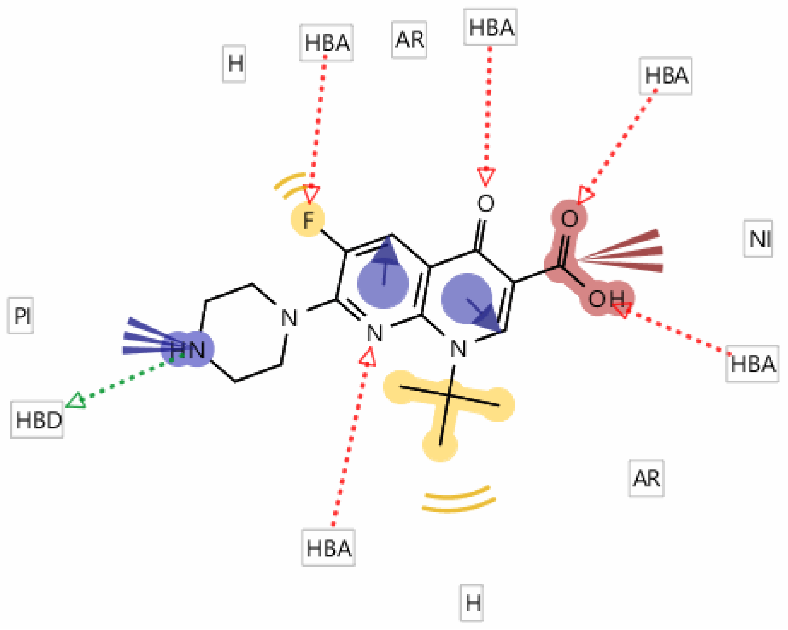
PD 137156		CHRPKMHVBYWRHT-FTYBWHBYSA-N	InChI=1S/2C19H22FN3O4/c2*1-10-7-22(5-4-21-10)15-6-14-16(13(9-24)17(15)20)18(25)12(19(26)27)8-23(14)11-2-3-11/h2*6,8,10-11,21,24H,2-5,7,9H2,1H3,(H,26,27)/t2*10-/m10/s1	C[C@H]1CN(C2=C(F)C(CO)=C3C(N(C4CC4)C=C(C(O)=O)C3=O)=C2)CCN1.C[C@@H]5CN(C6=C(F)C(CO)=C7C(N(C8CC8)C=C(C(O)=O)C7=O)=C6)CCN5	DNA gyrase and topoisomerase IV inhibitor	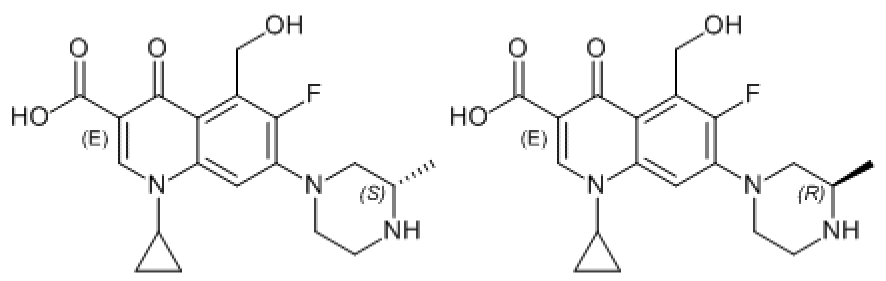	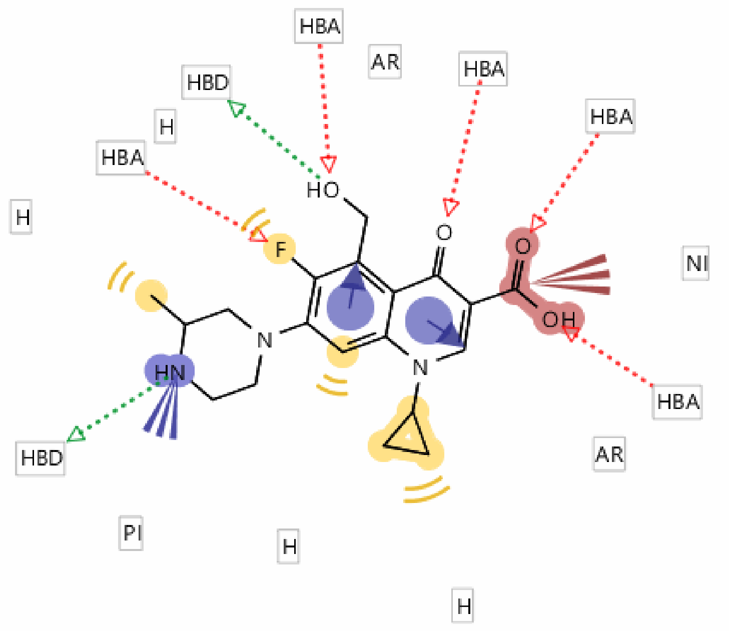
PD 138312	107334-06-5	WZGKUGHHTACDTE-SNVBAGLBSA-N	InChI=1S/C19H23FN4O3/c1-19(2,21)10-5-6-23(8-10)17-14(20)7-12-15(25)13(18(26)27)9-24(11-3-4-11)16(12)22-17/h7,9-11H,3-6,8,21H2,1-2H3,(H,26,27)/t10-/m1/s1	CC(C)([C@@H]1CCN(C2=C(F)C=C(C3=N2)C(C(C(O)=O)=CN3C4CC4)=O)C1)N	DNA gyrase and topoisomerase IV inhibitor	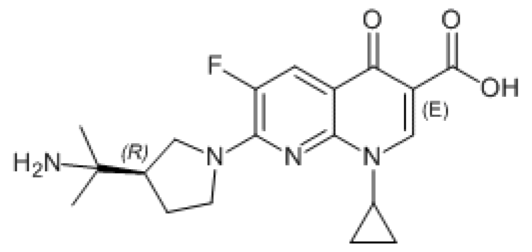	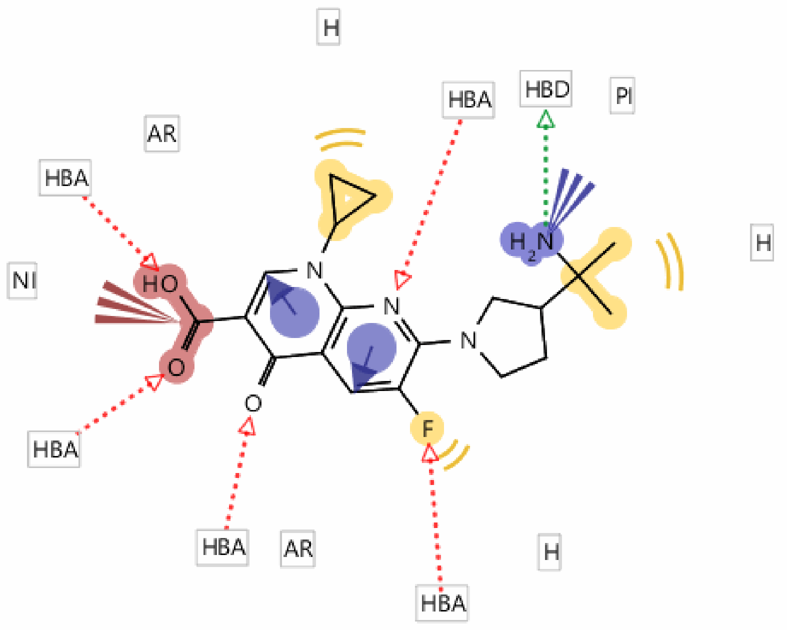

Common name	CAS Reg. No.	InChIKey	InChI	SMILES	Primary target	2D Structure	Pharmacophore[1]
PD 140248	143383-20-4	VMHBXZLMBJVYFA-WDEREUQCSA-N	InChI=1S/C21H19F3N4O3/c1-10(25)11-4-5-27(8-11)20-16(24)7-13-18(29)14(21(30)31)9-28(19(13)26-20)17-3-2-12(22)6-15(17)23/h2-3,6-7,9-11H,4-5,8,25H2,1H3,(H,30,31)/t10-,11+/m0/s1	C[C@@H]([C@@]1(CCN(C1)C2=C(C=C(C(C(C(O)=O)=CN3C4=C(C=C(C=C4)F)F)=O)C3=N2)F)[H])N	DNA gyrase and topoisomerase IV inhibitor	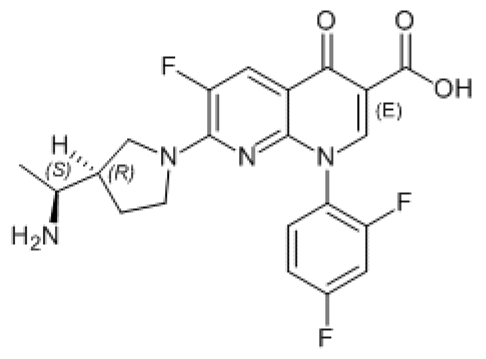	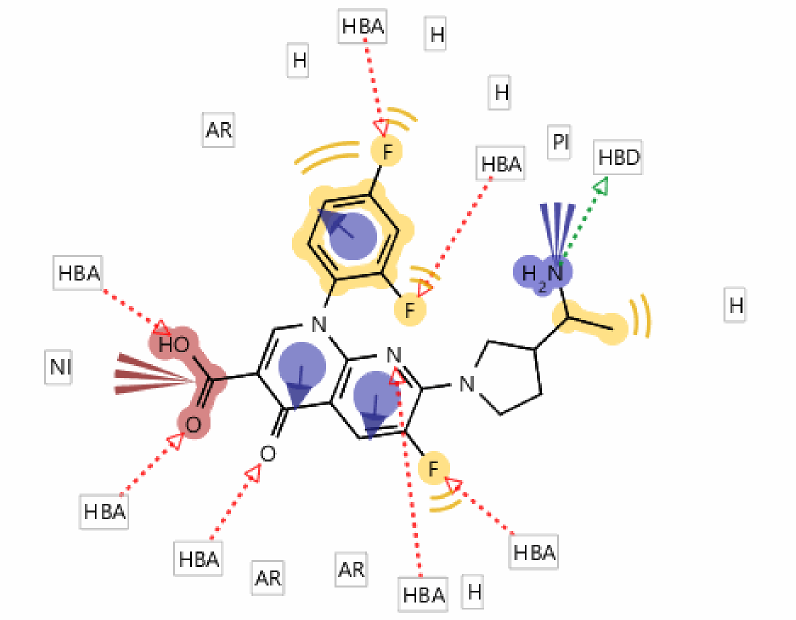
PD 163449	109347-94-6	KRKGVKYQENDSEV-UHFFFAOYSA-N	InChI=1S/C17H17BrFN3O3/c18-13-14-10(7-12(19)15(13)21-5-3-20-4-6-21)16(23)11(17(24)25)8-22(14)9-1-2-9/h7-9,20H,1-6H2,(H,24,25)	BrC1=C2C(C(C(C(O)=O)=CN2C3CC3)=O)=CC(F)=C1N4CCNCC4	DNA gyrase and topoisomerase IV inhibitor	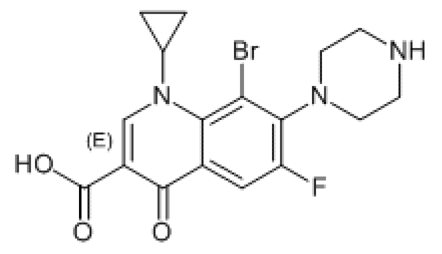	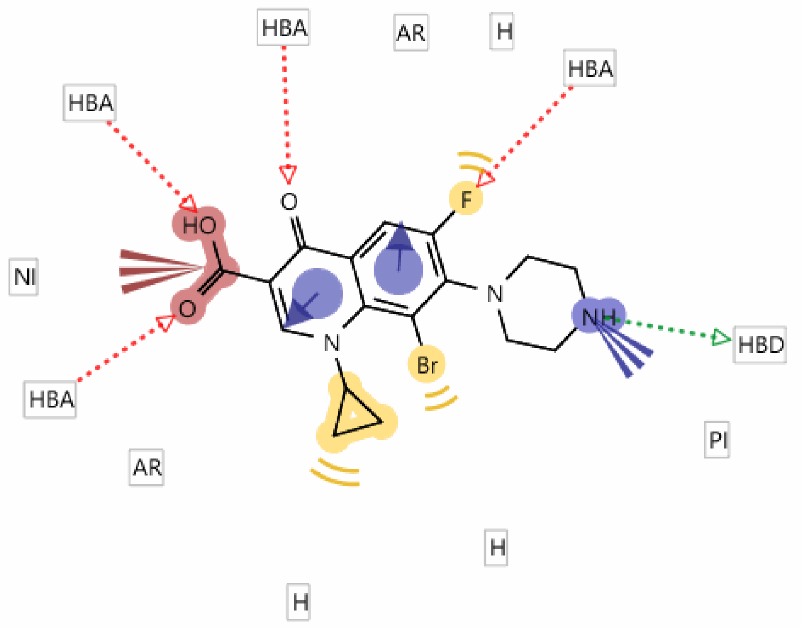
PD 164488	112811-83-3	YKKIBHKSHPPCEQ-UHFFFAOYSA-N	InChI=1S/C19H22FN3O4/c1-2-27-18-15-12(9-14(20)16(18)22-7-5-21-6-8-22)17(24)13(19(25)26)10-23(15)11-3-4-11/h9-11,21H,2-8H2,1H3,(H,25,26)	CCOC1=C2C(C(C(C(O)=O)=CN2C3CC3)=O)=CC(F)=C1N4CCNCC4	DNA gyrase and topoisomerase IV inhibitor	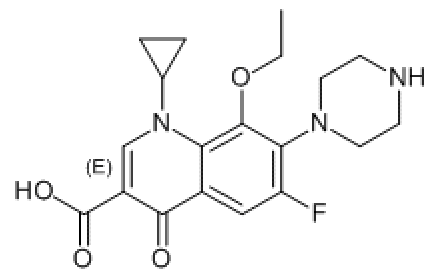	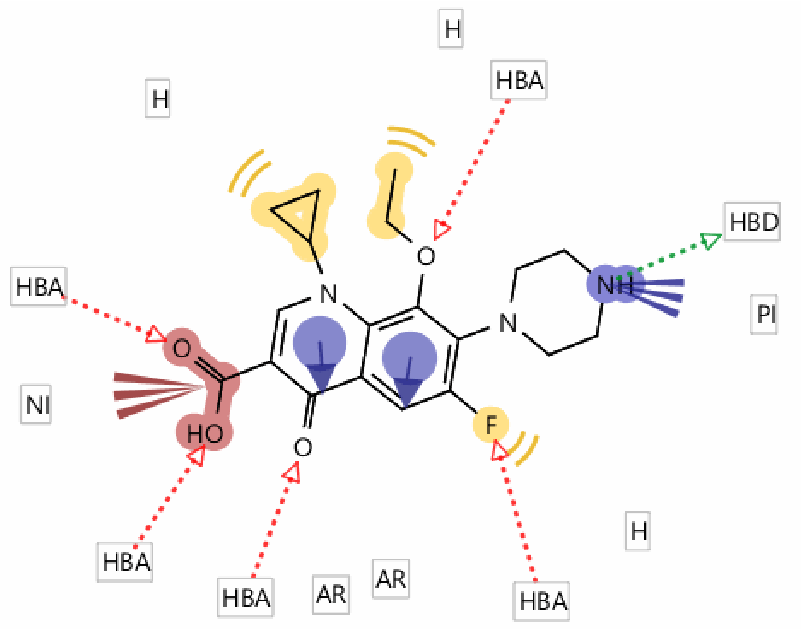
Pefloxacin	70458-92-3	FHFYDNQZQSQIAI-UHFFFAOYSA-N	InChI=1S/C17H20FN3O3/c1-3-20-10-12(17(23)24)16(22)11-8-13(18)15(9-14(11)20)21-6-4-19(2)5-7-21/h8-10H,3-7H2,1-2H3,(H,23,24)	CCN1C=C(C(O)=O)C(C2=CC(F)=C(N3CCN(CC3)C)C=C21)=O	DNA gyrase and topoisomerase IV inhibitor	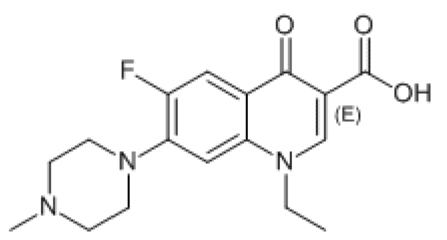	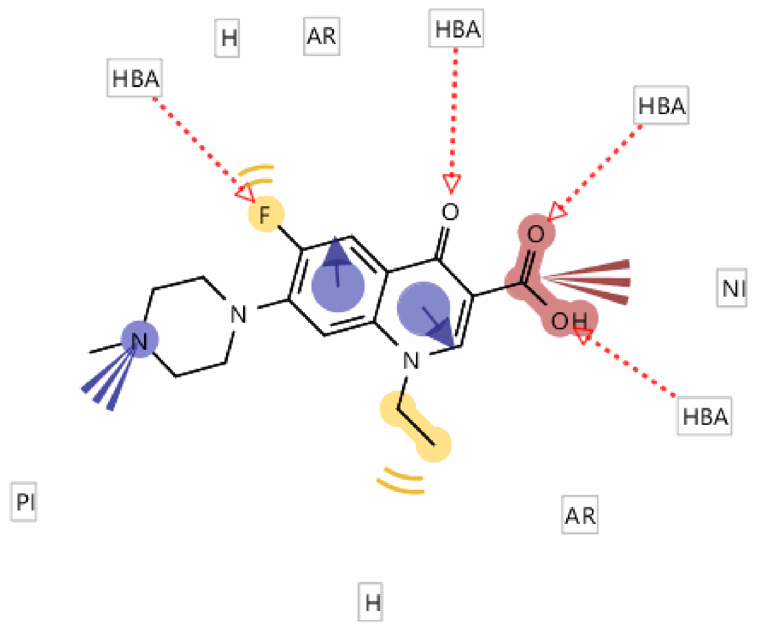
Pipemidic acid	51940-44-4	JOHZPMXAZQZXHR-UHFFFAOYSA-N	InChI=1S/C14H17N5O3/c1-2-18-8-10(13(21)22)11(20)9-7-16-14(17-12(9)18)19-5-3-15-4-6-19/h7-8,15H,2-6H2,1H3,(H,21,22)	CCN1C=C(C(O)=O)C(C2=CN=C(N3CCNCC3)N=C21)=O	DNA gyrase and topoisomerase IV inhibitor	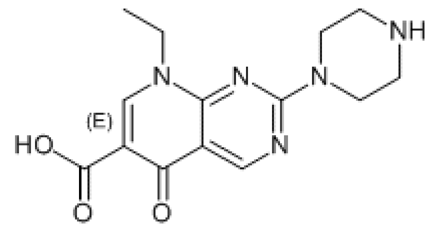	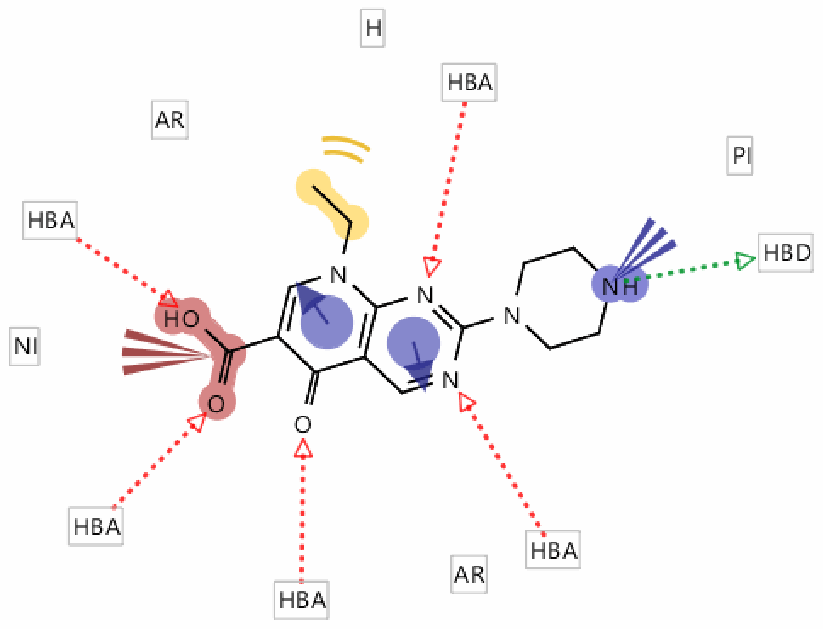

Common name	CAS Reg. No.	InChIKey	InChI	SMILES	Primary target	2D Structure	Pharmacophore[1]
Piromidic acid	19562-30-2	RCIMBBZXSXFZBV-UHFFFAOYSA-N	InChI=1S/C14H16N4O3/c1-2-17-8-10(13(20)21)11(19)9-7-15-14(16-12(9)17)18-5-3-4-6-18/h7-8H,2-6H2,1H3,(H,20,21)	CCN1C=C(C(C2=CN=C(N=C21)N3CCCC3)=O)C(O)=O	DNA gyrase and topoisomerase IV inhibitor	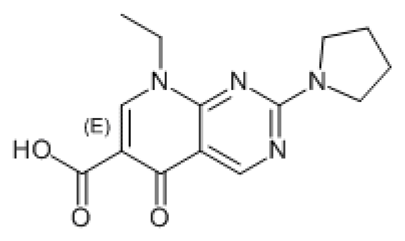	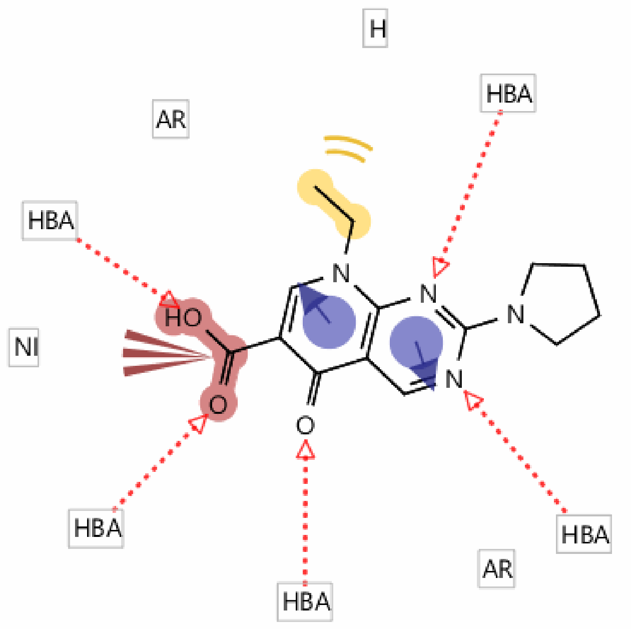
Piroxacin	93906-65-1	FELNOORDZDEBLV-UHFFFAOYSA-N	InChI=1S/C16H14N2O3/c1-2-17-10-13(16(20)21)15(19)12-6-5-11(9-14(12)17)18-7-3-4-8-18/h3-10H,2H2,1H3,(H,20,21)	CCN1C=C(C(O)=O)C(C2=C1C=C(N3C=CC=C3)C=C2)=O	DNA gyrase and topoisomerase IV inhibitor	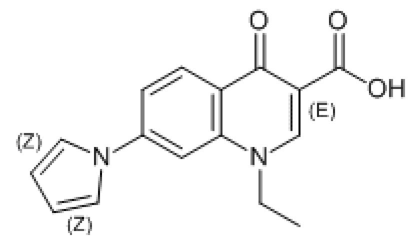	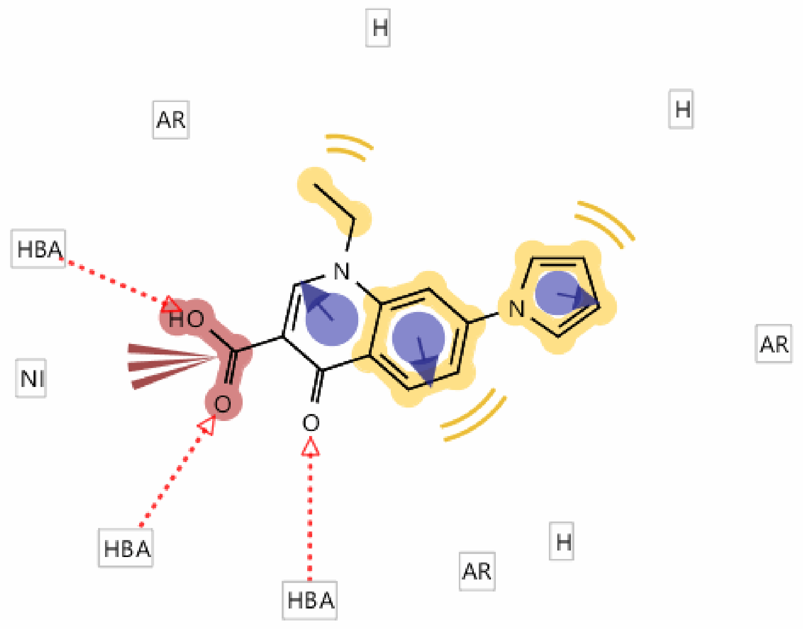
Pradofloxacin	195532-12-8	LZLXHGFNOWILIY-APPDUMDISA-N	InChI=1S/C21H21FN4O3/c22-16-6-13-18(26(12-3-4-12)9-15(20(13)27)21(28)29)14(7-23)19(16)25-8-11-2-1-5-24-17(11)10-25/h6,9,11-12,17,24H,1-5,8,10H2,(H,28,29)/t11-,17+/m0/s1	FC1=C(N2C[C@]3([H])CCCN[C@]3([H])C2)C(C#N)=C4C(C(C(C(O)=O)=CN4C5CC5)=O)=C1	DNA gyrase and topoisomerase IV inhibitor	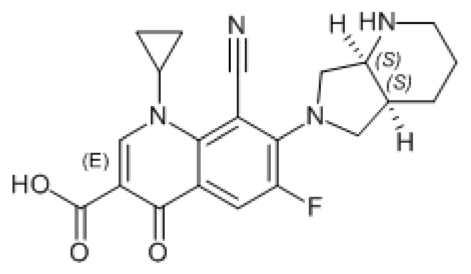	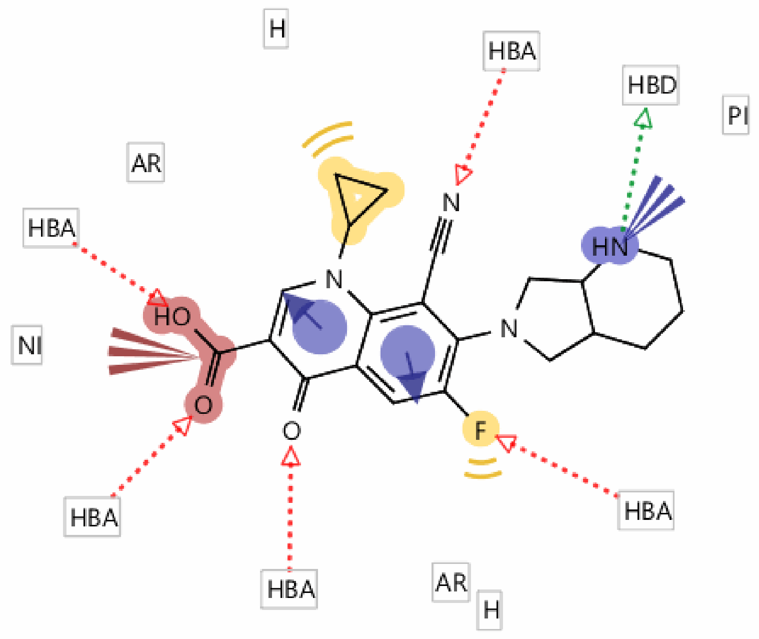
Premafloxacin	143383-65-7	SUQUWONDIBHQOZ-NWDGAFQWSA-N	InChI=1S/C21H26FN3O4/c1-11(23-2)12-6-7-24(9-12)18-16(22)8-14-17(20(18)29-3)25(13-4-5-13)10-15(19(14)26)21(27)28/h8,10-13,23H,4-7,9H2,1-3H3,(H,27,28)/t11-,12+/m0/s1	C[C@H](NC)[C@]1([H])CCN(C2=C(F)C=C(C(C(C(O)=O)=CN3C4CC4)=O)C3=C2OC)C1	DNA gyrase and topoisomerase IV inhibitor	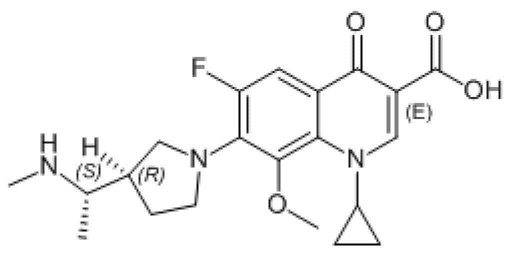	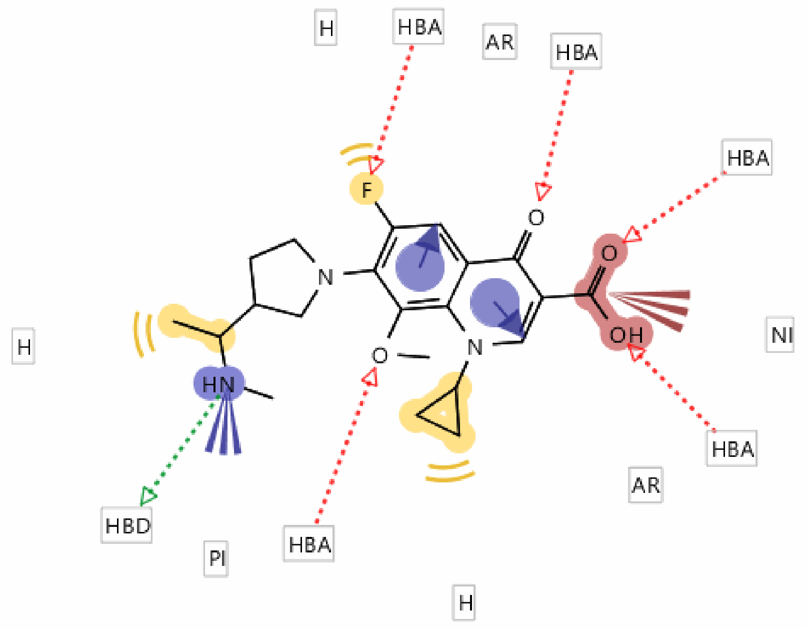
Prulifloxacin	123447-62-1	RIEHBOVIKTXYQN-FGYXOPSTSA-N	InChI=1S/2C21H20FN3O6S/c2*1-10-16(31-21(29)30-10)9-23-3-5-24(6-4-23)15-8-14-12(7-13(15)22)18(26)17(20(27)28)19-25(14)11(2)32-19/h2*7-8,11H,3-6,9H2,1-2H3,(H,27,28)/t2*11-/m10/s1	CC(O1)=C(CN2CCN(CC2)C3=C(C=C(C4=C3)C(C(C(O)=O)=C5N4[C@@H](C)S5)=O)F)OC1=O.CC(O6)=C(CN7CCN(CC7)C8=C(C=C(C9=C8)C(C(C(O)=O)=C%10N9[C@H](C)S%10)=O)F)OC6=O	DNA gyrase and topoisomerase IV inhibitor	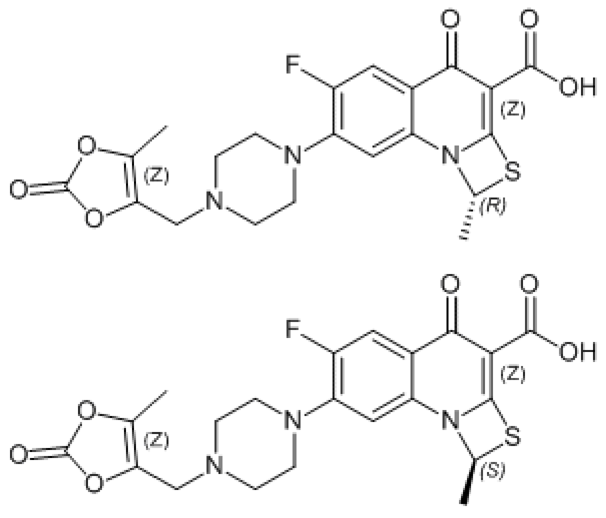	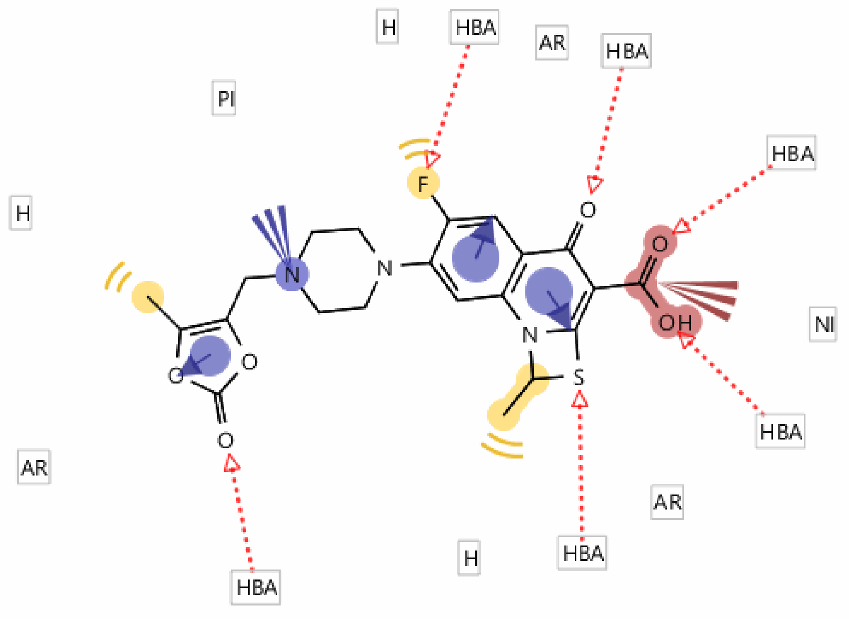

Common name	CAS Reg. No.	InChIKey	InChI	SMILES	Primary target	2D Structure	Pharmacophore[1]
PubChem CID-11531032		JRMYGOQEFDLQJN-UHFFFAOYSA-N	InChI=1S/C20H16FN3O2S/c21-15-7-14-16(8-13(15)11-3-1-10(9-22)2-4-11)24(12-5-6-12)20-17(18(14)25)19(26)23-27-20/h1-4,7-8,12H,5-6,9,22H2,(H,23,26)	O=C(C1=C2SNC1=O)C3=CC(F)=C(C4=CC=C(CN)C=C4)C=C3N2C5CC5	DNA gyrase and topoisomerase IV inhibitor	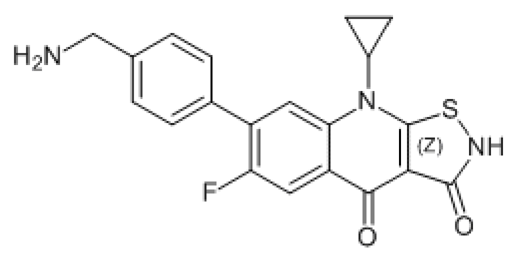	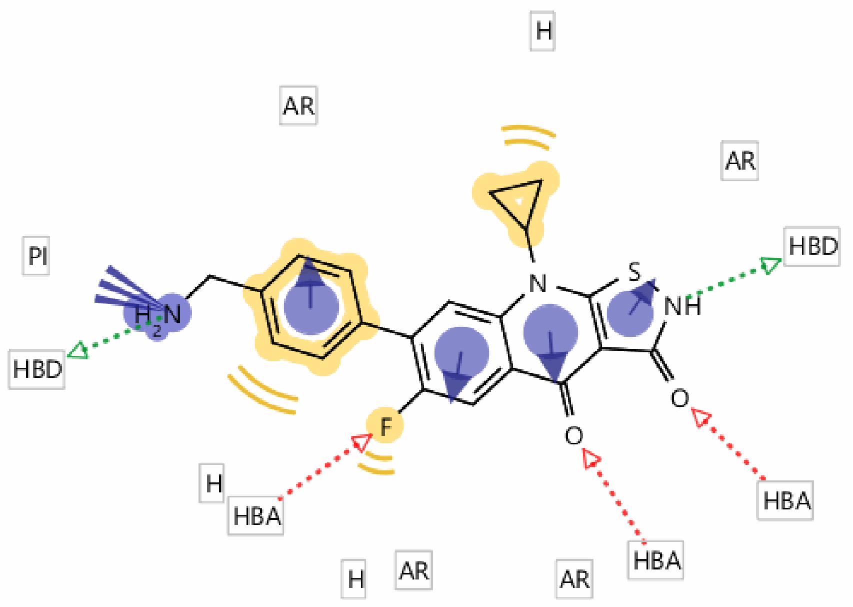
PubChem CID-11566845		RFAPGAPICNQXOM-UHFFFAOYSA-N	InChI=1S/C19H13FN2O3S/c20-14-7-13-15(8-12(14)9-2-1-3-11(23)6-9)22(10-4-5-10)19-16(17(13)24)18(25)21-26-19/h1-3,6-8,10,23H,4-5H2,(H,21,25)	O=C(C1=C2SNC1=O)C3=CC(F)=C(C4=CC(O)=CC=C4)C=C3N2C5CC5	DNA gyrase and topoisomerase IV inhibitor	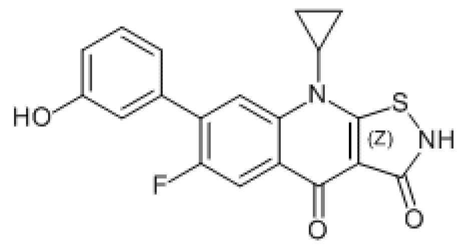	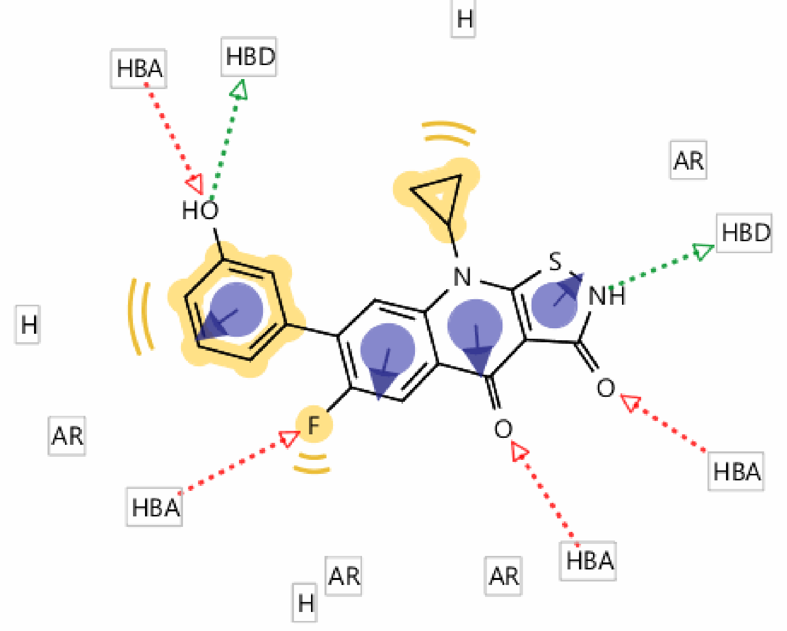
PubChem CID-11610627		FXVJVTDGWQCYOQ-UHFFFAOYSA-N	InChI=1S/C20H16FN3O2S/c21-15-7-14-16(8-13(15)11-3-1-2-10(6-11)9-22)24(12-4-5-12)20-17(18(14)25)19(26)23-27-20/h1-3,6-8,12H,4-5,9,22H2,(H,23,26)	O=C(C1=C2SNC1=O)C3=CC(F)=C(C4=CC(CN)=CC=C4)C=C3N2C5CC5	DNA gyrase and topoisomerase IV inhibitor	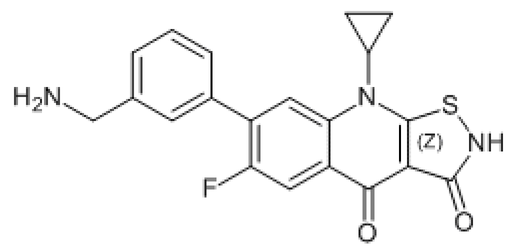	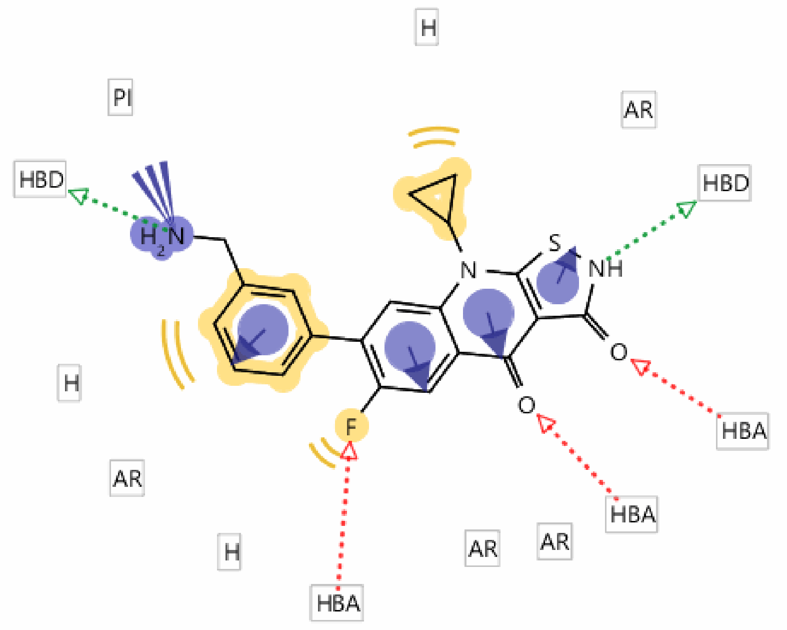
PubChem CID-11696318		KKUHZTLGKWLPKE-UHFFFAOYSA-N	InChI=1S/C19H14FN3O2S/c20-14-7-13-15(8-12(14)9-2-1-3-10(21)6-9)23(11-4-5-11)19-16(17(13)24)18(25)22-26-19/h1-3,6-8,11H,4-5,21H2,(H,22,25)	O=C(C1=C2SNC1=O)C3=CC(F)=C(C4=CC(N)=CC=C4)C=C3N2C5CC5	DNA gyrase and topoisomerase IV inhibitor	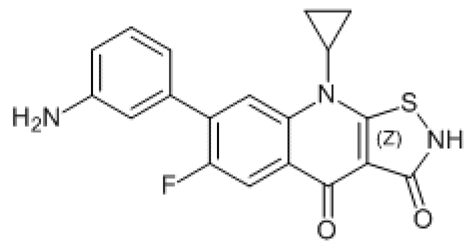	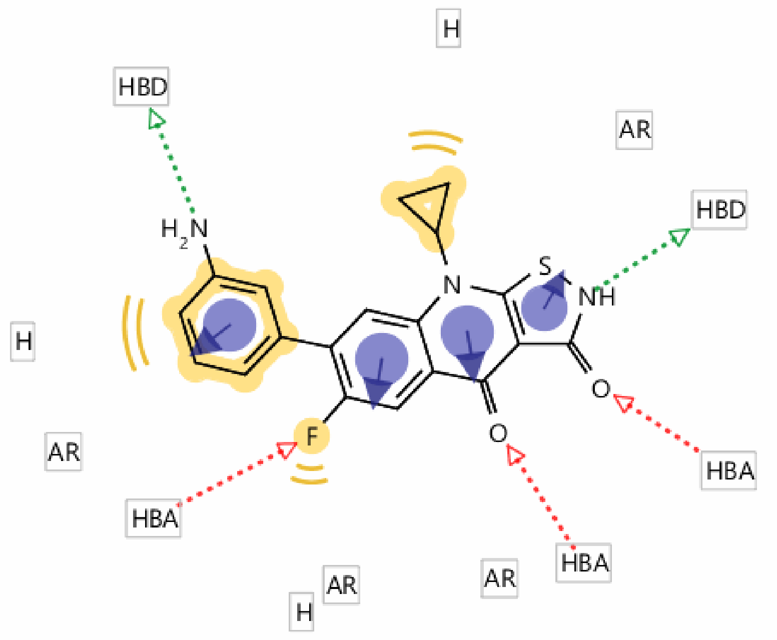
PubChem CID-11844920	906527-41-1	XWDPBLFYPNKPFN-UHFFFAOYSA-N	InChI=1S/C20H16FN3O3S/c1-9-7-10(5-6-22-9)14-13(21)8-12-16(18(14)27-2)24(11-3-4-11)20-15(17(12)25)19(26)23-28-20/h5-8,11H,3-4H2,1-2H3,(H,23,26)	CC1=NC=CC(C2=C(C=C3C(N(C4=C(C3=O)C(NS4)=O)C5CC5)=C2OC)F)=C1	DNA gyrase and topoisomerase IV inhibitor	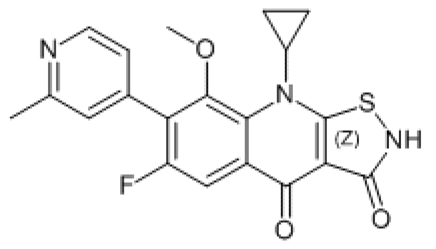	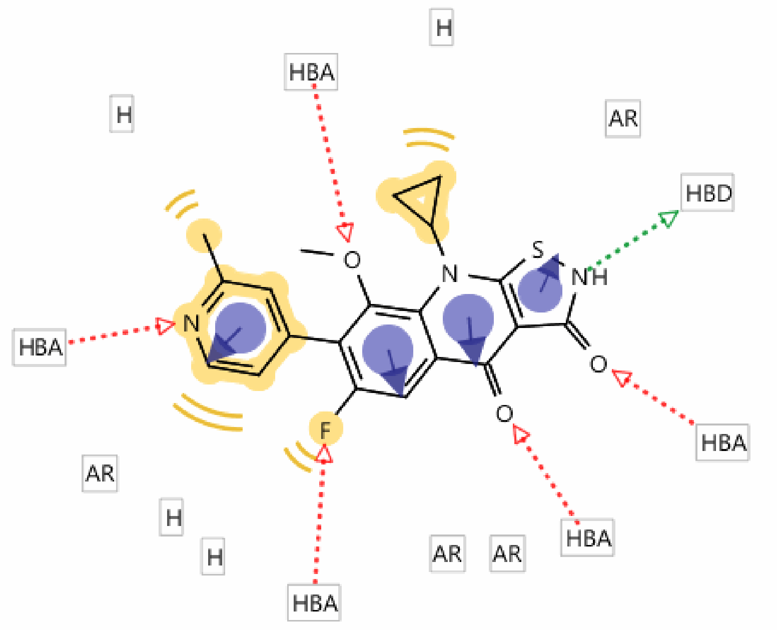
PubChem CID-11996799		YYAPVFVWLPPHKD-SNVBAGLBSA-N	InChI=1S/C23H20FN3O3S/c1-10-14-6-3-11(7-12(14)9-25-10)17-16(24)8-15-19(21(17)30-2)27(13-4-5-13)23-18(20(15)28)22(29)26-31-23/h3,6-8,10,13,25H,4-5,9H2,1-2H3,(H,26,29)/t10-/m1/s1	C[C@@H]1C2=C(CN1)C=C(C=C2)C3=C(C=C4C(N(C5=C(C4=O)C(NS5)=O)C6CC6)=C3OC)F	DNA gyrase and topoisomerase IV inhibitor	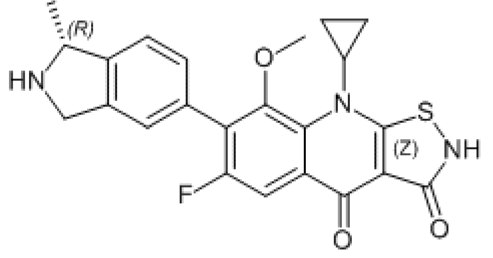	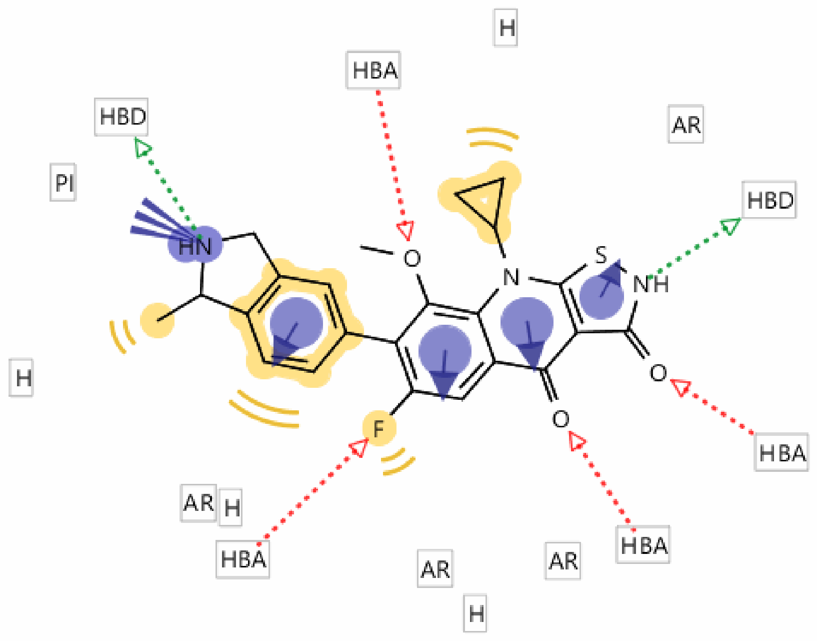

Common name	CAS Reg. No.	InChIKey	InChI	SMILES	Primary target	2D Structure	Pharmacophore[1]
PubChem CID-11996800		INFOMPWYYGJYFO-UHFFFAOYSA-N	InChI=1S/C22H18FN3O3S/c1-29-20-16(10-2-3-11-8-24-9-12(11)6-10)15(23)7-14-18(20)26(13-4-5-13)22-17(19(14)27)21(28)25-30-22/h2-3,6-7,13,24H,4-5,8-9H2,1H3,(H,25,28)	COC1=C2C(C(C3=C(N2C4CC4)SNC3=O)=O)=CC(F)=C1C5=CC6=C(CNC6)C=C5	DNA gyrase and topoisomerase IV inhibitor	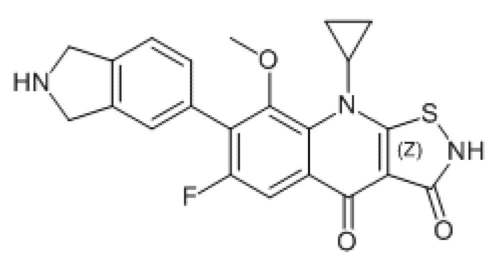	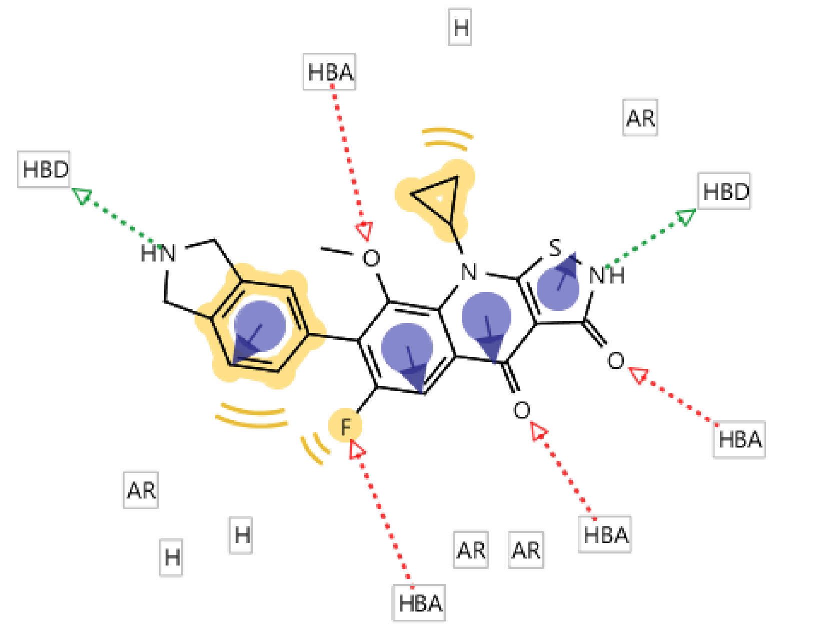
PubChem CID-11997263		BGJYJHMNNGVNLK-UHFFFAOYSA-N	InChI=1S/C21H18FN3O3S/c1-9-6-11(7-10(2)23-9)15-14(22)8-13-17(19(15)28-3)25(12-4-5-12)21-16(18(13)26)20(27)24-29-21/h6-8,12H,4-5H2,1-3H3,(H,24,27)	CC1=CC(C2=C(C=C3C(N(C4=C(C3=O)C(NS4)=O)C5CC5)=C2OC)F)=CC(C)=N1	DNA gyrase and topoisomerase IV inhibitor	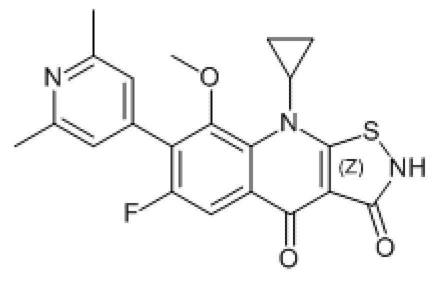	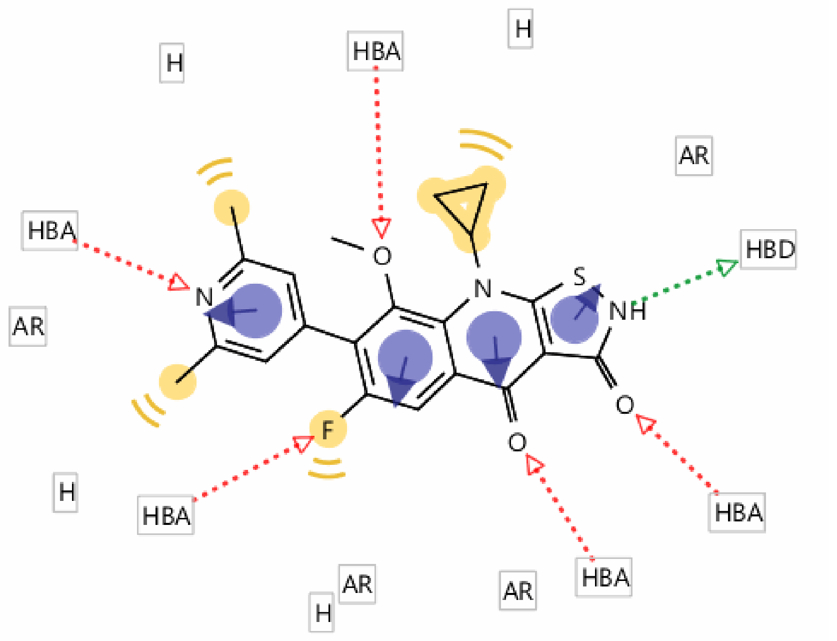
PubChem CID-25022869		OEFVEGDPRVNFMP-VHSXEESVSA-N	InChI=1S/C20H23FN4O3S/c1-9(22)10-5-6-24(8-10)16-13(21)7-12-15(18(16)28-2)25(11-3-4-11)20-14(17(12)26)19(27)23-29-20/h7,9-11H,3-6,8,22H2,1-2H3,(H,23,27)/t9-,10+/m0/s1	C[C@@H]([C@@]1(CCN(C1)C2=C(C=C(C3=C2OC)C(C4=C(N3C5CC5)SNC4=O)=O)F)[H])N	DNA gyrase and topoisomerase IV inhibitor	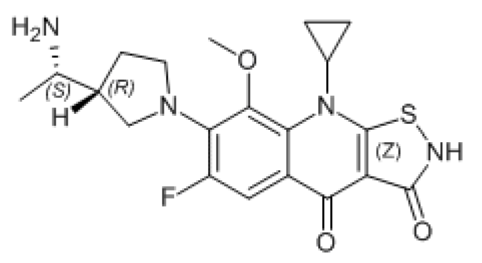	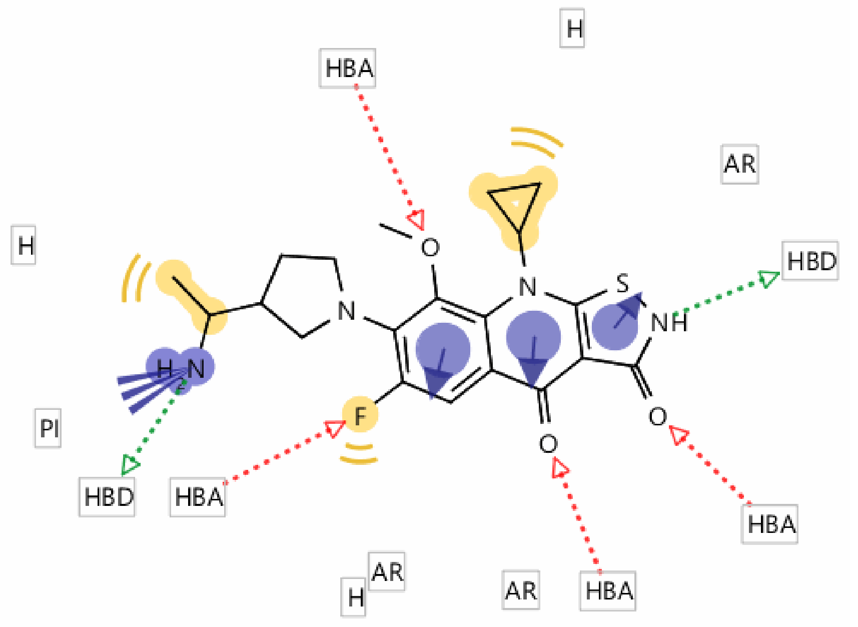
PubChem CID-44408626		DBHRNNUAKNLSTN-UHFFFAOYSA-N	InChI=1S/C19H13FN2O3S/c20-14-7-13-15(8-12(14)9-1-5-11(23)6-2-9)22(10-3-4-10)19-16(17(13)24)18(25)21-26-19/h1-2,5-8,10,23H,3-4H2,(H,21,25)	O=C(C1=C2SNC1=O)C3=CC(F)=C(C4=CC=C(O)C=C4)C=C3N2C5CC5	DNA gyrase and topoisomerase IV inhibitor	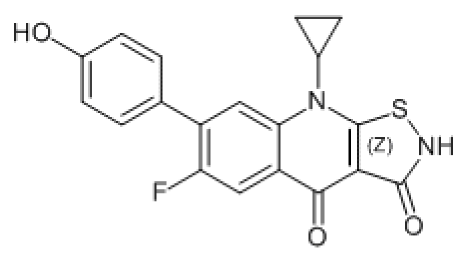	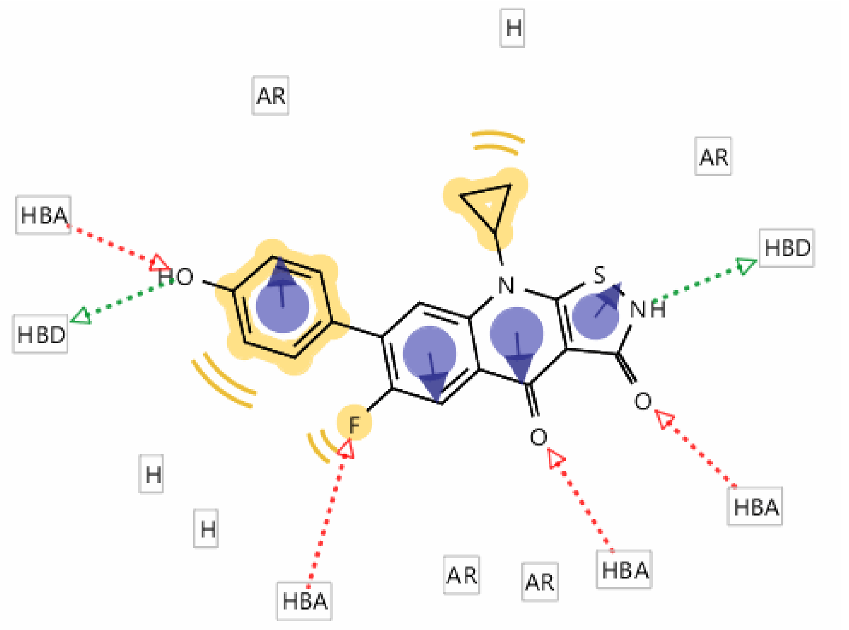
PubChem CID-44408894		XAJJUFKXNKKIQI-UHFFFAOYSA-N	InChI=1S/C20H15FN2O3S/c21-15-7-14-16(8-13(15)11-3-1-2-10(6-11)9-24)23(12-4-5-12)20-17(18(14)25)19(26)22-27-20/h1-3,6-8,12,24H,4-5,9H2,(H,22,26)	O=C(C1=C2SNC1=O)C3=CC(F)=C(C4=CC(CO)=CC=C4)C=C3N2C5CC5	DNA gyrase and topoisomerase IV inhibitor	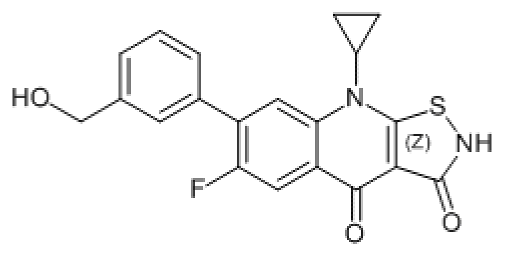	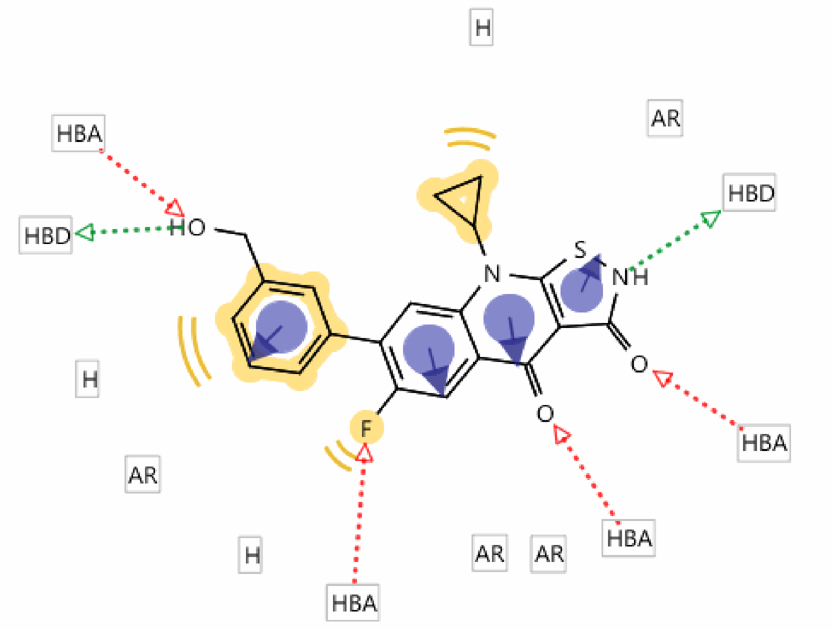
PubChem CID-44408896		YCVBALOHZCSOAU-UHFFFAOYSA-N	InChI=1S/C20H15FN2O3S/c21-15-7-14-16(8-13(15)11-3-1-10(9-24)2-4-11)23(12-5-6-12)20-17(18(14)25)19(26)22-27-20/h1-4,7-8,12,24H,5-6,9H2,(H,22,26)	O=C(C1=C2SNC1=O)C3=CC(F)=C(C4=CC=C(CO)C=C4)C=C3N2C5CC5	DNA gyrase and topoisomerase IV inhibitor	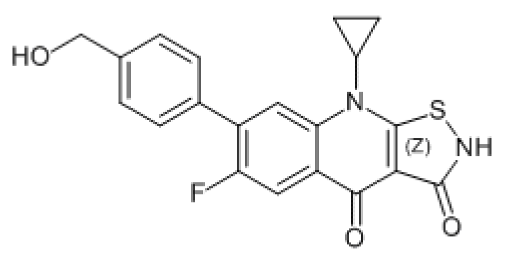	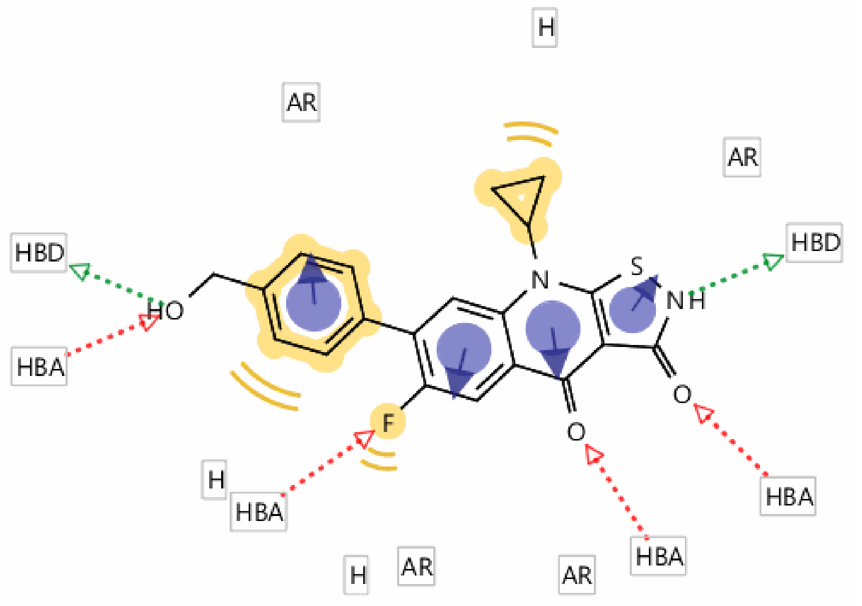

Common name	CAS Reg. No.	InChIKey	InChI	SMILES	Primary target	2D Structure	Pharmacophore[1]
PubChem CID-44408994		RYGQUNHPYQJBTQ-UHFFFAOYSA-N	InChI=1S/C19H12F2N2O2S/c20-10-3-1-2-9(6-10)12-8-15-13(7-14(12)21)17(24)16-18(25)22-26-19(16)23(15)11-4-5-11/h1-3,6-8,11H,4-5H2,(H,22,25)	O=C(C1=C2SNC1=O)C3=CC(F)=C(C4=CC(F)=CC=C4)C=C3N2C5CC5	DNA gyrase and topoisomerase IV inhibitor	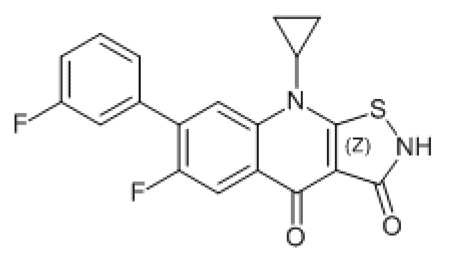	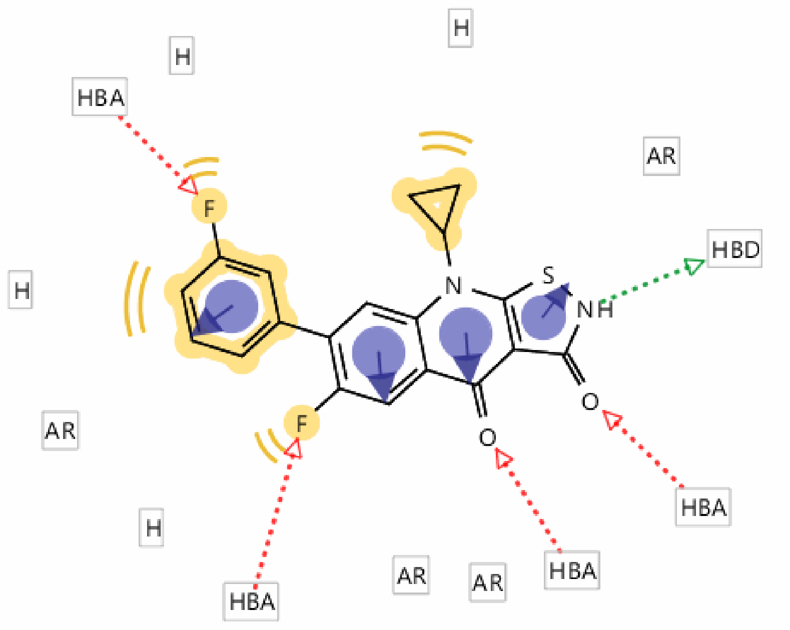
PubChem CID-44409001		HFRGSVIKEOBJHY-UHFFFAOYSA-N	InChI=1S/C19H13F2N3O2S/c20-12-4-1-8(5-14(12)22)10-7-15-11(6-13(10)21)17(25)16-18(26)23-27-19(16)24(15)9-2-3-9/h1,4-7,9H,2-3,22H2,(H,23,26)	O=C(C1=C2SNC1=O)C3=CC(F)=C(C4=CC(N)=C(F)C=C4)C=C3N2C5CC5	DNA gyrase and topoisomerase IV inhibitor	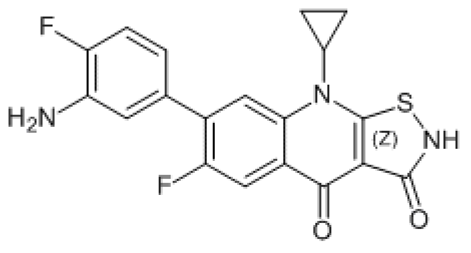	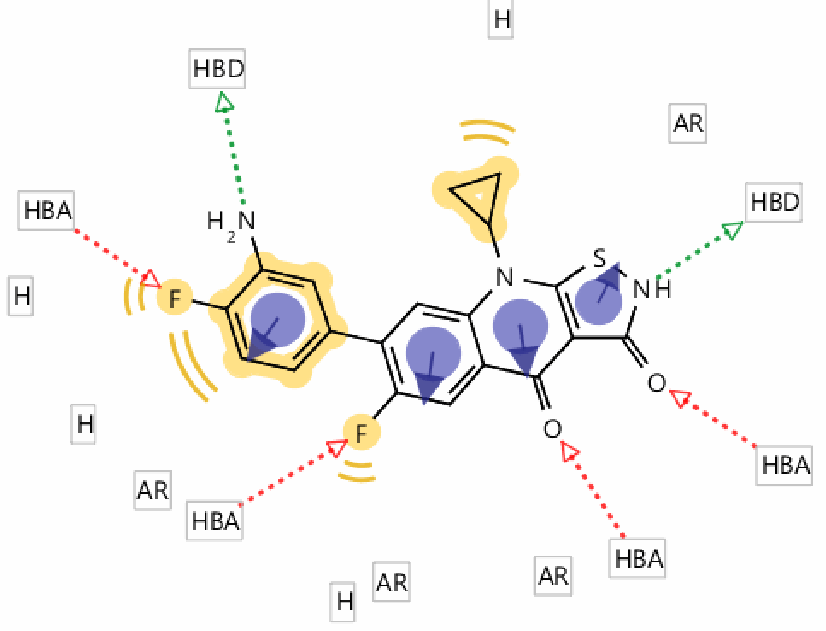
PubChem CID-44409010		JYDZVUWVVASPRQ-UHFFFAOYSA-N	InChI=1S/C19H14FN3O2S/c20-14-7-13-15(8-12(14)9-1-3-10(21)4-2-9)23(11-5-6-11)19-16(17(13)24)18(25)22-26-19/h1-4,7-8,11H,5-6,21H2,(H,22,25)	O=C(C1=C2SNC1=O)C3=CC(F)=C(C4=CC=C(N)C=C4)C=C3N2C5CC5	DNA gyrase and topoisomerase IV inhibitor	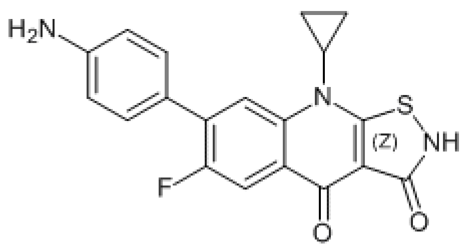	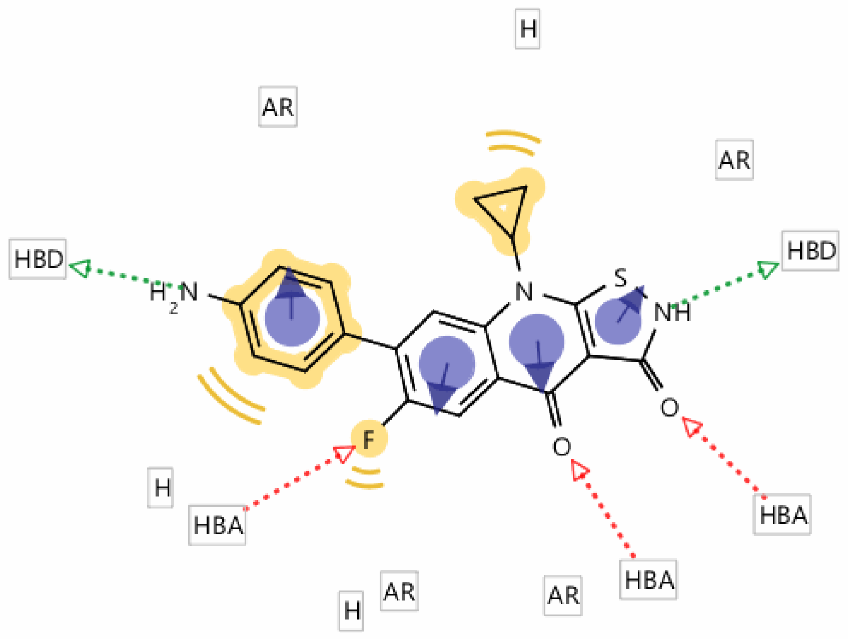
PubChem CID-53236573		SOJRULJEVFPUAA-WDEREUQCSA-N	InChI=1S/C21H25FN4O3S/c1-10(23-2)11-6-7-25(9-11)17-14(22)8-13-16(19(17)29-3)26(12-4-5-12)21-15(18(13)27)20(28)24-30-21/h8,10-12,23H,4-7,9H2,1-3H3,(H,24,28)/t10-,11+/m0/s1	C[C@@H]([C@@]1(CCN(C1)C2=C(C=C(C3=C2OC)C(C4=C(N3C5CC5)SNC4=O)=O)F)[H])NC	DNA gyrase and topoisomerase IV inhibitor	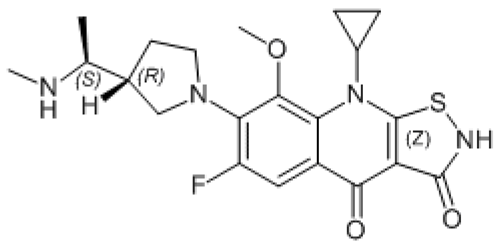	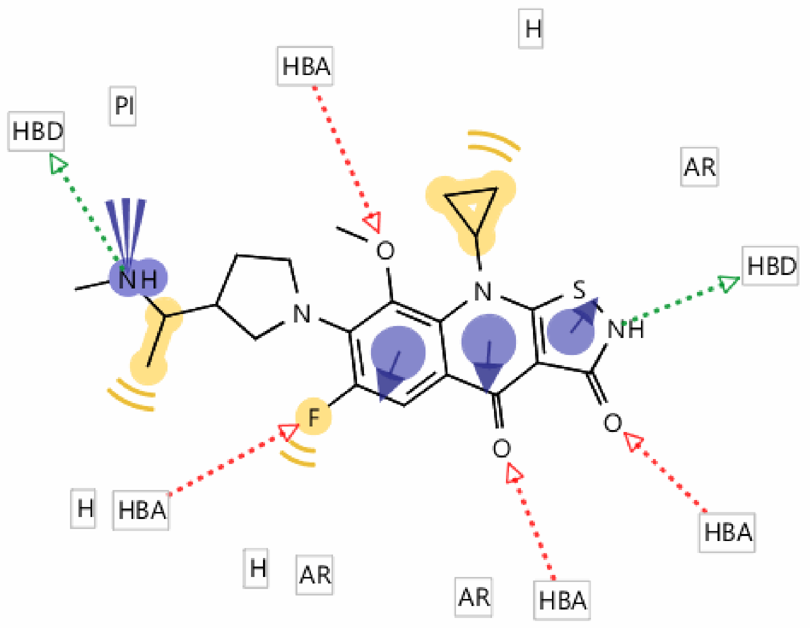
PubChem CID-53236796		HGRMJFQMMABBOE-NWDGAFQWSA-N	InChI=1S/C22H26F2N4O3S/c1-11(25-7-6-23)12-5-8-27(10-12)18-15(24)9-14-17(20(18)31-2)28(13-3-4-13)22-16(19(14)29)21(30)26-32-22/h9,11-13,25H,3-8,10H2,1-2H3,(H,26,30)/t11-,12+/m0/s1	C[C@@H]([C@@]1(CCN(C1)C2=C(C=C(C3=C2OC)C(C4=C(N3C5CC5)SNC4=O)=O)F)[H])NCCF	DNA gyrase and topoisomerase IV inhibitor	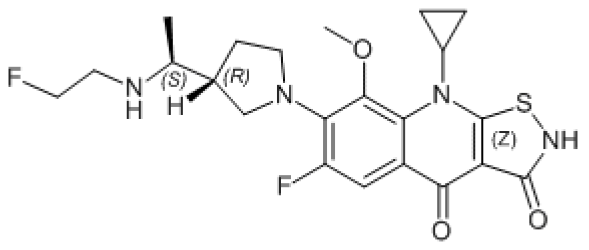	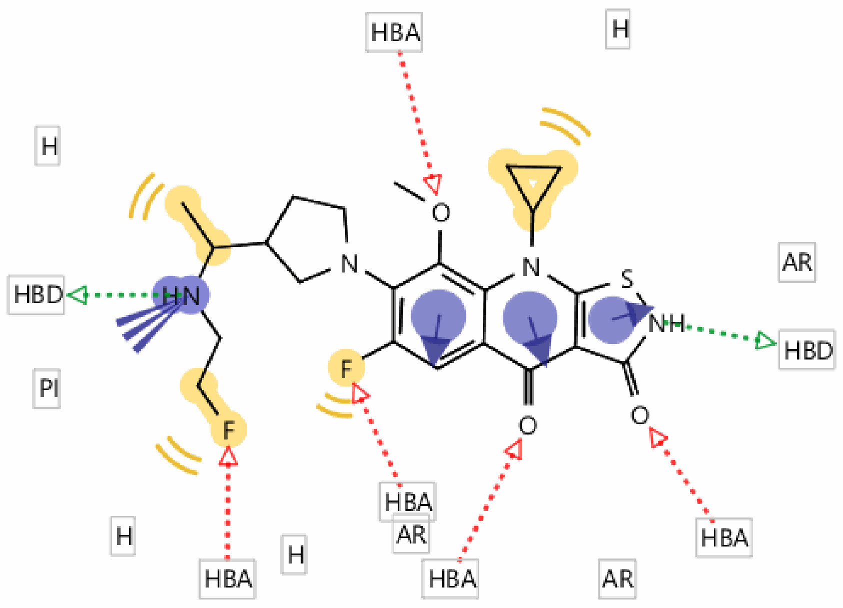
PubChem CID-122195336		XIQHTMHVFRRPKO-UHFFFAOYSA-N	InChI=1S/C21H20F3N5O3/c1-3-26-10-6-28(7-10)17-9(2)16-11(4-13(17)22)18(30)12(21(31)32)8-29(16)20-15(24)5-14(23)19(25)27-20/h4-5,8,10,26H,3,6-7H2,1-2H3,(H2,25,27)(H,31,32)	CC1=C2C(C(C(C(O)=O)=CN2C3=C(F)C=C(F)C(N)=N3)=O)=CC(F)=C1N4CC(C4)NCC	DNA gyrase and topoisomerase IV inhibitor	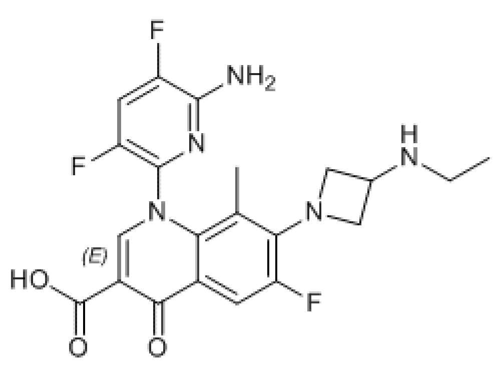	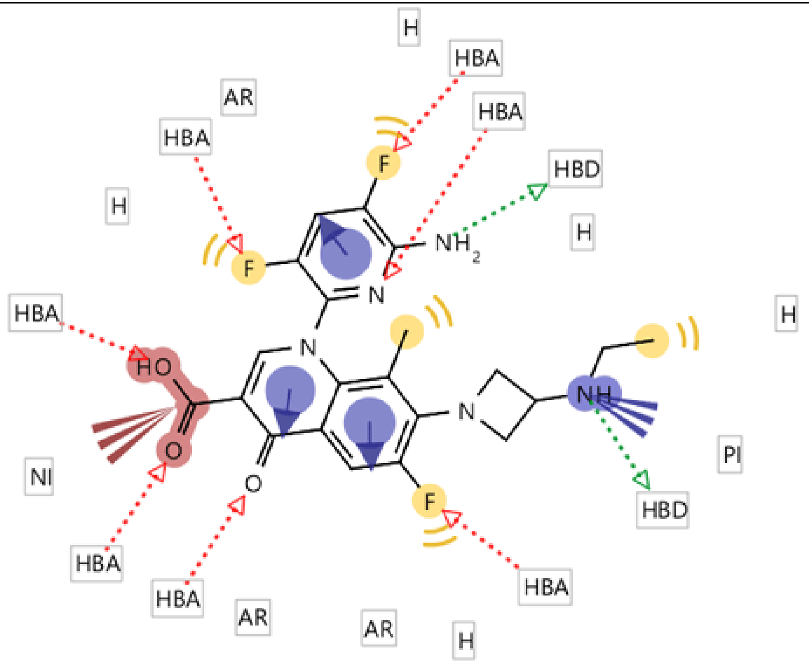

Common name	CAS Reg. No.	InChIKey	InChI	SMILES	Primary target	2D Structure	Pharmacophore[1]
PubChem CID-122195337		QYWWAPQGCKJESQ-UHFFFAOYSA-N	InChI=1S/C22H22F3N5O3/c1-3-4-27-11-7-29(8-11)18-10(2)17-12(5-14(18)23)19(31)13(22(32)33)9-30(17)21-16(25)6-15(24)20(26)28-21/h5-6,9,11,27H,3-4,7-8H2,1-2H3,(H2,26,28)(H,32,33)	CC1=C2C(C(C(C(O)=O)=CN2C3=C(F)C=C(F)C(N)=N3)=O)=CC(F)=C1N4CC(C4)NCCC	DNA gyrase and topoisomerase IV inhibitor	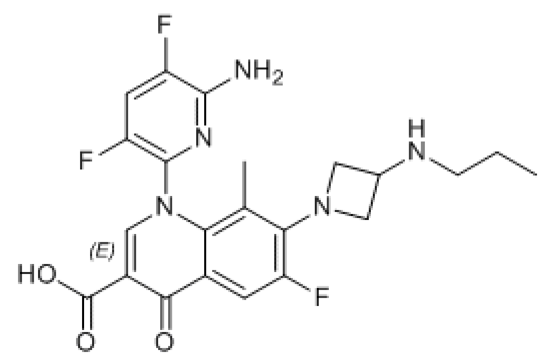	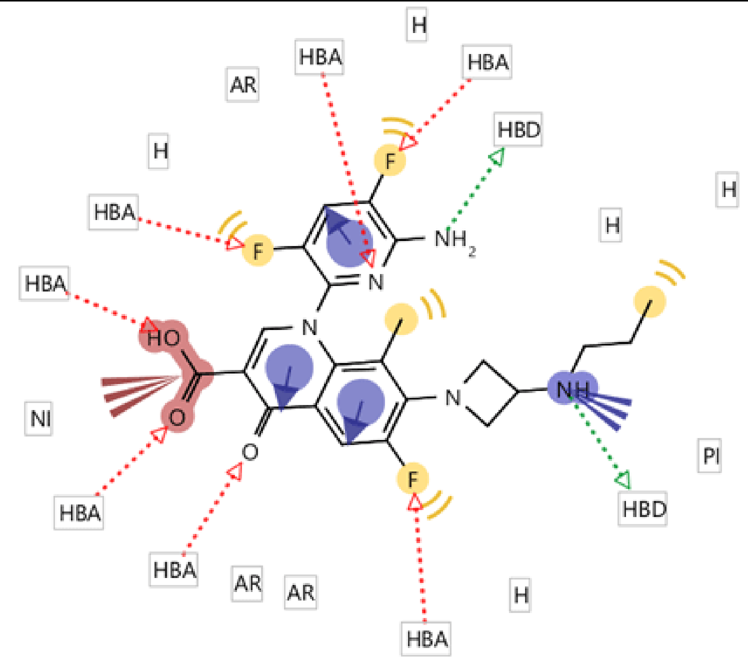
QA 241 free base		ZBUQTFNBHOYTSG-FTYBWHBYSA-N	InChI=1S/2C19H20FN3O4/c2*1-10-7-14(24)15-16-11(18(25)12(19(26)27)9-23(10)16)8-13(20)17(15)22-5-3-21(2)4-6-22/h2*8-10H,3-7H2,1-2H3,(H,26,27)/t2*10-/m10/s1	C[C@@H]1CC(C2=C3C(C(C(C(O)=O)=CN13)=O)=CC(F)=C2N4CCN(C)CC4)=O.C[C@H]5CC(C6=C7C(C(C(C(O)=O)=CN57)=O)=CC(F)=C6N8CCN(C)CC8)=O	DNA gyrase and topoisomerase IV inhibitor	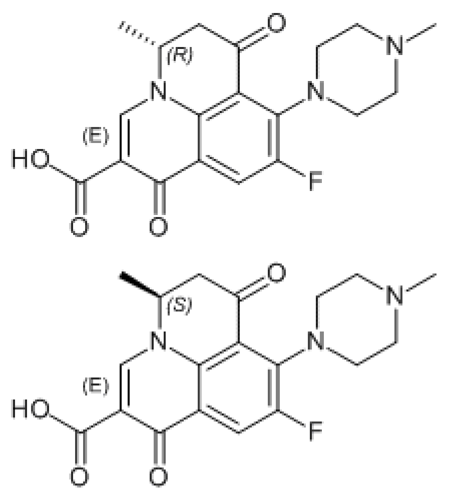	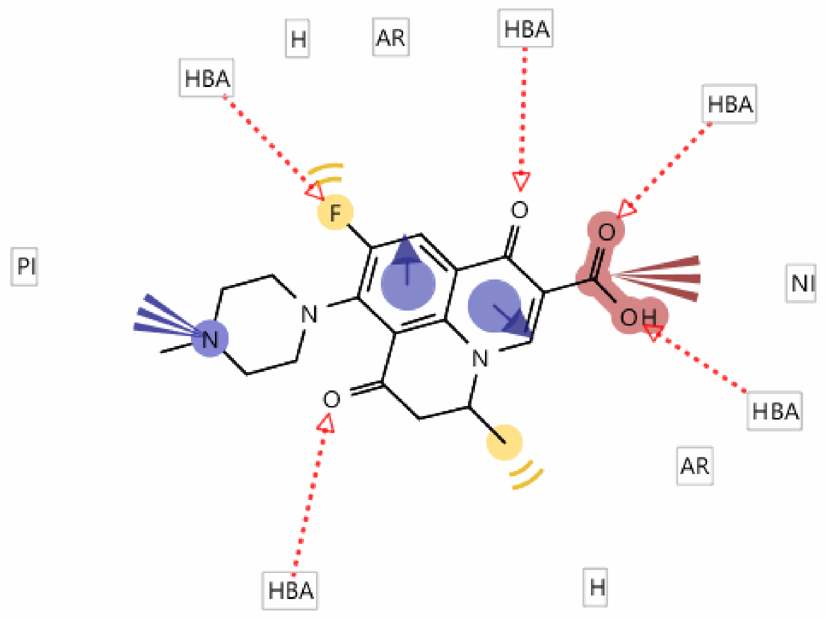
RO 13-5478	71575-96-7	AKBXSBHUDQHSJE-UHFFFAOYSA-N	InChI=1S/C16H18N2O3/c1-4-18-10-13(16(20)21)15(19)9-14(18)11-5-7-12(8-6-11)17(2)3/h5-10H,4H2,1-3H3,(H,20,21)	CCN1C=C(C(O)=O)C(C=C1C2=CC=C(N(C)C)C=C2)=O	DNA gyrase and topoisomerase IV inhibitor	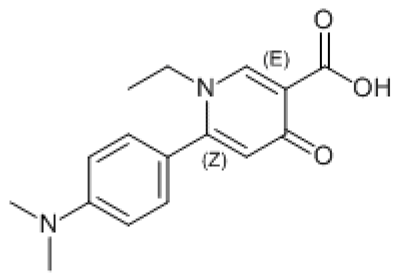	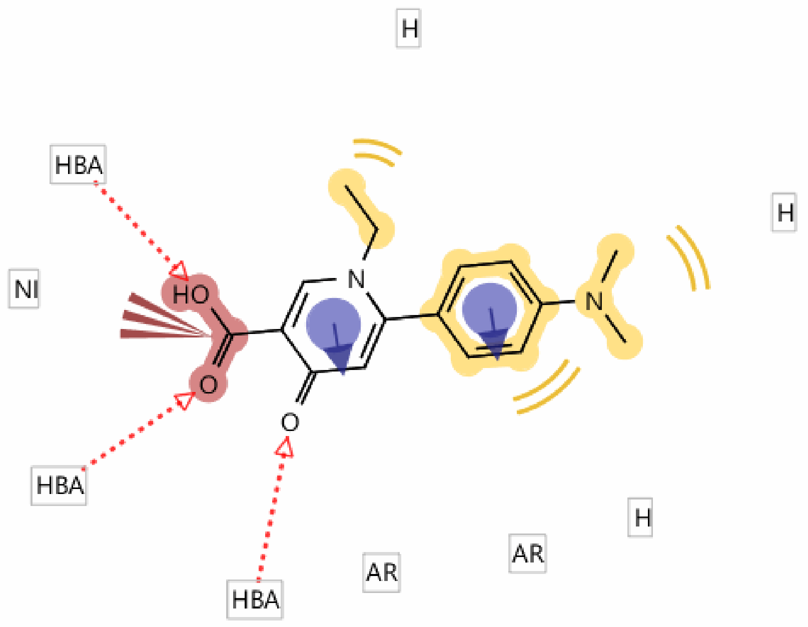
RO 14-9578	100891-41-6	QKRNJTOFEPJZFH-RMTNWKGQSA-N	InChI=1S/2C16H13NO5/c2*1-8-2-9-3-14-15(22-7-21-14)4-10(9)12-5-13(18)11(16(19)20)6-17(8)12/h2*3-6,8H,2,7H2,1H3,(H,19,20)/t2*8-/m10/s1	C[C@H]1CC2=CC3=C(C=C2C4=CC(C(C(O)=O)=CN14)=O)OCO3.C[C@@H]5CC6=CC7=C(C=C6C8=CC(C(C(O)=O)=CN58)=O)OCO7	DNA gyrase and topoisomerase IV inhibitor	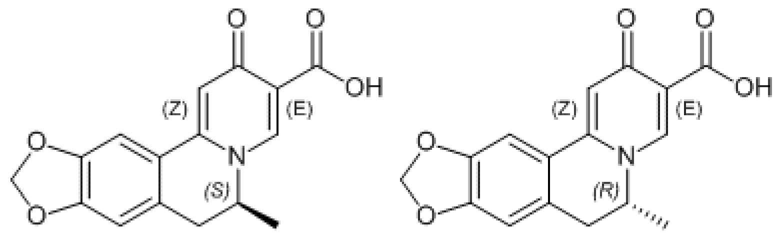	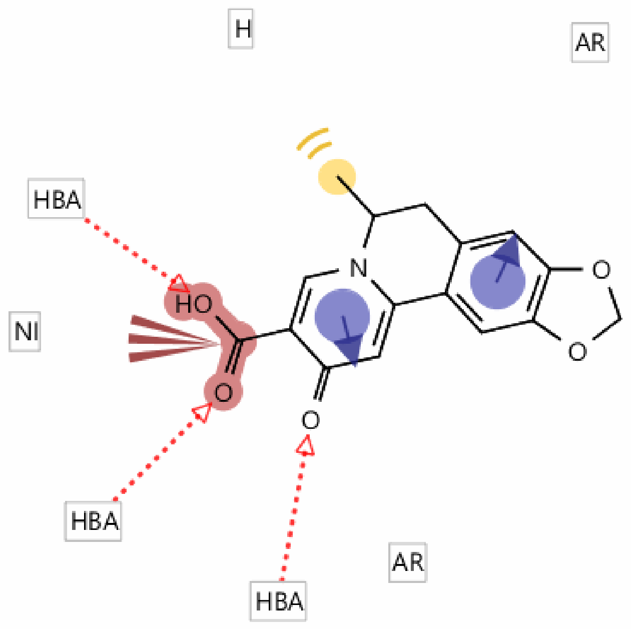
Rosoxacin	40034-42-2	XBPZXDSZHPDXQU-UHFFFAOYSA-N	InChI=1S/C17H14N2O3/c1-2-19-10-14(17(21)22)16(20)13-4-3-12(9-15(13)19)11-5-7-18-8-6-11/h3-10H,2H2,1H3,(H,21,22)	CCN1C=C(C(O)=O)C(C2=C1C=C(C3=CC=NC=C3)C=C2)=O	DNA gyrase and topoisomerase IV inhibitor	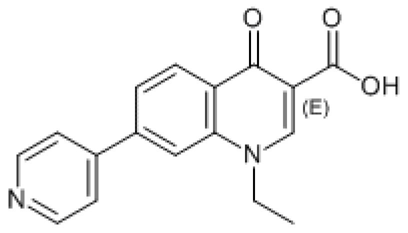	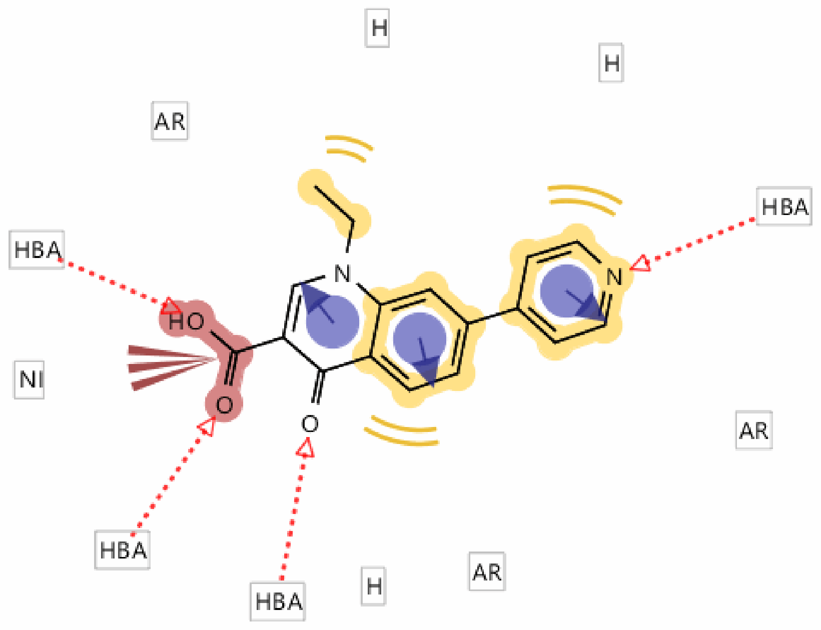
Rufloxacin	101363-10-4	NJCJBUHJQLFDSW-UHFFFAOYSA-N	InChI=1S/C17H18FN3O3S/c1-19-2-4-20(5-3-19)14-12(18)8-10-13-16(14)25-7-6-21(13)9-11(15(10)22)17(23)24/h8-9H,2-7H2,1H3,(H,23,24)	CN1CCN(C2=C(F)C=C3C4=C2SCCN4C=C(C(O)=O)C3=O)CC1	DNA gyrase and topoisomerase IV inhibitor	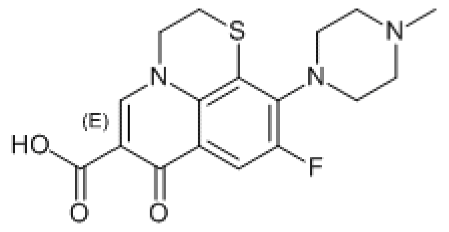	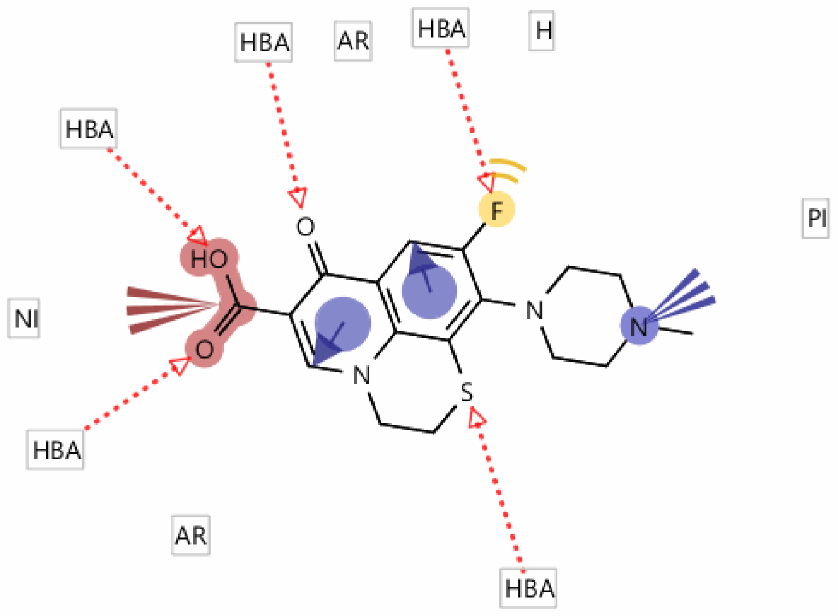

Common name	CAS Reg. No.	InChIKey	InChI	SMILES	Primary target	2D Structure	Pharmacophore[1]
S 25932	92358-02-6	CYAJHSDJRONVJG-IGXPJQGBSA-N	InChI=1S/2C17H14FN3O3/c2*1-9-2-3-10-14-11(16(22)12(17(23)24)7-21(9)14)6-13(18)15(10)20-5-4-19-8-20/h2*4-9H,2-3H2,1H3,(H,23,24)/t2*9-/m10/s1	C[C@@H]1CCC2=C3C(C(C(C(O)=O)=CN13)=O)=CC(F)=C2N4C=CN=C4.C[C@H]5CCC6=C7C(C(C(C(O)=O)=CN57)=O)=CC(F)=C6N8C=CN=C8	DNA gyrase and topoisomerase IV inhibitor	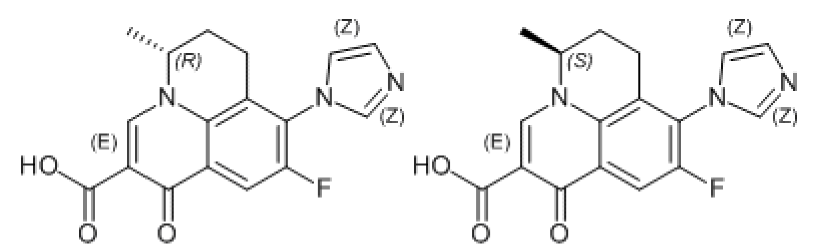	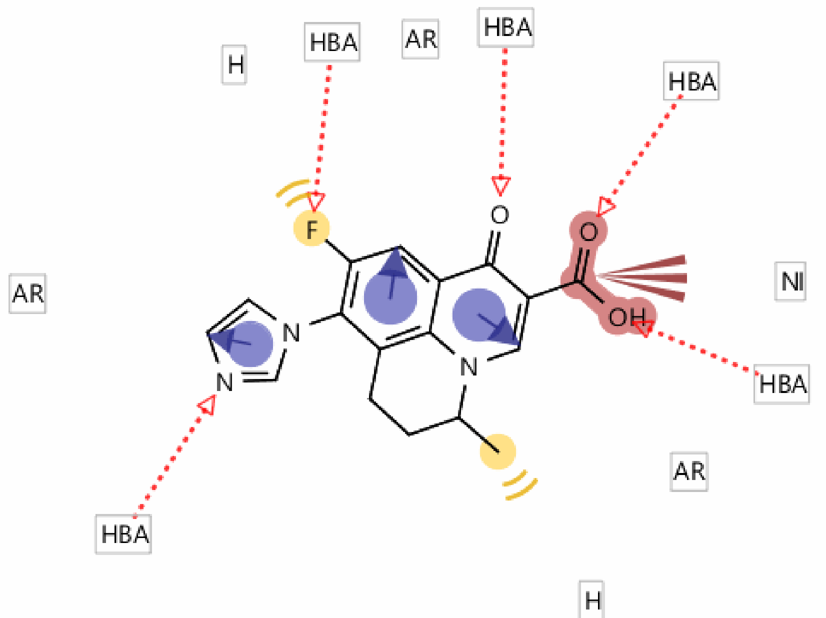
S 31076		PEQPFISMWLZTJZ-UHFFFAOYSA-N	InChI=1S/C19H20FN3O5/c1-28-18-15-12(17(24)13(19(25)26)9-23(15)11-2-3-11)8-14(20)16(18)22-6-4-10(21-27)5-7-22/h8-9,11,27H,2-7H2,1H3,(H,25,26)	COC1=C2C(C(C(C(O)=O)=CN2C3CC3)=O)=CC(F)=C1N4CC/C(CC4)=N/O	DNA gyrase and topoisomerase IV inhibitor	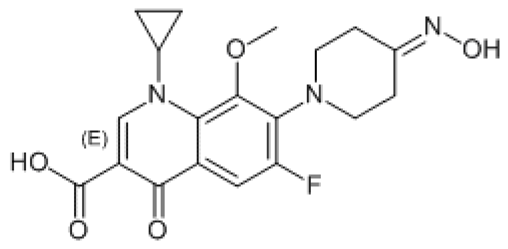	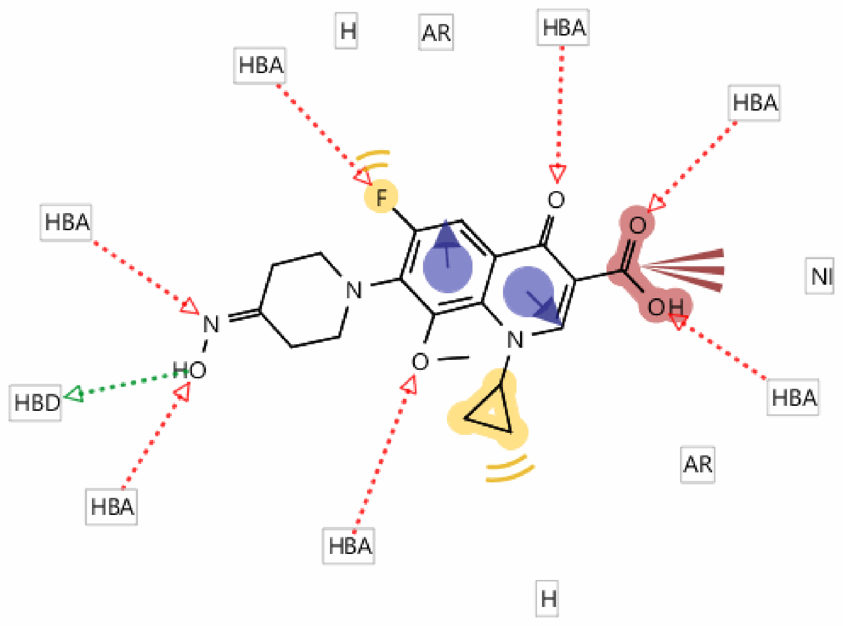
Sarafloxacin	98105-99-8	XBHBWNFJWIASRO-UHFFFAOYSA-N	InChI=1S/C20H17F2N3O3/c21-12-1-3-13(4-2-12)25-11-15(20(27)28)19(26)14-9-16(22)18(10-17(14)25)24-7-5-23-6-8-24/h1-4,9-11,23H,5-8H2,(H,27,28)	FC1=CC=C(C=C1)N2C=C(C(C3=CC(F)=C(C=C32)N4CCNCC4)=O)C(O)=O	DNA gyrase and topoisomerase IV inhibitor	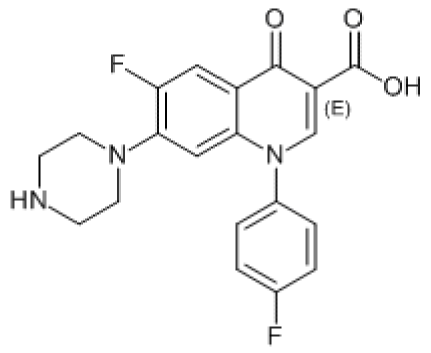	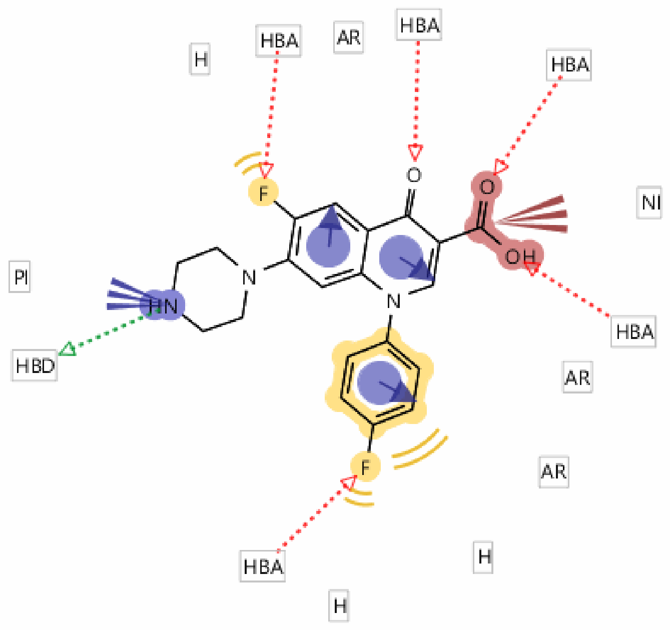
Sitafloxacin	127254-12-0	PNUZDKCDAWUEGK-CYZMBNFOSA-N	InChI=1S/C19H18ClF2N3O3/c20-14-15-8(17(26)9(18(27)28)5-25(15)12-4-10(12)21)3-11(22)16(14)24-6-13(23)19(7-24)1-2-19/h3,5,10,12-13H,1-2,4,6-7,23H2,(H,27,28)/t10-,12+,13+/m0/s1	ClC1=C2C(C(C(C(O)=O)=CN2[C@@H]3C[C@@H]3F)=O)=CC(F)=C1N4C[C@@H](N)C5(C4)CC5	DNA gyrase and topoisomerase IV inhibitor	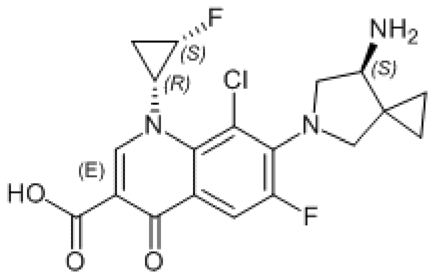	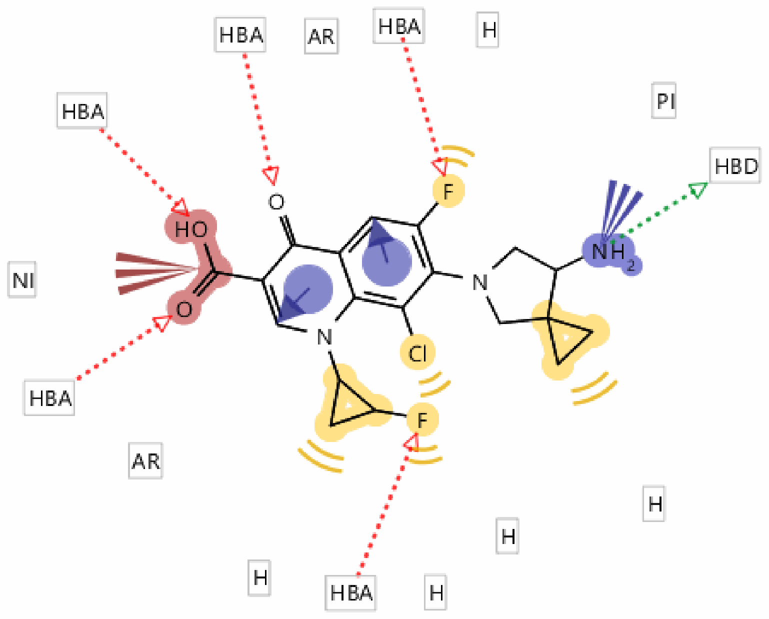
Sparfloxacin	110871-86-8	DZZWHBIBMUVIIW-DTORHVGOSA-N	InChI=1S/C19H22F2N4O3/c1-8-5-24(6-9(2)23-8)17-13(20)15(22)12-16(14(17)21)25(10-3-4-10)7-11(18(12)26)19(27)28/h7-10,23H,3-6,22H2,1-2H3,(H,27,28)/t8-,9+	C[C@@H]1N[C@H](C)CN(C2=C(C(N)=C(C3=C2F)C(C(C(O)=O)=CN3C4CC4)=O)F)C1	DNA gyrase and topoisomerase IV inhibitor	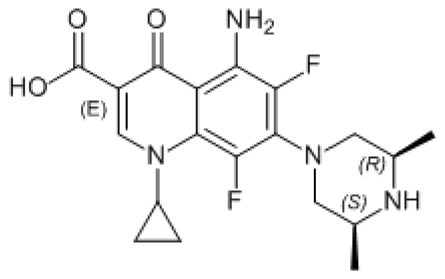	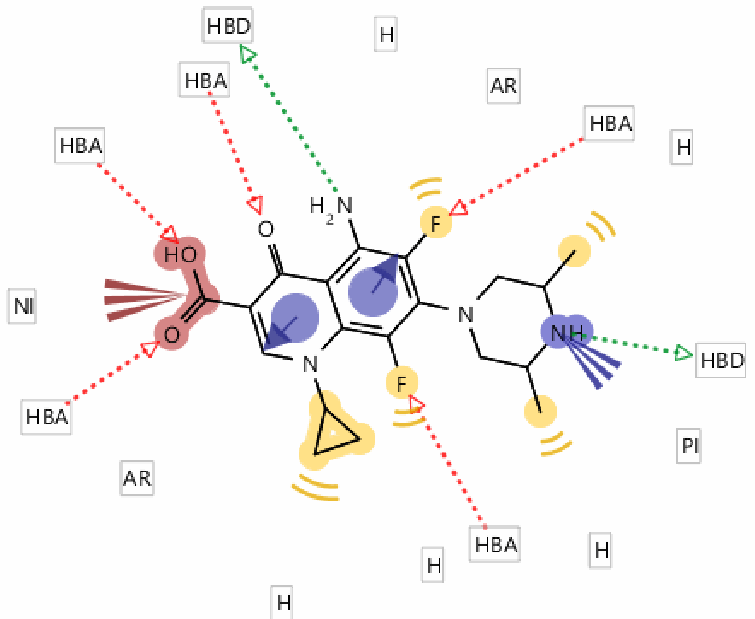
T 14097	55503-43-0	LQPXJLUABZECRT-UHFFFAOYSA-N	InChI=1S/C10H8ClNO3S/c1-2-12-4-6(10(14)15)8(13)5-3-7(11)16-9(5)12/h3-4H,2H2,1H3,(H,14,15)	CCN1C=C(C(O)=O)C(C2=C1SC(Cl)=C2)=O	DNA gyrase and topoisomerase IV inhibitor	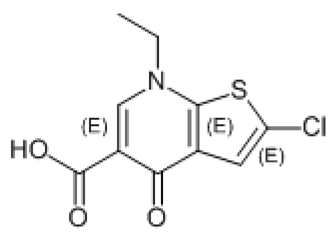	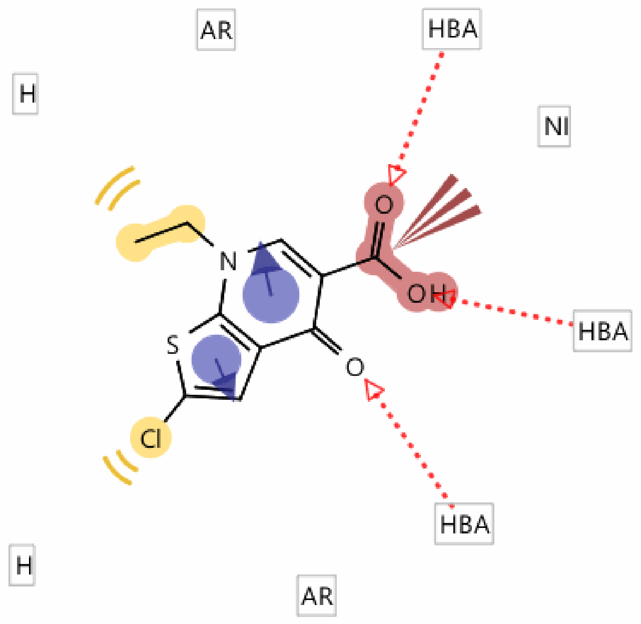

Common name	CAS Reg. No.	InChIKey	InChI	SMILES	Primary target	2D Structure	Pharmacophore[1]
Temafloxacin	108319-06-8	ASYIGCPXSRQVKE-FGYXOPSTSA-N	InChI=1S/2C21H18F3N3O3/c2*1-11-9-26(5-4-25-11)19-8-18-13(7-16(19)24)20(28)14(21(29)30)10-27(18)17-3-2-12(22)6-15(17)23/h2*2-3,6-8,10-11,25H,4-5,9H2,1H3,(H,29,30)/t2*11-/m10/s1	C[C@@H]1CN(CCN1)C2=C(C=C(C3=C2)C(C(C(O)=O)=CN3C4=C(C=C(C=C4)F)F)=O)F.C[C@H]5CN(CCN5)C6=C(C=C(C7=C6)C(C(C(O)=O)=CN7C8=C(C=C(C=C8)F)F)=O)F	DNA gyrase and topoisomerase IV inhibitor	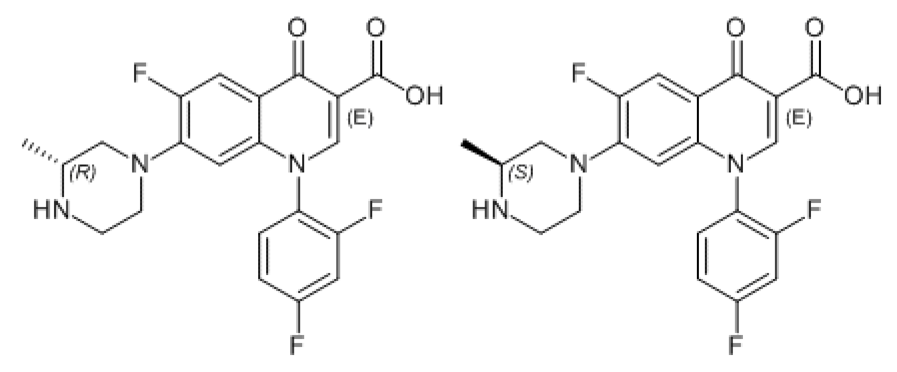	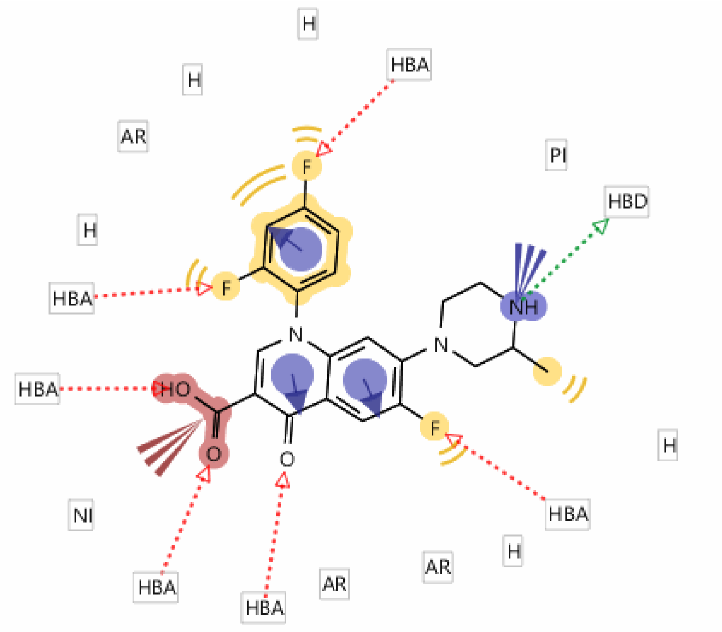
Tioxacin	34976-39-1	VUUPJGYIOAVFAN-UHFFFAOYSA-N	InChI=1S/C14H12N2O4S/c1-3-16-6-7(13(18)19)11(17)10-8(16)4-5-9-12(10)21-14(20)15(9)2/h4-6H,3H2,1-2H3,(H,18,19)	CCN(C1=C2C(S3)=C(N(C3=O)C)C=C1)C=C(C(O)=O)C2=O	DNA gyrase and topoisomerase IV inhibitor	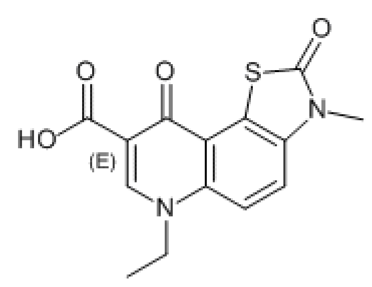	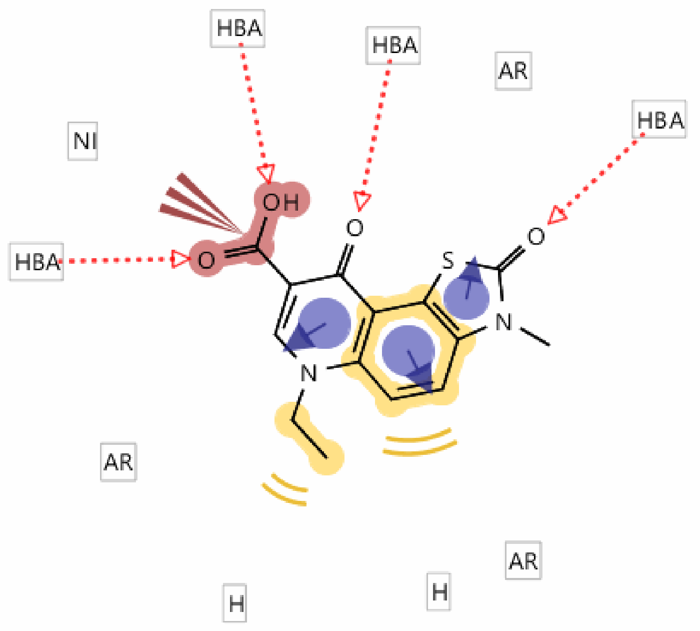
Tosufloxacin	100490-36-6	RBHGNYKXPNUOAL-FTYBWHBYSA-N	InChI=1S/2C19H15F3N4O3/c2*20-9-1-2-15(13(21)5-9)26-8-12(19(28)29)16(27)11-6-14(22)18(24-17(11)26)25-4-3-10(23)7-25/h2*1-2,5-6,8,10H,3-4,7,23H2,(H,28,29)/t2*10-/m10/s1	FC1=CC(F)=C(C=C1)N2C=C(C(C3=CC(F)=C(N=C32)N4CC[C@H](C4)N)=O)C(O)=O.FC5=CC(F)=C(C=C5)N6C=C(C(C7=CC(F)=C(N=C76)N8CC[C@@H](C8)N)=O)C(O)=O	DNA gyrase and topoisomerase IV inhibitor	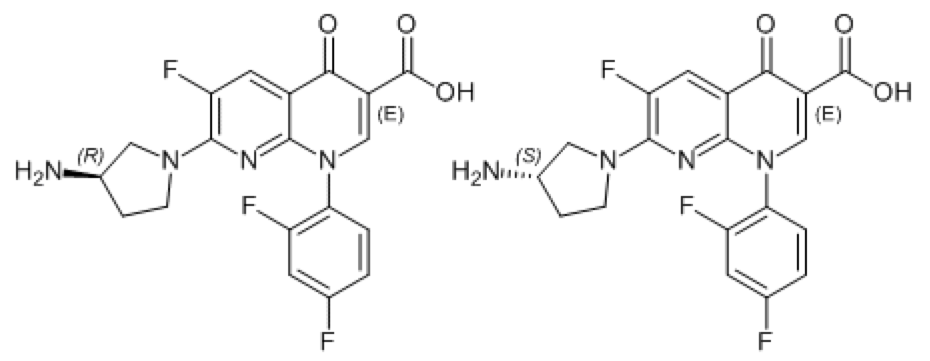	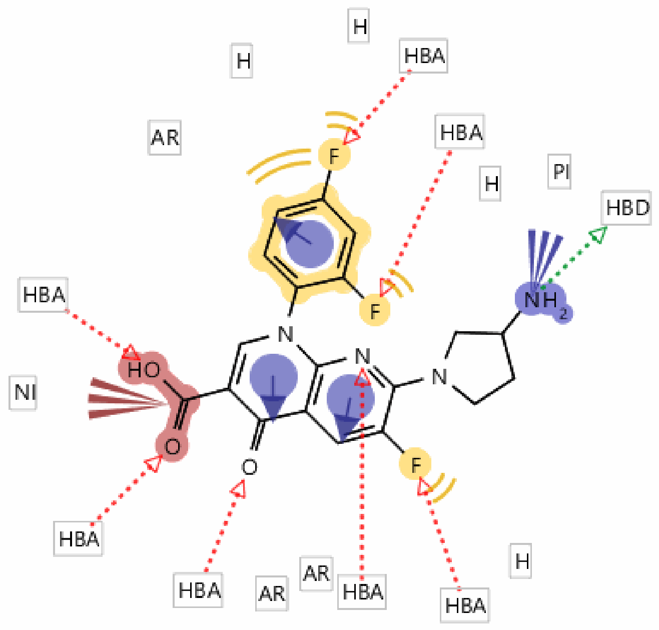
Trovafloxacin	147059-72-1	WVPSKSLAZQPAKQ-CDMJZVDBSA-N	InChI=1S/C20H15F3N4O3/c21-8-1-2-15(13(22)3-8)27-7-12(20(29)30)17(28)9-4-14(23)19(25-18(9)27)26-5-10-11(6-26)16(10)24/h1-4,7,10-11,16H,5-6,24H2,(H,29,30)/t10-,11+,16+	FC1=CC(F)=C(C=C1)N2C=C(C(C3=CC(F)=C(N=C32)N4C[C@@]([C@H]5N)([H])[C@]5(C4)[H])=O)C(O)=O	DNA gyrase and topoisomerase IV inhibitor	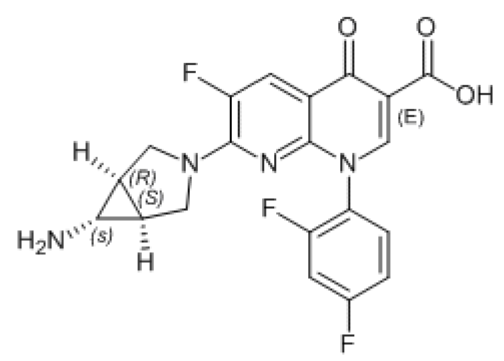	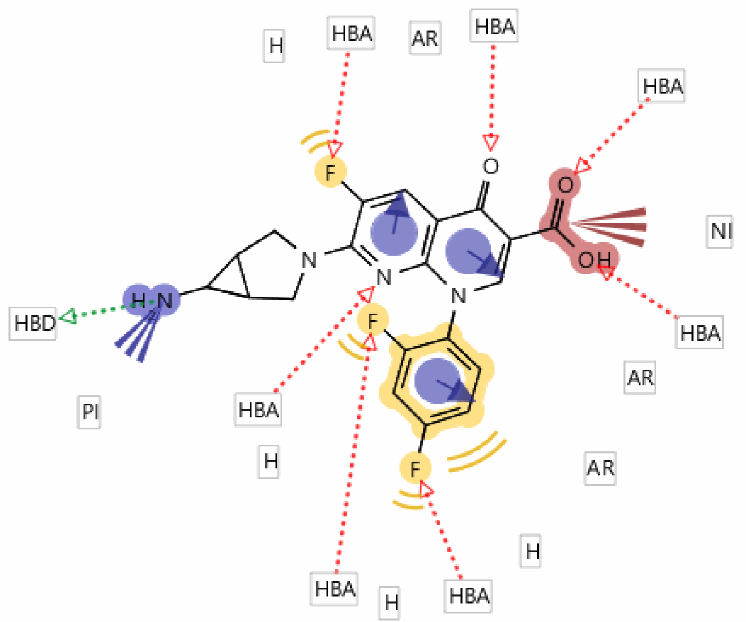
Ulifloxacin	112984-60-8	DGSHRYWXPXYLTN-RMTNWKGQSA-N	InChI=1S/2C16H16FN3O3S/c2*1-8-20-11-7-12(19-4-2-18-3-5-19)10(17)6-9(11)14(21)13(16(22)23)15(20)24-8/h2*6-8,18H,2-5H2,1H3,(H,22,23)/t2*8-/m10/s1	C[C@@H]1N2C3=CC(N4CCNCC4)=C(C=C3C(C(C(O)=O)=C2S1)=O)F.C[C@H]5N6C7=CC(N8CCNCC8)=C(C=C7C(C(C(O)=O)=C6S5)=O)F	DNA gyrase and topoisomerase IV inhibitor	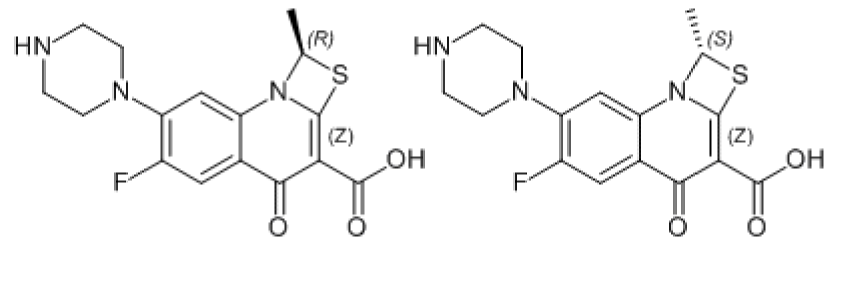	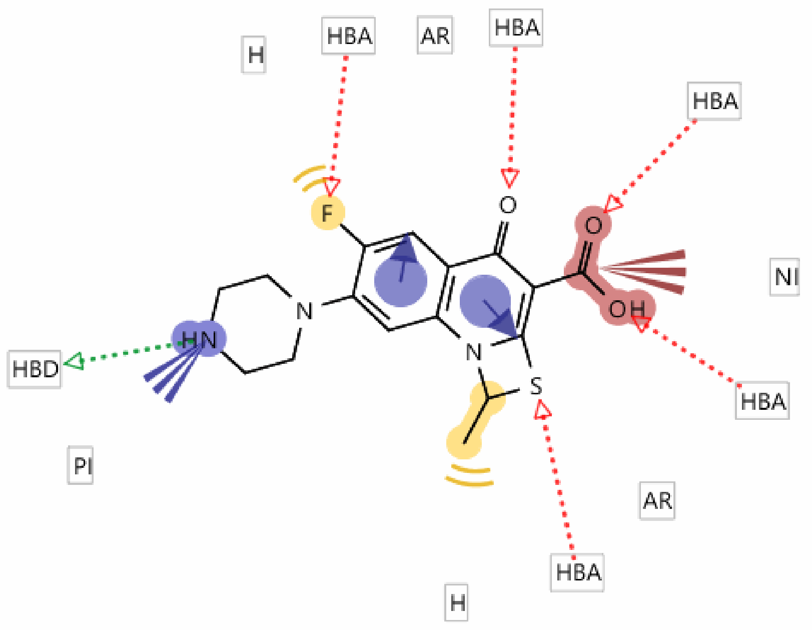
Vebufloxacin (benofloxacin)	79644-90-9	POFCJIUVXMKNOL-FGYXOPSTSA-N	InChI=1S/2C19H22FN3O3/c2*1-11-3-4-12-16-13(18(24)14(19(25)26)10-23(11)16)9-15(20)17(12)22-7-5-21(2)6-8-22/h2*9-11H,3-8H2,1-2H3,(H,25,26)/t2*11-/m10/s1	C[C@@H]1CCC2=C3C(C(C(C(O)=O)=CN13)=O)=CC(F)=C2N4CCN(C)CC4.C[C@H]5CCC6=C7C(C(C(C(O)=O)=CN57)=O)=CC(F)=C6N8CCN(C)CC8	DNA gyrase and topoisomerase IV inhibitor	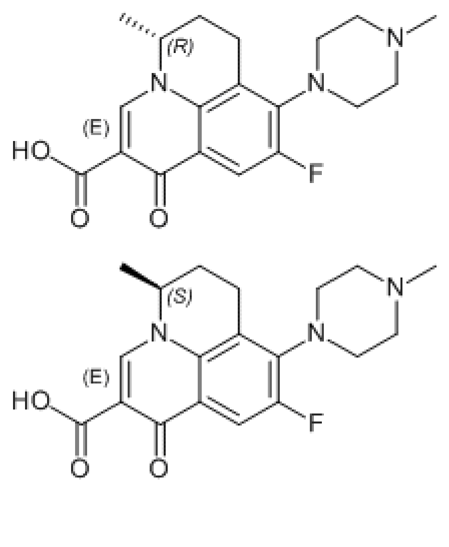	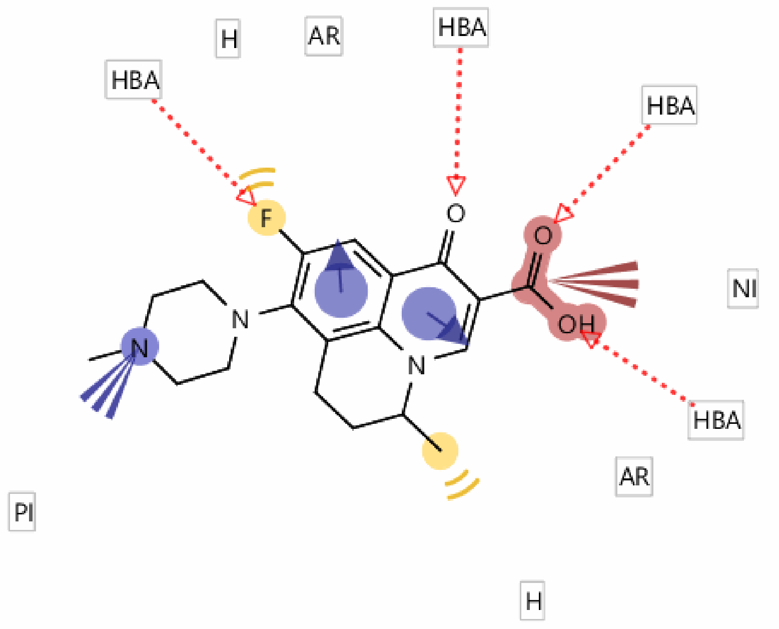

Common name	CAS Reg. No.	InChIKey	InChI	SMILES	Primary target	2D Structure	Pharmacophore[1]
VG 6/1		NSJJLSZVDLRPMW-UHFFFAOYSA-N	InChI=1S/C23H21N3O5/c1-13-20-17(22(27)18(23(28)29)12-25(20)16-6-7-16)10-19(26(30)31)21(13)24-9-8-14-4-2-3-5-15(14)11-24/h2-5,10,12,16H,6-9,11H2,1H3,(H,28,29)	CC1=C2C(C(C(C(O)=O)=CN2C3CC3)=O)=CC([N+]([O-])=O)=C1N4CCC5=CC=CC=C5C4	DNA gyrase and topoisomerase IV inhibitor	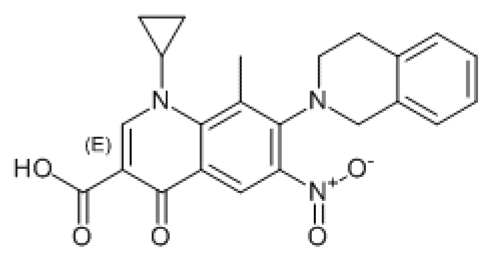	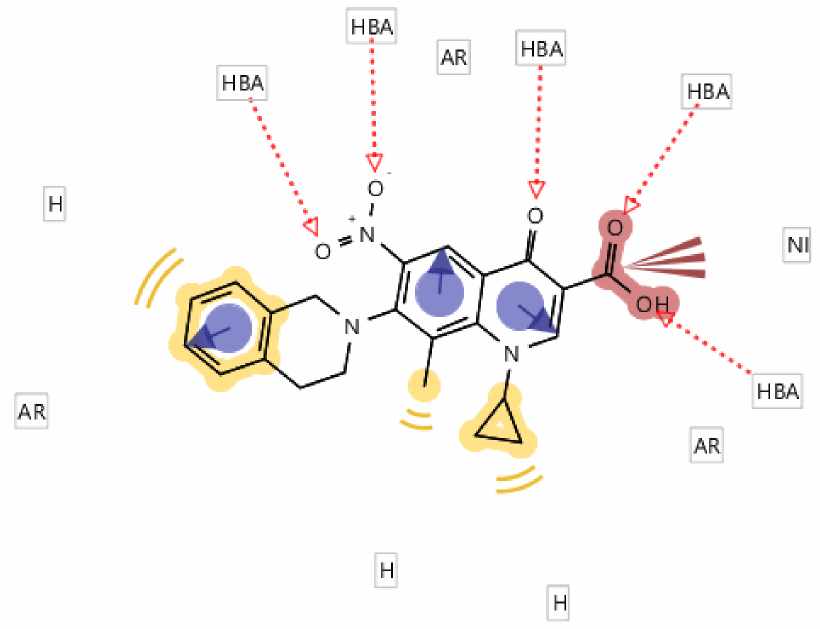
WCK 1152 free base	473839-20-2	PMBHAWNZPPNADR-HNNXBMFYSA-N	InChI=1S/C21H26FN3O4/c1-21(2)10-24(7-6-15(21)23)17-14(22)8-12-16(19(17)29-3)25(11-4-5-11)9-13(18(12)26)20(27)28/h8-9,11,15H,4-7,10,23H2,1-3H3,(H,27,28)/t15-/m0/s1	CC1(CN(C2=C(F)C=C(C(C(C(O)=O)=CN3C4CC4)=O)C3=C2OC)CC[C@@H]1N)C	DNA gyrase and topoisomerase IV inhibitor	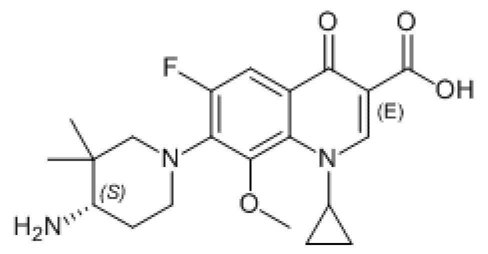	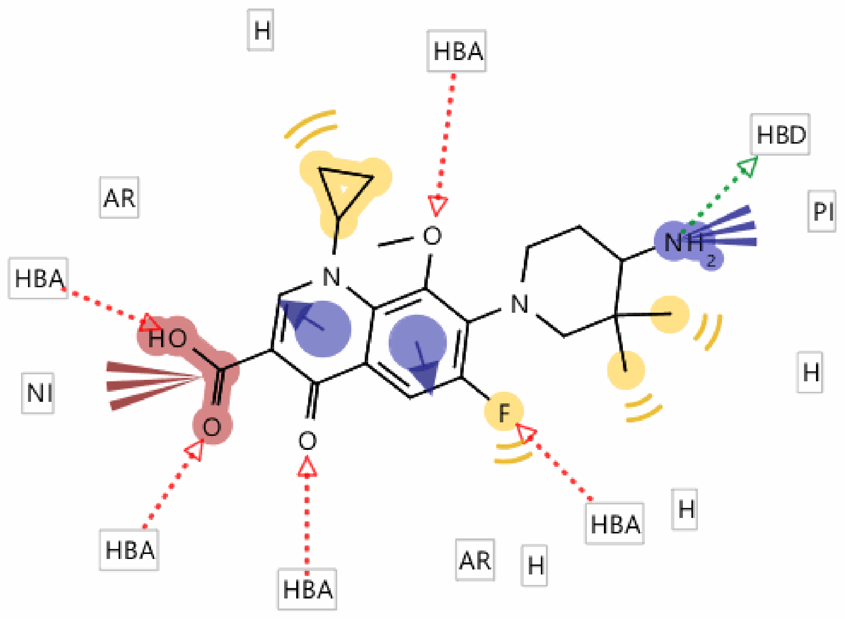
WIN 57273	123942-04-1	FNKLCGHPPNAPBJ-UHFFFAOYSA-N	InChI=1S/C20H17FN2O3/c1-10-5-12(6-11(2)22-10)14-8-18-15(7-17(14)21)19(24)16(20(25)26)9-23(18)13-3-4-13/h5-9,13H,3-4H2,1-2H3,(H,25,26)	CC1=NC(C)=CC(C2=CC3=C(C=C2F)C(C(C(O)=O)=CN3C4CC4)=O)=C1	DNA gyrase and topoisomerase IV inhibitor	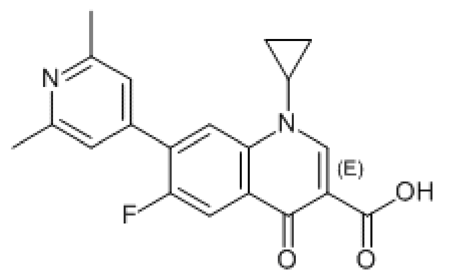	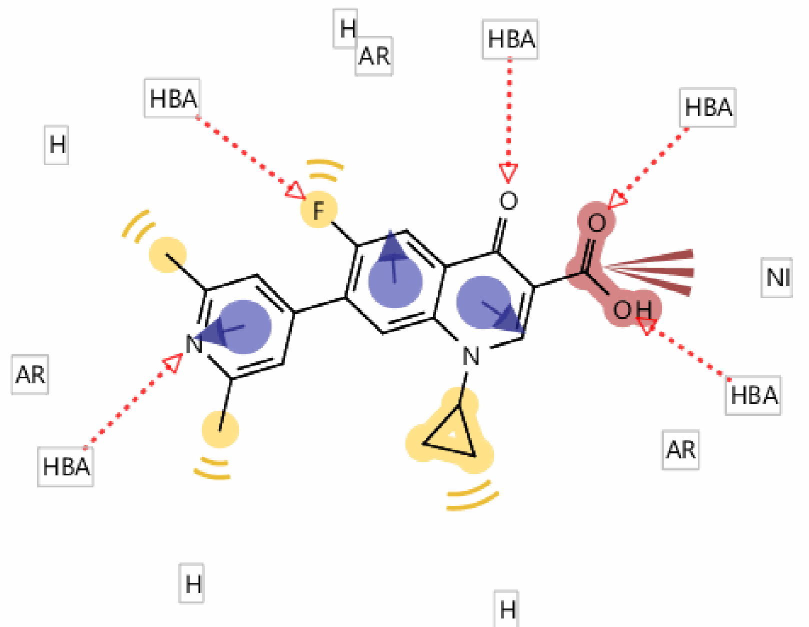
WIN 57294	123942-05-2	WHXJSJBKDGZVDA-UHFFFAOYSA-N	InChI=1S/C20H16F2N2O3/c1-9-5-11(6-10(2)23-9)16-15(21)7-13-18(17(16)22)24(12-3-4-12)8-14(19(13)25)20(26)27/h5-8,12H,3-4H2,1-2H3,(H,26,27)	CC1=NC(C)=CC(C2=C(F)C=C(C(C(C(O)=O)=CN3C4CC4)=O)C3=C2F)=C1	DNA gyrase and topoisomerase IV inhibitor	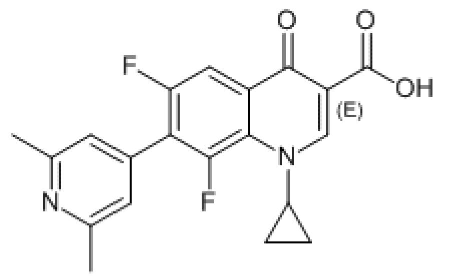	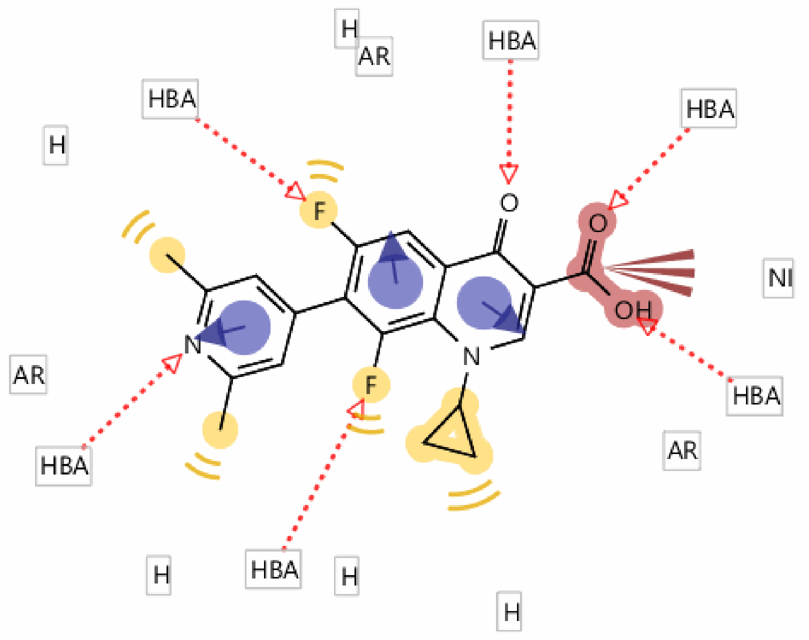
WIN 58161	122033-48-1	DNYDAUUCCWZVJY-NSHDSACASA-N	InChI=1S/C20H17FN2O3S/c1-9-4-12(5-10(2)22-9)16-15(21)6-13-17-19(16)27-8-11(3)23(17)7-14(18(13)24)20(25)26/h4-7,11H,8H2,1-3H3,(H,25,26)/t11-/m0/s1	CC1=NC(C)=CC(C2=C(C=C3C(C(C(O)=O)=CN4C3=C2SC[C@@H]4C)=O)F)=C1	DNA gyrase and topoisomerase IV inhibitor	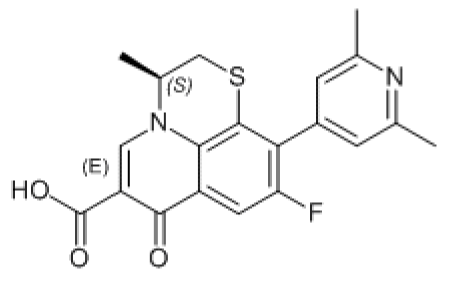	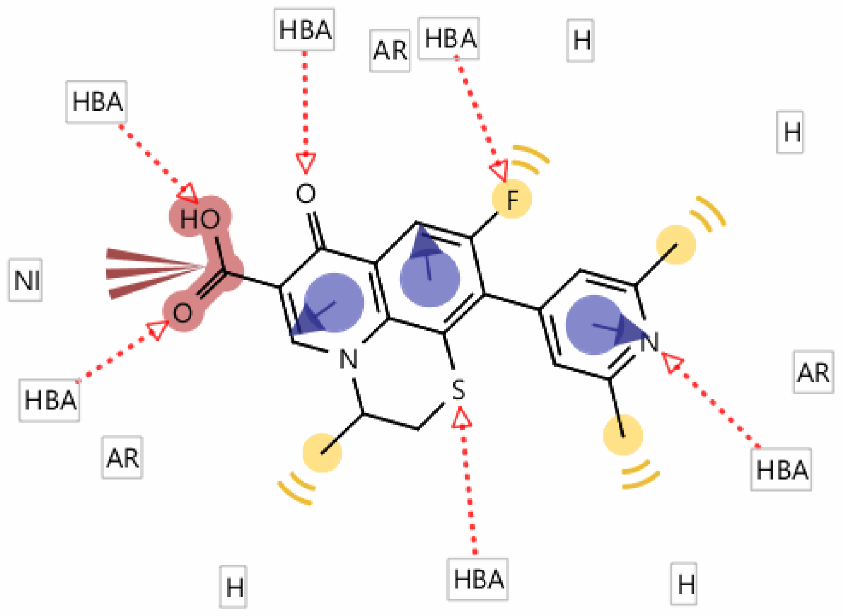
WQ 2743		LQDVQPGHLHYNSA-UHFFFAOYSA-N	InChI=1S/C19H15BrF3N5O3/c1-25-7-4-27(5-7)15-10(21)2-8-14(13(15)20)28(6-9(16(8)29)19(30)31)18-12(23)3-11(22)17(24)26-18/h2-3,6-7,25H,4-5H2,1H3,(H2,24,26)(H,30,31)	CNC1CN(C1)C2=C(C=C3C(N(C=C(C3=O)C(O)=O)C4=NC(N)=C(C=C4F)F)=C2Br)F	DNA gyrase and topoisomerase IV inhibitor	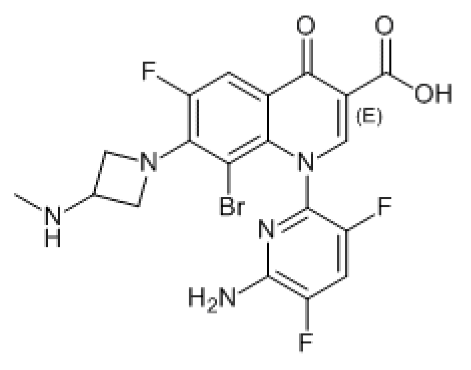	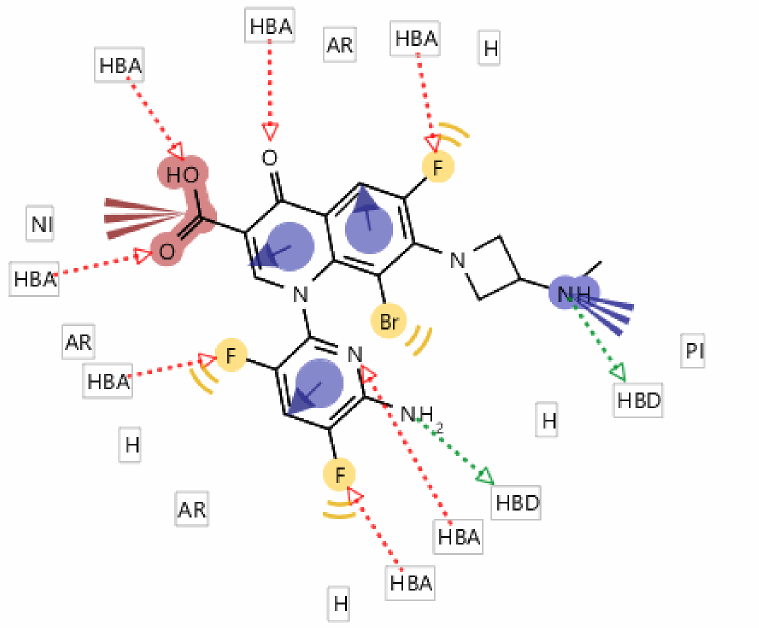

Common name	CAS Reg. No.	InChIKey	InChI	SMILES	Primary target	2D Structure	Pharmacophore[1]
WQ 2756		ATHBCDQPZRXUNJ-UHFFFAOYSA-N	InChI=1S/C19H14ClF3N4O3/c20-15-16-8(1-12(23)17(15)26-4-7(24)5-26)18(28)9(19(29)30)6-27(16)14-3-13(25)10(21)2-11(14)22/h1-3,6-7H,4-5,24-25H2,(H,29,30)	ClC1=C2C(C(C(C(O)=O)=CN2C3=C(C=C(C(N)=C3)F)F)=O)=CC(F)=C1N4CC(C4)N	DNA gyrase and topoisomerase IV inhibitor	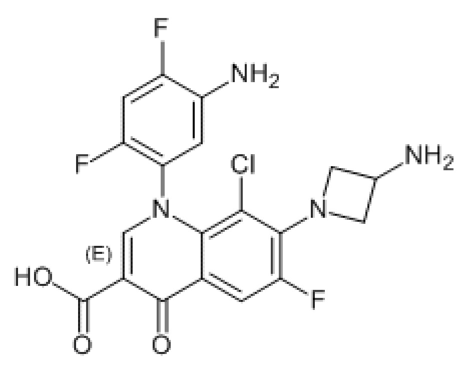	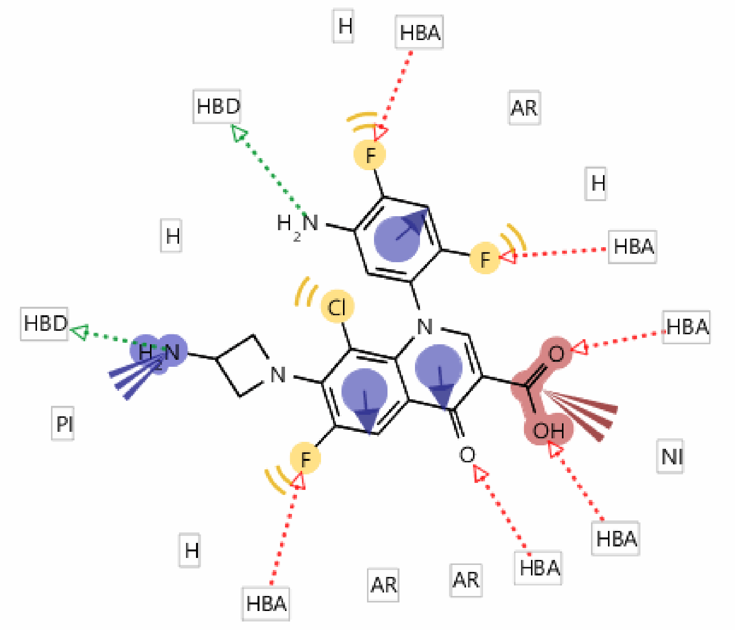
WQ 2908		DOXYSZFWQNMGOR-UHFFFAOYSA-N	InChI=1S/C20H17F3N4O3/c1-8-17-10(2-14(23)18(8)26-5-9(24)6-26)19(28)11(20(29)30)7-27(17)16-4-15(25)12(21)3-13(16)22/h2-4,7,9H,5-6,24-25H2,1H3,(H,29,30)	CC1=C2C(C(C(C(O)=O)=CN2C3=C(C=C(C(N)=C3)F)F)=O)=CC(F)=C1N4CC(C4)N	DNA gyrase and topoisomerase IV inhibitor	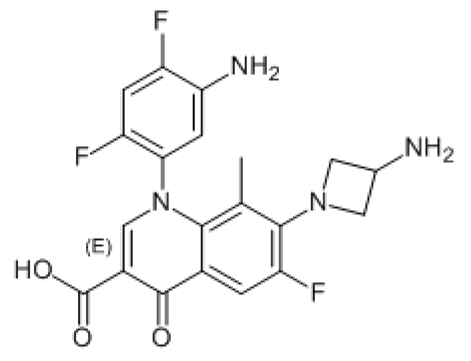	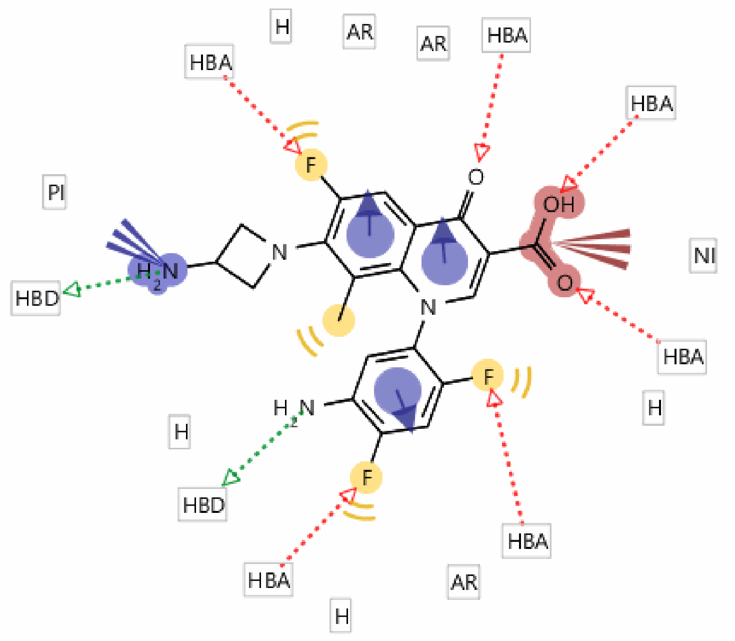
WQ 2942		ODKHMADULYCXOQ-UHFFFAOYSA-N	InChI=1S/C17H12F3N3O3/c1-6-14(22)11(20)2-7-15(6)23(5-8(16(7)24)17(25)26)13-4-12(21)9(18)3-10(13)19/h2-5H,21-22H2,1H3,(H,25,26)	CC1=C(C(F)=CC2=C1N(C=C(C2=O)C(O)=O)C3=C(C=C(C(N)=C3)F)F)N	DNA gyrase and topoisomerase IV inhibitor	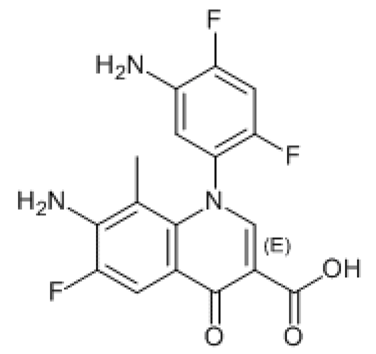	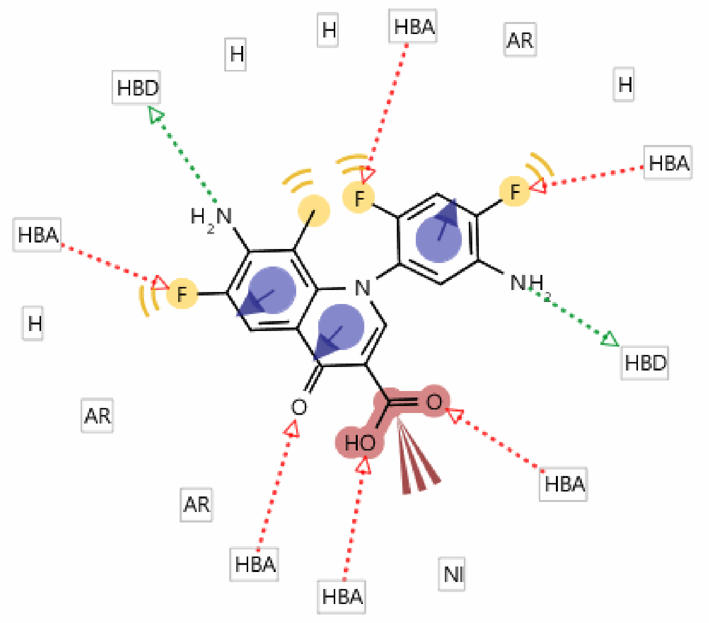
WQ 3330		FUQDVYUDQZJARD-UHFFFAOYSA-N	InChI=1S/C22H21F3N4O3/c1-3-27-11-7-28(8-11)20-10(2)19-12(4-16(20)25)21(30)13(22(31)32)9-29(19)18-6-17(26)14(23)5-15(18)24/h4-6,9,11,27H,3,7-8,26H2,1-2H3,(H,31,32)	CCNC1CN(C2=C(F)C=C(C(C(C(O)=O)=CN3C4=C(F)C=C(F)C(N)=C4)=O)C3=C2C)C1	DNA gyrase and topoisomerase IV inhibitor	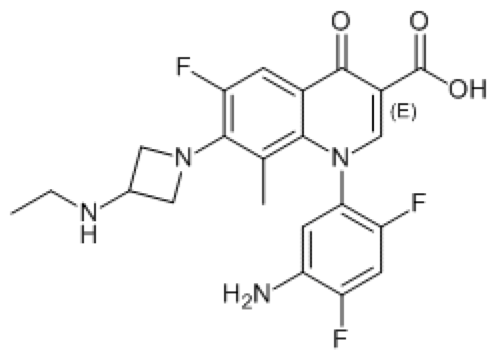	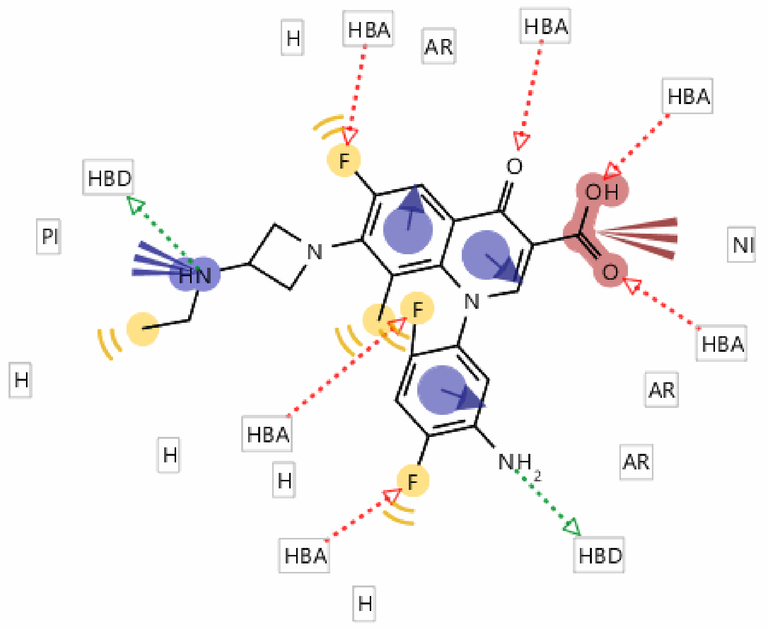
Y 688		AGRJUZPPATWGHI-FQEVSTJZSA-N	InChI=1S/C20H23F2N3O4/c1-29-18-15-12(17(26)13(19(27)28)7-25(15)11-2-3-11)6-14(22)16(18)24-5-4-20(8-21,9-23)10-24/h6-7,11H,2-5,8-10,23H2,1H3,(H,27,28)/t20-/m0/s1	COC1=C2C(C(C(C(O)=O)=CN2C3CC3)=O)=CC(F)=C1N4CC[C@](CF)(CN)C4	DNA gyrase and topoisomerase IV inhibitor	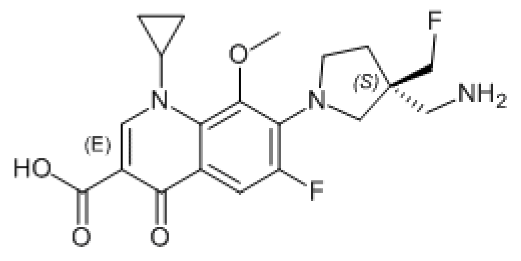	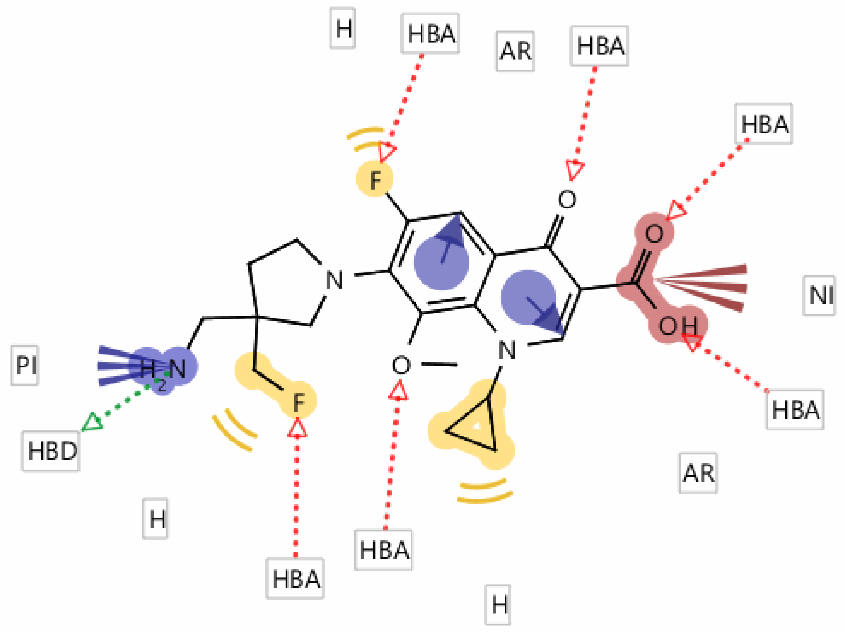
Zabofloxacin	219680-11-2	ZNPOCLHDJCAZAH-UHFFFAOYSA-N	InChI=1S/C19H20FN5O4/c1-29-23-14-6-24(9-19(14)7-21-8-19)17-13(20)4-11-15(26)12(18(27)28)5-25(10-2-3-10)16(11)22-17/h4-5,10,21H,2-3,6-9H2,1H3,(H,27,28)	CO/N=C1CN(C2=C(F)C=C(C3=N2)C(C(C(O)=O)=CN3C4CC4)=O)CC5/1CNC5	DNA gyrase and topoisomerase IV inhibitor	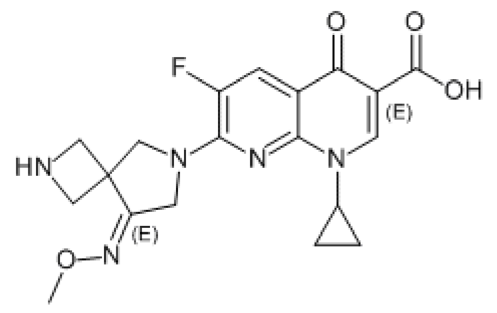	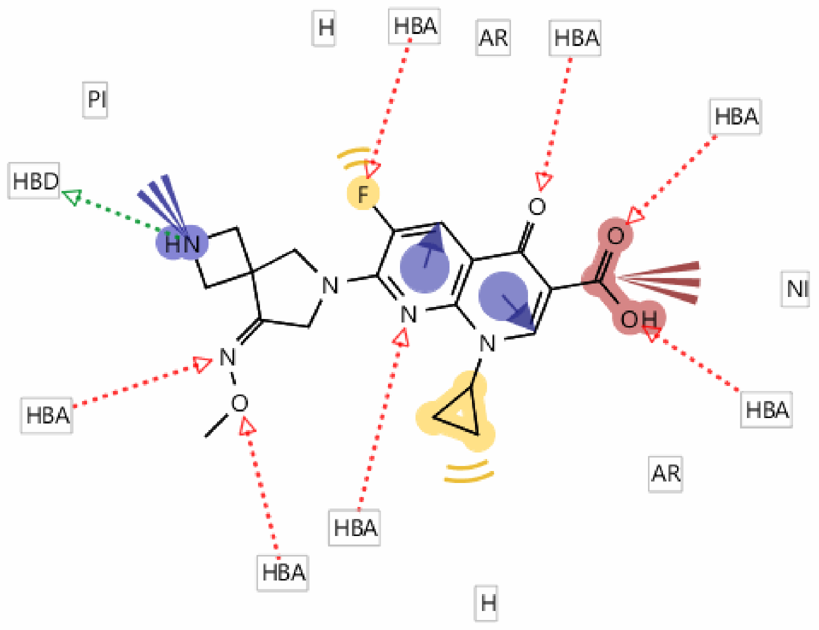

Common name	CAS Reg. No.	InChIKey	InChI	SMILES	Primary target	2D Structure	Pharmacophore[1]
Azidamfenicol	13838-08-9	SGRUZFCHLOFYHZ-MWLCHTKSSA-N	InChI=1S/C11H13N5O5/c12-15-13-5-10(18)14-9(6-17)11(19)7-1-3-8(4-2-7)16(20)21/h1-4,9,11,17,19H,5-6H2,(H,14,18)/t9-,11-/m1/s1	OC[C@@]([H])(NC(CN=[N+]=[N-])=O)[C@H](O)C1=CC=C([N+]([O-])=O)C=C1	Large ribosomal subunit inhibitor	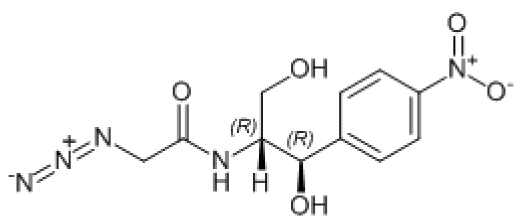	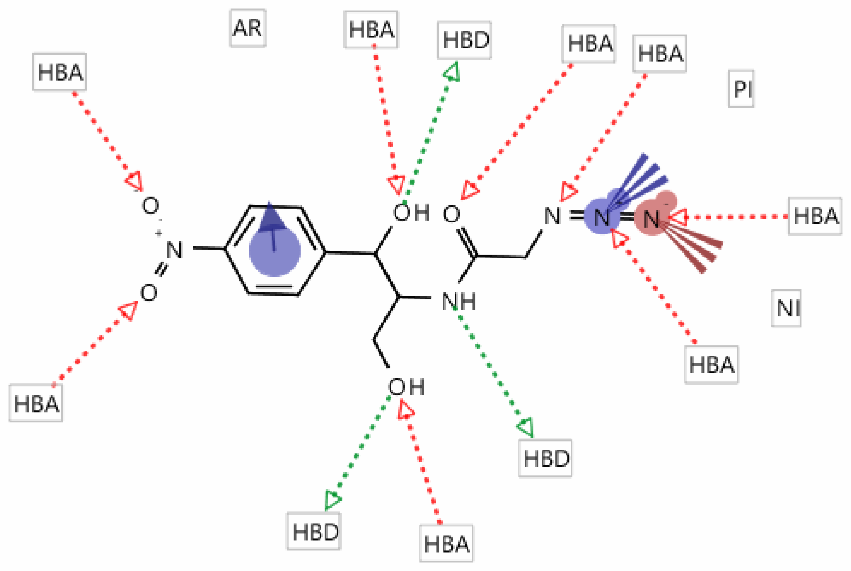
Bromamphenicol (bromoamphenicol)	40027-72-3	DCFUCPPWRWFUFZ-MWLCHTKSSA-N	InChI=1S/C11H13BrN2O5/c12-5-10(16)13-9(6-15)11(17)7-1-3-8(4-2-7)14(18)19/h1-4,9,11,15,17H,5-6H2,(H,13,16)/t9-,11-/m1/s1	OC[C@@]([H])(NC(CBr)=O)[C@H](O)C1=CC=C([N+]([O-])=O)C=C1	Large ribosomal subunit inhibitor	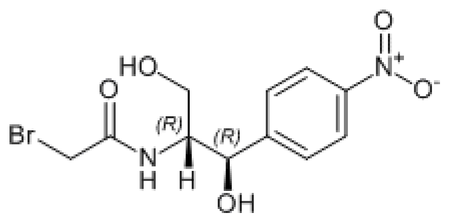	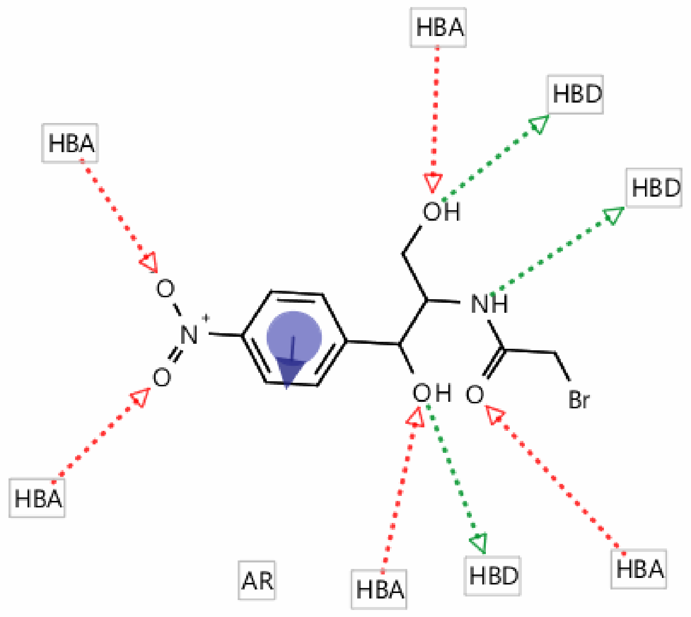
Cetofenicol (cetophenicol)	735-52-4	PKUBDVAOXLEWBF-GHMZBOCLSA-N	InChI=1S/C13H15Cl2NO4/c1-7(18)8-2-4-9(5-3-8)11(19)10(6-17)16-13(20)12(14)15/h2-5,10-12,17,19H,6H2,1H3,(H,16,20)/t10-,11-/m1/s1	CC(C1=CC=C(C=C1)[C@H]([C@@]([H])(CO)NC(C(Cl)Cl)=O)O)=O	Large ribosomal subunit inhibitor	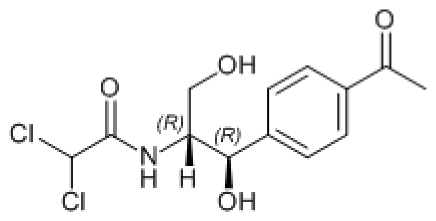	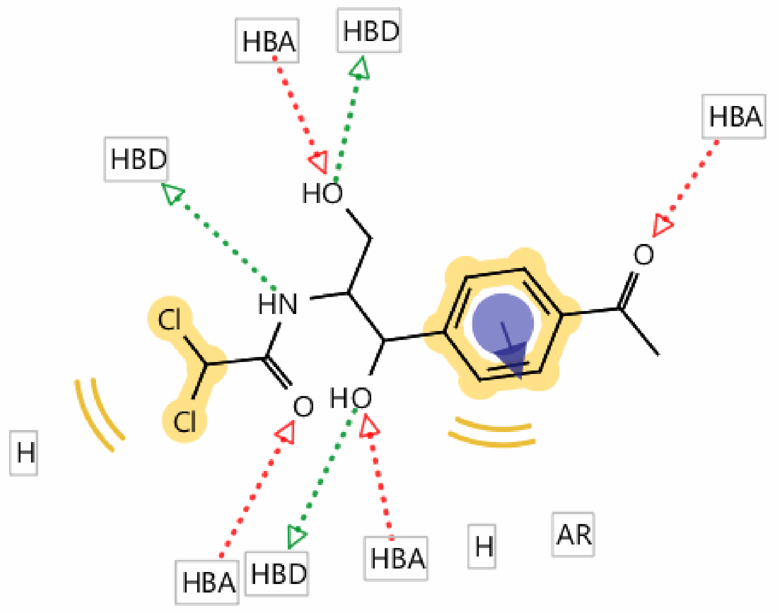
Chloramphenicol	56-75-7	WIIZWVCIJKGZOK-RKDXNWHRSA-N	InChI=1S/C11H12Cl2N2O5/c12-10(13)11(18)14-8(5-16)9(17)6-1-3-7(4-2-6)15(19)20/h1-4,8-10,16-17H,5H2,(H,14,18)/t8-,9-/m1/s1	OC[C@@]([C@@H](C1=CC=C(C=C1)[N+]([O-])=O)O)([H])NC(C(Cl)Cl)=O	Large ribosomal subunit inhibitor	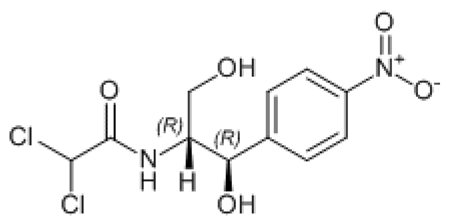	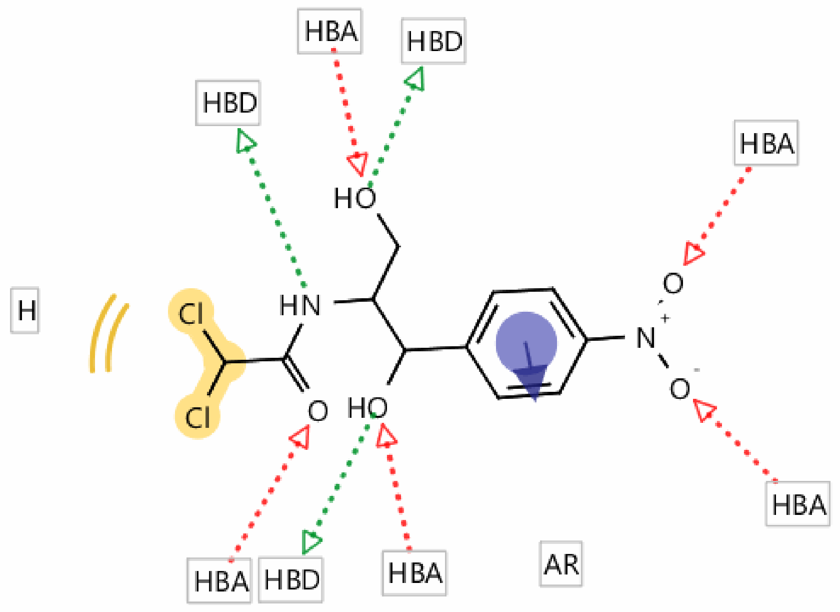
Florfenicol	73231-34-2	AYIRNRDRBQJXIF-NXEZZACHSA-N	InChI=1S/C12H14Cl2FNO4S/c1-21(19,20)8-4-2-7(3-5-8)10(17)9(6-15)16-12(18)11(13)14/h2-5,9-11,17H,6H2,1H3,(H,16,18)/t9-,10-/m1/s1	CS(=O)(C1=CC=C(C=C1)[C@H]([C@@]([H])(CF)NC(C(Cl)Cl)=O)O)=O	Large ribosomal subunit inhibitor	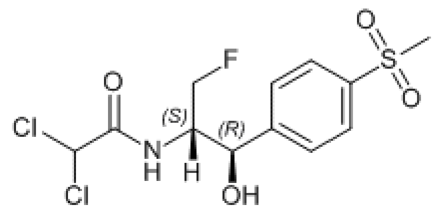	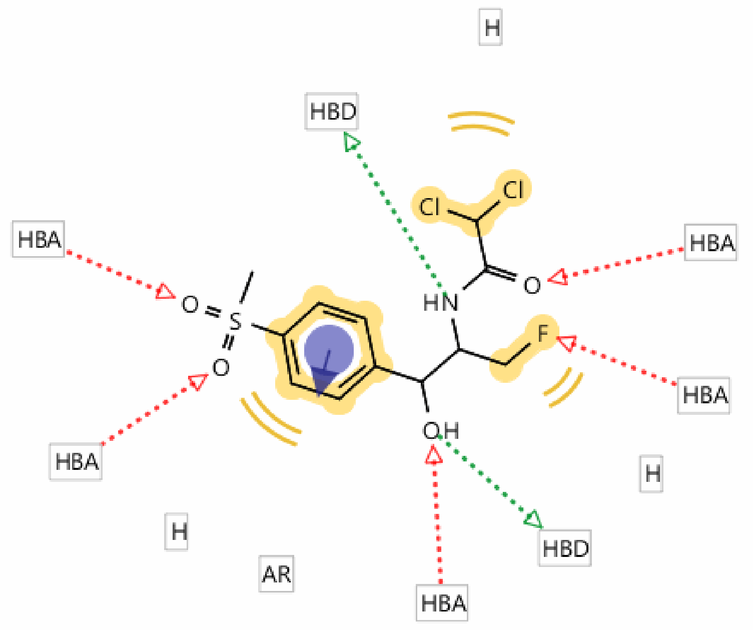
Monoiodoamphenicol	40027-73-4	YRWBLCUITDTYCT-MWLCHTKSSA-N	InChI=1S/C11H13IN2O5/c12-5-10(16)13-9(6-15)11(17)7-1-3-8(4-2-7)14(18)19/h1-4,9,11,15,17H,5-6H2,(H,13,16)/t9-,11-/m1/s1	OC[C@@]([H])(NC(CI)=O)[C@H](O)C1=CC=C([N+]([O-])=O)C=C1	Large ribosomal subunit inhibitor	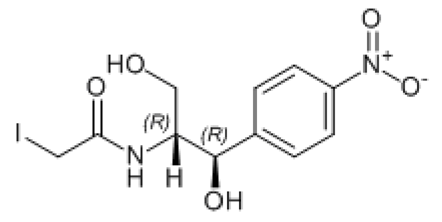	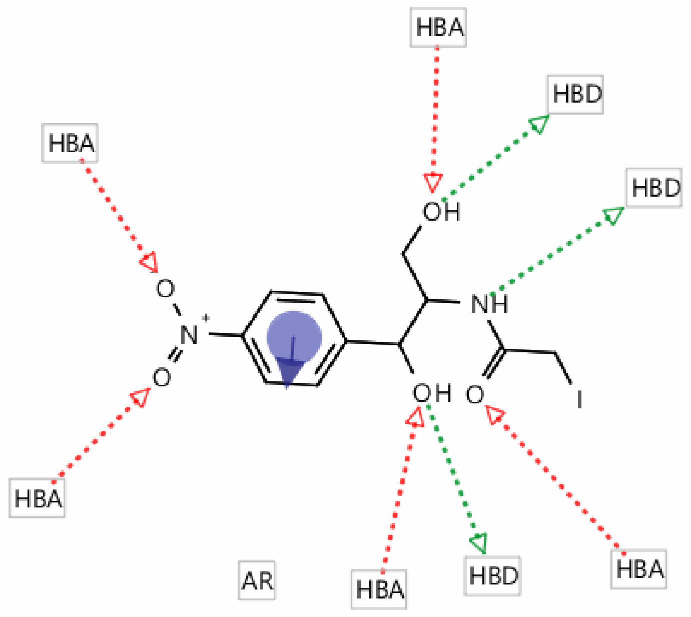

Common name	CAS Reg. No.	InChIKey	InChI	SMILES	Primary target	2D Structure	Pharmacophore[1]
Racefenicol (racephenicol)	847-25-6	JNKVGZRTMPMVAG-KSBPLQDLSA-N	InChI=1S/2C12H15Cl2NO5S/c2*1-21(19,20)8-4-2-7(3-5-8)10(17)9(6-16)15-12(18)11(13)14/h2*2-5,9-11,16-17H,6H2,1H3,(H,15,18)/t2*9-,10-/m10/s1	CS(C1=CC=C(C=C1)[C@H]([C@@]([H])(CO)NC(C(Cl)Cl)=O)O)(=O)=O.CS(C2=CC=C(C=C2)[C@@H]([C@]([H])(CO)NC(C(Cl)Cl)=O)O)(=O)=O	Large ribosomal subunit inhibitor	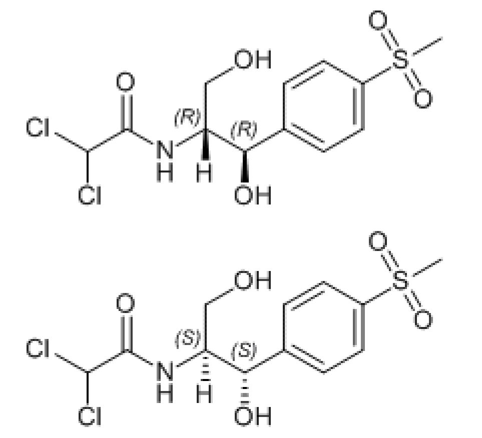	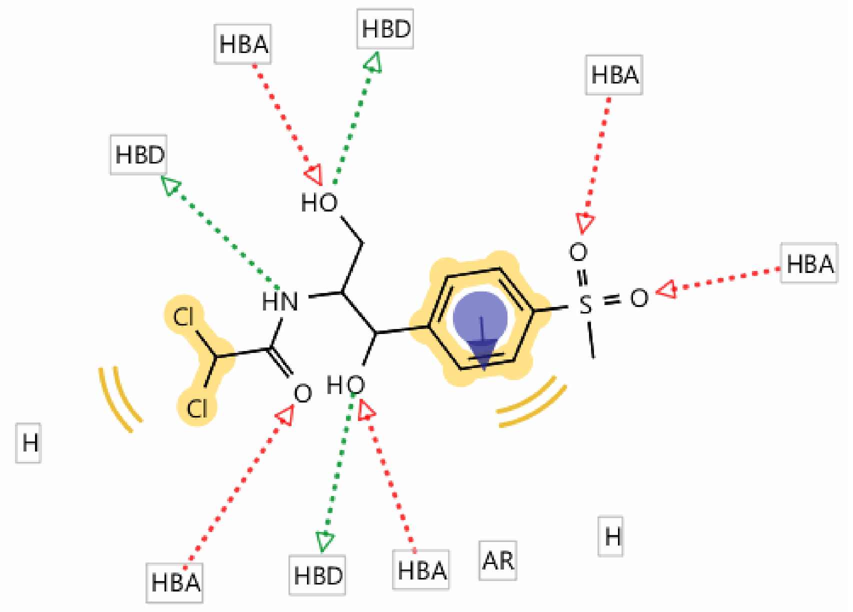
Tevenel	4302-95-8	HODRFAVLXIFVTR-RKDXNWHRSA-N	InChI=1S/C11H14Cl2N2O5S/c12-10(13)11(18)15-8(5-16)9(17)6-1-3-7(4-2-6)21(14,19)20/h1-4,8-10,16-17H,5H2,(H,15,18)(H2,14,19,20)/t8-,9-/m1/s1	OC[C@@]([H])(NC(C(Cl)Cl)=O)[C@H](O)C1=CC=C(S(=O)(N)=O)C=C1	Large ribosomal subunit inhibitor	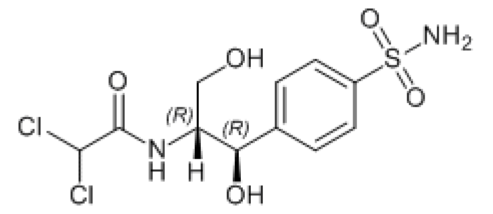	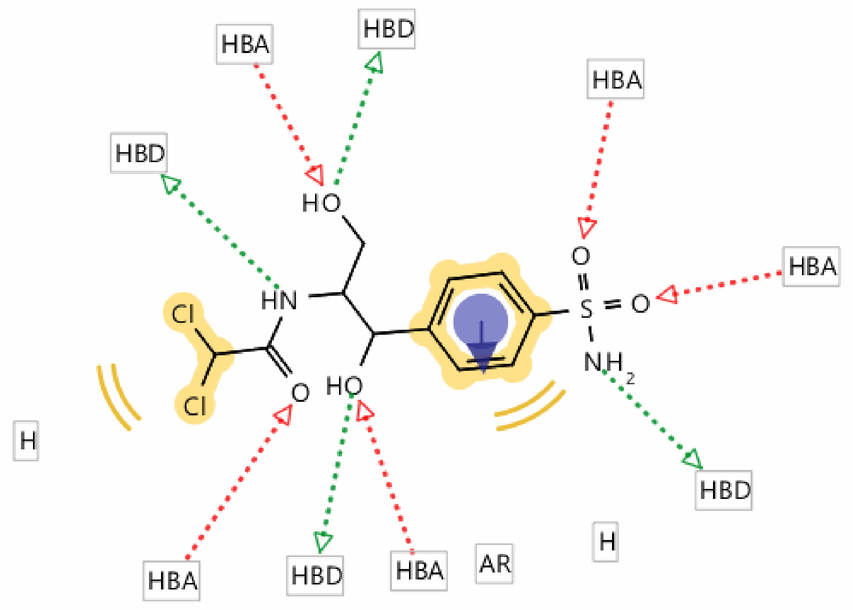
Thiamphenicol	15318-45-3	OTVAEFIXJLOWRX-NXEZZACHSA-N	InChI=1S/C12H15Cl2NO5S/c1-21(19,20)8-4-2-7(3-5-8)10(17)9(6-16)15-12(18)11(13)14/h2-5,9-11,16-17H,6H2,1H3,(H,15,18)/t9-,10-/m1/s1	CS(=O)(C1=CC=C([C@@H](O)[C@]([H])(NC(C(Cl)Cl)=O)CO)C=C1)=O	Large ribosomal subunit inhibitor	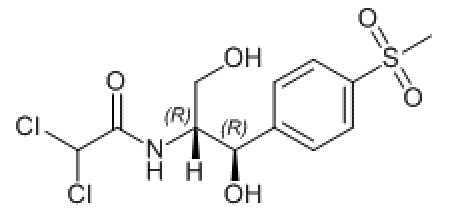	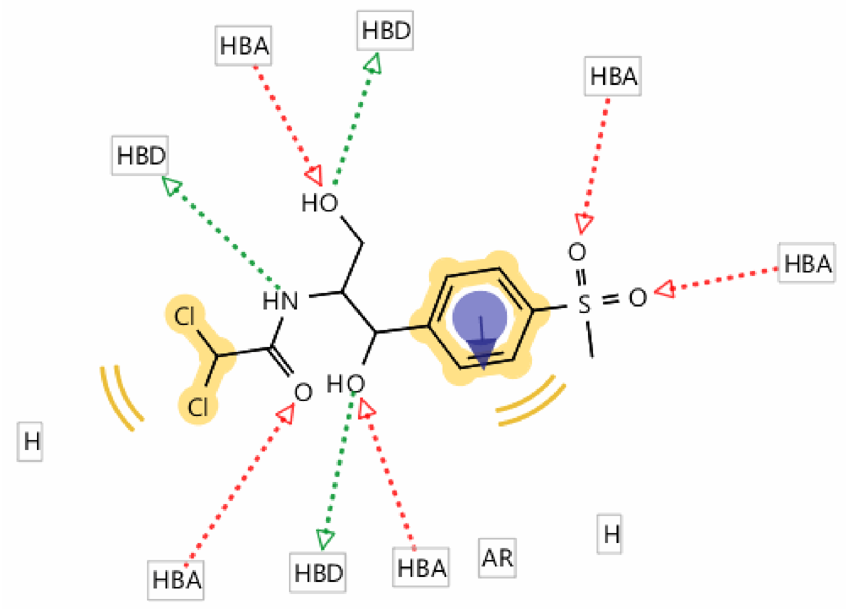
WIN 5094-2		HYMCEAYSFJCEJN-NXEZZACHSA-N	InChI=1S/C12H15Cl2NO3S/c1-19-8-4-2-7(3-5-8)10(17)9(6-16)15-12(18)11(13)14/h2-5,9-11,16-17H,6H2,1H3,(H,15,18)/t9-,10-/m1/s1	CSC1=CC=C(C=C1)[C@H]([C@@]([H])(CO)NC(C(Cl)Cl)=O)O	Large ribosomal subunit inhibitor	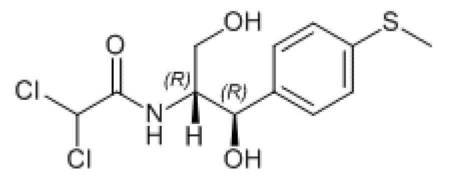	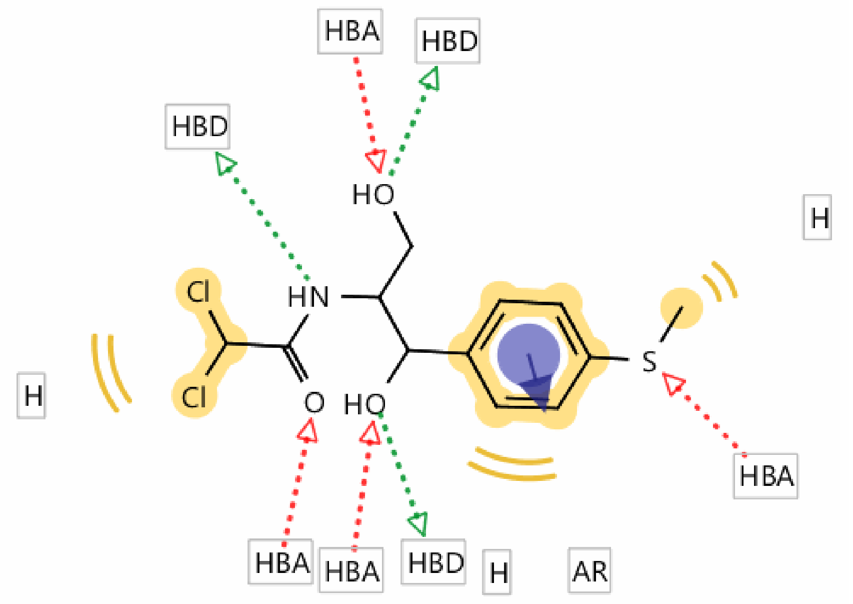
9-Methylstreptimidone (S 632A$_2$)		ATUBIBZJAGAIBW-WGEALTPQSA-N	InChI=1S/C17H25NO4/c1-4-5-11(2)6-12(3)15(20)10-14(19)7-13-8-16(21)18-17(22)9-13/h4-6,12-14,19H,7-10H2,1-3H3,(H,18,21,22)/b5-4-,11-6+/t12-,14+/m0/s1	O=C(N1)CC(C[C@@H](O)CC([C@@H](C)/C=C(C)/C=C\C)=O)CC1=O	Large ribosomal subunit inhibitor	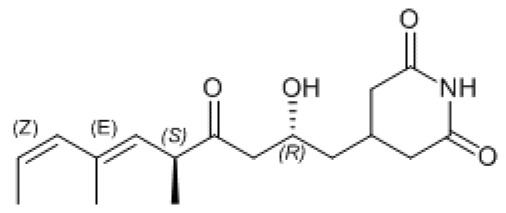	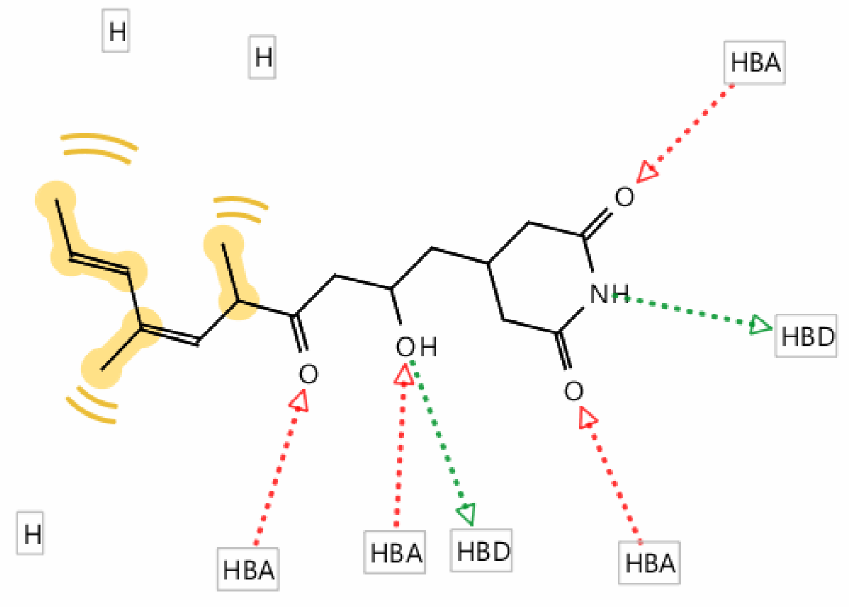
Acetoxycycloheximide (streptovitacin E-73)	2885-39-4	UFDHNJJHPSGMFX-SQUSCZTCSA-N	InChI=1S/C17H25NO6/c1-9-7-17(3,24-10(2)19)8-12(16(9)23)13(20)4-11-5-14(21)18-15(22)6-11/h9,11-13,20H,4-8H2,1-3H3,(H,18,21,22)/t9-,12-,13+,17+/m0/s1	C[C@H](C1=O)C[C@](C)(C[C@@]1([H])[C@H](O)CC(C2)CC(NC2=O)=O)OC(C)=O	Large ribosomal subunit inhibitor	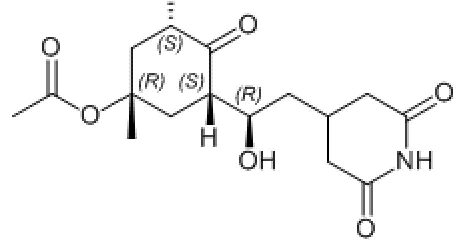	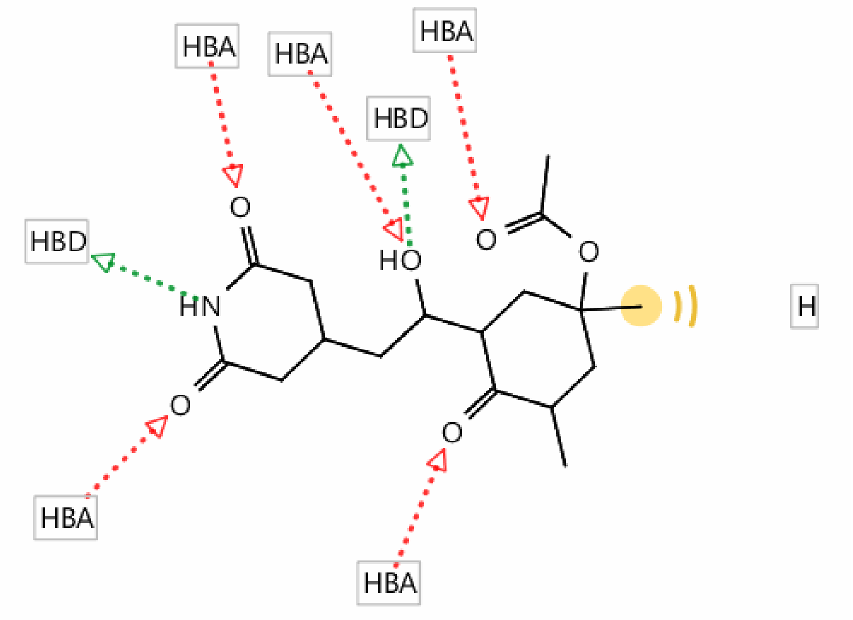

Common name	CAS Reg. No.	InChIKey	InChI	SMILES	Primary target	2D Structure	Pharmacophore[1]
Actiketal	133658-47-6	JGRHJZLSKWAAPV-HNNXBMFYSA-N	InChI=1S/C15H15NO5/c1-7-3-8(2)13-10(4-7)14(19)15(20,21-13)9-5-11(17)16-12(18)6-9/h3-4,9,20H,5-6H2,1-2H3,(H,16,17,18)/t15-/m0/s1	CC1=CC(C)=C(O[C@@](C(C2)CC(NC2=O)=O)(C3=O)O)C3=C1	Large ribosomal subunit inhibitor	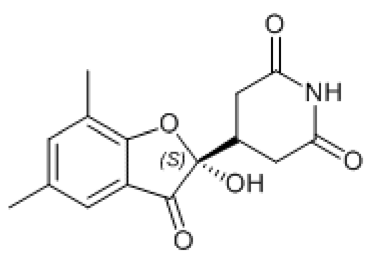	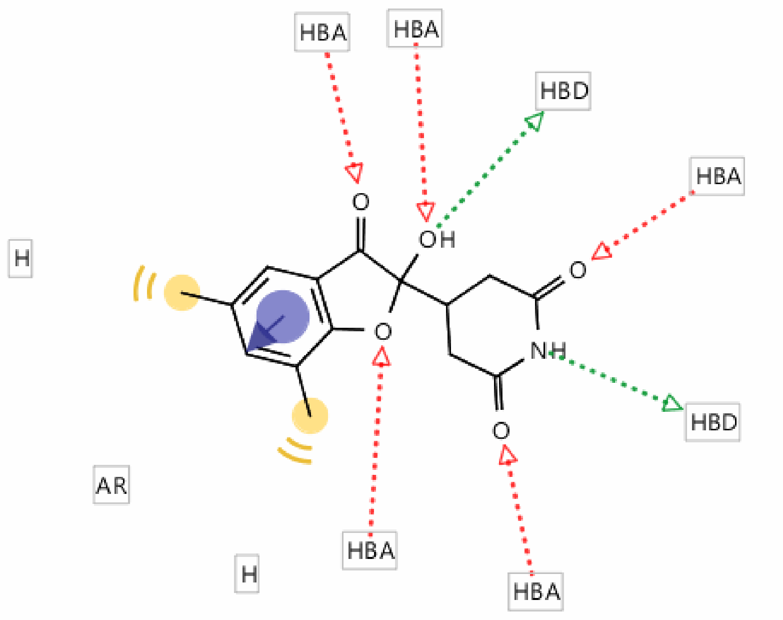
Actiphenol (actinophenol)	526-02-3	YTLMIHBTPWTPEV-UHFFFAOYSA-N	InChI=1S/C15H17NO4/c1-8-3-9(2)15(20)11(4-8)12(17)5-10-6-13(18)16-14(19)7-10/h3-4,10,20H,5-7H2,1-2H3,(H,16,18,19)	CC1=CC(C)=C(C(C(CC(CC(N2)=O)C C2=O)=O)=C1)O	Large ribosomal subunit inhibitor	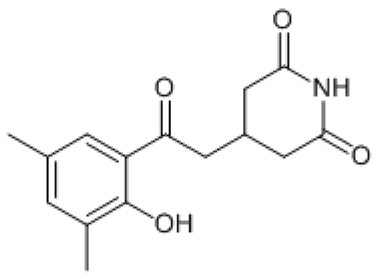	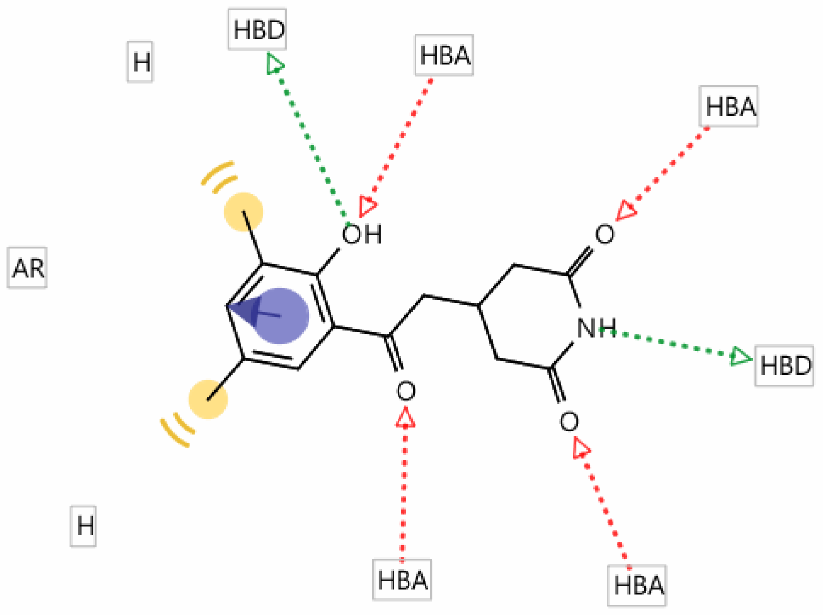
Cycloheximide	66-81-9	YPHMISFOHDHNIV-FSZOTQKASA-N	InChI=1S/C15H23NO4/c1-8-3-9(2)15(20)11(4-8)12(17)5-10-6-13(18)16-14(19)7-10/h8-12,17H,3-7H2,1-2H3,(H,16,18,19)/t8-,9-,11-,12+/m0/s1	C[C@H](C[C@@]1([H])[C@H](O)CC(C2)CC(NC2=O)=O)C[C@@H](C1=O)C	Large ribosomal subunit inhibitor	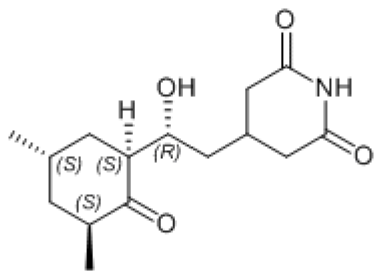	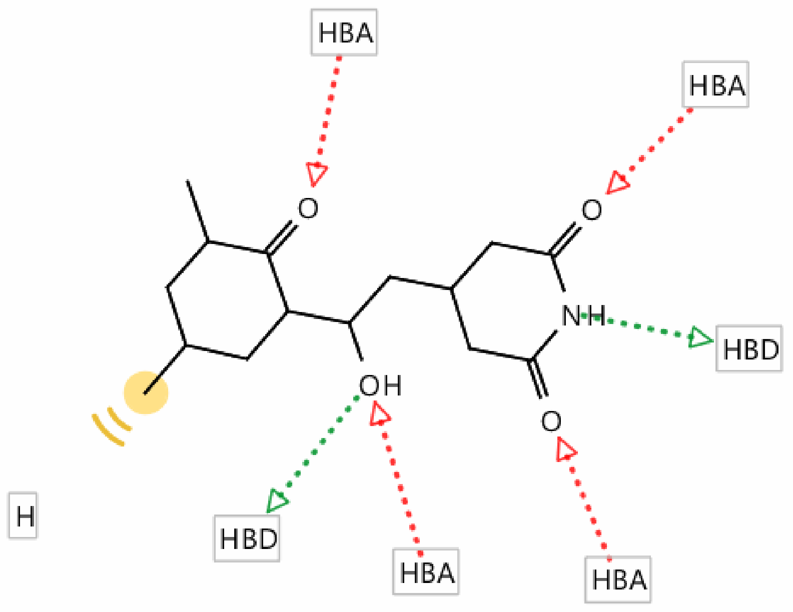
Epiderstatin	126602-16-2	WFHHLLAMGXGSRT-RIIAMCTJSA-N	InChI=1S/C15H20N2O4/c1-8-3-9(2)15(21)16-12(8)7-11(18)4-10-5-13(19)17-14(20)6-10/h7-10H,3-6H2,1-2H3,(H,16,21)(H,17,19,20)/b12-7-/t8-,9-/m1/s1	O=C(/C=C([C@@H](C[C@H]1C)C)\NC1=O)CC2CC(NC(C2)=O)=O	Large ribosomal subunit inhibitor	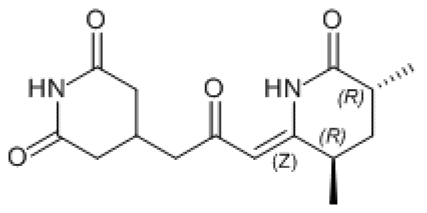	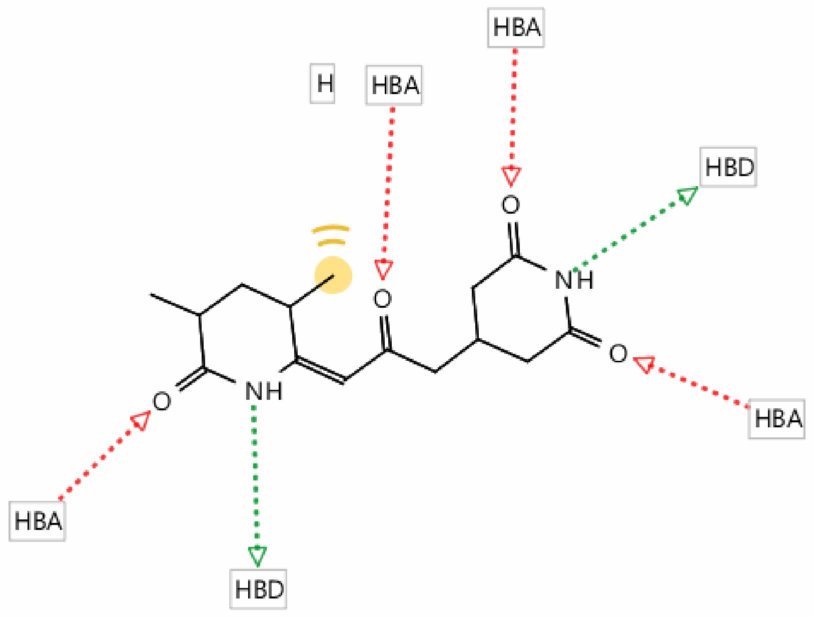
Inactone	487-34-3	GCCDDVHZHSSEMD-HOTUBEGUSA-N	InChI=1S/C15H21NO4/c1-8-3-9(2)15(20)11(4-8)12(17)5-10-6-13(18)16-14(19)7-10/h4,8-10,12,17H,3,5-7H2,1-2H3,(H,16,18,19)/t8-,9-,12+/m0/s1	O=C(N1)CC(C[C@H](C2=C[C@@H](C)C[C@H](C)C2=O)O)CC1=O	Large ribosomal subunit inhibitor	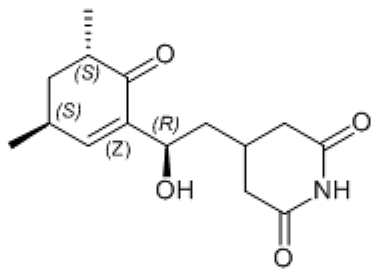	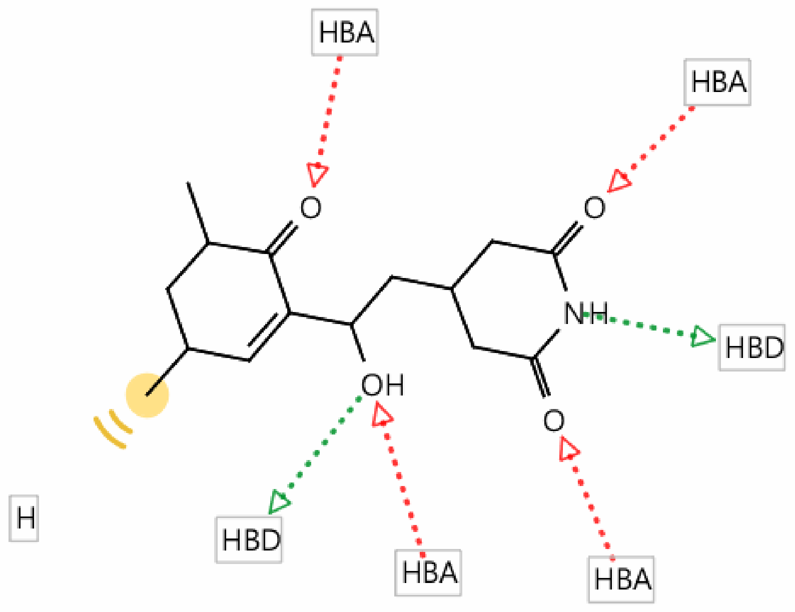
Isocycloheximide	6746-42-5	YPHMISFOHDHNIV-LUTQBAROSA-N	InChI=1S/C15H23NO4/c1-8-3-9(2)15(20)11(4-8)12(17)5-10-6-13(18)16-14(19)7-10/h8-12,17H,3-7H2,1-2H3,(H,16,18,19)/t8-,9+,11+,12+/m0/s1	C[C@H](C[C@]1([H])[C@H](O)CC(C2)CC(NC2=O)=O)C[C@H](C1=O)C	Large ribosomal subunit inhibitor	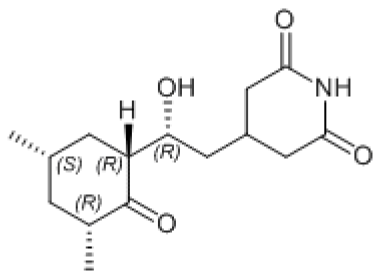	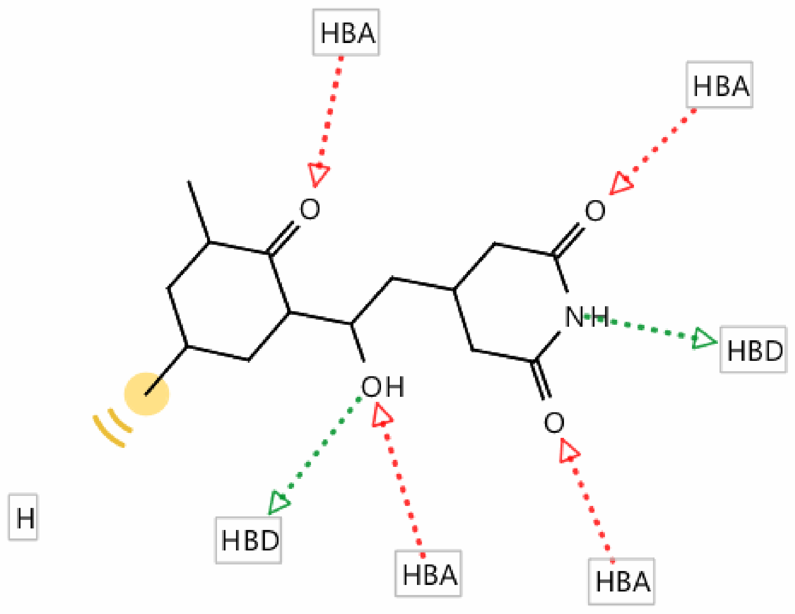

Common name	CAS Reg. No.	InChIKey	InChI	SMILES	Primary target	2D Structure	Pharmacophore[1]
Isomigrastatin	415952-70-4	TYTDEHCAAKKYOG-FEJRZXFSSA-N	InChI=1S/C27H39NO7/c1-17(21(29)11-9-10-20-15-23(30)28-24(31)16-20)14-18(2)27-19(3)26(33)22(34-4)12-7-5-6-8-13-25(32)35-27/h7-8,12-14,17,19-20,22,26-27,33H,5-6,9-11,15-16H2,1-4H3,(H,28,30,31)/b12-7+,13-8+,18-14+/t17-,19-,22-,26-,27-/m0/s1	C[C@H]1[C@H](O)[C@@H](OC)/C=C/CC/C=C/C(O[C@H]1/C(C)=C/[C@@H](C(CCCC2CC(NC(C2)=O)=O)=O)C)=O	Large ribosomal subunit inhibitor	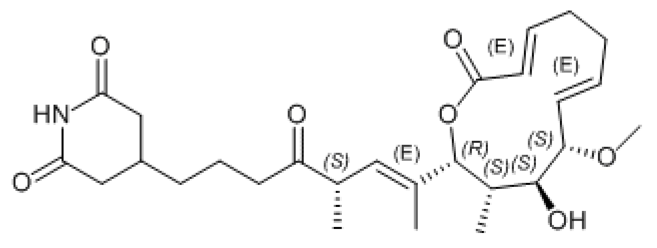	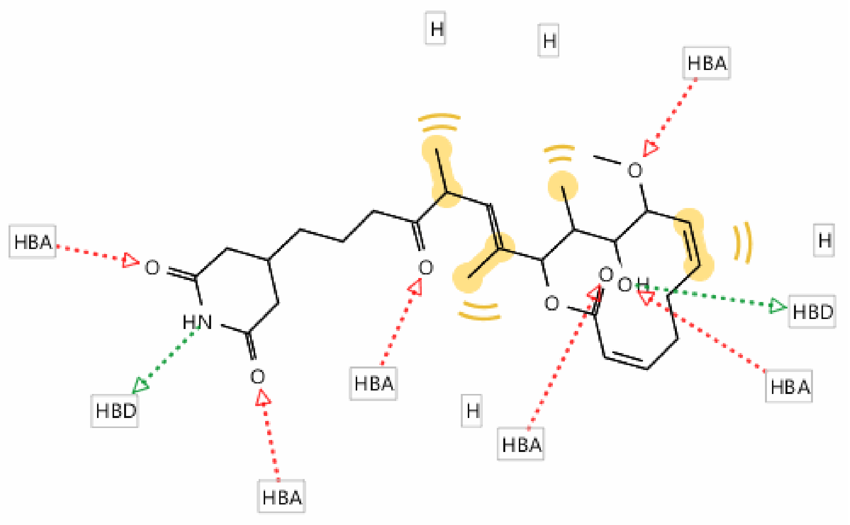
Lactimidomycin	134869-15-1	OYOKHBHOTQDIPM-BRHOHSSQSA-N	InChI=1S/C26H35NO6/c1-17-10-8-6-4-5-7-9-11-25(32)33-26(17)19(3)12-18(2)22(29)16-21(28)13-20-14-23(30)27-24(31)15-20/h4,6,8-12,17-18,20-21,26,28H,5,7,13-16H2,1-3H3,(H,27,30,31)/b6-4+,10-8-,11-9+,19-12+/t17-,18-,21+,26+/m0/s1	O=C1/C=C/CC/C=C/C=C\[C@H](C)[C@H](/C(C)=C/[C@H](C)C(C[C@H](O)CC2CC(NC(C2)=O)=O)=O)O1	Large ribosomal subunit inhibitor	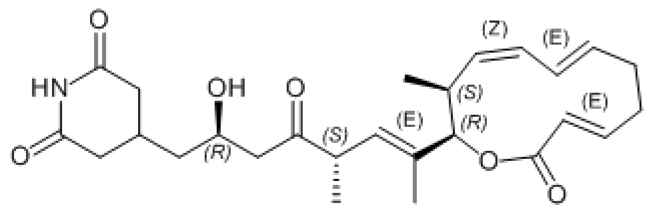	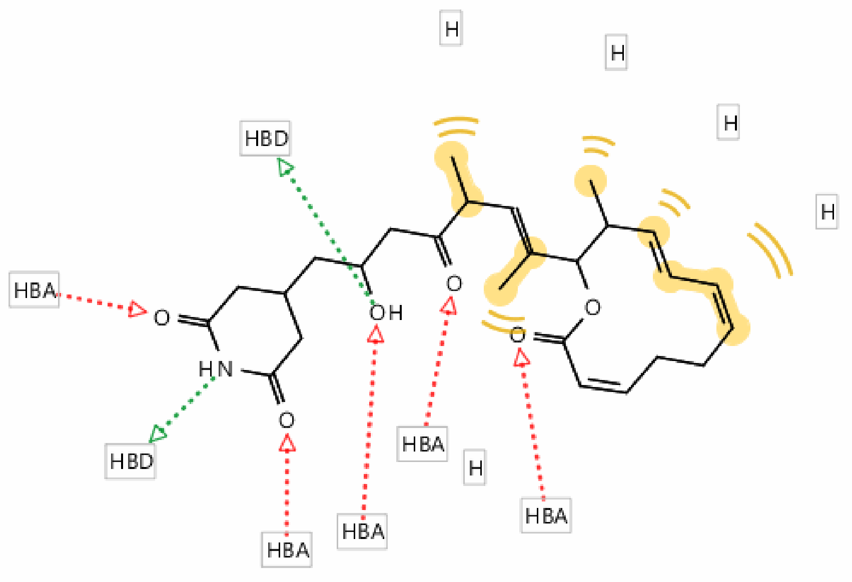
Naramycin B	642-81-9	YPHMISFOHDHNIV-GAIPPQHRSA-N	InChI=1S/C15H23NO4/c1-8-3-9(2)15(20)11(4-8)12(17)5-10-6-13(18)16-14(19)7-10/h8-12,17H,3-7H2,1-2H3,(H,16,18,19)/t8-,9-,11+,12+/m0/s1	C[C@@H](C[C@H](C)C1=O)C[C@@]1([C@@H](CC(CC(N2)=O)CC2=O)O)[H]	Large ribosomal subunit inhibitor	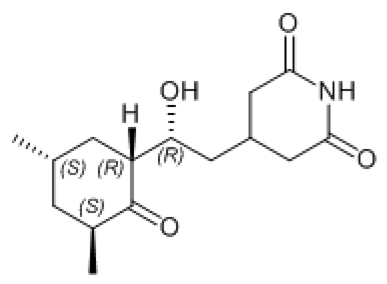	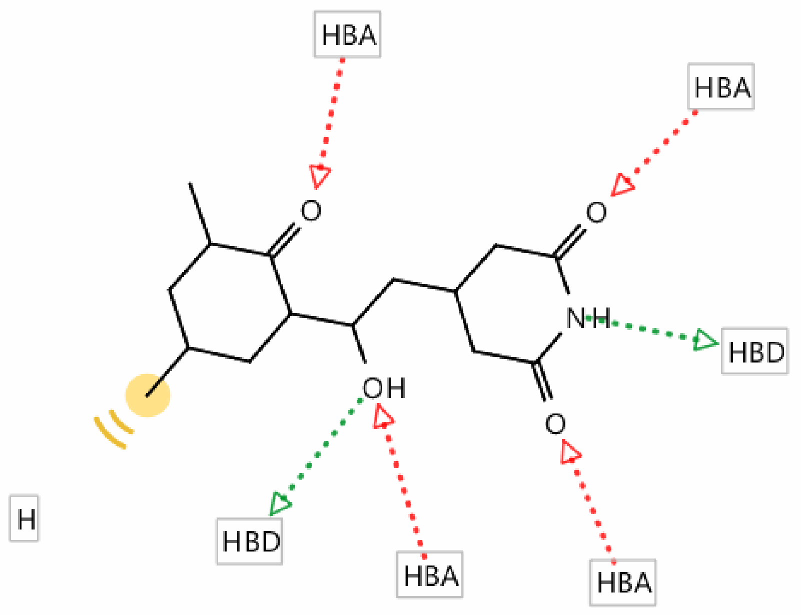
Neoisocycloheximide	94799-82-3	YPHMISFOHDHNIV-SPFNVWMYSA-N	InChI=1S/C15H23NO4/c1-8-3-9(2)15(20)11(4-8)12(17)5-10-6-13(18)16-14(19)7-10/h8-12,17H,3-7H2,1-2H3,(H,16,18,19)/t8-,9+,11+,12-/m0/s1	C[C@H](C[C@]1([H])[C@@H](O)CC(C2)CC(NC2=O)=O)C[C@H](C1=O)C	Large ribosomal subunit inhibitor	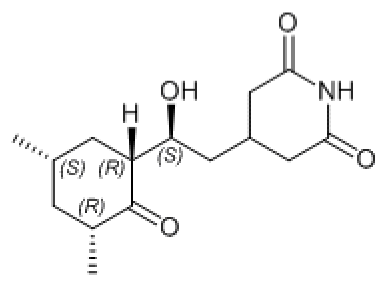	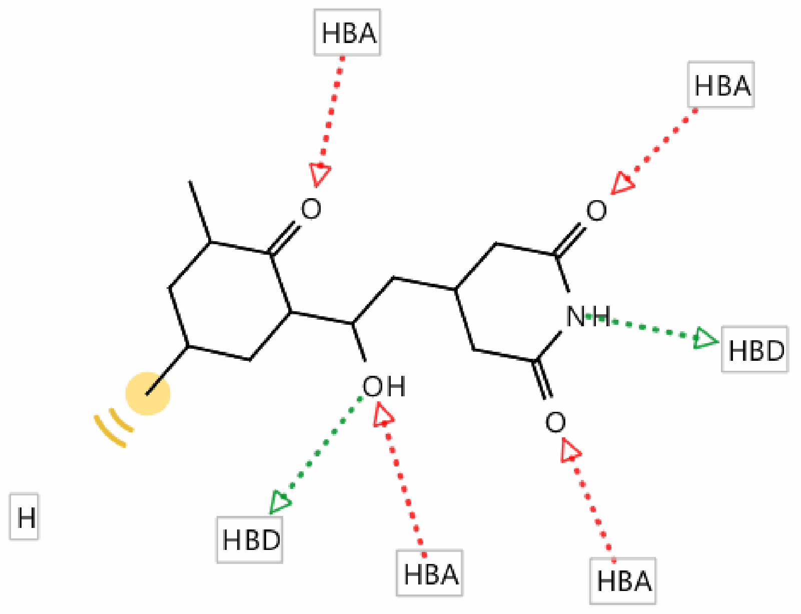
S 632A$_3$		ATUBIBZJAGAIBW-WCPFJIQSSA-N	InChI=1S/C17H25NO4/c1-4-5-11(2)6-12(3)15(20)10-14(19)7-13-8-16(21)18-17(22)9-13/h4-6,12-14,19H,7-10H2,1-3H3,(H,18,21,22)/b5-4+,11-6+/t12-,14+/m0/s1	O=C(N1)CC(C[C@@H](O)CC([C@@H](C)/C=C(C)/C=C/C)=O)CC1=O	Large ribosomal subunit inhibitor	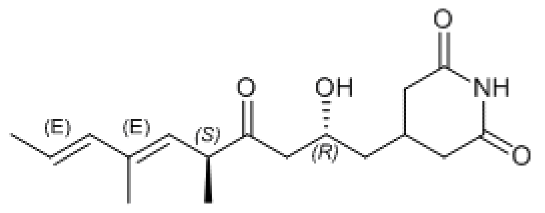	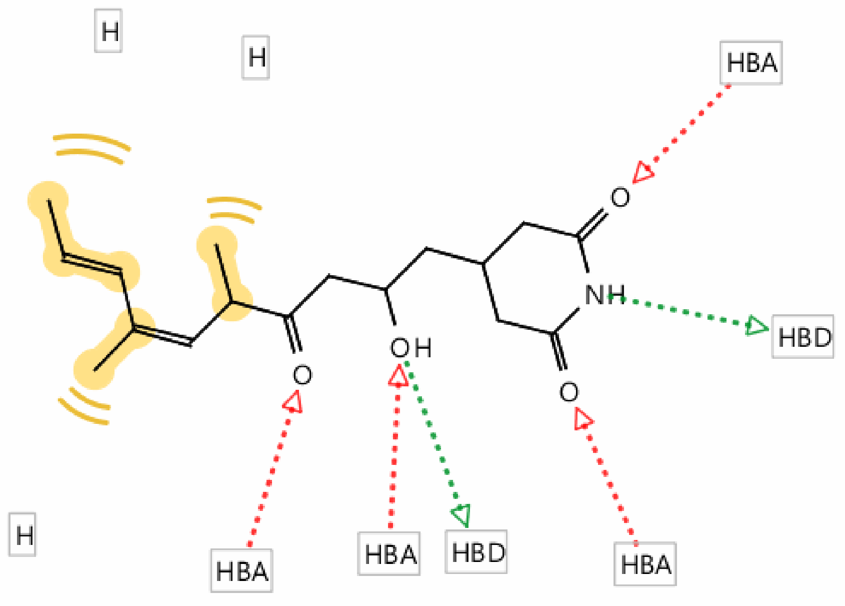
Streptimidone (S 632A$_1$)	738-72-7	ZRYKVDWGQVQRPG-ICABLKIKSA-N	InChI=1S/C16H23NO4/c1-4-10(2)5-11(3)14(19)9-13(18)6-12-7-15(20)17-16(21)8-12/h4-5,11-13,18H,1,6-9H2,2-3H3,(H,17,20,21)/b10-5-/t11-,13+/m0/s1	C[C@H](C(C[C@@H](CC1CC(NC(C1)=O)=O)O)=O)/C=C(C=C)/C	Large ribosomal subunit inhibitor	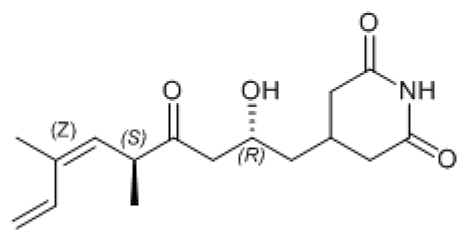	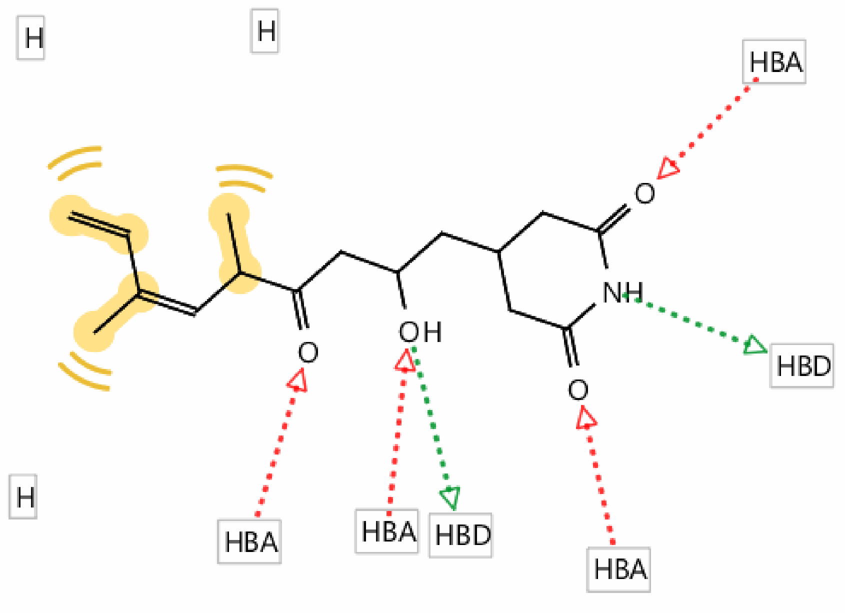

Common name	CAS Reg. No.	InChIKey	InChI	SMILES	Primary target	2D Structure	Pharmacophore[1]
Streptovitacin A	523-86-4	NFDQYBUVHVRNNY-VATFKXGISA-N	InChI=1S/C15H23NO5/c1-8-6-15(2,21)7-10(14(8)20)11(17)3-9-4-12(18)16-13(19)5-9/h8-11,17,21H,3-7H2,1-2H3,(H,16,18,19)/t8-,10-,11+,15+/m0/s1	C[C@@H](C[C@](O)(C)C[C@]1([C@@H](CC(CC(N2)=O)CC2=O)O)[H])C1=O	Large ribosomal subunit inhibitor	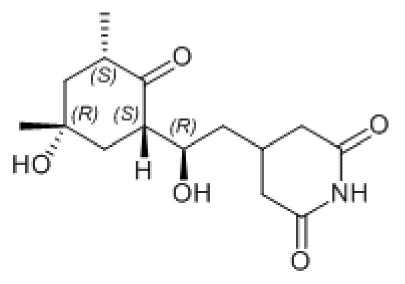	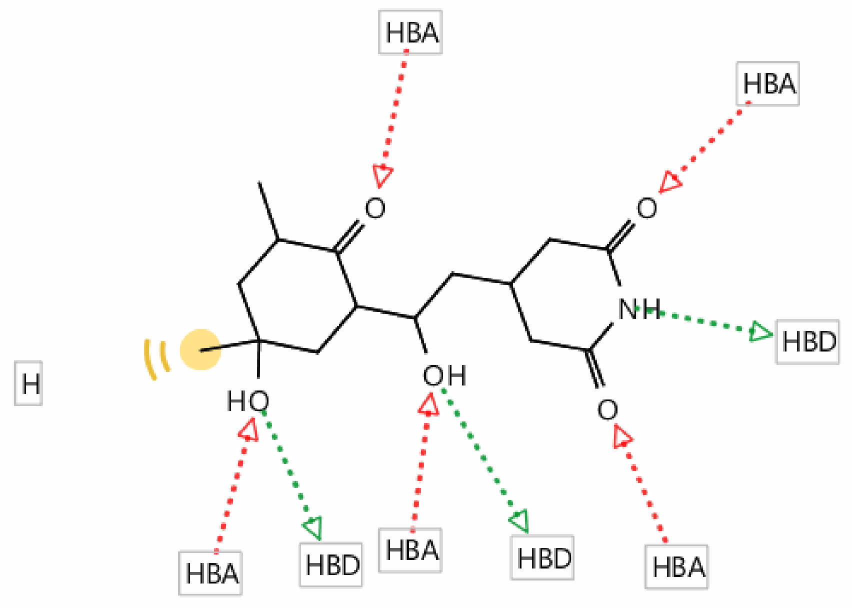
Streptovitacin B	634-50-4	JZQFWTNEUBVJCY-IBVXQHDCSA-N	InChI=1S/C15H23NO5/c1-7-3-10(15(21)8(2)14(7)20)11(17)4-9-5-12(18)16-13(19)6-9/h7-11,14,17,20H,3-6H2,1-2H3,(H,16,18,19)/t7-,8-,10+,11-,14+/m1/s1	C[C@H](C[C@]1([C@@H](CC(CC(N2)=O)CC2=O)O)[H])[C@@H]([C@@H](C)C1=O)O	Large ribosomal subunit inhibitor	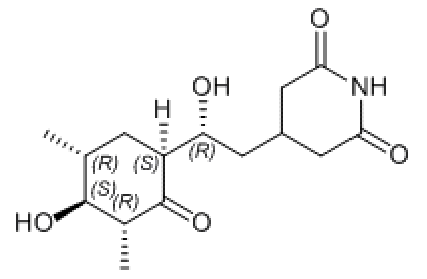	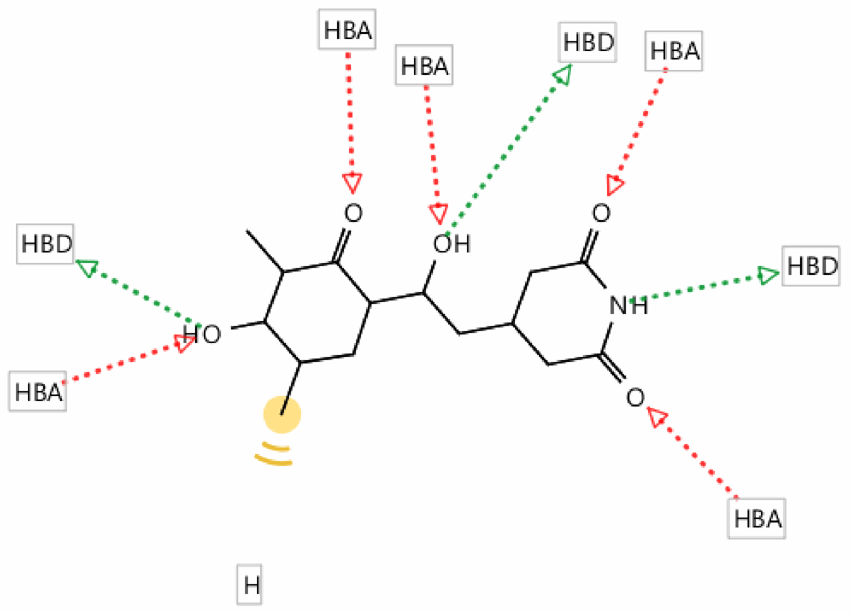
Streptovitacin C_2	521-26-6	SUOBVRUNPIKYHC-ORXWAGORSA-N	InChI=1S/C15H23NO5/c1-8-3-10(14(20)15(2,21)7-8)11(17)4-9-5-12(18)16-13(19)6-9/h8-11,17,21H,3-7H2,1-2H3,(H,16,18,19)/t8-,10-,11-,15-/m1/s1	C[C@@H](C[C@]1(O)C)C[C@]([C@H](O)CC(C2)CC(NC2=O)=O)(C1=O)[H]	Large ribosomal subunit inhibitor	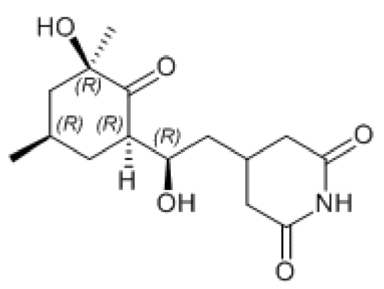	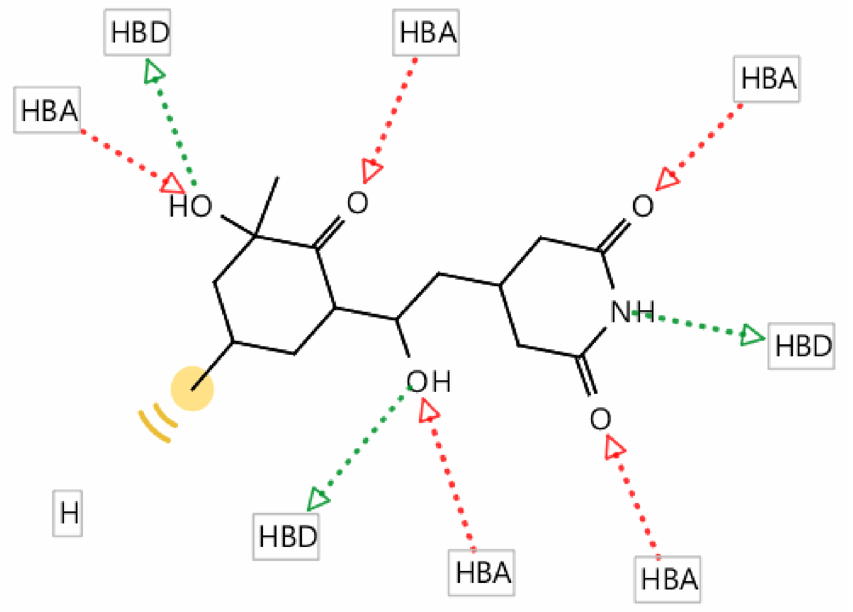
Amicetin (allomycin)	17650-86-1	HDNVYHWHCVTDIV-ZENIWSRCSA-N	InChI=1S/C29H42N6O9/c1-15-19(44-26-24(38)23(37)22(34(4)5)16(2)43-26)10-11-21(42-15)35-13-12-20(33-28(35)41)32-25(39)17-6-8-18(9-7-17)31-27(40)29(3,30)14-36/h6-9,12-13,15-16,19,21-24,26,36-38H,10-11,14,30H2,1-5H3,(H,31,40)(H,32,33,39,41)/t15-,16-,19+,21-,22-,23+,24-,26-,29+/m1/s1	C[C@H]1O[C@@]([H])(N2C=CC(NC(C3=CC=C(C=C3)NC([C@](N)(C)CO)=O)=O)=NC2=O)CC[C@]1([H])O[C@@]4([C@@H]([C@H]([C@@]([H])([C@@H](C)O4)N(C)C)O)O)[H]	Large ribosomal subunit inhibitor	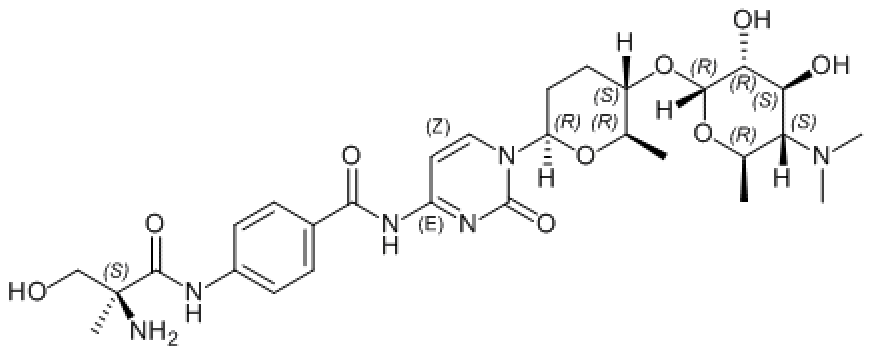	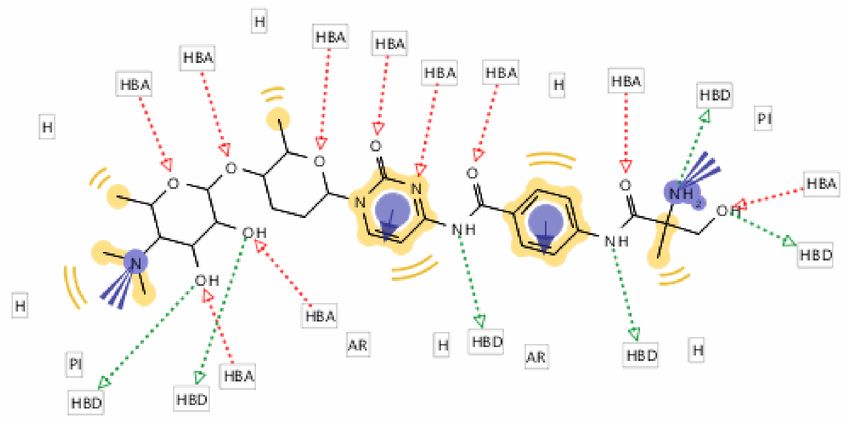
Antelmycin (anthelmycin)	12706-94-4	VQQSDVBOXQHCHU-SKPOXZENSA-N	InChI=1S/C21H37N5O14/c22-7-1-2-26(21(37)25-7)19-16(36)12(32)9(24)17(39-19)18(15(35)14(34)10(30)5(29)3-27)40-20-13(33)8(23)11(31)6(4-28)38-20/h1-2,5-6,8-20,27-36H,3-4,23-24H2,(H2,22,25,37)/t5-,6-,8+,9+,10-,11-,12+,13-,14+,15+,16-,17+,18-,19-,20+/m1/s1	NC(C=CN1[C@@]2([H])[C@H](O)[C@@H](O)[C@H](N)[C@@]([C@@]([C@@H](O)[C@@H](O)[C@H](O)[C@H](O)CO)(O[C@@]3([H])[C@H](O)[C@@H](N)[C@H](O)[C@H](O3)CO)[H])(O2)[H])=NC1=O	Large ribosomal subunit inhibitor	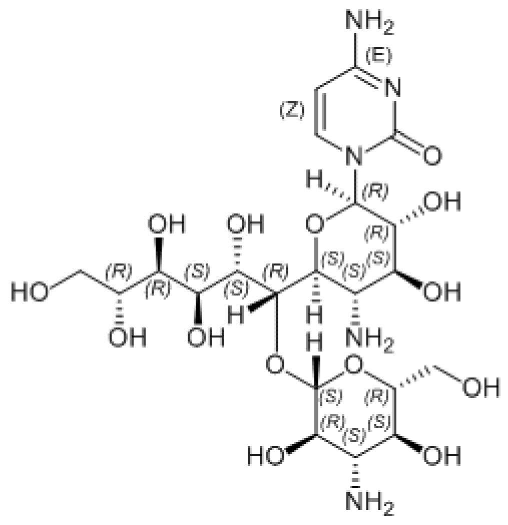	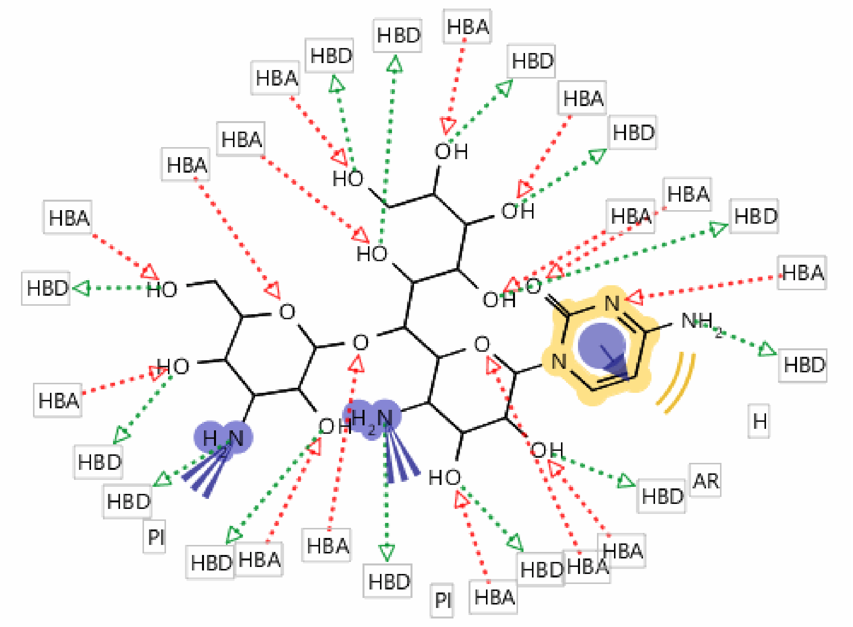
Arginomycin	106133-33-9	QHXNKYPHTJBRJV-UVHGVLLISA-N	InChI=1S/C18H28N8O5/c1-9(5-7-25(2)17(21)22)13(20)15(27)23-10-3-4-12(31-14(10)16(28)29)26-8-6-11(19)24-18(26)30/h3-4,6,8-10,12-14H,5,7,20H2,1-2H3,(H3,21,22)(H,23,27)(H,28,29)(H2,19,24,30)/t9-,10+,12-,13+,14+/m1/s1	C[C@H](CCN(C)C(N)=N)[C@@H](C(N[C@@]([H])(C=C[C@]([H])(N1C=CC(N)=NC1=O)O2)[C@]2(C(O)=O)[H])=O)N	Large ribosomal subunit inhibitor	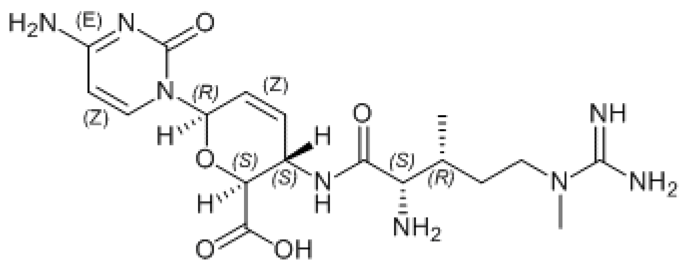	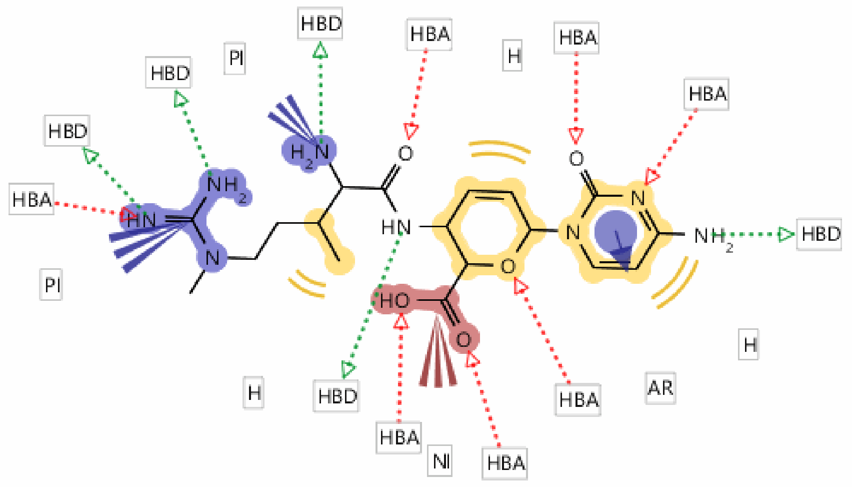

Common name	CAS Reg. No.	InChIKey	InChI	SMILES	Primary target	2D Structure	Pharmacophore[1]
Bagougeramine A	104840-35-9	UUOLIDLGSNSWHZ-QJHHURCWSA-N	InChI=1S/C17H28N10O7/c1-22-5-8(28)24-6(4-23-16(20)21)14(32)26-9-10(29)11(30)15(34-12(9)13(19)31)27-3-2-7(18)25-17(27)33/h2-3,6,9-12,15,22,29-30H,4-5H2,1H3,(H2,19,31)(H,24,28)(H,26,32)(H2,18,25,33)(H4,20,21,23)/t6-,9+,10+,11-,12+,15-/m1/s1	CNCC(N[C@]([H])(CNC(N)=N)C(N[C@]1([H])[C@@]([H])(C(N)=O)O[C@@]([H])(N2C=CC(N)=NC2=O)[C@H](O)[C@H]1O)=O)=O	Large ribosomal subunit inhibitor	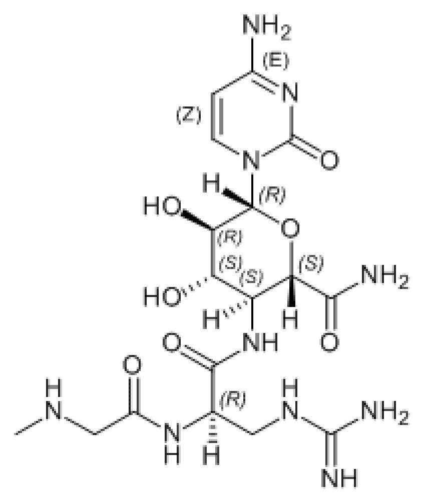	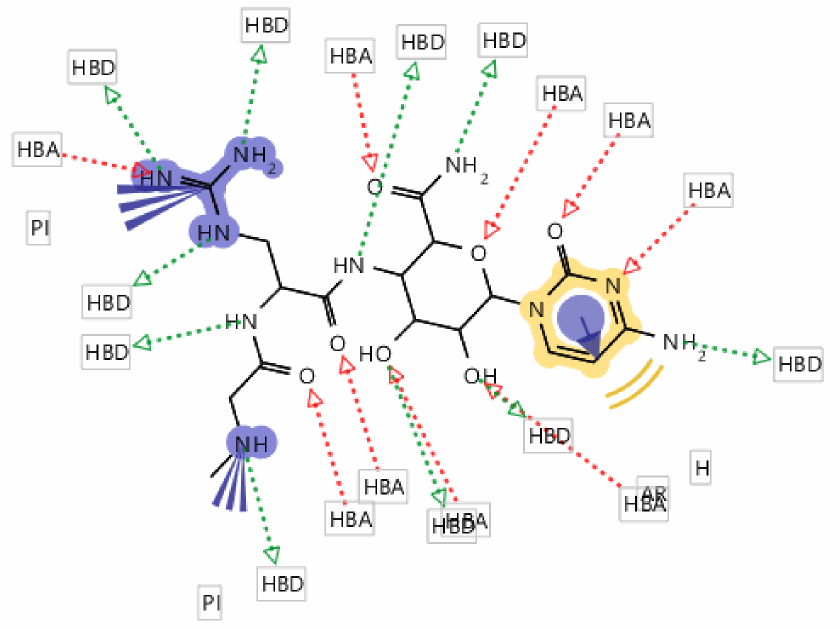
Bagougeramine B	104840-34-8	LCASODQCASDDLN-WEYBRJQASA-N	InChI=1S/C24H44N12O7/c1-29-12-15(37)33-13(11-32-23(27)28)20(40)35-16-17(38)18(39)22(36-10-5-14(26)34-24(36)42)43-19(16)21(41)31-9-4-8-30-7-3-2-6-25/h5,10,13,16-19,22,29-30,38-39H,2-4,6-9,11-12,25H2,1H3,(H,31,41)(H,33,37)(H,35,40)(H2,26,34,42)(H4,27,28,32)/t13-,16+,17+,18-,19+,22-/m1/s1	CNCC(N[C@]([H])(CNC(N)=N)C(N[C@]1([H])[C@@]([H])(C(NCCCNCCCCN)=O)O[C@@]([H])(N2C=CC(N)=NC2=O)[C@H](O)[C@H]1O)=O)=O	Large ribosomal subunit inhibitor	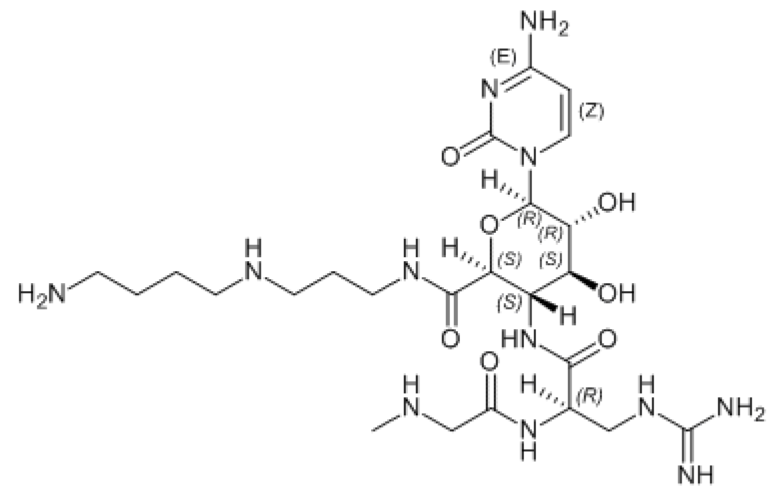	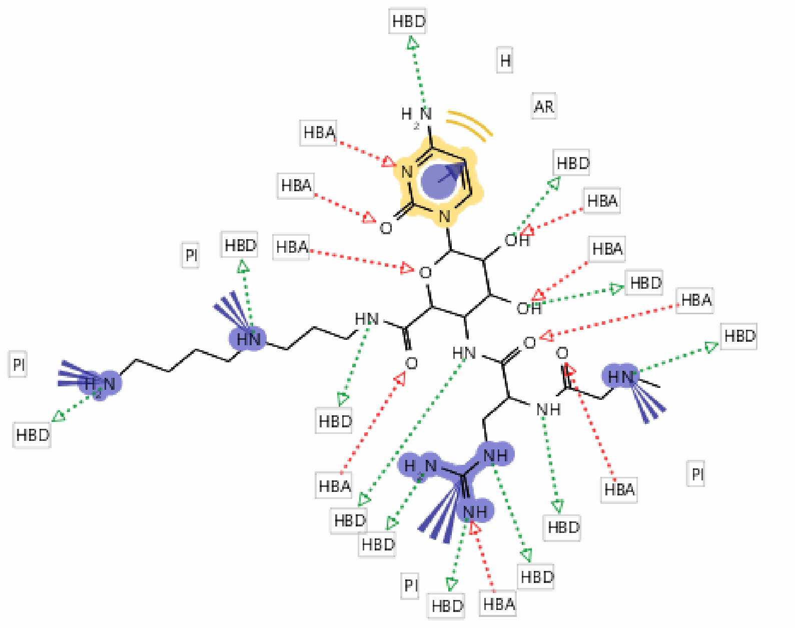
Bamicetin	43043-14-7	DLNZVFMLTYLOGI-DCPNSWTPSA-N	InChI=1S/C28H40N6O9/c1-14-18(43-25-23(37)22(36)21(30-4)15(2)42-25)9-10-20(41-14)34-12-11-19(33-27(34)40)32-24(38)16-5-7-17(8-6-16)31-26(39)28(3,29)13-35/h5-8,11-12,14-15,18,20-23,25,30,35-37H,9-10,13,29H2,1-4H3,(H,31,39)(H,32,33,38,40)/t14-,15-,18+,20-,21-,22+,23-,25-,28+/m1/s1	C[C@H]1O[C@@]([H])(N2C=CC(NC(C3=CC=C(C(C=C3)NC([C@](N)(C)CO)=O)=O)=NC2=O)CC[C@]1([H])O[C@@]4([C@@H]([C@H]([C@@H]([C@@H](C)O4)NC)O)O)[H]	Large ribosomal subunit inhibitor	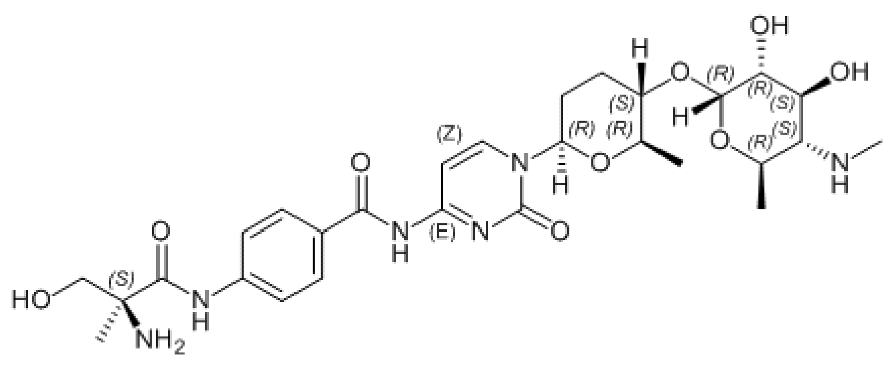	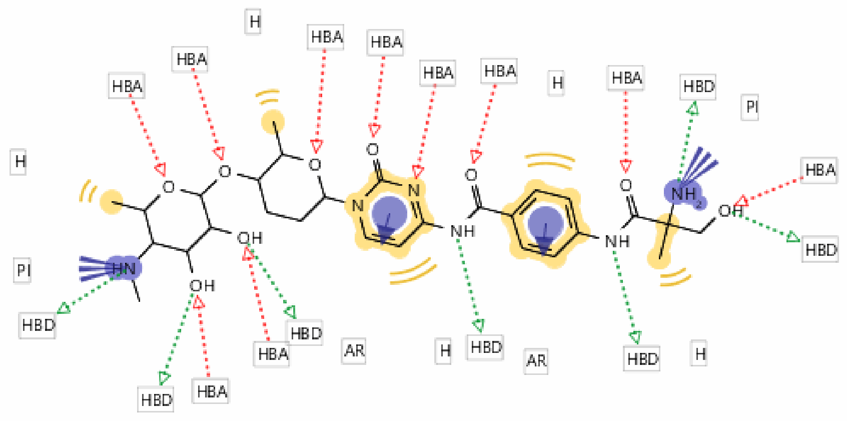
Blasticidin H	61461-71-0	DBZWUKQWLUXEEQ-IJPSMNBWSA-N	InChI=1S/C17H28N8O6/c1-24(16(20)21)4-2-8(18)6-12(27)22-9-7-10(26)14(31-13(9)15(28)29)25-5-3-11(19)23-17(25)30/h3,5,8-10,13-14,26H,2,4,6-7,18H2,1H3,(H3,20,21)(H,22,27)(H,28,29)(H2,19,23,30)/t8-,9-,10+,13-,14+/m0/s1	CN(CC[C@@H](CC(N[C@]1([H])[C@@]([H])(C(O)=O)O[C@@]([H])(N2C=CC(N)=NC2=O)[C@H](O)C1)=O)N)C(N)=N	Large ribosomal subunit inhibitor	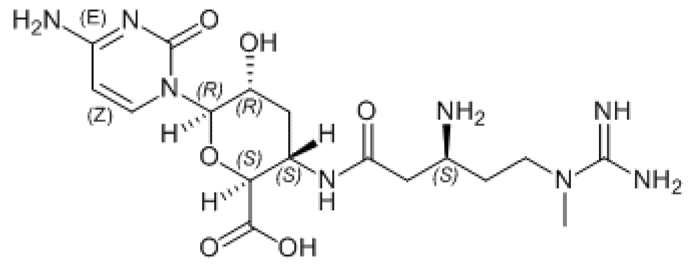	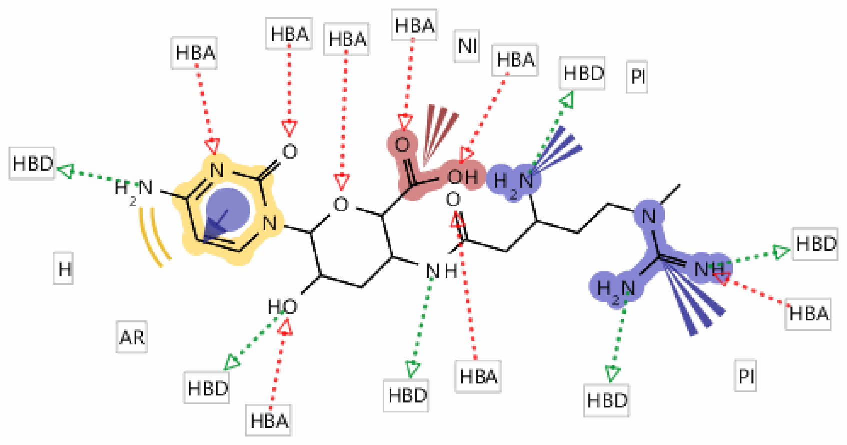
Blasticidin S	2079-00-7	CXNPLSGKWMLZPZ-ZNIXKSQXSA-N	InChI=1S/C17H26N8O5/c1-24(16(20)21)6-4-9(18)8-12(26)22-10-2-3-13(30-14(10)15(27)28)25-7-5-11(19)23-17(25)29/h2-3,5,7,9-10,13-14H,4,6,8,18H2,1H3,(H3,20,21)(H,22,26)(H,27,28)(H2,19,23,29)/t9-,10-,13+,14-/m0/s1	CN(CC[C@@H](CC(N[C@@]([H])(C=C[C@]([H])(N1C=CC(N)=NC1=O)O2)[C@]2(C(O)=O)[H])=O)N)C(N)=N	Large ribosomal subunit inhibitor	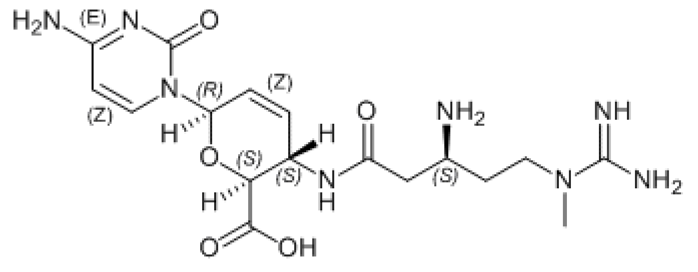	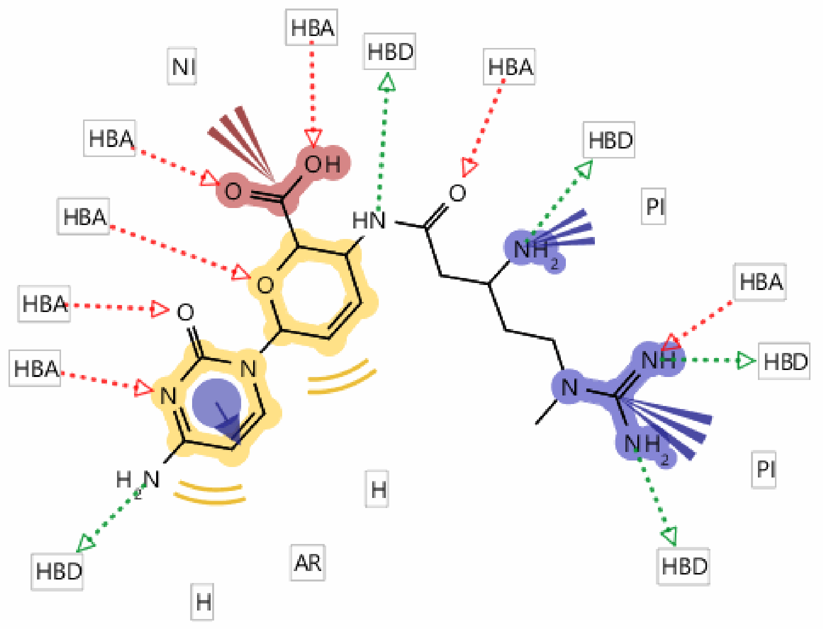
Cytimidine	51848-04-5	ZPVQPHLYBWRGBF-HNNXBMFYSA-N	InChI=1S/C15H17N5O4/c1-15(16,8-21)13(23)18-10-4-2-9(3-5-10)12(22)19-11-6-7-17-14(24)20-11/h2-7,21H,8,16H2,1H3,(H,18,23)(H2,17,19,20,22,24)/t15-/m0/s1	C[C@](C(NC1=CC=C(C(NC(C=CN2)=NC2=O)=O)C=C1)=O)(N)CO	Large ribosomal subunit inhibitor	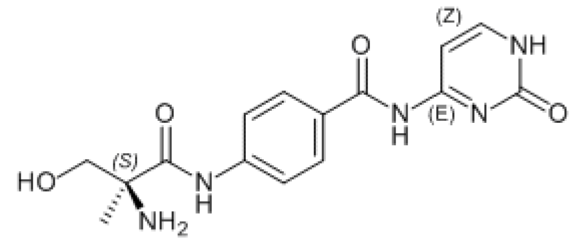	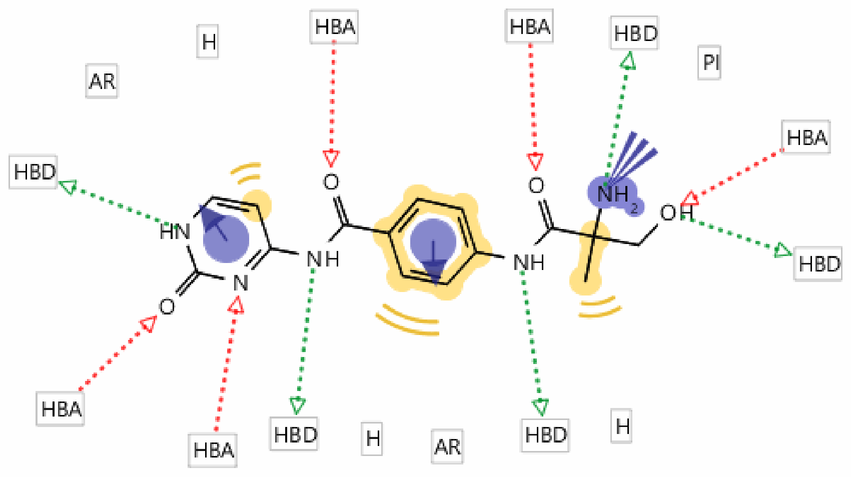

Common name	CAS Reg. No.	InChIKey	InChI	SMILES	Primary target	2D Structure	Pharmacophore[1]
Cytomycin (saitomycin)	2005-98-3	NXYZPLILSHUEBR-ZNIXKSQXSA-N	InChI=1S/C17H23N7O5/c1-23-6-4-9(20-16(23)19)8-12(25)21-10-2-3-13(29-14(10)15(26)27)24-7-5-11(18)22-17(24)28/h2-3,5,7,9-10,13-14H,4,6,8H2,1H3,(H2,19,20)(H,21,25)(H,26,27)(H2,18,22,28)/t9-,10-,13+,14-/m0/s1	CN(CC[C@@]([H])(CC(N[C@@]([H])(C=C[C@]([H])(N1C=CC(N)=NC1=O)O2)[C@]2(C(O)=O)[H])=O)N3)C3=N	Large ribosomal subunit inhibitor	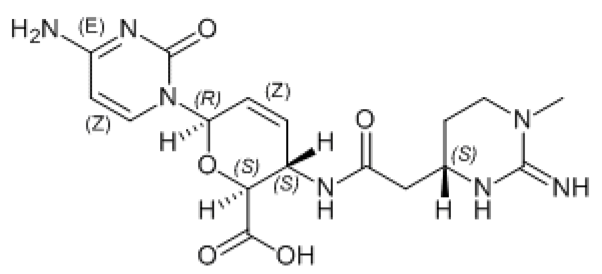	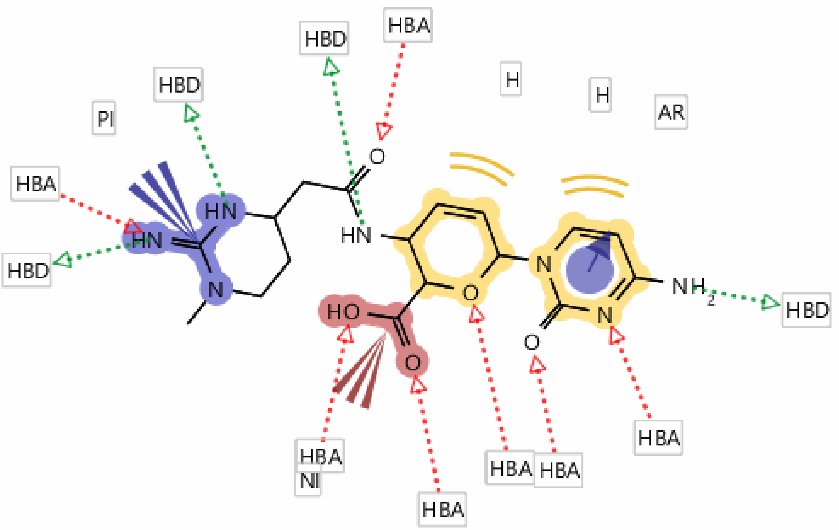
Cytosamine	26758-91-8	XWEBNXCXASNJFB-BHDDGSBGSA-N	InChI=1S/C18H30N4O6/c1-9-11(5-6-13(26-9)22-8-7-12(19)20-18(22)25)28-17-16(24)15(23)14(21(3)4)10(2)27-17/h7-11,13-17,23-24H,5-6H2,1-4H3,(H2,19,20,25)/t9-,10-,11+,13-,14-,15+,16-,17-/m1/s1	C[C@H]1O[C@@]([H])(N2C=CC(N)=NC2=O)CC[C@]1([H])O[C@@]3([C@@H]([C@H]([C@@]([H])([C@@H](C)O3)N(C)C)O)O)[H]	Large ribosomal subunit inhibitor	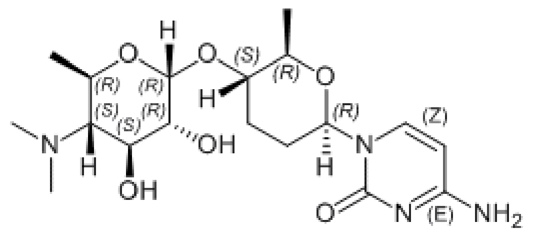	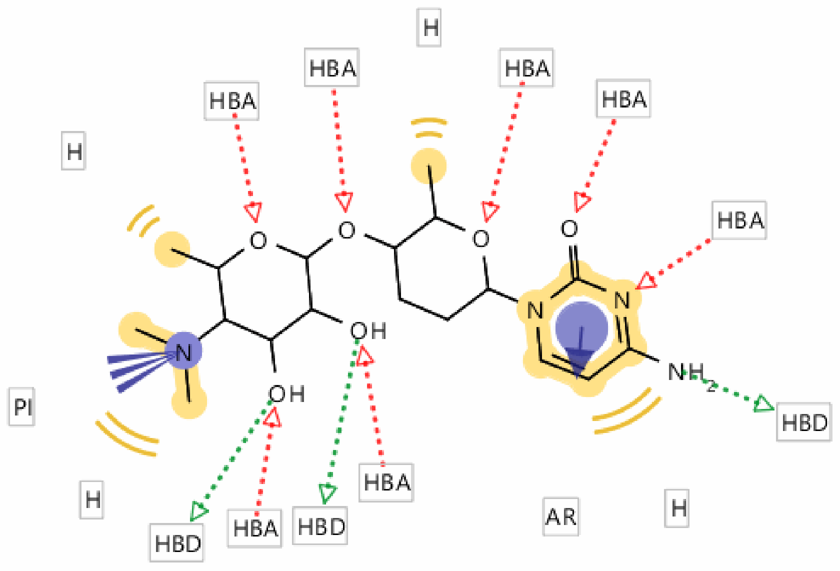
Cytosaminomycin A	157878-02-9	OEIFFRHFKRNPAL-ZQEFWBPISA-N	InChI=1S/C22H34N4O8S/c1-11-17(25(3)4)18(29)19(30)21(33-11)34-20-12(2)32-16(10-13(20)27)26-8-6-14(24-22(26)31)23-15(28)7-9-35-5/h6-9,11-13,16-21,27,29-30H,10H2,1-5H3,(H,23,24,28,31)/b9-7+/t11-,12-,13-,16-,17-,18+,19-,20-,21-/m1/s1	C[C@@H]1[C@](N(C)C)([H])[C@@H]([C@H]([C@](O[C@@]2([H])[C@@H](C)O[C@]([H])(C[C@H]2O)N3C=CC(NC(/C=C/SC)=O)=NC3=O)([H])O1)O)O	Large ribosomal subunit inhibitor	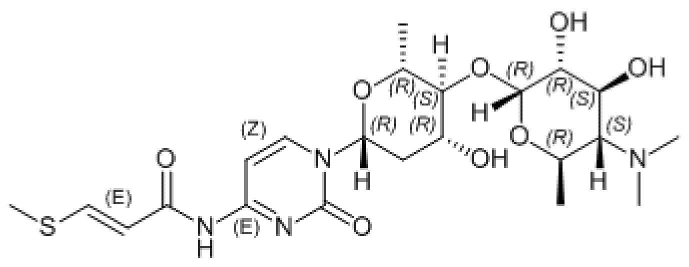	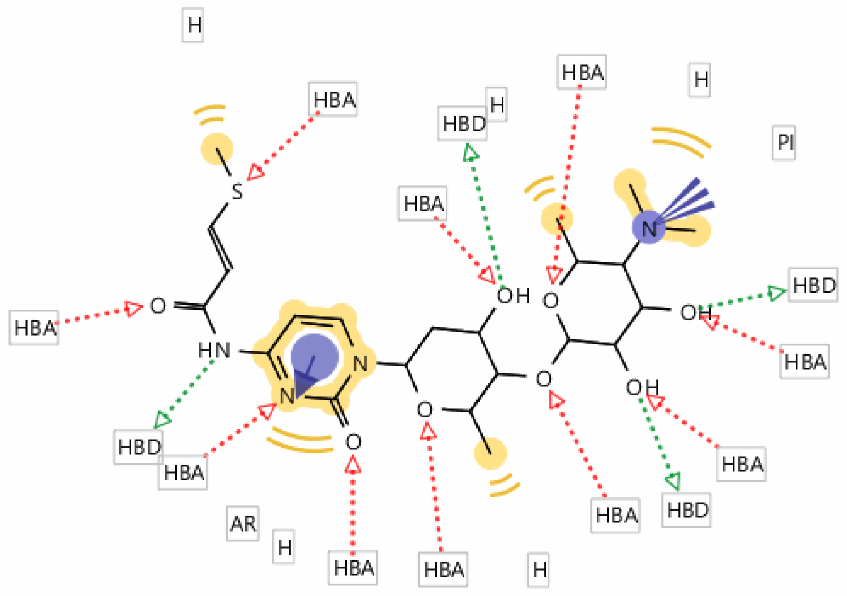
Cytosaminomycin B	157878-03-0	UFIWZSNSJFCLAC-KYFRGEGWSA-N	InChI=1S/C26H37N5O8/c1-13-20(30(4)5)21(33)22(34)25(38-13)39-23-14(2)37-19(12-17(23)32)31-11-10-18(29-26(31)36)28-24(35)15-6-8-16(27-3)9-7-15/h6-11,13-14,17,19-23,25,27,32-34H,12H2,1-5H3,(H,28,29,35,36)/t13-,14-,17-,19-,20-,21+,22-,23-,25-/m1/s1	C[C@@H]1[C@](N(C)C)([H])[C@H](O)[C@@H](O)[C@](O[C@]([C@H](O)C[C@]2([H])N3C=CC(NC(C4=CC=C(NC)C=C4)=O)=NC3=O)([H])[C@H](O2)C)([H])O1	Large ribosomal subunit inhibitor	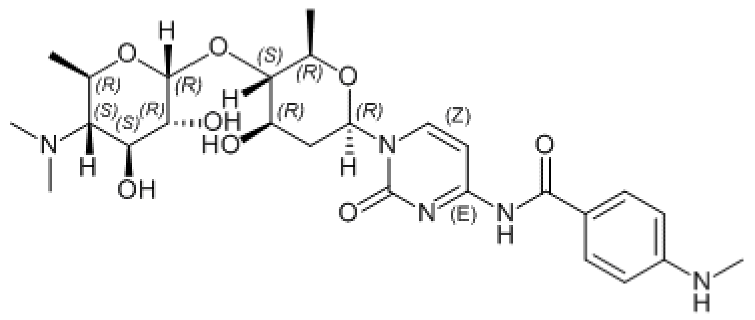	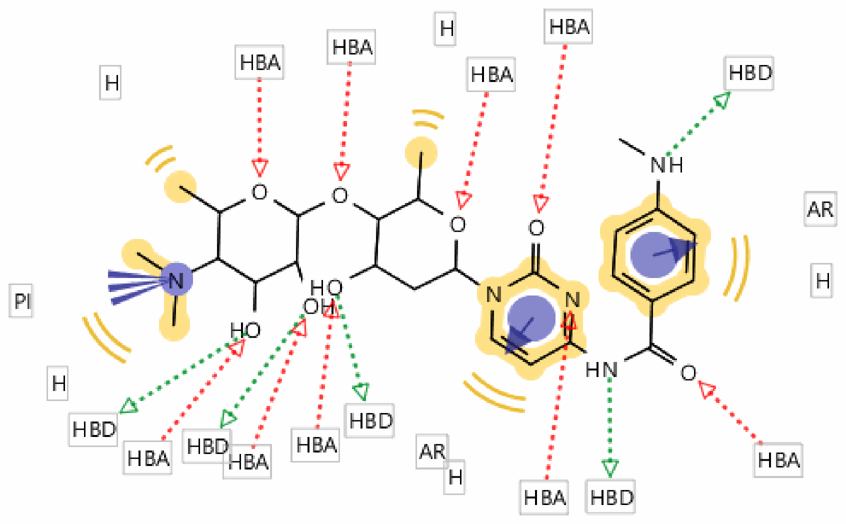
Cytosaminomycin C	157878-04-1	QIFBWQHIMSOVGC-NJGLGVPISA-N	InChI=1S/C23H36N4O8/c1-11(2)9-16(29)24-15-7-8-27(23(32)25-15)17-10-14(28)21(13(4)33-17)35-22-20(31)19(30)18(26(5)6)12(3)34-22/h7-9,12-14,17-22,28,30-31H,10H2,1-6H3,(H,24,25,29,32)/t12-,13-,14-,17-,18-,19+,20-,21-,22-/m1/s1	C/C(C)=C\C(NC(C=CN1[C@@]2([H])C[C@@H](O)[C@@]([C@H](O2)C)(O[C@]3([H])[C@H](O)[C@@H](O)[C@@]([C@H](O3)C)(N(C)C)[H])[H])=NC1=O)=O	Large ribosomal subunit inhibitor	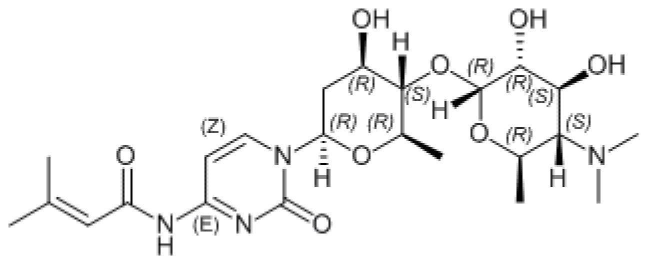	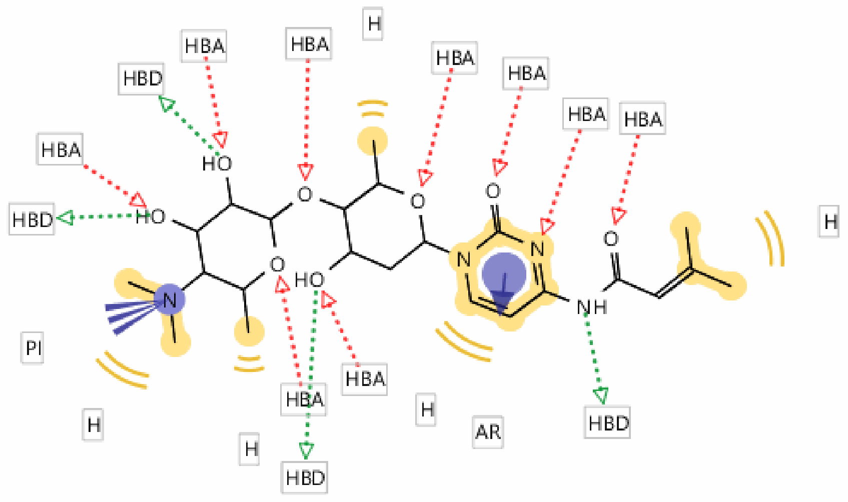
Cytosaminomycin D	157878-05-2	BIKHRIBBGRUYFE-MPFXDSPDSA-N	InChI=1S/C23H36N4O8/c1-7-11(2)21(31)24-15-8-9-27(23(32)25-15)16-10-14(28)20(13(4)33-16)35-22-19(30)18(29)17(26(5)6)12(3)34-22/h7-9,12-14,16-20,22,28-30H,10H2,1-6H3,(H,24,25,31,32)/b11-7+/t12-,13-,14-,16-,17-,18+,19-,20-,22-/m1/s1	C/C=C(C)\C(NC(C=CN1[C@]2(C[C@H]([C@@]([H])([C@@H](C)O2)O[C@@]3([C@@H]([C@H]([C@@]([H])([C@@H](C)O3)N(C)C)O)O)[H])O)[H])=NC1=O)=O	Large ribosomal subunit inhibitor	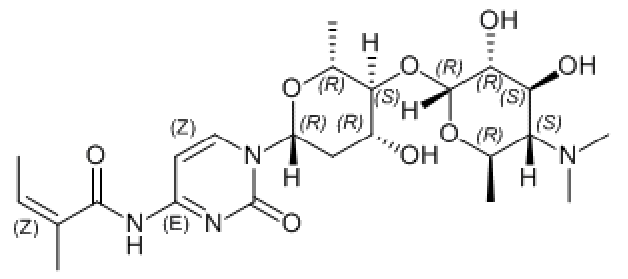	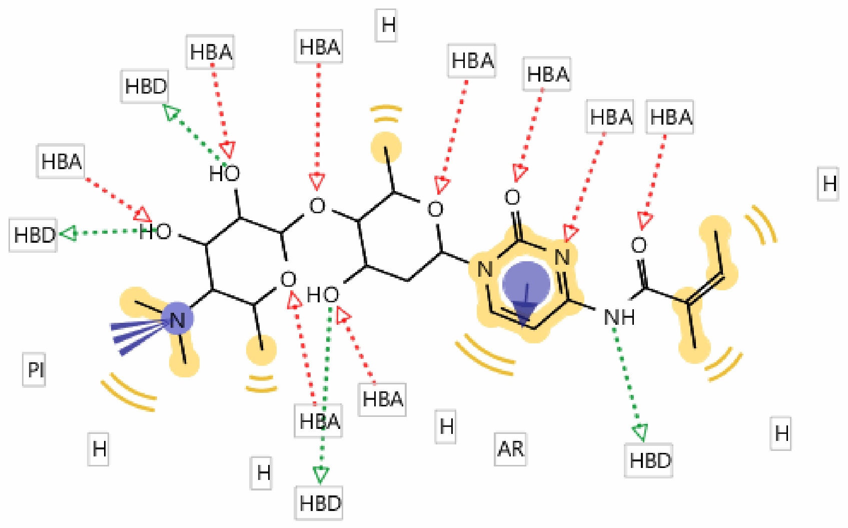

Common name	CAS Reg. No.	InChIKey	InChI	SMILES	Primary target	2D Structure	Pharmacophore[1]
Gougerotin	2096-42-6	AMNAZJFEONUVTD-QJHHURCWSA-N	InChI=1S/C16H25N7O8/c1-19-4-8(25)20-6(5-24)14(29)22-9-10(26)11(27)15(31-12(9)13(18)28)23-3-2-7(17)21-16(23)30/h2-3,6,9-12,15,19,24,26-27H,4-5H2,1H3,(H2,18,28)(H,20,25)(H,22,29)(H2,17,21,30)/t6-,9+,10+,11-,12+,15-/m1/s1	CNCC(N[C@](C(N[C@]1([H])[C@@](O[C@@]([C@H](O)[C@H]1O)([H])N2C=CC(N)=NC2=O)([H])C(N)=O)=O)([H])CO)=O	Large ribosomal subunit inhibitor	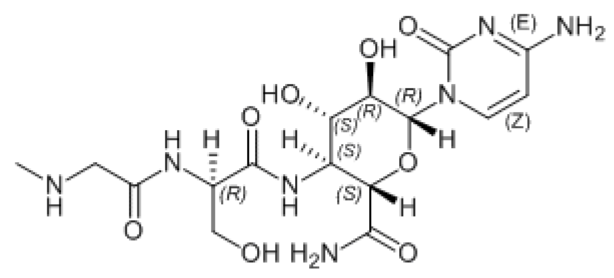	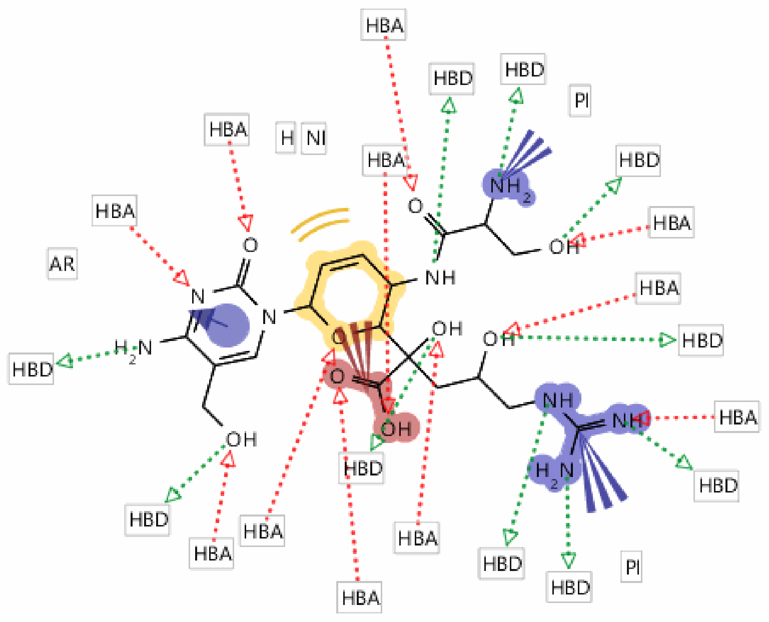
Mildiomycin	67527-71-3	QKJJCZYFXJCKRX-GCJHDDRKSA-N	InChI=1S/C19H30N8O9/c20-10(7-29)15(31)25-11-1-2-12(27-5-8(6-28)14(21)26-18(27)34)36-13(11)19(35,16(32)33)3-9(30)4-24-17(22)23/h1-2,5,9-13,28-30,35H,3-4,6-7,20H2,(H,25,31)(H,32,33)(H2,21,26,34)(H4,22,23,24)/t9-,10+,11+,12-,13+,19-/m1/s1	N[C@H](C(N[C@]1(C=C[C@]([H])(N2C=C(C(N)=NC2=O)CO)O[C@]1([H])[C@](O)(C[C@@H](O)CNC(N)=N)C(O)=O)[H])=O)CO	Large ribosomal subunit inhibitor	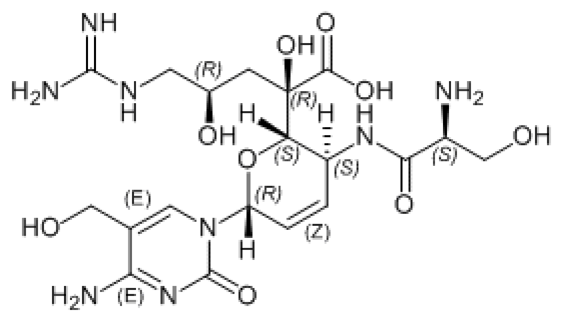	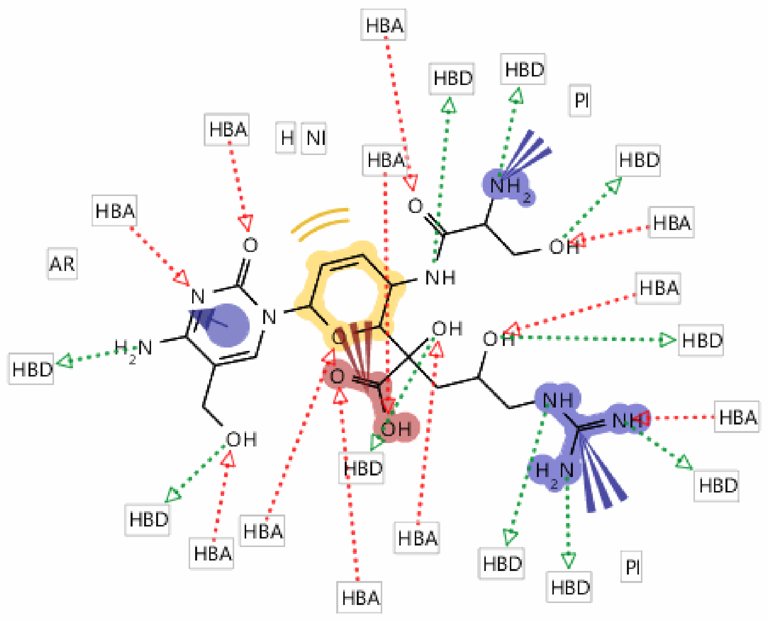
Mildiomycin B	78162-88-6	OKTYUQWLOZTONY-KKRREJBMSA-N	InChI=1S/C18H27BrN8O8/c19-8-5-27(17(33)26-13(8)21)11-2-1-10(25-14(30)9(20)6-28)12(35-11)18(34,15(31)32)3-7(29)4-24-16(22)23/h1-2,5,7,9-12,28-29,34H,3-4,6,20H2,(H,25,30)(H,31,32)(H2,21,26,33)(H4,22,23,24)/t7-,9+,10+,11-,12+,18-/m1/s1	BrC(C(N)=N1)=CN([C@]2([H])O[C@]([H])([C@](O)(C[C@H](CNC(N)=N)O)C(O)=O)[C@](NC([C@H](CO)N)=O)([H])C=C2)C1=O	Large ribosomal subunit inhibitor	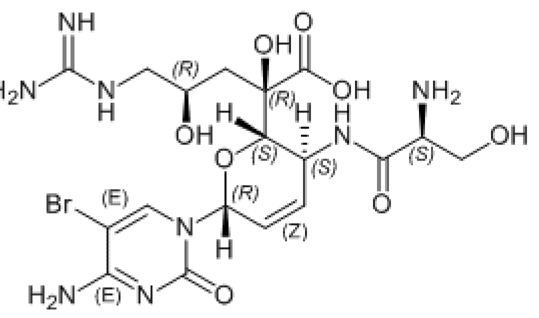	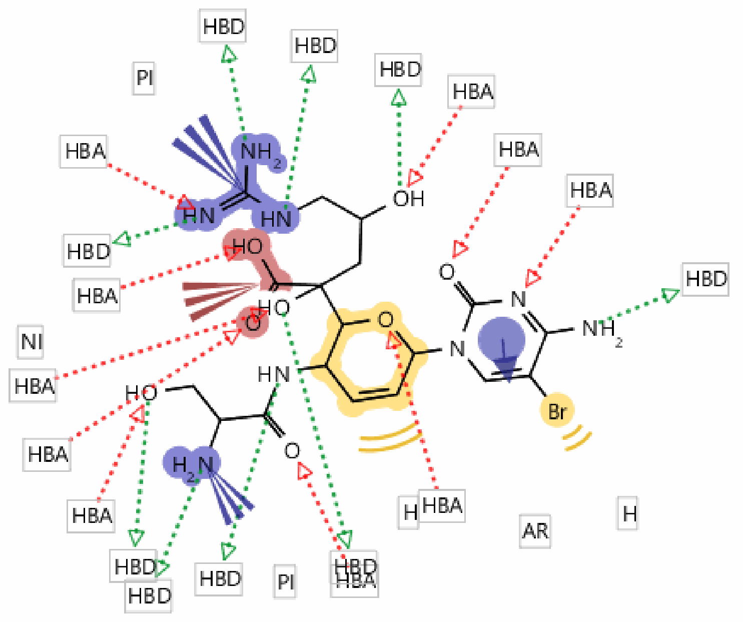
Mildiomycin C	78162-87-5	HNOHJRMHJUPCHB-VDBCCMNASA-N	InChI=1S/C18H28N8O8/c19-9(7-27)14(29)24-10-1-2-12(26-4-3-11(20)25-17(26)32)34-13(10)18(33,15(30)31)5-8(28)6-23-16(21)22/h1-4,8-10,12-13,27-28,33H,5-7,19H2,(H,24,29)(H,30,31)(H2,20,25,32)(H4,21,22,23)/t8-,9+,10+,12-,13+,18-/m1/s1	N[C@H](C(N[C@]1(C=C[C@](N2C=CC(N)=NC2=O)(O[C@]1([H])[C@](C[C@@H](O)CNC(N)=N)(C(O)=O)O)[H])[H])=O)CO	Large ribosomal subunit inhibitor	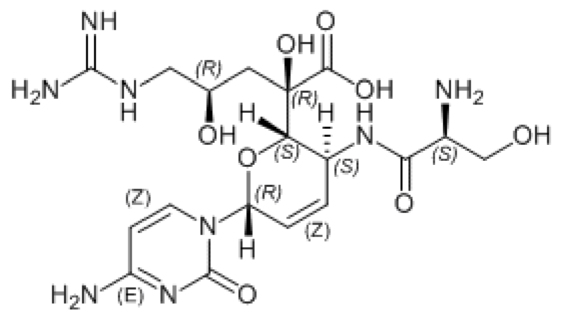	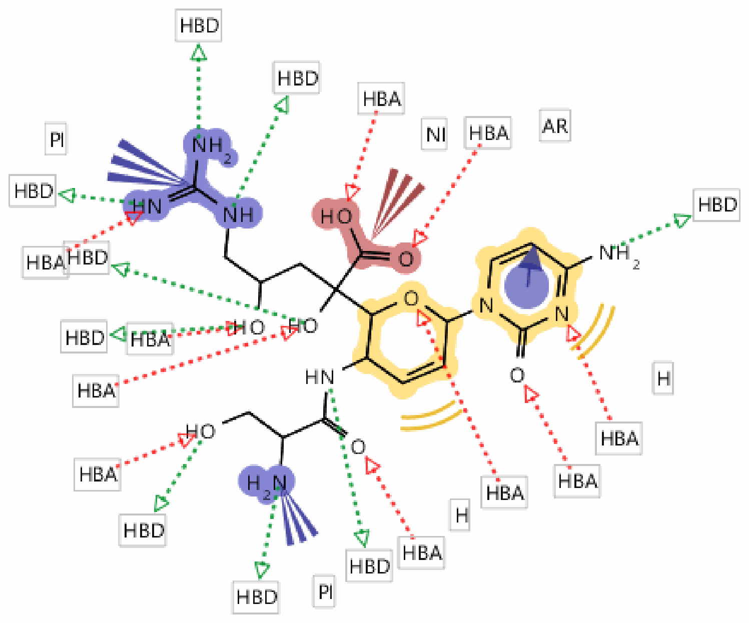
Mildiomycin D	86432-24-8	GVLLATLXQPLOAJ-LGSFDMRSSA-N	InChI=1S/C19H30N8O8/c20-10(8-29)15(30)25-11-2-3-12(27-6-9(7-28)14(21)26-18(27)33)35-13(11)19(34,16(31)32)4-1-5-24-17(22)23/h2-3,6,10-13,28-29,34H,1,4-5,7-8,20H2,(H,25,30)(H,31,32)(H2,21,26,33)(H4,22,23,24)/t10-,11-,12+,13-,19+/m0/s1	N[C@H](C(N[C@]1(C=C[C@](N2C=C(C(N)=NC2=O)CO)(O[C@]1([H])[C@](CCCNC(N)=N)(C(O)=O)O)[H])[H])=O)CO	Large ribosomal subunit inhibitor	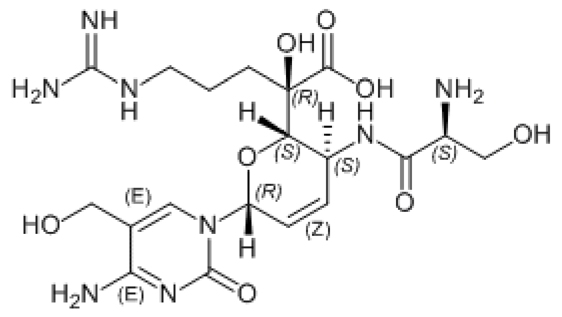	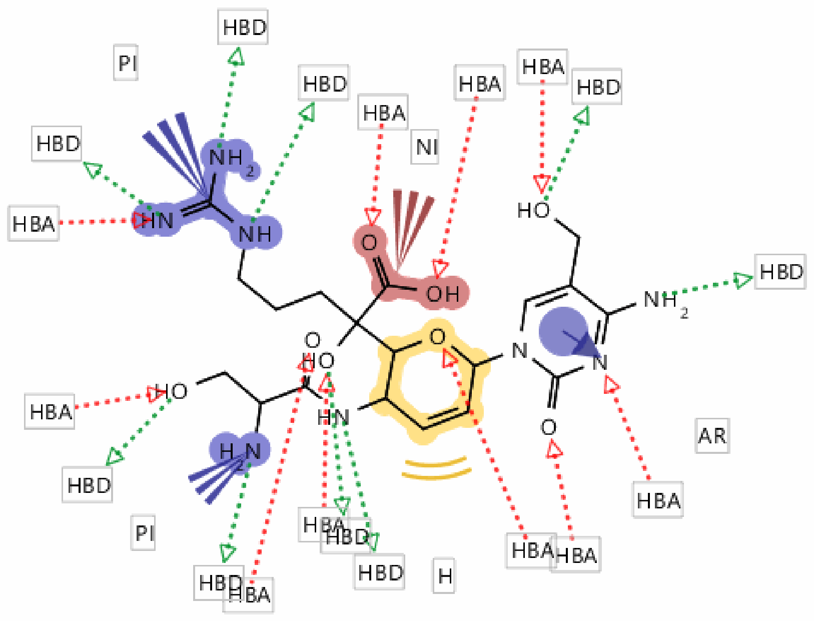
Mildiomycin M	78162-91-1	RXCXBCIOCUWGGD-GCJHDDRKSA-N	InChI=1S/C19H30N8O8/c1-8-6-27(18(33)26-14(8)21)12-3-2-11(25-15(30)10(20)7-28)13(35-12)19(34,16(31)32)4-9(29)5-24-17(22)23/h2-3,6,9-13,28-29,34H,4-5,7,20H2,1H3,(H,25,30)(H,31,32)(H2,21,26,33)(H4,22,23,24)/t9-,10+,11+,12-,13+,19-/m1/s1	CC(C(N)=N1)=CN([C@]2([H])O[C@]([H])([C@](O)(C[C@H](CNC(N)=N)O)C(O)=O)[C@](NC([C@H](CO)N)=O)([H])C=C2)C1=O	Large ribosomal subunit inhibitor	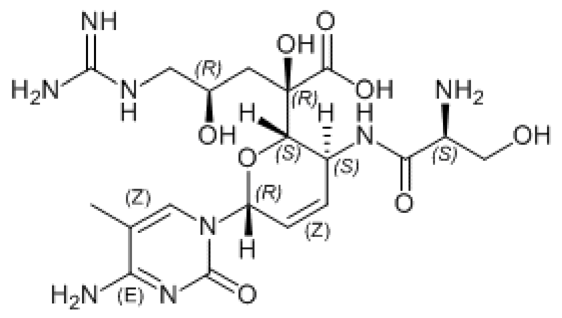	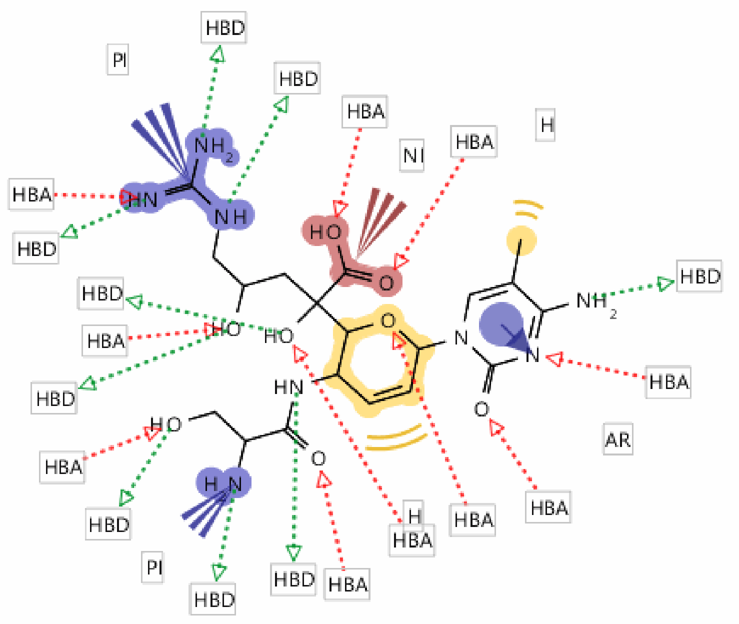

Common name	CAS Reg. No.	InChIKey	InChI	SMILES	Primary target	2D Structure	Pharmacophore[1]
Norplicacetin	64040-47-7	HJFXGPLQIPLHSS-UOUGBBRZSA-N	InChI=1S/C24H33N5O7/c1-12-16(36-23-21(31)20(30)19(26-3)13(2)35-23)8-9-18(34-12)29-11-10-17(28-24(29)33)27-22(32)14-4-6-15(25)7-5-14/h4-7,10-13,16,18-21,23,26,30-31H,8-9,25H2,1-3H3,(H,27,28,32,33)/t12-,13-,16+,18-,19-,20+,21-,23-/m1/s1	C[C@H]1O[C@@]([H])(N2C=CC(NC(C3=CC=C(C=C3)N)=O)=NC2=O)CC[C@]1([H])O[C@@]4([C@@H]([C@H]([C@@H]([C@@H](C)O4)NC)O)O)[H]	Large ribosomal subunit inhibitor	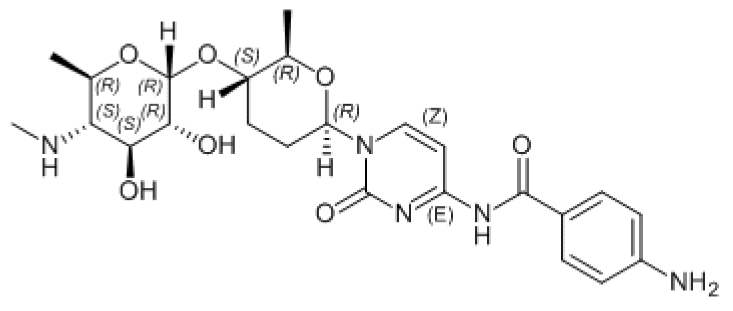	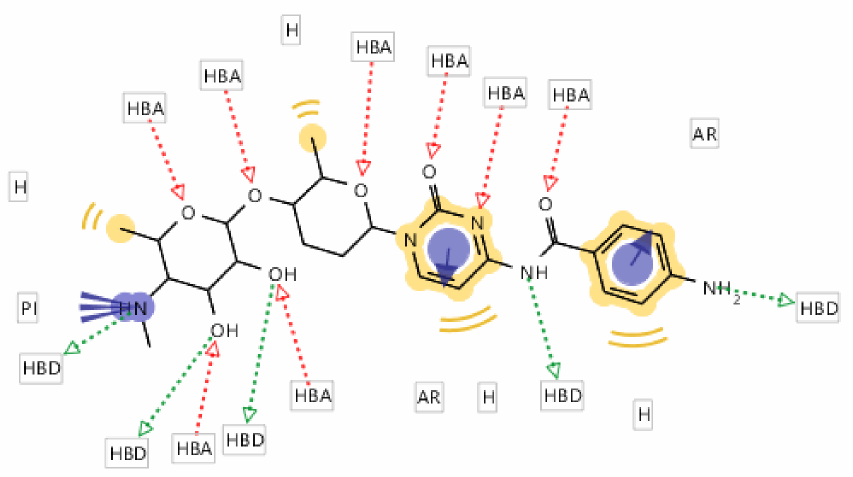
Oxamicetin	52665-75-5	UWVPICJGEAEOKF-OBFBSDRYSA-N	InChI=1S/C29H42N6O10/c1-14-21(34(4)5)22(38)23(39)26(44-14)45-24-15(2)43-20(12-18(24)37)35-11-10-19(33-28(35)42)32-25(40)16-6-8-17(9-7-16)31-27(41)29(3,30)13-36/h6-11,14-15,18,20-24,26,36-39H,12-13,30H2,1-5H3,(H,31,41)(H,32,33,40,42)/t14-,15-,18-,20-,21-,22+,23-,24-,26-,29+/m1/s1	C[C@@H]1[C@](N(C)C)([H])[C@@H]([C@H]([C@](O[C@]2([H])[C@@H](C)O[C@]([H])(C[C@H]2O)N3C=CC(NC(C4=CC=C(C=C4)NC([C@](CO)(N)C)=O)=O)=NC3=O)([H])O1)O)O	Large ribosomal subunit inhibitor	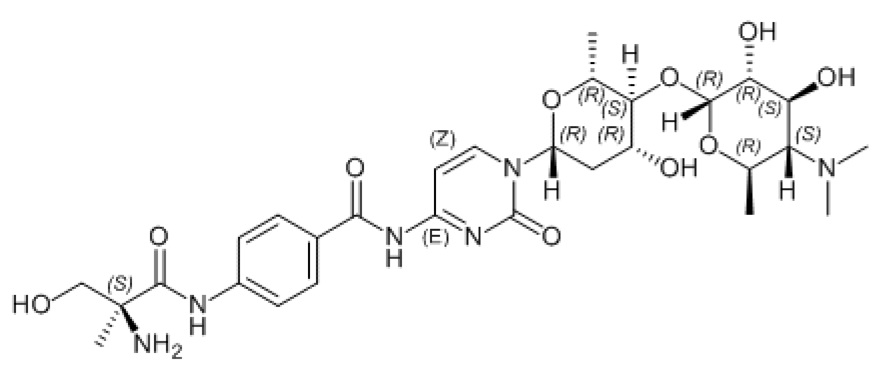	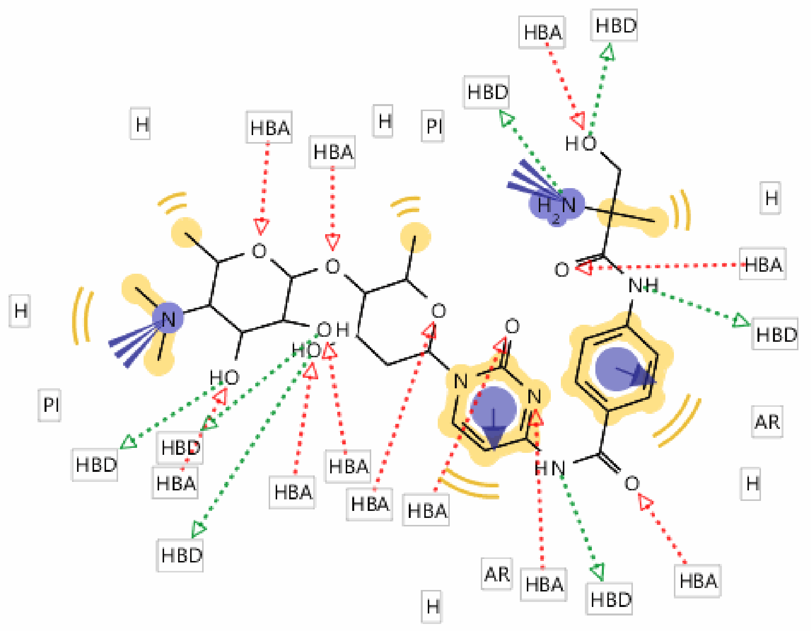
Oxyplicacetin (cytosaminomycin E)	100108-92-7	UYEYXSOGZWDEFY-LJEPMGMESA-N	InChI=1S/C25H35N5O8/c1-12-19(29(3)4)20(32)21(33)24(37-12)38-22-13(2)36-18(11-16(22)31)30-10-9-17(28-25(30)35)27-23(34)14-5-7-15(26)8-6-14/h5-10,12-13,16,18-22,24,31-33H,11,26H2,1-4H3,(H,27,28,34,35)/t12-,13-,16-,18-,19-,20+,21-,22-,24-/m1/s1	C[C@@H]1[C@](N(C)C)([H])[C@H](O)[C@@H](O)[C@](O[C@]([C@H](O)C[C@]2([H])N3C=CC(NC(C4=CC=C(N)C=C4)=O)=NC3=O)([H])[C@H](O2)C)([H])O1	Large ribosomal subunit inhibitor	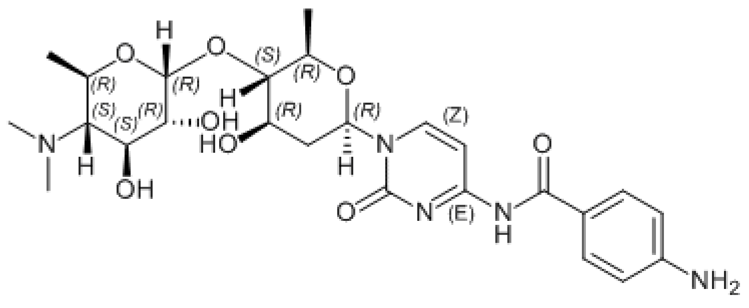	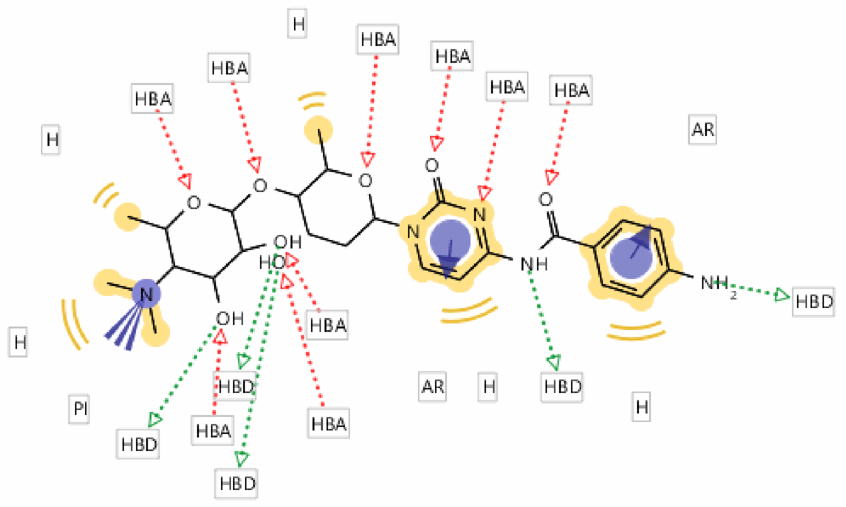
Plicacetin (amicetin B)	43043-15-8	NFOMJDXALJABQF-DDNYSEEGSA-N	InChI=1S/C25H35N5O7/c1-13-17(37-24-22(32)21(31)20(29(3)4)14(2)36-24)9-10-19(35-13)30-12-11-18(28-25(30)34)27-23(33)15-5-7-16(26)8-6-15/h5-8,11-14,17,19-22,24,31-32H,9-10,26H2,1-4H3,(H,27,28,33,34)/t13-,14-,17+,19-,20-,21+,22-,24-/m1/s1	C[C@H]1O[C@@](N2C=CC(NC(C3=CC=C(N)C=C3)=O)=NC2=O)(CC[C@@]1(O[C@]4([H])[C@H](O)[C@@H](O)[C@@]([C@H](O4)C)(N(C)C)[H])[H])[H]	Large ribosomal subunit inhibitor	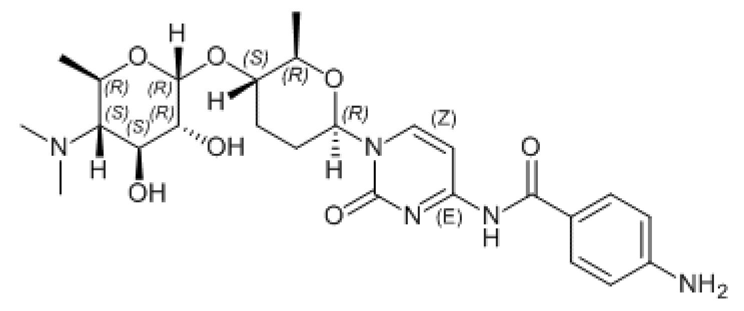	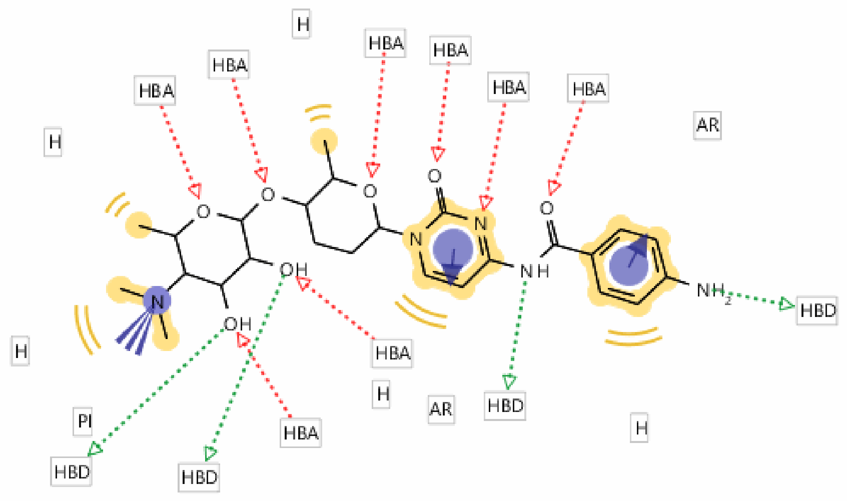
Rodaplutin	108351-49-1	MGPVTRZZQCEQDF-AKKLJKOSSA-N	InChI=1S/C24H39N9O7/c1-12(2)8-15(25)21(36)29-14(6-7-32(3)23(27)28)9-17(35)30-16-4-5-18(40-19(16)22(37)38)33-10-13(11-34)20(26)31-24(33)39/h4-5,10,12,14-16,18-19,34H,6-9,11,25H2,1-3H3,(H3,27,28)(H,29,36)(H,30,35)(H,37,38)(H2,26,31,39)/t14-,15+,16+,18-,19+/m1/s1	CC(C)C[C@@H](C(N[C@](CC(N[C@@]1([H])C=C[C@](O[C@@]1(C(O)=O)[H])([H])N2C=C(CO)C(N)=NC2=O)=O)([H])CCN(C)C(N)=N)=O)N	Large ribosomal subunit inhibitor	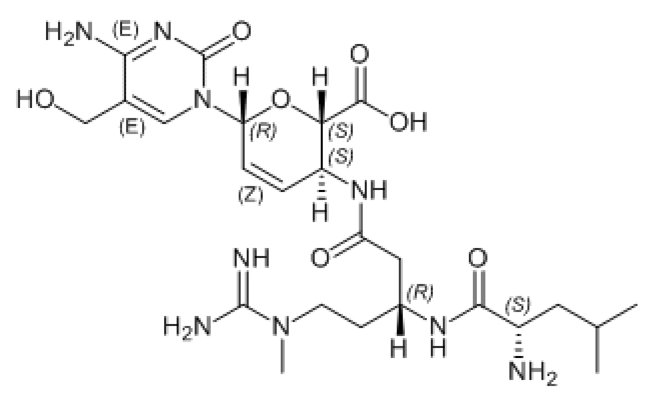	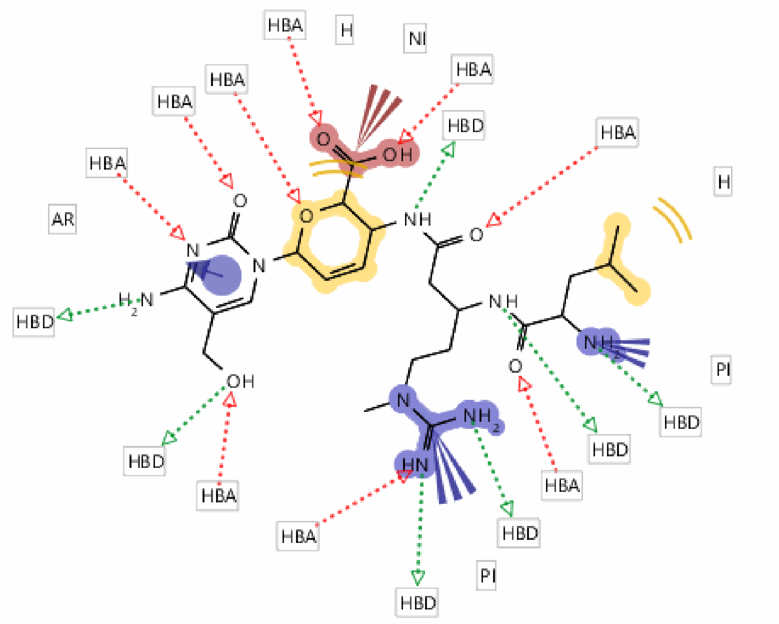
SCH 36605	113667-06-4	MGPVTRZZQCEQDF-FYAJOAKPSA-N	InChI=1S/C24H39N9O7/c1-12(2)8-15(25)21(36)29-14(6-7-32(3)23(27)28)9-17(35)30-16-4-5-18(40-19(16)22(37)38)33-10-13(11-34)20(26)31-24(33)39/h4-5,10,12,14-16,18-19,34H,6-9,11,25H2,1-3H3,(H3,27,28)(H,29,36)(H,30,35)(H,37,38)(H2,26,31,39)/t14-,15-,16-,18+,19-/m0/s1	CC(C)C[C@@H](C(N[C@@](CC(N[C@@]1([H])C=C[C@](O[C@@]1(C(O)=O)[H])([H])N2C=C(CO)C(N)=NC2=O)=O)([H])CCN(C)C(N)=N)=O)N	Large ribosomal subunit inhibitor	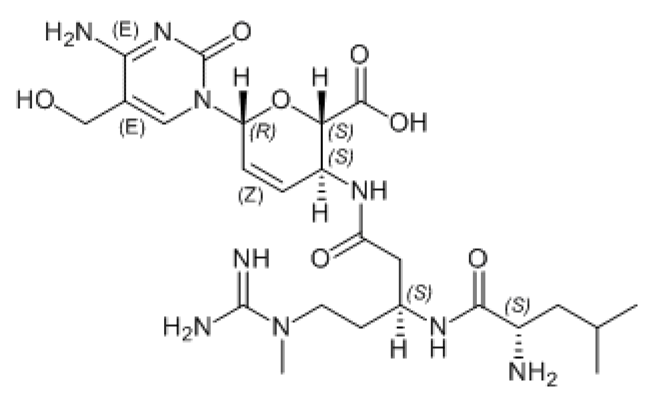	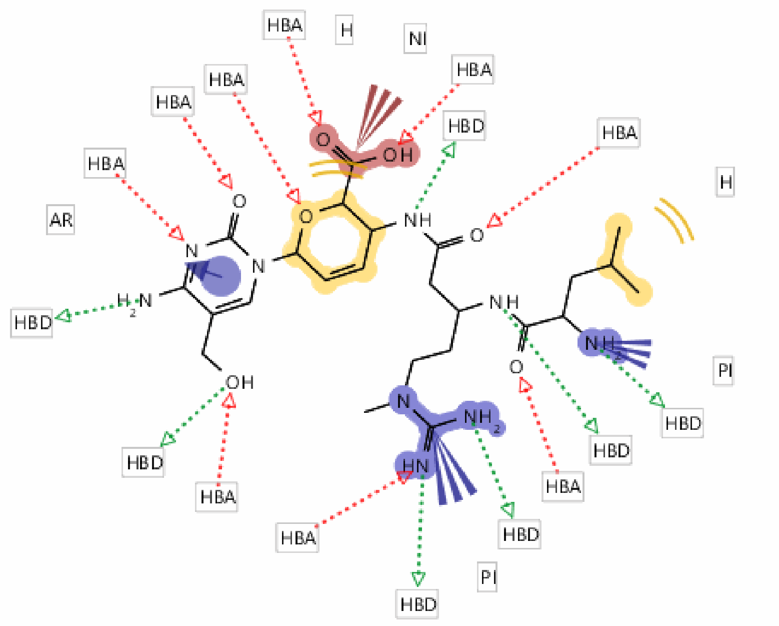

Common name	CAS Reg. No.	InChIKey	InChI	SMILES	Primary target	2D Structure	Pharmacophore[1]
SF 2457	115748-04-4	MRCNWCHLQHYMMZ-AOHSPZNCSA-N	InChI=1S/C27H38N6O9/c1-12(28)24(37)30-16-7-5-15(6-8-16)25(38)31-18-9-10-33(27(39)32-18)19-11-17(34)23(14(3)40-19)42-26-22(36)21(35)20(29-4)13(2)41-26/h5-10,12-14,17,19-23,26,29,34-36H,11,28H2,1-4H3,(H,30,37)(H,31,32,38,39)/t12-,13-,14-,17-,19-,20-,21+,22-,23-,26-/m1/s1	C[C@H](C(NC1=CC=C(C=C1)C(NC(C=CN2[C@]3(C[C@H]([C@@]([H])([C@@H](C)O3)O[C@@]4([C@@H]([C@H]([C@@H]([C@@H](C)O4)NC)O)O)[H])O)[H])=NC2=O)=O)=O)N	Large ribosomal subunit inhibitor	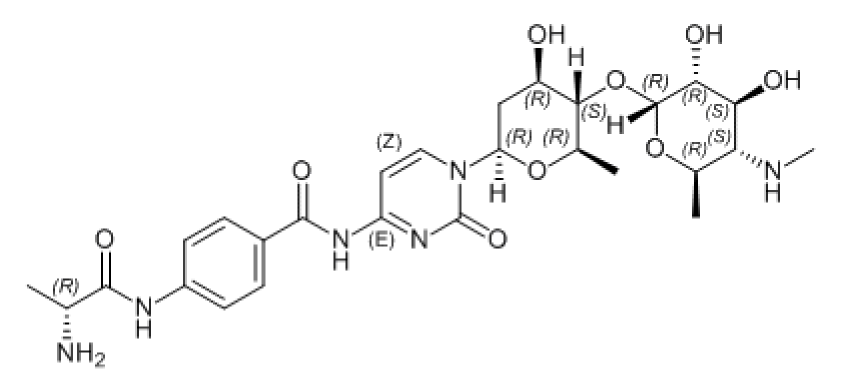	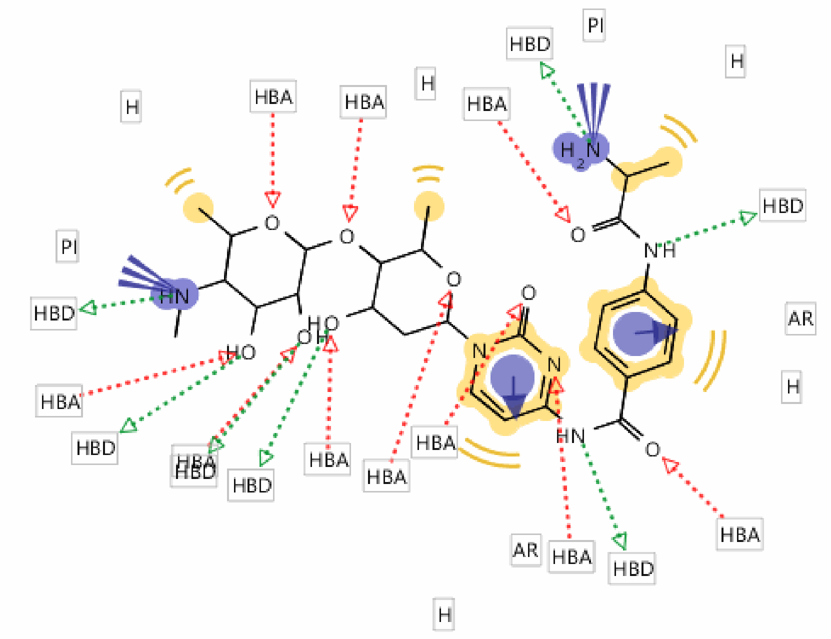
1-Epidactimicin	103531-05-1	VFBPKQSATYZKRX-AMFQOMFUSA-N	InChI=1S/C18H36N6O6/c1-8(20)10-5-4-9(21)18(29-10)30-16-12(22)14(26)17(28-3)13(15(16)27)24(2)11(25)6-23-7-19/h7-10,12-18,26-27H,4-6,20-22H2,1-3H3,(H2,19,23)/t8-,9+,10-,12+,13-,14-,15+,16+,17+,18+/m0/s1	C[C@H](N)[C@]1([H])CC[C@@H](N)[C@]([H])(O1)O[C@]2([H])[C@H](N)[C@H](O)[C@H](OC)[C@]([H])([C@H]2O)N(C(CNC=N)=O)C	Small ribosomal subunit inhibitor	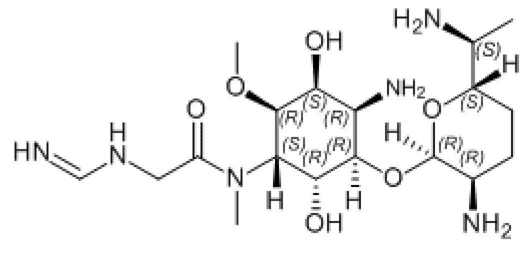	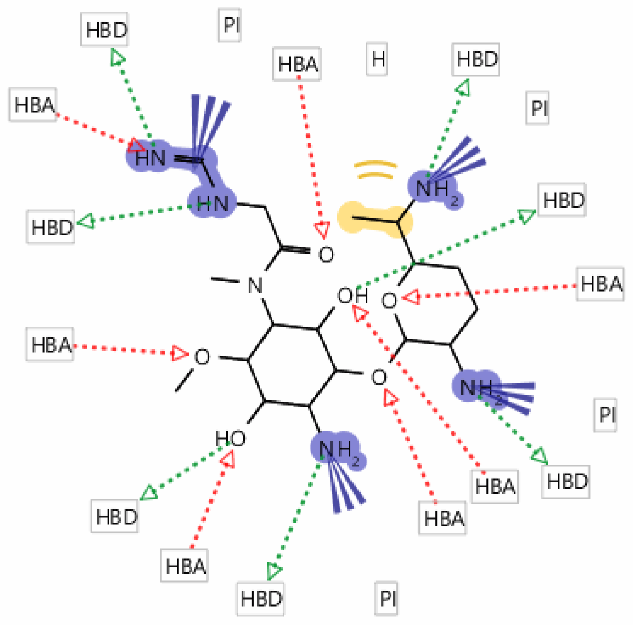
A 396I (SS 56D)	31357-30-9	LGHKNEMZGUSIBY-OREKOCCZSA-N	InChI=1S/2C19H35N3O13/c2*20-4-1-5(21)9(26)14(8(4)25)32-18-16-15(10(27)7(3-24)31-18)34-19(35-16)17(30)12(29)11(28)13(33-19)6(22)2-23/h2*4-18,23-30H,1-3,20-22H2/t4-,5+,6-,7+,8+,9-,10-,11+,12-,13+,14-,15-,16-,17+,18-,19+;4-,5+,6-,7+,8+,9-,10-,11+,12-,13+,14-,15-,16-,17+,18-,19-/m00/s1	N[C@H]1C[C@H]([C@@H]([C@]([H])([C@@H]1O)O[C@]2([C@@]3([H])O[C@@]4([C@@H]([C@H]([C@H]([C@@]([H])([C@H](CO)N)O4)O)O)O)O[C@]3([C@H]([C@@H](CO)O2)O)[H])[H])O)N.N[C@H]5C[C@H]([C@@H]([C@]([H])([C@@H]5O)O[C@]6([C@@]7([H])O[C@]8([C@@H]([C@H]([C@H]([C@@]([H])([C@H](CO)N)O8)O)O)O)O[C@]7([C@H]([C@@H](CO)O6)O)[H])[H])O)N	Small ribosomal subunit inhibitor	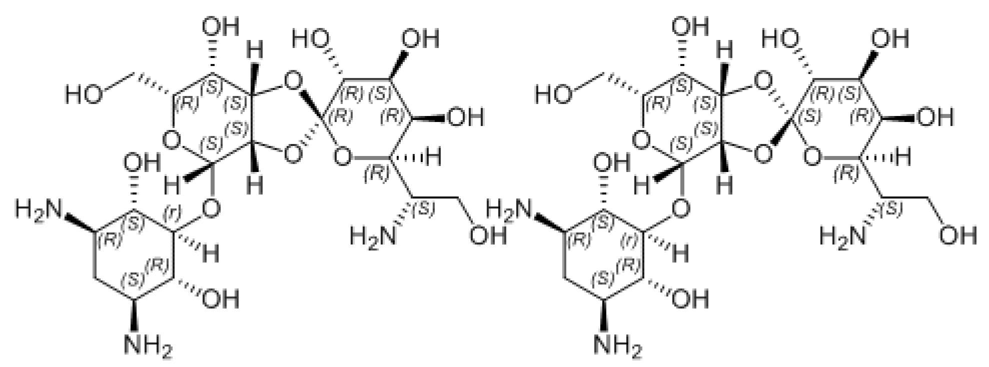	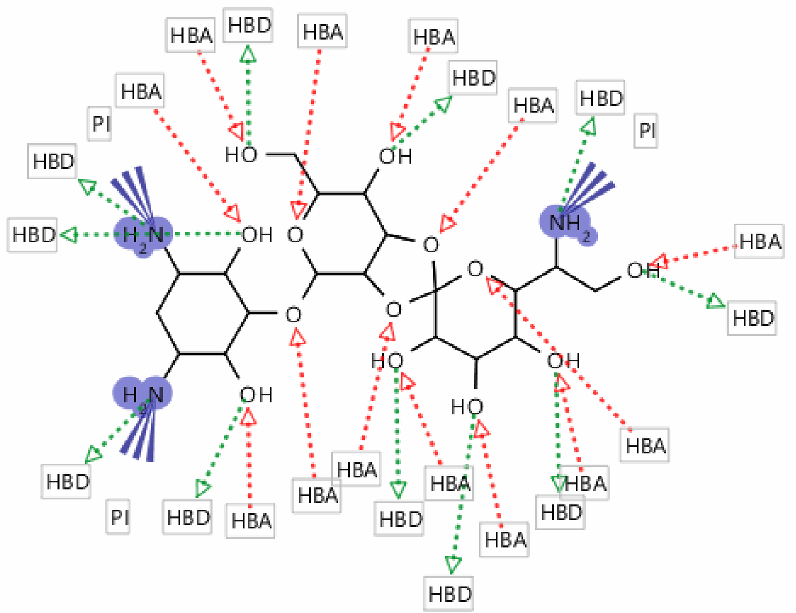
Ambistrin (streptoduocin)	8017-59-2	AWBXTNNIECFIHT-XZQQZIICSA-N	InChI=1S/C21H41N7O12.C21H39N7O12/c2*1-5-21(36,4-30)16(40-17-9(26-2)13(34)10(31)6(3-29)38-17)18(37-5)39-15-8(28-20(24)25)11(32)7(27-19(22)23)12(33)14(15)35/h5-18,26,29-36H,3-4H2,1-2H3,(H4,22,23,27)(H4,24,25,28);4-18,26,29,31-36H,3H2,1-2H3,(H4,22,23,27)(H4,24,25,28)/t2*5-,6-,7+,8-,9-,10-,11+,12-,13-,14+,15+,16-,17-,18-,21+/m00/s1	C[C@H]1[C@](O)(CO)[C@@H](O[C@@]2([H])[C@@H](NC)[C@H](O)[C@@H](O)[C@@H](O2)CO)[C@H](O[C@]3([H])[C@]([H])([C@H](O)[C@]([H])([C@H](O)[C@H]3O)NC(N)=N)NC(N)=N)O1.C[C@H]4[C@](O)(C=O)[C@@H](O[C@@]5([H])[C@@H](NC)[C@H](O)[C@@H](O)[C@@H](O5)CO)[C@H](O[C@]6([H])[C@]([H])([C@H](O)[C@]([H])([C@H](O)[C@H]6O)NC(N)=N)NC(N)=N)O4	Small ribosomal subunit inhibitor	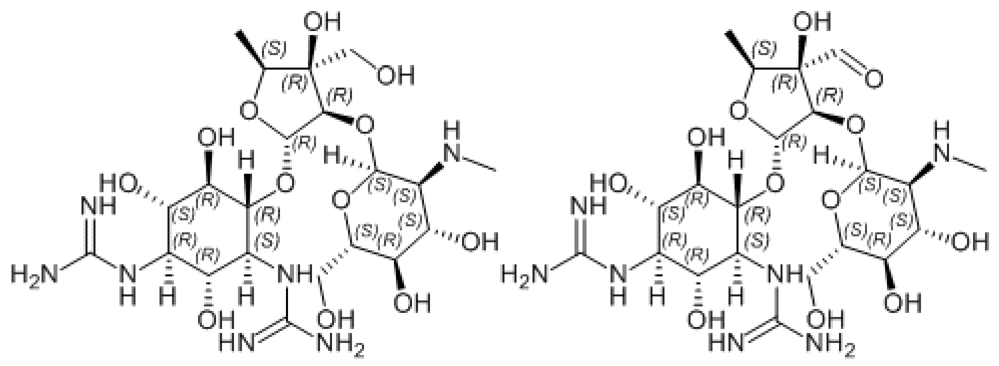	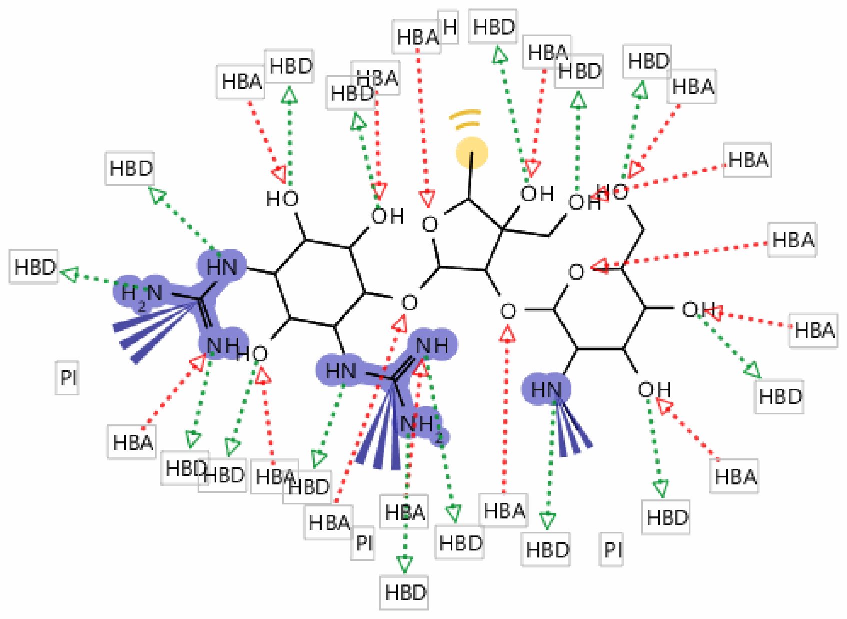
Amikacin	37517-28-5	LKCWBDHBTVXHDL-RMDFUYIESA-N	InChI=1S/C22H43N5O13/c23-2-1-8(29)20(36)27-7-3-6(25)18(39-22-16(34)15(33)13(31)9(4-24)37-22)17(35)19(7)40-21-14(32)11(26)12(30)10(5-28)38-21/h6-19,21-22,28-35H,1-5,23-26H2,(H,27,36)/t6-,7+,8-,9+,10+,11-,12+,13+,14+,15-,16+,17-,18+,19-,21+,22+/m0/s1	NCC[C@@H](C(N[C@@]1(C[C@@H]([C@]([H])([C@@H]([C@]1(O[C@@]2([C@@H]([C@H]([C@@H]([C@@H](CO)O2)O)N)O)[H])[H])O)O[C@@]3([C@@H]([C@H]([C@@H]([C@@H](CN)O3)O)O)O)[H])N)[H])=O)O	Small ribosomal subunit inhibitor	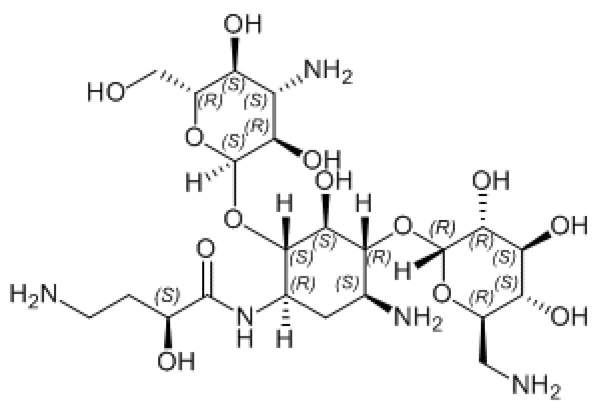	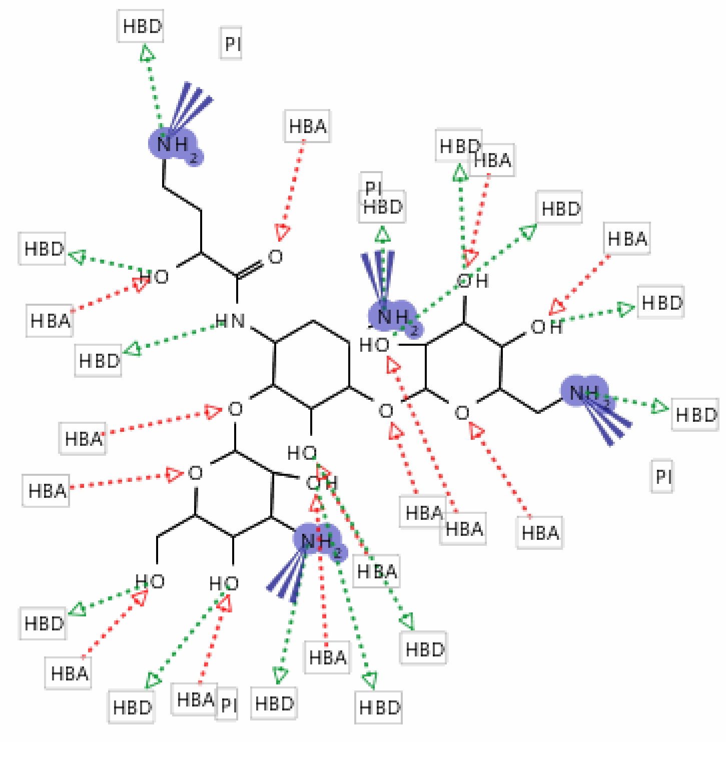

Common name	CAS Reg. No.	InChIKey	InChI	SMILES	Primary target	2D Structure	Pharmacophore[1]
Apramycin (nebramycin II)	37321-09-8	XZNUGFQTQHRASN-XQENGBIVSA-N	InChI=1S/C21H41N5O11/c1-26-11-14(30)18-8(33-20(11)37-21-16(32)13(29)10(25)9(4-27)34-21)3-7(24)19(36-18)35-17-6(23)2-5(22)12(28)15(17)31/h5-21,26-32H,2-4,22-25H2,1H3/t5-,6+,7-,8+,9-,10-,11+,12+,13+,14-,15-,16-,17-,18+,19+,20-,21-/m1/s1	CN[C@@H]1[C@]([H])(O[C@]2([H])[C@@](O[C@]([H])(O[C@@]3([C@H](C[C@H]([C@@H]([C@H]3O)O)N)N)[H])[C@H](N)C2)([H])[C@@H]1O)O[C@@]4([C@@H]([C@H]([C@@H]([C@@H](CO)O4)N)O)O)[H]	Small ribosomal subunit inhibitor	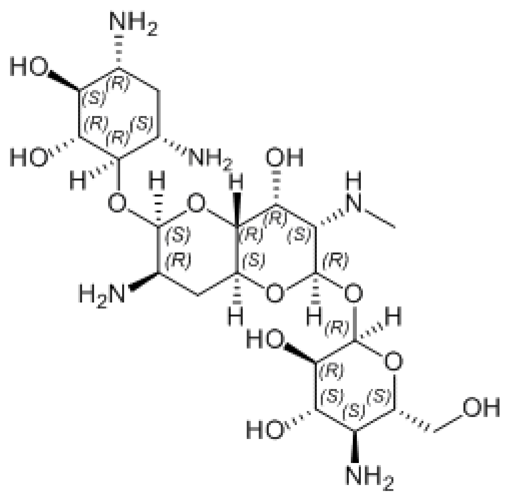	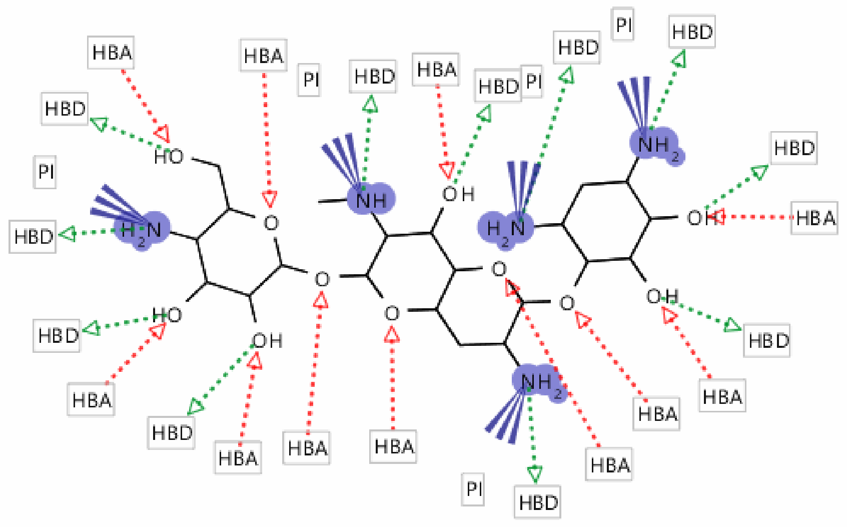
Aprosamine		SNJPWAGLRQBAAQ-MARGFZEESA-N	InChI=1S/C15H30N4O7/c1-19-8-10(21)13-7(24-14(8)23)3-6(18)15(26-13)25-12-5(17)2-4(16)9(20)11(12)22/h4-15,19-23H,2-3,16-18H2,1H3/t4-,5+,6-,7+,8+,9+,10-,11-,12-,13+,14+,15+/m1/s1	CN[C@H]1[C@H]([C@@]2([H])O[C@]([C@H](N)C[C@]2([H])O[C@@H]1O)([H])O[C@@]3([C@H](C[C@H]([C@@H]([C@H]3O)O)N)N)[H])O	Small ribosomal subunit inhibitor	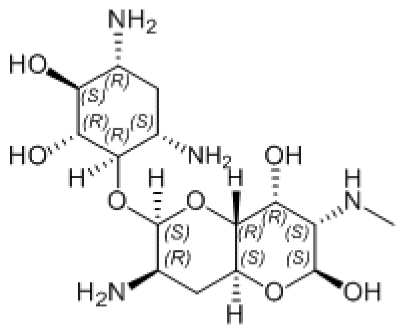	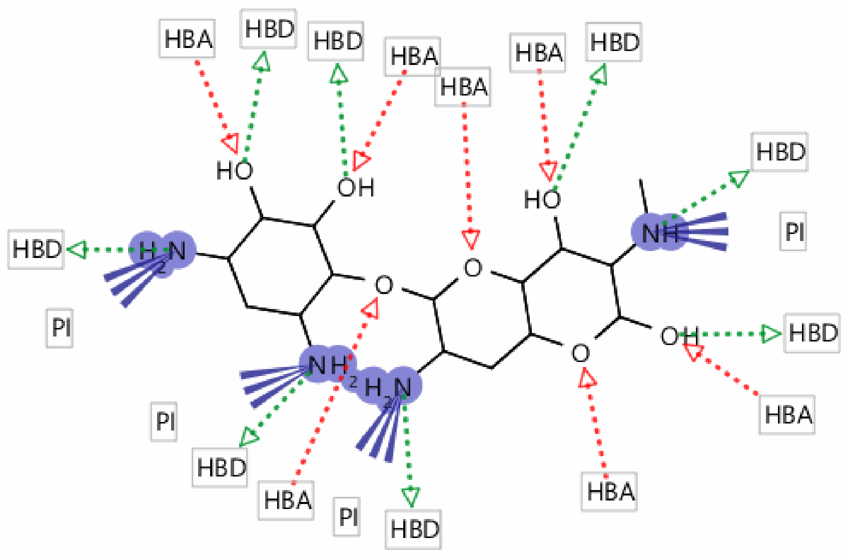
Arbekacin	51025-85-5	MKKYBZZTJQGVCD-XTCKQBCOSA-N	InChI=1S/C22H44N6O10/c23-4-3-12(30)20(34)28-11-5-10(26)18(37-21-9(25)2-1-8(6-24)35-21)17(33)19(11)38-22-16(32)14(27)15(31)13(7-29)36-22/h8-19,21-22,29-33H,1-7,23-27H2,(H,28,34)/t8-,9+,10-,11+,12-,13+,14-,15+,16+,17-,18+,19-,21+,22+/m0/s1	NCC[C@@H](C(N[C@@]1(C[C@@H]([C@]([H])([C@@H]([C@]1(O[C@@]2([C@@H]([C@H]([C@@H]([C@@H](CO)O2)O)N)O)[H])[H])O)O[C@@]3([C@@H](CC[C@@H](CN)O3)N)[H])N)[H])=O)O	Small ribosomal subunit inhibitor	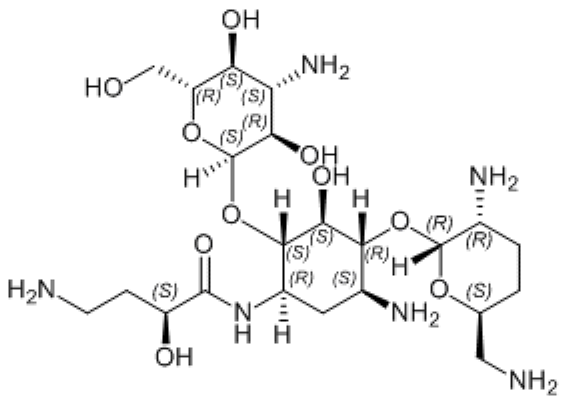	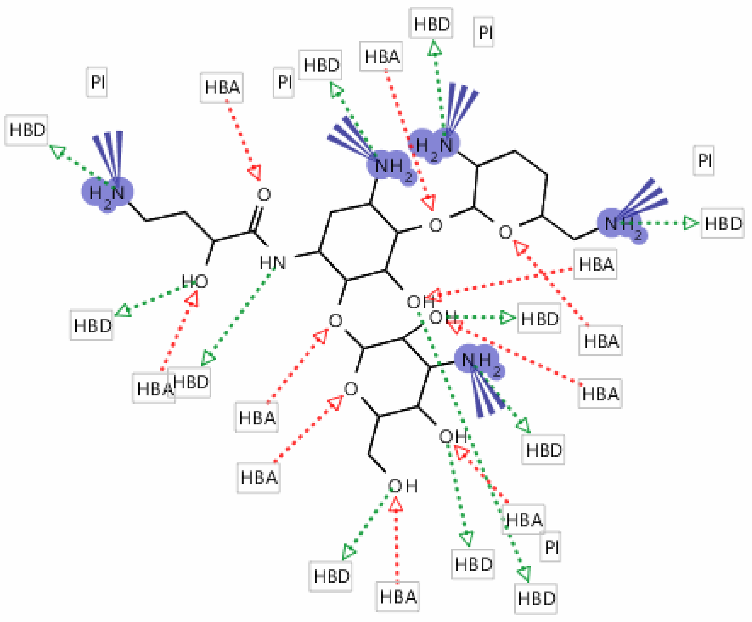
Astromicin (fortimicin A)	55779-06-1	BIDUPMYXGFNAEJ-APGVDKLISA-N	InChI=1S/C17H35N5O6/c1-7(19)9-5-4-8(20)17(27-9)28-15-11(21)13(24)16(26-3)12(14(15)25)22(2)10(23)6-18/h7-9,11-17,24-25H,4-6,18-21H2,1-3H3/t7-,8+,9-,11-,12-,13-,14+,15+,16+,17+/m0/s1	C[C@H](N)[C@]1([H])CC[C@@H](N)[C@](O1)(O[C@]2([H])[C@@H](N)[C@H](O)[C@H](OC)[C@]([C@H]2O)(N(C(CN)=O)C)[H])[H]	Small ribosomal subunit inhibitor	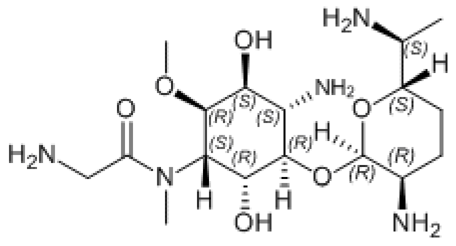	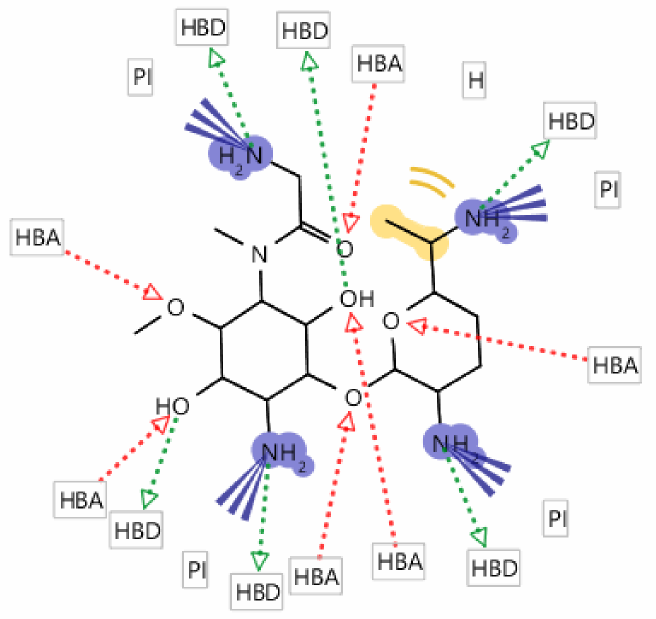
Astromicin B (fortimicin B)	54783-95-8	WFMQYKIRAVMXSU-LCVFDZPESA-N	InChI=1S/C15H32N4O5/c1-6(16)8-5-4-7(17)15(23-8)24-13-9(18)11(20)14(22-3)10(19-2)12(13)21/h6-15,19-21H,4-5,16-18H2,1-3H3/t6-,7+,8-,9-,10-,11-,12+,13+,14+,15+/m0/s1	C[C@@H]([C@@]1(CC[C@H]([C@](O[C@@]2([C@H]([C@@H]([C@@H]([C@H]([C@H]2O)NC)OC)O)N)[H])([H])O1)N)[H])N	Small ribosomal subunit inhibitor	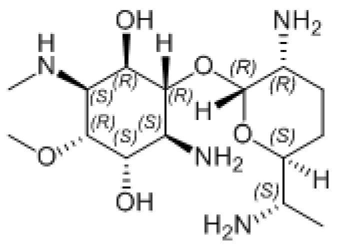	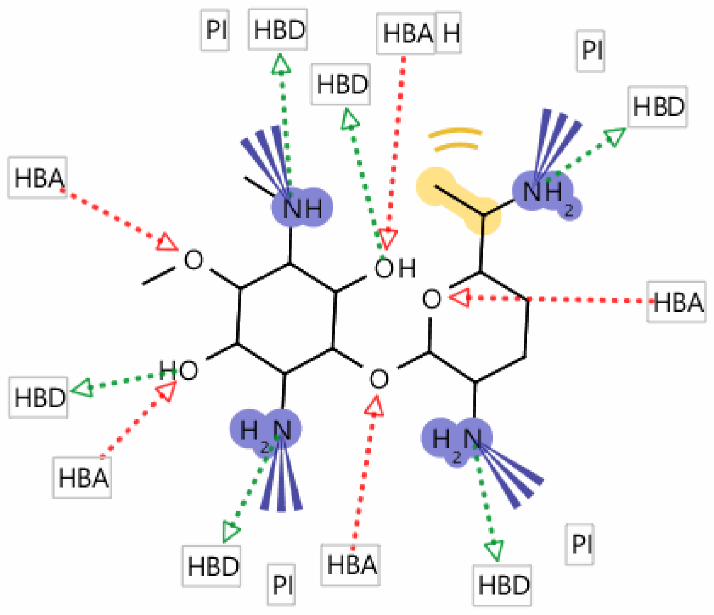
Bekanamycin (kanamycin B; nebramycin V)	4696-76-8	SKKLOUVUUNMCJE-FQSMHNGLSA-N	InChI=1S/C18H37N5O10/c19-2-6-11(26)12(27)9(23)17(30-6)32-15-4(20)1-5(21)16(14(15)29)33-18-13(28)8(22)10(25)7(3-24)31-18/h4-18,24-29H,1-3,19-23H2/t4-,5+,6+,7+,8-,9+,10+,11+,12+,13+,14-,15+,16-,17+,18+/m0/s1	NC[C@@H]1[C@H]([C@@H]([C@H]([C@]([H])(O1)O[C@@]2([C@H](C[C@H]([C@@]([H])([C@H]2O)O[C@@]3([C@@H]([C@H]([C@@H]([C@@H](CO)O3)O)N)O)[H])N)N)[H])N)O)O	Small ribosomal subunit inhibitor	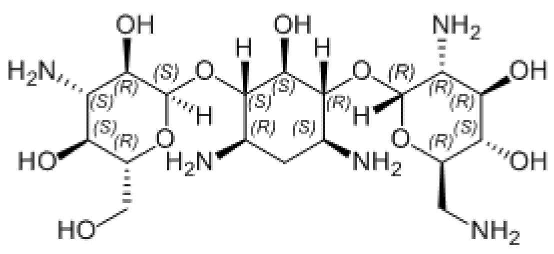	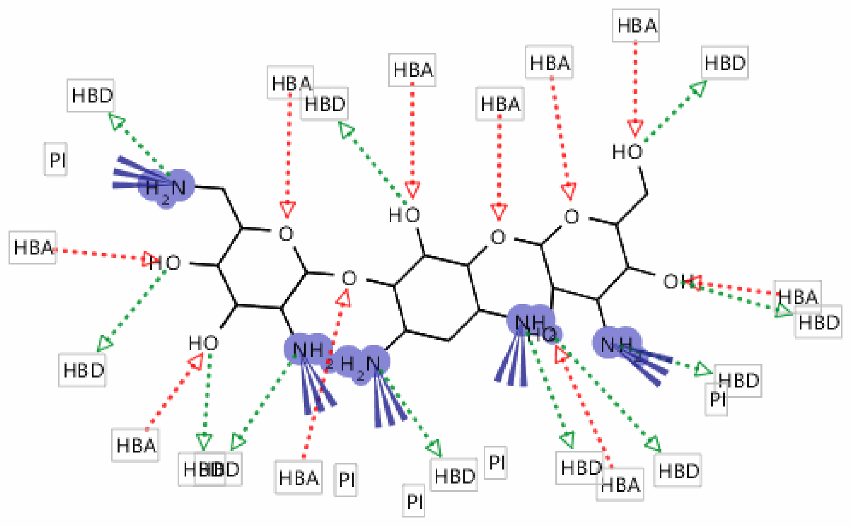

Common name	CAS Reg. No.	InChIKey	InChI	SMILES	Primary target	2D Structure	Pharmacophore[1]
Betamicin (gentamicin B)	36889-15-3	RHRAMPXHWHSKQB-GGEUKFTFSA-N	InChI=1S/C19H38N4O10/c1-19(29)5-30-17(13(28)16(19)23-2)32-14-6(21)3-7(22)15(12(14)27)33-18-11(26)10(25)9(24)8(4-20)31-18/h6-18,23-29H,3-5,20-22H2,1-2H3/t6-,7+,8-,9-,10+,11-,12-,13-,14+,15-,16-,17-,18-,19+/m1/s1	C[C@@]1(CO[C@@]([H])([C@@H]([C@H]1NC)O)O[C@]2([C@@H](C[C@@H]([C@]([H])([C@@H]2O)O[C@@]3([C@@H]([C@H]([C@@H]([C@@H](CN)O3)O)O)O)[H])N)N)[H])O	Small ribosomal subunit inhibitor	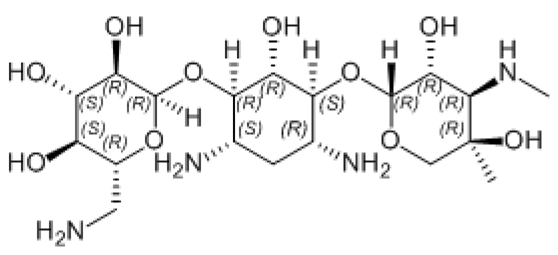	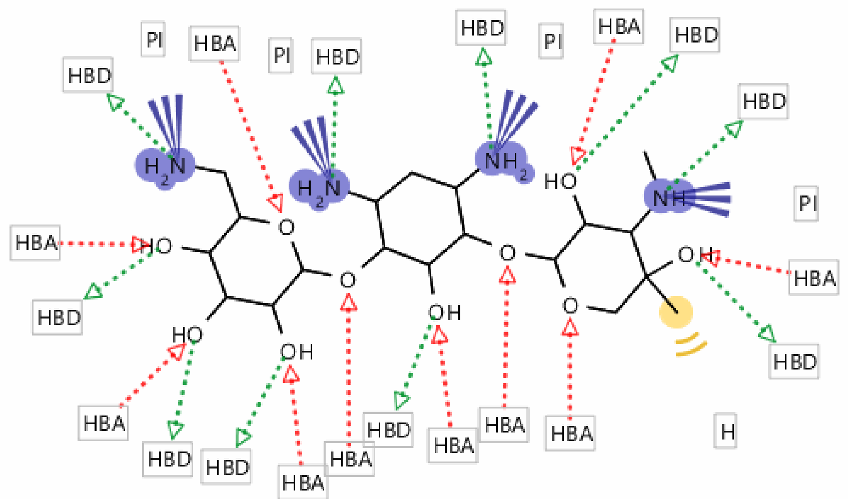
Butikacin	59733-86-7	OCFOTEIMZBKQFS-DGMGPCKZSA-N	InChI=1S/C22H45N5O12/c23-2-1-7(29)5-27-9-3-8(25)19(38-22-17(34)16(33)14(31)10(4-24)36-22)18(35)20(9)39-21-15(32)12(26)13(30)11(6-28)37-21/h7-22,27-35H,1-6,23-26H2/t7-,8-,9+,10+,11+,12-,13+,14+,15+,16-,17+,18-,19+,20-,21+,22+/m0/s1	NCC[C@H](O)CN[C@]1([H])C[C@H](N)[C@]([C@H](O)[C@@]1([H])O[C@]2([H])[C@H](O)[C@@H](N)[C@H](O)[C@H](O2)CO)(O[C@]3([H])[C@H](O)[C@@H](O)[C@H](O)[C@H](O3)CN)[H]	Small ribosomal subunit inhibitor	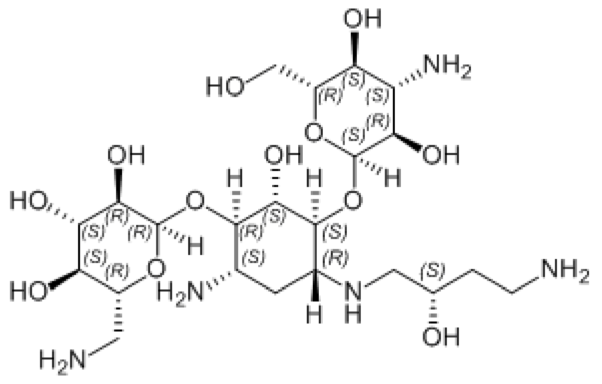	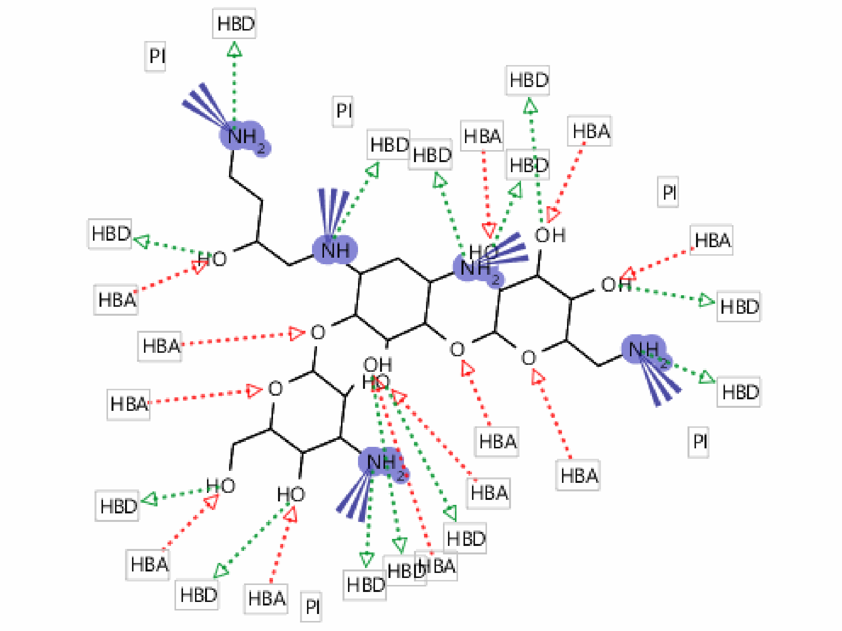
Butirosin A	34291-02-6	XEQLFNPSYWZPOW-SVRMBHBBSA-N	InChI=1S/C21H41N5O12/c22-2-1-8(28)19(34)26-7-3-6(24)17(37-20-11(25)15(32)13(30)9(4-23)35-20)18(12(7)29)38-21-16(33)14(31)10(5-27)36-21/h6-18,20-21,27-33H,1-5,22-25H2,(H,26,34)/t6-,7+,8-,9+,10+,11+,12-,13+,14-,15+,16+,17+,18+,20+,21-/m0/s1	NCC[C@H](O)C(N[C@]1([H])C[C@H](N)[C@]([C@@]([C@H]1O)(O[C@H]2[C@H](O)[C@@H](O)[C@H](O2)CO)[H])(O[C@]3([H])[C@H](N)[C@@H](O)[C@H](O)[C@H](O3)CN)[H])=O	Small ribosomal subunit inhibitor	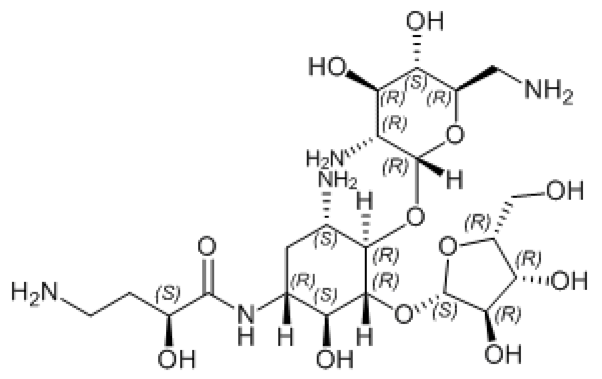	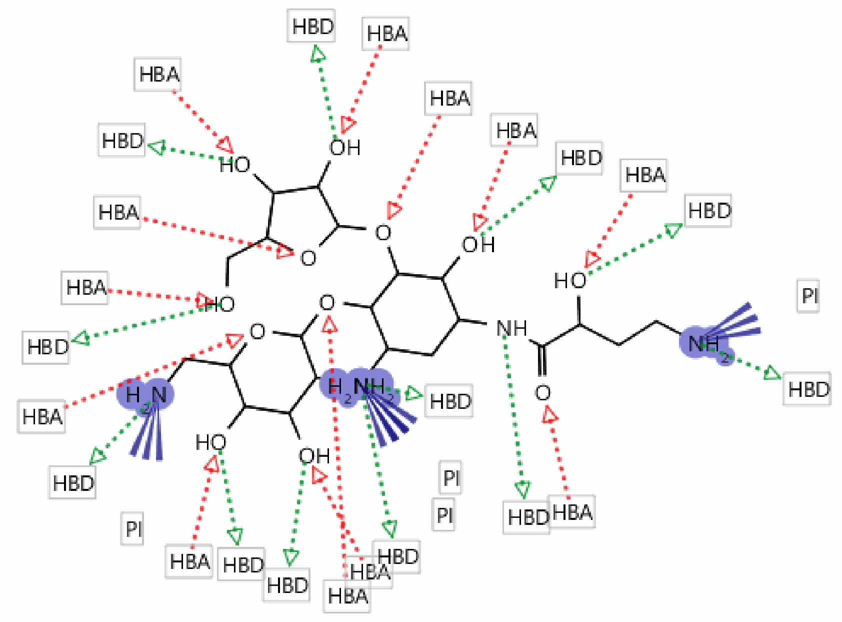
Butirosin B	34291-03-7	XEQLFNPSYWZPOW-HBYCGHPUSA-N	InChI=1S/C21H41N5O12/c22-2-1-8(28)19(34)26-7-3-6(24)17(37-20-11(25)15(32)13(30)9(4-23)35-20)18(12(7)29)38-21-16(33)14(31)10(5-27)36-21/h6-18,20-21,27-33H,1-5,22-25H2,(H,26,34)/t6-,7+,8-,9+,10+,11+,12-,13+,14+,15+,16+,17+,18+,20+,21-/m0/s1	NCC[C@@H](C(N[C@@]1(C[C@@H]([C@]([H])([C@@]([H])([C@H]1O)O[C@H]2[C@@H]([C@@H]([C@@H](CO)O2)O)O)O[C@@]3([C@@H]([C@H]([C@@H]([C@@H](CN)O3)O)O)N)[H])N)[H])=O)O	Small ribosomal subunit inhibitor	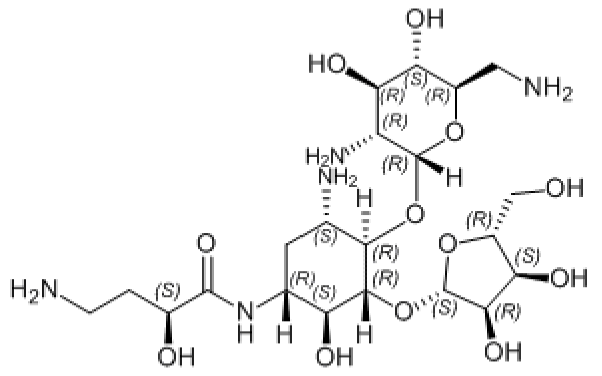	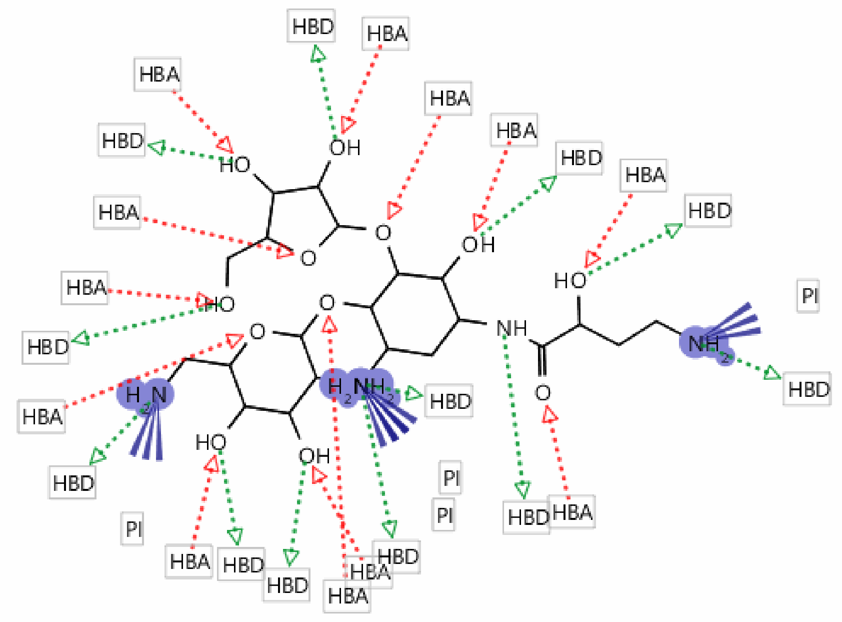
Dactimicin	73196-97-1	VFBPKQSATYZKRX-WKEGKRRDSA-N	InChI=1S/C18H36N6O6/c1-8(20)10-5-4-9(21)18(29-10)30-16-12(22)14(26)17(28-3)13(15(16)27)24(2)11(25)6-23-7-19/h7-10,12-18,26-27H,4-6,20-22H2,1-3H3,(H2,19,23)/t8-,9+,10-,12-,13-,14-,15+,16+,17+,18+/m0/s1	C[C@H](N)[C@]1([H])CC[C@@H](N)[C@](O1)(O[C@]2([H])[C@@H](N)[C@H](O)[C@H](OC)[C@]([C@H]2O)(N(C(CNC=N)=O)C)[H])[H]	Small ribosomal subunit inhibitor	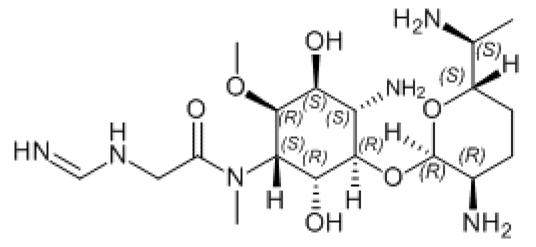	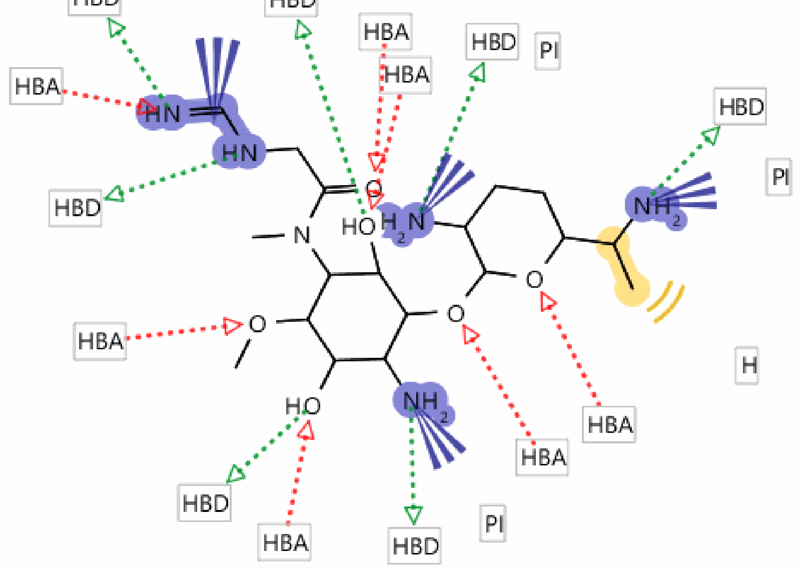
Destomycin A	14918-35-5	YELMTJULBWLPRL-RMZUDAPHSA-N	InChI=1S/2C20H37N3O13/c2*1-23-7-2-5(21)9(26)15(10(7)27)33-19-17-16(11(28)8(4-25)32-19)35-20(36-17)18(31)13(30)12(29)14(34-20)6(22)3-24/h2*5-19,23-31H,2-4,21-22H2,1H3/t5-,6-,7+,8+,9+,10-,11-,12+,13-,14+,15-,16-,17-,18+,19-,20+;5-,6-,7+,8+,9+,10-,11-,12+,13-,14+,15-,16-,17-,18+,19-,20-/m00/s1	CN[C@@H]1C[C@@H]([C@H]([C@@]([H])([C@H]1O)O[C@]2([C@@]3([H])O[C@@]4([C@@H]([C@H]([C@H]([C@@]([H])([C@H](CO)N)O4)O)O)O)O[C@]3([C@H]([C@@H](CO)O2)O)[H])[H])O)N.CN[C@@H]5C[C@@H]([C@H]([C@@]([H])([C@H]5O)O[C@]6([C@@]7([H])O[C@]8([C@@H]([C@H]([C@H]([C@@]([H])([C@H](CO)N)O8)O)O)O)O[C@]7([C@H]([C@@H](CO)O6)O)[H])[H])O)N	Small ribosomal subunit inhibitor	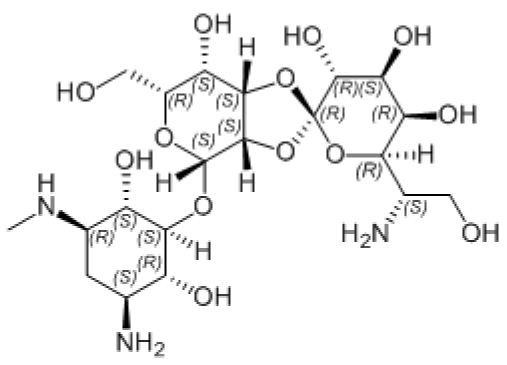 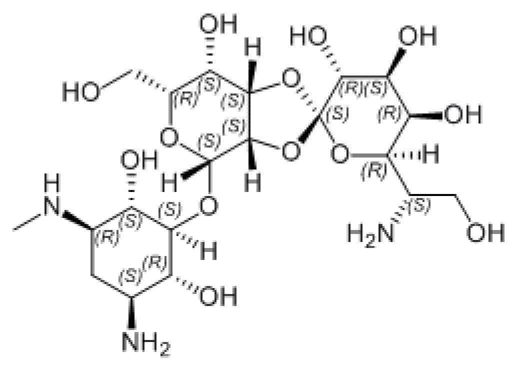	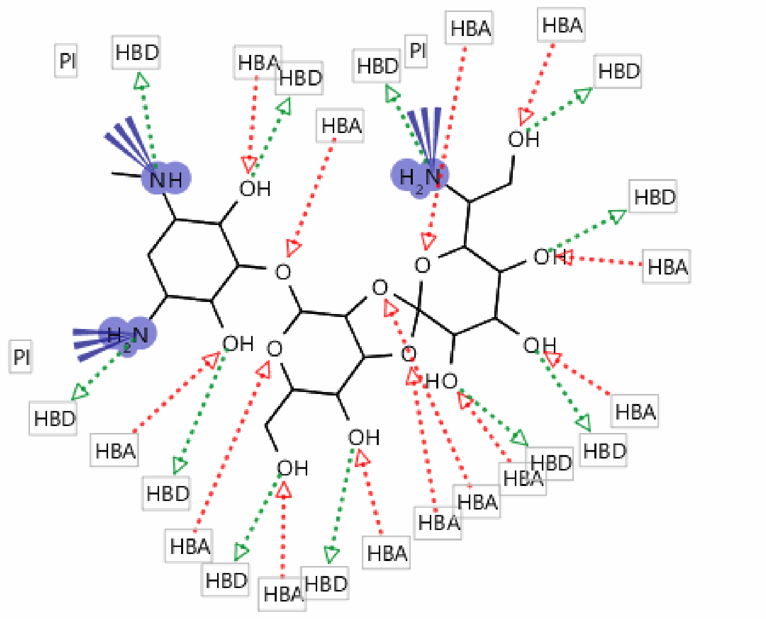

Common name	CAS Reg. No.	InChIKey	InChI	SMILES	Primary target	2D Structure	Pharmacophore[1]
Destomycin B	11005-98-4	VBRSUYJOBFNQTD-OVGJSIDVSA-N	InChI=1S/2C21H39N3O13/c2*1-23-7-3-8(24-2)11(28)16(10(7)27)34-20-18-17(12(29)9(5-26)33-20)36-21(37-18)19(32)14(31)13(30)15(35-21)6(22)4-25/h2*6-20,23-32H,3-5,22H2,1-2H3/t6-,7-,8+,9+,10+,11-,12+,13-,14-,15+,16-,17-,18-,19+,20-,21+;6-,7-,8+,9+,10+,11-,12+,13-,14-,15+,16-,17-,18-,19+,20-,21-/m00/s1	CN[C@H]1C[C@H]([C@@H]([C@]([H])([C@@H]1O)O[C@]2([C@@]3([H])O[C@@]4([C@@H]([C@H]([C@@H]([C@@]([H])([C@H](CO)N)O4)O)O)O)O[C@]3([C@@H]([C@@H](CO)O2)O)[H])[H])O)NC.CN[C@H]5C[C@H]([C@@H]([C@]([H])([C@@H]5O)O[C@]6([C@@]7([H])O[C@]8([C@@H]([C@H]([C@@H]([C@@]([H])([C@H](CO)N)O8)O)O)O)O[C@]7([C@@H]([C@@H](CO)O6)O)[H])[H])O)NC	Small ribosomal subunit inhibitor	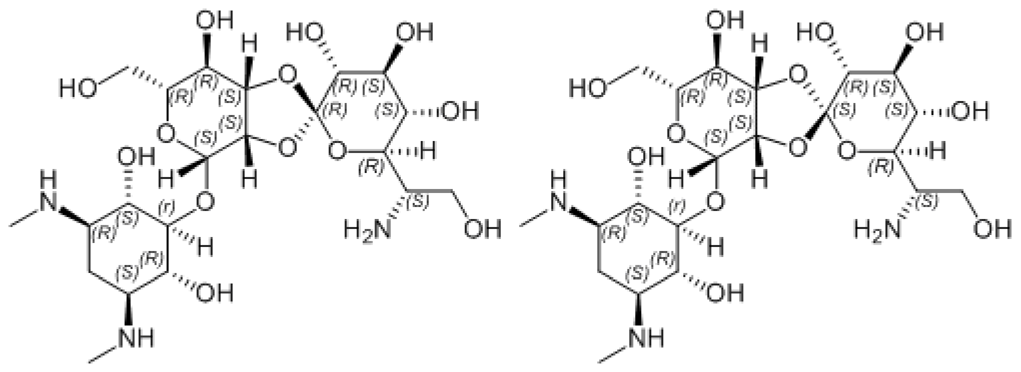	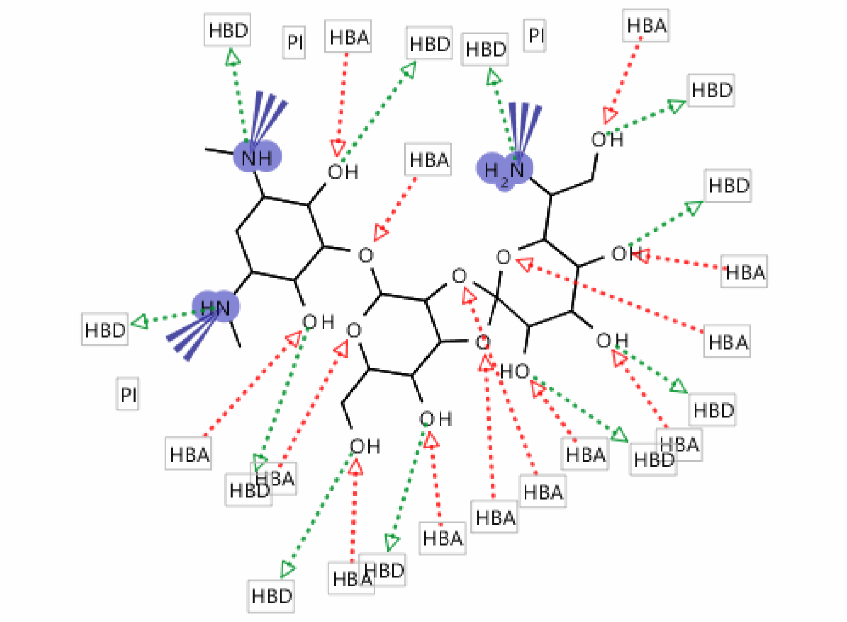
Dibekacin	34493-98-6	JJCQSGDBDPYCEO-XVZSLQNASA-N	InChI=1S/C18H37N5O8/c19-4-6-1-2-7(20)17(28-6)30-15-8(21)3-9(22)16(14(15)27)31-18-13(26)11(23)12(25)10(5-24)29-18/h6-18,24-27H,1-5,19-23H2/t6-,7+,8-,9+,10+,11-,12+,13+,14-,15+,16-,17+,18+/m0/s1	NC[C@@H]1CC[C@H]([C@]([H])(O1)O[C@@]2([C@H](C[C@H]([C@@]([H])([C@H]2O)O[C@@]3([C@@H]([C@H]([C@@H]([C@@H](CO)O3)O)N)O)[H])N)N)[H])N	Small ribosomal subunit inhibitor	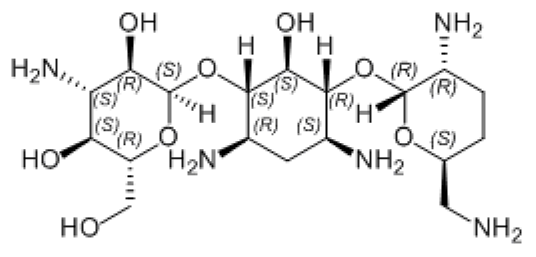	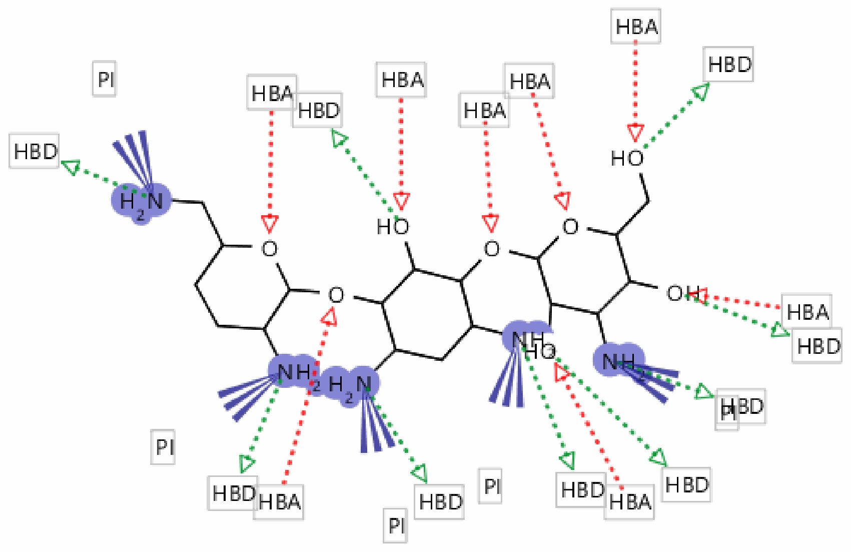
Dihydrostreptomycin	128-46-1	ASXBYYWOLISCLQ-HZYVHMACSA-N	InChI=1S/C21H41N7O12/c1-5-21(36,4-30)16(40-17-9(26-2)13(34)10(31)6(3-29)38-17)18(37-5)39-15-8(28-20(24)25)11(32)7(27-19(22)23)12(33)14(15)35/h5-18,26,29-36H,3-4H2,1-2H3,(H4,22,23,27)(H4,24,25,28)/t5-,6-,7+,8-,9-,10-,11+,12-,13-,14+,15+,16-,17-,18-,21+/m0/s1	C[C@H]1[C@](O)(CO)[C@@H](O[C@@]2([H])[C@@H](NC)[C@H](O)[C@@H](O)[C@@H](O2)CO)[C@H](O[C@]3([H])[C@]([H])([C@H](O)[C@]([H])([C@H](O)[C@H]3O)NC(N)=N)NC(N)=N)O1	Small ribosomal subunit inhibitor	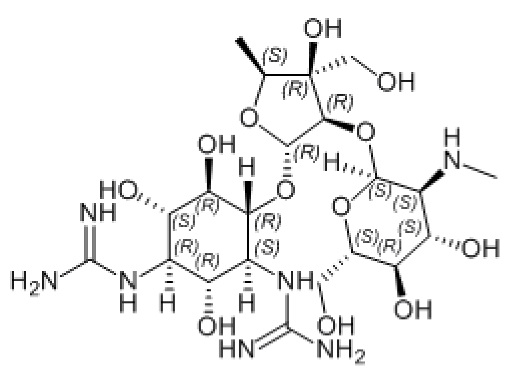	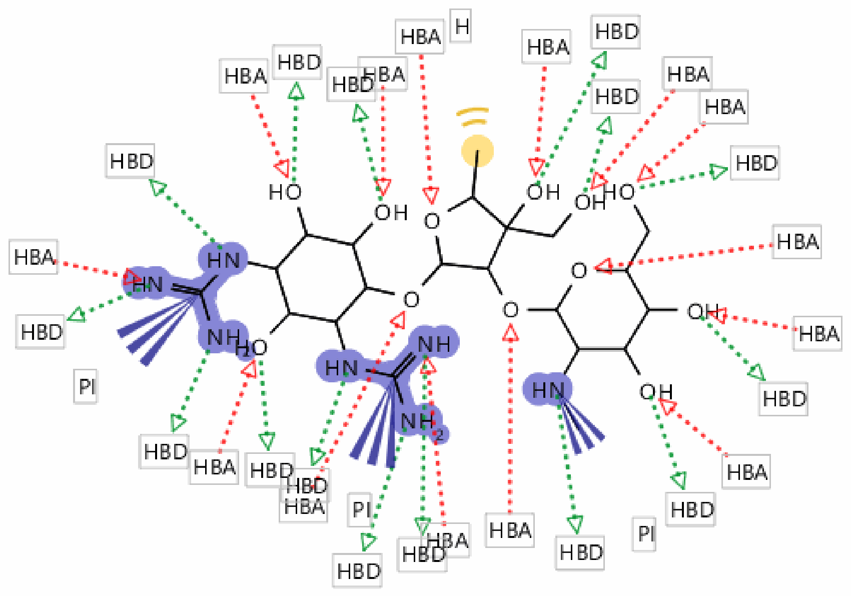
Etimicin	59711-96-5	NZGMVSJQULXLHF-RAKCNUBFSA-N	InChI=1S/C21H43N5O7/c1-4-26-13-7-12(24)16(32-19-11(23)6-5-10(8-22)31-19)14(27)17(13)33-20-15(28)18(25-3)21(2,29)9-30-20/h10-20,25-29H,4-9,22-24H2,1-3H3/t10-,11+,12-,13+,14-,15+,16+,17-,18+,19+,20+,21-/m0/s1	CCN[C@@]1(C[C@@H]([C@]([H])([C@@H]([C@]1(O[C@@]2([C@@H]([C@H]([C@](O)(C)CO2)NC)O)[H])[H])O)O[C@@]3([C@@H](CC[C@@H](CN)O3)N)[H])N)[H]	Small ribosomal subunit inhibitor	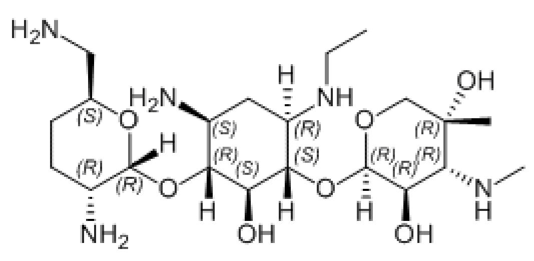	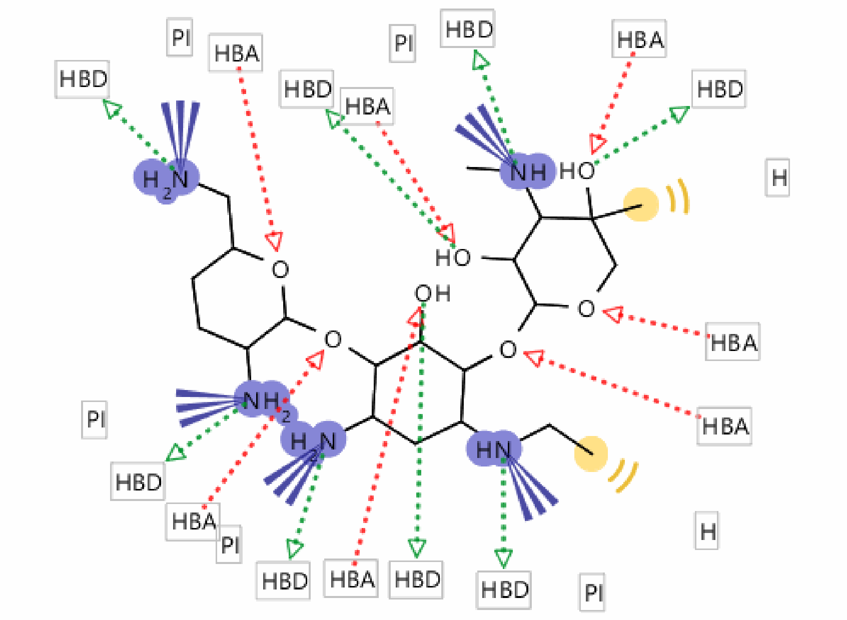
Fortimicin AE	72843-38-0	WFMQYKIRAVMXSU-OIVPKDKZSA-N	InChI=1S/C15H32N4O5/c1-6(16)8-5-4-7(17)15(23-8)24-13-9(18)11(20)14(22-3)10(19-2)12(13)21/h6-15,19-21H,4-5,16-18H2,1-3H3/t6-,7+,8-,9-,10+,11-,12+,13-,14-,15+/m0/s1	C[C@@H]([C@@]1(CC[C@H]([C@](O[C@]2([C@H]([C@@H]([C@H]([C@@H]([C@H]2O)NC)OC)O)N)[H])([H])O1)N)[H])N	Small ribosomal subunit inhibitor	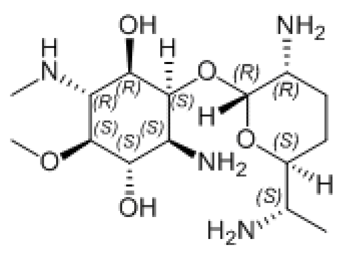	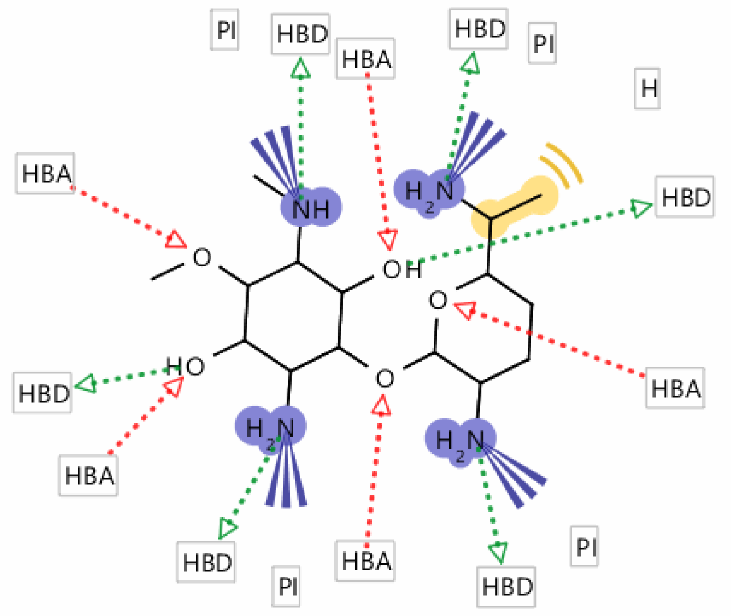
Fortimicin AH	74918-32-4	OSHGHQXTURHSOO-ZPPFTRLZSA-N	InChI=1S/C15H27N3O5/c1-6-8-5-4-7(18-6)15(22-8)23-13-9(16)11(19)14(21-3)10(17-2)12(13)20/h7-15,17,19-20H,4-5,16H2,1-3H3/t7-,8-,9+,10+,11+,12-,13-,14-,15-/m1/s1	CC1=N[C@@H]2CC[C@H]1O[C@@]2(O[C@@]3([C@H]([C@@H]([C@@H]([C@H]([C@H]3O)NC)OC)O)N)[H])[H]	Small ribosomal subunit inhibitor	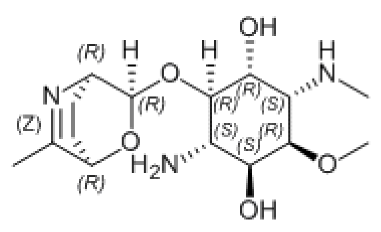	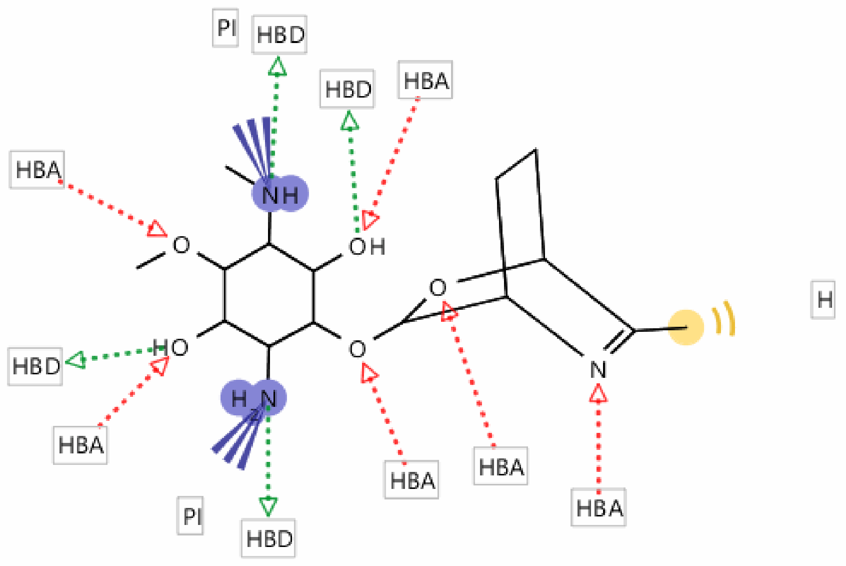

Common name	CAS Reg. No.	InChIKey	InChI	SMILES	Primary target	2D Structure	Pharmacophore[1]
Fortimicin AI	75419-92-0	OSHGHQXTURHSOO-CLIGTQAVSA-N	InChI=1S/C15H27N3O5/c1-6-8-5-4-7(18-6)15(22-8)23-13-9(16)11(19)14(21-3)10(17-2)12(13)20/h7-15,17,19-20H,4-5,16H2,1-3H3/t7-,8-,9+,10+,11+,12-,13-,14+,15-/m1/s1	CC1=N[C@@H]2CC[C@H]1O[C@@]2(O[C@@]3([C@H]([C@@H]([C@H]([C@H]([C@H]3O)NC)OC)O)N)[H])[H]	Small ribosomal subunit inhibitor	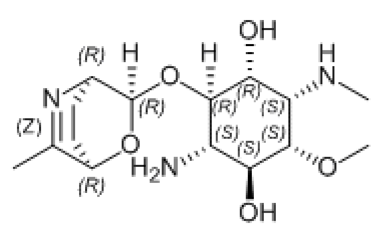	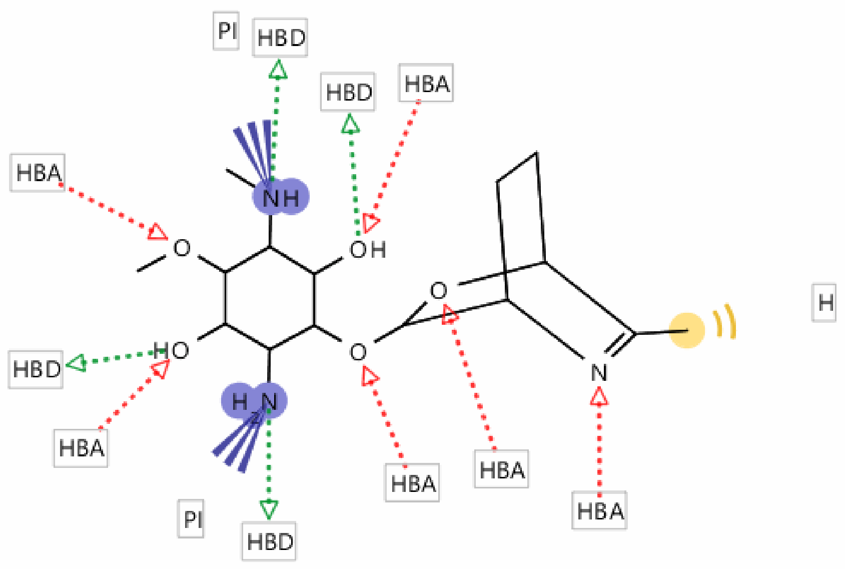
Fortimicin AK	74918-33-5	FLLSACKNCATULI-QUCDNLDZSA-N	InChI=1S/C14H29N3O6/c1-17-9-11(20)12(8(16)10(19)13(9)21-2)23-14-7(15)4-3-6(5-18)22-14/h6-14,17-20H,3-5,15-16H2,1-2H3/t6-,7+,8-,9+,10-,11+,12+,13+,14+/m0/s1	CN[C@@H]1[C@@H](O)[C@@]([C@@H](N)[C@H](O)[C@@H]1OC)(O[C@]2([H])[C@H](N)CC[C@H](O2)CO)[H]	Small ribosomal subunit inhibitor	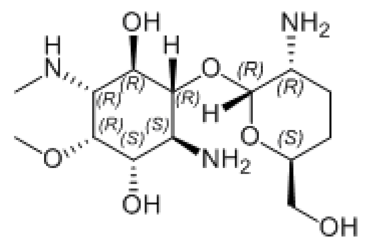	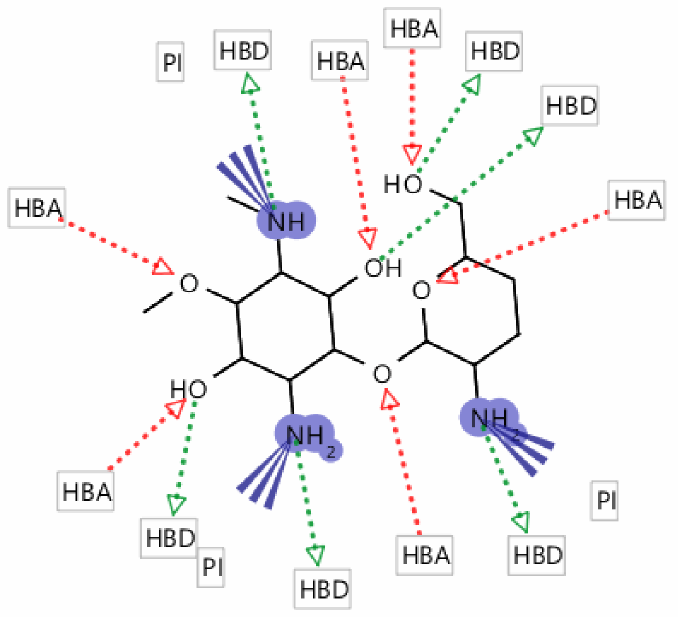
Fortimicin AL	74958-27-3	XYMQMTNLCXERSU-PVWCKTGBSA-N	InChI=1S/C14H28N4O5/c1-5(15)7-4-3-6(16)14(22-7)23-13-8(17)10(19)11(20)9(18-2)12(13)21/h4-6,8-14,18-21H,3,15-17H2,1-2H3/t5-,6+,8-,9-,10-,11-,12+,13+,14+/m0/s1	C[C@@H](C1=CC[C@H]([C@](O[C@@]2([C@H]([C@@H]([C@H]([C@@H]([C@H]2O)NC)O)O)N)[H])([H])O1)N)N	Small ribosomal subunit inhibitor	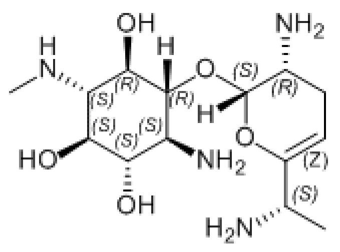	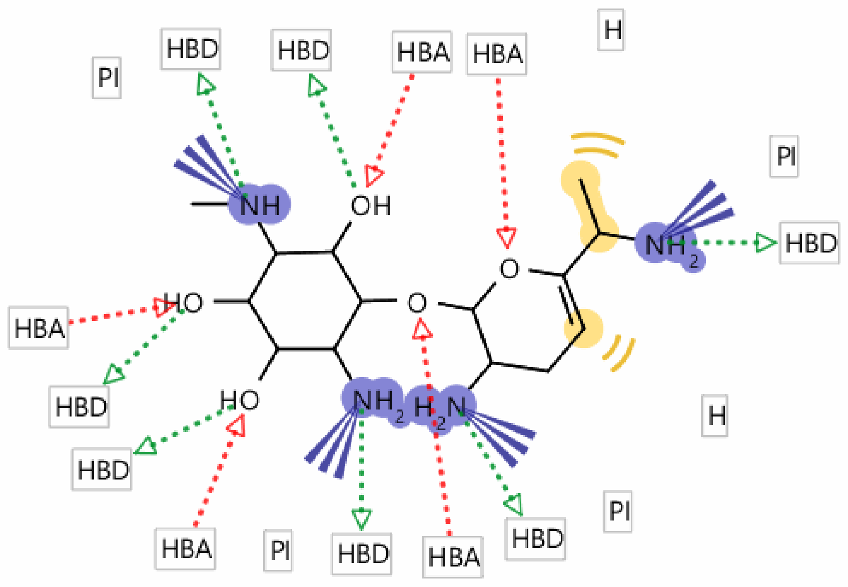
Fortimicin AM	74958-28-4	KBIFQFMZFNTDKE-NZCWFKHXSA-N	InChI=1S/C14H30N4O5/c1-5(15)7-4-3-6(16)14(22-7)23-13-8(17)10(19)11(20)9(18-2)12(13)21/h5-14,18-21H,3-4,15-17H2,1-2H3/t5-,6+,7-,8-,9-,10-,11+,12+,13+,14+/m0/s1	C[C@@H]([C@@]1(CC[C@H]([C@](O[C@@]2([C@H]([C@@H]([C@@H]([C@@H]([C@H]2O)NC)O)O)N)[H])([H])O1)N)[H])N	Small ribosomal subunit inhibitor	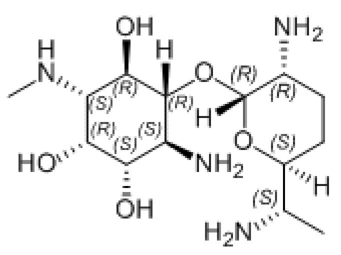	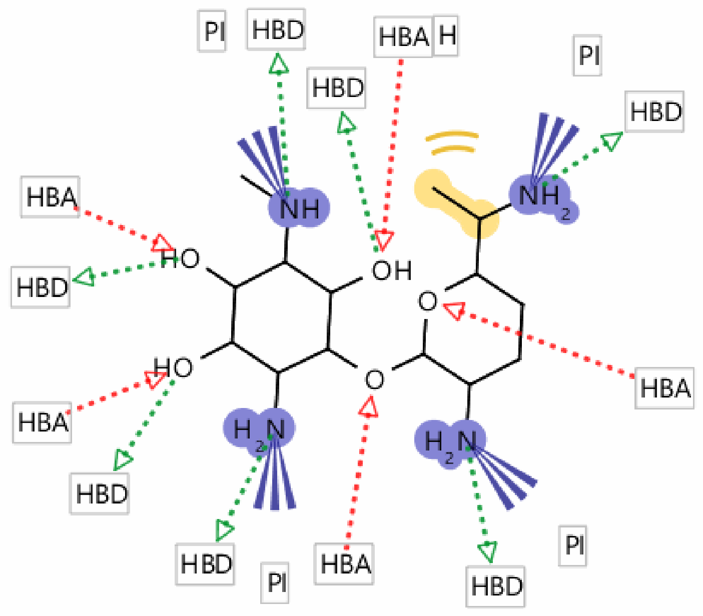
Fortimicin AN	74918-34-6	DDVCNPOLKJXLSW-OPMOAULHSA-N	InChI=1S/C16H33N5O6/c1-6(18)8-4-3-7(19)16(26-8)27-15-11(21-9(22)5-17)13(24)12(23)10(20-2)14(15)25/h6-8,10-16,20,23-25H,3-5,17-19H2,1-2H3,(H,21,22)/t6-,7+,8-,10+,11-,12+,13-,14+,15+,16+/m0/s1	C[C@H](N)[C@]1([H])CC[C@@H](N)[C@](O1)(O[C@]2([H])[C@]([C@H](O)[C@H](O)[C@@H](NC)[C@H]2O)(NC(CN)=O)[H])[H]	Small ribosomal subunit inhibitor	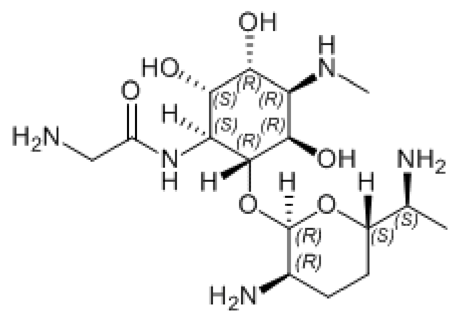	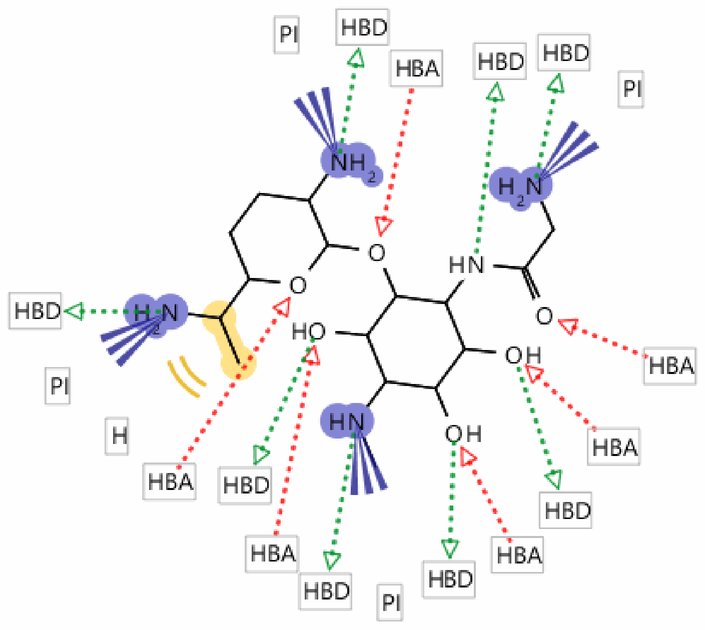
Fortimicin AO	74918-35-7	KWFHTRCEUOZBDZ-IXMAVWSQSA-N	InChI=1S/C13H27N3O8/c1-16-6-10(21)8(19)4(14)12(11(6)22)24-13-5(15)9(20)7(18)3(2-17)23-13/h3-13,16-22H,2,14-15H2,1H3/t3-,4+,5-,6+,7-,8+,9-,10+,11-,12-,13-/m1/s1	CN[C@H]1[C@@H]([C@H]([C@@H]([C@]([H])([C@@H]1O)O[C@@]2([C@@H]([C@H]([C@@H]([C@@H](CO)O2)O)O)N)[H])N)O)O	Small ribosomal subunit inhibitor	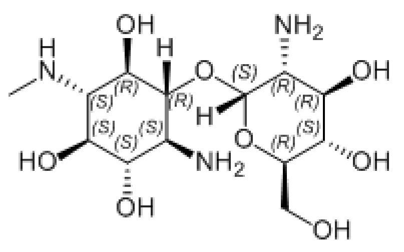	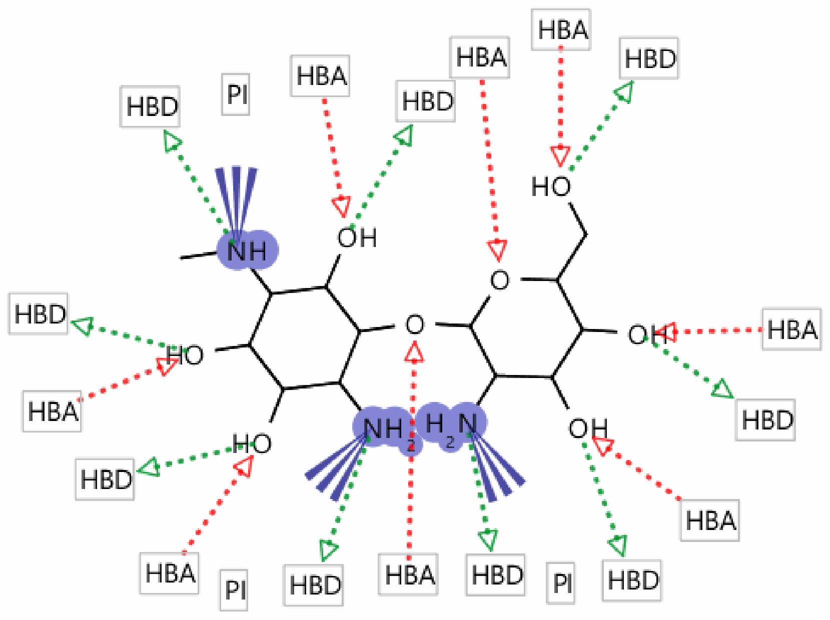

Common name	CAS Reg. No.	InChIKey	InChI	SMILES	Primary target	2D Structure	Pharmacophore[1]
Fortimicin AP	74958-29-5	KBIFQFMZFNTDKE-DHPLRXPASA-N	InChI=1S/C14H30N4O5/c1-5(15)7-4-3-6(16)14(22-7)23-13-8(17)10(19)11(20)9(18-2)12(13)21/h5-14,18-21H,3-4,15-17H2,1-2H3/t5-,6+,7-,8-,9-,10-,11-,12+,13+,14+/m0/s1	C[C@@H]([C@@]1(CC[C@H]([C@](O[C@@]2([C@H]([C@@H]([C@H]([C@@H]([C@H]2O)NC)O)O)N)[H])([H])O1)N)[H])N	Small ribosomal subunit inhibitor	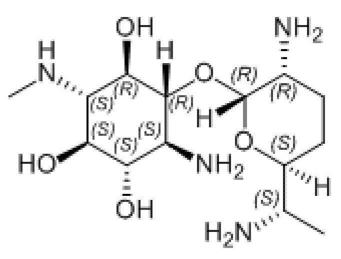	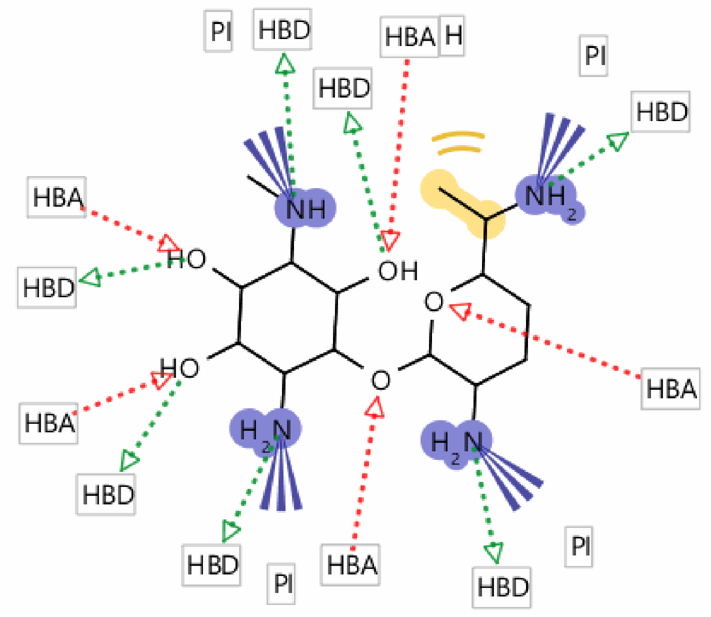
Fortimicin AQ	70952-84-0	VQQVKFYHAFUVPL-VJJPAKIZSA-N	InChI=1S/C16H34N4O5/c1-7(17)9-6-5-8(18)16(24-9)25-14-10(19)12(21)15(23-4)11(13(14)22)20(2)3/h7-16,21-22H,5-6,17-19H2,1-4H3/t7-,8+,9-,10-,11-,12-,13+,14+,15+,16+/m0/s1	C[C@@H]([C@@]1(CC[C@H]([C@](O[C@@]2([C@H]([C@@H]([C@@H]([C@](N(C)C)([H])[C@H]2O)OC)O)N)[H])([H])O1)N)[H])N	Small ribosomal subunit inhibitor	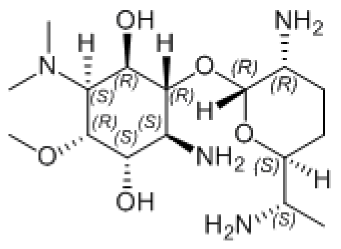	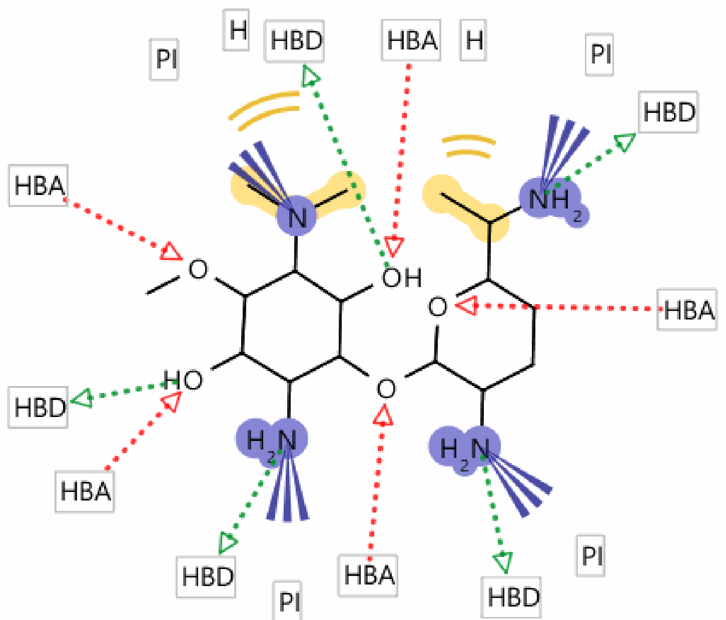
Fortimicin AS	66963-18-6	NVRQSAQBQUUGHN-NZOQUNFRSA-N	InChI=1S/C17H36N4O6/c1-8(18)10-5-4-9(19)17(26-10)27-15-11(20)13(23)16(25-3)12(14(15)24)21(2)6-7-22/h8-17,22-24H,4-7,18-20H2,1-3H3/t8-,9+,10-,11-,12-,13-,14+,15+,16+,17+/m0/s1	C[C@H](N)[C@]1([H])CC[C@@H](N)[C@](O1)(O[C@]2([H])[C@@H](N)[C@H](O)[C@H](OC)[C@]([C@H]2O)(N(CCO)C)[H])[H]	Small ribosomal subunit inhibitor	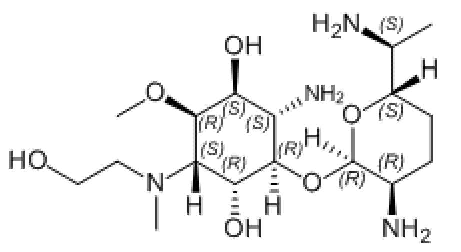	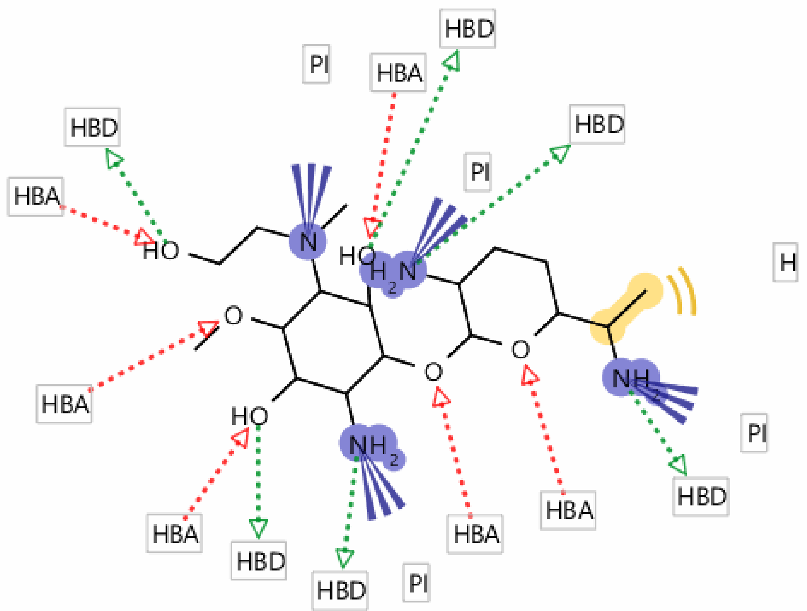
Fortimicin C	62874-51-5	VKGIGFQBOYWLHV-APGVDKLISA-N	InChI=1S/C18H36N6O7/c1-7(19)9-5-4-8(20)17(30-9)31-15-11(21)13(26)16(29-3)12(14(15)27)24(2)10(25)6-23-18(22)28/h7-9,11-17,26-27H,4-6,19-21H2,1-3H3,(H3,22,23,28)/t7-,8+,9-,11-,12-,13-,14+,15+,16+,17+/m0/s1	C[C@H](N)[C@]1([H])CC[C@@H](N)[C@](O1)(O[C@]2([H])[C@@H](N)[C@H](O)[C@H](OC)[C@]([C@H]2O)(N(C(CNC(N)=O)=O)C)[H])[H]	Small ribosomal subunit inhibitor	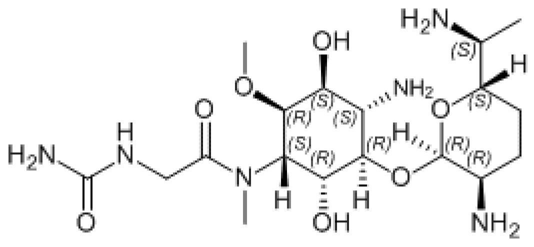	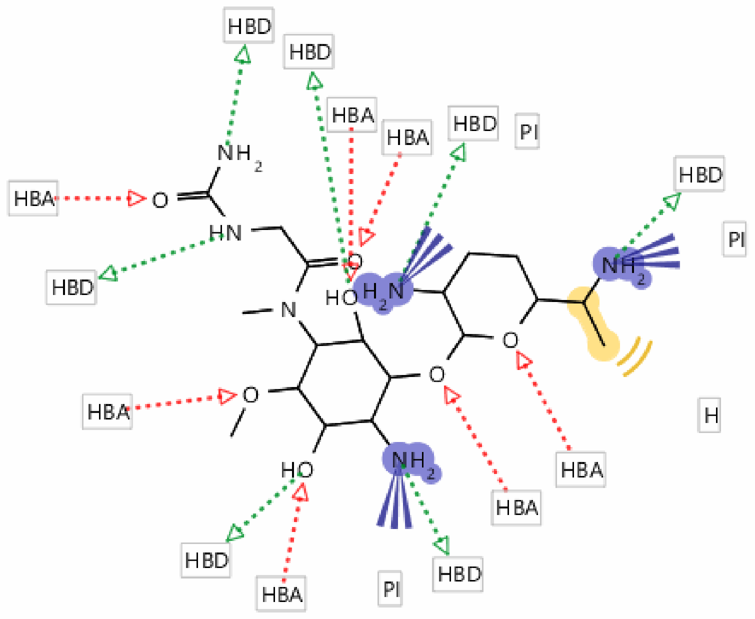
Fortimicin D	67330-20-5	VYLDQCCRMFPJHA-FXJXLBQDSA-N	InChI=1S/C16H33N5O6/c1-21(9(22)6-18)11-13(24)14(10(20)12(23)15(11)25-2)27-16-8(19)4-3-7(5-17)26-16/h7-8,10-16,23-24H,3-6,17-20H2,1-2H3/t7-,8+,10-,11-,12-,13+,14+,15+,16+/m0/s1	CN(C(CN)=O)[C@]1([C@H]([C@@]([H])([C@H]([C@@H]([C@@H]1OC)O)N)O[C@@]2([C@@H](CC[C@@H](CN)O2)N)[H])O)[H]	Small ribosomal subunit inhibitor	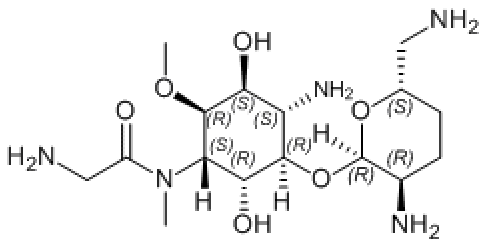	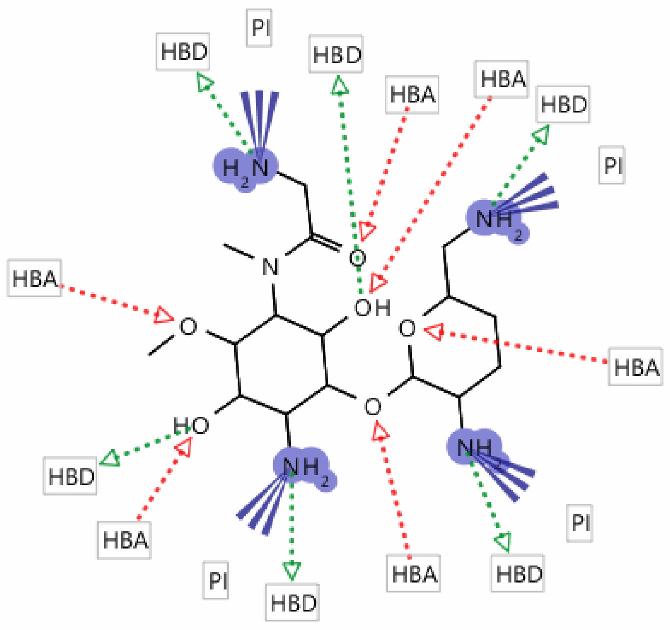
Fortimicin E (fortimicin KH)	71772-09-3	WFMQYKIRAVMXSU-QXCYNEMBSA-N	InChI=1S/C15H32N4O5/c1-6(16)8-5-4-7(17)15(23-8)24-13-9(18)11(20)14(22-3)10(19-2)12(13)21/h6-15,19-21H,4-5,16-18H2,1-3H3/t6-,7+,8-,9-,10+,11-,12+,13+,14-,15+/m0/s1	C[C@@H]([C@@]1(CC[C@H]([C@](O[C@@]2([C@H]([C@@H]([C@H]([C@@H]([C@H]2O)NC)OC)O)N)[H])([H])O1)N)[H])N	Small ribosomal subunit inhibitor	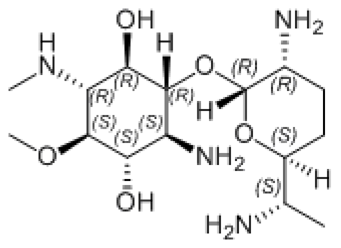	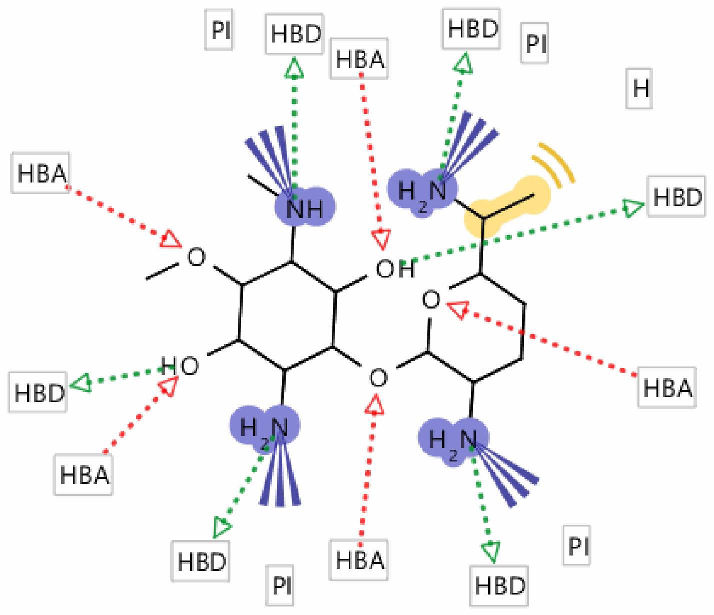

Common name	CAS Reg. No.	InChIKey	InChI	SMILES	Primary target	2D Structure	Pharmacophore[1]
Fortimicin KE	67330-21-6	SPZPNNYPYCPIPT-SDOPNMJFSA-N	InChI=1S/C14H30N4O5/c1-18-9-11(20)12(8(17)10(19)13(9)21-2)23-14-7(16)4-3-6(5-15)22-14/h6-14,18-20H,3-5,15-17H2,1-2H3/t6-,7+,8-,9-,10-,11+,12+,13+,14+/m0/s1	CN[C@H]1[C@H]([C@@]([H])([C@H]([C@@H]([C@@H]1OC)O)N)O[C@@]2([C@@H](CC[C@@H](CN)O2)N)[H])O	Small ribosomal subunit inhibitor	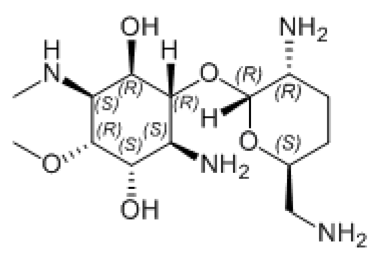	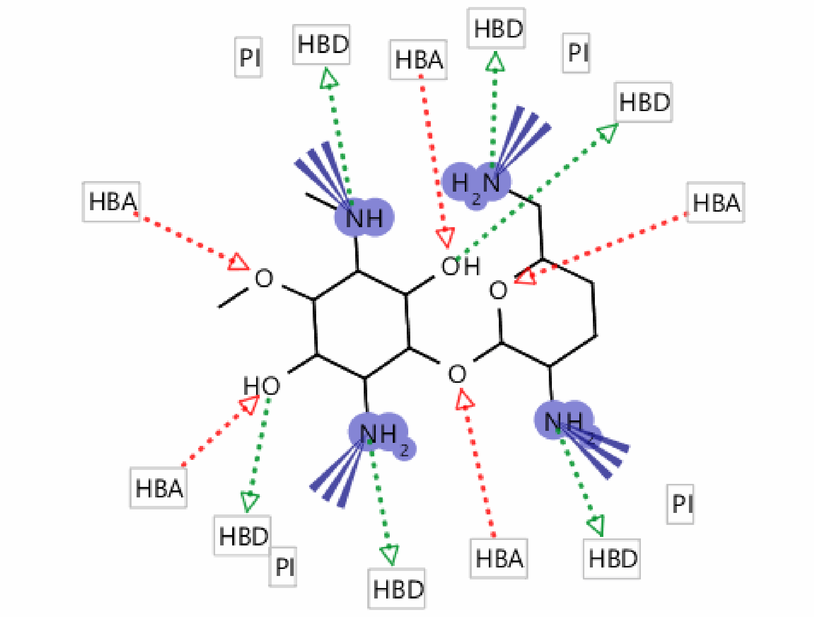
Fortimicin KF	71415-84-4	WCGAINYZGGJPMC-JOUGLVKWSA-N	InChI=1S/C14H28N4O5/c1-18-9-11(20)12(8(17)10(19)13(9)21-2)23-14-7(16)4-3-6(5-15)22-14/h3,7-14,18-20H,4-5,15-17H2,1-2H3/t7-,8+,9+,10+,11-,12-,13-,14-/m1/s1	CN[C@H]1[C@H]([C@@]([H])([C@H]([C@@H]([C@@H]1OC)O)N)O[C@@]2([C@@H](CC=C(CN)O2)N)[H])O	Small ribosomal subunit inhibitor	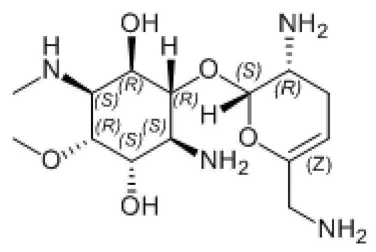	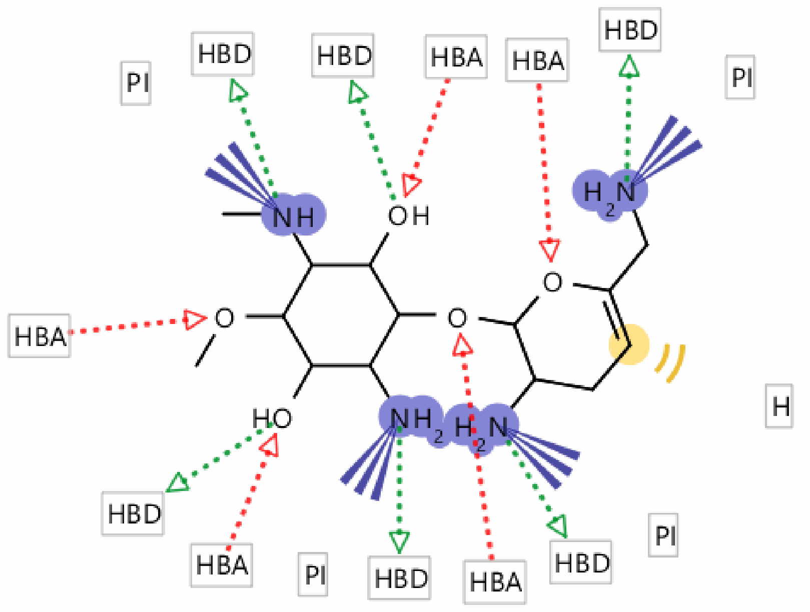
Fortimicin KG	71415-85-5	DCTIDBRLUYVPBC-XLENIPLDSA-N	InChI=1S/C15H30N4O5/c1-6(16)8-5-4-7(17)15(23-8)24-13-9(18)11(20)14(22-3)10(19-2)12(13)21/h5-7,9-15,19-21H,4,16-18H2,1-3H3/t6-,7+,9-,10-,11-,12+,13+,14+,15+/m0/s1	C[C@@H](C1=CC[C@H]([C@](O[C@@]2([C@H]([C@@H]([C@@H]([C@H]([C@H]2O)NC)OC)O)N)[H])([H])O1)N)N	Small ribosomal subunit inhibitor	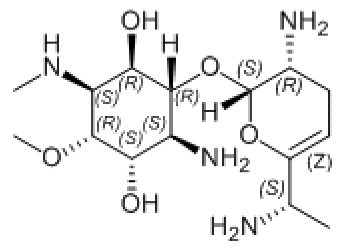	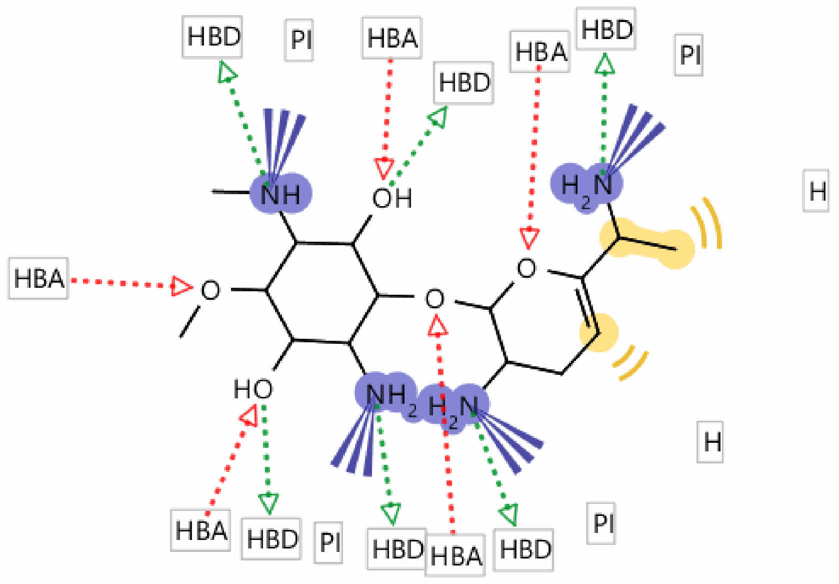
Fortimicin KL$_1$		BYPFOZNNHPOWJK-IXMAVWSQSA-N	InChI=1S/C13H28N4O7/c1-17-6-10(21)8(19)4(15)12(11(6)22)24-13-5(16)9(20)7(18)3(2-14)23-13/h3-13,17-22H,2,14-16H2,1H3/t3-,4+,5-,6+,7-,8+,9-,10+,11-,12-,13-/m1/s1	CN[C@H]1[C@@H]([C@H]([C@@H]([C@]([H])([C@@H]1O)O[C@@]2([C@@H]([C@H]([C@@H]([C@@H](CN)O2)O)O)N)[H])N)O)O	Small ribosomal subunit inhibitor	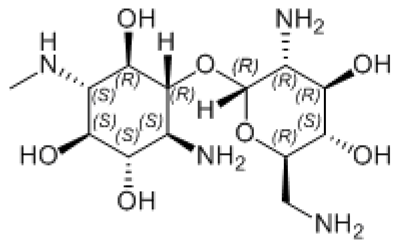	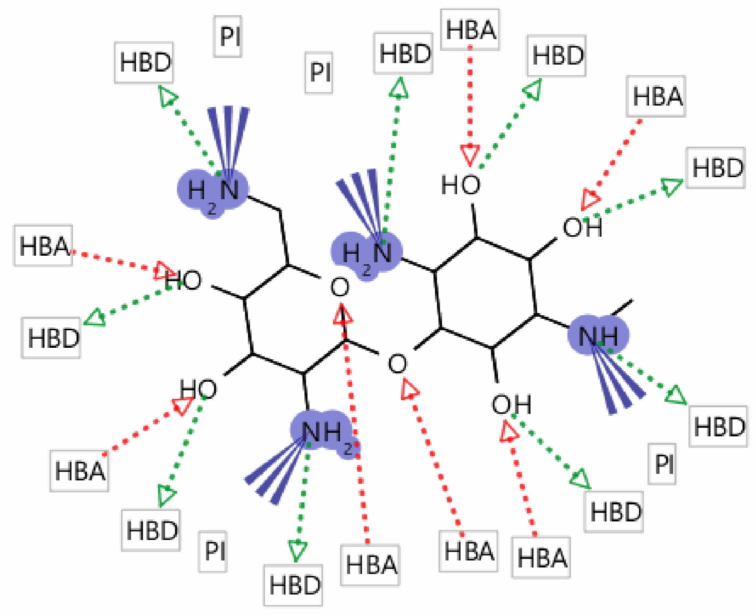
Fortimicin KR	122517-24-2	WFMQYKIRAVMXSU-GBKVUIFDSA-N	InChI=1S/C15H32N4O5/c1-6(16)8-5-4-7(17)15(23-8)24-13-9(18)11(20)14(22-3)10(19-2)12(13)21/h6-15,19-21H,4-5,16-18H2,1-3H3/t6-,7+,8-,9-,10+,11-,12+,13+,14+,15+/m0/s1	C[C@@H]([C@@]1(CC[C@H]([C@](O[C@@]2([C@H]([C@@H]([C@@H]([C@@H]([C@H]2O)NC)OC)O)N)[H])([H])O1)N)[H])N	Small ribosomal subunit inhibitor	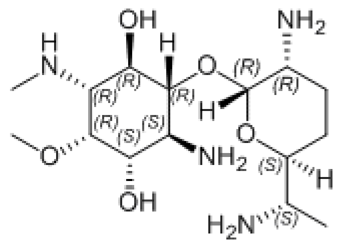	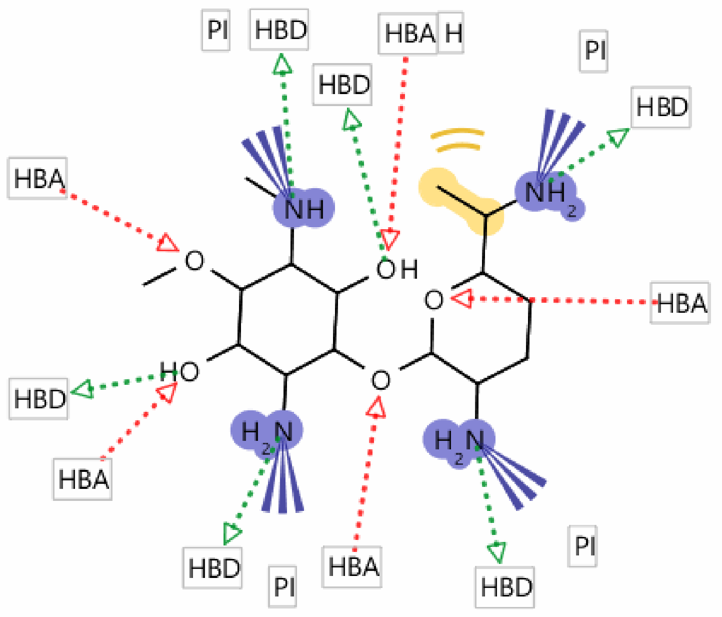
Framycetin (neomycin B)	119-04-0	PGBHMTALBVVCIT-VCIWKGPPSA-N	InChI=1S/C23H46N6O13/c24-2-7-13(32)15(34)10(28)21(37-7)40-18-6(27)1-5(26)12(31)20(18)42-23-17(36)19(9(4-30)39-23)41-22-11(29)16(35)14(33)8(3-25)38-22/h5-23,30-36H,1-4,24-29H2/t5-,6+,7-,8+,9-,10-,11-,12+,13-,14-,15-,16-,17-,18-,19-,20-,21-,22-,23+/m1/s1	NC[C@@H]1[C@H]([C@@H]([C@H]([C@]([H])(O1)O[C@@]2([C@H](C[C@H]([C@@H]([C@]2(O[C@H]3[C@@H]([C@@H]([C@@H](CO)O3)O[C@@]4([C@@H]([C@H]([C@@H]([C@H](CN)O4)O)O)N)[H])O)[H])O)N)N)[H])N)O)O	Small ribosomal subunit inhibitor	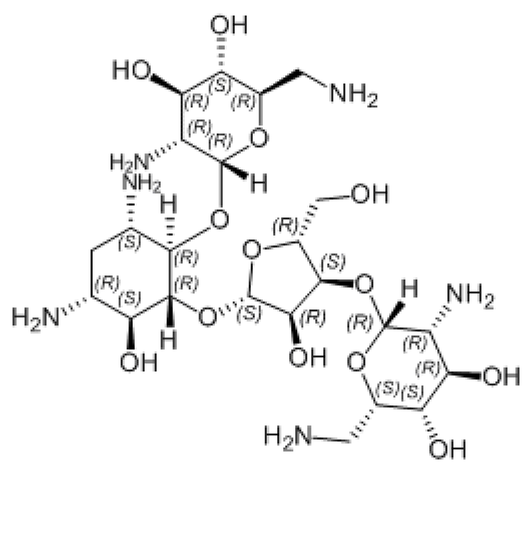	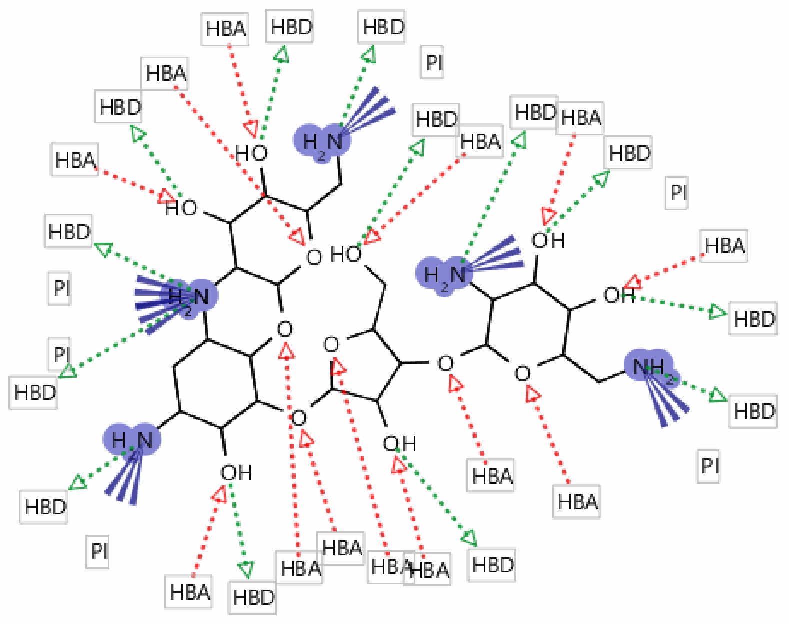

Common name	CAS Reg. No.	InChIKey	InChI	SMILES	Primary target	2D Structure	Pharmacophore[1]
Geneticin (gentamicin G-418)	49863-47-0	BRZYSWJRSDMWLG-DJWUNRQOSA-N	InChI=1S/C20H40N4O10/c1-6(25)14-11(27)10(26)9(23)18(32-14)33-15-7(21)4-8(22)16(12(15)28)34-19-13(29)17(24-3)20(2,30)5-31-19/h6-19,24-30H,4-5,21-23H2,1-3H3/t6-,7+,8-,9-,10-,11+,12+,13-,14-,15-,16+,17-,18-,19-,20+/m1/s1	C[C@H]([C@@]1([C@H]([C@@H]([C@H]([C@]([H])(O1)O[C@@]2([C@H](C[C@H]([C@@]([H])([C@H]2O)O[C@@]3([C@@H]([C@H]([C@](O)(C)CO3)NC)O)[H])N)N)[H])N)O)O)[H])O	Small ribosomal subunit inhibitor	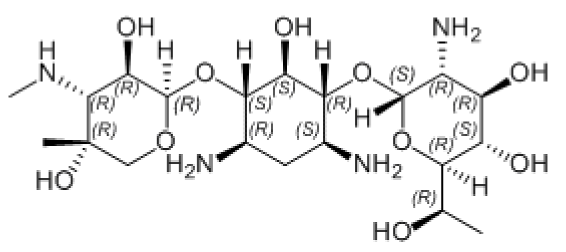	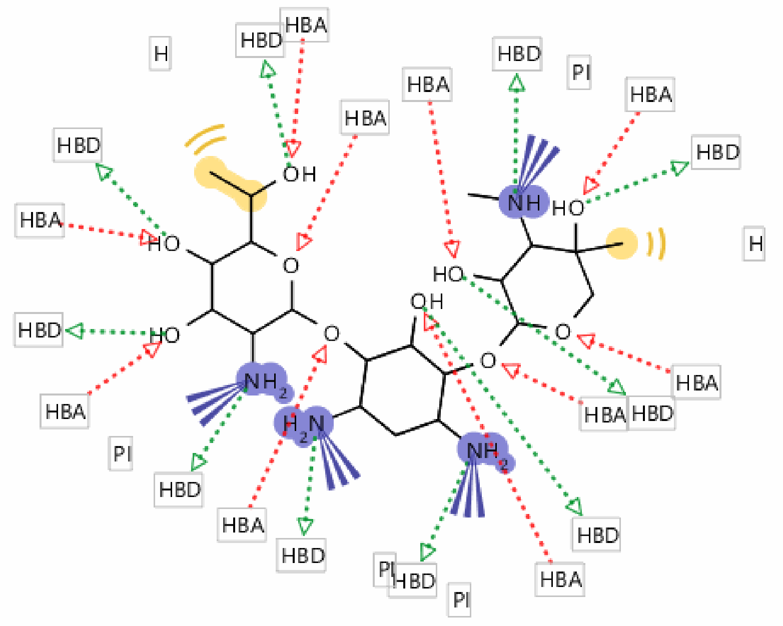
Gentamicin A	13291-74-2	LKKVGKXCMYHKSL-QVNYEEQUSA-N	InChI=1S/C18H36N4O10/c1-22-10-7(24)4-29-18(13(10)27)32-16-6(20)2-5(19)15(14(16)28)31-17-9(21)12(26)11(25)8(3-23)30-17/h5-18,22-28H,2-4,19-21H2,1H3/t5-,6+,7+,8+,9+,10-,11+,12+,13+,14-,15+,16-,17+,18+/m0/s1	CN[C@H]1[C@@H](CO[C@@]([H])([C@@H]1O)O[C@]2([C@@H](C[C@@H]([C@]([H])([C@@H]2O)O[C@@]3([C@@H]([C@H]([C@@H]([C@@H](CO)O3)O)O)N)[H])N)N)[H])O	Small ribosomal subunit inhibitor	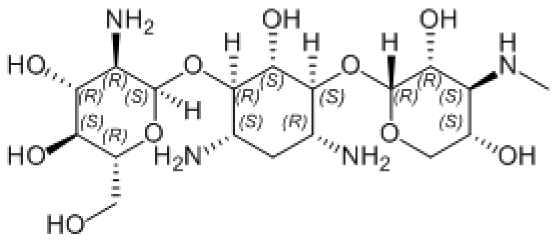	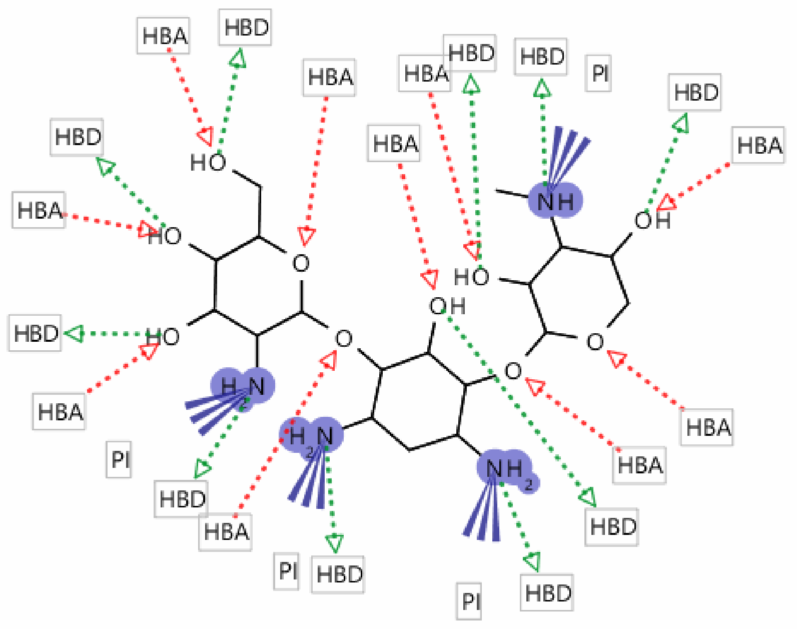
Gentamicin A_1	55925-13-8	LKKVGKXCMYHKSL-OWLCRXJJSA-N	InChI=1S/C18H36N4O10/c1-22-10-7(24)4-29-18(13(10)27)32-16-6(20)2-5(19)15(14(16)28)31-17-9(21)12(26)11(25)8(3-23)30-17/h5-18,22-28H,2-4,19-21H2,1H3/t5-,6+,7-,8+,9+,10-,11+,12+,13+,14-,15+,16-,17+,18+/m0/s1	CN[C@H]1[C@H](CO[C@@]([H])([C@@H]1O)O[C@]2([C@@H](C[C@@H]([C@]([H])([C@@H]2O)O[C@@]3([C@@H]([C@H]([C@@H]([C@@H](CO)O3)O)O)N)[H])N)N)[H])O	Small ribosomal subunit inhibitor	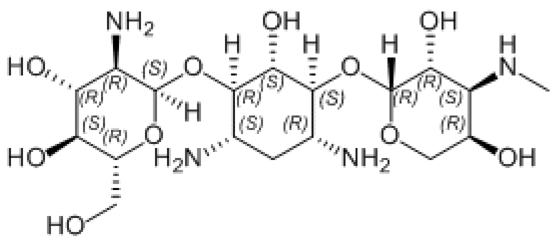	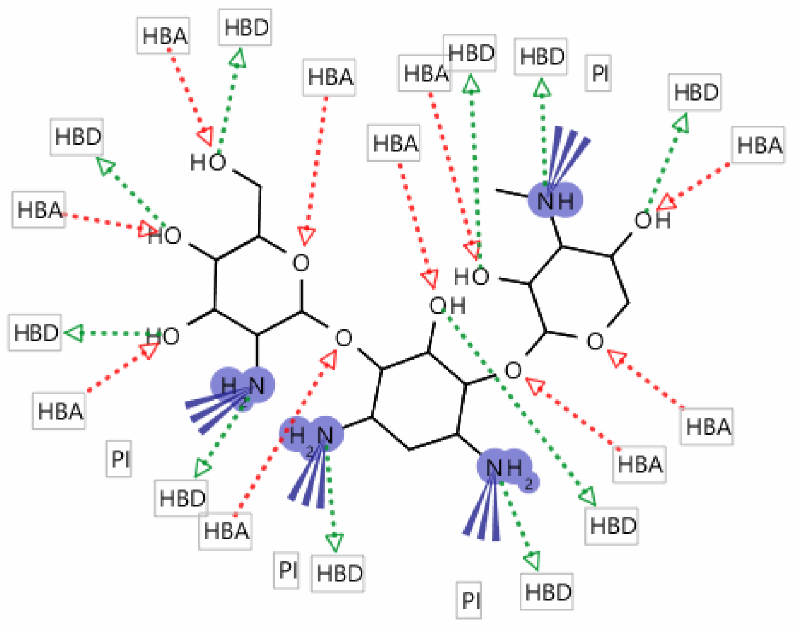
Gentamicin A_2	55715-66-7	BIVUTZYWJNTGDG-GTMVCPGISA-N	InChI=1S/C17H33N3O11/c18-4-1-5(19)15(31-17-12(26)9(23)6(22)3-28-17)13(27)14(4)30-16-8(20)11(25)10(24)7(2-21)29-16/h4-17,21-27H,1-3,18-20H2/t4-,5+,6+,7+,8+,9-,10+,11+,12+,13-,14+,15-,16+,17+/m0/s1	N[C@H]1C[C@H]([C@@]([H])([C@H]([C@@]1(O[C@@]2([C@@H]([C@H]([C@@H]([C@@H](CO)O2)O)O)N)[H])[H])O)O[C@@]3([C@@H]([C@H]([C@@H](CO3)O)O)O)[H])N	Small ribosomal subunit inhibitor	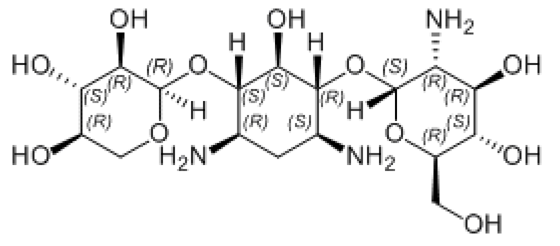	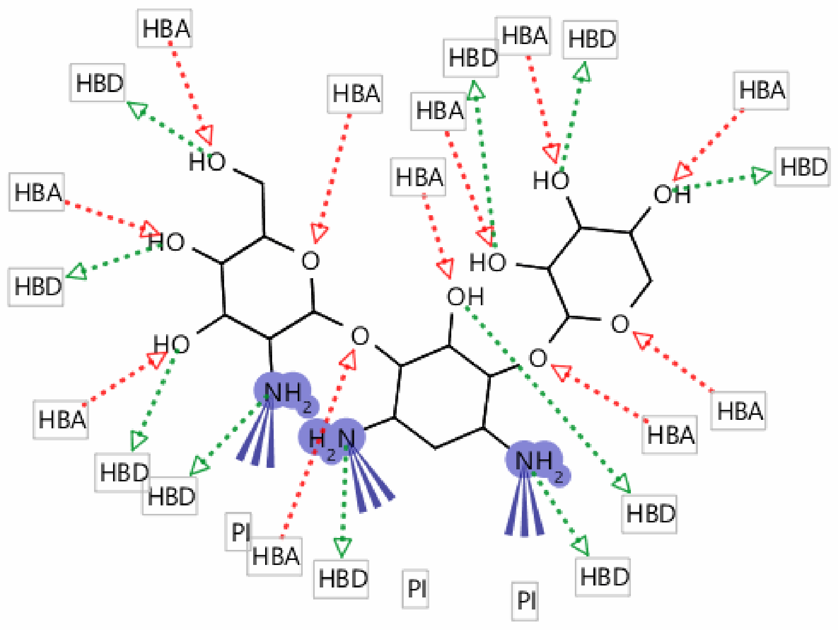
Gentamicin A_3	55715-67-8	BDTQHFBWYNCGHN-CUYCKOLISA-N	InChI=1S/C18H36N4O10/c1-22-9-7(23)4-29-17(11(9)25)31-15-5(20)2-6(21)16(14(15)28)32-18-13(27)12(26)10(24)8(3-19)30-18/h5-18,22-28H,2-4,19-21H2,1H3/t5-,6+,7+,8-,9+,10-,11-,12+,13-,14-,15+,16-,17-,18-/m1/s1	CN[C@H]1[C@H](CO[C@@]([H])([C@@H]1O)O[C@]2([C@@H](C[C@@H]([C@]([H])([C@@H]2O)O[C@@]3([C@@H]([C@H]([C@@H]([C@@H](CN)O3)O)O)O)[H])N)N)[H])O	Small ribosomal subunit inhibitor	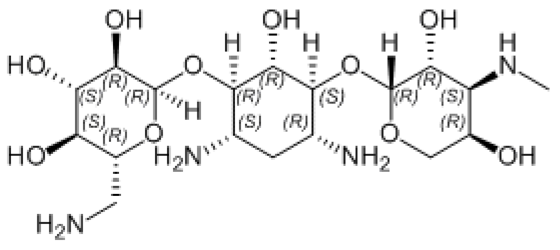	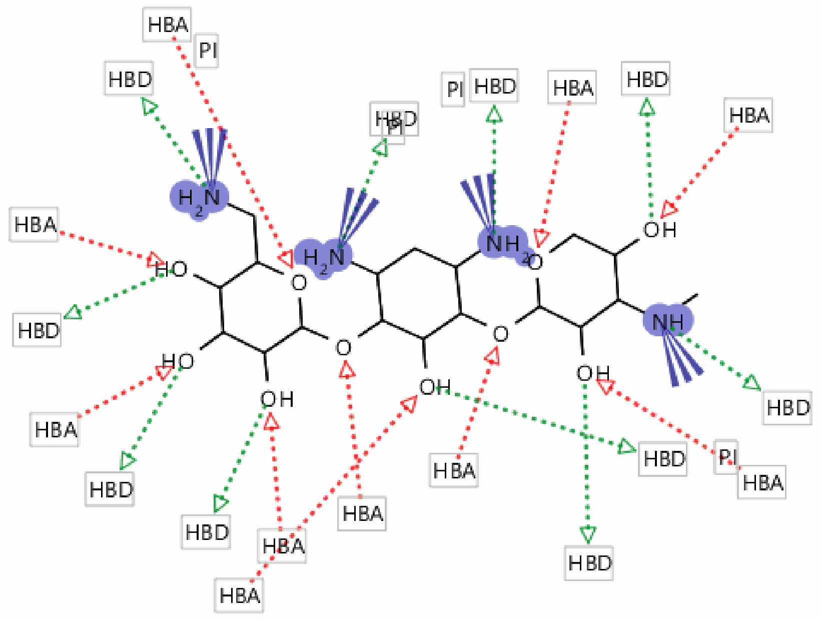
Gentamicin A_4	55904-33-1	LAVFZFGFAAANON-NTLRYLFOSA-N	InChI=1S/C19H36N4O11/c1-23(5-25)11-8(26)4-31-19(14(11)29)34-17-7(21)2-6(20)16(15(17)30)33-18-10(22)13(28)12(27)9(3-24)32-18/h5-19,24,26-30H,2-4,20-22H2,1H3/t6-,7+,8+,9+,10+,11-,12+,13+,14+,15-,16+,17-,18+,19+/m0/s1	CN(C=O)[C@]1([C@@H](CO[C@@]([H])([C@@H]1O)O[C@]2([C@@H](C[C@@H]([C@]([H])([C@@H]2O)O[C@@]3([C@@H]([C@H]([C@@H]([C@@H](CO)O3)O)O)N)[H])N)N)[H])O)[H]	Small ribosomal subunit inhibitor	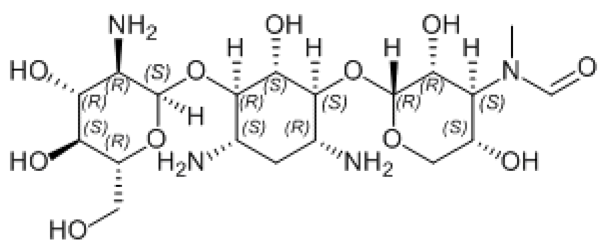	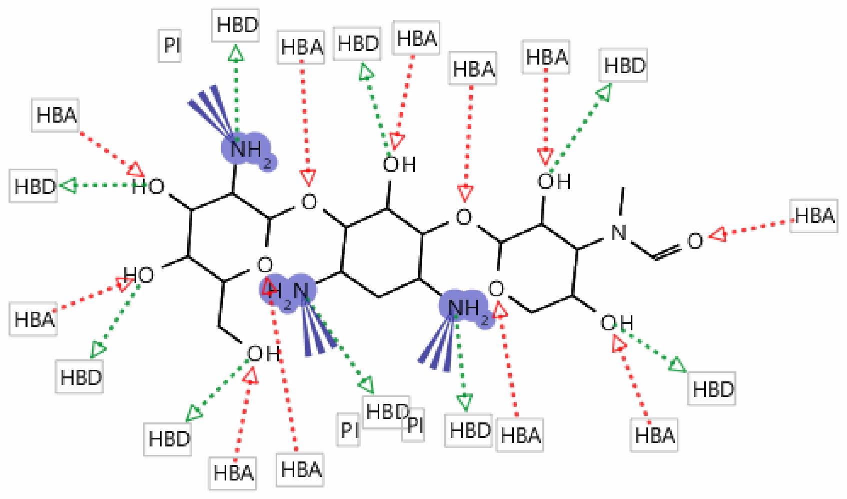

Common name	CAS Reg. No.	InChIKey	InChI	SMILES	Primary target	2D Structure	Pharmacophore[1]
Gentamicin B_1	36889-16-4	ABCLPPNEPBAKRL-FGMXNJDGSA-N	InChI=1S/C20H40N4O10/c1-6(21)14-10(26)9(25)11(27)19(32-14)34-16-8(23)4-7(22)15(12(16)28)33-18-13(29)17(24-3)20(2,30)5-31-18/h6-19,24-30H,4-5,21-23H2,1-3H3/t6-,7-,8+,9+,10+,11-,12-,13-,14-,15+,16-,17-,18-,19-,20+/m1/s1	C[C@@H](N)[C@]1([H])[C@@H](O)[C@H](O)[C@@H](O)[C@](O1)(O[C@]2([H])[C@@H](N)C[C@@H](N)[C@@]([C@H]2O)(O[C@]3([H])[C@H](O)[C@@H](NC)[C@](C)(CO3)O)[H])[H]	Small ribosomal subunit inhibitor	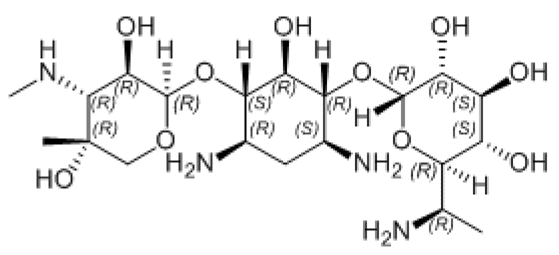	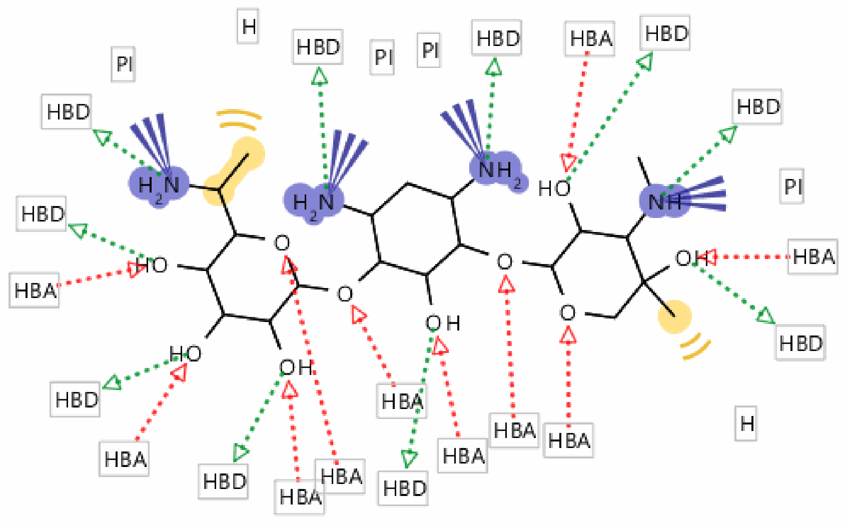
Gentamicin C_1	25876-10-2	CEAZRRDELHUEMR-CAMVTXANSA-N	InChI=1S/C21H43N5O7/c1-9(25-3)13-6-5-10(22)19(31-13)32-16-11(23)7-12(24)17(14(16)27)33-20-15(28)18(26-4)21(2,29)8-30-20/h9-20,25-29H,5-8,22-24H2,1-4H3/t9-,10-,11+,12-,13+,14+,15-,16-,17+,18-,19-,20-,21+/m1/s1	C[C@@H](NC)[C@]1([H])CC[C@@H](N)[C@](O1)(O[C@]2([H])[C@@H](N)C[C@@H](N)[C@@]([C@H]2O)(O[C@]3([H])[C@H](O)[C@@H](NC)[C@](C)(CO3)O)[H])[H]	Small ribosomal subunit inhibitor	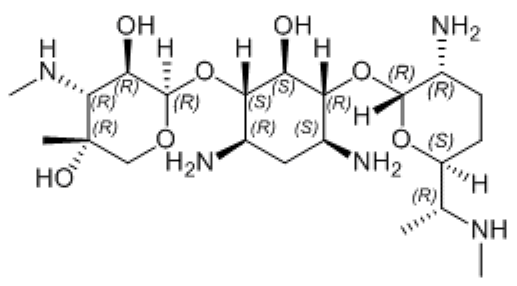	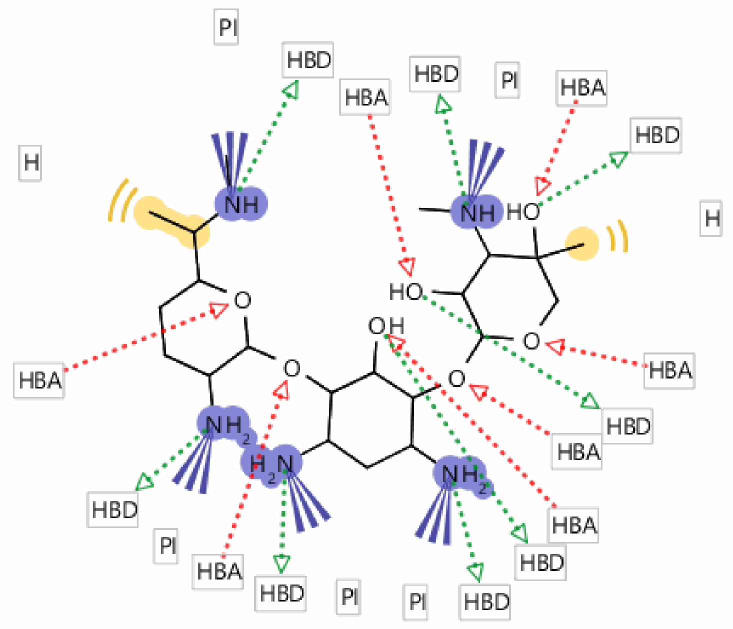
Gentamicin C_{1a}	26098-04-4	VEGXETMJINRLTH-BOZYPMBZSA-N	InChI=1S/C19H39N5O7/c1-19(27)7-28-18(13(26)16(19)24-2)31-15-11(23)5-10(22)14(12(15)25)30-17-9(21)4-3-8(6-20)29-17/h8-18,24-27H,3-7,20-23H2,1-2H3/t8-,9+,10-,11+,12-,13+,14+,15-,16+,17+,18+,19-/m0/s1	C[C@]1(O)CO[C@@]([C@H](O)[C@H]1NC)(O[C@@]2([H])[C@H](N)C[C@H](N)[C@]([C@@H]2O)(O[C@]3([H])[C@H](N)CC[C@H](O3)CN)[H])[H]	Small ribosomal subunit inhibitor	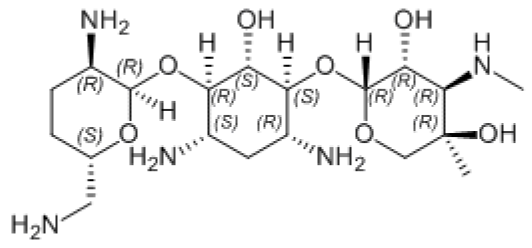	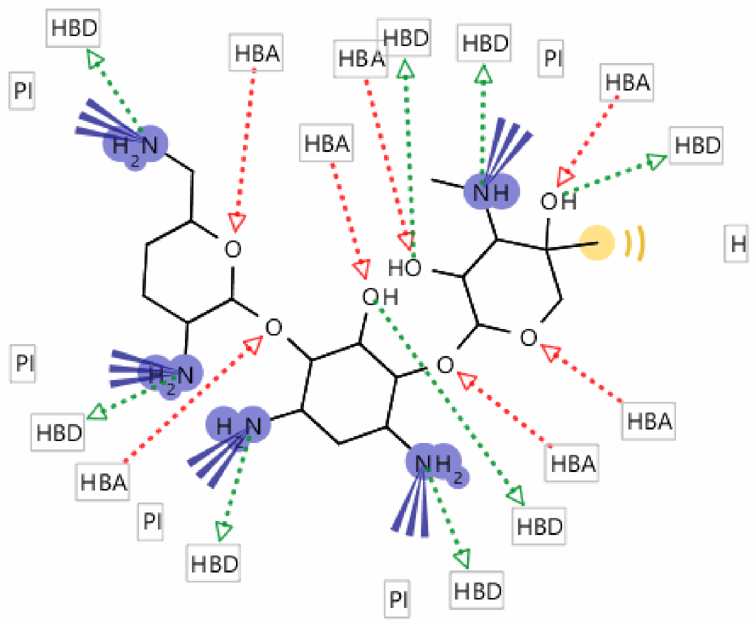
Gentamicin C_2	25876-11-3	XUFIWSHGXVLULG-IDLVJFIQSA-N	InChI=1S/C20H41N5O7/c1-8(21)12-5-4-9(22)18(30-12)31-15-10(23)6-11(24)16(13(15)26)32-19-14(27)17(25-3)20(2,28)7-29-19/h8-19,25-28H,4-7,21-24H2,1-3H3/t8-,9-,10+,11-,12+,13+,14-,15-,16+,17-,18-,19-,20+/m1/s1	C[C@H]([C@@]1(CC[C@H]([C@]([H])(O1)O[C@@]2([C@H](C[C@H]([C@@]([H])([C@H]2O)O[C@@]3([C@@H]([C@H]([C@](O)(C)CO3)NC)O)[H])N)N)[H])N)[H])N	Small ribosomal subunit inhibitor	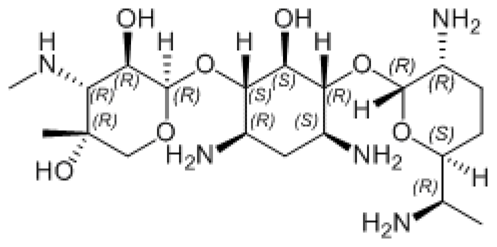	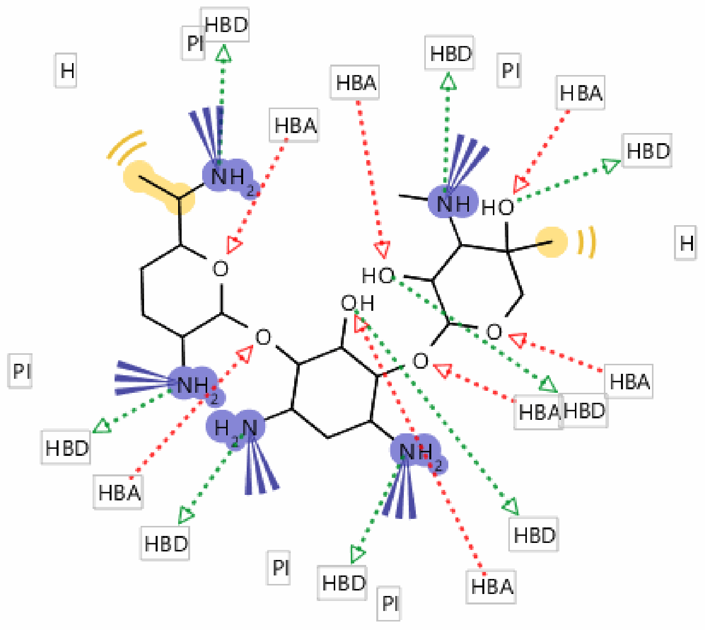
Gentamicin C_{2a}	59751-72-3	XUFIWSHGXVLULG-BSBKYKEKSA-N	InChI=1S/C20H41N5O7/c1-8(21)12-5-4-9(22)18(30-12)31-15-10(23)6-11(24)16(13(15)26)32-19-14(27)17(25-3)20(2,28)7-29-19/h8-19,25-28H,4-7,21-24H2,1-3H3/t8-,9+,10-,11+,12-,13-,14+,15+,16-,17+,18+,19+,20-/m0/s1	C[C@@H]([C@@]1(CC[C@H]([C@]([H])(O1)O[C@@]2([C@H](C[C@H]([C@@]([H])([C@H]2O)O[C@@]3([C@@H]([C@H]([C@](O)(C)CO3)NC)O)[H])N)N)[H])N)[H])N	Small ribosomal subunit inhibitor	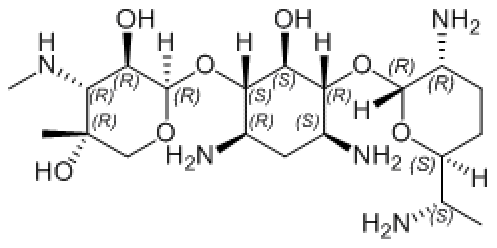	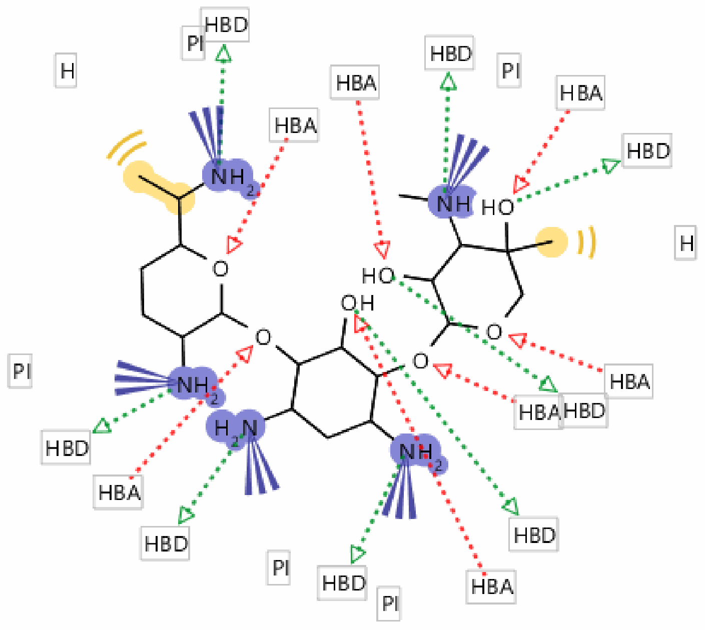
Gentamicin X_2	36889-17-5	HFLKNINDVFJPQT-ZFAMMYHGSA-N	InChI=1S/C19H38N4O10/c1-19(29)5-30-18(13(28)16(19)23-2)33-15-7(21)3-6(20)14(12(15)27)32-17-9(22)11(26)10(25)8(4-24)31-17/h6-18,23-29H,3-5,20-22H2,1-2H3/t6-,7+,8+,9+,10+,11+,12-,13+,14+,15-,16+,17+,18+,19-/m0/s1	C[C@@]1(CO[C@@]([H])([C@@H]([C@H]1NC)O)O[C@]2([C@@H](C[C@@H]([C@]([H])([C@@H]2O)O[C@@]3([C@@H]([C@H]([C@@H]([C@@H](CO)O3)O)O)N)[H])N)N)[H])O	Small ribosomal subunit inhibitor	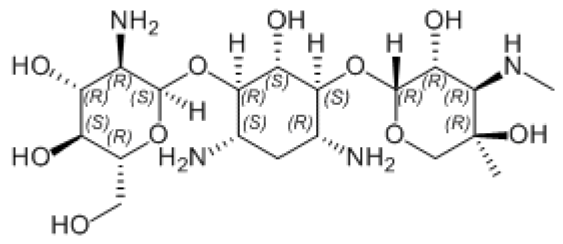	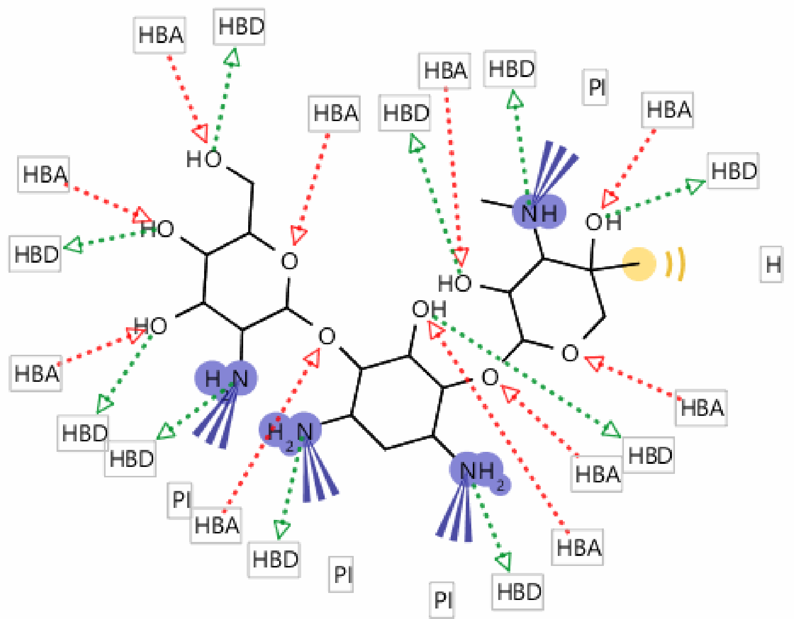

Common name	CAS Reg. No.	InChIKey	InChI	SMILES	Primary target	2D Structure	Pharmacophore[1]
Hybrimycin A_1	22332-07-6	AQNVGROASUJDKE-PBZLBJBQSA-N	InChI=1S/C23H46N6O14/c24-1-4-11(31)14(34)9(28)21(38-4)41-18-6(3-30)40-23(17(18)37)43-20-16(36)7(26)13(33)8(27)19(20)42-22-10(29)15(35)12(32)5(2-25)39-22/h4-23,30-37H,1-3,24-29H2/t4-,5+,6+,7+,8-,9+,10+,11+,12+,13+,14+,15+,16-,17+,18+,19+,20+,21+,22+,23-/m0/s1	NC[C@H]1[C@H]([C@@H]([C@H]([C@]([H])(O1)O[C@H]2[C@H]([C@@H](O[C@@H]2CO)O[C@@]3([C@H]([C@@H]([C@H]([C@@H]([C@]3(O[C@@]4([C@@H]([C@H]([C@@H]([C@@H](CN)O4)O)O)N)[H])[H])N)O)N)O)[H])O)N)O)O	Small ribosomal subunit inhibitor	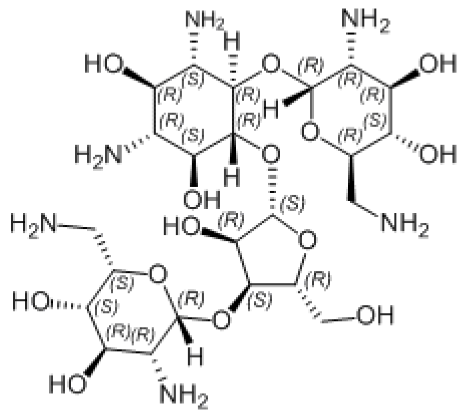	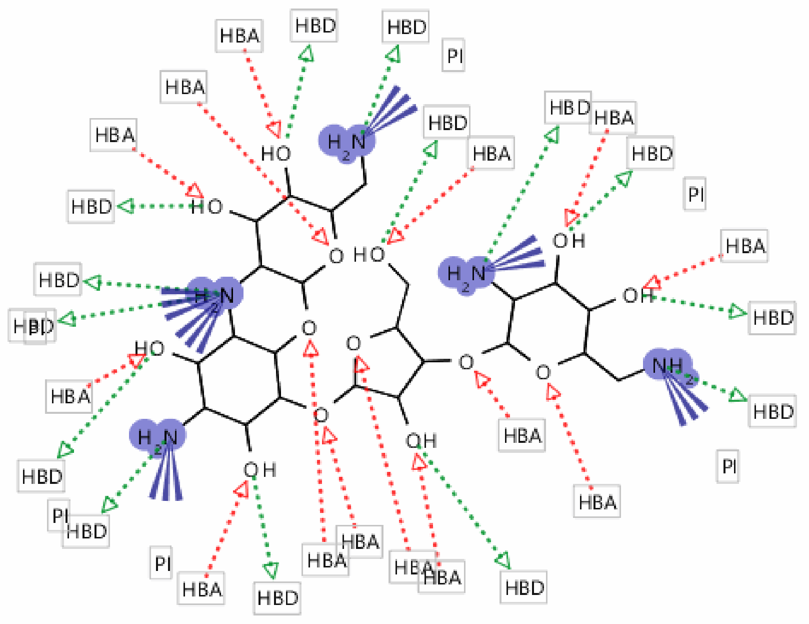
Hybrimycin A_2	22400-60-8	AQNVGROASUJDKE-DVUGITRJSA-N	InChI=1S/C23H46N6O14/c24-1-4-11(31)14(34)9(28)21(38-4)41-18-6(3-30)40-23(17(18)37)43-20-16(36)7(26)13(33)8(27)19(20)42-22-10(29)15(35)12(32)5(2-25)39-22/h4-23,30-37H,1-3,24-29H2/t4-,5-,6-,7-,8+,9-,10-,11-,12-,13-,14-,15-,16+,17-,18-,19-,20-,21-,22-,23+/m1/s1	NC[C@@H]1[C@H]([C@@H]([C@H]([C@]([H])(O1)O[C@H]2[C@H]([C@@H](O[C@@H]2CO)O[C@@]3([C@H]([C@@H]([C@H]([C@@H]([C@]3(O[C@@]4([C@@H]([C@H]([C@@H]([C@@H](CN)O4)O)O)N)[H])[H])N)O)N)O)[H])O)N)O)O	Small ribosomal subunit inhibitor	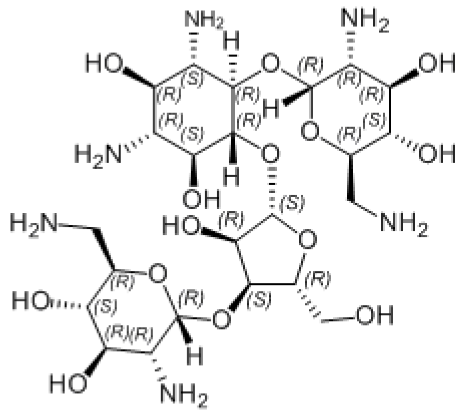	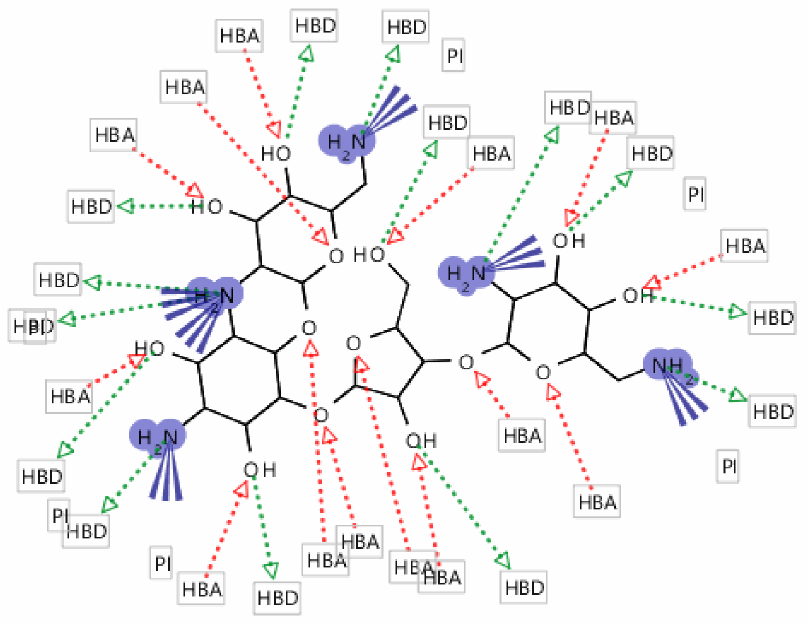
Hybrimycin B_1	22332-08-7	AQNVGROASUJDKE-XKPKFMCTSA-N	InChI=1S/C23H46N6O14/c24-1-4-11(31)14(34)9(28)21(38-4)41-18-6(3-30)40-23(17(18)37)43-20-16(36)7(26)13(33)8(27)19(20)42-22-10(29)15(35)12(32)5(2-25)39-22/h4-23,30-37H,1-3,24-29H2/t4-,5+,6+,7+,8-,9+,10+,11+,12+,13-,14+,15+,16-,17+,18+,19+,20+,21+,22+,23-/m0/s1	NC[C@H]1[C@H]([C@@H]([C@H]([C@]([H])(O1)O[C@H]2[C@H]([C@@H](O[C@@H]2CO)O[C@@]3([C@H]([C@@H]([C@@H]([C@@H]([C@]3(O[C@@]4([C@@H]([C@H]([C@@H]([C@@H](CN)O4)O)O)N)[H])[H])N)O)N)O)[H])O)N)O)O	Small ribosomal subunit inhibitor	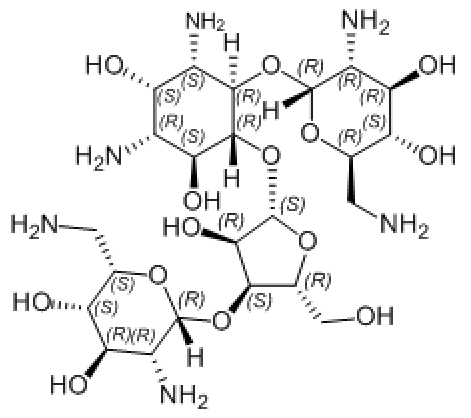	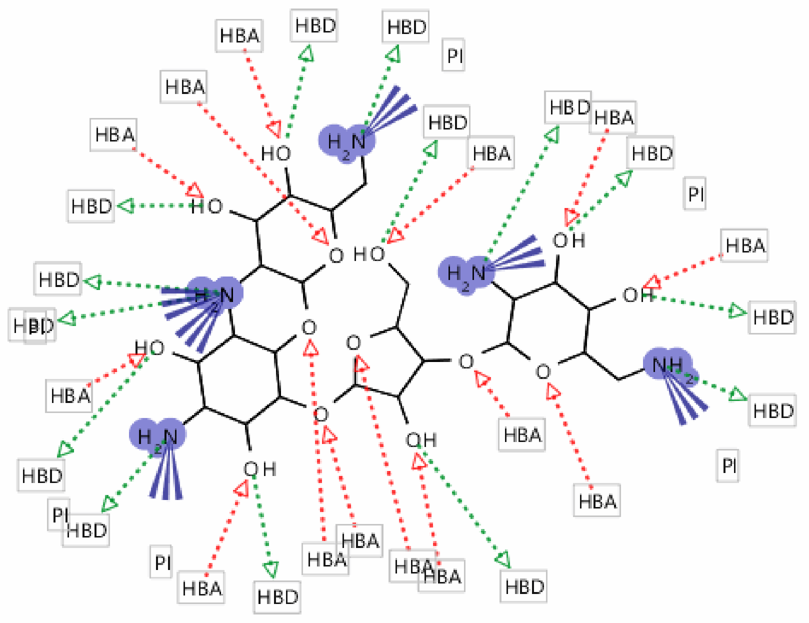
Hybrimycin B_2	27425-78-1	AQNVGROASUJDKE-UUWSLUEDSA-N	InChI=1S/C23H46N6O14/c24-1-4-11(31)14(34)9(28)21(38-4)41-18-6(3-30)40-23(17(18)37)43-20-16(36)7(26)13(33)8(27)19(20)42-22-10(29)15(35)12(32)5(2-25)39-22/h4-23,30-37H,1-3,24-29H2/t4-,5-,6-,7-,8+,9-,10-,11-,12-,13+,14-,15-,16+,17-,18-,19-,20-,21-,22-,23+/m1/s1	NC[C@@H]1[C@H]([C@@H]([C@H]([C@]([H])(O1)O[C@H]2[C@H]([C@@H](O[C@@H]2CO)O[C@@]3([C@H]([C@@H]([C@@H]([C@@H]([C@]3(O[C@@]4([C@@H]([C@H]([C@@H]([C@@H](CN)O4)O)O)N)[H])[H])N)O)N)O)[H])O)N)O)O	Small ribosomal subunit inhibitor	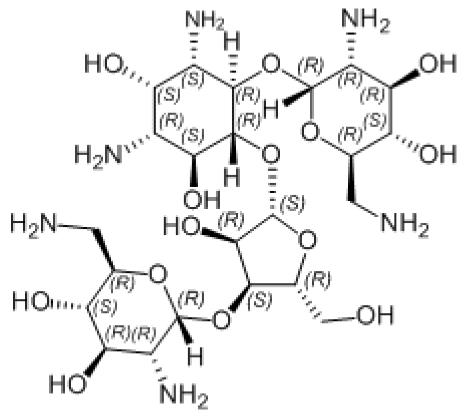	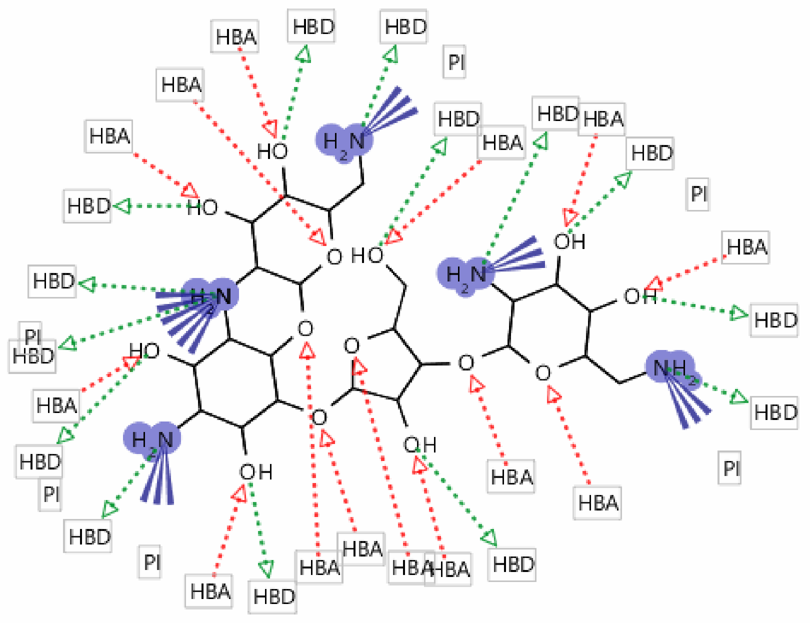
Hybrimycin C_1	38965-79-6	HVKBOZJCNGRWQJ-PBZLBJBQSA-N	InChI=1S/C23H45N5O15/c24-1-4-11(31)14(34)9(27)21(38-4)41-18-6(3-30)40-23(17(18)37)43-20-16(36)7(25)13(33)8(26)19(20)42-22-10(28)15(35)12(32)5(2-29)39-22/h4-23,29-37H,1-3,24-28H2/t4-,5+,6+,7+,8-,9+,10+,11+,12+,13+,14+,15+,16-,17+,18+,19+,20+,21+,22+,23-/m0/s1	NC[C@H]1[C@H]([C@@H]([C@H]([C@]([H])(O1)O[C@H]2[C@H]([C@@H](O[C@@H]2CO)O[C@@]3([C@H]([C@@H]([C@H]([C@@H]([C@]3(O[C@@]4([C@@H]([C@H]([C@@H]([C@@H](CO)O4)O)O)N)[H])[H])N)O)N)O)[H])O)N)O)O	Small ribosomal subunit inhibitor	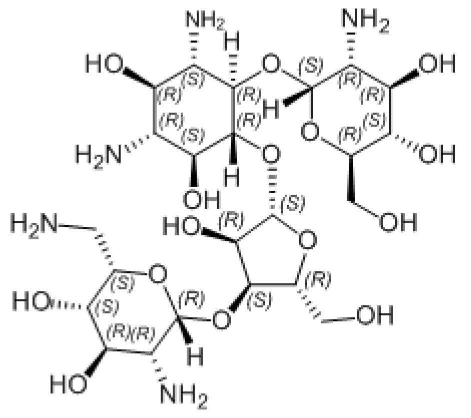	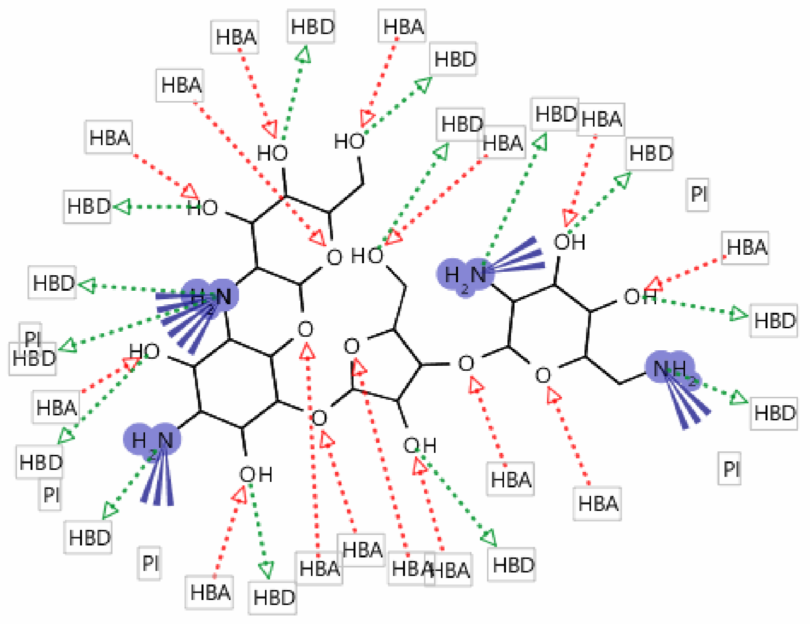
Hybrimycin C_2	39004-63-2	HVKBOZJCNGRWQJ-DVUGITRJSA-N	InChI=1S/C23H45N5O15/c24-1-4-11(31)14(34)9(27)21(38-4)41-18-6(3-30)40-23(17(18)37)43-20-16(36)7(25)13(33)8(26)19(20)42-22-10(28)15(35)12(32)5(2-29)39-22/h4-23,29-37H,1-3,24-28H2/t4-,5-,6-,7-,8+,9-,10-,11-,12-,13-,14-,15-,16+,17-,18-,19-,20-,21-,22-,23+/m1/s1	NC[C@@H]1[C@H]([C@@H]([C@H]([C@]([H])(O1)O[C@H]2[C@H]([C@@H](O[C@@H]2CO)O[C@@]3([C@H]([C@@H]([C@H]([C@@H]([C@]3(O[C@@]4([C@@H]([C@H]([C@@H]([C@@H](CO)O4)O)O)N)[H])[H])N)O)N)O)[H])O)N)O)O	Small ribosomal subunit inhibitor	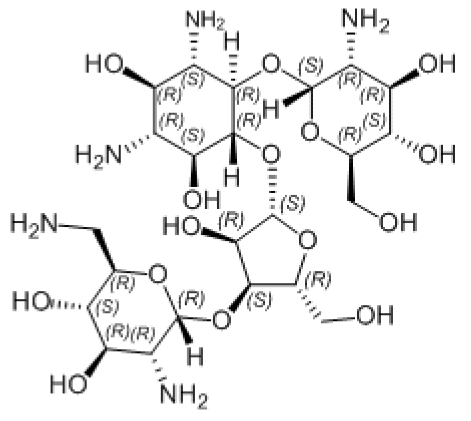	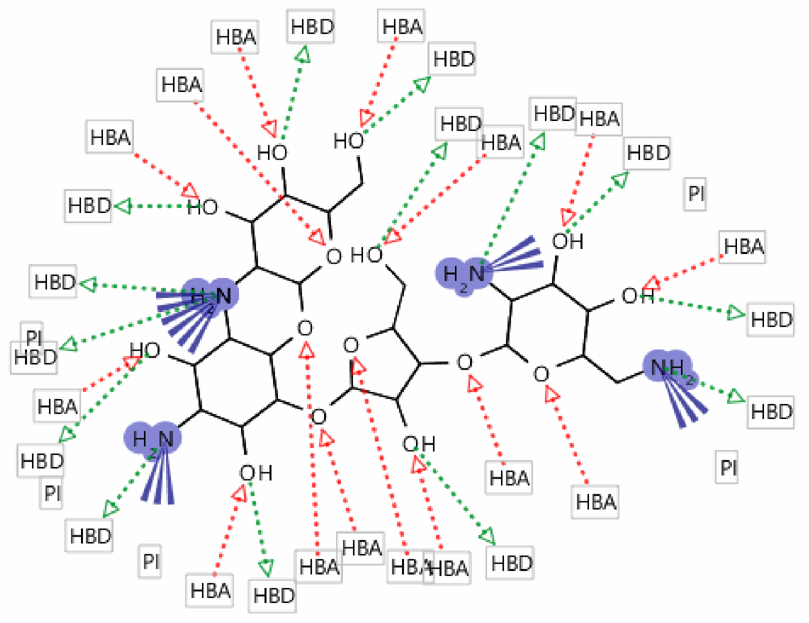

Common name	CAS Reg. No.	InChIKey	InChI	SMILES	Primary target	2D Structure	Pharmacophore[1]
Hybrimycin D	52198-61-5	OVRZSYBFMXXXDW-SKMGLNHUSA-N	InChI=1S/C18H35N3O13/c19-5-8(24)3(1-22)31-17(11(5)27)33-15-6(20)10(26)7(21)16(14(15)30)34-18-13(29)12(28)9(25)4(2-23)32-18/h3-18,22-30H,1-2,19-21H2/t3-,4-,5+,6-,7+,8-,9-,10+,11-,12+,13-,14-,15+,16-,17-,18-/m1/s1	N[C@@H]1[C@H]([C@]([H])(O[C@H](CO)[C@H]1O)O[C@]2([C@@H]([C@@H]([C@@H]([C@]([H])([C@@H]2O)O[C@@]3([C@@H]([C@H]([C@@H]([C@@H](CO)O3)O)O)O)[H])N)O)N)[H])O	Small ribosomal subunit inhibitor	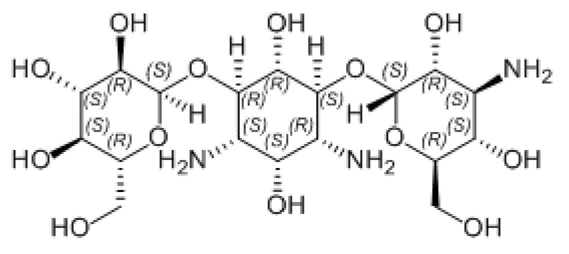	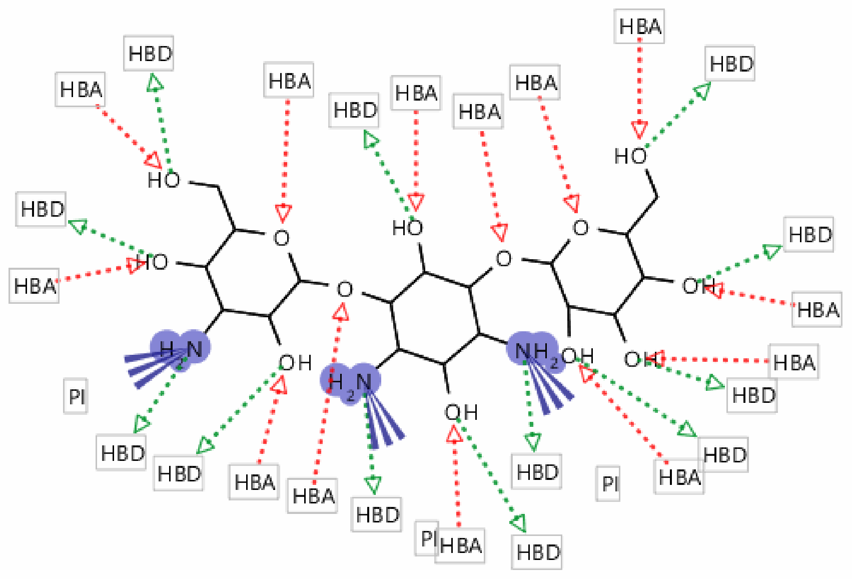
Hygromycin B (A 396II)	31282-04-9	YELMTJULBWLPRL-FYIYYDQBSA-N	InChI=1S/2C20H37N3O13/c2*1-23-7-2-5(21)9(26)15(10(7)27)33-19-17-16(11(28)8(4-25)32-19)35-20(36-17)18(31)13(30)12(29)14(34-20)6(22)3-24/h2*5-19,23-31H,2-4,21-22H2,1H3/t5-,6+,7+,8-,9+,10-,11+,12-,13+,14-,15-,16+,17+,18-,19+,20+;5-,6+,7+,8-,9+,10-,11+,12-,13+,14-,15-,16+,17+,18-,19+,20-/m11/s1	CN[C@H]1C[C@H]([C@@H]([C@]([H])([C@@H]1O)O[C@]2([C@@]3([H])O[C@]4([C@@H]([C@H]([C@H]([C@@]([H])([C@H](CO)N)O4)O)O)O)O[C@]3([C@H]([C@@H](CO)O2)O)[H])[H])O)N.CN[C@H]5C[C@H]([C@@H]([C@]([H])([C@@H]5O)O[C@]6([C@@]7([H])O[C@@]8([C@@H]([C@H]([C@H]([C@@]([H])([C@H](CO)N)O8)O)O)O)O[C@]7([C@H]([C@@H](CO)O6)O)[H])[H])O)N	Small ribosomal subunit inhibitor	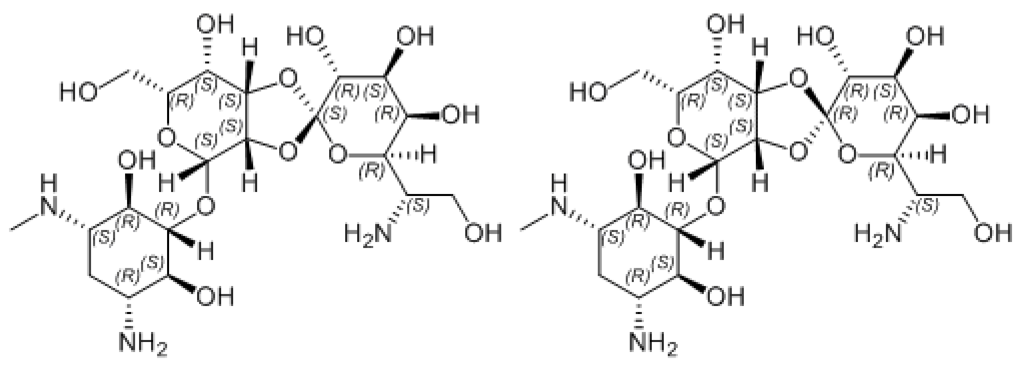	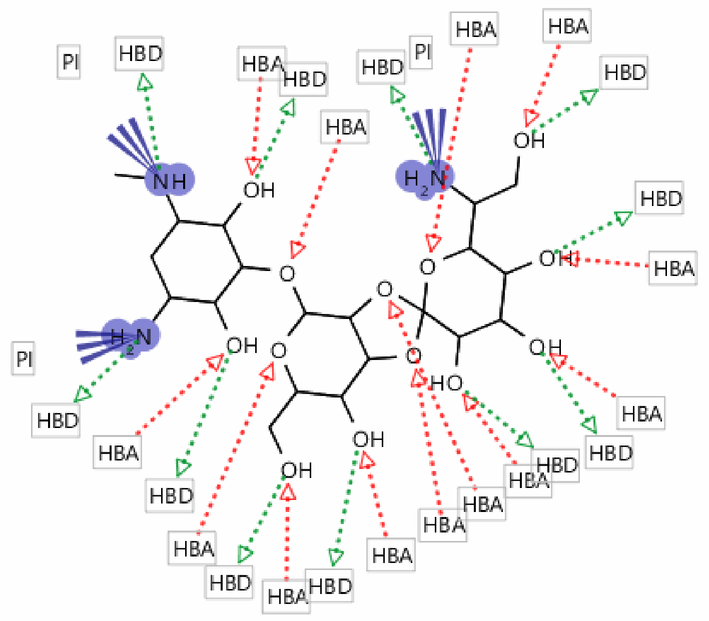
Isepamicin	58152-03-7	UDIIBEDMEYAVNG-ZKFPOVNWSA-N	InChI=1S/C22H43N5O12/c1-22(35)6-36-20(15(33)18(22)26-2)39-17-8(27-19(34)9(28)4-23)3-7(25)16(14(17)32)38-21-13(31)12(30)11(29)10(5-24)37-21/h7-18,20-21,26,28-33,35H,3-6,23-25H2,1-2H3,(H,27,34)/t7-,8+,9-,10+,11+,12-,13+,14-,15+,16+,17-,18+,20+,21+,22-/m0/s1	C[C@@]1(CO[C@@]([H])([C@@H]([C@H]1NC)O)O[C@]2([C@@]([H])(C[C@@H]([C@]([H])([C@@H]2O)O[C@@]3([C@H](O)[C@H]([C@@H]([C@@H](CN)O3)O)O)[H])N)NC([C@H](CN)O)=O)[H])O	Small ribosomal subunit inhibitor	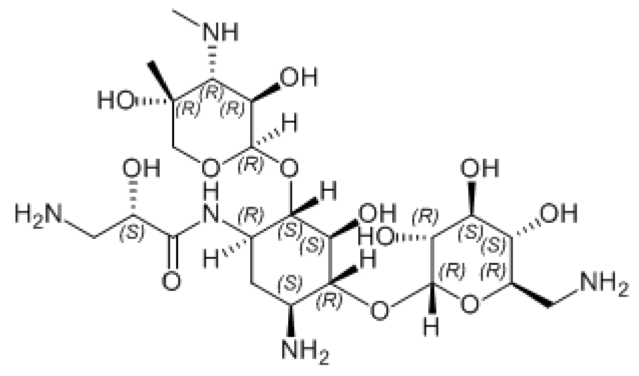	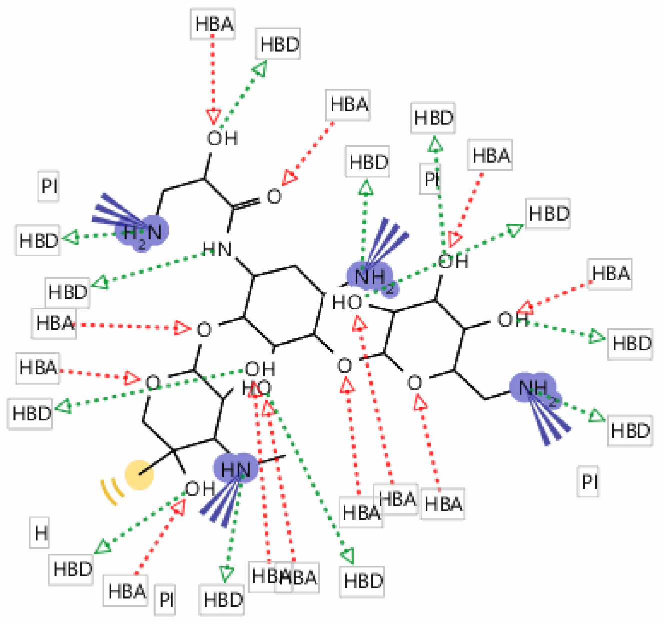
Istamycin A (sannamycin A)	72503-79-8	NEFDRWXEVITQMN-JWYRXTSNSA-N	InChI=1S/C17H35N5O5/c1-21-8-9-4-5-10(19)17(26-9)27-16-11(20)6-12(25-3)14(15(16)24)22(2)13(23)7-18/h9-12,14-17,21,24H,4-8,18-20H2,1-3H3/t9-,10+,11-,12-,14+,15+,16+,17+/m0/s1	CNC[C@@]1(CC[C@H]([C@]([H])(O1)O[C@@]2([C@H](C[C@@H]([C@]([H])([C@H]2O)N(C)C(CN)=O)OC)N)[H])N)[H]	Small ribosomal subunit inhibitor	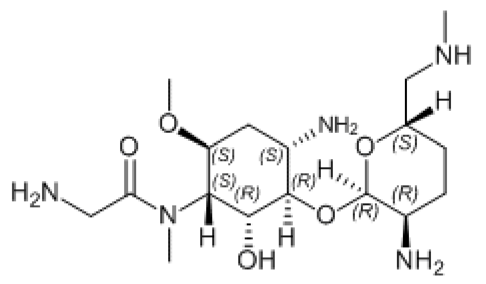	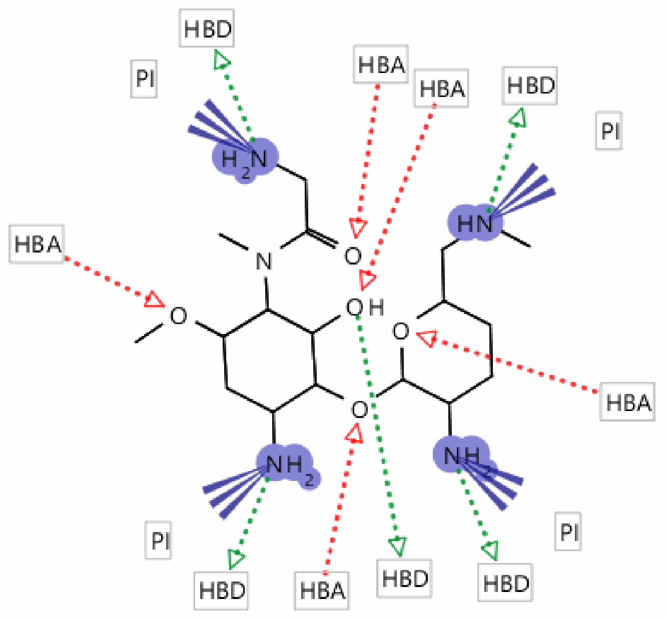
Istamycin A$_0$ (sannamycin B)	72503-80-1	GKYYNFPFPFRFFN-LHPGNBRISA-N	InChI=1S/C15H32N4O4/c1-18-7-8-4-5-9(16)15(22-8)23-14-10(17)6-11(21-3)12(19-2)13(14)20/h8-15,18-20H,4-7,16-17H2,1-3H3/t8-,9+,10-,11-,12+,13+,14+,15+/m0/s1	CNC[C@@]1(CC[C@H]([C@]([H])(O1)O[C@@]2([C@H](C[C@@H]([C@H]([C@H]2O)NC)OC)N)[H])N)[H]	Small ribosomal subunit inhibitor	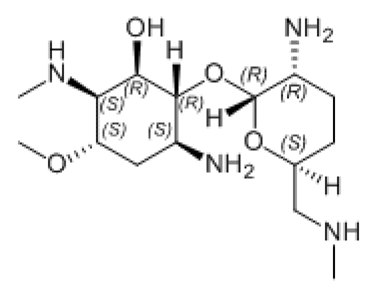	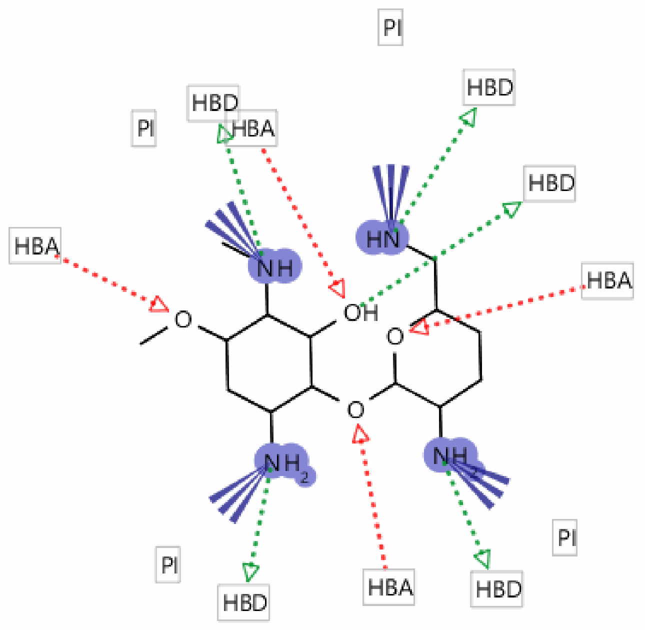

Common name	CAS Reg. No.	InChIKey	InChI	SMILES	Primary target	2D Structure	Pharmacophore[1]
Istamycin A₁	83728-96-5	IHPJPQIRNONWGH-GTSDWETFSA-N	InChI=1S/C18H35N5O6/c1-21-7-10-4-5-11(19)18(28-10)29-17-12(20)6-13(27-3)15(16(17)26)23(2)14(25)8-22-9-24/h9-13,15-18,21,26H,4-8,19-20H2,1-3H3,(H,22,24)/t10-,11+,12-,13-,15+,16+,17+,18+/m0/s1	CNC[C@@]1(CC[C@H]([C@]([H])(O1)O[C@@]2([C@H](C[C@@H]([C@]([H])([C@H]2O)N(C)C(CNC=O)=O)OC)N)[H])N)[H]	Small ribosomal subunit inhibitor	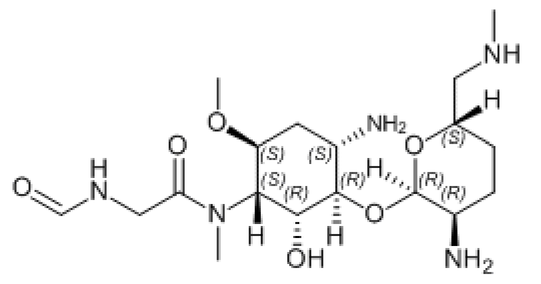	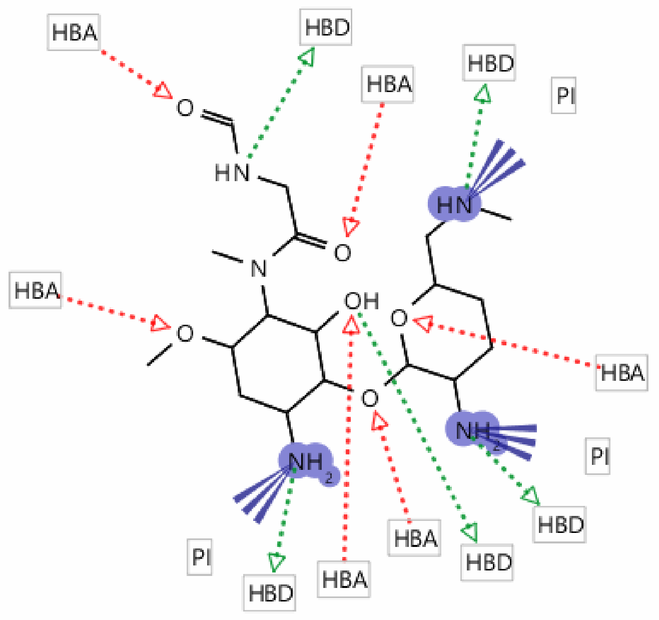
Istamycin A₂	83728-97-6	BBWHQSZBCQYJHR-JWYRXTSNSA-N	InChI=1S/C18H36N6O6/c1-22-7-9-4-5-10(19)17(29-9)30-16-11(20)6-12(28-3)14(15(16)26)24(2)13(25)8-23-18(21)27/h9-12,14-17,22,26H,4-8,19-20H2,1-3H3,(H3,21,23,27)/t9-,10+,11-,12-,14+,15+,16+,17+/m0/s1	CNC[C@@]1(CC[C@H]([C@]([H])(O1)O[C@@]2([C@H](C[C@@H]([C@]([H])([C@H]2O)N(C)C(CNC(N)=O)=O)OC)N)[H])N)[H]	Small ribosomal subunit inhibitor	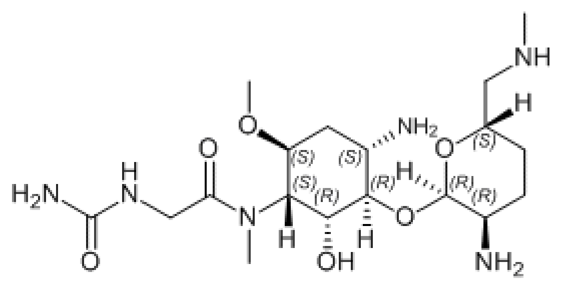	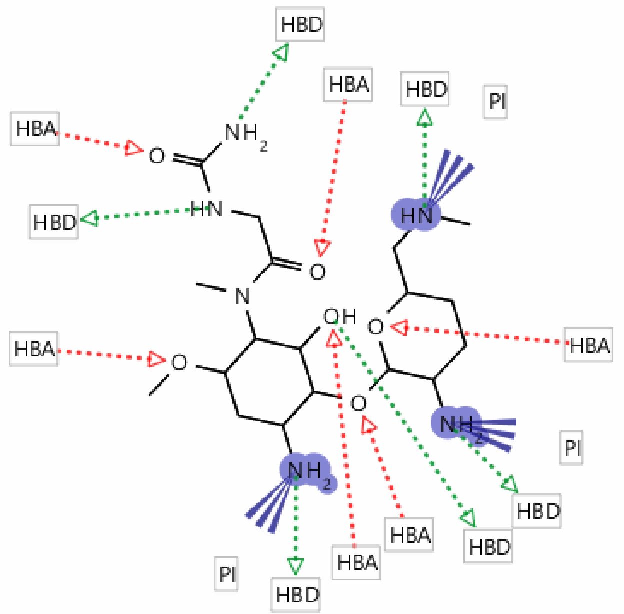
Istamycin A₃	81202-83-7	FBYTVIISAJWXNX-GTSDWETFSA-N	InChI=1S/C18H36N6O5/c1-22-7-10-4-5-11(20)18(28-10)29-17-12(21)6-13(27-3)15(16(17)26)24(2)14(25)8-23-9-19/h9-13,15-18,22,26H,4-8,20-21H2,1-3H3,(H2,19,23)/t10-,11+,12-,13-,15+,16+,17+,18+/m0/s1	CNC[C@@]1(CC[C@H]([C@]([H])(O1)O[C@@]2([C@H](C[C@@H]([C@]([H])([C@H]2O)N(C)C(CNC=N)=O)OC)N)[H])N)[H]	Small ribosomal subunit inhibitor	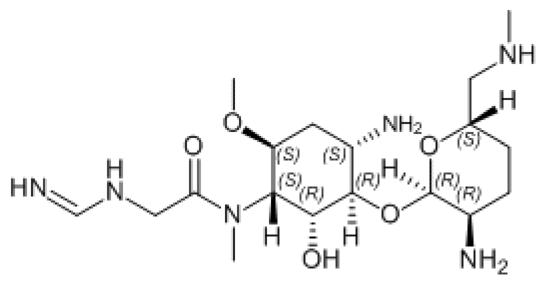	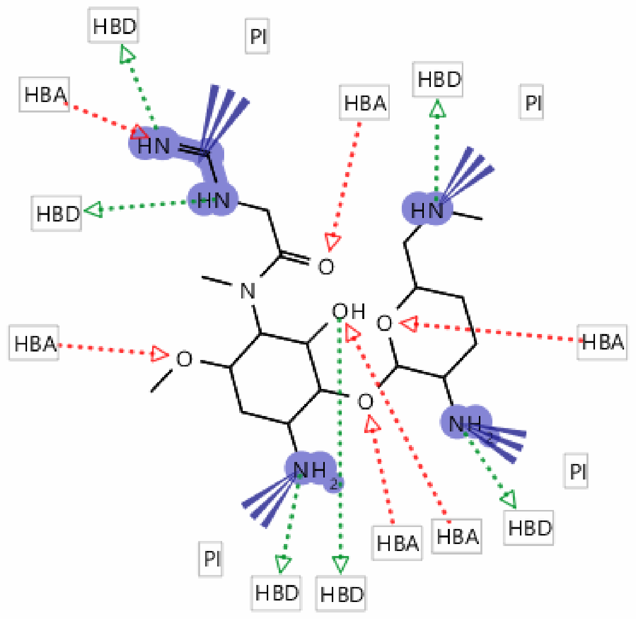
Istamycin AO		SHVSVMWFARPGSO-NVLSRCHWSA-N	InChI=1S/C13H27N3O7/c1-16-8-5(18)2-4(14)12(11(8)21)23-13-7(15)10(20)9(19)6(3-17)22-13/h4-13,16-21H,2-3,14-15H2,1H3/t4-,5+,6+,7+,8-,9+,10+,11+,12+,13+/m0/s1	CN[C@H]1[C@H](O)C[C@H](N)[C@]([C@@H]1O)(O[C@]2([H])[C@H](N)[C@@H](O)[C@H](O)[C@H](O2)CO)[H]	Small ribosomal subunit inhibitor	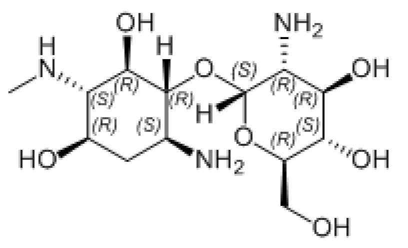	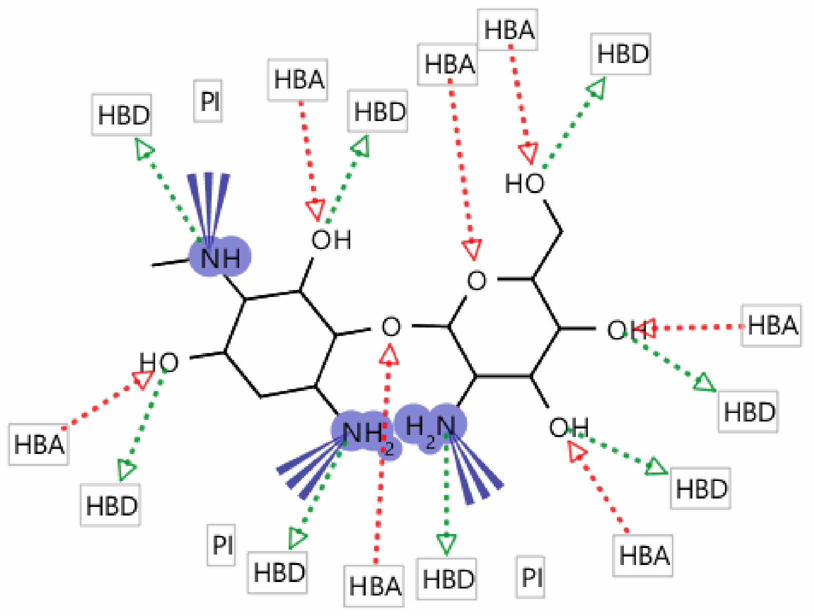
Istamycin AP (sannamycin E)	73051-92-0	CPVYVUXPFHPEFB-LJKYVLQWSA-N	InChI=1S/C13H28N4O4/c1-17-10-9(18)4-8(16)12(11(10)19)21-13-7(15)3-2-6(5-14)20-13/h6-13,17-19H,2-5,14-16H2,1H3/t6-,7+,8-,9+,10-,11+,12+,13+/m0/s1	CN[C@H]1[C@@H](C[C@@H]([C@]([H])([C@@H]1O)O[C@@]2([C@@H](CC[C@@H](CN)O2)N)[H])N)O	Small ribosomal subunit inhibitor	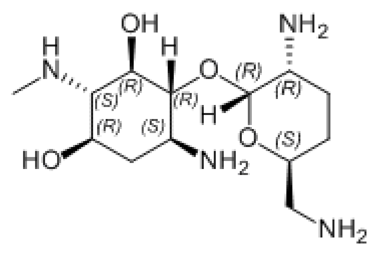	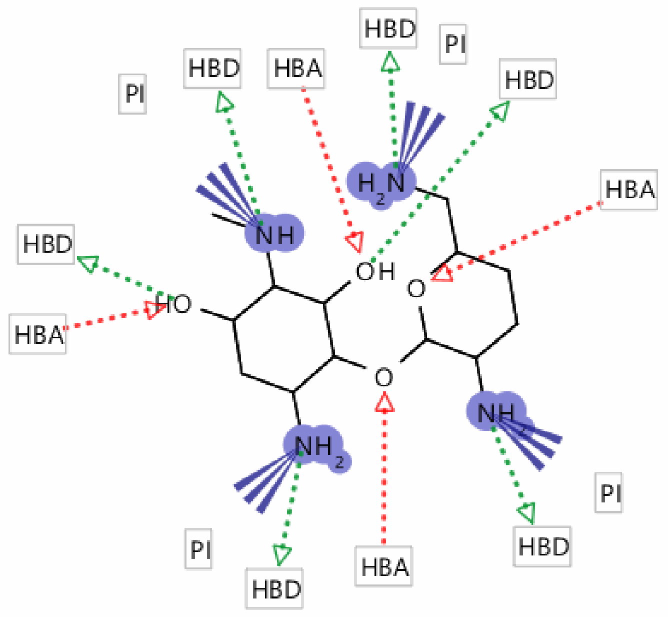
Istamycin B	72523-64-9	NEFDRWXEVITQMN-MKRRRRENSA-N	InChI=1S/C17H35N5O5/c1-21-8-9-4-5-10(19)17(26-9)27-16-11(20)6-12(25-3)14(15(16)24)22(2)13(23)7-18/h9-12,14-17,21,24H,4-8,18-20H2,1-3H3/t9-,10+,11+,12-,14+,15+,16+,17+/m0/s1	CNC[C@]1([H])CC[C@@H](N)[C@](O[C@]2([H])[C@H](N)C[C@H](OC)[C@](N(C(CN)=O)C)([H])[C@H]2O)([H])O1	Small ribosomal subunit inhibitor	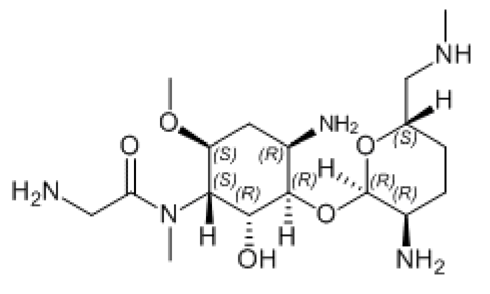	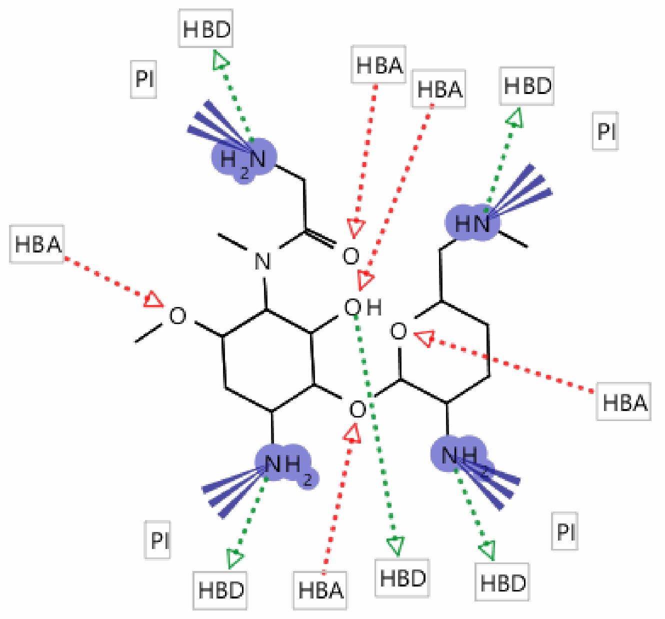

Common name	CAS Reg. No.	InChIKey	InChI	SMILES	Primary target	2D Structure	Pharmacophore[1]
Istamycin B_0	76497-51-3	GKYYNFPFPFRFFN-FWCUKHODSA-N	InChI=1S/C15H32N4O4/c1-18-7-8-4-5-9(16)15(22-8)23-14-10(17)6-11(21-3)12(19-2)13(14)20/h8-15,18-20H,4-7,16-17H2,1-3H3/t8-,9+,10+,11-,12+,13+,14+,15+/m0/s1	CNC[C@]1([H])CC[C@@H](N)[C@](O1)(O[C@]2([H])[C@H](N)C[C@H](OC)[C@@H](NC)[C@H]2O)[H]	Small ribosomal subunit inhibitor	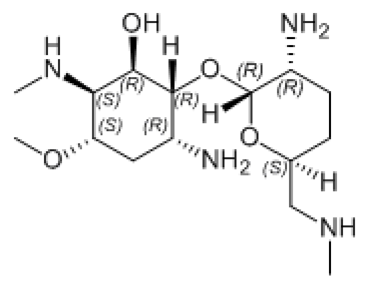	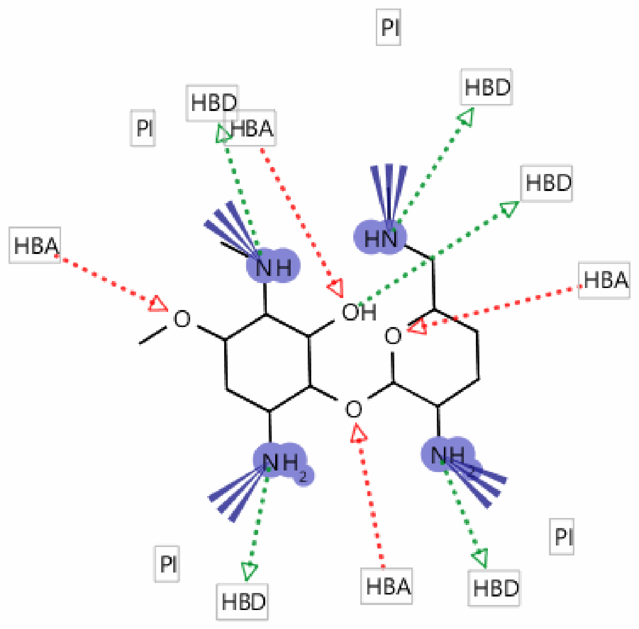
Istamycin B_1	83780-76-1	IHPJPQIRNONWGH-WLWVOYFZSA-N	InChI=1S/C18H35N5O6/c1-21-7-10-4-5-11(19)18(28-10)29-17-12(20)6-13(27-3)15(16(17)26)23(2)14(25)8-22-9-24/h9-13,15-18,21,26H,4-8,19-20H2,1-3H3,(H,22,24)/t10-,11+,12+,13-,15+,16+,17+,18+/m0/s1	CNC[C@@]1(CC[C@H]([C@]([H])(O1)O[C@@]2([C@@H](C[C@@H]([C@]([H])([C@H]2O)N(C)C(CNC=O)=O)OC)N)[H])N)[H]	Small ribosomal subunit inhibitor	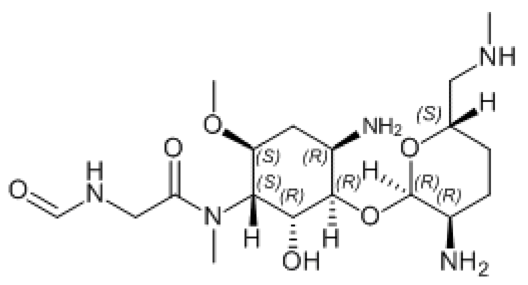	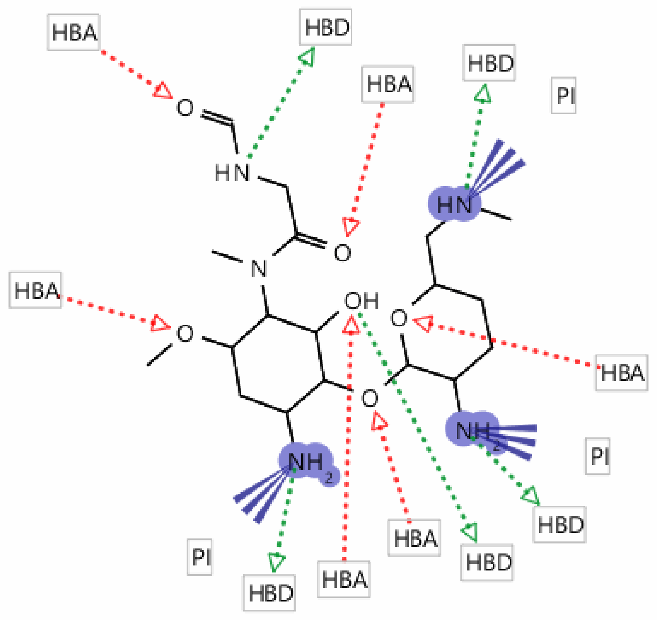
Istamycin B_3	77312-55-1	FBYTVIISAJWXNX-WLWVOYFZSA-N	InChI=1S/C18H36N6O5/c1-22-7-10-4-5-11(20)18(28-10)29-17-12(21)6-13(27-3)15(16(17)26)24(2)14(25)8-23-9-19/h9-13,15-18,22,26H,4-8,20-21H2,1-3H3,(H2,19,23)/t10-,11+,12+,13-,15+,16+,17+,18+/m0/s1	CNC[C@@]1(CC[C@H]([C@]([H])(O1)O[C@@]2([C@@H](C[C@@H]([C@]([H])([C@H]2O)N(C)C(CNC=N)=O)OC)N)[H])N)[H]	Small ribosomal subunit inhibitor	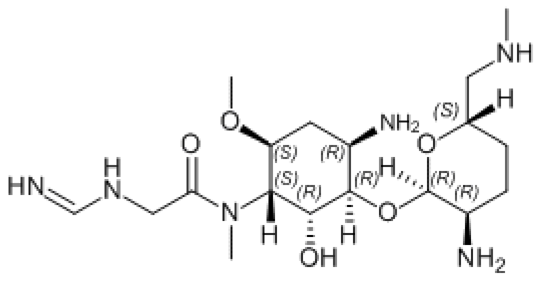	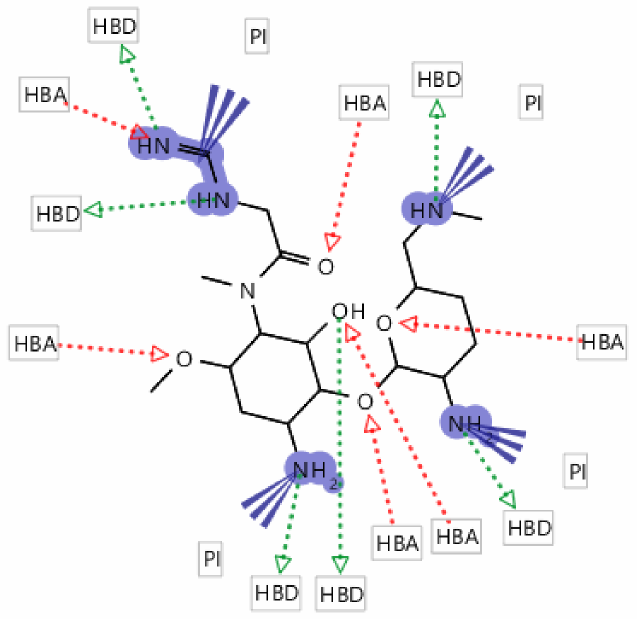
Istamycin C	83728-93-2	XKGUGTMUSMUVIP-GTSDWETFSA-N	InChI=1S/C18H37N5O5/c1-4-22-9-10-5-6-11(20)18(27-10)28-17-12(21)7-13(26-3)15(16(17)25)23(2)14(24)8-19/h10-13,15-18,22,25H,4-9,19-21H2,1-3H3/t10-,11+,12-,13-,15+,16+,17+,18+/m0/s1	CCNC[C@]1([H])CC[C@@H](N)[C@](O1)(O[C@]2([H])[C@@H](N)C[C@H](OC)[C@]([C@H]2O)(N(C(CN)=O)C)[H])[H]	Small ribosomal subunit inhibitor	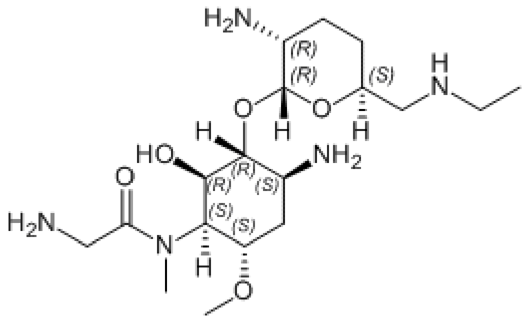	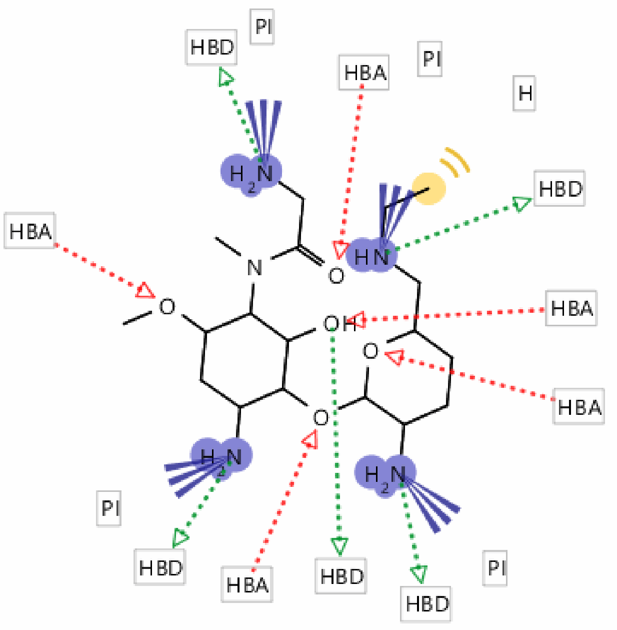
Istamycin C_0	83728-94-3	MBIKACINXASGTE-PFCCBWJBSA-N	InChI=1S/C16H34N4O4/c1-4-20-8-9-5-6-10(17)16(23-9)24-15-11(18)7-12(22-3)13(19-2)14(15)21/h9-16,19-21H,4-8,17-18H2,1-3H3/t9-,10+,11-,12-,13+,14+,15+,16+/m0/s1	CCNC[C@@]1(CC[C@H]([C@]([H])(O1)O[C@@]2([C@H](C[C@@H]([C@H]([C@H]2O)NC)OC)N)[H])N)[H]	Small ribosomal subunit inhibitor	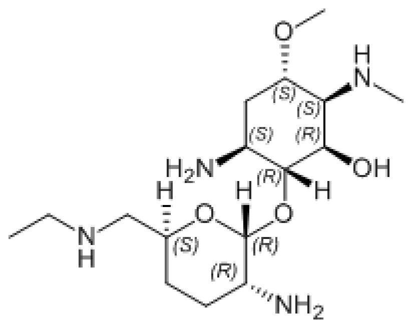	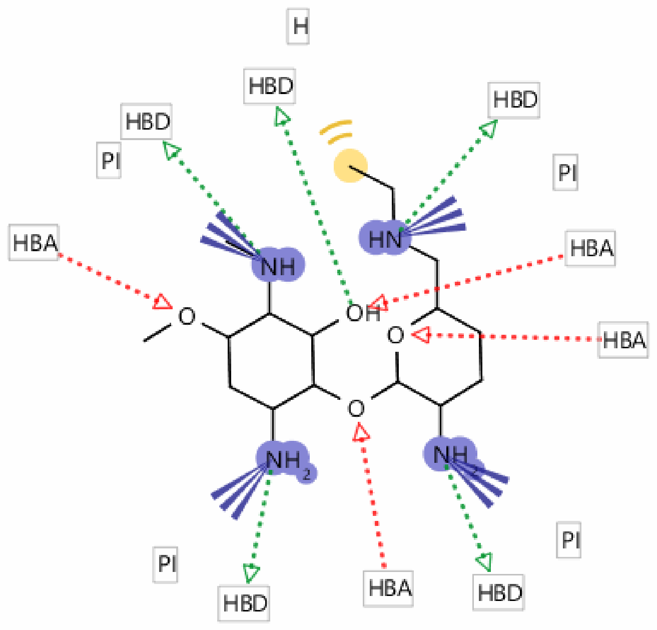
Istamycin C_1	83728-95-4	HEQBQGYOPQNTBN-XIDLQPTCSA-N	InChI=1S/C19H37N5O6/c1-4-22-8-11-5-6-12(20)19(29-11)30-18-13(21)7-14(28-3)16(17(18)27)24(2)15(26)9-23-10-25/h10-14,16-19,22,27H,4-9,20-21H2,1-3H3,(H,23,25)/t11-,12+,13-,14-,16+,17+,18+,19+/m0/s1	CCNC[C@@]1(CC[C@H]([C@]([H])(O1)O[C@@]2([C@H](C[C@@H]([C@]([H])([C@H]2O)N(C)C(CNC=O)=O)OC)N)[H])N)[H]	Small ribosomal subunit inhibitor	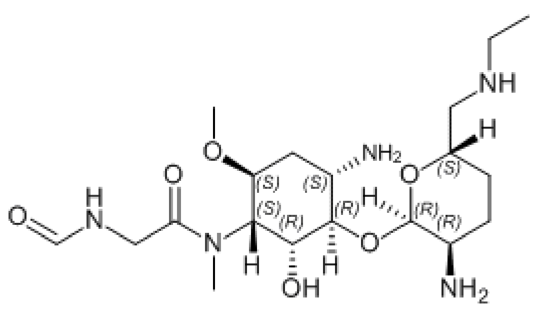	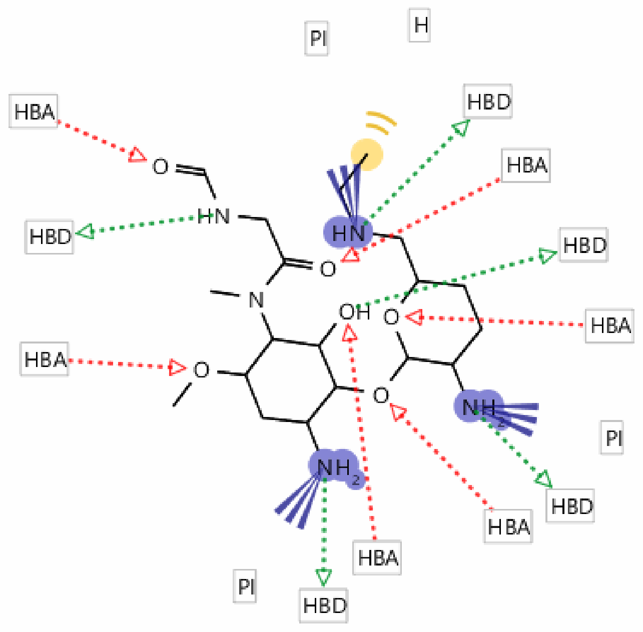

Common name	CAS Reg. No.	InChIKey	InChI	SMILES	Primary target	2D Structure	Pharmacophore[1]
Istamycin KL1		QOLRPJXGRXMWJL-NVLSRCHWSA-N	InChI=1S/C13H28N4O6/c1-17-8-5(18)2-4(15)12(11(8)21)23-13-7(16)10(20)9(19)6(3-14)22-13/h4-13,17-21H,2-3,14-16H2,1H3/t4-,5+,6+,7+,8-,9+,10+,11+,12+,13+/m0/s1	CN[C@H]1[C@@H](C[C@@H]([C@]([H])([C@@H]1O)O[C@@]2([C@@H]([C@H]([C@@H]([C@@H](CN)O2)O)O)N)[H])N)O	Small ribosomal subunit inhibitor	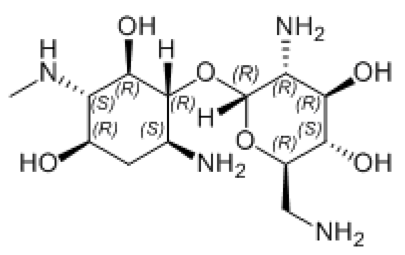	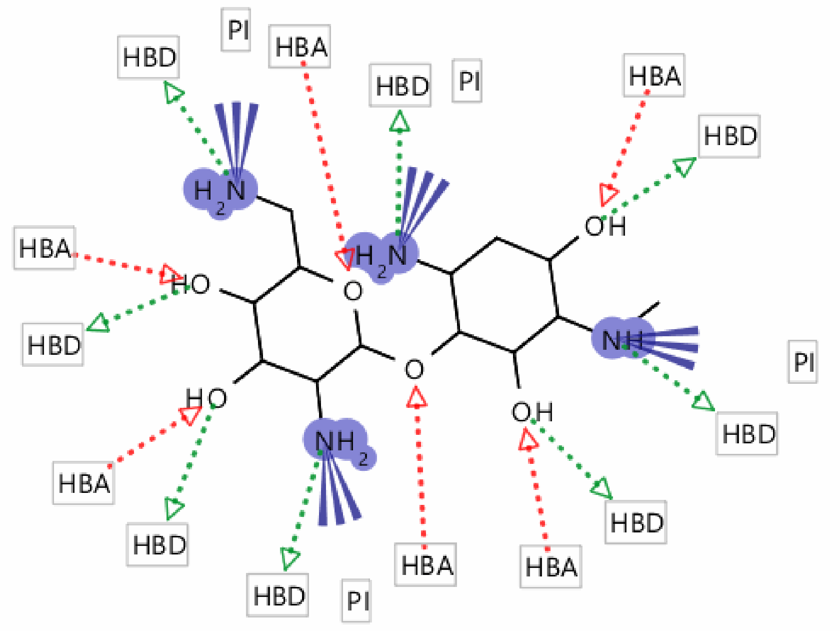
Istamycin X0 (sannamycin G)	73522-72-2	LVWCJJGPEHPHJN-OFIDPPGASA-N	InChI=1S/C14H30N4O4/c1-18-11-10(20-2)5-9(17)13(12(11)19)22-14-8(16)4-3-7(6-15)21-14/h7-14,18-19H,3-6,15-17H2,1-2H3/t7-,8+,9-,10-,11-,12+,13+,14+/m0/s1	CN[C@H]1[C@H](C[C@@H]([C@]([H])([C@@H]1O)O[C@@]2([C@@H](CC[C@@H](CN)O2)N)[H])N)OC	Small ribosomal subunit inhibitor	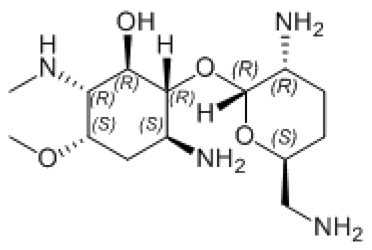	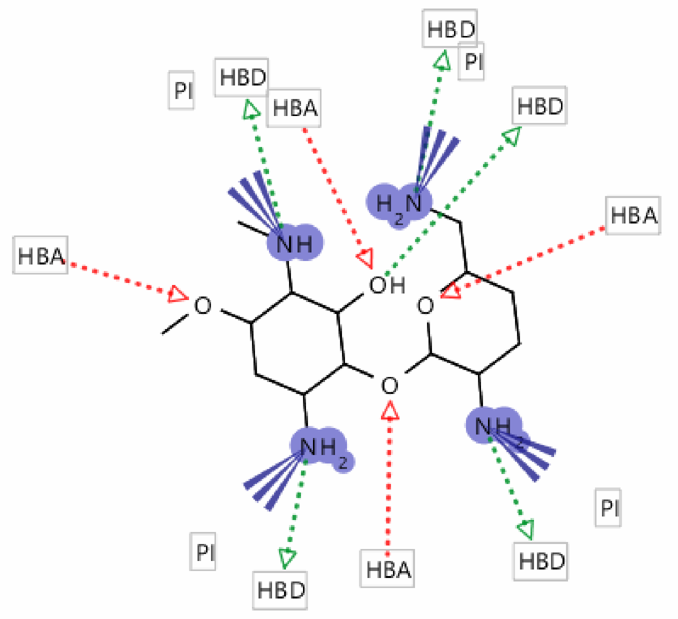
Istamycin Y0 (sannamycin H)	73491-61-9	LVWCJJGPEHPHJN-MNMRKGMHSA-N	InChI=1S/C14H30N4O4/c1-18-11-10(20-2)5-9(17)13(12(11)19)22-14-8(16)4-3-7(6-15)21-14/h7-14,18-19H,3-6,15-17H2,1-2H3/t7-,8+,9-,10+,11-,12+,13+,14+/m0/s1	CN[C@H]1[C@@H](C[C@@H]([C@]([H])([C@@H]1O)O[C@@]2([C@@H](CC[C@@H](CN)O2)N)[H])N)OC	Small ribosomal subunit inhibitor	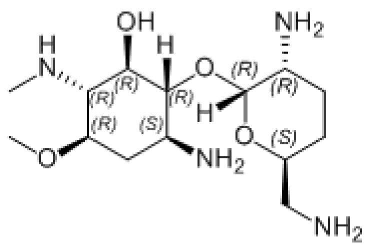	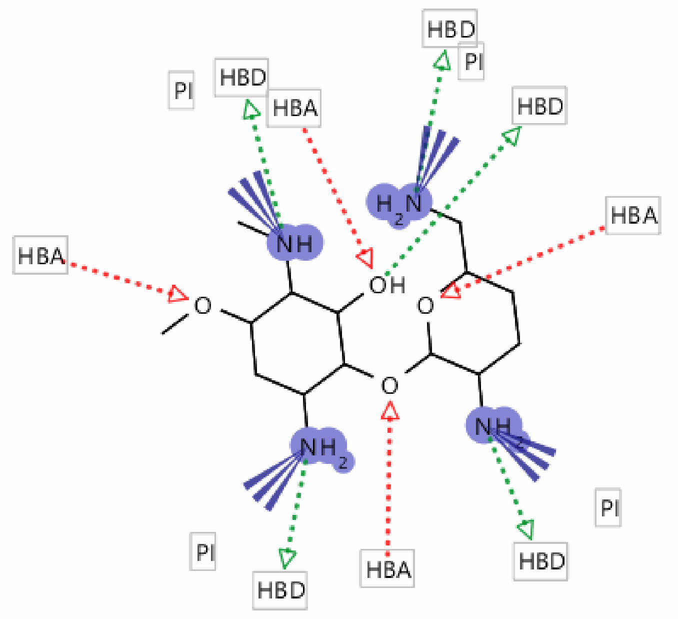
Kanamycin (kanamycin A)	59-01-8	SBUJHOSQTJFQJX-NOAMYHISSA-N	InChI=1S/C18H36N4O11/c19-2-6-10(25)12(27)13(28)18(30-6)33-16-5(21)1-4(20)15(14(16)29)32-17-11(26)8(22)9(24)7(3-23)31-17/h4-18,23-29H,1-3,19-22H2/t4-,5+,6-,7-,8+,9-,10-,11-,12+,13-,14-,15+,16-,17-,18-/m1/s1	NC[C@@H]1[C@@H](O)[C@H](O)[C@@H](O)[C@](O1)(O[C@]2([H])[C@@H](N)C[C@@H](N)[C@@]([C@H]2O)(O[C@]3([H])[C@H](O)[C@@H](N)[C@H](O)[C@H](O3)CO)[H])[H]	Small ribosomal subunit inhibitor	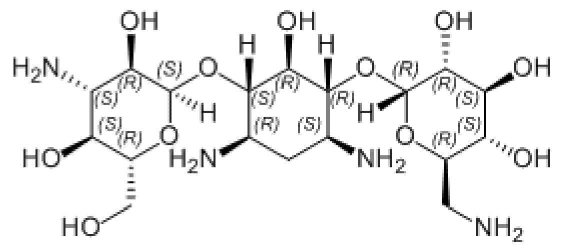	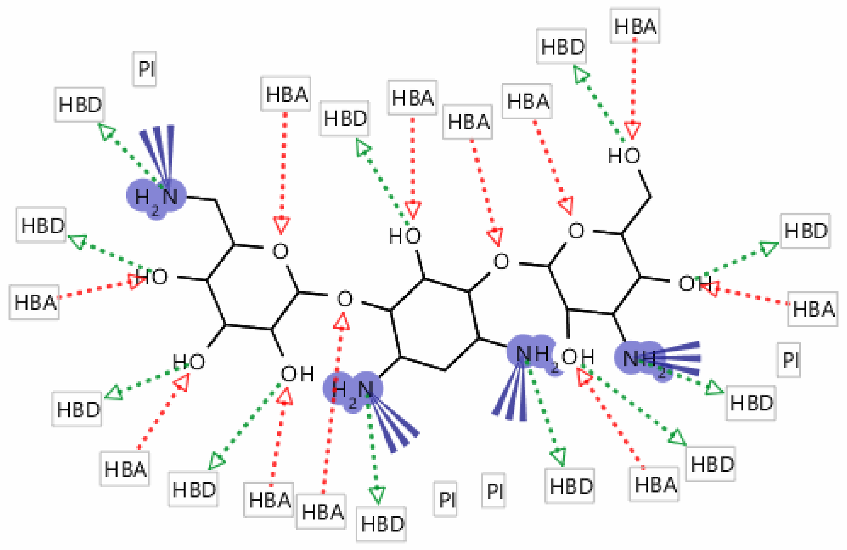
Kanamycin C	2280-32-2	WZDRWYJKESFZMB-FQSMHNGLSA-N	InChI=1S/C18H36N4O11/c19-4-1-5(20)16(33-18-13(28)8(21)10(25)6(2-23)31-18)14(29)15(4)32-17-9(22)12(27)11(26)7(3-24)30-17/h4-18,23-29H,1-3,19-22H2/t4-,5+,6+,7+,8-,9+,10+,11+,12+,13+,14-,15+,16-,17+,18+/m0/s1	N[C@H]1C[C@H]([C@@]([H])([C@H]([C@@]1(O[C@@]2([C@@H]([C@H]([C@@H]([C@@H](CO)O2)O)O)N)[H])[H])O)O[C@@]3([C@@H]([C@H]([C@@H]([C@@H](CO)O3)O)N)O)[H])N	Small ribosomal subunit inhibitor	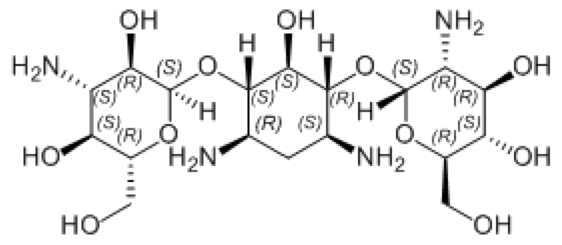	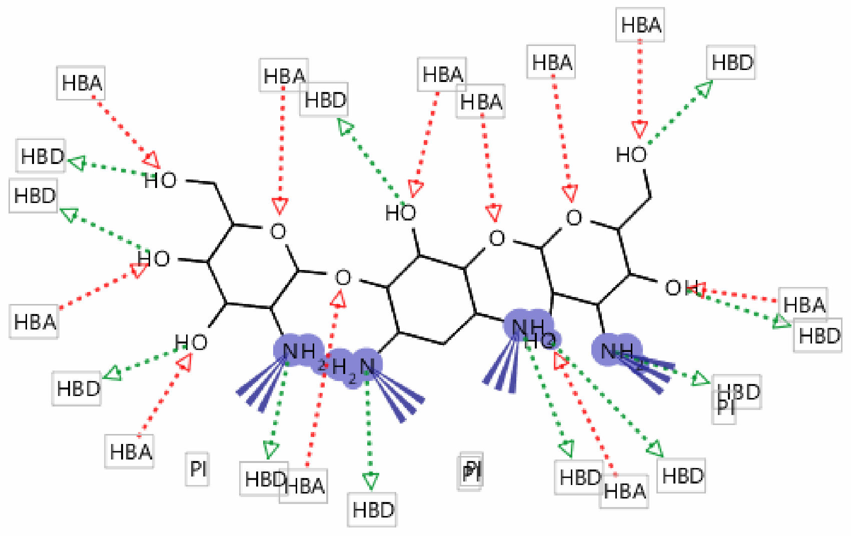
Kanamycin D		NZCOZAMBHLSNDW-HNDNCJINSA-N	InChI=1S/C18H35N3O12/c19-2-6-8(23)10(25)12(27)17(30-6)32-15-4(20)1-5(21)16(14(15)29)33-18-13(28)11(26)9(24)7(3-22)31-18/h4-18,22-29H,1-3,19-21H2/t4-,5+,6+,7+,8+,9+,10-,11-,12+,13+,14-,15+,16-,17+,18+/m0/s1	NC[C@@H]1[C@H]([C@@H]([C@H]([C@]([H])(O1)O[C@@]2([C@H](C[C@H]([C@@]([H])([C@H]2O)O[C@@]3([C@@H]([C@H]([C@@H]([C@@H](CO)O3)O)O)O)[H])N)N)[H])O)O)O	Small ribosomal subunit inhibitor	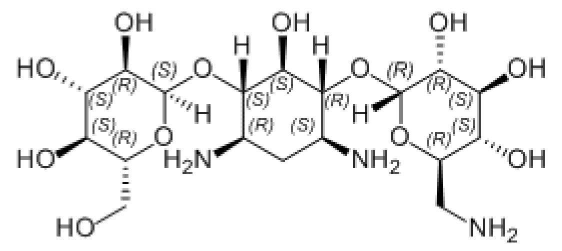	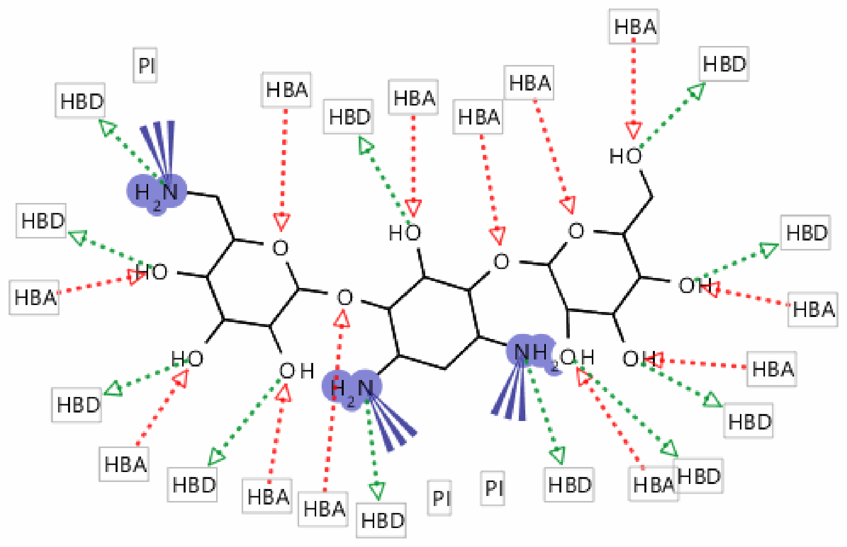

Common name	CAS Reg. No.	InChIKey	InChI	SMILES	Primary target	2D Structure	Pharmacophore[1]
Kanamycin X		OHNBRQGGOHMIAP-NOAMYHISSA-N	InChI=1S/C18H35N3O12/c19-4-1-5(20)16(33-18-13(28)12(27)10(25)7(3-23)31-18)14(29)15(4)32-17-11(26)8(21)9(24)6(2-22)30-17/h4-18,22-29H,1-3,19-21H2/t4-,5+,6-,7-,8+,9-,10-,11-,12+,13-,14-,15+,16-,17-,18-/m1/s1	N[C@@H]1C[C@@H]([C@]([H])([C@@H]([C@]1(O[C@@]2([C@@H]([C@H]([C@@H]([C@@H](CO)O2)O)N)O)[H])[H])O)O[C@@]3([C@@H]([C@H]([C@@H]([C@@H](CO)O3)O)O)O)[H])N	Small ribosomal subunit inhibitor	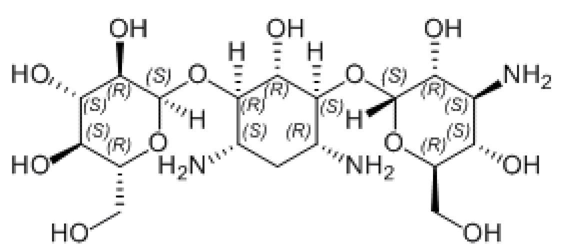	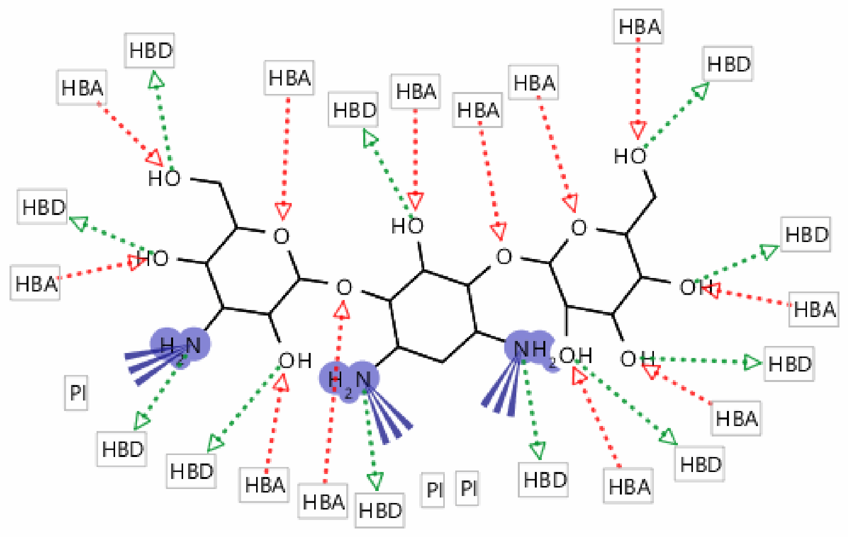
Lividamine (nebramycin IX)	36019-33-7	WFYNQZAEUGGNRH-RROBTUQGSA-N	InChI=1S/C12H25N3O6/c13-4-1-5(14)11(10(19)9(4)18)21-12-6(15)2-7(17)8(3-16)20-12/h4-12,16-19H,1-3,13-15H2/t4-,5+,6-,7+,8-,9+,10-,11-,12-/m1/s1	N[C@@H]1C[C@H](N)[C@]([C@H](O)[C@H]1O)(O[C@]2([H])[C@H](N)C[C@H](O)[C@H](O2)CO)[H]	Small ribosomal subunit inhibitor	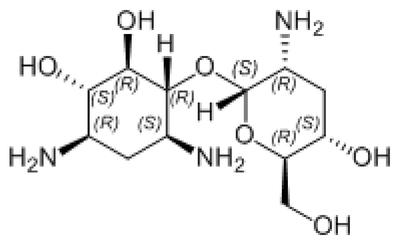	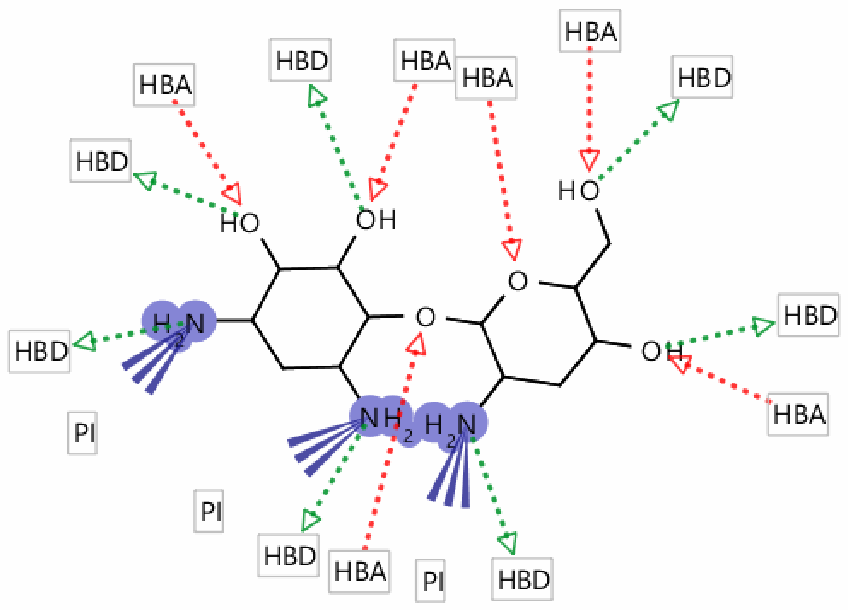
Lividomycin	36441-41-5	DBLVDAUGBTYDFR-SWMBIRFSSA-N	InChI=1S/C29H55N5O18/c30-3-11-23(51-28-20(43)19(42)17(40)13(5-36)47-28)18(41)15(34)27(45-11)50-24-14(6-37)48-29(21(24)44)52-25-16(39)7(31)1-8(32)22(25)49-26-9(33)2-10(38)12(4-35)46-26/h7-29,35-44H,1-6,30-34H2/t7-,8+,9-,10+,11+,12-,13-,14-,15-,16+,17-,18-,19+,20+,21-,22-,23-,24-,25-,26-,27-,28-,29+/m1/s1	NC[C@H]1[C@]([H])([C@@H]([C@H]([C@]([H])(O1)O[C@H]2[C@H]([C@@H](O[C@@H]2CO)O[C@@]3([C@H]([C@@H](C[C@@H]([C@]3(O[C@@]4([C@@H](C[C@@H]([C@@H](CO)O4)O)N)[H])[H])N)N)O)[H])O)N)O)O[C@@]5([C@@H](O)[C@H]([C@@H]([C@@H](CO)O5)O)O)[H]	Small ribosomal subunit inhibitor	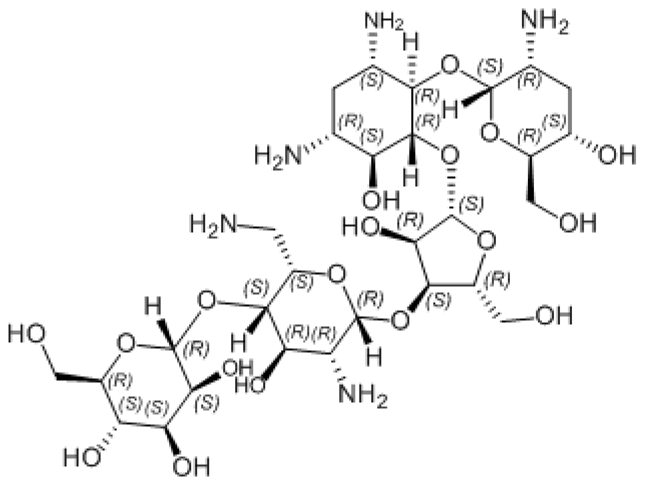	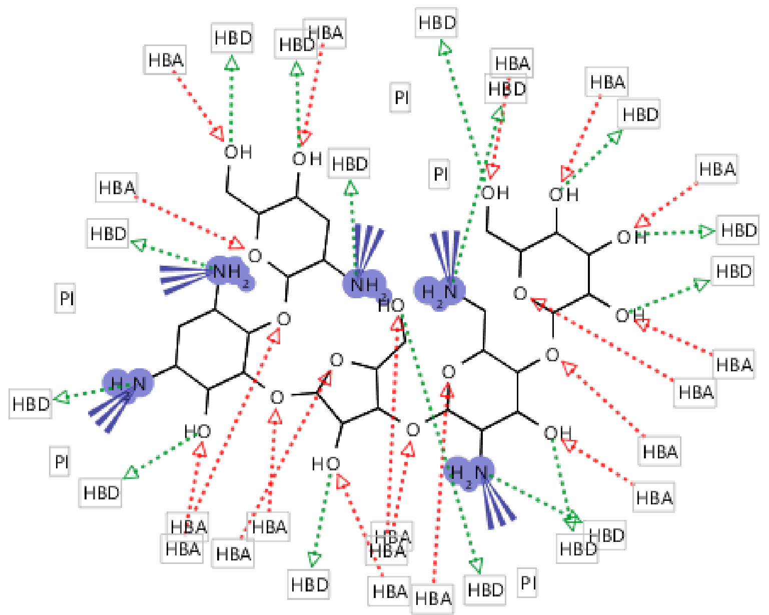
Lividomycin B (3'-deoxyparomomycin I)	37636-51-4	BRSBFYLXCMGALM-CDEWKRIDSA-N	InChI=1S/C23H45N5O13/c24-3-10-15(33)16(34)13(28)22(36-10)40-19-12(5-30)38-23(17(19)35)41-20-14(32)6(25)1-7(26)18(20)39-21-8(27)2-9(31)11(4-29)37-21/h6-23,29-35H,1-5,24-28H2/t6-,7+,8-,9+,10+,11-,12-,13-,14+,15-,16-,17-,18-,19-,20-,21-,22-,23+/m1/s1	NC[C@H]1[C@H]([C@@H]([C@H]([C@]([H])(O1)O[C@H]2[C@H]([C@@H](O[C@@H]2CO)O[C@@]3([C@H]([C@@H](C[C@@H]([C@]3(O[C@@]4([C@@H](C[C@@H]([C@@H](CO)O4)O)N)[H])[H])N)N)O)[H])O)N)O)O	Small ribosomal subunit inhibitor	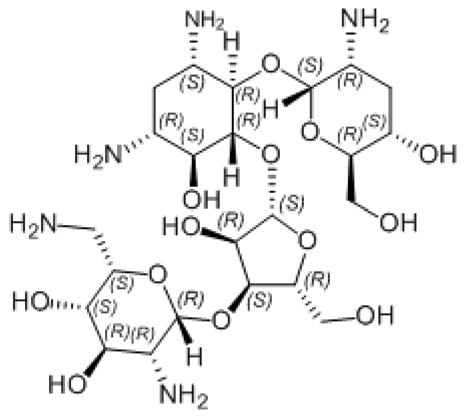	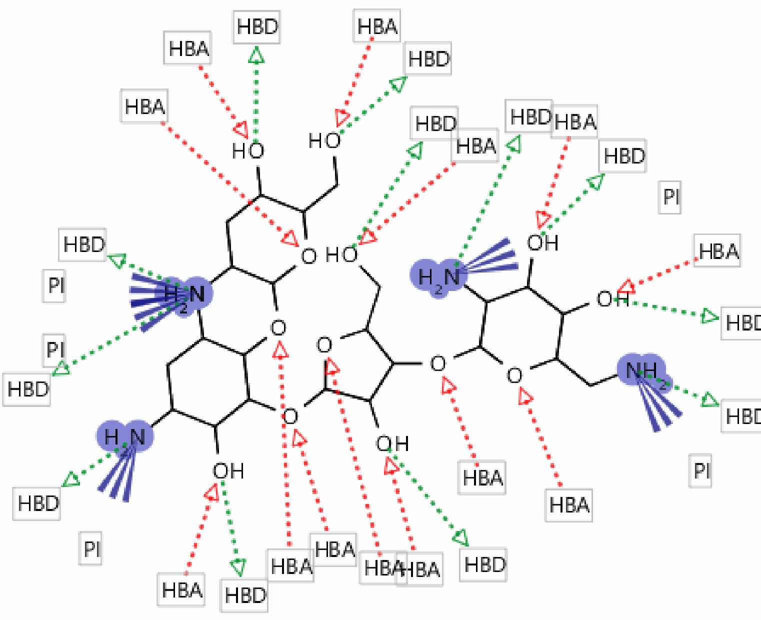
Mannosylparomomycin	36019-37-1	GESYZSHWHHOJFW-JDDJXUFZSA-N	InChI=1S/C29H55N5O19/c30-2-8-23(52-28-20(44)19(43)16(40)10(4-36)48-28)18(42)13(34)27(46-8)51-24-11(5-37)49-29(21(24)45)53-25-14(38)6(31)1-7(32)22(25)50-26-12(33)17(41)15(39)9(3-35)47-26/h6-29,35-45H,1-5,30-34H2/t6-,7+,8+,9-,10-,11-,12-,13-,14+,15-,16-,17-,18-,19+,20+,21-,22-,23-,24-,25-,26-,27-,28-,29+/m1/s1	NC[C@H]1[C@]([H])([C@H](O)[C@H]([C@]([H])(O1)O[C@H]2[C@H]([C@@H](O[C@@H]2CO)O[C@@]3([C@H]([C@@H](C[C@@H]([C@]3(O[C@@]4([C@@H]([C@H]([C@@H]([C@@H](CO)O4)O)O)N)[H])[H])N)N)O)[H])O)N)O[C@@]5([C@H]([C@H]([C@@H]([C@@H](CO)O5)O)O)O)[H]	Small ribosomal subunit inhibitor	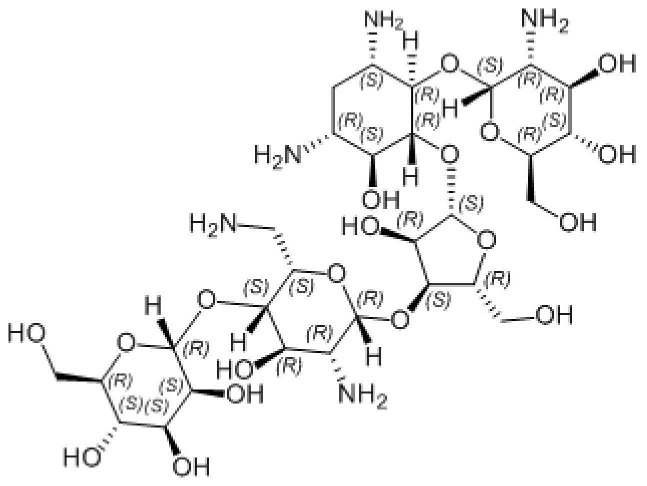	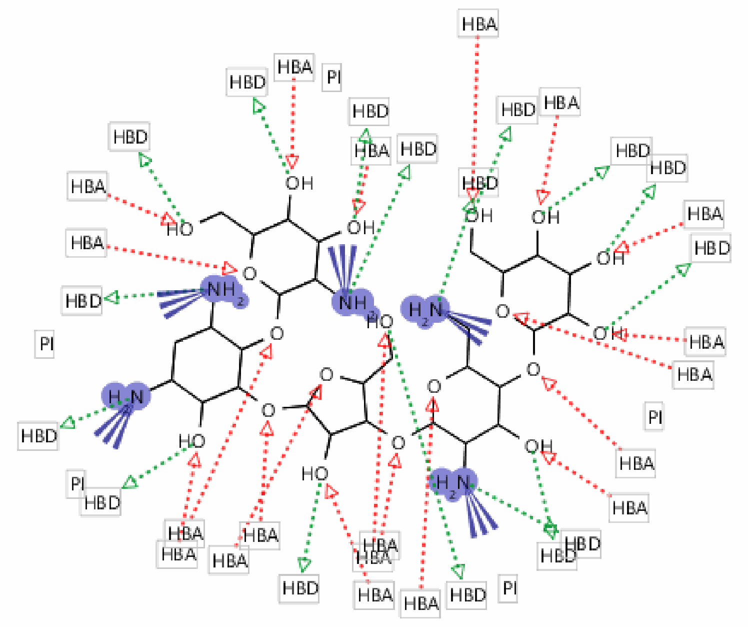
Micronomicin (gentamicin C_{2b})	52093-21-7	DNYGXMICFMACRA-XHEDQWPISA-N	InChI=1S/C20H41N5O7/c1-20(28)8-29-19(14(27)17(20)25-3)32-16-12(23)6-11(22)15(13(16)26)31-18-10(21)5-4-9(30-18)7-24-2/h9-19,24-28H,4-8,21-23H2,1-3H3/t9-,10+,11-,12+,13-,14+,15+,16-,17+,18+,19+,20-/m0/s1	C[C@@]1(CO[C@@]([H])([C@@H]([C@H]1NC)O)O[C@]2([C@@H](C[C@@H]([C@]([H])([C@@H]2O)O[C@@]3([C@@H](CC[C@@]([H])(CNC)O3)N)[H])N)N)[H])O	Small ribosomal subunit inhibitor	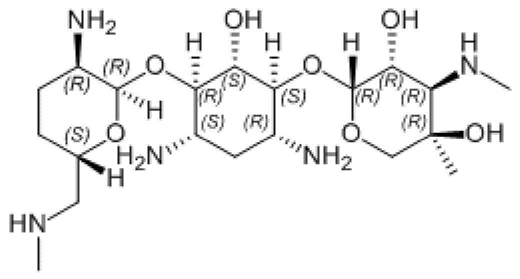	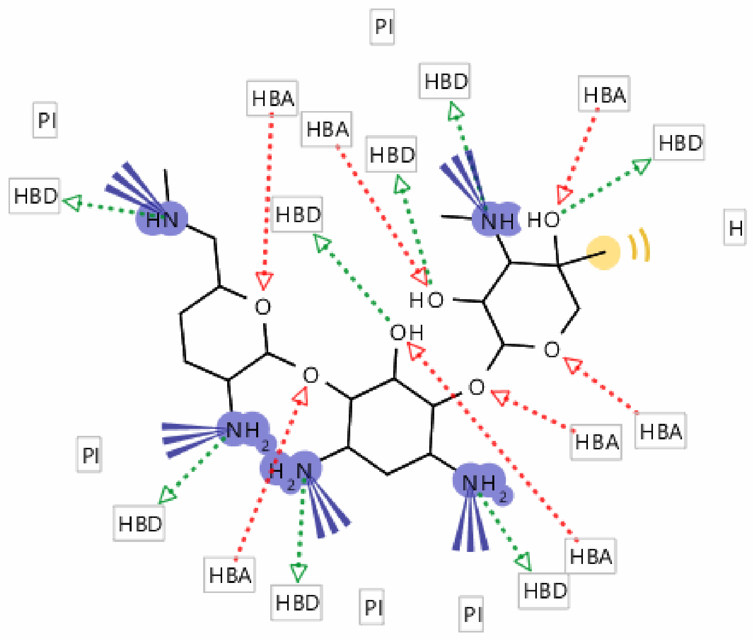

Common name	CAS Reg. No.	InChIKey	InChI	SMILES	Primary target	2D Structure	Pharmacophore[1]
Neamine (neomycin A; nebramycin X)	3947-65-7	SYJXFKPQNSDJLI-HKEUSBCWSA-N	InChI=1S/C12H26N4O6/c13-2-5-8(18)9(19)6(16)12(21-5)22-11-4(15)1-3(14)7(17)10(11)20/h3-12,17-20H,1-2,13-16H2/t3-,4+,5-,6-,7+,8-,9-,10-,11-,12-/m1/s1	NC[C@@H]1[C@H]([C@@H]([C@H]([C@]([H])(O1)O[C@@]2([C@H](C[C@H]([C@@H]([C@H]2O)O)N)N)[H])N)O)O	Small ribosomal subunit inhibitor	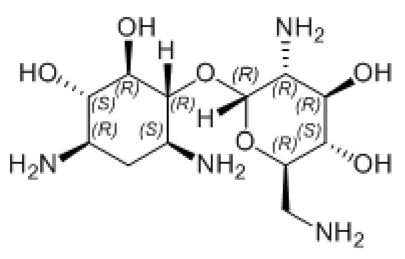	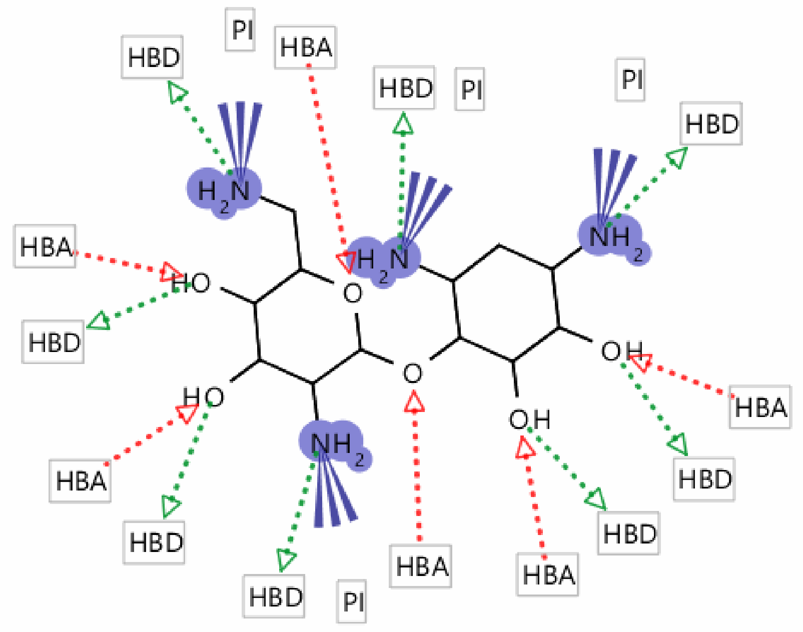
Nebramine (nebramycin VIII)	34051-04-2	QBWLTQZEVUXXSR-RROBTUQGSA-N	InChI=1S/C12H26N4O5/c13-3-8-7(17)2-6(16)12(20-8)21-11-5(15)1-4(14)9(18)10(11)19/h4-12,17-19H,1-3,13-16H2/t4-,5+,6-,7+,8-,9+,10-,11-,12-/m1/s1	NC[C@@H]1[C@H](C[C@H]([C@]([H])(O1)O[C@@]2([C@H](C[C@H]([C@@H]([C@H]2O)O)N)N)[H])N)O	Small ribosomal subunit inhibitor	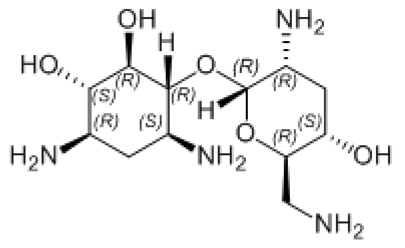	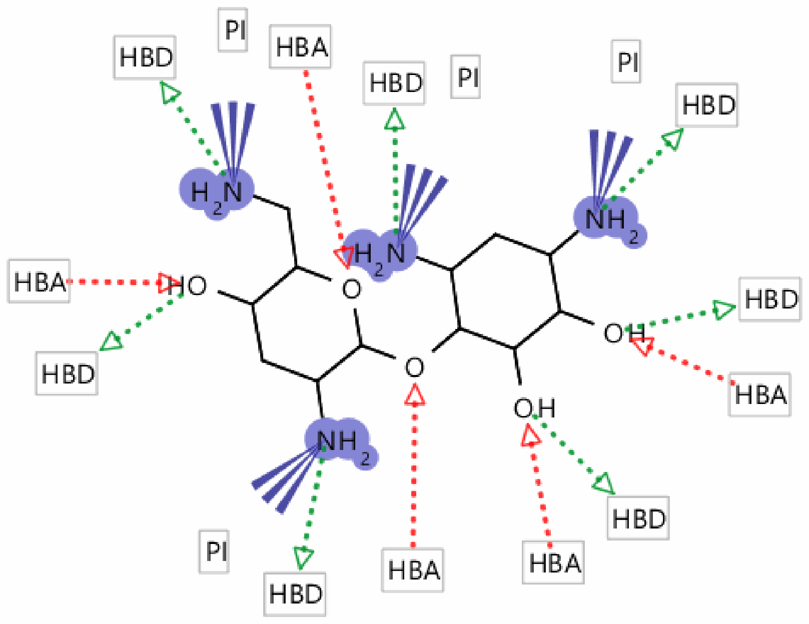
Nebramycin III	31077-70-0	MOWMHIINUAQFMU-DNBVWFFRSA-N	InChI=1S/C18H36N4O11/c19-2-6-9(24)11(26)8(22)17(30-6)32-15-4(20)1-5(21)16(14(15)29)33-18-13(28)12(27)10(25)7(3-23)31-18/h4-18,23-29H,1-3,19-22H2/t4-,5+,6+,7+,8+,9+,10+,11+,12-,13+,14-,15+,16-,17+,18+/m0/s1	NC[C@@H]1[C@H]([C@@H]([C@H]([C@]([H])(O1)O[C@@]2([C@H](C[C@H]([C@@]([H])([C@H]2O)O[C@@]3([C@@H]([C@H]([C@@H]([C@@H](CO)O3)O)O)O)[H])N)N)[H])N)O)O	Small ribosomal subunit inhibitor	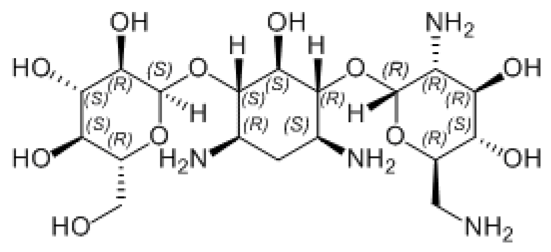	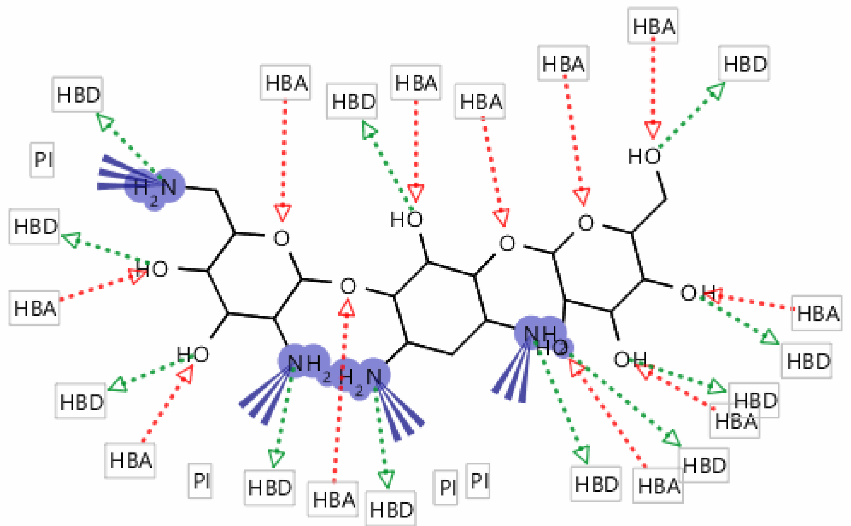
Nebramycin IV	51736-76-6	XCSTZNJIQFIVPE-FQSMHNGLSA-N	InChI=1S/C19H38N6O11/c20-2-6-11(27)12(28)9(24)17(33-6)35-15-4(21)1-5(22)16(14(15)30)36-18-13(29)8(23)10(26)7(34-18)3-32-19(25)31/h4-18,26-30H,1-3,20-24H2,(H2,25,31)/t4-,5+,6+,7+,8-,9+,10+,11+,12+,13+,14-,15+,16-,17+,18+/m0/s1	NC[C@@H]1[C@H]([C@@H]([C@H]([C@]([H])(O1)O[C@@]2([C@H](C[C@H]([C@@]([H])([C@H]2O)O[C@@]3([C@@H]([C@H]([C@@H]([C@@]([H])(COC(N)=O)O3)O)N)O)[H])N)N)[H])N)O)O	Small ribosomal subunit inhibitor	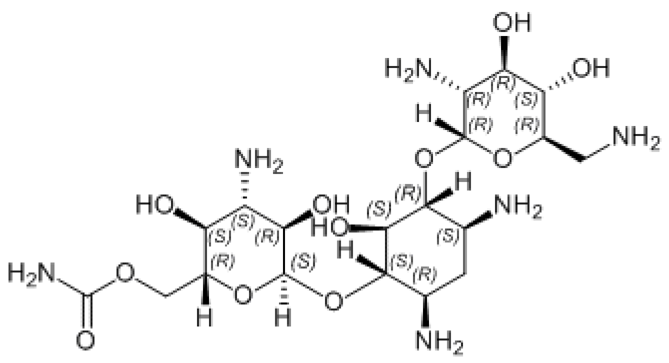	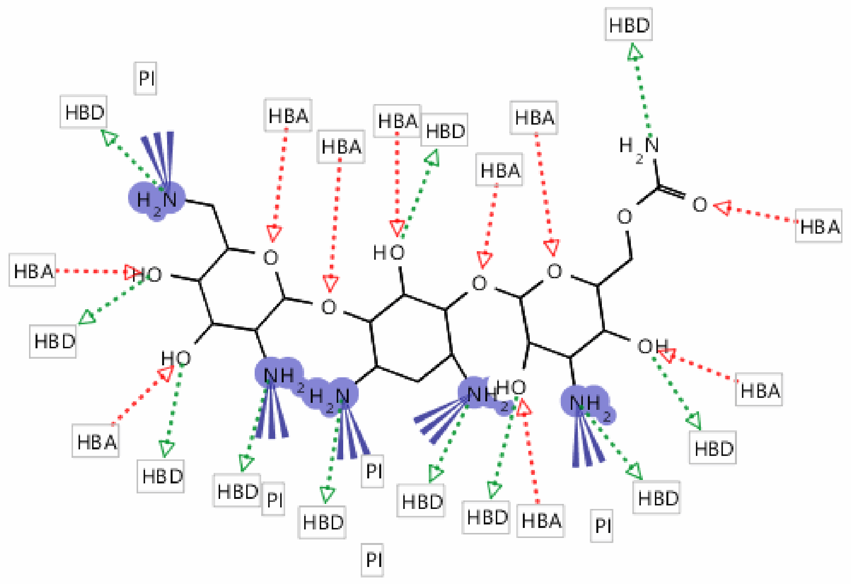
Nebramycin V'	51736-77-7	YPPFEJHOHNPKLT-PBSUHMDJSA-N	InChI=1S/C19H38N6O10/c20-3-9-8(26)2-7(23)17(32-9)34-15-5(21)1-6(22)16(14(15)29)35-18-13(28)11(24)12(27)10(33-18)4-31-19(25)30/h5-18,26-29H,1-4,20-24H2,(H2,25,30)/t5-,6+,7+,8-,9+,10+,11-,12+,13+,14-,15+,16-,17+,18+/m0/s1	NC[C@@H]1[C@H](C[C@H]([C@]([H])(O1)O[C@@]2([C@H](C[C@H]([C@@]([H])([C@H]2O)O[C@@]3([C@H](O)[C@H]([C@@H]([C@@]([H])(COC(N)=O)O3)O)N)[H])N)N)[H])N)O	Small ribosomal subunit inhibitor	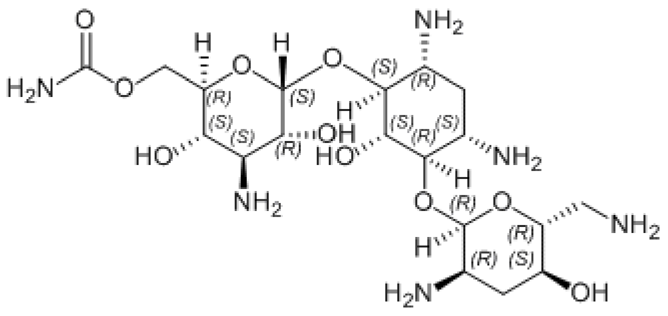	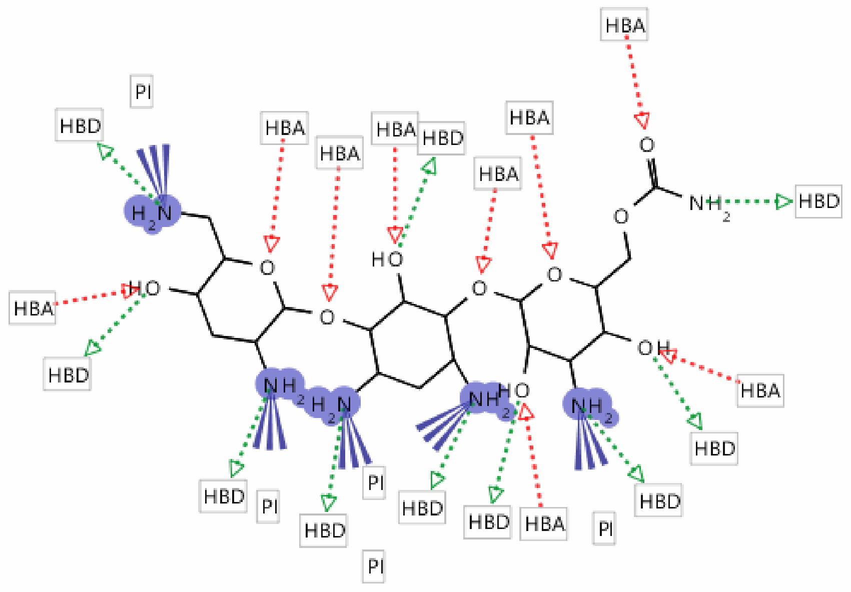
Nebramycin XI	64332-33-8	ACQKFHZCTOKZKP-PBSUHMDJSA-N	InChI=1S/C19H38N6O10/c20-3-9-8(27)2-7(25-19(24)31)17(32-9)34-15-5(21)1-6(22)16(14(15)30)35-18-13(29)11(23)12(28)10(4-26)33-18/h5-18,26-30H,1-4,20-23H2,(H3,24,25,31)/t5-,6+,7+,8-,9+,10+,11-,12+,13+,14-,15+,16-,17+,18+/m0/s1	NC[C@@H]1[C@@H](O)C[C@]([C@](O1)(O[C@]2([H])[C@@H](N)C[C@@H](N)[C@@]([C@H]2O)(O[C@]3([H])[C@H](O)[C@@H](N)[C@H](O)[C@H](O3)CO)[H])[H])(NC(N)=O)[H]	Small ribosomal subunit inhibitor	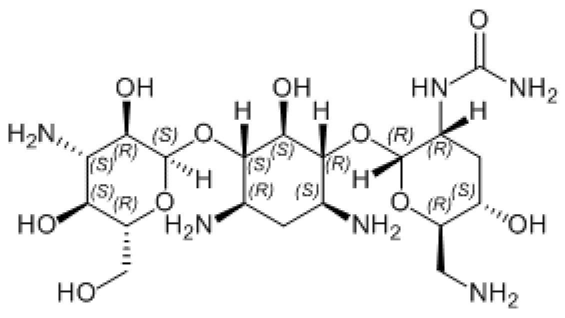	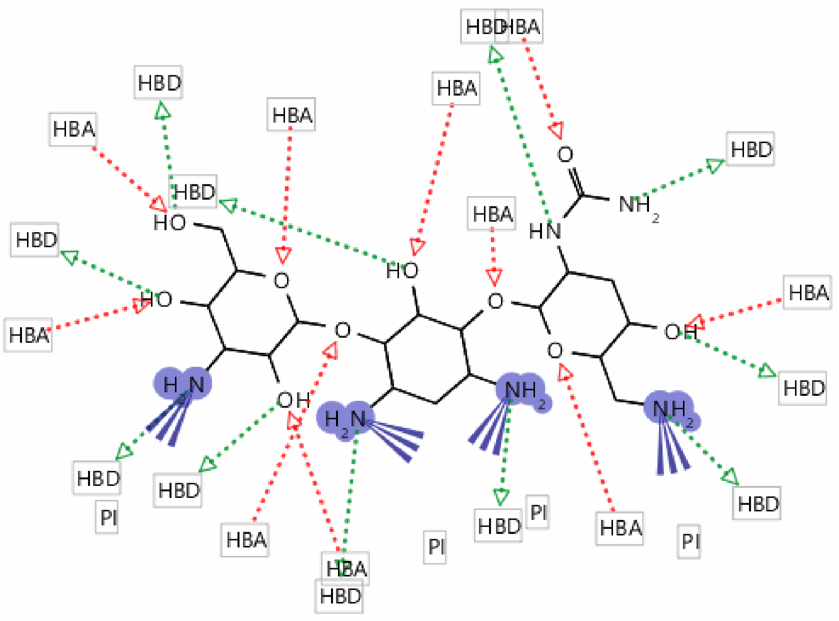

Common name	CAS Reg. No.	InChIKey	InChI	SMILES	Primary target	2D Structure	Pharmacophore[1]
Nebramycin XII	64332-34-9	RTOAPJRCIZBHKA-HFSBJZBZSA-N	InChI=1S/C18H36N4O10/c19-3-9-8(24)2-7(22)17(29-9)31-15-5(20)1-6(21)16(14(15)28)32-18-13(27)12(26)11(25)10(4-23)30-18/h5-18,23-28H,1-4,19-22H2/t5-,6+,7+,8-,9+,10+,11+,12-,13+,14-,15+,16-,17+,18+/m0/s1	NC[C@@H]1[C@@H](O)C[C@@H](N)[C@](O1)(O[C@]2([H])[C@@H](N)C[C@@H](N)[C@@]([C@H]2O)(O[C@]3([H])[C@H](O)[C@@H](O)[C@H](O)[C@H](O3)CO)[H])[H]	Small ribosomal subunit inhibitor	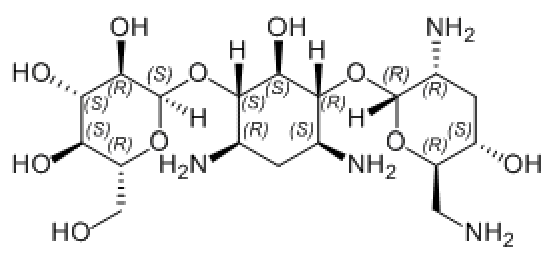	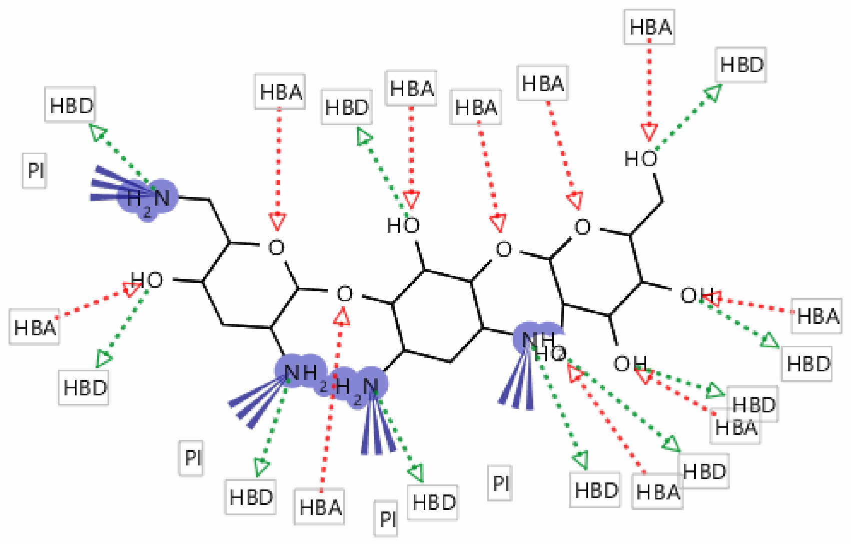
Nebramycin XIII	64332-35-0	BXUDMXDCTTUXLQ-PBSUHMDJSA-N	InChI=1S/C19H38N6O10/c20-5-1-6(21)16(35-18-13(29)11(23)12(28)10(4-26)33-18)14(30)15(5)34-17-7(22)2-8(27)9(32-17)3-25-19(24)31/h5-18,26-30H,1-4,20-23H2,(H3,24,25,31)/t5-,6+,7+,8-,9+,10+,11-,12+,13+,14-,15+,16-,17+,18+/m0/s1	N[C@H]1C[C@H]([C@@]([H])([C@H]([C@@]1(O[C@@]2([C@@H](C[C@@H]([C@@]([H])(CNC(N)=O)O2)O)N)[H])[H])O)O[C@@]3([C@@H]([C@H]([C@@H]([C@@H](CO)O3)O)N)O)[H])N	Small ribosomal subunit inhibitor	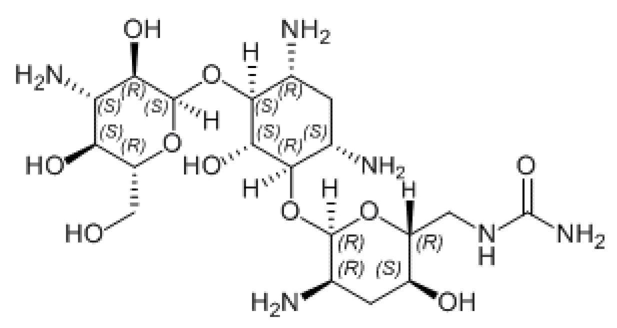	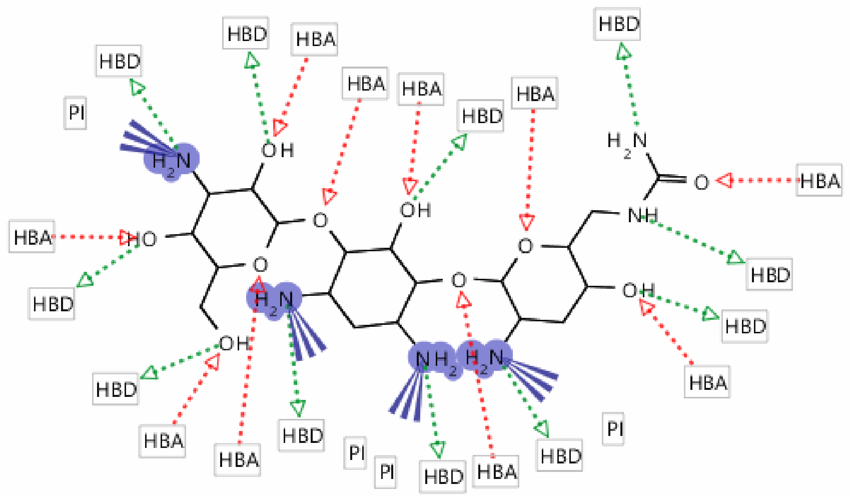
Neomycin C	66-86-4	PGBHMTALBVVCIT-VZXHOKRSSA-N	InChI=1S/C23H46N6O13/c24-2-7-13(32)15(34)10(28)21(37-7)40-18-6(27)1-5(26)12(31)20(18)42-23-17(36)19(9(4-30)39-23)41-22-11(29)16(35)14(33)8(3-25)38-22/h5-23,30-36H,1-4,24-29H2/t5-,6+,7-,8-,9-,10-,11-,12+,13-,14-,15-,16-,17-,18-,19-,20-,21-,22-,23+/m1/s1	NC[C@@H]1[C@H]([C@@H]([C@H]([C@]([H])(O1)O[C@@]2([C@H](C[C@H]([C@@H]([C@]2(O[C@H]3[C@@H]([C@@H]([C@@H](CO)O3)O[C@@]4([C@@H]([C@H]([C@@H]([C@@H](CN)O4)O)O)N)[H])O)[H])O)N)N)[H])N)O)O	Small ribosomal subunit inhibitor	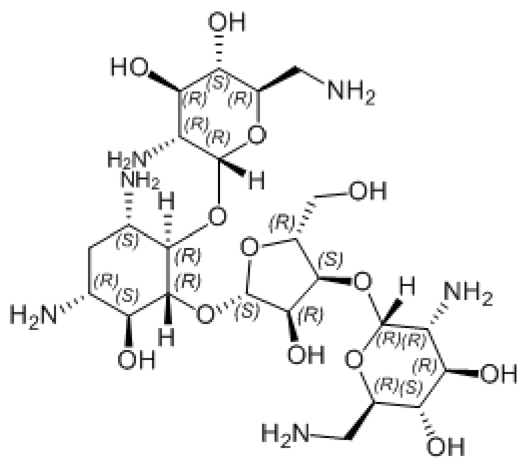	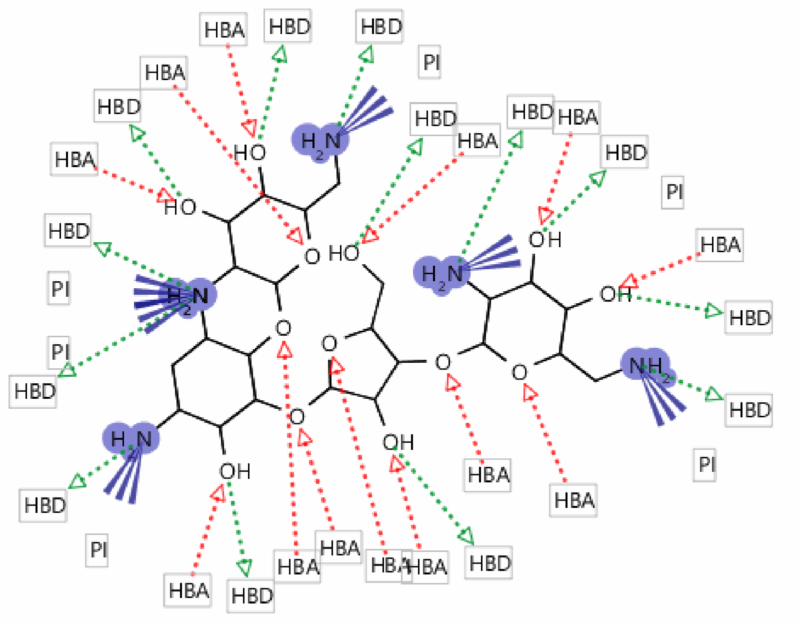
Neomycin F (paromomycin II)	51795-47-2	UOZODPSAJZTQNH-VZXHOKRSSA-N	InChI=1S/C23H45N5O14/c24-2-7-13(32)15(34)10(27)21(37-7)41-19-9(4-30)39-23(17(19)36)42-20-12(31)5(25)1-6(26)18(20)40-22-11(28)16(35)14(33)8(3-29)38-22/h5-23,29-36H,1-4,24-28H2/t5-,6+,7-,8-,9-,10-,11-,12+,13-,14-,15-,16-,17-,18-,19-,20-,21-,22-,23+/m1/s1	NC[C@@H]1[C@H]([C@@H]([C@H]([C@]([H])(O1)O[C@H]2[C@H]([C@@H](O[C@@H]2CO)O[C@@]3([C@H]([C@@H](C[C@@H]([C@]3(O[C@@]4([C@@H]([C@H]([C@@H]([C@@H](CO)O4)O)O)N)[H])[H])N)N)O)[H])O)N)O)O	Small ribosomal subunit inhibitor	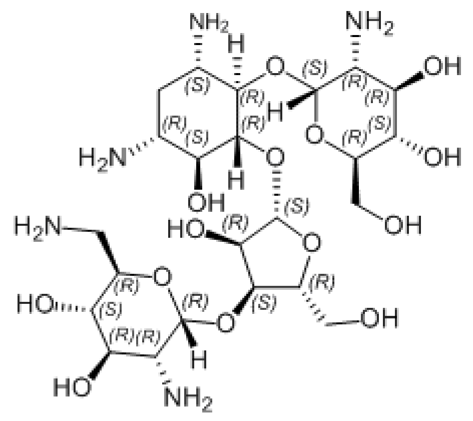	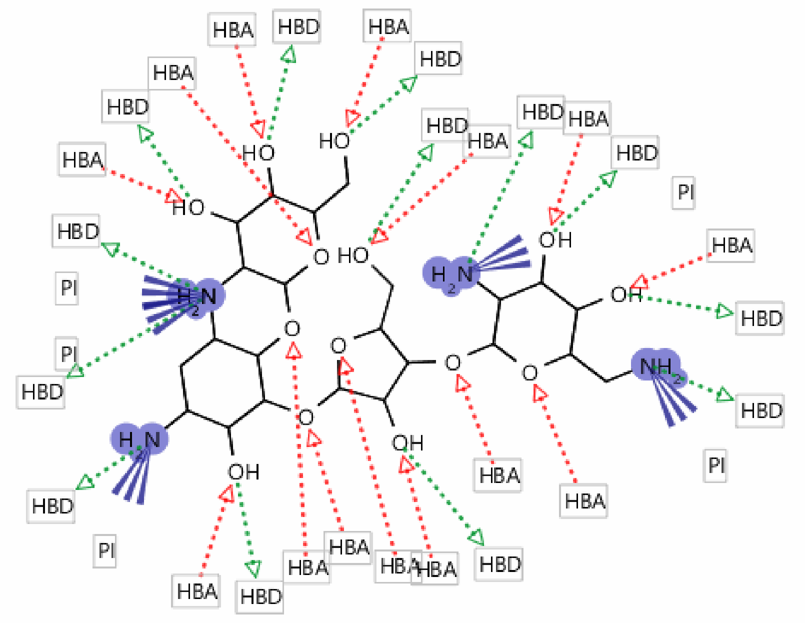
Netilmicin	56391-56-1	CIDUJQMULVCIBT-MQDUPKMGSA-N	InChI=1S/C21H41N5O7/c1-4-26-13-7-12(24)16(32-19-11(23)6-5-10(8-22)31-19)14(27)17(13)33-20-15(28)18(25-3)21(2,29)9-30-20/h5,11-20,25-29H,4,6-9,22-24H2,1-3H3/t11-,12+,13-,14+,15-,16-,17+,18-,19-,20-,21+/m1/s1	CCN[C@@]1(C[C@@H]([C@]([H])([C@@H]([C@]1(O[C@@]2([C@@H]([C@H]([C@](O)(C)CO2)NC)O)[H])[H])O)O[C@@]3([C@@H](CC=C(CN)O3)N)[H])N)[H]	Small ribosomal subunit inhibitor	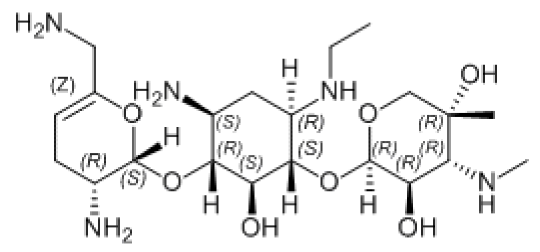	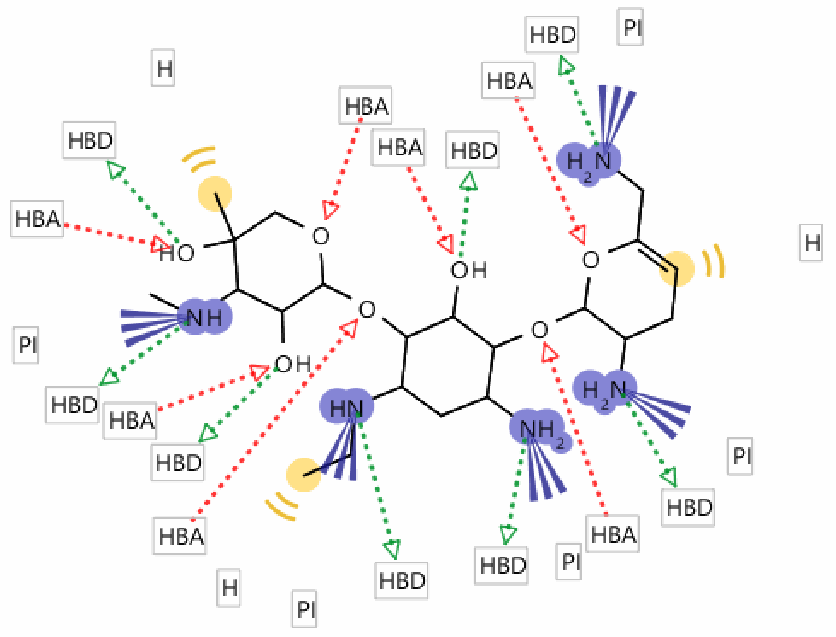
NK 1001	53025-93-7	NZCOZAMBHLSNDW-GUKOCFKPSA-N	InChI=1S/C18H35N3O12/c19-2-6-8(23)10(25)12(27)17(30-6)32-15-4(20)1-5(21)16(14(15)29)33-18-13(28)11(26)9(24)7(3-22)31-18/h4-18,22-29H,1-3,19-21H2/t4-,5+,6-,7-,8-,9-,10+,11+,12-,13-,14-,15+,16-,17-,18-/m1/s1	NC[C@@H]1[C@@H](O)[C@H](O)[C@@H](O)[C@](O1)(O[C@@]2([H])[C@H](N)C[C@H](N)[C@]([C@@H]2O)(O[C@]3([H])[C@H](O)[C@@H](O)[C@H](O)[C@H](O3)CO)[H])[H]	Small ribosomal subunit inhibitor	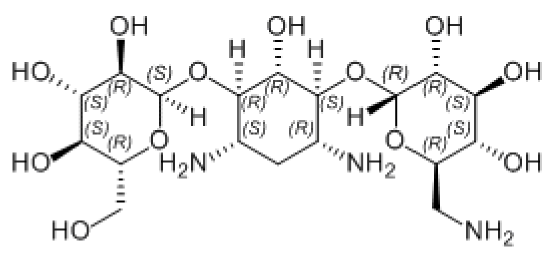	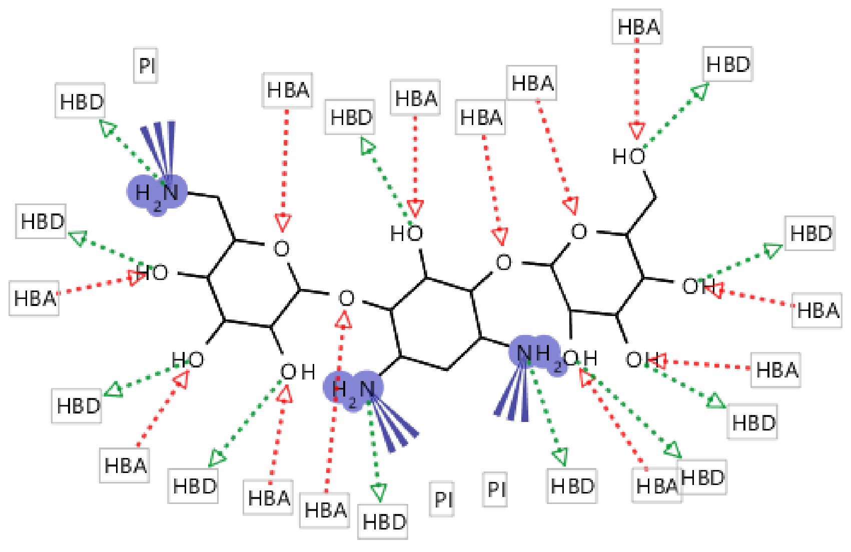

Common name	CAS Reg. No.	InChIKey	InChI	SMILES	Primary target	2D Structure	Pharmacophore[1]
Oxyapramycin (nebramycin VII)	56283-52-4	VDGAVKNERSZOPW-UZVIDTGHSA-N	InChI=1S/C21H41N5O12/c1-26-9-13(31)18-17(37-20(9)38-21-15(33)11(29)7(24)6(3-27)34-21)12(30)8(25)19(36-18)35-16-5(23)2-4(22)10(28)14(16)32/h4-21,26-33H,2-3,22-25H2,1H3/t4-,5+,6-,7-,8-,9+,10+,11+,12-,13-,14-,15-,16-,17+,18-,19+,20-,21-/m1/s1	CN[C@@H]1[C@]([H])(O[C@@]2([C@@](O[C@]([H])(O[C@]3([H])[C@@H](N)C[C@@H](N)[C@H](O)[C@H]3O)[C@@H]([C@H]2O)N)([H])[C@@H]1O)[H])O[C@]4([H])[C@H](O)[C@@H](O)[C@H](N)[C@H](O4)CO	Small ribosomal subunit inhibitor	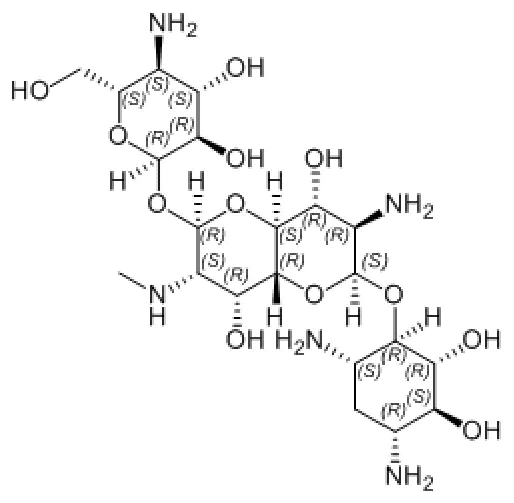	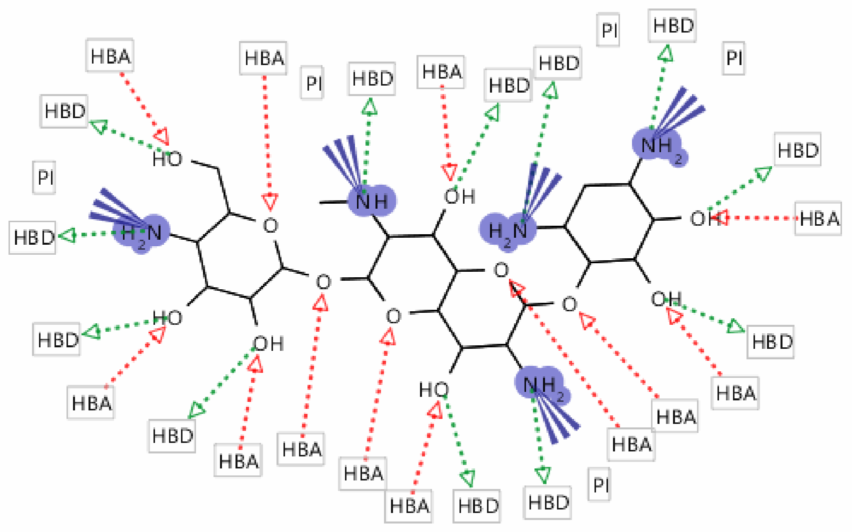
Paromamine (neomycin D)	534-47-4	JGSMDVGTXBPWIM-HKEUSBCWSA-N	InChI=1S/C12H25N3O7/c13-3-1-4(14)11(10(20)7(3)17)22-12-6(15)9(19)8(18)5(2-16)21-12/h3-12,16-20H,1-2,13-15H2/t3-,4+,5-,6-,7+,8-,9-,10-,11-,12-/m1/s1	N[C@@H]1C[C@@H]([C@]([H])([C@@H]([C@H]1O)O)O[C@@]2([C@@H]([C@H]([C@@H]([C@@H](CO)O2)O)O)N)[H])N	Small ribosomal subunit inhibitor	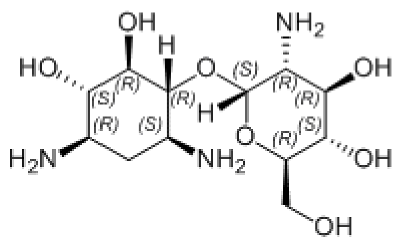	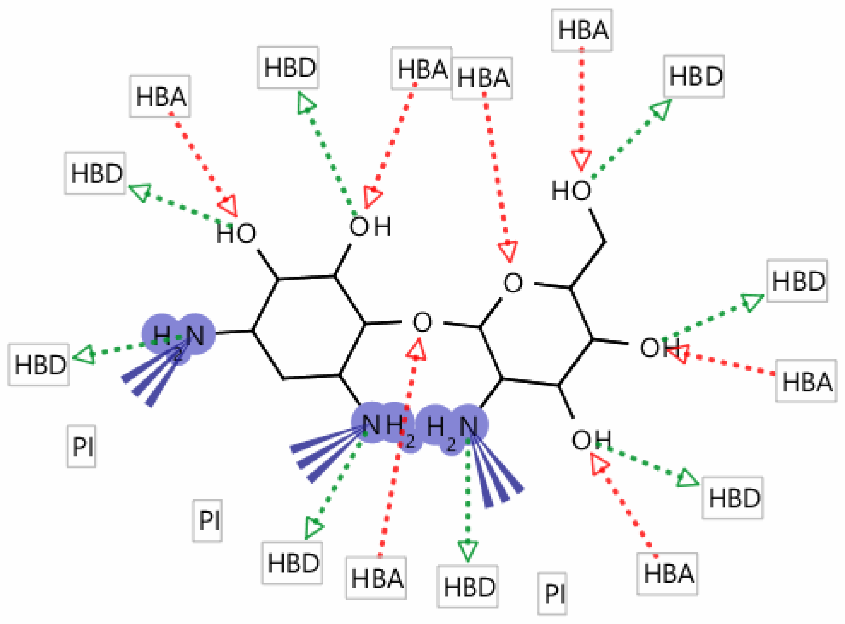
Paromomycin (paromomycin I; neomycin E)	7542-37-2	UOZODPSAJZTQNH-LSWIJEOBSA-N	InChI=1S/C23H45N5O14/c24-2-7-13(32)15(34)10(27)21(37-7)41-19-9(4-30)39-23(17(19)36)42-20-12(31)5(25)1-6(26)18(20)40-22-11(28)16(35)14(33)8(3-29)38-22/h5-23,29-36H,1-4,24-28H2/t5-,6+,7+,8-,9-,10-,11-,12+,13-,14-,15-,16-,17-,18-,19-,20-,21-,22-,23+/m1/s1	NC[C@H]1[C@H]([C@@H]([C@H]([C@]([H])(O1)O[C@H]2[C@H]([C@@H](O[C@@H]2CO)O[C@@]3([C@H]([C@@H](C[C@@H]([C@]3(O[C@@]4([C@@H]([C@H]([C@@H]([C@@H](CO)O4)O)O)N)[H])[H])N)N)O)[H])O)N)O)O	Small ribosomal subunit inhibitor	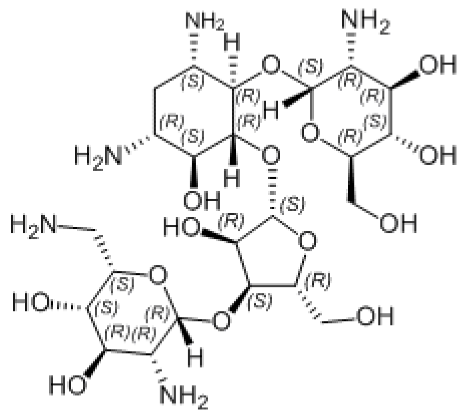	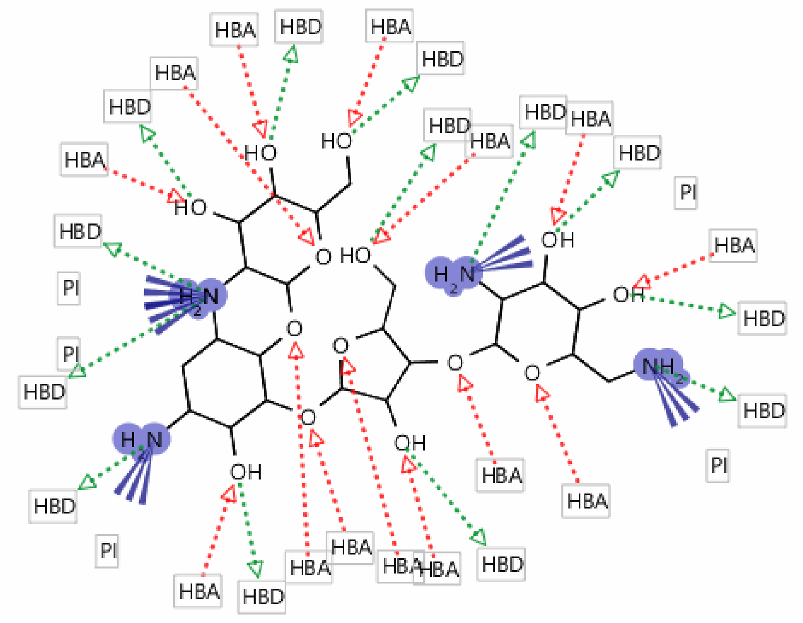
Pentisomicin	55870-64-9	URWAJWIAIPFPJE-VHLNBGGKSA-N	InChI=1S/C19H37N5O7/c1-19(27)7-28-18(13(26)16(19)24-2)31-15-11(23)5-10(22)14(12(15)25)30-17-9(21)4-3-8(6-20)29-17/h3,9-18,24-27H,4-7,20-23H2,1-2H3/t9-,10+,11-,12-,13-,14-,15+,16-,17-,18-,19+/m1/s1	C[C@]1(O)CO[C@@]([C@H](O)[C@H]1NC)(O[C@@]2([H])[C@H](N)C[C@H](N)[C@]([C@H]2O)(O[C@]3([H])[C@H](N)CC=C(O3)CN)[H])[H]	Small ribosomal subunit inhibitor	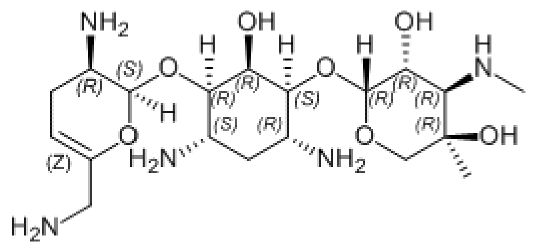	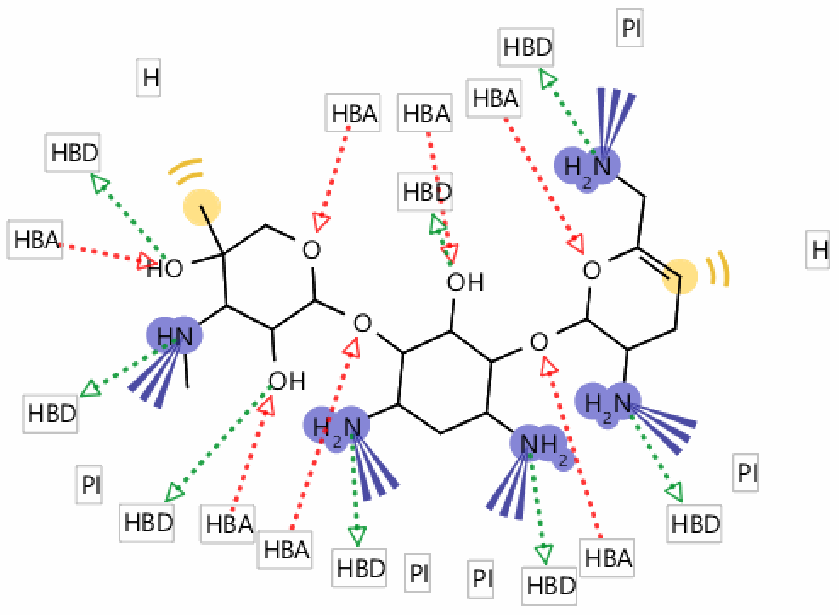
Plazomicin	1154757-24-0	IYDYFVUFSPQPPV-PEXOCOHZSA-N	InChI=1S/C25H48N6O10/c1-25(37)11-38-24(18(35)21(25)29-2)41-20-15(31-22(36)16(33)5-6-26)9-14(28)19(17(20)34)40-23-13(27)4-3-12(39-23)10-30-7-8-32/h3,13-21,23-24,29-30,32-35,37H,4-11,26-28H2,1-2H3,(H,31,36)/t13-,14+,15-,16+,17+,18-,19-,20+,21-,23-,24-,25+/m1/s1	C[C@@]1(CO[C@@]([H])([C@@H]([C@H]1NC)O)O[C@]2([C@@]([H])(C[C@@H]([C@]([H])([C@@H]2O)O[C@@]3([C@@H](CC=C(CNCCO)O3)N)[H])N)NC([C@H](CCN)O)=O)[H])O	Small ribosomal subunit inhibitor	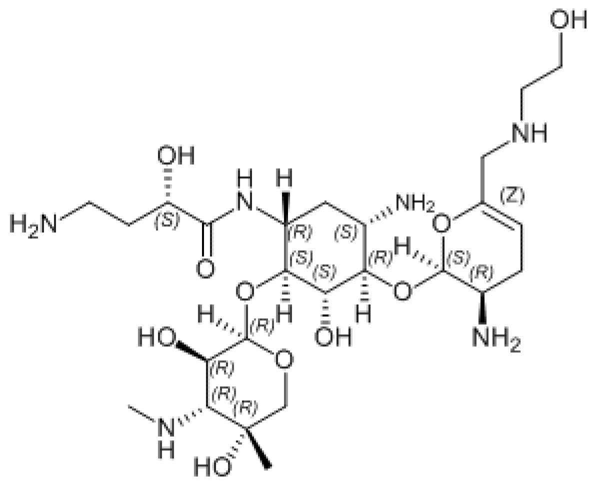	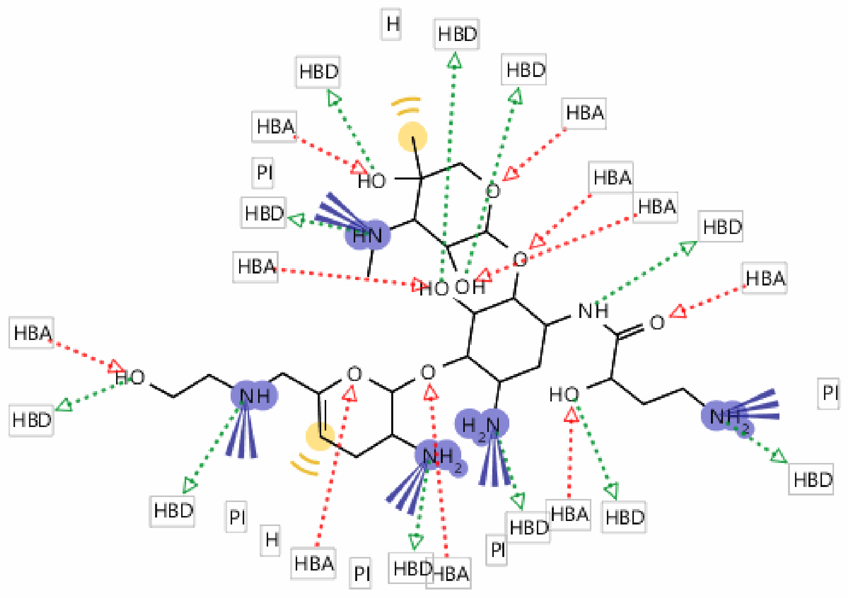
Propikacin	66887-96-5	ZEFUFVWPRPISAD-FLUPTLLOSA-N	InChI=1S/C21H43N5O12/c22-2-9-14(31)15(32)12(25)20(35-9)37-18-7(23)1-8(26-6(3-27)4-28)19(17(18)34)38-21-16(33)11(24)13(30)10(5-29)36-21/h6-21,26-34H,1-5,22-25H2/t7-,8+,9+,10+,11-,12+,13+,14+,15+,16+,17-,18+,19-,20+,21+/m0/s1	NC[C@@H]1[C@@H](O)[C@H](O)[C@@H](N)[C@](O1)(O[C@]2([H])[C@@H](N)C[C@]([C@@]([C@H]2O)(O[C@]3([H])[C@H](O)[C@@H](N)[C@H](O)[C@H](O3)CO)[H])(NC(CO)CO)[H])[H]	Small ribosomal subunit inhibitor	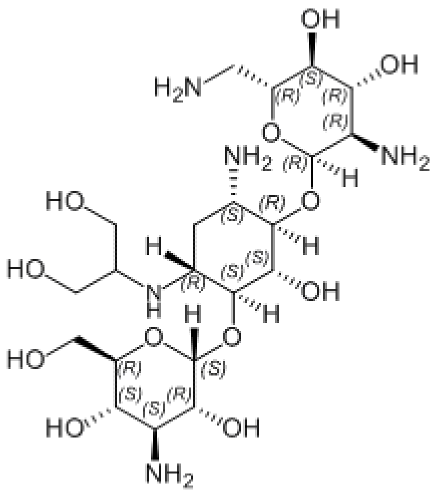	

Common name	CAS Reg. No.	InChIKey	InChI	SMILES	Primary target	2D Structure	Pharmacophore[1]
Pyrankacin		XUIOIKYDMYXBFJ-ZWQGTLNRSA-N	InChI=1S/C22H44N6O9/c1-8-14(27)16(31)17(32)22(34-8)37-19-15(30)12(28-20(33)13(29)4-5-23)6-11(26)18(19)36-21-10(25)3-2-9(7-24)35-21/h8-19,21-22,29-32H,2-7,23-27H2,1H3,(H,28,33)/t8-,9+,10-,11+,12-,13+,14-,15+,16+,17-,18-,19-,21-,22+/m1/s1	C[C@@H]1[C@H]([C@@H]([C@H]([C@@]([H])(O1)O[C@@]2([C@H]([C@@]([H])(C[C@@H]([C@]2(O[C@@]3([C@@H](CC[C@@H](CN)O3)N)[H])[H])N)NC([C@H](CCN)O)=O)O)[H])O)O)N	Small ribosomal subunit inhibitor	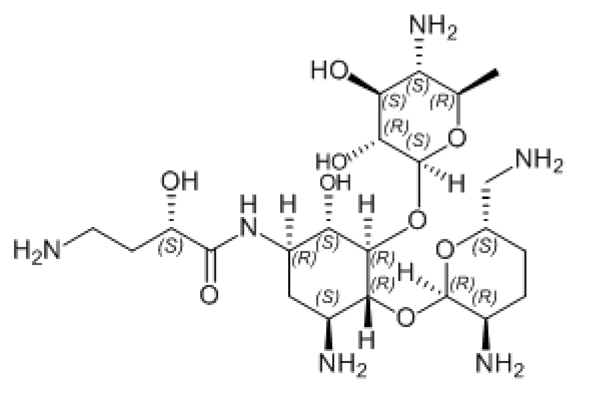	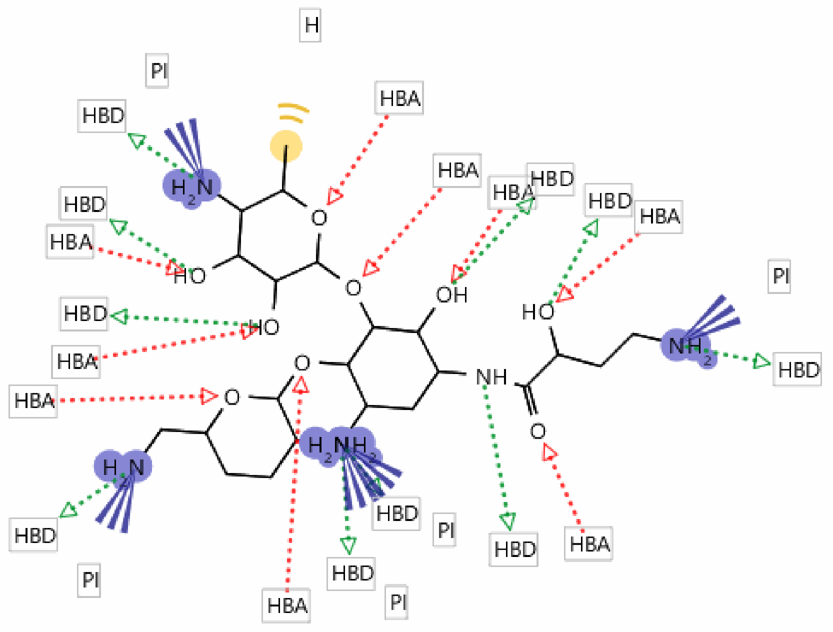
Ribostamycin	25546-65-0	NSKGQURZWSPSBC-VVPCINPTSA-N	InChI=1S/C17H34N4O10/c18-2-6-10(24)12(26)8(21)16(28-6)30-14-5(20)1-4(19)9(23)15(14)31-17-13(27)11(25)7(3-22)29-17/h4-17,22-27H,1-3,18-21H2/t4-,5+,6-,7-,8-,9+,10-,11-,12-,13-,14-,15-,16-,17+/m1/s1	NC[C@@H]1[C@H]([C@@H]([C@H]([C@]([H])(O1)O[C@@]2([C@H](C[C@H]([C@@H]([C@]2(O[C@H]3[C@@H]([C@@H]([C@@H](CO)O3)O)O)[H])O)N)N)[H])N)O)O	Small ribosomal subunit inhibitor	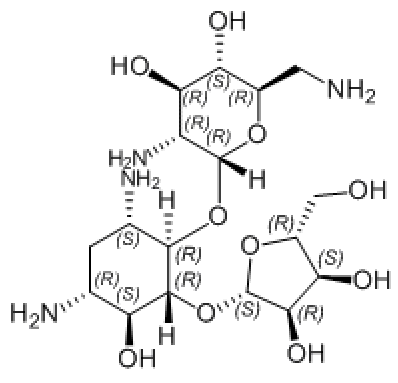	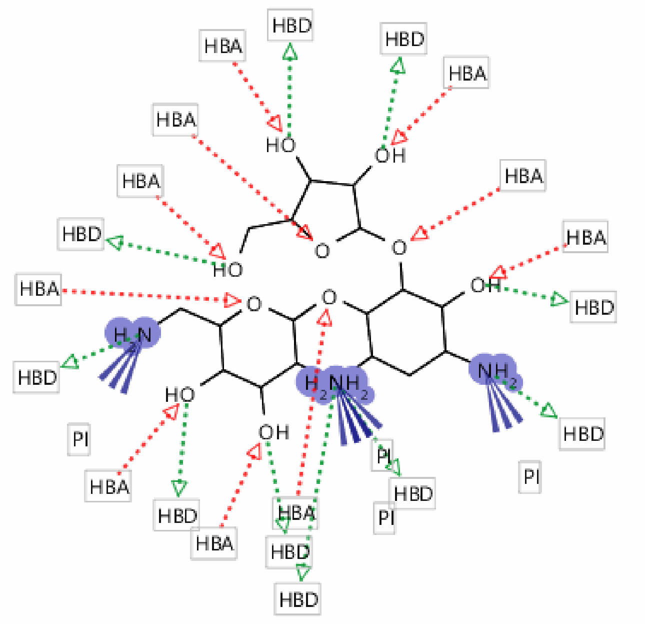
Saccharocin (KA 5685)	86630-31-1	WKKBQRRWYZAXFF-VJCYLLSFSA-N	InChI=1S/C21H40N4O12/c1-25-10-13(29)18-8(33-20(10)37-21-16(32)14(30)12(28)9(4-26)34-21)3-7(24)19(36-18)35-17-6(23)2-5(22)11(27)15(17)31/h5-21,25-32H,2-4,22-24H2,1H3/t5-,6+,7-,8+,9-,10+,11+,12-,13-,14+,15-,16-,17-,18+,19+,20-,21-/m1/s1	CN[C@@H]1[C@](O[C@@]2([C@@]([H])([C@@H]1O)O[C@](O[C@]3([H])[C@@H](N)C[C@@H](N)[C@H](O)[C@H]3O)([C@@H](C2)N)[H])[H])(O[C@]4([H])[C@H](O)[C@@H](O)[C@H](O)[C@H](O4)CO)[H]	Small ribosomal subunit inhibitor	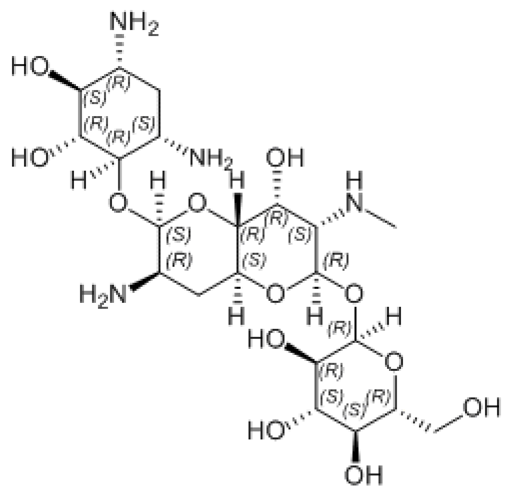	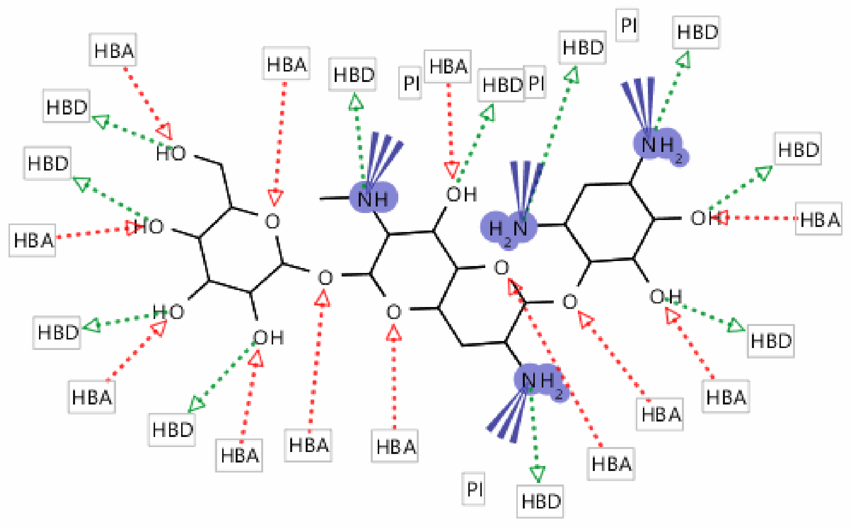
Sannamycin C	73522-71-1	GKYYNFPFPFRFFN-YPPZSDNLSA-N	InChI=1S/C15H32N4O4/c1-18-7-8-4-5-9(16)15(22-8)23-14-10(17)6-11(21-3)12(19-2)13(14)20/h8-15,18-20H,4-7,16-17H2,1-3H3/t8-,9+,10-,11+,12+,13+,14+,15+/m0/s1	CNC[C@@]1(CC[C@H]([C@]([H])(O1)O[C@@]2([C@H](C[C@H]([C@H]([C@H]2O)NC)OC)N)[H])N)[H]	Small ribosomal subunit inhibitor	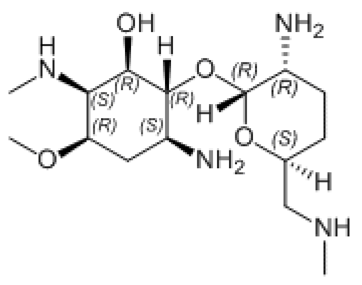	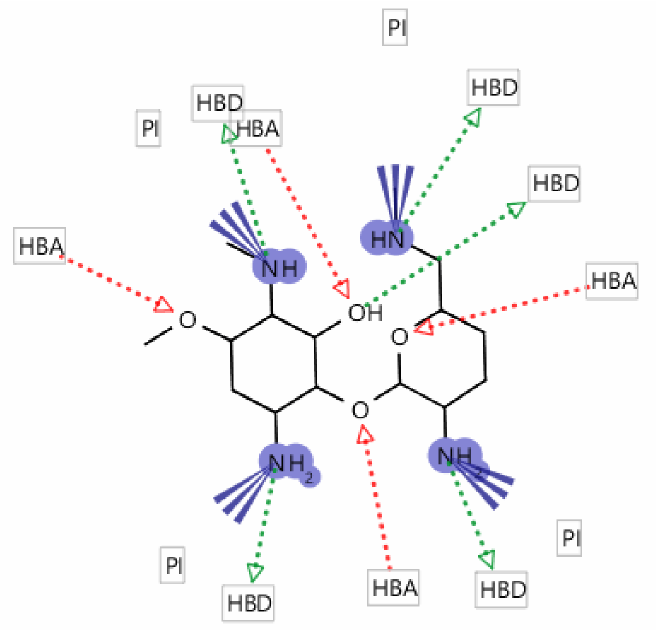
Sannamycin F	83931-91-3	NKRBCHAZRNKYCR-UMFUCVETSA-N	InChI=1S/C18H35N5O6/c1-20-7-10-4-5-12(23-14(25)8-22-9-24)18(28-10)29-17-11(19)6-13(27-3)15(21-2)16(17)26/h9-13,15-18,20-21,26H,4-8,19H2,1-3H3,(H,22,24)(H,23,25)/t10-,11-,12+,13+,15+,16+,17+,18+/m0/s1	CNC[C@@]1(CC[C@]([H])([C@]([H])(O1)O[C@@]2([C@H](C[C@H]([C@H]([C@H]2O)NC)OC)N)[H])NC(CNC=O)=O)[H]	Small ribosomal subunit inhibitor	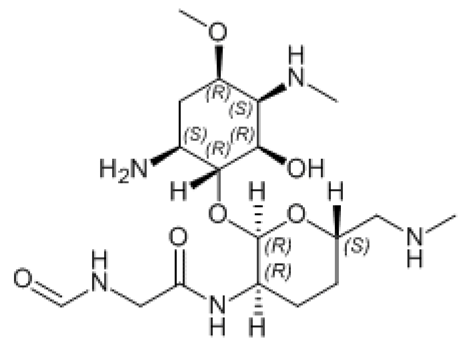	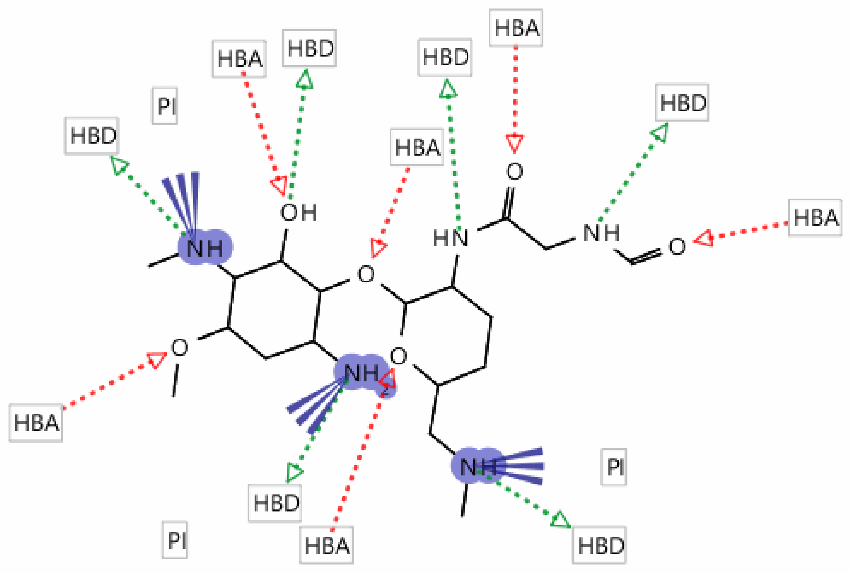
Sannamycin J	83997-42-6	LVWCJJGPEHPHJN-ZRXDKONOSA-N	InChI=1S/C14H30N4O4/c1-18-11-10(20-2)5-9(17)13(12(11)19)22-14-8(16)4-3-7(6-15)21-14/h7-14,18-19H,3-6,15-17H2,1-2H3/t7-,8+,9-,10+,11+,12+,13+,14+/m0/s1	CN[C@@H]1[C@@H](C[C@@H]([C@]([H])([C@@H]1O)O[C@@]2([C@@H](CC[C@@H](CN)O2)N)[H])N)OC	Small ribosomal subunit inhibitor	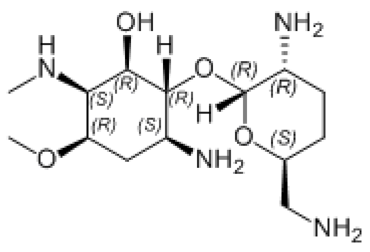	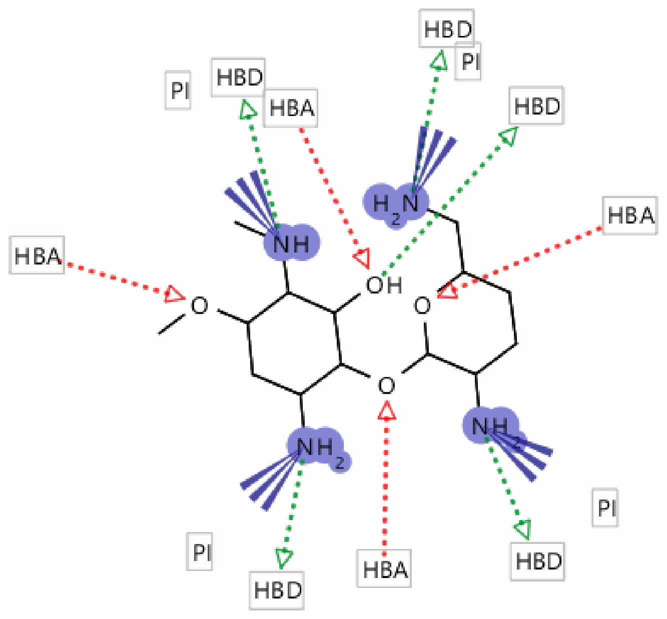

Common name	CAS Reg. No.	InChIKey	InChI	SMILES	Primary target	2D Structure	Pharmacophore[1]
Sannamycin K	83919-30-6	RVSJRSRXTISNGT-NKVKSULBSA-N	InChI=1S/C13H26N4O4/c1-17-10-9(18)4-8(16)12(11(10)19)21-13-7(15)3-2-6(5-14)20-13/h2,7-13,17-19H,3-5,14-16H2,1H3/t7-,8+,9-,10+,11-,12-,13-/m1/s1	CN[C@H]1[C@@H](C[C@@H]([C@]([H])([C@@H]1O)O[C@@]2([C@@H](CC=C(CN)O2)N)[H])N)O	Small ribosomal subunit inhibitor	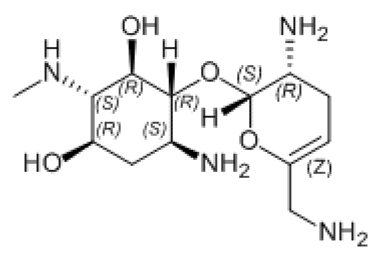	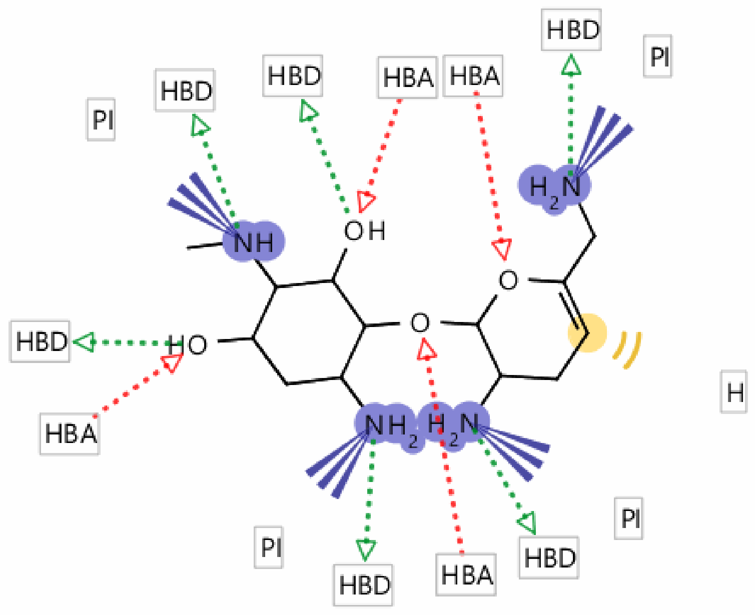
Sannamycin KR		GKYYNFPFPFRFFN-YAAFGFCESA-N	InChI=1S/C15H32N4O4/c1-18-7-8-4-5-9(16)15(22-8)23-14-10(17)6-11(21-3)12(19-2)13(14)20/h8-15,18-20H,4-7,16-17H2,1-3H3/t8-,9+,10-,11-,12-,13+,14+,15+/m0/s1	CNC[C@@]1(CC[C@H]([C@]([H])(O1)O[C@@]2([C@H](C[C@@H]([C@@H]([C@H]2O)NC)OC)N)[H])N)[H]	Small ribosomal subunit inhibitor	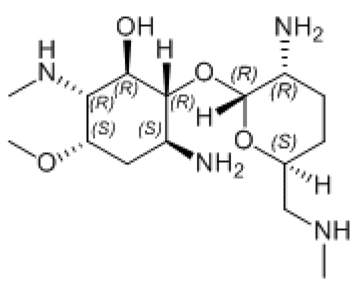	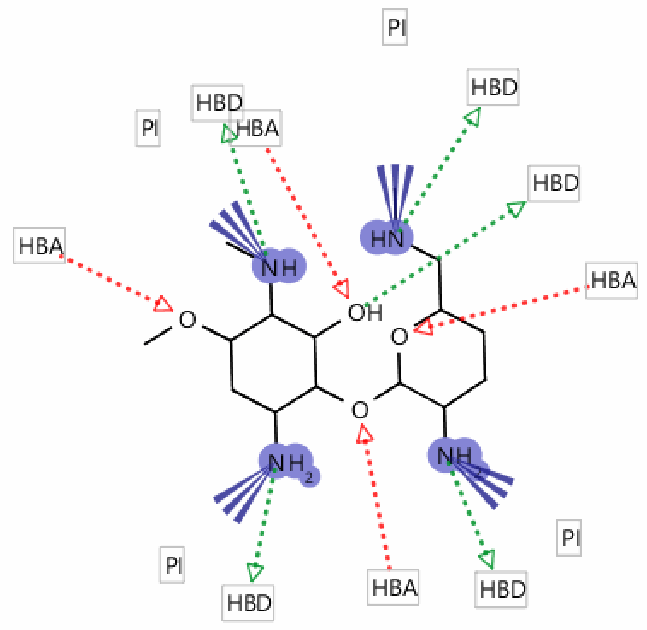
Sannamycin L	83946-32-1	RHKAQLAIYZGWPT-VXXVKXLQSA-N	InChI=1S/C12H26N4O4/c13-4-5-1-2-6(14)12(19-5)20-11-7(15)3-8(17)9(16)10(11)18/h5-12,17-18H,1-4,13-16H2/t5-,6+,7-,8+,9-,10+,11+,12+/m0/s1	NC[C@@H]1CC[C@H]([C@]([H])(O1)O[C@@]2([C@H](C[C@H]([C@@H]([C@H]2O)N)O)N)[H])N	Small ribosomal subunit inhibitor	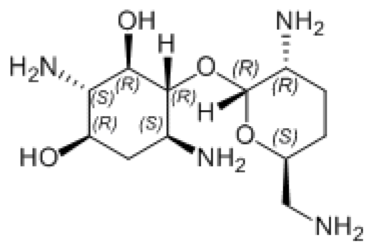	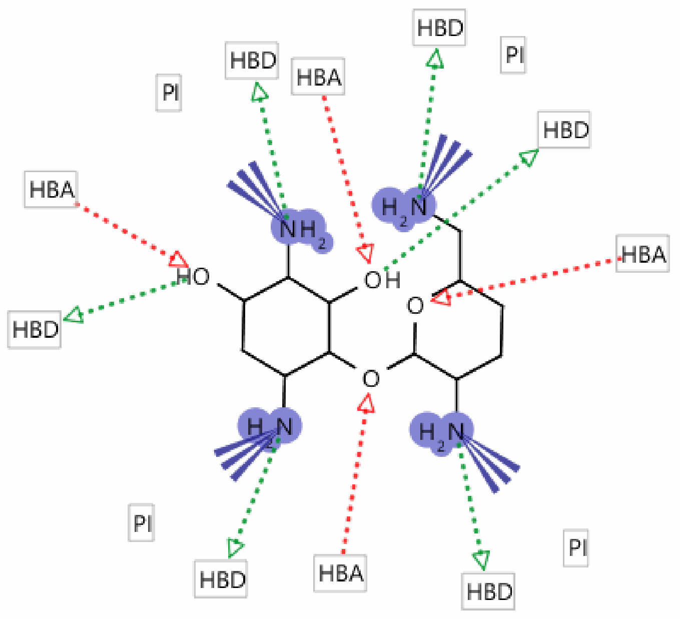
Seldomycin	75635-18-6	HJKXMQLJTKUBMG-INVUDJPSSA-N	InChI=1S/C18H38N6O7/c1-27-10-5-28-17(13(24)12(10)23)30-15-7(20)3-8(21)16(14(15)26)31-18-11(22)9(25)2-6(4-19)29-18/h6-18,25-26H,2-5,19-24H2,1H3/t6-,7+,8-,9-,10+,11+,12-,13-,14+,15-,16+,17+,18+/m0/s1	CO[C@@H]1CO[C@@]([H])([C@H]([C@H]1N)N)O[C@]2([C@@H](C[C@@H]([C@]([H])([C@@H]2O)O[C@@]3([C@@H]([C@H](C[C@@H](CN)O3)O)N)[H])N)N)[H]	Small ribosomal subunit inhibitor	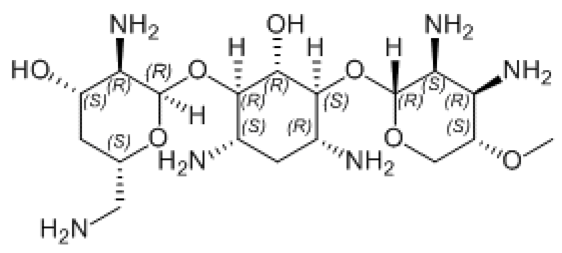	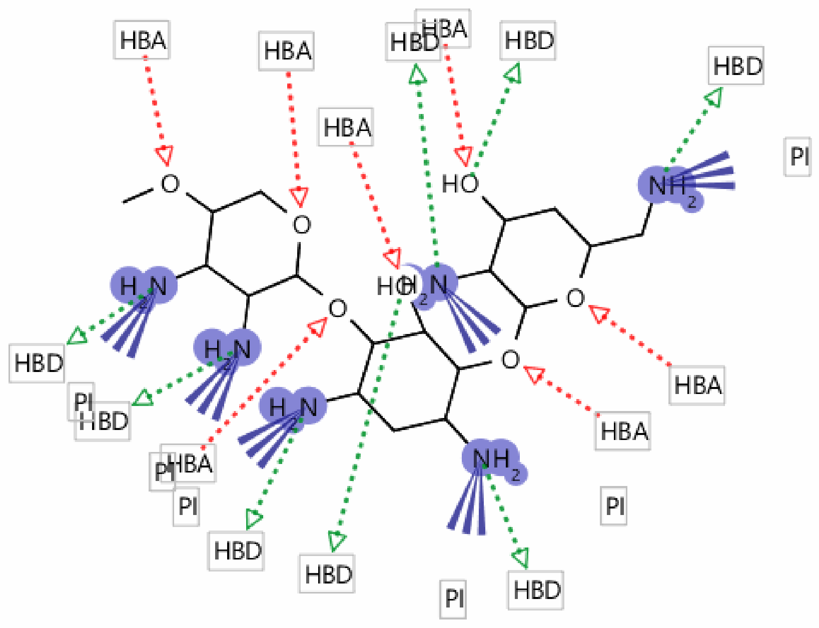
Seldomycin 1 (seldomycin factor 1)	56276-04-1	YNMRKGLUALDHIR-PZQDWFEXSA-N	InChI=1S/C17H34N4O10/c18-4-1-5(19)15(31-17-9(21)12(26)11(25)7(2-22)29-17)13(27)14(4)30-16-8(20)10(24)6(23)3-28-16/h4-17,22-27H,1-3,18-21H2/t4-,5+,6-,7-,8-,9-,10+,11-,12-,13-,14+,15-,16-,17-/m1/s1	N[C@@H]1C[C@@H]([C@]([H])([C@@H]([C@]1(O[C@@]2([C@@H]([C@H]([C@@H](CO2)O)O)N)[H])[H])O)O[C@@]3([C@@H]([C@H]([C@@H]([C@@H](CO)O3)O)O)N)[H])N	Small ribosomal subunit inhibitor	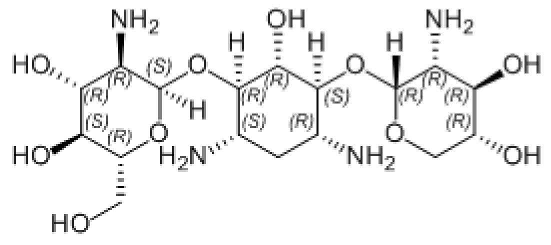	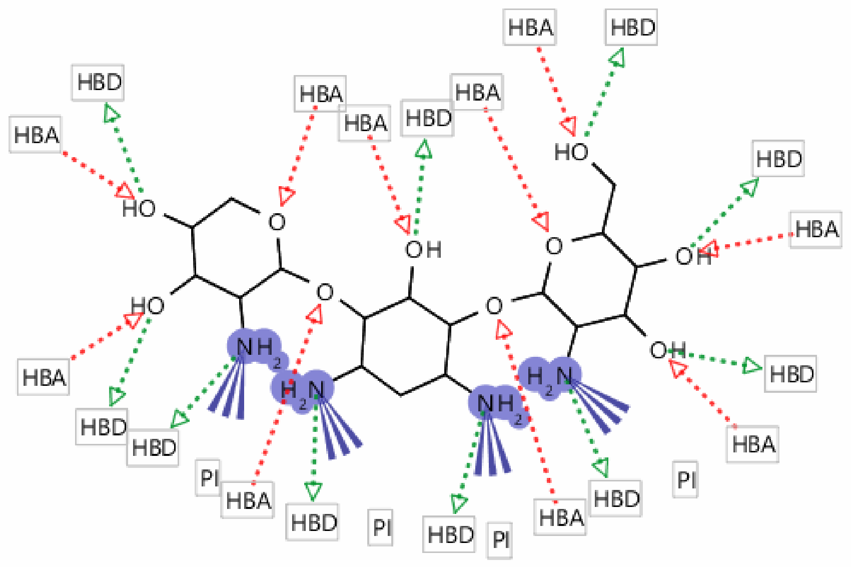
Seldomycin 2 (seldomycin factor 2)	54333-78-7	NOBINJIDZDKGAE-GVYSDAGYSA-N	InChI=1S/C12H26N4O5/c13-3-4-1-7(17)8(16)12(20-4)21-11-6(15)2-5(14)9(18)10(11)19/h4-12,17-19H,1-3,13-16H2/t4-,5+,6-,7-,8+,9-,10+,11+,12+/m0/s1	NC[C@@H]1C[C@@H]([C@H]([C@]([H])(O1)O[C@@]2([C@H](C[C@H]([C@@H]([C@H]2O)O)N)N)[H])N)O	Small ribosomal subunit inhibitor	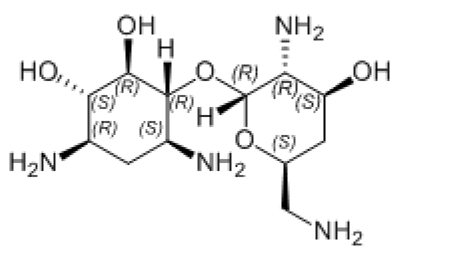	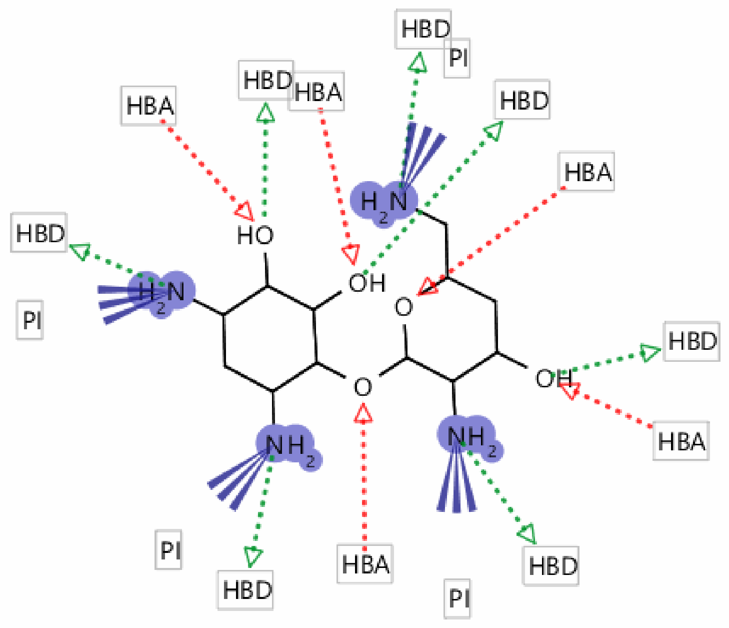

Common name	CAS Reg. No.	InChIKey	InChI	SMILES	Primary target	2D Structure	Pharmacophore[1]
Seldomycin 3 (seldomycin factor 3)	56276-05-2	ZAOUGJBZKPMBDW-PZQDWFEXSA-N	InChI=1S/C17H35N5O9/c18-2-7-11(25)12(26)9(22)17(29-7)31-15-5(20)1-4(19)14(13(15)27)30-16-8(21)10(24)6(23)3-28-16/h4-17,23-27H,1-3,18-22H2/t4-,5+,6-,7-,8-,9-,10+,11-,12-,13-,14+,15-,16-,17-/m1/s1	NC[C@@H]1[C@H]([C@@H]([C@H]([C@]([H])(O1)O[C@@]2([C@H](C[C@H]([C@@]([H])([C@H]2O)O[C@@]3([C@@H]([C@H]([C@@H](CO3)O)O)N)[H])N)N)[H])N)O)O	Small ribosomal subunit inhibitor	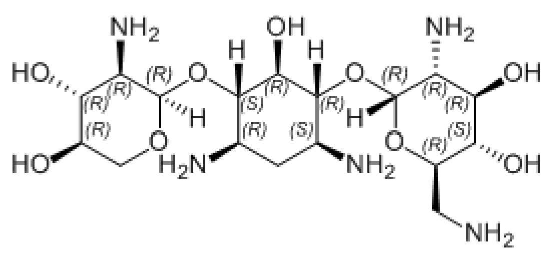	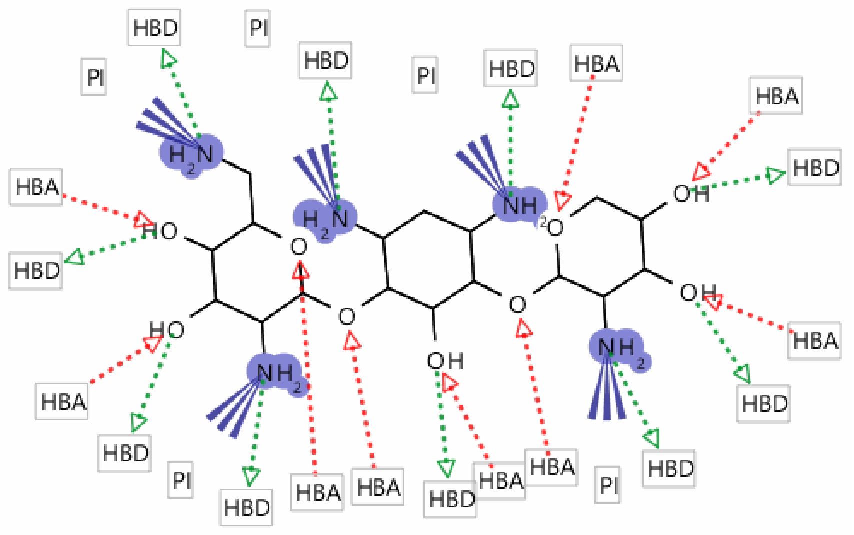
Seldomycin 5 (seldomycin factor 5)	56276-26-7	HJKXMQLJTKUBMG-BAOVJYBRSA-N	InChI=1S/C18H38N6O7/c1-27-10-5-28-17(13(24)12(10)23)30-15-7(20)3-8(21)16(14(15)26)31-18-11(22)9(25)2-6(4-19)29-18/h6-18,25-26H,2-5,19-24H2,1H3/t6-,7+,8-,9-,10+,11+,12-,13+,14+,15-,16+,17+,18+/m0/s1	CO[C@@H]1CO[C@@]([H])([C@@H]([C@H]1N)N)O[C@]2([C@@H](C[C@@H]([C@]([H])([C@@H]2O)O[C@@]3([C@@H]([C@H](C[C@@H](CN)O3)O)N)[H])N)N)[H]	Small ribosomal subunit inhibitor	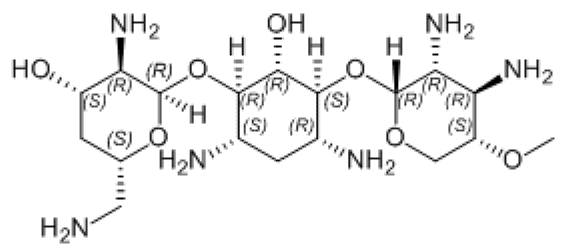	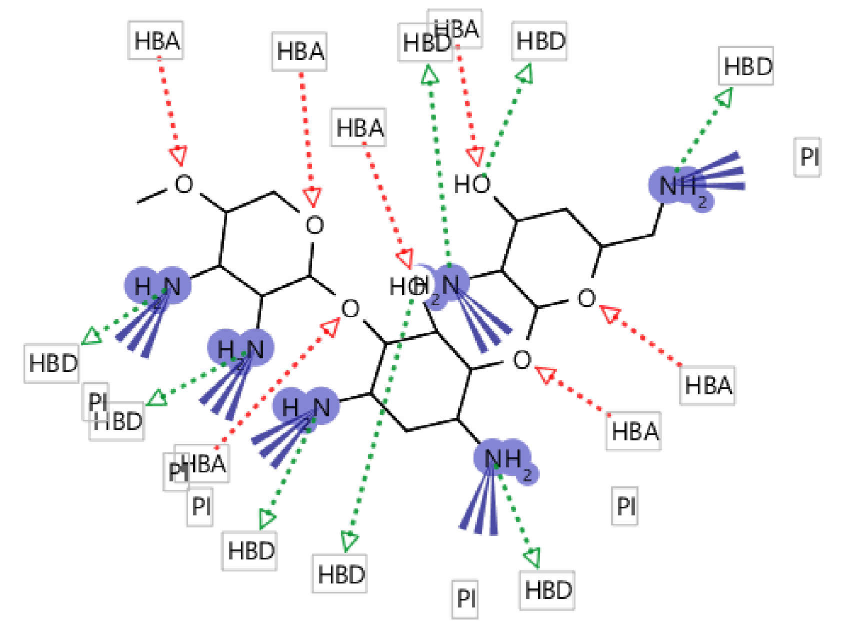
Sisomicin	32385-11-8	URWAJWIAIPFPJE-YFMIWBNJSA-N	InChI=1S/C19H37N5O7/c1-19(27)7-28-18(13(26)16(19)24-2)31-15-11(23)5-10(22)14(12(15)25)30-17-9(21)4-3-8(6-20)29-17/h3,9-18,24-27H,4-7,20-23H2,1-2H3/t9-,10+,11-,12+,13-,14-,15+,16-,17-,18-,19+/m1/s1	C[C@@]1(CO[C@@]([H])([C@@H]([C@H]1NC)O)O[C@]2([C@@H](C[C@@H]([C@]([H])([C@@H]2O)O[C@@]3([C@@H](CC=C(CN)O3)N)[H])N)N)[H])O	Small ribosomal subunit inhibitor	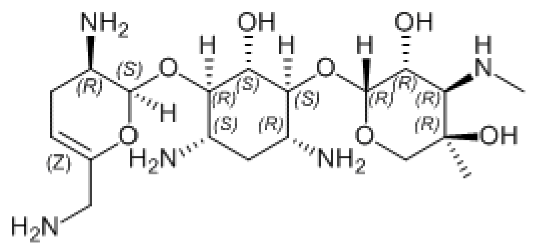	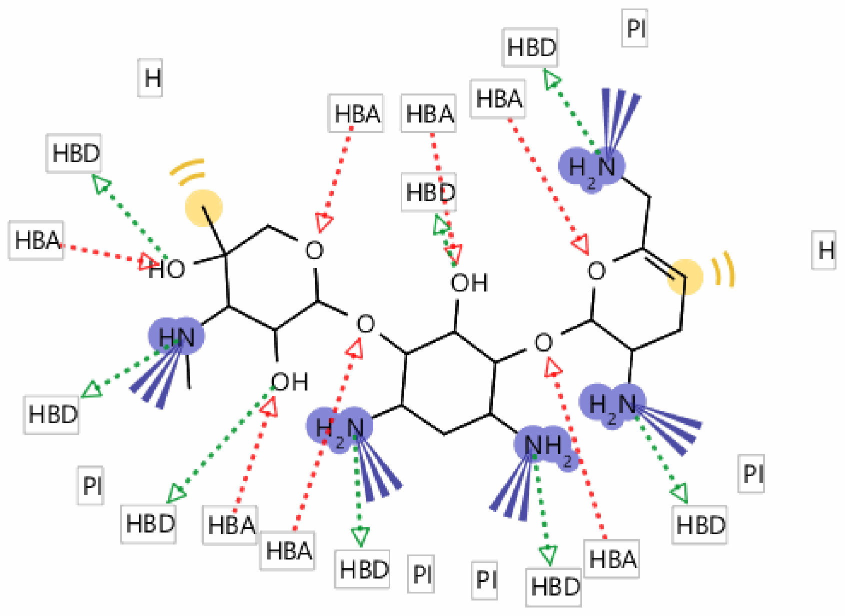
Sisomicin B	53797-16-3	DAKDDLIZULPEFW-WXGYIBDGSA-N	InChI=1S/C18H35N5O7/c1-23-12-11(24)6-27-18(13(12)25)30-16-10(22)4-9(21)15(14(16)26)29-17-8(20)3-2-7(5-19)28-17/h2,8-18,23-26H,3-6,19-22H2,1H3/t8-,9+,10-,11-,12+,13-,14+,15-,16+,17-,18-/m1/s1	CN[C@H]1[C@@H](CO[C@@]([H])([C@@H]1O)O[C@]2([C@@H](C[C@@H]([C@]([H])([C@@H]2O)O[C@@]3([C@@H](CC=C(CN)O3)N)[H])N)N)[H])O	Small ribosomal subunit inhibitor	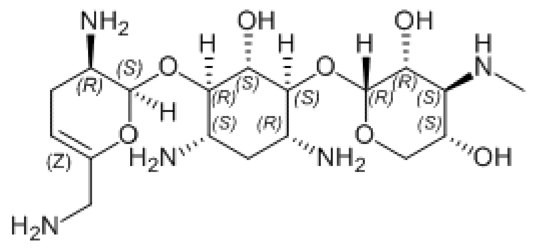	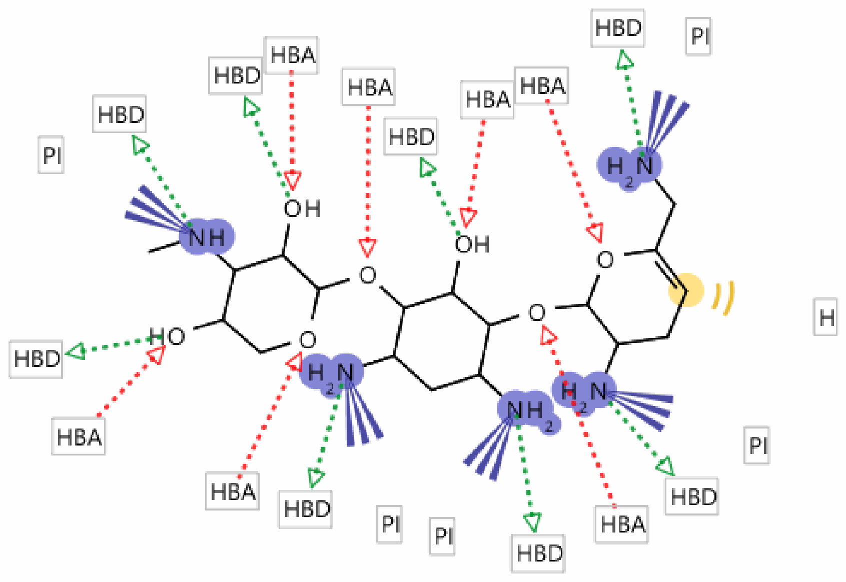
Sisomicin D	53759-50-5	DAKDDLIZULPEFW-JVRQZOTMSA-N	InChI=1S/C18H35N5O7/c1-23-12-11(24)6-27-18(13(12)25)30-16-10(22)4-9(21)15(14(16)26)29-17-8(20)3-2-7(5-19)28-17/h2,8-18,23-26H,3-6,19-22H2,1H3/t8-,9+,10-,11+,12+,13-,14+,15-,16+,17-,18-/m1/s1	CN[C@H]1[C@H](CO[C@@]([H])([C@@H]1O)O[C@]2([C@@H](C[C@@H]([C@]([H])([C@@H]2O)O[C@@]3([C@@H](CC=C(CN)O3)N)[H])N)N)[H])O	Small ribosomal subunit inhibitor	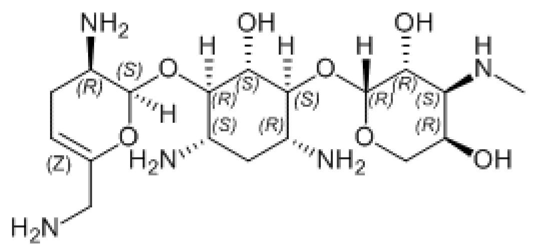	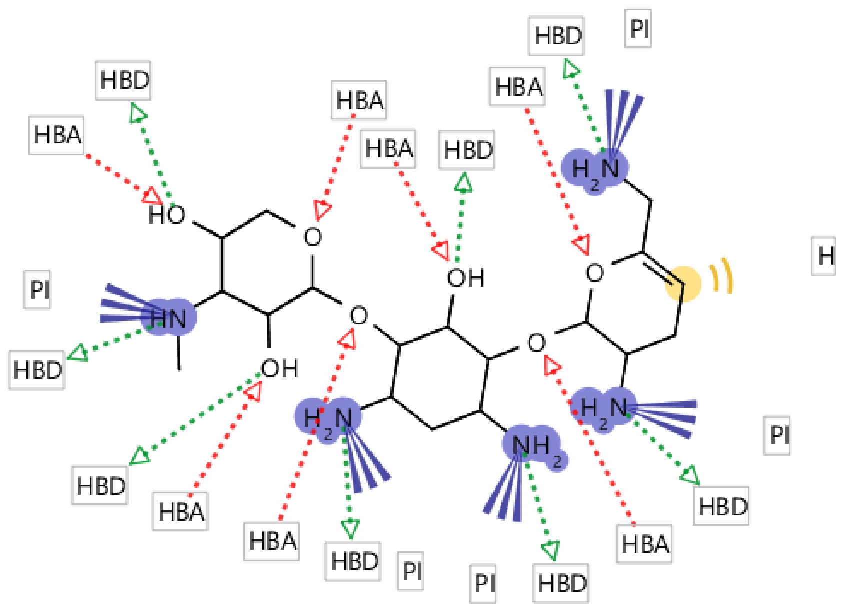
Spectinomycin	1695-77-8	UNFWWIHTNXNPBV-WXKVUWSESA-N	InChI=1S/C14H24N2O7/c1-5-4-6(17)14(20)13(21-5)22-12-10(19)7(15-2)9(18)8(16-3)11(12)23-14/h5,7-13,15-16,18-20H,4H2,1-3H3/t5-,7-,8+,9+,10+,11-,12-,13+,14+/m1/s1	C[C@@H]1CC([C@]2([C@@]([H])(O1)O[C@@]3([C@H]([C@@H]([C@@H]([C@@H]([C@]3(O2)[H])NC)O)NC)O)[H])O)=O	Small ribosomal subunit inhibitor	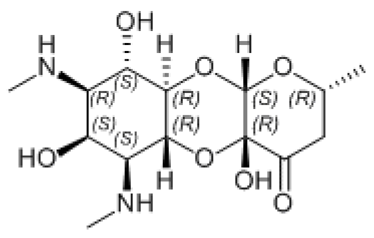	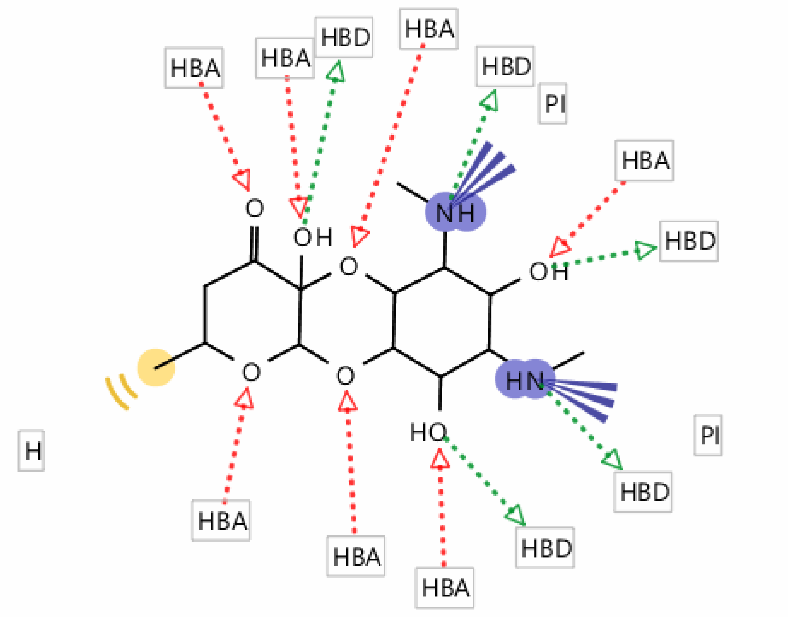

Common name	CAS Reg. No.	InChIKey	InChI	SMILES	Primary target	2D Structure	Pharmacophore[1]
Sporaricin A	68743-79-3	VMUGJEXITMIYRW-WQCXHKNXSA-N	InChI=1S/C17H35N5O5/c1-8(19)11-5-4-9(20)17(26-11)27-16-10(21)6-12(25-3)14(15(16)24)22(2)13(23)7-18/h8-12,14-17,24H,4-7,18-21H2,1-3H3/t8-,9+,10+,11-,12-,14+,15+,16+,17+/m0/s1	C[C@H](N)[C@]1([H])CC[C@@H](N)[C@]([H])(O1)O[C@]2([H])[C@H](N)C[C@H](OC)[C@]([H])([C@H]2O)N(C(CN)=O)C	Small ribosomal subunit inhibitor	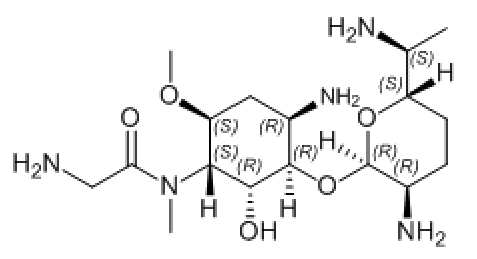	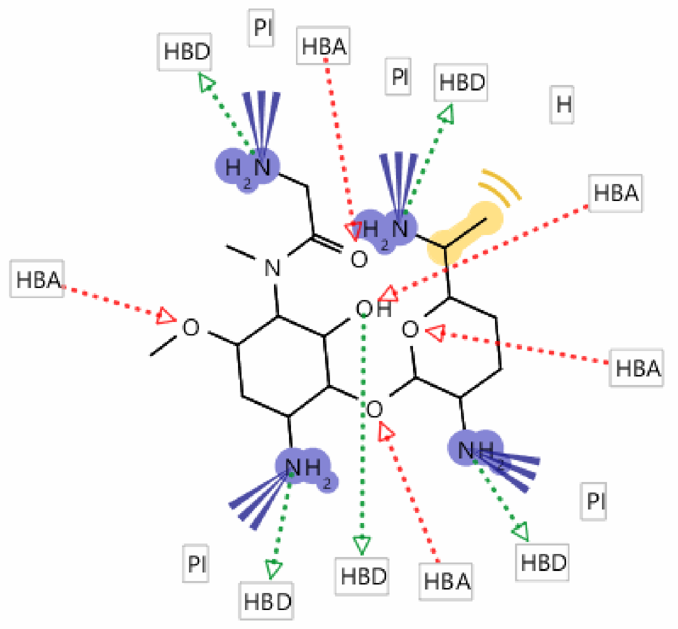
Sporaricin B	68743-78-2	YGRYQFMCQIZSDG-SSHYVVCRSA-N	InChI=1S/C15H32N4O4/c1-7(16)10-5-4-8(17)15(22-10)23-14-9(18)6-11(21-3)12(19-2)13(14)20/h7-15,19-20H,4-6,16-18H2,1-3H3/t7-,8+,9+,10-,11-,12+,13+,14+,15+/m0/s1	C[C@@H]([C@@]1(CC[C@H]([C@]([H])(O1)O[C@@]2([C@@H](C[C@@H]([C@H]([C@H]2O)NC)OC)N)[H])N)[H])N	Small ribosomal subunit inhibitor	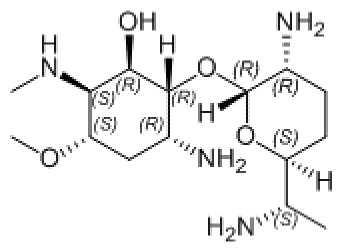	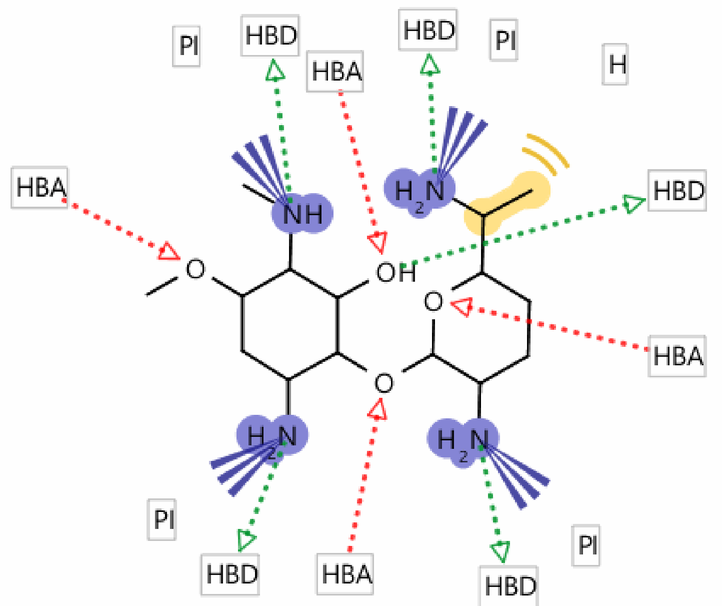
Sporaricin C	68743-80-6	PUURLTDHGIJACO-WQCXHKNXSA-N	InChI=1S/C18H36N6O6/c1-8(19)11-5-4-9(20)17(29-11)30-16-10(21)6-12(28-3)14(15(16)26)24(2)13(25)7-23-18(22)27/h8-12,14-17,26H,4-7,19-21H2,1-3H3,(H3,22,23,27)/t8-,9+,10+,11-,12-,14+,15+,16+,17+/m0/s1	C[C@H](N)[C@]1([H])CC[C@@H](N)[C@](O1)(O[C@]2([H])[C@H](N)C[C@H](OC)[C@]([C@H]2O)(N(C(CNC(N)=O)=O)C)[H])[H]	Small ribosomal subunit inhibitor	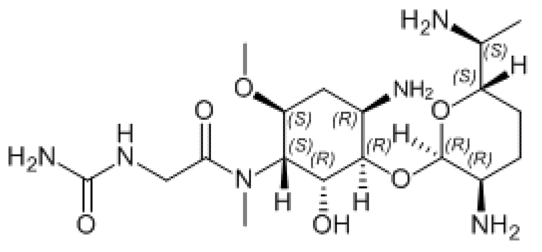	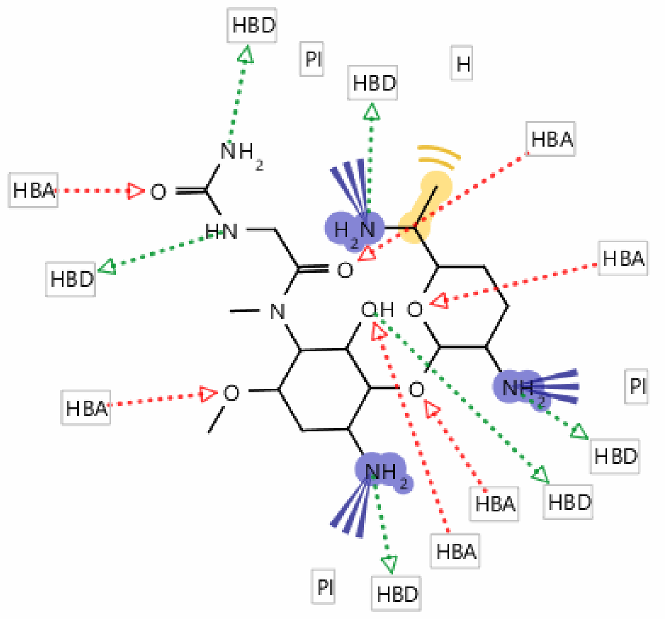
Sporaricin D	68743-81-7	VFIPOZSOMJAAJD-QKRADUSQSA-N	InChI=1S/C18H35N5O6/c1-9(19)12-5-4-10(20)18(28-12)29-17-11(21)6-13(27-3)15(16(17)26)23(2)14(25)7-22-8-24/h8-13,15-18,26H,4-7,19-21H2,1-3H3,(H,22,24)/t9-,10+,11+,12-,13-,15+,16+,17+,18+/m0/s1	C[C@H](N)[C@]1([H])CC[C@@H](N)[C@]([H])(O1)O[C@]2([H])[C@H](N)C[C@H](OC)[C@]([H])([C@H]2O)N(C(CNC=O)=O)C	Small ribosomal subunit inhibitor	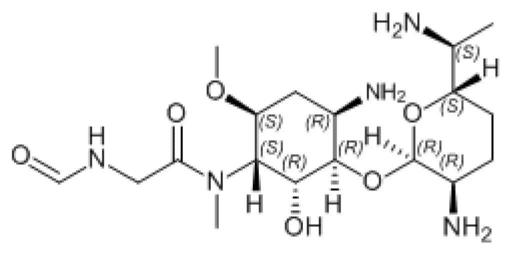	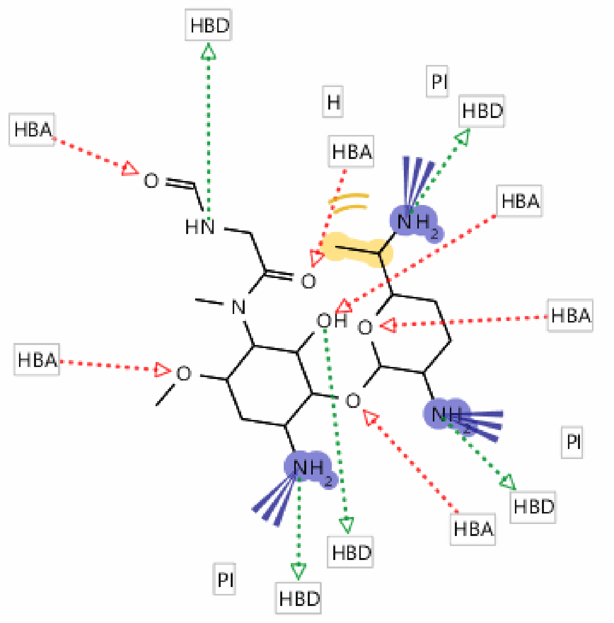
Sporaricin E	71657-28-8	YGRYQFMCQIZSDG-RSAZALMYSA-N	InChI=1S/C15H32N4O4/c1-7(16)10-5-4-8(17)15(22-10)23-14-9(18)6-11(21-3)12(19-2)13(14)20/h7-15,19-20H,4-6,16-18H2,1-3H3/t7-,8+,9-,10-,11-,12+,13+,14+,15+/m0/s1	C[C@@H]([C@@]1(CC[C@H]([C@]([H])(O1)O[C@@]2([C@H](C[C@@H]([C@H]([C@H]2O)NC)OC)N)[H])N)[H])N	Small ribosomal subunit inhibitor	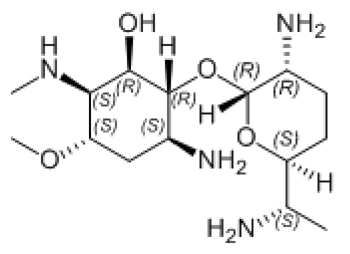	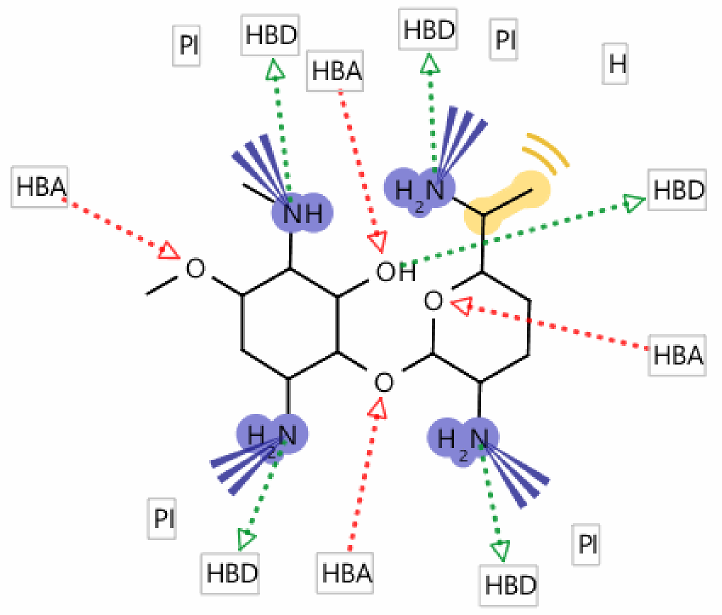
SS 56A	39471-53-9	UMXWTYBXHADELM-QSOWAMBTSA-N	InChI=1S/C12H24N2O8/c13-3-1-4(14)7(17)11(6(3)16)22-12-10(20)9(19)8(18)5(2-15)21-12/h3-12,15-20H,1-2,13-14H2/t3-,4+,5-,6+,7-,8-,9+,10+,11-,12+/m1/s1	N[C@@H]1C[C@@H]([C@H]([C@@]([H])([C@H]1O)O[C@]2([C@H]([C@H]([C@@H]([C@@H](CO)O2)O)O)O)[H])O)N	Small ribosomal subunit inhibitor	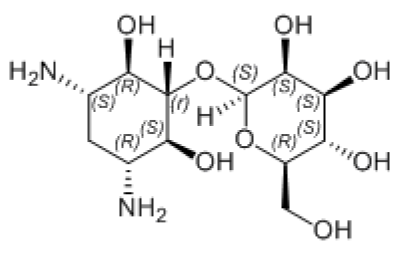	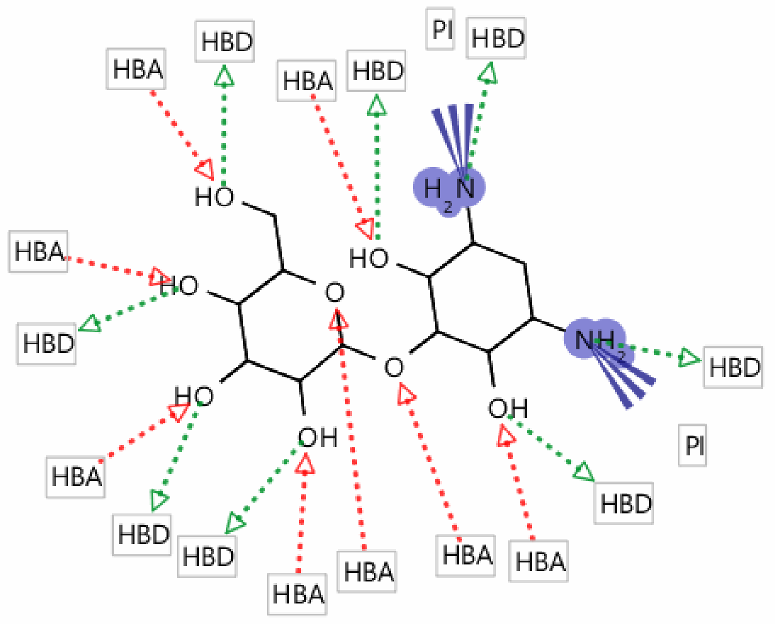

Common name	CAS Reg. No.	InChIKey	InChI	SMILES	Primary target	2D Structure	Pharmacophore[1]
SS 56B	39471-54-0	UMXWTYBXHADELM-BAFSKJBISA-N	InChI=1S/C12H24N2O8/c13-3-1-4(14)7(17)11(6(3)16)22-12-10(20)9(19)8(18)5(2-15)21-12/h3-12,15-20H,1-2,13-14H2/t3-,4+,5-,6+,7-,8+,9+,10+,11-,12+/m1/s1	N[C@@H]1C[C@@H]([C@H]([C@@]([H])([C@H]1O)O[C@]2([C@H]([C@H]([C@H]([C@@H](CO)O2)O)O)O)[H])O)N	Small ribosomal subunit inhibitor	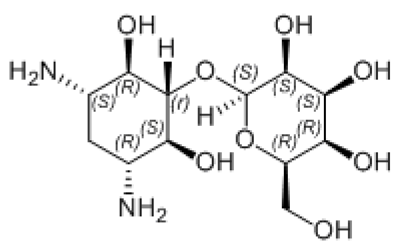	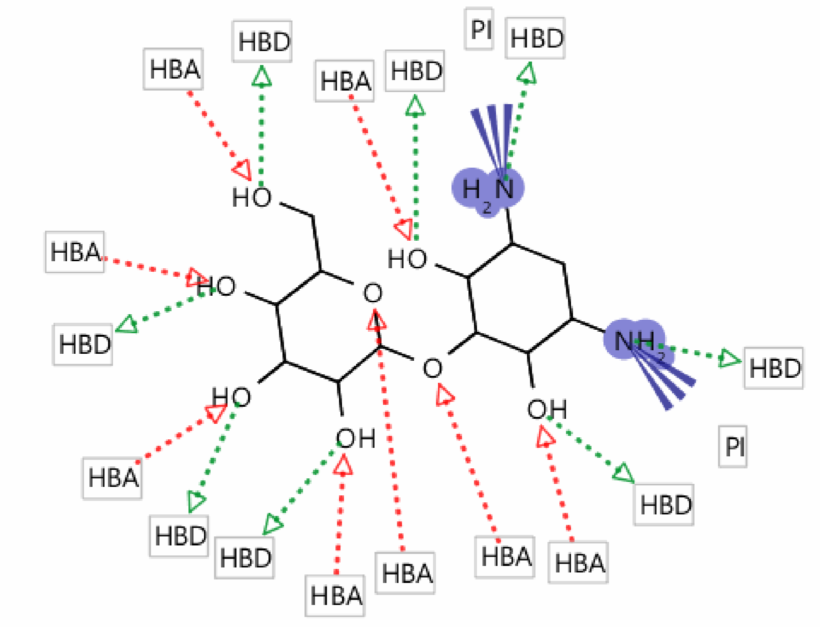
SS 56C	39471-55-1	AQZVLCYCZRQKRV-YFTWSAESSA-N	InChI=1S/2C19H35N3O14/c2*20-3(1-23)13-11(29)12(30)17(31)19(34-13)35-15-7(25)4(2-24)32-18(16(15)36-19)33-14-9(27)5(21)8(26)6(22)10(14)28/h2*3-18,23-31H,1-2,20-22H2/t3-,4+,5-,6+,7-,8+,9+,10-,11+,12-,13+,14-,15-,16-,17+,18-,19+;3-,4+,5-,6+,7-,8+,9+,10-,11+,12-,13+,14-,15-,16-,17+,18-,19-/m00/s1	N[C@@H](CO)[C@@]1([C@@H]([C@@H]([C@H]([C@@]2(O1)O[C@]3([H])[C@@]([H])([C@@]([H])(O[C@H](CO)[C@@H]3O)O[C@]4([C@@H]([C@H]([C@H]([C@H]([C@@H]4O)N)O)N)O)[H])O2)O)O)O)[H].N[C@@H](CO)[C@@]5([C@@H]([C@@H]([C@H]([C@]6(O5)O[C@]7([H])[C@@]([H])([C@@]([H])(O[C@H](CO)[C@@H]7O)O[C@]8([C@@H]([C@H]([C@H]([C@H]([C@@H]8O)N)O)N)O)[H])O6)O)O)O)[H]	Small ribosomal subunit inhibitor	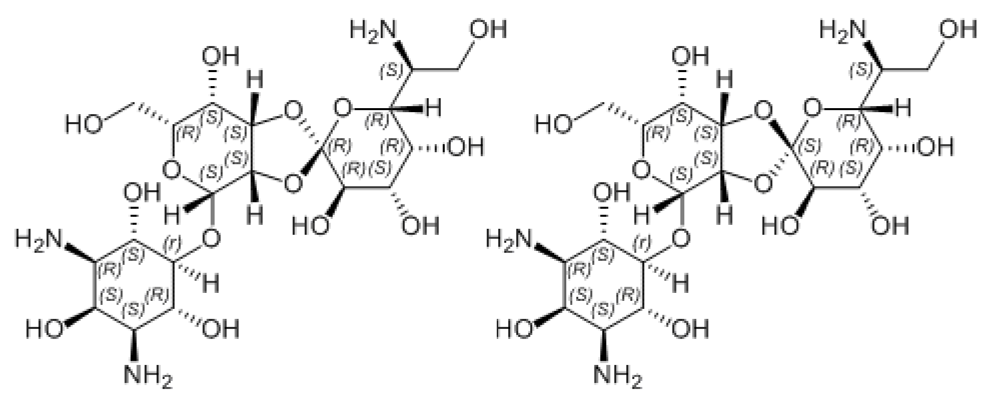	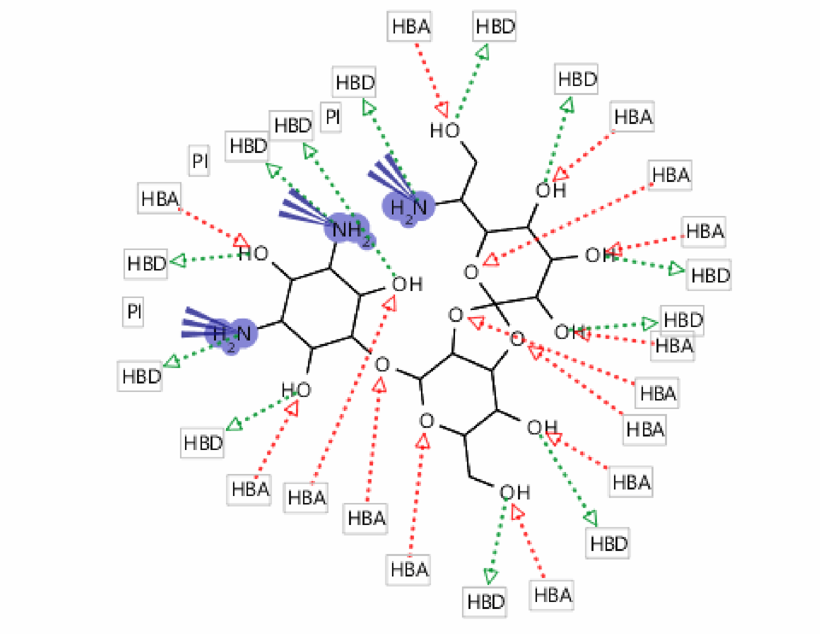
Streptomycin	57-92-1	UCSJYZPVAKXKNQ-HZYVHMACSA-N	InChI=1S/C21H39N7O12/c1-5-21(36,4-30)16(40-17-9(26-2)13(34)10(31)6(3-29)38-17)18(37-5)39-15-8(28-20(24)25)11(32)7(27-19(22)23)12(33)14(15)35/h4-18,26,29,31-36H,3H2,1-2H3,(H4,22,23,27)(H4,24,25,28)/t5-,6-,7+,8-,9-,10-,11+,12-,13-,14+,15+,16-,17-,18-,21+/m0/s1	C[C@H]1[C@](C=O)([C@@H](O[C@@]2([H])[C@@H](NC)[C@H](O)[C@@H](O)[C@@H](O2)CO)[C@H](O[C@]3([H])[C@]([C@H](O)[C@]([C@H](O)[C@H]3O)(NC(N)=N)[H])(NC(N)=N)[H])O1)O	Small ribosomal subunit inhibitor	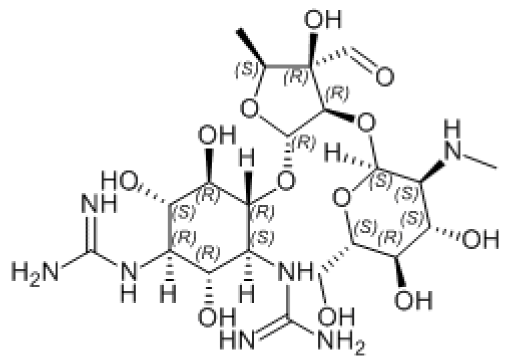	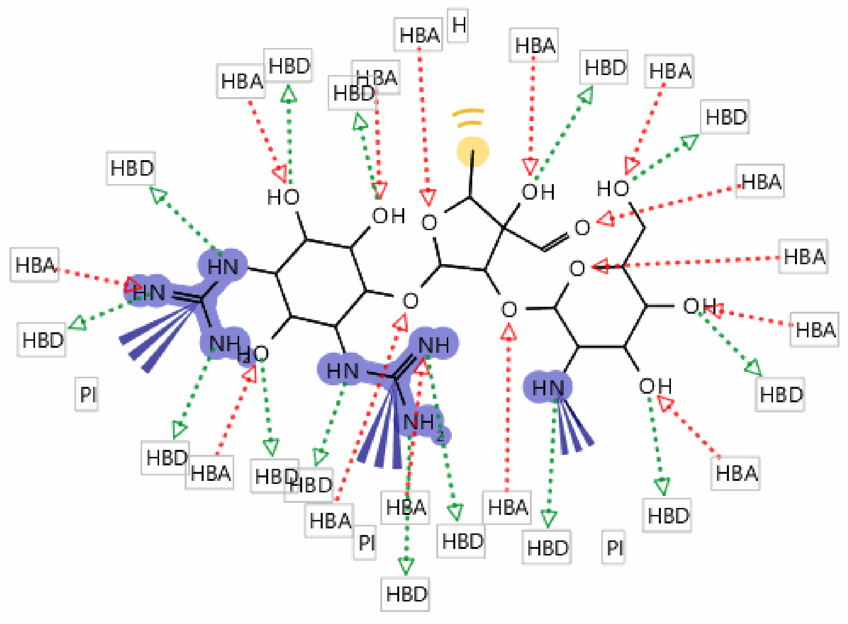
Streptoniazid (streptonicozid)	4480-58-4	JCBKYEIGGFKWPQ-HKWRBIJSSA-N	InChI=1S/C27H44N10O12/c1-9-27(45,8-34-37-22(44)10-3-5-33-6-4-10)21(49-23-14(32-2)18(42)15(39)11(7-38)47-23)24(46-9)48-20-13(36-26(30)31)16(40)12(35-25(28)29)17(41)19(20)43/h3-6,8-9,11-21,23-24,32,38-43,45H,7H2,1-2H3,(H,37,44)(H4,28,29,35)(H4,30,31,36)/b34-8+/t9-,11-,12+,13-,14-,15-,16+,17-,18-,19+,20+,21-,23-,24-,27+/m0/s1	CN[C@H]1[C@@H]([C@H]([C@@H](O[C@]1(O[C@H]2[C@@H](O[C@H]([C@@]2(/C=N/NC(C3=CC=NC=C3)=O)O)C)O[C@]4([C@@H]([C@H]([C@@]([H])([C@H]([C@@]4(NC(N)=N)[H])O)NC(N)=N)O)O)[H])[H])CO)O)O	Small ribosomal subunit inhibitor	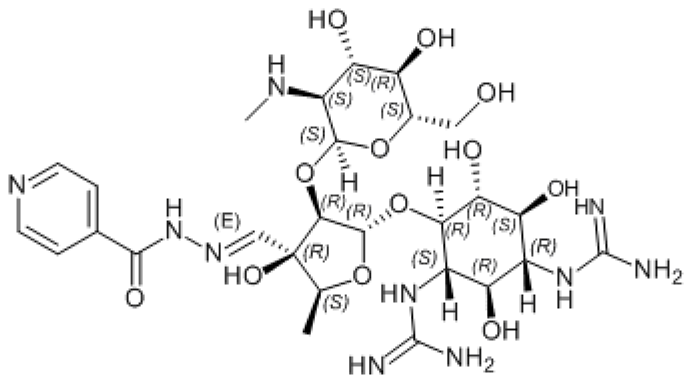	
Tobramycin (nebramycin VI)	32986-56-4	NLVFBUXFDBBNBW-PBSUHMDJSA-N	InChI=1S/C18H37N5O9/c19-3-9-8(25)2-7(22)17(29-9)31-15-5(20)1-6(21)16(14(15)28)32-18-13(27)11(23)12(26)10(4-24)30-18/h5-18,24-28H,1-4,19-23H2/t5-,6+,7+,8-,9+,10+,11-,12+,13+,14-,15+,16-,17+,18+/m0/s1	NC[C@@H]1[C@H](C[C@H]([C@@]([H])(O1)O[C@@]2([C@H](C[C@H]([C@@]([H])([C@H]2O)O[C@@]3([C@@H]([C@H]([C@@H]([C@@H](CO)O3)O)N)O)[H])N)N)[H])N)O	Small ribosomal subunit inhibitor	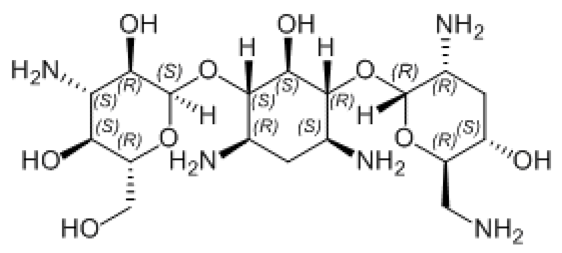	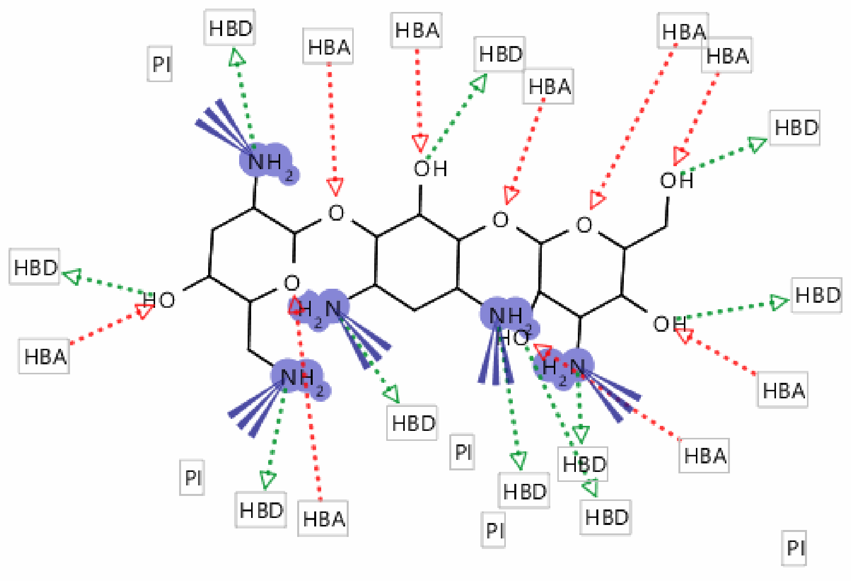
Trospectomycin	88669-04-9	KHAUBYTYGDOYRU-IRXASZMISA-N	InChI=1S/C17H30N2O7/c1-4-5-6-8-7-9(20)17(23)16(24-8)25-15-13(22)10(18-2)12(21)11(19-3)14(15)26-17/h8,10-16,18-19,21-23H,4-7H2,1-3H3/t8-,10-,11+,12+,13+,14-,15-,16+,17+/m1/s1	CCCC[C@@]1(CC([C@]2([C@@]([H])(O1)O[C@@]3([C@H]([C@@H]([C@@H]([C@@H]([C@]3(O2)[H])NC)O)NC)O)[H])O)=O)[H]	Small ribosomal subunit inhibitor	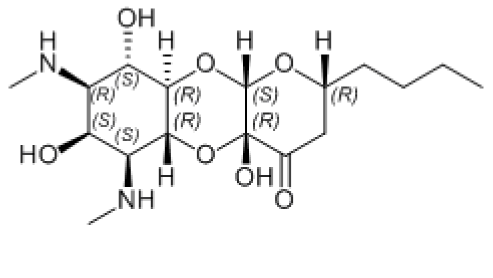	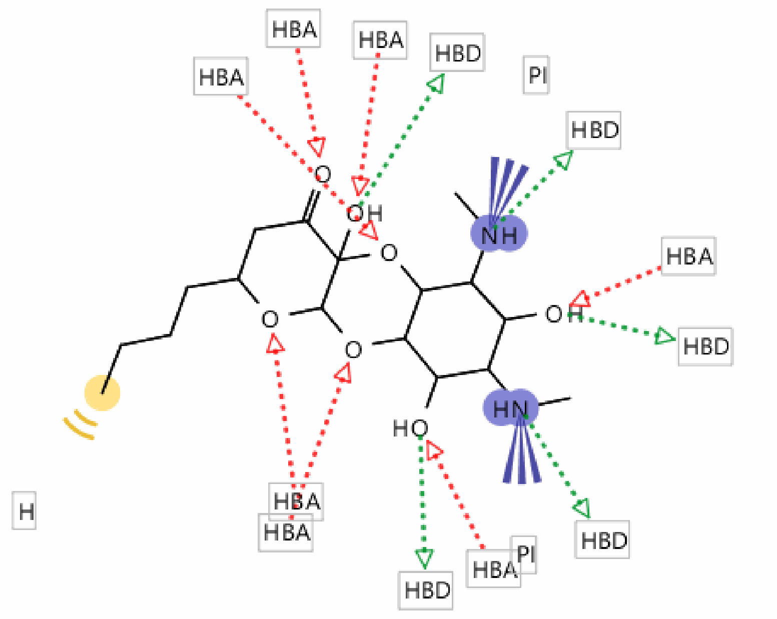

Common name	CAS Reg. No.	InChIKey	InChI	SMILES	Primary target	2D Structure	Pharmacophore[1]
Verdamicin	49863-48-1	XUSXOPRDIDWMFO-CTMSJIKGSA-N	InChI=1S/C20H39N5O7/c1-8(21)12-5-4-9(22)18(30-12)31-15-10(23)6-11(24)16(13(15)26)32-19-14(27)17(25-3)20(2,28)7-29-19/h5,8-11,13-19,25-28H,4,6-7,21-24H2,1-3H3/t8-,9+,10-,11+,13-,14+,15+,16-,17+,18+,19+,20-/m0/s1	C[C@@H](C1=CC[C@H]([C@]([H])(O1)O[C@@]2([C@H](C[C@H]([C@@]([H])([C@H]2O)O[C@@]3([C@@H]([C@H]([C@](O)(C)CO3)NC)O)[H])N)N)[H])N)N	Small ribosomal subunit inhibitor	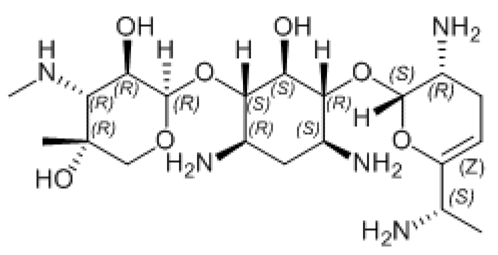	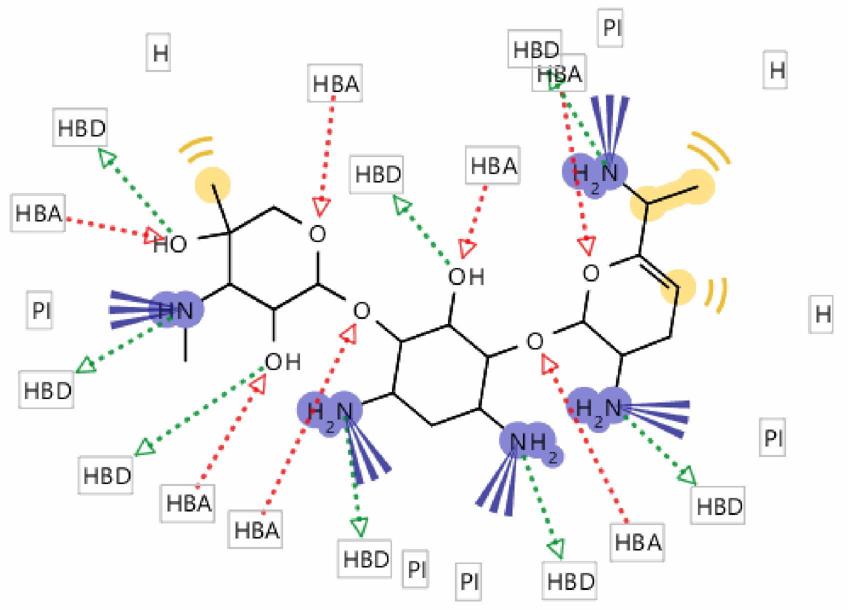
Verdamicin C₂		XUSXOPRDIDWMFO-KJYPRNKJSA-N	InChI=1S/C20H39N5O7/c1-8(21)12-5-4-9(22)18(30-12)31-15-10(23)6-11(24)16(13(15)26)32-19-14(27)17(25-3)20(2,28)7-29-19/h5,8-11,13-19,25-28H,4,6-7,21-24H2,1-3H3/t8-,9-,10+,11-,13+,14-,15-,16+,17-,18-,19-,20+/m1/s1	C[C@H](C1=CC[C@H]([C@]([H])(O1)O[C@@]2([C@H](C[C@H]([C@@]([H])([C@H]2O)O[C@@]3([C@@H]([C@H]([C@](O)(C)CO3)NC)O)[H])N)N)[H])N)N	Small ribosomal subunit inhibitor	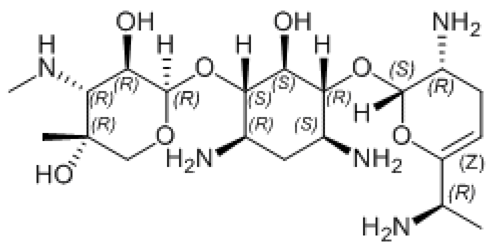	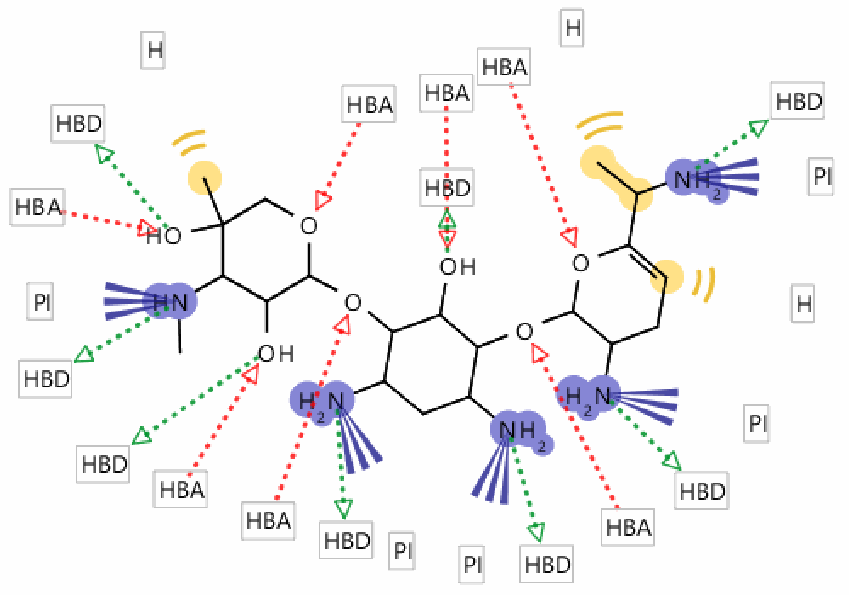
Vertilmicin	59711-97-6	QVRLHIRFLRXFIW-KBXIASADSA-N	InChI=1S/C22H43N5O7/c1-5-27-13-8-12(25)17(33-20-11(24)6-7-14(32-20)10(2)23)15(28)18(13)34-21-16(29)19(26-4)22(3,30)9-31-21/h7,10-13,15-21,26-30H,5-6,8-9,23-25H2,1-4H3/t10-,11+,12-,13+,15-,16+,17+,18-,19+,20+,21+,22-/m0/s1	CCN[C@@]1(C[C@@H]([C@]([H])([C@@H]([C@]1(O[C@@]2([C@@H]([C@H]([C@](O)(C)CO2)NC)O)[H])[H])O)O[C@@]3([C@@H](CC=C([C@H](C)N)O3)N)[H])N)[H]	Small ribosomal subunit inhibitor	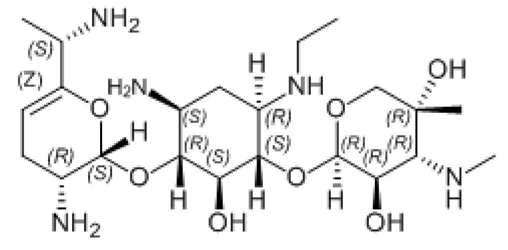	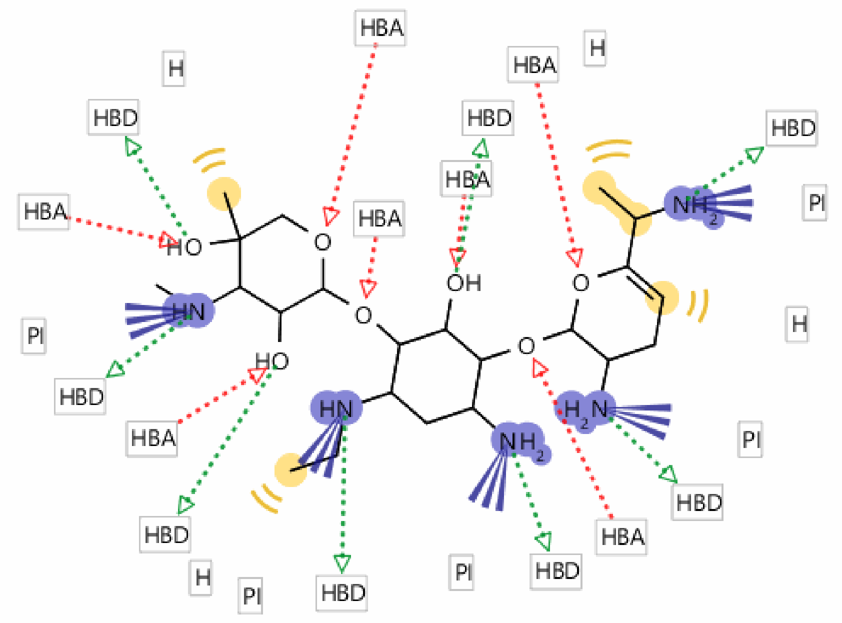
7-Iodosancycline	113164-67-3	HTASTNARVGFMKI-UVPAEMEASA-N	InChI=1S/C21H21IN2O7/c1-24(2)15-9-6-7-5-8-10(22)3-4-11(25)13(8)16(26)12(7)18(28)21(9,31)19(29)14(17(15)27)20(23)30/h3-4,7,9,15,25,27-28,31H,5-6H2,1-2H3,(H2,23,30)/t7-,9-,15-,21-/m0/s1	CN(C)[C@]1([C@@]2(C[C@]([H])(CC3=C(C=CC(O)=C3C4=O)I)C4=C([C@@]2(C(C(C(N)=O)=C1O)=O)O)O)[H])[H]	Small ribosomal subunit inhibitor	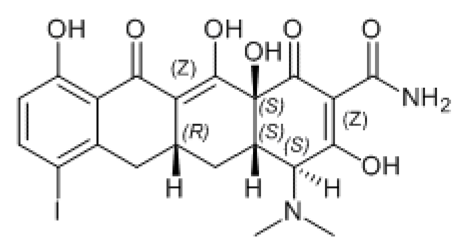	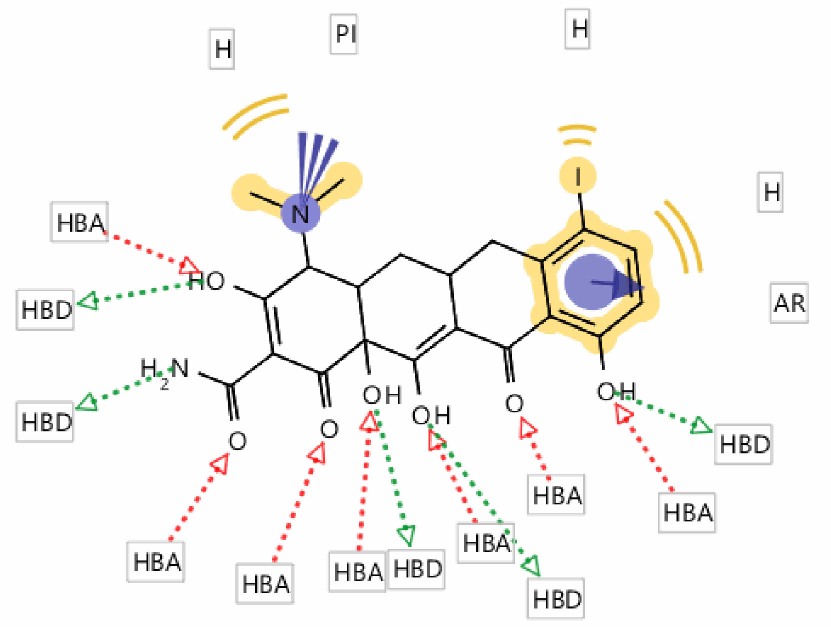
Amicycline	5874-95-3	LRJNBPUJLBQLEI-CROFIWJMSA-N	InChI=1S/C21H23N3O7/c1-24(2)14-9-6-8-5-7-3-4-10(22)15(25)11(7)16(26)12(8)18(28)21(9,31)19(29)13(17(14)27)20(23)30/h3-4,8-9,14,25,27-28,31H,5-6,22H2,1-2H3,(H2,23,30)/t8-,9-,14-,21-/m0/s1	CN(C)[C@]1([C@@]2(C[C@]([H])(CC3=C(C(O)=C(C=C3)N)C4=O)C4=C([C@@]2(C(C(C(N)=O)=C1O)=O)O)O)[H])[H]	Small ribosomal subunit inhibitor	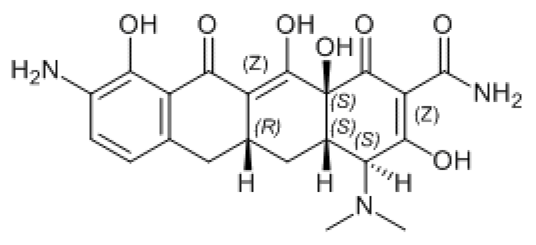	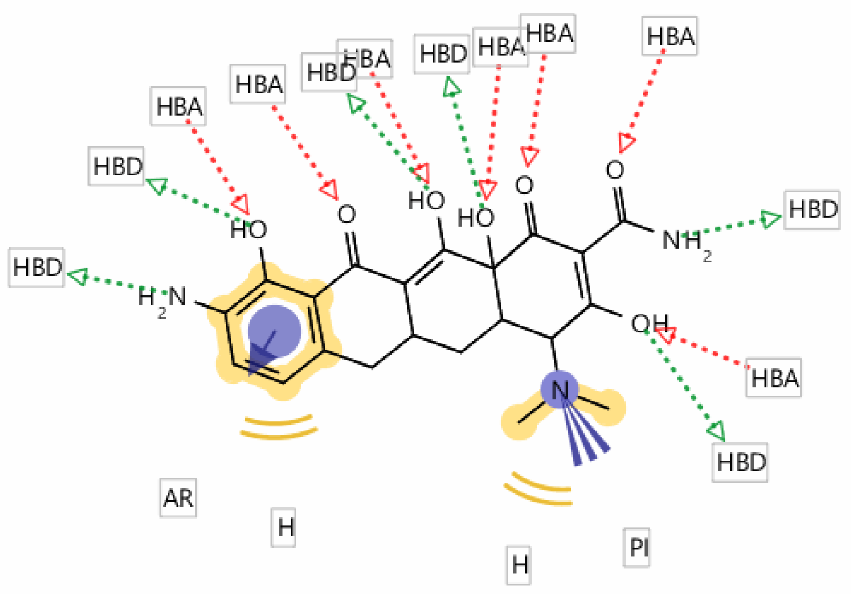
Apicycline	15599-51-6	LBMIIJSEZPRYDF-BZPGYHNTSA-N	InChI=1S/2C30H38N4O11/c2*1-29(44)14-5-4-6-17(36)18(14)22(37)19-15(29)13-16-21(32(2)3)23(38)20(25(40)30(16,45)24(19)39)27(41)31-26(28(42)43)34-9-7-33(8-10-34)11-12-35/h2*4-6,15-16,21,26,35-36,38-39,44-45H,7-13H2,1-3H3,(H,31,41)(H,42,43)/t2*15-,16-,21-,26-,29+,30-/m10/s1	C[C@](O)(C1=C(C(O)=CC=C1)C2=O)[C@]([H])(C[C@@]3([C@]([H])(C(O)=C(C4=O)C(N[C@@]([H])(C(O)=O)N5CCN(CC5)CCO)=O)N(C)C)[H])C2=C([C@]34O)O.C[C@@](O)(C6=C(C(O)=CC=C6)C7=O)[C@@]([H])(C[C@]8([C@@]([H])(C(O)=C(C9=O)C(N[C@]([H])(C(O)=O)N%10CCN(CC%10)CCO)=O)N(C)C)[H])C7=C([C@@]89O)O	Small ribosomal subunit inhibitor	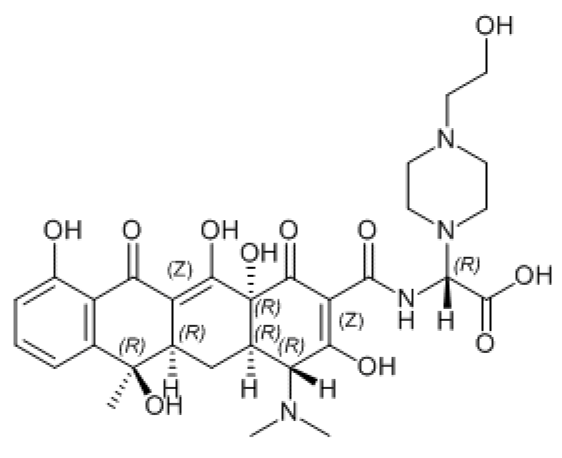 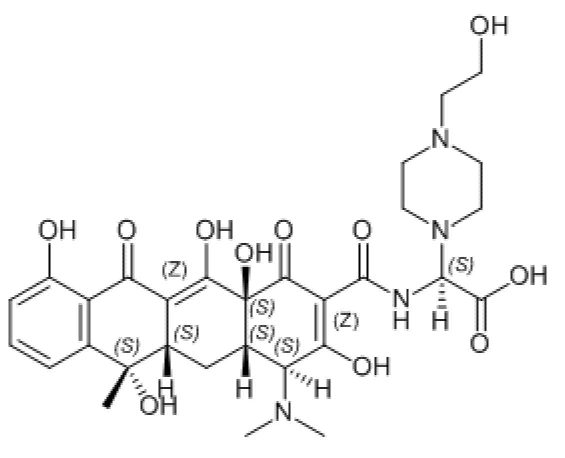	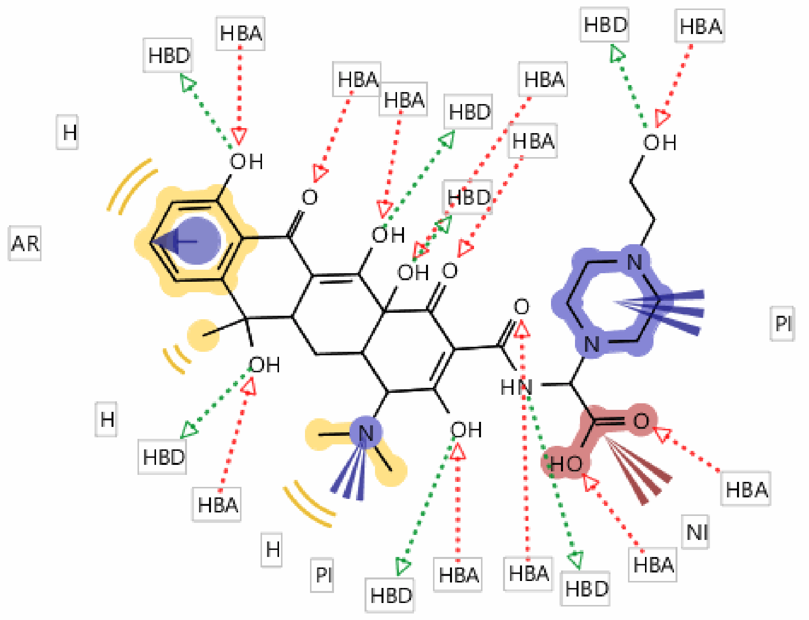

Common name	CAS Reg. No.	InChIKey	InChI	SMILES	Primary target	2D Structure	Pharmacophore[1]
Bromotetracycline (bromtetracycline)	4572-56-9	GYBYOGBOLCMVCQ-XRNKAMNCSA-N	InChI=1S/C22H23BrN2O8/c1-21(32)7-6-8-15(25(2)3)17(28)13(20(24)31)19(30)22(8,33)18(29)11(7)16(27)12-10(26)5-4-9(23)14(12)21/h4-5,7-8,15,26,28-29,32-33H,6H2,1-3H3,(H2,24,31)/t7-,8-,15-,21-,22-/m0/s1	C[C@](O)([C@@]([H])(C[C@]1([C@@]([H])(C(O)=C(C2=O)C(N)=O)N(C)C)[H])C3=C([C@@]12O)O)C4=C(C=CC(O)=C4C3=O)Br	Small ribosomal subunit inhibitor	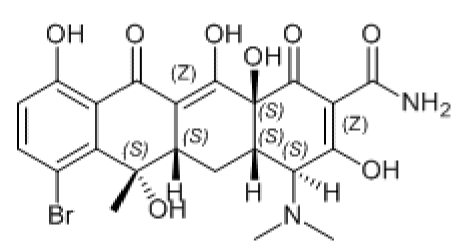	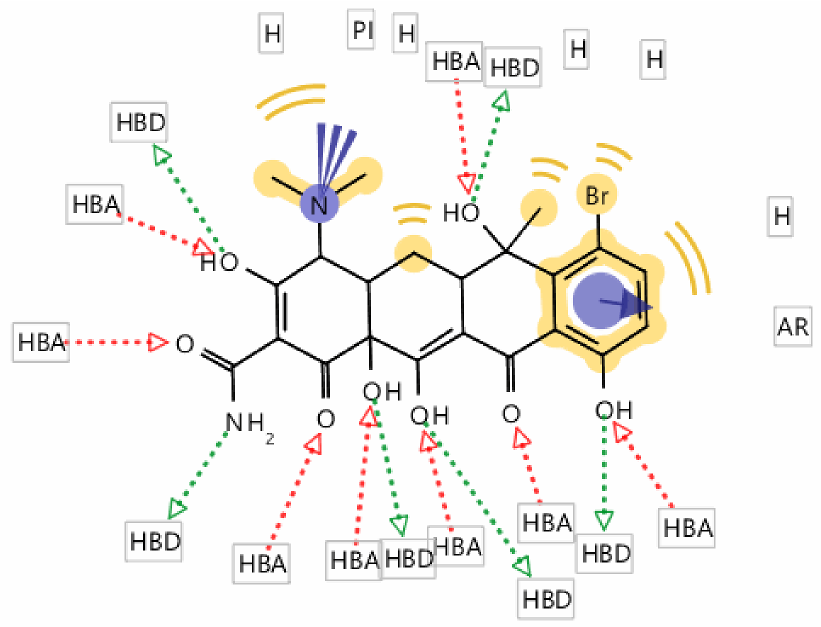
Chlortetracycline (chlorotetracycline)	57-62-5	CYDMQBQPVICBEU-XRNKAMNCSA-N	InChI=1S/C22H23ClN2O8/c1-21(32)7-6-8-15(25(2)3)17(28)13(20(24)31)19(30)22(8,33)18(29)11(7)16(27)12-10(26)5-4-9(23)14(12)21/h4-5,7-8,15,26,28-29,32-33H,6H2,1-3H3,(H2,24,31)/t7-,8-,15-,21-,22-/m0/s1	C[C@](O)([C@@]([H])(C[C@]1([C@@]([H])(C(O)=C(C2=O)C(N)=O)N(C)C)[H])C3=C([C@@]12O)O)C4=C(C=CC(O)=C4C3=O)Cl	Small ribosomal subunit inhibitor	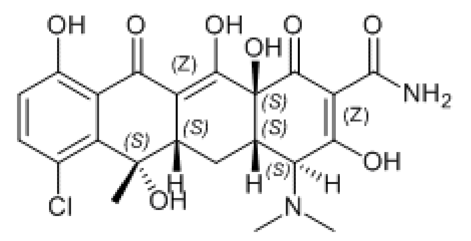	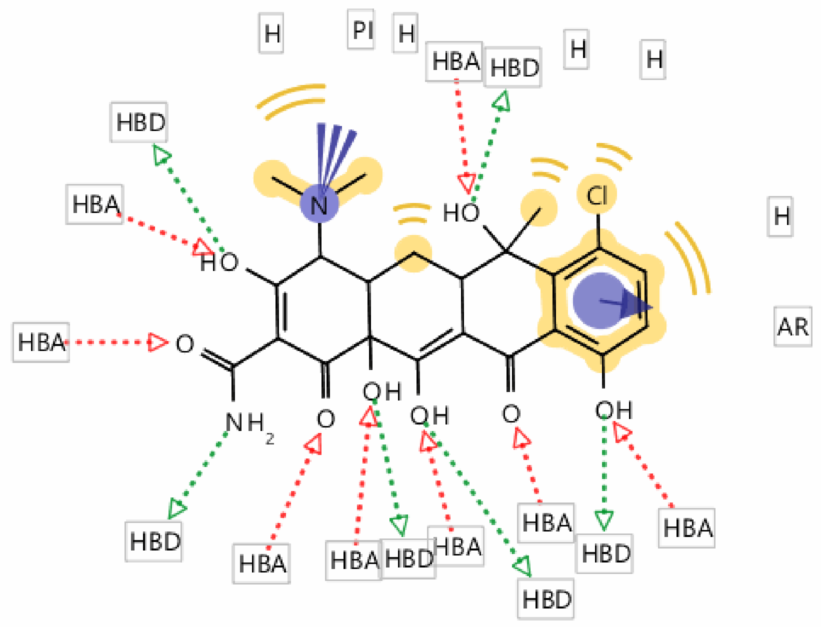
Clomocycline	1181-54-0	GJGDLRSSCNAKGL-KMVLDZISSA-N	InChI=1S/C23H25ClN2O9/c1-22(34)8-6-9-16(26(2)3)18(30)14(21(33)25-7-27)20(32)23(9,35)19(31)12(8)17(29)13-11(28)5-4-10(24)15(13)22/h4-5,8-9,16,27-28,30-31,34-35H,6-7H2,1-3H3,(H,25,33)/t8-,9-,16-,22-,23-/m0/s1	C[C@](O)([C@@]([H])(C[C@]1([C@@]([H])(C(O)=C(C2=O)C(NCO)=O)N(C)C)[H])C3=C([C@@]12O)O)C4=C(C=CC(O)=C4C3=O)Cl	Small ribosomal subunit inhibitor	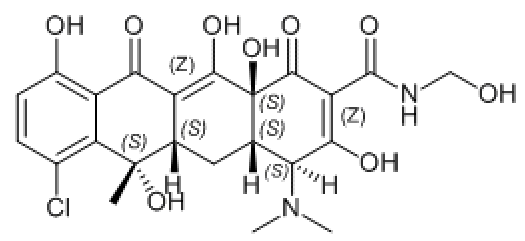	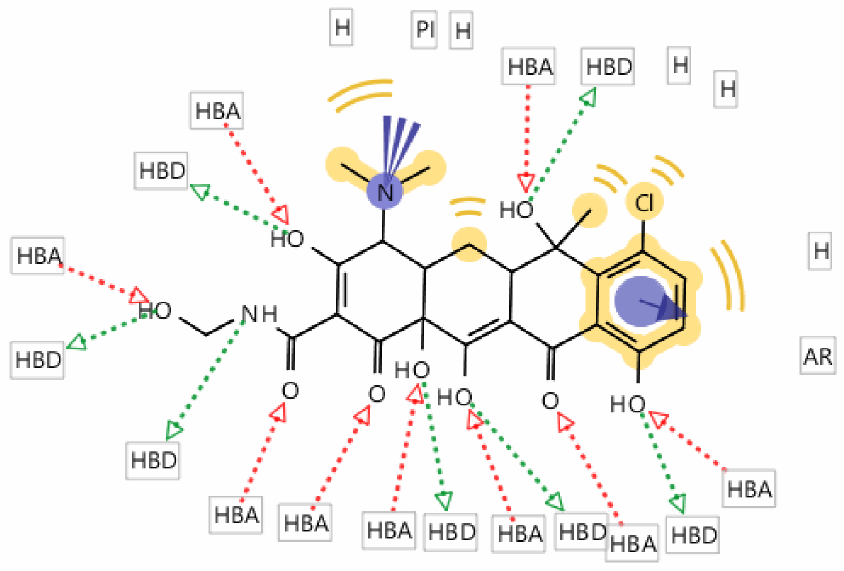
Demeclocycline	127-33-3	FMTDIUIBLCQGJB-SEYHBJAFSA-N	InChI=1S/C21H21ClN2O8/c1-24(2)14-7-5-6-10(16(27)12-9(25)4-3-8(22)11(12)15(6)26)18(29)21(7,32)19(30)13(17(14)28)20(23)31/h3-4,6-7,14-15,25-26,28-29,32H,5H2,1-2H3,(H2,23,31)/t6-,7-,14-,15-,21-/m0/s1	CN(C)[C@]1([C@@]2(C[C@@]([H])(C3=C([C@@]2(C(C(C(N)=O)=C1O)=O)O)O)[C@@H](C4=C(C=CC(O)=C4C3=O)Cl)O)[H])[H]	Small ribosomal subunit inhibitor	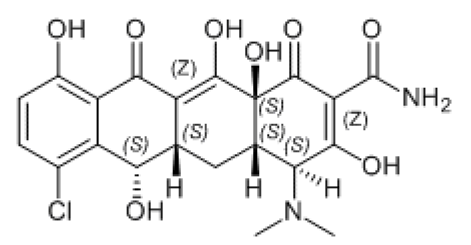	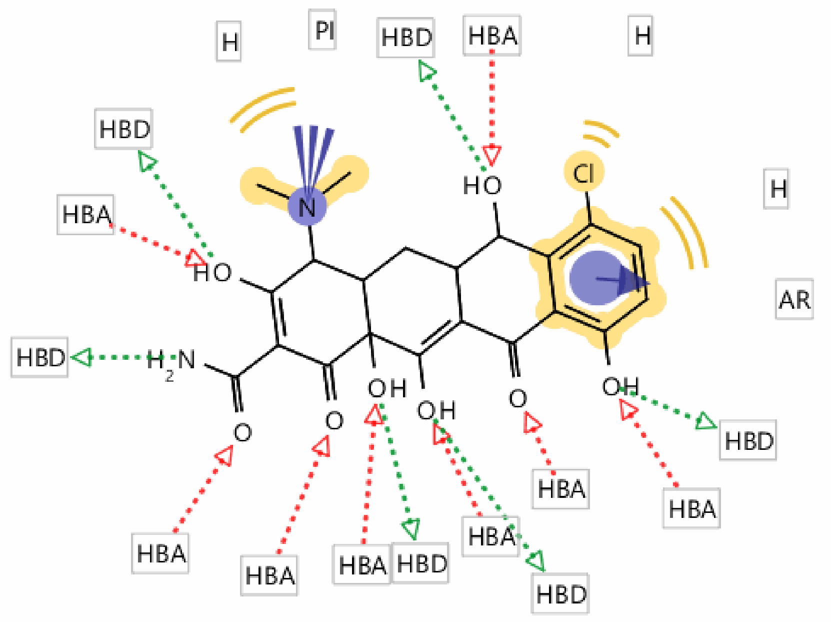
Demecycline	987-02-0	JCSGAUKCDAVARS-SOUFLCLCSA-N	InChI=1S/C21H22N2O8/c1-23(2)14-9-6-8-12(16(26)11-7(15(8)25)4-3-5-10(11)24)18(28)21(9,31)19(29)13(17(14)27)20(22)30/h3-5,8-9,14-15,24-25,27-28,31H,6H2,1-2H3,(H2,22,30)/t8-,9-,14-,15+,21-/m0/s1	CN(C)[C@]1([C@@]2(C[C@@]([H])(C3=C([C@@]2(C(C(C(N)=O)=C1O)=O)O)O)[C@@H](C4=C(C(O)=CC=C4)C3=O)O)[H])[H]	Small ribosomal subunit inhibitor	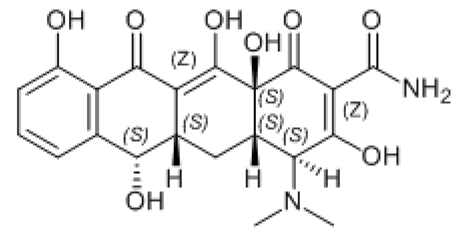	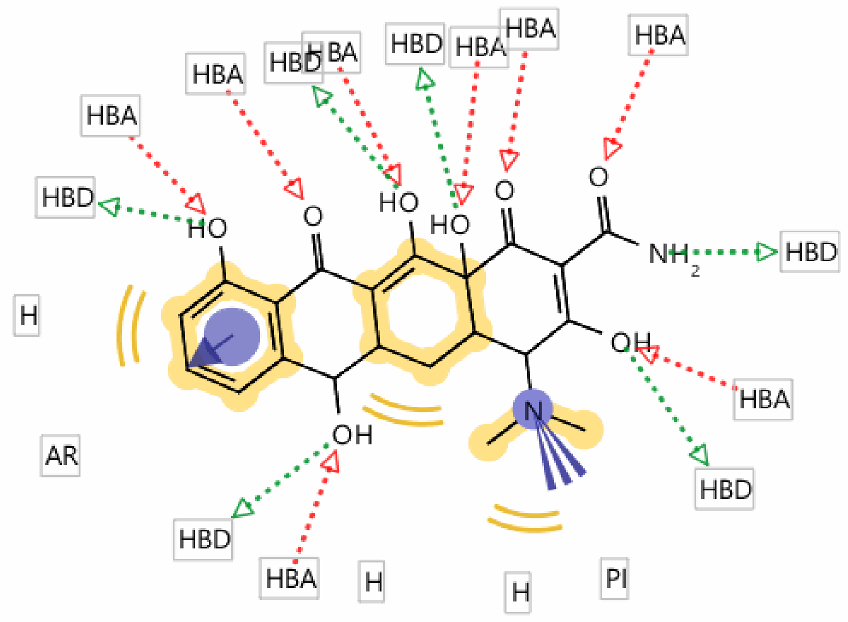
DMG-DMDOT (DMG-DMDOT)	151922-17-7	OAZYAPOSQALFER-CLPWGHEBSA-N	InChI=1S/C25H30N4O8/c1-28(2)9-14(30)27-13-6-5-10-7-11-8-12-18(29(3)4)21(33)17(24(26)36)23(35)25(12,37)22(34)16(11)20(32)15(10)19(13)31/h5-6,11-12,18,31,33-34,37H,7-9H2,1-4H3,(H2,26,36)(H,27,30)/t11-,12-,18-,25-/m0/s1	CN(C)CC(NC1=C(C(C2=O)=C(C=C1)C[C@@]([H])(C[C@]3([C@@]([H])(C(O)=C(C4=O)C(N)=O)N(C)C)[H])C2=C([C@@]34O)O)O)=O	Small ribosomal subunit inhibitor	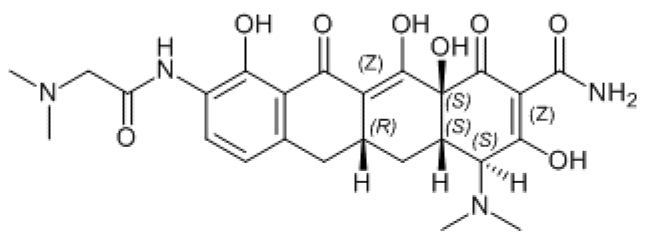	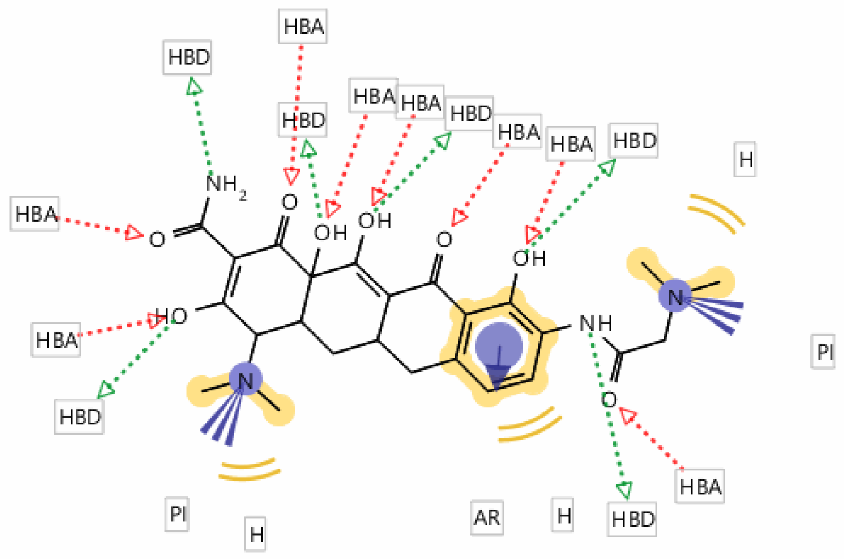

Common name	CAS Reg. No.	InChIKey	InChI	SMILES	Primary target	2D Structure	Pharmacophore[1]
DMG-MINO	151922-16-6	CTFJMTPWQZXWGH-ISIOAQNYSA-N	InChI=1S/C27H35N5O8/c1-30(2)10-16(33)29-14-9-15(31(3)4)12-7-11-8-13-20(32(5)6)23(36)19(26(28)39)25(38)27(13,40)24(37)17(11)22(35)18(12)21(14)34/h9,11,13,20,34,36-37,40H,7-8,10H2,1-6H3,(H2,28,39)(H,29,33)/t11-,13-,20-,27-/m0/s1	CN(CC(NC1=CC(N(C)C)=C(C2=C1O)C[C@@](C[C@@]3([H])[C@@](C(O)=C(C(N)=O)C4=O)(N(C)C)[H])(C(C2=O)=C(O)[C@@]34O)[H])=O)C	Small ribosomal subunit inhibitor	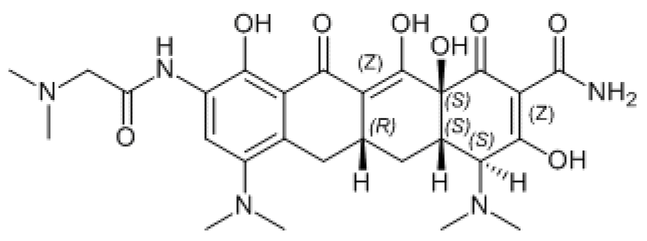	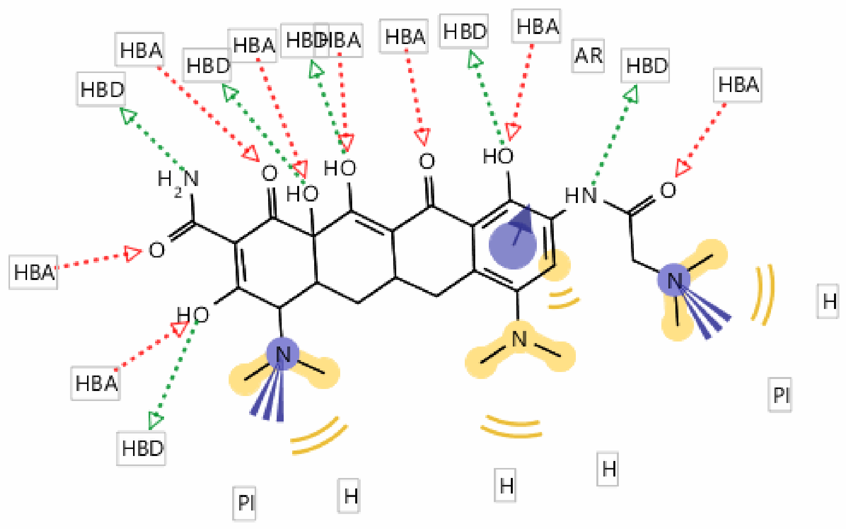
Doxycycline	564-25-0	JBIWCJUYHHGXTC-AKNGSSGZSA-N	InChI=1S/C22H24N2O8/c1-7-8-5-4-6-9(25)11(8)16(26)12-10(7)17(27)14-15(24(2)3)18(28)13(21(23)31)20(30)22(14,32)19(12)29/h4-7,10,14-15,17,25,27-29,32H,1-3H3,(H2,23,31)/t7-,10+,14+,15-,17-,22-/m0/s1	C[C@H]([C@](C1=C(O)[C@@]2(O)[C@]3([H])[C@@](C(O)=C(C(N)=O)C2=O)(N(C)C)[H])([C@@H]3O)[H])C4=C(C1=O)C(O)=CC=C4	Small ribosomal subunit inhibitor	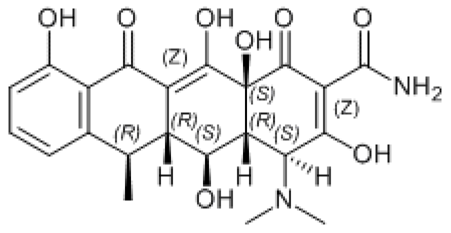	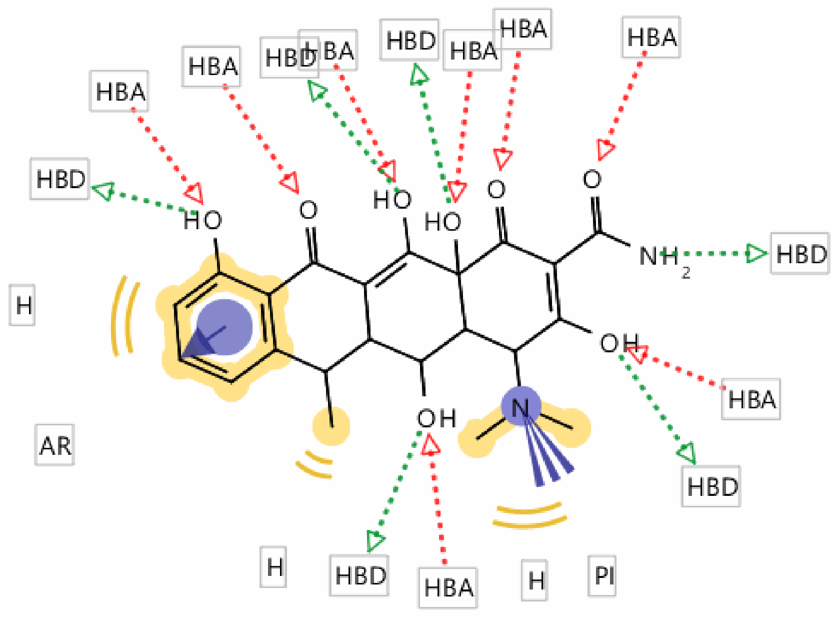
Eravacycline	1207283-85-9	HLFSMUUOKPBTSM-ISIOAQNYSA-N	InChI=1S/C27H31FN4O8/c1-31(2)20-13-8-11-7-12-14(28)9-15(30-16(33)10-32-5-3-4-6-32)21(34)18(12)22(35)17(11)24(37)27(13,40)25(38)19(23(20)36)26(29)39/h9,11,13,20,34,36-37,40H,3-8,10H2,1-2H3,(H2,29,39)(H,30,33)/t11-,13-,20-,27-/m0/s1	CN(C)[C@]1([C@@]2(C[C@]([H])(CC3=C4C(O)=C(C=C3F)NC(CN5CCCC5)=O)C(C4=O)=C([C@@]2(C(C(C(N)=O)=C1O)=O)O)O)[H])[H]	Small ribosomal subunit inhibitor	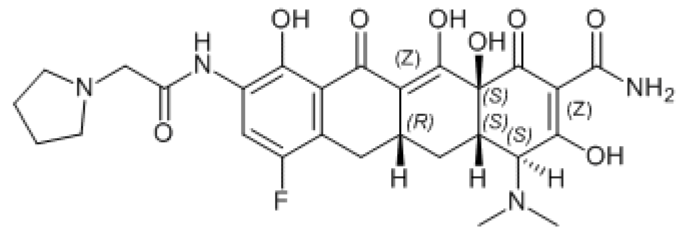	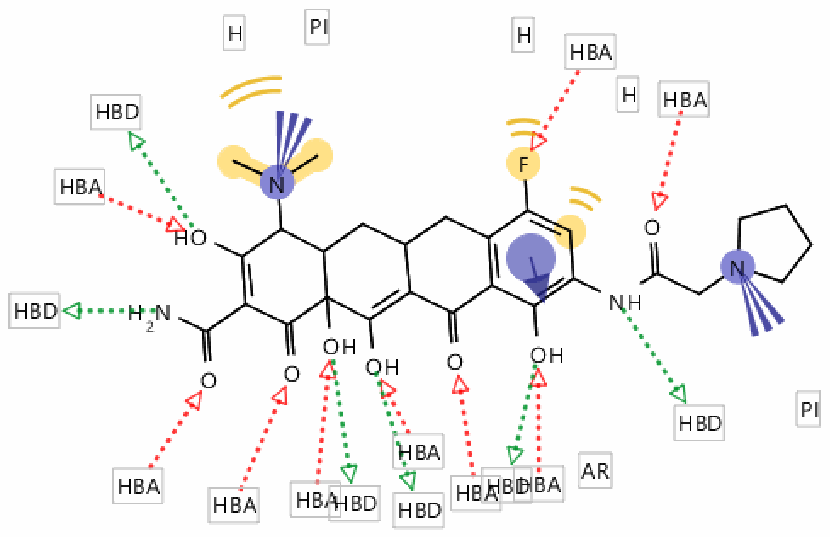
Etamocycline	15590-00-8	BZUKDLNEDCDORL-ULRSWZSCSA-N	InChI=1S/C50H60N6O16/c1-47(69)21-11-9-13-27(57)29(21)37(59)31-23(47)17-25-35(53(3)4)39(61)33(43(65)49(25,71)41(31)63)45(67)51-19-55(7)15-16-56(8)20-52-46(68)34-40(62)36(54(5)6)26-18-24-32(42(64)50(26,72)44(34)66)38(60)30-22(48(24,2)70)12-10-14-28(30)58/h9-14,23-26,35-36,57-58,61-64,69-72H,15-20H2,1-8H3,(H,51,67)(H,52,68)/t23-,24-,25-,26-,35-,36-,47+,48+,49-,50-/m0/s1	C[C@@](O)(C1=C(C(O)=CC=C1)C2=O)[C@@]([H])(C[C@]3([C@@]([H])(C(O)=C(C4=O)C(NCN(C)CCN(C)CNC(C5=C([C@]([H])([C@@]6(C[C@]7(C(C(C8=C(C=CC=C8O)[C@@]7(C)O)=O)=C([C@@]6(C5=O)O)O)[H])[H])N(C)C)O)=O)=O)N(C)C)[H])C2=C([C@@]34O)O	Small ribosomal subunit inhibitor	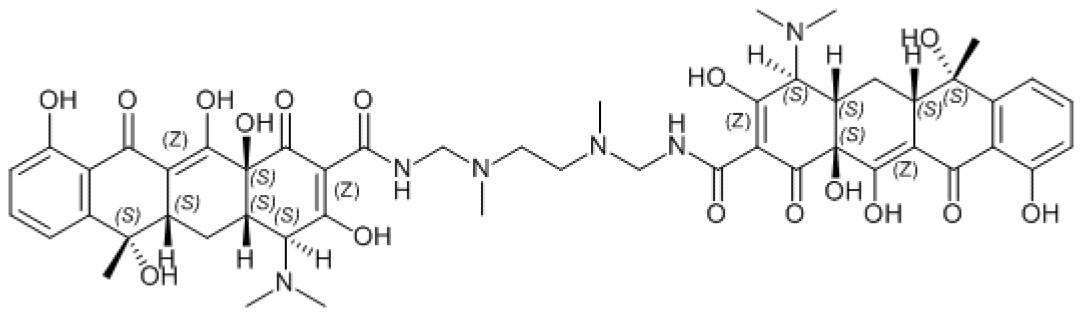	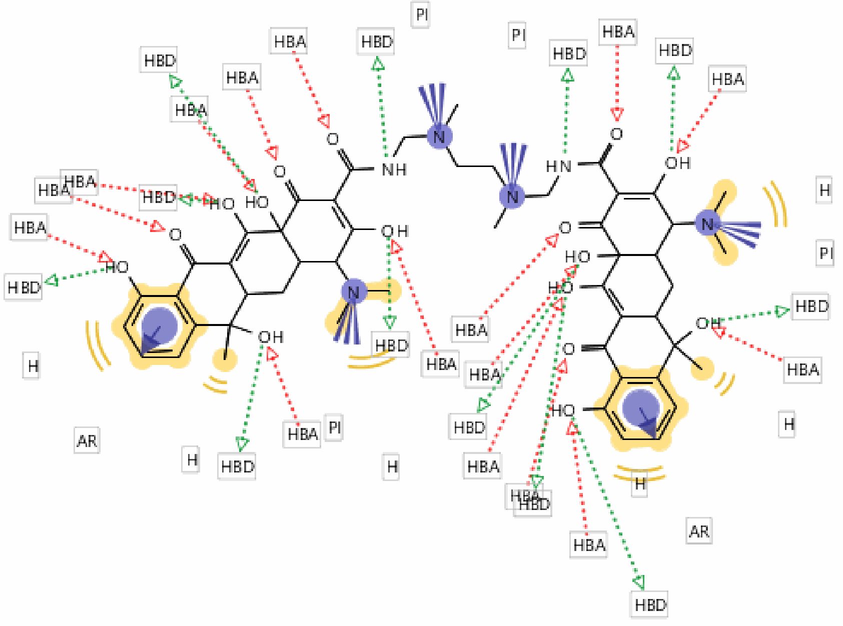
Glycocycline	751-98-4	ASQHOYTXXZGROZ-ZVKUQKJHSA-N	InChI=1S/C25H29N3O10/c1-24(37)10-5-4-6-13(29)15(10)19(32)16-11(24)7-12-18(28(2)3)20(33)17(22(35)25(12,38)21(16)34)23(36)27-9-26-8-14(30)31/h4-6,11-12,18,26,29,33-34,37-38H,7-9H2,1-3H3,(H,27,36)(H,30,31)/t11-,12-,18-,24+,25-/m0/s1	C[C@@](O)(C1=C(C(O)=CC=C1)C2=O)[C@@]([H])(C[C@]3([C@@]([H])(C(O)=C(C4=O)C(NCNCC(O)=O)=O)N(C)C)[H])C2=C([C@@]34O)O	Small ribosomal subunit inhibitor	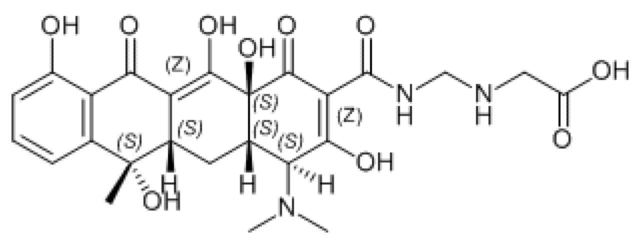	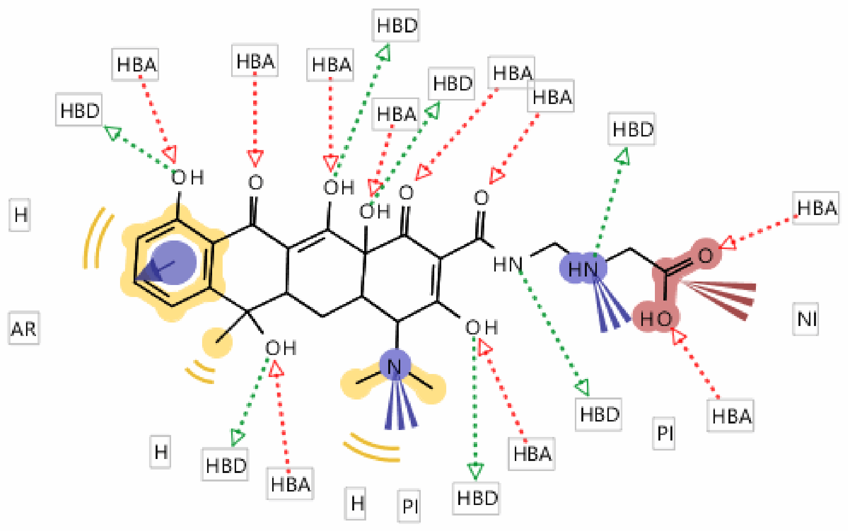
Guamecycline	16545-11-2	DIRJDIBCAHCCFL-AQFAATAFSA-N	InChI=1S/C29H38N8O8/c1-28(44)13-5-4-6-16(38)17(13)21(39)18-14(28)11-15-20(35(2)3)22(40)19(24(42)29(15,45)23(18)41)25(43)33-12-36-7-9-37(10-8-36)27(32)34-26(30)31/h4-6,14-15,20,38,40-41,44-45H,7-12H2,1-3H3,(H,33,43)(H5,30,31,32,34)/t14-,15-,20-,28+,29-/m0/s1	C[C@@]([C@@](C1=C(O)[C@@]23O)([H])C[C@@]2([H])[C@@](N(C)C)([H])C(O)=C(C(NCN4CCN(C(NC(N)=N)=N)CC4)=O)C3=O)(O)C5=C(C1=O)C(O)=CC=C5	Small ribosomal subunit inhibitor	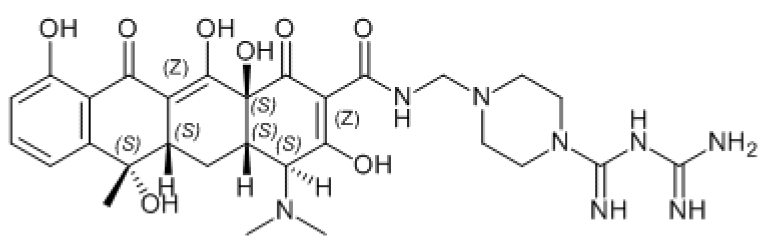	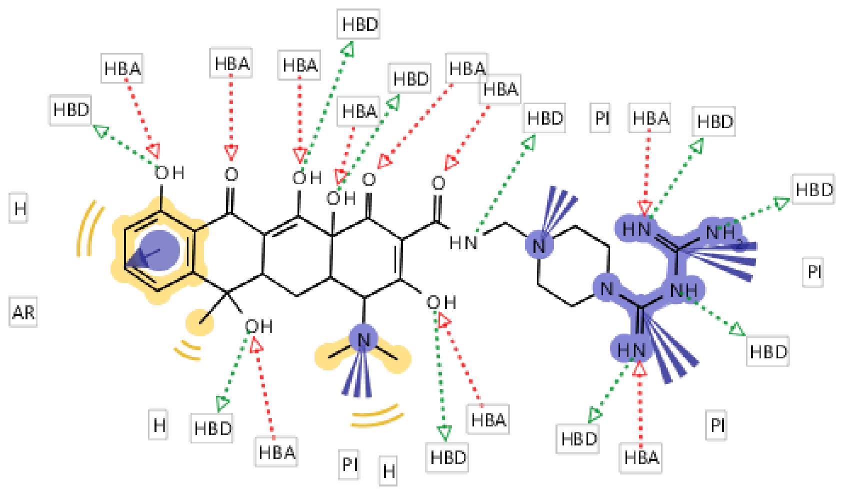

Common name	CAS Reg. No.	InChIKey	InChI	SMILES	Primary target	2D Structure	Pharmacophore[1]
Lymecycline	992-21-2	AHEVKYYGXVEWNO-UEPZRUIBSA-N	InChI=1S/C29H38N4O10/c1-28(42)13-7-6-9-17(34)18(13)22(35)19-14(28)11-15-21(33(2)3)23(36)20(25(38)29(15,43)24(19)37)26(39)32-12-31-10-5-4-8-16(30)27(40)41/h6-7,9,14-16,21,31,34,36-37,42-43H,4-5,8,10-12,30H2,1-3H3,(H,32,39)(H,40,41)/t14-,15-,16-,21-,28+,29-/m0/s1	C[C@@](O)(C1=C(C(O)=CC=C1)C2=O)[C@@]([H])(C[C@]3([C@@]([H])(C(O)=C(C4=O)C(NCNCCCC[C@@H](C(O)=O)N)=O)N(C)C)[H])C2=C([C@@]34O)O	Small ribosomal subunit inhibitor	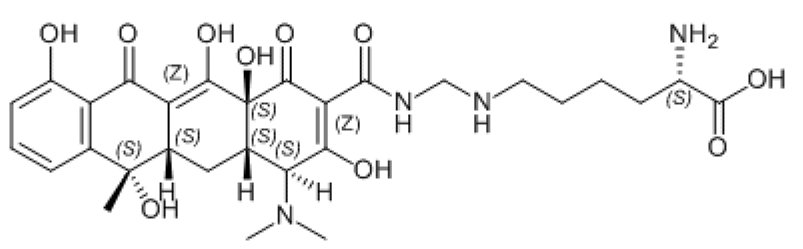	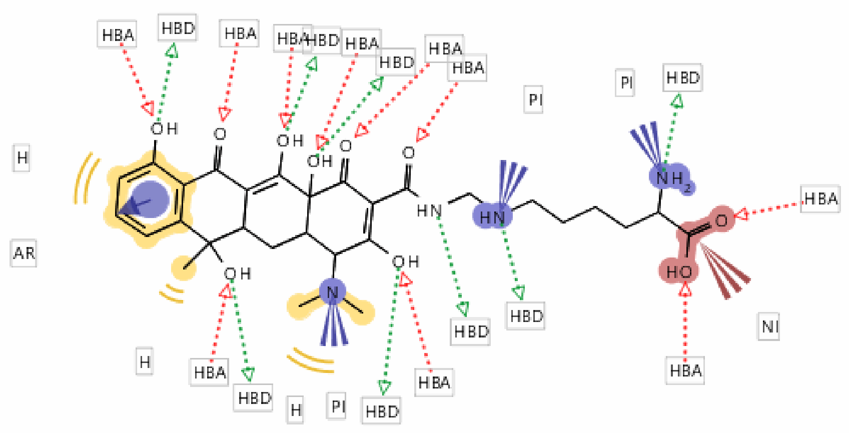
Meclocycline	2013-58-3	GGQJXCQBBONZFX-IWVLMIASSA-N	InChI=1S/C22H21ClN2O8/c1-6-9-7(23)4-5-8(26)11(9)16(27)12-10(6)17(28)14-15(25(2)3)18(29)13(21(24)32)20(31)22(14,33)19(12)30/h4-5,10,14-15,17,26,28-30,33H,1H2,2-3H3,(H2,24,32)/t10-,14-,15+,17+,22+/m1/s1	C=C(C1=C(C=CC(O)=C1C2=O)Cl)[C@]([H])(C2=C([C@]3([C@@]4([C@@]([H])(C(O)=C(C3=O)C(N)=O)N(C)C)[H])O)O)[C@@H]4O	Small ribosomal subunit inhibitor	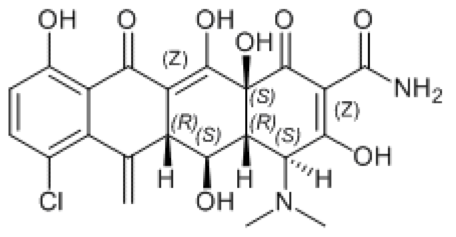	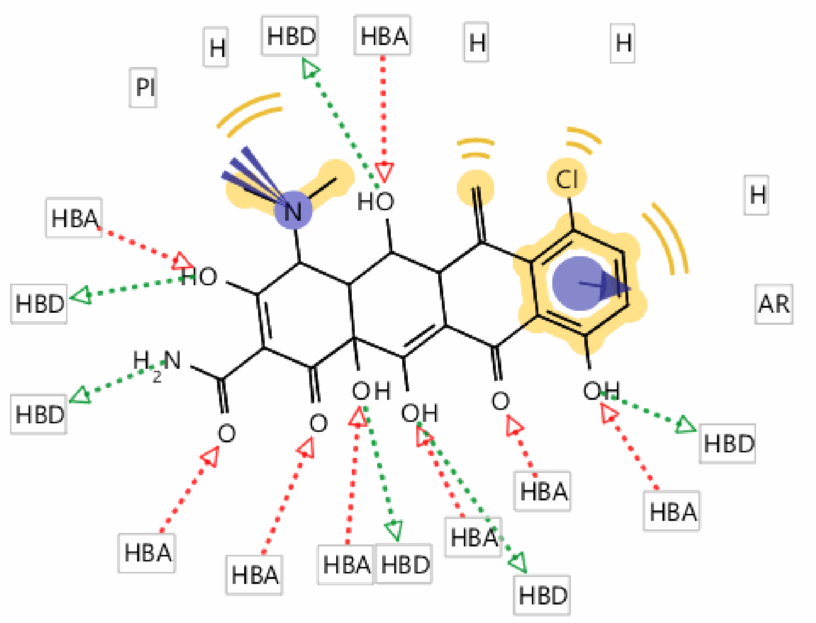
Meglucycline	31770-79-3	QQFZVDWDMZYSQX-FUUYDGDCSA-N	InChI=1S/C29H37N3O13/c1-28(43)10-5-4-6-13(34)15(10)21(36)16-11(28)7-12-19(32(2)3)22(37)17(25(40)29(12,44)24(16)39)26(41)31-9-30-18-23(38)20(35)14(8-33)45-27(18)42/h4-6,11-12,14,18-20,23,27,30,33-35,37-39,42-44H,7-9H2,1-3H3,(H,31,41)/t11-,12-,14+,18+,19-,20+,23+,27+,28+,29-/m0/s1	C[C@@](O)(C1=C(C(O)=CC=C1)C2=O)[C@@]([H])(C[C@]3([C@@]([H])(C(O)=C(C4=O)C(NCN[C@]([H])([C@H]([C@@H]([C@@H](CO)O5)O)O)[C@@H]5O)=O)N(C)C)[H])C2=C([C@@]34O)O	Small ribosomal subunit inhibitor	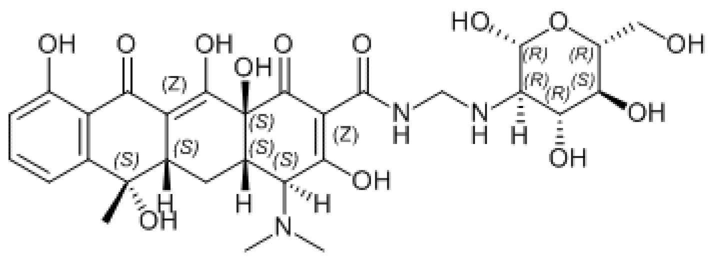	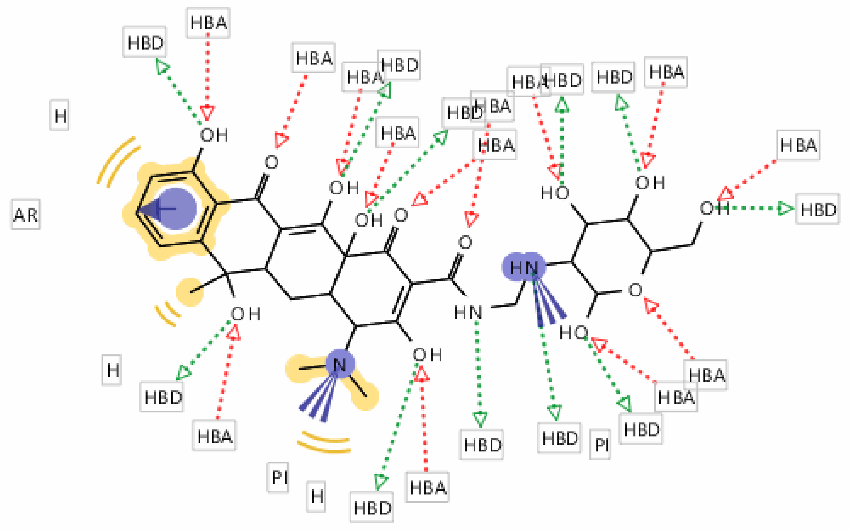
Metacycline (methacycline)	914-00-1	MHIGBKBJSQVXNH-IWVLMIASSA-N	InChI=1S/C22H22N2O8/c1-7-8-5-4-6-9(25)11(8)16(26)12-10(7)17(27)14-15(24(2)3)18(28)13(21(23)31)20(30)22(14,32)19(12)29/h4-6,10,14-15,17,25,27-29,32H,1H2,2-3H3,(H2,23,31)/t10-,14-,15+,17+,22+/m1/s1	C=C(C1=C(C(O)=CC=C1)C2=O)[C@]([H])(C2=C([C@]3([C@@]4([C@@]([H])(C(O)=C(C3=O)C(N)=O)N(C)C)[H])O)O)[C@@H]4O	Small ribosomal subunit inhibitor	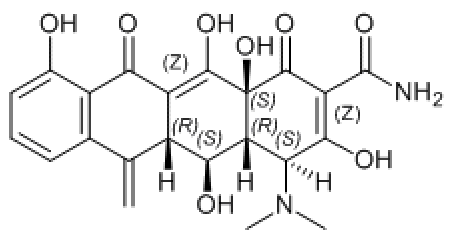	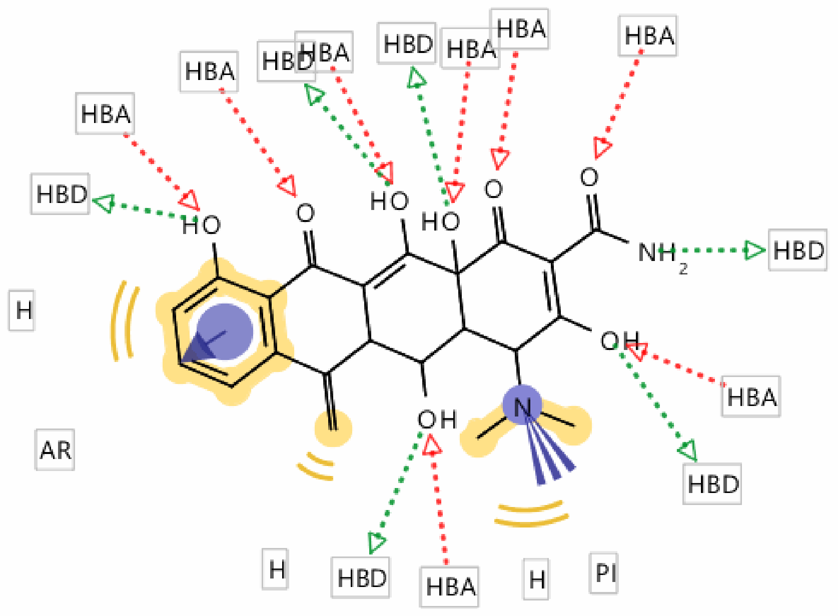
Minocycline	10118-90-8	DYKFCLLONBREIL-KVUCHLLUSA-N	InChI=1S/C23H27N3O7/c1-25(2)12-5-6-13(27)15-10(12)7-9-8-11-17(26(3)4)19(29)16(22(24)32)21(31)23(11,33)20(30)14(9)18(15)28/h5-6,9,11,17,27,29-30,33H,7-8H2,1-4H3,(H2,24,32)/t9-,11-,17-,23-/m0/s1	CN(C)C1=C(C[C@@]([H])(C[C@]2([C@@]([H])(C(O)=C(C3=O)C(N)=O)N(C)C)[H])C4=C([C@@]23O)O)C(C4=O)=C(O)C=C1	Small ribosomal subunit inhibitor	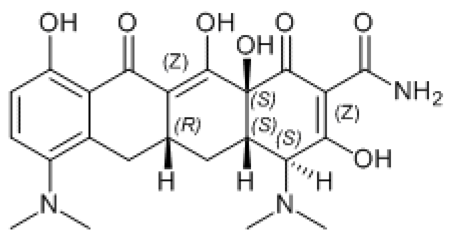	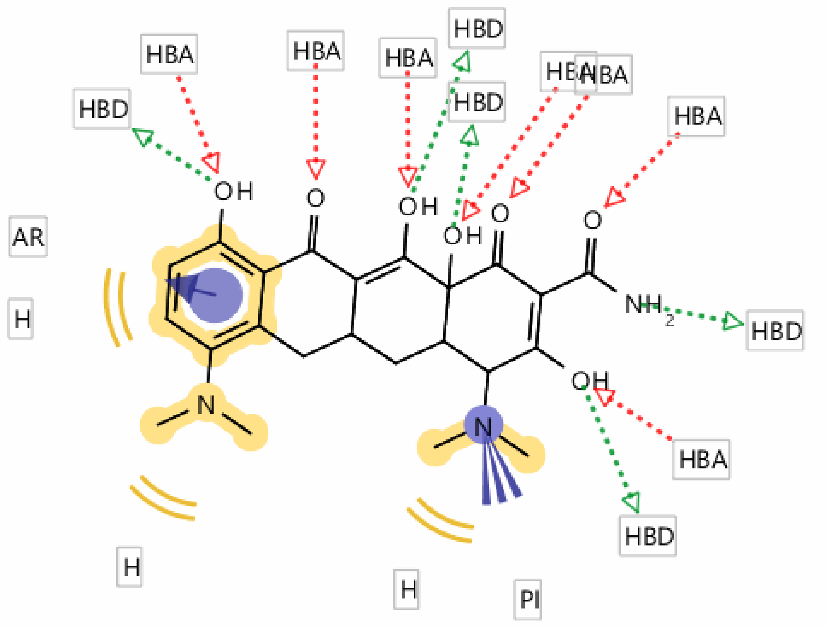
Morphocycline	3098-60-0	BXWBMZALVNZQBE-IAHYZSEUSA-N	InChI=1S/C27H33N3O9/c1-26(37)13-5-4-6-16(31)17(13)21(32)18-14(26)11-15-20(29(2)3)22(33)19(24(35)27(15,38)23(18)34)25(36)28-12-30-7-9-39-10-8-30/h4-6,14-15,20,31,33-34,37-38H,7-12H2,1-3H3,(H,28,36)/t14-,15-,20-,26+,27-/m0/s1	C[C@@](O)(C1=C(C(O)=CC=C1)C2=O)[C@@]([H])(C[C@]3([C@@]([H])(C(O)=C(C4=O)C(NCN5CCOCC5)=O)N(C)C)[H])C2=C([C@@]34O)O	Small ribosomal subunit inhibitor	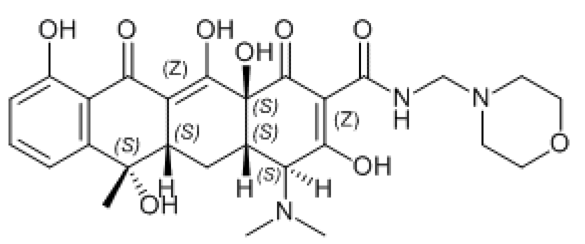	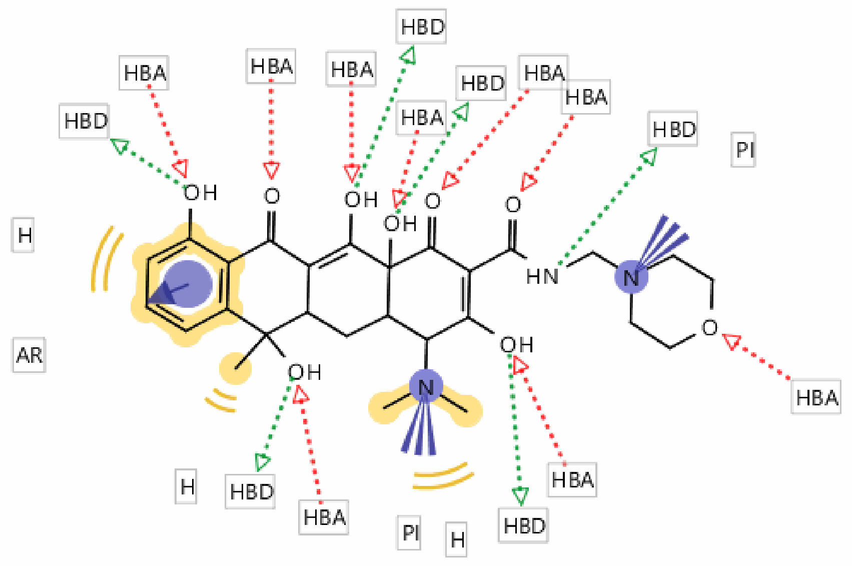

Common name	CAS Reg. No.	InChIKey	InChI	SMILES	Primary target	2D Structure	Pharmacophore[1]
Nitrocycline	5585-59-1	PHMPSHIUYYLXKR-UVPAEMEASA-N	InChI=1S/C21H21N3O9/c1-23(2)15-9-6-7-5-8-10(24(32)33)3-4-11(25)13(8)16(26)12(7)18(28)21(9,31)19(29)14(17(15)27)20(22)30/h3-4,7,9,15,25,27-28,31H,5-6H2,1-2H3,(H2,22,30)/t7-,9-,15-,21-/m0/s1	CN(C)[C@]1([C@@]2(C[C@]([H])(CC3=C(C=CC(O)=C3C4=O)[N+]([O-])=O)C4=C([C@@]2(C(C(C(N)=O)=C1O)=O)O)O)[H])[H]	Small ribosomal subunit inhibitor	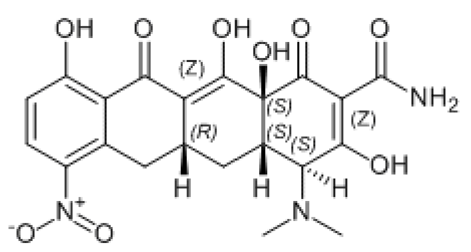	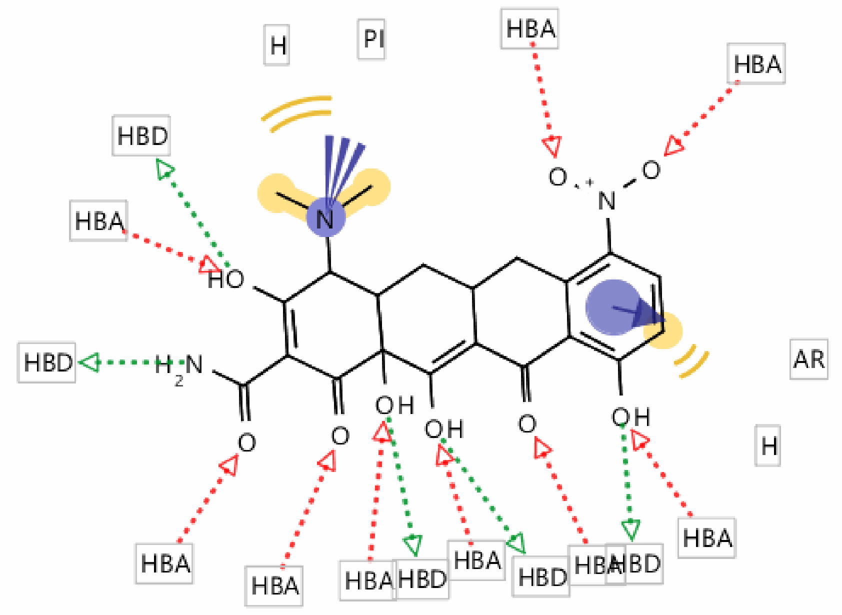
Omadacycline	389139-89-3	JEECQCWWSTZDCK-IQZGDKDPSA-N	InChI=1S/C29H40N4O7/c1-28(2,3)12-31-11-14-10-17(32(4)5)15-8-13-9-16-21(33(6)7)24(36)20(27(30)39)26(38)29(16,40)25(37)18(13)23(35)19(15)22(14)34/h10,13,16,21,31,34,36-37,40H,8-9,11-12H2,1-7H3,(H2,30,39)/t13-,16-,21-,29-/m0/s1	CC(CNCC1=CC(N(C)C)=C(C[C@@]([H])(C[C@]2([C@@]([H])(C(O)=C(C3=O)C(N)=O)N(C)C)[H])C(C4=O)=C([C@@]23O)O)C4=C1O)(C)C	Small ribosomal subunit inhibitor	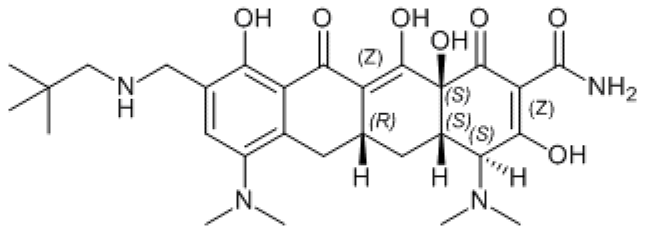	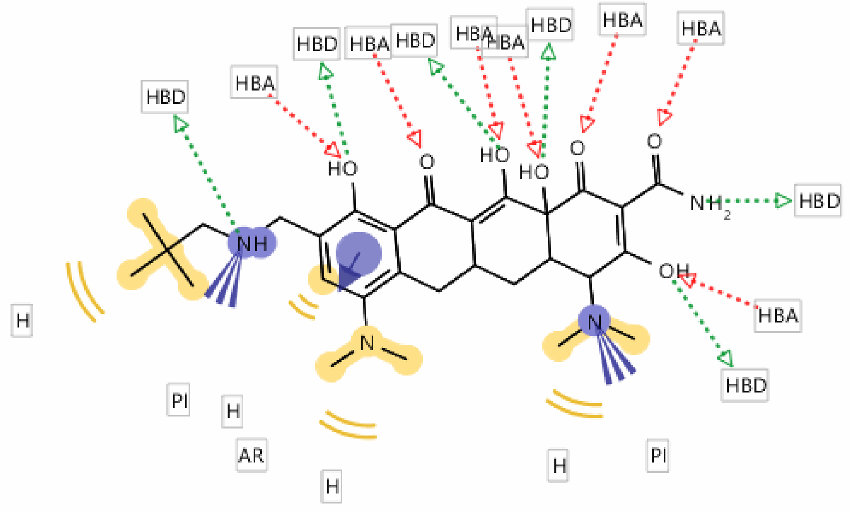
Oxytetracycline	79-57-2	IWVCMVBTMGNXQD-PXOLEDIWSA-N	InChI=1S/C22H24N2O9/c1-21(32)7-5-4-6-8(25)9(7)15(26)10-12(21)17(28)13-14(24(2)3)16(27)11(20(23)31)19(30)22(13,33)18(10)29/h4-6,12-14,17,25,27-29,32-33H,1-3H3,(H2,23,31)/t12-,13-,14+,17+,21-,22+/m1/s1	C[C@@](O)(C1=C(C(O)=CC=C1)C2=O)[C@]([H])(C2=C([C@]3([C@@]4([C@@]([H])(C(O)=C(C3=O)C(N)=O)N(C)C)[H])O)O)[C@@H]4O	Small ribosomal subunit inhibitor	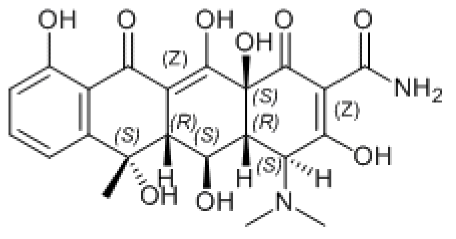	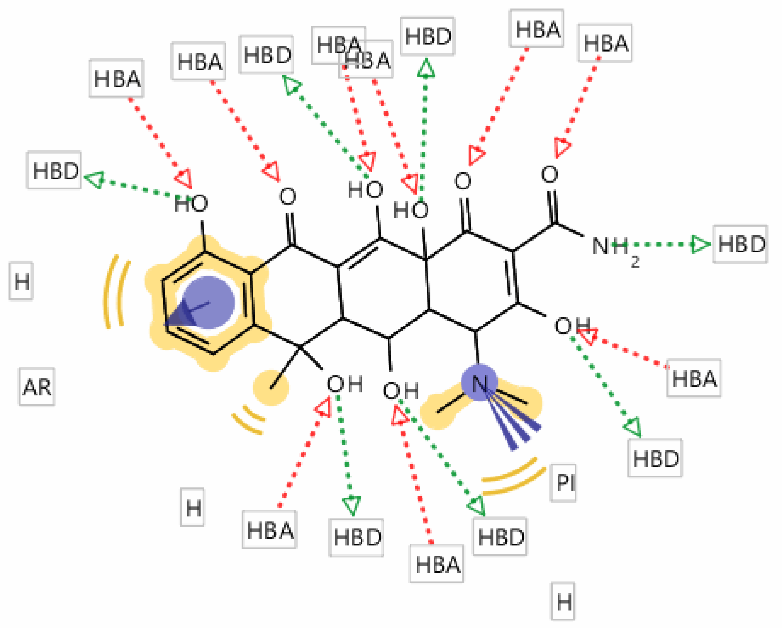
Pecocycline	15301-82-3	YSPSTEBORJKXSI-LQRCXKKPSA-N	InChI=1S/2C29H35N3O10/c2*1-28(41)14-7-4-8-17(33)18(14)22(34)19-15(28)10-16-21(31(2)3)23(35)20(25(37)29(16,42)24(19)36)26(38)30-12-32-9-5-6-13(11-32)27(39)40/h2*4,7-8,13,15-16,21,33,35-36,41-42H,5-6,9-12H2,1-3H3,(H,30,38)(H,39,40)/t13-,15+,16+,21+,28-,29+;13-,15-,16-,21-,28+,29-/m10/s1	C[C@@](O)(C1=C(C(O)=CC=C1)C2=O)[C@@]([H])(C[C@]3([C@@]([H])(C(O)=C(C4=O)C(NCN5CCC[C@]([H])(C5)C(O)=O)=O)N(C)C)[H])C2=C([C@@]34O)O.C[C@@](O)(C6=C(C(O)=CC=C6)C7=O)[C@@]([H])(C[C@]8([C@@]([H])(C(O)=C(C9=O)C(NCN%10CCC[C@@]([H])(C%10)C(O)=O)=O)N(C)C)[H])C7=C([C@@]89O)O	Small ribosomal subunit inhibitor	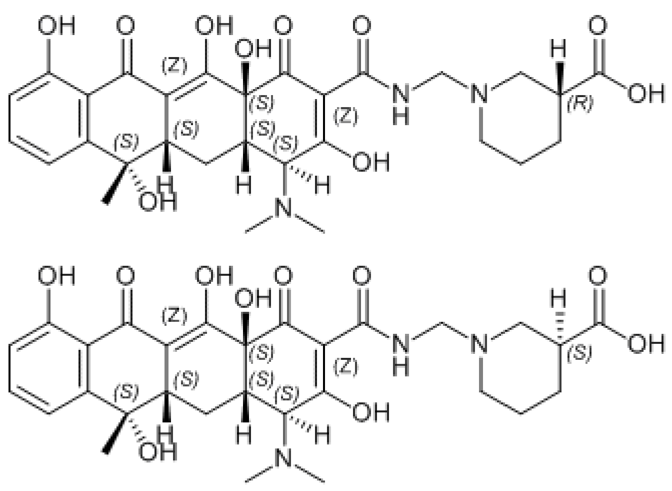	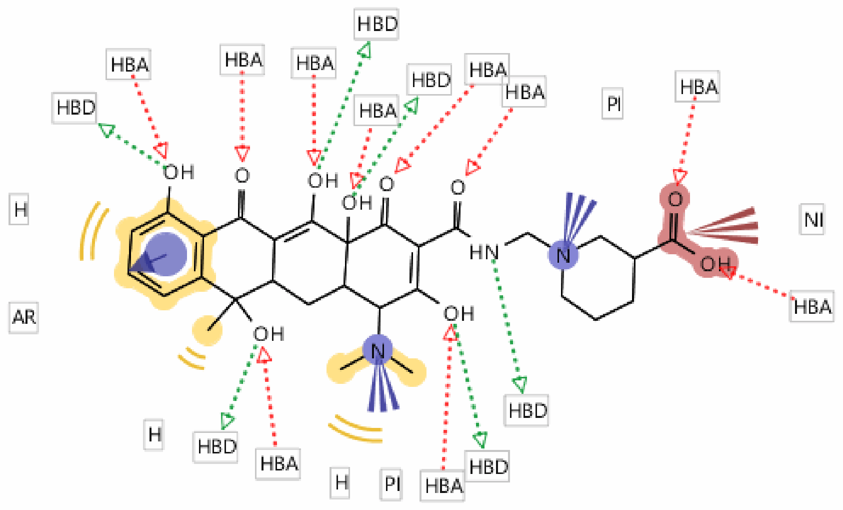
Penimepicycline	4599-60-4	MEGKRPMNPGTIIG-VNYBMUHKSA-N	InChI=1S/C29H38N4O9.C16H18N2O5S/c1-28(41)15-5-4-6-18(35)19(15)23(36)20-16(28)13-17-22(31(2)3)24(37)21(26(39)29(17,42)25(20)38)27(40)30-14-33-9-7-32(8-10-33)11-12-34;1-16(2)12(15(21)22)18-13(20)11(14(18)24-16)17-10(19)8-23-9-6-4-3-5-7-9/h4-6,16-17,22,34-35,37-38,41-42H,7-14H2,1-3H3,(H,30,40);3-7,11-12,14H,8H2,1-2H3,(H,17,19)(H,21,22)/t16-,17-,22-,28+,29-;11-,12+,14-/m01/s1	C[C@@](C1=C(C2=O)C(O)=CC=C1)([C@@](C[C@@]3([H])[C@@](C(O)=C(C(NCN4CCN(CCO)CC4)=O)C5=O)(N(C)C)[H])(C2=C(O)[C@@]35O)[H])O.CC6([C@@H](N7C([C@@H](NC(COC8=CC=CC=C8)=O)[C@@]7([H])S6)=O)C(O)=O)C	Small ribosomal subunit inhibitor	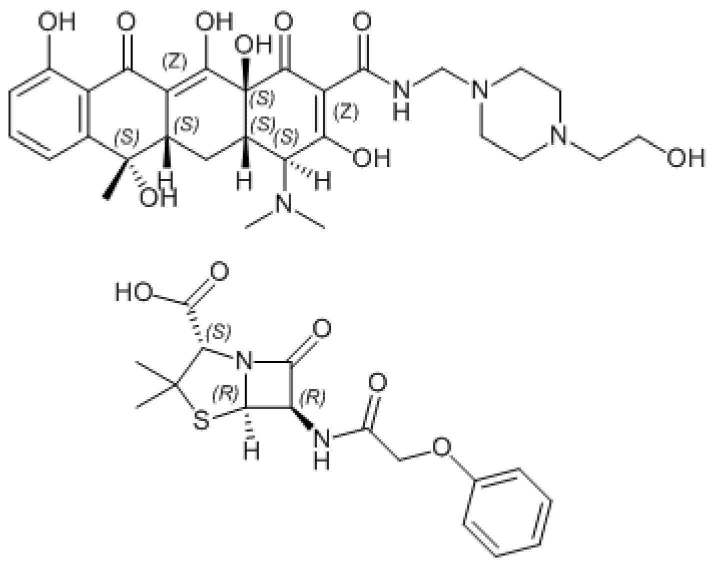	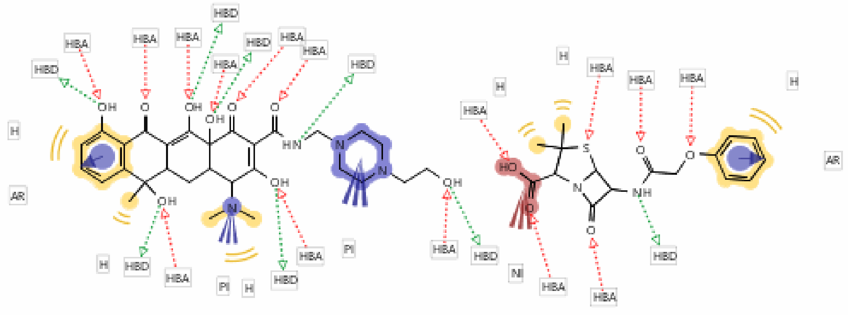
Penimocycline	16259-34-0	ZVVMCQBZCQRRME-YABKDXSZSA-N	InChI=1S/C39H43N5O12S/c1-37(2)29(36(53)54)44-34(52)25(35(44)57-37)42-33(51)24(16-10-7-6-8-11-16)40-15-41-32(50)23-28(47)26(43(4)5)19-14-18-22(30(48)39(19,56)31(23)49)27(46)21-17(38(18,3)55)12-9-13-20(21)45/h6-13,18-19,24-26,29,35,40,45,47-48,55-56H,14-15H2,1-5H3,(H,41,50)(H,42,51)(H,53,54)/t18-,19-,24+,25+,26-,29-,35+,38+,39-/m0/s1	CC1(C)[C@H](C(O)=O)N2C([C@H]([C@]2(S1)[H])NC([C@@]([H])(C3=CC=CC=C3)NCNC(C4=C([C@]([H])([C@@]5(C[C@]6(C(C(C7=C(C=CC=C7O)[C@@]6(C)O)=O)=C([C@@]5(C4=O)O)O)[H])[H])N(C)C)O)=O)=O)=O	Small ribosomal subunit inhibitor	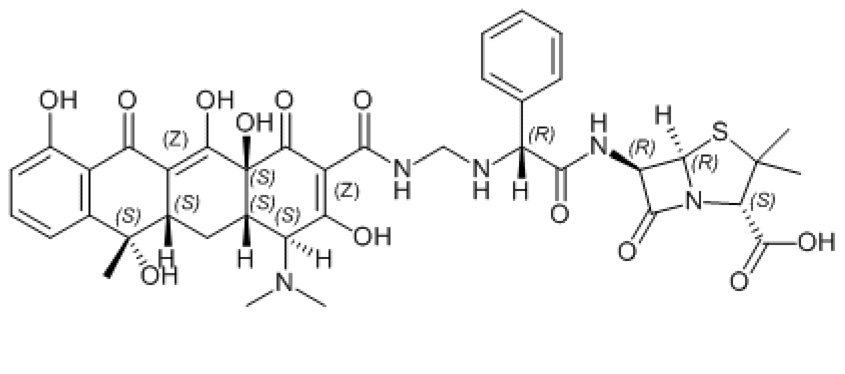	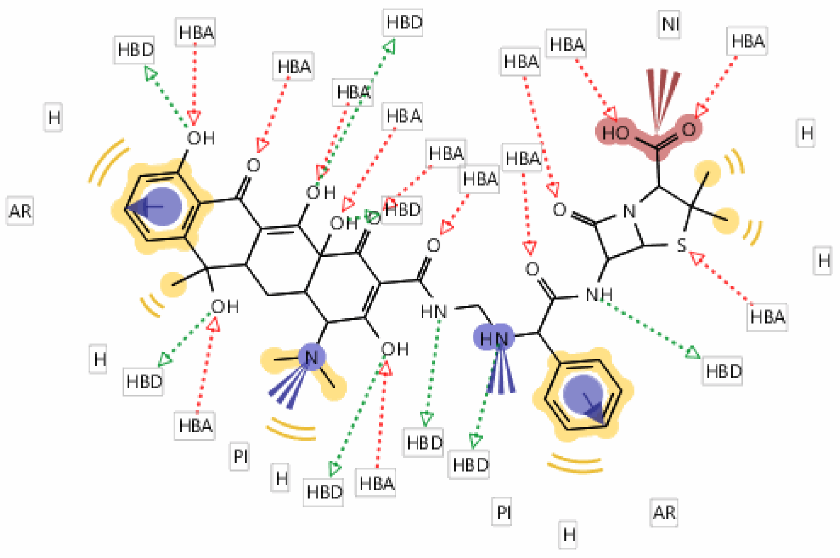

Common name	CAS Reg. No.	InChIKey	InChI	SMILES	Primary target	2D Structure	Pharmacophore[1]
Pipacycline	1110-80-1	XATZHCXBMKRRDO-REHNUXHNSA-N	InChI=1S/C29H38N4O9/c1-28(41)15-5-4-6-18(35)19(15)23(36)20-16(28)13-17-22(31(2)3)24(37)21(26(39)29(17,42)25(20)38)27(40)30-14-33-9-7-32(8-10-33)11-12-34/h4-6,16-17,22,34-35,37-38,41-42H,7-14H2,1-3H3,(H,30,40)/t16-,17-,22-,28+,29-/m0/s1	C[C@@](O)(C1=C(C(O)=CC=C1)C2=O)[C@@]([H])(C[C@]3([C@@]([H])(C(O)=C(C4=O)C(NCN5CCN(CC5)CCO)=O)N(C)C)[H])C2=C([C@@]34O)O	Small ribosomal subunit inhibitor	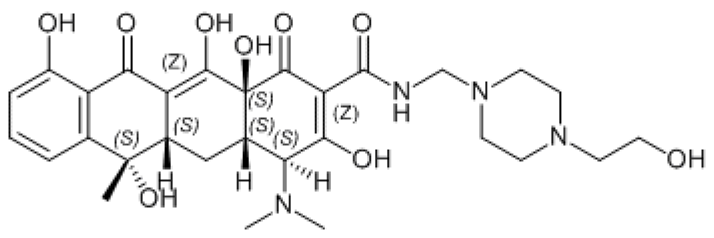	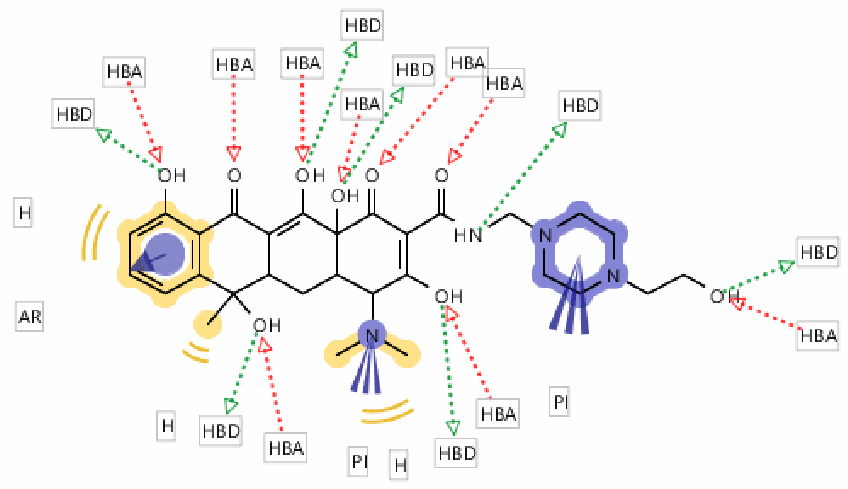
Rolitetracycline	751-97-3	HMEYVGGHISAPJR-IAHYZSEUSA-N	InChI=1S/C27H33N3O8/c1-26(37)13-7-6-8-16(31)17(13)21(32)18-14(26)11-15-20(29(2)3)22(33)19(24(35)27(15,38)23(18)34)25(36)28-12-30-9-4-5-10-30/h6-8,14-15,20,31,33-34,37-38H,4-5,9-12H2,1-3H3,(H,28,36)/t14-,15-,20-,26+,27-/m0/s1	C[C@@](O)(C1=C(C(O)=CC=C1)C2=O)[C@@]([H])(C[C@]3([C@@]([H])(C(O)=C(C4=O)C(NCN5CCCC5)=O)N(C)C)[H])C2=C([C@@]34O)O	Small ribosomal subunit inhibitor	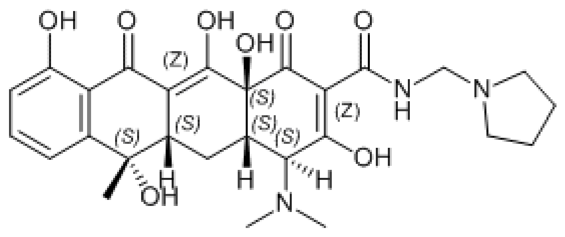	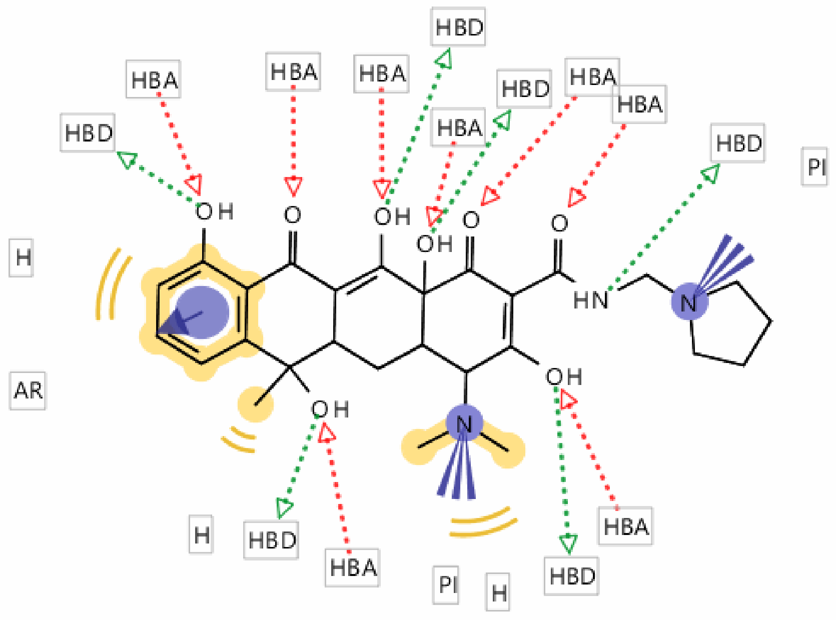
Sancycline	808-26-4	XDVCLKFLRAWGIT-ADOAZJKMSA-N	InChI=1S/C21H22N2O7/c1-23(2)15-10-7-9-6-8-4-3-5-11(24)12(8)16(25)13(9)18(27)21(10,30)19(28)14(17(15)26)20(22)29/h3-5,9-10,15,24,26-27,30H,6-7H2,1-2H3,(H2,22,29)/t9-,10-,15-,21-/m0/s1	CN(C)[C@]1([C@@]2(C[C@]([H])(CC3=C(C(O)=CC=C3)C4=O)C4=C([C@@]2(C(C(C(N)=O)=C1O)=O)O)O)[H])[H]	Small ribosomal subunit inhibitor	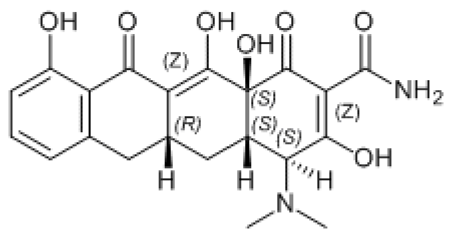	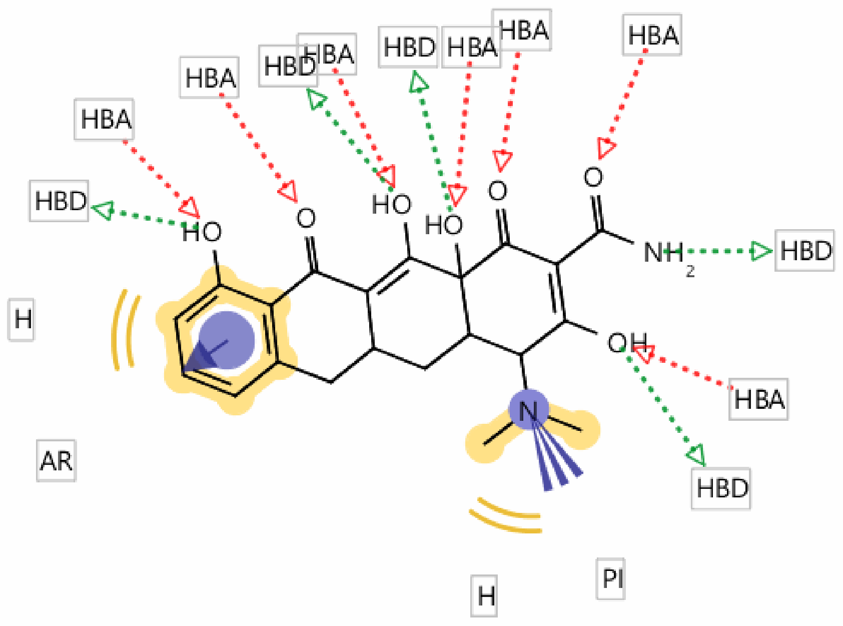
Sarecycline	1035654-66-0	PQJQFLNBMSCUSH-SBAJWEJLSA-N	InChI=1S/C24H29N3O8/c1-26(2)18-13-8-11-7-12-10(9-27(3)35-4)5-6-14(28)16(12)19(29)15(11)21(31)24(13,34)22(32)17(20(18)30)23(25)33/h5-6,11,13,18,28,30-31,34H,7-9H2,1-4H3,(H2,25,33)/t11-,13-,18-,24-/m0/s1	CN(C)[C@]1([C@@]2(C[C@]([H])(CC3=C(C=CC(O)=C3C4=O)CN(C)OC)C4=C([C@@]2(C(C(C(N)=O)=C1O)=O)O)O)[H])[H]	Small ribosomal subunit inhibitor	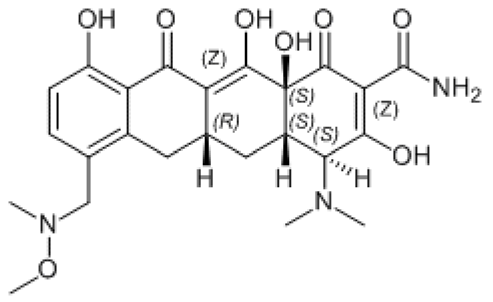	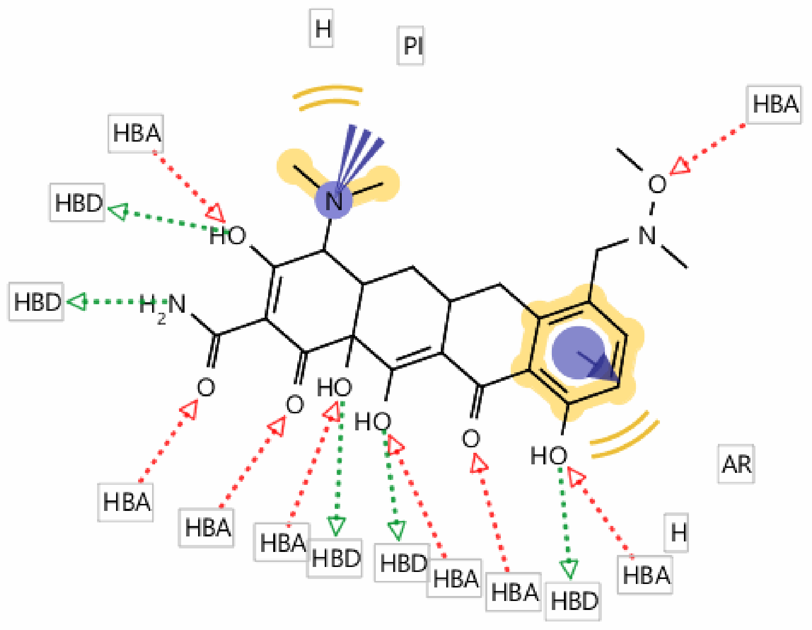
Tetracycline	60-54-8	OFVLGDICTFRJMM-WESIUVDSSA-N	InChI=1S/C22H24N2O8/c1-21(31)8-5-4-6-11(25)12(8)16(26)13-9(21)7-10-15(24(2)3)17(27)14(20(23)30)19(29)22(10,32)18(13)28/h4-6,9-10,15,25,27-28,31-32H,7H2,1-3H3,(H2,23,30)/t9-,10-,15-,21+,22-/m0/s1	C[C@@](O)(C1=C(C(O)=CC=C1)C2=O)[C@@]([H])(C[C@]3([C@@]([H])(C(O)=C(C4=O)C(N)=O)N(C)C)[H])C2=C([C@@]34O)O	Small ribosomal subunit inhibitor	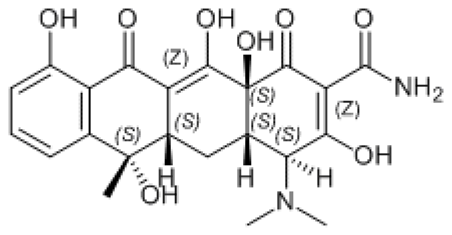	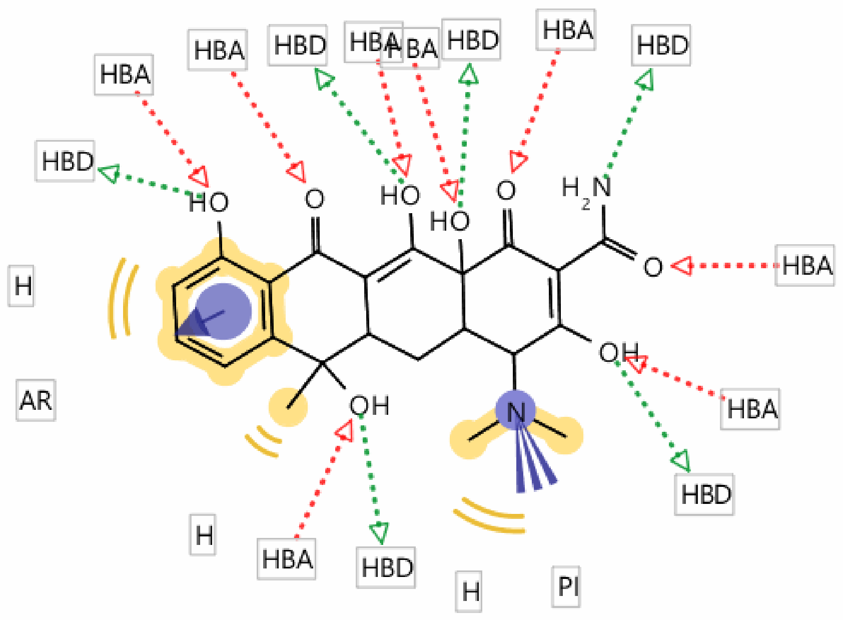
Tigecycline	220620-09-7	FPZLLRFZJZRHSY-HJYUBDRYSA-N	InChI=1S/C29H39N5O8/c1-28(2,3)31-11-17(35)32-15-10-16(33(4)5)13-8-12-9-14-21(34(6)7)24(38)20(27(30)41)26(40)29(14,42)25(39)18(12)23(37)19(13)22(15)36/h10,12,14,21,31,36,38-39,42H,8-9,11H2,1-7H3,(H2,30,41)(H,32,35)/t12-,14-,21-,29-/m0/s1	CC(NCC(NC1=CC(N(C)C)=C(C[C@@]([H])(C[C@]2([C@@]([H])(C(O)=C(C3=O)C(N)=O)N(C)C)[H])C(C4=O)=C([C@@]23O)O)C4=C1O)=O)(C)C	Small ribosomal subunit inhibitor	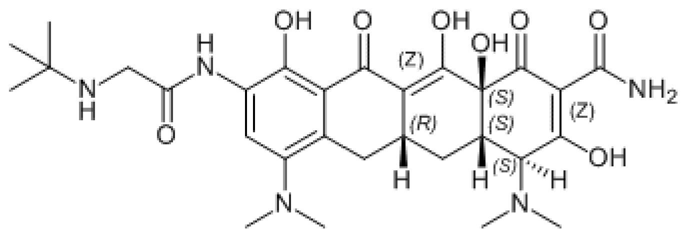	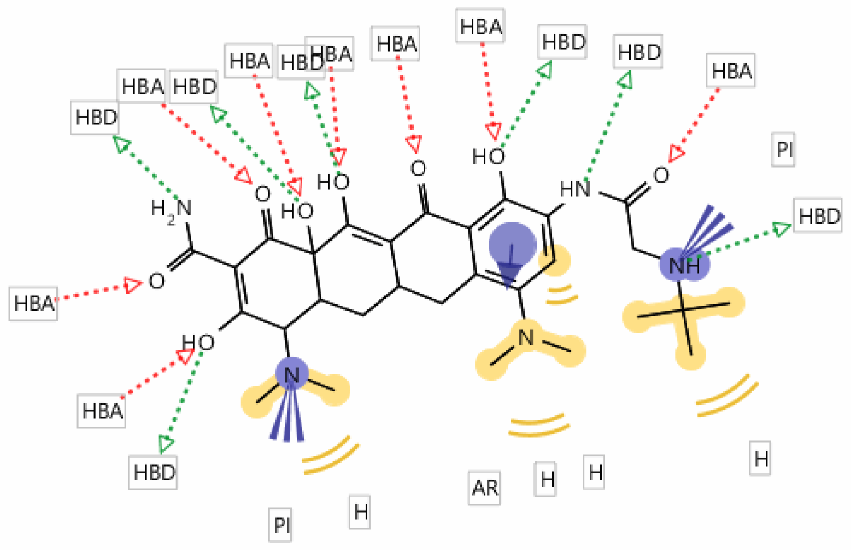

Common name	CAS Reg. No.	InChIKey	InChI	SMILES	Primary target	2D Structure	Pharmacophore[1]
TP 271	1207284-17-0	IXMIZHVJXGKPJI-HMFHYXQTSA-N	InChI=1S/C27H31FN4O8/c1-31(2)19-12-8-10-7-11-13(28)9-14(30-26(39)15-5-4-6-32(15)3)20(33)17(11)21(34)16(10)23(36)27(12,40)24(37)18(22(19)35)25(29)38/h9-10,12,15,19,33,35-36,40H,4-8H2,1-3H3,(H2,29,38)(H,30,39)/t10-,12-,15-,19-,27-/m0/s1	CN(C)[C@]1([C@@]2(C[C@]([H])(CC3=C4C(O)=C(C=C3F)NC([C@@H]5CCCN5C)=O)C(C4=O)=C([C@@]2(C(C(C(N)=O)=C1O)=O)O)O)[H])[H]	Small ribosomal subunit inhibitor	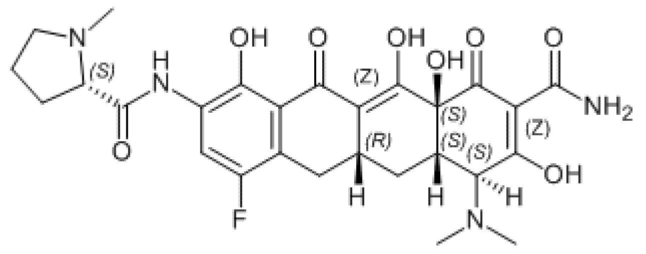	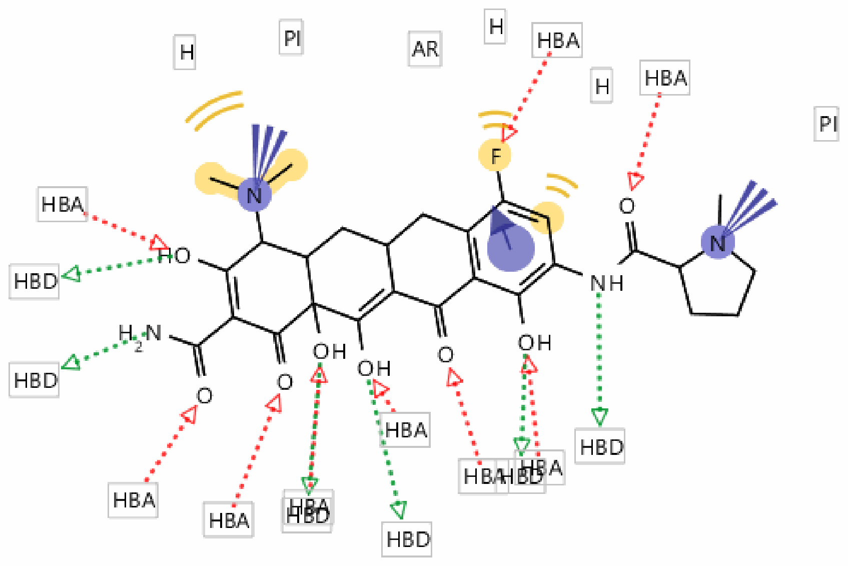
Albaconazole	187949-02-6	UHIXWHUVLCAJQL-MPBGBICISA-N	InChI=1S/C20H16ClF2N5O2/c1-12(28-11-25-18-6-13(21)2-4-15(18)19(28)29)20(30,8-27-10-24-9-26-27)16-5-3-14(22)7-17(16)23/h2-7,9-12,30H,8H2,1H3/t12-,20-/m1/s1	C[C@H]([C@](O)(CN1C=NC=N1)C2=C(C=C(C=C2)F)F)N3C=NC4=C(C=CC(Cl)=C4)C3=O	Sterol 14α-demethylase inhibitor	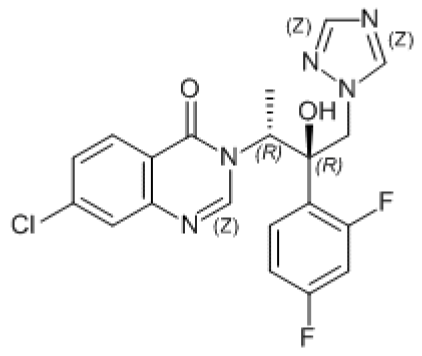	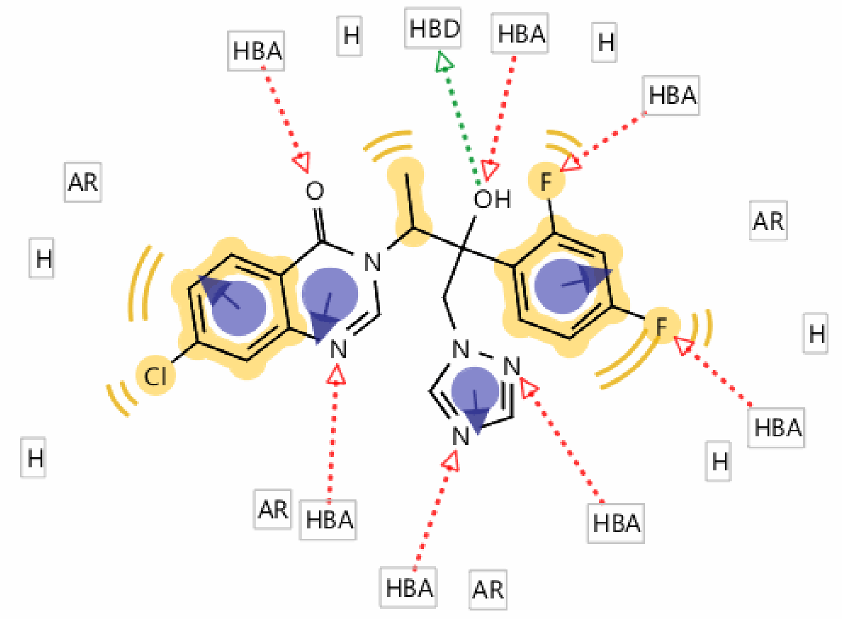
Alteconazole	93479-96-0	AXDYHXZBKXITAA-TYDMEHBZSA-N	InChI=1S/2C17H12Cl3N3O/c2*18-12-3-1-11(2-4-12)17(8-23-10-21-9-22-23)16(24-17)14-6-5-13(19)7-15(14)20/h2*1-7,9-10,16H,8H2/t2*16-,17-/m10/s1	ClC1=CC=C(C=C1)[C@]2(CN3C=NC=N3)[C@@H](C4=C(C=C(C=C4)Cl)Cl)O2.ClC5=CC=C(C=C5)[C@@]6(CN7C=NC=N7)[C@H](C8=C(C=C(C=C8)Cl)Cl)O6	Sterol 14α-demethylase inhibitor	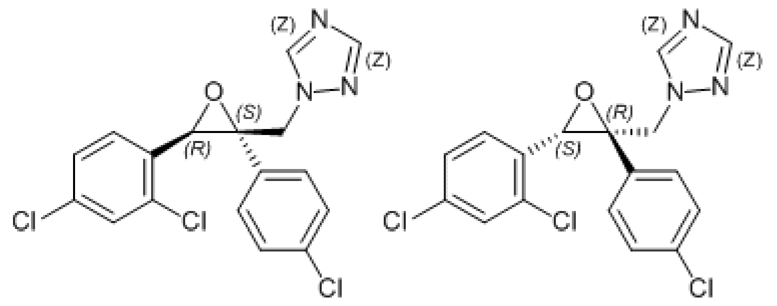	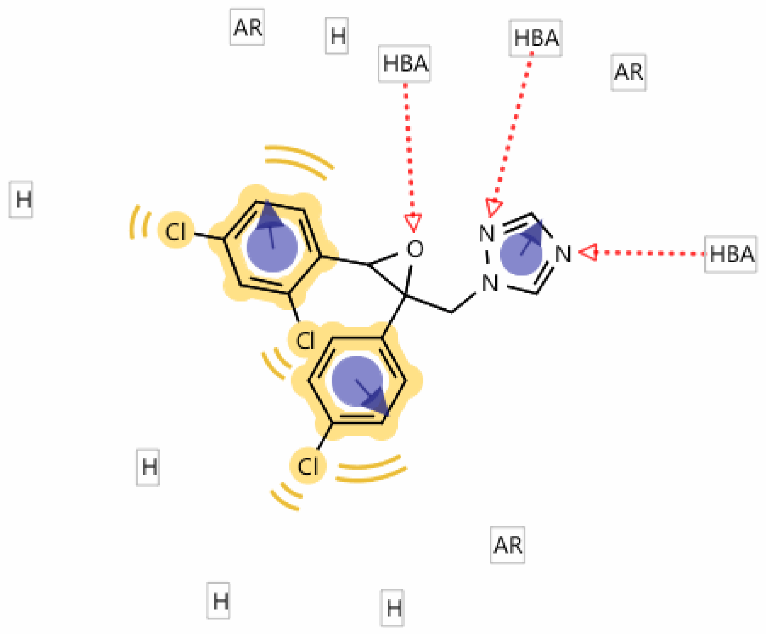
Azaconazole	60207-31-0	AKNQMEBLVAMSNZ-UHFFFAOYSA-N	InChI=1S/C12H11Cl2N3O2/c13-9-1-2-10(11(14)5-9)12(18-3-4-19-12)6-17-8-15-7-16-17/h1-2,5,7-8H,3-4,6H2	ClC1=CC(Cl)=C(C2(OCCO2)CN3C=NC=N3)C=C1	Sterol 14α-demethylase inhibitor	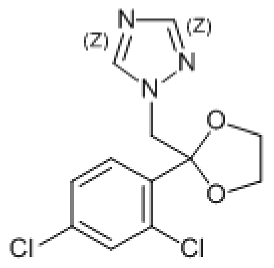	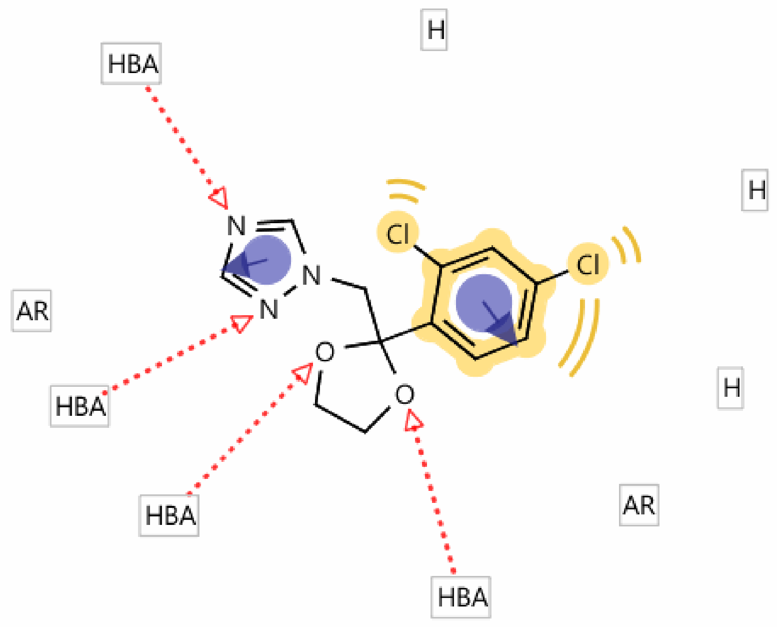
BAS 110	83223-83-0	UOSZFOHRMATYAF-ROIIEEENSA-N	InChI=1S/4C12H13Cl2N3O2/c4*1-12(18,9-4-3-8(13)5-10(9)14)11(19-2)17-7-15-6-16-17/h4*3-7,11,18H,1-2H3/t2*11-,12+;2*11-,12-/m1010/s1	C[C@](O)(C1=C(C=C(C=C1)Cl)Cl)[C@]([H])(N2C=NC=N2)OC.C[C@@](O)(C3=C(C=C(C=C3)Cl)Cl)[C@@]([H])(N4C=NC=N4)OC.C[C@@](O)(C5=C(C=C(C=C5)Cl)Cl)[C@]([H])(N6C=NC=N6)OC.C[C@](O)(C7=C(C=C(C=C7)Cl)Cl)[C@@]([H])(N8C=NC=N8)OC	Sterol 14α-demethylase inhibitor	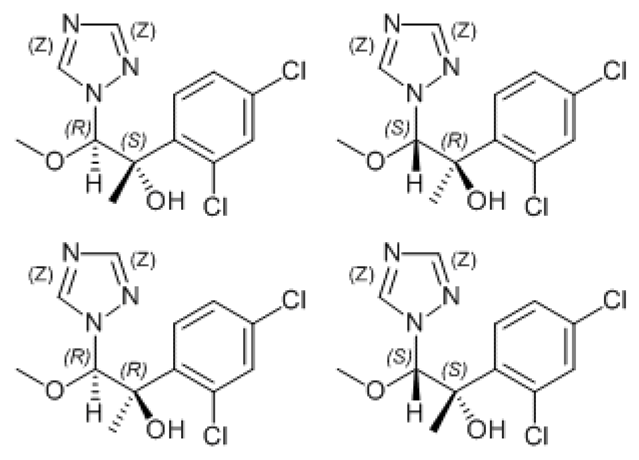	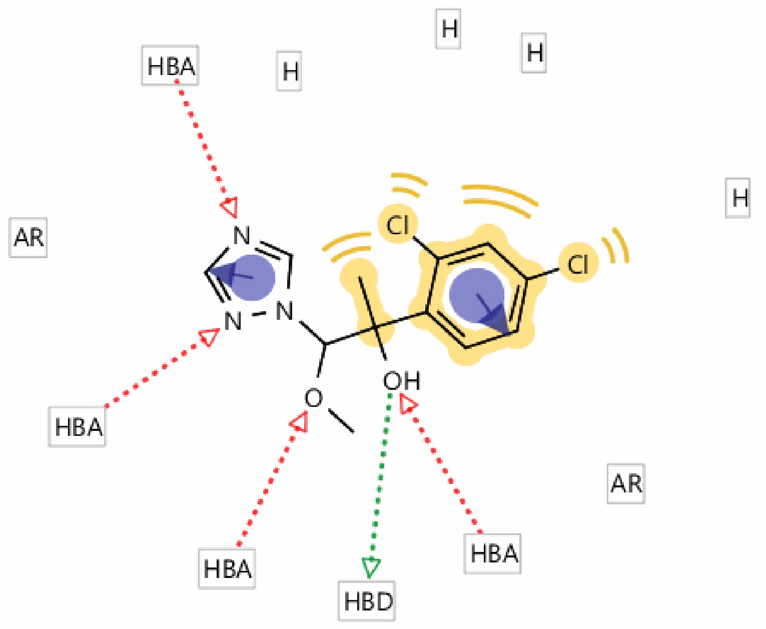
BAS 111	80553-79-3	KAEVVAWQVXIYPH-MVMUQWOCSA-N	InChI=1S/4C16H23N3O2/c4*1-16(2,3)15(20)14(19-12-17-11-18-19)9-10-21-13-7-5-4-6-8-13/h4*4-8,11-12,14-15,20H,9-10H2,1-3H3/t2*14-,15+;2*14-,15-/m1010/s1	CC([C@H]([C@@]([H])(CCOC1=CC=CC=C1)N2C=NC=N2)O)(C)C.CC([C@@H]([C@]([H])(CCOC3=CC=CC=C3)N4C=NC=N4)O)(C)C.CC([C@@H]([C@@]([H])(CCOC5=CC=CC=C5)N6C=NC=N6)O)(C)C.CC([C@H]([C@]([H])(CCOC7=CC=CC=C7)N8C=NC=N8)O)(C)C	Sterol 14α-demethylase inhibitor	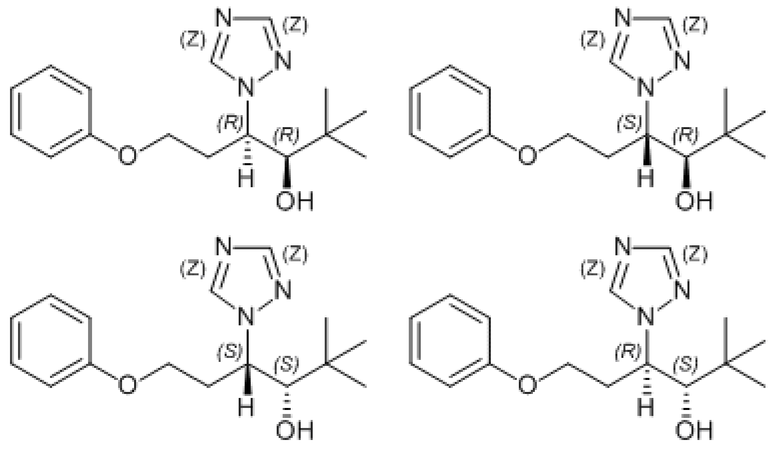	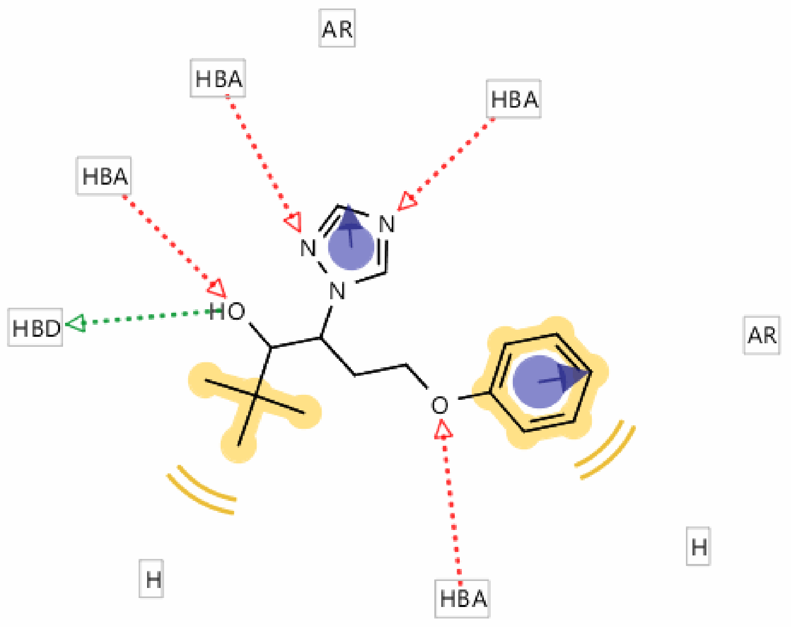

Common name	CAS Reg. No.	InChIKey	InChI	SMILES	Primary target	2D Structure	Pharmacophore[1]
BAS 45406F	77562-07-3	PFSFZMGHUUOHON-XLNRJJMWSA-N	InChI=1S/C17H14Cl2N4O/c18-14-6-7-15(16(19)8-14)17(9-23-12-20-11-21-23)22-24-10-13-4-2-1-3-5-13/h1-8,11-12H,9-10H2/b22-17-	ClC1=CC(Cl)=C(/C(CN2C=NC=N2)=N\OCC3=CC=CC=C3)C=C1	Sterol 14α-demethylase inhibitor	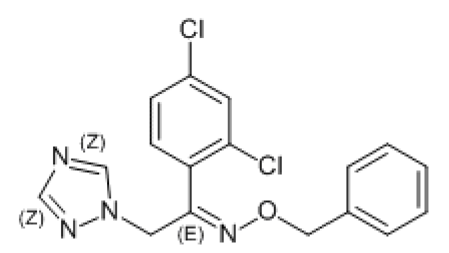	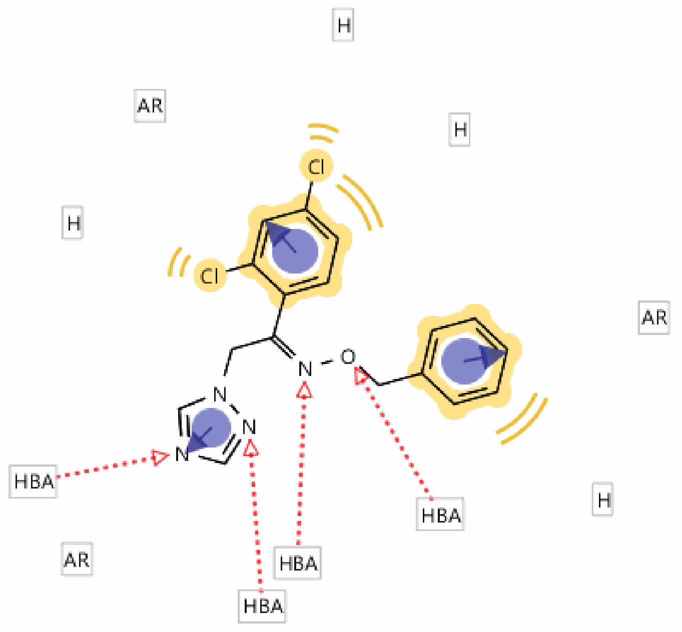
Bitertanol	55179-31-2	SPVIDRSYTTWGHG-UWFRYYLNSA-N	InChI=1S/4C20H23N3O2/c4*1-20(2,3)18(24)19(23-14-21-13-22-23)25-17-11-9-16(10-12-17)15-7-5-4-6-8-15/h4*4-14,18-19,24H,1-3H3/t2*18-,19+;2*18-,19-/m1010/s1	CC(C)(C)[C@H](O)[C@@](N1C=NC=N1)(OC2=CC=C(C3=CC=CC=C3)C=C2)[H].CC(C)(C)[C@@H](O)[C@](N4C=NC=N4)(OC5=CC=C(C6=CC=CC=C6)C=C5)[H].CC(C)(C)[C@H](O)[C@](N7C=NC=N7)(OC8=CC=C(C9=CC=CC=C9)C=C8)[H].CC(C)(C)[C@@H](O)[C@@](N%10C=NC=N%10)(OC%11=CC=C(C%12=CC=CC=C%12)C=C%11)[H]	Sterol 14α-demethylase inhibitor	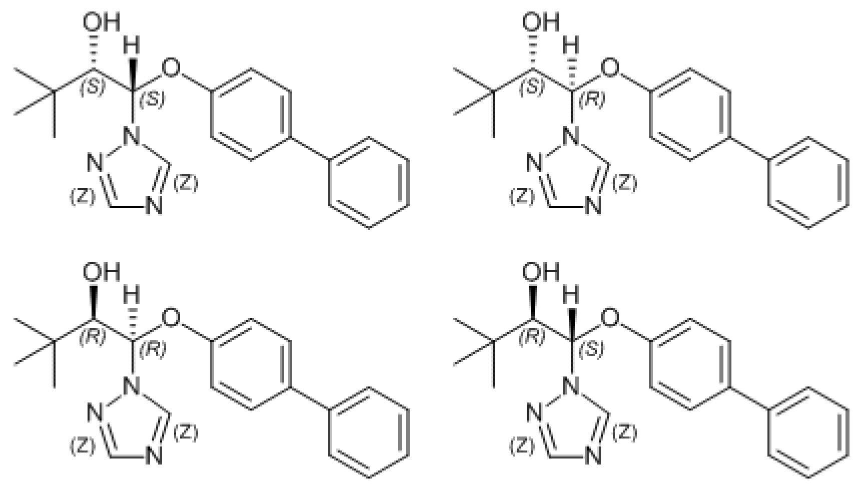	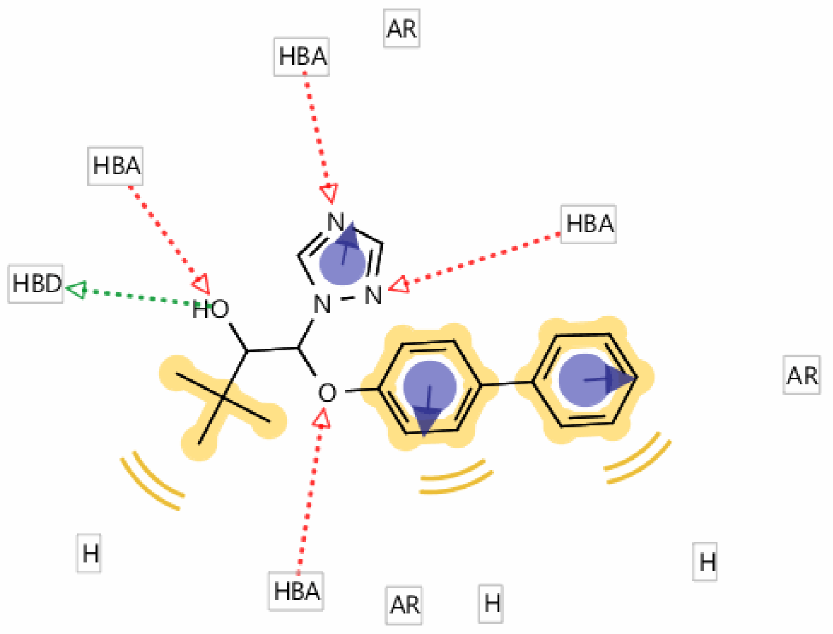
Bromuconazole	116255-48-2	RQBZXFVUTSGWJQ-RLWGQDEYSA-N	InChI=1S/4C13H12BrCl2N3O/c4*14-9-4-13(20-5-9,6-19-8-17-7-18-19)11-2-1-10(15)3-12(11)16/h4*1-3,7-9H,4-6H2/t2*9-,13+;2*9-,13-/m1010/s1	Br[C@H]1CO[C@](C2=C(C=C(C=C2)Cl)Cl)(CN3C=NC=N3)C1.Br[C@@H]4CO[C@@](C5=C(C=C(C=C5)Cl)Cl)(CN6C=NC=N6)C4.Br[C@H]7CO[C@@](C8=C(C=C(C=C8)Cl)Cl)(CN9C=NC=N9)C7.Br[C@@H]%10CO[C@](C%11=C(C=C(C=C%11)Cl)Cl)(CN%12C=NC=N%12)C%10	Sterol 14α-demethylase inhibitor	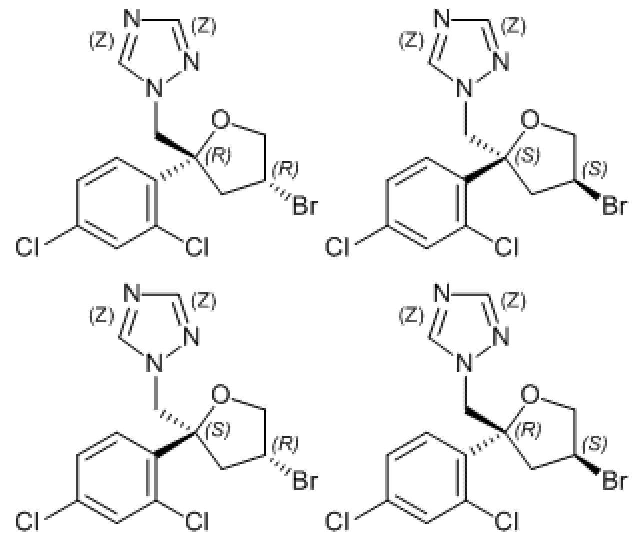	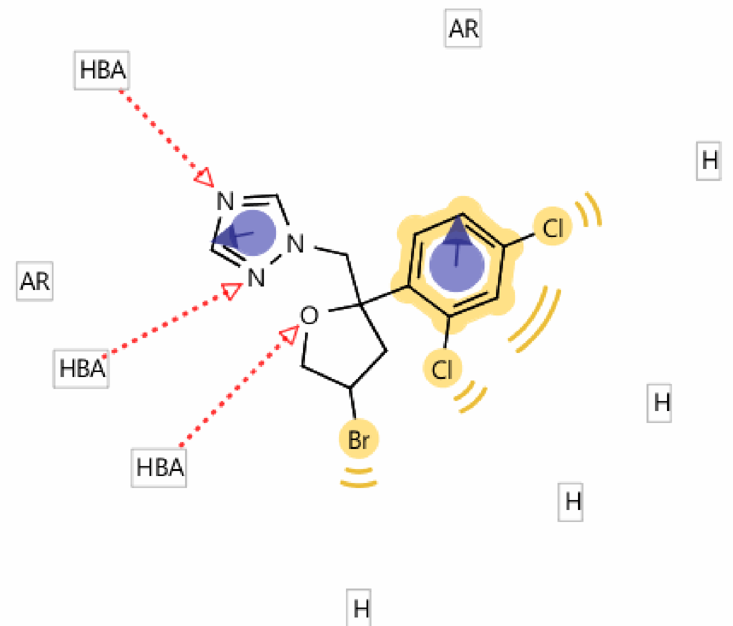
Cyproconazole	94361-06-5	LEZZHEWSNKPMOI-MACVOLPTSA-N	InChI=1S/4C15H18ClN3O/c4*1-11(12-2-3-12)15(20,8-19-10-17-9-18-19)13-4-6-14(16)7-5-13/h4*4-7,9-12,20H,2-3,8H2,1H3/t2*11-,15+;2*11-,15-/m1010/s1	C[C@H](C1CC1)[C@@](O)(CN2C=NC=N2)C3=CC=C(C=C3)Cl.C[C@@H](C4CC4)[C@](O)(CN5C=NC=N5)C6=CC=C(C=C6)Cl.C[C@H](C7CC7)[C@](O)(CN8C=NC=N8)C9=CC=C(C=C9)Cl.C[C@@H](C%10CC%10)[C@@](O)(CN%11C=NC=N%11)C%12=CC=C(C=C%12)Cl	Sterol 14α-demethylase inhibitor	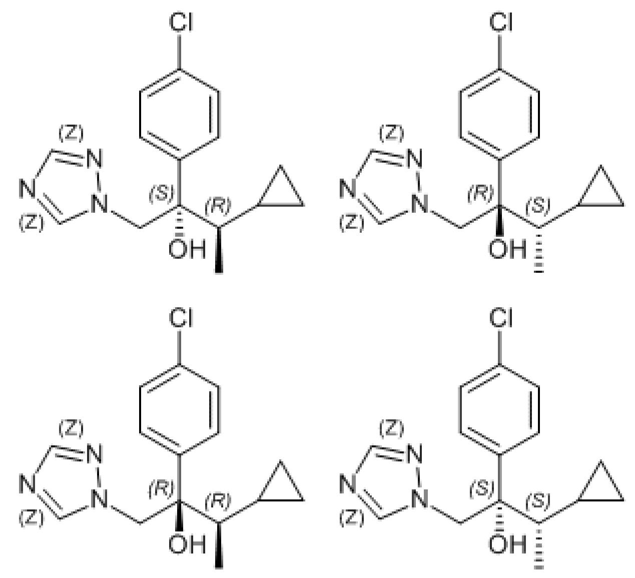	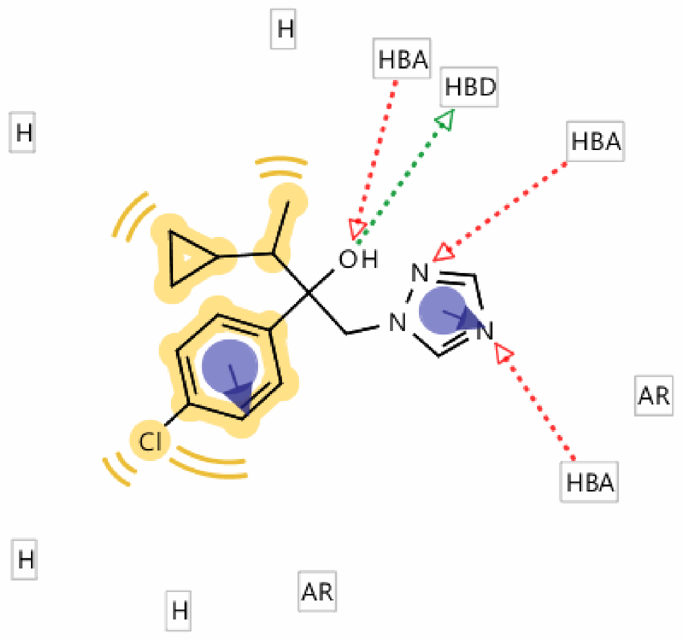
D 0870	149715-95-7	FZEJTXCSLUORDW-BGLDCGIESA-N	InChI=1S/C24H20F6N6O2/c25-17-4-7-19(20(26)9-17)23(37,10-35-14-31-13-33-35)11-36-15-32-21(34-36)8-3-16-1-5-18(6-2-16)38-12-24(29,30)22(27)28/h1-9,13-15,22,37H,10-12H2/b8-3-/t23-/m1/s1	FC1=CC(F)=C([C@@](CN2C=NC=N2)(CN3C=NC(/C=C\C4=CC=C(OCC(C(F)F)(F)F)C=C4)=N3)O)C=C1	Sterol 14α-demethylase inhibitor	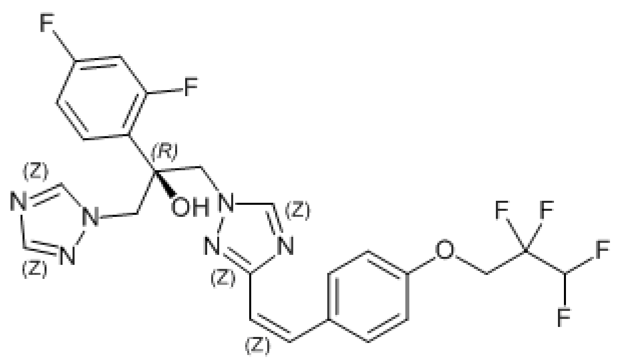	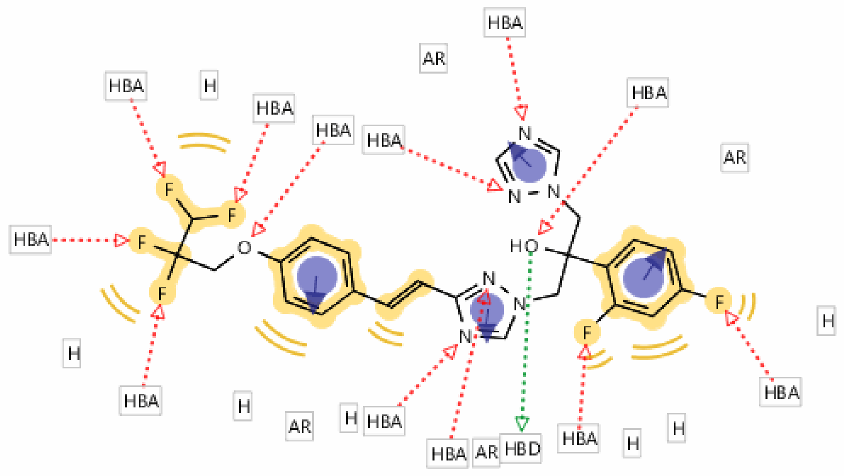
Diclobutrazol	75736-33-3	PLPRPHZNQBHGSP-DZYJZKBLSA-N	InChI=1S/2C15H19Cl2N3O/c2*1-15(2,3)14(21)13(20-9-18-8-19-20)6-10-4-5-11(16)7-12(10)17/h2*4-5,7-9,13-14,21H,6H2,1-3H3/t2*13-,14+/m10/s1	CC([C@H]([C@@]([H])(CC1=C(C=C(C=C1)Cl)Cl)N2C=NC=N2)O)(C)C.CC([C@@H]([C@]([H])(CC3=C(C=C(C=C3)Cl)Cl)N4C=NC=N4)O)(C)C	Sterol 14α-demethylase inhibitor	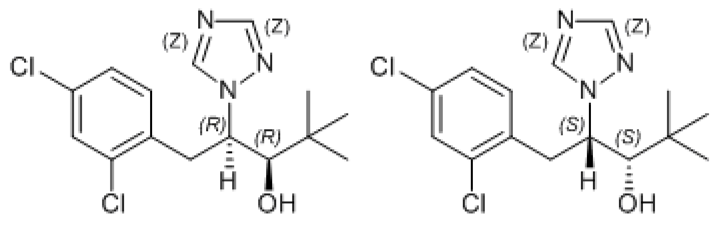	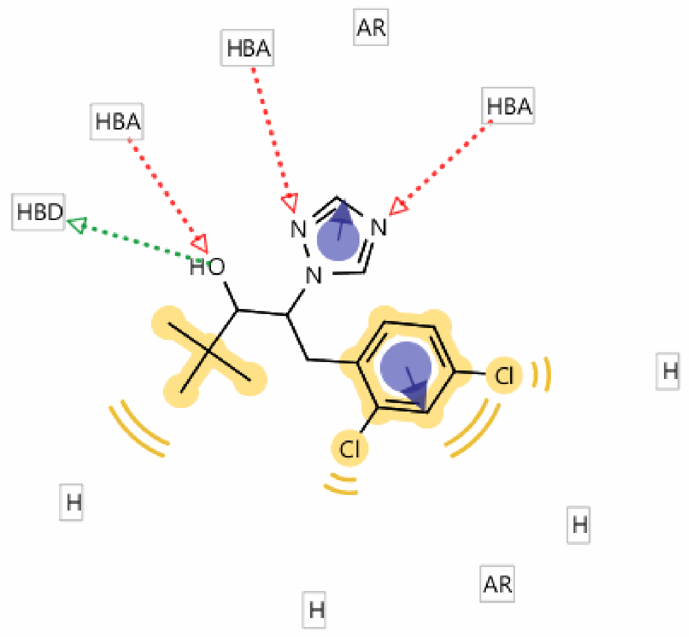

Common name	CAS Reg. No.	InChIKey	InChI	SMILES	Primary target	2D Structure	Pharmacophore[1]
Difenoconazole	119446-68-3	FQNUBDIMKXZYAC-HMLBIANXSA-N	InChI=1S/4C19H17Cl2N3O3/c4*1-13-9-25-19(27-13,10-24-12-22-11-23-24)17-7-6-16(8-18(17)21)26-15-4-2-14(20)3-5-15/h4*2-8,11-13H,9-10H2,1H3/t2*13-,19+;2*13-,19-/m1010/s1	C[C@H]1O[C@](CN2C=NC=N2)(OC1)C3=C(Cl)C=C(OC4=CC=C(Cl)C=C4)C=C3.C[C@@H]5O[C@@](CN6C=NC=N6)(OC5)C7=C(Cl)C=C(OC8=CC=C(Cl)C=C8)C=C7.C[C@H]9O[C@@](CN%10C=NC=N%10)(OC9)C%11=C(Cl)C=C(OC%12=CC=C(Cl)C=C%12)C=C%11.C[C@@H]%13O[C@](CN%14C=NC=N%14)(OC%13)C%15=C(Cl)C=C(OC%16=CC=C(Cl)C=C%16)C=C%15	Sterol 14α-demethylase inhibitor	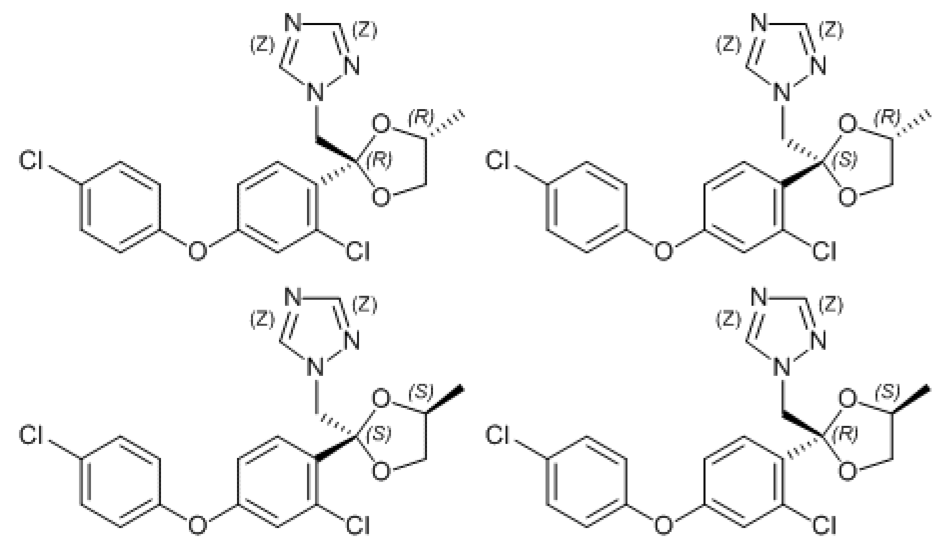	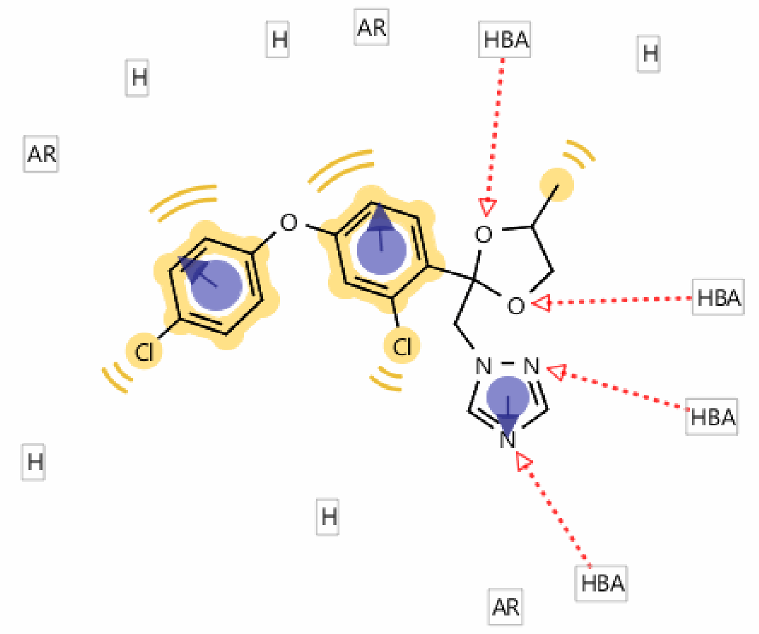
Diniconazole	83657-24-3	HFLXXQLOUZRCHU-FATSGVOGSA-N	InChI=1S/2C15H17Cl2N3O/c2*1-15(2,3)14(21)13(20-9-18-8-19-20)6-10-4-5-11(16)7-12(10)17/h2*4-9,14,21H,1-3H3/b2*13-6+/t2*14-/m10/s1	O[C@H](/C(N1C=NC=N1)=C\C2=C(Cl)C=C(Cl)C=C2)C(C)(C)C.O[C@@H](/C(N3C=NC=N3)=C\C4=C(Cl)C=C(Cl)C=C4)C(C)(C)C	Sterol 14α-demethylase inhibitor	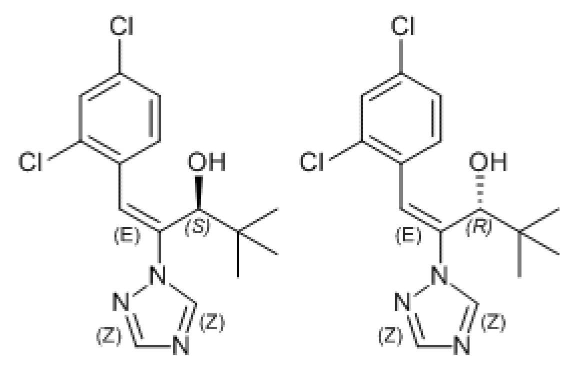	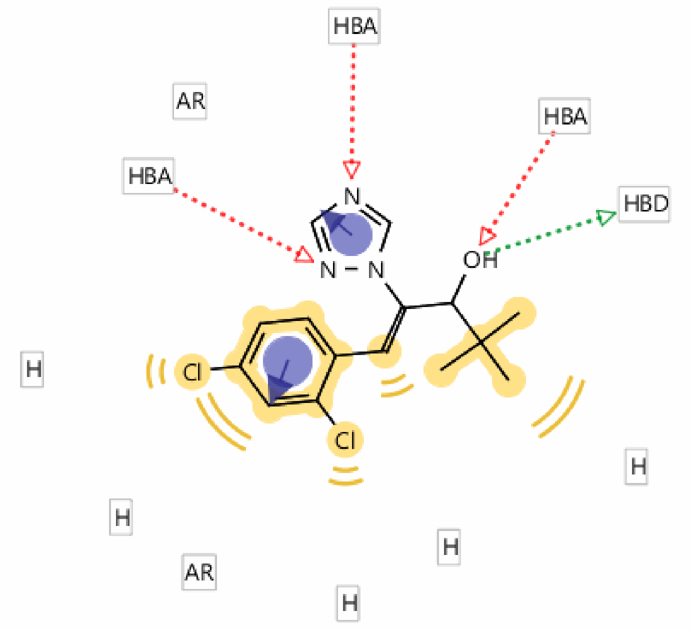
Efinaconazole	164650-44-6	NFEZZTICAUWDHU-RDTXWAMCSA-N	InChI=1S/C18H22F2N4O/c1-13-5-7-23(8-6-13)14(2)18(25,10-24-12-21-11-22-24)16-4-3-15(19)9-17(16)20/h3-4,9,11-12,14,25H,1,5-8,10H2,2H3/t14-,18-/m1/s1	C=C1CCN(CC1)[C@H](C)[C@](O)(CN2C=NC=N2)C3=C(C=C(C=C3)F)F	Sterol 14α-demethylase inhibitor	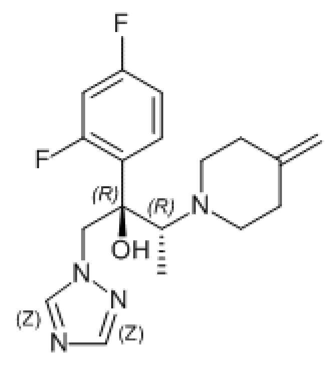	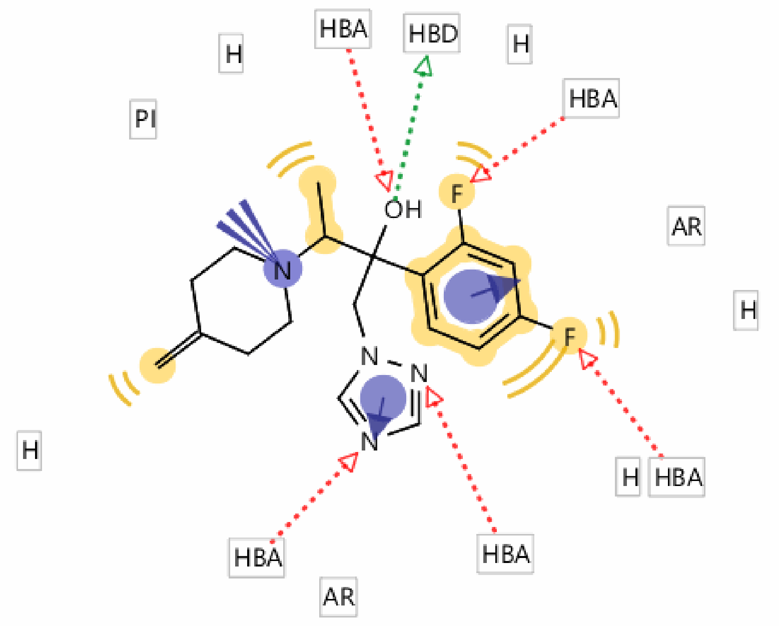
Embeconazole	329744-44-7	XSRKBFUVSRRJDX-GGZJQHSXSA-N	InChI=1S/C27H25F3N4O3S/c1-18(27(35,15-34-17-32-16-33-34)23-9-8-21(28)11-25(23)30)38-22-13-36-26(37-14-22)5-3-2-4-20-7-6-19(12-31)10-24(20)29/h2-11,16-18,22,26,35H,13-15H2,1H3/b4-2+,5-3+/t18-,22-,26-,27-/m1/s1	C[C@H]([C@](O)(CN1C=NC=N1)C2=C(C=C(C=C2)F)F)S[C@@]3([H])CO[C@]([H])(/C=C/C=C/C4=C(C=C(C=C4)C#N)F)OC3	Sterol 14α-demethylase inhibitor	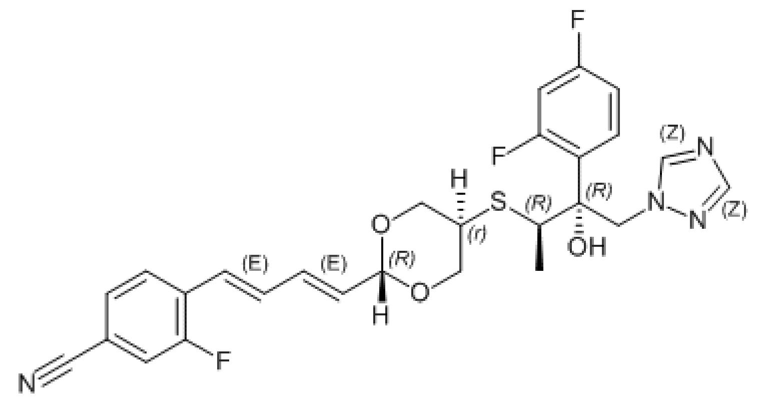	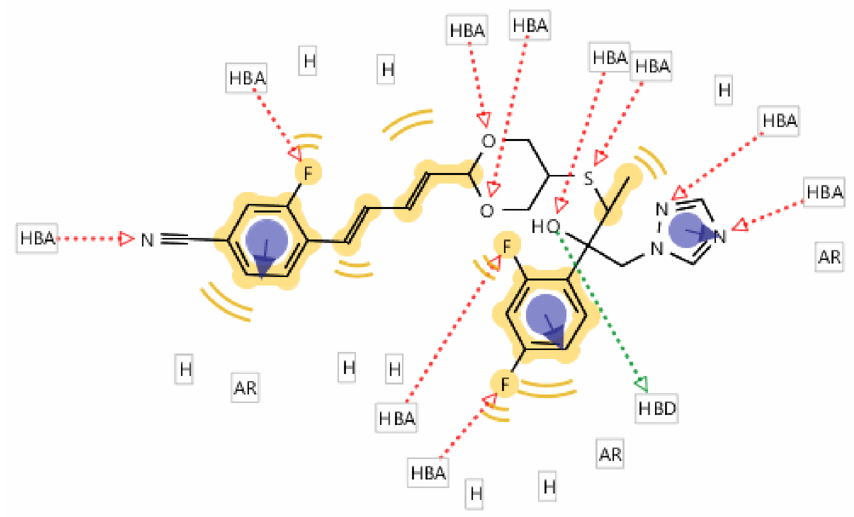
Epoxiconazole	133855-98-8	FEVYAAHIKWPJNV-TYDMEHBZSA-N	InChI=1S/2C17H13ClFN3O/c2*18-15-4-2-1-3-14(15)16-17(23-16,9-22-11-20-10-21-22)12-5-7-13(19)8-6-12/h2*1-8,10-11,16H,9H2/t2*16-,17-/m10/s1	ClC1=CC=CC=C1[C@H]2O[C@]2(CN3C=NC=N3)C4=CC=C(C=C4)F.ClC5=CC=CC=C5[C@@H]6O[C@@]6(CN7C=NC=N7)C8=CC=C(C=C8)F	Sterol 14α-demethylase inhibitor	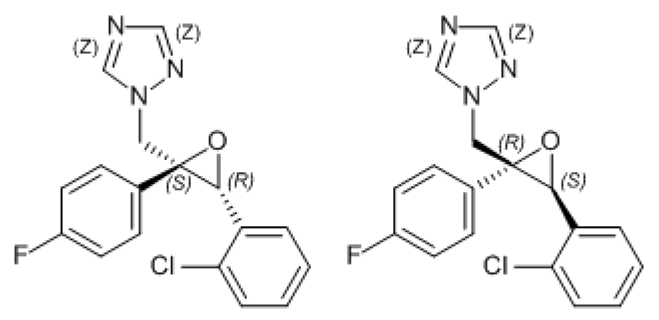	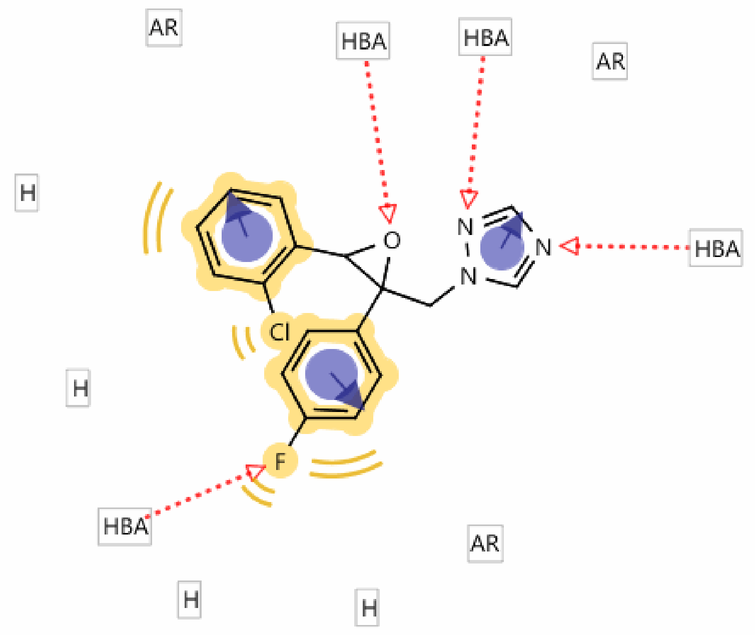
Etaconazole	60207-93-4	JFMUAODQVWMYRW-KKRXSGBFSA-N	InChI=1S/4C14H15Cl2N3O2/c4*1-2-11-6-20-14(21-11,7-19-9-17-8-18-19)12-4-3-10(15)5-13(12)16/h4*3-5,8-9,11H,2,6-7H2,1H3/t2*11-,14+;2*11-,14-/m1010/s1	CC[C@H]1O[C@](C2=C(C=C(C=C2)Cl)Cl)(CN3C=NC=N3)OC1.CC[C@@H]4O[C@@](C5=C(C=C(C=C5)Cl)Cl)(CN6C=NC=N6)OC4.CC[C@H]7O[C@@](C8=C(C=C(C=C8)Cl)Cl)(CN9C=NC=N9)OC7.CC[C@@H]%10O[C@](C%11=C(C=C(C=C%11)Cl)Cl)(CN%12C=NC=N%12)OC%10	Sterol 14α-demethylase inhibitor	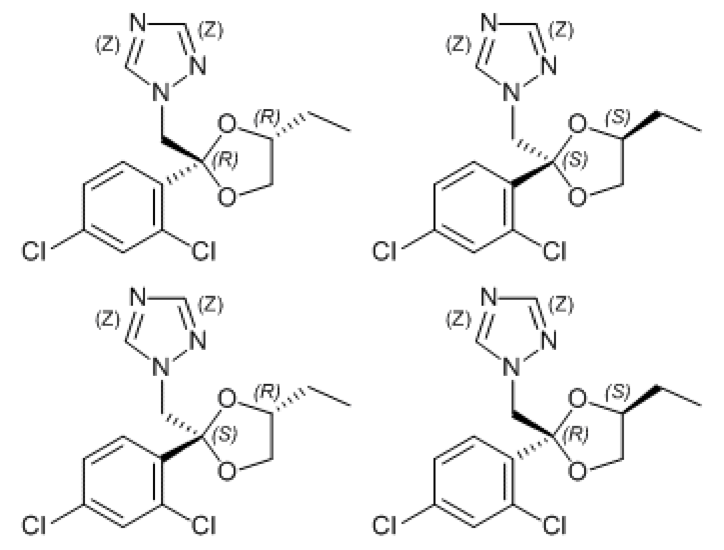	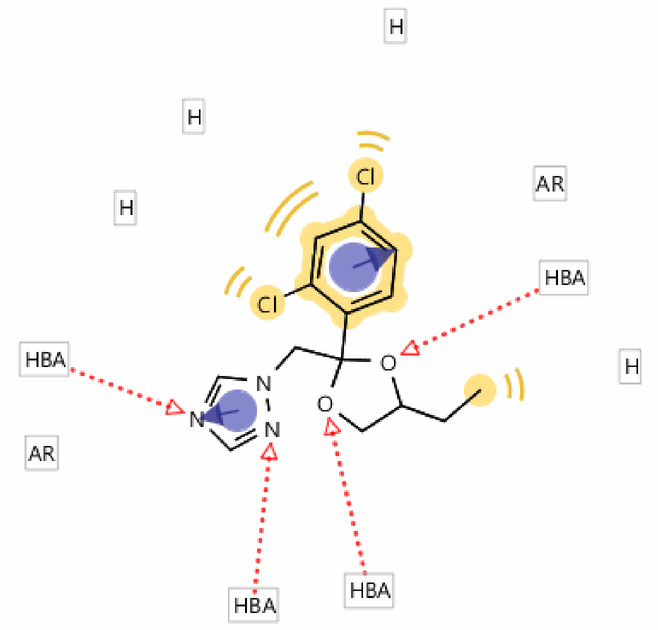

Common name	CAS Reg. No.	InChIKey	InChI	SMILES	Primary target	2D Structure	Pharmacophore[1]
Fenbuconazole	114369-43-6	QSYNIAKLTBPGTE-OYPHMNEHSA-N	InChI=1S/2C19H17ClN4/c2*20-18-8-6-16(7-9-18)10-11-19(12-21,13-24-15-22-14-23-24)17-4-2-1-3-5-17/h2*1-9,14-15H,10-11,13H2/t2*19-/m10/s1	ClC1=CC=C(C=C1)CC[C@@](C2=CC=CC=C2)(C#N)CN3C=NC=N3.ClC4=CC=C(C=C4)CC[C@](C5=CC=CC=C5)(C#N)CN6C=NC=N6	Sterol 14α-demethylase inhibitor	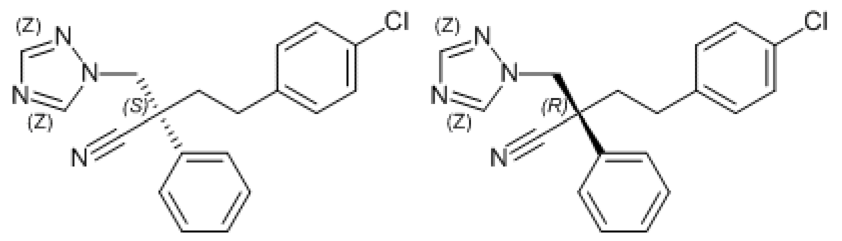	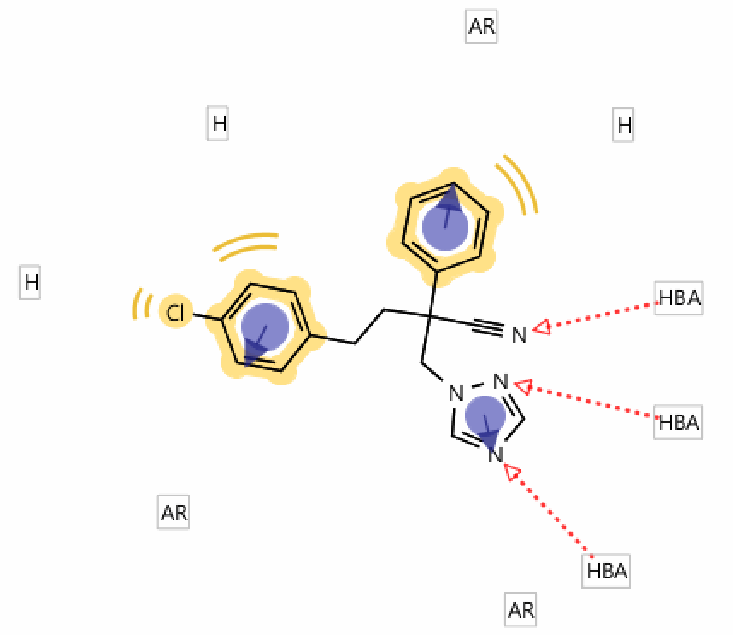
Fluconazole	86386-73-4	RFHAOTPXVQNOHP-UHFFFAOYSA-N	InChI=1S/C13H12F2N6O/c14-10-1-2-11(12(15)3-10)13(22,4-20-8-16-6-18-20)5-21-9-17-7-19-21/h1-3,6-9,22H,4-5H2	FC1=CC(F)=C(C(CN2C=NC=N2)(CN3C=NC=N3)O)C=C1	Sterol 14α-demethylase inhibitor	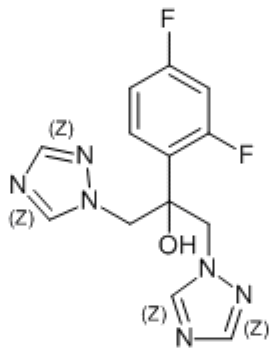	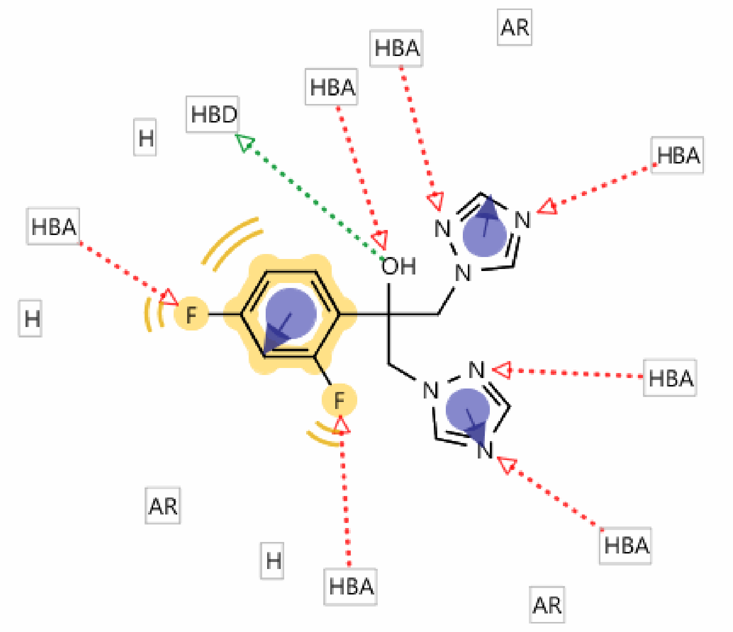
Fluotrimazole	31251-03-3	LXMQMMSGERCRSU-UHFFFAOYSA-N	InChI=1S/C22H16F3N3/c23-22(24,25)20-13-7-12-19(14-20)21(28-16-26-15-27-28,17-8-3-1-4-9-17)18-10-5-2-6-11-18/h1-16H	FC(F)(C1=CC=CC(C(N2C=NC=N2)(C3=CC=CC=C3)C4=CC=CC=C4)=C1)F	Sterol 14α-demethylase inhibitor	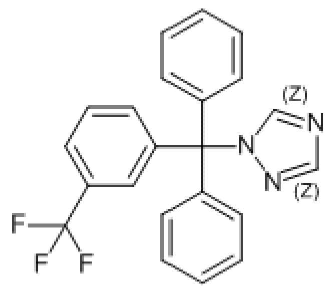	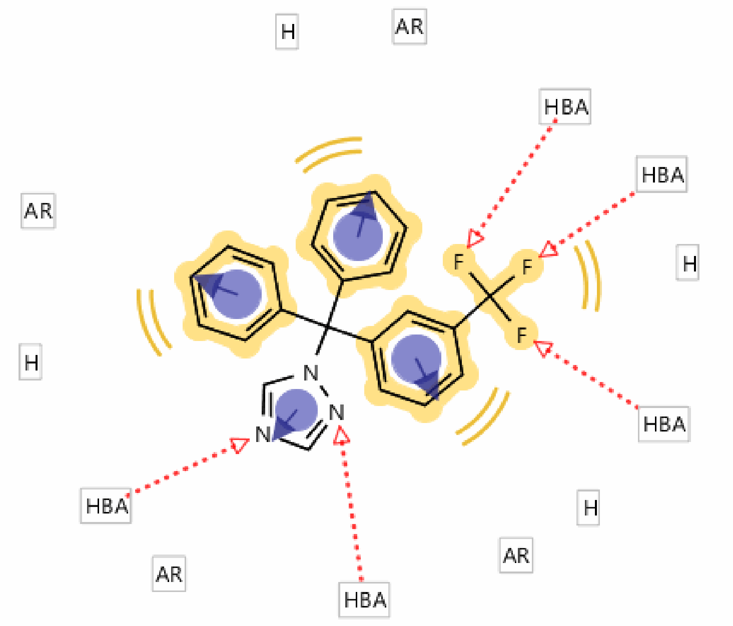
Fluquinconazole	136426-54-5	IJJVMEJXYNJXOJ-UHFFFAOYSA-N	InChI=1S/C16H8Cl2FN5O/c17-9-1-4-14(12(18)5-9)24-15(25)11-6-10(19)2-3-13(11)22-16(24)23-8-20-7-21-23/h1-8H	ClC1=CC(Cl)=C(C=C1)N2C(N3C=NC=N3)=NC(C=CC(F)=C4)=C4C2=O	Sterol 14α-demethylase inhibitor	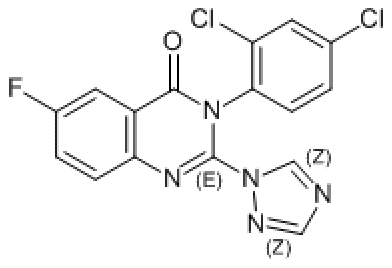	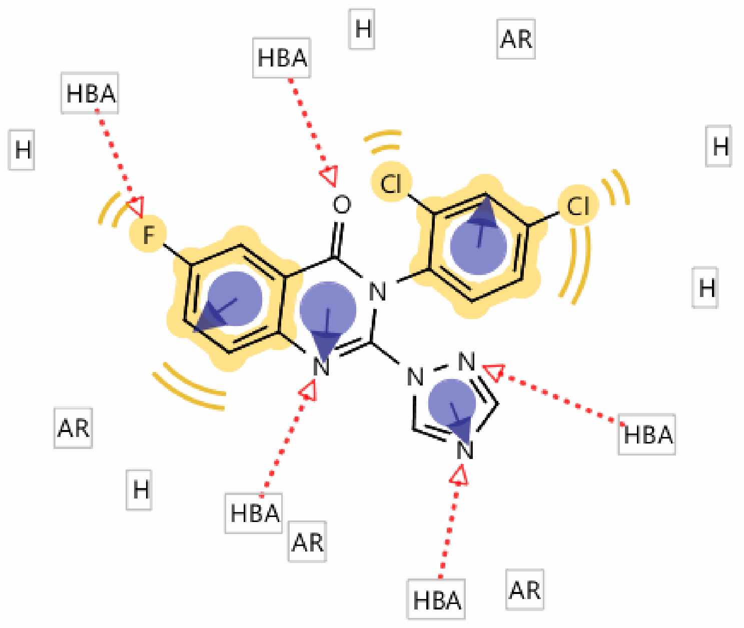
Flusilazole	85509-19-9	FQKUGOMFVDPBIZ-UHFFFAOYSA-N	InChI=1S/C16H15F2N3Si/c1-22(12-21-11-19-10-20-21,15-6-2-13(17)3-7-15)16-8-4-14(18)5-9-16/h2-11H,12H2,1H3	C[Si](C1=CC=C(C=C1)F)(CN2C=NC=N2)C3=CC=C(C=C3)F	Sterol 14α-demethylase inhibitor	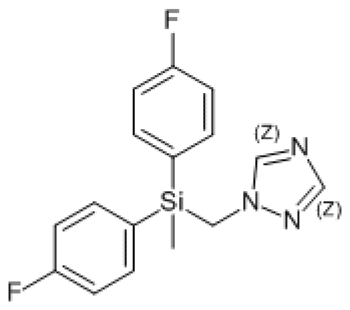	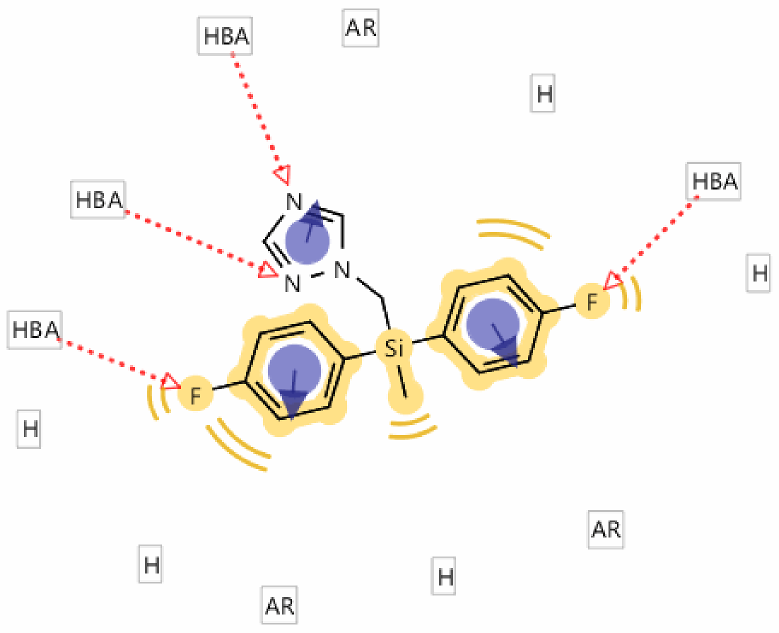
Flutriafol	76674-21-0	CSXFHZGGPUTMPM-RRHAQCGESA-N	InChI=1S/2C16H13F2N3O/c2*17-13-7-5-12(6-8-13)16(22,9-21-11-19-10-20-21)14-3-1-2-4-15(14)18/h2*1-8,10-11,22H,9H2/t2*16-/m10/s1	FC1=CC=C(C=C1)[C@](O)(CN2C=NC=N2)C3=CC=CC=C3F.FC4=CC=C(C=C4)[C@@](O)(CN5C=NC=N5)C6=CC=CC=C6F	Sterol 14α-demethylase inhibitor	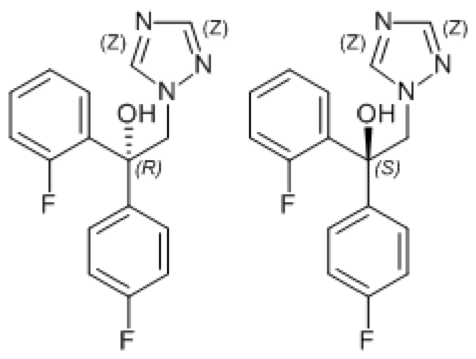	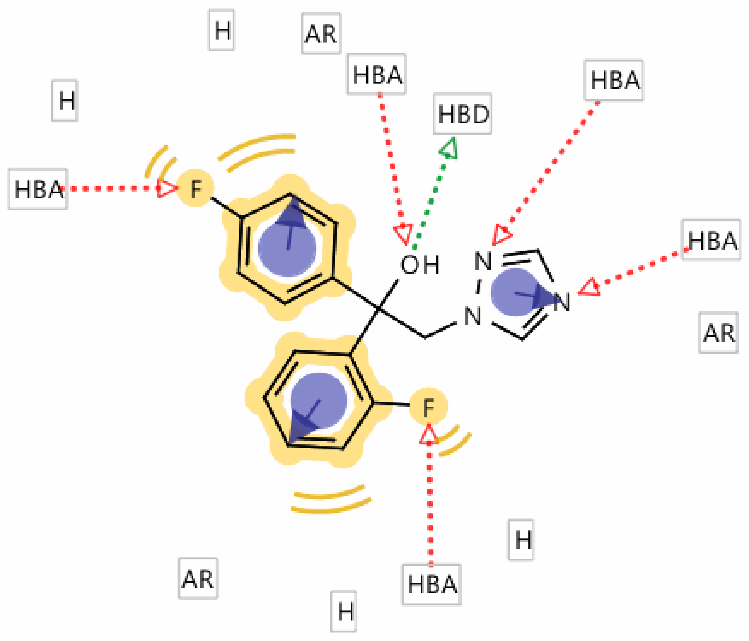

Common name	CAS Reg. No.	InChIKey	InChI	SMILES	Primary target	2D Structure	Pharmacophore[1]
Fosfluconazole	194798-83-9	GHJWNRRCRIGGIO-UHFFFAOYSA-N	InChI=1S/C13H13F2N6O4P/c14-10-1-2-11(12(15)3-10)13(25-26(22,23)24,4-20-8-16-6-18-20)5-21-9-17-7-19-21/h1-3,6-9H,4-5H2,(H2,22,23,24)	FC1=CC(F)=C(C=C1)C(OP(O)(O)=O)(CN2C=NC=N2)CN3C=NC=N3	Sterol 14α-demethylase inhibitor	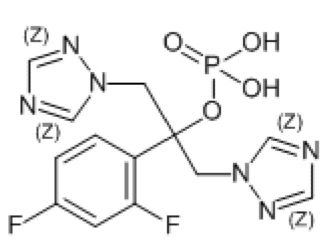	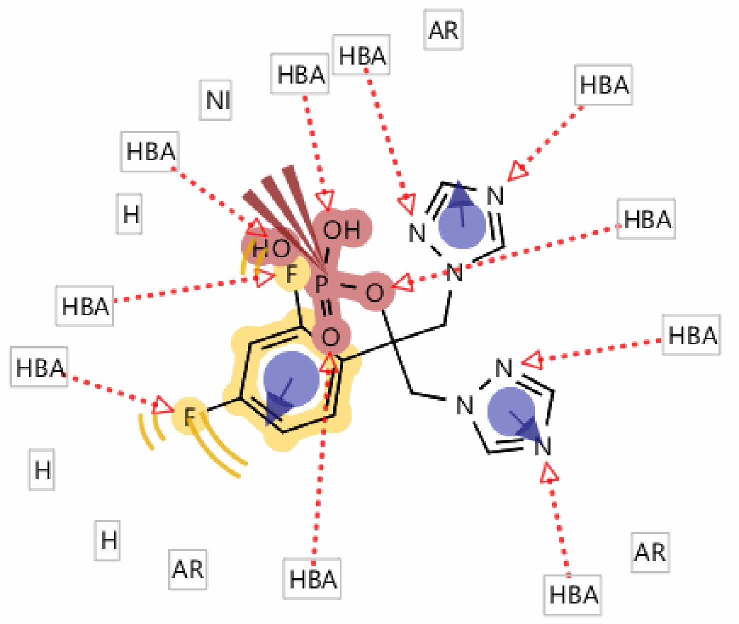
Furconazole	112839-33-5	RQHFGSMAIYYONL-BQMCQWBFSA-N	InChI=1S/4C15H14Cl2F3N3O2/c4*16-10-1-2-11(12(17)5-10)14(6-23-9-21-8-22-23)4-3-13(25-14)24-7-15(18,19)20/h4*1-2,5,8-9,13H,3-4,6-7H2/t2*13-,14+;2*13-,14-/m1010/s1	ClC1=CC(Cl)=C([C@@]2(CN3C=NC=N3)CC[C@@H](O2)OCC(F)(F)F)C=C1.ClC4=CC(Cl)=C([C@]5(CN6C=NC=N6)CC[C@H](O5)OCC(F)(F)F)C=C4.ClC7=CC(Cl)=C([C@]8(CN9C=NC=N9)CC[C@@H](O8)OCC(F)(F)F)C=C7.ClC%10=CC(Cl)=C([C@@]%11(CN%12C=NC=N%12)CC[C@H](O%11)OCC(F)(F)F)C=C%10	Sterol 14α-demethylase inhibitor	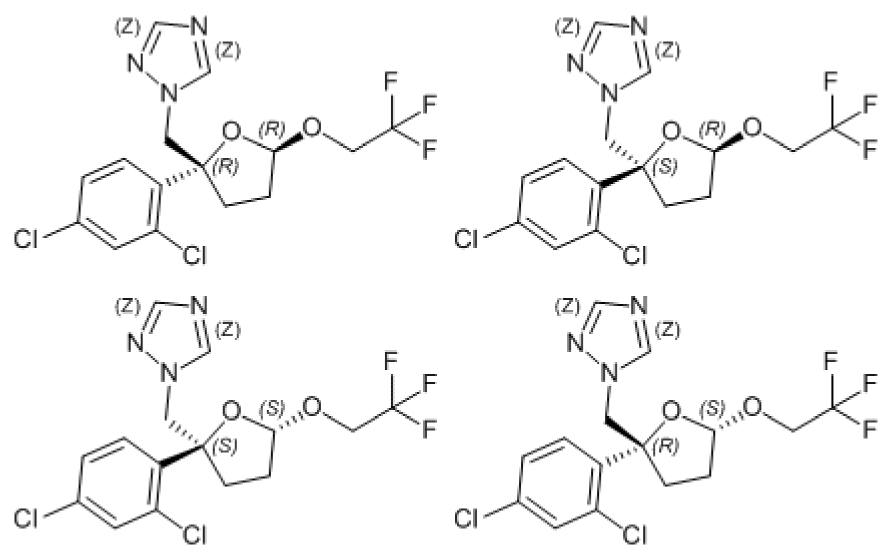	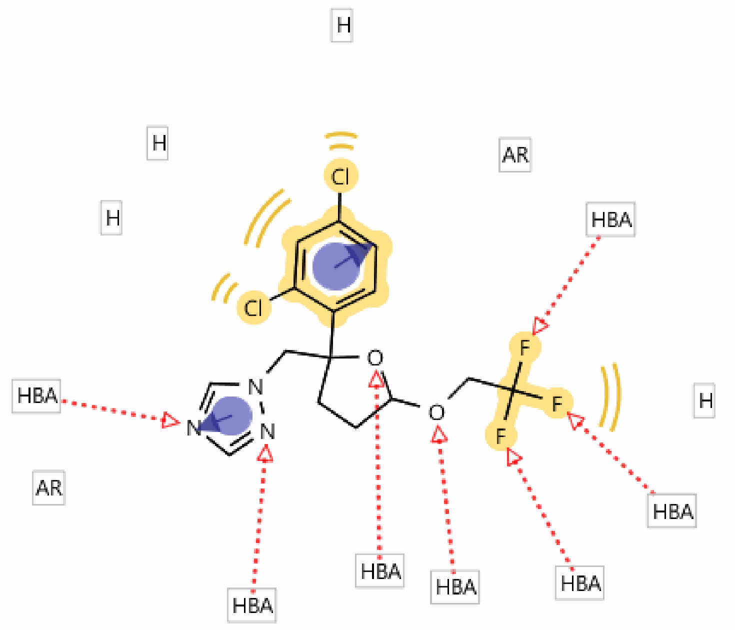
Genaconazole (SCH 39304)	120924-80-3	HBWHJEKUXXZRSF-IPJDUMPVSA-N	InChI=1S/2C13H15F2N3O3S/c2*1-9(22(2,20)21)13(19,6-18-8-16-7-17-18)11-4-3-10(14)5-12(11)15/h2*3-5,7-9,19H,6H2,1-2H3/t2*9-,13-/m10/s1	C[C@H]([C@](O)(CN1C=NC=N1)C2=C(C=C(C=C2)F)F)S(C)(=O)=O.C[C@@H]([C@@](O)(CN3C=NC=N3)C4=C(C=C(C=C4)F)F)S(C)(=O)=O	Sterol 14α-demethylase inhibitor	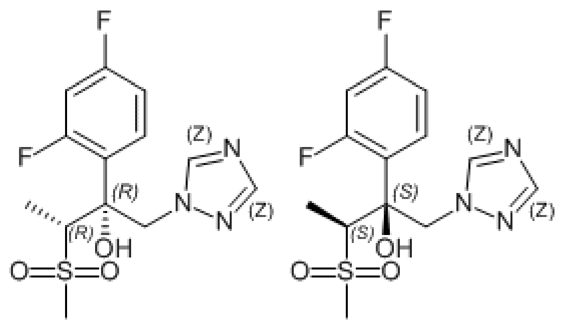	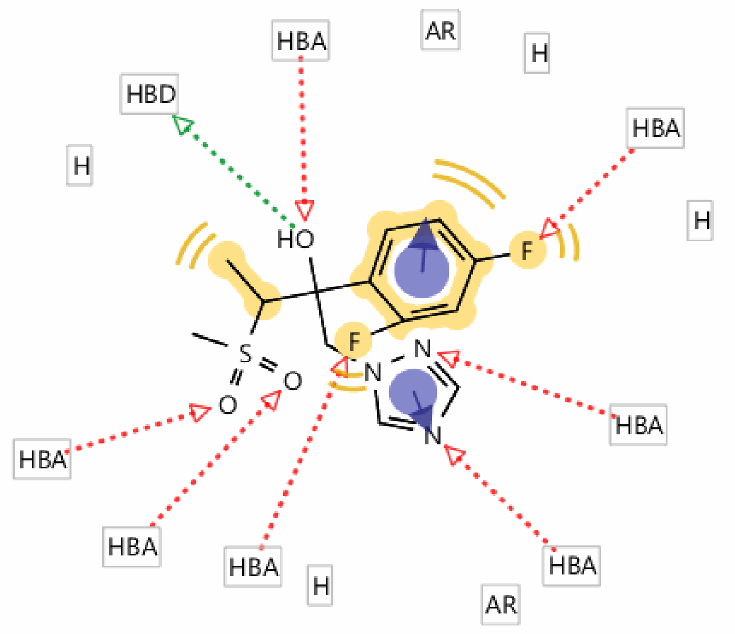
Hexaconazole	79983-71-4	XYUVNMJFMCHWHP-IKFJUQJOSA-N	InChI=1S/2C14H17Cl2N3O/c2*1-2-3-6-14(20,8-19-10-17-9-18-19)12-5-4-11(15)7-13(12)16/h2*4-5,7,9-10,20H,2-3,6,8H2,1H3/t2*14-/m10/s1	CCCC[C@@](O)(CN1C=NC=N1)C2=C(C=C(C=C2)Cl)Cl.CCCC[C@](O)(CN3C=NC=N3)C4=C(C=C(C=C4)Cl)Cl	Sterol 14α-demethylase inhibitor	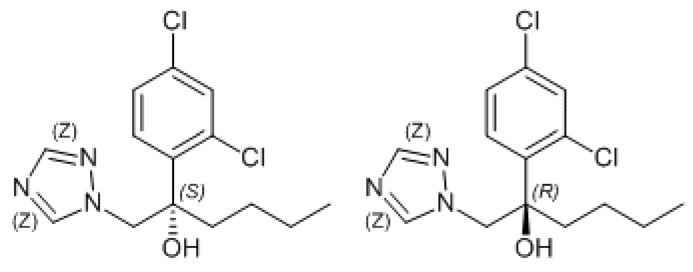	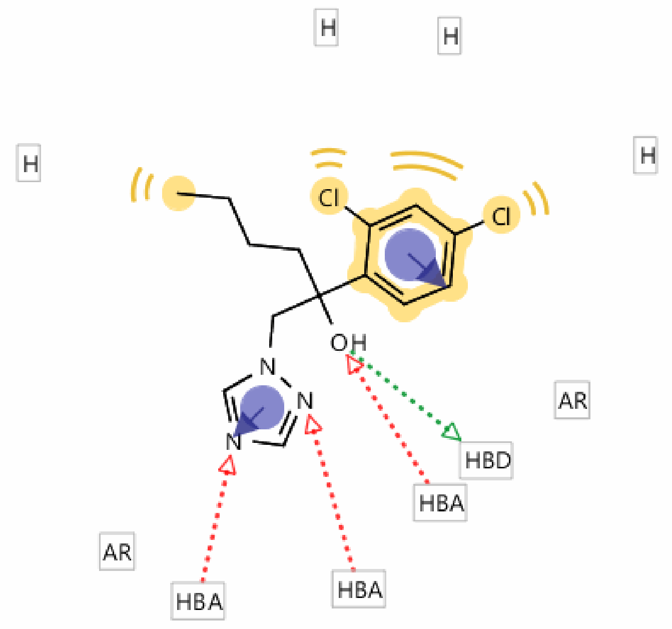
ICI 153066	76674-22-1	UGARSCJPQHNMKB-RRHAQCGESA-N	InChI=1S/2C16H12Cl2FN3O/c2*17-12-3-6-14(15(18)7-12)16(23,8-22-10-20-9-21-22)11-1-4-13(19)5-2-11/h2*1-7,9-10,23H,8H2/t2*16-/m10/s1	ClC1=CC(Cl)=C(C=C1)[C@@](O)(CN2C=NC=N2)C3=CC=C(C=C3)F.ClC4=CC(Cl)=C(C=C4)[C@](O)(CN5C=NC=N5)C6=CC=C(C=C6)F	Sterol 14α-demethylase inhibitor	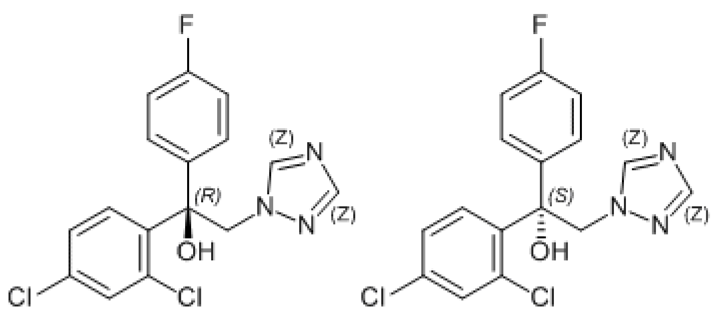	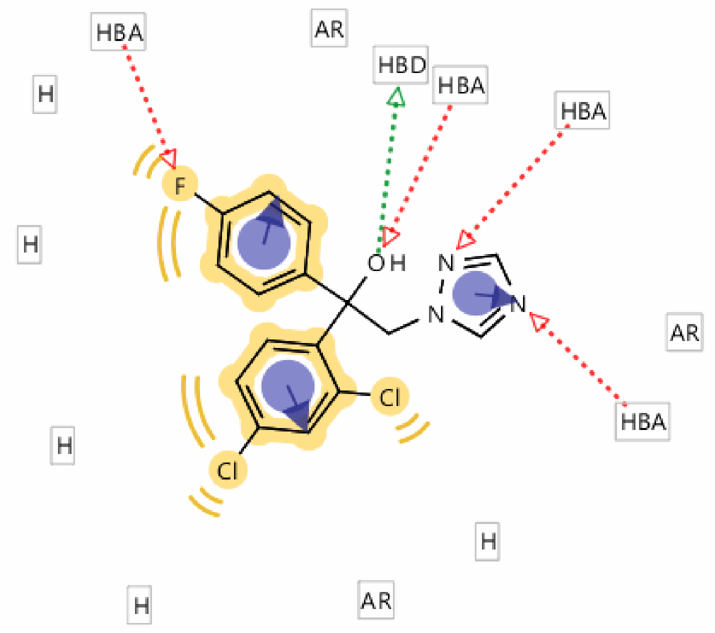
ICI 195739	103961-78-0	KSWVFWCEIOKHHC-HCLNXLRASA-N	InChI=1S/2C24H20F6N6O2/c2*25-17-4-7-19(20(26)9-17)23(37,10-35-14-31-13-33-35)11-36-15-32-21(34-36)8-3-16-1-5-18(6-2-16)38-12-24(29,30)22(27)28/h2*1-9,13-15,22,37H,10-12H2/b2*8-3-/t2*23-/m10/s1	FC1=CC(F)=C(C=C1)[C@@](O)(CN2C=NC=N2)CN3C=NC(/C=C\C4=CC=C(C=C4)OCC(F)(C(F)F)F)=N3.FC5=CC(F)=C(C=C5)[C@](O)(CN6C=NC=N6)CN7C=NC(/C=C\C8=CC=C(C=C8)OCC(F)(C(F)F)F)=N7	Sterol 14α-demethylase inhibitor	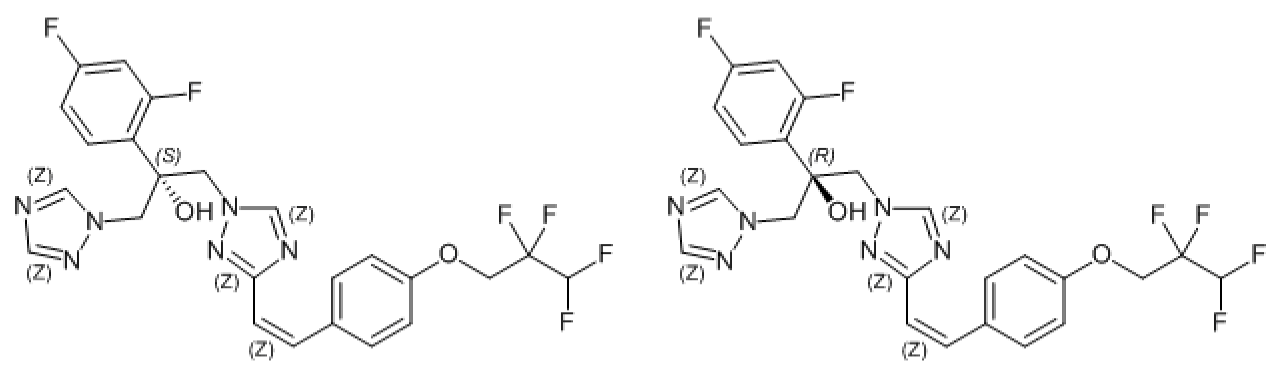	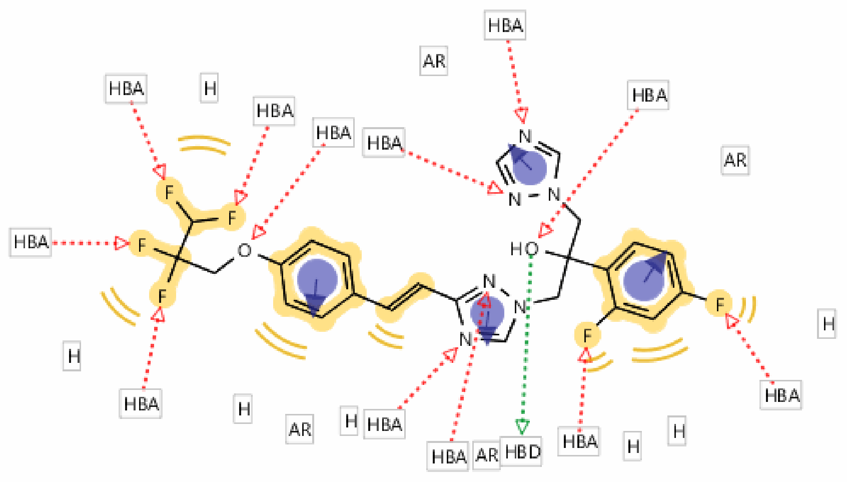

Common name	CAS Reg. No.	InChIKey	InChI	SMILES	Primary target	2D Structure	Pharmacophore[1]
Imibenconazole	86598-92-7	AGKSTYPVMZODRV-UHFFFAOYSA-N	InChI=1S/C17H13Cl3N4S/c18-13-3-1-12(2-4-13)9-25-17(8-24-11-21-10-22-24)23-16-6-5-14(19)7-15(16)20/h1-7,10-11H,8-9H2	ClC1=C(/N=C(CN2N=CN=C2)/SCC3=CC=C(Cl)C=C3)C=CC(Cl)=C1	Sterol 14α-demethylase inhibitor	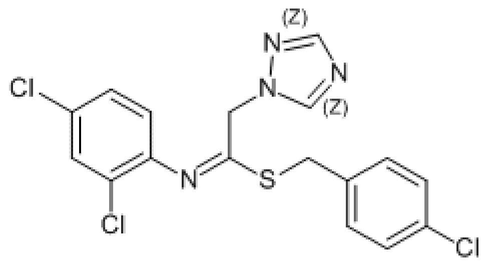	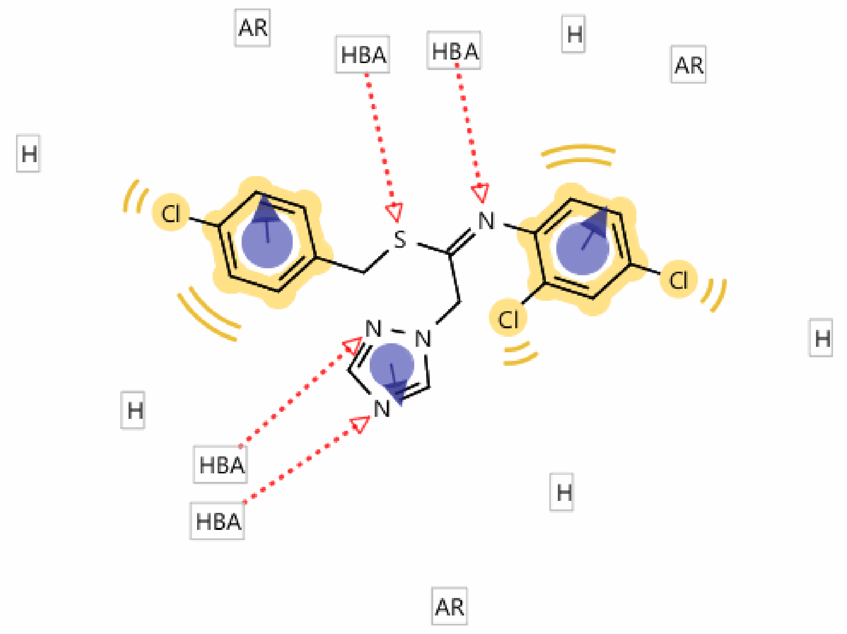
Ipconazole	125225-28-7	TUZZSXYFOCWDAS-BIEISDBSSA-N	InChI=1S/4C18H24ClN3O/c4*1-13(2)17-8-5-15(9-14-3-6-16(19)7-4-14)18(17,23)10-22-12-20-11-21-22/h4*3-4,6-7,11-13,15,17,23H,5,8-10H2,1-2H3/t2*15-,17+,18+;2*15-,17-,18+/m1010/s1	CC(C)[C@@H]1CC[C@H](CC2=CC=C(C=C2)Cl)[C@]1(CN3C=NC=N3)O.CC(C)[C@H]4CC[C@@H](CC5=CC=C(C=C5)Cl)[C@@]4(CN6C=NC=N6)O.CC(C)[C@H]7CC[C@H](CC8=CC=C(C=C8)Cl)[C@]7(CN9C=NC=N9)O.CC(C)[C@@H]%10CC[C@@H](CC%11=CC=C(C=C%11)Cl)[C@@]%10(CN%12C=NC=N%12)O	Sterol 14α-demethylase inhibitor	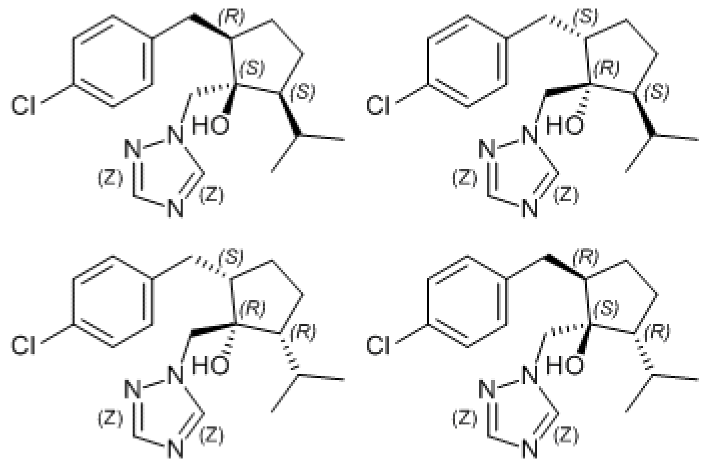	
Ipfentrifluconazole	1417782-08-1	QCLZMYAIZNMPAH-OYPHMNEHSA-N	InChI=1S/2C20H19ClF3N3O2/c2*1-13(2)19(28,10-27-12-25-11-26-27)17-8-7-16(9-18(17)20(22,23)24)29-15-5-3-14(21)4-6-15/h2*3-9,11-13,28H,10H2,1-2H3/t2*19-/m10/s1	CC([C@](CN1C=NC=N1)(C2=C(C(F)(F)F)C=C(OC3=CC=C(Cl)C=C3)C=C2)O)C.CC([C@@](CN4C=NC=N4)(C5=C(C(F)(F)F)C=C(OC6=CC=C(Cl)C=C6)C=C5)O)C	Sterol 14α-demethylase inhibitor	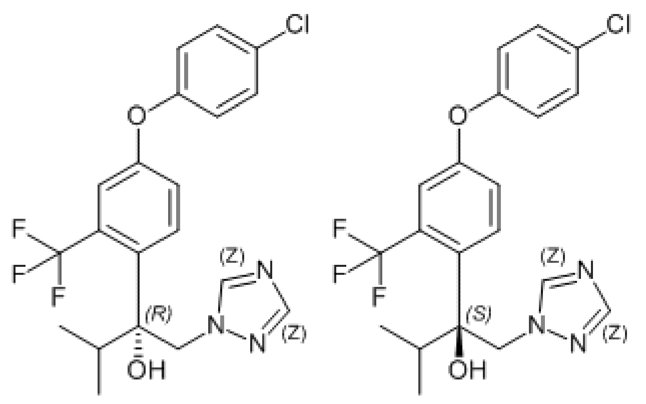	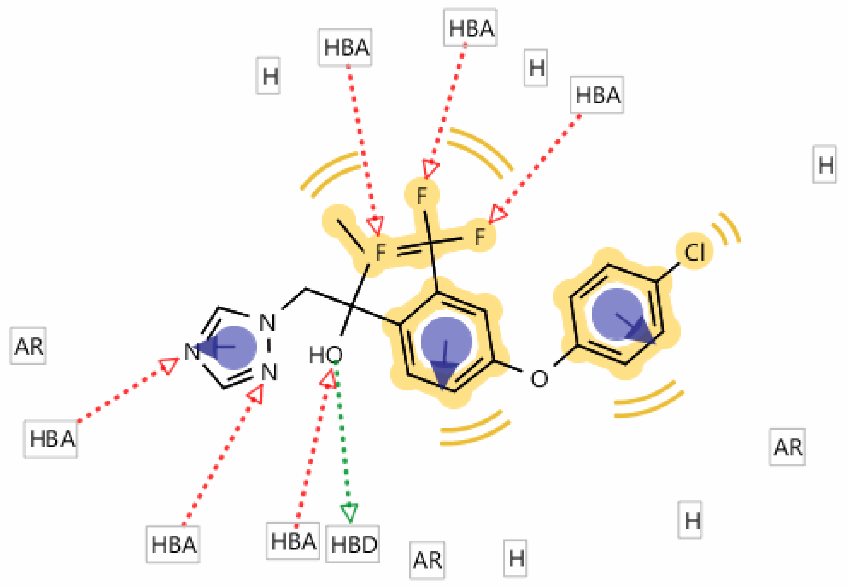
Isavuconazole	241479-67-4	DDFOUSQFMYRUQK-RCDICMHDSA-N	InChI=1S/C22H17F2N5OS/c1-14(21-28-20(10-31-21)16-4-2-15(9-25)3-5-16)22(30,11-29-13-26-12-27-29)18-8-17(23)6-7-19(18)24/h2-8,10,12-14,30H,11H2,1H3/t14-,22+/m0/s1	C[C@H]([C@](CN1C=NC=N1)(C2=C(F)C=CC(F)=C2)O)C3=NC(C4=CC=C(C#N)C=C4)=CS3	Sterol 14α-demethylase inhibitor	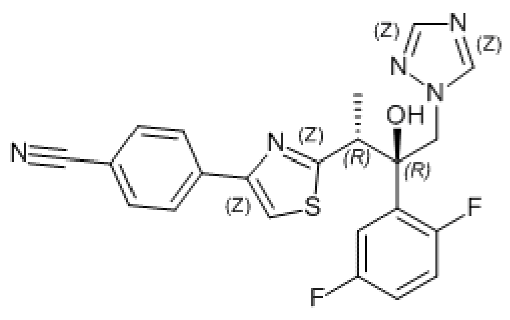	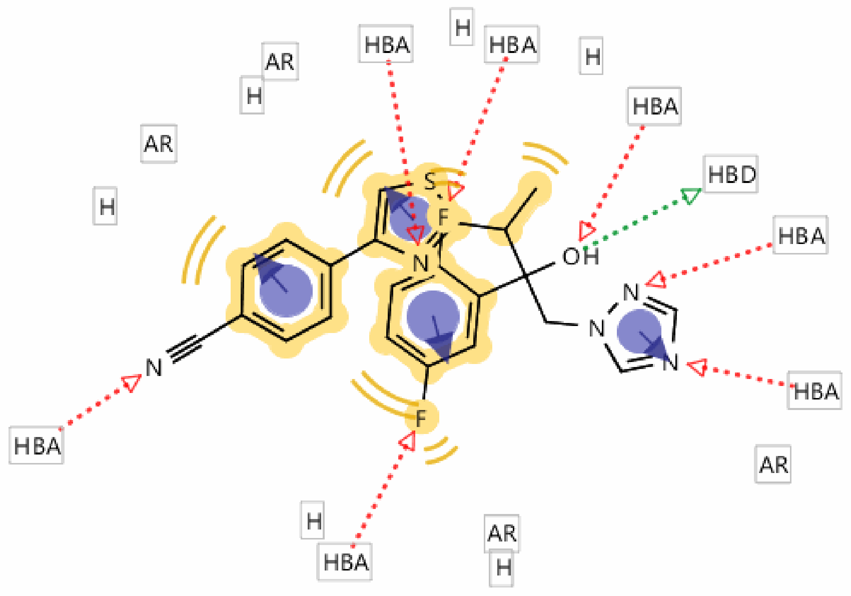
Itraconazole	84625-61-6	LSTCZAXKMOLFGK-PPUJANMKSA-N	InChI=1S/4C35H38Cl2N8O4/c4*1-3-25(2)45-34(46)44(24-40-45)29-7-5-27(6-8-29)41-14-16-42(17-15-41)28-9-11-30(12-10-28)47-19-31-20-48-35(49-31,21-43-23-38-22-39-43)32-13-4-26(36)18-33(32)37/h4*4-13,18,22-25,31H,3,14-17,19-21H2,1-2H3/t2*25-,31+,35+;2*25-,31-,35-/m1010/s1	CC[C@@H](C)N(C1=O)N=CN1C2=CC=C(C=C2)N3CCN(CC3)C4=CC=C(C=C4)OC[C@H]5CO[C@@](O5)(C6=C(C=C(C=C6)Cl)Cl)CN7N=CN=C7.CC[C@H](C)N(C8=O)N=CN8C9=CC=C(C=C9)N%10CCN(CC%10)C%11=CC=C(C=C%11)OC[C@H]%12CO[C@@](O%12)(C%13=C(C=C(C=C%13)Cl)Cl)CN%14N=CN=C%14.CC[C@@H](C)N(C%15=O)N=CN%15C%16=CC=C(C=C%16)N%17CCN(CC%17)C%18=CC=C(C=C%18)OC[C@@H]%19CO[C@](O%19)(C%20=C(C=C(C=C%20)Cl)Cl)CN%21N=CN=C%21.CC[C@H](C)N(C%22=O)N=CN%22C%23=CC=C(C=C%23)N%24CCN(CC%24)C%25=CC=C(C=C%25)OC[C@@H]%26CO[C@](O%26)(C%27=C(C=C(C=C%27)Cl)Cl)CN%28N=CN=C%28	Sterol 14α-demethylase inhibitor	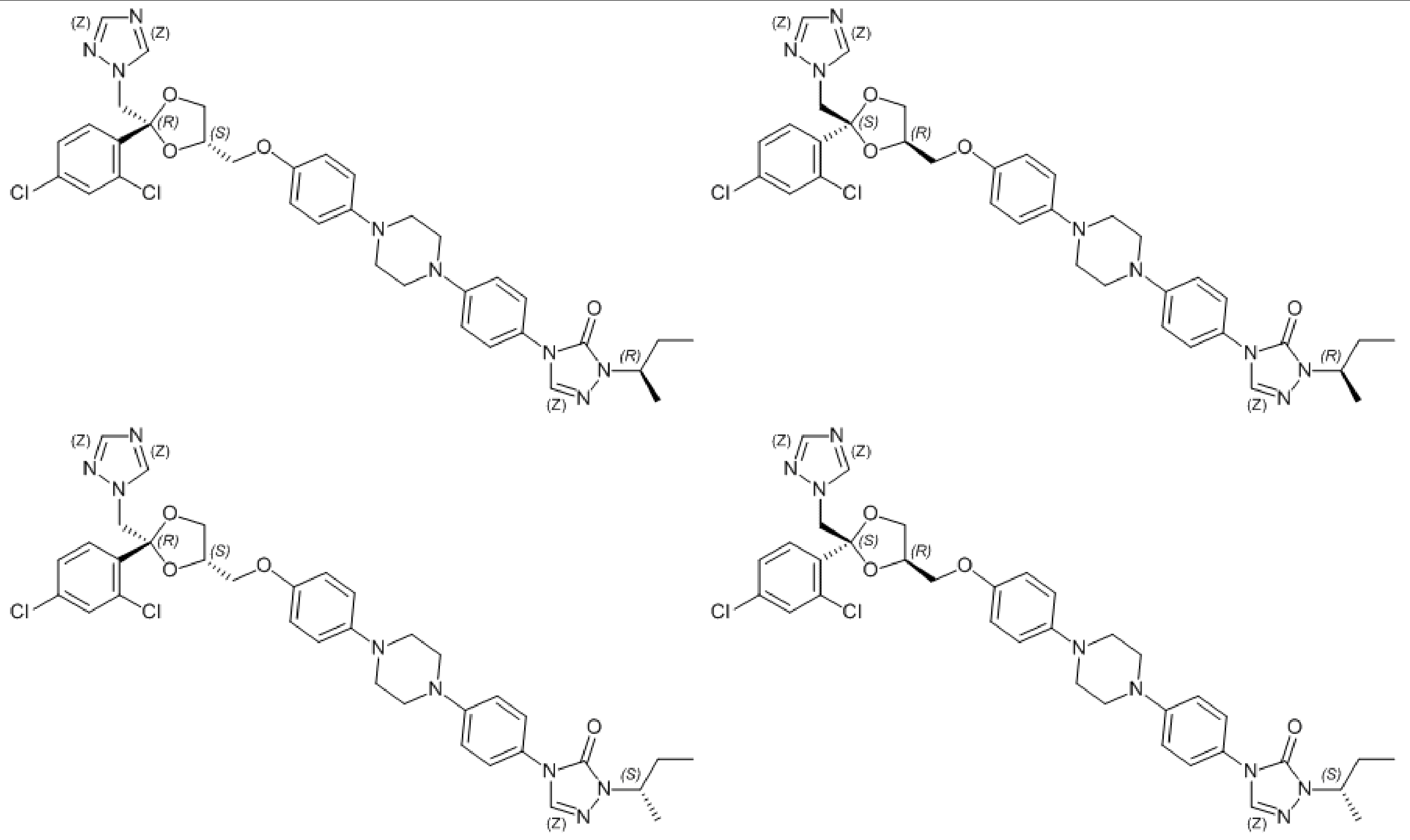	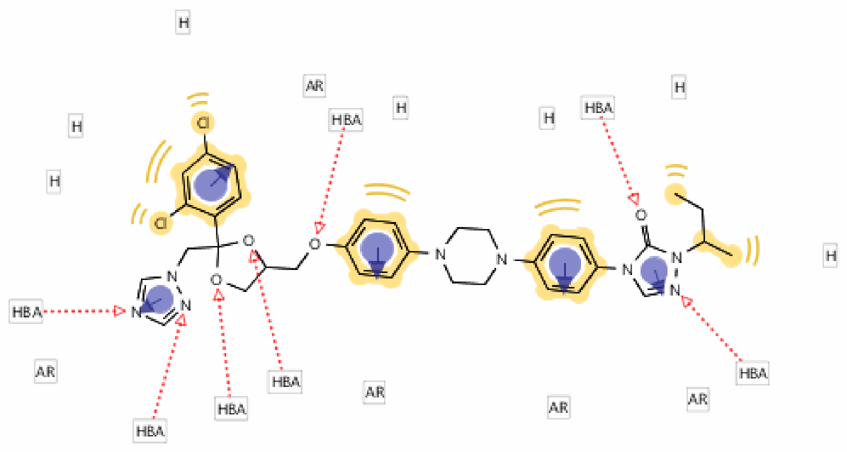
LAB 158241F	95059-87-3	CRVPQJOCTGXDRB-UHFFFAOYSA-N	InChI=1S/C14H15Cl2N3O/c1-14(2,3)7-13(19-9-17-8-18-19)20-12-5-4-10(15)6-11(12)16/h4-9H,1-3H3	CC(/C=C(N1C=NC=N1)/OC2=C(C=C(C=C2)Cl)Cl)(C)C	Sterol 14α-demethylase inhibitor	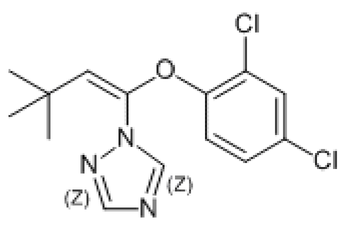	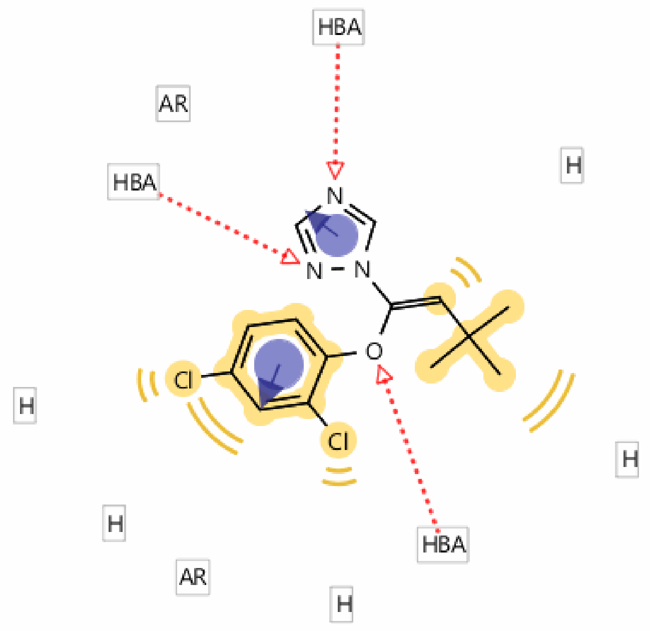

Common name	CAS Reg. No.	InChIKey	InChI	SMILES	Primary target	2D Structure	Pharmacophore[1]
LAB 170250F	88630-25-5	FQMDEELSZJOSAQ-TYDMEHBZSA-N	InChI=1S/2C17H14ClN3O/c2*18-15-8-6-14(7-9-15)17(10-21-12-19-11-20-21)16(22-17)13-4-2-1-3-5-13/h2*1-9,11-12,16H,10H2/t2*16-,17-/m10/s1	ClC1=CC=C(C=C1)[C@]2(CN3C=NC=N3)[C@@H](C4=CC=CC=C4)O2.ClC5=CC=C(C=C5)[C@@]6(CN7C=NC=N7)[C@H](C8=CC=CC=C8)O6	Sterol 14α-demethylase inhibitor	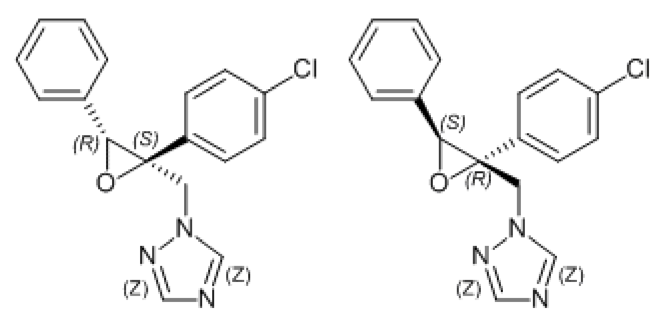	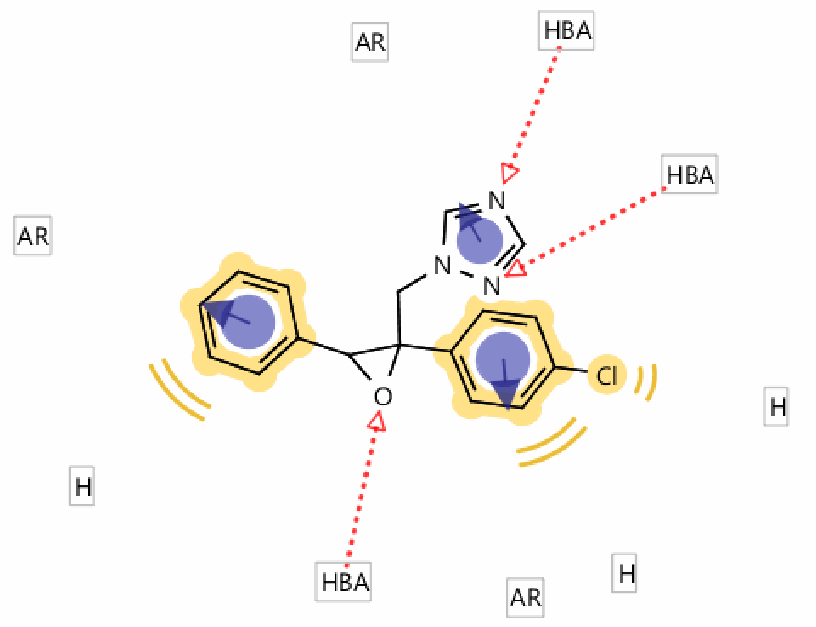
Mefentrifluconazole	1417782-03-6	JVJPWUXCSKFHQH-QAOGLABXSA-N	InChI=1S/2C18H15ClF3N3O2/c2*1-17(26,9-25-11-23-10-24-25)15-7-6-14(8-16(15)18(20,21)22)27-13-4-2-12(19)3-5-13/h2*2-8,10-11,26H,9H2,1H3/t2*17-/m10/s1	C[C@@](O)(CN1C=NC=N1)C2=C(C=C(C=C2)OC3=CC=C(C=C3)Cl)C(F)(F)F.C[C@](O)(CN4C=NC=N4)C5=C(C=C(C=C5)OC6=CC=C(C=C6)Cl)C(F)(F)F	Sterol 14α-demethylase inhibitor	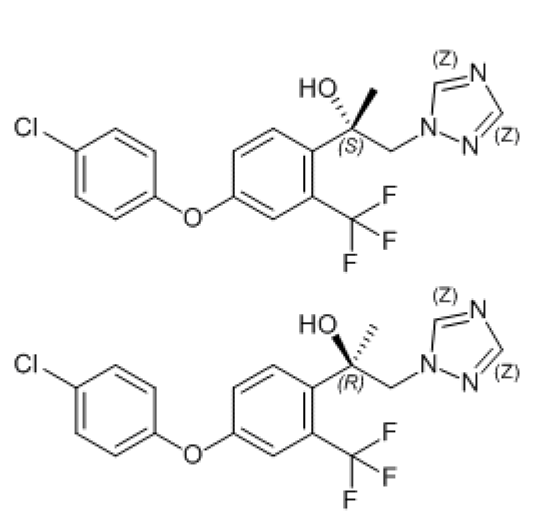	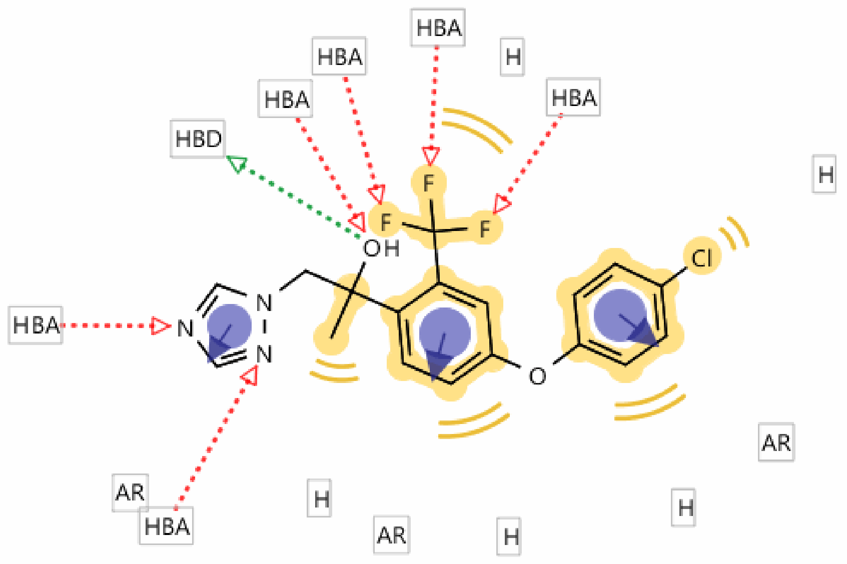
Metconazole	125116-23-6	MYDROAMIYTWIDB-SNBRXXPMSA-N	InChI=1S/4C17H22ClN3O/c4*1-16(2)8-7-14(9-13-3-5-15(18)6-4-13)17(16,22)10-21-12-19-11-20-21/h4*3-6,11-12,14,22H,7-10H2,1-2H3/t2*14-,17+;2*14-,17-/m1010/s1	CC1(C)CC[C@H](CC2=CC=C(C=C2)Cl)[C@]1(CN3C=NC=N3)O.CC4(C)CC[C@@H](CC5=CC=C(C=C5)Cl)[C@@]4(CN6C=NC=N6)O.CC7(C)CC[C@H](CC8=CC=C(C=C8)Cl)[C@@]7(CN9C=NC=N9)O.CC%10(C)CC[C@@H](CC%11=CC=C(C=C%11)Cl)[C@]%10(CN%12C=NC=N%12)O	Sterol 14α-demethylase inhibitor	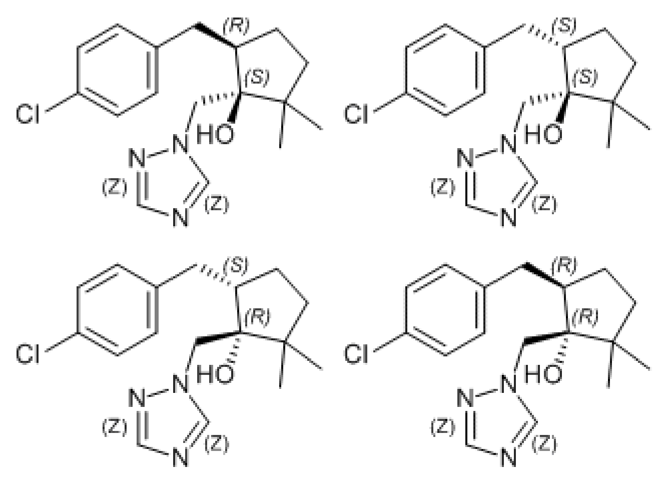	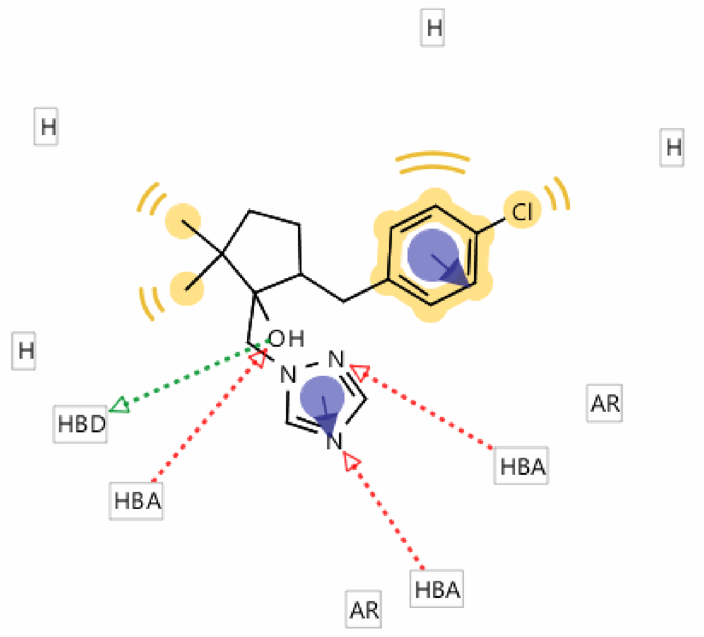
Myclobutanil	88671-89-0	ZRLAPEUZHQWKBJ-ZWZQDMJTSA-N	InChI=1S/2C15H17ClN4/c2*1-2-3-8-15(9-17,10-20-12-18-11-19-20)13-4-6-14(16)7-5-13/h2*4-7,11-12H,2-3,8,10H2,1H3/t2*15-/m10/s1	CCCC[C@@](C#N)(CN1C=NC=N1)C2=CC=C(Cl)C=C2.CCCC[C@](C#N)(CN3C=NC=N3)C4=CC=C(Cl)C=C4	Sterol 14α-demethylase inhibitor	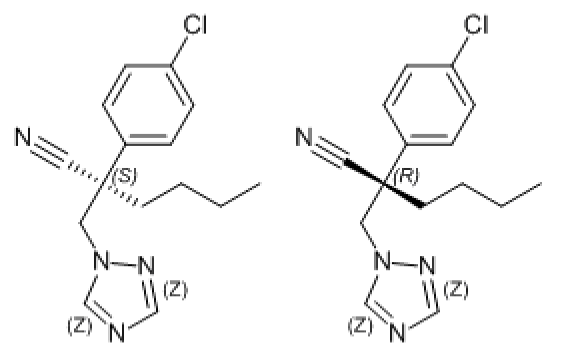	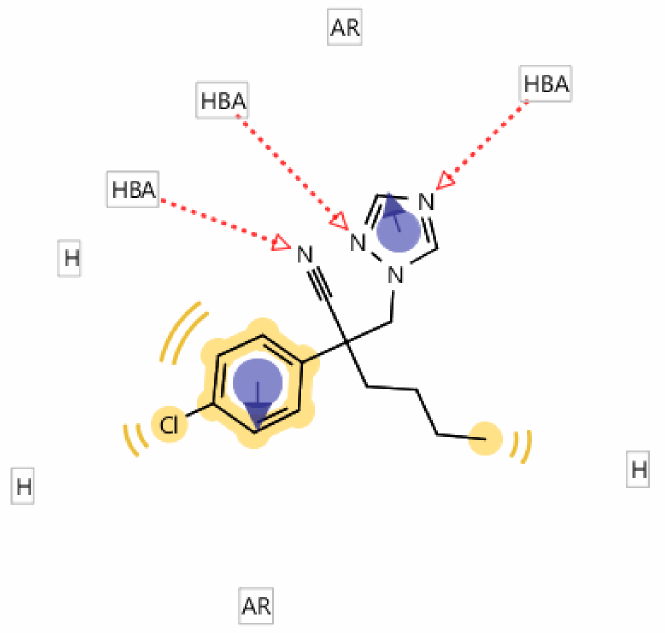
Penconazole	66246-88-6	ZVCSHSPUPZBCBS-FTYBWHBYSA-N	InChI=1S/2C13H15Cl2N3/c2*1-2-3-10(7-18-9-16-8-17-18)12-5-4-11(14)6-13(12)15/h2*4-6,8-10H,2-3,7H2,1H3/t2*10-/m10/s1	CCC[C@]([H])(CN1C=NC=N1)C2=C(C=C(C=C2)Cl)Cl.CCC[C@@]([H])(CN3C=NC=N3)C4=C(C=C(C=C4)Cl)Cl	Sterol 14α-demethylase inhibitor	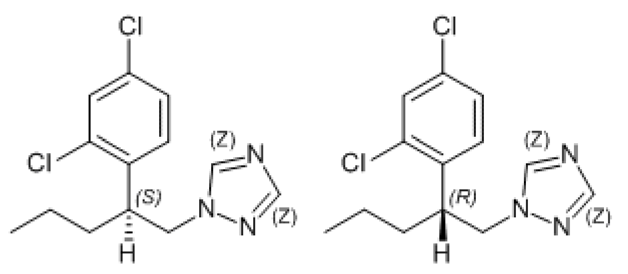	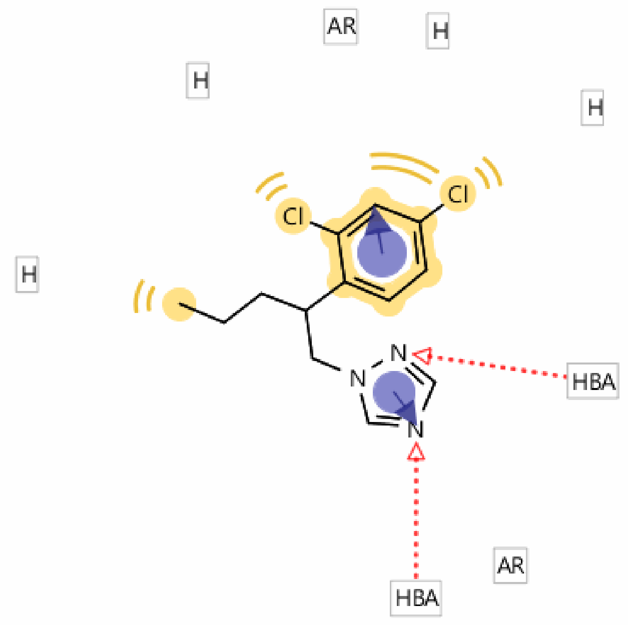

Common name	CAS Reg. No.	InChIKey	InChI	SMILES	Primary target	2D Structure	Pharmacophore[1]
Posaconazole	171228-49-2	RAGOYPUPXAKGKH-XAKZXMRKSA-N	InChI=1S/C37H42F2N8O4/c1-3-35(26(2)48)47-36(49)46(25-42-47)31-7-5-29(6-8-31)43-14-16-44(17-15-43)30-9-11-32(12-10-30)50-20-27-19-37(51-21-27,22-45-24-40-23-41-45)33-13-4-28(38)18-34(33)39/h4-13,18,23-27,35,48H,3,14-17,19-22H2,1-2H3/t26-,27+,35-,37-/m0/s1	CC[C@@]([C@@H](O)C)(N(N=CN1C2=CC=C(N3CCN(C4=CC=C(OC[C@@H]5CO[C@](CN6C=NC=N6)(C5)C7=C(F)C=C(F)C=C7)C=C4)CC3)C=C2)C1=O)[H]	Sterol 14α-demethylase inhibitor	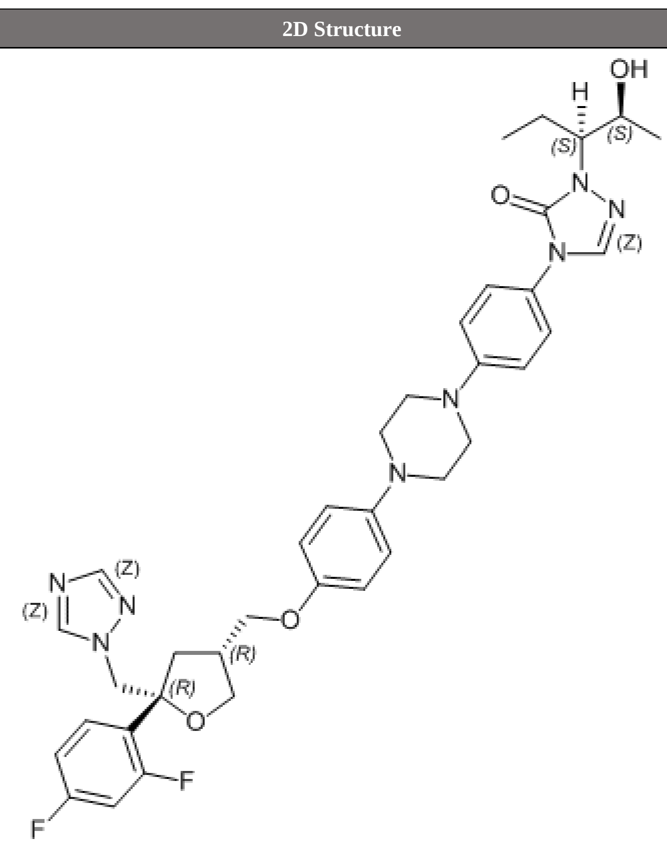	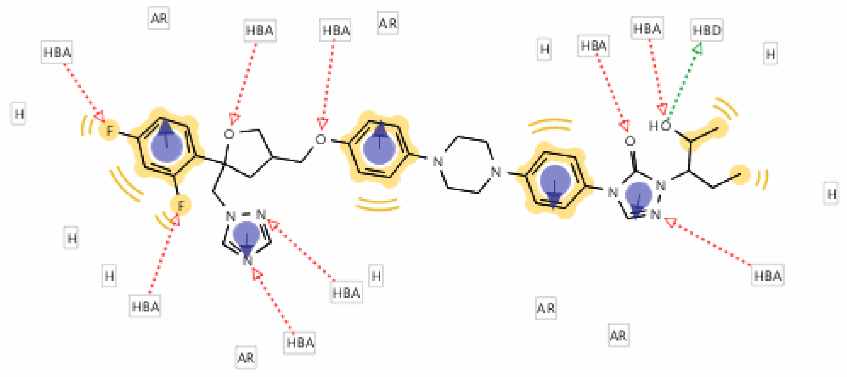
PP 969	69141-50-0	XMTNXPUOWZZIPL-RNDCMBHTSA-N	InChI=1S/4C14H25N3O2/c4*1-13(2,3)11(18)7-10(12(19)14(4,5)6)17-9-15-8-16-17/h4*8-10,12,19H,7H2,1-6H3/t2*10-,12+;2*10-,12-/m1010/s1	CC(C)(C)C(C[C@@]([C@@H](O)C(C)(C)C)(N1C=NC=N1)[H])=O.CC(C)(C)C(C[C@@]([C@H](O)C(C)(C)C)(N2C=NC=N2)[H])=O.CC(C)(C)C(C[C@]([C@H](O)C(C)(C)C)(N3C=NC=N3)[H])=O.CC(C)(C)C(C[C@]([C@@H](O)C(C)(C)C)(N4C=NC=N4)[H])=O	Sterol 14α-demethylase inhibitor	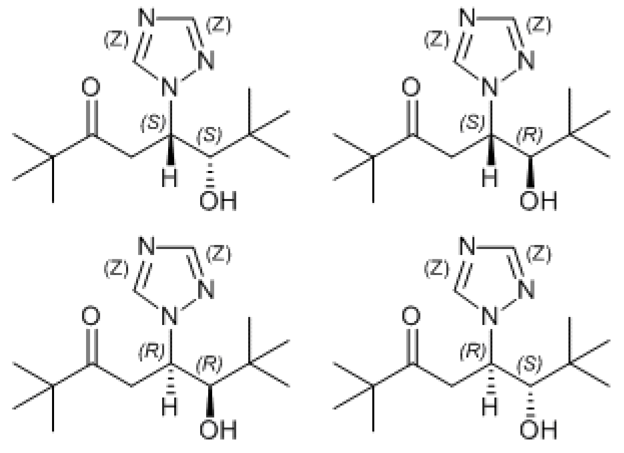	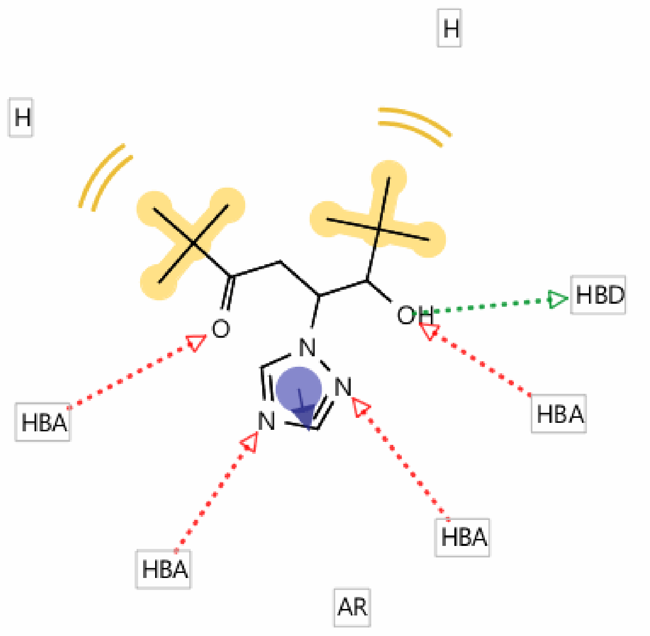
Pramiconazole	219923-85-0	AEKNYBWUEYNWMJ-QWOOXDRHSA-N	InChI=1S/C35H39F2N7O4/c1-25(2)43-17-18-44(34(43)45)29-6-4-27(5-7-29)40-13-15-41(16-14-40)28-8-10-30(11-9-28)46-20-33-47-22-35(48-33,21-42-24-38-23-39-42)31-12-3-26(36)19-32(31)37/h3-12,19,23-25,33H,13-18,20-22H2,1-2H3/t33-,35+/m0/s1	CC(C)N(CCN1C2=CC=C(C=C2)N3CCN(CC3)C4=CC=C(C=C4)OC[C@@H]5O[C@](C6=C(C=C(C=C6)F)F)(CN7C=NC=N7)CO5)C1=O	Sterol 14α-demethylase inhibitor	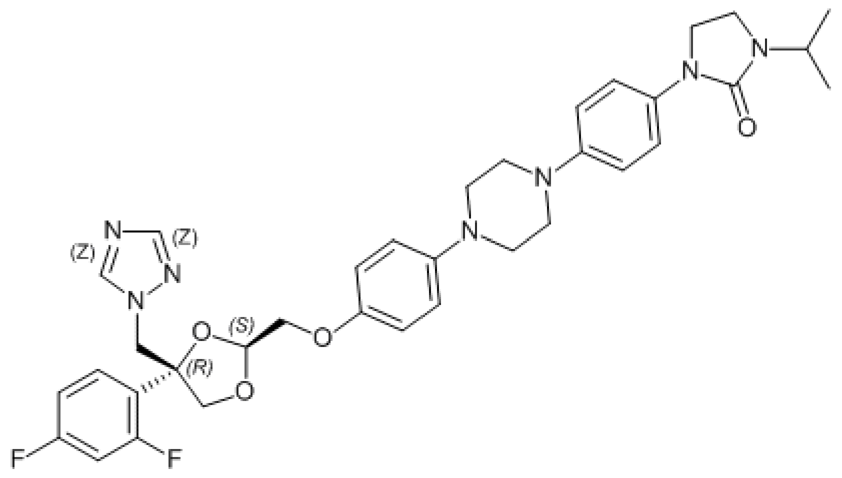	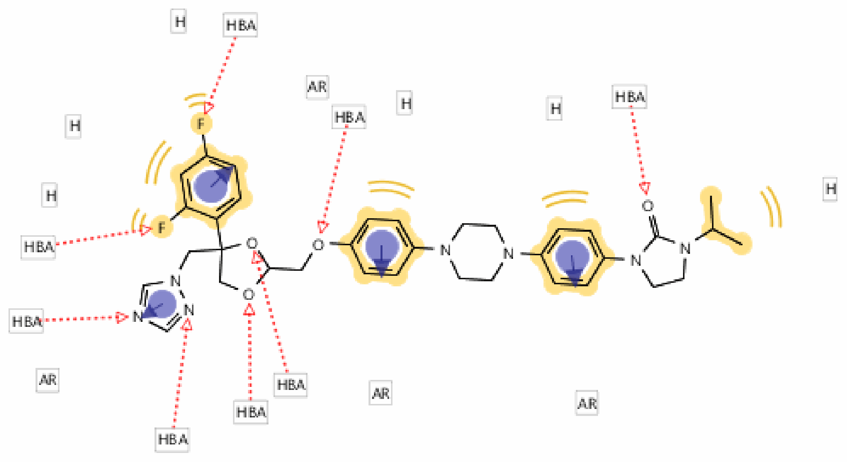
Propiconazole	60207-90-1	DNROXMGWOGXFNI-XWYXKSRUSA-N	InChI=1S/4C15H17Cl2N3O2/c4*1-2-3-12-7-21-15(22-12,8-20-10-18-9-19-20)13-5-4-11(16)6-14(13)17/h4*4-6,9-10,12H,2-3,7-8H2,1H3/t2*12-,15+;2*12-,15-/m1010/s1	CCC[C@H]1O[C@](C2=C(C=C(C=C2)Cl)Cl)(CN3C=NC=N3)OC1.CCC[C@@H]4O[C@@](C5=C(C=C(C=C5)Cl)Cl)(CN6C=NC=N6)OC4.CCC[C@H]7O[C@@](C8=C(C=C(C=C8)Cl)Cl)(CN9C=NC=N9)OC7.CCC[C@@H]%10O[C@](C%11=C(C=C(C=C%11)Cl)Cl)(CN%12C=NC=N%12)OC%10	Sterol 14α-demethylase inhibitor	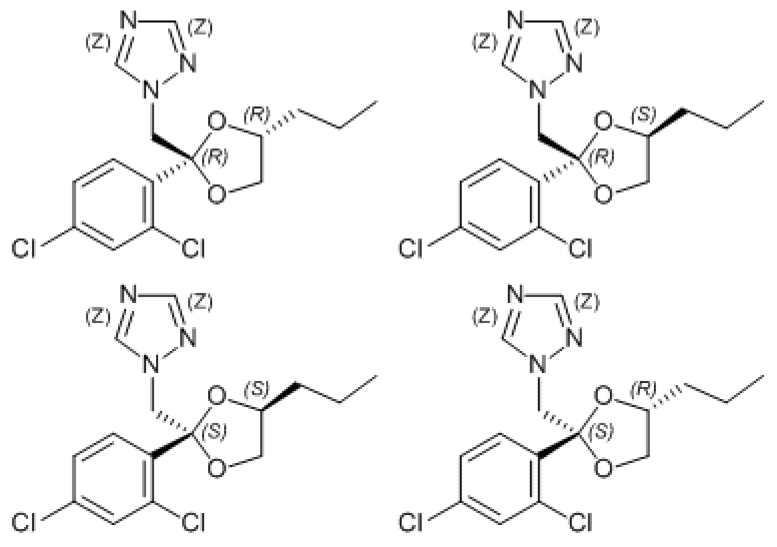	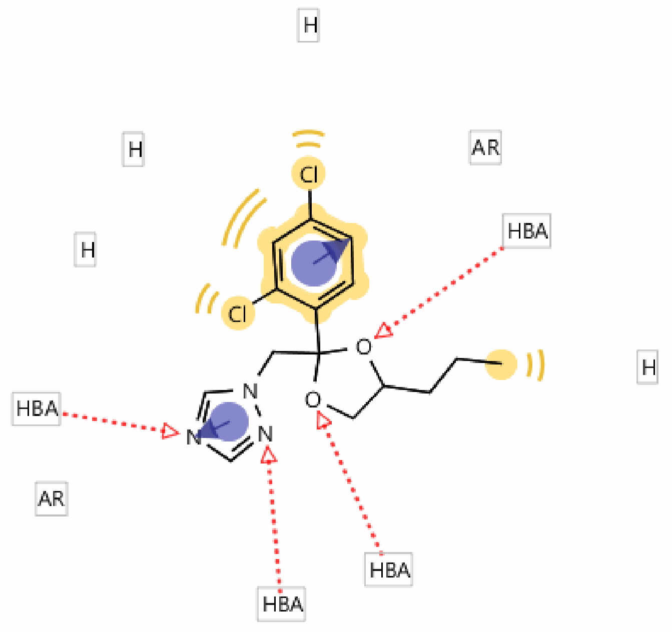
Quinconazole	103970-75-8	OVFHHJZHXHZIHT-UHFFFAOYSA-N	InChI=1S/C16H9Cl2N5O/c17-10-5-6-14(12(18)7-10)23-15(24)11-3-1-2-4-13(11)21-16(23)22-9-19-8-20-22/h1-9H	ClC1=CC(Cl)=C(C=C1)N2C(C3=CC=CC=C3N=C2N4C=NC=N4)=O	Sterol 14α-demethylase inhibitor	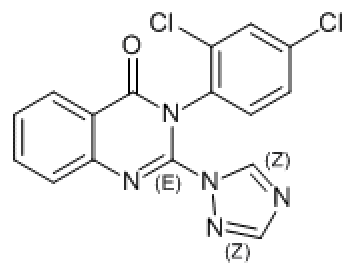	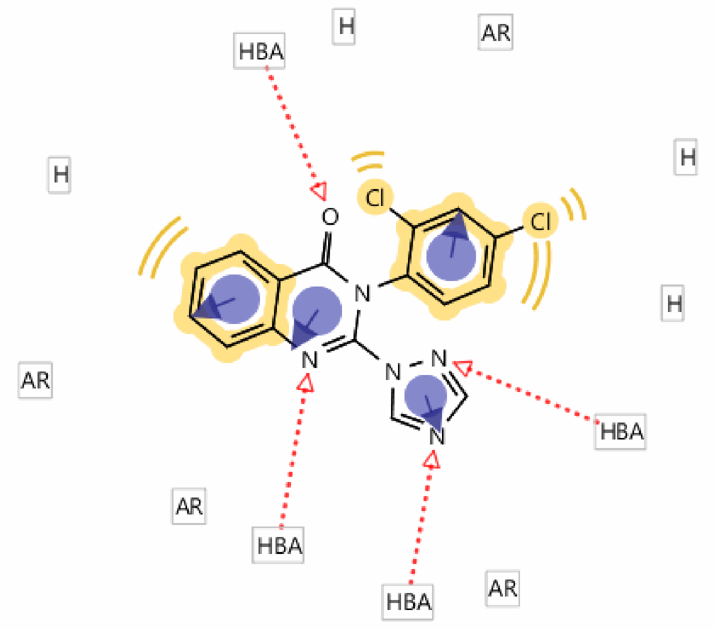

Common name	CAS Reg. No.	InChIKey	InChI	SMILES	Primary target	2D Structure	Pharmacophore[1]
Ravuconazole	182760-06-1	OPAHEYNNJWPQPX-RCDICMHDSA-N	InChI=1S/C22H17F2N5OS/c1-14(21-28-20(10-31-21)16-4-2-15(9-25)3-5-16)22(30,11-29-13-26-12-27-29)18-7-6-17(23)8-19(18)24/h2-8,10,12-14,30H,11H2,1H3/t14-,22+/m0/s1	C[C@@H](C1=NC(C2=CC=C(C(=C2)C#N)=CS1)[C@](O)(CN3C=NC=N3)C4=C(C=C(C=C4)F)F	Sterol 14α-demethylase inhibitor	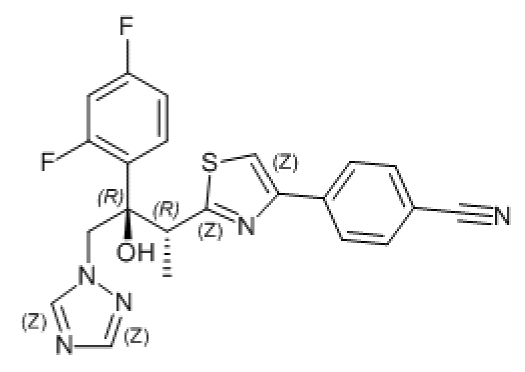	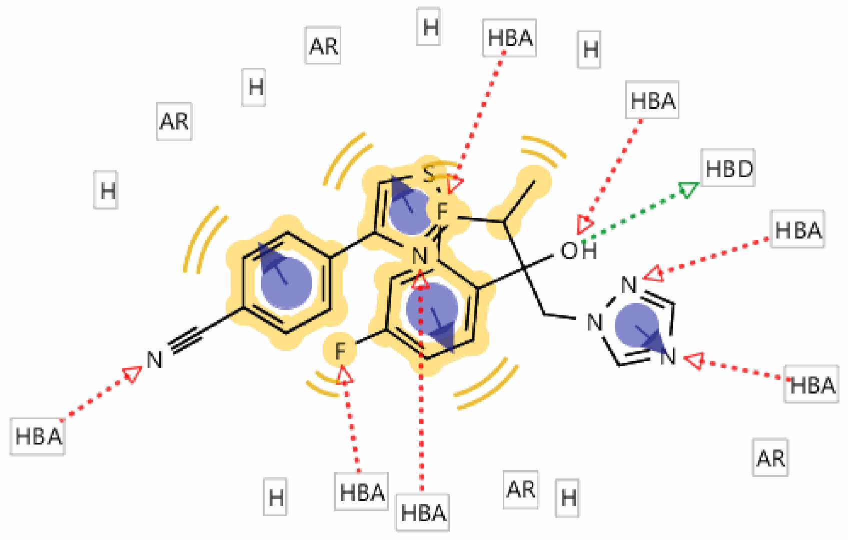
Saperconazole	110588-57-3	ZEODQGNTFDNXMH-PPUJANMKSA-N	InChI=1S/4C35H38F2N8O4/c4*1-3-25(2)45-34(46)44(24-40-45)29-7-5-27(6-8-29)41-14-16-42(17-15-41)28-9-11-30(12-10-28)47-19-31-20-48-35(49-31,21-43-23-38-22-39-43)32-13-4-26(36)18-33(32)37/h4*4-13,18,22-25,31H,3,14-17,19-21H2,1-2H3/t2*25-,31+,35+;2*25-,31-,35-/m1010/s1	CC[C@@H](C)N(C1=O)N=CN1C2=CC=C(C=C2)N3CCN(CC3)C4=CC=C(C=C4)OC[C@@H]5CO[C@@](CN6N=CN=C6)(C7=C(C=C(C=C7)F)F)O5.CC[C@H](C)N(C8=O)N=CN8C9=CC=C(C=C9)N%10CCN(CC%10)C%11=CC=C(C=C%11)OC[C@@H]%12CO[C@@](CN%13N=CN=C%13)(C%14=C(C=C(C=C%14)F)F)O%12.CC[C@H](C)N(C%15=O)N=CN%15C%16=CC=C(C=C%16)N%17CCN(CC%17)C%18=CC=C(C=C%18)OC[C@H]%19CO[C@](O%19)(CN%20N=CN=C%20)C%21=C(C=C(C=C%21)F)F.CC[C@@H](C)N(C%22=O)N=CN%22C%23=CC=C(C=C%23)N%24CCN(CC%24)C%25=CC=C(C=C%25)OC[C@H]%26CO[C@](O%26)(CN%27N=CN=C%27)C%28=C(C=C(C=C%28)F)F	Sterol 14α-demethylase inhibitor	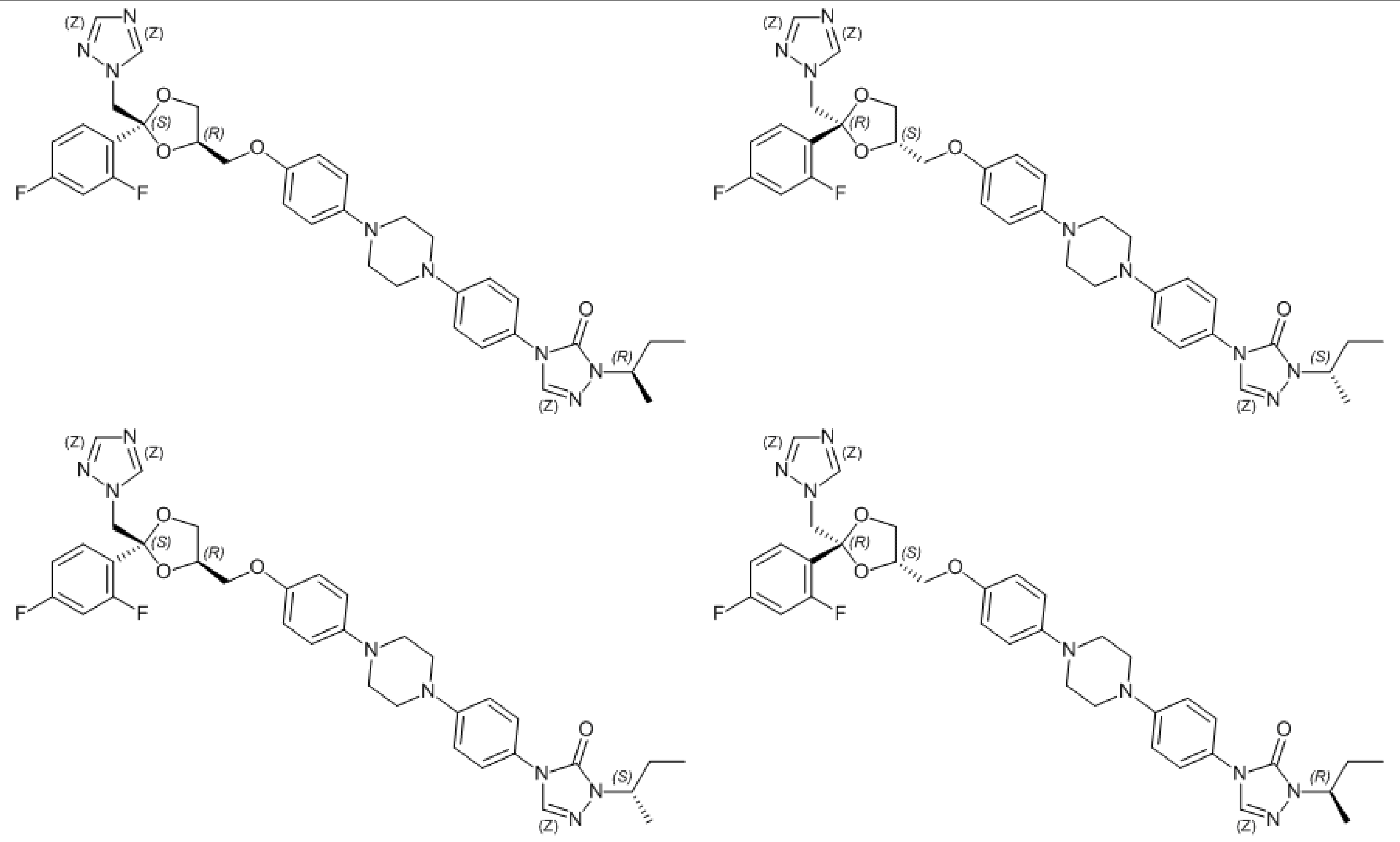	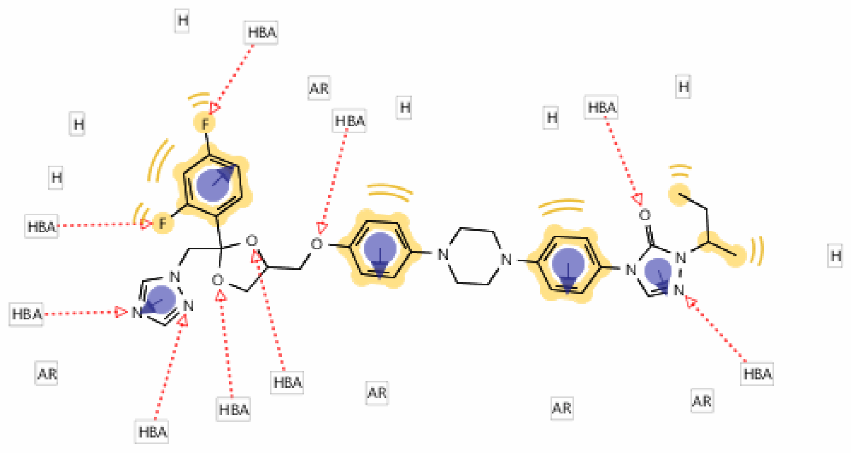
SCH 42427	121650-83-7	HFGZFHCWKKQGIS-NOZJJQNGSA-N	InChI=1S/C13H15F2N3O3S/c1-9(22(2,20)21)13(19,6-18-8-16-7-17-18)11-4-3-10(14)5-12(11)15/h3-5,7-9,19H,6H2,1-2H3/t9-,13-/m1/s1	C[C@@H](S(C)(=O)=O)[C@](CN1C=NC=N1)(C2=C(F)C=C(F)C=C2)O	Sterol 14α-demethylase inhibitor	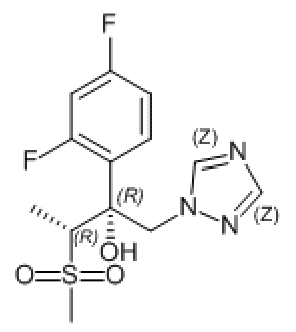	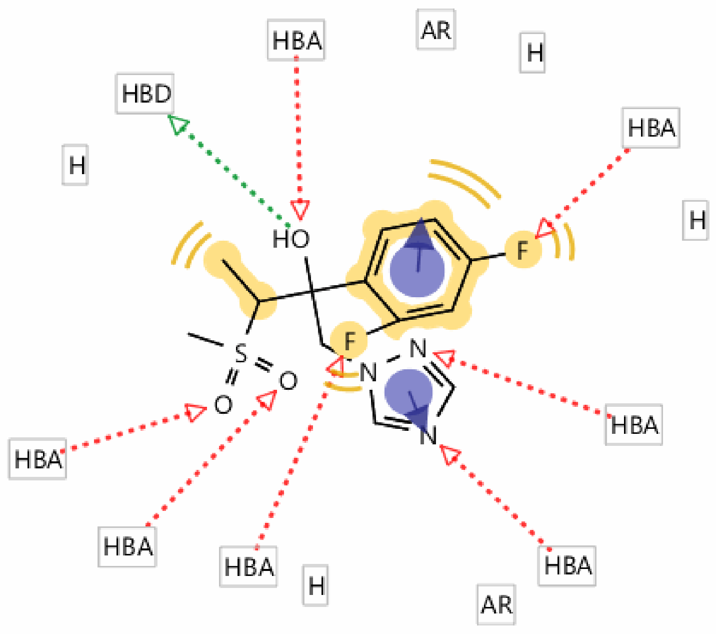
SCH 51048	161532-65-6	OPFHZSVWSCMEPV-AYAMJOBCSA-N	InChI=1S/C37H42F2N8O3/c1-3-29(4-2)47-36(48)46(26-42-47)32-8-6-30(7-9-32)43-15-17-44(18-16-43)31-10-12-33(13-11-31)49-21-27-20-37(50-22-27,23-45-25-40-24-41-45)34-14-5-28(38)19-35(34)39/h5-14,19,24-27,29H,3-4,15-18,20-23H2,1-2H3/t27-,37+/m1/s1	CCC(N(N=CN1C2=CC=C(N3CCN(C4=CC=C(OC[C@H]5C[C@](C6=C(F)C=C(F)C=C6)(OC5)CN7N=CN=C7)C=C4)CC3)C=C2)C1=O)CC	Sterol 14α-demethylase inhibitor	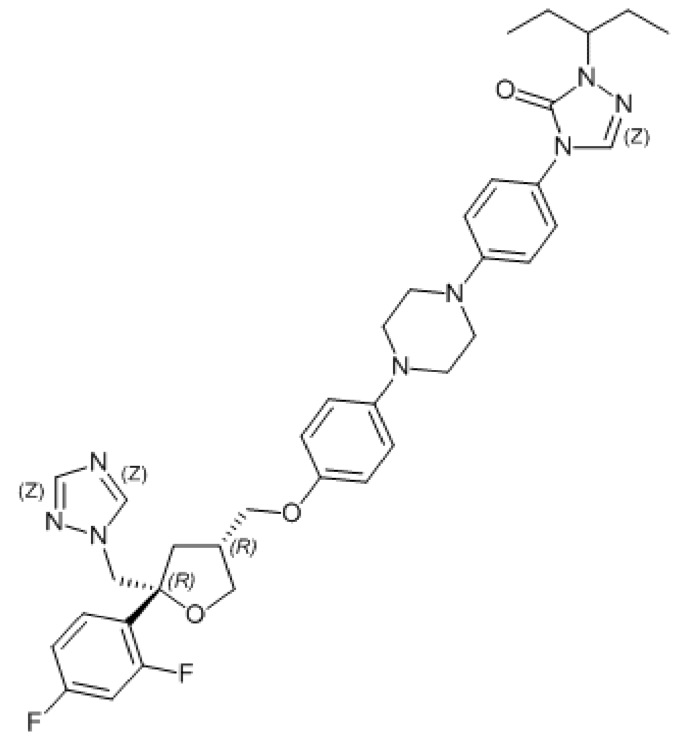	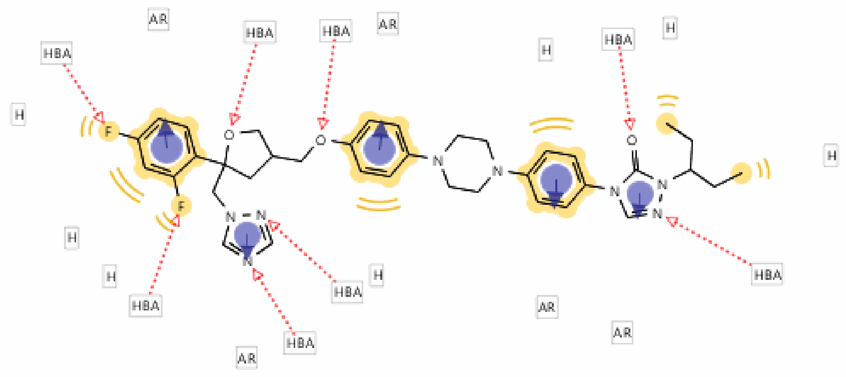
SDZ 89-485	103183-65-9	LHDGDQZODHEIPT-INIZCTEOSA-N	InChI=1S/C16H20ClN3O/c1-15(2,12-3-4-12)16(21,9-20-11-18-10-19-20)13-5-7-14(17)8-6-13/h5-8,10-12,21H,3-4,9H2,1-2H3/t16-/m0/s1	CC([C@](O)(CN1C=NC=N1)C2=CC=C(C=C2)Cl)(C)C3CC3	Sterol 14α-demethylase inhibitor	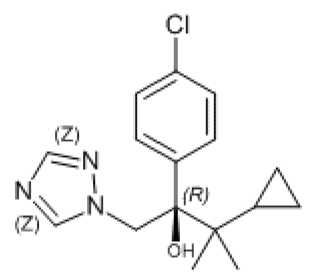	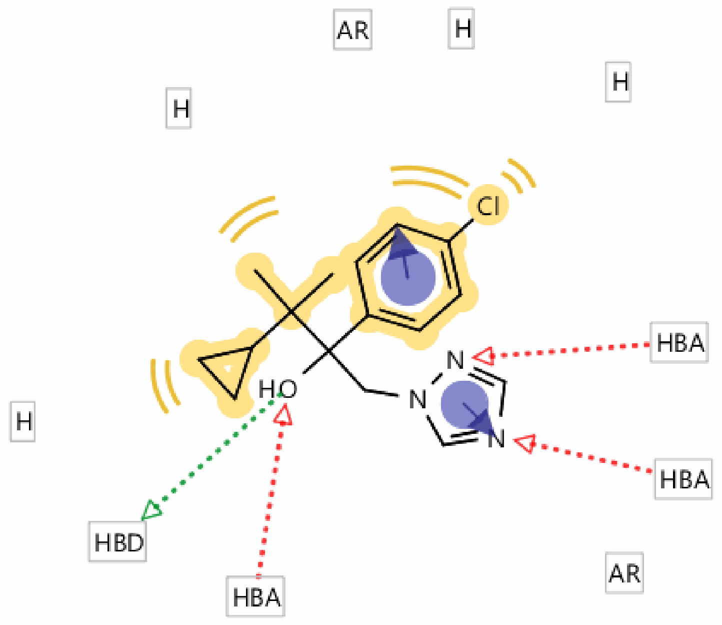

Common name	CAS Reg. No.	InChIKey	InChI	SMILES	Primary target	2D Structure	Pharmacophore[1]
Simeconazole	149508-90-7	FECFVNIPATZKGO-IKFJUQJOSA-N	InChI=1S/2C14H20FN3OSi/c2*1-20(2,3)9-14(19,8-18-11-16-10-17-18)12-4-6-13(15)7-5-12/h2*4-7,10-11,19H,8-9H2,1-3H3/t2*14-/m10/s1	C[Si](C[C@](O)(CN1C=NC=N1)C2=CC=C(C=C2)F)(C)C.C[Si](C[C@@](O)(CN3C=NC=N3)C4=CC=C(C=C4)F)(C)C	Sterol 14α-demethylase inhibitor	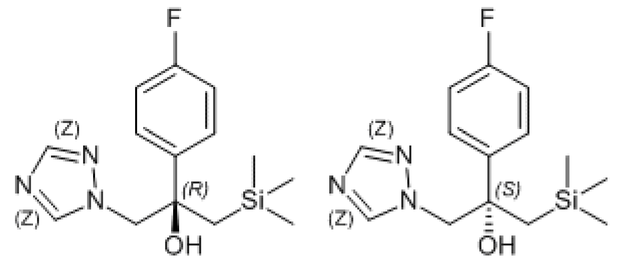	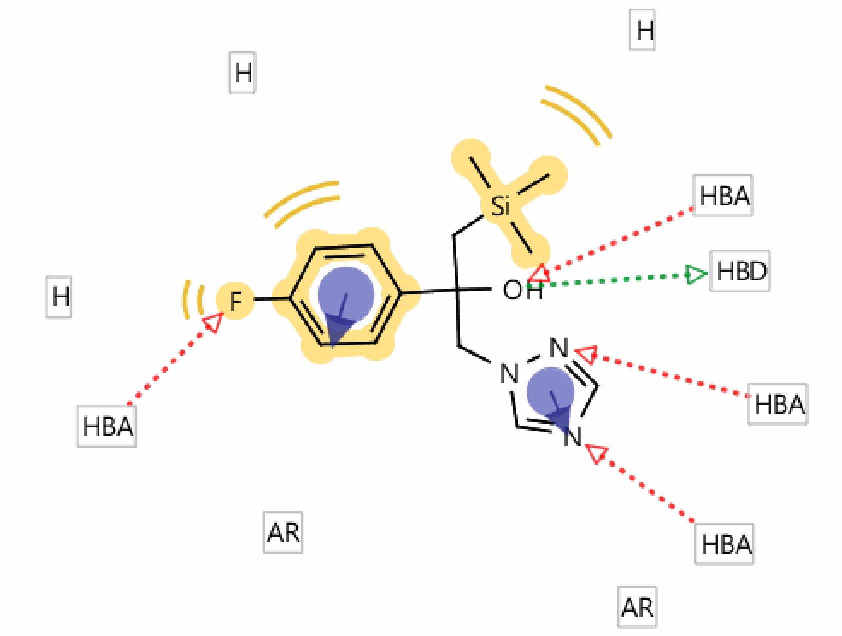
SSF 109 (huanjunzuo)	129586-32-9	WSBKLNJAJZVBAF-PNSAJKJISA-N	InChI=1S/2C15H18ClN3O/c2*16-13-7-5-12(6-8-13)15(20)9-3-1-2-4-14(15)19-11-17-10-18-19/h2*5-8,10-11,14,20H,1-4,9H2/t2*14-,15+/m10/s1	ClC1=CC=C([C@@]2(O)CCCCC[C@H]2N3C=NC=N3)C=C1.ClC4=CC=C([C@]5(O)CCCCC[C@@H]5N6C=NC=N6)C=C4	Sterol 14α-demethylase inhibitor	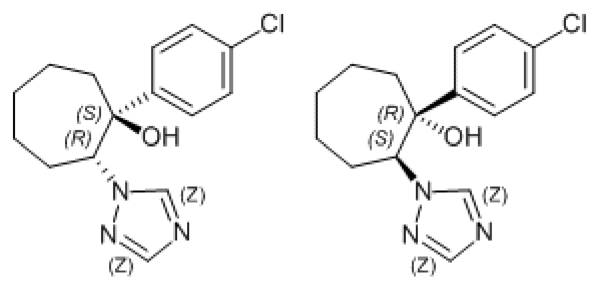	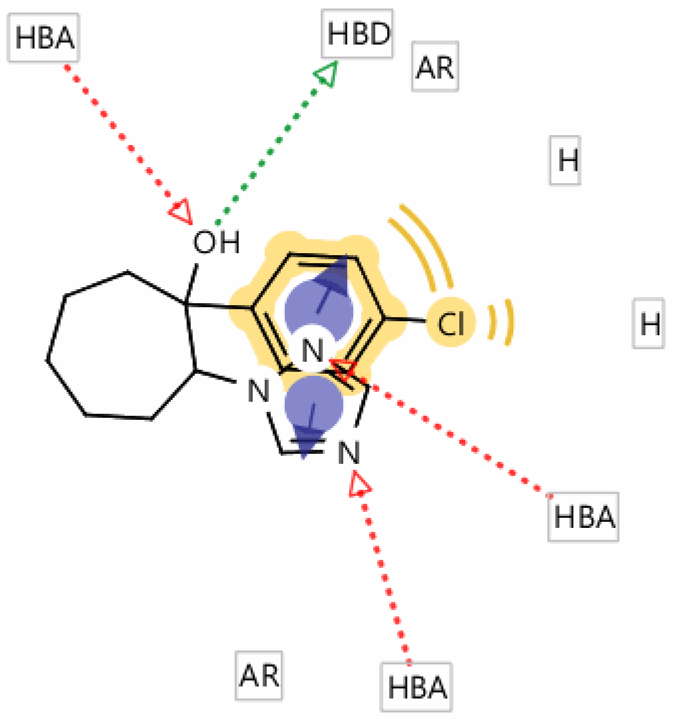
SSY 726	136067-88-4	NZEBWFSFYQECNY-AWEZNQCLSA-N	InChI=1S/C15H18F3N3O3S/c1-13(2,25(3,23)24)14(22,8-21-10-19-9-20-21)11-4-6-12(7-5-11)15(16,17)18/h4-7,9-10,22H,8H2,1-3H3/t14-/m0/s1	CC(S(C)(=O)=O)(C)[C@](O)(CN1C=NC=N1)C2=CC=C(C=C2)C(F)(F)F	Sterol 14α-demethylase inhibitor	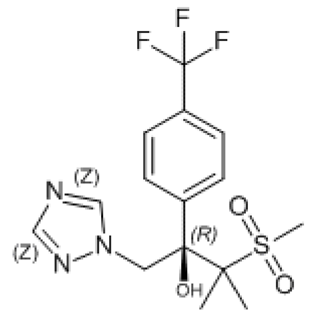	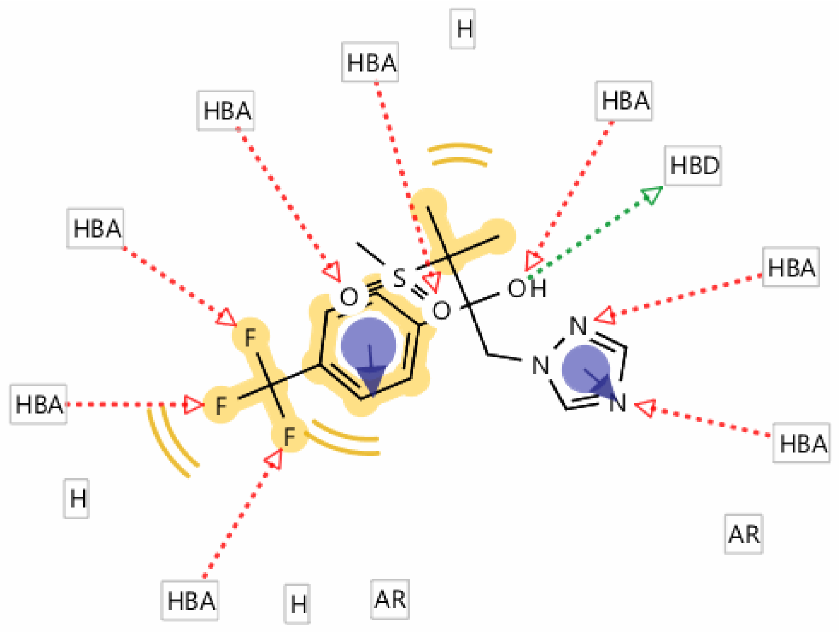
SYN 2506		IUFJTCWVBOVKJY-ONOMSOESSA-N	InChI=1S/C29H36F2N8O2/c1-4-23(5-2)39-28(40)38(20-34-39)25-9-7-24(8-10-25)36-14-12-35(13-15-36)21(3)29(41,17-37-19-32-18-33-37)26-11-6-22(30)16-27(26)31/h6-11,16,18-21,23,41H,4-5,12-15,17H2,1-3H3/t21-,29-/m1/s1	CCC(N(N=CN1C2=CC=C(N3CCN([C@@H]([C@](CN4C=NC=N4)(C5=C(F)C=C(F)C=C5)O)C)CC3)C=C2)C1=O)CC	Sterol 14α-demethylase inhibitor	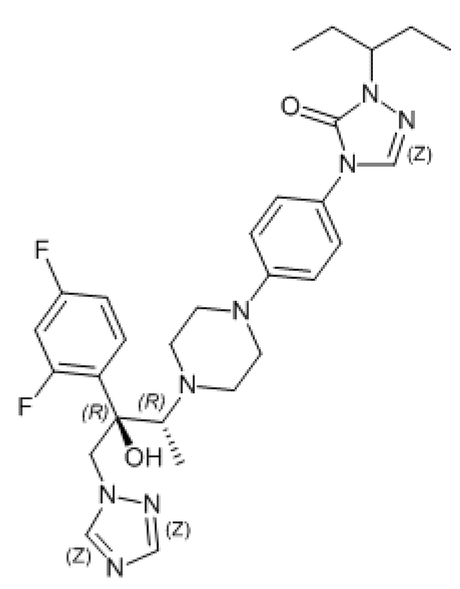	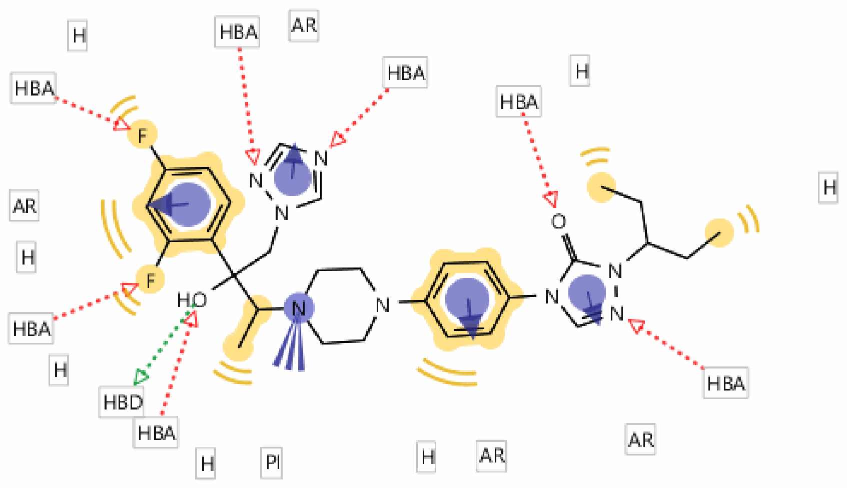
SYN 2836	210562-94-0	RKIFSGOQLRESME-JPZYQRIQSA-N	InChI=1S/C32H31F5N8O2/c1-22(31(47,18-43-20-38-19-39-43)28-11-6-25(33)16-29(28)34)41-12-14-42(15-13-41)26-7-9-27(10-8-26)44-21-40-45(30(44)46)17-23-2-4-24(5-3-23)32(35,36)37/h2-11,16,19-22,47H,12-15,17-18H2,1H3/t22-,31-/m1/s1	C[C@H]([C@](O)(CN1C=NC=N1)C2=C(C=C(C=C2)F)F)N3CCN(CC3)C4=CC=C(C=C4)N(C=NN5CC6=CC=C(C=C6)C(F)(F)F)C5=O	Sterol 14α-demethylase inhibitor	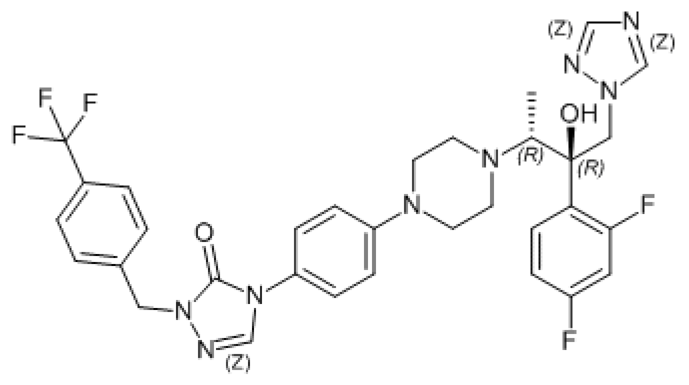	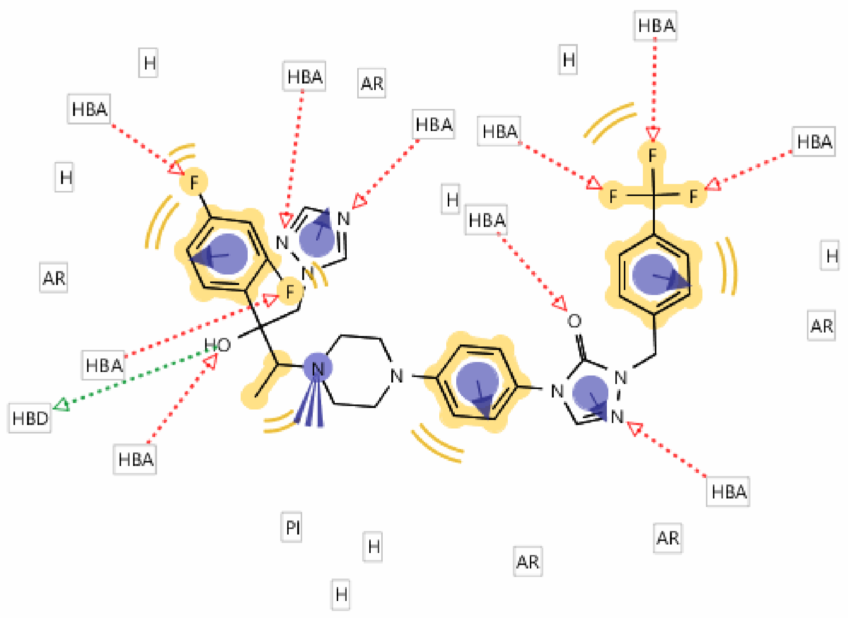

Common name	CAS Reg. No.	InChIKey	InChI	SMILES	Primary target	2D Structure	Pharmacophore[1]
SYN 2869	210562-98-4	HEAOYZUDBIUSJN-JPZYQRIQSA-N	InChI=1S/C32H31F5N8O3/c1-22(31(47,18-43-20-38-19-39-43)28-11-4-24(33)16-29(28)34)41-12-14-42(15-13-41)25-5-7-26(8-6-25)44-21-40-45(30(44)46)17-23-2-9-27(10-3-23)48-32(35,36)37/h2-11,16,19-22,47H,12-15,17-18H2,1H3/t22-,31-/m1/s1	C[C@H]([C@](O)(CN1C=NC=N1)C2=C(C=C(C=C2)F)F)N3CCN(CC3)C4=CC=C(C=C4)N(C=NN5CC6=CC=C(C=C6)OC(F)(F)F)C5=O	Sterol 14α-demethylase inhibitor	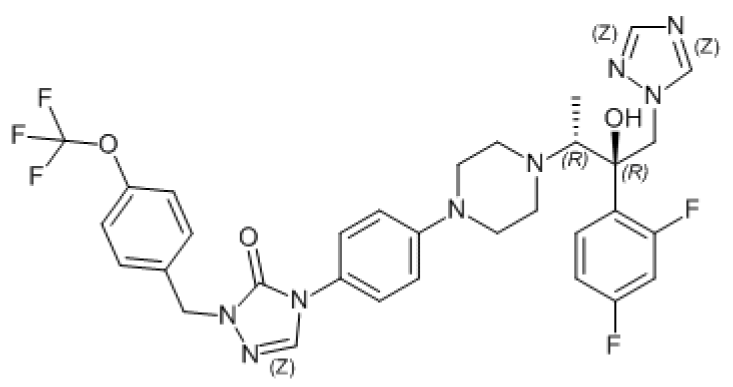	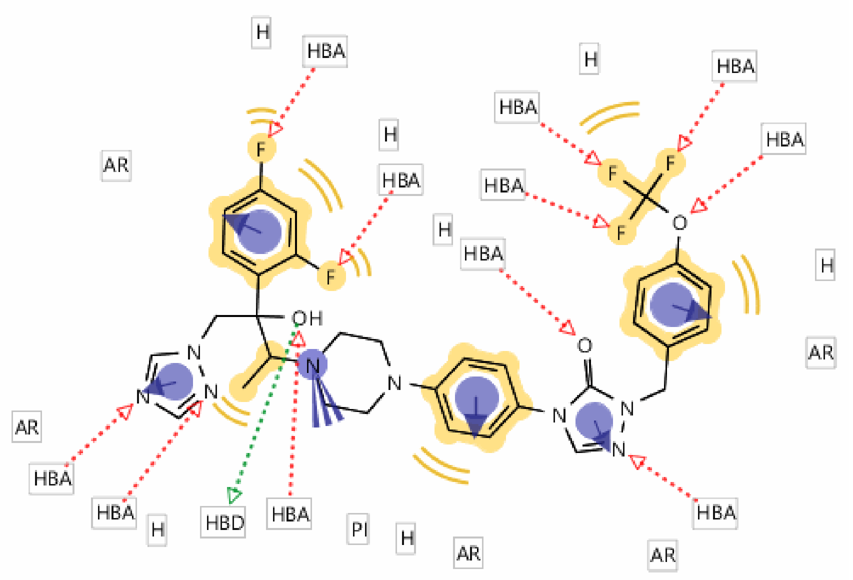
SYN 2903	210563-03-4	PCYZZKYRNGSDMF-ROTAYESASA-N	InChI=1S/C32H30F6N8O2/c1-21(31(48,17-44-19-39-18-40-44)26-8-6-24(33)14-27(26)34)42-10-12-43(13-11-42)29-9-7-25(15-28(29)35)45-20-41-46(30(45)47)16-22-2-4-23(5-3-22)32(36,37)38/h2-9,14-15,18-21,48H,10-13,16-17H2,1H3/t21-,31-/m1/s1	C[C@H]([C@](O)(CN1C=NC=N1)C2=C(C=C(C=C2)F)F)N3CCN(CC3)C4=C(C=C(C=C4)N(C=NN5CC6=CC=C(C=C6)C(F)(F)F)C5=O)F	Sterol 14α-demethylase inhibitor	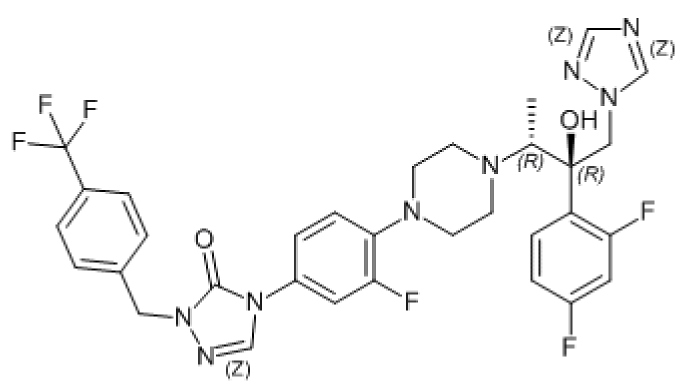	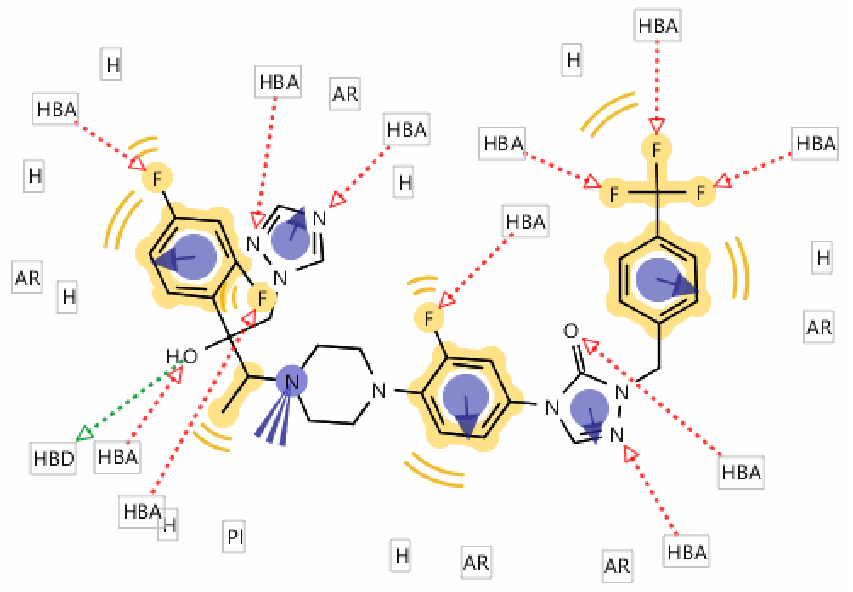
SYN 2921	227103-93-7	WYVLGGLILXPIQP-ROTAYESASA-N	InChI=1S/C32H30F6N8O3/c1-21(31(48,17-44-19-39-18-40-44)26-8-4-23(33)14-27(26)34)42-10-12-43(13-11-42)29-9-5-24(15-28(29)35)45-20-41-46(30(45)47)16-22-2-6-25(7-3-22)49-32(36,37)38/h2-9,14-15,18-21,48H,10-13,16-17H2,1H3/t21-,31-/m1/s1	C[C@H]([C@](O)(CN1C=NC=N1)C2=C(C=C(C=C2)F)F)N3CCN(CC3)C4=C(C=C(C=C4)N(C=NN5CC6=CC=C(C=C6)OC(F)(F)F)C5=O)F	Sterol 14α-demethylase inhibitor	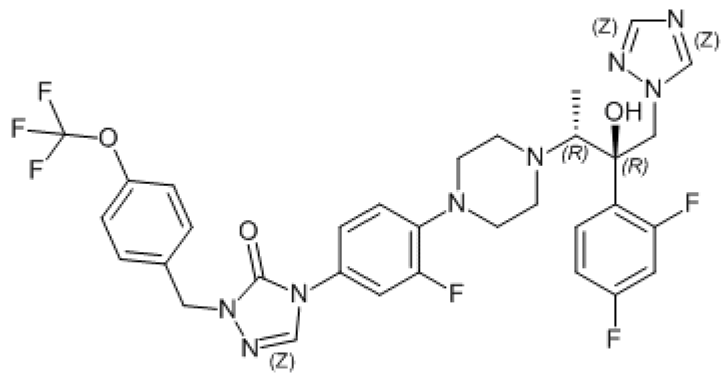	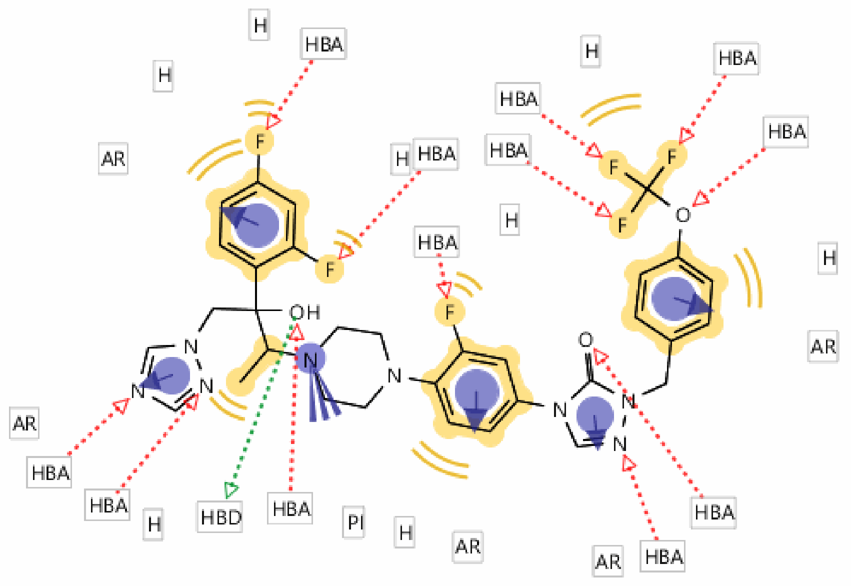
T 8581		KDHNDVXHMRYACP-NSHDSACASA-N	InChI=1S/C12H10F4N4O2/c13-7-1-2-8(9(14)3-7)11(22,12(15,16)10(17)21)4-20-6-18-5-19-20/h1-3,5-6,22H,4H2,(H2,17,21)/t11-/m0/s1	FC1=CC(F)=C(C=C1)[C@@](O)(CN2C=NC=N2)C(F)(C(N)=O)F	Sterol 14α-demethylase inhibitor	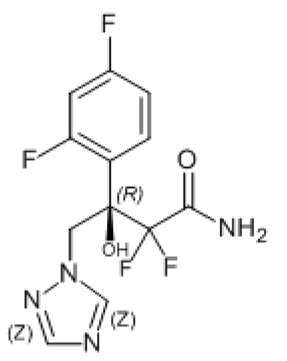	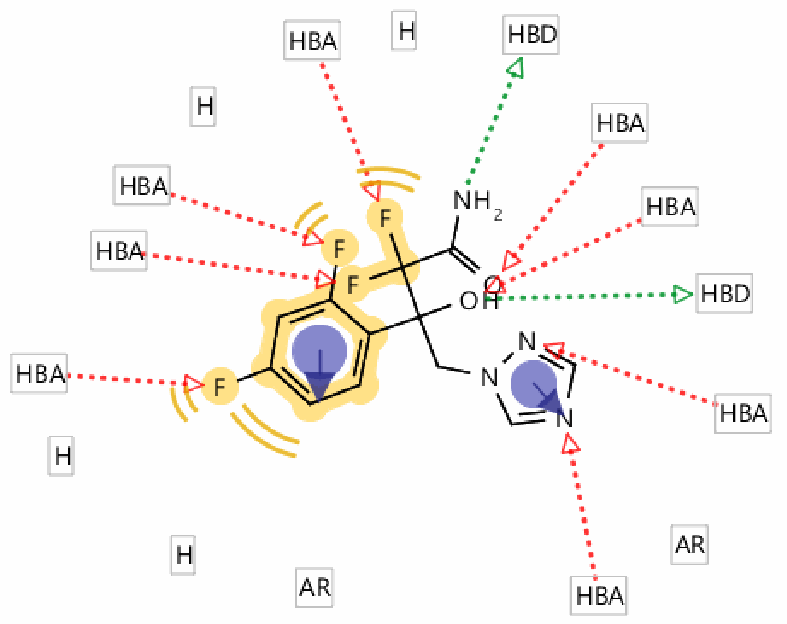
TAK 187	155432-64-7	CBHTUKXHISWMTH-JLCFBVMHSA-N	InChI=1S/C23H20F6N6O3/c1-14(22(37,9-33-12-30-11-31-33)18-7-2-15(24)8-19(18)25)35-21(36)34(13-32-35)16-3-5-17(6-4-16)38-10-23(28,29)20(26)27/h2-8,11-14,20,37H,9-10H2,1H3/t14-,22-/m1/s1	C[C@@H](N(N=CN1C2=CC=C(OCC(C(F)F)(F)F)C=C2)C1=O)[C@](CN3C=NC=N3)(C4=C(F)C=C(F)C=C4)O	Sterol 14α-demethylase inhibitor	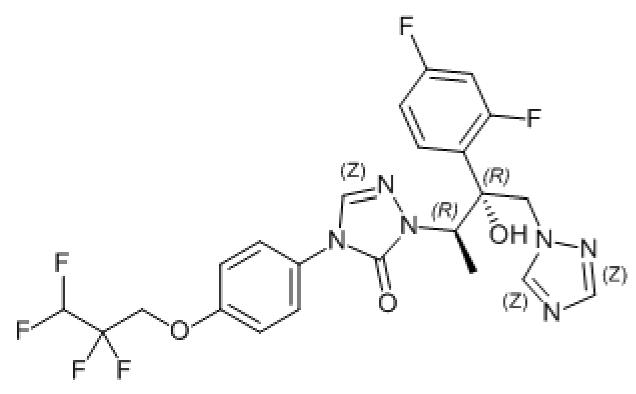	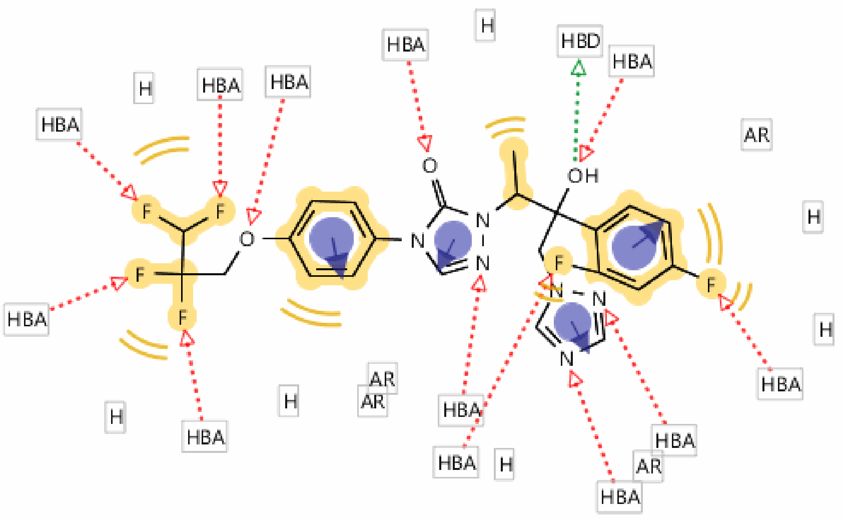
TAK 456	181869-54-5	JTNIHNJCRCYZSU-IVZQSRNASA-N	InChI=1S/C22H21F2N9O2/c1-15(22(35,11-30-13-25-12-27-30)19-7-2-16(23)10-20(19)24)31-8-9-32(21(31)34)17-3-5-18(6-4-17)33-14-26-28-29-33/h2-7,10,12-15,35H,8-9,11H2,1H3/t15-,22-/m1/s1	C[C@H]([C@](O)(CN1C=NC=N1)C2=C(C=C(C=C2)F)F)N(CCN3C4=CC=C(C=C4)N5C=NN=N5)C3=O	Sterol 14α-demethylase inhibitor	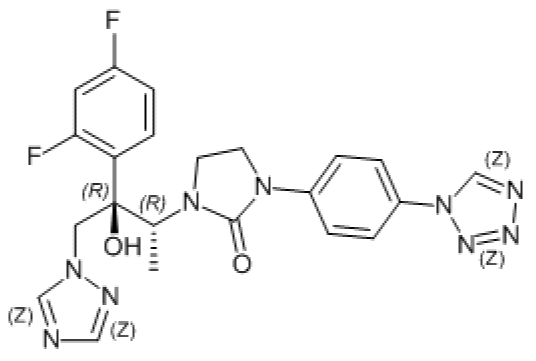	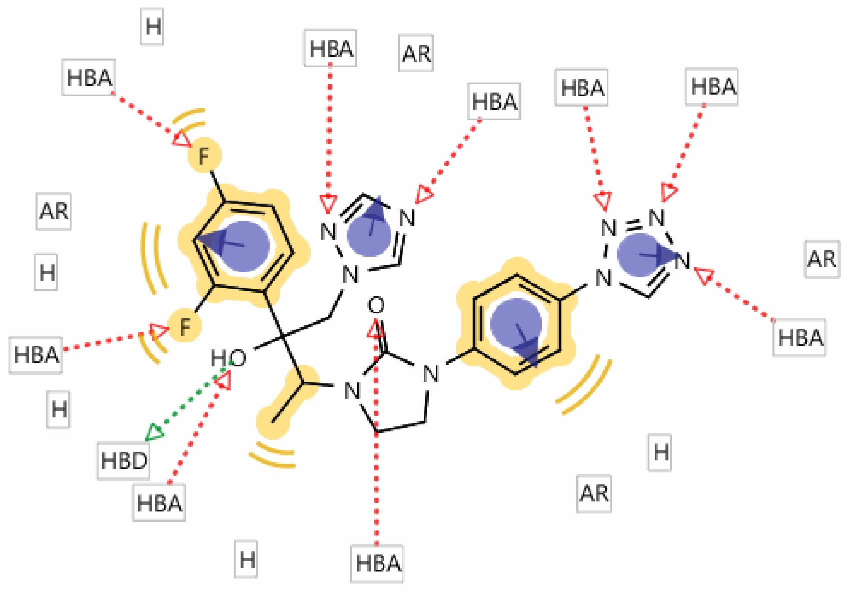

Common name	CAS Reg. No.	InChIKey	InChI	SMILES	Primary target	2D Structure	Pharmacophore[1]
Tebuconazole	107534-96-3	WTDBZJUHDCAMBD-RRHAQCGESA-N	InChI=1S/2C16H22ClN3O/c2*1-15(2,3)16(21,10-20-12-18-11-19-20)9-8-13-4-6-14(17)7-5-13/h2*4-7,11-12,21H,8-10H2,1-3H3/t2*16-/m10/s1	CC([C@@](O)(CCC1=CC=C(C=C1)Cl)CN2C=NC=N2)(C)C.CC([C@](O)(CCC3=CC=C(C=C3)Cl)CN4C=NC=N4)(C)C	Sterol 14α-demethylase inhibitor	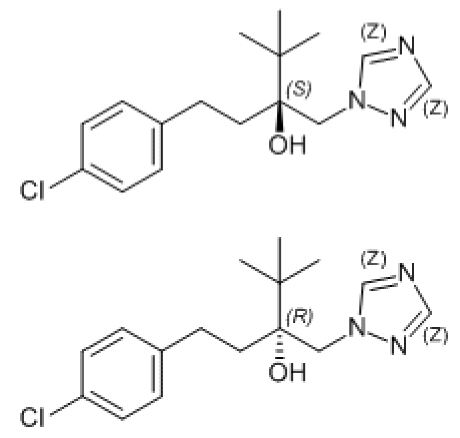	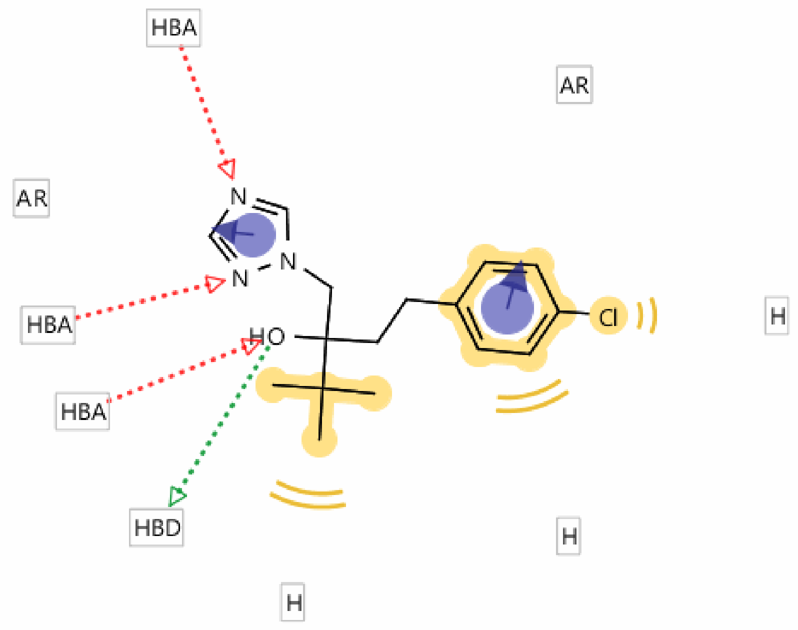
Terconazole (triaconazole)	67915-31-5	AJIBSZMJDYUTCJ-ICCBHQQHSA-N	InChI=1S/2C26H31Cl2N5O3/c2*1-19(2)31-9-11-32(12-10-31)21-4-6-22(7-5-21)34-14-23-15-35-26(36-23,16-33-18-29-17-30-33)24-8-3-20(27)13-25(24)28/h2*3-8,13,17-19,23H,9-12,14-16H2,1-2H3/t2*23-,26-/m10/s1	CC(C)N1CCN(CC1)C2=CC=C(C=C2)OC[C@H]3O[C@@](C4=C(C=C(C=C4)Cl)Cl)(CN5C=NC=N5)OC3.CC(C)N6CCN(CC6)C7=CC=C(C=C7)OC[C@@H]8O[C@](C9=C(C=C(C=C9)Cl)Cl)(CN%10C=NC=N%10)OC8	Sterol 14α-demethylase inhibitor	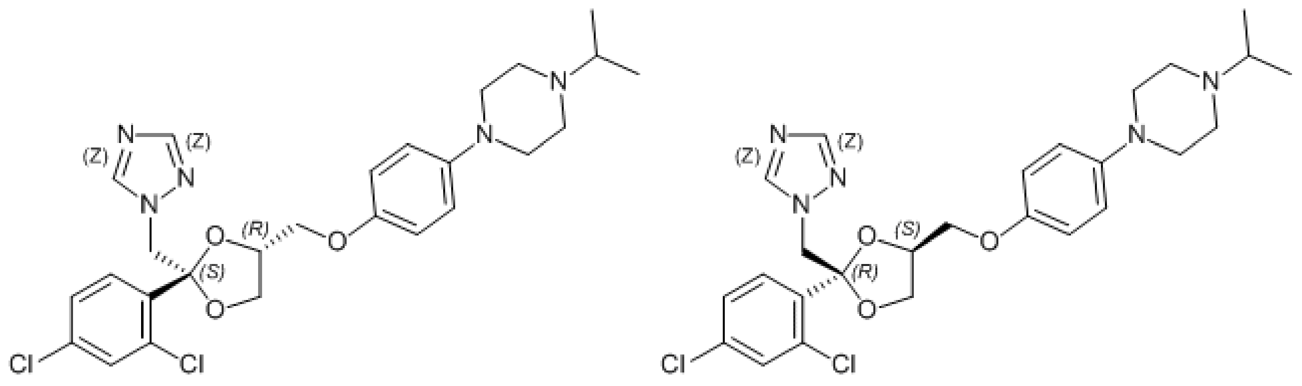	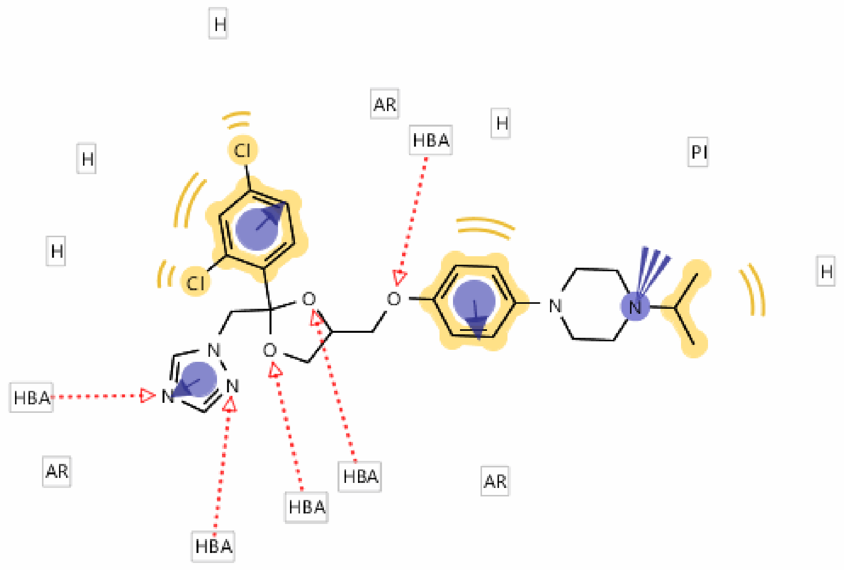
Tetraconazole	112281-77-3	ZXGSSOGNBOQRJO-RMTNWKGQSA-N	InChI=1S/2C13H11Cl2F4N3O/c2*14-9-1-2-10(11(15)3-9)8(4-22-7-20-6-21-22)5-23-13(18,19)12(16)17/h2*1-3,6-8,12H,4-5H2/t2*8-/m10/s1	ClC1=CC(Cl)=C([C@](CN2C=NC=N2)(COC(C(F)F)(F)F)[H])C=C1.ClC3=CC(Cl)=C([C@@](CN4C=NC=N4)(COC(C(F)F)(F)F)[H])C=C3	Sterol 14α-demethylase inhibitor	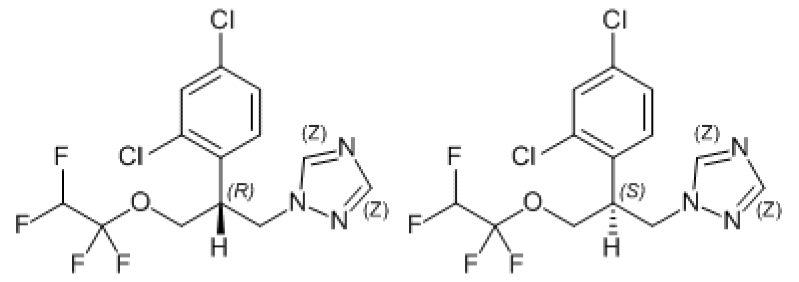	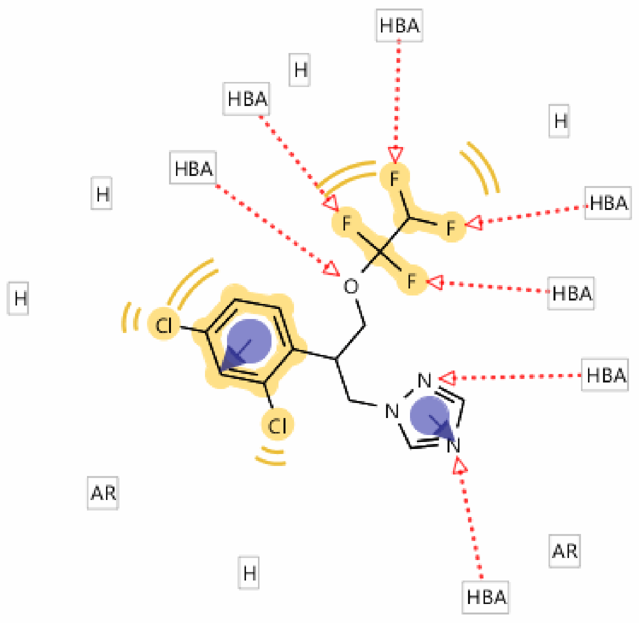
Triadimefon	43121-43-3	JXTANTZZZMKBPO-JACLSRQLSA-N	InChI=1S/2C14H16ClN3O2/c2*1-14(2,3)12(19)13(18-9-16-8-17-18)20-11-6-4-10(15)5-7-11/h2*4-9,13H,1-3H3/t2*13-/m10/s1	CC(C([C@]([H])(N1C=NC=N1)OC2=CC=C(C=C2)Cl)=O)(C)C.CC(C([C@@]([H])(N3C=NC=N3)OC4=CC=C(C=C4)Cl)=O)(C)C	Sterol 14α-demethylase inhibitor	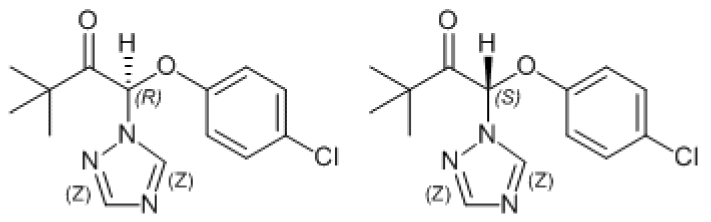	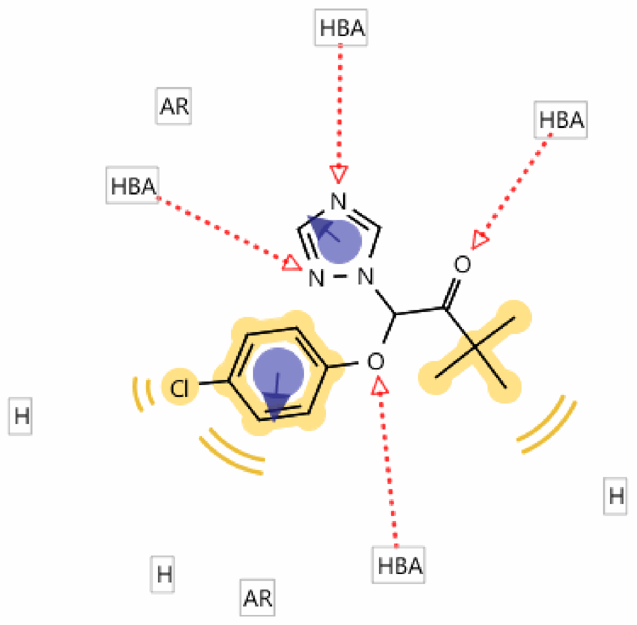
Triadimenol	55219-65-3	PZDHSXRELTYDNI-FSCVMSEGSA-N	InChI=1S/4C14H18ClN3O2/c4*1-14(2,3)12(19)13(18-9-16-8-17-18)20-11-6-4-10(15)5-7-11/h4*4-9,12-13,19H,1-3H3/t2*12-,13+;2*12-,13-/m1010/s1	CC(C)(C)[C@H](O)[C@@](N1C=NC=N1)(OC2=CC=C(Cl)C=C2)[H].CC(C)(C)[C@@H](O)[C@](N3C=NC=N3)(OC4=CC=C(Cl)C=C4)[H].CC(C)(C)[C@H](O)[C@](N5C=NC=N5)(OC6=CC=C(Cl)C=C6)[H].CC(C)(C)[C@@H](O)[C@@](N7C=NC=N7)(OC8=CC=C(Cl)C=C8)[H]	Sterol 14α-demethylase inhibitor	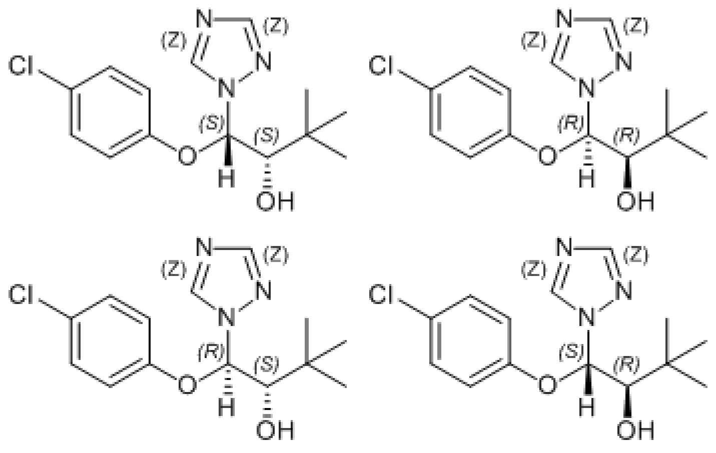	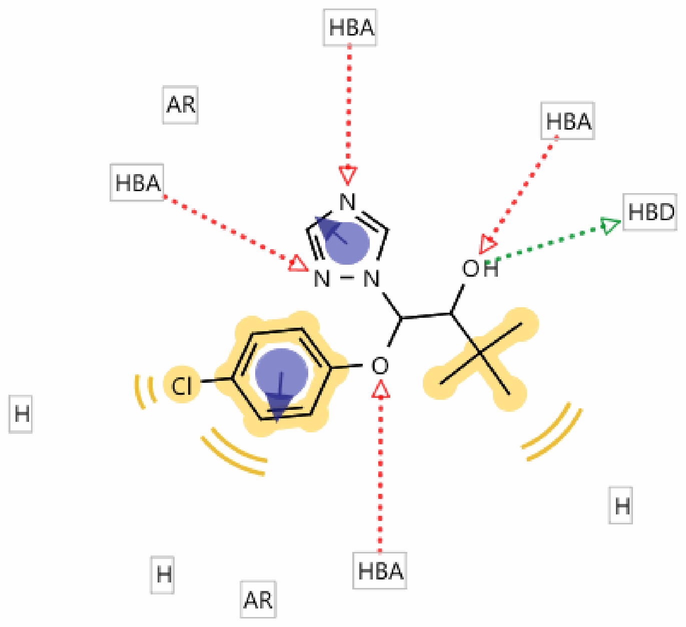

Common name	CAS Reg. No.	InChIKey	InChI	SMILES	Primary target	2D Structure	Pharmacophore[1]
Triticonazole	131983-72-7	CBFIZWGJMDQBAI-CYNNTGGJSA-N	InChI=1S/2C17H20ClN3O/c2*1-16(2)8-7-14(9-13-3-5-15(18)6-4-13)17(16,22)10-21-12-19-11-20-21/h2*3-6,9,11-12,22H,7-8,10H2,1-2H3/b2*14-9+/t2*17-/m10/s1	CC1(C)CC/C([C@]1(O)CN2C=NC=N2)=C\C3=CC=C(C=C3)Cl.CC4(C)CC/C([C@@]4(O)CN5C=NC=N5)=C\C6=CC=C(C=C6)Cl	Sterol 14α-demethylase inhibitor	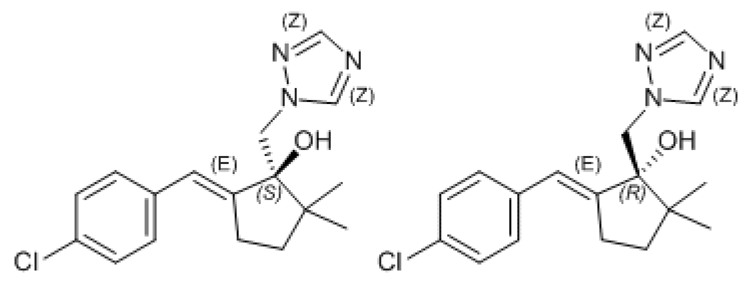	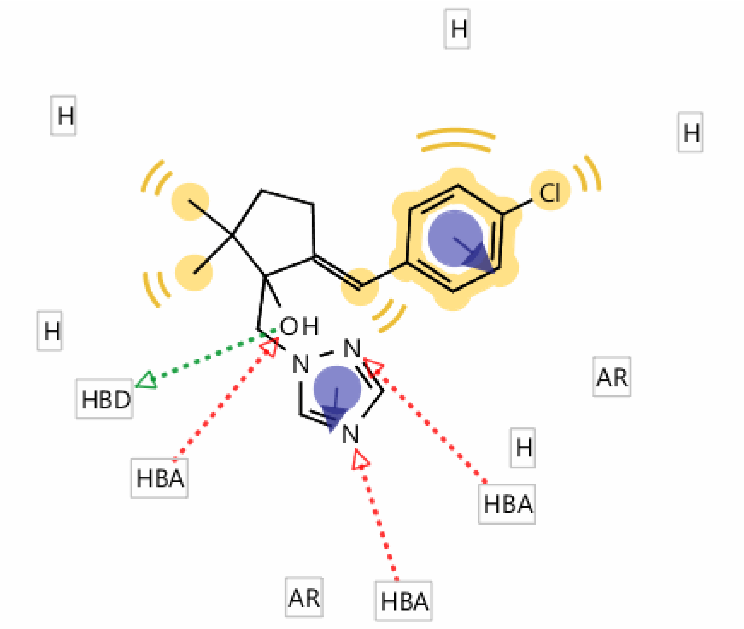
UK 47265	81886-52-4	CMGCKUPKNSSPER-UHFFFAOYSA-N	InChI=1S/C13H12Cl2N6O/c14-10-1-2-11(12(15)3-10)13(22,4-20-8-16-6-18-20)5-21-9-17-7-19-21/h1-3,6-9,22H,4-5H2	ClC1=CC(Cl)=C(C=C1)C(O)(CN2C=NC=N2)CN3C=NC=N3	Sterol 14α-demethylase inhibitor	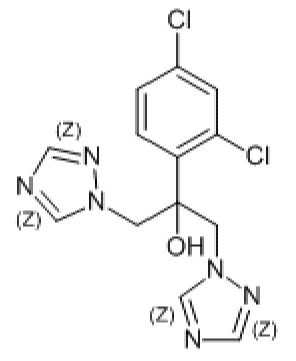	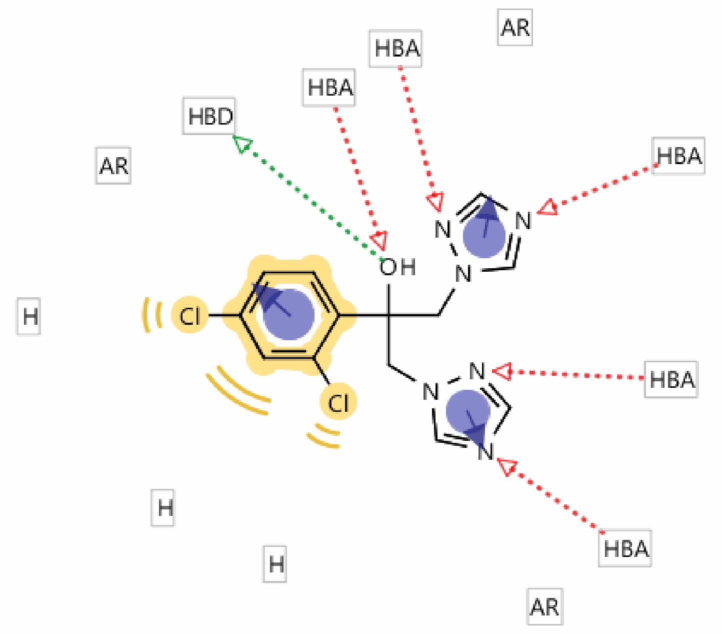
UK 51486		LZPGGXYCXQYBEI-IZIBOJBPSA-N	InChI=1S/2C12H12Cl2N4O2/c2*13-8-1-2-9(10(14)3-8)12(20,4-11(15)19)5-18-7-16-6-17-18/h2*1-3,6-7,20H,4-5H2,(H2,15,19)/t2*12-/m10/s1	ClC1=CC(Cl)=C(C=C1)[C@@](O)(CC(N)=O)CN2C=NC=N2.ClC3=CC(Cl)=C(C=C3)[C@](O)(CC(N)=O)CN4C=NC=N4	Sterol 14α-demethylase inhibitor	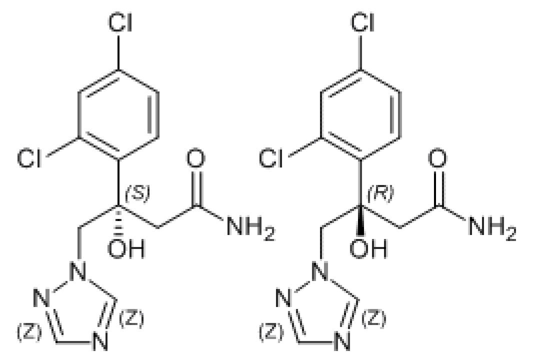	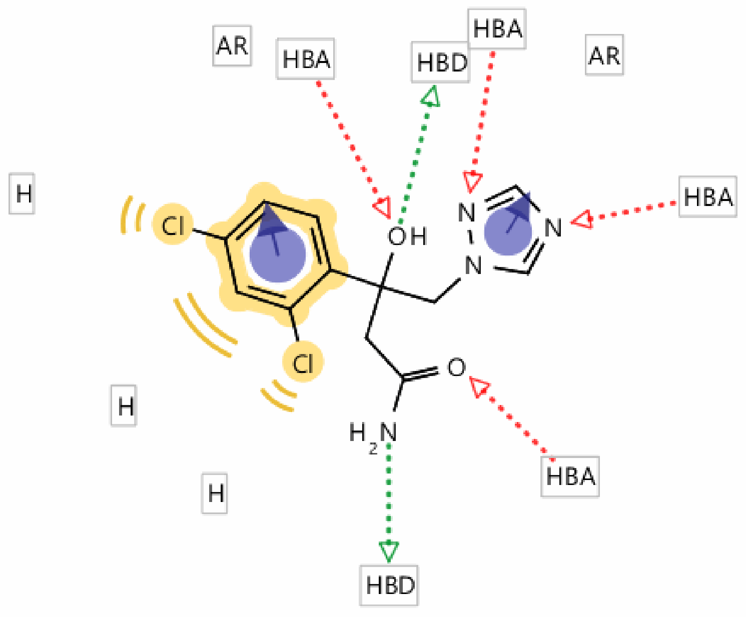
Uniconazole	83657-22-1	UKWGFELUGYSORA-JCPQPXMASA-N	InChI=1S/2C15H18ClN3O/c2*1-15(2,3)14(20)13(19-10-17-9-18-19)8-11-4-6-12(16)7-5-11/h2*4-10,14,20H,1-3H3/b2*13-8+/t2*14-/m10/s1	CC(C)([C@@H](/C(N1N=CN=C1)=C\C2=CC=C(C=C2)Cl)O)C.CC(C)([C@H](/C(N3N=CN=C3)=C\C4=CC=C(C=C4)Cl)O)C	Sterol 14α-demethylase inhibitor	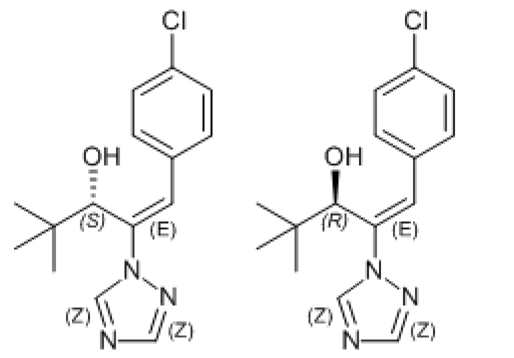	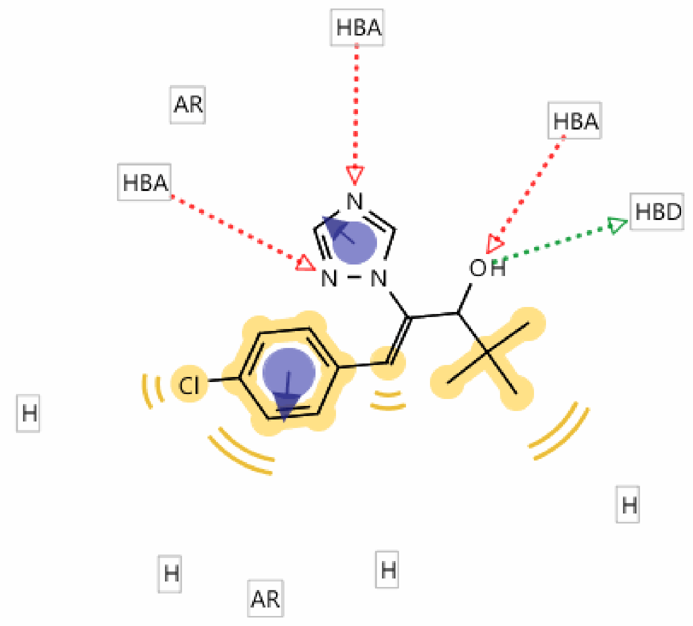
UR 9746		QJHNXGWLYVMQTP-YIILKADPSA-N	InChI=1S/C22H19F5N4O3/c1-13-21(10-30-12-28-11-29-30,17-7-6-16(23)8-18(17)24)34-19(32)9-31(13)20(33)14-2-4-15(5-3-14)22(25,26)27/h2-8,11-13,19,32H,9-10H2,1H3/t13-,19+,21-/m1/s1	C[C@H]1N(C[C@@H](O)O[C@@]1(CN2C=NC=N2)C3=C(C=C(C=C3)F)F)C(C4=CC=C(C=C4)C(F)(F)F)=O	Sterol 14α-demethylase inhibitor	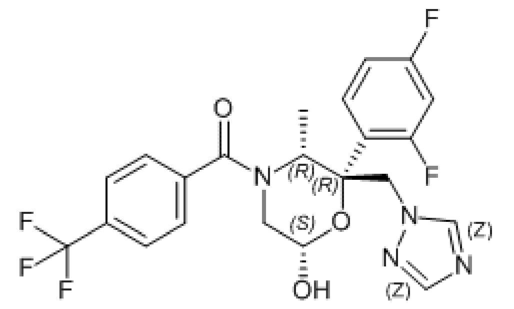	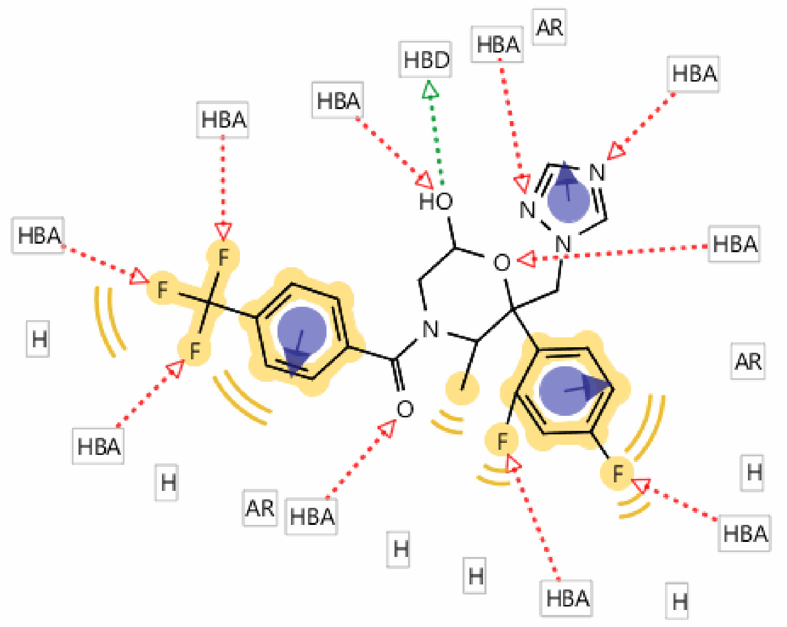
UR 9751		QSVFWYIWGYYGQN-SPLOXXLWSA-N	InChI=1S/C22H19F5N4O2/c1-14-21(11-30-13-28-12-29-30,18-7-6-17(23)10-19(18)24)33-9-8-31(14)20(32)15-2-4-16(5-3-15)22(25,26)27/h2-7,10,12-14H,8-9,11H2,1H3/t14-,21-/m1/s1	C[C@H]1N(CCO[C@@]1(CN2C=NC=N2)C3=C(C=C(C=C3)F)F)C(C4=CC=C(C=C4)C(F)(F)F)=O	Sterol 14α-demethylase inhibitor	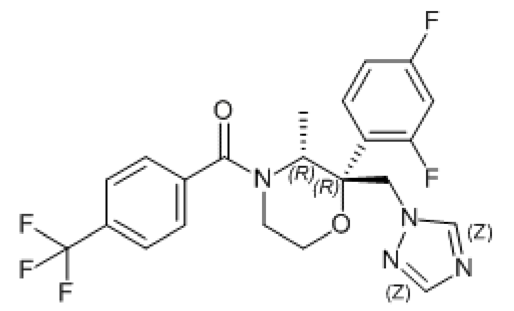	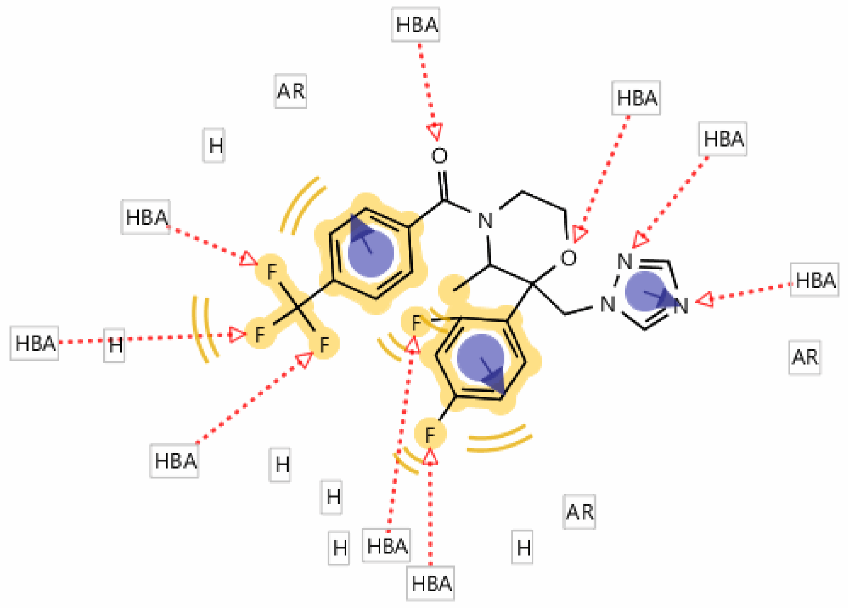

Common name	CAS Reg. No.	InChIKey	InChI	SMILES	Primary target	2D Structure	Pharmacophore[1]
Vibunazole (BAY N-7133)	80456-55-9	BGYFURWVZLQDTR-ZWZQDMJTSA-N	InChI=1S/2C15H20ClN3O2/c2*1-14(2,3)15(20,8-19-11-17-10-18-19)9-21-13-6-4-12(16)5-7-13/h2*4-7,10-11,20H,8-9H2,1-3H3/t2*15-/m10/s1	CC([C@@](O)(CN1C=NC=N1)COC2=CC=C(C=C2)Cl)(C)C.CC([C@](O)(CN3C=NC=N3)COC4=CC=C(C=C4)Cl)(C)C	Sterol 14α-demethylase inhibitor	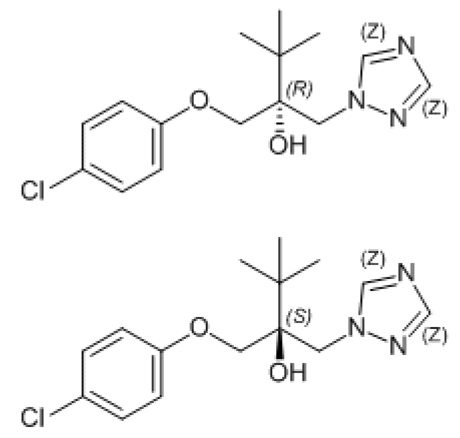	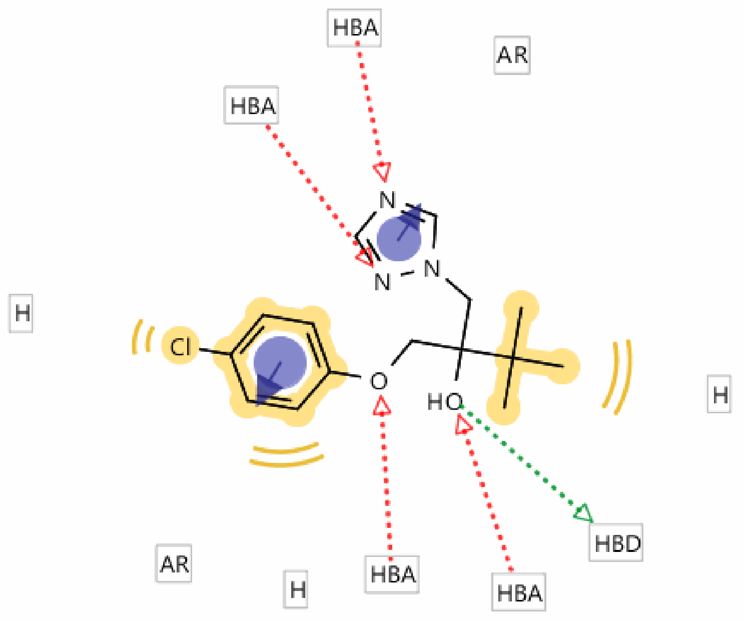
Voriconazole	137234-62-9	BCEHBSKCWLPMDN-MGPLVRAMSA-N	InChI=1S/C16H14F3N5O/c1-10(15-14(19)5-20-7-22-15)16(25,6-24-9-21-8-23-24)12-3-2-11(17)4-13(12)18/h2-5,7-10,25H,6H2,1H3/t10-,16+/m0/s1	C[C@@H](C1=NC=NC=C1F)[C@](O)(CN2C=NC=N2)C3=C(C=C(C=C3)F)F	Sterol 14α-demethylase inhibitor	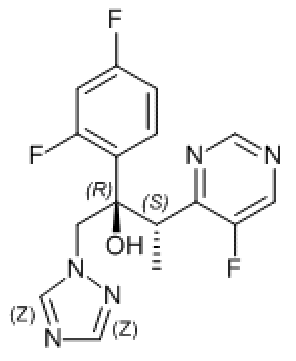	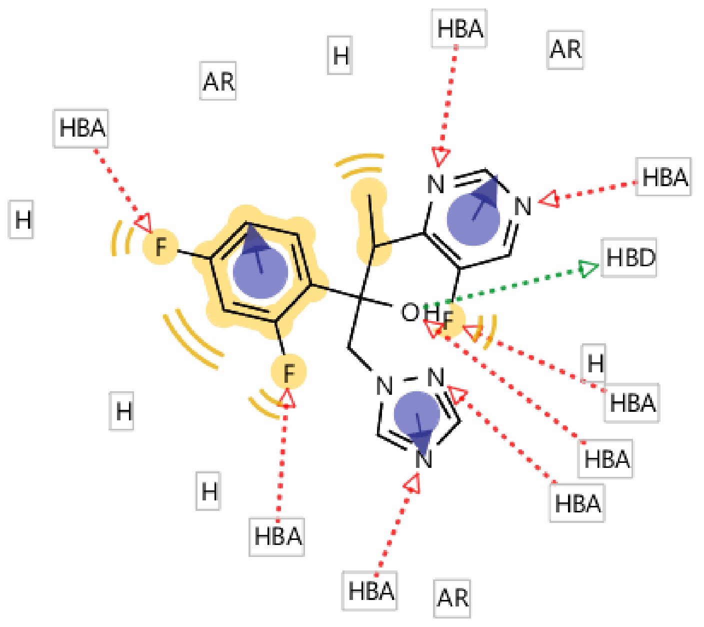
YH 1715R		BVWQNHXMENIJIP-RFAUZJTJSA-N	InChI=1S/C15H15F2N5O2S2/c1-9(25-14-20-13(24-2)21-26-14)15(23,6-22-8-18-7-19-22)11-4-3-10(16)5-12(11)17/h3-5,7-9,23H,6H2,1-2H3/t9-,15-/m1/s1	C[C@H]([C@](O)(CN1C=NC=N1)C2=C(C=C(C=C2)F)F)SC3=NC(OC)=NS3	Sterol 14α-demethylase inhibitor	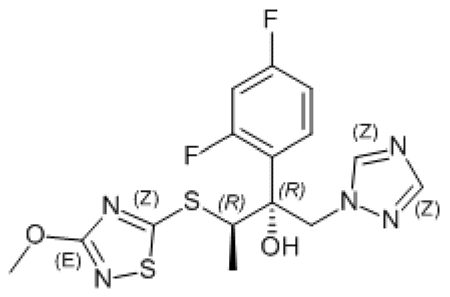	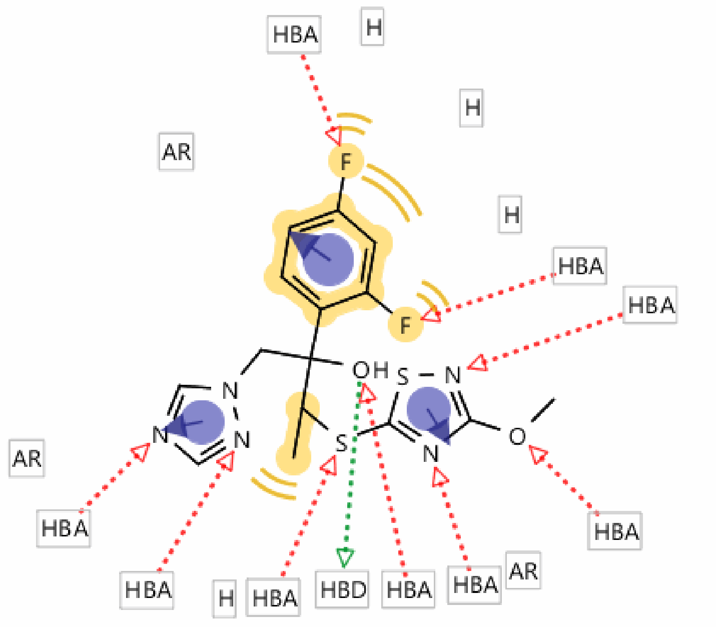
1-Dodecylimidazole (N-dodecylimidazole)	4303-67-7	JMTFLSQHQSFNTE-UHFFFAOYSA-N	InChI=1S/C15H28N2/c1-2-3-4-5-6-7-8-9-10-11-13-17-14-12-16-15-17/h12,14-15H,2-11,13H2,1H3	CCCCCCCCCCCCN1C=CN=C1	Sterol 14α-demethylase inhibitor	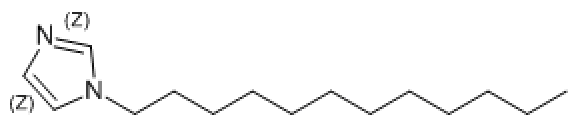	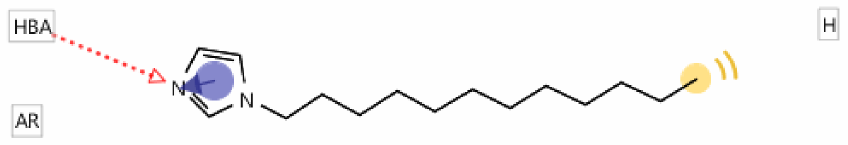
AFK 108	135330-85-7	VEBBPRCZRACGDG-BWXGWXQGSA-N	InChI=1S/2C21H26Cl2N2O/c2*1-16(2)5-4-6-17(3)9-12-26-21(14-25-11-10-24-15-25)19-8-7-18(22)13-20(19)23/h2*5,7-11,13,15,21H,4,6,12,14H2,1-3H3/b2*17-9+/t2*21-/m10/s1	C/C(C)=C\CC/C(C)=C/CO[C@@]([H])(C1=C(Cl)C=C(Cl)C=C1)CN2C=CN=C2.C/C(C)=C\CC/C(C)=C/CO[C@]([H])(C3=C(Cl)C=C(Cl)C=C3)CN4C=CN=C4	Sterol 14α-demethylase inhibitor	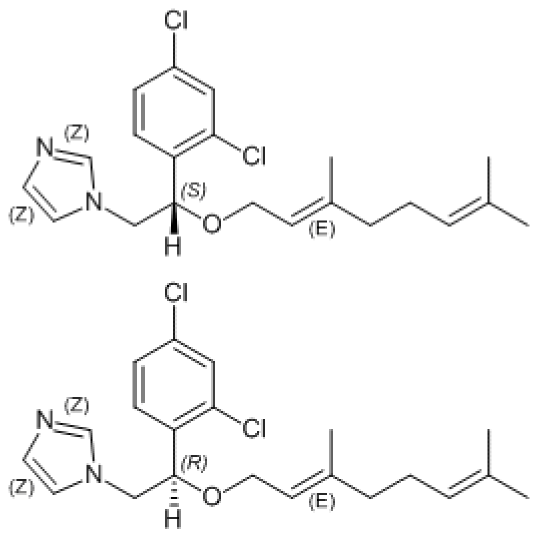	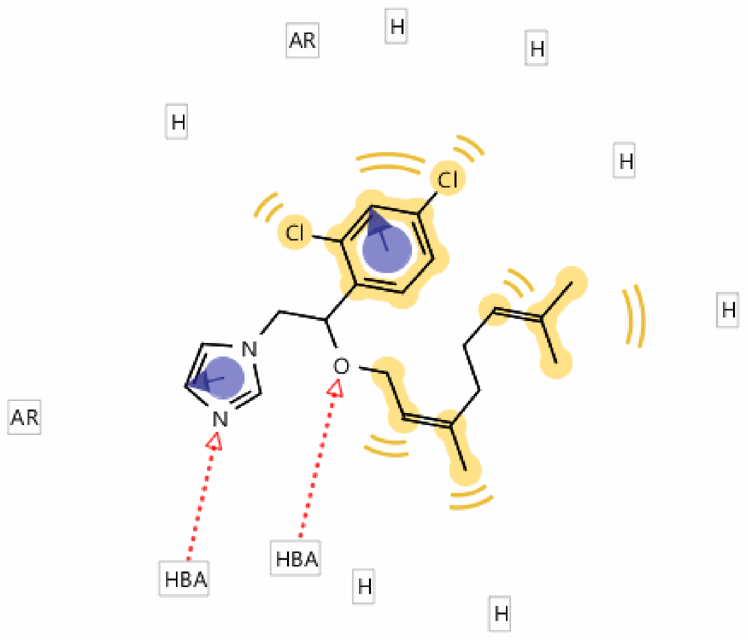
Aliconazole	63824-12-4	WNGFKUOERJDDIY-OQLLNIDSSA-N	InChI=1S/C18H13Cl3N2/c19-16-4-1-13(2-5-16)15(11-23-8-7-22-12-23)9-14-3-6-17(20)10-18(14)21/h1-10,12H,11H2/b15-9+	ClC1=CC=C(/C=C(CN2C=NC=C2)/C3=CC=C(Cl)C=C3)C(Cl)=C1	Sterol 14α-demethylase inhibitor	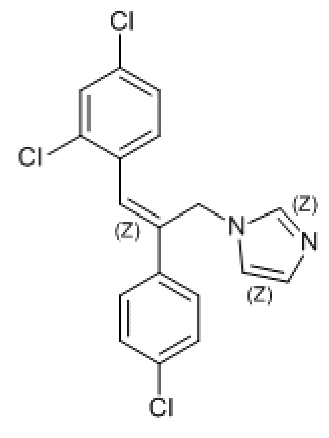	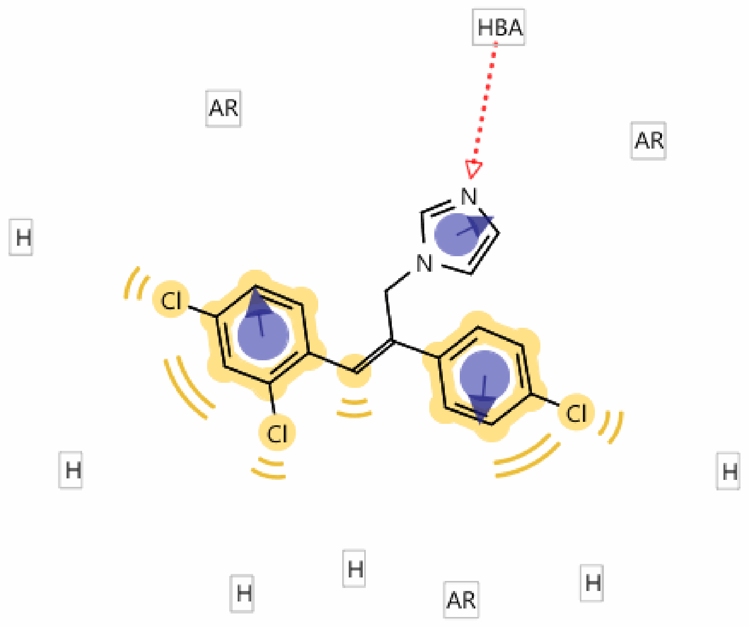

Common name	CAS Reg. No.	InChIKey	InChI	SMILES	Primary target	2D Structure	Pharmacophore[1]
Arasertaconazole	583057-48-1	JLGKQTAYUIMGRK-IBGZPJMESA-N	InChI=1S/C20H15Cl3N2OS/c21-14-4-5-16(18(23)8-14)19(9-25-7-6-24-12-25)26-10-13-11-27-20-15(13)2-1-3-17(20)22/h1-8,11-12,19H,9-10H2/t19-/m0/s1	ClC1=CC(Cl)=C(C=C1)[C@]([H])(CN2C=CN=C2)OCC3=CSC4=C3C=CC=C4Cl	Sterol 14α-demethylase inhibitor	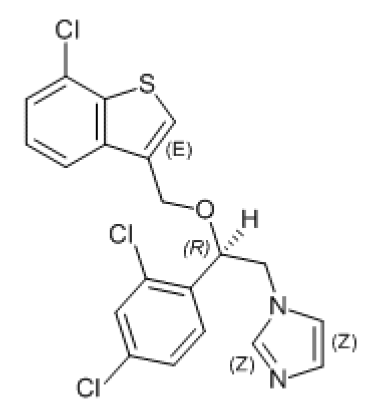	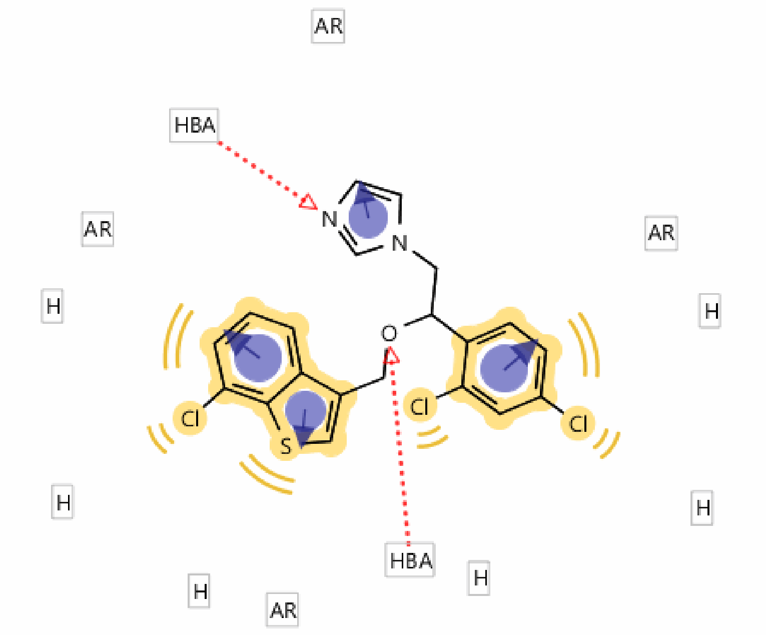
Azalanstat	143393-27-5	VYNIUBZKEWJOJP-UNMCSNQZSA-N	InChI=1S/C22H24ClN3O2S/c23-18-3-1-17(2-4-18)9-10-22(15-26-12-11-25-16-26)27-13-20(28-22)14-29-21-7-5-19(24)6-8-21/h1-8,11-12,16,20H,9-10,13-15,24H2/t20-,22-/m0/s1	ClC1=CC=C(CC[C@@]2(OC[C@H](O2)CSC3=CC=C(N)C=C3)CN4C=CN=C4)C=C1	Sterol 14α-demethylase inhibitor	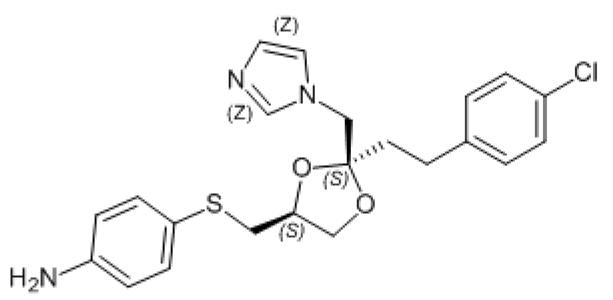	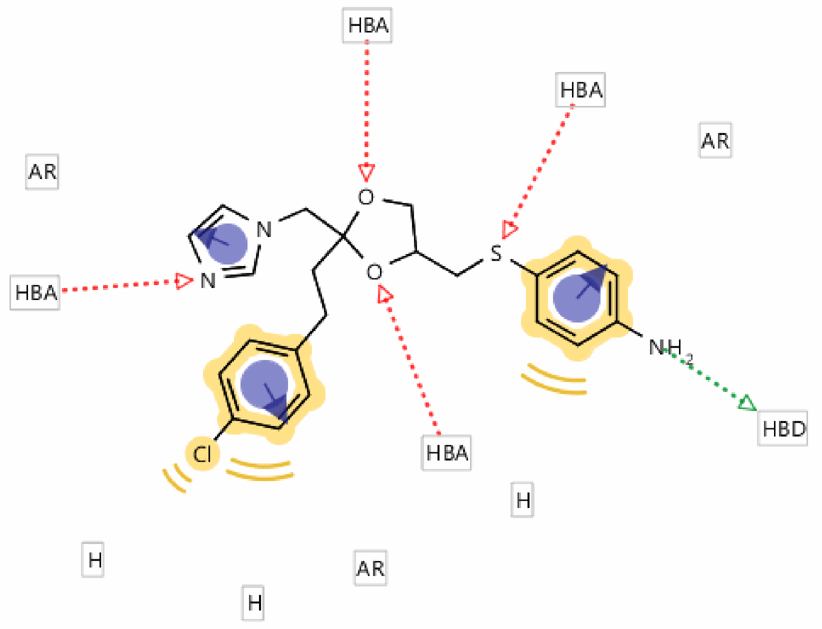
BAY C-9263	56290-29-0	QAGZXMQDYRBQLN-UHFFFAOYSA-N	InChI=1S/C18H16N2O2.ClH/c1-22-17(21)18(20-13-12-19-14-20,15-8-4-2-5-9-15)16-10-6-3-7-11-16;/h2-14H,1H3;1H	COC(C(N1C=CN=C1)(C2=CC=CC=C2)C3=CC=CC=C3)=O.Cl	Sterol 14α-demethylase inhibitor	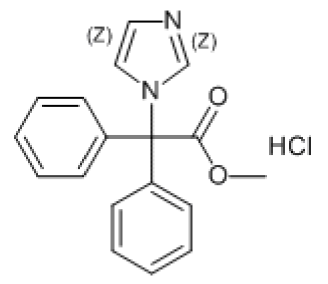	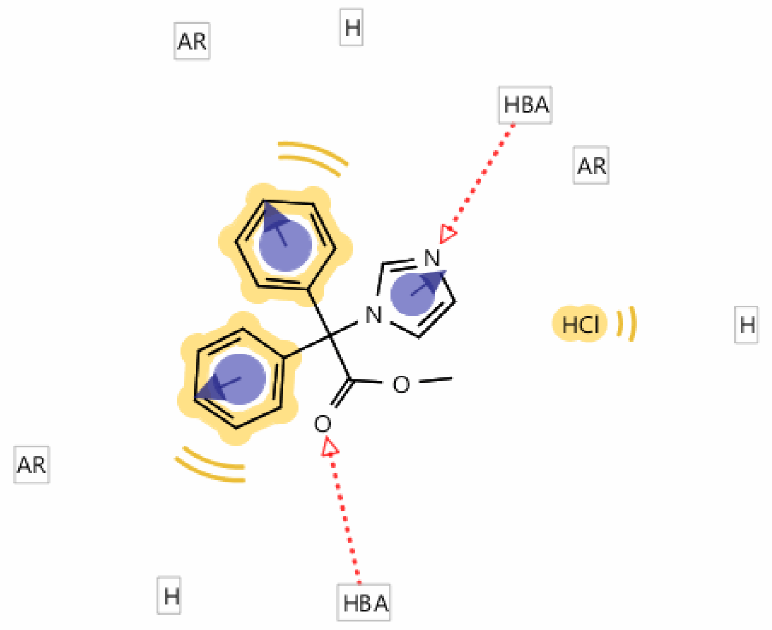
BAY D-9603	36698-20-1	MTXDFCPHZOIELM-UHFFFAOYSA-N	InChI=1S/C18H14N2/c1-2-18(20-14-13-19-15-20,16-9-5-3-6-10-16)17-11-7-4-8-12-17/h1,3-15H	C#CC(N1C=CN=C1)(C2=CC=CC=C2)C3=CC=CC=C3	Sterol 14α-demethylase inhibitor	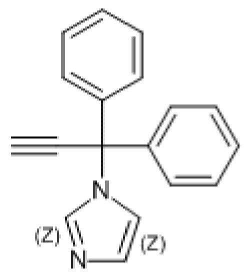	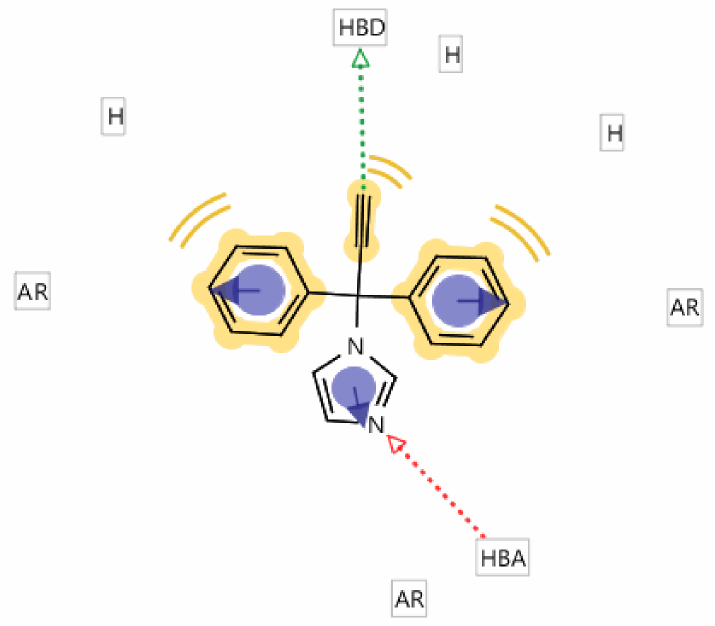
Becliconazole	112893-26-2	NZSHKJYRRPWFGN-JJMXQKMNSA-N	InChI=1S/2C18H12Cl2N2O/c2*19-13-5-6-16-12(9-13)10-17(23-16)18(22-8-7-21-11-22)14-3-1-2-4-15(14)20/h2*1-11,18H/t2*18-/m10/s1	ClC1=CC=C2OC([C@@]([H])(C3=CC=CC=C3Cl)N4C=CN=C4)=CC2=C1.ClC5=CC=C6OC([C@]([H])(C7=CC=CC=C7Cl)N8C=CN=C8)=CC6=C5	Sterol 14α-demethylase inhibitor	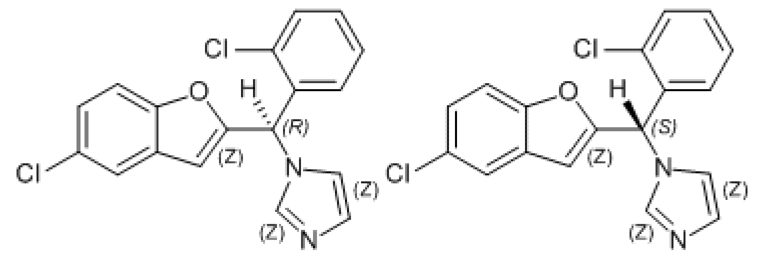	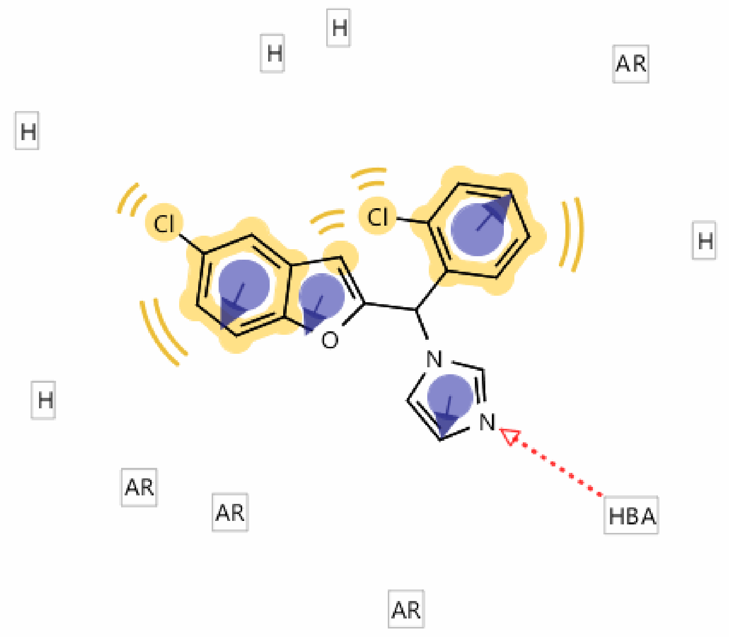
Bifonazole	60628-96-8	MNYJKGRCRFPXGL-IKXJNKEISA-N	InChI=1S/2C22H18N2/c2*1-3-7-18(8-4-1)19-11-13-21(14-12-19)22(24-16-15-23-17-24)20-9-5-2-6-10-20/h2*1-17,22H/t2*22-/m10/s1	[H][C@@](C1=CC=CC=C1)(C2=CC=C(C3=CC=CC=C3)C=C2)N4C=CN=C4.[H][C@](C5=CC=CC=C5)(C6=CC=C(C7=CC=CC=C7)C=C6)N8C=CN=C8	Sterol 14α-demethylase inhibitor	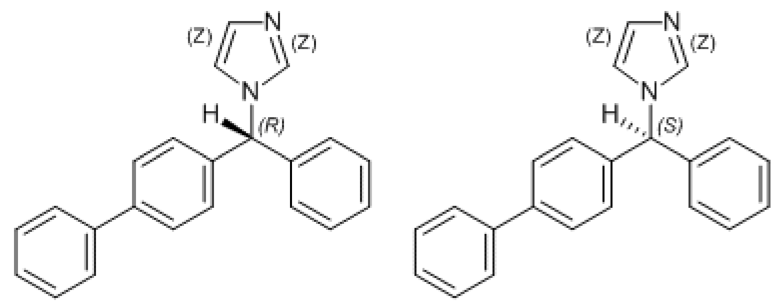	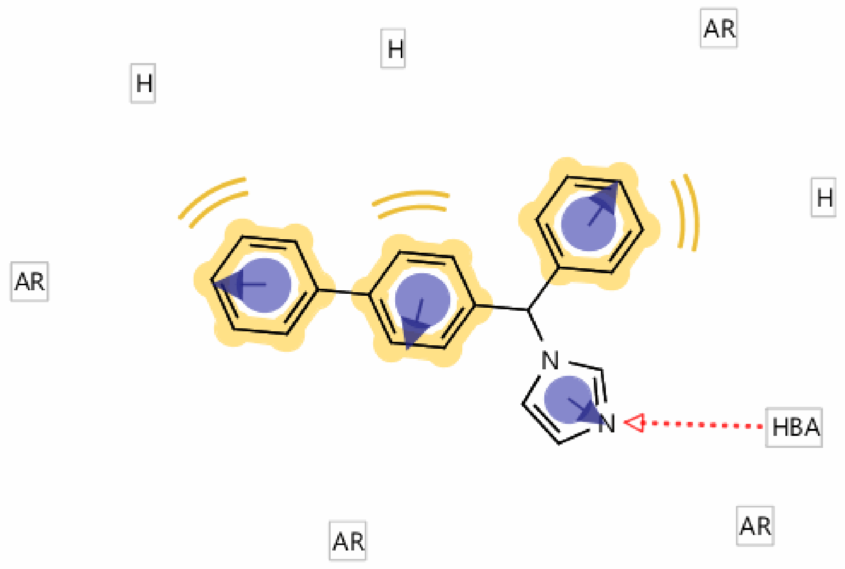

Common name	CAS Reg. No.	InChIKey	InChI	SMILES	Primary target	2D Structure	Pharmacophore[1]
Brolaconazole	108894-40-2	HPIJVBZJFDBDIB-QAOGLABXSA-N	InChI=1S/2C17H15BrN2/c2*18-16-8-6-15(7-9-16)17(12-20-11-10-19-13-20)14-4-2-1-3-5-14/h2*1-11,13,17H,12H2/t2*17-/m10/s1	BrC1=CC=C([C@](CN2C=CN=C2)(C3=CC=CC=C3)[H])C=C1.BrC4=CC=C([C@@](CN5C=CN=C5)(C6=CC=CC=C6)[H])C=C4	Sterol 14α-demethylase inhibitor	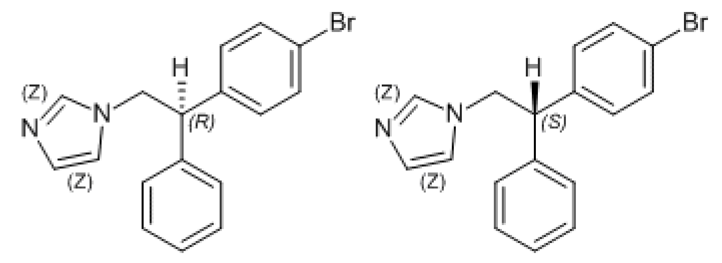	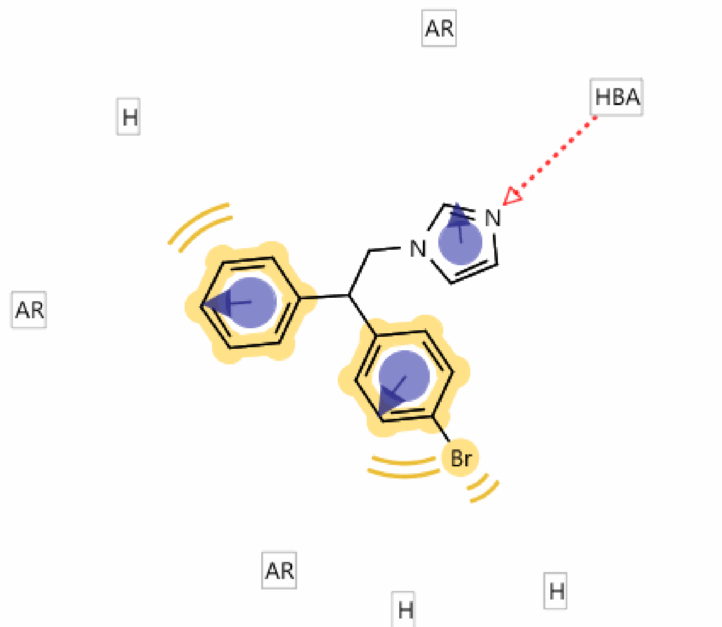
Butoconazole	64872-76-0	GFQXTUUCDYFXOL-RRHAQCGESA-N	InChI=1S/2C19H17Cl3N2S/c2*20-15-7-4-14(5-8-15)6-9-16(12-24-11-10-23-13-24)25-19-17(21)2-1-3-18(19)22/h2*1-5,7-8,10-11,13,16H,6,9,12H2/t2*16-/m10/s1	ClC1=CC=C(C=C1)CC[C@](SC2=C(C=CC=C2Cl)Cl)([H])CN3C=CN=C3.ClC4=CC=C(C=C4)CC[C@@](SC5=C(C=CC=C5Cl)Cl)([H])CN6C=CN=C6	Sterol 14α-demethylase inhibitor	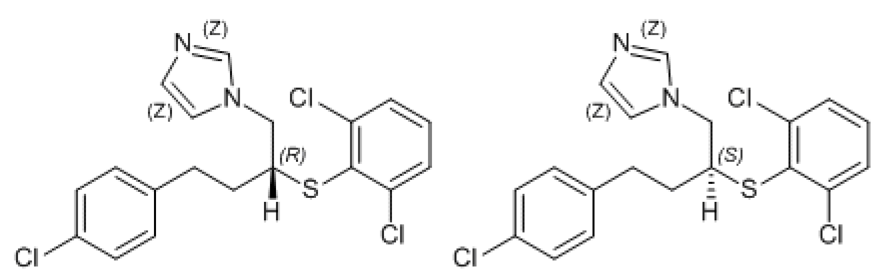	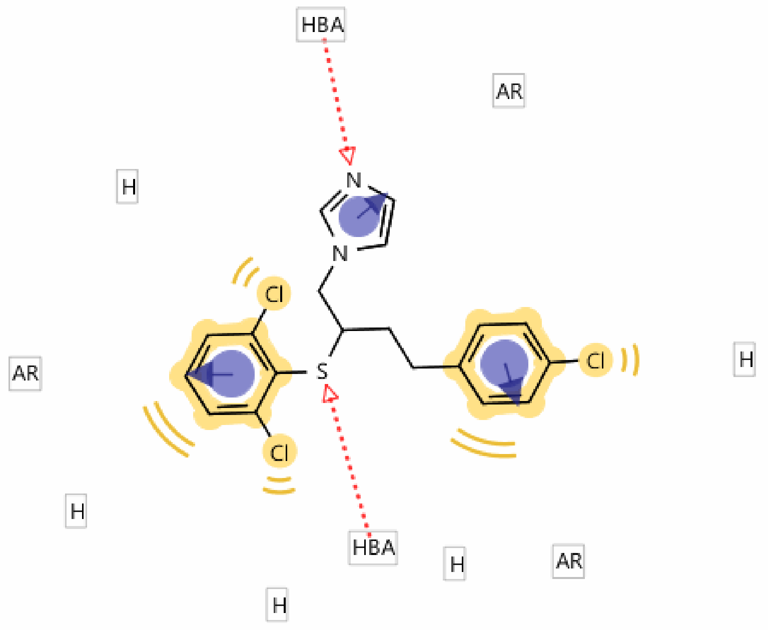
Cisconazole	104456-79-3	UEEKYUQRQVJODD-JQDMTEHVSA-N	InChI=1S/2C19H15F3N2OS/c2*20-12-4-5-17-13(8-12)19(18(26-17)9-24-7-6-23-11-24)25-10-14-15(21)2-1-3-16(14)22/h2*1-8,11,18-19H,9-10H2/t2*18-,19-/m10/s1	FC1=CC2=C(C=C1)S[C@H](CN3C=CN=C3)[C@@H]2OCC4=C(C=CC=C4F)F.FC5=CC6=C(C=C5)S[C@@H](CN7C=CN=C7)[C@H]6OCC8=C(C=CC=C8F)F	Sterol 14α-demethylase inhibitor	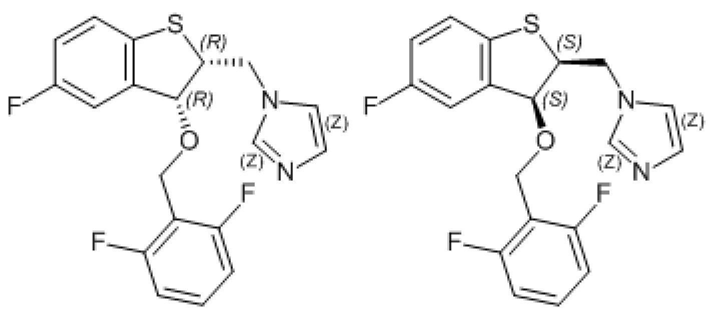	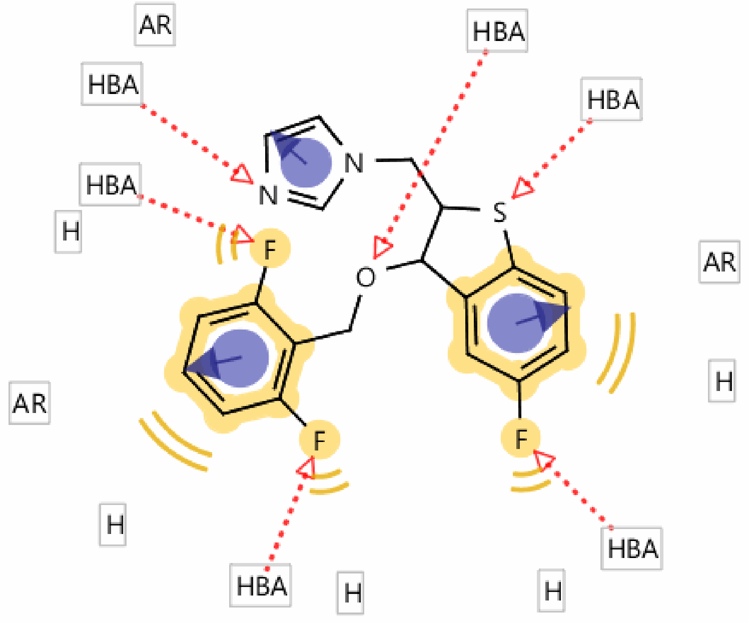
Climbazole	38083-17-9	DOGQEYHNANWDJU-IKFJUQJOSA-N	InChI=1S/2C15H17ClN2O2/c2*1-15(2,3)13(19)14(18-9-8-17-10-18)20-12-6-4-11(16)5-7-12/h2*4-10,14H,1-3H3/t2*14-/m10/s1	CC(C([C@]([H])(N1C=CN=C1)OC2=CC=C(C=C2)Cl)=O)(C)C.CC(C([C@@]([H])(N3C=CN=C3)OC4=CC=C(C=C4)Cl)=O)(C)C	Sterol 14α-demethylase inhibitor	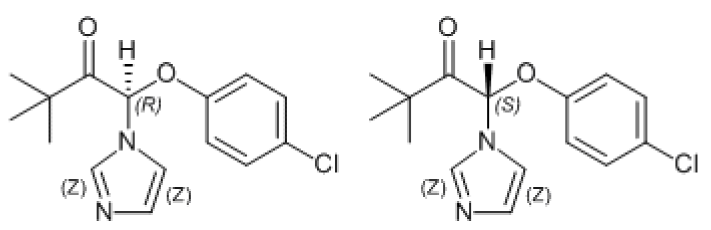	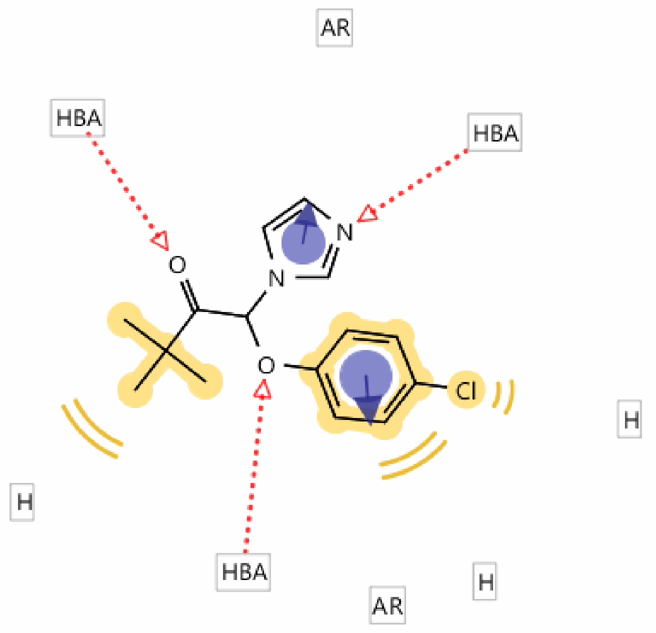
Clotrimazole	23593-75-1	VNFPBHJOKIVQEB-UHFFFAOYSA-N	InChI=1S/C22H17ClN2/c23-21-14-8-7-13-20(21)22(25-16-15-24-17-25,18-9-3-1-4-10-18)19-11-5-2-6-12-19/h1-17H	ClC1=CC=CC=C1C(N2C=CN=C2)(C3=CC=CC=C3)C4=CC=CC=C4	Sterol 14α-demethylase inhibitor	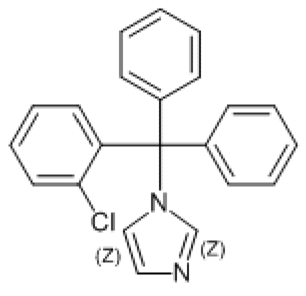	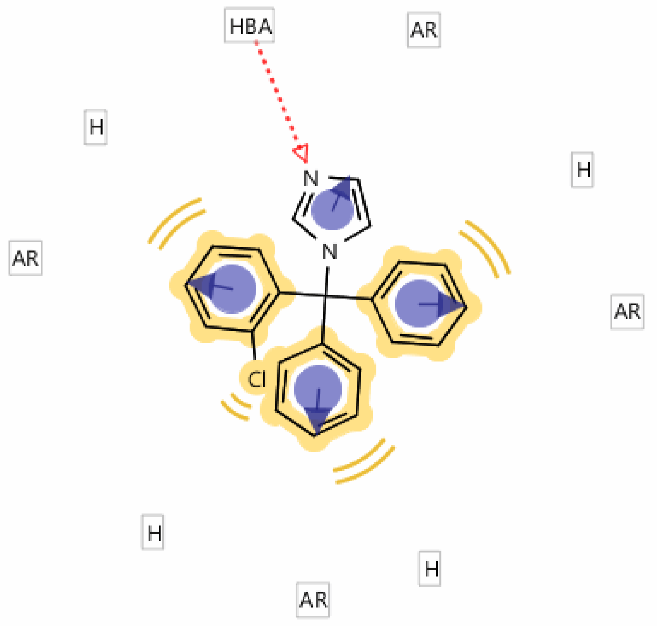
Croconazole	77175-51-0	WHPAGCJNPTUGGD-UHFFFAOYSA-N	InChI=1S/C18H15ClN2O/c1-14(21-10-9-20-13-21)17-7-2-3-8-18(17)22-12-15-5-4-6-16(19)11-15/h2-11,13H,1,12H2	C=C(C1=CC=CC=C1OCC2=CC(Cl)=CC=C2)N3C=CN=C3	Sterol 14α-demethylase inhibitor	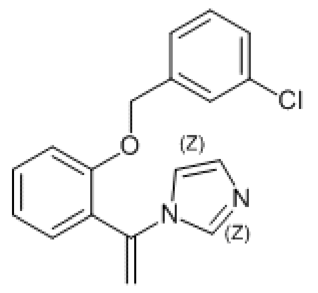	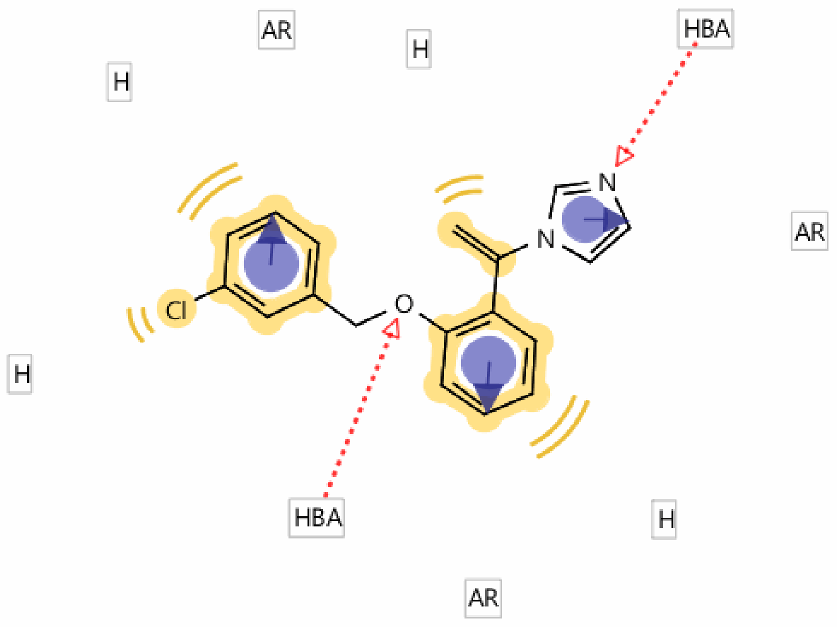

Common name	CAS Reg. No.	InChIKey	InChI	SMILES	Primary target	2D Structure	Pharmacophore[1]
Democonazole	70161-09-0	ABVFVJRTKMVJMV-XDHOZWIPSA-N	InChI=1S/C19H15Cl3N2O2/c20-14-1-4-16(5-2-14)25-9-10-26-19(12-24-8-7-23-13-24)17-6-3-15(21)11-18(17)22/h1-8,11-13H,9-10H2/b19-12+	ClC1=C(C=CC(Cl)=C1)/C(OCCOC2=CC=C(C=C2)Cl)=C\N3C=NC=C3	Sterol 14α-demethylase inhibitor	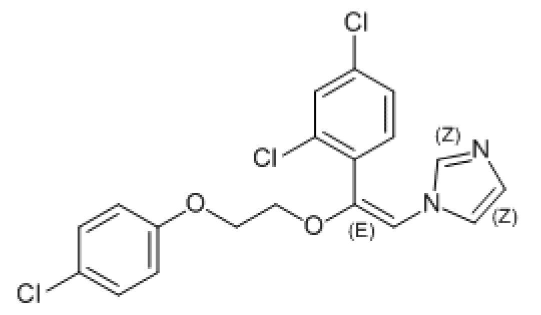	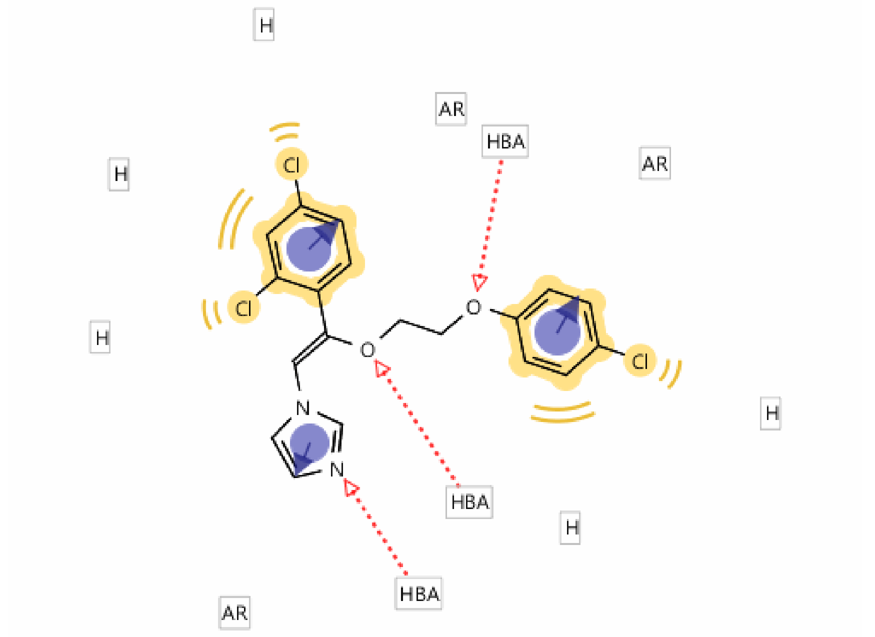
Dichlorophenyl imidazoldioxolan (elubiol)	67914-69-6	ZISRNZMDAFGHDW-BTOYNABYSA-N	InChI=1S/2C27H30Cl2N4O5/c2*1-2-35-26(34)33-13-11-32(12-14-33)21-4-6-22(7-5-21)36-16-23-17-37-27(38-23,18-31-10-9-30-19-31)24-8-3-20(28)15-25(24)29/h2*3-10,15,19,23H,2,11-14,16-18H2,1H3/t2*23-,27-/m10/s1	CCOC(N1CCN(CC1)C2=CC=C(C=C2)OC[C@H]3CO[C@](CN4C=NC=C4)(C5=C(C=C(C=C5)Cl)Cl)O3)=O.CCOC(N6CCN(CC6)C7=CC=C(C=C7)OC[C@@H]8CO[C@@](CN9C=NC=C9)(C%10=C(C=C(C=C%10)Cl)Cl)O8)=O	Sterol 14α-demethylase inhibitor	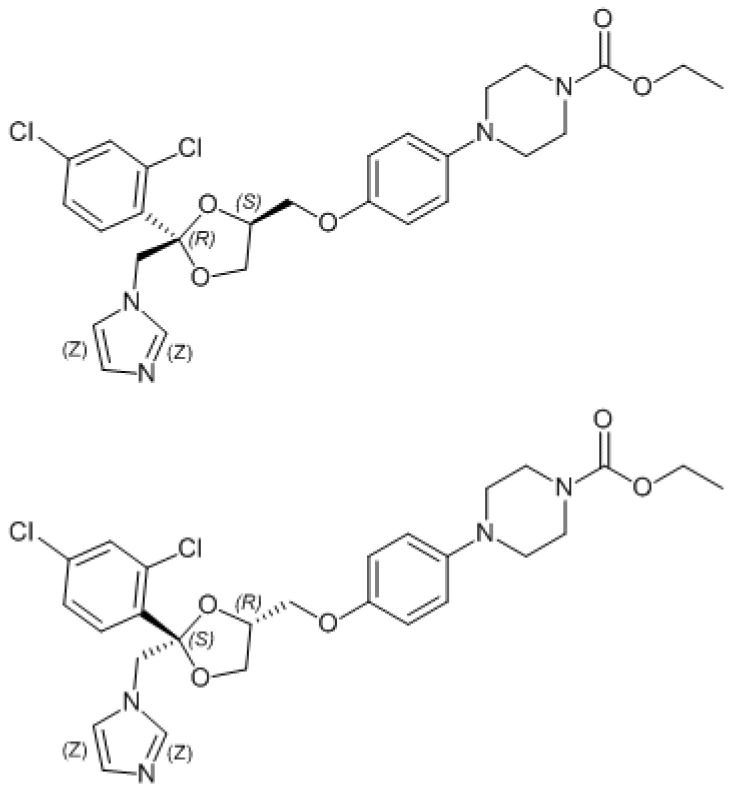	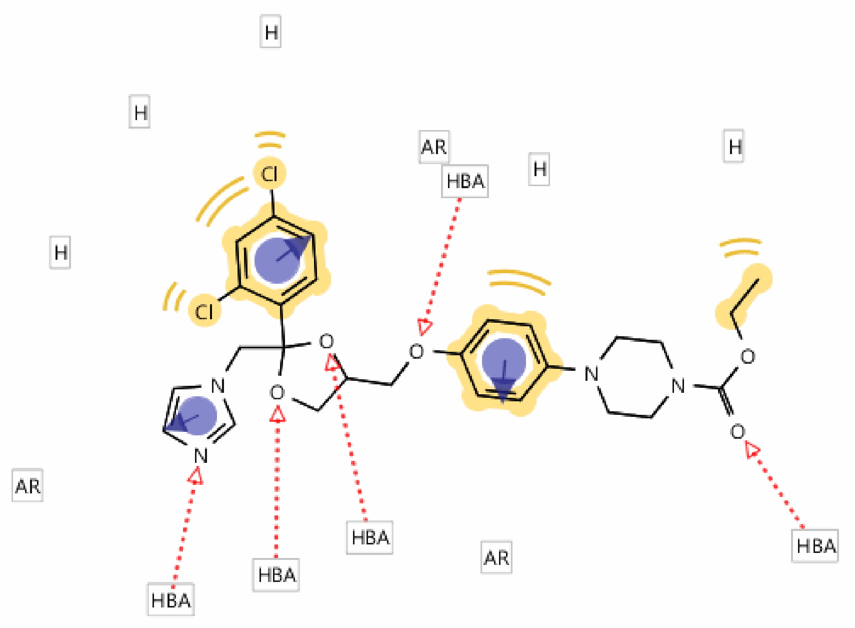
Doconazole	59831-63-9	UXSJEKCVVMAKOL-ICCBHQQHSA-N	InChI=1S/2C26H22Cl2N2O3/c2*27-21-8-11-24(25(28)14-21)26(17-30-13-12-29-18-30)32-16-23(33-26)15-31-22-9-6-20(7-10-22)19-4-2-1-3-5-19/h2*1-14,18,23H,15-17H2/t2*23-,26-/m10/s1	ClC1=CC(Cl)=C([C@@]2(OC[C@H](O2)COC3=CC=C(C4=CC=CC=C4)C=C3)CN5C=CN=C5)C=C1.ClC6=CC(Cl)=C([C@]7(OC[C@@H](O7)COC8=CC=C(C9=CC=CC=C9)C=C8)CN%10C=CN=C%10)C=C6	Sterol 14α-demethylase inhibitor	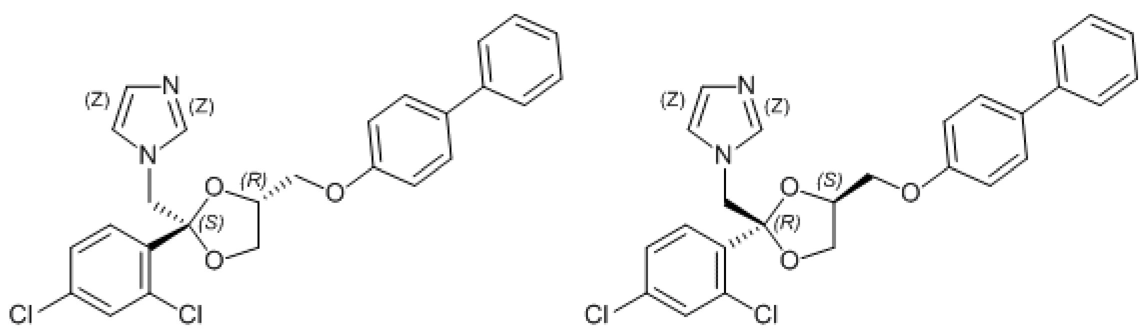	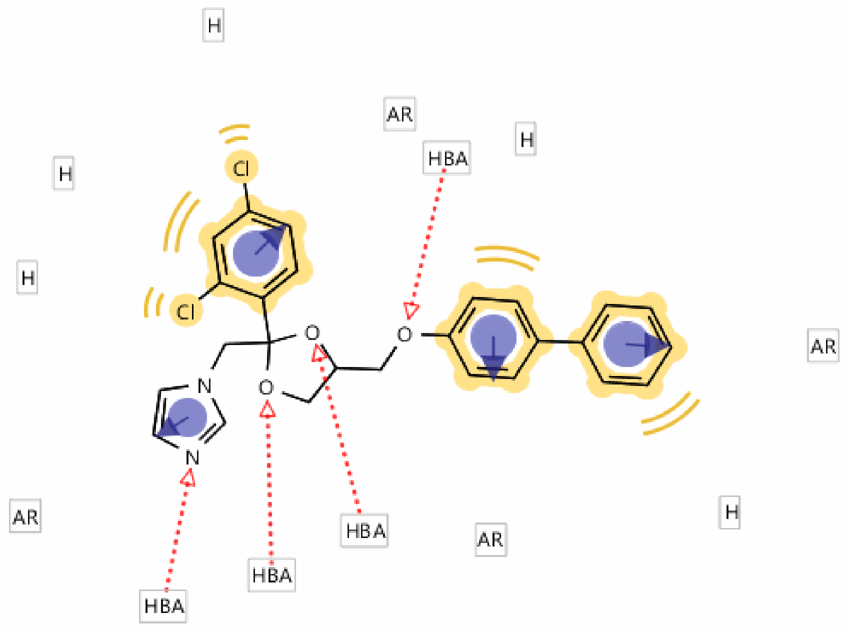
Eberconazole	128326-82-9	YPJVLGCHLGMCIW-JJMXQKMNSA-N	InChI=1S/2C18H14Cl2N2/c2*19-14-9-13-6-5-12-3-1-2-4-15(12)18(17(13)16(20)10-14)22-8-7-21-11-22/h2*1-4,7-11,18H,5-6H2/t2*18-/m10/s1	ClC1=CC(Cl)=C2C(CCC3=CC=CC=C3[C@H]2N4C=CN=C4)=C1.ClC5=CC(Cl)=C6C(CCC7=CC=CC=C7[C@@H]6N8C=CN=C8)=C5	Sterol 14α-demethylase inhibitor	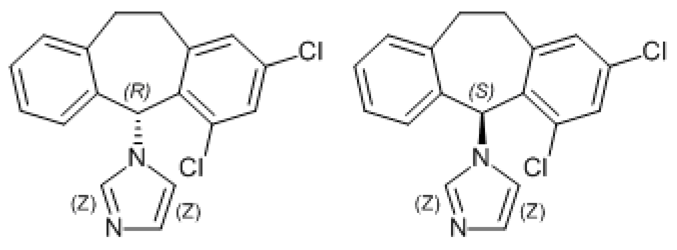	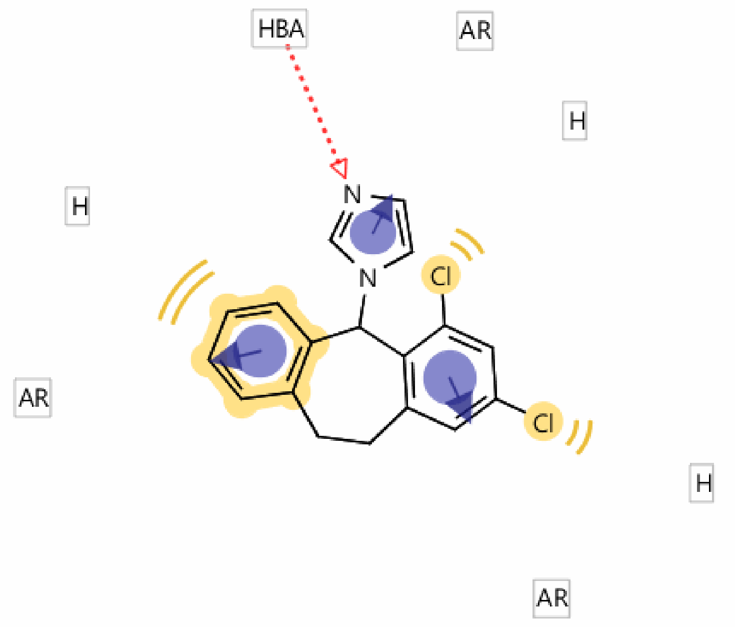
Econazole	27220-47-9	XPKMQAONTXFFGN-JJMXQKMNSA-N	InChI=1S/2C18H15Cl3N2O/c2*19-14-3-1-13(2-4-14)11-24-18(10-23-8-7-22-12-23)16-6-5-15(20)9-17(16)21/h2*1-9,12,18H,10-11H2/t2*18-/m10/s1	ClC1=CC=C(C=C1)CO[C@]([H])(CN2C=CN=C2)C3=C(C=C(C=C3)Cl)Cl.ClC4=CC=C(C=C4)CO[C@@]([H])(CN5C=CN=C5)C6=C(C=C(C=C6)Cl)Cl	Sterol 14α-demethylase inhibitor	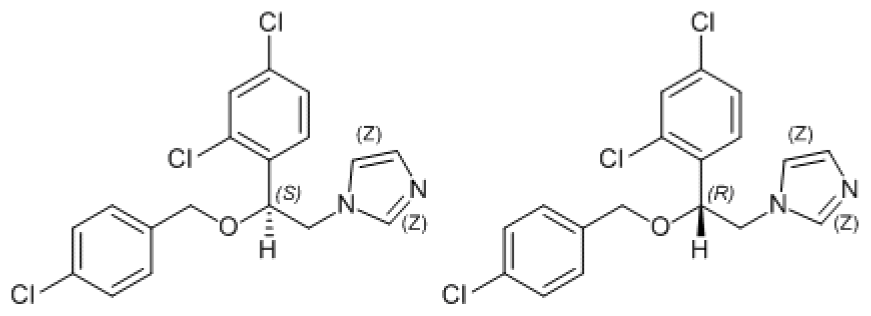	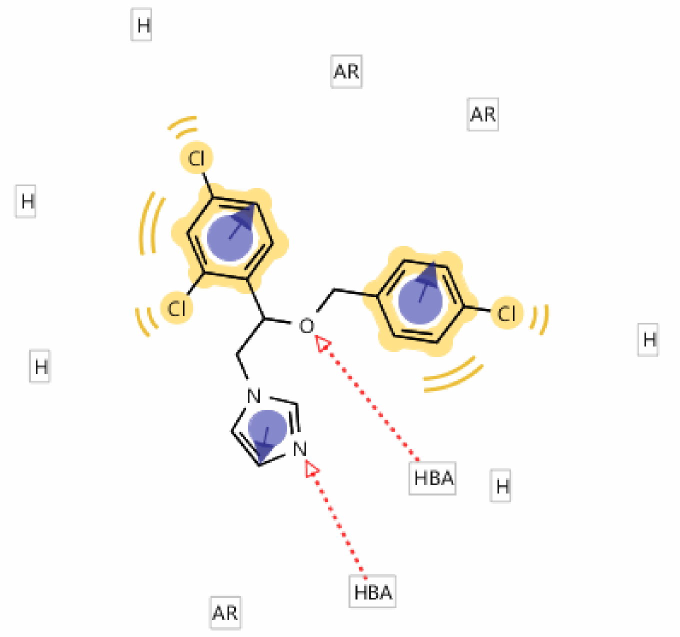

Common name	CAS Reg. No.	InChIKey	InChI	SMILES	Primary target	2D Structure	Pharmacophore[1]
Fenapanil	61019-78-1	RIELFAOFGOMSQE-RRHAQCGESA-N	InChI=1S/2C16H19N3/c2*1-2-3-9-16(12-17,13-19-11-10-18-14-19)15-7-5-4-6-8-15/h2*4-8,10-11,14H,2-3,9,13H2,1H3/t2*16-/m10/s1	CCCC[C@@](C1=CC=CC=C1)(C#N)CN2C=CN=C2.CCCC[C@](C3=CC=CC=C3)(C#N)CN4C=CN=C4	Sterol 14α-demethylase inhibitor	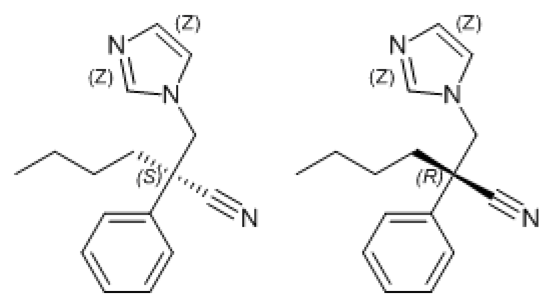	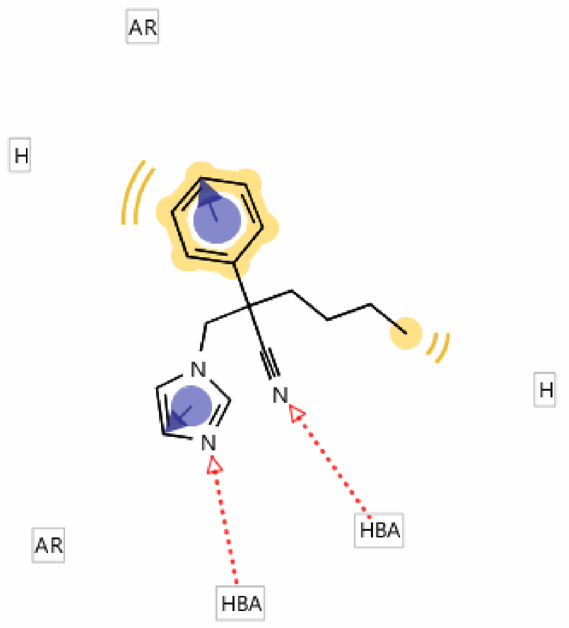
Fenticonazole	72479-26-6	BVAMTFZPMBAWRF-STCDVQFJSA-N	InChI=1S/2C24H20Cl2N2OS/c2*25-19-8-11-22(23(26)14-19)24(15-28-13-12-27-17-28)29-16-18-6-9-21(10-7-18)30-20-4-2-1-3-5-20/h2*1-14,17,24H,15-16H2/t2*24-/m10/s1	ClC1=CC(Cl)=C(C=C1)[C@@]([H])(CN2C=CN=C2)OCC3=CC=C(C=C3)SC4=CC=CC=C4.ClC5=CC(Cl)=C(C=C5)[C@]([H])(CN6C=CN=C6)OCC7=CC=C(C=C7)SC8=CC=CC=C8	Sterol 14α-demethylase inhibitor	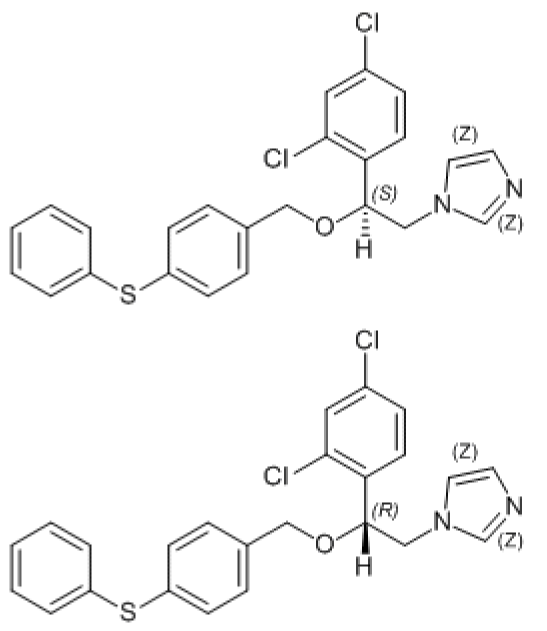	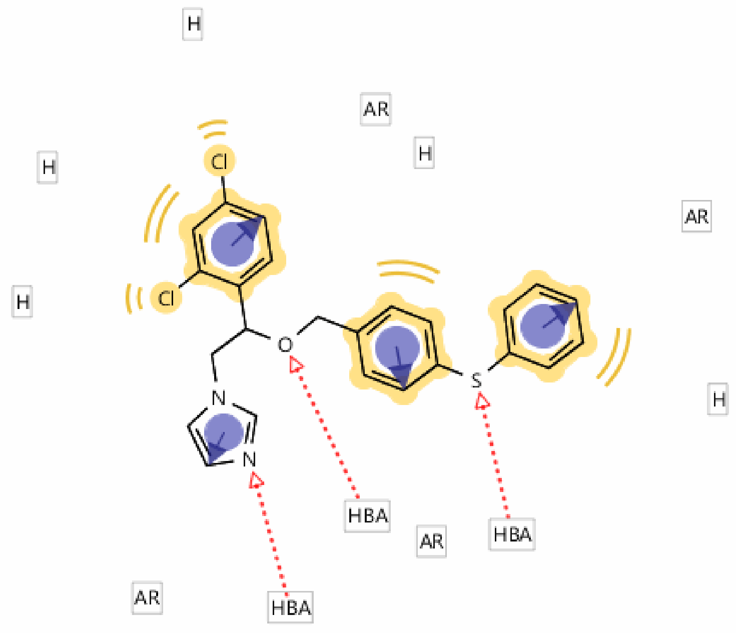
Flutrimazole	119006-77-8	WNVBMMUIAABPGP-IKXJNKEISA-N	InChI=1S/2C22H16F2N2/c2*23-19-12-10-18(11-13-19)22(26-15-14-25-16-26,17-6-2-1-3-7-17)20-8-4-5-9-21(20)24/h2*1-16H/t2*22-/m10/s1	FC1=CC=C(C=C1)[C@](N2C=CN=C2)(C3=CC=CC=C3)C4=CC=CC=C4F.FC5=CC=C(C=C5)[C@@](N6C=CN=C6)(C7=CC=CC=C7)C8=CC=CC=C8F	Sterol 14α-demethylase inhibitor	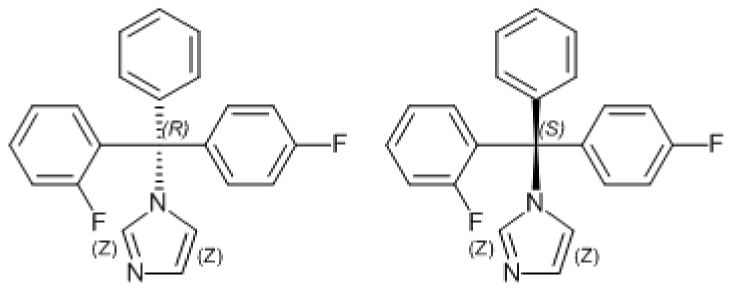	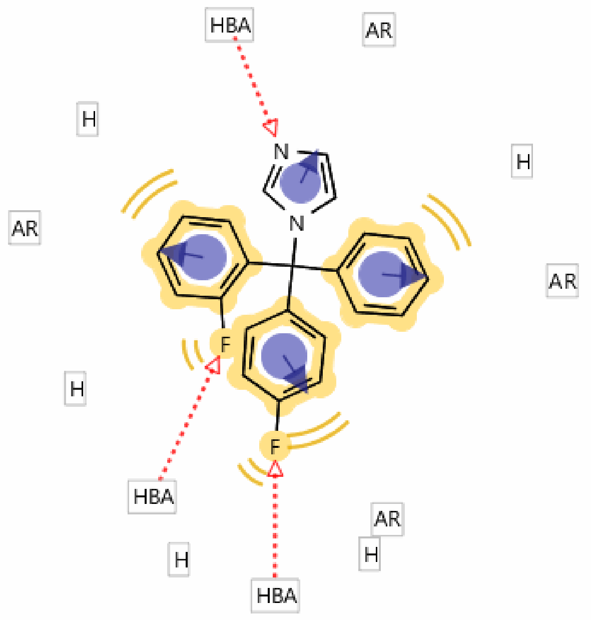
Imazalil (enilconazole)	35554-44-0	BZKXPDQNGYLUSH-IKFJUQJOSA-N	InChI=1S/2C14H14Cl2N2O/c2*1-2-7-19-14(9-18-6-5-17-10-18)12-4-3-11(15)8-13(12)16/h2*2-6,8,10,14H,1,7,9H2/t2*14-/m10/s1	C=CCO[C@]([H])(CN1C=CN=C1)C2=C(C=C(C=C2)Cl)Cl.C=CCO[C@@]([H])(CN3C=CN=C3)C4=C(C=C(C=C4)Cl)Cl	Sterol 14α-demethylase inhibitor	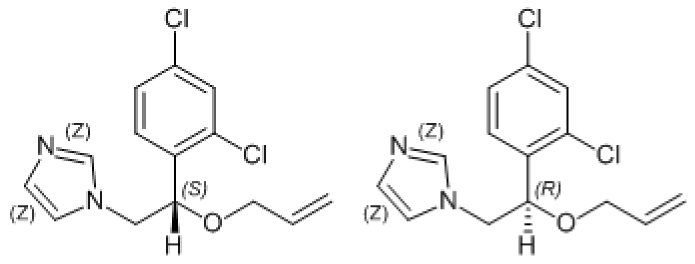	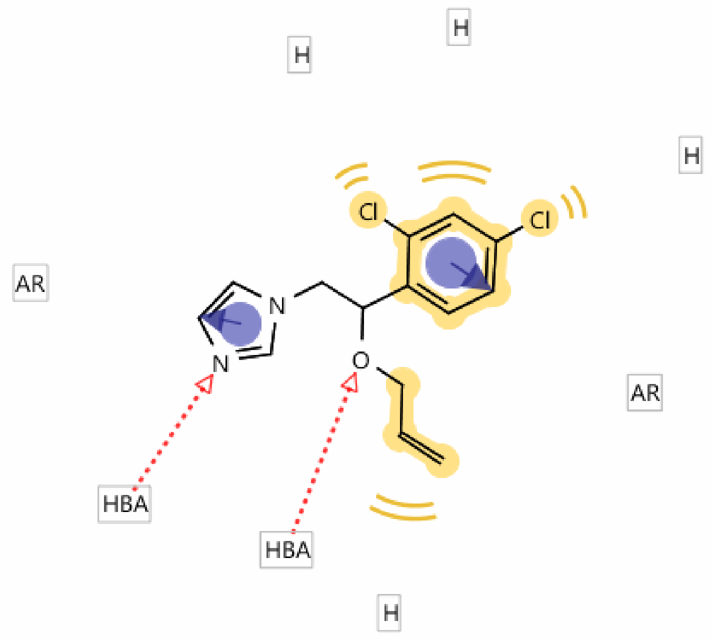
Isoconazole	27523-40-6	RIOHVOBBAZPSRG-JJMXQKMNSA-N	InChI=1S/2C18H14Cl4N2O/c2*19-12-4-5-13(17(22)8-12)18(9-24-7-6-23-11-24)25-10-14-15(20)2-1-3-16(14)21/h2*1-8,11,18H,9-10H2/t2*18-/m10/s1	ClC1=CC(Cl)=C(C=C1)[C@@]([H])(CN2C=CN=C2)OCC3=C(C=CC=C3Cl)Cl.ClC4=CC(Cl)=C(C=C4)[C@]([H])(CN5C=CN=C5)OCC6=C(C=CC=C6Cl)Cl	Sterol 14α-demethylase inhibitor	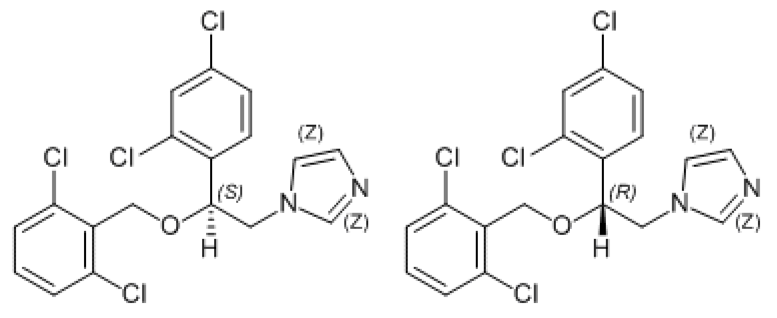	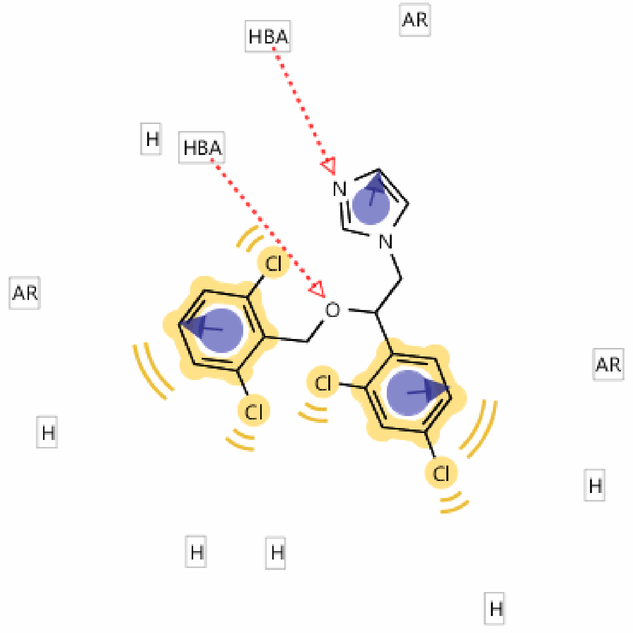
Ketaminazole		OXONPOTUQMJCNH-ICCBHQQHSA-N	InChI=1S/2C26H29Cl2N5O3/c2*1-19(34)32-10-12-33(13-11-32)22-5-3-21(4-6-22)30-15-23-16-35-26(36-23,17-31-9-8-29-18-31)24-7-2-20(27)14-25(24)28/h2*2-9,14,18,23,30H,10-13,15-17H2,1H3/t2*23-,26-/m10/s1	CC(N1CCN(C2=CC=C(NC[C@H]3O[C@@](CN4C=CN=C4)(OC3)C5=C(Cl)C=C(Cl)C=C5)C=C2)CC1)=O.CC(N6CCN(C7=CC=C(NC[C@@H]8O[C@](CN9C=CN=C9)(OC8)C%10=C(Cl)C=C(Cl)C=C%10)C=C7)CC6)=O	Sterol 14α-demethylase inhibitor	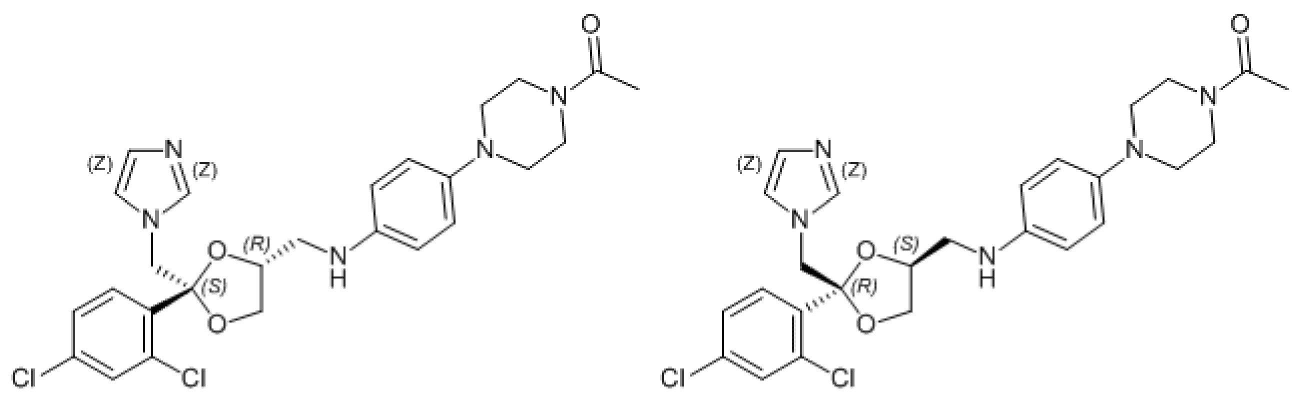	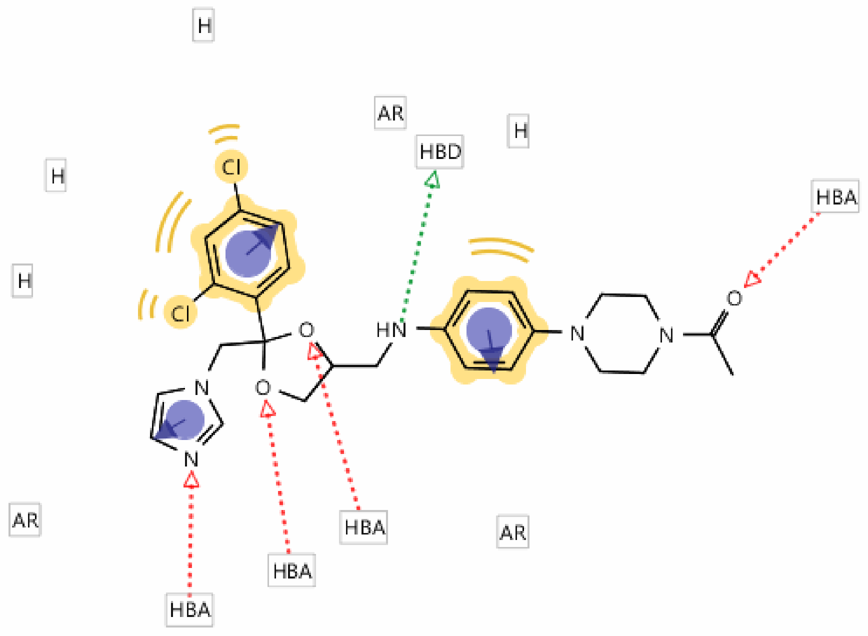

Common name	CAS Reg. No.	InChIKey	InChI	SMILES	Primary target	2D Structure	Pharmacophore[1]
Ketoconazole	65277-42-1	RPECMBIVPHZCSC-ICCBHQQHSA-N	InChI=1S/2C26H28Cl2N4O4/c2*1-19(33)31-10-12-32(13-11-31)21-3-5-22(6-4-21)34-15-23-16-35-26(36-23,17-30-9-8-29-18-30)24-7-2-20(27)14-25(24)28/h2*2-9,14,18,23H,10-13,15-17H2,1H3/t2*23-,26-/m10/s1	CC(N1CCN(CC1)C2=CC=C(C(C=C2)OC[C@H]3O[C@@](C4=C(C=C(C=C4)Cl)Cl)(CN5C=CN=C5)OC3)=O.CC(N6CCN(CC6)C7=CC=C(C=C7)OC[C@@H]8O[C@](C9=C(C=C(C=C9)Cl)Cl)(CN%10C=CN=C%10)OC8)=O	Sterol 14α-demethylase inhibitor	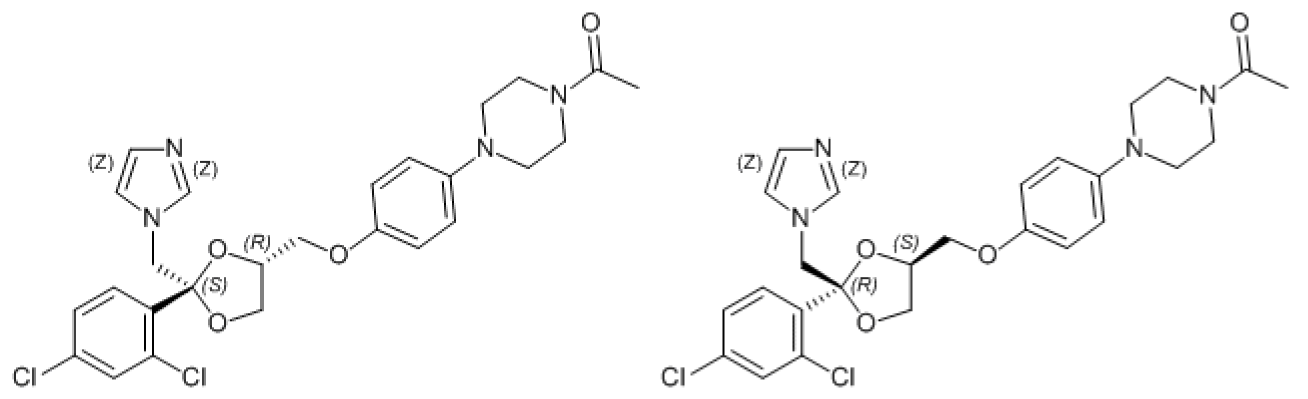	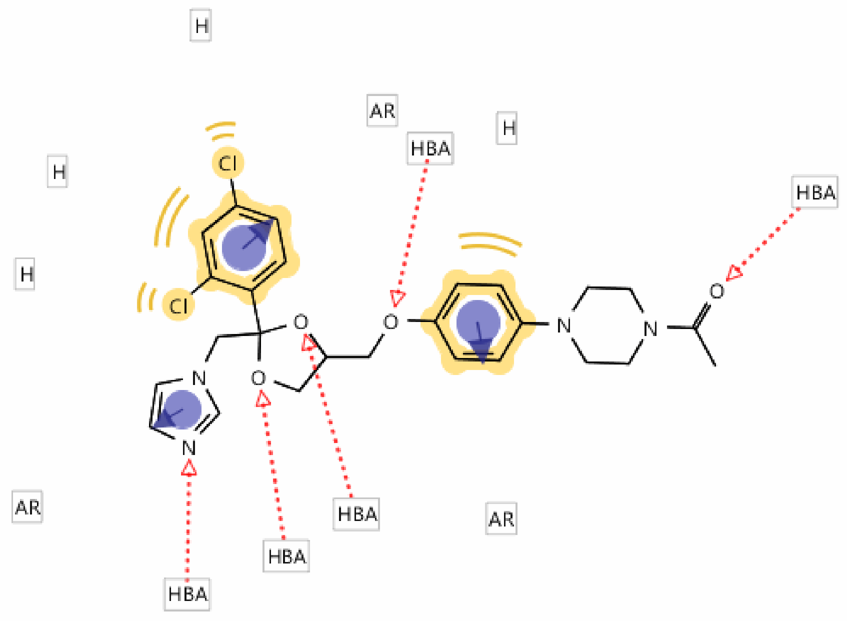
Lanoconazole	101530-10-3	WQFFFCGJNONTPF-QGXXPBGRSA-N	InChI=1S/2C14H10ClN3S2/c2*15-11-4-2-1-3-10(11)13-8-19-14(20-13)12(7-16)18-6-5-17-9-18/h2*1-6,9,13H,8H2/b2*14-12+/t2*13-/m10/s1	ClC1=CC=CC=C1[C@@H](CS/2)SC2=C(C#N)\N3C=CN=C3.ClC4=CC=CC=C4[C@H](CS/5)SC5=C(C#N)\N6C=CN=C6	Sterol 14α-demethylase inhibitor	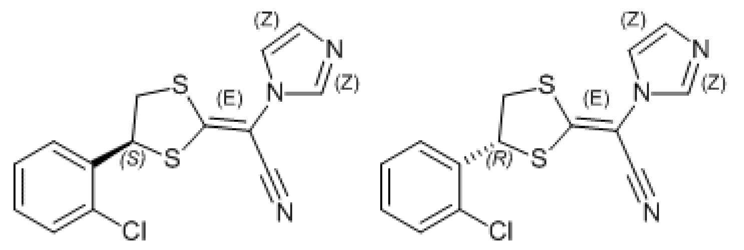	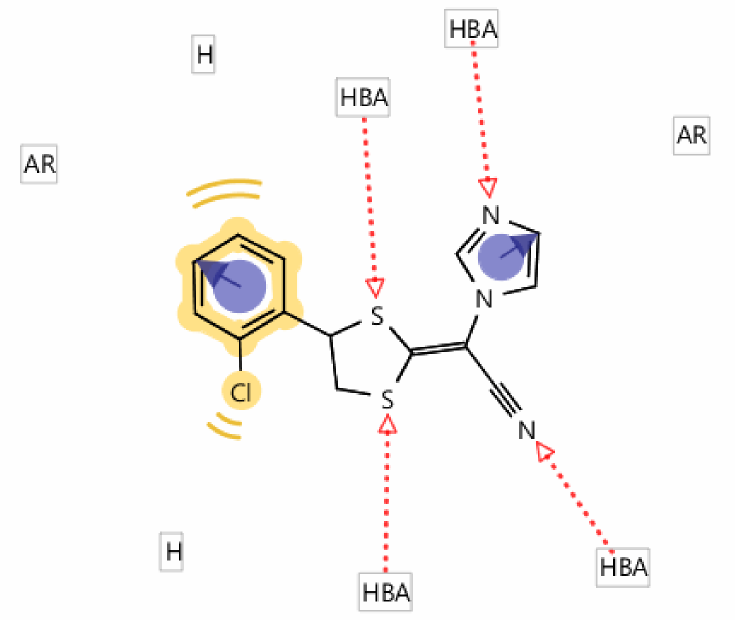
Lombazole	60628-98-0	QSDKVULGCBWAJP-IKXJNKEISA-N	InChI=1S/2C22H17ClN2/c2*23-21-9-5-4-8-20(21)22(25-15-14-24-16-25)19-12-10-18(11-13-19)17-6-2-1-3-7-17/h2*1-16,22H/t2*22-/m10/s1	ClC1=CC=CC=C1[C@@](N2C=CN=C2)([H])C3=CC=C(C=C3)C4=CC=CC=C4.ClC5=CC=CC=C5[C@](N6C=CN=C6)([H])C7=CC=C(C=C7)C8=CC=CC=C8	Sterol 14α-demethylase inhibitor	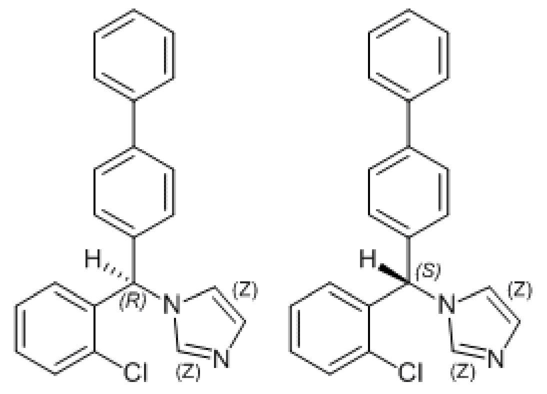	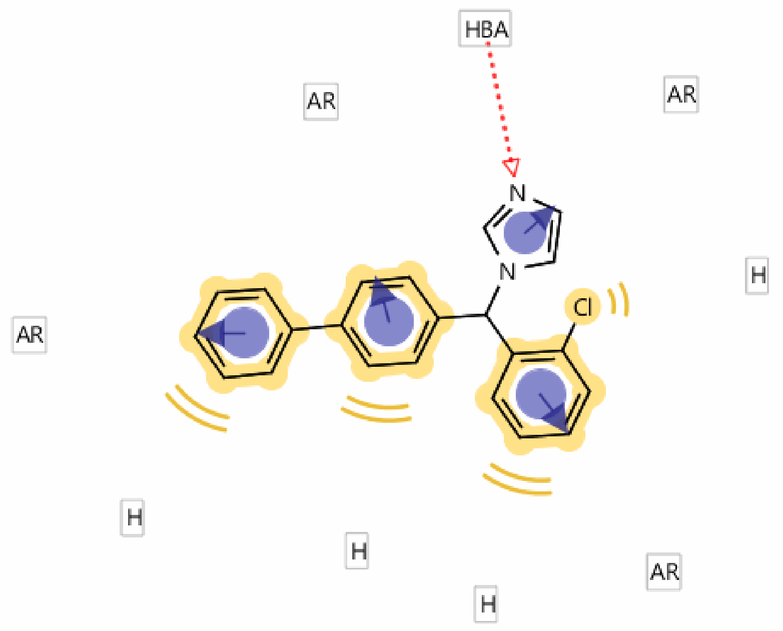
Luliconazole	187164-19-8	YTAOBBFIOAEMLL-REQDGWNSSA-N	InChI=1S/C14H9Cl2N3S2/c15-9-1-2-10(11(16)5-9)13-7-20-14(21-13)12(6-17)19-4-3-18-8-19/h1-5,8,13H,7H2/b14-12+/t13-/m0/s1	ClC1=CC(Cl)=C(C=C1)[C@H](CS/2)SC2=C(C#N)\N3C=CN=C3	Sterol 14α-demethylase inhibitor	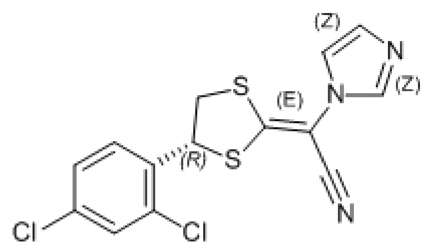	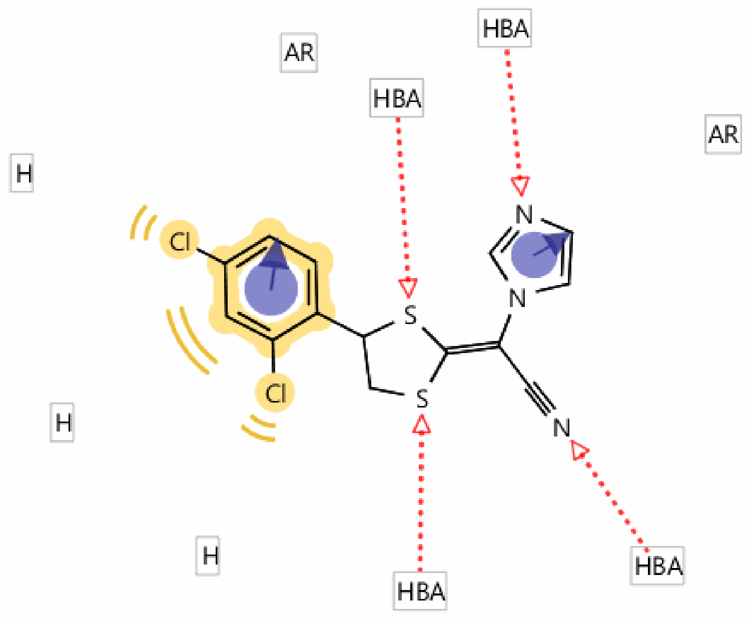
MH 0685	105688-63-9	DDSQEQZRLZZOJR-UHFFFAOYSA-N	InChI=1S/C16H13ClN2OS/c1-12(19-9-8-18-11-19)14-4-2-3-5-15(14)20-10-13-6-7-16(17)21-13/h2-9,11H,1,10H2	C=C(N1C=CN=C1)C2=CC=CC=C2OCC3=CC=C(Cl)S3	Sterol 14α-demethylase inhibitor	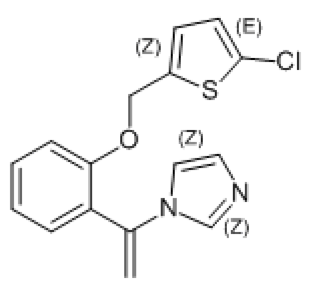	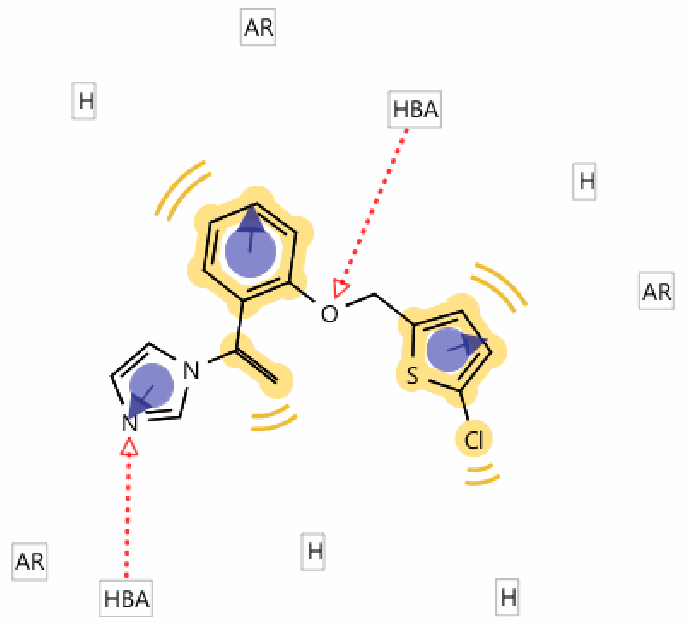
Miconazole	22916-47-8	APCOSEGPTCWPHP-JJMXQKMNSA-N	InChI=1S/2C18H14Cl4N2O/c2*19-13-2-1-12(16(21)7-13)10-25-18(9-24-6-5-23-11-24)15-4-3-14(20)8-17(15)22/h2*1-8,11,18H,9-10H2/t2*18-/m10/s1	ClC1=CC(Cl)=C(C=C1)CO[C@]([H])(CN2C=CN=C2)C3=C(C=C(C=C3)Cl)Cl.ClC4=CC(Cl)=C(C=C4)CO[C@@]([H])(CN5C=CN=C5)C6=C(C=C(C=C6)Cl)Cl	Sterol 14α-demethylase inhibitor	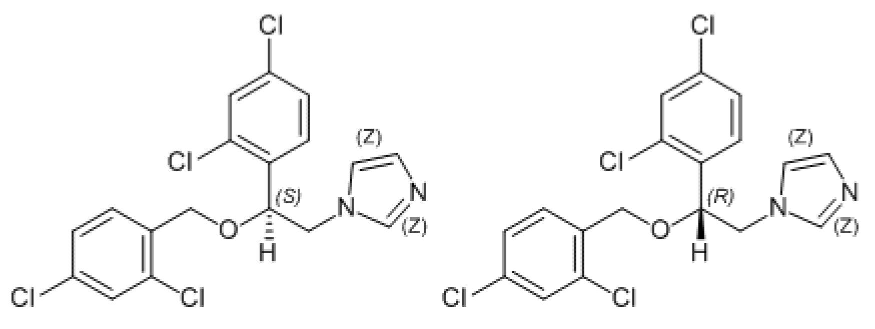	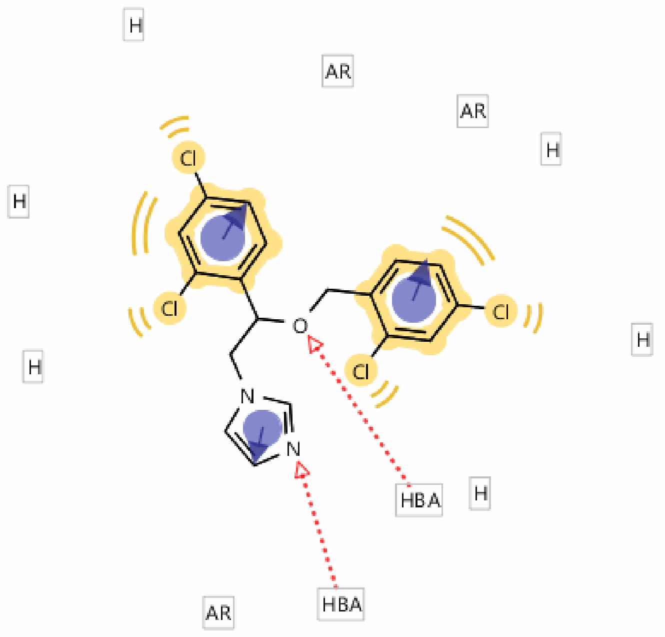

Common name	CAS Reg. No.	InChIKey	InChI	SMILES	Primary target	2D Structure	Pharmacophore[1]
Neticonazole	130726-68-0	VWOIKFDZQQLJBJ-DTQAZKPQSA-N	InChI=1S/C17H22N2OS/c1-3-4-7-12-20-17-9-6-5-8-15(17)16(13-21-2)19-11-10-18-14-19/h5-6,8-11,13-14H,3-4,7,12H2,1-2H3/b16-13+	CCCCCOC1=CC=CC=C1/C(N2C=NC=C2)=C\SC	Sterol 14α-demethylase inhibitor	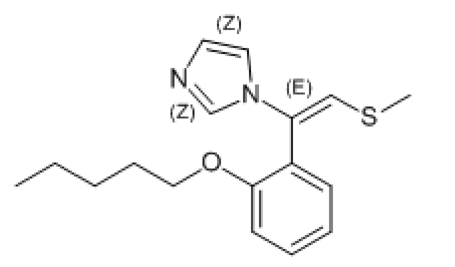	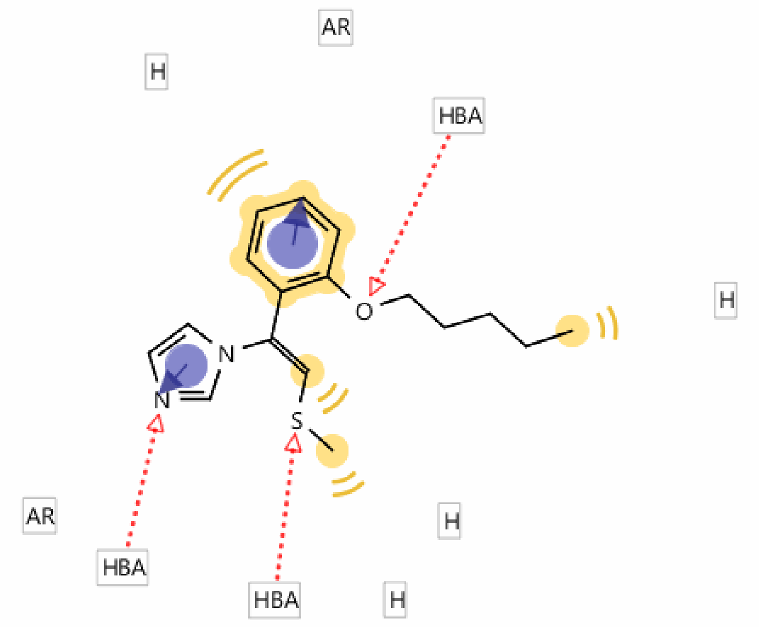
OK 8705	56764-91-9	ZTPSVWPAOCNTDE-ZWZQDMJTSA-N	InChI=1S/2C17H22N2O4/c2*1-17(2,3)15(23-16(20)19-10-9-18-12-19)11-22-14-7-5-13(21-4)6-8-14/h2*5-10,12,15H,11H2,1-4H3/t2*15-/m10/s1	CC([C@@]([H])(COC1=CC=C(C=C1)OC)OC(N2C=CN=C2)=O)(C)C.CC([C@]([H])(COC3=CC=C(C=C3)OC)OC(N4C=CN=C4)=O)(C)C	Sterol 14α-demethylase inhibitor	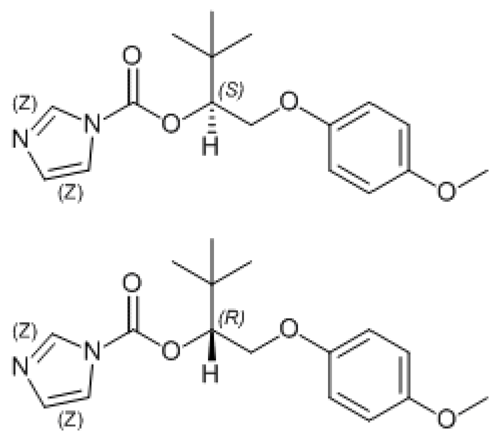	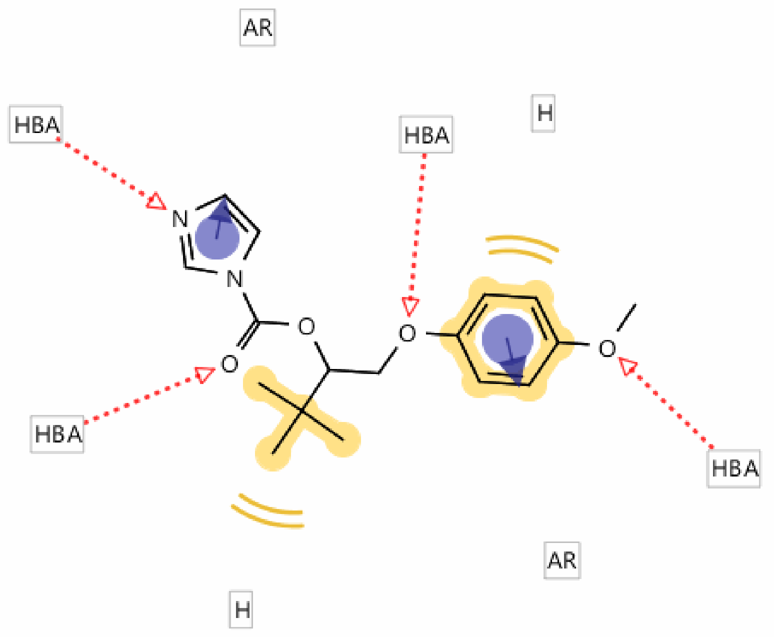
OK 8801	156764-92-0	OWTBSSUBJUMAAH-ZWZQDMJTSA-N	InChI=1S/2C17H22N2O3S/c2*1-17(2,3)15(22-16(23)19-10-9-18-12-19)11-21-14-7-5-13(20-4)6-8-14/h2*5-10,12,15H,11H2,1-4H3/t2*15-/m10/s1	CC(C)([C@@](OC(N1C=CN=C1)=S)([H])COC2=CC=C(C=C2)OC)C.CC(C)([C@](OC(N3C=CN=C3)=S)([H])COC4=CC=C(C=C4)OC)C	Sterol 14α-demethylase inhibitor	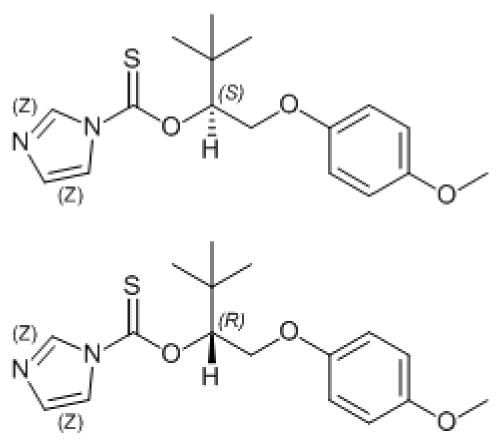	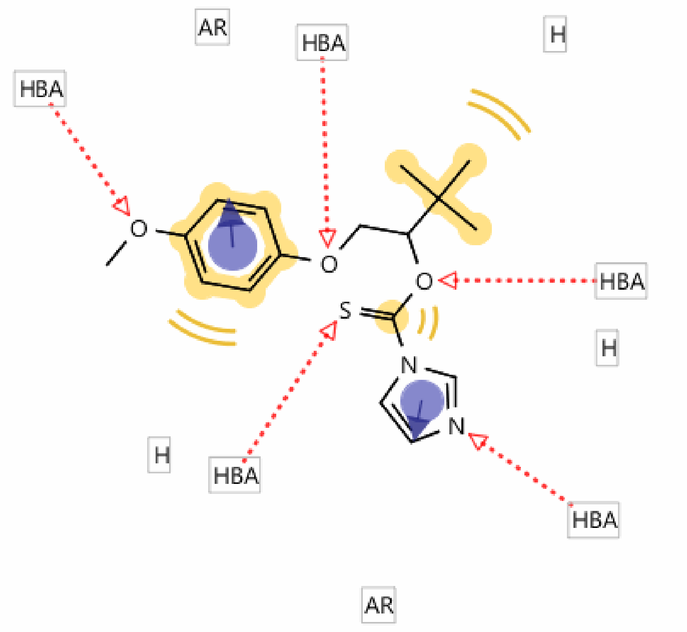
Omoconazole	74512-12-2	JMFOSJNGKJCTMJ-ZHZULCJRSA-N	InChI=1S/C20H17Cl3N2O2/c1-14(25-9-8-24-13-25)20(18-7-4-16(22)12-19(18)23)27-11-10-26-17-5-2-15(21)3-6-17/h2-9,12-13H,10-11H2,1H3/b20-14-	C/C(N1C=NC=C1)=C(C2=C(C=C(C=C2)Cl)Cl)/OCCOC3=CC=C(Cl)C=C3	Sterol 14α-demethylase inhibitor	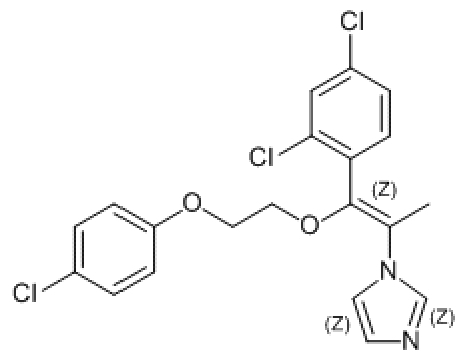	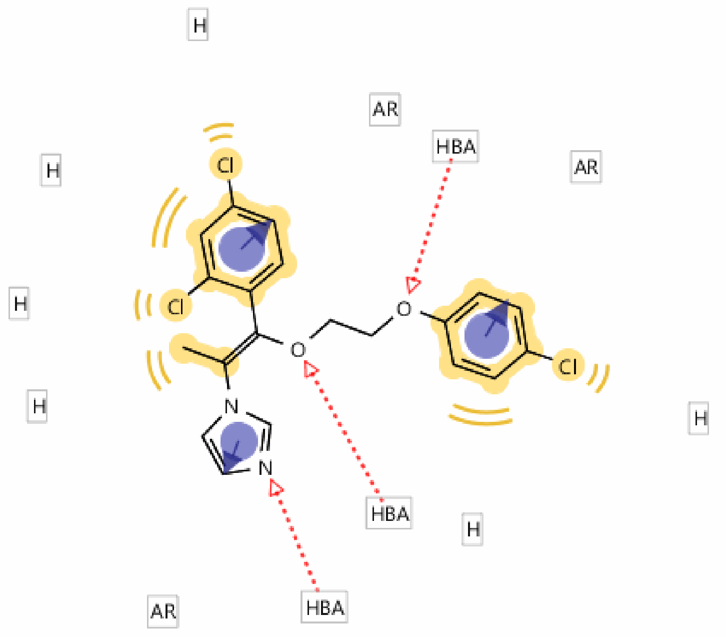
Orconazole	66778-37-8	ZBBFBKFWRSNBEF-JJMXQKMNSA-N	InChI=1S/2C18H15Cl3N2O/c2*19-14-6-4-13(5-7-14)18(10-23-9-8-22-12-23)24-11-15-16(20)2-1-3-17(15)21/h2*1-9,12,18H,10-11H2/t2*18-/m10/s1	ClC1=CC=C(C=C1)[C@@]([H])(CN2C=CN=C2)OCC3=C(C=CC=C3Cl)Cl.ClC4=CC=C(C=C4)[C@]([H])(CN5C=CN=C5)OCC6=C(C=CC=C6Cl)Cl	Sterol 14α-demethylase inhibitor	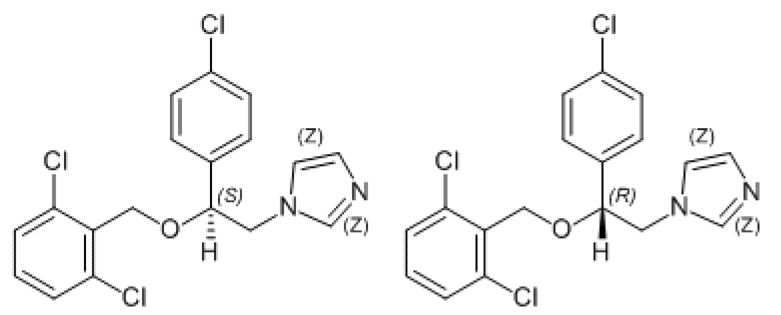	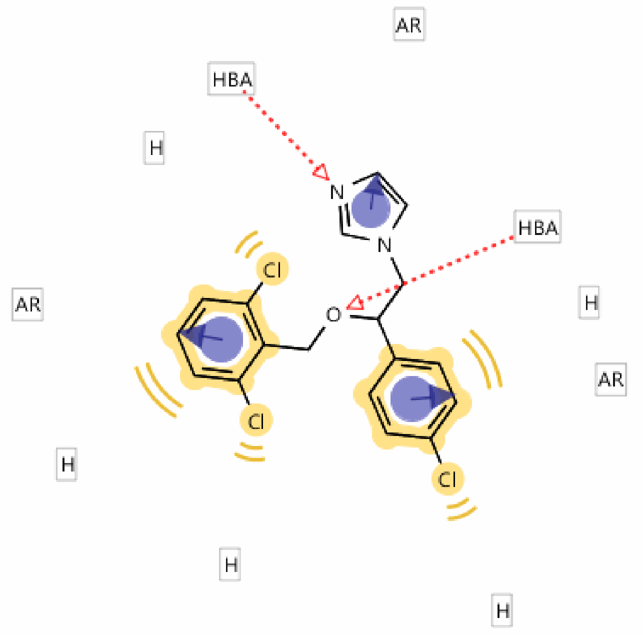

Common name	CAS Reg. No.	InChIKey	InChI	SMILES	Primary target	2D Structure	Pharmacophore[1]
Oxiconazole	64211-45-6	QRJJEGAJXVEBNE-HKOYGPOVSA-N	InChI=1S/C18H13Cl4N3O/c19-13-2-1-12(16(21)7-13)10-26-24-18(9-25-6-5-23-11-25)15-4-3-14(20)8-17(15)22/h1-8,11H,9-10H2/b24-18+	ClC1=CC(Cl)=C(C=C1)CO/N=C(CN2C=CN=C2)/C3=C(C=C(C=C3)Cl)Cl	Sterol 14α-demethylase inhibitor	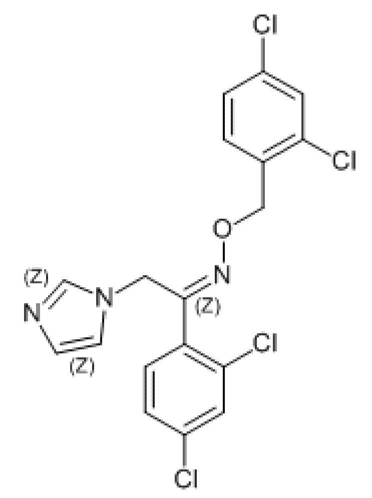	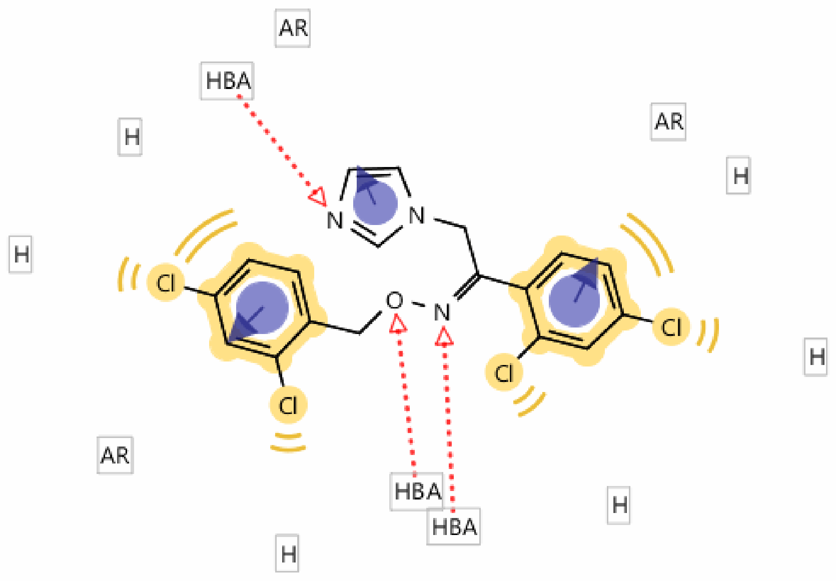
Oxpoconazole	134074-64-9	BOLPVRVYVDAPEO-OYPHMNEHSA-N	InChI=1S/2C19H24ClN3O2/c2*1-18(2)13-25-19(3,23(18)17(24)22-12-11-21-14-22)10-4-5-15-6-8-16(20)9-7-15/h2*6-9,11-12,14H,4-5,10,13H2,1-3H3/t2*19-/m10/s1	CC(C)(CO[C@@]1(CCCC2=CC=C(Cl)C=C2)C)N1C(N3C=CN=C3)=O.CC(C)(CO[C@]4(CCCC5=CC=C(Cl)C=C5)C)N4C(N6C=CN=C6)=O	Sterol 14α-demethylase inhibitor	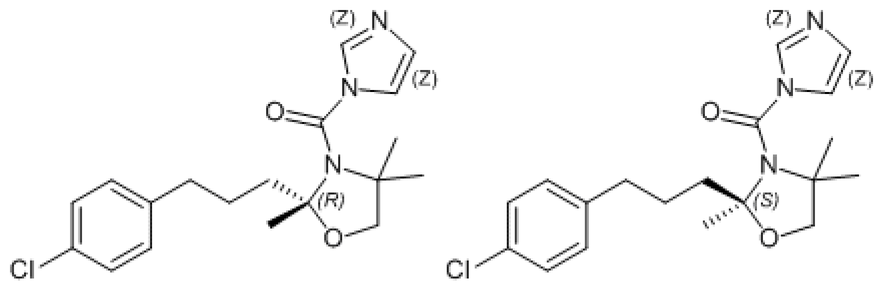	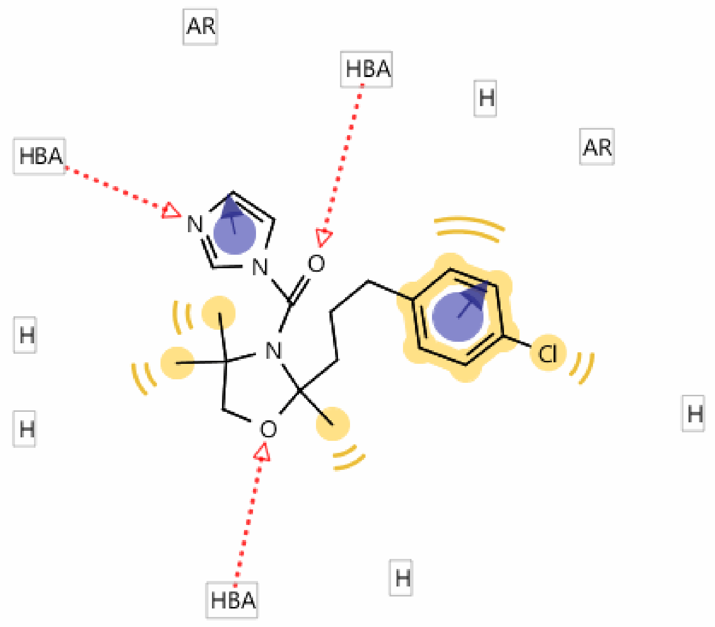
Parconazole	61400-59-7	NLLNYRPYKMKFKS-QMGGJZHNSA-N	InChI=1S/2C17H16Cl2N2O3/c2*1-2-7-22-9-14-10-23-17(24-14,11-21-6-5-20-12-21)15-4-3-13(18)8-16(15)19/h2*1,3-6,8,12,14H,7,9-11H2/t2*14-,17-/m10/s1	C#CCOC[C@H]1O[C@@](C2=C(C=C(C=C2)Cl)Cl)(CN3C=CN=C3)OC1.C#CCOC[C@@H]4O[C@](C5=C(C=C(C=C5)Cl)Cl)(CN6C=CN=C6)OC4	Sterol 14α-demethylase inhibitor	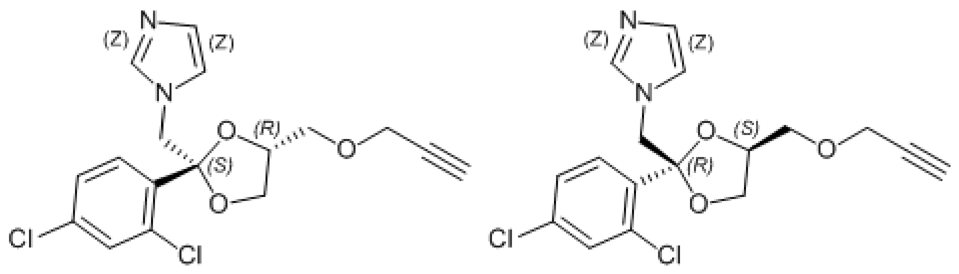	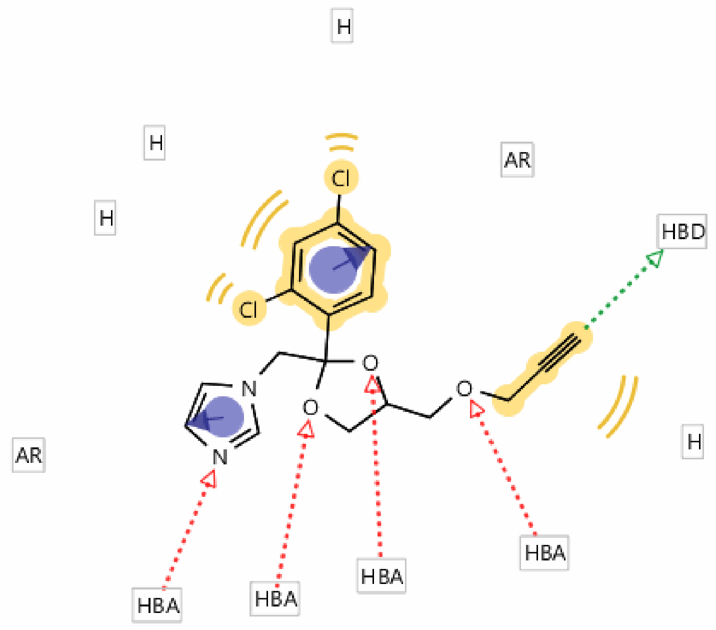
Pefurazoate	101903-30-4	WWWXSKRXDKHXKA-RRHAQCGESA-N	InChI=1S/2C18H23N3O4/c2*1-3-5-6-11-25-17(22)16(4-2)21(13-15-8-7-12-24-15)18(23)20-10-9-19-14-20/h2*3,7-10,12,14,16H,1,4-6,11,13H2,2H3/t2*16-/m10/s1	C=CCCCOC([C@@](CC)(N(C(N1C=CN=C1)=O)CC2=CC=CO2)[H])=O.C=CCCCOC([C@](CC)(N(C(N3C=CN=C3)=O)CC4=CC=CO4)[H])=O	Sterol 14α-demethylase inhibitor	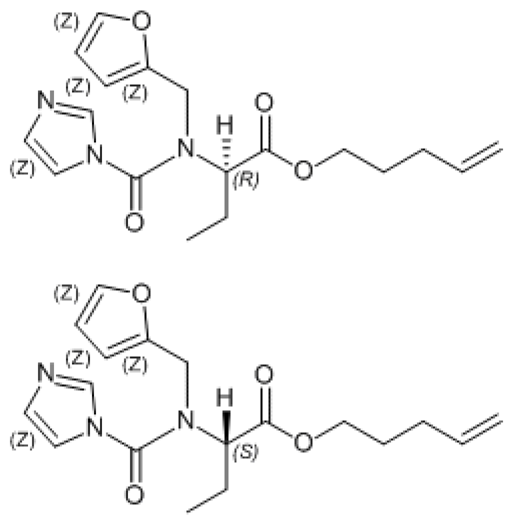	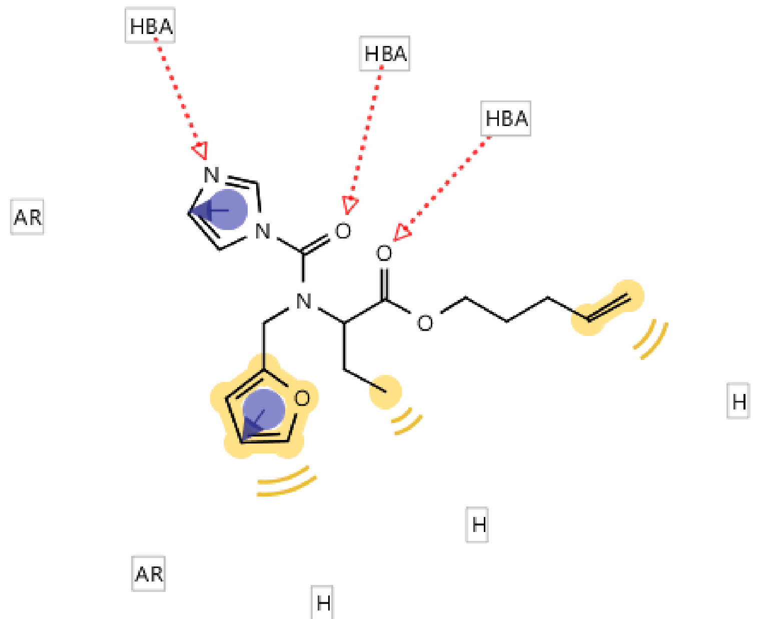
PR 967-234	113614-50-9	SMZRSICFVRMOFE-WKZUIDESSA-N	InChI=1S/2C20H19Cl2N3O/c2*1-24-20(13-25-11-10-23-14-25,16-4-8-18(22)9-5-16)12-19(26-24)15-2-6-17(21)7-3-15/h2*2-11,14,19H,12-13H2,1H3/t2*19-,20-/m10/s1	CN1[C@](C2=CC=C(C=C2)Cl)(C[C@H](C3=CC=C(C=C3)Cl)O1)CN4C=CN=C4.CN5[C@@](C6=CC=C(C=C6)Cl)(C[C@@H](C7=CC=C(C=C7)Cl)O5)CN8C=CN=C8	Sterol 14α-demethylase inhibitor	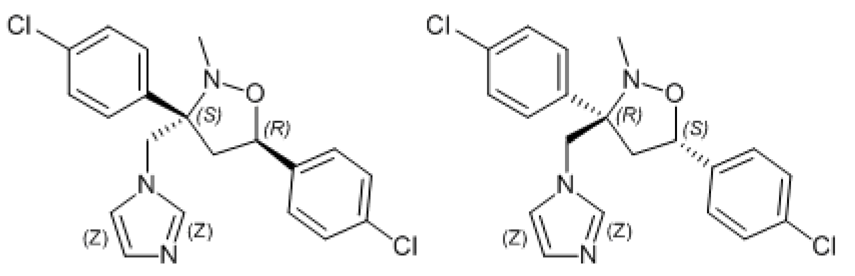	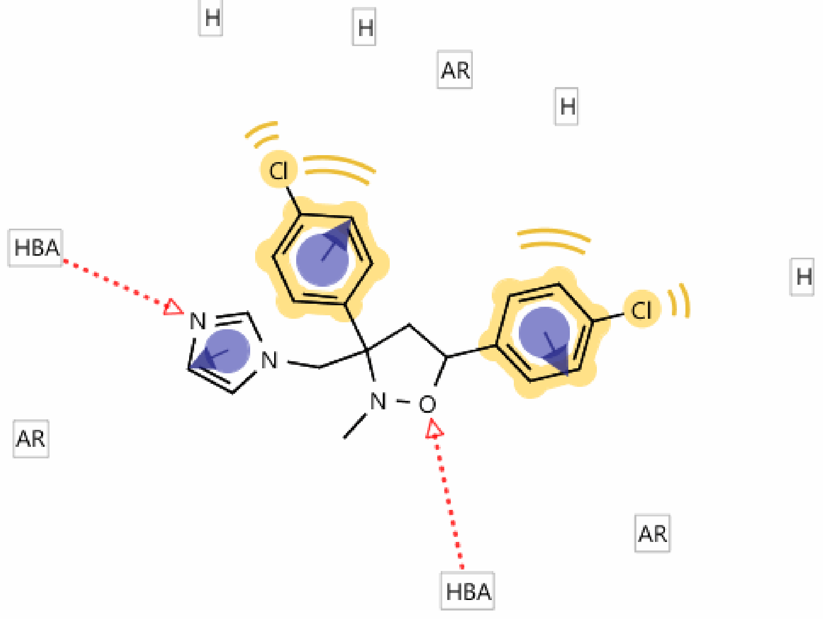
Prochloraz	67747-09-5	TVLSRXXIMLFWEO-UHFFFAOYSA-N	InChI=1S/C15H16Cl3N3O2/c1-2-4-20(15(22)21-5-3-19-10-21)6-7-23-14-12(17)8-11(16)9-13(14)18/h3,5,8-10H,2,4,6-7H2,1H3	CCCN(CCOC1=C(C=C(C=C1Cl)Cl)Cl)C(N2C=CN=C2)=O	Sterol 14α-demethylase inhibitor	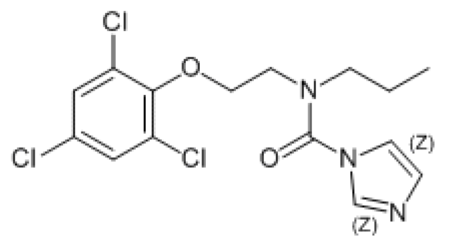	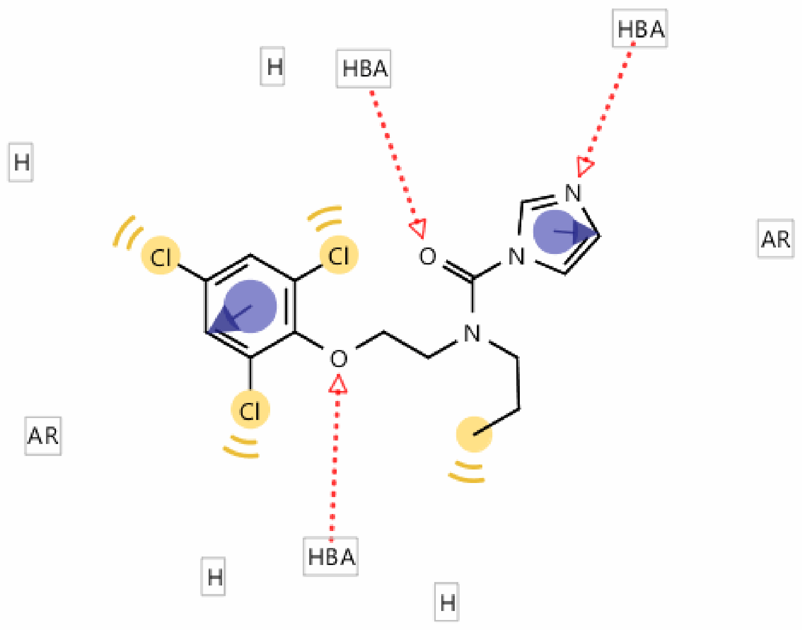

Common name	CAS Reg. No.	InChIKey	InChI	SMILES	Primary target	2D Structure	Pharmacophore[1]
R 31000	59364-79-3	GIPWYOKMIORAMK-GMHWIHOYSA-N	InChI=1S/2C20H17BrCl2N2O3/c2*21-14-1-4-16(5-2-14)26-10-17-11-27-20(28-17,12-25-8-7-24-13-25)18-6-3-15(22)9-19(18)23/h2*1-9,13,17H,10-12H2/t2*17-,20-/m10/s1	ClC1=CC(Cl)=C([C@@]2(OC[C@H](O2)COC3=CC=C(Br)C=C3)CN4C=CN=C4)C=C1.ClC5=CC(Cl)=C([C@]6(OC[C@@H](O6)COC7=CC=C(Br)C=C7)CN8C=CN=C8)C=C5	Sterol 14α-demethylase inhibitor	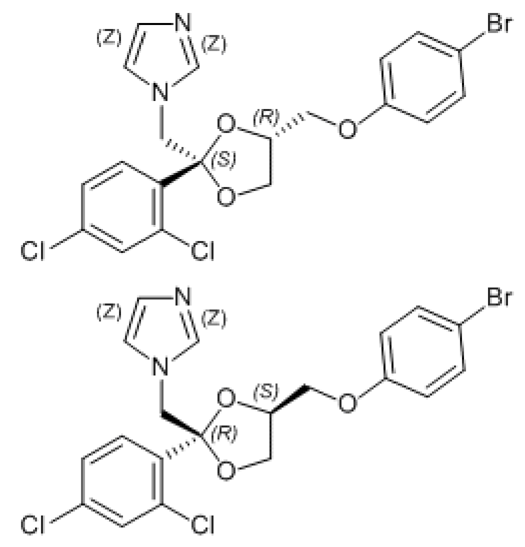	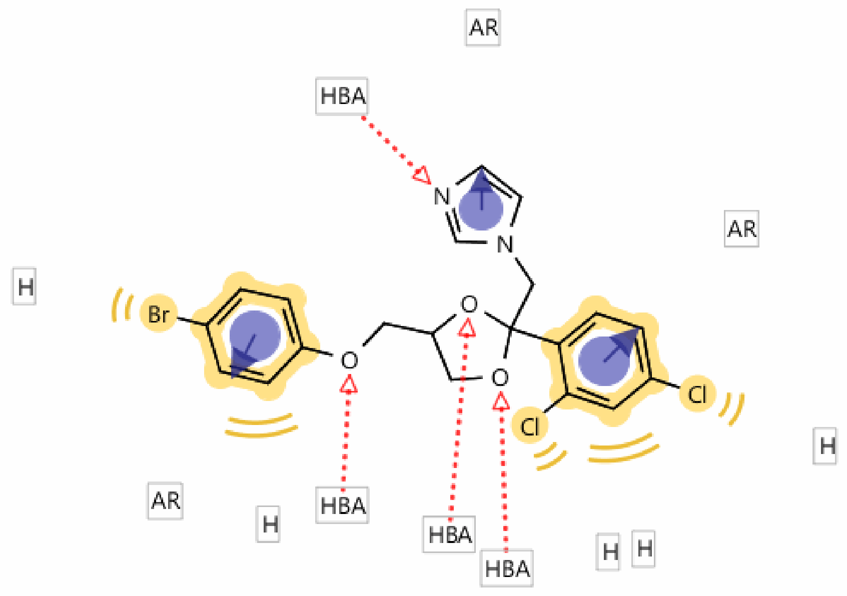
Sertaconazole	99592-32-2	LJLKIDAGGMBTKK-OYPHMNEHSA-N	InChI=1S/2C20H15Cl3N2OS/c2*21-14-4-5-16(18(23)8-14)19(9-25-7-6-24-12-25)26-10-13-11-27-20-15(13)2-1-3-17(20)22/h2*1-8,11-12,19H,9-10H2/t2*19-/m10/s1	ClC1=CC(Cl)=C(C=C1)[C@@]([H])(CN2C=CN=C2)OCC3=CSC4=C3C=CC=C4Cl.ClC5=CC(Cl)=C(C=C5)[C@]([H])(CN6C=CN=C6)OCC7=CSC8=C7C=CC=C8Cl	Sterol 14α-demethylase inhibitor	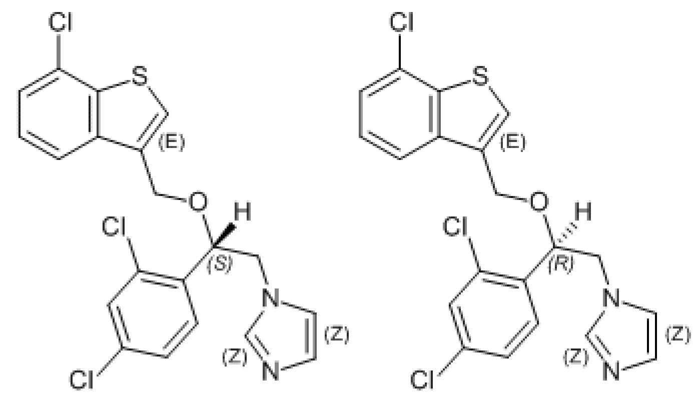	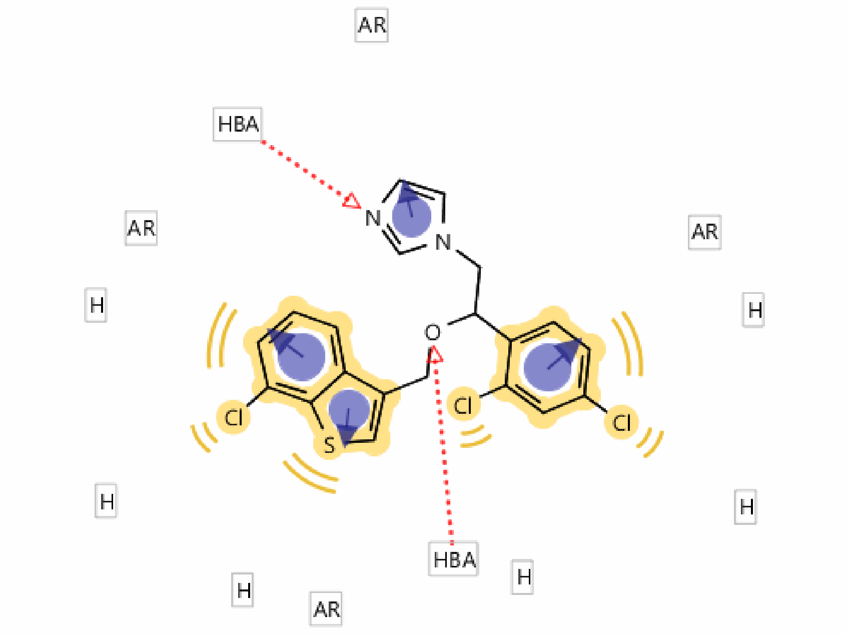
SM 4470	89433-57-8	GYJPRBFOYUKMDU-PKLMIRHRSA-N	InChI=1S/C16H20Cl2N2OS.ClH/c1-2-3-8-22-11-16(21,10-20-7-6-19-12-20)14-5-4-13(17)9-15(14)18;/h4-7,9,12,21H,2-3,8,10-11H2,1H3;1H/t16-;/m1./s1	CCCCSC[C@](O)(CN1C=CN=C1)C2=C(C=C(C=C2)Cl)Cl.Cl	Sterol 14α-demethylase inhibitor	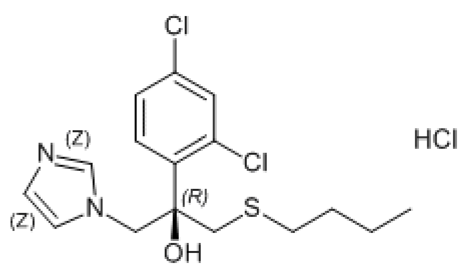	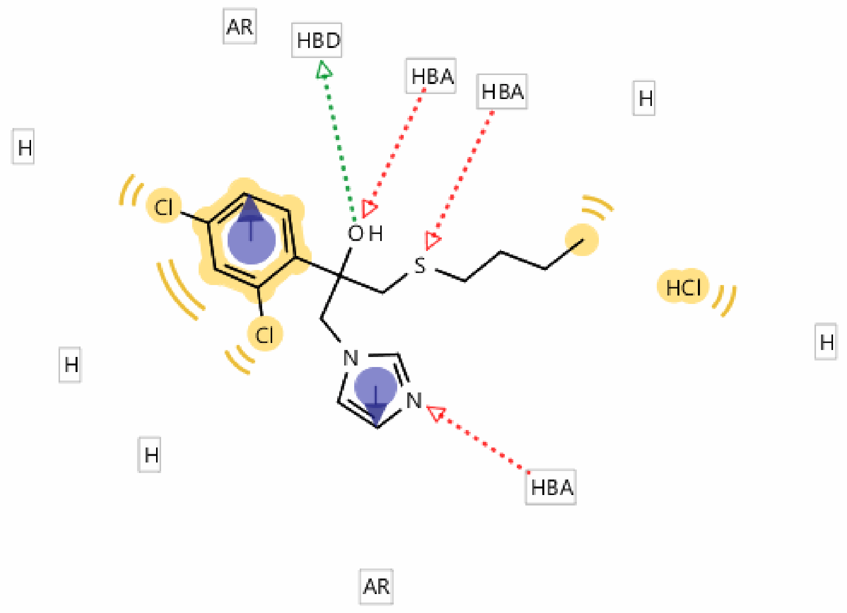
SSF 105	92678-39-2	WSJCEFBEHAVGML-UHFFFAOYSA-N	InChI=1S/C18H14Cl2N2O/c1-13(22-9-8-21-12-22)16-4-2-3-5-18(16)23-11-14-6-7-15(19)10-17(14)20/h2-10,12H,1,11H2	C=C(C1=CC=CC=C1OCC2=C(C=C(C=C2)Cl)Cl)N3C=CN=C3	Sterol 14α-demethylase inhibitor	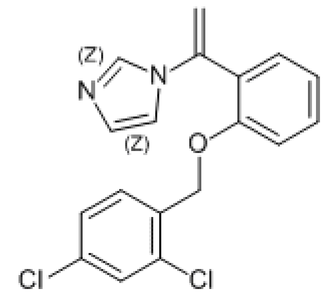	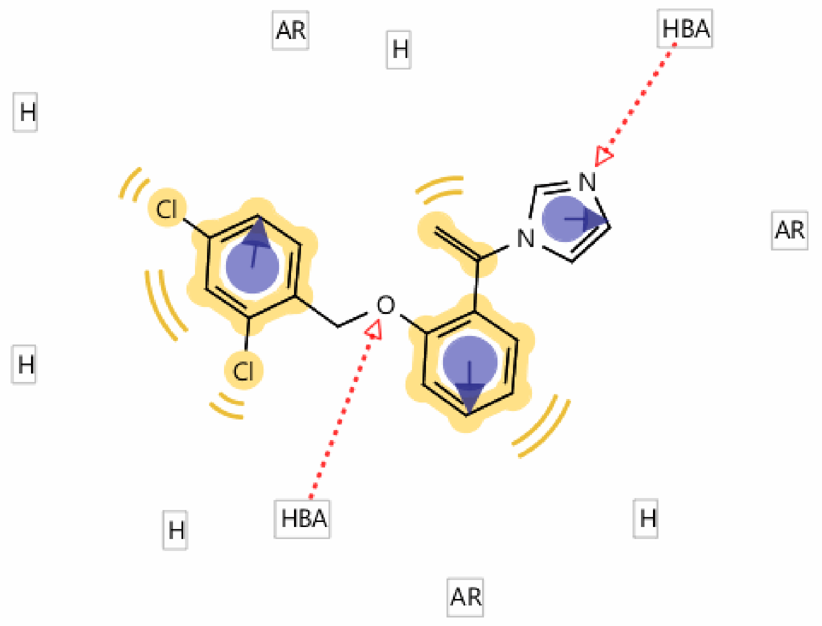
Sulconazole	61318-90-9	CTIGODQDRBKBMD-JJMXQKMNSA-N	InChI=1S/2C18H15Cl3N2S/c2*19-14-3-1-13(2-4-14)11-24-18(10-23-8-7-22-12-23)16-6-5-15(20)9-17(16)21/h2*1-9,12,18H,10-11H2/t2*18-/m10/s1	ClC1=CC=C(C=C1)CS[C@]([H])(CN2C=CN=C2)C3=C(C=C(C=C3)Cl)Cl.ClC4=CC=C(C=C4)CS[C@@]([H])(CN5C=CN=C5)C6=C(C=C(C=C6)Cl)Cl	Sterol 14α-demethylase inhibitor	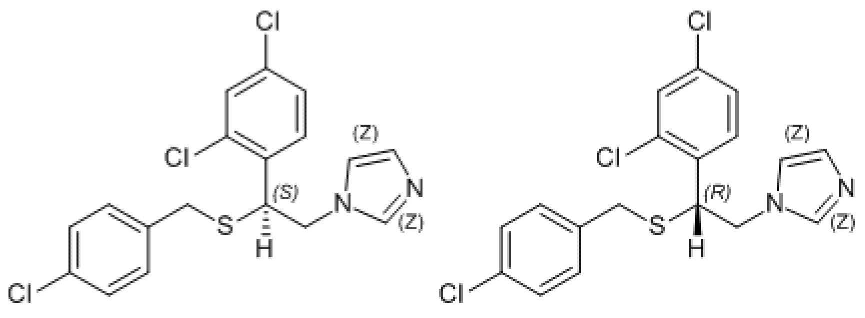	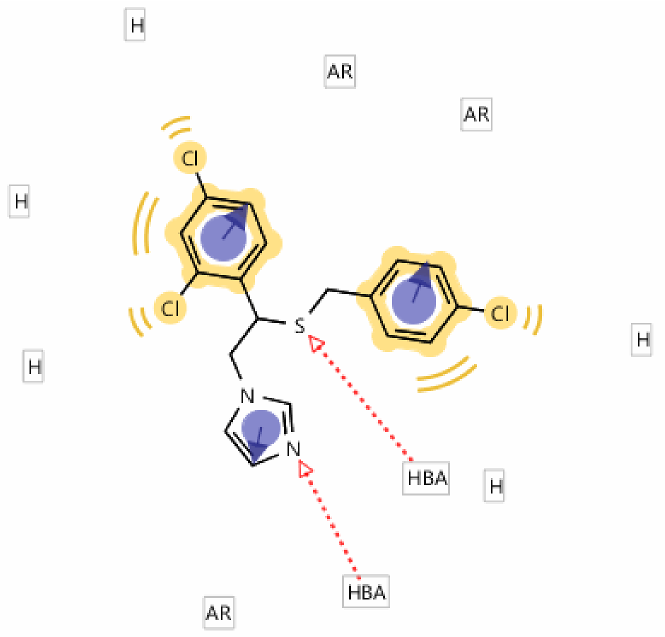
Tioconazole	65899-73-2	TWBXKPYJOIBYBR-ZWZQDMJTSA-N	InChI=1S/2C16H13Cl3N2OS/c2*17-12-1-2-13(14(18)7-12)15(8-21-5-4-20-10-21)22-9-11-3-6-23-16(11)19/h2*1-7,10,15H,8-9H2/t2*15-/m10/s1	ClC1=CC(Cl)=C(C=C1)[C@@]([H])(CN2C=CN=C2)OCC3=C(Cl)SC=C3.ClC4=CC(Cl)=C(C=C4)[C@]([H])(CN5C=CN=C5)OCC6=C(Cl)SC=C6	Sterol 14α-demethylase inhibitor	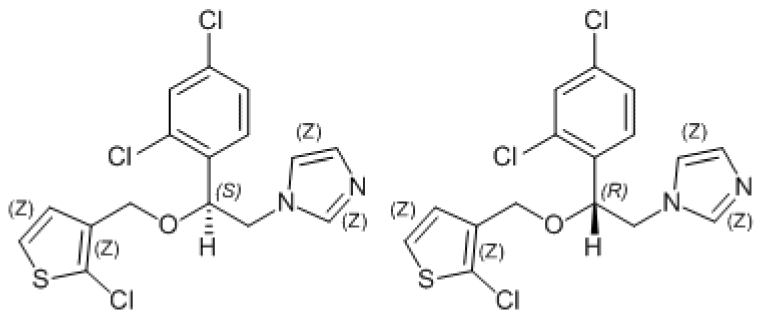	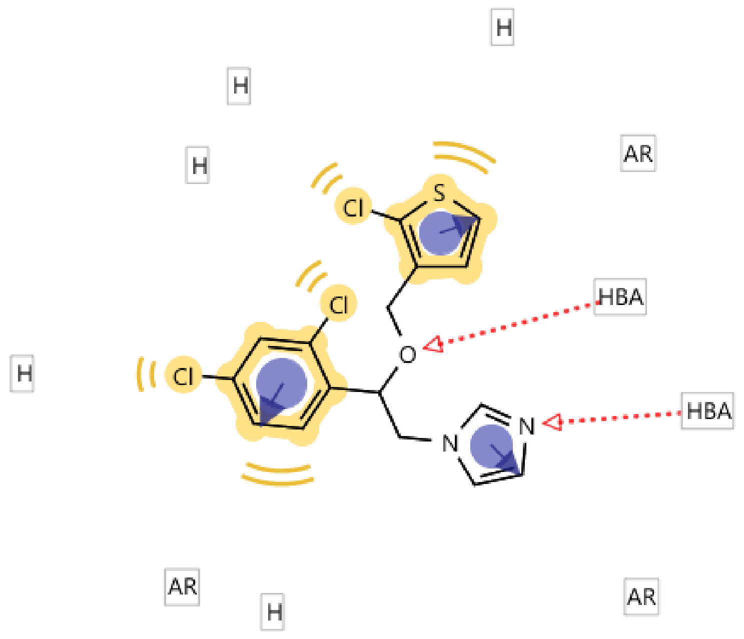

Common name	CAS Reg. No.	InChIKey	InChI	SMILES	Primary target	2D Structure	Pharmacophore[1]
Triflumizole	68694-11-1	HSMVPDGQOIQYSR-KGENOOAVSA-N	InChI=1S/C15H15ClF3N3O/c1-2-7-23-9-14(22-6-5-20-10-22)21-13-4-3-11(16)8-12(13)15(17,18)19/h3-6,8,10H,2,7,9H2,1H3/b21-14+	CCCOC/C(N1C=CN=C1)=N\C2=C(C=C(C=C2)Cl)C(F)(F)F	Sterol 14α-demethylase inhibitor	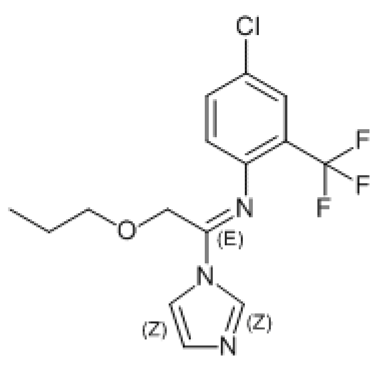	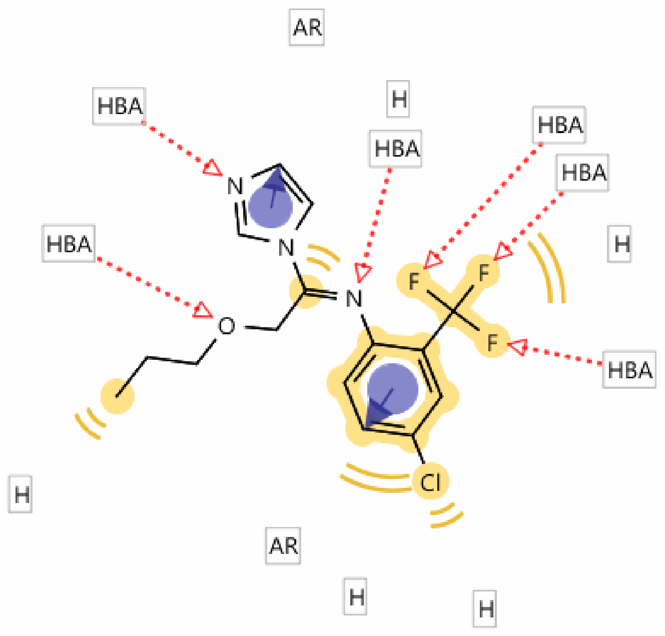
UK 38667		UBVGNXYFFHOQEO-IKFJUQJOSA-N	InChI=1S/2C14H16Cl2N2O/c2*1-2-5-14(19,9-18-7-6-17-10-18)12-4-3-11(15)8-13(12)16/h2*3-4,6-8,10,19H,2,5,9H2,1H3/t2*14-/m10/s1	CCC[C@@](O)(CN1C=CN=C1)C2=C(C=C(C=C2)Cl)Cl.CCC[C@](O)(CN3C=CN=C3)C4=C(C=C(C=C4)Cl)Cl	Sterol 14α-demethylase inhibitor	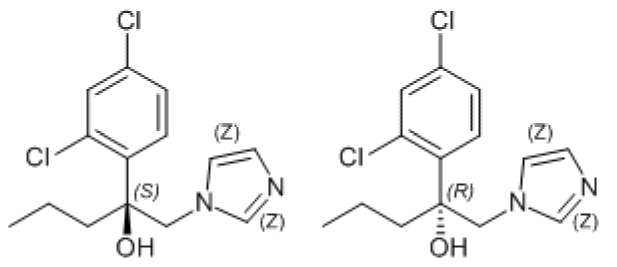	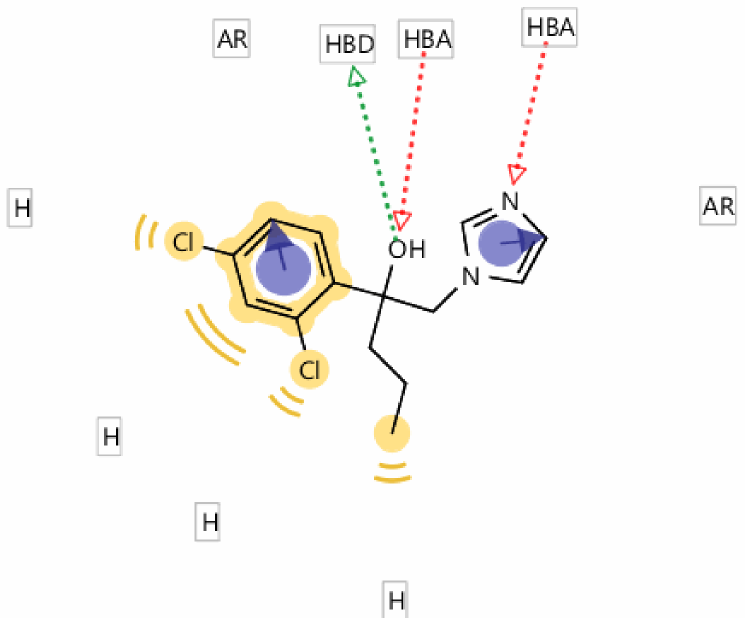
Valconazole	56097-80-4	QMLYQGYOBJCNOZ-IKFJUQJOSA-N	InChI=1S/2C16H18Cl2N2O2/c2*1-16(2,3)15(21)14(9-20-7-6-19-10-20)22-13-5-4-11(17)8-12(13)18/h2*4-8,10,14H,9H2,1-3H3/t2*14-/m10/s1	CC(C)(C)C([C@@](CN1C=CN=C1)(OC2=C(Cl)C=C(Cl)C=C2)[H])=O.CC(C)(C)C([C@](CN3C=CN=C3)(OC4=C(Cl)C=C(Cl)C=C4)[H])=O	Sterol 14α-demethylase inhibitor	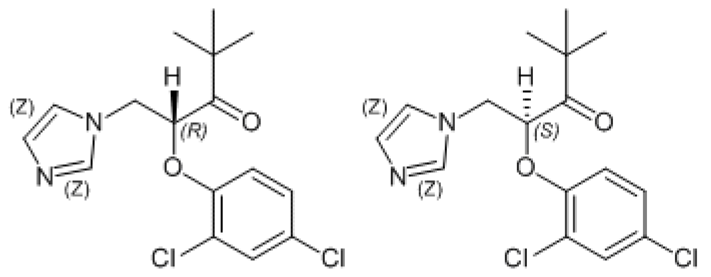	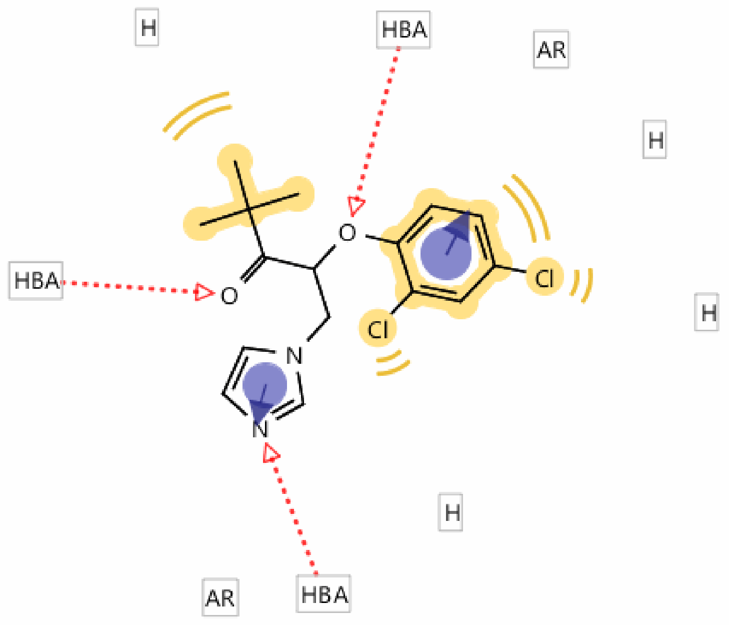
Zinoconazole	84697-21-2	KCHHCAJDHQEGNL-UDWIEESQSA-N	InChI=1S/C15H11Cl3N4S/c16-10-2-1-3-11(17)15(10)21-20-12(8-22-7-6-19-9-22)13-4-5-14(18)23-13/h1-7,9,21H,8H2/b20-12+	ClC1=C(N/N=C(C2=CC=C(S2)Cl)\CN3C=CN=C3)C(Cl)=CC=C1	Sterol 14α-demethylase inhibitor	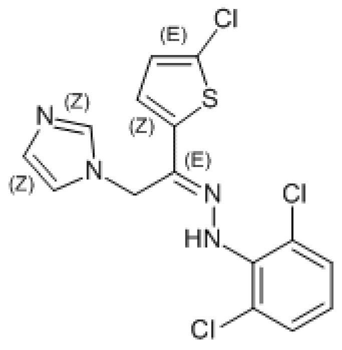	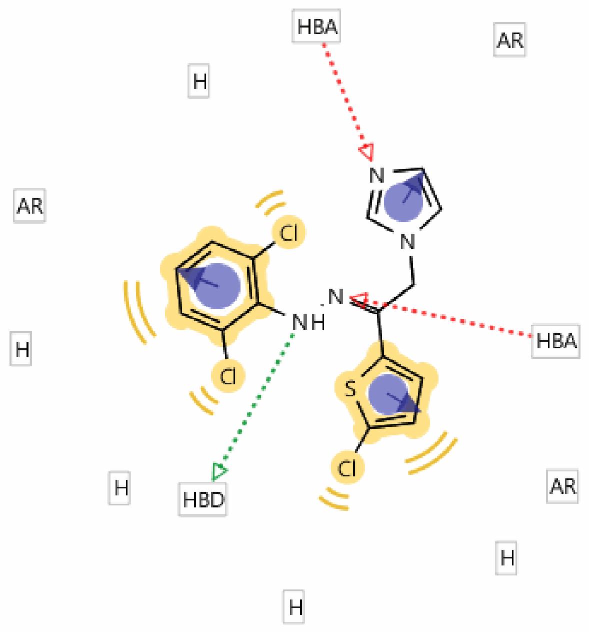
Zoficonazole	71097-23-9	DHNBWYKFUTZUFX-ZCLATKBISA-N	InChI=1S/2C20H19Cl3N2O2/c2*21-15-2-5-17(6-3-15)26-10-1-11-27-20(13-25-9-8-24-14-25)18-7-4-16(22)12-19(18)23/h2*2-9,12,14,20H,1,10-11,13H2/t2*20-/m10/s1	ClC1=CC=C(C=C1)OCCCO[C@]([H])(CN2C=CN=C2)C3=C(C=C(C=C3)Cl)Cl.ClC4=CC=C(C=C4)OCCCO[C@@]([H])(CN5C=CN=C5)C6=C(C=C(C=C6)Cl)Cl	Sterol 14α-demethylase inhibitor	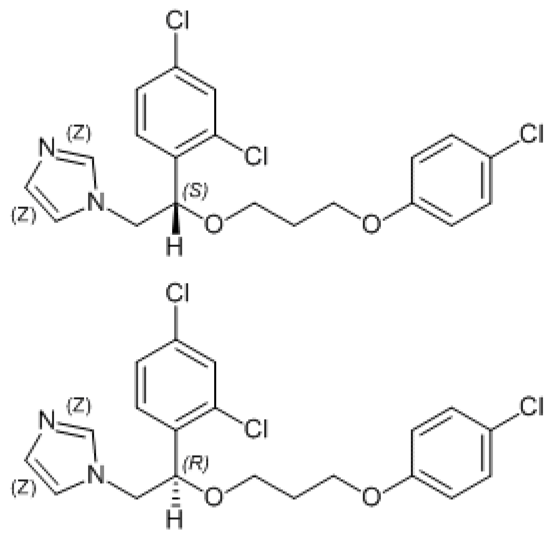	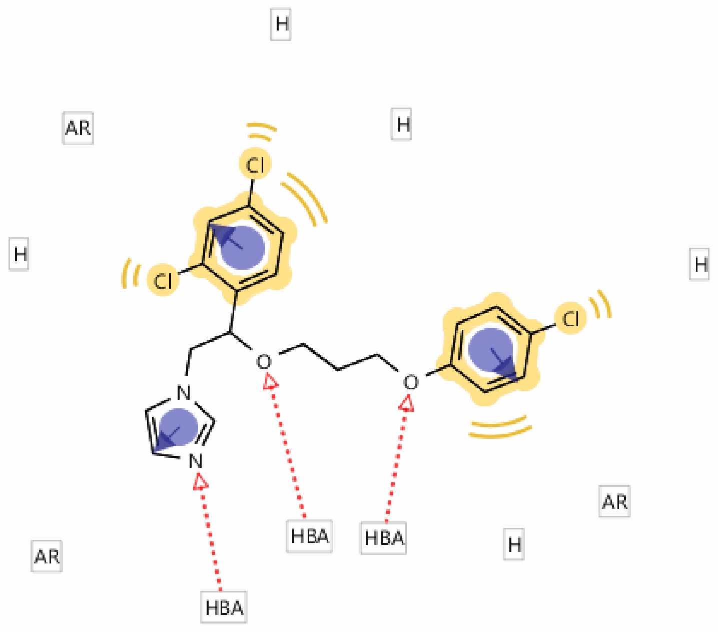

Reference

1. Wolber, G., Dornhofer, A. A. & Langer, T. Efficient overlay of small organic molecules using 3D pharmacophores. *J. Comput. Aided Mol. Des.* **20**, 773–788 (2006).

Table S2. Primary targets published by scientific literature for bioactive molecules of the present study

Bioactive molecule	Primary target	Source
A 57132	Unknown	Unknown
A 57241	DNA gyrase inhibitor	Shen *et al*., 1989
A 57274 (A 62917)	DNA gyrase inhibitor	Shen *et al*., 1989; Domagala, 1994
A 60919 (PD 118106)	DNA gyrase inhibitor	Domagala, 1994
A 61867 (BRN 4276829)	DNA gyrase inhibitor	Shen *et al*., 1989
A 62251 (A 57531; PD 137954)	DNA gyrase inhibitor	Shen *et al*., 1989
A 62255	DNA gyrase inhibitor	Shen *et al*., 1989
A 62824	DNA gyrase inhibitor<br>DNA gyrase and topoisomerase IV inhibitor	Domagala, 1994<br>Wiles *et al*., 2006
A 65326	DNA gyrase inhibitor	Rosen *et al*., 1988
ACH 702	DNA gyrase inhibitor<br>DNA gyrase and topoisomerase IV inhibitor	Pucci *et al*., 2010<br>Kim *et al*., 2011
Acorafloxacin (avarofloxacin)	DNA gyrase and topoisomerase IV inhibitor	Morrow *et al*., 2010; Butler *et al*., 2013
ADDNC (A 65485)	DNA gyrase inhibitor	Rosen *et al*., 1988
Alalevonadifloxacin	DNA gyrase and topoisomerase IV inhibitor	Butler *et al*., 2013
Alatrofloxacin	DNA gyrase and topoisomerase IV inhibitor	Brighty & Gootz, 1997
Amifloxacin	DNA gyrase inhibitor	Domagala *et al*., 1986; Zweerink & Edison, 1986
Antofloxacin	DNA gyrase inhibitor	Yu *et al*., 2016
AT 4929	Unknown	Unknown
Balofloxacin	DNA gyrase inhibitor	Ito *et al*., 1992; Domagala, 1994
BAY Y-3118 free base	DNA gyrase inhibitor	Domagala, 1994; Georgopoulos *et al*., 1998
Besifloxacin	DNA gyrase inhibitor	Ward *et al*., 2007
Binfloxacin	Unknown	Unknown
BMY 40062	DNA gyrase inhibitor	Bazile *et al*., 1992; Domagala, 1994
BMY 40397	DNA gyrase inhibitor	Bazile *et al*., 1992
BMY 42230	DNA gyrase inhibitor	Bazile *et al*., 1992
BMY 43261	DNA gyrase inhibitor	Bazile *et al*., 1992
BMY 43748	Unknown	Unknown
BMY 45243	Unknown	Unknown
BMY 45706	DNA gyrase inhibitor	Bazile *et al*., 1992
BRN 4913428 (PD 131199)	DNA gyrase inhibitor	Domagala, 1994
Cadrofloxacin	DNA gyrase and topoisomerase IV inhibitor	Takenouchi *et al*., 1996
Cetefloxacin	DNA gyrase inhibitor	Georgopoulos *et al*., 1998
Chinfloxacin	Unknown	Unknown
CI 990 (PD 131112)	DNA gyrase inhibitor	Domagala, 1994
Ciprofloxacin	DNA gyrase inhibitor<br>DNA gyrase and topoisomerase IV inhibitor	Shen & Pernet, 1985; Domagala *et al*., 1986; Zweerink & Edison, 1986; Barrett *et al*., 1989; Shen *et al*., 1989; Kotera *et al*., 1990; Ozaki *et al*., 1991; Piddock *et al*., 1991; Bazile *et al*., 1992; Ito *et al*., 1992; Rohner *et al*., 1992; Muratani *et al*., 1992; Domagala, 1994; Albertini *et al*., 1995; Coughlin *et al*., 1995; Takahashi *et al*., 1997; Choi *et al*., 1998; Georgopoulos *et al*., 1998<br>Hoshino *et al*., 1994; Brighty & Gootz, 1997; Takenouchi *et al*., 1996; Ince & Hooper, 2000; Moreillon & Entenza, 2001; Kishii *et al*., 2003; Otani *et al*., 2003; de Souza *et al*., 2005; Strahilevitz *et al*., 2005; Morrow *et al*., 2010; Higuchi *et al*., 2013; Kazamori *et al*., 2014; Kishii *et al*., 2017
Clinafloxacin	DNA gyrase inhibitor	Piddock *et al*., 1991; Domagala, 1994
CP 100964	Unknown	Unknown
CP 104830	DNA gyrase inhibitor	Gootz *et al*., 1994
CP 105532 (PD 125275)	DNA gyrase inhibitor	Gootz *et al*., 1994
CP 115953	DNA gyrase inhibitor	Robinson *et al*., 1991; Robinson *et al*., 1992
CP 115955	DNA gyrase inhibitor	Robinson *et al*., 1992
CP 135803	Unknown	Unknown
CP 67015	DNA gyrase inhibitor	Barrett *et al*., 1989
CP 67804	DNA gyrase inhibitor	Robinson *et al*., 1991; Albertini *et al*., 1995
CP 74667	Unknown	Unknown
CP 92121	Unknown	Unknown
CP 99433	Unknown	Unknown
Danofloxacin	DNA gyrase inhibitor<br>DNA gyrase and topoisomerase IV inhibitor	McGuirk *et al*., 1992<br>Sato *et al*., 2013
DC 159a free base	DNA gyrase and topoisomerase IV inhibitor	Hoshino *et al*., 2008
Delafloxacin	DNA gyrase and topoisomerase IV inhibitor	Butler *et al*., 2013

Bioactive molecule	Primary target	Source
Desfluorociprofloxacin (SQ 4004)	Unknown	Unknown
Difloxacin	DNA gyrase inhibitor	Shen *et al*., 1989
DJ 6783	Unknown	Unknown
DK 507k	DNA gyrase and topoisomerase IV inhibitor	Otani *et al*., 2003
DN 9494	DNA gyrase inhibitor	Hoshino *et al*., 1991
Droxacin	Unknown	Unknown
DS 8587 free base	DNA gyrase and topoisomerase IV inhibitor	Higuchi *et al*., 2013
DU 6611	DNA gyrase inhibitor	Kitamura *et al*., 1995
DU 6668	DNA gyrase inhibitor	Kitamura *et al*., 1995
DV 7751a (DV 7751)	DNA gyrase inhibitor	Hoshino *et al*., 1994
DW 8186	DNA gyrase inhibitor	Kitamura *et al*., 1995
DX 619	DNA gyrase and topoisomerase IV inhibitor	Strahilevitz *et al*., 2005
E 3604	Unknown	Unknown
E 3846	Unknown	Unknown
E 4441	Unknown	Unknown
E 4474	Unknown	Unknown
E 4480	Unknown	Unknown
E 4497	Unknown	Unknown
E 4501	Unknown	Unknown
E 4502	Unknown	Unknown
E 4527	Unknown	Unknown
E 4528	Unknown	Unknown
E 4534	Unknown	Unknown
E 4535	Unknown	Unknown
E 4695	Unknown	Unknown
Ecenofloxacin	Unknown	Unknown
EN 272	Unknown	Unknown
Enoxacin	DNA gyrase inhibitor<br>DNA gyrase and topoisomerase IV inhibitor	Domagala *et al*., 1986; Piddock *et al*., 1991; Domagala, 1994; Albertini *et al*., 1995<br>Takenouchi *et al*., 1996
Enrofloxacin	DNA gyrase inhibitor<br>DNA gyrase and topoisomerase IV inhibitor	Albertini *et al*., 1995<br>Sato *et al*., 2013
Esafloxacin	Unknown	Unknown
FA 103	Unknown	Unknown
Fandofloxacin	DNA gyrase inhibitor	Choi *et al*., 1998
Finafloxacin	DNA gyrase and topoisomerase IV inhibitor	Butler *et al*., 2013
Fleroxacin	DNA gyrase inhibitor<br>DNA gyrase and topoisomerase IV inhibitor	Domagala *et al*., 1986; Rohner *et al*., 1992; Domagala, 1994; Albertini *et al*., 1995<br>Takenouchi *et al*., 1996
Flumequine	DNA gyrase inhibitor	Zweerink & Edison, 1986
Garenoxacin	DNA gyrase inhibitor<br>DNA gyrase and topoisomerase IV inhibitor	Bhagwat *et al*., 2006<br>Strahilevitz *et al*., 2005; Kishii *et al*., 2017
Gatifloxacin	DNA gyrase inhibitor<br>DNA gyrase and topoisomerase IV inhibitor	Domagala, 1994<br>Takenouchi *et al*., 1996; Otani *et al*., 2003; Kishii *et al*., 2003
Gemifloxacin	DNA gyrase and topoisomerase IV inhibitor	Strahilevitz *et al*., 2005; Morrow *et al*., 2010; Kazamori *et al*., 2014
Grepafloxacin	DNA gyrase inhibitor	Domagala, 1994
Ibafloxacin	DNA gyrase inhibitor	Wolfson *et al*., 1987
Irloxacin	Unknown	Unknown
K 12	Unknown	Unknown
KB 5246	DNA gyrase inhibitor	Kotera *et al*., 1990
KPI 10 free base (WQ 3810)	DNA gyrase and topoisomerase IV inhibitor	Kazamori *et al*., 2014
Lascufloxacin	DNA gyrase and topoisomerase IV inhibitor	Kishii *et al*., 2017
Levofloxacin	DNA gyrase inhibitor<br>DNA gyrase and topoisomerase IV inhibitor	Hoshino *et al*., 1991; Takahashi et al., 1997; Yu *et al*., 2016<br>Hoshino *et al*., 1994; Moreillon & Entenza, 2001;Yamakawa *et al*., 2002; Otani *et al*., 2003; Fecik *et al*., 2005; Kazamori *et al*., 2014; Higuchi *et al*., 2013; Kishii *et al*., 2017
Levonadifloxacin	DNA gyrase and topoisomerase IV inhibitor	Patel *et al*., 2004; de Souza *et al*., 2005
Levonadifloxacin arginine (WCK 771)	DNA gyrase inhibitor<br>DNA gyrase and topoisomerase IV inhibitor	Bhagwat *et al*., 2006<br>Patel *et al*., 2004; Al-Lahham *et al*., 2005; de Souza *et al*., 2005; Butler *et al*., 2013
Lomefloxacin	DNA gyrase inhibitor<br>DNA gyrase and topoisomerase IV inhibitor	Shen *et al*., 1989; Ito *et al*., 1992; Domagala, 1994; Albertini *et al*., 1995; Georgopoulos *et al*., 1998<br>Takenouchi *et al*., 1996

Bioactive molecule	Primary target	Source
Marbofloxacin	DNA gyrase IV inhibitor	Albertini *et al*., 1995
Merafloxacin	DNA gyrase inhibitor	Shen *et al*., 1989; Domagala, 1994
Metioxate	Unknown	Unknown
MF 5101	Unknown	Unknown
MF 5103	Unknown	Unknown
MF 5112 free base	DNA gyrase inhibitor	Cecchetti *et al*., 1996
MF 5126	DNA gyrase inhibitor	Cecchetti *et al*., 1996
MF 5137	DNA gyrase inhibitor	Cecchetti *et al*., 1996
MF 5143	DNA gyrase inhibitor	Cecchetti *et al*., 1996
MF 5168	Unknown	Unknown
Miloxacin	DNA gyrase inhibitor	Domagala *et al*., 1986
Moxifloxacin	DNA gyrase inhibitor<br>DNA gyrase and topoisomerase IV inhibitor	Bhagwat *et al*., 2006; Yu *et al*., 2016<br>Moreillon & Entenza, 2001; Otani *et al*., 2003; Morrow *et al*., 2010; Kazamori *et al*., 2014
Nadifloxacin	DNA gyrase inhibitor<br>DNA gyrase and topoisomerase IV inhibitor	Fecik *et al*., 2005<br>Yamakawa *et al*., 2002
Nalidixic acid	DNA gyrase inhibitor<br>DNA gyrase and topoisomerase IV inhibitor	Gellert *et al*., 1977; Sugino *et al*., 1977; Shen & Pernet, 1985; Domagala *et al*., 1986; Zweerink & Edison, 1986; Barrett *et al*., 1989; Shen *et al*., 1989; Piddock *et al*., 1991; Albertini *et al*., 1995; Georgopoulos *et al*., 1998<br>Hoshino *et al*., 1994
Nemonoxacin	DNA gyrase and topoisomerase IV inhibitor	Butler *et al*., 2013
Norfloxacin	DNA gyrase inhibitor<br>DNA gyrase and topoisomerase IV inhibitor	Shen & Pernet, 1985; Domagala *et al*., 1986; Zweerink & Edison, 1986; Barrett *et al*., 1989; Shen *et al*., 1989; Kotera *et al*., 1990; Ozaki *et al*., 1991; Muratani *et al*., 1992; Domagala, 1994; Albertini *et al*., 1995; Coughlin *et al*., 1995; Georgopoulos *et al*., 1998<br>Takenouchi *et al*., 1996; Otani *et al*., 2003
NSFQ 104	DNA gyrase inhibitor	Alovero *et al*., 1998
NSFQ 105	DNA gyrase inhibitor	Alovero *et al*., 1998
Ofloxacin	DNA gyrase inhibitor<br>DNA gyrase and topoisomerase IV inhibitor	Zweerink & Edison, 1986; Domagala *et al*., 1986; Wolfson *et al*., 1987; Shen *et al*., 1989; Kotera *et al*., 1990; Hoshino *et al*., 1991; Ozaki *et al*., 1991; Muratani *et al*., 1992; Albertini *et al*., 1995; Coughlin *et al*., 1995; Ito *et al*., 1992; Domagala, 1994; Georgopoulos *et al*., 1998<br>Takenouchi *et al*., 1996; Yamakawa *et al*., 2002; Yu *et al*., 2016
Olamufloxacin	DNA gyrase inhibitor	Takahashi et al., 1997
Orbifloxacin	DNA gyrase and topoisomerase IV inhibitor	Sato *et al*., 2013
Oxolinic acid	DNA gyrase inhibitor<br>DNA gyrase and topoisomerase IV inhibitor	Gellert *et al*., 1977; Sugino *et al*., 1977; Shen & Pernet, 1985; Domagala *et al*., 1986; Zweerink & Edison, 1986; Shen *et al*., 1989; Barrett *et al*., 1989; Albertini *et al*., 1995<br>Takenouchi *et al*., 1996
Ozenoxacin	DNA gyrase and topoisomerase IV inhibitor	Yamakawa *et al*., 2002; Butler *et al*., 2013
Pazufloxacin	DNA gyrase inhibitor	Muratani *et al*., 1992; Fecik *et al*., 2005
PD 111834	Unknown	Unknown
PD 112388	Unknown	Unknown
PD 114111	Unknown	Unknown
PD 115311	Unknown	Unknown
PD 116507	Unknown	Unknown
PD 117596	DNA gyrase inhibitor	Piddock *et al*., 1991
PD 118362	Unknown	Unknown
PD 119344	Unknown	Unknown
PD 129626	Unknown	Unknown
PD 131628	DNA gyrase inhibitor	Domagala, 1994
PD 135042 (AM 1147)	DNA gyrase and topoisomerase IV inhibitor	Kishii *et al*., 2003
PD 135144 (BMY 33315)	DNA gyrase inhibitor	Bazile *et al*., 1992
PD 137156	Unknown	Unknown
PD 138312	DNA gyrase inhibitor	Domagala, 1994
PD 140248	DNA gyrase inhibitor	Domagala, 1994
PD 163449	DNA gyrase and topoisomerase IV inhibitor	Zhao *et al*., 1998
PD 164488	DNA gyrase and topoisomerase IV inhibitor	Zhao *et al*., 1998
Pefloxacin	DNA gyrase inhibitor	Shen & Pernet, 1985; Zweerink & Edison, 1986; Domagala *et al*., 1986; Shen *et al*., 1989; Bazile *et al*., 1992; Domagala, 1994; Albertini *et al*., 1995; Georgopoulos *et al*., 1998
Pipemidic acid	DNA gyrase inhibitor<br>DNA gyrase and topoisomerase IV inhibitor	Shen & Pernet, 1985; Zweerink & Edison, 1986; Domagala *et al*., 1986; Georgopoulos *et al*., 1998<br>Takenouchi *et al*., 1996
Piromidic acid	DNA gyrase inhibitor<br>DNA gyrase and topoisomerase IV inhibitor	Domagala *et al*., 1986<br>Takenouchi *et al*., 1996

Bioactive molecule	Primary target	Source
Piroxacin	Unknown	Unknown
Pradofloxacin	Topoisomerase IV inhibitor	Köerber *et al*., 2002
Premafloxacin	DNA gyrase and topoisomerase IV inhibitor	Ince & Hooper, 2000
Prulifloxacin	DNA gyrase inhibitor	Yoshida & Mitsuhashi, 1993
PubChem CID-11531032	DNA gyrase and topoisomerase IV inhibitor	Wiles *et al*., 2006
PubChem CID-11566845	DNA gyrase and topoisomerase IV inhibitor	Wiles *et al*., 2006
PubChem CID-11610627	DNA gyrase and topoisomerase IV inhibitor	Wiles *et al*., 2006
PubChem CID-11696318	DNA gyrase and topoisomerase IV inhibitor	Wiles *et al*., 2006
PubChem CID-11844920	DNA gyrase and topoisomerase IV inhibitor	Cheng *et al*., 2007; Wang *et al*., 2007
PubChem CID-11996799	DNA gyrase and topoisomerase IV inhibitor	Cheng *et al*., 2007
PubChem CID-11996800	DNA gyrase and topoisomerase IV inhibitor	Cheng *et al*., 2007
PubChem CID-11997263	DNA gyrase and topoisomerase IV inhibitor	Cheng *et al*., 2007; Wang *et al*., 2007
PubChem CID-25022869	DNA gyrase and topoisomerase IV inhibitor	Kim *et al*., 2011
PubChem CID-44408626	DNA gyrase and topoisomerase IV inhibitor	Wiles *et al*., 2006
PubChem CID-44408894	DNA gyrase and topoisomerase IV inhibitor	Wiles *et al*., 2006
PubChem CID-44408896	DNA gyrase and topoisomerase IV inhibitor	Wiles *et al*., 2006
PubChem CID-44408994	DNA gyrase and topoisomerase IV inhibitor	Wiles *et al*., 2006
PubChem CID-44409001	DNA gyrase and topoisomerase IV inhibitor	Wiles *et al*., 2006
PubChem CID-44409010	DNA gyrase and topoisomerase IV inhibitor	Wiles *et al*., 2006
PubChem CID-53236573	DNA gyrase and topoisomerase IV inhibitor	Kim *et al*., 2011
PubChem CID-53236796	DNA gyrase and topoisomerase IV inhibitor	Kim *et al*., 2011
PubChem CID-122195336	DNA gyrase and topoisomerase IV inhibitor	Unknown
PubChem CID-122195337	DNA gyrase and topoisomerase IV inhibitor	Unknown
QA 241 free base	Unknown	Unknown
RO 13-5478	DNA gyrase inhibitor	Georgopapadakou *et al*., 1987
RO 14-9578	DNA gyrase inhibitor	Georgopapadakou *et al*., 1987
Rosoxacin	DNA gyrase inhibitor	Shen & Pernet, 1985; Zweerink & Edison, 1986; Domagala *et al*., 1986; Shen *et al*., 1989
Rufloxacin	DNA gyrase inhibitor	Georgopoulos *et al*., 1998; Choi *et al*., 1998
S 25932	DNA gyrase inhibitor	Fecik *et al*., 2005
S 31076	Unknown	Unknown
Sarafloxacin	DNA gyrase inhibitor<br>DNA gyrase and topoisomerase IV inhibitor	Shen *et al*., 1989<br>Takenouchi *et al*., 1996
Sitafloxacin	DNA gyrase inhibitor<br>DNA gyrase and topoisomerase IV inhibitor	Domagala, 1994; Kitamura *et al*., 1995<br>Hoshino *et al*., 1994; Otani *et al*., 2003
Sparfloxacin	DNA gyrase inhibitor<br>DNA gyrase and topoisomerase IV inhibitor	Ito *et al*., 1992; Rohner *et al*., 1992; Bazile *et al*., 1992; Domagala, 1994; Gootz *et al*., 1994; Takahashi *et al*., 1997; Georgopoulos *et al*., 1998; Choi *et al*., 1998<br>Hoshino *et al*., 1994; Otani *et al*., 2003
T 14097	Unknown	Unknown
Temafloxacin	DNA gyrase inhibitor	Shen *et al*., 1989; Rohner *et al*., 1992; Domagala, 1994
Tioxacin	Unknown	Unknown
Tosufloxacin	DNA gyrase inhibitor<br>DNA gyrase and topoisomerase IV inhibitor	Ito *et al*., 1992; Rosen *et al*., 1988; Muratani *et al*., 1992; Domagala, 1994<br>Takenouchi *et al*., 1996
Trovafloxacin	DNA gyrase inhibitor<br>DNA gyrase and topoisomerase IV inhibitor	Brighty & Gootz, 1997; Moreillon & Entenza, 2001<br>Bhagwat *et al*., 2006
Ulifloxacin	DNA gyrase inhibitor	Ozaki *et al*., 1991; Yoshida & Mitsuhashi, 1993
Vebufloxacin (benofloxacin)	DNA gyrase inhibitor	Fecik *et al*., 2005
VG 6/1	Unknown	Unknown
WCK 1152 free base	DNA gyrase and topoisomerase IV inhibitor	Al-Lahham *et al*., 2005
WIN 57273	DNA gyrase inhibitor	Rohner *et al*., 1992
WIN 57294	Unknown	Unknown
WIN 58161	DNA gyrase inhibitor	Coughlin *et al*., 1995; Fecik *et al*., 2005
WQ 2743	Unknown	Unknown
WQ 2756	Unknown	Unknown
WQ 2908	Unknown	Unknown
WQ 2942	Unknown	Unknown
WQ 3330	Unknown	Unknown
Y 688	DNA gyrase and topoisomerase IV inhibitor	Moreillon & Entenza, 2001
Zabofloxacin	DNA gyrase and topoisomerase IV inhibitor	Butler *et al*., 2013
Azidamfenicol	50S ribosomal subunit inhibitor	Schwarz *et al*., 2004
Bromamphenicol (bromoamphenicol)	50S ribosomal subunit inhibitor<br>50S ribosomal subunits and peptidyl transferase inhibitor	Bald *et al*., 1972<br>Sonenberg *et al*., 1973

Bioactive molecule	Primary target	Source
Cetofenicol (cetophenicol)	Unknown	Unknown
Chloramphenicol	50S ribosomal subunit inhibitor<br>50S ribosomal subunits and peptidyl transferase inhibitor	Vazquez, 1964; Schwarz *et al*., 2004; Bald *et al*., 1972; Salas & Hardisson, 1979<br>Sonenberg *et al*., 1973
Florfenicol	50S ribosomal subunit inhibitor	Schwarz *et al*., 2004
Monoiodoamphenicol	50S ribosomal subunit inhibitor	Bald et al., 1972
Racefenicol (racephenicol)	50S ribosomal subunit inhibitor	Salas & Hardisson, 1979
Tevenel	50S ribosomal subunit inhibitor	Salas & Hardisson, 1979
Thiamphenicol	50S ribosomal subunit inhibitor	Schwarz *et al*., 2004; Salas & Hardisson, 1979
WIN 5094-2	50S ribosomal subunit inhibitor	Salas & Hardisson, 1979
9-Methylstreptimidone (S 632A_2)	Unknown	Unknown
Acetoxycycloheximide (streptovitacin E-73)	60S ribosomal subunit inhibitor	Rao & Grollman, 1967
Actiketal	60S ribosomal subunit inhibitor	Lackie, 2010
Actiphenol (actinophenol)	50S ribosomal subunit inhibitor<br>60S ribosomal subunit inhibitor	Sisler & Siegel, 1967<br>Vazquez *et al*., 1974
Cycloheximide	60S ribosomal subunit inhibitor	Rao & Grollman, 1967; Vazquez *et al*., 1974; Schneider-Poetsch *et al*., 2010
Epiderstatin	60S ribosomal subunit inhibitor	Lackie , 2010
Inactone	50S ribosomal subunit inhibitor	Sisler & Siegel, 1967
Isocycloheximide	50S ribosomal subunit inhibitor	Sisler & Siegel, 1967
Isomigrastatin	60S ribosomal subunit inhibitor	Schneider-Poetsch *et al*., 2010
Lactimidomycin	60S ribosomal subunit inhibitor	Schneider-Poetsch *et al*., 2010
Naramycin B	50S ribosomal subunit inhibitor	Sisler & Siegel, 1967
Neoisocycloheximide	50S ribosomal subunit inhibitor	Sisler & Siegel, 1967
S 632A_3	Unknown	Unknown
Streptimidone (S 632A_1)	50S ribosomal subunit inhibitor<br>60S ribosomal subunit inhibitor	Sisler & Siegel, 1967<br>Rao & Grollman, 1967; Vazquez *et al*., 1974
Streptovitacin A	50S ribosomal subunit inhibitor<br>60S ribosomal subunit inhibitor	Sisler & Siegel, 1967<br>Rao & Grollman, 1967; Vazquez *et al*., 1974
Streptovitacin B	50S ribosomal subunit inhibitor<br>60S ribosomal subunit inhibitor	Sisler & Siegel, 1967<br>Lackie, 2010
Streptovitacin C_2	50S ribosomal subunit inhibitor<br>60S ribosomal subunit inhibitor	Sisler & Siegel, 1967<br>Lackie, 2010
Amicetin (allomycin)	Ribosomal peptidyl transferase inhibitor	Celma *et al*., 1970; Lichtenthaler *et al*., 1975
Antelmycin (anthelmycin)	Ribosomal peptidyl transferase inhibitor	Uchida & Wolf, 1974
Arginomycin	50S ribosomal subunit inhibitor	Gould, 1997
Bagougeramine A	50S ribosomal subunit inhibitor	Gould, 1997
Bagougeramine B	50S ribosomal subunit inhibitor	Gould, 1997
Bamicetin	Ribosomal peptidyl transferase inhibitor	Celma *et al*., 1970; Lichtenthaler *et al*., 1975
Blasticidin H	50S ribosomal subunit inhibitor	Gould, 1997
Blasticidin S	50S ribosomal subunit inhibitor<br>Ribosomal peptidyl transferase inhibitor	Kinoshita *et al*., 1970; Li *et al*., 2013; Gould, 1997<br>Celma *et al*., 1970
Cytimidine	Ribosomal peptidyl transferase inhibitor	Lichtenthaler *et al*., 1975
Cytomycin (saitomycin)	Unknown	Unknown
Cytosamine	Ribosomal peptidyl transferase inhibitor	Lichtenthaler *et al*., 1975
Cytosaminomycin A	Binding to the peptidyl transferase centre	Fu *et al*., 2018
Cytosaminomycin B	Binding to the peptidyl transferase centre	Fu *et al*., 2018
Cytosaminomycin C	Binding to the peptidyl transferase centre	Fu *et al*., 2018
Cytosaminomycin D	Binding to the peptidyl transferase centre	Fu *et al*., 2018
Gougerotin	50S ribosomal subunit inhibitor<br>Ribosomal peptidyl transferase inhibitor	Gould, 1997<br>Celma *et al*., 1970
Mildiomycin	50S ribosomal subunit inhibitor<br>Ribosomal peptidyl transferase inhibitor	Li *et al*., 2013; Gould, 1997<br>Feduchi *et al*., 1985
Mildiomycin B	Unknown	Unknown
Mildiomycin C	50S ribosomal subunit inhibitor	Gould, 1997
Mildiomycin D	50S ribosomal subunit inhibitor	Gould, 1997
Mildiomycin M	Large ribosomal subunit inhibitor	Unknown
Norplicacetin	Binding to the peptidyl transferase centre	Fu *et al*., 2018
Oxamicetin	Ribosomal peptidyl transferase inhibitor	Lichtenthaler *et al*., 1975; Fu *et al*., 2018

Bioactive molecule	Primary target	Source
Oxyplicacetin (cytosaminomycin E)	Binding to the peptidyl transferase centre	Fu *et al.*, 2018
Plicacetin (amicetin B)	Ribosomal peptidyl transferase inhibitor	Celma *et al.*, 1970; Lichtenthaler *et al.*, 1975
Rodaplutin	Unknown	Unknown
SCH 36605	50S ribosomal subunit inhibitor	Gould, 1997
SF 2457	binding to the peptidyl transferase centre	Fu *et al.*, 2018
1-Epidactimicin	Unknown	Unknown
A 396I (SS 56D)	30S ribosomal subunit inhibitor	Tanaka, 1975
Ambistrin (streptoduocin)	Unknown	Unknown
Amikacin	30S ribosomal subunit inhibitor<br>Binding to the A site in the 16S rRNA	Guo *et al.*, 1981; Tanaka, 1983<br>Kondo *et al.*, 2006
Apramycin (nebramycin II)	16S ribosomal RNA inhibitor<br>30S ribosomal subunit inhibitor<br>Ribosomal A-site inhibitor	von Ahsen *et al.*, 1992<br>Han *et al.*, 2005; Le Goffic *et al.*, 1980<br>Matt *et al.*, 2012; Mandhapati *et al.*, 2014
Aprosamine	30S ribosomal subunit inhibitor<br>Ribosomal A-site inhibitor	Han *et al.*, 2005<br>Matt *et al.*, 2012
Arbekacin	30S ribosomal subunit inhibitor	Guo *et al.*, 1981; Tanaka *et al.*, 1983
Astromicin (fortimicin A)	30S ribosomal subunit inhibitor	Guo *et al.*, 1981; Tanaka, 1983; Moreau *et al.*, 1984
Astromicin B (fortimicin B)	30S ribosomal subunit inhibitor	Moreau *et al.*, 1984
Bekanamycin (kanamycin B; nebramycin V)	16S ribosomal RNA inhibitor<br>30S ribosomal subunit inhibitor	von Ahsen *et al.*, 1991<br>Benveniste & Davies, 1973; Tanaka, 1975; Campuzano *et al.*, 1979; Le Goffic *et al.*, 1980; Tanaka, 1983
Betamicin (gentamicin B)	16S ribosomal RNA inhibitor<br>30S ribosomal subunit inhibitor	von Ahsen *et al.*, 1991<br>Guo *et al.*, 1981
Butikacin	30S ribosomal subunit inhibitor	Le Goffic *et al.*, 1980
Butirosin A	30S ribosomal subunit inhibitor	Tanaka, 1975
Butirosin B	30S ribosomal subunit inhibitor	Benveniste & Davies, 1973; Tanaka, 1975
Dactimicin	Unknown	Unknown
Destomycin A	30S ribosomal subunit inhibitor	Tanaka, 1975
Destomycin B	30S ribosomal subunit inhibitor	Tanaka, 1975
Dibekacin	30S ribosomal subunit inhibitor	Guo *et al.*, 1981; Tanaka *et al.*, 1983; Tanaka, 1983; Le Goffic *et al.*, 1980
Dihydrostreptomycin	30S ribosomal subunit inhibitor	Tanaka, 1975; Tanaka, 1983; Campuzano *et al.*, 1979
Etimicin	binding to 16S ribosomal RNA (rRNA) in the 30S subunit	Xin et al., 2006
Fortimicin AE	Unknown	Unknown
Fortimicin AH	Unknown	Unknown
Fortimicin AI	Unknown	Unknown
Fortimicin AK	Unknown	Unknown
Fortimicin AM	Unknown	Unknown
Fortimicin AN	Unknown	Unknown
Fortimicin AO	Unknown	Unknown
Fortimicin AP	Unknown	Unknown
Fortimicin AQ	Unknown	Unknown
Fortimicin AS	Unknown	Unknown
Fortimicin C	Unknown	Unknown
Fortimicin D	30S ribosomal subunit inhibitor	Guo *et al.*, 1981
Fortimicin E (fortimicin KH)	Unknown	Unknown
Fortimicin KE	Unknown	Unknown
Fortimicin KF	Unknown	Unknown
Fortimicin KG	Unknown	Unknown
Fortimicin KL_1	Unknown	Unknown
Fortimicin KR	Unknown	Unknown
Fortimicin AL	Unknown	Unknown
Framycetin (neomycin B)	16S ribosomal RNA inhibitor<br>30S ribosomal subunit inhibitor<br>Ribosomal A-site inhibitor	von Ahsen *et al.*, 1991<br>Benveniste & Davies, 1973; Tanaka, 1975; Campuzano *et al.*, 1979; Tanaka, 1983; Moreau *et al.*, 1984<br>Matt *et al.*, 2012
Geneticin (gentamicin G-418)	16S ribosomal RNA inhibitor	von Ahsen *et al.*, 1991
Gentamicin A	30S ribosomal subunit inhibitor<br>Ribosomal A-site inhibitor	Benveniste & Davies, 1973; Tanaka, 1975; Moreau *et al.*, 1984<br>Matt *et al.*, 2012
Gentamicin A_1	Unknown	Unknown
Gentamicin A_2	Unknown	Unknown

Bioactive molecule	Primary target	Source
Gentamicin A_3	Unknown	Unknown
Gentamicin A_4	Unknown	Unknown
Gentamicin B_1	Bindig ribosome A site	Baradaran-Heravi *et al*.,2017
Gentamicin C_1	16S ribosomal RNA inhibitor<br>30S ribosomal subunit inhibitor	von Ahsen *et al*., 1991<br>Benveniste & Davies, 1973; Tanaka, 1975; Campuzano *et al*., 1979; Guo *et al*., 1981; Tanaka, 1983
Gentamicin C_{1a}	16S ribosomal RNA inhibitor<br>30S ribosomal subunit inhibitor	von Ahsen *et al*., 1991<br>Benveniste & Davies, 1973; Tanaka, 1975; Campuzano *et al*., 1979; Le Goffic *et al*., 1980; Guo *et al*., 1981; Tanaka, 1983
Gentamicin C_2	16S ribosomal RNA inhibitor<br>30S ribosomal subunit inhibitor	von Ahsen *et al*., 1991<br>Benveniste & Davies, 1973; Tanaka, 1975; Guo *et al*., 1981; Tanaka, 1983
Gentamicin C_{2a}	Unknown	Unknown
Gentamicin X_2	Unknown	Unknown
Hybrimycin A_1	30S ribosomal subunit inhibitor	Tanaka, 1975
Hybrimycin A_2	30S ribosomal subunit inhibitor	Tanaka, 1975
Hybrimycin B_1	30S ribosomal subunit inhibitor	Tanaka, 1975
Hybrimycin B_2	30S ribosomal subunit inhibitor	Tanaka, 1975
Hybrimycin C_1	Unknown	Unknown
Hybrimycin C_2	Unknown	Unknown
Hybrimycin D	Unknown	Unknown
Hygromycin B (A 396II)	30S ribosomal subunit inhibitor	Tanaka, 1975
Isepamicin	binding to 16S ribosomal RNA (rRNA) in 30S subunit	Guo *et al*., 1981; Yokoyama *et al*.,2003
Istamycin A (sannamycin A)	30S ribosomal subunit inhibitor	Guo *et al*., 1981
Istamycin A_0 (sannamycin B)	30S ribosomal subunit inhibitor	Guo *et al*., 1981
Istamycin A_1	Unknown	Unknown
Istamycin A_2	Unknown	Unknown
Istamycin A_3	Unknown	Unknown
Istamycin AO	Unknown	Unknown
Istamycin AP (sannamycin E)	Unknown	Unknown
Istamycin B	Unknown	Unknown
Istamycin B_0	Unknown	Unknown
Istamycin B_1	Unknown	Unknown
Istamycin B_3	Unknown	Unknown
Istamycin C	Unknown	Unknown
Istamycin C_0	Unknown	Unknown
Istamycin C_1	Unknown	Unknown
Istamycin KL_1	Unknown	Unknown
Istamycin X_0 (sannamycin G)	Unknown	Unknown
Istamycin Y_0 (sannamycin H)	Unknown	Unknown
Kanamycin (kanamycin A)	16S ribosomal RNA inhibitor<br>30S ribosomal subunit inhibitor<br>Ribosomal A-site inhibitor	von Ahsen *et al*., 1991<br>Masukawa *et al*., 1968; Tanaka, 1975; Campuzano *et al*., 1979; Le Goffic *et al*., 1980; Guo *et al*., 1981; Tanaka, 1983; Moreau *et al*., 1984<br>Matt *et al*., 2012
Kanamycin C	16S ribosomal RNA inhibitor<br>30S ribosomal subunit inhibitor	von Ahsen *et al*., 1991<br>Benveniste & Davies, 1973; Tanaka, 1975; Le Goffic *et al*., 1980
Kanamycin D	Unknown	Unknown
Kanamycin X	Unknown	Unknown
Lividamine (nebramycin IX)	Unknown	Unknown
Lividomycin	30S ribosomal subunit inhibitor	Tanaka, 1975; Guo *et al*., 1981; Tanaka, 1983
Lividomycin B (3'-deoxyparomomycin I)	30S ribosomal subunit inhibitor	Tanaka, 1975; Tanaka, 1983
Mannosylparomomycin	30S ribosomal subunit inhibitor	Tanaka, 1975
Micronomicin (gentamicin C_{2b})	30S ribosomal subunit inhibitor	Tanaka, 1983
Neamine (neomycin A; nebramycin X)	16S ribosomal RNA inhibitor<br>30S ribosomal subunit inhibitor	von Ahsen *et al*., 1991<br>Tanaka, 1975; Campuzano *et al*., 1979; Guo *et al*., 1981
Nebramine (nebramycin VIII)	30S ribosomal subunit inhibitor	Benveniste & Davies, 1973
Nebramycin III	30S ribosomal subunit inhibitor	Tanaka, 1975
Nebramycin IV	Unknown	Unknown
Nebramycin V'	Unknown	Unknown
Nebramycin XI	Unknown	Unknown
Nebramycin XII	Unknown	Unknown

Bioactive molecule	Primary target	Source
Nebramycin XIII	Unknown	Unknown
Neomycin C	30S ribosomal subunit inhibitor	Benveniste & Davies, 1973; Tanaka, 1975; Campuzano *et al.*, 1979; Tanaka, 1983
Neomycin F (paromomycin II)	30S ribosomal subunit inhibitor	Tanaka, 1975; Tanaka, 1983
Netilmicin	30S ribosomal subunit inhibitor	Guo *et al.*, 1981; Tanaka, 1983
NK 1001	30S ribosomal subunit inhibitor	Tanaka, 1975
Oxyapramycin (nebramycin VII)	Ribosomal A-site inhibitor	Mandhapati *et al.*, 2014
Paromamine (neomycin D)	30S ribosomal subunit inhibitor	Tanaka, 1975
Paromomycin (paromomycin I; neomycin E)	16S ribosomal RNA inhibitor<br>30S ribosomal subunit inhibitor	von Ahsen *et al.*, 1991<br>Benveniste & Davies, 1973; Tanaka, 1975; Campuzano *et al.*, 1979; Guo *et al.*, 1981; Tanaka, 1983
Pentisomicin	16S ribosomal RNA inhibitor<br>30S ribosomal subunit inhibitor	von Ahsen *et al.*,1992<br>Tanaka, 1983
Plazomicin	16S ribosomal RNA inhibitor	Livermore *et al.*,2010
Propikacin	30S ribosomal subunit inhibitor	Tanaka, 1983
Pyrankacin	Binding A-site of 16S RNA	Udumula *et al.*,2013
Ribostamycin	16S ribosomal RNA inhibitor<br>30S ribosomal subunit inhibitor	von Ahsen et al., 1991<br>Benveniste & Davies, 1973; Tanaka, 1975; Campuzano *et al.*, 1979; Le Goffic *et al.*, 1980; Guo *et al.*, 1981; Tanaka, 1983
Saccharocin (KA 5685)	Ribosomal A-site inhibitor	Mandhapati *et al.*, 2014
Sannamycin C	Unknown	Unknown
Sannamycin F	Unknown	Unknown
Sannamycin J	Unknown	Unknown
Sannamycin K	Unknown	Unknown
Sannamycin KR	Unknown	Unknown
Sannamycin L	Unknown	Unknown
Seldomycin	30S ribosomal subunit inhibitor	Le Goffic *et al.*, 1980
Seldomycin 1 (seldomycin factor 1)	Unknown	Unknown
Seldomycin 2 (seldomycin factor 2)	Unknown	Unknown
Seldomycin 3 (seldomycin factor 3)	Unknown	Unknown
Seldomycin 5 (seldomycin factor 5)	Unknown	Unknown
Sisomicin	30S ribosomal subunit inhibitor	Benveniste & Davies, 1973; Tanaka, 1975; Campuzano *et al.*, 1979; Guo et al., 1981; Tanaka, 1983
Sisomicin B	Unknown	Unknown
Sisomicin D	Unknown	Unknown
Spectinomycin	30S ribosomal subunit inhibitor	Davies *et al.*, 1965; Tanaka, 1975; Tanaka, 1983; Barry *et al.*, 1989
Sporaricin A	Unknown	Unknown
Sporaricin B	Unknown	Unknown
Sporaricin C	Unknown	Unknown
Sporaricin D	Unknown	Unknown
Sporaricin E	Unknown	Unknown
SS 56A	Unknown	Unknown
SS 56B	Unknown	Unknown
SS 56C	Unknown	Unknown
Streptomycin	Small ribosomal subunit inhibitor	Davies, 1964; Tanaka, 1975; Tanaka, 1983; Moreau *et al.*, 1984; Campuzano *et al.*, 1979
Streptoniazid (streptonicozid)	Unknown	Unknown
Tobramycin (nebramycin VI)	16S ribosomal RNA inhibitor<br>30S ribosomal subunit inhibitor<br>Ribosomal A-site inhibitor	von Ahsen *et al.*, 1991<br>Benveniste & Davies, 1973; Tanaka, 1975; Campuzano *et al.*, 1979; Guo *et al.*, 1981; Tanaka, 1983; Moreau *et al.*, 1984<br>Matt *et al.*, 2012
Trospectomycin	30S ribosomal subunit inhibitor	Barry *et al.*, 1989
Verdamicin	30S ribosomal subunit inhibitor	Campuzano *et al.*, 1979
Verdamicin C_2	Unknown	Unknown
Vertilmicin	Unknown	Unknown
7-Iodosancycline	30S ribosomal subunit inhibitor	Podlogar *et al.*, 2003
Amicycline	30S ribosomal subunit inhibitor	Sum, 2004
Apicycline	30S ribosomal subunit inhibitor	Alagarsamy, 2012
Bromotetracycline (bromtetracycline)	30S ribosomal subunit inhibitor	Alagarsamy, 2012
Chlortetracycline (chlorotetracycline)	30S ribosomal subunit inhibitor	Franklin, 1963; Podlogar *et al.*, 2003; Sum, 2004; Alagarsamy, 2012

Bioactive molecule	Primary target	Source
Clomocycline	30S ribosomal subunit inhibitor	Alagarsamy, 2012
Demeclocycline	30S ribosomal subunit inhibitor	Epe & Woolley, 1984; Sum, 2004
Demecycline	30S ribosomal subunit inhibitor	Sum, 2004
DMG-DMDOT (DMG-DM DOT)	30S ribosomal subunit inhibitor	Sum, 2004; Sum *et al*., 2006
DMG-MINO	30S ribosomal subunit inhibitor	Sum, 2004; Sum *et al*., 2006
Doxycycline	30S ribosomal subunit inhibitor	Podlogar *et al*., 2003; Sum, 2004; Alagarsamy, 2012
Eravacycline	30S ribosomal subunit inhibitor	Grossman *et al*., 2012
Etamocycline	Unknown	Unknown
Glycocycline	Unknown	Unknown
Guamecycline	30S ribosomal subunit inhibitor	Alagarsamy, 2012
Lymecycline	30S ribosomal subunit inhibitor	Alagarsamy, 2012
Meclocycline	30S ribosomal subunit inhibitor	Alagarsamy, 2012
Meglucycline	30S ribosomal subunit inhibitor	Alagarsamy, 2012
Metacycline (methacycline)	30S ribosomal subunit inhibitor	Sum, 2004; Alagarsamy, 2012
Minocycline	30S ribosomal subunit inhibitor	Podlogar *et al*., 2003; Sum, 2004; Sum *et al*., 2006; Alagarsamy, 2012
Morphocycline	Unknown	Unknown
Nitrocycline	Unknown	Unknown
Omadacycline	30S ribosomal subunit inhibitor	Draper *et al*., 2014; Sum, 2004
Oxytetracycline	30S ribosomal subunit inhibitor	Franklin, 1963; Podlogar *et al*., 2003; Sum, 2004; Alagarsamy, 2012
Pecocycline	Unknown	Unknown
Penimepicycline	Unknown	Unknown
Penimocycline	Unknown	Unknown
Pipacycline	30S ribosomal subunit inhibitor	Alagarsamy, 2012
Rolitetracycline	30S ribosomal subunit inhibitor	Alagarsamy, 2012
Sancycline	30S ribosomal subunit inhibitor	Podlogar *et al*., 2003; Alagarsamy, 2012
Sarecycline	Unknown	Unknown
Tetracycline	30S ribosomal subunit inhibitor	Franklin, 1963; Connamacher & Mandel, 1965; Podlogar *et al*., 2003; Sum, 2004; Alagarsamy, 2012
Tigecycline	30S ribosomal subunit inhibitor	Podlogar *et al*., 2003; Sum *et al*., 2006
TP 271	30S ribosomal subunit inhibitor	Grossman *et al*., 2017
Albaconazole	Sterol C14α-demethylase inhibitor	da Matta Guedes *et al*., 2004
Alteconazole	Unknown	Unknown
Azaconazole	Sterol 14α-demethylase inhibitor	Vanden Bossche *et al*., 1987
BAS 110	Obtusifoliol 14a-methyl demethylase inhibitor	Taton *et al*., 1988
BAS 111	Obtusifoliol 14a-methyl demethylase inhibitor	Taton *et al*., 1988
BAS 45406F	Unknown	Unknown
Bitertanol	Sterol C-14 demethylation inhibitor	Köller, 1987; De Waard & Van Nistelrooy, 1990
Bromuconazole	Sterol C-14 demethylation inhibitor	Roberts *et al*., 1999; Elliott, 1999
Cyproconazole	Sterol C-14 demethylation inhibitor	Leroux *et al*., 1988; Elliott, 1999
D 0870	Sterol C-14 demethylase inhibitor	Kojima et al., 1999
Diclobutrazol	Sterol C-14 demethylation inhibitor	Köller, 1987; Leroux *et al*., 1988; De Waard & Van Nistelrooy, 1990
Difenoconazole	Sterol C-14 demethylation inhibitor	Kunz *et al*., 1997; Roberts *et al*., 1999
Diniconazole	Sterol C-14 demethylation inhibitor	Köller, 1987; Leroux *et al*., 1988
Efinaconazole	Sterol 14α-demethylase inhibitor	Tatsumi *et al*., 2013
Embeconazole	Lanosterol 14α-demethylase inhibitor	Sorbera *et al*., 2003
Epoxiconazole	Sterol 14α-demethylase inhibitor	Akers *et al*., 1990
Etaconazole	Sterol C-14 demethylation inhibitor	Ebert *et al*., 1983; Köller, 1987; De Waard & Van Nistelrooy, 1990
Fenbuconazole	Sterol C-14 demethylation inhibitor	Reynolds *et al*., 1997; Elliott, 1999
Fluconazole	Lanosterol 14α-demethylase inhibitor<br>Sterol 14α-demethylase inhibitor<br>Lanosterol C-14 demethylation inhibitor<br>Sterol C-14 demethylation inhibitor	Tsuchimori *et al*., 2002<br>Vanden Bossche *et al*., 1987; Mellado *et al*., 2005; Warrilow *et al*., 2010; Warrilow *et al*., 2016<br>Barrett-Bee *et al*.,1988<br>Richardson *et al*., 1990; Fromtling, 1988
Fluotrimazole	Sterol C-14 demethylation inhibitor	Buchenauer, 1978
Fluquinconazole	Sterol C-14 demethylation inhibitor	Roberts *et al*., 1999
Flusilazole	Sterol C-14 demethylation inhibitor	Leroux *et al*., 1988; Kunz *et al*., 1997
Flutriafol	Sterol C-14 demethylation inhibitor	Köller, 1987; Leroux et al., 1988
Fosfluconazole	Cytochrome P450 sterol C-14 alpha-demethylation inhibitor	Sobue *et al*., 2004
Furconazole	Sterol C-14 demethylation inhibitor	Ziogas *et al*., 2015
Genaconazole (SCH 39304)	Sterol 14α-demethylase inhibitor	Venkateswarlu & Kelly, 1997; Bartroli *et al*., 1995

Bioactive molecule	Primary target	Source
Hexaconazole	Sterol C-14 demethylation inhibitor	Leroux *et al*., 1988
ICI 153066	Sterol 14α-demethylase inhibitor<br>Lanosterol C-14-demethylation inhibitor	Hitchcock *et al*., 1987<br>Barrett-Bee *et al*., 1989
ICI 195739	Sterol 14α-demethylase inhibitor	Barrett-Bee et al.,1988; Bartroli et al., 1995; Fromtling, 1988
Imibenconazole	Sterol C-14 demethylation inhibitor	Roberts *et al*., 1999
Ipconazole	Sterol C-14 demethylation inhibitor	Saishoji *et al*., 1998
Ipfentrifluconazole	Unknown	Unknown
Isavuconazole	Cytochrom P450 sterol 14α-demethylase (CYP51) inhibitor	Guinea & Bouza, 2008
Itraconazole	Lanosterol 14α-demethylase inhibitor<br>Sterol 14α-demethylase inhibitor<br>Sterol C-14 demethylation inhibitor	Tsuchimori *et al*., 2002<br>Vanden Bossche *et al*., 1987; Mellado *et al*., 2005; Warrilow *et al*., 2010; Warrilow *et al*., 2016<br>Fromtling, 1988
LAB 158241F	Obtusifoliol 14a-methyl demethylase inhibitor	Taton *et al*., 1988
LAB 170250F	Obtusifoliol 14a-methyl demethylase inhibitor	Taton *et al*., 1988
Mefentrifluconazole	Unknown	Unknown
Metconazole	Cytochrome P450 C-14 demethylation inhibitor	Ito *et al*., 1999
Myclobutanil	Sterol 14-demethylase inhibitor<br>Sterol C-14 demethylation inhibitor	Quinn *et al*., 1986<br>Elliott, 2000
Penconazole	Sterol 14α-demethylase inhibitor<br>Sterol C-14 demethylation inhibitor	Vanden Bossche *et al*., 1987<br>Köller, 1987; Leroux *et al*., 1988; De Waard & Van Nistelrooy, 1990
Posaconazole	Sterol C-14 demethylation inhibitor	Munayyer et al., 1996
PP 969	Unknown	Unknown
Pramiconazole	Sterol 14α-demethylase inhibitor	Vanden Bossche *et al*., 2004
Propiconazole	Obtusifoliol 14a-methyl demethylase inhibitor<br>Sterol 14α-demethylase inhibitor<br>Sterol C-14 demethylation inhibitor	Taton *et al*., 1988<br>Vanden Bossche et al., 1987<br>Köller, 1987; Leroux *et al*., 1988; De Waard & Van Nistelrooy, 1990; Reynolds *et al*., 1997; Elliott, 1999
Quinconazole	Sterol 14α-demethylase inhibitor	Kapteyn *et al*., 1994
Ravuconazole	Sterol 14α-demethylase inhibitor	Mellado *et al*., 2005
Saperconazole	Sterol 14α-demethylase inhibitor	Vanden Bossche *et al*., 1989
SCH 42427<br>SCH 42427	C14 lanosterol demethylase inhibitor<br>Sterol 14α-demethylase inhibitor	Hesk *et al*., 1992<br>Bartroli *et al*., 1996
SCH 51048	Sterol 14α-demethylase inhibitor	Bartroli *et al*., 1995; Lovey *et al*., 2002
SDZ 89-485	Lanosterol 14-demethylation inhibitor	Ryder, 1990
Simeconazole	Sterol C-14 demethylation inhibitor	Tsuda *et al*., 2004
SSF 109 (huanjunzuo)	Sterol 14α-demethylase inhibitor	Shirane *et al*., 1990
SSY 726	Unknown	Unknown
SYN 2506	Unknown	Unknown
SYN 2836	Unknown	Unknown
SYN 2869	Unknown	Unknown
SYN 2903	Unknown	Unknown
SYN 2921	Unknown	Unknown
T 8581	Unknown	Unknown
TAK 187	Sterol 14α-demethylase inhibitor	Tasaka *et al*., 1997
TAK 456	Lanosterol 14α-demethylase inhibitor	Tsuchimori *et al*., 2002
Tebuconazole	Sterol C-14 demethylation inhibitor	Leroux *et al*., 1988; De Waard & Van Nistelrooy, 1990; Kunz *et al*., 1997; Elliott, 1999
Terconazole (triaconazole)	Sterol 14α-demethylase inhibitor	Pfaller *et al*., 1990
Tetraconazole	Sterol 14α-demethylase inhibitor	Gozzo *et al*., 1995
Triadimefon	Sterol C-14 demethylation inhibitor	Barug & Kerkenaar, 1984; Köller, 1987; Leroux *et al*., 1988
Triadimenol	Sterol C-14 demethylation inhibitor	Leroux et al., 1988; Köller, 1987
Triticonazole	Sterol demethylation inhibitor	Mugnier *et al*., 1993
UK 47265	Sterol C-14 demethylation inhibitor	Richardson et al., 1988
UK 51486	Unknown	Unknown
Uniconazole	Sterol C-14 demethylation inhibitor	Köller, 1987
UR 9746	Sterol 14α-demethylase inhibitor	Bartroli *et al*., 1995
UR 9751	Sterol 14α-demethylase inhibitor	Bartroli *et al*., 1995
Vibunazole (BAY N-7133)	Sterol C-14 demethylation inhibitor	Köller, 1987; Fromtling, 1988
Voriconazole	Lanosterol 14α-demethylase inhibitor<br>Sterol 14α-demethylase inhibitor	Tsuchimori *et al*., 2002<br>Sanati *et al*., 1997; Mellado *et al*., 2005; Warrilow *et al*., 2010; Warrilow *et al*., 2016

Bioactive molecule	Primary target	Source
YH 1715R	Lanosterol 14α-demethylase inhibitor	Park *et al*., 2004
1-Dodecylimidazole (N-dodecylimidazole)	Sterol C14 demethylation inhibitor	Henry& Sisler, 1979
AFK 108	Lanosterol 14α-demethylase inhibitor	Yuri *et al*.,1992
Aliconazole	Unknown	Unknown
Arasertaconazole	Unknown	Unknown
Azalanstat	Lanosterol 14α-demethylase inhibitor	Burton *et al*., 1995
BAY C-9263	Unknown	Unknown
BAY D-9603	Unknown	Unknown
Becliconazole	Unknown	Unknown
Bifonazole	Sterol 14α-demethylase inhibitor<br>Sterol C-14 demethylation inhibitor	Vanden Bossche *et al*., 1987; Niwano *et al*., 1999<br>Köller, 1987; Fromtling, 1988
Brolaconazole	Unknown	Unknown
Butoconazole	Sterol C-14 demethylation inhibitor	Pye & Marriott, 1982; Fromtling, 1988
Cisconazole	Unknown	Unknown
Climbazole	Sterol 14α-demethylase inhibitor	Barug & Kerkenaar, 1984
Clotrimazole	Sterol 14α-demethylase inhibitor<br>Sterol C-14 demethylation inhibitor	Vanden Bossche *et al*., 1987; Warrilow et al., 2016<br>Buchenauer, 1978; Pye & Marriott, 1982; Köller, 1987; Fromtling, 1988; Leroux *et al*., 1988; Warrilow *et al*., 2010
Croconazole	Sterol C-14 demethylation inhibitor	Hiratani & Yamaguchi, 1985; Fromtling, 1988
Democonazole	Unknown	Unknown
Dichlorophenyl imidazoldioxolan (elubiol)	Sterol 14α-demethylase inhibitor	Ries & Hess, 2000
Doconazole	Unknown	Unknown
Eberconazole	Lanosterol 14α-demethylase inhibitor	Moodahadu *et al*., 2014
Econazole	Sterol 14α-demethylase inhibitor<br>Sterol C-14 demethylation inhibitor	Warrilow *et al*., 2010<br>Pye & Marriott, 1982; Fromtling, 1988
Fenapanil	Sterol C-14 demethylation inhibitor	Köller, 1987; De Waard & Van Nistelrooy, 1990
Fenticonazole	Sterol 14α-demethylase inhibitor	Griffith, 2012
Flutrimazole	Lanosterol 14 alpha-demethylase inhibitor	García et al., 1992
Imazalil (enilconazole)	Sterol 14α-demethylase inhibitor<br>Sterol C-14 demethylation inhibitor	Vanden Bossche *et al*., 1987<br>Barug & Kerkenaar, 1984; Köller, 1987; Leroux et al., 1988
Isoconazole	Sterol 14α-demethylase inhibitor	Veraldi, 2013; Griffith, 2012
Ketaminazole	Sterol 14α-demethylase inhibitor	Warrilow et al., 2016
Ketoconazole	Lanosterol C-14 demethylation inhibitor<br>Sterol 14α-demethylase inhibitor<br>Sterol C-14 demethylation inhibitor	Barrett-Bee *et al*.,1988<br>Vanden Bossche *et al*., 1987; Warrilow *et al*., 2010; Warrilow *et al*., 2016<br>Pye & Marriott, 1982; Köller, 1987; Fromtling, 1988; Mellado *et al*., 2005
Lanoconazole	Sterol 14α-demethylase inhibitor<br>Sterol C-14 demethylation inhibitor	Niwano *et al*., 1999<br>Ohmi *et al*., 1992
Lombazole	Sterol C-14 demethylation inhibitor	Barug & Bastiaanse, 1985; Barug & Kerkenaar, 1984
Luliconazole	Sterol 14α-demethylase inhibitor	Niwano *et al*., 1999
MH 0685	Unknown	Unknown
Miconazole	Sterol 14α-demethylase inhibitor<br>Sterol C-14 demethylation inhibitor	Vanden Bossche *et al*., 1987; Warrilow *et al*., 2010<br>Pye & Marriott, 1982; Köller, 1987; Fromtling, 1988
Neticonazole	Sterol 14α-demethylase inhibitor	Griffith, 2012
OK 8705	Sterol 14α-demethylase inhibitor	Imai *et al*., 1993
OK 8801	Sterol 14α-demethylase inhibitor	Imai *et al*., 1993
Omoconazole	Sterol 14α-demethylase inhibitor	Griffith, 2012
Orconazole	Unknown	Unknown
Oxiconazole	Sterol 14α-demethylase inhibitor	Griffith, 2012
Oxpoconazole	Lanosterol 14α-demethylase ($P450_{14DM}$)	Hayashi *et al*., 2003
Parconazole	Sterol C-14 demethylation inhibitor	Pye & Marriott, 1982
Pefurazoate	Sterol C-14 demethylation inhibitor	Wada *et al*., 1991
PR 967-234	Sterol 14α-demethylase inhibitor	Padeiskaya & Baklanova, 1993
Prochloraz	Sterol 14α-demethylase inhibitor<br>Sterol C-14 demethylation inhibitor	Kapteyn *et al*., 1994<br>Leroux *et al*., 1989
R 31000	Unknown	Unknown
Sertaconazole	Ergosterol synthesis inhibitor	Agut *et al*., 1992
SM 4470	Sterol 14α-demethylase inhibitor	Padeiskaya & Baklanova, 1993
SSF 105	Unknown	Unknown
Sulconazole	Sterol 14α-demethylase inhibitor	Griffith, 2012
Tioconazole	Sterol 14α-demethylase inhibitor	Pye & Marriott, 1982; Köller, 1987; Fromtling, 1988

Bioactive molecule	Primary target	Source
Triflumizole	Sterol C-14 demethylation inhibitor	Leroux *et al*., 1988
UK 38667	Unknown	Unknown
Valconazole	Unknown	Unknown
Zinoconazole	Sterol 14α-demethylase inhibitor	Zampieri et al., 2009
Zoficonazole	Unknown	Unknown

References

Agut, J., C. Palacín, A. Sacristan & J. A. Ortiz (1992) Inhibition of ergosterol synthesis by sertaconazole in *Candida albicans*. *Arzneimittel-Forschung,* 42**,** 718-720.

Akers, A., H. H. Köhle & R. E. Gold. 1990. Uptake, transport and mode of action of BAS 480 F, a new triazole fungicide. In *Brighton Crop Protection Conference, Pests and Diseases*, 837-845. British Crop Protection Council.

Alagarsamy, V. 2012. *Textbook of Medicinal Chemistry Vol II-E-Book*. Elsevier Health Sciences.

Albertini, S., A.-A. Chételat, B. Miller, W. Muster, E. Pujadas, R. Strobel & E. Gocke (1995) Genotoxicity of 17 gyrase-and four mammalian topoisomerase II-poisons in prokaryotic and eukaryotic test systems. *Mutagenesis,* 10**,** 343-351.

Al-Lahham, A., N. J. de Souza, M. Patel & R. René Reinert (2005) Activity of the new quinolones WCK 771, WCK 1152 and WCK 1153 against clinical isolates of *Streptococcus pneumoniae* and *Streptococcus pyogenes*. *Journal of Antimicrobial Chemotherapy,* 56**,** 1130-1133.

Alovero, F., M. Nieto, M. R. Mazzieri, R. Then & R. H. Manzo (1998) Mode of action of sulfanilyl fluoroquinolones. *Antimicrobial Agents and Chemotherapy,* 42**,** 1495-1498.

Bald, R., V. A. Erdmann & O. Pongs (1972) Irreversible binding of chloramphenicol analogues to *E. coli* ribosomes. *FEBS Letters,* 28**,** 149-152.

Baradaran-Heravi, A., J. Niesser, A. D. Balgi, K. Choi, C. Zimmerman, A. P. South, H. J. Anderson, N. C. Strynadka, M. B. Bally & M. Roberge (2017) Gentamicin B1 is a minor gentamicin component with major nonsense mutation suppression activity. *Proceedings of the National Academy of Sciences of the United States of America,* 114**,** 3479–3484.

Barrett, J. F., T. D. Gootz, P. R. McGuirk, C. A. Farrell & S. A. Sokolowski (1989) Use of *in vitro* topoisomerase II assays for studying quinolone antibacterial agents. *Antimicrobial Agents and Chemotherapy,* 33**,** 1697-1703.

Barrett-Bee, K., J. Lees, P. Pinder, J. Campbell & L. Newboult (1988) Biochemical studies with a novel antifungal agent, ICI 195,739. *Annals of the New York Academy of Sciences,* 544**,** 231-244.

Barry, A. L., R. N. Jones & C. Thornsberry (1989) Antibacterial activity of trospectomycin (U-63366F) and initial evaluations of disk diffusion susceptibility tests. *Antimicrobial Agents and Chemotherapy,* 33**,** 569-572.

Bartroli, J., E. Turmo, M. Alguero, E. Boncompte, M. L. Vericat, J. Garcia-Rafanell & J. Forn (1995) Synthesis and antifungal activity of new azole derivatives containing an N-acylmorpholine ring. *Journal of Medicinal Chemistry,* 38**,** 3918-3932.

Barug, D. & H. B. Bastiaanse (1985) On the antibacterial mode of action of lombazole. *Antonie van Leeuwenhoek,* 51**,** 582-583.

Barug, D. & A. Kerkenaar (1984) Resistance in mutagen-induced mutants of *Ustilago maydis* to fungicides which inhibit ergosterol biosynthesis. *Pest Management Science,* 15**,** 78-84.

Bazile, S., N. Moreau, D. Bouzard & M. Essiz (1992) Relationships among antibacterial activity, inhibition of DNA gyrase, and intracellular accumulation of 11 fluoroquinolones. *Antimicrobial Agents and Chemotherapy,* 36**,** 2622-2627.

Benveniste, R. & J. Davies (1973) Structure-activity relationships among the aminoglycoside antibiotics: role of hydroxyl and amino groups. *Antimicrobial Agents and Chemotherapy,* 4**,** 402-409.

Bhagwat, S. S., L. A. Mundkur, S. V. Gupte, M. V. Patel & H. F. Khorakiwala (2006) The anti-methicillin-resistant Staphylococcus aureus quinolone WCK 771 has potent activity against sequentially selected mutants, has a narrow mutant selection window against quinolone-resistant *Staphylococcus aureus*, and preferentially targets DNA gyrase. *Antimicrobial Agents and Chemotherapy,* 50**,** 3568-3579.

Brighty, K. E. & T. D. Gootz (1997) The chemistry and biological profile of trovafloxacin. *Journal of Antimicrobial Chemotherapy,* 39**,** 1-14.

Buchenauer, H. (1978) Analogy in the mode of action of fluotrimazole and clotrimazole in *Ustilago avenae*. *Pesticide Biochemistry and Physiology,* 8**,** 15-25.

Burton, P. M., D. C. Swinney, R. Heller, B. Dunlap, M. Chiou, E. Malonzo, J. Haller, K. A. M. Walker, A. Salari, S. Murakami, G. Mendizabal & L. Tokes (1995) Azalanstat (RS-21607), a lanosterol 14α-demethylase inhibitor with cholesterol-lowering activity. *Biochemical Pharmacology,* 50**,** 529-544.

Butler, M. S., M. A. Blaskovich & M. A. Cooper (2013) Antibiotics in the clinical pipeline in 2013. *Journal of Antibiotics,* 66**,** 571-591.

Campuzano, S., D. Vázquez & J. Modolell (1979) Functional interaction of neomycin B and related antibiotics with 30S and 50S ribosomal subunits. *Biochemical and Biophysical Research Communications,* 87**,** 960-966.

Cecchetti, V., A. Fravolini, M. C. Lorenzini, O. Tabarrini, P. Terni & T. Xin (1996) Studies on 6-aminoquinolones: synthesis and antibacterial evaluation of 6-amino-8-methylquinolones. *Journal of Medicinal Chemistry,* 39**,** 436-445.

Celma, M. L., R. E. Monro & D. Vazquez (1970) Substrate and antibiotic binding sites at the peptidyl transferase centre of *E. coli* ribosomes. *FEBS letters,* 6**,** 273-277.

Cheng, J., J. A. Thanassi, C. L. Thoma, B. J. Bradbury, M. Deshpande & M. J. Pucci (2007) Dual targeting of DNA gyrase and topoisomerase IV: target interactions of heteroaryl isothiazolones in *Staphylococcus aureus*. *Antimicrobial Agents and Chemotherapy,* 51**,** 2445-2453.

Choi, K.-H., M.-C. Baek, B.-K. Kim & E.-C. Choi (1998) Resistance mechanism of *Acinetobacter* spp. strains resistant to DW-116, a new quinolone. *Archives of Pharmacal Research,* 21**,** 310-314.

Connamacher, R. H. & H. G. Mandel (1965) Binding of tetracycline to the 30S ribosomes and to polyuridylic acid. *Biochemical and Biophysical Research Communications,* 20**,** 98-103.

Coughlin, S. A., D. W. Danz, R. G. Robinson, K. M. Klingbeil, M. P. Wentland, T. H. Corbett, W. R. Waud, L. A. Zwelling, E. Altschuler, E. Bales & J. B. Rake (1995) Mechanism of action and antitumor activity of (*S*)-10-(2, 6-dimethyl-4-pyridinyl)-9-fluoro-3-methyl-7-oxo-2, 3-dihydro-7*H*-pyridol [1, 2, 3-*de*]-[1, 4] benzothiazine-6-carboxylic acid (WIN 58161). *Biochemical Pharmacology,* 50**,** 111-122.

da Matta Guedes, P. M., J. A. Urbina, M. de Lana, L. C. Afonso, V. M. Veloso, W. L. Tafuri, G. L. Machado-Coelho, E. Chiari & M. T. Bahia (2004) Activity of the new triazole derivative albaconazole against *Trypanosoma* (*Schizotrypanum*) *cruzi* in dog hosts. *Antimicrobial Agents and Chemotherapy,* 48**,** 4286-4292.

Davies, J., P. Anderson & B. D. Davis (1965) Inhibition of protein synthesis by spectinomycin. *Science,* 149**,** 1096-1098.

Davies, J. E. (1964) Studies on the ribosomes of streptomycin-sensitive and resistant strains of *Escherichia coli*. *Proceedings of the National Academy of Sciences of the United States of America,* 51**,** 659-664.

de Souza, N. J., S. V. Gupte, P. K. Deshpande, V. N. Desai, S. B. Bhawsar, R. D. Yeole, M. C. Shukla, J. Strahilevitz, D. C. Hooper, B. Bozdogan, P. C. Appelbaum, M. R. Jacobs, N. Shetty, M. V. Patel, R. Jha & H. F. Khorakiwala (2005) A Chiral Benzoquinolizine-2-carboxylic Acid Arginine Salt Active against Vancomycin-Resistant *Staphylococcus aureus*. *Journal of Medicinal Chemistry,* 48**,** 5232-5242.

De Waard, M. A. & J. G. M. Van Nistelrooy (1990) Stepwise development of laboratory resistance to DMI-fungicides in *Penicillium italicum*. *Netherlands Journal of Plant Pathology,* 96**,** 321-329.

Domagala, J. M. (1994) Structure-activity and structure-side-effect relationships for the quinolone antibacterials. *Journal of Antimicrobial Chemotherapy,* 33**,** 685-706.

Domagala, J. M., L. D. Hanna, C. L. Heifetz, M. P. Hutt, T. F. Mich, J. P. Sanchez & M. Solomon (1986) New structure-activity relationships of the quinolone antibacterials using the target enzyme. The development and application of a DNA gyrase assay. *Journal of medicinal chemistry,* 29**,** 394-404.

Draper, M. P., S. Weir, A. Macone, J. Donatelli, C. A. Trieber, S. K. Tanaka & S. B. Levy (2014) Mechanism of action of the novel aminomethylcycline antibiotic omadacycline. *Antimicrobial Agents and Chemotherapy,* 58**,** 1279-1283.

Ebert, E., J. Gaudin, W. Muecke, K. Ramsteiner, C. Vogel & H. Führer (1983) Inhibition of ergosterol biosynthesis by etaconazole in *Ustilago maydis*. *Zeitschrift*

für Naturforschung - Section C Journal of Biosciences, 38**,** 28-34.
Elliott, M. (1999) Effect of Demethylation Inhibiting Fungicides on 'Tifgreen' Bermudagrass Quality. *HortTechnology,* 9**,** 195-197.
Epe, B. & P. Woolley (1984) The binding of 6-demethylchlortetracycline to 70S, 50S and 30S ribosomal particles: a quantitative study by fluorescence anisotropy. *EMBO Journal,* 3**,** 121-126.
Fecik, R. A., P. Devasthale, S. Pillai, A. Keschavarz-Shokri, L. Shen & L. A. Mitscher (2005) Chiral DNA Gyrase Inhibitors. 3. Probing the Chiral Preference of the Active Site of DNA Gyrase. Synthesis of 10-Fluoro-6-methyl-6,7-dihydro-9-piperazinyl-2*H*-benzo[*a*]quinolizin-20-one-3-carboxylic Acid Analogues. *Journal of Medicinal Chemistry,* 48**,** 1229-1236.
Feduchi, E., M. Cosin & L. Carrasco (1985) Mildiomycin: a nucleoside antibiotic that inhibits protein synthesis. *Journal of Antibiotics,* 38**,** 415-419.
Franklin, T. J. (1963) The inhibition of incorporation of leucine into protein of cell-free systems from rat liver and *Escherichia coli* by chlortetracycline. *Biochemical Journal,* 87**,** 449-453.
Fromtling, R. A. (1988) Overview of medically important antifungal azole derivatives. *Clinical Microbiology Reviews,* 1**,** 187-217.
Fu, J., S. Laval & B. Yu (2018) Total Synthesis of Nucleoside Antibiotics Plicacetin and Streptcytosine A. *Journal of Organic Chemistry*.
García, R. J., M. A. Dronda, M. Merlos, J. Forn, J. M. Torres, M. I. Zapatero & N. Basi (1992) *In vitro* and *in vivo* studies with flutrimazole, a new imidazole derivative with antifungal activity. *Arzneimittel-Forschung,* 42**,** 836-840.
Gellert, M., K. Mizuuchi, M. H. O'Dea, T. Itoh & J.-I. Tomizawa (1977) Nalidixic acid resistance: a second genetic character involved in DNA gyrase activity. *Proceedings of the National Academy of Sciences of the United States of America,* 74**,** 4772-4776.
Georgopapadakou, N. H., B. A. Dix, P. Angehrn, A. Wick & G. L. Olson (1987) Monocyclic and tricyclic analogs of quinolones: mechanism of action. *Antimicrobial Agents and Chemotherapy,* 31**,** 614-616.
Georgopoulos, A., R. Schein, A. Buxbaum, S. Tzotzos, A. Hirschl & W. Graninger (1998) Mechanisms of quinolone resistance in clinical isolates of *Enterobacter cloacae*. *Clinical Microbiology and Infection,* 4**,** 75-81.
Gootz, T. D., P. R. McGuirk, M. S. Moynihan & S. L. Haskell (1994) Placement of alkyl substituents on the C-7 piperazine ring of fluoroquinolones: dramatic differential effects on mammalian topoisomerase II and DNA gyrase. *Antimicrobial Agents and Chemotherapy,* 38**,** 130-133.
Gould, S. J. 1997. Blasticidin S and related peptidyl nucleoside antibiotics. In *Biotechnology of Antibiotics,* ed. W. R. Strohl, 703-731. New York: Marcel Dekker, Inc.
Gozzo, F., A. Carelli, R. Carzaniga, G. Farina, A. Arnoldi, D. Lamb & S. L. Kelly (1995) Stereoselective interaction of tetraconazole with 14α-demethylase in fungi. *Pesticide Biochemistry and Physiology,* 53**,** 10-22.
Griffith, R. K. 2012. Antifungal Agents. In *Foye's principles of medicinal chemistry : Seventh edition,* eds. T. L. Lemke, D. A. Williams, V. F. Roche & S. W. Zito, 1158-1174. Lippincott Williams & Wilkins.
Grossman, T. H., C. Fyfe, W. O'Brien, M. Hackel, M. B. Minyard, K. B. Waites, J. Dubois, T. M. Murphy, A. M. Slee, W. J. Weiss & J. A. Sutcliffe (2017) Fluorocycline TP-271 Is Potent against Complicated Community-Acquired Bacterial Pneumonia Pathogens. *mSphere,* 2**,** e00004-17.
Grossman, T. H., A. L. Starosta, C. Fyfe, W. O'Brien, D. M. Rothstein, A. Mikolajka, D. N. Wilson & J. A. Sutcliffe (2012) Target-and resistance-based mechanistic studies with TP-434, a novel fluorocycline antibiotic. *Antimicrobial Agents and Chemotherapy,* 56**,** 2559-2564.
Guinea, J. & E. Bouza (2008) Isavuconazole: a new and promising antifungal triazole for the treatment of invasive fungal infections. *Future Microbiology,* 3**,** 603-615.
Guo, L., Y. Wan, X. Wang, P. George Wang & W. Zhao (2012) Development of aminoglycoside antibiotics by carbohydrate chemistry. *Mini-Reviews in Medicinal Chemistry,* 12**,** 1533-1541.
Han, Q., Q. Zhao, S. Fish, K. B. Simonsen, D. Vourloumis, J. M. Froelich, D. Wall & T. Hermann (2005) Molecular recognition by glycoside pseudo base pairs and triples in an apramycin–RNA complex. *Angewandte Chemie - International Edition,* 44**,** 2694-2700.
Hayashi, K., H. j. Schoonbeek & M. A. De Waard (2003) Modulators of membrane drug transporters potentiate the activity of the DMI fungicide oxpoconazole against *Botrytis cinerea*. *Pest Management Science,* 59**,** 294-302.
Henry, M. J. & H. D. Sisler (1979) Effects of miconazole and dodecylimidazole on sterol biosynthesis in *Ustilago maydis*. *Antimicrobial Agents and Chemotherapy,* 15**,** 603-607.
Hesk, D., C. Bowlen, T. Duelfer, D. Koharski, P. McNamara & S. Saluja (1992) Synthesis of SCH 42427 labelled with ^{14}C in two different positions. *Journal of Labelled Compounds and Radiopharmaceuticals,* 31**,** 445-454.
Higuchi, S., Y. Onodera, M. Chiba, K. Hoshino & N. Gotoh (2013) Potent *in vitro* antibacterial activity of DS-8587, a novel broad-spectrum quinolone, against *Acinetobacter baumannii*. *Antimicrobial Agents and Chemotherapy,* 57**,** 1978-1981.
Hiratani, T. & H. Yamaguchi (1985) Studies on the mechanism of action of a new imidazole antimycotic 710674-S. *Chemotherapy,* 33**,** 579-591.
Hitchcock, C. A., K. J. Barrett-Bee & N. J. Russell (1987) Inhibition of 14 α-sterol demethylase activity in *Candida albicans* Darlington does not correlate with resistance to azole. *Journal of Medical and Veterinary Mycology,* 25**,** 329-333.
Hoshino, K., K. Inoue, Y. Murakami, Y. Kurosaka, K. Namba, Y. Kashimoto, S. Uoyama, R. Okumura, S. Higuchi & T. Otani (2008) *In vitro* and *in vivo* antibacterial activities of DC-159a, a new fluoroquinolone. *Antimicrobial Agents and Chemotherapy,* 52**,** 65-76.
Hoshino, K., A. Kitamura, I. Morrissey, K. Sato, J.-I. Kato & H. Ikeda (1994) Comparison of inhibition of *Escherichia coli* topoisomerase IV by quinolones with DNA gyrase inhibition. *Antimicrobial Agents and Chemotherapy,* 38**,** 2623-2627.
Hoshino, K., K. Sato, K. Akahane, A. Yoshida, I. Hayakawa, M. Sato, T. Une & Y. Osada (1991) Significance of the methyl group on the oxazine ring of ofloxacin derivatives in the inhibition of bacterial and mammalian type II topoisomerases. *Antimicrobial Agents and Chemotherapy,* 35**,** 309-312.
Imai, T., T. Uchida, K. Yamaguchi, H. Takao & T. Goto (1993) Preparation of enantiomers of the new imidazole fungicides OK-8705 and OK-8801 and their antifungal activity against *Botrytis einerea* and *Gibberella fujikuroi*. *Journal of Pesticide Science,* 18**,** 375-380.
Ince, D. & D. C. Hooper (2000) Mechanisms and frequency of resistance to premafloxacin in *Staphylococcus aureus*: novel mutations suggest novel drug-target interactions. *Antimicrobial Agents and Chemotherapy,* 44**,** 3344-3350.
Ito, A., T. Saishoji, S. Kumazawa & H. Chuman (1999) Structure-activity relationships of the azole fungicide metconazole and its related azolylmethylcycloalkanols. *Journal of Pesticide Science,* 24**,** 262-269.
Ito, T., M. Otsuki & T. Nishino (1992) *In vitro* antibacterial activity of Q-35, a new fluoroquinolone. *Antimicrobial Agents and Chemotherapy,* 36**,** 1708-1714.
Kapteyn, J. C., R. J. Milling, D. J. Simpson & M. A. De Waard (1994) Inhibition of sterol biosynthesis in cell-free extracts of *Botrytis cinerea* by prochloraz and prochloraz analogues. *Pest Management Science,* 40**,** 313-319.
Kazamori, D., H. Aoi, K. Sugimoto, T. Ueshima, H. Amano, K. Itoh, Y. Kuramoto & A. Yazaki (2014) *In vitro* activity of WQ-3810, a novel fluoroquinolone, against multidrug-resistant and fluoroquinolone-resistant pathogens. *International Journal of Antimicrobial Agents,* 44**,** 443-449.
Kim, H. Y., J. A. Wiles, Q. Wang, G. C. Pais, E. Lucien, A. Hashimoto, D. M. Nelson, J. A. Thanassi, S. D. Podos, M. Deshpande, M. J. Pucci & B. J. Bradbury (2011) Exploration of the activity of 7-pyrrolidino-8-methoxyisothiazoloquinolones against methicillin-resistant *Staphylococcus aureus* (MRSA). *Journal of Medicinal Chemistry,* 54**,** 3268-3282.
Kinoshita, T., N. Tanaka & H. Umezawa (1970) Binding of blasticidin S to ribosomes. *Journal of Antibiotics,* 23**,** 288-290.
Kishii, R., M. Takei, H. Fukuda, K. Hayashi & M. Hosaka (2003) Contribution of the 8-methoxy group to the activity of gatifloxacin against type II topoisomerases of *Streptococcus pneumoniae*. *Antimicrobial Agents and Chemotherapy,* 47**,** 77-81.
Kishii, R., Y. Yamaguchi & M. Takei (2017) *In vitro* activities and spectrum of the novel fluoroquinolone lascufloxacin (KRP-AM1977). *Antimicrobial Agents and Chemotherapy,* 61**,** e00120-17.
Kitamura, A., K. Hoshino, Y. Kimura, I. Hayakawa & K. Sato (1995) Contribution of the C-8 substituent of DU-6859a, a new potent fluoroquinolone, to its activity against DNA gyrase mutants of *Pseudomonas aeruginosa*. *Antimicrobial Agents and Chemotherapy,* 39**,** 1467-1471.
Köerber, B., E. Luhmer, H.-G. Wetzstein & P. Heisig. 2002. Bactericidal mechanisms of pradofloxacin, a novel 8-cyanofluoroquinolone. In *Program and Abstracts of the 42nd Interscience Conference on Antimicrobial Agents and Chemotherapy,* 188. San Diego, CA: American Society for Microbiology.
Kojima, M., H. Yamada, S. Nakamura, M. Kikuchi, T. Nakanishi, Y. Takahashi, H. Mochizuki & Y. Mikami (1999) Antifungal Activities of D0870 against Fluconazole-Resistant *Candida albicans*. *Nippon Ishinkin Gakkai Zasshi,* 40**,** 209-215.

Köller, W. (1987) Isomers of sterol synthesis inhibitors: fungicidal effects and plant growth regulator activities. *Pest Management Science,* 18**,** 129-147.
Kondo, J., B. François, R. J. M. Russell, J. B. Murray & E. Westhof (2006) Crystal structure of the bacterial ribosomal decoding site complexed with amikacin containing the γ-amino-α-hydroxybutyryl (haba) group. *Biochimie,* 88**,** 1027-1031.
Kotera, Y., M. Inoue & S. Mitsuhashi (1990) Activity of KB-5246 against outer membrane mutants of *Escherichia coli* and *Salmonella typhimurium*. *Antimicrobial Agents and Chemotherapy,* 34**,** 1323-1325.
Kunz, S., H. Deising & K. Mendgen (1997) Acquisition of resistance to sterol demethylation inhibitors by populations of *Venturia inaequalis*. *Phytopathology,* 87**,** 1272-1278.
Lackie, J. 2010. Glutarimide antibiotics. In *A dictionary of biomedicine,* ed. C. A. O'Callaghan, 608. Oxford University Press.
Le Goffic, F., M.-L. Capmau, E. Tangy & E. Caminade (1980) Have deoxystreptamine aminoglycoside antibiotics the same binding site on bacterial ribosomes? *Journal of Antibiotics,* 33**,** 895-899.
Leroux, P., M. Gredt & P. Boeda (1988) Resistance to inhibitors of sterol biosynthesis in field isolates or laboratory strains of the eyespot pathogen *Pseudocercosporella herpotrichoides*. *Pest Management Science,* 23**,** 119-129.
Li, L., J. Wu, Z. Deng, T. M. Zabriskie & X. He (2013) *Streptomyces lividans* blasticidin S deaminase and its application in engineering a blasticidin S-producing strain for ease of genetic manipulation. *Applied and Environmental Microbiology,* 79**,** 2349-2357.
Lichtenthaler, F. W., J. Černá & I. Rychlik (1975) The effect of oxamicetin and some amicetin analogs on ribosomal peptidyl transferase. *FEBS letters,* 53**,** 184-187.
Livermore, D. M., S. Mushtaq, M. Warner, J.-C. Zhang, S. Maharjan, M. Doumith & N. Woodford (2010) Activity of aminoglycosides, including ACHN-490, against carbapenem-resistant Enterobacteriaceae isolates. *Journal of Antimicrobial Chemotherapy,* 66**,** 48-53.
Lovey, R. G., A. K. Saksena, V. M. Girijavallabhan, P. Blundell, H. Guzik, D. Loebenberg, R. M. Parmegiani & A. Cacciapuoti (2002) Synthesis and antifungal activity of the 2, 2, 5-tetrahydrofuran regioisomers of SCH 51048. *Bioorganic and Medicinal Chemistry Letters,* 12**,** 1739-1742.
Mandhapati, A. R., D. Shcherbakov, S. Duscha, A. Vasella, E. C. Böttger & D. Crich (2014) Importance of the 6′-Hydroxy Group and Its Configuration for Apramycin Activity. *ChemMedChem,* 9**,** 2074-2083.
Masukawa, H., N. Tanaka & H. Umezawa (1968) Localization of kanamycin sensitivity in the 23S core of 30S ribosomes of *E. coli*. *Journal of Antibiotics,* 21**,** 517-518.
Matt, T., C. L. Ng, K. Lang, S.-H. Sha, R. Akbergenov, D. Shcherbakov, M. Meyer, S. Duscha, J. Xie, S. R. Dubbaka, D. Perez-Fernandez, A. Vasella, V. Ramakrishnan, J. Schacht & E. C. Böttger (2012) Dissociation of antibacterial activity and aminoglycoside ototoxicity in the 4-monosubstituted 2-deoxystreptamine apramycin. *Proceedings of the National Academy of Sciences of the United States of America,* 109**,** 10984-10989.
McGuirk, P. R., M. R. Jefson, D. D. Mann, N. C. Elliott, P. Chang, E. P. Cisek, C. P. Cornell, T. D. Gootz, S. L. Haskell, L. J. LaFleur, M. J. Rosenfeld, T. R. Shryock, A. M. Silvia & F. H. Weber (1992) Synthesis and structure-activity relationships of 7-diazabicycloalkylquinolones, including danofloxacin, a new quinolone antibacterial agent for veterinary medicine. *Journal of Medicinal Chemistry,* 35**,** 611-620.
Mellado, E., G. Garcia-Effron, M. J. Buitrago, L. Alcazar-Fuoli, M. Cuenca-Estrella & J. L. Rodriguez-Tudela (2005) Targeted gene disruption of the 14-α sterol demethylase (*cyp51A*) in Aspergillus fumigatus and its role in azole drug susceptibility. *Antimicrobial Agents and Chemotherapy,* 49**,** 2536-2538.
Moodahadu, L. S., A. Patnaik, V. V. Arvind, R. M. Bhide, K. Katta, B. Krishnankutty & S. Bellary (2014) Preclinical pharmacological profile of Eberconazole: A review and update. *Muller Journal of Medical Sciences and Research,* 5**,** 159-165.
Moreau, N., C. Jaxel & F. Le Goffic (1984) Comparison of fortimicins with other aminoglycosides and effects on bacterial ribosome and protein synthesis. *Antimicrobial Agents and Chemotherapy,* 26**,** 857-862.
Moreillon, P. & J. M. Entenza (2001) Antibiotic resistance: learning from animal feeds and animal experimentation. *Clinical Microbiology and Infection,* 7**,** 13-18.
Morrow, B. J., W. He, K. M. Amsler, B. D. Foleno, M. J. Macielag, A. S. Lynch & K. Bush (2010) *In vitro* antibacterial activities of JNJ-Q2, a new broad-spectrum fluoroquinolone. *Antimicrobial Agents and Chemotherapy,* 54**,** 1955-1964.
Mugnier, J., J. Gouot, J. Hutt, A. Greiner, M. Chazalet, J. M. Gaulliard & G. Ingram. 1993. RPA 400727, a new seed treatment effective against cereal leaf diseases. In *45th International Symposium on Crop Protection*, 1411-1419. Ghent, Belgium: Mededelingen van de Faculteit Landbouwwetenschappen, Universiteit Gent.
Munayyer, H., K. J. Shaw, R. S. Hare, B. Salisbury, L. Heimark, B. Pramanik & J. R. Greene. 1996. SCH 56592 is a potent inhibitor of sterol C14 demethylation in fungi, abstr. F92. In *Program and Abstracts of the 36th Interscience Conference on Antimicrobial Agents and Chemotherapy. American Society for Microbiology, Washington, DC,* 115. American Society for Microbiology, Washington, D.C.
Muratani, T., M. Inoue & S. Mitsuhashi (1992) *In vitro* activity of T-3761, a new fluoroquinolone. *Antimicrobial Agents and Chemotherapy,* 36**,** 2293-2303.
Niwano, Y., H. Koga, H. Kodama, K. Kanai, T. Miyazaki & H. Yamaguchi (1999) Inhibition of sterol 14 α-demethylation of *Candida albicans* with NND-502, a novel optically active imidazole antimycotic agent. *Medical Mycology,* 37**,** 351-355.
Ohmi, T., S. Sakumoto, Y. Niwano, H. Oka, T. Suzuki, M. Uchida & H. Yamaguchi (1992) Inhibitory Effect of NND-318 on Ergosterol Synthesis in *Trichophyton mentagrophytes* and *Candida albicans*. *Nippon Ishinkin Gakkai Zasshi,* 33**,** 349-354.
Otani, T., M. Tanaka, E. Ito, Y. Kurosaka, Y. Murakami, K. Onodera, T. Akasaka & K. Sato (2003) *In vitro* and *in vivo* antibacterial activities of DK-507k, a novel fluoroquinolone. *Antimicrobial Agents and Chemotherapy,* 47**,** 3750-3759.
Ozaki, M., M. Matsuda, Y. Tomii, K. Kimura, K. Kazuno, M. Kitano, M. Kise, K. Shibata, M. Otsuki & T. Nishino (1991) *In vitro* antibacterial activity of a new quinolone, NM394. *Antimicrobial Agents and Chemotherapy,* 35**,** 2490-2495.
Padeiskaya, E. N. & O. V. Baklanova (1993) Synthetic chemotherapeutic drugs for the treatment of mycoses (review). *Pharmaceutical Chemistry Journal,* 27**,** 227-241.
Park, K.-S., H.-I. Kang, J. W. Lee & Y.-K. Paik (2004) Anti-Candida activity of YH-1715R, a new triazole derivative. *Journal of Microbiology and Biotechnology,* 14**,** 693-697.
Patel, M. V., N. J. de Souza, S. V. Gupte, M. A. Jafri, S. S. Bhagwat, Y. Chugh, H. F. Khorakiwala, M. R. Jacobs & P. C. Appelbaum (2004) Antistaphylococcal activity of WCK 771, a tricyclic fluoroquinolone, in animal infection models. *Antimicrobial Agents and Chemotherapy,* 48**,** 4754-4761.
Pfaller, M. A., J. Riley & T. Koerner (1990) Effects of terconazole and other azole antifungal agents on the sterol and carbohydrate composition of *Candida albicans*. *Diagnostic Microbiology and Infectious Disease,* 13**,** 31-35.
Piddock, L. J. V., M. C. Hall & R. N. Walters (1991) Phenotypic characterization of quinolone-resistant mutants of Enterobacteriaceae selected from wild type, *gyrA* type and multiplyresistant (*marA*) type strains. *Journal of Antimicrobial Chemotherapy,* 28**,** 185-198.
Podlogar, B. L., K. A. Ohemeng & J. F. Barrett (2003) Patents on tetracycline and tetracycline derivatives as antimicrobials: January 1998–October 2002. *Expert Opinion on Therapeutic Patents,* 13**,** 467-478.
Pucci, M. J., M. Ackerman, J. A. Thanassi, C. M. Shoen & M. H. Cynamon (2010) *In vitro* antituberculosis activities of ACH-702, a novel isothiazoloquinolone, against quinolone-susceptible and quinolone-resistant isolates. *Antimicrobial Agents and Chemotherapy,* 54**,** 3478-3480.
Pye, G. W. & M. S. Marriott (1982) Inhibition of sterol C14 demethylation by imidazole-containing antifungals. *Sabouraudia: Journal of Medical and Veterinary Mycology,* 20**,** 325-329.
Quinn, J. A., T. T. Fujimoto, A. R. Egan & S. H. Shaber (1986) The properties of RH 3866, a new triazole fungicide. *Pest Management Science,* 17**,** 357-362.
Rao, S. S. & A. P. Grollman (1967) Cycloheximide resistance in yeast: a property of the 60s ribosomal subunit. *Biochemical and Biophysical Research Communications,* 29**,** 696-704.
Reynolds, K. L., T. B. Brenneman & P. F. Bertrand (1997) Sensitivity of *Cladosporium caryigenum* to propiconazole and fenbuconazole. *Plant Disease,* 81**,** 163-166.
Richardson, K., K. Cooper, M. S. Marriott, M. H. Tarbit, F. Troke & P. J. Whittle (1990) Discovery of fluconazole, a novel antifungal agent. *Reviews of Infectious Diseases,* 12**,** S267-S271.
Richardson, K., K. Cooper, M. S. Marriott, M. H. Tarbit, P. F. Troke & P. J. Whittle (1988) Design and evaluation of a systemically active agent, fluconazole. *Annals of the New York Academy of Sciences,* 544**,** 4-11.
Ries, G. & R. Hess (2000) Safety and efficacy of elubiol in cosmetic use. *Journal of Toxicology: Cutaneous and Ocular Toxicology,* 19**,** 199-206.
Roberts, T. R., D. H. Hutson, P. J. Jewess, P. W. Lee, P. H. Nicholls & J. R. Plimmer. 1999. *Metabolic pathways of agrochemicals: insecticides and fungicides*. Great Britain: Royal Society of Chemistry.

Robinson, M. J., B. A. Martin, T. D. Gootz, P. R. McGuirk, M. Moynihan, J. A. Sutcliffe & N. Osheroff (1991) Effects of quinolone derivatives on eukaryotic topoisomerase II. A novel mechanism for enhancement of enzyme-mediated DNA cleavage. *Journal of Biological Chemistry,* 266**,** 14585-14592.
Robinson, M. J., B. A. Martin, T. D. Gootz, P. R. Mcguirk & N. Osheroff (1992) Effects of novel fluoroquinolones on the catalytic activities of eukaryotic topoisomerase II: Influence of the C-8 fluorine group. *Antimicrobial Agents and Chemotherapy,* 36**,** 751-756.
Rohner, P., M. Peebo, D. P. Lew, R. Auckenthaler & J.-C. Pechère (1992) Comparative *in-vitro* activity of new quinolones against clinical isolates and resistant mutants. *Journal of Antimicrobial Chemotherapy,* 29**,** 41-48.
Rosen, T., D. T. W. Chu, I. M. Lico, P. B. Fernandes, K. Marsh, L. Shen, V. G. Cepa & A. G. Pernet (1988) Design, synthesis, and properties of (4*S*)-7-(4-amino-2-substituted-pyrrolidin-1-yl) quinolone-3-carboxylic acids. *Journal of Medicinal Chemistry,* 31**,** 1598-1611.
Ryder, N. S. (1990) Biochemical model of action and enantiomeric selectivity of SDZ 89–485, a new triazole antimycotic. *Journal of Medical and Veterinary Mycology,* 28**,** 385-394.
Saishoji, T., A. Ito, S. Kumazawa & H. Chuman (1998) Structure-Activity Relationships of Enantiomers of the Azole Fungicide Ipconazole and Its Related Compounds: Fungicidal and Plant Growth Inhibitory Activities. *Journal of Pesticide Science,* 23**,** 129-136.
Salas, J. A. & C. Hardisson (1979) Inhibition of the respiration of dormant and swollen *Streptomyces* spores by chloramphenicol. *Current Microbiology,* 2**,** 273-276.
Sanati, H., P. Belanger, R. Fratti & M. Ghannoum (1997) A new triazole, voriconazole (UK-109,496), blocks sterol biosynthesis in *Candida albicans* and *Candida krusei*. *Antimicrobial Agents and Chemotherapy,* 41**,** 2492-2496.
Sato, T., T. Okubo, M. Usui, H. Higuchi & Y. Tamura (2013) Amino acid substitutions in GyrA and ParC are associated with fluoroquinolone resistance in *Mycoplasma bovis* isolates from Japanese dairy calves. *Journal of Veterinary Medical Science,* 75**,** 1063-1065.
Schneider-Poetsch, T., J. Ju, D. E. Eyler, Y. Dang, S. Bhat, W. C. Merrick, R. Green, B. Shen & J. O. Liu (2010) Inhibition of eukaryotic translation elongation by cycloheximide and lactimidomycin. *Nature Chemical Biology,* 6**,** 209-217.
Schwarz, S., C. Kehrenberg, B. Doublet & A. Cloeckaert (2004) Molecular basis of bacterial resistance to chloramphenicol and florfenicol. *FEMS Microbiology Reviews,* 28**,** 519-542.
Shen, L. L., L. A. Mitscher, P. N. Sharma, T. J. O'donnell, D. W. T. Chu, C. S. Cooper, T. Rosen & A. G. Pernet (1989) Mechanism of inhibition of DNA gyrase by quinolone antibacterials: a cooperative drug-DNA binding model. *Biochemistry,* 28**,** 3886-3894.
Shen, L. L. & A. G. Pernet (1985) Mechanism of inhibition of DNA gyrase by analogues of nalidixic acid: the target of the drugs is DNA. *Proceedings of the National Academy of Sciences of the United States of America,* 82**,** 307-311.
Shirane, N., A. Murabayashi, M. Masuko, A. Uomori, Y. Yoshimura, S. Seo, K. Uchida & K. i. Takeda (1990) Effect on ergosterol biosynthesis of a fungicide, SSF-109, in *Botrytis cinerea*. *Phytochemistry,* 29**,** 2513-2520.
Sisler, H. D. & M. R. Siegel. 1967. Cycloheximide and other glutarimide antibiotics. In *Mechanism of Action,* eds. D. Gottlieb & P. D. Shaw, 283-307. Berlin, Heidelberg Springer.
Sobue, S., K. Tan, L. Shaw, G. Layton & R. Hust (2004) Comparison of the pharmacokinetics of fosfluconazole and fluconazole after single intravenous administration of fosfluconazole in healthy Japanese and Caucasian volunteers. *European Journal of Clinical Pharmacology,* 60**,** 247-253.
Sonenberg, N., M. Wilchek & A. Zamir (1973) Mapping of *Escherichia coli* ribosomal components involved in peptidyl transferase activity. *Proceedings of the National Academy of Sciences of the United States of America,* 70**,** 1423-1426.
Sorbera, L. A., M. del Fresno & X. Rabasseda (2003) CS-758. Antifungal lanosterol 14α-demethylase inhibitor. *Drugs of the Future,* 28**,** 217-223.
Strahilevitz, J., Q. C. Truong-Bolduc & D. C. Hooper (2005) DX-619, a novel des-fluoro (6) quinolone manifesting low frequency of selection of resistant *Staphylococcus aureus* mutants: quinolone resistance beyond modification of type II topoisomerases. *Antimicrobial Agents and Chemotherapy,* 49**,** 5051-5057.
Sugino, A., C. L. Peebles, K. N. Kreuzer & N. R. Cozzarelli (1977) Mechanism of action of nalidixic acid: purification of *Escherichia coli nalA* gene product and its relationship to DNA gyrase and a novel nicking-closing enzyme. *Proceedings of the National Academy of Sciences of the United States of America,* 74**,** 4767-4771.
Sum, P. E. 2004. Tetracyclines. In *Kirk-Othmer Encyclopedia of Chemical Technology,* eds. E. R. Kirk, D. F. Othmer, J. I. Kroschwitz & M. Howe-Grant. New York: John Wiley & Sons, Inc.
Sum, P.-E., A. T. Ross, P. J. Petersen & R. T. Testa (2006) Synthesis and antibacterial activity of 9-substituted minocycline derivatives. *Bioorganic and Medicinal Chemistry Letters,* 16**,** 400-403.
Takahashi, Y., N. Masuda, M. Otsuki, M. Miki & T. Nishino (1997) *In vitro* activity of HSR-903, a new quinolone. *Antimicrobial Agents and Chemotherapy,* 41**,** 1326-1330.
Takenouchi, T., F. Tabata, Y. Iwata, H. Hanzawa, M. Sugawara & S. Ohya (1996) Hydrophilicity of quinolones is not an exclusive factor for decreased activity in efflux-mediated resistant mutants of *Staphylococcus aureus*. *Antimicrobial Agents and Chemotherapy,* 40**,** 1835-1842.
Tanaka, N. 1975. Aminoglycoside antibiotics. In *Mechanism of Action of Antimicrobial and Antitumor Agents,* eds. J. W. Corcoran, F. E. Hahn, J. F. Snell & K. L. Arora, 340-364. Berlin, Heidelberg: Springer.
Tanaka, N. (1983) Mechanism of action of and resistance to aminoglycoside antibiotics. *Archives of Pharmacal Research,* 6**,** 93-102.
Tanaka, N., K. Matsunaga, A. Hirata, Y. Matsuhisa & T. Nishimura (1983) Mechanism of action of habekacin, a novel amino acid-containing aminoglycoside antibiotic. *Antimicrobial Agents and Chemotherapy,* 24**,** 797-802.
Tasaka, A., T. Kitazaki, N. Tsuchimori, Y. Matsushita, R. Hayashi, K. Okonogi & K. Itoh (1997) Optically Active Antifungal Azoles. VII. Synthesis and Antifungal Activity of Stereoisomers of 2-[(1R, 2R)-2-(2, 4-Difluorophenyl)-2-hydroxy-1-methyl-3-(1H-1, 2, 4-triazol-1-yl) propyl]-4-[4-(2, 2, 3, 3-tetrafluoropropoxy) phenyl]-3 (2H, 4H)-1, 2, 4-triazolone (TAK-187). *Chemical and Pharmaceutical Bulletin,* 45**,** 321-326.
Taton, M., P. Ullmann, P. Benveniste & A. Rahier (1988) Interaction of triazole fungicides and plant growth regulators with microsomal cytochrome *P*-450-dependent obtusifoliol 14α-methyl demethylase. *Pesticide Biochemistry and Physiology,* 30**,** 178-189.
Tatsumi, Y., M. Nagashima, T. Shibanushi, A. Iwata, Y. Kangawa, F. Inui, W. J. J. Siu, R. Pillai & Y. Nishiyama (2013) Mechanism of action of efinaconazole, a novel triazole antifungal agent. *Antimicrobial Agents and Chemotherapy,* 57**,** 2405-2409.
Tsuchimori, N., R. Hayashi, N. Kitamoto, K. Asai, T. Kitazaki, Y. Iizawa, K. Itoh & K. Okonogi (2002) *In vitro* and *in vivo* antifungal activities of TAK-456, a novel oral triazole with a broad antifungal spectrum. *Antimicrobial Agents and Chemotherapy,* 46**,** 1388-1393.
Tsuda, M., H. Itoh & S. Kato (2004) Evaluation of the systemic activity of simeconazole in comparison with that of other DMI fungicides. *Pest Management Science,* 60**,** 875-880.
Uchida, K. & H. Wolf (1974) Metabolic products of microorganisms 133. Inhibition of ribosomal peptidyl transferase by hikizimycin, a nucleoside antibiotic. *Journal of Antibiotics,* 27**,** 783-787.
Udumula, V., Y. W. Ham, M. Y. Fosso, K. Y. Chan, R. Rai, J. Zhang, J. Li & C.-W. T. Chang (2013) Investigation of antibacterial mode of action for traditional and amphiphilic aminoglycosides. *Bioorganic and Medicinal Chemistry Letters,* 23**,** 1671-1675.
Vanden Bossche, H., J. Ausma, H. Bohets, K. Vermuyten, G. Willemsens, P. Marichal, L. Meerpoel, F. Odds & M. Borgers (2004) The novel azole R126638 is a selective inhibitor of ergosterol synthesis in *Candida albicans, Trichophyton* spp., and *Microsporum canis*. *Antimicrobial Agents and Chemotherapy,* 48**,** 3272-3278.
Vanden Bossche, H., P. Marichal, J. Gorrens, D. Bellens, H. Verhoeven, M. C. Coene, W. Lauwers & P. A. J. Janssen (1987) Interaction of azole derivatives with cytochrome P-450 isozymes in yeast, fungi, plants and mammalian cells. *Pest Management Science,* 21**,** 289-306.
Vanden Bossche, H., P. Marichal, G. Willemsens, D. Bellens, J. Gorrens, I. Roels, M.-C. Coene, L. Le Jeune & P. A. J. Janssen (1990) Saperconazole: A selective inhibitor of the cytochrome P-450-dependent ergosterol synthesis in *Candida albicans*, *Aspergillus fumigatus* and *Trichophyton mentagrophytes*. *Mycoses,* 33**,** 335-352.
Vazquez, D. (1964) The binding of chloramphenicol by ribosomes from *Bacillus megaterium*. *Biochemical and Biophysical Research Communications,* 15**,** 464-468.
Vazquez, D., M. Barbacid & L. Carrasco (1974) Inhibitors of mammalian protein synthesis. *Hamatol Bluttransfus,* 14**,** 327-340.
Venkateswarlu, K. & S. L. Kelly (1997) Stereoselective interaction of SCH 39304, a triazole, with sterol 14alpha-demethylase of *Aspergillus fumigatus*. *Journal of Antimicrobial Chemotherapy,* 39**,** 597-601.

Veraldi, S. (2013) Isoconazole nitrate: a unique broad-spectrum antimicrobial azole effective in the treatment of dermatomycoses, both as monotherapy and in combination with corticosteroids. *Mycoses,* 56**,** 3-15.

von Ahsen, U., J. Davies & R. Schroeder (1991) Antibiotic inhibition of group I ribozyme function. *Nature,* 353**,** 368-370.

von Ahsen, U., J. Davies & R. Schroeder (1992) Non-competitive inhibition of group I intron RNA self-splicing by aminoglycoside antibiotics. *Journal of Molecular Biology,* 226**,** 935-941.

Wada, T., S. Kuzuma, M. Takenaka & Y. Hirota (1991) Fungitoxic properties and mechanism of action of pefurazoate. *Japanese Journal of Phytopathology,* 57**,** 153-159.

Wang, Q., E. Lucien, A. Hashimoto, G. C. G. Pais, D. M. Nelson, Y. Song, J. A. Thanassi, C. W. Marlor, C. L. Thoma, J. Cheng, S. D. Podos, Y. Ou, M. Deshpande, M. J. Pucci, D. D. Buechter, B. J. Bradbury & J. A. Wiles (2007) Isothiazoloquinolones with enhanced antistaphylococcal activities against multidrug-resistant strains: effects of structural modifications at the 6-, 7-, and 8-positions. *Journal of Medicinal Chemistry,* 50**,** 199-210.

Ward, K. W., J.-F. Lepage & J.-Y. Driot (2007) Nonclinical pharmacodynamics, pharmacokinetics, and safety of BOL-303224-A, a novel fluoroquinolone antimicrobial agent for topical ophthalmic use. *Journal of Ocular Pharmacology and Therapeutics,* 23**,** 243-256.

Warrilow, A. G. S., C. M. Martel, J. E. Parker, N. Melo, D. C. Lamb, W. D. Nes, D. E. Kelly & S. L. Kelly (2010) Azole binding properties of *Candida albicans* sterol 14-α demethylase (CaCYP51). *Antimicrobial Agents and Chemotherapy,* 54**,** 4235-4245.

Warrilow, A. G. S., C. L. Price, J. E. Parker, N. J. Rolley, C. J. Smyrniotis, D. D. Hughes, V. Thoss, W. D. Nes, D. E. Kelly, T. R. Holman & S. L. Kelly (2016) Azole antifungal sensitivity of sterol 14α-demethylase (CYP51) and CYP5218 from *Malassezia globosa*. *Scientific reports,* 6**,** 1-10.

Wiles, J. A., Q. Wang, E. Lucien, A. Hashimoto, Y. Song, J. Cheng, C. W. Marlor, Y. Ou, S. D. Podos, J. A. Thanassi, C. L. Thoma, M. Deshpande, M. J. Pucci & B. J. Bradbury (2006) Isothiazoloquinolones containing functionalized aromatic hydrocarbons at the 7-position: synthesis and *in vitro* activity of a series of potent antibacterial agents with diminished cytotoxicity in human cells. *Bioorganic and Medicinal Chemistry Letters,* 16**,** 1272-1276.

Wolfson, J. S., D. C. Hooper, E. Y. Ng, K. S. Souza, G. L. McHugh & M. N. Swartz (1987) Antagonism of wild-type and resistant *Escherichia coli* and its DNA gyrase by the tricyclic 4-quinolone analogs ofloxacin and S-25930 stereoisomers. *Antimicrobial Agents and Chemotherapy,* 31**,** 1861-1863.

Xin, L., D. Pu, H. Deliang, Y. Huijun, L. Weiming, Y. Fei, Z. Xin, K. Dongyang, C. Juyang, Y. Weiyan, H. Dongyi, J. Zhengce & G. Minxin (2006) Mitochondrial DNA A1555G mutation screening using a testing kit method and its significance in preventing aminoglycoside-related hearing loss. *Journal of Otology,* 1**,** 61-64.

Yamakawa, T., J. Mitsuyama & K. Hayashi (2002) *In vitro* and *in vivo* antibacterial activity of T-3912, a novel non-fluorinated topical quinolone. *Journal of Antimicrobial Chemotherapy,* 49**,** 455-465.

Yokoyama, K., Y. Doi, K. Yamane, H. Kurokawa, N. Shibata, K. Shibayama, T. Yagi, H. Kato & Y. Arakawa (2003) Acquisition of 16S rRNA methylase gene in *Pseudomonas aeruginosa*. *The Lancet,* 362**,** 1888-1893.

Yoshida, T. & S. Mitsuhashi (1993) Antibacterial activity of NM394, the active form of prodrug NM441, a new quinolone. *Antimicrobial Agents and Chemotherapy,* 37**,** 793-800.

Yu, X., G. Wang, S. Chen, G. Wei, Y. Shang, L. Dong, T. Schön, D. Moradigaravand, J. Parkhill, S. J. Peacock, C. U. Köser & H. Huang (2016) Mycobacterium tuberculosis wild-type and non-wild-type MIC distributions for the novel fluoroquinolone antofloxacin compared with ofloxacin, levofloxacin, and moxifloxacin. *Antimicrobial Agents and Chemotherapy***,** AAC. 00393-16.

Yuri, A., I. Koichi, H. Kimihiko, S. Akira, K. Michinari & Y. Yuzo (1992) Inhibition by a novel azole antifungal agent with a geranyl group on lanosterol 14α-demethylase of yeast. *Biochemical Pharmacology,* 44**,** 1701-1705.

Zampieri, D., M. G. Mamolo, E. Laurini, G. Scialino, E. Banfi & L. Vio (2009) 2-Aryl-3-(1*za*-Azol-1-yl)-1*H*-Indole derivatives: a new class of antimycobacterial compounds–conventional heating in comparison with MW-assisted synthesis. *Archiv der Pharmazie,* 342**,** 716-722.

Zhao, X., J.-Y. Wang, C. Xu, Y. Dong, J. Zhou, J. Domagala & K. Drlica (1998) Killing of *Staphylococcus aureus* by C-8-methoxy fluoroquinolones. *Antimicrobial Agents and Chemotherapy,* 42**,** 956-958.

Ziogas, B. N. & A. A. Malandrakis. 2015. Sterol biosynthesis inhibitors: C14 demethylation (DMIs). In *Fungicide Resistance in Plant Pathogens,* eds. H. Ishii & D. Hollomon, 199-216. Tokyo: Springer.

Zweerink, M. M. & A. Edison (1986) Inhibition of *Micrococcus luteus* DNA gyrase by norfloxacin and 10 other quinolone carboxylic acids. *Antimicrobial Agents and Chemotherapy,* 29**,** 598-601.

Table S3. Primary targets annotated by PubChem, ChEMBL, KEGG and DrugBank databases for bioactive molecules of the present study

Bioactive molecule	PubChem[3]	ChEMBL[1]	KEGG[2]	DrugBank[4]
A 57132	Unknown	Unknown	Unknown	Unknown
A 57241	Unknown	Unknown	Unknown	Unknown
A 57274 (A 62917)	Unknown	Unknown	Unknown	Unknown
A 60919 (PD 118106)	Unknown	Unknown	Unknown	Unknown
A 61867 (BRN 4276829)	Unknown	Unknown	Unknown	Unknown
A 62251 (A 57531; PD 137954)	Unknown	Unknown	Unknown	Unknown
A 62255	Unknown	Unknown	Unknown	Unknown
A 62824	Unknown	Unknown	Unknown	Unknown
A 65326	Unknown	Unknown	Unknown	Unknown
ACH 702	Unknown	Unknown	Unknown	Unknown
Acorafloxacin (avarofloxacin)	Unknown	Unknown	DNA gyrase and topoisomerase IV inhibitor	Unknown
ADDNC (A 65485)	Unknown	Unknown	Unknown	Unknown
Alalevonadifloxacin	Unknown	Unknown	Unknown	Unknown
Alatrofloxacin	Topoisomerase II (eukaryotic & prokaryotic forms) inhibitor	Bacterial DNA gyrase and topoisomerase IV inhibitor	DNA gyrase and topoisomerase IV inhibitor	Unknown
Amifloxacin	Topoisomerase II (eukaryotic & prokaryotic forms) inhibitor	Unknown	Unknown	Unknown
Antofloxacin	Topoisomerase II (eukaryotic & prokaryotic forms) inhibitor	Unknown	Unknown	Unknown
AT 4929	Unknown	Unknown	Unknown	Unknown
Balofloxacin	Topoisomerase II (eukaryotic & prokaryotic forms) inhibitor	Unknown	DNA gyrase and topoisomerase IV inhibitor	Unknown
BAY Y-3118 free base	Unknown	Unknown	Unknown	Unknown
Besifloxacin	Topoisomerase II (eukaryotic & prokaryotic forms) inhibitor	Bacterial DNA gyrase and topoisomerase IV inhibitor	DNA gyrase and topoisomerase IV inhibitor	Bacterial DNA gyrase and topoisomerase IV inhibitor
Binfloxacin	Unknown	Unknown	Unknown	Unknown
BMY 40062	Unknown	Unknown	Unknown	Unknown
BMY 40397	Unknown	Unknown	Unknown	Unknown
BMY 42230	Unknown	Unknown	Unknown	Unknown
BMY 43261	Unknown	Unknown	Unknown	Unknown
BMY 43748	Unknown	Unknown	Unknown	Unknown
BMY 45243	Unknown	Unknown	Unknown	Unknown
BMY 45706	Unknown	Unknown	Unknown	Unknown
BRN 4913428 (PD 131199)	Unknown	Unknown	Unknown	Unknown
Cadrofloxacin	Unknown	Unknown	Unknown	Unknown
Cetefloxacin	Unknown	Unknown	Unknown	Unknown
Chinfloxacin	Unknown	Unknown	Unknown	Unknown
CI 990 (PD 131112)	Unknown	Unknown	Unknown	Unknown
Ciprofloxacin	Topoisomerase II (eukaryotic & prokaryotic forms) inhibitor	Bacterial DNA gyrase inhibitor	DNA gyrase inhibitor	Bacterial DNA gyrase and topoisomerase IV inhibitor
Clinafloxacin	Topoisomerase II (eukaryotic & prokaryotic forms) inhibitor	Unknown	DNA gyrase and topoisomerase IV inhibitor	Unknown
CP 100964	Unknown	Unknown	Unknown	Unknown
CP 104830	Unknown	Unknown	Unknown	Unknown
CP 105532 (PD 125275)	Unknown	Unknown	Unknown	Unknown
CP 115953	Unknown	Unknown	Unknown	Unknown
CP 115955	Unknown	Unknown	Unknown	Unknown
CP 135803	Unknown	Unknown	Unknown	Unknown
CP 67015	Unknown	Unknown	Unknown	Unknown
CP 67804	Unknown	Unknown	Unknown	Unknown
CP 74667	Unknown	Unknown	Unknown	Unknown
CP 92121	Unknown	Unknown	Unknown	Unknown
CP 99433	Unknown	Unknown	Unknown	Unknown
Danofloxacin	Unknown	Unknown	DNA gyrase inhibitor	Unknown
DC 159a free base	Unknown	Unknown	Unknown	Unknown

Bioactive molecule	PubChem[3]	ChEMBL[1]	KEGG[2]	DrugBank[4]
Delafloxacin	Bacterial DNA gyrase and topoisomerase IV inhibitor	Unknown	DNA gyrase and topoisomerase IV inhibitor	Bacterial DNA gyrase and topoisomerase IV inhibitor
Desfluorociprofloxacin (SQ 4004)	Unknown	Unknown	Unknown	Unknown
Difloxacin	Unknown	Unknown	DNA gyrase inhibitor	Unknown
DJ 6783	Unknown	Unknown	Unknown	Unknown
DK 507k	Unknown	Unknown	Unknown	Unknown
DN 9494	Unknown	Unknown	Unknown	Unknown
Droxacin	Unknown	Unknown	Unknown	Unknown
DS 8587 free base	Unknown	Unknown	Unknown	Unknown
DU 6611	Unknown	Unknown	Unknown	Unknown
DU 6668	Unknown	Unknown	Unknown	Unknown
DV 7751a (DV 7751)	Unknown	Unknown	Unknown	Unknown
DW 8186	Unknown	Unknown	Unknown	Unknown
DX 619	Unknown	Unknown	Unknown	Unknown
E 3604	Unknown	Unknown	Unknown	Unknown
E 3846	Unknown	Unknown	Unknown	Unknown
E 4441	Unknown	Unknown	Unknown	Unknown
E 4474	Unknown	Unknown	Unknown	Unknown
E 4480	Unknown	Unknown	Unknown	Unknown
E 4497	Unknown	Unknown	Unknown	Unknown
E 4501	Unknown	Unknown	Unknown	Unknown
E 4502	Unknown	Unknown	Unknown	Unknown
E 4527	Unknown	Unknown	Unknown	Unknown
E 4528	Unknown	Unknown	Unknown	Unknown
E 4534	Unknown	Unknown	Unknown	Unknown
E 4535	Unknown	Unknown	Unknown	Unknown
E 4695	Unknown	Unknown	Unknown	Unknown
Ecenofloxacin	Unknown	Unknown	Unknown	Unknown
EN 272	Unknown	Unknown	Unknown	Unknown
Enoxacin	Topoisomerase II (eukaryotic & prokaryotic forms) inhibitor	DNA gyrase and topoisomerase IV inhibitor	DNA gyrase inhibitor	Bacterial DNA gyrase inhibitor
Enrofloxacin	Bacterial DNA gyrase inhibitor	Unknown	DNA gyrase and topoisomerase IV inhibitor	Unknown
Esafloxacin	Unknown	Unknown	Unknown	Unknown
FA 103	Unknown	Unknown	Unknown	Unknown
Fandofloxacin	Unknown	Unknown	Unknown	Unknown
Finafloxacin	Bacterial DNA gyrase and topoisomerase IV inhibitor	DNA gyrase and topoisomerase IV inhibitor	DNA gyrase and topoisomerase IV inhibitor	Bacterial DNA gyrase and topoisomerase IV inhibitor
Fleroxacin	DNA gyrase inhibitor	Unknown	DNA gyrase and topoisomerase IV inhibitor	DNA gyrase inhibitor
Flumequine	Topoisomerase II (eukaryotic & prokaryotic forms) inhibitor	Unknown	Unknown	Unknown
Garenoxacin	Topoisomerase II (eukaryotic & prokaryotic forms) inhibitor	Unknown	DNA gyrase and topoisomerase IV inhibitor	Unknown
Gatifloxacin	Bacterial DNA gyrase and topoisomerase IV inhibitor	DNA gyrase and topoisomerase IV inhibitor	DNA gyrase and topoisomerase IV inhibitor	Bacterial DNA gyrase and topoisomerase IV inhibitor
Gemifloxacin	Bacterial DNA gyrase and topoisomerase IV inhibitor	DNA gyrase and topoisomerase IV inhibitor	DNA gyrase and topoisomerase IV inhibitor	Bacterial DNA gyrase and topoisomerase IV inhibitor
Grepafloxacin	Bacterial DNA gyrase and topoisomerase IV inhibitor	DNA gyrase and topoisomerase IV inhibitor	Unknown	Bacterial DNA gyrase and topoisomerase IV inhibitor
Ibafloxacin	Unknown	Unknown	Unknown	Unknown
Irloxacin	Unknown	Unknown	Unknown	Unknown
K 12	Unknown	Unknown	Unknown	Unknown
KB 5246	Unknown	Unknown	Unknown	Unknown
KPI 10 free base (WQ 3810)	Unknown	Unknown	Unknown	Unknown
Lascufloxacin	Unknown	Unknown	Unknown	Unknown
Levofloxacin	Bacterial DNA gyrase and topoisomerase IV inhibitor	DNA gyrase and topoisomerase IV inhibitor	DNA gyrase and topoisomerase IV inhibitor	Bacterial DNA gyrase and topoisomerase IV inhibitor
Levonadifloxacin	Unknown	Unknown	Unknown	Unknown
Levonadifloxacin arginine (WCK 771)	Unknown	Unknown	Unknown	Unknown
Lomefloxacin	Bacterial DNA gyrase and topoisomerase IV inhibitor	Bacterial DNA gyrase inhibitor	DNA gyrase inhibitor	Bacterial DNA gyrase and topoisomerase IV inhibitor

Bioactive molecule	PubChem[3]	ChEMBL[1]	KEGG[2]	DrugBank[4]
Marbofloxacin	Unknown	Unknown	Unknown	Unknown
Merafloxacin	Unknown	Unknown	Unknown	Unknown
Metioxate	Unknown	Unknown	Unknown	Unknown
MF 5101	Unknown	Unknown	Unknown	Unknown
MF 5103	Unknown	Unknown	Unknown	Unknown
MF 5112 free base	Unknown	Unknown	Unknown	Unknown
MF 5126	Unknown	Unknown	Unknown	Unknown
MF 5137	Unknown	Unknown	Unknown	Unknown
MF 5143	Unknown	Unknown	Unknown	Unknown
MF 5168	Unknown	Unknown	Unknown	Unknown
Miloxacin	Unknown	Unknown	Unknown	Unknown
Moxifloxacin	Bacterial DNA gyrase and topoisomerase IV inhibitor	Bacterial DNA gyrase and topoisomerase IV inhibitor	DNA gyrase and topoisomerase IV inhibitor	Bacterial DNA gyrase and topoisomerase IV inhibitor
Nadifloxacin	Topoisomerase II (eukaryotic & prokaryotic forms) inhibitor	Unknown	DNA gyrase inhibitor	Unknown
Nalidixic acid	Topoisomerase II (eukaryotic & prokaryotic forms) inhibitor	Bacterial DNA gyrase inhibitor	DNA gyrase inhibitor	Bacterial DNA gyrase inhibitor
Nemonoxacin	Unknown	Unknown	Unknown	Unknown
Norfloxacin	Bacterial DNA gyrase and topoisomerase IV inhibitor	Bacterial DNA gyrase and topoisomerase IV inhibitor	DNA gyrase inhibitor	Bacterial DNA gyrase and topoisomerase IV inhibitor
NSFQ 104	Unknown	Unknown	Unknown	Unknown
NSFQ 105	Unknown	Unknown	Unknown	Unknown
Ofloxacin	DNA gyrase and topoisomerase IV inhibitor	Bacterial DNA gyrase inhibitor	DNA gyrase and topoisomerase IV inhibitor	DNA gyrase and topoisomerase IV inhibitor
Olamufloxacin	Unknown	Unknown	Unknown	Unknown
Orbifloxacin	Unknown	Unknown	DNA gyrase inhibitor	Unknown
Oxolinic acid	Topoisomerase II (eukaryotic & prokaryotic forms) inhibitor	DNA topoisomerase type II (gyrase)	DNA gyrase and topoisomerase IV inhibitor	Unknown
Ozenoxacin	Bacterial DNA gyrase A and topoisomerase IV inhibitor	Unknown	DNA gyrase and topoisomerase IV inhibitor	Bacterial DNA gyrase A and topoisomerase IV inhibitor
Pazufloxacin	Topoisomerase II (eukaryotic & prokaryotic forms) inhibitor	Unknown	DNA gyrase and topoisomerase IV inhibitor	Unknown
PD 111834	Unknown	Unknown	Unknown	Unknown
PD 112388	Unknown	Unknown	Unknown	Unknown
PD 114111	Unknown	Unknown	Unknown	Unknown
PD 115311	Unknown	Unknown	Unknown	Unknown
PD 116507	Unknown	Unknown	Unknown	Unknown
PD 117596	Unknown	Unknown	Unknown	Unknown
PD 118362	Unknown	Unknown	Unknown	Unknown
PD 119344	Unknown	Unknown	Unknown	Unknown
PD 129626	Unknown	Unknown	Unknown	Unknown
PD 131628	Unknown	Unknown	Unknown	Unknown
PD 135042 (AM 1147)	Topoisomerase II (eukaryotic & prokaryotic forms) inhibitor	Unknown	Unknown	Unknown
PD 135144 (BMY 33315)	Unknown	Unknown	Unknown	Unknown
PD 137156	Unknown	Unknown	Unknown	Unknown
PD 138312	Topoisomerase II (eukaryotic & prokaryotic forms) inhibitor	Unknown	Unknown	Unknown
PD 140248	Unknown	Unknown	Unknown	Unknown
PD 163449	Unknown	Unknown	Unknown	Unknown
PD 164488	Unknown	Unknown	Unknown	Unknown
Pefloxacin	Bacterial DNA gyrase A and topoisomerase IV inhibitor	Unknown	DNA gyrase and topoisomerase IV inhibitor	Bacterial DNA gyrase A and topoisomerase IV inhibitor
Pipemidic acid	Unknown	Unknown	DNA gyrase inhibitor	Unknown
Piromidic acid	Unknown	Unknown	DNA gyrase inhibitor	Unknown
Piroxacin	Unknown	Unknown	Unknown	Unknown
Pradofloxacin	Unknown	Unknown	Unknown	Unknown
Premafloxacin	Unknown	Unknown	DNA gyrase and topoisomerase IV inhibitor	Unknown
Prulifloxacin	Topoisomerase II (eukaryotic & prokaryotic forms) inhibitor	Unknown	DNA gyrase inhibitor	Unknown
PubChem CID-11531032	Unknown	Unknown	Unknown	Unknown

Bioactive molecule	PubChem[3]	ChEMBL[1]	KEGG[2]	DrugBank[4]
PubChem CID-11566845	Unknown	Unknown	Unknown	Unknown
PubChem CID-11610627	Unknown	Unknown	Unknown	Unknown
PubChem CID-11696318	Unknown	Unknown	Unknown	Unknown
PubChem CID-11844920	Unknown	Unknown	Unknown	Unknown
PubChem CID-11996799	Unknown	Unknown	Unknown	Unknown
PubChem CID-11996800	Unknown	Unknown	Unknown	Unknown
PubChem CID-11997263	Unknown	Unknown	Unknown	Unknown
PubChem CID-25022869	Unknown	Unknown	Unknown	Unknown
PubChem CID-44408626	Unknown	Unknown	Unknown	Unknown
PubChem CID-44408894	Unknown	Unknown	Unknown	Unknown
PubChem CID-44408896	Unknown	Unknown	Unknown	Unknown
PubChem CID-44408994	Unknown	Unknown	Unknown	Unknown
PubChem CID-44409001	Unknown	Unknown	Unknown	Unknown
PubChem CID-44409010	Unknown	Unknown	Unknown	Unknown
PubChem CID-53236573	Unknown	Unknown	Unknown	Unknown
PubChem CID-53236796	Unknown	Unknown	Unknown	Unknown
PubChem CID-122195336	Unknown	Unknown	Unknown	Unknown
PubChem CID-122195337	Unknown	Unknown	Unknown	Unknown
QA 241 free base	Unknown	Unknown	Unknown	Unknown
RO 13-5478	Unknown	Unknown	Unknown	Unknown
RO 14-9578	Unknown	Unknown	Unknown	Unknown
Rosoxacin	Bacterial DNA gyrase and topoisomerase IV inhibitor	Unknown	DNA gyrase inhibitor	Bacterial DNA gyrase and topoisomerase IV inhibitor
Rufloxacin	Topoisomerase II (eukaryotic & prokaryotic forms) inhibitor	Unknown	DNA gyrase and topoisomerase IV inhibitor	Unknown
S 25932	Unknown	Unknown	Unknown	Unknown
S 31076	Unknown	Unknown	Unknown	Unknown
Sarafloxacin	Unknown	Unknown	DNA gyrase and topoisomerase IV inhibitor	Unknown
Sitafloxacin	Topoisomerase II (eukaryotic & prokaryotic forms) inhibitor	Unknown	DNA gyrase and topoisomerase IV inhibitor	Unknown
Sparfloxacin	Bacterial DNA gyrase and topoisomerase IV inhibitor	Bacterial DNA gyrase and topoisomerase IV inhibitor	DNA gyrase and topoisomerase IV inhibitor	Bacterial DNA gyrase and topoisomerase IV inhibitor
T 14097	Unknown	Unknown	Unknown	Unknown
Temafloxacin	Bacterial DNA gyrase and topoisomerase IV inhibitor	Unknown	DNA gyrase and topoisomerase IV inhibitor	Bacterial DNA gyrase and topoisomerase IV inhibitor
Tioxacin	Unknown	Unknown	Unknown	Unknown
Tosufloxacin	Unknown	Unknown	DNA gyrase and topoisomerase IV inhibitor	Unknown
Trovafloxacin	Bacterial DNA gyrase and topoisomerase IV inhibitor	Bacterial DNA gyrase and topoisomerase IV inhibitor	DNA gyrase and topoisomerase IV inhibitor	Bacterial DNA gyrase and topoisomerase IV inhibitor
Ulifloxacin	Topoisomerase II (eukaryotic & prokaryotic forms) inhibitor	Unknown	Unknown	Unknown
Vebufloxacin (benofloxacin)	Unknown	Unknown	Unknown	Unknown
VG 6/1	Unknown	Unknown	Unknown	Unknown
WCK 1152 free base	Unknown	Unknown	Unknown	Unknown
WIN 57273	Unknown	Unknown	Unknown	Unknown
WIN 57294	Topoisomerase II (eukaryotic & prokaryotic forms) inhibitor	Unknown	Unknown	Unknown
WIN 58161	Unknown	Unknown	Unknown	Unknown
WQ 2743	Unknown	Unknown	Unknown	Unknown
WQ 2756	Unknown	Unknown	Unknown	Unknown
WQ 2908	Unknown	Unknown	Unknown	Unknown
WQ 2942	Unknown	Unknown	Unknown	Unknown
WQ 3330	Unknown	Unknown	Unknown	Unknown
Y 688	Unknown	Unknown	Unknown	Unknown
Zabofloxacin	Unknown	Unknown	Unknown	Unknown
Azidamfenicol	Unknown	Unknown	50S ribosomal subunit inhibitor	Unknown
Bromamphenicol (bromoamphenicol)	Unknown	Unknown	Unknown	Unknown

Bioactive molecule	PubChem[3]	ChEMBL[1]	KEGG[2]	DrugBank[4]
Cetofenicol (cetophenicol)	Unknown	Unknown	50S ribosomal subunit inhibitor	Unknown
Chloramphenicol	50S ribosomal protein L16	Unknown	50S ribosomal subunit inhibitor	50S ribosomal protein L16
Florfenicol	Unknown	Unknown	50S ribosomal subunit inhibitor	Unknown
Monoiodoamphenicol	Unknown	Unknown	Unknown	Unknown
Racefenicol (racephenicol)	Unknown	Unknown	50S ribosomal subunit inhibitor	Unknown
Tevenel	Unknown	Unknown	Unknown	Unknown
Thiamphenicol	Unknown	Unknown	50S ribosomal subunit inhibitor	Unknown
WIN 5094-2	Unknown	Unknown	Unknown	Unknown
9-Methylstreptimidone (S 632A$_2$)	Unknown	Unknown	Unknown	Unknown
Acetoxycycloheximide (streptovitacin E-73)	Unknown	Unknown	Unknown	Unknown
Actiketal	Unknown	Unknown	Unknown	Unknown
Actiphenol (actinophenol)	Unknown	Unknown	Unknown	Unknown
Cycloheximide	Unknown	Unknown	Unknown	Unknown
Epiderstatin	Unknown	Unknown	Unknown	Unknown
Inactone	Unknown	Unknown	Unknown	Unknown
Isocycloheximide	Unknown	Unknown	Unknown	Unknown
Isomigrastatin	Unknown	Unknown	Unknown	Unknown
Lactimidomycin	Unknown	Unknown	Unknown	Unknown
Naramycin B	Unknown	Unknown	Unknown	Unknown
Neoisocycloheximide	Unknown	Unknown	Unknown	Unknown
S 632A$_3$	Unknown	Unknown	Unknown	Unknown
Streptimidone (S 632A$_1$)	Unknown	Unknown	Unknown	Unknown
Streptovitacin A	Unknown	Unknown	Unknown	Unknown
Streptovitacin B	Unknown	Unknown	Unknown	Unknown
Streptovitacin C$_2$	Unknown	Unknown	Unknown	Unknown
Amicetin (allomycin)	Unknown	Unknown	Unknown	Unknown
Antelmycin (anthelmycin)	Unknown	Unknown	Unknown	Unknown
Arginomycin	Unknown	Unknown	Unknown	Unknown
Bagougeramine A	Unknown	Unknown	Unknown	Unknown
Bagougeramine B	Unknown	Unknown	Unknown	Unknown
Bamicetin	Unknown	Unknown	Unknown	Unknown
Blasticidin H	Unknown	Unknown	Unknown	Unknown
Blasticidin S	Inhibition of peptide bound formation in the ribosomal machinery	Unknown	Unknown	Unknown
Cytimidine	Unknown	Unknown	Unknown	Unknown
Cytomycin (saitomycin)	Unknown	Unknown	Unknown	Unknown
Cytosamine	Unknown	Unknown	Unknown	Unknown
Cytosaminomycin A	Unknown	Unknown	Unknown	Unknown
Cytosaminomycin B	Unknown	Unknown	Unknown	Unknown
Cytosaminomycin C	Unknown	Unknown	Unknown	Unknown
Cytosaminomycin D	Unknown	Unknown	Unknown	Unknown
Gougerotin	Unknown	Unknown	Unknown	Unknown
Mildiomycin	Unknown	Unknown	Unknown	Unknown
Mildiomycin B	Unknown	Unknown	Unknown	Unknown
Mildiomycin C	Unknown	Unknown	Unknown	Unknown
Mildiomycin D	Unknown	Unknown	Unknown	Unknown
Mildiomycin M	Unknown	Unknown	Unknown	Unknown
Norplicacetin	Unknown	Unknown	Unknown	Unknown
Oxamicetin	Unknown	Unknown	Unknown	Unknown
Oxyplicacetin (cytosaminomycin E)	Unknown	Unknown	Unknown	Unknown
Plicacetin (amicetin B)	Unknown	Unknown	Unknown	Unknown
Rodaplutin	Unknown	Unknown	Unknown	Unknown
SCH 36605	Unknown	Unknown	Unknown	Unknown
SF 2457	Unknown	Unknown	Unknown	Unknown
1-Epidactimicin	Unknown	Unknown	Unknown	Unknown
A 396I (SS 56D)	Unknown	Unknown	Unknown	Unknown

Bioactive molecule	PubChem[3]	ChEMBL[1]	KEGG[2]	DrugBank[4]
Ambistrin (streptoduocin)	Unknown	Unknown	Unknown	Unknown
Amikacin	30S ribosomal protein S12 and 16S rRNA	Unknown	30S ribosomal subunit	30S ribosomal protein S12 and 16S rRNA
Apramycin (nebramycin II)	Unknown	Unknown	30S ribosomal subunit	Unknown
Aprosamine	Unknown	Unknown	Unknown	Unknown
Arbekacin	30S ribosomal protein S12 and 16S rRNA	Unknown	30S ribosomal subunit	30S ribosomal protein S12 and 16S rRNA
Astromicin (fortimicin A)	Unknown	Unknown	30S ribosomal subunit	Unknown
Astromicin B (fortimicin B)	Unknown	Unknown	Unknown	Unknown
Bekanamycin (kanamycin B; nebramycin V)	Bacterial 30S ribosomal subunit	Unknown	30S ribosomal subunit	Unknown
Betamicin (gentamicin B)	Unknown	Unknown	Unknown	Unknown
Butikacin	Unknown	Unknown	Unknown	Unknown
Butirosin A	Unknown	Unknown	Unknown	Unknown
Butirosin B	Unknown	Unknown	Unknown	Unknown
Dactimicin	Unknown	Unknown	Unknown	Unknown
Destomycin A	Unknown	Unknown	Unknown	Unknown
Destomycin B	Unknown	Unknown	Unknown	Unknown
Dibekacin	Unknown	Unknown	30S ribosomal subunit	Unknown
Dihydrostreptomycin	Bacterial 30S ribosomal subunit	Unknown	30S ribosomal subunit	Unknown
Etimicin	Unknown	Unknown	Unknown	Unknown
Fortimicin AE	Unknown	Unknown	Unknown	Unknown
Fortimicin AH	Unknown	Unknown	Unknown	Unknown
Fortimicin AI	Unknown	Unknown	Unknown	Unknown
Fortimicin AK	Unknown	Unknown	Unknown	Unknown
Fortimicin AM	Unknown	Unknown	Unknown	Unknown
Fortimicin AN	Unknown	Unknown	Unknown	Unknown
Fortimicin AO	Unknown	Unknown	Unknown	Unknown
Fortimicin AP	Unknown	Unknown	Unknown	Unknown
Fortimicin AQ	Unknown	Unknown	Unknown	Unknown
Fortimicin AS	Unknown	Unknown	Unknown	Unknown
Fortimicin C	Unknown	Unknown	Unknown	Unknown
Fortimicin D	Unknown	Unknown	Unknown	Unknown
Fortimicin E (fortimicin KH)	Unknown	Unknown	Unknown	Unknown
Fortimicin KE	Unknown	Unknown	Unknown	Unknown
Fortimicin KF	Unknown	Unknown	Unknown	Unknown
Fortimicin KG	Unknown	Unknown	Unknown	Unknown
Fortimicin KL_1	Unknown	Unknown	Unknown	Unknown
Fortimicin KR	Unknown	Unknown	Unknown	Unknown
Fortimicin AL	Unknown	Unknown	Unknown	Unknown
Framycetin (neomycin B)	30S ribosomal protein S12 and 16S rRNA	Unknown	30S ribosomal subunit	30S ribosomal protein S12 and 16S rRNA
Geneticin (gentamicin G-418)	Unknown	Unknown	Unknown	Unknown
Gentamicin A	Unknown	Unknown	Unknown	Unknown
Gentamicin A_1	Unknown	Unknown	Unknown	Unknown
Gentamicin A_2	Unknown	Unknown	Unknown	Unknown
Gentamicin A_3	Unknown	Unknown	Unknown	Unknown
Gentamicin A_4	Unknown	Unknown	Unknown	Unknown
Gentamicin B_1	Unknown	Unknown	Unknown	Unknown
Gentamicin C_1	30S ribosomal protein S12 and 16S rRNA	Unknown	30S ribosomal subunit	30S ribosomal protein S12 and 16S rRNA
Gentamicin C_{1a}	30S ribosomal protein S12 and 16S rRNA	Unknown	30S ribosomal subunit	30S ribosomal protein S12 and 16S rRNA
Gentamicin C_2	30S ribosomal protein S12 and 16S rRNA	Unknown	30S ribosomal subunit	30S ribosomal protein S12 and 16S rRNA
Gentamicin C_{2a}	30S ribosomal protein S12 and 16S rRNA	Unknown	30S ribosomal subunit	30S ribosomal protein S12 and 16S rRNA
Gentamicin X_2	Unknown	Unknown	Unknown	Unknown
Hybrimycin A_1	Unknown	Unknown	Unknown	Unknown
Hybrimycin A_2	Unknown	Unknown	Unknown	Unknown
Hybrimycin B_1	Unknown	Unknown	Unknown	Unknown
Hybrimycin B_2	Unknown	Unknown	Unknown	Unknown
Hybrimycin C_1	Unknown	Unknown	Unknown	Unknown
Hybrimycin C_2	Unknown	Unknown	Unknown	Unknown

Bioactive molecule	PubChem[3]	ChEMBL[1]	KEGG[2]	DrugBank[4]
Hybrimycin D	Unknown	Unknown	Unknown	Unknown
Hygromycin B (A 396II)	Unknown	Unknown	Unknown	Unknown
Isepamicin	Unknown	Unknown	30S ribosomal subunit	Unknown
Istamycin A (sannamycin A)	Unknown	Unknown	Unknown	Unknown
Istamycin A_0 (sannamycin B)	Unknown	Unknown	Unknown	Unknown
Istamycin A_1	Unknown	Unknown	Unknown	Unknown
Istamycin A_2	Unknown	Unknown	Unknown	Unknown
Istamycin A_3	Unknown	Unknown	Unknown	Unknown
Istamycin AO	Unknown	Unknown	Unknown	Unknown
Istamycin AP (sannamycin E)	Unknown	Unknown	Unknown	Unknown
Istamycin B	Unknown	Unknown	Unknown	Unknown
Istamycin B_0	Unknown	Unknown	Unknown	Unknown
Istamycin B_1	Unknown	Unknown	Unknown	Unknown
Istamycin B_3	Unknown	Unknown	Unknown	Unknown
Istamycin C	Unknown	Unknown	Unknown	Unknown
Istamycin C_0	Unknown	Unknown	Unknown	Unknown
Istamycin C_1	Unknown	Unknown	Unknown	Unknown
Istamycin KL_1	Unknown	Unknown	Unknown	Unknown
Istamycin X_0 (sannamycin G)	Unknown	Unknown	Unknown	Unknown
Istamycin Y_0 (sannamycin H)	Unknown	Unknown	Unknown	Unknown
Kanamycin (kanamycin A)	30S ribosomal protein S12 and 16S rRNA	Unknown	30S ribosomal subunit	30S ribosomal protein S12 and 16S rRNA
Kanamycin C	Unknown	Unknown	Unknown	Unknown
Kanamycin D	Unknown	Unknown	Unknown	Unknown
Kanamycin X	Unknown	Unknown	Unknown	Unknown
Lividamine (nebramycin IX)	Unknown	Unknown	Unknown	Unknown
Lividomycin	Unknown	Unknown	Unknown	Unknown
Lividomycin B (3'-deoxyparomomycin I)	Unknown	Unknown	Unknown	Unknown
Mannosylparomomycin	Unknown	Unknown	Unknown	Unknown
Micronomicin (gentamicin C_{2b})	Unknown	Unknown	30S ribosomal subunit	Unknown
Neamine (neomycin A; nebramycin X)	Unknown	Unknown	30S ribosomal subunit	Unknown
Nebramine (nebramycin VIII)	Unknown	Unknown	Unknown	Unknown
Nebramycin III	Unknown	Unknown	Unknown	Unknown
Nebramycin IV	Unknown	Unknown	Unknown	Unknown
Nebramycin V'	Unknown	Unknown	Unknown	Unknown
Nebramycin XI	Unknown	Unknown	Unknown	Unknown
Nebramycin XII	Unknown	Unknown	Unknown	Unknown
Nebramycin XIII	Unknown	Unknown	Unknown	Unknown
Neomycin C	Unknown	Unknown	Unknown	Unknown
Neomycin F (paromomycin II)	Unknown	Unknown	Unknown	Unknown
Netilmicin	30S ribosomal protein S12 and 16S rRNA	Unknown	30S ribosomal subunit	30S ribosomal protein S12 and 16S rRNA
NK 1001	Unknown	Unknown	Unknown	Unknown
Oxyapramycin (nebramycin VII)	Unknown	Unknown	Unknown	Unknown
Paromamine (neomycin D)	Unknown	Unknown	Unknown	Unknown
Paromomycin (paromomycin I; neomycin E)	16S ribosomal RNA	Unknown	30S ribosomal subunit	16S ribosomal RNA
Pentisomicin	Unknown	Unknown	Unknown	Unknown
Plazomicin	Unknown	Unknown	30S ribosomal subunit	Unknown
Propikacin	Unknown	Unknown	30S ribosomal subunit	Unknown
Pyrankacin	Unknown	Unknown	Unknown	Unknown
Ribostamycin	30S ribosomal subunit	Unknown	30S ribosomal subunit	30S ribosomal subunit
Saccharocin (KA 5685)	Unknown	Unknown	Unknown	Unknown
Sannamycin C	Unknown	Unknown	Unknown	Unknown
Sannamycin F	Unknown	Unknown	Unknown	Unknown
Sannamycin J	Unknown	Unknown	Unknown	Unknown

Bioactive molecule	PubChem[3]	ChEMBL[1]	KEGG[2]	DrugBank[4]
Sannamycin K	Unknown	Unknown	Unknown	Unknown
Sannamycin KR	Unknown	Unknown	Unknown	Unknown
Sannamycin L	Unknown	Unknown	Unknown	Unknown
Seldomycin	Unknown	Unknown	Unknown	Unknown
Seldomycin 1 (seldomycin factor 1)	Unknown	Unknown	Unknown	Unknown
Seldomycin 2 (seldomycin factor 2)	Unknown	Unknown	Unknown	Unknown
Seldomycin 3 (seldomycin factor 3)	Unknown	Unknown	Unknown	Unknown
Seldomycin 5 (seldomycin factor 5)	Unknown	Unknown	Unknown	Unknown
Sisomicin	Unknown	Unknown	30S ribosomal subunit	Unknown
Sisomicin B	Unknown	Unknown	Unknown	Unknown
Sisomicin D	Unknown	Unknown	Unknown	Unknown
Spectinomycin	30S ribosomal subunit	Unknown	30S ribosomal subunit	30S ribosomal subunit
Sporaricin A	Unknown	Unknown	Unknown	Unknown
Sporaricin B	Unknown	Unknown	Unknown	Unknown
Sporaricin C	Unknown	Unknown	Unknown	Unknown
Sporaricin D	Unknown	Unknown	Unknown	Unknown
Sporaricin E	Unknown	Unknown	Unknown	Unknown
SS 56A	Unknown	Unknown	Unknown	Unknown
SS 56B	Unknown	Unknown	Unknown	Unknown
SS 56C	Unknown	Unknown	Unknown	Unknown
Streptomycin	30S ribosomal protein S12 and 16S rRNA	Unknown	30S ribosomal subunit	30S ribosomal protein S12 and 16S rRNA
Streptoniazid (streptonicozid)	Unknown	Unknown	Unknown	Unknown
Tobramycin (nebramycin VI)	30S ribosomal subunit and 16S rRNA	Unknown	Unknown	30S ribosomal subunit and 16S rRNA
Trospectomycin	Unknown	Unknown	30S ribosomal subunit	Unknown
Verdamicin	Unknown	Unknown	Unknown	Unknown
Verdamicin C_2	Unknown	Unknown	Unknown	Unknown
Vertilmicin	Unknown	Unknown	Unknown	Unknown
7-Iodosancycline	Unknown	Unknown	Unknown	Unknown
Amicycline	Unknown	Unknown	Unknown	Unknown
Apicycline	Unknown	Unknown	Unknown	Unknown
Bromotetracycline (bromtetracycline)	Unknown	Unknown	Unknown	Unknown
Chlortetracycline (chlorotetracycline)	Unknown	Unknown	30S ribosomal subunit	Unknown
Clomocycline	30S ribosomal subunit	Unknown	30S ribosomal subunit	30S ribosomal subunit
Demeclocycline	30S ribosomal subunit	Unknown	30S ribosomal subunit	Unknown
Demecycline	Unknown	Unknown	Unknown	Unknown
DMG-DMDOT (DMG-DM DOT)	Unknown	Unknown	Unknown	Unknown
DMG-MINO	Unknown	Unknown	Unknown	Unknown
Doxycycline	Unknown	Unknown	30S ribosomal subunit	Unknown
Eravacycline	Unknown	Unknown	30S ribosomal subunit	Unknown
Etamocycline	Unknown	Unknown	Unknown	Unknown
Glycocycline	Unknown	Unknown	Unknown	Unknown
Guamecycline	Unknown	Unknown	Unknown	Unknown
Lymecycline	30S ribosomal subunit	Unknown	30S ribosomal subunit	30S ribosomal subunit
Meclocycline	30S ribosomal protein S7	Unknown	30S ribosomal subunit	30S ribosomal protein S7
Meglucycline	Unknown	Unknown	Unknown	Unknown
Metacycline (methacycline)	30S ribosomal subunit and 16S rRNA	Unknown	30S ribosomal subunit	30S ribosomal subunit and 16S rRNA
Minocycline	30S ribosomal subunit	Unknown	30S ribosomal subunit	30S ribosomal subunit
Morphocycline	Unknown	Unknown	Unknown	Unknown
Nitrocycline	Unknown	Unknown	Unknown	Unknown
Omadacycline	Unknown	Unknown	30S ribosomal subunit	Unknown
Oxytetracycline	30S ribosomal subunit	Unknown	30S ribosomal subunit	30S ribosomal subunit
Pecocycline	Unknown	Unknown	Unknown	Unknown
Penimepicycline	Unknown	Unknown	Unknown	Unknown
Penimocycline	Unknown	Unknown	Unknown	Unknown
Pipacycline	Unknown	Unknown	Unknown	Unknown

Bioactive molecule	PubChem[3]	ChEMBL[1]	KEGG[2]	DrugBank[4]
Rolitetracycline	30S ribosomal subunit	Unknown	30S ribosomal subunit	30S ribosomal subunit
Sancycline	Unknown	Unknown	Unknown	Unknown
Sarecycline	Unknown	Unknown	30S ribosomal subunit	Unknown
Tetracycline	30S ribosomal subunit	Unknown	30S ribosomal subunit	30S ribosomal subunit
Tigecycline	30S ribosomal subunit	Unknown	30S ribosomal subunit	30S ribosomal subunit
TP 271	Unknown	Unknown	Unknown	Unknown
Albaconazole	Unknown	Unknown	Sterol 14alpha-demethylase	Unknown
Alteconazole	Unknown	Unknown	Sterol 14alpha-demethylase	Unknown
Azaconazole	Unknown	C14-demethylase in sterol biosynthesis	Sterol 14alpha-demethylase	Unknown
BAS 110	Unknown	Unknown	Unknown	Unknown
BAS 111	Unknown	Unknown	Unknown	Unknown
BAS 45406F	Unknown	Unknown	Unknown	Unknown
Bitertanol	Unknown	C14-demethylase in sterol biosynthesis	Unknown	Unknown
Bromuconazole	Unknown	C14-demethylase in sterol biosynthesis	Unknown	Unknown
Cyproconazole	Unknown	C14-demethylase in sterol biosynthesis	Unknown	Unknown
D 0870	Unknown	Unknown	Unknown	Unknown
Diclobutrazol	Unknown	Unknown	Unknown	Unknown
Difenoconazole	14alpha-demethylation of sterols	C14-demethylase in sterol biosynthesis	Unknown	Unknown
Diniconazole	Unknown	C14-demethylase in sterol biosynthesis	Unknown	Unknown
Efinaconazole	Lanosterol 14α-demethylase	Lanosterol 14-alpha demethylase inhibitor	Sterol 14alpha-demethylase	Lanosterol 14α-demethylase
Embeconazole	Unknown	Unknown	Unknown	Unknown
Epoxiconazole	Unknown	C14-demethylase in sterol biosynthesis	Unknown	Unknown
Etaconazole	Unknown	Unknown	Unknown	Unknown
Fenbuconazole	Unknown	C14-demethylase in sterol biosynthesis	Unknown	Unknown
Fluconazole	Lanosterol 14α-demethylase	Cytochrome P450 51 inhibitor	Sterol 14alpha-demethylase	Lanosterol 14α-demethylase
Fluotrimazole	Unknown	Unknown	Unknown	Unknown
Fluquinconazole	Unknown	C14-demethylase in sterol biosynthesis	Unknown	Unknown
Flusilazole	Unknown	C14-demethylase in sterol biosynthesis	Unknown	Unknown
Flutriafol	Unknown	C14-demethylase in sterol biosynthesis	Unknown	Unknown
Fosfluconazole	Sterol 14-demethylase	Unknown	Sterol 14alpha-demethylase	Unknown
Furconazole	Unknown	Unknown	Unknown	Unknown
Genaconazole (SCH 39304)	Unknown	Unknown	Unknown	Unknown
Hexaconazole	Unknown	C14-demethylase in sterol biosynthesis	Unknown	Unknown
ICI 153066	Unknown	Unknown	Unknown	Unknown
ICI 195739	Unknown	Unknown	Unknown	Unknown
Imibenconazole	Unknown	Unknown	Unknown	Unknown
Ipconazole	Unknown	Unknown	Unknown	Unknown
Ipfentrifluconazole	Unknown	Unknown	Unknown	Unknown
Isavuconazole	Lanosterol 14α-demethylase	Unknown	Sterol 14alpha-demethylase	Lanosterol 14α-demethylase
Itraconazole	Lanosterol 14α-demethylase	Cytochrome P450 51 inhibitor	Sterol 14alpha-demethylase	Lanosterol 14α-demethylase
LAB 158241F	Unknown	Unknown	Unknown	Unknown
LAB 170250F	Unknown	Unknown	Unknown	Unknown
Mefentrifluconazole	Unknown	Unknown	Unknown	Unknown
Metconazole	Unknown	C14-demethylase in sterol biosynthesis	Unknown	Unknown
Myclobutanil	Unknown	C14-demethylase in sterol biosynthesis	Unknown	Unknown
Penconazole	Unknown	C14-demethylase in sterol biosynthesis	Unknown	Unknown
Posaconazole	Sterol 14α-demethylase	Cytochrome P450 51 inhibitor	Sterol 14alpha-demethylase	Sterol 14α-demethylase
PP 969	Unknown	Unknown	Unknown	Unknown
Pramiconazole	Unknown	Unknown	Sterol 14alpha-demethylase	Unknown

Bioactive molecule	PubChem[3]	ChEMBL[1]	KEGG[2]	DrugBank[4]
Propiconazole	Unknown	C14-demethylase in sterol biosynthesis	Unknown	Unknown
Quinconazole	Unknown	Unknown	Unknown	Unknown
Ravuconazole	14α-demethylase in sterol synthesis	Unknown	Unknown	Unknown
Saperconazole	Cytochrome P450 sterol 14 alpha-demethylase (CYP51)	Unknown	Sterol 14alpha-demethylase	Unknown
SCH 42427	Unknown	Unknown	Unknown	Unknown
SCH 51048	Unknown	Unknown	Unknown	Unknown
SDZ 89-485	Unknown	Unknown	Unknown	Unknown
Simeconazole	Unknown	Unknown	Unknown	Unknown
SSF 109 (huanjunzuo)	Unknown	Unknown	Unknown	Unknown
SSY 726	Unknown	Unknown	Unknown	Unknown
SYN 2506	Unknown	Unknown	Unknown	Unknown
SYN 2836	Unknown	Unknown	Unknown	Unknown
SYN 2869	Unknown	Unknown	Unknown	Unknown
SYN 2903	Unknown	Unknown	Unknown	Unknown
SYN 2921	Unknown	Unknown	Unknown	Unknown
T 8581	Unknown	Unknown	Unknown	Unknown
TAK 187	Unknown	Unknown	Unknown	Unknown
TAK 456	Unknown	Unknown	Unknown	Unknown
Tebuconazole	Unknown	C14-demethylase in sterol biosynthesis	Unknown	Unknown
Terconazole (triaconazole)	Lanosterol 14α-demethylase	Cytochrome P450 51 inhibitor	Sterol 14alpha-demethylase	Lanosterol 14α-demethylase
Tetraconazole	Unknown	C14-demethylase in sterol biosynthesis	Unknown	Unknown
Triadimefon	Unknown	C14-demethylase in sterol biosynthesis	Unknown	Unknown
Triadimenol	Unknown	C14-demethylase in sterol biosynthesis	Unknown	Unknown
Triticonazole	Unknown	Unknown	Unknown	Unknown
UK 47265	Unknown	Unknown	Unknown	Unknown
UK 51486	Unknown	Unknown	Unknown	Unknown
Uniconazole	Unknown	Unknown	Unknown	Unknown
UR 9746	Unknown	Unknown	Unknown	Unknown
UR 9751	Unknown	Unknown	Unknown	Unknown
Vibunazole (BAY N-7133)	Unknown	Unknown	Sterol 14alpha-demethylase	Unknown
Voriconazole	14-alpha sterol demethylase	Cytochrome P450 51 inhibitor	Sterol 14alpha-demethylase	14-alpha sterol demethylase
YH 1715R	Unknown	Unknown	Unknown	Unknown
1-Dodecylimidazole (N-dodecylimidazole)	Unknown	Unknown	Unknown	Unknown
AFK 108	Unknown	Unknown	Unknown	Unknown
Aliconazole	Unknown	Unknown	Sterol 14alpha-demethylase	Unknown
Arasertaconazole	Unknown	Unknown	Unknown	Unknown
Azalanstat	Lanosterol 14α-demethylase	Unknown	CYP51A1	Unknown
BAY C-9263	Unknown	Unknown	Unknown	Unknown
BAY D-9603	Unknown	Unknown	Unknown	Unknown
Becliconazole	Unknown	Unknown	Unknown	Unknown
Bifonazole	Lanosterol 14α-demethylase	Unknown	Sterol 14alpha-demethylase	Lanosterol 14α-demethylase
Brolaconazole	Unknown	Unknown	Unknown	Unknown
Butoconazole	Cytochrome P450 14α-demethylase	Cytochrome P450 51 inhibitor	Sterol 14alpha-demethylase	Cytochrome P450 14α-demethylase
Cisconazole	Unknown	Unknown	Sterol 14alpha-demethylase	Unknown
Climbazole	Unknown	Unknown	Unknown	Unknown
Clotrimazole	Lanosterol 14α-demethylase	Cytochrome P450 51 inhibitor	Sterol 14alpha-demethylase	Lanosterol 14α-demethylase
Croconazole	Unknown	Unknown	Sterol 14alpha-demethylase	Unknown
Democonazole	Unknown	Unknown	Unknown	Unknown
Dichlorophenyl imidazoldioxolan (elubiol)	Unknown	Unknown	Unknown	Unknown
Doconazole	Unknown	Unknown	Sterol 14alpha-demethylase	Unknown
Eberconazole	Unknown	Unknown	Sterol 14alpha-demethylase	Unknown
Econazole	Lanosterol 14α-demethylase	Cytochrome P450 51 inhibitor	Sterol 14alpha-demethylase	Lanosterol 14α-demethylase

Bioactive molecule	PubChem[3]	ChEMBL[1]	KEGG[2]	DrugBank[4]
Fenapanil	Unknown	Unknown	Unknown	Unknown
Fenticonazole	Unknown	Unknown	Sterol 14alpha-demethylase	Unknown
Flutrimazole	Unknown	Unknown	Sterol 14alpha-demethylase	Unknown
Imazalil (enilconazole)	Unknown	C14-demethylase in sterol biosynthesis	Sterol 14alpha-demethylase	Unknown
Isoconazole	Unknown	Unknown	Sterol 14alpha-demethylase	Unknown
Ketaminazole	Unknown	Unknown	Unknown	Unknown
Ketoconazole	Lanosterol 14α-demethylase	Cytochrome P450 51 inhibitor	Sterol 14alpha-demethylase	Lanosterol 14α-demethylase
Lanoconazole	Unknown	Unknown	Sterol 14alpha-demethylase	Unknown
Lombazole	Unknown	Unknown	Unknown	Unknown
Luliconazole	Lanosterol 14α-demethylase	Cytochrome P450 51 inhibitor	Sterol 14alpha-demethylase	Lanosterol 14α-demethylase
MH 0685	Unknown	Unknown	Unknown	Unknown
Miconazole	Lanosterol 14α-demethylase	Cytochrome P450 51 inhibitor	Sterol 14alpha-demethylase	Lanosterol 14α-demethylase
Neticonazole	Unknown	Unknown	Sterol 14alpha-demethylase	Unknown
OK 8705	Unknown	Unknown	Unknown	Unknown
OK 8801	Unknown	Unknown	Unknown	Unknown
Omoconazole	Unknown	Unknown	Sterol 14alpha-demethylase	Unknown
Orconazole	Unknown	Unknown	Sterol 14alpha-demethylase	Unknown
Oxiconazole	Lanosterol 14α-demethylase	Cytochrome P450 51 inhibitor	Sterol 14alpha-demethylase	Lanosterol 14α-demethylase
Oxpoconazole	Unknown	Unknown	Unknown	Unknown
Parconazole	Lanosterol 14α-demethylase	Unknown	Sterol 14alpha-demethylase	Unknown
Pefurazoate	Unknown	C14-demethylase in sterol biosynthesis	Unknown	Unknown
PR 967-234	Unknown	Unknown	Unknown	Unknown
Prochloraz	Unknown	C14-demethylase in sterol biosynthesis	Unknown	Unknown
R 31000	Unknown	Unknown	Unknown	Unknown
Sertaconazole	Lanosterol 14α-demethylase	Cytochrome P450 51 inhibitor	Sterol 14alpha-demethylase	Lanosterol 14α-demethylase
SM 4470	Unknown	Unknown	Unknown	Unknown
SSF 105	Unknown	Unknown	Unknown	Unknown
Sulconazole	Unknown	Cytochrome P450 51 inhibitor	Sterol 14alpha-demethylase	Unknown
Tioconazole	Lanosterol 14α-demethylase	Cytochrome P450 51 inhibitor	Sterol 14alpha-demethylase	Lanosterol 14α-demethylase
Triflumizole	Unknown	C14-demethylase in sterol biosynthesis	Unknown	Unknown
UK 38667	Unknown	Unknown	Unknown	Unknown
Valconazole	Unknown	Unknown	Unknown	Unknown
Zinoconazole	Unknown	Unknown	Sterol 14alpha-demethylase	Unknown
Zoficonazole	Unknown	Unknown	Unknown	Unknown

References

1. Gaulton, A. *et al.* The ChEMBL database in 2017. *Nucleic Acids Res.* **45**, D945–D954 (2017).
2. Kanehisa, M., Furumichi, M., Tanabe, M., Sato, Y. & Morishima, K. KEGG: new perspectives on genomes, pathways, diseases and drugs. *Nucleic Acids Res.* **45**, D353–D361 (2017).
3. Kim, S. *et al*. PubChem 2019 update: improved access to chemical data. *Nucleic Acids Res.* **47**, D1102–D1109 (2019).
4. Wishart, D. S. *et al.* DrugBank 5.0: a major update to the DrugBank database for 2018. *Nucleic Acids Res.* **46**, D1074–D1082 (2018).

Table S4. Activity values of the molecules in ChEMBL database with a structural similarity of 85% and more to molecules in new predictions found with predicted targets of the proposed method

Bioactive molecule	Similar molecule	Similar molecule target	Activity value of similar molecule (µM)	Reference
A 57132	CHEMBL121176	DNA gyrase	IC_{50} = 4.62	J. Med. Chem., (2003) 46:17:3655
	CHEMBL142729		IC_{50} = 8.66	J. Med. Chem., (1991) 34:2:656
	CHEMBL160468		IC_{50} = 18.3	
	CHEMBL2110225		IC_{50} = 1.16	J. Med. Chem., (1993) 36:7:871
	CHEMBL273348		IC_{50} = 7.42	J. Med. Chem., (1993) 36:14:1964
	CHEMBL288421		IC_{50} = 17.93	J. Med. Chem., (1988) 31:8:1598
	CHEMBL294975		IC_{50} = 1.43	
	CHEMBL345900		IC_{50} = 17.58	J. Med. Chem., (1991) 34:2:656
	CHEMBL411723		IC_{50} = 12.46	J. Med. Chem., (1993) 36:14:1964
	CHEMBL420884		IC_{50} = 68.37	J. Med. Chem., (2003) 46:17:3655
	CHEMBL42646		IC_{50} = 1.72	J. Med. Chem., (1988) 31:8:1598
	CHEMBL428	Topoisomerase IV subunit A	IC_{50} = 10	Nucleic Acids Res., (2017) 45:D1:D945
	CHEMBL541279	DNA gyrase	IC_{50} = 0.4	J. Med. Chem., (1988) 31:8:1586
	CHEMBL544247		IC_{50} = 2.97	
	CHEMBL61949		IC_{50} = 150.45	J. Med. Chem., (1991) 34:2:656
			IC_{50} > 75.22	J. Med. Chem., (1993) 36:14:1964
	CHEMBL826		IC_{50} = 87.41	J. Med. Chem., (1986) 29:3:394
		DNA gyrase subunit A/subunit B	IC_{50} = 14	Antimicrob. Agents Chemother., (2008) 52:8:2909
		Topoisomerase IV subunit A	IC_{50} = 10	Nucleic Acids Res., (2017) 45:D1:D945
AT 4929	CHEMBL1201760	Topoisomerase IV subunit A	IC_{50} = 10	Nucleic Acids Res., (2017) 45:D1:D945
	CHEMBL1202	DNA gyrase	IC_{50} = 0.6	J. Med. Chem., (1992) 35:1:94
			IC_{50} = 0.72	J. Med. Chem., (2011) 54:6:1539
	CHEMBL120948		IC_{50} = 4.1	J. Med. Chem., (2003) 46:17:3655
	CHEMBL1210954	Topoisomerase IV subunit A	IC_{50} = 10	Nucleic Acids Res., (2017) 45:D1:D945
	CHEMBL1213456			
	CHEMBL122691	DNA gyrase	IC_{50} = 8.84	J. Med. Chem., (2003) 46:17:3655
	CHEMBL122698		IC_{50} = 0.51	
	CHEMBL123246		IC_{50} = 4.26	
	CHEMBL1276635		IC_{50} = 1.1	Antimicrob. Agents Chemother., (2010) 54:7:3011
	CHEMBL136779		IC_{50} = 6.97	J. Med. Chem., (1996) 39:25:4952
	CHEMBL141308		IC_{50} = 7.64	
	CHEMBL149425		IC_{50} = 36.47	J. Med. Chem., (1988) 31:3:503
	CHEMBL15087		IC_{50} = 0.75	J. Med. Chem., (1993) 36:7:871
			IC_{50} = 7.85	J. Med. Chem., (1993) 36:14:1964
	CHEMBL15186		IC_{50} = 7.51	J. Med. Chem., (1988) 31:3:503
	CHEMBL15247		IC_{50} = 6.64	
	CHEMBL15391		IC_{50} = 7.66	
	CHEMBL15511	Topoisomerase IV subunit A	IC_{50} = 10	Nucleic Acids Res., (2017) 45:D1:D945
	CHEMBL15549	DNA gyrase	IC_{50} = 8.02	J. Med. Chem., (1988) 31:3:503
	CHEMBL15613		IC_{50} = 5.6	Antimicrob. Agents Chemother., (2007) 51:1:119

Bioactive molecule	Similar molecule	Similar molecule target	Activity value of similar molecule (μM)	Reference
		Topoisomerase IV	IC_{50} = 4.2	
	CHEMBL157548	Topoisomerase IV subunit A	IC_{50} = 10	Nucleic Acids Res., (2017) 45:D1:D945
	CHEMBL159024	DNA gyrase	IC_{50} = 16.78	J. Med. Chem., (1991) 34:2:656
	CHEMBL159030		IC_{50} = 4.85	
	CHEMBL159188		IC_{50} = 36.67	
	CHEMBL160192		IC_{50} = 14.09	
	CHEMBL160302		IC_{50} = 2.23	
	CHEMBL160661		IC_{50} = 7.75	
	CHEMBL160662		IC_{50} = 8.15	
	CHEMBL160838		IC_{50} = 4.2	
	CHEMBL184167	Topoisomerase IV subunit A	IC_{50} = 0.73	Bioorg. Med. Chem. Lett., (2004) 14:20:5193
	CHEMBL186870		IC_{50} = 0.41	
	CHEMBL187796		IC_{50} = 0.72	
	CHEMBL2110220	DNA gyrase	IC_{50} = 1.39	J. Med. Chem., (1993) 36:7:871
			IC_{50} = 20.87	
	CHEMBL2110319			
	CHEMBL274469		IC_{50} = 7.68	J. Med. Chem., (1988) 31:3:503
				J. Med. Chem., (1991) 34:2:656
	CHEMBL275174		IC_{50} = 7.65	J. Med. Chem., (1988) 31:3:503
	CHEMBL277100	Topoisomerase IV subunit A	IC_{50} = 10	Nucleic Acids Res., (2017) 45:D1:D945
	CHEMBL278255	DNA gyrase	IC_{50} = 0.55	J. Med. Chem., (2003) 46:17:3655
			IC_{50} = 3.14	J. Med. Chem., (2008) 51:11:3238
		Topoisomerase IV	IC_{50} = 5.17	
		Topoisomerase IV subunit A	IC_{50} = 10	Nucleic Acids Res., (2017) 45:D1:D945
	CHEMBL295433			
	CHEMBL31	DNA gyrase	IC_{50} = 0.5	Bioorg. Med. Chem. Lett., (2006) 16:5:1272
				J. Med. Chem., (2006) 49:22:6435
			IC_{50} = 8.58	Bioorg. Med. Chem., (2009) 17:19:6879
		DNA gyrase subunit A	IC_{50} = 9.4	Antimicrob. Agents Chemother., (2010) 54:8:3478
		Topoisomerase IV subunit A	IC_{50} = 1.2	Bioorg. Med. Chem. Lett., (2006) 16:5:1272
			IC_{50} = 10	Nucleic Acids Res., (2017) 45:D1:D945
	CHEMBL32	DNA gyrase	IC_{50} = 0.3	Bioorg. Med. Chem. Lett., (2006) 16:5:1272
			IC_{50} = 10.91	Bioorg. Med. Chem., (2009) 17:19:6879
			IC_{50} = 18	J. Med. Chem., (2011) 54:9:3418
			IC_{50} = 27.5	Antimicrob. Agents Chemothe., (2007) 51:7:2445
			IC_{50} = 28	J. Med. Chem., (2007) 50:2:199
			IC_{50} = 7.7	J. Med. Chem., (2011) 54:9:3268
		DNA gyrase subunit A	IC_{50} = 9.2	Antimicrob. Agents Chemother., (2010) 54:8:3478
		DNA gyrase subunit A/subunit B	IC_{50} = 2.5	Antimicrob. Agents Chemothe., (2008) 52:8:2909
		DNA gyrase subunit B	IC_{50} > 50	Bioorg. Med. Chem., (2014) 22:17:4924
				Bioorg. Med. Chem., (2015) 23:9:2062

Bioactive molecule	Similar molecule	Similar molecule target	Activity value of similar molecule (μM)	Reference
				Eur. J. Med. Chem., (2015) 103:null:1
		Topoisomerase IV subunit A	IC_{50} = 0.8	Bioorg. Med. Chem. Lett., (2006) 16:5:1272
			IC_{50} = 10	Nucleic Acids Res., (2017) 45:D1:D945
	CHEMBL330779	DNA gyrase	IC_{50} < 1.38	J. Med. Chem., (1991) 34:2:656
	CHEMBL331076		IC_{50} = 8.81	J. Med. Chem., (2003) 46:17:3655
	CHEMBL331987		IC_{50} = 7.93	
	CHEMBL3350260		IC_{50} = 2.09	J. Med. Chem., (1993) 36:7:871
	CHEMBL342273		IC_{50} = 83.48	J. Med. Chem., (1996) 39:25:4952
	CHEMBL343603		IC_{50} = 16.42	
	CHEMBL344447		IC_{50} = 9.37	
	CHEMBL348879		IC_{50} = 7.45	J. Med. Chem., (1991) 34:2:656
	CHEMBL352000		IC_{50} = 16.18	
	CHEMBL357882		IC_{50} = 97.17	J. Med. Chem., (1996) 39:25:4952
	CHEMBL359193		IC_{50} = 32.82	J. Med. Chem., (1988) 31:3:503
	CHEMBL416273		IC_{50} = 10.66	J. Med. Chem., (1996) 39:25:4952
	CHEMBL420897		IC_{50} = 17.91	J. Med. Chem., (2003) 46:17:3655
	CHEMBL421101		IC_{50} = 8.3	
	CHEMBL421277		IC_{50} = 2.32	J. Med. Chem., (1991) 34:2:656
	CHEMBL422131		IC_{50} > 246.07	J. Med. Chem., (1988) 31:3:503
	CHEMBL42299		IC_{50} > 492.09	
	CHEMBL429961		IC_{50} = 4.34	J. Med. Chem., (1996) 39:25:4952
	CHEMBL47028		IC_{50} = 7.4	J. Med. Chem., (1988) 31:3:503
	CHEMBL540589		IC_{50} = 5.53	J. Med. Chem., (1991) 34:2:656
	CHEMBL544407		IC_{50} = 7.99	
	CHEMBL561	Topoisomerase IV subunit A	IC_{50} = 10	Nucleic Acids Res., (2017) 45:D1:D945
	CHEMBL583			
	CHEMBL6210	DNA gyrase	IC_{50} = 3.58	J. Med. Chem., (1988) 31:3:503
	CHEMBL8		IC_{50} = 0.2	J. Med. Chem., (2006) 49:22:6435
			IC_{50} = 0.3	Bioorg. Med. Chem. Lett., (2006) 16:5:1272
			IC_{50} = 0.45	Antimicrob. Agents Chemother., (2010) 54:7:3011
			IC_{50} = 0.97	Bioorg. Med. Chem. Lett., (2012) 22:7:2428
			IC_{50} = 61.7	Antimicrob. Agents Chemother., (2007) 51:7:2445
			IC_{50} = 62	J. Med. Chem., (2007) 50:2:199
		Topoisomerase IV	IC_{50} = 8	J. Med. Chem., (2013) 56:18:7396
		Topoisomerase IV subunit A	IC_{50} = 10	Nucleic Acids Res., (2017) 45:D1:D945
	CHEMBL850	DNA gyrase	IC_{50} = 0.64	J. Med. Chem., (2004) 47:14:3693
		Topoisomerase IV subunit A	IC_{50} = 10	Nucleic Acids Res., (2017) 45:D1:D945
BMY 43748	CHEMBL102572	DNA gyrase	IC_{50} = 16.38	J. Med. Chem., (1986) 29:3:394
	CHEMBL121176		IC_{50} = 4.62	J. Med. Chem., (2003) 46:17:3655
	CHEMBL15314		IC_{50} = 2.17	J. Med. Chem., (1993) 36:7:871
	CHEMBL2110221		IC_{50} = 2.08	

Bioactive molecule	Similar molecule	Similar molecule target	Activity value of similar molecule (μM)	Reference
	CHEMBL2110225		IC_{50} = 1.16	
	CHEMBL2110321		IC_{50} = 2.08	
			IC_{50} = 6.94	
	CHEMBL273348		IC_{50} = 7.42	J. Med. Chem., (1993) 36:14:1964
	CHEMBL276583		IC_{50} = 2.26	J. Med. Chem., (1993) 36:7:871
			IC_{50} = 9.03	J. Med. Chem., (1993) 36:14:1964
	CHEMBL3350261		IC_{50} = 6.94	J. Med. Chem., (1993) 36:7:871
	CHEMBL420884		IC_{50} = 68.37	J. Med. Chem., (2003) 46:17:3655
	CHEMBL428	Topoisomerase IV subunit A	IC_{50} = 10	Nucleic Acids Res., (2017) 45:D1:D945
	CHEMBL541279	DNA gyrase	IC_{50} = 0.4	J. Med. Chem., (1988) 31:8:1586
	CHEMBL542832		IC_{50} = 0.99	
	CHEMBL544247		IC_{50} = 2.97	
	CHEMBL826		IC_{50} = 87.41	J. Med. Chem., (1986) 29:3:394
		DNA gyrase subunit A/subunit B	IC_{50} = 14	Antimicrob. Agents Chemother., (2008) 52:8:2909
		Topoisomerase IV subunit A	IC_{50} = 10	Nucleic Acids Res., (2017) 45:D1:D945
BMY 45243	CHEMBL273348	DNA gyrase	IC_{50} = 7.42	J. Med. Chem., (1993) 36:14:1964
	CHEMBL276583		IC_{50} = 2.26	J. Med. Chem., (1993) 36:7:871
			IC_{50} = 9.03	J. Med. Chem., (1993) 36:14:1964
	CHEMBL541279		IC_{50} = 0.4	J. Med. Chem., (1988) 31:8:1586
	CHEMBL544247		IC_{50} = 2.97	
Chinfloxacin	CHEMBL32	DNA gyrase	IC_{50} = 0.3	Bioorg. Med. Chem. Lett., (2006) 16:5:1272
			IC_{50} = 10.91	Bioorg. Med. Chem., (2009) 17:19:6879
			IC_{50} = 18	J. Med. Chem., (2011) 54:9:3418
			IC_{50} = 27.5	Antimicrob. Agents Chemothe., (2007) 51:7:2445
			IC_{50} = 28	J. Med. Chem., (2007) 50:2:199
			IC_{50} = 7.7	J. Med. Chem., (2011) 54:9:3268
		DNA gyrase subunit A	IC_{50} = 9.2	Antimicrob. Agents Chemother., (2010) 54:8:3478
		DNA gyrase subunit A/subunit B	IC_{50} = 2.5	Antimicrob. Agents Chemothe., (2008) 52:8:2909
		DNA gyrase subunit B	IC_{50} > 50	Bioorg. Med. Chem., (2014) 22:17:4924
				Bioorg. Med. Chem., (2015) 23:9:2062
				Eur. J. Med. Chem., (2015) 103:null:1
		Topoisomerase IV subunit A	IC_{50} = 0.8	Bioorg. Med. Chem. Lett., (2006) 16:5:1272
			IC_{50} = 10	Nucleic Acids Res., (2017) 45:D1:D945
CP 135803	CHEMBL1201760	Topoisomerase IV subunit A	IC_{50} = 10	Nucleic Acids Res., (2017) 45:D1:D945
	CHEMBL1202	DNA gyrase	IC_{50} = 0.6	J. Med. Chem., (1992) 35:1:94
			IC_{50} = 0.72	J. Med. Chem., (2011) 54:6:1539
	CHEMBL120948		IC_{50} = 4.1	J. Med. Chem., (2003) 46:17:3655
	CHEMBL1210954	Topoisomerase IV subunit A	IC_{50} = 10	Nucleic Acids Res., (2017) 45:D1:D945
	CHEMBL1213456			

Bioactive molecule	Similar molecule	Similar molecule target	Activity value of similar molecule (μM)	Reference
	CHEMBL122691	DNA gyrase	IC_{50} = 8.84	J. Med. Chem., (2003) 46:17:3655
	CHEMBL122698		IC_{50} = 0.51	
	CHEMBL123246		IC_{50} = 4.26	
	CHEMBL1257096	Topoisomerase IV subunit A	IC_{50} = 10	Nucleic Acids Res., (2017) 45:D1:D945
	CHEMBL136779	DNA gyrase	IC_{50} = 6.97	J. Med. Chem., (1996) 39:25:4952
	CHEMBL141308		IC_{50} = 7.64	
	CHEMBL149425		IC_{50} = 36.47	J. Med. Chem., (1988) 31:3:503
	CHEMBL15087		IC_{50} = 0.75	J. Med. Chem., (1993) 36:7:871
			IC_{50} = 7.85	J. Med. Chem., (1993) 36:14:1964
	CHEMBL15186		IC_{50} = 7.51	J. Med. Chem., (1988) 31:3:503
	CHEMBL15247		IC_{50} = 6.64	
	CHEMBL15296		IC_{50} = 1.45	J. Med. Chem., (1993) 36:7:871
	CHEMBL15391		IC_{50} = 7.66	J. Med. Chem., (1988) 31:3:503
	CHEMBL15511	Topoisomerase IV subunit A	IC_{50} = 10	Nucleic Acids Res., (2017) 45:D1:D945
	CHEMBL15549	DNA gyrase	IC_{50} = 8.02	J. Med. Chem., (1988) 31:3:503
				J. Med. Chem., (1991) 34:2:656
	CHEMBL15613		IC_{50} = 5.6	Antimicrob. Agents Chemother., (2007) 51:1:119
		Topoisomerase IV	IC_{50} = 4.2	
	CHEMBL157548	Topoisomerase IV subunit A	IC_{50} = 10	Nucleic Acids Res., (2017) 45:D1:D945
	CHEMBL159024	DNA gyrase	IC_{50} = 16.78	J. Med. Chem., (1991) 34:2:656
	CHEMBL159030		IC_{50} = 4.85	
	CHEMBL159188		IC_{50} = 36.67	
	CHEMBL160192		IC_{50} = 14.09	
	CHEMBL160302		IC_{50} = 2.23	
	CHEMBL160661		IC_{50} = 7.75	
	CHEMBL160662		IC_{50} = 8.15	
	CHEMBL160838		IC_{50} = 4.2	
	CHEMBL184167	Topoisomerase IV subunit A	IC_{50} = 0.73	Bioorg. Med. Chem. Lett., (2004) 14:20:5193
	CHEMBL186870		IC_{50} = 0.41	
	CHEMBL187796		IC_{50} = 0.72	
	CHEMBL2110220	DNA gyrase	IC_{50} = 1.39	J. Med. Chem., (1993) 36:7:871
			IC_{50} = 20.87	
	CHEMBL2110319			
	CHEMBL274469		IC_{50} = 7.68	J. Med. Chem., (1988) 31:3:503
				J. Med. Chem., (1991) 34:2:656
	CHEMBL275174		IC_{50} = 7.65	J. Med. Chem., (1988) 31:3:503
	CHEMBL277100	Topoisomerase IV subunit A	IC_{50} = 10	Nucleic Acids Res., (2017) 45:D1:D945
	CHEMBL278255	DNA gyrase	IC_{50} = 0.55	J. Med. Chem., (2003) 46:17:3655
			IC_{50} = 3.14	J. Med. Chem., (2008) 51:11:3238
		Topoisomerase IV	IC_{50} = 5.17	
		Topoisomerase IV subunit A	IC_{50} = 10	Nucleic Acids Res., (2017) 45:D1:D945
	CHEMBL295433			
	CHEMBL31	DNA gyrase	IC_{50} = 0.5	Bioorg. Med. Chem. Lett., (2006) 16:5:1272

Bioactive molecule	Similar molecule	Similar molecule target	Activity value of similar molecule (μM)	Reference
				J. Med. Chem., (2006) 49:22:6435
			IC_{50} = 8.58	Bioorg. Med. Chem., (2009) 17:19:6879
		DNA gyrase subunit A	IC_{50} = 9.4	Antimicrob. Agents Chemother., (2010) 54:8:3478
		Topoisomerase IV subunit A	IC_{50} = 1.2	Bioorg. Med. Chem. Lett., (2006) 16:5:1272
			IC_{50} = 10	Nucleic Acids Res., (2017) 45:D1:D945
	CHEMBL32	DNA gyrase	IC_{50} = 0.3	Bioorg. Med. Chem. Lett., (2006) 16:5:1272
			IC_{50} = 10.91	Bioorg. Med. Chem., (2009) 17:19:6879
			IC_{50} = 18	J. Med. Chem., (2011) 54:9:3418
			IC_{50} = 27.5	Antimicrob. Agents Chemothe., (2007) 51:7:2445
			IC_{50} = 28	J. Med. Chem., (2007) 50:2:199
			IC_{50} = 7.7	J. Med. Chem., (2011) 54:9:3268
		DNA gyrase subunit A	IC_{50} = 9.2	Antimicrob. Agents Chemother., (2010) 54:8:3478
		DNA gyrase subunit A/subunit B	IC_{50} = 2.5	Antimicrob. Agents Chemothe., (2008) 52:8:2909
		DNA gyrase subunit B	IC_{50} > 50	Bioorg. Med. Chem., (2014) 22:17:4924
				Eur. J. Med. Chem., (2015) 103:null:1
		Topoisomerase IV subunit A	IC_{50} = 0.8	Bioorg. Med. Chem. Lett., (2006) 16:5:1277
	CHEMBL330779	DNA gyrase	IC_{50} < 1.38	J. Med. Chem., (1991) 34:2:656
	CHEMBL331076		IC_{50} = 8.81	J. Med. Chem., (2003) 46:17:3655
	CHEMBL331987		IC_{50} = 7.93	
	CHEMBL3350260		IC_{50} = 2.09	J. Med. Chem., (1993) 36:7:871
	CHEMBL342273		IC_{50} = 83.48	J. Med. Chem., (1996) 39:25:4952
	CHEMBL343603		IC_{50} = 16.42	
	CHEMBL344447		IC_{50} = 9.37	
	CHEMBL348879		IC_{50} = 7.45	J. Med. Chem., (1991) 34:2:656
	CHEMBL352000		IC_{50} = 16.18	
	CHEMBL356984		IC_{50} > 222.98	J. Med. Chem., (1988) 31:3:503
	CHEMBL357882		IC_{50} = 97.17	J. Med. Chem., (1996) 39:25:4952
	CHEMBL359193		IC_{50} = 32.82	J. Med. Chem., (1988) 31:3:503
	CHEMBL416273		IC_{50} = 10.66	J. Med. Chem., (1996) 39:25:4952
	CHEMBL420897		IC_{50} = 17.91	J. Med. Chem., (2003) 46:17:3655
	CHEMBL421101		IC_{50} = 8.3	
	CHEMBL421277		IC_{50} = 2.32	J. Med. Chem., (1991) 34:2:656
	CHEMBL422131		IC_{50} > 246.07	J. Med. Chem., (1988) 31:3:503
	CHEMBL42299		IC_{50} > 492.09	
	CHEMBL429961		IC_{50} = 4.34	J. Med. Chem., (1996) 39:25:4952
	CHEMBL47028		IC_{50} = 7.4	J. Med. Chem., (1988) 31:3:503
	CHEMBL540589		IC_{50} = 5.53	J. Med. Chem., (1991) 34:2:656
	CHEMBL544407		IC_{50} = 7.99	
	CHEMBL561	Topoisomerase IV subunit A	IC_{50} = 10	Nucleic Acids Res., (2017) 45:D1:D945
	CHEMBL583			
	CHEMBL6210	DNA gyrase	IC_{50} = 3.58	J. Med. Chem., (1988) 31:3:503

Bioactive molecule	Similar molecule	Similar molecule target	Activity value of similar molecule (μM)	Reference
	CHEMBL8		IC_{50} = 0.2	J. Med. Chem., (2006) 49:22:6435
			IC_{50} = 0.3	Bioorg. Med. Chem. Lett., (2006) 16:5:1272
			IC_{50} = 0.45	Antimicrob. Agents Chemother., (2010) 54:7:3011
			IC_{50} = 0.97	Bioorg. Med. Chem. Lett., (2012) 22:7:2428
			IC_{50} = 61.7	Antimicrob. Agents Chemother., (2007) 51:7:2445
			IC_{50} = 62	J. Med. Chem., (2007) 50:2:199
		Topoisomerase IV	IC_{50} = 8	J. Med. Chem., (2013) 56:18:7396
		Topoisomerase IV subunit A	IC_{50} = 10	Nucleic Acids Res., (2017) 45:D1:D945
	CHEMBL850	DNA gyrase	IC_{50} = 0.64	J. Med. Chem., (2004) 47:14:3693
		Topoisomerase IV subunit A	IC_{50} = 10	Nucleic Acids Res., (2017) 45:D1:D945
CP 74667	CHEMBL1201760	Topoisomerase IV subunit A	IC_{50} = 10	Nucleic Acids Res., (2017) 45:D1:D945
	CHEMBL15511			
	CHEMBL157548			
	CHEMBL159030	DNA gyrase	IC_{50} = 4.85	J. Med. Chem., (1991) 34:2:656
	CHEMBL159188		IC_{50} = 36.67	
	CHEMBL160244		IC_{50} = 15.8	
	CHEMBL160302		IC_{50} = 2.23	
	CHEMBL160415		IC_{50} = 15.1	
	CHEMBL160661		IC_{50} = 7.75	
	CHEMBL160662		IC_{50} = 8.15	
	CHEMBL160786		IC_{50} = 9.1	
	CHEMBL184489	Topoisomerase IV subunit A	IC_{50} = 0.85	Bioorg. Med. Chem. Lett., (2004) 14:20:5193
	CHEMBL32	DNA gyrase	IC_{50} = 0.3	Bioorg. Med. Chem. Lett., (2006) 16:5:1272
			IC_{50} = 10.91	Bioorg. Med. Chem., (2009) 17:19:6879
			IC_{50} = 18	J. Med. Chem., (2011) 54:9:3418
			IC_{50} = 27.5	Antimicrob. Agents Chemothe., (2007) 51:7:2445
			IC_{50} = 28	J. Med. Chem., (2007) 50:2:199
			IC_{50} = 7.7	J. Med. Chem., (2011) 54:9:3268
		DNA gyrase subunit A	IC_{50} = 9.2	Antimicrob. Agents Chemother., (2010) 54:8:3478
		DNA gyrase subunit A/subunit B	IC_{50} = 2.5	Antimicrob. Agents Chemothe., (2008) 52:8:2909
		DNA gyrase subunit B	IC_{50} > 50	Bioorg. Med. Chem., (2014) 22:17:4924
				Bioorg. Med. Chem., (2015) 23:9:2062
				Eur. J. Med. Chem., (2015) 103:null:1
		Topoisomerase IV subunit A	IC_{50} = 0.8	Bioorg. Med. Chem. Lett., (2006) 16:5:1272
			IC_{50} = 10	Nucleic Acids Res., (2017) 45:D1:D945
	CHEMBL343603	DNA gyrase	IC_{50} = 16.42	J. Med. Chem., (1996) 39:25:4952
	CHEMBL345171		IC_{50} = 8.39	J. Med. Chem., (1991) 34:2:656
	CHEMBL348879		IC_{50} = 7.45	
	CHEMBL349801		IC_{50} = 9.76	

Bioactive molecule	Similar molecule	Similar molecule target	Activity value of similar molecule (µM)	Reference
	CHEMBL351693		IC_{50} = 8.08	
	CHEMBL352000		IC_{50} = 16.18	
	CHEMBL365638	Topoisomerase IV subunit A	IC_{50} = 0.31	Bioorg. Med. Chem. Lett., (2004) 14:20:5193
	CHEMBL429961	DNA gyrase	IC_{50} = 4.34	J. Med. Chem., (1996) 39:25:4952
	CHEMBL433517		IC_{50} = 14.09	J. Med. Chem., (1991) 34:2:656
	CHEMBL435838		IC_{50} = 7.73	
	CHEMBL540589		IC_{50} = 5.53	
	CHEMBL544407		IC_{50} = 7.99	
	CHEMBL553807		IC_{50} = 9.76	
	CHEMBL555158		IC_{50} = 43.81	
CP 99433	CHEMBL121176	DNA gyrase	IC_{50} = 4.62	J. Med. Chem., (2003) 46:17:3655
	CHEMBL142729		IC_{50} = 8.66	J. Med. Chem., (1991) 34:2:656
	CHEMBL15314		IC_{50} = 2.17	J. Med. Chem., (1993) 36:7:871
	CHEMBL157587		IC_{50} = 15.73	J. Med. Chem., (1991) 34:2:656
	CHEMBL160468		IC_{50} = 18.3	
	CHEMBL160497		IC_{50} = 14.77	
	CHEMBL2110221		IC_{50} = 2.08	J. Med. Chem., (1993) 36:7:871
	CHEMBL2110225		IC_{50} = 1.16	
	CHEMBL2110321		IC_{50} = 2.08	
			IC_{50} = 6.94	
	CHEMBL273348		IC_{50} = 7.42	J. Med. Chem., (1993) 36:14:1964
	CHEMBL276583		IC_{50} = 2.26	J. Med. Chem., (1993) 36:7:871
	CHEMBL276583		IC_{50} = 9.03	J. Med. Chem., (1993) 36:14:1964
	CHEMBL3350261		IC_{50} = 6.94	J. Med. Chem., (1993) 36:7:871
	CHEMBL345900		IC_{50} = 17.58	J. Med. Chem., (1991) 34:2:656
	CHEMBL348355		IC_{50} = 15.73	
	CHEMBL420884		IC_{50} = 68.37	J. Med. Chem., (2003) 46:17:3655
	CHEMBL428	Topoisomerase IV subunit A	IC_{50} = 10	Nucleic Acids Res., (2017) 45:D1:D945
	CHEMBL430	DNA gyrase	IC_{50} = 0.5	Bioorg. Med. Chem. Lett., (2006) 16:5:1272
			IC_{50} = 5.6	Antimicrob. Agents Chemother., (2007) 51:7:2445
				J. Med. Chem., (2007) 50:2:199
				J. Med. Chem., (2011) 54:9:3268
				J. Med. Chem., (2011) 54:9:3418
		Topoisomerase IV subunit A	IC_{50} = 0.3	Bioorg. Med. Chem. Lett., (2006) 16:5:1272
			IC_{50} = 10	Nucleic Acids Res., (2017) 45:D1:D945
	CHEMBL541279	DNA gyrase	IC_{50} = 0.4	J. Med. Chem., (1988) 31:8:1586
	CHEMBL542832		IC_{50} = 0.99	
	CHEMBL544247		IC_{50} = 2.97	
	CHEMBL544647		IC_{50} = 47	J. Med. Chem., (1991) 34:2:656
	CHEMBL544878		IC_{50} = 20.14	
	CHEMBL545344		IC_{50} = 8.06	
	CHEMBL558413		IC_{50} = 10.6	
	CHEMBL58300		IC_{50} = 11.54	J. Med. Chem., (1993) 36:14:1964

Bioactive molecule	Similar molecule	Similar molecule target	Activity value of similar molecule (μM)	Reference
	CHEMBL58513			
	CHEMBL61949		IC_{50} = 150.45	J. Med. Chem., (1991) 34:2:656
			IC_{50} > 75.22	J. Med. Chem., (1993) 36:14:1964
Desfluorociprofloxacin (SQ 4004)	CHEMBL1202	DNA gyrase	IC_{50} = 0.6	J. Med. Chem., (1992) 35:1:94
			IC_{50} = 0.72	J. Med. Chem., (2011) 54:6:1539
	CHEMBL120948		IC_{50} = 4.1	J. Med. Chem., (2003) 46:17:3655
	CHEMBL1213456	Topoisomerase IV subunit A	IC_{50} = 10	Nucleic Acids Res., (2017) 45:D1:D945
	CHEMBL122691	DNA gyrase	IC_{50} = 8.84	J. Med. Chem., (2003) 46:17:3655
	CHEMBL122698		IC_{50} = 0.51	
	CHEMBL123246		IC_{50} = 4.26	
	CHEMBL145932		IC_{50} = 10.11	J. Med. Chem., (1996) 39:25:4952
	CHEMBL149425		IC_{50} = 36.47	J. Med. Chem., (1988) 31:3:503
	CHEMBL15087		IC_{50} = 0.75	J. Med. Chem., (1993) 36:7:871
			IC_{50} = 7.85	J. Med. Chem., (1993) 36:14:1964
	CHEMBL15511	Topoisomerase IV subunit A	IC_{50} = 10	Nucleic Acids Res., (2017) 45:D1:D945
	CHEMBL15549	DNA gyrase	IC_{50} = 8.02	J. Med. Chem., (1988) 31:3:503
				J. Med. Chem., (1991) 34:2:656
	CHEMBL274469		IC_{50} = 7.68	J. Med. Chem., (1988) 31:3:503
				J. Med. Chem., (1991) 34:2:656
	CHEMBL275698		IC_{50} = 11.5	J. Med. Chem., (1993) 36:14:1964
	CHEMBL295433	Topoisomerase IV subunit A	IC_{50} = 10	Nucleic Acids Res., (2017) 45:D1:D945
	CHEMBL31	DNA gyrase	IC_{50} = 0.5	Bioorg. Med. Chem. Lett., (2006) 16:5:1272
				J. Med. Chem., (2006) 49:22:6435
			IC_{50} = 8.58	Bioorg. Med. Chem., (2009) 17:19:6879
		DNA gyrase subunit A	IC_{50} = 9.4	Antimicrob. Agents Chemother., (2010) 54:8:3478
		Topoisomerase IV subunit A	IC_{50} = 1.2	Bioorg. Med. Chem. Lett., (2006) 16:5:1272
			IC_{50} = 10	Nucleic Acids Res., (2017) 45:D1:D945
	CHEMBL330779	DNA gyrase	IC_{50} < 1.38	J. Med. Chem., (1991) 34:2:656
	CHEMBL331076		IC_{50} = 8.81	J. Med. Chem., (2003) 46:17:3655
	CHEMBL342273		IC_{50} = 83.48	J. Med. Chem., (1996) 39:25:4952
	CHEMBL343603		IC_{50} = 16.42	
	CHEMBL344447		IC_{50} = 9.37	
	CHEMBL357882		IC_{50} = 97.17	
	CHEMBL416273		IC_{50} = 10.66	
	CHEMBL420897		IC_{50} = 17.91	J. Med. Chem., (2003) 46:17:3655
	CHEMBL421101		IC_{50} = 8.3	
	CHEMBL421277		IC_{50} = 2.32	J. Med. Chem., (1991) 34:2:656
	CHEMBL42299		IC_{50} > 492.09	J. Med. Chem., (1988) 31:3:503
	CHEMBL429961		IC_{50} = 4.34	J. Med. Chem., (1996) 39:25:4952
	CHEMBL47028		IC_{50} = 7.4	J. Med. Chem., (1988) 31:3:503
	CHEMBL58226		IC_{50} = 2.74	J. Med. Chem., (1993) 36:14:1964
	CHEMBL583	Topoisomerase IV subunit A	IC_{50} = 10	Nucleic Acids Res., (2017) 45:D1:D945

Bioactive molecule	Similar molecule	Similar molecule target	Activity value of similar molecule (μM)	Reference
	CHEMBL8	DNA gyrase	IC_{50} = 0.2	J. Med. Chem., (2006) 49:22:6435
			IC_{50} = 0.3	Bioorg. Med. Chem. Lett., (2006) 16:5:1272
			IC_{50} = 0.45	Antimicrob. Agents Chemother., (2010) 54:7:3011
			IC_{50} = 0.97	Bioorg. Med. Chem. Lett., (2012) 22:7:2428
			IC_{50} = 61.7	Antimicrob. Agents Chemother., (2007) 51:7:2445
			IC_{50} = 62	J. Med. Chem., (2007) 50:2:199
		Topoisomerase IV	IC_{50} = 8	J. Med. Chem., (2013) 56:18:7396
		Topoisomerase IV subunit A	IC_{50} = 10	Nucleic Acids Res., (2017) 45:D1:D945
E 3604	CHEMBL68262	DNA gyrase	IC_{50} = 93.24	J. Med. Chem., (1986) 29:3:394
		Topoisomerase IV subunit A	IC_{50} = 10	Nucleic Acids Res., (2017) 45:D1:D945
E 3846	CHEMBL68262	DNA gyrase	IC_{50} = 93.24	J. Med. Chem., (1986) 29:3:394
		Topoisomerase IV subunit A	IC_{50} = 10	Nucleic Acids Res., (2017) 45:D1:D945
E 4474	CHEMBL278255	DNA gyrase	IC_{50} = 0.55	J. Med. Chem., (2003) 46:17:3655
			IC_{50} = 3.14	J. Med. Chem., (2008) 51:11:3238
		Topoisomerase IV	IC_{50} = 5.17	
		Topoisomerase IV subunit A	IC_{50} = 10	Nucleic Acids Res., (2017) 45:D1:D945
	CHEMBL331076	DNA gyrase	IC_{50} = 8.81	J. Med. Chem., (2003) 46:17:3655
E 4534	CHEMBL132468	Topoisomerase IV subunit A	IC_{50} = 10	Nucleic Acids Res., (2017) 45:D1:D945
E 4535	CHEMBL132468	Topoisomerase IV subunit A	IC_{50} = 10	Nucleic Acids Res., (2017) 45:D1:D945
E 4695	CHEMBL276583	DNA gyrase	IC_{50} = 2.26	J. Med. Chem., (1993) 36:7:871
			IC_{50} = 9.03	J. Med. Chem., (1993) 36:14:1964
	CHEMBL3633518		IC_{50} = 0.29	Eur. J. Med. Chem., (2015) 103:null:354
	CHEMBL420884		IC_{50} = 68.37	J. Med. Chem., (2003) 46:17:3655
Esafloxacin	CHEMBL102572	DNA gyrase	IC_{50} = 16.38	J. Med. Chem., (1986) 29:3:394
	CHEMBL121176		IC_{50} = 4.62	J. Med. Chem., (2003) 46:17:3655
	CHEMBL142729		IC_{50} = 8.66	J. Med. Chem., (1991) 34:2:656
	CHEMBL15314		IC_{50} = 2.17	J. Med. Chem., (1993) 36:7:871
	CHEMBL160468		IC_{50} = 18.3	J. Med. Chem., (1991) 34:2:656
	CHEMBL2110221		IC_{50} = 2.08	J. Med. Chem., (1993) 36:7:871
	CHEMBL2110225		IC_{50} = 1.16	
	CHEMBL2110321		IC_{50} = 2.08	
			IC_{50} = 6.94	
	CHEMBL273348		IC_{50} = 7.42	J. Med. Chem., (1993) 36:14:1964
	CHEMBL276583		IC_{50} = 2.26	J. Med. Chem., (1993) 36:7:871
			IC_{50} = 9.03	J. Med. Chem., (1993) 36:14:1964
	CHEMBL3350261		IC_{50} = 6.94	J. Med. Chem., (1993) 36:7:871
	CHEMBL420884		IC_{50} = 68.37	J. Med. Chem., (2003) 46:17:3655
	CHEMBL430		IC_{50} = 0.5	Bioorg. Med. Chem. Lett., (2006) 16:5:1272
			IC_{50} = 5.6	Antimicrob. Agents Chemother., (2007) 51:7:2445
				J. Med. Chem., (2007) 50:2:199
				J. Med. Chem., (2011) 54:9:3268

Bioactive molecule	Similar molecule	Similar molecule target	Activity value of similar molecule (µM)	Reference
				J. Med. Chem., (2011) 54:9:3418
		Topoisomerase IV subunit A	IC_{50} = 0.3	Bioorg. Med. Chem. Lett., (2006) 16:5:1272
			IC_{50} = 10	Nucleic Acids Res., (2017) 45:D1:D945
	CHEMBL541279	DNA gyrase	IC_{50} = 0.4	J. Med. Chem., (1988) 31:8:1586
	CHEMBL542832		IC_{50} = 0.99	
	CHEMBL544247		IC_{50} = 2.97	
	CHEMBL61949		IC_{50} = 150.45	J. Med. Chem., (1991) 34:2:656
			IC_{50} > 75.22	J. Med. Chem., (1993) 36:14:1964
	CHEMBL826		IC_{50} = 87.41	J. Med. Chem., (1986) 29:3:394
		DNA gyrase subunit A/subunit B	IC_{50} = 14	Antimicrob. Agents Chemother., (2008) 52:8:2909
		Topoisomerase IV subunit A	IC_{50} = 10	Nucleic Acids Res., (2017) 45:D1:D945
FA 103	CHEMBL1201760	Topoisomerase IV subunit A	IC_{50} = 10	Nucleic Acids Res., (2017) 45:D1:D945
	CHEMBL149425	DNA gyrase	IC_{50} = 36.47	J. Med. Chem., (1988) 31:3:503
	CHEMBL159030		IC_{50} = 4.85	J. Med. Chem., (1991) 34:2:656
	CHEMBL160302		IC_{50} = 2.23	
	CHEMBL274469		IC_{50} = 7.68	J. Med. Chem., (1988) 31:3:503
				J. Med. Chem., (1991) 34:2:656
	CHEMBL348879		IC_{50} = 7.45	
	CHEMBL422131		IC_{50} > 246.07	J. Med. Chem., (1988) 31:3:503
Fortimicin AK	CHEMBL431061	30S ribosomal subunit	IC_{50} = 3.9	Bioorg. Med. Chem. Lett., (2002) 12:23:3367
Fortimicin AM	CHEMBL2206195	30S ribosomal subunit	IC_{50} = 1.9	J. Med. Chem., (2012) 55:23:10630
	CHEMBL431061		IC_{50} = 3.9	Bioorg. Med. Chem. Lett., (2002) 12:23:3367
Fortimicin AN	CHEMBL2206184	30S ribosomal subunit	IC_{50} = 1.8	J. Med. Chem., (2012) 55:23:10630
	CHEMBL2206185			
	CHEMBL2206189		IC_{50} = 1	
	CHEMBL2206192		IC_{50} = 2.3	
	CHEMBL2206193		IC_{50} = 0.8	
Fortimicin AO	CHEMBL2206188	30S ribosomal subunit	IC_{50} = 1.1	J. Med. Chem., (2012) 55:23:10630
	CHEMBL2206190		IC_{50} = 2	
	CHEMBL2206191		IC_{50} = 2.1	
	CHEMBL373500		IC_{50} = 0.5	
	CHEMBL431061		IC_{50} = 3.9	Bioorg. Med. Chem. Lett., (2002) 12:23:3367
Fortimicin AP	CHEMBL2206194	30S ribosomal subunit	IC_{50} = 1.1	J. Med. Chem., (2012) 55:23:10630
	CHEMBL2206195		IC_{50} = 1.9	
	CHEMBL431061		IC_{50} = 3.9	
Fortimicin KL_1	CHEMBL2206188	30S ribosomal subunit	IC_{50} = 1.1	J. Med. Chem., (2012) 55:23:10630
	CHEMBL2206190		IC_{50} = 2	
	CHEMBL2206191		IC_{50} = 2.1	
	CHEMBL373500		IC_{50} = 0.5	
	CHEMBL431061		IC_{50} = 3.9	Bioorg. Med. Chem. Lett., (2002) 12:23:3367
Gentamicin A_1	CHEMBL2206187	30S ribosomal subunit	IC_{50} = 0.2	J. Med. Chem., (2012) 55:23:10630

Bioactive molecule	Similar molecule	Similar molecule target	Activity value of similar molecule (μM)	Reference
	CHEMBL2206188		IC_{50} = 1.1	
	CHEMBL2206189		IC_{50} = 1	
	CHEMBL2206190		IC_{50} = 2	
	CHEMBL2206191		IC_{50} = 2.1	
	CHEMBL2206192		IC_{50} = 2.3	
	CHEMBL2206193		IC_{50} = 0.8	
	CHEMBL2206194		IC_{50} = 1.1	
	CHEMBL2206195		IC_{50} = 1.9	
	CHEMBL373500		IC_{50} = 0.5	
	CHEMBL431061		IC_{50} = 3.9	Bioorg. Med. Chem. Lett., (2002) 12:23:3367
Gentamicin A_2	CHEMBL2206187	30S ribosomal subunit	IC_{50} = 0.2	J. Med. Chem., (2012) 55:23:10630
	CHEMBL2206188		IC_{50} = 1.1	
	CHEMBL2206190		IC_{50} = 2	
	CHEMBL2206191		IC_{50} = 2.1	
	CHEMBL2206192		IC_{50} = 2.3	
	CHEMBL2206193		IC_{50} = 0.8	
	CHEMBL2206194		IC_{50} = 1.1	
	CHEMBL2206195		IC_{50} = 1.9	
	CHEMBL373500		IC_{50} = 0.5	
	CHEMBL431061		IC_{50} = 3.9	Bioorg. Med. Chem. Lett., (2002) 12:23:3367
Gentamicin A_3	CHEMBL2206187	30S ribosomal subunit	IC_{50} = 0.2	J. Med. Chem., (2012) 55:23:10630
	CHEMBL2206188		IC_{50} = 1.1	
	CHEMBL2206189		IC_{50} = 1	
	CHEMBL2206190		IC_{50} = 2	
	CHEMBL2206191		IC_{50} = 2.1	
	CHEMBL2206192		IC_{50} = 2.3	
	CHEMBL2206193		IC_{50} = 0.8	
	CHEMBL2206194		IC_{50} = 1.1	
	CHEMBL2206195		IC_{50} = 1.9	
	CHEMBL373500		IC_{50} = 0.5	
	CHEMBL431061		IC_{50} = 3.9	Bioorg. Med. Chem. Lett., (2002) 12:23:3367
Gentamicin A_4	CHEMBL2206184	30S ribosomal subunit	IC_{50} = 1.8	J. Med. Chem., (2012) 55:23:10630
	CHEMBL2206185			
	CHEMBL2206187		IC_{50} = 0.2	
	CHEMBL2206188		IC_{50} = 1.1	
	CHEMBL2206189		IC_{50} = 1	
	CHEMBL2206190		IC_{50} = 2	
	CHEMBL2206191		IC_{50} = 2.1	
	CHEMBL2206192		IC_{50} = 2.3	
	CHEMBL2206193		IC_{50} = 0.8	
	CHEMBL373500		IC_{50} = 0.5	
Gentamicin X_2	CHEMBL3039594	30S ribosomal subunit	IC_{50} = 0.03	J. Med. Chem., (2012) 55:23:10630
Hybrimycin C_1	CHEMBL2206184	30S ribosomal subunit	IC_{50} = 1.8	J. Med. Chem., (2012) 55:23:10630

Bioactive molecule	Similar molecule	Similar molecule target	Activity value of similar molecule (μM)	Reference
	CHEMBL2206185			
	CHEMBL2206187		IC_{50} = 0.2	
	CHEMBL2206188		IC_{50} = 1.1	
	CHEMBL2206189		IC_{50} = 1	
	CHEMBL2206190		IC_{50} = 2	
	CHEMBL2206191		IC_{50} = 2.1	
	CHEMBL2206192		IC_{50} = 2.3	
	CHEMBL2206193		IC_{50} = 0.8	
	CHEMBL2206194		IC_{50} = 1.1	
	CHEMBL2206195		IC_{50} = 1.9	
	CHEMBL2206196		IC_{50} = 0.05	
	CHEMBL373500		IC_{50} = 0.5	
	CHEMBL431061		IC_{50} = 3.9	Bioorg. Med. Chem. Lett., (2002) 12:23:3367
Hybrimycin C_2	CHEMBL2206184	30S ribosomal subunit	IC_{50} = 1.8	J. Med. Chem., (2012) 55:23:10630
	CHEMBL2206185			
	CHEMBL2206187		IC_{50} = 0.2	
	CHEMBL2206188		IC_{50} = 1.1	
	CHEMBL2206189		IC_{50} = 1	
	CHEMBL2206190		IC_{50} = 2	
	CHEMBL2206191		IC_{50} = 2.1	
	CHEMBL2206192		IC_{50} = 2.3	
	CHEMBL2206193		IC_{50} = 0.8	
	CHEMBL2206194		IC_{50} = 1.1	
	CHEMBL2206195		IC_{50} = 1.9	
	CHEMBL2206196		IC_{50} = 0.05	
	CHEMBL373500		IC_{50} = 0.5	
	CHEMBL431061		IC_{50} = 3.9	Bioorg. Med. Chem. Lett., (2002) 12:23:3367
Istamycin AO	CHEMBL2206188	30S ribosomal subunit	IC_{50} = 1.1	J. Med. Chem., (2012) 55:23:10630
	CHEMBL2206190		IC_{50} = 2	
	CHEMBL2206191		IC_{50} = 2.1	
	CHEMBL2206194		IC_{50} = 1.1	
	CHEMBL2206195		IC_{50} = 1.9	
	CHEMBL2206196		IC_{50} = 0.05	
	CHEMBL373500		IC_{50} = 0.5	
	CHEMBL431061		IC_{50} = 3.9	Bioorg. Med. Chem. Lett., (2002) 12:23:3367
Istamycin AP (sannamycin E)	CHEMBL431061	30S ribosomal subunit	IC_{50} = 3.9	Bioorg. Med. Chem. Lett., (2002) 12:23:3367
Istamycin KL_1	CHEMBL2206188	30S ribosomal subunit	IC_{50} = 1.1	J. Med. Chem., (2012) 55:23:10630
	CHEMBL2206190		IC_{50} = 2	
	CHEMBL2206191		IC_{50} = 2.1	
	CHEMBL2206194		IC_{50} = 1.1	
	CHEMBL2206195		IC_{50} = 1.9	
	CHEMBL2206196		IC_{50} = 0.05	
	CHEMBL373500		IC_{50} = 0.5	

Bioactive molecule	Similar molecule	Similar molecule target	Activity value of similar molecule (µM)	Reference
	CHEMBL431061		IC_{50} = 3.9	Bioorg. Med. Chem. Lett., (2002) 12:23:3367
Kanamycin D	CHEMBL2206187	30S ribosomal subunit	IC_{50} = 0.2	J. Med. Chem., (2012) 55:23:10630
	CHEMBL2206188		IC_{50} = 1.1	
	CHEMBL2206189		IC_{50} = 1	
	CHEMBL2206190		IC_{50} = 2	
	CHEMBL2206191		IC_{50} = 2.1	
	CHEMBL2206192		IC_{50} = 2.3	
	CHEMBL2206193		IC_{50} = 0.8	
	CHEMBL2206194		IC_{50} = 1.1	
	CHEMBL2206195		IC_{50} = 1.9	
	CHEMBL2206196		IC_{50} = 0.05	
	CHEMBL373500		IC_{50} = 0.5	
	CHEMBL431061		IC_{50} = 3.9	Bioorg. Med. Chem. Lett., (2002) 12:23:3367
Kanamycin X	CHEMBL2206187	30S ribosomal subunit	IC_{50} = 0.2	J. Med. Chem., (2012) 55:23:10630
	CHEMBL2206188		IC_{50} = 1.1	
	CHEMBL2206190		IC_{50} = 2	
	CHEMBL2206191		IC_{50} = 2.1	
	CHEMBL2206194		IC_{50} = 1.1	
	CHEMBL2206195		IC_{50} = 1.9	
	CHEMBL2206196		IC_{50} = 0.05	
	CHEMBL373500		IC_{50} = 0.5	
	CHEMBL431061		IC_{50} = 3.9	Bioorg. Med. Chem. Lett., (2002) 12:23:3367
Lividamine (nebramycin IX)	CHEMBL2206188	30S ribosomal subunit	IC_{50} = 1.1	J. Med. Chem., (2012) 55:23:10630
	CHEMBL2206190		IC_{50} = 2	
	CHEMBL2206191		IC_{50} = 2.1	
	CHEMBL2206196		IC_{50} = 0.05	
	CHEMBL373500		IC_{50} = 0.5	
	CHEMBL431061		IC_{50} = 3.9	Bioorg. Med. Chem. Lett., (2002) 12:23:3367
MF 5101	CHEMBL1202	DNA gyrase	IC_{50} = 0.6	J. Med. Chem., (1992) 35:1:94
			IC_{50} = 0.72	J. Med. Chem., (2011) 54:6:1539
	CHEMBL120948		IC_{50} = 4.1	J. Med. Chem., (2003) 46:17:3655
	CHEMBL1210954	Topoisomerase IV subunit A	IC_{50} = 10	Nucleic Acids Res., (2017) 45:D1:D945
	CHEMBL1213456			
	CHEMBL122691	DNA gyrase	IC_{50} = 8.84	J. Med. Chem., (2003) 46:17:3655
	CHEMBL122698		IC_{50} = 0.51	
	CHEMBL123246		IC_{50} = 4.26	
	CHEMBL124970		IC_{50} = 9.24	J. Med. Chem., (1996) 39:25:4952
	CHEMBL136779		IC_{50} = 6.97	
	CHEMBL145932		IC_{50} = 10.11	
	CHEMBL148052		IC_{50} = 20.83	
	CHEMBL149425		IC_{50} = 36.47	J. Med. Chem., (1988) 31:3:503
	CHEMBL15087		IC_{50} = 0.75	J. Med. Chem., (1993) 36:7:871
			IC_{50} = 7.85	J. Med. Chem., (1993) 36:14:1964

Bioactive molecule	Similar molecule	Similar molecule target	Activity value of similar molecule (μM)	Reference
	CHEMBL15186		IC_{50} = 7.51	J. Med. Chem., (1988) 31:3:503
	CHEMBL15296		IC_{50} = 1.45	J. Med. Chem., (1993) 36:7:871
	CHEMBL15511	Topoisomerase IV subunit A	IC_{50} = 10	Nucleic Acids Res., (2017) 45:D1:D945
	CHEMBL15549	DNA gyrase	IC_{50} = 8.02	J. Med. Chem., (1988) 31:3:503
				J. Med. Chem., (1991) 34:2:656
	CHEMBL2110220		IC_{50} = 1.39	J. Med. Chem., (1993) 36:7:871
			IC_{50} = 20.87	
	CHEMBL2110319			
	CHEMBL274469		IC_{50} = 7.68	J. Med. Chem., (1988) 31:3:503
				J. Med. Chem., (1991) 34:2:656
	CHEMBL275698		IC_{50} = 11.5	J. Med. Chem., (1993) 36:14:1964
	CHEMBL278255		IC_{50} = 0.55	J. Med. Chem., (2003) 46:17:3655
			IC_{50} = 3.14	J. Med. Chem., (2008) 51:11:3238
		Topoisomerase IV	IC_{50} = 5.17	
		Topoisomerase IV subunit A	IC_{50} = 10	Nucleic Acids Res., (2017) 45:D1:D945
	CHEMBL295433			
	CHEMBL31	DNA gyrase	IC_{50} = 0.5	Bioorg. Med. Chem. Lett., (2006) 16:5:1272
				J. Med. Chem., (2006) 49:22:6435
			IC_{50} = 8.58	Bioorg. Med. Chem., (2009) 17:19:6879
		DNA gyrase subunit A	IC_{50} = 9.4	Antimicrob. Agents Chemother., (2010) 54:8:3478
		Topoisomerase IV subunit A	IC_{50} = 1.2	Bioorg. Med. Chem. Lett., (2006) 16:5:1272
			IC_{50} = 10	Nucleic Acids Res., (2017) 45:D1:D945
	CHEMBL330779	DNA gyrase	IC_{50} < 1.38	J. Med. Chem., (1991) 34:2:656
	CHEMBL331076		IC_{50} = 8.81	J. Med. Chem., (2003) 46:17:3655
	CHEMBL331987		IC_{50} = 7.93	
	CHEMBL3350260		IC_{50} = 2.09	J. Med. Chem., (1993) 36:7:871
	CHEMBL342273		IC_{50} = 83.48	J. Med. Chem., (1996) 39:25:4952
	CHEMBL343603		IC_{50} = 16.42	
	CHEMBL344447		IC_{50} = 9.37	
	CHEMBL357882		IC_{50} = 97.17	
	CHEMBL416273		IC_{50} = 10.66	
	CHEMBL420897		IC_{50} = 17.91	J. Med. Chem., (2003) 46:17:3655
	CHEMBL421101		IC_{50} = 8.3	
	CHEMBL421277		IC_{50} = 2.32	J. Med. Chem., (1991) 34:2:656
	CHEMBL42299		IC_{50} > 492.09	J. Med. Chem., (1988) 31:3:503
	CHEMBL429961		IC_{50} = 4.34	J. Med. Chem., (1996) 39:25:4952
	CHEMBL47028		IC_{50} = 7.4	J. Med. Chem., (1988) 31:3:503
	CHEMBL583	Topoisomerase IV subunit A	IC_{50} = 10	Nucleic Acids Res., (2017) 45:D1:D945
	CHEMBL8	DNA gyrase	IC_{50} = 0.2	J. Med. Chem., (2006) 49:22:6435
			IC_{50} = 0.3	Bioorg. Med. Chem. Lett., (2006) 16:5:1272
			IC_{50} = 0.45	Antimicrob. Agents Chemother., (2010) 54:7:3011
			IC_{50} = 0.97	Bioorg. Med. Chem. Lett., (2012) 22:7:2428

Bioactive molecule	Similar molecule	Similar molecule target	Activity value of similar molecule (µM)	Reference
			IC_{50} = 61.7	Antimicrob. Agents Chemother., (2007) 51:7:2445
			IC_{50} = 62	J. Med. Chem., (2007) 50:2:199
		Topoisomerase IV	IC_{50} = 8	J. Med. Chem., (2013) 56:18:7396
		Topoisomerase IV subunit A	IC_{50} = 10	Nucleic Acids Res., (2017) 45:D1:D945
	CHEMBL850	DNA gyrase	IC_{50} = 0.64	J. Med. Chem., (2004) 47:14:3693
		Topoisomerase IV subunit A	IC_{50} = 10	Nucleic Acids Res., (2017) 45:D1:D945
MF 5103	CHEMBL1201760	Topoisomerase IV subunit A	IC_{50} = 10	Nucleic Acids Res., (2017) 45:D1:D945
	CHEMBL1202	DNA gyrase	IC_{50} = 0.6	J. Med. Chem., (1992) 35:1:94
			IC_{50} = 0.72	J. Med. Chem., (2011) 54:6:1539
	CHEMBL120948		IC_{50} = 4.1	J. Med. Chem., (2003) 46:17:3655
	CHEMBL1210954	Topoisomerase IV subunit A	IC_{50} = 10	Nucleic Acids Res., (2017) 45:D1:D945
	CHEMBL1213456			
	CHEMBL122691	DNA gyrase	IC_{50} = 8.84	J. Med. Chem., (2003) 46:17:3655
	CHEMBL122698		IC_{50} = 0.51	
	CHEMBL123246		IC_{50} = 4.26	
	CHEMBL124970		IC_{50} = 9.24	J. Med. Chem., (1996) 39:25:4952
	CHEMBL1257096	Topoisomerase IV subunit A	IC_{50} = 10	Nucleic Acids Res., (2017) 45:D1:D945
	CHEMBL1276635	DNA gyrase	IC_{50} = 1.1	Antimicrob. Agents Chemother., (2010) 54:7:3011
	CHEMBL136779		IC_{50} = 6.97	J. Med. Chem., (1996) 39:25:4952
	CHEMBL141308		IC_{50} = 7.64	
	CHEMBL148033		IC_{50} = 28.54	
	CHEMBL149425		IC_{50} = 36.47	J. Med. Chem., (1988) 31:3:503
	CHEMBL15087		IC_{50} = 0.75	J. Med. Chem., (1993) 36:7:871
			IC_{50} = 7.85	J. Med. Chem., (1993) 36:14:1964
	CHEMBL15186		IC_{50} = 7.51	J. Med. Chem., (1988) 31:3:503
	CHEMBL15247		IC_{50} = 6.64	
	CHEMBL15296		IC_{50} = 1.45	J. Med. Chem., (1993) 36:7:871
	CHEMBL15391		IC_{50} = 7.66	J. Med. Chem., (1988) 31:3:503
	CHEMBL15511	Topoisomerase IV subunit A	IC_{50} = 10	Nucleic Acids Res., (2017) 45:D1:D945
	CHEMBL15549	DNA gyrase	IC_{50} = 8.02	J. Med. Chem., (1988) 31:3:503
				J. Med. Chem., (1991) 34:2:656
	CHEMBL157548	Topoisomerase IV subunit A	IC_{50} = 10	Nucleic Acids Res., (2017) 45:D1:D945
	CHEMBL159024	DNA gyrase	IC_{50} = 16.78	J. Med. Chem., (1991) 34:2:656
	CHEMBL159188		IC_{50} = 36.67	
	CHEMBL160192		IC_{50} = 14.09	
	CHEMBL160661		IC_{50} = 7.75	
	CHEMBL160662		IC_{50} = 8.15	
	CHEMBL160838		IC_{50} = 4.2	
	CHEMBL184167	Topoisomerase IV subunit A	IC_{50} = 0.73	Bioorg. Med. Chem. Lett., (2004) 14:20:5193
	CHEMBL186870		IC_{50} = 0.41	
	CHEMBL187796		IC_{50} = 0.72	

Bioactive molecule	Similar molecule	Similar molecule target	Activity value of similar molecule (μM)	Reference
	CHEMBL192226	DNA gyrase	IC_{50} = 196.55	J. Med. Chem., (2005) 48:9:3194
	CHEMBL2110220		IC_{50} = 1.39	J. Med. Chem., (1993) 36:7:871
			IC_{50} = 20.87	
	CHEMBL2110319			
	CHEMBL230599	Topoisomerase IV subunit A	IC_{50} = 10	Nucleic Acids Res., (2017) 45:D1:D945
	CHEMBL258788	DNA gyrase subunit B	IC_{50} > 108.81	Bioorg. Med. Chem., (2008) 16:5:2558
	CHEMBL267648	DNA gyrase	IC_{50} = 16.5	J. Med. Chem., (1986) 29:3:394
	CHEMBL274469		IC_{50} = 7.68	J. Med. Chem., (1988) 31:3:503
				J. Med. Chem., (1991) 34:2:656
	CHEMBL275174		IC_{50} = 7.65	J. Med. Chem., (1988) 31:3:503
	CHEMBL275698		IC_{50} = 11.5	J. Med. Chem., (1993) 36:14:1964
	CHEMBL278255		IC_{50} = 0.55	J. Med. Chem., (2003) 46:17:3655
			IC_{50} = 3.14	J. Med. Chem., (2008) 51:11:3238
		Topoisomerase IV	IC_{50} = 5.17	
	CHEMBL278255	Topoisomerase IV subunit A	IC_{50} = 10	Nucleic Acids Res., (2017) 45:D1:D945
	CHEMBL295433			
	CHEMBL31	DNA gyrase	IC_{50} = 0.5	Bioorg. Med. Chem. Lett., (2006) 16:5:1272
				J. Med. Chem., (2006) 49:22:6435
			IC_{50} = 8.58	Bioorg. Med. Chem., (2009) 17:19:6879
		DNA gyrase subunit A	IC_{50} = 9.4	Antimicrob. Agents Chemother., (2010) 54:8:3478
		Topoisomerase IV subunit A	IC_{50} = 1.2	Bioorg. Med. Chem. Lett., (2006) 16:5:1272
			IC_{50} = 10	Nucleic Acids Res., (2017) 45:D1:D945
	CHEMBL32	DNA gyrase	IC_{50} = 0.3	Bioorg. Med. Chem. Lett., (2006) 16:5:1272
			IC_{50} = 10.91	Bioorg. Med. Chem., (2009) 17:19:6879
			IC_{50} = 18	J. Med. Chem., (2011) 54:9:3418
			IC_{50} = 27.5	Antimicrob. Agents Chemothe., (2007) 51:7:2445
			IC_{50} = 28	J. Med. Chem., (2007) 50:2:199
			IC_{50} = 7.7	J. Med. Chem., (2011) 54:9:3268
		DNA gyrase subunit A	IC_{50} = 9.2	Antimicrob. Agents Chemother., (2010) 54:8:3478
		DNA gyrase subunit A/subunit B	IC_{50} = 2.5	Antimicrob. Agents Chemothe., (2008) 52:8:2909
		DNA gyrase subunit B	IC_{50} > 50	Bioorg. Med. Chem., (2014) 22:17:4924
				Bioorg. Med. Chem., (2015) 23:9:2062
				Eur. J. Med. Chem., (2015) 103:null:1
		Topoisomerase IV subunit A	IC_{50} = 0.8	Bioorg. Med. Chem. Lett., (2006) 16:5:1272
			IC_{50} = 10	Nucleic Acids Res., (2017) 45:D1:D945
	CHEMBL330779	DNA gyrase	IC_{50} < 1.38	J. Med. Chem., (1991) 34:2:656
	CHEMBL331076		IC_{50} = 8.81	J. Med. Chem., (2003) 46:17:3655
	CHEMBL331987		IC_{50} = 7.93	
	CHEMBL3350260		IC_{50} = 2.09	J. Med. Chem., (1993) 36:7:871

Bioactive molecule	Similar molecule	Similar molecule target	Activity value of similar molecule (μM)	Reference
	CHEMBL342273		IC_{50} = 83.48	J. Med. Chem., (1996) 39:25:4952
	CHEMBL343603		IC_{50} = 16.42	
	CHEMBL344447		IC_{50} = 9.37	
	CHEMBL352000		IC_{50} = 16.18	J. Med. Chem., (1991) 34:2:656
	CHEMBL357882		IC_{50} = 97.17	J. Med. Chem., (1996) 39:25:4952
	CHEMBL359193		IC_{50} = 32.82	J. Med. Chem., (1988) 31:3:503
	CHEMBL371124		IC_{50} = 1807.64	J. Med. Chem., (2005) 48:9:3194
	CHEMBL416273		IC_{50} = 10.66	J. Med. Chem., (1996) 39:25:4952
	CHEMBL420897		IC_{50} = 17.91	J. Med. Chem., (2003) 46:17:3655
	CHEMBL421101		IC_{50} = 8.3	
	CHEMBL421277		IC_{50} = 2.32	J. Med. Chem., (1991) 34:2:656
	CHEMBL422131		IC_{50} > 246.07	J. Med. Chem., (1988) 31:3:503
	CHEMBL42299		IC_{50} > 492.09	
	CHEMBL429961		IC_{50} = 4.34	J. Med. Chem., (1996) 39:25:4952
	CHEMBL47028		IC_{50} = 7.4	J. Med. Chem., (1988) 31:3:503
	CHEMBL540589		IC_{50} = 5.53	J. Med. Chem., (1991) 34:2:656
	CHEMBL544407		IC_{50} = 7.99	
	CHEMBL583	Topoisomerase IV subunit A	IC_{50} = 10	Nucleic Acids Res., (2017) 45:D1:D945
	CHEMBL6210	DNA gyrase	IC_{50} = 3.58	J. Med. Chem., (1988) 31:3:503
	CHEMBL6273		IC_{50} = 10.29	J. Med. Chem., (1986) 29:3:394
			IC_{50} = 185.47	J. Med. Chem., (2005) 48:9:3194
	CHEMBL8		IC_{50} = 0.2	J. Med. Chem., (2006) 49:22:6435
			IC_{50} = 0.3	Bioorg. Med. Chem. Lett., (2006) 16:5:1272
			IC_{50} = 0.45	Antimicrob. Agents Chemother., (2010) 54:7:3011
			IC_{50} = 0.97	Bioorg. Med. Chem. Lett., (2012) 22:7:2428
			IC_{50} = 61.7	Antimicrob. Agents Chemother., (2007) 51:7:2445
			IC_{50} = 62	J. Med. Chem., (2007) 50:2:199
		Topoisomerase IV	IC_{50} = 8	J. Med. Chem., (2013) 56:18:7396
		Topoisomerase IV subunit A	IC_{50} = 10	Nucleic Acids Res., (2017) 45:D1:D945
	CHEMBL850	DNA gyrase	IC_{50} = 0.64	J. Med. Chem., (2004) 47:14:3693
		Topoisomerase IV subunit A	IC_{50} = 10	Nucleic Acids Res., (2017) 45:D1:D945
Nebramycin IV	CHEMBL2206187	30S ribosomal subunit	IC_{50} = 0.2	J. Med. Chem., (2012) 55:23:10630
	CHEMBL2206192		IC_{50} = 2.3	
	CHEMBL2206193		IC_{50} = 0.8	
	CHEMBL2206196		IC_{50} = 0.05	
	CHEMBL373500		IC_{50} = 0.5	
Nebramycin V'	CHEMBL2206187	30S ribosomal subunit	IC_{50} = 0.2	J. Med. Chem., (2012) 55:23:10630
	CHEMBL2206196		IC_{50} = 0.05	
	CHEMBL373500		IC_{50} = 0.5	
Nebramycin XI	CHEMBL2206187	30S ribosomal subunit	IC_{50} = 0.2	J. Med. Chem., (2012) 55:23:10630
Nebramycin XII	CHEMBL2206187	30S ribosomal subunit	IC_{50} = 0.2	J. Med. Chem., (2012) 55:23:10630
	CHEMBL2206188		IC_{50} = 1.1	

Bioactive molecule	Similar molecule	Similar molecule target	Activity value of similar molecule (μM)	Reference
	CHEMBL2206189		IC_{50} = 1	
	CHEMBL2206190		IC_{50} = 2	
	CHEMBL2206191		IC_{50} = 2.1	
	CHEMBL2206192		IC_{50} = 2.3	
	CHEMBL2206193		IC_{50} = 0.8	
	CHEMBL2206194		IC_{50} = 1.1	
	CHEMBL2206195		IC_{50} = 1.9	
	CHEMBL2206196		IC_{50} = 0.05	
	CHEMBL373500		IC_{50} = 0.5	
	CHEMBL431061		IC_{50} = 3.9	Bioorg. Med. Chem. Lett., (2002) 12:23:3367
Nebramycin XIII	CHEMBL2206187	30S ribosomal subunit	IC_{50} = 0.2	J. Med. Chem., (2012) 55:23:10630
	CHEMBL2206196		IC_{50} = 0.05	
PD 114111	CHEMBL267648	DNA gyrase	IC_{50} = 16.5	J. Med. Chem., (1986) 29:3:394
	CHEMBL277523		IC_{50} = 18.68	
	CHEMBL6273		IC_{50} = 10.29	
			IC_{50} = 185.47	J. Med. Chem., (2005) 48:9:3194
	CHEMBL9		IC_{50} = 0.6	J. Med. Chem., (2006) 49:1:39
			IC_{50} = 17.22	J. Med. Chem., (1986) 29:3:394
		Topoisomerase IV subunit A	IC_{50} = 10	Nucleic Acids Res., (2017) 45:D1:D945
			IC_{50} = 3.5	J. Med. Chem., (2006) 49:1:39
PD 116507	CHEMBL2105637	Topoisomerase IV subunit A	IC_{50} = 10	Nucleic Acids Res., (2017) 45:D1:D945
	CHEMBL3633518	DNA gyrase	IC_{50} = 0.29	Eur. J. Med. Chem., (2015) 103:null:354
	CHEMBL3633519		IC_{50} = 0.55	
	CHEMBL3633520		IC_{50} = 0.17	
	CHEMBL3633521		IC_{50} = 0.41	
	CHEMBL3633522		IC_{50} = 0.43	
	CHEMBL3633523		IC_{50} = 0.7	
PD 119344	CHEMBL120948	DNA gyrase	IC_{50} = 4.1	J. Med. Chem., (2003) 46:17:3655
	CHEMBL1210954	Topoisomerase IV subunit A	IC_{50} = 10	Nucleic Acids Res., (2017) 45:D1:D945
	CHEMBL122691	DNA gyrase	IC_{50} = 8.84	J. Med. Chem., (2003) 46:17:3655
	CHEMBL122698		IC_{50} = 0.51	
	CHEMBL123246		IC_{50} = 4.26	
	CHEMBL1276635		IC_{50} = 1.1	Antimicrob. Agents Chemother., (2010) 54:7:3011
	CHEMBL15087		IC_{50} = 0.75	J. Med. Chem., (1993) 36:7:871
			IC_{50} = 7.85	J. Med. Chem., (1993) 36:14:1964
	CHEMBL15186		IC_{50} = 7.51	J. Med. Chem., (1988) 31:3:503
	CHEMBL15247		IC_{50} = 6.64	
	CHEMBL15296		IC_{50} = 1.45	J. Med. Chem., (1993) 36:7:871
	CHEMBL15391		IC_{50} = 7.66	J. Med. Chem., (1988) 31:3:503
	CHEMBL15511	Topoisomerase IV subunit A	IC_{50} = 10	Nucleic Acids Res., (2017) 45:D1:D945
	CHEMBL15549	DNA gyrase	IC_{50} = 8.02	J. Med. Chem., (1988) 31:3:503
				J. Med. Chem., (1991) 34:2:656

Bioactive molecule	Similar molecule	Similar molecule target	Activity value of similar molecule (μM)	Reference
	CHEMBL184167	Topoisomerase IV subunit A	IC_{50} = 0.73	Bioorg. Med. Chem. Lett., (2004) 14:20:5193
	CHEMBL186870		IC_{50} = 0.41	
	CHEMBL2110220	DNA gyrase	IC_{50} = 1.39	J. Med. Chem., (1993) 36:7:871
			IC_{50} = 20.87	
	CHEMBL2110319			
	CHEMBL230599	Topoisomerase IV subunit A	IC_{50} = 10	Nucleic Acids Res., (2017) 45:D1:D945
	CHEMBL275174	DNA gyrase	IC_{50} = 7.65	J. Med. Chem., (1988) 31:3:503
	CHEMBL278255		IC_{50} = 0.55	J. Med. Chem., (2003) 46:17:3655
			IC_{50} = 3.14	J. Med. Chem., (2008) 51:11:3238
		Topoisomerase IV	IC_{50} = 5.17	
		Topoisomerase IV subunit A	IC_{50} = 10	Nucleic Acids Res., (2017) 45:D1:D945
	CHEMBL282758	DNA gyrase	IC_{50} = 85.44	J. Med. Chem., (1986) 29:3:394
	CHEMBL330779		IC_{50} < 1.38	J. Med. Chem., (1991) 34:2:656
	CHEMBL331076		IC_{50} = 8.81	J. Med. Chem., (2003) 46:17:3655
	CHEMBL331987		IC_{50} = 7.93	
	CHEMBL3350260		IC_{50} = 2.09	J. Med. Chem., (1993) 36:7:871
	CHEMBL356984		IC_{50} > 222.98	J. Med. Chem., (1988) 31:3:503
	CHEMBL357882		IC_{50} = 97.17	J. Med. Chem., (1996) 39:25:4952
	CHEMBL359193		IC_{50} = 32.82	J. Med. Chem., (1988) 31:3:503
	CHEMBL421277		IC_{50} = 2.32	J. Med. Chem., (1991) 34:2:656
	CHEMBL6210		IC_{50} = 3.58	J. Med. Chem., (1988) 31:3:503
PD 129626	CHEMBL6210	DNA gyrase	IC_{50} = 3.58	J. Med. Chem., (1988) 31:3:503
PD 129627	CHEMBL410627	DNA gyrase subunit B	IC_{50} = 114.83	Bioorg. Med. Chem., (2008) 16:5:2558
PD 137156	CHEMBL1202	DNA gyrase	IC_{50} = 0.6	J. Med. Chem., (1992) 35:1:94
			IC_{50} = 0.72	J. Med. Chem., (2011) 54:6:1539
	CHEMBL120948		IC_{50} = 4.1	J. Med. Chem., (2003) 46:17:3655
	CHEMBL1210954	Topoisomerase IV subunit A	IC_{50} = 10	Nucleic Acids Res., (2017) 45:D1:D945
	CHEMBL1213456			
	CHEMBL122691	DNA gyrase	IC_{50} = 8.84	J. Med. Chem., (2003) 46:17:3655
	CHEMBL122698		IC_{50} = 0.51	
	CHEMBL123246		IC_{50} = 4.26	
	CHEMBL1276635		IC_{50} = 1.1	Antimicrob. Agents Chemother., (2010) 54:7:3011
	CHEMBL136779		IC_{50} = 6.97	J. Med. Chem., (1996) 39:25:4952
	CHEMBL141308		IC_{50} = 7.64	
	CHEMBL148033		IC_{50} = 28.54	
	CHEMBL149425		IC_{50} = 36.47	J. Med. Chem., (1988) 31:3:503
	CHEMBL15087		IC_{50} = 0.75	J. Med. Chem., (1993) 36:7:871
			IC_{50} = 7.85	J. Med. Chem., (1993) 36:14:1964
	CHEMBL15186		IC_{50} = 7.51	J. Med. Chem., (1988) 31:3:503
	CHEMBL15247		IC_{50} = 6.64	
	CHEMBL15296		IC_{50} = 1.45	J. Med. Chem., (1993) 36:7:871
	CHEMBL15391		IC_{50} = 7.66	J. Med. Chem., (1988) 31:3:503
	CHEMBL15511	Topoisomerase IV subunit A	IC_{50} = 10	Nucleic Acids Res., (2017) 45:D1:D945

Bioactive molecule	Similar molecule	Similar molecule target	Activity value of similar molecule (μM)	Reference
	CHEMBL15549	DNA gyrase	IC_{50} = 8.02	J. Med. Chem., (1988) 31:3:503
				J. Med. Chem., (1991) 34:2:656
	CHEMBL157548	Topoisomerase IV subunit A	IC_{50} = 10	Nucleic Acids Res., (2017) 45:D1:D945
	CHEMBL159024	DNA gyrase	IC_{50} = 16.78	J. Med. Chem., (1991) 34:2:656
	CHEMBL159030		IC_{50} = 4.85	
	CHEMBL159188		IC_{50} = 36.67	
	CHEMBL160192		IC_{50} = 14.09	
	CHEMBL160661		IC_{50} = 7.75	
	CHEMBL160662		IC_{50} = 8.15	
	CHEMBL160838		IC_{50} = 4.2	
	CHEMBL2110220		IC_{50} = 1.39	J. Med. Chem., (1993) 36:7:871
			IC_{50} = 20.87	
	CHEMBL2110319			
	CHEMBL274469		IC_{50} = 7.68	J. Med. Chem., (1988) 31:3:503
				J. Med. Chem., (1991) 34:2:656
	CHEMBL275174		IC_{50} = 7.65	J. Med. Chem., (1988) 31:3:503
	CHEMBL275698		IC_{50} = 11.5	J. Med. Chem., (1993) 36:14:1964
	CHEMBL277100	Topoisomerase IV subunit A	IC_{50} = 10	Nucleic Acids Res., (2017) 45:D1:D945
	CHEMBL278255	DNA gyrase	IC_{50} = 0.55	J. Med. Chem., (2003) 46:17:3655
			IC_{50} = 3.14	J. Med. Chem., (2008) 51:11:3238
		Topoisomerase IV	IC_{50} = 5.17	
		Topoisomerase IV subunit A	IC_{50} = 10	Nucleic Acids Res., (2017) 45:D1:D945
	CHEMBL295433			
	CHEMBL31	DNA gyrase	IC_{50} = 0.5	Bioorg. Med. Chem. Lett., (2006) 16:5:1272
				J. Med. Chem., (2006) 49:22:6435
			IC_{50} = 8.58	Bioorg. Med. Chem., (2009) 17:19:6879
		DNA gyrase subunit A	IC_{50} = 9.4	Antimicrob. Agents Chemother., (2010) 54:8:3478
		Topoisomerase IV subunit A	IC_{50} = 1.2	Bioorg. Med. Chem. Lett., (2006) 16:5:1272
			IC_{50} = 10	Nucleic Acids Res., (2017) 45:D1:D945
	CHEMBL32	DNA gyrase	IC_{50} = 0.3	Bioorg. Med. Chem. Lett., (2006) 16:5:1272
			IC_{50} = 10.91	Bioorg. Med. Chem., (2009) 17:19:6879
			IC_{50} = 18	J. Med. Chem., (2011) 54:9:3418
			IC_{50} = 27.5	Antimicrob. Agents Chemothe., (2007) 51:7:2445
			IC_{50} = 28	J. Med. Chem., (2007) 50:2:199
			IC_{50} = 7.7	J. Med. Chem., (2011) 54:9:3268
		DNA gyrase subunit A	IC_{50} = 9.2	Antimicrob. Agents Chemother., (2010) 54:8:3478
		DNA gyrase subunit A/subunit B	IC_{50} = 2.5	Antimicrob. Agents Chemothe., (2008) 52:8:2909
		DNA gyrase subunit B	IC_{50} > 50	Bioorg. Med. Chem., (2014) 22:17:4924
				Bioorg. Med. Chem., (2015) 23:9:2062
				Eur. J. Med. Chem., (2015) 103:null:1
		Topoisomerase IV subunit A	IC_{50} = 0.8	Bioorg. Med. Chem. Lett., (2006) 16:5:1272

Bioactive molecule	Similar molecule	Similar molecule target	Activity value of similar molecule (μM)	Reference
			IC_{50} = 10	Nucleic Acids Res., (2017) 45:D1:D945
	CHEMBL330779	DNA gyrase	IC_{50} < 1.38	J. Med. Chem., (1991) 34:2:656
	CHEMBL331076		IC_{50} = 8.81	J. Med. Chem., (2003) 46:17:3655
	CHEMBL331987		IC_{50} = 7.93	
	CHEMBL3350260		IC_{50} = 2.09	J. Med. Chem., (1993) 36:7:871
	CHEMBL342273		IC_{50} = 83.48	J. Med. Chem., (1996) 39:25:4952
	CHEMBL343603		IC_{50} = 16.42	
	CHEMBL344447		IC_{50} = 9.37	
	CHEMBL348879		IC_{50} = 7.45	J. Med. Chem., (1991) 34:2:656
	CHEMBL352000		IC_{50} = 16.18	
	CHEMBL357882		IC_{50} = 97.17	J. Med. Chem., (1996) 39:25:4952
	CHEMBL359193		IC_{50} = 32.82	J. Med. Chem., (1988) 31:3:503
	CHEMBL416273		IC_{50} = 10.66	J. Med. Chem., (1996) 39:25:4952
	CHEMBL420897		IC_{50} = 17.91	J. Med. Chem., (2003) 46:17:3655
	CHEMBL421101		IC_{50} = 8.3	
	CHEMBL421277		IC_{50} = 2.32	J. Med. Chem., (1991) 34:2:656
	CHEMBL422131		IC_{50} > 246.07	J. Med. Chem., (1988) 31:3:503
	CHEMBL42299		IC_{50} > 492.09	
	CHEMBL429961		IC_{50} = 4.34	J. Med. Chem., (1996) 39:25:4952
	CHEMBL47028		IC_{50} = 7.4	J. Med. Chem., (1988) 31:3:503
	CHEMBL540589		IC_{50} = 5.53	J. Med. Chem., (1991) 34:2:656
	CHEMBL544407		IC_{50} = 7.99	
	CHEMBL561	Topoisomerase IV subunit A	IC_{50} = 10	Nucleic Acids Res., (2017) 45:D1:D945
	CHEMBL583			
	CHEMBL6210	DNA gyrase	IC_{50} = 3.58	J. Med. Chem., (1988) 31:3:503
	CHEMBL8		IC_{50} = 0.2	J. Med. Chem., (2006) 49:22:6435
			IC_{50} = 0.3	Bioorg. Med. Chem. Lett., (2006) 16:5:1272
			IC_{50} = 0.45	Antimicrob. Agents Chemother., (2010) 54:7:3011
			IC_{50} = 0.97	Bioorg. Med. Chem. Lett., (2012) 22:7:2428
			IC_{50} = 61.7	Antimicrob. Agents Chemother., (2007) 51:7:2445
			IC_{50} = 62	J. Med. Chem., (2007) 50:2:199
		Topoisomerase IV	IC_{50} = 8	J. Med. Chem., (2013) 56:18:7396
		Topoisomerase IV subunit A	IC_{50} = 10	Nucleic Acids Res., (2017) 45:D1:D945
	CHEMBL850	DNA gyrase	IC_{50} = 0.64	J. Med. Chem., (2004) 47:14:3693
		Topoisomerase IV subunit A	IC_{50} = 10	Nucleic Acids Res., (2017) 45:D1:D945
Piroxacin	CHEMBL68262	DNA gyrase	IC_{50} = 93.24	J. Med. Chem., (1986) 29:3:394
		Topoisomerase IV subunit A	IC_{50} = 10	Nucleic Acids Res., (2017) 45:D1:D945
QA 241 free base	CHEMBL146339	DNA gyrase	IC_{50} = 69.08	J. Med. Chem., (1988) 31:3:503
	CHEMBL187677		IC_{50} = 13.87	J. Med. Chem., (2005) 48:16:5232
		Topoisomerase IV subunit A		

Bioactive molecule	Similar molecule	Similar molecule target	Activity value of similar molecule (µM)	Reference
	CHEMBL33	DNA gyrase	IC_{50} = 0.47	Eur. J. Med. Chem., (2015) 103:null:354
			IC_{50} = 2136.31	J. Med. Chem., (2008) 51:11:3238
			IC_{50} = 25.76	
			IC_{50} = 26.04	Bioorg. Med. Chem., (2009) 17:19:6879
		DNA gyrase subunit A	IC_{50} = 52	Antimicrob. Agents Chemother., (2010) 54:8:3478
	CHEMBL346948	Topoisomerase IV subunit A	IC_{50} = 10	Nucleic Acids Res., (2017) 45:D1:D945
	CHEMBL363449	DNA gyrase	IC_{50} = 27.75	J. Med. Chem., (2005) 48:16:5232
		Topoisomerase IV subunit A	IC_{50} > 27.75	
	CHEMBL4	DNA gyrase	IC_{50} = 17.43	J. Med. Chem., (1986) 29:3:394
			IC_{50} = 20.75	J. Med. Chem., (1988) 31:3:503
			IC_{50} = 4.98	Bioorg. Med. Chem. Lett., (1998) 8:1:97
		DNA gyrase subunit A/subunit B	IC_{50} = 1.5	Antimicrob. Agents Chemother., (2008) 52:8:2909
R 31000	CHEMBL295698	Cytochrome P450 51	IC_{50} = 0.047	J. Med. Chem., (1992) 35:15:2818
			IC_{50} = 0.119	
			IC_{50} = 0.19	Drug Metab. Dispos., (2007) 35:3:493
			Ki = 0.0245	J. Med. Chem., (1993) 36:15:2235
			Ki = 0.0635	
			Ki = 0.065	
	CHEMBL319160		IC_{50} = 1.6	J. Med. Chem., (1992) 35:15:2818
	CHEMBL328863		IC_{50} = 1.37	
S 31076	CHEMBL1202	DNA gyrase	IC_{50} = 0.6	J. Med. Chem., (1992) 35:1:94
			IC_{50} = 0.72	J. Med. Chem., (2011) 54:6:1539
	CHEMBL120948		IC_{50} = 4.1	J. Med. Chem., (2003) 46:17:3655
	CHEMBL1210954	Topoisomerase IV subunit A	IC_{50} = 10	Nucleic Acids Res., (2017) 45:D1:D945
	CHEMBL1213456			
	CHEMBL122691	DNA gyrase	IC_{50} = 8.84	J. Med. Chem., (2003) 46:17:3655
	CHEMBL122698		IC_{50} = 0.51	
	CHEMBL123246		IC_{50} = 4.26	
	CHEMBL1257096	Topoisomerase IV subunit A	IC_{50} = 10	Nucleic Acids Res., (2017) 45:D1:D945
	CHEMBL1276635	DNA gyrase	IC_{50} = 1.1	Antimicrob. Agents Chemother., (2010) 54:7:3011
	CHEMBL136779		IC_{50} = 6.97	J. Med. Chem., (1996) 39:25:4952
	CHEMBL141308		IC_{50} = 7.64	
	CHEMBL148033		IC_{50} = 28.54	
	CHEMBL149425		IC_{50} = 36.47	J. Med. Chem., (1988) 31:3:503
	CHEMBL15296		IC_{50} = 1.45	J. Med. Chem., (1993) 36:7:871
	CHEMBL15511	Topoisomerase IV subunit A	IC_{50} = 10	Nucleic Acids Res., (2017) 45:D1:D945
	CHEMBL15549	DNA gyrase	IC_{50} = 8.02	J. Med. Chem., (1988) 31:3:503
				J. Med. Chem., (1991) 34:2:656
	CHEMBL2110220		IC_{50} = 1.39	J. Med. Chem., (1993) 36:7:871
			IC_{50} = 20.87	
	CHEMBL2110319			
	CHEMBL230599	Topoisomerase IV subunit A	IC_{50} = 10	Nucleic Acids Res., (2017) 45:D1:D945

Bioactive molecule	Similar molecule	Similar molecule target	Activity value of similar molecule (µM)	Reference
	CHEMBL258788	DNA gyrase subunit B	IC_{50} > 108.81	Bioorg. Med. Chem., (2008) 16:5:2558
	CHEMBL274469	DNA gyrase	IC_{50} = 7.68	J. Med. Chem., (1988) 31:3:503
				J. Med. Chem., (1991) 34:2:656
	CHEMBL278255		IC_{50} = 0.55	J. Med. Chem., (2003) 46:17:3655
			IC_{50} = 3.14	J. Med. Chem., (2008) 51:11:3238
		Topoisomerase IV	IC_{50} = 5.17	
		Topoisomerase IV subunit A	IC_{50} = 10	Nucleic Acids Res., (2017) 45:D1:D945
	CHEMBL31	DNA gyrase	IC_{50} = 0.5	Bioorg. Med. Chem. Lett., (2006) 16:5:1272
				J. Med. Chem., (2006) 49:22:6435
			IC_{50} = 8.58	Bioorg. Med. Chem., (2009) 17:19:6879
		DNA gyrase subunit A	IC_{50} = 9.4	Antimicrob. Agents Chemother., (2010) 54:8:3478
		Topoisomerase IV subunit A	IC_{50} = 1.2	Bioorg. Med. Chem. Lett., (2006) 16:5:1272
			IC_{50} = 10	Nucleic Acids Res., (2017) 45:D1:D945
	CHEMBL32	DNA gyrase	IC_{50} = 0.3	Bioorg. Med. Chem. Lett., (2006) 16:5:1272
			IC_{50} = 10.91	Bioorg. Med. Chem., (2009) 17:19:6879
			IC_{50} = 18	J. Med. Chem., (2011) 54:9:3418
			IC_{50} = 27.5	Antimicrob. Agents Chemothe., (2007) 51:7:2445
			IC_{50} = 28	J. Med. Chem., (2007) 50:2:199
			IC_{50} = 7.7	J. Med. Chem., (2011) 54:9:3268
		DNA gyrase subunit A	IC_{50} = 9.2	Antimicrob. Agents Chemother., (2010) 54:8:3478
		DNA gyrase subunit A/subunit B	IC_{50} = 2.5	Antimicrob. Agents Chemothe., (2008) 52:8:2909
		DNA gyrase subunit B	IC_{50} > 50	Bioorg. Med. Chem., (2014) 22:17:4924
				Bioorg. Med. Chem., (2015) 23:9:2062
				Eur. J. Med. Chem., (2015) 103:null:1
		Topoisomerase IV subunit A	IC_{50} = 0.8	Bioorg. Med. Chem. Lett., (2006) 16:5:1272
			IC_{50} = 10	Nucleic Acids Res., (2017) 45:D1:D945
	CHEMBL330779	DNA gyrase	IC_{50} < 1.38	J. Med. Chem., (1991) 34:2:656
	CHEMBL331076		IC_{50} = 8.81	J. Med. Chem., (2003) 46:17:3655
	CHEMBL331987		IC_{50} = 7.93	
	CHEMBL3350260		IC_{50} = 2.09	J. Med. Chem., (1993) 36:7:871
	CHEMBL343603		IC_{50} = 16.42	J. Med. Chem., (1996) 39:25:4952
	CHEMBL357882		IC_{50} = 97.17	
	CHEMBL420897		IC_{50} = 17.91	J. Med. Chem., (2003) 46:17:3655
	CHEMBL421101		IC_{50} = 8.3	
	CHEMBL421277		IC_{50} = 2.32	J. Med. Chem., (1991) 34:2:656
	CHEMBL42299		IC_{50} > 492.09	J. Med. Chem., (1988) 31:3:503
	CHEMBL429961		IC_{50} = 4.34	J. Med. Chem., (1996) 39:25:4952
	CHEMBL47028		IC_{50} = 7.4	J. Med. Chem., (1988) 31:3:503
	CHEMBL6210		IC_{50} = 3.58	
	CHEMBL8		IC_{50} = 0.2	J. Med. Chem., (2006) 49:22:6435

Bioactive molecule	Similar molecule	Similar molecule target	Activity value of similar molecule (μM)	Reference
			IC_{50} = 0.3	Bioorg. Med. Chem. Lett., (2006) 16:5:1272
			IC_{50} = 0.45	Antimicrob. Agents Chemother., (2010) 54:7:3011
			IC_{50} = 0.97	Bioorg. Med. Chem. Lett., (2012) 22:7:2428
			IC_{50} = 61.7	Antimicrob. Agents Chemother., (2007) 51:7:2445
			IC_{50} = 62	J. Med. Chem., (2007) 50:2:199
		Topoisomerase IV	IC_{50} = 8	J. Med. Chem., (2013) 56:18:7396
		Topoisomerase IV subunit A	IC_{50} = 10	Nucleic Acids Res., (2017) 45:D1:D945
Sannamycin L	CHEMBL431061	30S ribosomal subunit	IC_{50} = 3.9	Bioorg. Med. Chem. Lett., (2002) 12:23:3367
Seldomycin 1 (seldomycin factor 1)	CHEMBL2206187	30S ribosomal subunit	IC_{50} = 0.2	J. Med. Chem., (2012) 55:23:10630
	CHEMBL2206188		IC_{50} = 1.1	
	CHEMBL2206189		IC_{50} = 1	
	CHEMBL2206190		IC_{50} = 2	
	CHEMBL2206191		IC_{50} = 2.1	
	CHEMBL2206192		IC_{50} = 2.3	
	CHEMBL2206193		IC_{50} = 0.8	
	CHEMBL2206194		IC_{50} = 1.1	
	CHEMBL2206195		IC_{50} = 1.9	
	CHEMBL2206196		IC_{50} = 0.05	
	CHEMBL373500		IC_{50} = 0.5	
	CHEMBL431061		IC_{50} = 3.9	Bioorg. Med. Chem. Lett., (2002) 12:23:3367
Seldomycin 2 (seldomycin factor 2)	CHEMBL2206188	30S ribosomal subunit	IC_{50} = 1.1	J. Med. Chem., (2012) 55:23:10630
	CHEMBL2206190		IC_{50} = 2	
	CHEMBL2206191		IC_{50} = 2.1	
	CHEMBL2206196		IC_{50} = 0.05	
	CHEMBL373500		IC_{50} = 0.5	
	CHEMBL431061		IC_{50} = 3.9	Bioorg. Med. Chem. Lett., (2002) 12:23:3367
Seldomycin 3 (seldomycin factor 3)	CHEMBL2206187	30S ribosomal subunit	IC_{50} = 0.2	J. Med. Chem., (2012) 55:23:10630
	CHEMBL2206188		IC_{50} = 1.1	
	CHEMBL2206189		IC_{50} = 1	
	CHEMBL2206190		IC_{50} = 2	
	CHEMBL2206191		IC_{50} = 2.1	
	CHEMBL2206192		IC_{50} = 2.3	
	CHEMBL2206193		IC_{50} = 0.8	
	CHEMBL2206194		IC_{50} = 1.1	
	CHEMBL2206195		IC_{50} = 1.9	
	CHEMBL2206196		IC_{50} = 0.05	
	CHEMBL373500		IC_{50} = 0.5	
	CHEMBL431061		IC_{50} = 3.9	Bioorg. Med. Chem. Lett., (2002) 12:23:3367
Seldomycin 5 (seldomycin factor 5)	CHEMBL2206188	30S ribosomal subunit	IC_{50} = 1.1	J. Med. Chem., (2012) 55:23:10630
	CHEMBL2206190		IC_{50} = 2	
	CHEMBL2206191		IC_{50} = 2.1	

Bioactive molecule	Similar molecule	Similar molecule target	Activity value of similar molecule (μM)	Reference
	CHEMBL2206194	30S ribosomal subunit	IC_{50} = 1.1	
	CHEMBL2206195		IC_{50} = 1.9	
	CHEMBL2206196		IC_{50} = 0.05	
	CHEMBL373500		IC_{50} = 0.5	
	CHEMBL431061		IC_{50} = 3.9	Bioorg. Med. Chem. Lett., (2002) 12:23:3367
SS 56A	CHEMBL2206188	30S ribosomal subunit	IC_{50} = 1.1	J. Med. Chem., (2012) 55:23:10630
	CHEMBL2206190		IC_{50} = 2	
	CHEMBL2206191		IC_{50} = 2.1	
	CHEMBL2206196		IC_{50} = 0.05	
	CHEMBL373500		IC_{50} = 0.5	
	CHEMBL431061		IC_{50} = 3.9	Bioorg. Med. Chem. Lett., (2002) 12:23:3367
SS 56B	CHEMBL2206188	30S ribosomal subunit	IC_{50} = 1.1	J. Med. Chem., (2012) 55:23:10630
	CHEMBL2206190		IC_{50} = 2	
	CHEMBL2206191		IC_{50} = 2.1	
	CHEMBL2206196		IC_{50} = 0.05	
	CHEMBL373500		IC_{50} = 0.5	
	CHEMBL431061		IC_{50} = 3.9	Bioorg. Med. Chem. Lett., (2002) 12:23:3367
SS 56C	CHEMBL3138729	30S ribosomal subunit	IC_{50} = 123.22	Antimicrob. Agents Chemother., (2007) 51:2:591
Verdamicin C_2	CHEMBL2206186	30S ribosomal subunit	IC_{50} = 0.01	J. Med. Chem., (2012) 55:23:10630
Vertilmicin	CHEMBL2206186	30S ribosomal subunit	IC_{50} = 0.01	J. Med. Chem., (2012) 55:23:10630
VG 6/1	CHEMBL124970	DNA gyrase	IC_{50} = 9.24	J. Med. Chem., (1996) 39:25:4952
	CHEMBL148052		IC_{50} = 20.83	
WQ 2743	CHEMBL2105637	Topoisomerase IV subunit A	IC_{50} = 10	Nucleic Acids Res., (2017) 45:D1:D945
	CHEMBL3633518	DNA gyrase	IC_{50} = 0.29	Eur. J. Med. Chem., (2015) 103:null:354
	CHEMBL3633519		IC_{50} = 0.55	
	CHEMBL3633520		IC_{50} = 0.17	
	CHEMBL3633521		IC_{50} = 0.41	
	CHEMBL3633522		IC_{50} = 0.43	
	CHEMBL3633523		IC_{50} = 0.7	
WQ 2756	CHEMBL132468	Topoisomerase IV subunit A	IC_{50} = 10	Nucleic Acids Res., (2017) 45:D1:D945
WQ 2908	CHEMBL132468	Topoisomerase IV subunit A	IC_{50} = 10	Nucleic Acids Res., (2017) 45:D1:D945
	CHEMBL3633518	DNA gyrase	IC_{50} = 0.29	Eur. J. Med. Chem., (2015) 103:null:354
	CHEMBL3633519		IC_{50} = 0.55	
	CHEMBL3633520		IC_{50} = 0.17	
	CHEMBL3633522		IC_{50} = 0.43	
WQ 3330	CHEMBL132468	Topoisomerase IV subunit A	IC_{50} = 10	Nucleic Acids Res., (2017) 45:D1:D945
	CHEMBL3633518	DNA gyrase	IC_{50} = 0.29	Eur. J. Med. Chem., (2015) 103:null:354
	CHEMBL3633519		IC_{50} = 0.55	
	CHEMBL3633520		IC_{50} = 0.17	
	CHEMBL3633521		IC_{50} = 0.41	
	CHEMBL3633522		IC_{50} = 0.43	

Bioactive molecule	Similar molecule	Similar molecule target	Activity value of similar molecule (µM)	Reference
Zoficonazole	CHEMBL808	Cytochrome P450 51	IC_{50} = 0.05	Drug Metab. Dispos., (2007) 35:3:493

Table S5. Primary targets predicted by the computational target prediction tools for bioactive molecules of the present study

Bioactive molecule	PASS online[4]			Similarity Ensemble Approach (SEA)[2]				ChemProt[3]		SuperPred[6]		Polypharmacology Browser (PPB)[1]			SPiDER[7]		HitPick[5]			PharmMapper[9]			TargetHunter[8]	
	Activity	Pa	Pi	Organism	Target	P-Value	Max TC	Organism	Target	Organism	Target	Organism	Target	P-Value	Target	Confidence level	Target	Precision (%)	Tc similarity	Organism	Target	Job Id	Target	Reference
A 57132	DNA gyrase inhibitor	0.246	0.002	*Escherichia coli*	DNA gyrase subunit A	7.45E-25	0.46	*Bacillus subtilis (strain 168)*	DNA gyrase subunit A		Unknown	*Escherichia coli K-12*	DNA gyrase	0	Unknown		Unknown				Unknown	18048160938; 180424145316	Unknown	
	DNA topoisomerase IV inhibitor	0.198	0.002		DNA gyrase subunit B	7.33E-15	0.46		DNA gyrase subunit B			*Staphylococcus aureus subsp. aureus Mu50*	DNA gyrase	0										
				Mycobacterium tuberculosis	DNA gyrase subunit A	7.61E-39	0.39		DNA topoisomerase IV subunit A				DNA gyrase subunit B	0										
				Staphylococcus aureus	DNA topoisomerase 4 subunit A	1.15E-07	0.46	*Escherichia coli*	DNA gyrase subunit A															
					DNA topoisomerase 4 subunit B	3.95E-06	0.46		DNA gyrase subunit B															
								Escherichia coli K-12	DNA gyrase subunit A															
									DNA gyrase subunit B															
									DNA topoisomerase IV subunit A															
								Pseudomonas aeruginosa (strain ATCC 15692 / PAO1 / 1C / PRS 101 / LMG12228)	DNA gyrase subunit A															
									DNA gyrase subunit B															
								Mycobacterium tuberculosis	DNA gyrase subunit A															
									DNA gyrase subunit B															
								Staphylococcus aureus subsp. aureus Mu50	DNA gyrase subunit A															
									DNA gyrase subunit B															
									DNA topoisomerase IV subunit A															
								Staphylococcus aureus subsp. aureus N315	DNA gyrase subunit B															
A 57241	DNA gyrase inhibitor	0.431	0.001	*Escherichia coli*	DNA gyrase subunit A	1.42E-52	0.53	*Bacillus subtilis (strain 168)*	DNA gyrase subunit A		Unknown	*Escherichia coli K-12*	DNA gyrase	0	Unknown		Unknown				Unknown	18048201624; 180424145353	Unknown	
	DNA topoisomerase IV inhibitor	0.195	0.002		DNA gyrase subunit B	3.28E-31	0.53		DNA gyrase subunit B			*Staphylococcus aureus subsp. aureus Mu50*	DNA gyrase	0										
				Mycobacterium tuberculosis	DNA gyrase subunit A	5.33E-36	0.35		DNA topoisomerase IV subunit A				DNA gyrase subunit B	0										
				Staphylococcus aureus	DNA topoisomerase 4 subunit A	7.18E-08	0.50		DNA topoisomerase IV subunit B															
					DNA topoisomerase 4 subunit B	1.34E-06	0.50	*Escherichia coli*	DNA gyrase subunit A															
									DNA gyrase subunit B															
								Escherichia coli K-12	DNA gyrase subunit A															
									DNA gyrase subunit B															
									DNA topoisomerase IV subunit A															
								Pseudomonas aeruginosa (strain ATCC 15692 / PAO1 / 1C / PRS 101 / LMG12228)	DNA gyrase subunit A															
									DNA gyrase subunit B															
								Mycobacterium tuberculosis	DNA gyrase subunit B															
								Staphylococcus aureus subsp. aureus Mu50	DNA gyrase subunit A															
									DNA gyrase subunit B															
									DNA topoisomerase IV subunit A															
								Staphylococcus aureus subsp. aureus N315	DNA gyrase subunit B															
A 57274 (A 62917)	DNA gyrase inhibitor	0.329	0.002	*Escherichia coli*	DNA gyrase subunit A	1.74E-40	0.49	*Bacillus subtilis (strain 168)*	DNA gyrase subunit A		Unknown	*Staphylococcus aureus subsp. aureus Mu50*	DNA gyrase	0	Unknown		Unknown				Unknown	18048172953; 180424145427	Unknown	
	DNA topoisomerase IV inhibitor	0.247	0.002		DNA gyrase subunit B	4.42E-24	0.49		DNA gyrase subunit B				DNA gyrase subunit B	0										
				Mycobacterium tuberculosis	DNA gyrase subunit A	7.36E-38	0.38		DNA topoisomerase IV subunit A															
				Staphylococcus aureus	DNA topoisomerase 4 subunit A	7.16E-08	0.49	*Escherichia coli*	DNA gyrase subunit A															
					DNA topoisomerase 4 subunit B	1.67E-06	0.49		DNA gyrase subunit B															
								Escherichia coli K-12	DNA gyrase subunit A															
									DNA gyrase subunit B															
									DNA topoisomerase IV subunit A															
								Pseudomonas aeruginosa (strain ATCC 15692 / PAO1 / 1C / PRS 101 / LMG12228)	DNA gyrase subunit A															
									DNA gyrase subunit B															
								Mycobacterium tuberculosis	DNA gyrase subunit A															
									DNA gyrase subunit B															
								Staphylococcus aureus subsp. aureus Mu50	DNA gyrase subunit A															
									DNA gyrase subunit B															
									DNA topoisomerase IV subunit A															
								Staphylococcus aureus subsp. aureus N315	DNA gyrase subunit B															
A 60919 (PD 118106)	DNA gyrase inhibitor	0.414	0.001	*Escherichia coli*	DNA gyrase subunit A	2.04E-37	0.64	*Bacillus subtilis (strain 168)*	DNA gyrase subunit A		Unknown	*Escherichia coli K-12*	DNA gyrase	0	Unknown		Unknown				Unknown	18048201702; 180424145510	Unknown	
	DNA topoisomerase IV inhibitor	0.227	0.002		DNA gyrase subunit B	2.85E-22	0.64		DNA gyrase subunit B			*Staphylococcus aureus subsp. aureus Mu50*	DNA gyrase	0										
				Mycobacterium tuberculosis	DNA gyrase subunit A	2.07E-53	0.54		DNA topoisomerase IV subunit A				DNA gyrase subunit B	0										
				Staphylococcus aureus	DNA topoisomerase 4 subunit A	2.07E-10	0.64		DNA topoisomerase IV subunit B															
					DNA topoisomerase 4 subunit B	2.74E-08	0.64	*Escherichia coli*	DNA gyrase subunit A															
									DNA gyrase subunit B															
								Escherichia coli K-12	DNA gyrase subunit A															
									DNA gyrase subunit B															
									DNA topoisomerase IV subunit A															
								Pseudomonas aeruginosa (strain ATCC 15692 / PAO1 / 1C / PRS 101 / LMG12228)	DNA gyrase subunit A															
									DNA gyrase subunit B															
								Mycobacterium tuberculosis	DNA gyrase subunit A															
									DNA gyrase subunit B															
								Staphylococcus aureus subsp. aureus Mu50	DNA gyrase subunit A															
									DNA gyrase subunit B															
									DNA topoisomerase IV subunit A															
								Staphylococcus aureus subsp. aureus N315	DNA gyrase subunit B															
A 61867 (BRN 4276829)	DNA gyrase inhibitor	0.435	0.001	*Escherichia coli*	DNA gyrase subunit A	1.30E-49	0.48	*Bacillus subtilis (strain 168)*	DNA gyrase subunit A		Unknown	*Escherichia coli K-12*	DNA gyrase	0	Unknown		Unknown			*Staphylococcus aureus (strain MSSA476)*	DNA topoisomerase 4 subunit A	18048201741; 180424145550	Unknown	
	DNA topoisomerase IV inhibitor	0.189	0.002		DNA gyrase subunit B	1.83E-29	0.48		DNA gyrase subunit B			*Staphylococcus aureus subsp. aureus Mu50*	DNA gyrase	0										
				Mycobacterium tuberculosis	DNA gyrase subunit A	2.81E-34	0.33		DNA topoisomerase IV subunit A				DNA gyrase subunit B	0										
				Staphylococcus aureus	DNA topoisomerase 4 subunit A	1.57E-07	0.48		DNA topoisomerase IV subunit B															
					DNA topoisomerase 4 subunit B	2.52E-06	0.48	*Escherichia coli*	DNA gyrase subunit A															
									DNA gyrase subunit B															
								Escherichia coli K-12	DNA gyrase subunit A															
									DNA gyrase subunit B															
									DNA topoisomerase IV subunit A															
								Pseudomonas aeruginosa (strain ATCC 15692 / PAO1 / 1C / PRS 101 / LMG12228)	DNA gyrase subunit A															
									DNA gyrase subunit B															
								Mycobacterium tuberculosis	DNA gyrase subunit B															
								Staphylococcus aureus subsp. aureus Mu50	DNA gyrase subunit A															
									DNA gyrase subunit B															
									DNA topoisomerase IV subunit A															
								Staphylococcus aureus subsp. aureus N315	DNA gyrase subunit B															
A 62251 (A 57531; PD 137954)	DNA gyrase inhibitor	0.326	0.002	*Escherichia coli*	DNA gyrase subunit A	1.98E-51	0.64	*Bacillus subtilis (strain 168)*	DNA gyrase subunit A		Unknown	*Escherichia coli K-12*	DNA gyrase	0	Unknown		Unknown			*Staphylococcus aureus (strain MSSA476)*	DNA topoisomerase 4 subunit A	18048201814; 180424145628	Unknown	
	DNA topoisomerase IV inhibitor	0.130	0.003		DNA gyrase subunit B	1.55E-30	0.64		DNA gyrase subunit B			*Staphylococcus aureus subsp. aureus Mu50*	DNA gyrase	0										
				Mycobacterium tuberculosis	DNA gyrase subunit A	3.61E-35	0.35		DNA topoisomerase IV subunit A				DNA gyrase subunit B	0										
				Staphylococcus aureus	DNA topoisomerase 4 subunit A	5.62E-09	0.64		DNA topoisomerase IV subunit B															
					DNA topoisomerase 4 subunit B	2.74E-08	0.64	*Escherichia coli*	DNA gyrase subunit A															
									DNA gyrase subunit B															
								Escherichia coli K-12	DNA gyrase subunit A															
									DNA gyrase subunit B															
									DNA topoisomerase IV subunit A															
								Pseudomonas aeruginosa (strain ATCC 15692 / PAO1 / 1C / PRS 101 / LMG12228)	DNA gyrase subunit A															
									DNA gyrase subunit B															
								Mycobacterium tuberculosis	DNA gyrase subunit B															
								Staphylococcus aureus subsp. aureus Mu50	DNA gyrase subunit A															
									DNA gyrase subunit B															
									DNA topoisomerase IV subunit A															
								Staphylococcus aureus subsp. aureus N315	DNA gyrase subunit B															
A 62255	DNA gyrase inhibitor	0.398	0.001	*Escherichia coli*	DNA gyrase subunit A	1.12E-50	0.52	*Bacillus subtilis (strain 168)*	DNA gyrase subunit A		Unknown	*Escherichia coli K-12*	DNA gyrase	0	Unknown		Unknown				Unknown	18048201841; 180424145711	Unknown	
	DNA topoisomerase IV inhibitor	0.140	0.003		DNA gyrase subunit B	4.31E-30	0.52		DNA gyrase subunit B			*Staphylococcus aureus subsp. aureus Mu50*	DNA gyrase subunit B	0										
				Mycobacterium tuberculosis	DNA gyrase subunit A	9.99E-45	0.49		DNA topoisomerase IV subunit A															
				Staphylococcus aureus	DNA topoisomerase 4 subunit A	2.29E-08	0.52		DNA topoisomerase IV subunit B															
					DNA topoisomerase 4 subunit B	7.01E-07	0.52	*Escherichia coli*	DNA gyrase subunit A															
									DNA gyrase subunit B															
								Escherichia coli K-12	DNA gyrase subunit A															
									DNA gyrase subunit B															
									DNA topoisomerase IV subunit A															
								Pseudomonas aeruginosa (strain ATCC 15692 / PAO1 / 1C / PRS 101 / LMG12228)	DNA gyrase subunit A															
									DNA gyrase subunit B															
								Mycobacterium tuberculosis	DNA gyrase subunit B															
								Staphylococcus aureus	DNA topoisomerase IV subunit A															
								Staphylococcus aureus subsp. aureus Mu50	DNA gyrase subunit A															
									DNA gyrase subunit B															
									DNA topoisomerase IV subunit A															
								Staphylococcus aureus subsp. aureus N315	DNA gyrase subunit B															
A 62824	DNA gyrase inhibitor	0.680	0.001	*Escherichia coli*	DNA gyrase subunit A	2.16E-23	0.52	*Escherichia coli K-12*	DNA gyrase subunit A		Unknown	*Escherichia coli K-12*	DNA gyrase	0	Unknown		Unknown			*Staphylococcus aureus (strain MSSA476)*	DNA topoisomerase 4 subunit A	18049164042	DNA gyrase	Bioorg. Med. Chem. Lett.,(2006)16:5:1272
	DNA topoisomerase IV inhibitor	0.938	0.000		DNA gyrase subunit B	5.33E-14	0.52		DNA gyrase subunit B			*Staphylococcus aureus*	Topoisomerase IV subunit A	0									DNA gyrase subunit A	Antimicrob. Agents Chemother.,(2010)54:8:3478
				Mycobacterium tuberculosis	DNA gyrase subunit A	2.11E-25	0.47																Topoisomerase IV subunit A	Bioorg. Med. Chem. Lett.,(2006)16:5:1272; Bioorg. Med. Chem. Lett.,(2006)16:5:1277
				Staphylococcus aureus	DNA topoisomerase 4 subunit A	6.23E-97	0.61																	
					DNA topoisomerase 4 subunit B	6.87E-07	0.52																	

Bioactive molecule	PASS online[4]			Similarity Ensemble Approach (SEA)[2]				ChemProt[3]		SuperPred[6]		Polypharmacology Browser (PPB)[1]			SPiDER[7]		HitPick[5]			PharmMapper[9]			TargetHunter[8]	
	Activity	Pa	Pi	Organism	Target	P-Value	Max TC	Organism	Target	Organism	Target	Organism	Target	P-Value	Target	Confidence level	Target	Precision (%)	Tc similarity	Organism	Target	Job Id	Target	Reference
A 65326	DNA gyrase inhibitor	0.379	0.001	*Escherichia coli*	DNA gyrase subunit A	6.28E-43	0.39	*Escherichia coli K-12*	DNA gyrase subunit A		Unknown	*Escherichia coli K-12*	DNA gyrase	0	Unknown		Unknown				Unknown	18048173733; 180424145804	Unknown	
	DNA topoisomerase IV inhibitor	0.126	0.003		DNA gyrase subunit B	1.60E-25	0.39		DNA gyrase subunit B			*Staphylococcus aureus subsp. aureus Mu50*	DNA gyrase	0										
				Mycobacterium tuberculosis	DNA gyrase subunit A	6.12E-34	0.34	*Pseudomonas aeruginosa (strain ATCC 15692 / PAO1 / 1C / PRS 101 / LMG12228)*	DNA gyrase subunit A				DNA gyrase subunit B	0										
				Staphylococcus aureus	DNA topoisomerase 4 subunit A	1.03E-06	0.39		DNA gyrase subunit B															
								Staphylococcus aureus subsp. aureus Mu50	DNA gyrase subunit A															
									DNA gyrase subunit B															
									DNA topoisomerase IV subunit A															
								Staphylococcus aureus subsp. aureus N315	DNA gyrase subunit B															
ACH 702	DNA gyrase inhibitor	0.886	0.000	*Mycobacterium tuberculosis*	DNA gyrase subunit A	1.92E-94	1.00		Unknown		Unknown	*Escherichia coli K-12*	DNA gyrase	0	Unknown		Unknown				Unknown	18049164103	DNA gyrase	Bioorg. Med. Chem. Lett.,(2006)16:5:1272
	DNA topoisomerase IV inhibitor	0.848	0.000	*Staphylococcus aureus*	DNA topoisomerase 4 subunit A	1.10E-76	0.50					*Staphylococcus aureus subsp. aureus Mu50*	DNA gyrase	0.01 to 0									DNA gyrase subunit A	Antimicrob. Agents Chemother.,(2010)54:8:3478
													DNA gyrase subunit B	0									Topoisomerase IV subunit A	Bioorg. Med. Chem. Lett.,(2006)16:5:1272; Bioorg. Med. Chem. Lett.,(2006)16:5:1277
												Staphylococcus aureus	Topoisomerase IV subunit A	0										
Acorafloxacin (avarofloxacin)	DNA gyrase inhibitor	0.365	0.001	*Escherichia coli*	DNA gyrase subunit A	2.54E-32	0.40	*Escherichia coli*	DNA gyrase subunit A		Unknown	*Staphylococcus aureus subsp. aureus Mu50*	DNA gyrase subunit B	0	Unknown		Unknown				Unknown	18048111050; 180424145836	DNA gyrase	Bioorg. Med. Chem. Lett.,(2006)16:5:1272; Bioorg. Med. Chem. Lett.,(2008)18:3:1229; J. Med. Chem.,(1993)36:7:871; J. Med. Chem.,(2003)46:17:3655; J. Med. Chem.,(2006)49:22:6435
	DNA topoisomerase IV inhibitor	0.284	0.002		DNA gyrase subunit B	2.89E-19	0.40		DNA gyrase subunit B														DNA gyrase subunit A	Antimicrob. Agents Chemother.,(2010)54:8:3478
				Mycobacterium tuberculosis	DNA gyrase subunit A	1.16E-82	0.61	*Escherichia coli K-12*	DNA gyrase subunit A														DNA gyrase subunit B	Bioorg. Med. Chem.,(2008)16:5:2558; Bioorg. Med. Chem.,(2014)22:17:4924; Bioorg. Med. Chem.,(2015)23:9:2062; J. Med. Chem.,(1992)35:25:4745
				Staphylococcus aureus	DNA topoisomerase 4 subunit A	2.77E-09	0.59		DNA gyrase subunit B														Topoisomerase IV subunit A	Bioorg. Med. Chem. Lett.,(2006)16:5:1272; Bioorg. Med. Chem. Lett.,(2006)16:5:1277
								Mycobacterium smegmatis (strain ATCC 700084 / mc(2)155)	DNA gyrase subunit B															
								Mycobacterium tuberculosis	DNA gyrase subunit A															
									DNA gyrase subunit B															
								Staphylococcus aureus subsp. aureus Mu50	DNA gyrase subunit B															
									DNA topoisomerase IV subunit A															
ADDNC (A 65485)	DNA gyrase inhibitor	0.360	0.001	*Escherichia coli*	DNA gyrase subunit A	3.97E-25	0.33	*Escherichia coli K-12*	DNA gyrase subunit A		Unknown	*Staphylococcus aureus subsp. aureus Mu50*	DNA gyrase	0	Unknown		Unknown				Unknown	18048142430	Unknown	
	DNA topoisomerase IV inhibitor	0.228	0.002		DNA gyrase subunit B	5.00E-15	0.33		DNA gyrase subunit B				DNA gyrase subunit B	0										
				Mycobacterium tuberculosis	DNA gyrase subunit A	8.85E-35	0.35	*Pseudomonas aeruginosa (strain ATCC 15692 / PAO1 / 1C / PRS 101 / LMG12228)*	DNA gyrase subunit A															
				Staphylococcus aureus	DNA topoisomerase 4 subunit A	3.29E-06	0.33		DNA gyrase subunit B															
								Staphylococcus aureus subsp. aureus Mu50	DNA gyrase subunit A															
								Staphylococcus aureus subsp. aureus N315	DNA gyrase subunit B															
Alalevonadifloxacin	DNA gyrase inhibitor	0.085	0.004	*Escherichia coli*	DNA gyrase subunit A	1.83E-18	0.36	*Escherichia coli*	DNA gyrase subunit A		Unknown	*Escherichia coli K-12*	DNA gyrase	0	Unknown		Unknown			*Staphylococcus aureus (strain MSSA476)*	DNA topoisomerase 4 subunit A	18048152557; 180424145941	DNA gyrase subunit B	Bioorg. Med. Chem.,(2008)16:5:2558; J. Med. Chem.,(1992)35:25:4745
	DNA topoisomerase IV inhibitor	0.041	0.004		DNA gyrase subunit B	4.29E-11	0.36		DNA gyrase subunit B			*Staphylococcus aureus subsp. aureus Mu50*	DNA gyrase subunit B	0									Topoisomerase IV subunit A	Bioorg. Med. Chem. Lett.,(2004)14:20:5193; J. Med. Chem.,(2005)48:16:5232
				Mycobacterium tuberculosis	DNA gyrase subunit A	8.63E-45	0.45	*Escherichia coli K-12*	DNA gyrase subunit A															
				Staphylococcus aureus	DNA topoisomerase 4 subunit A	2.82E-07	0.40		DNA gyrase subunit B															
								Mycobacterium tuberculosis	DNA gyrase subunit B															
								Staphylococcus aureus	DNA topoisomerase IV subunit A															
								Staphylococcus aureus subsp. aureus Mu50	DNA gyrase subunit A															
									DNA gyrase subunit B															
								Staphylococcus aureus subsp. aureus N315	DNA gyrase subunit B															
Alatrofloxacin	DNA gyrase inhibitor	0.161	0.003	*Escherichia coli*	DNA gyrase subunit A	1.11E-16	0.31	*Escherichia coli K-12*	DNA gyrase subunit A		Unknown		Unknown		Unknown		Unknown				Unknown	18048123814; 180424174929	DNA gyrase	J. Med. Chem.,(1991)34:3:1142; J. Med. Chem.,(1993)36:7:871; J. Med. Chem.,(1993)36:14:1964; J. Med. Chem.,(1996)39:16:3070; J. Med. Chem.,(2003)46:17:3655
	DNA topoisomerase IV inhibitor	0.077	0.003		DNA gyrase subunit B	5.71E-10	0.31		DNA gyrase subunit B														DNA gyrase subunit B	J. Med. Chem.,(1992)35:25:4745
				Mycobacterium tuberculosis	DNA gyrase subunit A	2.59E-33	0.32	*Pseudomonas aeruginosa (strain ATCC 15692 / PAO1 / 1C / PRS 101 / LMG12228)*	DNA gyrase subunit A														Topoisomerase IV subunit A	Bioorg. Med. Chem. Lett.,(2004)14:20:5193; Bioorg. Med. Chem. Lett.,(2006)16:5:1272
				Staphylococcus aureus	DNA topoisomerase 4 subunit A	5.77E-06	0.31		DNA gyrase subunit B															
								Staphylococcus aureus subsp. aureus Mu50	DNA gyrase subunit A															
								Staphylococcus aureus subsp. aureus N315	DNA gyrase subunit B															
Amifloxacin	DNA gyrase inhibitor	0.048	0.005	*Escherichia coli*	DNA gyrase subunit A	8.98E-51	0.52	*Escherichia coli*	DNA gyrase subunit A		Unknown	*Escherichia coli K-12*	DNA gyrase	0	Unknown		Unknown				Unknown	18048103621; 180424174959	Unknown	
	DNA topoisomerase IV inhibitor	0.046	0.004		DNA gyrase subunit B	3.79E-30	0.52		DNA gyrase subunit B			*Staphylococcus aureus subsp. aureus Mu50*	DNA gyrase subunit B	0										
				Mycobacterium tuberculosis	DNA gyrase subunit A	3.50E-34	0.33	*Escherichia coli K-12*	DNA gyrase subunit A															
				Staphylococcus aureus	DNA topoisomerase 4 subunit A	7.23E-08	0.52		DNA gyrase subunit B															
					DNA topoisomerase 4 subunit B	8.39E-07	0.52	*Mycobacterium tuberculosis*	DNA gyrase subunit A															
									DNA gyrase subunit B															
								Staphylococcus aureus subsp. aureus Mu50	DNA gyrase subunit A															
									DNA gyrase subunit B															
								Staphylococcus aureus subsp. aureus N315	DNA gyrase subunit B															
Antofloxacin	DNA gyrase inhibitor	0.427	0.001	*Mycobacterium tuberculosis*	DNA gyrase subunit A	4.29E-17	0.31	*Escherichia coli*	DNA gyrase subunit A		Unknown	*Escherichia coli K-12*	DNA gyrase	0	Unknown		Unknown				Unknown	18048142456; 180424175030	Unknown	
	DNA topoisomerase IV inhibitor	0.147	0.002						DNA gyrase subunit B			*Staphylococcus aureus subsp. aureus Mu50*	DNA gyrase subunit B	0.01 to 0										
								Escherichia coli K-12	DNA gyrase subunit A															
									DNA gyrase subunit B															
								Mycobacterium smegmatis	DNA topoisomerase (ATP-hydrolysing) (DNA gyrase)															
								Mycobacterium tuberculosis	DNA gyrase subunit A															
									DNA gyrase subunit B															
								Staphylococcus aureus subsp. aureus Mu50	DNA gyrase subunit A															
									DNA gyrase subunit B															
								Staphylococcus aureus subsp. aureus N315	DNA gyrase subunit B															
AT 4929	DNA gyrase inhibitor	0.453	0.001	*Escherichia coli*	DNA gyrase subunit A	1.21E-36	0.52	*Bacillus subtilis (strain 168)*	DNA gyrase subunit A		Unknown	*Escherichia coli K-12*	DNA gyrase	0	Unknown		Unknown				Unknown	18049133116	Unknown	
	DNA topoisomerase IV inhibitor	0.166	0.002		DNA gyrase subunit B	8.17E-22	0.52		DNA gyrase subunit B			*Staphylococcus aureus subsp. aureus Mu50*	DNA gyrase subunit B	0										
				Mycobacterium tuberculosis	DNA gyrase subunit A	2.12E-44	0.46	*Bacillus subtilis (strain 168)*	DNA topoisomerase IV subunit A															
				Staphylococcus aureus	DNA topoisomerase 4 subunit A	1.65E-08	0.52		DNA topoisomerase IV subunit B															
					DNA topoisomerase 4 subunit B	8.52E-07	0.52	*Escherichia coli K-12*	DNA gyrase subunit A															
									DNA gyrase subunit B															
									DNA topoisomerase IV subunit A															
								Pseudomonas aeruginosa (strain ATCC 15692 / PAO1 / 1C / PRS 101 / LMG12228)	DNA gyrase subunit A															
									DNA gyrase subunit B															
								Mycobacterium tuberculosis	DNA gyrase subunit B															
								Staphylococcus aureus subsp. aureus Mu50	DNA gyrase subunit A															
									DNA gyrase subunit B															
									DNA topoisomerase IV subunit A															
								Staphylococcus aureus subsp. aureus N315	DNA gyrase subunit B															
Balofloxacin	DNA gyrase inhibitor	0.549	0.001	*Escherichia coli*	DNA gyrase subunit A	2.74E-34	0.43	*Bacillus subtilis (strain 168)*	DNA gyrase subunit A		Unknown	*Escherichia coli K-12*	DNA gyrase	0.01 to 0	Unknown		Unknown				Unknown	18048101205; 180424175110	Unknown	
	DNA topoisomerase IV inhibitor	0.307	0.002		DNA gyrase subunit B	2.00E-20	0.43		DNA gyrase subunit B			*Staphylococcus aureus subsp. aureus Mu50*	DNA gyrase subunit B	0										
				Mycobacterium tuberculosis	DNA gyrase subunit A	8.05E-94	0.73		DNA topoisomerase IV subunit A															
				Staphylococcus aureus	DNA topoisomerase 4 subunit A	8.80E-13	0.66	*Escherichia coli*	DNA gyrase subunit A															
					DNA topoisomerase 4 subunit B	7.92E-06	0.43		DNA gyrase subunit B															
								Escherichia coli K-12	DNA gyrase subunit A															
									DNA gyrase subunit B															
									DNA topoisomerase IV subunit A															
								Pseudomonas aeruginosa (strain ATCC 15692 / PAO1 / 1C / PRS 101 / LMG12228)	DNA gyrase subunit A															
									DNA gyrase subunit B															
								Mycobacterium tuberculosis	DNA gyrase subunit B															
								Staphylococcus aureus subsp. aureus Mu50	DNA gyrase subunit A															
									DNA gyrase subunit B															
									DNA topoisomerase IV subunit A															
								Staphylococcus aureus subsp. aureus N315	DNA gyrase subunit B															
BAY Y-3118 free base	DNA gyrase inhibitor	0.626	0.001	*Escherichia coli*	DNA gyrase subunit A	2.56E-33	0.46	*Bacillus subtilis (strain 168)*	DNA gyrase subunit A		Unknown	*Escherichia coli K-12*	DNA gyrase	0	Unknown		Unknown			*Staphylococcus aureus (strain MSSA476)*	DNA topoisomerase 4 subunit A	18048161009; 180424175236	Unknown	
	DNA topoisomerase IV inhibitor	0.417	0.001		DNA gyrase subunit B	7.45E-20	0.46		DNA gyrase subunit B			*Staphylococcus aureus subsp. aureus Mu50*	DNA gyrase	0										
				Mycobacterium tuberculosis	DNA gyrase subunit A	3.18E-70	0.78		DNA topoisomerase IV subunit A			*Staphylococcus aureus subsp. aureus Mu50*	DNA gyrase subunit B	0										
				Staphylococcus aureus	DNA topoisomerase 4 subunit A	5.03E-14	0.78		DNA topoisomerase IV subunit B															
					DNA topoisomerase 4 subunit B	4.31E-06	0.46	*Escherichia coli*	DNA gyrase subunit A															
									DNA gyrase subunit B															
								Escherichia coli K-12	DNA gyrase subunit A															
									DNA gyrase subunit B															
									DNA topoisomerase IV subunit A															
								Pseudomonas aeruginosa (strain ATCC 15692 / PAO1 / 1C / PRS 101 / LMG12228)	DNA gyrase subunit A															
									DNA gyrase subunit B															
								Mycobacterium tuberculosis	DNA gyrase subunit A															
									DNA gyrase subunit B															
								Staphylococcus aureus subsp. aureus Mu50	DNA gyrase subunit A															
									DNA gyrase subunit B															
									DNA topoisomerase IV subunit A															
								Staphylococcus aureus subsp. aureus N315	DNA gyrase subunit B															
Besifloxacin	DNA gyrase inhibitor	0.678	0.001	*Escherichia coli*	DNA gyrase subunit A	4.24E-36	0.46	*Bacillus subtilis (strain 168)*	DNA gyrase subunit A		Unknown	*Staphylococcus aureus subsp. aureus Mu50*	DNA gyrase subunit B	0	Unknown		Unknown			*Staphylococcus aureus (strain MSSA476)*	DNA topoisomerase 4 subunit A	18048095852; 180424175314	Unknown	
	DNA topoisomerase IV inhibitor	0.428	0.001		DNA gyrase subunit B	1.71E-21	0.46		DNA gyrase subunit B															
				Mycobacterium tuberculosis	DNA gyrase subunit A	4.12E-71	0.54		DNA topoisomerase IV subunit A															
				Staphylococcus aureus	DNA topoisomerase 4 subunit A	5.13E-09	0.49		DNA topoisomerase IV subunit B															
					DNA topoisomerase 4 subunit B	3.71E-06	0.46	*Escherichia coli K-12*	DNA gyrase subunit A															
									DNA gyrase subunit B															
									DNA topoisomerase IV subunit A															
								Pseudomonas aeruginosa (strain ATCC 15692 / PAO1 / 1C / PRS 101 / LMG12228)	DNA gyrase subunit A															
									DNA gyrase subunit B															
								Staphylococcus aureus subsp. aureus Mu50	DNA gyrase subunit A															
									DNA gyrase subunit B															
									DNA topoisomerase IV subunit A															
								Staphylococcus aureus subsp. aureus N315	DNA gyrase subunit B															

Bioactive molecule	PASS online[4] Activity	Pa	Pi	Similarity Ensemble Approach (SEA)[2] Organism	Target	P-Value	Max TC	ChemProt[3] Organism	Target
Binfloxacin	DNA gyrase inhibitor	0.292	0.002	*Escherichia coli*	DNA gyrase subunit A	5.84E-46	0.50	*Escherichia coli*	DNA gyrase subunit A
	DNA topoisomerase IV inhibitor	0.075	0.003		DNA gyrase subunit B	2.61E-27	0.50		DNA gyrase subunit B
				Mycobacterium tuberculosis	DNA gyrase subunit A	3.95E-40	0.38	*Escherichia coli K-12*	DNA gyrase subunit A
				Staphylococcus aureus	DNA topoisomerase 4 subunit A	2.81E-08	0.50		DNA gyrase subunit B
					DNA topoisomerase 4 subunit B	1.34E-06	0.50	*Mycobacterium tuberculosis*	DNA gyrase subunit A
									DNA gyrase subunit B
								Staphylococcus aureus subsp. aureus Mu50	DNA gyrase subunit A
									DNA gyrase subunit B
								Staphylococcus aureus subsp. aureus N315	DNA gyrase subunit B
BMY 40062	DNA gyrase inhibitor	0.105	0.003	*Escherichia coli*	DNA gyrase subunit A	7.77E-13	0.34	*Escherichia coli K-12*	DNA gyrase subunit A
	DNA topoisomerase IV inhibitor	0.056	0.003		DNA gyrase subunit B	8.95E-08	0.34		DNA gyrase subunit B
				Mycobacterium tuberculosis	DNA gyrase subunit A	9.05E-41	0.39		
				Staphylococcus aureus	DNA topoisomerase 4 subunit A	6.53E-07	0.38		
BMY 40397	DNA gyrase inhibitor	0.129	0.003	*Mycobacterium tuberculosis*	DNA gyrase subunit A	3.16E-39	0.38		Unknown
	DNA topoisomerase IV inhibitor	0.069	0.003	*Staphylococcus aureus*	DNA topoisomerase 4 subunit A	8.60E-07	0.35		
BMY 42230	DNA gyrase inhibitor	0.066	0.004	*Escherichia coli*	DNA gyrase subunit A	3.59E-22	0.44	*Bacillus subtilis (strain 168)*	DNA gyrase subunit A
	DNA topoisomerase IV inhibitor	0.057	0.003		DNA gyrase subunit B	2.79E-13	0.44		DNA gyrase subunit B
				Mycobacterium tuberculosis	DNA gyrase subunit A	3.33E-39	0.39		DNA topoisomerase IV subunit A
				Staphylococcus aureus	DNA topoisomerase 4 subunit A	1.62E-07	0.44	*Escherichia coli*	DNA gyrase subunit A
					DNA topoisomerase 4 subunit B	6.99E-06	0.44		DNA gyrase subunit B
								Escherichia coli K-12	DNA gyrase subunit A
									DNA gyrase subunit B
									DNA topoisomerase IV subunit A
								Pseudomonas aeruginosa (strain ATCC 15692 / PAO1 / 1C / PRS 101 / LMG12228)	DNA gyrase subunit A
									DNA gyrase subunit B
								Mycobacterium tuberculosis	DNA gyrase subunit A
									DNA gyrase subunit B
								Staphylococcus aureus subsp. aureus Mu50	DNA gyrase subunit A
									DNA gyrase subunit B
								Staphylococcus aureus subsp. aureus N315	DNA gyrase subunit B
BMY 43261	DNA gyrase inhibitor	0.095	0.003	*Escherichia coli*	DNA gyrase subunit A	1.59E-10	0.45	*Bacillus subtilis (strain 168)*	DNA gyrase subunit A
	DNA topoisomerase IV inhibitor	0.077	0.003		DNA gyrase subunit B	2.07E-06	0.45		DNA gyrase subunit B
				Mycobacterium tuberculosis	DNA gyrase subunit A	7.36E-38	0.38		DNA topoisomerase IV subunit A
				Staphylococcus aureus	DNA topoisomerase 4 subunit A	1.81E-07	0.45	*Escherichia coli*	DNA gyrase subunit A
					DNA topoisomerase 4 subunit B	5.81E-06	0.45		DNA gyrase subunit B
								Escherichia coli K-12	DNA gyrase subunit A
									DNA gyrase subunit B
									DNA topoisomerase IV subunit A
								Pseudomonas aeruginosa (strain ATCC 15692 / PAO1 / 1C / PRS 101 / LMG12228)	DNA gyrase subunit A
									DNA gyrase subunit B
								Mycobacterium tuberculosis	DNA gyrase subunit A
									DNA gyrase subunit B
								Staphylococcus aureus subsp. aureus Mu50	DNA gyrase subunit A
									DNA gyrase subunit B
								Staphylococcus aureus subsp. aureus N315	DNA gyrase subunit B
BMY 43748	DNA gyrase inhibitor	0.367	0.001	*Escherichia coli*	DNA gyrase subunit A	2.73E-29	0.32	*Escherichia coli*	DNA gyrase subunit A
	DNA topoisomerase IV inhibitor	0.164	0.002		DNA gyrase subunit B	1.77E-17	0.32		DNA gyrase subunit B
				Mycobacterium tuberculosis	DNA gyrase subunit A	5.76E-32	0.31		DNA topoisomerase IV subunit A
				Staphylococcus aureus	DNA topoisomerase 4 subunit A	5.15E-06	0.32	*Escherichia coli K-12*	DNA gyrase subunit A
									DNA gyrase subunit B
									DNA topoisomerase IV subunit A
								Pseudomonas aeruginosa (strain ATCC 15692 / PAO1 / 1C / PRS 101 / LMG12228)	DNA gyrase subunit A
									DNA gyrase subunit B
								Mycobacterium smegmatis	DNA topoisomerase (ATP-hydrolysing) (DNA gyrase)
								Mycobacterium smegmatis (strain ATCC 700084 / mc(2)155)	DNA gyrase subunit B
								Mycobacterium tuberculosis	DNA gyrase subunit A
									DNA gyrase subunit B
								Staphylococcus aureus	DNA topoisomerase IV subunit A
								Staphylococcus aureus subsp. aureus Mu50	DNA gyrase subunit A
									DNA gyrase subunit B
									DNA topoisomerase IV subunit A
								Staphylococcus aureus subsp. aureus N315	DNA gyrase subunit B
BMY 45243	DNA gyrase inhibitor	0.195	0.002	*Mycobacterium tuberculosis*	DNA gyrase subunit A	1.33E-15	0.28	*Escherichia coli K-12*	DNA gyrase subunit A
	DNA topoisomerase IV inhibitor	0.069	0.003						DNA gyrase subunit B
								Pseudomonas aeruginosa (strain ATCC 15692 / PAO1 / 1C / PRS 101 / LMG12228)	DNA gyrase subunit A
									DNA gyrase subunit B
								Staphylococcus aureus subsp. aureus Mu50	DNA gyrase subunit A
									DNA gyrase subunit B
									DNA topoisomerase IV subunit A
								Staphylococcus aureus subsp. aureus N315	DNA gyrase subunit B
BMY 45706	DNA gyrase inhibitor	0.032	0.017	*Mycobacterium tuberculosis*	DNA gyrase subunit A	9.44E-35	0.34		Unknown
	DNA topoisomerase IV inhibitor	0.041	0.004	*Staphylococcus aureus*	DNA topoisomerase 4 subunit A	4.67E-06	0.31		
BRN 4913428 (PD 131199)	DNA gyrase inhibitor	0.565	0.001	*Escherichia coli*	DNA gyrase subunit A	4.66E-34	0.45	*Bacillus subtilis (strain 168)*	DNA gyrase subunit A
	DNA topoisomerase IV inhibitor	0.314	0.002		DNA gyrase subunit B	2.73E-20	0.45		DNA gyrase subunit B
				Mycobacterium tuberculosis	DNA gyrase subunit A	7.17E-72	0.54		DNA topoisomerase IV subunit A
				Staphylococcus aureus	DNA topoisomerase 4 subunit A	9.08E-09	0.48		DNA topoisomerase IV subunit B
					DNA topoisomerase 4 subunit B	5.56E-06	0.45	*Escherichia coli*	DNA gyrase subunit A
									DNA gyrase subunit B
								Escherichia coli K-12	DNA gyrase subunit A
									DNA gyrase subunit B
									DNA topoisomerase IV subunit A
								Pseudomonas aeruginosa (strain ATCC 15692 / PAO1 / 1C / PRS 101 / LMG12228)	DNA gyrase subunit A
									DNA gyrase subunit B
								Mycobacterium tuberculosis	DNA gyrase subunit A
									DNA gyrase subunit B
								Staphylococcus aureus subsp. aureus Mu50	DNA gyrase subunit A
									DNA gyrase subunit B
									DNA topoisomerase IV subunit A
								Staphylococcus aureus subsp. aureus N315	DNA gyrase subunit B
Cadrofloxacin	DNA gyrase inhibitor	0.423	0.001	*Escherichia coli*	DNA gyrase subunit A	3.55E-34	0.49	*Bacillus subtilis (strain 168)*	DNA gyrase subunit A
	DNA topoisomerase IV inhibitor	0.327	0.002		DNA gyrase subunit B	2.32E-20	0.49		DNA gyrase subunit B
				Mycobacterium tuberculosis	DNA gyrase subunit A	3.95E-92	0.82		DNA topoisomerase IV subunit A
				Staphylococcus aureus	DNA topoisomerase 4 subunit A	4.00E-10	0.60		DNA topoisomerase IV subunit B
					DNA topoisomerase 4 subunit B	2.00E-06	0.49	*Escherichia coli*	DNA gyrase subunit A
									DNA gyrase subunit B
								Escherichia coli K-12	DNA gyrase subunit A
									DNA gyrase subunit B
									DNA topoisomerase IV subunit A
								Pseudomonas aeruginosa (strain ATCC 15692 / PAO1 / 1C / PRS 101 / LMG12228)	DNA gyrase subunit A
									DNA gyrase subunit B
								Mycobacterium tuberculosis	DNA gyrase subunit B
								Staphylococcus aureus subsp. aureus Mu50	DNA gyrase subunit A
									DNA gyrase subunit B
								Staphylococcus aureus subsp. aureus N315	DNA gyrase subunit B
Cetefloxacin	DNA gyrase inhibitor	0.359	0.001	*Escherichia coli*	DNA gyrase subunit A	6.84E-44	0.41		Unknown
	DNA topoisomerase IV inhibitor	0.087	0.003		DNA gyrase subunit B	4.33E-26	0.41		
				Mycobacterium tuberculosis	DNA gyrase subunit A	9.35E-35	0.35		
				Staphylococcus aureus	DNA topoisomerase 4 subunit A	7.11E-07	0.41		

Bioactive molecule	SuperPred[6] Organism	Target	Polypharmacology Browser (PPB)[1] Organism	Target	P-Value	SPiDER[7] Target	Confidence level	HitPick[5] Target	Precision (%)	Tc similarity	PharmMapper[9] Organism	Target	Job Id	TargetHunter[8] Target	Reference
Binfloxacin		Unknown	*Escherichia coli K-12*	DNA gyrase	0	Unknown		Unknown			*Staphylococcus aureus (strain MSSA476)*	DNA topoisomerase 4 subunit A	18048103650; 180424175348	Unknown	
			Staphylococcus aureus subsp. aureus Mu50	DNA gyrase subunit B	0										
BMY 40062		Unknown	*Escherichia coli K-12*	DNA gyrase	0	Unknown		Unknown				Unknown	18048142537	Unknown	
			Staphylococcus aureus subsp. aureus Mu50	DNA gyrase	0										
				DNA gyrase subunit B	0										
BMY 40397		Unknown	*Escherichia coli K-12*	DNA gyrase	0	Unknown		Unknown			*Staphylococcus aureus (strain MSSA476)*	DNA topoisomerase 4 subunit A	18048173822; 180424175440	Unknown	
			Staphylococcus aureus subsp. aureus Mu50	DNA gyrase subunit B	0										
BMY 42230		Unknown	*Escherichia coli K-12*	DNA gyrase	0	Unknown		Unknown				Unknown	18048173940; 180424175520	Unknown	
			Staphylococcus aureus subsp. aureus Mu50	DNA gyrase subunit B	0										
BMY 43261		Unknown	*Escherichia coli K-12*	DNA gyrase	0	Unknown		Unknown				Unknown	18048175956; 180424175554	Unknown	
			Staphylococcus aureus subsp. aureus Mu50	DNA gyrase subunit B	0										
BMY 43748		Unknown	*Staphylococcus aureus subsp. aureus Mu50*	DNA gyrase	0	Unknown		Unknown				Unknown	18048132514; 180424181555	DNA gyrase	J. Med. Chem.,(1991)34:3:1155; J. Med. Chem.,(1992)35:10:1764; J. Med. Chem.,(1993)36:7:871; J. Med. Chem.,(1996)39:16:3070; J. Med. Chem.,(1993)36:14:1964
				DNA gyrase subunit B	0									DNA gyrase subunit B	J. Med. Chem.,(1992)35:25:4745
														Topoisomerase IV subunit A	Bioorg. Med. Chem. Lett.,(2006)16:5:1272
BMY 45243		Unknown	*Escherichia coli K-12*	DNA gyrase	0	Unknown		Unknown				Unknown	18048180036; 180424181651	Unknown	
			Staphylococcus aureus subsp. aureus Mu50	DNA gyrase subunit B	0										
BMY 45706		Unknown	*Escherichia coli K-12*	DNA gyrase	0	Unknown		Unknown				Unknown	18048180134; 180424181731	Unknown	
			Staphylococcus aureus subsp. aureus Mu50	DNA gyrase subunit B	0										
BRN 4913428 (PD 131199)		Unknown	*Escherichia coli K-12*	DNA gyrase	0	Unknown		Unknown				Unknown	18049133145	Unknown	
			Staphylococcus aureus subsp. aureus Mu50	DNA gyrase	0										
				DNA gyrase subunit B	0										
Cadrofloxacin		Unknown	*Escherichia coli K-12*	DNA gyrase	0.01 to 0	Unknown		Unknown			*Staphylococcus aureus (strain MSSA476)*	DNA topoisomerase 4 subunit A	18048111142; 180424181804	Unknown	
			Staphylococcus aureus subsp. aureus Mu50	DNA gyrase subunit B	0										
Cetefloxacin		Unknown	*Escherichia coli K-12*	DNA gyrase	0	Unknown		Unknown				Unknown	18048123900; 180424181848	Unknown	
			Staphylococcus aureus subsp. aureus Mu50	DNA gyrase	0										
				DNA gyrase subunit B	0										

Bioactive molecule	PASS online[4]			Similarity Ensemble Approach (SEA)[2]				ChemProt[3]		SuperPred[6]		Polypharmacology Browser (PPB)[1]			SPiDER[7]		HitPick[5]			PharmMapper[9]			TargetHunter[8]	
	Activity	Pa	Pi	Organism	Target	P-Value	Max TC	Organism	Target	Organism	Target	Organism	Target	P-Value	Target	Confidence level	Target	Precision (%)	Tc similarity	Organism	Target	Job Id	Target	Reference
Chinfloxacin	DNA gyrase inhibitor<br>DNA topoisomerase IV inhibitor	0.392<br>0.375	0.001<br>0.002	*Escherichia coli*<br><br>*Mycobacterium tuberculosis*<br>*Staphylococcus aureus*	DNA gyrase subunit A<br>DNA gyrase subunit B<br>DNA gyrase subunit A<br>DNA topoisomerase 4 subunit A<br>DNA topoisomerase 4 subunit B	1.73E-32<br>2.30E-19<br>5.26E-89<br>4.09E-14<br>8.45E-06	0.43<br>0.43<br>0.82<br>0.82<br>0.43	*Bacillus subtilis (strain 168)*<br><br><br><br>*Escherichia coli*<br><br>*Escherichia coli K-12*<br><br><br>*Pseudomonas aeruginosa (strain ATCC 15692 / PAO1 / 1C / PRS 101 / LMG12228)*<br><br>*Mycobacterium tuberculosis*<br>*Staphylococcus aureus subsp. aureus Mu50*<br><br><br>*Staphylococcus aureus subsp. aureus N315*	DNA gyrase subunit A<br>DNA gyrase subunit B<br>DNA topoisomerase IV subunit A<br>DNA topoisomerase IV subunit B<br>DNA gyrase subunit A<br>DNA gyrase subunit B<br>DNA gyrase subunit A<br>DNA gyrase subunit B<br>DNA topoisomerase IV subunit A<br>DNA gyrase subunit A<br>DNA gyrase subunit B<br>DNA gyrase subunit B<br>DNA gyrase subunit A<br>DNA gyrase subunit B<br>DNA topoisomerase IV subunit A<br>DNA gyrase subunit B		Unknown		Unknown		Unknown		Unknown			*Staphylococcus aureus (strain MSSA476)*	DNA topoisomerase 4 subunit A	18048152640; 180424181949	Unknown	
CI 990 (PD 131112)	DNA gyrase inhibitor<br>DNA topoisomerase IV inhibitor	0.414<br>0.122	0.001<br>0.003	*Escherichia coli*<br><br>*Mycobacterium tuberculosis*<br>*Staphylococcus aureus*	DNA gyrase subunit A<br>DNA gyrase subunit B<br>DNA gyrase subunit A<br>DNA topoisomerase 4 subunit A	3.59E-31<br>1.38E-18<br>4.09E-45<br>7.89E-08	0.41<br>0.41<br>0.44<br>0.41	*Escherichia coli*<br><br>*Escherichia coli K-12*<br><br>*Pseudomonas aeruginosa (strain ATCC 15692 / PAO1 / 1C / PRS 101 / LMG12228)*<br><br>*Mycobacterium tuberculosis*<br><br>*Staphylococcus aureus*<br>*Staphylococcus aureus subsp. aureus Mu50*<br><br>*Staphylococcus aureus subsp. aureus N315*	DNA gyrase subunit A<br>DNA gyrase subunit B<br>DNA gyrase subunit A<br>DNA gyrase subunit B<br>DNA gyrase subunit A<br>DNA gyrase subunit B<br>DNA gyrase subunit A<br>DNA gyrase subunit B<br>DNA topoisomerase IV subunit A<br>DNA gyrase subunit A<br>DNA gyrase subunit B<br>DNA gyrase subunit B		Unknown	*Escherichia coli K-12*<br>*Staphylococcus aureus subsp. aureus Mu50*<br>*Staphylococcus aureus*	DNA gyrase<br>DNA gyrase subunit B<br>Topoisomerase IV subunit A	0<br>0<br>0	Unknown		Unknown				Unknown	18048142608	Unknown	
Ciprofloxacin	DNA gyrase inhibitor<br>DNA topoisomerase IV inhibitor	0.488<br>0.239	0.001<br>0.002	*Escherichia coli*<br><br>*Mycobacterium tuberculosis*<br>*Staphylococcus aureus*	DNA gyrase subunit A<br>DNA gyrase subunit B<br>DNA gyrase subunit A<br>DNA topoisomerase 4 subunit A<br>DNA topoisomerase 4 subunit B	9.96E-65<br>2.23E-38<br>2.30E-50<br>6.66E-16<br>1.64E-12	1.00<br>1.00<br>0.51<br>1.00<br>1.00	*Bacillus subtilis (strain 168)*<br><br>*Escherichia coli*<br><br>*Escherichia coli K-12*<br><br><br>*Pseudomonas aeruginosa (strain ATCC 15692 / PAO1 / 1C / PRS 101 / LMG12228)*<br><br>*Mycobacterium tuberculosis*<br><br>*Staphylococcus aureus subsp. aureus Mu50*<br><br>*Staphylococcus aureus subsp. aureus N315*	DNA gyrase subunit A<br>DNA gyrase subunit B<br>DNA gyrase subunit A<br>DNA gyrase subunit B<br>DNA gyrase subunit A<br>DNA gyrase subunit B<br>DNA topoisomerase IV subunit A<br>DNA gyrase subunit A<br>DNA gyrase subunit B<br>DNA gyrase subunit A<br>DNA gyrase subunit B<br>DNA gyrase subunit A<br>DNA gyrase subunit B<br>DNA gyrase subunit B		Unknown	*Staphylococcus aureus subsp. aureus Mu50*	DNA gyrase	0	Unknown		Unknown				Unknown	180327120710	Unknown	
Clinafloxacin	DNA gyrase inhibitor<br>DNA topoisomerase IV inhibitor	0.722<br>0.446	0.000<br>0.001	*Escherichia coli*<br><br>*Mycobacterium tuberculosis*<br>*Staphylococcus aureus*	DNA gyrase subunit A<br>DNA gyrase subunit B<br>DNA gyrase subunit A<br>DNA topoisomerase 4 subunit A<br>DNA topoisomerase 4 subunit B	3.03E-35<br>5.44E-21<br>5.46E-73<br>5.27E-09<br>3.82E-06	0.46<br>0.46<br>0.56<br>0.49<br>0.46	*Bacillus subtilis (strain 168)*<br><br><br><br>*Escherichia coli*<br><br>*Escherichia coli K-12*<br><br><br>*Pseudomonas aeruginosa (strain ATCC 15692 / PAO1 / 1C / PRS 101 / LMG12228)*<br><br>*Mycobacterium tuberculosis*<br><br>*Staphylococcus aureus subsp. aureus Mu50*<br><br><br>*Staphylococcus aureus subsp. aureus N315*	DNA gyrase subunit A<br>DNA gyrase subunit B<br>DNA topoisomerase IV subunit A<br>DNA topoisomerase IV subunit B<br>DNA gyrase subunit A<br>DNA gyrase subunit B<br>DNA gyrase subunit A<br>DNA gyrase subunit B<br>DNA topoisomerase IV subunit A<br>DNA gyrase subunit A<br>DNA gyrase subunit B<br>DNA gyrase subunit A<br>DNA gyrase subunit B<br>DNA gyrase subunit A<br>DNA gyrase subunit B<br>DNA topoisomerase IV subunit A<br>DNA gyrase subunit B		Unknown	*Escherichia coli K-12*<br>*Staphylococcus aureus subsp. aureus Mu50*	DNA gyrase<br>DNA gyrase subunit B	0<br>0	Unknown		Unknown				Unknown	18048101306; 180424182040	Unknown	
CP 100964	DNA gyrase inhibitor<br>DNA topoisomerase IV inhibitor	0.311<br>0.066	0.002<br>0.003	*Escherichia coli*<br><br>*Mycobacterium tuberculosis*<br>*Staphylococcus aureus*	DNA gyrase subunit A<br>DNA gyrase subunit B<br>DNA gyrase subunit A<br>DNA topoisomerase 4 subunit A	2.00E-21<br>7.69E-13<br>5.42E-36<br>4.50E-06	0.33<br>0.33<br>0.36<br>0.31		Unknown		Unknown	*Escherichia coli K-12*<br>*Staphylococcus aureus*	DNA gyrase<br>Topoisomerase IV subunit A	0.01 to 0<br>0	Unknown		Unknown				Unknown	18048180424; 180424182119	DNA gyrase<br>DNA gyrase subunit A<br>DNA gyrase subunit B	Bioorg. Med. Chem. Lett.,(1998)8:1:97; Bioorg. Med. Chem. Lett.,(2008)18:3:1229; J. Med. Chem.,(1986)29:4:445; J. Med. Chem.,(1988)31:3:503; J. Med. Chem.,(1991)34:3:1142<br>Antimicrob. Agents Chemother.,(2010)54:8:3478<br>Bioorg. Med. Chem.,(2008)16:5:2558; J. Med. Chem.,(1992)35:25:4745
CP 104830	DNA gyrase inhibitor<br>DNA topoisomerase IV inhibitor	0.562<br>0.300	0.001<br>0.002	*Escherichia coli*<br><br>*Mycobacterium tuberculosis*<br>*Staphylococcus aureus*	DNA gyrase subunit A<br>DNA gyrase subunit B<br>DNA gyrase subunit A<br>DNA topoisomerase 4 subunit A<br>DNA topoisomerase 4 subunit B	3.79E-27<br>3.33E-16<br>8.12E-47<br>1.03E-08<br>1.07E-06	0.51<br>0.51<br>0.47<br>0.51<br>0.51	*Bacillus subtilis (strain 168)*<br><br><br><br>*Escherichia coli*<br><br>*Escherichia coli K-12*<br><br><br>*Pseudomonas aeruginosa (strain ATCC 15692 / PAO1 / 1C / PRS 101 / LMG12228)*<br><br>*Mycobacterium tuberculosis*<br>*Staphylococcus aureus subsp. aureus Mu50*<br><br><br>*Staphylococcus aureus subsp. aureus N315*	DNA gyrase subunit A<br>DNA gyrase subunit B<br>DNA topoisomerase IV subunit A<br>DNA topoisomerase IV subunit B<br>DNA gyrase subunit A<br>DNA gyrase subunit B<br>DNA gyrase subunit A<br>DNA gyrase subunit B<br>DNA topoisomerase IV subunit A<br>DNA gyrase subunit A<br>DNA gyrase subunit B<br>DNA gyrase subunit B<br>DNA gyrase subunit A<br>DNA gyrase subunit B<br>DNA topoisomerase IV subunit A<br>DNA gyrase subunit B		Unknown	*Escherichia coli K-12*<br>*Staphylococcus aureus subsp. aureus Mu50*	DNA gyrase<br>DNA gyrase subunit B	0<br>0	Unknown		Unknown				Unknown	18048204116; 180424182214	Unknown	
CP 105532 (PD 125275)	DNA gyrase inhibitor<br>DNA topoisomerase IV inhibitor	0.679<br>0.289	0.001<br>0.002	*Escherichia coli*<br><br>*Mycobacterium tuberculosis*<br>*Staphylococcus aureus*	DNA gyrase subunit A<br>DNA gyrase subunit B<br>DNA gyrase subunit A<br>DNA topoisomerase 4 subunit A<br>DNA topoisomerase 4 subunit B	2.16E-36<br>1.15E-21<br>1.34E-92<br>1.56E-10<br>4.39E-07	0.54<br>0.54<br>0.82<br>0.59<br>0.54	*Bacillus subtilis (strain 168)*<br><br><br><br>*Escherichia coli*<br><br>*Escherichia coli K-12*<br><br><br>*Pseudomonas aeruginosa (strain ATCC 15692 / PAO1 / 1C / PRS 101 / LMG12228)*<br><br>*Mycobacterium tuberculosis*<br><br>*Staphylococcus aureus*<br>*Staphylococcus aureus subsp. aureus Mu50*<br><br><br>*Staphylococcus aureus subsp. aureus N315*	DNA gyrase subunit A<br>DNA gyrase subunit B<br>DNA topoisomerase IV subunit A<br>DNA topoisomerase IV subunit B<br>DNA gyrase subunit A<br>DNA gyrase subunit B<br>DNA gyrase subunit A<br>DNA gyrase subunit B<br>DNA topoisomerase IV subunit A<br>DNA gyrase subunit A<br>DNA gyrase subunit B<br>DNA gyrase subunit A<br>DNA gyrase subunit B<br>DNA topoisomerase IV subunit A<br>DNA gyrase subunit A<br>DNA gyrase subunit B<br>DNA topoisomerase IV subunit A<br>DNA gyrase subunit B		Unknown	*Escherichia coli K-12*<br>*Staphylococcus aureus subsp. aureus Mu50*	DNA gyrase<br>DNA gyrase subunit B	0<br>0	Unknown		Unknown				Unknown	18048204153; 180424182306	Unknown	
CP 115953	DNA gyrase inhibitor<br>DNA topoisomerase IV inhibitor	0.344<br>0.168	0.001<br>0.002	*Escherichia coli*<br><br>*Mycobacterium tuberculosis*<br>*Staphylococcus aureus*	DNA gyrase subunit A<br>DNA gyrase subunit B<br>DNA gyrase subunit A<br>DNA topoisomerase 4 subunit A<br>DNA topoisomerase 4 subunit B	3.74E-38<br>1.05E-22<br>2.30E-50<br>1.13E-13<br>2.62E-06	0.48<br>0.48<br>0.51<br>0.48<br>0.48		Unknown		Unknown	*Escherichia coli K-12*<br>*Staphylococcus aureus subsp. aureus Mu50*<br><br>*Staphylococcus aureus*	DNA gyrase<br>DNA gyrase<br>DNA gyrase subunit B<br>Topoisomerase IV subunit A	0<br>0<br>0<br>0	Unknown		Unknown				Unknown	18048132724; 180424182351	Unknown	
CP 115955	DNA gyrase inhibitor<br>DNA topoisomerase IV inhibitor	0.420<br>0.217	0.001<br>0.002	*Escherichia coli*<br><br>*Mycobacterium tuberculosis*<br>*Staphylococcus aureus*	DNA gyrase subunit A<br>DNA gyrase subunit B<br>DNA gyrase subunit A<br>DNA topoisomerase 4 subunit A<br>DNA topoisomerase 4 subunit B	1.97E-52<br>3.99E-31<br>5.39E-47<br>1.86E-65<br>1.56E-07	0.58<br>0.58<br>0.46<br>0.58<br>0.58		Unknown		Unknown	*Escherichia coli K-12*<br>*Staphylococcus aureus subsp. aureus Mu50*<br><br>*Staphylococcus aureus*	DNA gyrase<br>DNA gyrase<br>DNA gyrase subunit B<br>Topoisomerase IV subunit A	0<br>0<br>0<br>0	Unknown		Unknown				Unknown	18048132801; 180425104324	DNA gyrase<br>DNA gyrase subunit B<br>Topoisomerase IV subunit A	J. Med. Chem.,(1986)29:3:394; J. Med. Chem.,(1986)29:4:445; J. Med. Chem.,(1991)34:3:1155; J. Med. Chem.,(1991)34:2:656; J. Med. Chem.,(1993)36:14:1964<br>J. Med. Chem.,(1992)35:25:4745<br>Bioorg. Med. Chem. Lett.,(2006)16:5:1272; Bioorg. Med. Chem. Lett.,(2006)16:5:1277; J. Med. Chem.,(2006)49:1:39; J. Med. Chem.,(2005)48:16:5232
CP 135803	DNA gyrase inhibitor<br>DNA topoisomerase IV inhibitor	0.701<br>0.283	0.000<br>0.002	*Escherichia coli*<br><br>*Mycobacterium tuberculosis*<br>*Staphylococcus aureus*	DNA gyrase subunit A<br>DNA gyrase subunit B<br>DNA gyrase subunit A<br>DNA topoisomerase 4 subunit A	3.06E-18<br>5.81E-11<br>4.02E-55<br>4.18E-08	0.42<br>0.42<br>0.61<br>0.43	*Bacillus subtilis (strain 168)*<br><br><br>*Escherichia coli*<br><br>*Escherichia coli K-12*<br><br><br>*Pseudomonas aeruginosa (strain ATCC 15692 / PAO1 / 1C / PRS 101 / LMG12228)*<br><br>*Mycobacterium tuberculosis*<br><br>*Staphylococcus aureus subsp. aureus Mu50*<br><br><br>*Staphylococcus aureus subsp. aureus N315*	DNA gyrase subunit A<br>DNA gyrase subunit B<br>DNA topoisomerase IV subunit A<br>DNA gyrase subunit A<br>DNA gyrase subunit B<br>DNA gyrase subunit A<br>DNA gyrase subunit B<br>DNA topoisomerase IV subunit A<br>DNA gyrase subunit A<br>DNA gyrase subunit B<br>DNA gyrase subunit A<br>DNA gyrase subunit B<br>DNA gyrase subunit A<br>DNA gyrase subunit B<br>DNA topoisomerase IV subunit A<br>DNA gyrase subunit B		Unknown	*Escherichia coli K-12*<br>*Staphylococcus aureus subsp. aureus Mu50*	DNA gyrase<br>DNA gyrase subunit B	0<br>0	Unknown		Unknown				Unknown	18048204246; 180425104410	Unknown	

Bioactive molecule	PASS online[4]			Similarity Ensemble Approach (SEA)[2]				ChemProt[3]		SuperPred[6]		Polypharmacology Browser (PPB)[1]			SPiDER[7]		HitPick[5]			PharmMapper[9]			TargetHunter[8]	
	Activity	Pa	Pi	Organism	Target	P-Value	Max TC	Organism	Target	Organism	Target	Organism	Target	P-Value	Target	Confidence level	Target	Precision (%)	Tc similarity	Organism	Target	Job Id	Target	Reference
CP 67015	DNA gyrase inhibitor<br>DNA topoisomerase IV inhibitor	0.148<br>0.048	0.003<br>0.004	*Escherichia coli*<br>*Mycobacterium tuberculosis*<br>*Staphylococcus aureus*	DNA gyrase subunit A<br>DNA gyrase subunit B<br>DNA gyrase subunit A<br>DNA topoisomerase 4 subunit A	1.67E-20<br>2.69E-12<br>2.27E-37<br>1.82E-06	0.32<br>0.32<br>0.36<br>0.35	*Escherichia coli K-12*	DNA gyrase subunit A<br>DNA gyrase subunit B		Unknown	*Escherichia coli K-12*<br>*Staphylococcus aureus subsp. aureus Mu50*<br>*Staphylococcus aureus*	DNA gyrase<br>DNA gyrase subunit B<br>Topoisomerase IV subunit A	0<br>0<br>0	Unknown		Unknown				Unknown	18048132604; 180425104502	Unknown	
CP 67804	DNA gyrase inhibitor<br>DNA topoisomerase IV inhibitor	0.148<br>0.039	0.003<br>0.004	*Escherichia coli*<br>*Mycobacterium tuberculosis*<br>*Staphylococcus aureus*	DNA gyrase subunit A<br>DNA gyrase subunit B<br>DNA gyrase subunit A<br>DNA topoisomerase 4 subunit A	6.67E-25<br>6.88E-15<br>5.82E-37<br>2.15E-06	0.32<br>0.32<br>0.36<br>0.33	*Escherichia coli K-12*	DNA gyrase subunit A<br>DNA gyrase subunit B		Unknown	*Escherichia coli K-12*<br>*Staphylococcus aureus subsp. aureus Mu50*<br>*Staphylococcus aureus*	DNA gyrase<br>DNA gyrase subunit B<br>Topoisomerase IV subunit A	0<br>0<br>0	Unknown		Unknown				Unknown	18048132650; 180425104541	DNA gyrase<br>DNA gyrase subunit B<br>Topoisomerase IV subunit A	J. Med. Chem.,(1986)29:3:394; J. Med. Chem.,(1986)29:4:445; J. Med. Chem.,(1991)34:3:1155; J. Med. Chem.,(1991)34:2:656; J. Med. Chem.,(1993)36:14:1964<br>J. Med. Chem.,(1992)35:25:4745<br>Bioorg. Med. Chem. Lett.,(2004)14:20:5193; Bioorg. Med. Chem. Lett.,(2006)16:5:1272; Bioorg. Med. Chem. Lett.,(2006)16:5:1277; J. Med. Chem.,(2006)49:1:39; J. Med. Chem.,(2005)48:16:5232
CP 74667	DNA gyrase inhibitor<br>DNA topoisomerase IV inhibitor	0.365<br>0.213	0.001<br>0.002	*Escherichia coli*<br>*Mycobacterium tuberculosis*<br>*Staphylococcus aureus*	DNA gyrase subunit A<br>DNA gyrase subunit B<br>DNA gyrase subunit A<br>DNA topoisomerase 4 subunit A<br>DNA topoisomerase 4 subunit B	4.07E-59<br>4.55E-35<br>1.75E-48<br>9.19E-11<br>3.64E-09	0.72<br>0.72<br>0.48<br>0.72<br>0.72	*Escherichia coli K-12*<br>*Pseudomonas aeruginosa (strain ATCC 15692 / PAO1 / 1C / PRS 101 / LMG12228)*<br>*Staphylococcus aureus subsp. aureus Mu50*<br>*Staphylococcus aureus subsp. aureus N315*	DNA gyrase subunit A<br>DNA gyrase subunit B<br>DNA gyrase subunit A<br>DNA gyrase subunit B<br>DNA gyrase subunit A<br>DNA gyrase subunit B<br>DNA topoisomerase IV subunit A<br>DNA gyrase subunit B		Unknown	*Escherichia coli K-12*<br>*Staphylococcus aureus subsp. aureus Mu50*	DNA gyrase<br>DNA gyrase<br>DNA gyrase subunit B	0<br>0.01 to 0<br>0	Unknown		Unknown			*Staphylococcus aureus (strain MSSA476)*	DNA topoisomerase 4 subunit A	18048180211; 180425104722	Unknown	
CP 92121	DNA gyrase inhibitor<br>DNA topoisomerase IV inhibitor	0.361<br>0.106	0.001<br>0.003	*Escherichia coli*<br>*Mycobacterium tuberculosis*<br>*Staphylococcus aureus*	DNA gyrase subunit A<br>DNA gyrase subunit B<br>DNA gyrase subunit A<br>DNA topoisomerase 4 subunit A	4.48E-26<br>1.44E-15<br>1.50E-40<br>2.47E-09	0.35<br>0.35<br>0.41<br>0.36		Unknown		Unknown	*Escherichia coli K-12*<br>*Staphylococcus aureus*	DNA gyrase<br>Topoisomerase IV subunit A	0<br>0	Unknown		Unknown				Unknown	18048180328; 180425104842	DNA gyrase<br>DNA gyrase subunit A<br>DNA gyrase subunit B<br>Topoisomerase IV subunit A	Bioorg. Med. Chem. Lett.,(1998)8:1:97; Bioorg. Med. Chem. Lett.,(2008)18:3:1229; J. Med. Chem.,(1986)29:4:445; J. Med. Chem.,(1988)31:3:503; J. Med. Chem.,(1991)34:3:1142<br>Antimicrob. Agents Chemother.,(2010)54:8:3478<br>Bioorg. Med. Chem.,(2008)16:5:2558; J. Med. Chem.,(1992)35:25:4745<br>Bioorg. Med. Chem. Lett.,(2006)16:5:1272
CP 99433	DNA gyrase inhibitor<br>DNA topoisomerase IV inhibitor	0.528<br>0.309	0.001<br>0.002	*Escherichia coli*<br>*Mycobacterium tuberculosis*<br>*Staphylococcus aureus*	DNA gyrase subunit A<br>DNA gyrase subunit B<br>DNA gyrase subunit A<br>DNA topoisomerase 4 subunit A<br>DNA topoisomerase 4 subunit B	3.57E-36<br>1.54E-21<br>1.33E-48<br>1.33E-08<br>3.14E-06	0.47<br>0.47<br>0.48<br>0.47<br>0.47	*Escherichia coli*<br>*Pseudomonas aeruginosa (strain ATCC 15692 / PAO1 / 1C / PRS 101 / LMG12228)*<br>*Staphylococcus aureus subsp. aureus Mu50*	DNA gyrase subunit A<br>DNA gyrase subunit B<br>DNA gyrase subunit A<br>DNA gyrase subunit B<br>DNA gyrase subunit A<br>DNA gyrase subunit B<br>DNA topoisomerase IV subunit A		Unknown	*Escherichia coli K-12*<br>*Staphylococcus aureus subsp. aureus Mu50*	DNA gyrase<br>DNA gyrase<br>DNA gyrase subunit B	0<br>0<br>0	Unknown		Unknown				Unknown	18048180356; 180425104910	Unknown	
Danofloxacin	DNA gyrase inhibitor<br>DNA topoisomerase IV inhibitor	0.284<br>0.160	0.002<br>0.002	*Escherichia coli*<br>*Mycobacterium tuberculosis*<br>*Staphylococcus aureus*	DNA gyrase subunit A<br>DNA gyrase subunit B<br>DNA gyrase subunit A<br>DNA topoisomerase 4 subunit A<br>DNA topoisomerase 4 subunit B	1.36E-53<br>8.24E-32<br>1.61E-46<br>9.10E-10<br>5.01E-08	0.62<br>0.62<br>0.46<br>0.62<br>0.62	*Escherichia coli K-12*<br>*Pseudomonas aeruginosa (strain ATCC 15692 / PAO1 / 1C / PRS 101 / LMG12228)*<br>*Staphylococcus aureus subsp. aureus Mu50*<br>*Staphylococcus aureus subsp. aureus N315*	DNA gyrase subunit A<br>DNA gyrase subunit B<br>DNA gyrase subunit A<br>DNA gyrase subunit B<br>DNA gyrase subunit A<br>DNA gyrase subunit B<br>DNA gyrase subunit B		Unknown	*Escherichia coli K-12*<br>*Staphylococcus aureus subsp. aureus Mu50*	DNA gyrase<br>DNA gyrase<br>DNA gyrase subunit B	0<br>0<br>0	Unknown		Unknown			*Staphylococcus aureus (strain MSSA476)*	DNA topoisomerase 4 subunit A	18048101351; 180425104936	Unknown	
DC 159a free base	DNA gyrase inhibitor<br>DNA topoisomerase IV inhibitor	0.369<br>0.170	0.001<br>0.002	*Escherichia coli*<br>*Mycobacterium tuberculosis*<br>*Staphylococcus aureus*	DNA gyrase subunit A<br>DNA gyrase subunit A<br>DNA topoisomerase 4 subunit A	4.58E-09<br>1.74E-69<br>8.20E-08	0.34<br>0.52<br>0.48	*Escherichia coli*<br>*Escherichia coli K-12*<br>*Pseudomonas aeruginosa (strain ATCC 15692 / PAO1 / 1C / PRS 101 / LMG12228)*<br>*Mycobacterium tuberculosis*<br>*Staphylococcus aureus subsp. aureus Mu50*<br>*Staphylococcus aureus subsp. aureus N315*	DNA gyrase subunit A<br>DNA gyrase subunit B<br>DNA gyrase subunit A<br>DNA gyrase subunit B<br>DNA gyrase subunit A<br>DNA gyrase subunit B<br>DNA gyrase subunit B<br>DNA gyrase subunit A<br>DNA gyrase subunit B<br>DNA topoisomerase IV subunit A<br>DNA gyrase subunit B		Unknown	*Staphylococcus aureus subsp. aureus Mu50*	DNA gyrase<br>DNA gyrase subunit B	0.01 to 0<br>0	Unknown		Unknown				Unknown	18048180502; 180425105028	Unknown	
Delafloxacin	DNA gyrase inhibitor<br>DNA topoisomerase IV inhibitor	0.305<br>0.408	0.002<br>0.001	*Escherichia coli*<br>*Mycobacterium tuberculosis*<br>*Staphylococcus aureus*	DNA gyrase subunit A<br>DNA gyrase subunit A<br>DNA topoisomerase 4 subunit A	9.04E-09<br>6.23E-44<br>5.63E-07	0.32<br>0.42<br>0.41		Unknown		Unknown	*Escherichia coli K-12*<br>*Staphylococcus aureus subsp. aureus Mu50*	DNA gyrase<br>DNA gyrase<br>DNA gyrase subunit B	0<br>0.01 to 0<br>0	Unknown		Unknown			*Staphylococcus aureus (strain MSSA476)*	DNA topoisomerase 4 subunit A	18048103721; 180425105052	DNA gyrase<br>DNA gyrase subunit B	J. Med. Chem.,(1988)31:5:991; J. Med. Chem.,(1991)34:3:1142; J. Med. Chem.,(1991)34:3:1155; J. Med. Chem.,(2003)46:17:3655<br>J. Med. Chem.,(1992)35:25:4745
Desfluorociprofloxacin (SQ 4004)	DNA gyrase inhibitor<br>DNA topoisomerase IV inhibitor	0.353<br>0.152	0.001<br>0.002	*Escherichia coli*<br>*Mycobacterium tuberculosis*<br>*Staphylococcus aureus*	DNA gyrase subunit A<br>DNA gyrase subunit B<br>DNA gyrase subunit A<br>DNA topoisomerase 4 subunit A<br>DNA topoisomerase 4 subunit B	5.51E-39<br>3.39E-23<br>4.91E-43<br>9.67E-10<br>1.70E-08	0.66<br>0.66<br>0.43<br>0.66<br>0.66	*Bacillus subtilis (strain 168)*<br>*Escherichia coli K-12*<br>*Pseudomonas aeruginosa (strain ATCC 15692 / PAO1 / 1C / PRS 101 / LMG12228)*<br>*Staphylococcus aureus subsp. aureus Mu50*<br>*Staphylococcus aureus subsp. aureus N315*	DNA gyrase subunit A<br>DNA gyrase subunit B<br>DNA topoisomerase IV subunit A<br>DNA topoisomerase IV subunit B<br>DNA gyrase subunit A<br>DNA gyrase subunit B<br>DNA topoisomerase IV subunit A<br>DNA gyrase subunit A<br>DNA gyrase subunit B<br>DNA gyrase subunit A<br>DNA gyrase subunit B<br>DNA gyrase subunit B		Unknown	*Escherichia coli K-12*<br>*Staphylococcus aureus subsp. aureus Mu50*	DNA gyrase<br>DNA gyrase	0<br>0	Unknown		Unknown				Unknown	18048195949; 180425105128	DNA gyrase<br>DNA gyrase subunit A<br>DNA gyrase subunit B<br>Topoisomerase IV subunit A	J. Med. Chem.,(1986)29:3:394; J. Med. Chem.,(1988)31:3:503; J. Med. Chem.,(1988)31:5:991; J. Med. Chem.,(1993)36:14:1964; J. Med. Chem.,(1996)39:25:4952<br>Antimicrob. Agents Chemother.,(2010)54:8:3478<br>Bioorg. Med. Chem.,(2008)16:5:2558; J. Med. Chem.,(1992)35:25:4745<br>Bioorg. Med. Chem. Lett.,(2004)14:20:5193; Bioorg. Med. Chem. Lett.,(2006)16:5:1272; Bioorg. Med. Chem. Lett.,(2006)16:5:1277; J. Med. Chem.,(2005)48:16:5232; J. Med. Chem.,(2006)49:1:39
Difloxacin	DNA gyrase inhibitor<br>DNA topoisomerase IV inhibitor	0.189<br>0.108	0.002<br>0.003	*Escherichia coli*<br>*Mycobacterium tuberculosis*<br>*Staphylococcus aureus*	DNA gyrase subunit A<br>DNA gyrase subunit B<br>DNA gyrase subunit A<br>DNA topoisomerase 4 subunit A<br>DNA topoisomerase 4 subunit B	6.00E-55<br>1.31E-32<br>3.12E-34<br>8.51E-08<br>1.07E-06	0.58<br>0.58<br>0.33<br>0.51<br>0.51	*Escherichia coli*<br>*Escherichia coli K-12*<br>*Pseudomonas aeruginosa (strain ATCC 15692 / PAO1 / 1C / PRS 101 / LMG12228)*<br>*Mycobacterium smegmatis*<br>*Mycobacterium tuberculosis*<br>*Staphylococcus aureus*<br>*Staphylococcus aureus subsp. aureus Mu50*<br>*Staphylococcus aureus subsp. aureus N315*	DNA gyrase subunit A<br>DNA gyrase subunit B<br>DNA gyrase subunit A<br>DNA gyrase subunit B<br>DNA gyrase subunit A<br>DNA gyrase subunit B<br>DNA topoisomerase (ATP-hydrolysing) (DNA gyrase)<br>DNA gyrase subunit A<br>DNA gyrase subunit B<br>DNA topoisomerase IV subunit A<br>DNA gyrase subunit A<br>DNA gyrase subunit B<br>DNA gyrase subunit B		Unknown	*Escherichia coli K-12*<br>*Staphylococcus aureus subsp. aureus Mu50*	DNA gyrase<br>DNA gyrase<br>DNA gyrase subunit B	0<br>0<br>0	Unknown		Unknown			*Staphylococcus aureus (strain MSSA476)*	DNA topoisomerase 4 subunit A	18048103804; 180425130656	Unknown	
DJ 6783	DNA gyrase inhibitor<br>DNA topoisomerase IV inhibitor	0.028<br>0.026	0.027<br>0.009		Unknown			*Bacillus subtilis (strain 168)*<br>*Escherichia coli*<br>*Escherichia coli K-12*<br>*Mycobacterium tuberculosis*	DNA gyrase subunit A<br>DNA gyrase subunit B<br>DNA topoisomerase IV subunit A<br>DNA gyrase subunit A<br>DNA gyrase subunit B<br>DNA gyrase subunit A<br>DNA gyrase subunit B<br>DNA gyrase subunit B		Unknown	*Escherichia coli K-12*	DNA gyrase	0	Unknown		Unknown			*Staphylococcus aureus (strain MSSA476)*	DNA topoisomerase 4 subunit A	18048152712; 180425130723	DNA gyrase	Bioorg. Med. Chem. Lett.,(1992)2:7:643; Bioorg. Med. Chem. Lett.,(1993)3:2:225; Bioorg. Med. Chem. Lett.,(1998)8:1:97; J. Med. Chem.,(1986)29:4:445; J. Med. Chem.,(1986)29:3:394
DK 507k	DNA gyrase inhibitor<br>DNA topoisomerase IV inhibitor	0.677<br>0.213	0.001<br>0.002	*Escherichia coli*<br>*Mycobacterium tuberculosis*<br>*Staphylococcus aureus*	DNA gyrase subunit A<br>DNA gyrase subunit B<br>DNA gyrase subunit A<br>DNA topoisomerase 4 subunit A	5.41E-13<br>7.23E-08<br>1.90E-71<br>6.94E-08	0.33<br>0.33<br>0.53<br>0.49	*Escherichia coli*<br>*Escherichia coli K-12*<br>*Pseudomonas aeruginosa (strain ATCC 15692 / PAO1 / 1C / PRS 101 / LMG12228)*<br>*Mycobacterium tuberculosis*<br>*Staphylococcus aureus subsp. aureus Mu50*<br>*Staphylococcus aureus subsp. aureus N315*	DNA gyrase subunit A<br>DNA gyrase subunit B<br>DNA gyrase subunit A<br>DNA gyrase subunit B<br>DNA gyrase subunit A<br>DNA gyrase subunit B<br>DNA gyrase subunit B<br>DNA gyrase subunit A<br>DNA gyrase subunit B<br>DNA topoisomerase IV subunit A<br>DNA gyrase subunit B		Unknown	*Staphylococcus aureus subsp. aureus Mu50*<br>*Staphylococcus aureus subsp. aureus Mu50*	DNA gyrase<br>DNA gyrase subunit B	0.01 to 0<br>0	Unknown		Unknown				Unknown	18048180549; 180425130753	Unknown	
DN 9494	DNA gyrase inhibitor<br>DNA topoisomerase IV inhibitor	0.109<br>0.056	0.003<br>0.003	*Escherichia coli*<br>*Mycobacterium tuberculosis*<br>*Staphylococcus aureus*	DNA gyrase subunit A<br>DNA gyrase subunit B<br>DNA gyrase subunit A<br>DNA topoisomerase 4 subunit A	2.86E-27<br>2.22E-16<br>4.44E-40<br>8.90E-07	0.35<br>0.35<br>0.39<br>0.36	*Escherichia coli*<br>*Escherichia coli K-12*<br>*Mycobacterium smegmatis*<br>*Mycobacterium tuberculosis*<br>*Staphylococcus aureus subsp. aureus Mu50*<br>*Staphylococcus aureus subsp. aureus N315*	DNA gyrase subunit A<br>DNA gyrase subunit B<br>DNA gyrase subunit A<br>DNA gyrase subunit B<br>DNA topoisomerase (ATP-hydrolysing) (Dna gyrase)<br>DNA gyrase subunit A<br>DNA gyrase subunit A<br>DNA gyrase subunit B<br>DNA gyrase subunit B		Unknown		Unknown		Unknown		Unknown				Unknown	18048161042; 180425130833	Unknown	
Droxacin	DNA gyrase inhibitor<br>DNA topoisomerase IV inhibitor	0.068<br>0.032	0.004<br>0.005	*Escherichia coli*	DNA gyrase subunit A<br>DNA gyrase subunit B	6.42E-13<br>7.99E-08	0.32<br>0.32	*Escherichia coli K-12*	DNA gyrase subunit A<br>DNA gyrase subunit B		Unknown	*Escherichia coli K-12*	DNA gyrase	0	Unknown		Unknown				Unknown	18048123933; 180425130901	DNA gyrase	J. Med. Chem.,(1986)29:3:394; J. Med. Chem.,(1986)29:4:445; J. Med. Chem.,(1993)36:14:1964

Bioactive molecule	PASS online[4]			Similarity Ensemble Approach (SEA)[2]				ChemProt[3]		SuperPred[6]		Polypharmacology Browser (PPB)[1]			SPiDER[7]		HitPick[5]			PharmMapper[9]			TargetHunter[8]	
	Activity	Pa	Pi	Organism	Target	P-Value	Max TC	Organism	Target	Organism	Target	Organism	Target	P-Value	Target	Confidence level	Target	Precision (%)	Tc similarity	Organism	Target	Job Id	Target	Reference
DS 8587 free base	DNA gyrase inhibitor	0.153	0.003	*Mycobacterium tuberculosis*	DNA gyrase subunit A	4.22E-40	0.38	*Bacillus subtilis (strain 168)*	DNA gyrase subunit A		Unknown	*Escherichia coli K-12*	DNA gyrase	0	Unknown		Unknown			*Staphylococcus aureus (strain MSSA476)*	DNA topoisomerase 4 subunit A	18048142656	Unknown	
	DNA topoisomerase IV inhibitor	0.072	0.003	*Staphylococcus aureus*	DNA topoisomerase 4 subunit A	8.07E-07	0.37		DNA gyrase subunit B			*Staphylococcus aureus subsp. aureus Mu50*	DNA gyrase	0.01 to 0										
									DNA topoisomerase IV subunit A				DNA gyrase subunit B	0										
									DNA topoisomerase IV subunit B															
								Escherichia coli	DNA gyrase subunit A															
									DNA gyrase subunit B															
								Escherichia coli K-12	DNA gyrase subunit A															
									DNA gyrase subunit B															
									DNA topoisomerase IV subunit A															
								Pseudomonas aeruginosa (strain ATCC 15692 / PAO1 / 1C / PRS 101 / LMG12228)	DNA gyrase subunit A															
									DNA gyrase subunit B															
								Mycobacterium tuberculosis	DNA gyrase subunit A															
									DNA gyrase subunit B															
								Staphylococcus aureus	DNA topoisomerase IV subunit A															
								Staphylococcus aureus subsp. aureus Mu50	DNA gyrase subunit A															
									DNA gyrase subunit B															
									DNA topoisomerase IV subunit A															
								Staphylococcus aureus subsp. aureus N315	DNA gyrase subunit B															
DU 6611	DNA gyrase inhibitor	0.639	0.001	*Escherichia coli*	DNA gyrase subunit A	1.38E-07	0.39	*Bacillus subtilis (strain 168)*	DNA gyrase subunit A		Unknown	*Staphylococcus aureus subsp. aureus Mu50*	DNA gyrase	0	Unknown		Unknown				Unknown	18048204322; 180425130933	Unknown	
	DNA topoisomerase IV inhibitor	0.354	0.002		DNA gyrase subunit B	4.58E-10	0.39		DNA gyrase subunit B			*Staphylococcus aureus subsp. aureus Mu50*	DNA gyrase subunit B	0										
				Mycobacterium tuberculosis	DNA gyrase subunit A	1.11E-56	0.46		DNA topoisomerase IV subunit A															
				Staphylococcus aureus	DNA gyrase subunit B	1.47E-07	0.49		DNA topoisomerase IV subunit B															
					DNA topoisomerase 4 subunit A	3.46E-14	0.46	*Escherichia coli*	DNA gyrase subunit A															
									DNA gyrase subunit B															
								Escherichia coli K-12	DNA gyrase subunit A															
									DNA gyrase subunit B															
									DNA topoisomerase IV subunit A															
								Pseudomonas aeruginosa (strain ATCC 15692 / PAO1 / 1C / PRS 101 / LMG12228)	DNA gyrase subunit A															
									DNA gyrase subunit B															
								Mycobacterium tuberculosis	DNA gyrase subunit A															
									DNA gyrase subunit B															
								Staphylococcus aureus subsp. aureus Mu50	DNA gyrase subunit A															
									DNA gyrase subunit B															
									DNA topoisomerase IV subunit A															
								Staphylococcus aureus subsp. aureus N315	DNA gyrase subunit B															
DU 6668	DNA gyrase inhibitor	0.728	0.000	*Escherichia coli*	DNA gyrase subunit A	7.27E-34	0.43	*Bacillus subtilis (strain 168)*	DNA gyrase subunit A		Unknown	*Escherichia coli K-12*	DNA gyrase	0	Unknown		Unknown				Unknown	18048182131; 180425131001	Unknown	
	DNA topoisomerase IV inhibitor	0.356	0.002		DNA gyrase subunit B	3.55E-20	0.43		DNA gyrase subunit B			*Staphylococcus aureus subsp. aureus Mu50*	DNA gyrase	0										
				Mycobacterium tuberculosis	DNA gyrase subunit A	1.24E-65	0.50		DNA topoisomerase IV subunit A				DNA gyrase subunit B	0										
				Staphylococcus aureus	DNA topoisomerase 4 subunit A	1.94E-08	0.46		DNA topoisomerase IV subunit B															
					DNA topoisomerase 4 subunit B	9.38E-06	0.43	*Escherichia coli*	DNA gyrase subunit A															
									DNA gyrase subunit B															
								Escherichia coli K-12	DNA gyrase subunit A															
									DNA gyrase subunit B															
									DNA topoisomerase IV subunit A															
								Pseudomonas aeruginosa (strain ATCC 15692 / PAO1 / 1C / PRS 101 / LMG12228)	DNA gyrase subunit A															
									DNA gyrase subunit B															
								Mycobacterium tuberculosis	DNA gyrase subunit A															
									DNA gyrase subunit B															
								Staphylococcus aureus subsp. aureus Mu50	DNA gyrase subunit A															
									DNA gyrase subunit B															
									DNA topoisomerase IV subunit A															
								Staphylococcus aureus subsp. aureus N315	DNA gyrase subunit B															
DV 7751a (DV 7751)	DNA gyrase inhibitor	0.753	0.000	*Escherichia coli*	DNA gyrase subunit A	9.35E-21	0.33	*Escherichia coli*	DNA gyrase subunit A		Unknown	*Escherichia coli K-12*	DNA gyrase	0	Unknown		Unknown				Unknown	18048204350; 180425131034	Unknown	
	DNA topoisomerase IV inhibitor	0.121	0.003		DNA gyrase subunit B	1.91E-12	0.33		DNA gyrase subunit B			*Staphylococcus aureus subsp. aureus Mu50*	DNA gyrase	0										
				Mycobacterium tuberculosis	DNA gyrase subunit A	2.96E-42	0.41	*Escherichia coli K-12*	DNA gyrase subunit A															
				Staphylococcus aureus	DNA topoisomerase 4 subunit A	6.95E-07	0.38		DNA gyrase subunit B															
								Mycobacterium tuberculosis	DNA gyrase subunit A															
									DNA gyrase subunit B															
								Staphylococcus aureus	DNA topoisomerase IV subunit A															
								Staphylococcus aureus subsp. aureus Mu50	DNA gyrase subunit B															
									DNA topoisomerase IV subunit A															
DW 8186	DNA gyrase inhibitor	0.636	0.001	*Escherichia coli*	DNA gyrase subunit A	7.82E-46	0.50	*Bacillus subtilis (strain 168)*	DNA gyrase subunit A		Unknown	*Escherichia coli K-12*	DNA gyrase	0	Unknown		Unknown				Unknown	18048204423; 180425131110	Unknown	
	DNA topoisomerase IV inhibitor	0.102	0.003		DNA gyrase subunit B	3.10E-27	0.50		DNA gyrase subunit B			*Staphylococcus aureus subsp. aureus Mu50*	DNA gyrase	0										
				Mycobacterium tuberculosis	DNA gyrase subunit A	1.00E-34	0.34		DNA topoisomerase IV subunit A															
				Staphylococcus aureus	DNA topoisomerase 4 subunit A	9.03E-08	0.50		DNA topoisomerase IV subunit B															
					DNA topoisomerase 4 subunit B	1.34E-06	0.50	*Escherichia coli*	DNA gyrase subunit A															
									DNA gyrase subunit B															
								Escherichia coli K-12	DNA gyrase subunit A															
									DNA gyrase subunit B															
									DNA topoisomerase IV subunit A															
								Pseudomonas aeruginosa (strain ATCC 15692 / PAO1 / 1C / PRS 101 / LMG12228)	DNA gyrase subunit A															
									DNA gyrase subunit B															
								Mycobacterium tuberculosis	DNA gyrase subunit A															
									DNA gyrase subunit B															
								Staphylococcus aureus subsp. aureus Mu50	DNA gyrase subunit A															
									DNA gyrase subunit B															
									DNA topoisomerase IV subunit A															
								Staphylococcus aureus subsp. aureus N315	DNA gyrase subunit B															
DX 619	DNA gyrase inhibitor	0.261	0.002	*Escherichia coli*	DNA gyrase subunit A	2.22E-16	0.30	*Escherichia coli*	DNA gyrase subunit A		Unknown	*Escherichia coli K-12*	DNA gyrase	0	Unknown		Unknown			*Staphylococcus aureus (strain MSSA476)*	DNA topoisomerase 4 subunit A	18048182712; 180425131135	DNA gyrase	Bioorg. Med. Chem. Lett.,(1992)2:7:643; Bioorg. Med. Chem. Lett.,(2006)16:5:1272; J. Med. Chem.,(1993)36:7:871; J. Med. Chem.,(2003)46:17:3655; J. Med. Chem.,(2006)49:22:6435
	DNA topoisomerase IV inhibitor	0.100	0.003		DNA gyrase subunit B	8.00E-10	0.30		DNA gyrase subunit B			*Staphylococcus aureus subsp. aureus Mu50*	DNA gyrase	0.01 to 0									DNA gyrase subunit A	Antimicrob. Agents Chemother.,(2010)54:8:3478
				Mycobacterium tuberculosis	DNA gyrase subunit A	9.58E-40	0.39	*Escherichia coli K-12*	DNA gyrase subunit A				DNA gyrase subunit B	0									DNA gyrase subunit B	Bioorg. Med. Chem.,(2008)16:5:2558; Bioorg. Med. Chem.,(2014)22:17:4924; Bioorg. Med. Chem.,(2015)23:9:2062; J. Med. Chem.,(1992)35:25:4745
				Staphylococcus aureus	DNA topoisomerase 4 subunit A	1.85E-06	0.36		DNA gyrase subunit B														Topoisomerase IV subunit A	Bioorg. Med. Chem. Lett.,(2004)14:20:5193; Bioorg. Med. Chem. Lett.,(2006)16:5:1272; Bioorg. Med. Chem. Lett.,(2006)16:5:1277
								Pseudomonas aeruginosa (strain ATCC 15692 / PAO1 / 1C / PRS 101 / LMG12228)	DNA gyrase subunit A															
									DNA gyrase subunit B															
								Mycobacterium tuberculosis	DNA gyrase subunit B															
								Staphylococcus aureus subsp. aureus Mu50	DNA gyrase subunit A															
									DNA gyrase subunit B															
									DNA topoisomerase IV subunit A															
								Staphylococcus aureus subsp. aureus N315	DNA gyrase subunit B															
E 3604	DNA gyrase inhibitor	0.074	0.004	*Escherichia coli*	DNA gyrase subunit A	6.04E-43	0.43	*Escherichia coli K-12*	DNA gyrase subunit A		Unknown	*Escherichia coli K-12*	DNA gyrase	0	Unknown		Unknown			*Staphylococcus aureus (strain MSSA476)*	DNA topoisomerase 4 subunit A	18048182739; 180425131207	Unknown	
	DNA topoisomerase IV inhibitor	0.064	0.003		DNA gyrase subunit B	1.57E-25	0.43		DNA gyrase subunit B			*Staphylococcus aureus subsp. aureus Mu50*	DNA gyrase subunit B	0										
				Mycobacterium tuberculosis	DNA gyrase subunit A	1.33E-15	0.28	*Staphylococcus aureus subsp. aureus Mu50*	DNA gyrase subunit B															
E 3846	DNA gyrase inhibitor	0.409	0.001	*Escherichia coli*	DNA gyrase subunit A	2.03E-57	0.69	*Escherichia coli K-12*	DNA gyrase subunit A		Unknown	*Escherichia coli K-12*	DNA gyrase	0	Unknown		Unknown				Unknown	18048182807; 180425142938	Unknown	
	DNA topoisomerase IV inhibitor	0.300	0.002		DNA gyrase subunit B	4.57E-34	0.69		DNA gyrase subunit B			*Staphylococcus aureus subsp. aureus Mu50*	DNA gyrase	0										
				Mycobacterium tuberculosis	DNA gyrase subunit A	5.12E-44	0.43	*Pseudomonas aeruginosa (strain ATCC 15692 / PAO1 / 1C / PRS 101 / LMG12228)*	DNA gyrase subunit A				DNA gyrase subunit B	0										
				Staphylococcus aureus	DNA topoisomerase 4 subunit A	7.46E-13	0.69		DNA gyrase subunit B			*Staphylococcus aureus*	Topoisomerase IV subunit A	0										
					DNA topoisomerase 4 subunit B	7.13E-09	0.69	*Staphylococcus aureus subsp. aureus Mu50*	DNA gyrase subunit A															
									DNA gyrase subunit B															
								Staphylococcus aureus subsp. aureus N315	DNA gyrase subunit B															
E 4441	DNA gyrase inhibitor	0.368	0.001	*Escherichia coli*	DNA gyrase subunit A	3.11E-35	0.48	*Bacillus subtilis (strain 168)*	DNA gyrase subunit A		Unknown	*Escherichia coli K-12*	DNA gyrase	0	Unknown		Unknown				Unknown	18048182839; 180425143014	Unknown	
	DNA topoisomerase IV inhibitor	0.214	0.002		DNA gyrase subunit B	5.53E-21	0.48		DNA gyrase subunit B			*Staphylococcus aureus subsp. aureus Mu50*	DNA gyrase	0.01 to 0										
				Mycobacterium tuberculosis	DNA gyrase subunit A	6.30E-73	0.55		DNA topoisomerase IV subunit A				DNA gyrase subunit B	0										
				Staphylococcus aureus	DNA topoisomerase 4 subunit A	2.96E-09	0.51		DNA topoisomerase IV subunit B															
					DNA topoisomerase 4 subunit B	2.57E-06	0.48	*Escherichia coli K-12*	DNA gyrase subunit A															
									DNA gyrase subunit B															
								Pseudomonas aeruginosa (strain ATCC 15692 / PAO1 / 1C / PRS 101 / LMG12228)	DNA gyrase subunit A															
									DNA gyrase subunit B															
								Staphylococcus aureus subsp. aureus Mu50	DNA gyrase subunit A															
									DNA gyrase subunit B															
								Staphylococcus aureus subsp. aureus N315	DNA gyrase subunit B															
E 4474	DNA gyrase inhibitor	0.591	0.001	*Escherichia coli*	DNA gyrase subunit A	5.96E-37	0.49	*Bacillus subtilis (strain 168)*	DNA gyrase subunit A		Unknown	*Escherichia coli K-12*	DNA gyrase	0	Unknown		Unknown				Unknown	18048182920; 180425143045	Unknown	
	DNA topoisomerase IV inhibitor	0.340	0.002		DNA gyrase subunit B	5.37E-22	0.49		DNA gyrase subunit B			*Staphylococcus aureus subsp. aureus Mu50*	DNA gyrase	0										
				Mycobacterium tuberculosis	DNA gyrase subunit A	1.27E-79	0.60		DNA topoisomerase IV subunit A				DNA gyrase subunit B	0										
				Staphylococcus aureus	DNA topoisomerase 4 subunit A	6.43E-10	0.57		DNA topoisomerase IV subunit B															
					DNA topoisomerase 4 subunit B	1.68E-06	0.49	*Escherichia coli K-12*	DNA gyrase subunit A															
									DNA gyrase subunit B															
									DNA topoisomerase IV subunit A															
								Pseudomonas aeruginosa (strain ATCC 15692 / PAO1 / 1C / PRS 101 / LMG12228)	DNA gyrase subunit A															
									DNA gyrase subunit B															
								Staphylococcus aureus subsp. aureus Mu50	DNA gyrase subunit A															
									DNA gyrase subunit B															
									DNA topoisomerase IV subunit A															
								Staphylococcus aureus subsp. aureus N315	DNA gyrase subunit B															

Bioactive molecule	PASS online[4]			Similarity Ensemble Approach (SEA)[2]				ChemProt[3]		SuperPred[6]		Polypharmacology Browser (PPB)[1]			SPiDER[7]		HitPick[5]			PharmMapper[9]			TargetHunter[8]	
	Activity	Pa	Pi	Organism	Target	P-Value	Max TC	Organism	Target	Organism	Target	Organism	Target	P-Value	Target	Confidence level	Target	Precision (%)	Tc similarity	Organism	Target	Job Id	Target	Reference
E 4480	DNA gyrase inhibitor	0.361	0.001	*Escherichia coli*	DNA gyrase subunit A	2.09E-56	0.69	*Bacillus subtilis (strain 168)*	DNA gyrase subunit A		Unknown	*Escherichia coli K-12*	DNA gyrase	0	Unknown		Unknown				Unknown	18048182942; 180425143118	Unknown	
	DNA topoisomerase IV inhibitor	0.174	0.002		DNA gyrase subunit B	1.81E-33	0.69		DNA gyrase subunit B			*Staphylococcus aureus subsp. aureus Mu50*	DNA gyrase	0										
				Mycobacterium tuberculosis	DNA gyrase subunit A	4.31E-45	0.44		DNA topoisomerase IV subunit A				DNA gyrase subunit B	0										
				Staphylococcus aureus	DNA topoisomerase 4 subunit A	2.88E-10	0.69		DNA topoisomerase IV subunit B															
					DNA topoisomerase 4 subunit B	7.41E-09	0.69	*Escherichia coli K-12*	DNA gyrase subunit A															
									DNA gyrase subunit B															
								Pseudomonas aeruginosa (strain ATCC 15692 / PAO1 / 1C / PRS 101 / LMG12228)	DNA gyrase subunit A															
									DNA gyrase subunit B															
								Staphylococcus aureus subsp. aureus Mu50	DNA gyrase subunit A															
									DNA gyrase subunit B															
								Staphylococcus aureus subsp. aureus N315	DNA gyrase subunit B															
E 4497	DNA gyrase inhibitor	0.376	0.001	*Escherichia coli*	DNA gyrase subunit A	1.07E-17	0.36		Unknown		Unknown	*Escherichia coli K-12*	DNA gyrase	0	Unknown		Unknown				Unknown	18048183003; 180425143154	Unknown	
	DNA topoisomerase IV inhibitor	0.119	0.003		DNA gyrase subunit B	1.21E-10	0.36					*Staphylococcus aureus subsp. aureus Mu50*	DNA gyrase subunit B	0										
				Mycobacterium tuberculosis	DNA gyrase subunit A	3.07E-43	0.43																	
				Staphylococcus aureus	DNA topoisomerase 4 subunit A	3.72E-07	0.38																	
E 4501	DNA gyrase inhibitor	0.590	0.001	*Escherichia coli*	DNA gyrase subunit A	2.71E-36	0.49	*Bacillus subtilis (strain 168)*	DNA gyrase subunit A		Unknown	*Escherichia coli K-12*	DNA gyrase	0	Unknown		Unknown				Unknown	18048183026; 180425143238	Unknown	
	DNA topoisomerase IV inhibitor	0.316	0.002		DNA gyrase subunit B	1.31E-21	0.49		DNA gyrase subunit B			*Staphylococcus aureus subsp. aureus Mu50*	DNA gyrase subunit B	0										
				Mycobacterium tuberculosis	DNA gyrase subunit A	1.01E-78	0.60		DNA topoisomerase IV subunit A															
				Staphylococcus aureus	DNA topoisomerase 4 subunit A	6.43E-10	0.57		DNA topoisomerase IV subunit B															
					DNA topoisomerase 4 subunit B	1.68E-06	0.49	*Escherichia coli K-12*	DNA gyrase subunit A															
									DNA gyrase subunit B															
									DNA topoisomerase IV subunit A															
								Pseudomonas aeruginosa (strain ATCC 15692 / PAO1 / 1C / PRS 101 / LMG12228)	DNA gyrase subunit A															
									DNA gyrase subunit B															
								Staphylococcus aureus subsp. aureus Mu50	DNA gyrase subunit A															
									DNA gyrase subunit B															
								Staphylococcus aureus subsp. aureus N315	DNA gyrase subunit B															
E 4502	DNA gyrase inhibitor	0.561	0.001	*Escherichia coli*	DNA gyrase subunit A	6.69E-58	0.72	*Bacillus subtilis (strain 168)*	DNA gyrase subunit A		Unknown	*Escherichia coli K-12*	DNA gyrase	0	Unknown		Unknown				Unknown	18048183104; 180425143323	Unknown	
	DNA topoisomerase IV inhibitor	0.250	0.002		DNA gyrase subunit B	2.37E-34	0.72		DNA gyrase subunit B			*Staphylococcus aureus subsp. aureus Mu50*	DNA gyrase subunit B	0										
				Mycobacterium tuberculosis	DNA gyrase subunit A	1.75E-48	0.48		DNA topoisomerase IV subunit A															
				Staphylococcus aureus	DNA topoisomerase 4 subunit A	9.19E-11	0.72		DNA topoisomerase IV subunit B															
					DNA topoisomerase 4 subunit B	3.64E-09	0.72	*Escherichia coli K-12*	DNA gyrase subunit A															
									DNA gyrase subunit B															
									DNA topoisomerase IV subunit A															
								Pseudomonas aeruginosa (strain ATCC 15692 / PAO1 / 1C / PRS 101 / LMG12228)	DNA gyrase subunit A															
									DNA gyrase subunit B															
								Staphylococcus aureus subsp. aureus Mu50	DNA gyrase subunit A															
									DNA gyrase subunit B															
								Staphylococcus aureus subsp. aureus N315	DNA gyrase subunit B															
E 4527	DNA gyrase inhibitor	0.357	0.001	*Escherichia coli*	DNA gyrase subunit A	2.03E-34	0.46	*Bacillus subtilis (strain 168)*	DNA gyrase subunit A		Unknown	*Escherichia coli K-12*	DNA gyrase	0	Unknown		Unknown				Unknown	18048204456; 180425143429	Unknown	
	DNA topoisomerase IV inhibitor	0.200	0.002		DNA gyrase subunit B	1.67E-20	0.46		DNA gyrase subunit B			*Staphylococcus aureus subsp. aureus Mu50*	DNA gyrase subunit B	0										
				Mycobacterium tuberculosis	DNA gyrase subunit A	4.16E-70	0.54		DNA topoisomerase IV subunit A															
				Staphylococcus aureus	DNA topoisomerase 4 subunit A	5.27E-09	0.49		DNA topoisomerase IV subunit B															
					DNA topoisomerase 4 subunit B	3.82E-06	0.46	*Escherichia coli K-12*	DNA gyrase subunit A															
									DNA gyrase subunit B															
								Staphylococcus aureus subsp. aureus Mu50	DNA gyrase subunit B															
E 4528	DNA gyrase inhibitor	0.349	0.001	*Escherichia coli*	DNA gyrase subunit A	5.47E-55	0.67	*Bacillus subtilis (strain 168)*	DNA gyrase subunit A		Unknown	*Escherichia coli K-12*	DNA gyrase	0	Unknown		Unknown				Unknown	18048204546; 180425143514	Unknown	
	DNA topoisomerase IV inhibitor	0.160	0.002		DNA gyrase subunit B	1.24E-32	0.67		DNA gyrase subunit B			*Staphylococcus aureus subsp. aureus Mu50*	DNA gyrase	0										
				Mycobacterium tuberculosis	DNA gyrase subunit A	6.59E-44	0.43		DNA topoisomerase IV subunit A				DNA gyrase subunit B	0										
				Staphylococcus aureus	DNA topoisomerase 4 subunit A	5.80E-10	0.67		DNA topoisomerase IV subunit B															
					DNA topoisomerase 4 subunit B	1.43E-08	0.67	*Escherichia coli K-12*	DNA gyrase subunit A															
									DNA gyrase subunit B															
								Staphylococcus aureus subsp. aureus Mu50	DNA gyrase subunit B															
E 4534	DNA gyrase inhibitor	0.596	0.001	*Escherichia coli*	DNA gyrase subunit A	6.13E-34	0.45	*Bacillus subtilis (strain 168)*	DNA gyrase subunit A		Unknown	*Escherichia coli K-12*	DNA gyrase	0	Unknown		Unknown			*Staphylococcus aureus (strain MSSA476)*	DNA topoisomerase 4 subunit A	18048200028; 180425143536	Unknown	
	DNA topoisomerase IV inhibitor	0.248	0.002		DNA gyrase subunit B	3.21E-20	0.45		DNA gyrase subunit B			*Staphylococcus aureus subsp. aureus Mu50*	DNA gyrase subunit B	0										
				Mycobacterium tuberculosis	DNA gyrase subunit A	2.58E-72	0.57		DNA topoisomerase IV subunit A															
				Staphylococcus aureus	DNA topoisomerase 4 subunit A	6.26E-09	0.50		DNA topoisomerase IV subunit B															
					DNA topoisomerase 4 subunit B	5.81E-06	0.45	*Escherichia coli K-12*	DNA gyrase subunit A															
									DNA gyrase subunit B															
								Pseudomonas aeruginosa (strain ATCC 15692 / PAO1 / 1C / PRS 101 / LMG12228)	DNA gyrase subunit A															
									DNA gyrase subunit B															
								Staphylococcus aureus subsp. aureus Mu50	DNA gyrase subunit A															
								Staphylococcus aureus subsp. aureus N315	DNA gyrase subunit B															
E 4535	DNA gyrase inhibitor	0.569	0.001	*Escherichia coli*	DNA gyrase subunit A	1.36E-53	0.62	*Bacillus subtilis (strain 168)*	DNA gyrase subunit A		Unknown	*Escherichia coli K-12*	DNA gyrase	0	Unknown		Unknown			*Staphylococcus aureus (strain MSSA476)*	DNA topoisomerase 4 subunit A	18048204616; 180425143607	Unknown	
	DNA topoisomerase IV inhibitor	0.202	0.002		DNA gyrase subunit B	8.24E-32	0.62		DNA gyrase subunit B			*Staphylococcus aureus subsp. aureus Mu50*	DNA gyrase subunit B	0										
				Mycobacterium tuberculosis	DNA gyrase subunit A	1.28E-47	0.48		DNA topoisomerase IV subunit A															
				Staphylococcus aureus	DNA topoisomerase 4 subunit A	9.10E-10	0.62		DNA topoisomerase IV subunit B															
					DNA topoisomerase 4 subunit B	5.01E-08	0.62	*Escherichia coli K-12*	DNA gyrase subunit A															
									DNA gyrase subunit B															
								Pseudomonas aeruginosa (strain ATCC 15692 / PAO1 / 1C / PRS 101 / LMG12228)	DNA gyrase subunit A															
									DNA gyrase subunit B															
								Staphylococcus aureus subsp. aureus Mu50	DNA gyrase subunit A															
								Staphylococcus aureus subsp. aureus N315	DNA gyrase subunit B															
E 4695	DNA gyrase inhibitor	0.416	0.001	*Escherichia coli*	DNA gyrase subunit A	8.42E-33	0.43		Unknown		Unknown	*Escherichia coli K-12*	DNA gyrase	0	Unknown		Unknown			*Staphylococcus aureus (strain MSSA476)*	DNA topoisomerase 4 subunit A	18048200106; 180425143634	Unknown	
	DNA topoisomerase IV inhibitor	0.200	0.002		DNA gyrase subunit B	1.50E-19	0.43					*Staphylococcus aureus subsp. aureus Mu50*	DNA gyrase	0										
				Mycobacterium tuberculosis	DNA gyrase subunit A	2.14E-48	0.49						DNA gyrase subunit B	0										
				Staphylococcus aureus	DNA topoisomerase 4 subunit A	3.46E-08	0.43																	
					DNA topoisomerase 4 subunit B	8.35E-06	0.43																	
Ecenofloxacin	DNA gyrase inhibitor	0.276	0.002	*Escherichia coli*	DNA gyrase subunit A	6.47E-32	0.41	*Escherichia coli K-12*	DNA gyrase subunit A		Unknown	*Escherichia coli K-12*	DNA gyrase	0	Unknown		Unknown				Unknown	18048124011; 180426135552	Unknown	
	DNA topoisomerase IV inhibitor	0.125	0.003		DNA gyrase subunit B	5.01E-19	0.41		DNA gyrase subunit B			*Staphylococcus aureus subsp. aureus Mu50*	DNA gyrase	0										
				Mycobacterium tuberculosis	DNA gyrase subunit A	6.33E-47	0.44	*Pseudomonas aeruginosa (strain ATCC 15692 / PAO1 / 1C / PRS 101 / LMG12228)*	DNA gyrase subunit A				DNA gyrase subunit B	0										
				Staphylococcus aureus	DNA topoisomerase 4 subunit A	3.91E-08	0.44		DNA gyrase subunit B															
								Staphylococcus aureus subsp. aureus Mu50	DNA gyrase subunit A															
									DNA gyrase subunit B															
									DNA topoisomerase IV subunit A															
								Staphylococcus aureus subsp. aureus N315	DNA gyrase subunit B															
EN 272	DNA topoisomerase IV inhibitor	0.023	0.012	*Escherichia coli*	DNA gyrase subunit A	4.77E-09	0.34		Unknown		Unknown	*Escherichia coli K-12*	DNA gyrase	0	Unknown		Unknown				Unknown	18048152742; 180426135618	DNA gyrase	J. Med. Chem.,(1986)29:3:394; J. Med. Chem.,(1986)29:4:445
Enoxacin	DNA gyrase inhibitor	0.203	0.002	*Escherichia coli*	DNA gyrase subunit A	1.07E-10	0.46	*Bacillus subtilis (strain 168)*	DNA gyrase subunit A		Unknown	*Escherichia coli K-12*	DNA gyrase	0	Unknown		Unknown				Unknown	18048111237; 180426135721	Unknown	
	DNA topoisomerase IV inhibitor	0.127	0.003		DNA gyrase subunit B	1.63E-06	0.46		DNA gyrase subunit B			*Staphylococcus aureus subsp. aureus Mu50*	DNA gyrase subunit B	0										
				Mycobacterium tuberculosis	DNA gyrase subunit A	1.12E-41	0.41		DNA topoisomerase IV subunit A															
				Staphylococcus aureus	DNA topoisomerase 4 subunit A	5.68E-08	0.46		DNA topoisomerase IV subunit B															
					DNA topoisomerase 4 subunit B	3.95E-06	0.46	*Escherichia coli*	DNA gyrase subunit A															
									DNA gyrase subunit B															
								Escherichia coli K-12	DNA gyrase subunit A															
									DNA gyrase subunit B															
								Escherichia coli K-12	DNA topoisomerase IV subunit A															
								Pseudomonas aeruginosa (strain ATCC 15692 / PAO1 / 1C / PRS 101 / LMG12228)	DNA gyrase subunit A															
									DNA gyrase subunit B															
								Mycobacterium tuberculosis	DNA gyrase subunit A															
									DNA gyrase subunit B															
								Staphylococcus aureus subsp. aureus Mu50	DNA gyrase subunit A															
									DNA gyrase subunit B															
								Staphylococcus aureus subsp. aureus N315	DNA gyrase subunit B															
Enrofloxacin	DNA gyrase inhibitor	0.392	0.001	*Escherichia coli*	DNA gyrase subunit A	6.54E-67	0.72	*Escherichia coli K-12*	DNA gyrase subunit A		Unknown	*Staphylococcus aureus subsp. aureus Mu50*	DNA gyrase	0.01 to 0	Unknown		Unknown			*Staphylococcus aureus (strain MSSA476)*	DNA topoisomerase 4 subunit A	18048103830; 180426135814	Unknown	
	DNA topoisomerase IV inhibitor	0.214	0.002		DNA gyrase subunit B	1.15E-39	0.72		DNA gyrase subunit B				DNA gyrase subunit B	0										
				Mycobacterium tuberculosis	DNA gyrase subunit A	4.31E-45	0.44	*Staphylococcus aureus subsp. aureus Mu50*	DNA gyrase subunit A															
				Staphylococcus aureus	DNA topoisomerase 4 subunit A	1.53E-10	0.72		DNA gyrase subunit B															
					DNA topoisomerase 4 subunit B	3.16E-09	0.72	*Staphylococcus aureus subsp. aureus N315*	DNA gyrase subunit B															
Esafloxacin	DNA gyrase inhibitor	0.366	0.001	*Escherichia coli*	DNA gyrase subunit A	2.94E-21	0.35	*Escherichia coli*	DNA gyrase subunit A		Unknown	*Escherichia coli K-12*	DNA gyrase	0	Unknown		Unknown				Unknown	18048124047; 180426140112	Unknown	
	DNA topoisomerase IV inhibitor	0.185	0.002		DNA gyrase subunit B	9.65E-13	0.35		DNA gyrase subunit B			*Staphylococcus aureus subsp. aureus Mu50*	DNA gyrase subunit B	0										
				Mycobacterium tuberculosis	DNA gyrase subunit A	1.86E-40	0.39	*Escherichia coli K-12*	DNA gyrase subunit A															
				Staphylococcus aureus	DNA topoisomerase 4 subunit A	5.21E-07	0.38		DNA gyrase subunit B															
								Pseudomonas aeruginosa (strain ATCC 15692 / PAO1 / 1C / PRS 101 / LMG12228)	DNA gyrase subunit A															
									DNA gyrase subunit B															
								Mycobacterium tuberculosis	DNA gyrase subunit A															
									DNA gyrase subunit B															
								Staphylococcus aureus subsp. aureus Mu50	DNA gyrase subunit A															
									DNA gyrase subunit B															
									DNA topoisomerase IV subunit A															
								Staphylococcus aureus subsp. aureus N315	DNA gyrase subunit B															

Bioactive molecule	PASS online[4]			Similarity Ensemble Approach (SEA)[2]				ChemProt[3]		SuperPred[6]		Polypharmacology Browser (PPB)[1]			SPiDER[7]		HitPick[5]			PharmMapper[9]			TargetHunter[8]	
	Activity	Pa	Pi	Organism	Target	P-Value	Max TC	Organism	Target	Organism	Target	Organism	Target	P-Value	Target	Confidence level	Target	Precision (%)	Tc similarity	Organism	Target	Job Id	Target	Reference
FA 103	DNA gyrase inhibitor	0.601	0.001	*Escherichia coli*	DNA gyrase subunit A	4.05E-10	0.41	*Bacillus subtilis (strain 168)*	DNA gyrase subunit A		Unknown	*Escherichia coli K-12*	DNA gyrase	0	Unknown		Unknown				Unknown	18048200134; 180426140325	Unknown	
	DNA topoisomerase IV inhibitor	0.226	0.002		DNA gyrase subunit B	3.58E-06	0.41		DNA gyrase subunit B			*Staphylococcus aureus subsp. aureus Mu50*	DNA gyrase subunit B	0										
				Mycobacterium tuberculosis	DNA gyrase subunit A	8.50E-44	0.44		DNA topoisomerase IV subunit A															
				Staphylococcus aureus	DNA topoisomerase 4 subunit A	1.37E-07	0.41		DNA topoisomerase IV subunit B															
								Escherichia coli K-12	DNA gyrase subunit A															
									DNA gyrase subunit B															
									DNA topoisomerase IV subunit A															
								Pseudomonas aeruginosa (strain ATCC 15692 / PAO1 / 1C / PRS 101 / LMG12228)	DNA gyrase subunit A															
									DNA gyrase subunit B															
								Staphylococcus aureus subsp. aureus Mu50	DNA gyrase subunit A															
									DNA gyrase subunit B															
									DNA topoisomerase IV subunit A															
								Staphylococcus aureus subsp. aureus N315	DNA gyrase subunit B															
Fandofloxacin	DNA gyrase inhibitor	0.083	0.004	*Escherichia coli*	DNA gyrase subunit A	1.95E-50	0.47	*Escherichia coli*	DNA gyrase subunit A		Unknown	*Escherichia coli K-12*	DNA gyrase	0	Unknown		Unknown				Unknown	18048132828; 180426140521	Unknown	
	DNA topoisomerase IV inhibitor	0.182	0.002		DNA gyrase subunit B	5.99E-30	0.47		DNA gyrase subunit B			*Staphylococcus aureus subsp. aureus Mu50*	DNA gyrase	0										
				Mycobacterium tuberculosis	DNA gyrase subunit A	3.25E-32	0.31	*Escherichia coli K-12*	DNA gyrase subunit A				DNA gyrase subunit B	0										
				Staphylococcus aureus	DNA topoisomerase 4 subunit A	4.14E-07	0.45		DNA gyrase subunit B															
					DNA topoisomerase 4 subunit B	5.56E-06	0.45	*Pseudomonas aeruginosa (strain ATCC 15692 / PAO1 / 1C / PRS 101 / LMG12228)*	DNA gyrase subunit A															
									DNA gyrase subunit B															
								Mycobacterium tuberculosis	DNA gyrase subunit A															
									DNA gyrase subunit B															
								Staphylococcus aureus	DNA topoisomerase IV subunit A															
								Staphylococcus aureus subsp. aureus Mu50	DNA gyrase subunit A															
									DNA gyrase subunit B															
								Staphylococcus aureus subsp. aureus N315	DNA gyrase subunit B															
Finafloxacin	DNA gyrase inhibitor	0.263	0.002	*Escherichia coli*	DNA gyrase subunit A	2.63E-31	0.43	*Bacillus subtilis (strain 168)*	DNA gyrase subunit A		Unknown	*Escherichia coli K-12*	DNA gyrase	0.01 to 0	Unknown		Unknown				Unknown	18048095955; 180426140559	Unknown	
	DNA topoisomerase IV inhibitor	0.101	0.003		DNA gyrase subunit B	1.15E-18	0.43		DNA gyrase subunit B			*Staphylococcus aureus subsp. aureus Mu50*	DNA gyrase	0										
				Mycobacterium tuberculosis	DNA gyrase subunit A	3.31E-56	0.55		DNA topoisomerase IV subunit A				DNA gyrase subunit B	0										
				Staphylococcus aureus	DNA topoisomerase 4 subunit A	3.30E-09	0.55		DNA topoisomerase IV subunit B															
					DNA topoisomerase 4 subunit B	9.88E-06	0.43	*Escherichia coli*	DNA gyrase subunit A															
									DNA gyrase subunit B															
								Escherichia coli K-12	DNA gyrase subunit A															
									DNA gyrase subunit B															
									DNA topoisomerase IV subunit A															
								Pseudomonas aeruginosa (strain ATCC 15692 / PAO1 / 1C / PRS 101 / LMG12228)	DNA gyrase subunit A															
									DNA gyrase subunit B															
								Mycobacterium tuberculosis	DNA gyrase subunit A															
									DNA gyrase subunit B															
								Staphylococcus aureus	DNA topoisomerase IV subunit A															
								Staphylococcus aureus subsp. aureus Mu50	DNA gyrase subunit A															
									DNA gyrase subunit B															
									DNA topoisomerase IV subunit A															
								Staphylococcus aureus subsp. aureus N315	DNA gyrase subunit B															
Fleroxacin	DNA gyrase inhibitor	0.085	0.004	*Escherichia coli*	DNA gyrase subunit A	2.31E-29	0.35	*Escherichia coli*	DNA gyrase subunit A		Unknown	*Escherichia coli K-12*	DNA gyrase	0	Unknown		Unknown			*Staphylococcus aureus (strain MSSA476)*	DNA topoisomerase 4 subunit A	18048100030; 180426140626	Unknown	
	DNA topoisomerase IV inhibitor	0.060	0.003		DNA gyrase subunit B	1.61E-17	0.35		DNA gyrase subunit B			*Staphylococcus aureus subsp. aureus Mu50*	DNA gyrase subunit B	0										
				Mycobacterium tuberculosis	DNA gyrase subunit A	2.48E-42	0.41	*Escherichia coli K-12*	DNA gyrase subunit A															
				Staphylococcus aureus	DNA topoisomerase 4 subunit A	3.98E-07	0.39		DNA gyrase subunit B															
								Pseudomonas aeruginosa (strain ATCC 15692 / PAO1 / 1C / PRS 101 / LMG12228)	DNA gyrase subunit A															
									DNA gyrase subunit B															
								Mycobacterium tuberculosis	DNA gyrase subunit A															
									DNA gyrase subunit B															
								Staphylococcus aureus	DNA topoisomerase IV subunit A															
								Staphylococcus aureus subsp. aureus Mu50	DNA gyrase subunit A															
									DNA gyrase subunit B															
								Staphylococcus aureus subsp. aureus N315	DNA gyrase subunit B															
Flumequine	DNA gyrase inhibitor	0.179	0.002	*Escherichia coli*	DNA gyrase subunit A	8.50E-31	0.41		Unknown		Unknown	*Escherichia coli K-12*	DNA gyrase	0	Unknown		Unknown				Unknown	180330133120	DNA gyrase	Antimicrob. Agents Chemother.,(2007)51:9:3410; Bioorg. Med. Chem. Lett.,(1992)2:7:643; Bioorg. Med. Chem. Lett.,(2003)13:23:4229; J. Med. Chem.,(1986)29:3:394; J. Med. Chem.,(1986)29:4:445
	DNA topoisomerase IV inhibitor	0.052	0.003		DNA gyrase subunit B	2.29E-18	0.41					*Staphylococcus aureus subsp. aureus Mu50*	DNA gyrase subunit B	0									DNA gyrase subunit B	J. Med. Chem.,(1992)35:25:4745
				Mycobacterium tuberculosis	DNA gyrase subunit A	3.81E-42	0.42																Topoisomerase IV subunit A	Bioorg. Med. Chem. Lett.,(2006)16:5:1272; Bioorg. Med. Chem. Lett.,(2006)16:5:1277; J. Med. Chem.,(2005)48:16:5232; J. Med. Chem.,(2006)49:1:39; J. Med. Chem.,(2013)56:18:7396
				Staphylococcus aureus	DNA topoisomerase 4 subunit A	1.80E-07	0.41																	
Garenoxacin	DNA gyrase inhibitor	0.116	0.003	*Escherichia coli*	DNA gyrase subunit A	8.41E-26	0.34		Unknown		Unknown	*Escherichia coli K-12*	DNA gyrase	0.01 to 0	Unknown		Unknown			*Staphylococcus aureus (strain MSSA476)*	DNA topoisomerase 4 subunit A	18048101433; 180426140701	Unknown	
	DNA topoisomerase IV inhibitor	0.058	0.003		DNA gyrase subunit B	2.00E-15	0.34					*Staphylococcus aureus subsp. aureus Mu50*	DNA gyrase subunit B	0										
				Mycobacterium tuberculosis	DNA gyrase subunit A	2.90E-46	0.46					*Staphylococcus aureus*	Topoisomerase IV subunit A	0										
				Staphylococcus aureus	DNA topoisomerase 4 subunit A	3.05E-07	0.41																	
Gatifloxacin	DNA gyrase inhibitor	0.708	0.000	*Escherichia coli*	DNA gyrase subunit A	3.70E-35	0.51	*Bacillus subtilis (strain 168)*	DNA gyrase subunit A		Unknown		Unknown		Unknown		Unknown				Unknown	18048100104; 180426152529	Unknown	
	DNA topoisomerase IV inhibitor	0.372	0.002		DNA gyrase subunit B	6.12E-21	0.51		DNA gyrase subunit B															
				Mycobacterium tuberculosis	DNA gyrase subunit A	1.79E-112	1.00		DNA topoisomerase IV subunit A															
				Staphylococcus aureus	DNA topoisomerase 4 subunit A	5.46E-14	0.72	*Escherichia coli*	DNA gyrase subunit A															
					DNA topoisomerase 4 subunit B	1.09E-06	0.51		DNA gyrase subunit B															
								Escherichia coli K-12	DNA gyrase subunit A															
									DNA gyrase subunit B															
									DNA topoisomerase IV subunit A															
								Pseudomonas aeruginosa (strain ATCC 15692 / PAO1 / 1C / PRS 101 / LMG12228)	DNA gyrase subunit A															
									DNA gyrase subunit B															
								Mycobacterium tuberculosis	DNA gyrase subunit B															
								Staphylococcus aureus subsp. aureus Mu50	DNA gyrase subunit A															
									DNA gyrase subunit B															
								Staphylococcus aureus subsp. aureus N315	DNA gyrase subunit B															
Gemifloxacin	DNA gyrase inhibitor	0.704	0.000	*Escherichia coli*	DNA gyrase subunit A	8.35E-32	0.39	*Escherichia coli K-12*	DNA gyrase subunit A		Unknown	*Escherichia coli K-12*	DNA gyrase	0	Unknown		Unknown				Unknown	18048111310; 180426152559	DNA gyrase	Bioorg. Med. Chem. Lett.,(2006)16:5:1272; J. Med. Chem.,(1991)34:2:656; J. Med. Chem.,(1992)35:10:1764; J. Med. Chem.,(1993)36:7:871; J. Med. Chem.,(1993)36:14:1964
	DNA topoisomerase IV inhibitor	0.524	0.001		DNA gyrase subunit B	5.82E-19	0.39		DNA gyrase subunit B			*Staphylococcus aureus subsp. aureus Mu50*	DNA gyrase subunit B	0									DNA gyrase subunit B	J. Med. Chem.,(1992)35:25:4745
				Mycobacterium tuberculosis	DNA gyrase subunit A	2.90E-46	0.46					*Staphylococcus aureus*	Topoisomerase IV subunit A	0									Topoisomerase IV subunit A	Bioorg. Med. Chem. Lett.,(2006)16:5:1272
				Staphylococcus aureus	DNA topoisomerase 4 subunit A	1.05E-07	0.41																	
Grepafloxacin	DNA gyrase inhibitor	0.662	0.001	*Escherichia coli*	DNA gyrase subunit A	2.38E-36	0.52	*Bacillus subtilis (strain 168)*	DNA gyrase subunit A		Unknown	*Escherichia coli K-12*	DNA gyrase	0	Unknown		Unknown				Unknown	180327134053	Unknown	
	DNA topoisomerase IV inhibitor	0.145	0.003		DNA gyrase subunit B	1.21E-21	0.52		DNA gyrase subunit B			*Staphylococcus aureus subsp. aureus Mu50*	DNA gyrase subunit B	0										
				Mycobacterium tuberculosis	DNA gyrase subunit A	8.67E-49	0.53		DNA topoisomerase IV subunit A															
				Staphylococcus aureus	DNA topoisomerase 4 subunit A	1.15E-08	0.52	*Escherichia coli*	DNA gyrase subunit A															
					DNA topoisomerase 4 subunit B	7.01E-07	0.52		DNA gyrase subunit B															
								Escherichia coli K-12	DNA gyrase subunit A															
									DNA gyrase subunit B															
									DNA topoisomerase IV subunit A															
								Pseudomonas aeruginosa (strain ATCC 15692 / PAO1 / 1C / PRS 101 / LMG12228)	DNA gyrase subunit A															
									DNA gyrase subunit B															
								Mycobacterium tuberculosis	DNA gyrase subunit B															
								Staphylococcus aureus subsp. aureus Mu50	DNA gyrase subunit A															
									DNA gyrase subunit B															
									DNA topoisomerase IV subunit A															
								Staphylococcus aureus subsp. aureus N315	DNA gyrase subunit B															
Ibafloxacin	DNA gyrase inhibitor	0.166	0.003	*Escherichia coli*	DNA gyrase subunit A	1.51E-31	0.42		Unknown		Unknown	*Escherichia coli K-12*	DNA gyrase	0	Unknown		Unknown				Unknown	18048103912; 180426152626	DNA gyrase	J. Med. Chem.,(1986)29:3:394; J. Med. Chem.,(1993)36:14:1964; J. Med. Chem.,(1996)39:25:4952; J. Med. Chem.,(2005)48:16:5232
	DNA topoisomerase IV inhibitor	0.046	0.004		DNA gyrase subunit B	8.26E-19	0.42					*Staphylococcus aureus subsp. aureus Mu50*	DNA gyrase subunit B	0									DNA gyrase subunit B	J. Med. Chem.,(1992)35:25:4745
				Mycobacterium tuberculosis	DNA gyrase subunit A	2.61E-46	0.48																Topoisomerase IV subunit A	J. Med. Chem.,(2005)48:16:5232
				Staphylococcus aureus	DNA topoisomerase 4 subunit A	8.55E-08	0.42																	
Irloxacin	DNA gyrase inhibitor	0.201	0.002	*Escherichia coli*	DNA gyrase subunit A	2.90E-42	0.43	*Escherichia coli K-12*	DNA gyrase subunit A		Unknown	*Escherichia coli K-12*	DNA gyrase	0	Unknown		Unknown			*Staphylococcus aureus (strain MSSA476)*	DNA topoisomerase 4 subunit A	18048124120; 180426152657	Unknown	
	DNA topoisomerase IV inhibitor	0.125	0.003		DNA gyrase subunit B	3.95E-25	0.43		DNA gyrase subunit B			*Staphylococcus aureus subsp. aureus Mu50*	DNA gyrase subunit B	0										
				Mycobacterium tuberculosis	DNA gyrase subunit A	7.47E-32	0.30																	
				Staphylococcus aureus	DNA topoisomerase 4 subunit A	5.88E-07	0.43																	
K 12	DNA gyrase inhibitor	0.079	0.004	*Escherichia coli*	DNA gyrase subunit A	3.47E-36	0.37	*Escherichia coli*	DNA gyrase subunit A		Unknown	*Escherichia coli K-12*	DNA gyrase	0	Unknown		Unknown			*Staphylococcus aureus (strain MSSA476)*	DNA topoisomerase 4 subunit A	18048200158; 180426152724	Unknown	
	DNA topoisomerase IV inhibitor	0.120	0.003		DNA gyrase subunit B	1.52E-21	0.37		DNA gyrase subunit B			*Staphylococcus aureus subsp. aureus Mu50*	DNA gyrase subunit B	0										
				Mycobacterium tuberculosis	DNA gyrase subunit A	2.39E-41	0.40	*Escherichia coli K-12*	DNA gyrase subunit A															
				Staphylococcus aureus	DNA topoisomerase 4 subunit A	9.71E-07	0.38		DNA gyrase subunit B															
								Mycobacterium tuberculosis	DNA gyrase subunit A															
									DNA gyrase subunit B															
								Staphylococcus aureus subsp. aureus Mu50	DNA gyrase subunit A															
									DNA gyrase subunit B															
								Staphylococcus aureus subsp. aureus N315	DNA gyrase subunit B															
KB 5246	DNA gyrase inhibitor	0.111	0.003	*Mycobacterium tuberculosis*	DNA gyrase subunit A	6.66E-16	0.29	*Escherichia coli*	DNA gyrase subunit A		Unknown	*Staphylococcus aureus subsp. aureus Mu50*	DNA gyrase subunit B	0.04	Unknown		Unknown				Unknown	18048201936; 180426152756	Unknown	
	DNA topoisomerase IV inhibitor	0.038	0.004						DNA gyrase subunit B															
								Escherichia coli K-12	DNA gyrase subunit A															
									DNA gyrase subunit B															
								Mycobacterium smegmatis	DNA topoisomerase (ATP-hydrolysing) (DNA gyrase)															
								Mycobacterium tuberculosis	DNA gyrase subunit A															
									DNA gyrase subunit B															
								Staphylococcus aureus subsp. aureus Mu50	DNA gyrase subunit B															

Bioactive molecule	PASS online[4]			Similarity Ensemble Approach (SEA)[2]				ChemProt[3]		SuperPred[6]		Polypharmacology Browser (PPB)[1]			SPiDER[7]		HitPick[5]			PharmMapper[9]			TargetHunter[8]	
	Activity	Pa	Pi	Organism	Target	P-Value	Max TC	Organism	Target	Organism	Target	Organism	Target	P-Value	Target	Confidence level	Target	Precision (%)	Tc similarity	Organism	Target	Job Id	Target	Reference
KPI 10 free base (WQ 3810)	DNA gyrase inhibitor	0.307	0.002	*Mycobacterium tuberculosis*	DNA gyrase subunit A	1.06E-41	0.40	*Bacillus subtilis (strain 168)*	DNA gyrase subunit A		Unknown	*Escherichia coli K-12*	DNA gyrase	0	Unknown		Unknown				Unknown	18048142730	Unknown	
	DNA topoisomerase IV inhibitor	0.322	0.002	*Staphylococcus aureus*	DNA topoisomerase 4 subunit A	1.15E-06	0.40		DNA gyrase subunit B			*Staphylococcus aureus subsp. aureus Mu50*	DNA gyrase	0.01 to 0										
									DNA topoisomerase IV subunit A				DNA gyrase subunit B	0										
									DNA topoisomerase IV subunit B															
								Escherichia coli K-12	DNA gyrase subunit A															
									DNA gyrase subunit B															
								Staphylococcus aureus subsp. aureus Mu50	DNA gyrase subunit B															
Lascufloxacin	DNA gyrase inhibitor	0.228	0.002	*Escherichia coli*	DNA gyrase subunit A	1.21E-08	0.30	*Bacillus subtilis (strain 168)*	DNA gyrase subunit A		Unknown	*Escherichia coli K-12*	DNA gyrase	0	Unknown		Unknown			*Staphylococcus aureus (strain MSSA476)*	DNA topoisomerase 4 subunit A	18048152816; 180426152839	Unknown	
	DNA topoisomerase IV inhibitor	0.056	0.003	*Mycobacterium tuberculosis*	DNA gyrase subunit A	7.90E-67	0.48		DNA gyrase subunit B			*Staphylococcus aureus subsp. aureus Mu50*	DNA gyrase subunit B	0										
				Staphylococcus aureus	DNA topoisomerase 4 subunit A	1.77E-07	0.48		DNA topoisomerase IV subunit A															
									DNA topoisomerase IV subunit B															
								Escherichia coli	DNA gyrase subunit A															
									DNA gyrase subunit B															
									DNA topoisomerase IV subunit A															
								Escherichia coli K-12	DNA gyrase subunit A															
									DNA gyrase subunit B															
								Pseudomonas aeruginosa (strain ATCC 15692 / PAO1 / 1C / PRS 101 / LMG12228)	DNA gyrase subunit A															
									DNA gyrase subunit B															
								Mycobacterium tuberculosis	DNA gyrase subunit B															
								Staphylococcus aureus subsp. aureus Mu50	DNA gyrase subunit A															
									DNA gyrase subunit B															
									DNA topoisomerase IV subunit A															
								Staphylococcus aureus subsp. aureus N315	DNA gyrase subunit B															
Levofloxacin	DNA gyrase inhibitor	0.708	0.000	*Escherichia coli*	DNA gyrase subunit A	4.29E-35	0.38	*Escherichia coli*	DNA gyrase subunit A		Unknown		Unknown		Unknown		Unknown				Unknown	18048100135; 180426152908	Unknown	
	DNA topoisomerase IV inhibitor	0.372	0.002		DNA gyrase subunit B	6.68E-21	0.38		DNA gyrase subunit B															
				Mycobacterium tuberculosis	DNA gyrase subunit A	5.90E-45	0.45	*Escherichia coli K-12*	DNA gyrase subunit A															
				Staphylococcus aureus	DNA topoisomerase 4 subunit A	2.01E-07	0.40		DNA gyrase subunit B															
								Mycobacterium smegmatis	DNA topoisomerase (ATP-hydrolysing) (DNA gyrase)															
								Mycobacterium smegmatis (strain ATCC 700084 / mc(2)155)	DNA gyrase subunit B															
								Mycobacterium tuberculosis	DNA gyrase subunit B															
								Staphylococcus aureus subsp. aureus Mu50	DNA gyrase subunit A															
									DNA gyrase subunit B															
								Staphylococcus aureus subsp. aureus N315	DNA gyrase subunit B															
Levonadifloxacin	DNA gyrase inhibitor	0.278	0.002	*Escherichia coli*	DNA gyrase subunit A	2.69E-23	0.40	*Escherichia coli K-12*	DNA gyrase subunit A		Unknown	*Escherichia coli K-12*	DNA gyrase	0	Unknown		Unknown			*Staphylococcus aureus (strain MSSA476)*	DNA topoisomerase 4 subunit A	18048101520; 180426153036	Unknown	
	DNA topoisomerase IV inhibitor	0.063	0.003		DNA gyrase subunit B	6.05E-14	0.40		DNA gyrase subunit B			*Staphylococcus aureus subsp. aureus Mu50*	DNA gyrase subunit B	0										
				Mycobacterium tuberculosis	DNA gyrase subunit A	8.95E-65	0.50	*Staphylococcus aureus*	DNA topoisomerase IV subunit A															
				Staphylococcus aureus	DNA topoisomerase 4 subunit A	5.02E-08	0.44	*Staphylococcus aureus subsp. aureus Mu50*	DNA gyrase subunit A															
									DNA gyrase subunit B															
								Staphylococcus aureus subsp. aureus N315	DNA gyrase subunit B															
Levonadifloxacin arginine (WCK 771)	DNA gyrase inhibitor	0.278	0.002	*Escherichia coli*	DNA gyrase subunit A	1.66E-17	0.32		Unknown		Unknown	*Escherichia coli K-12*	DNA gyrase	0	Unknown		Unknown				Unknown	18048152856; 180426153128	DNA gyrase	J. Med. Chem.,(1986)29:3:394; J. Med. Chem.,(1991)34:3:1155; J. Med. Chem.,(1993)36:7:871; J. Med. Chem.,(1996)39:25:4952; J. Med. Chem.,(2005)48:16:5232
	DNA topoisomerase IV inhibitor	0.063	0.003		DNA gyrase subunit B	1.58E-10	0.32					*Staphylococcus aureus subsp. aureus Mu50*	DNA gyrase subunit B	0									DNA gyrase subunit B	J. Med. Chem.,(1992)35:25:4745
				Mycobacterium tuberculosis	DNA gyrase subunit A	1.40E-40	0.40																Topoisomerase IV subunit A	Bioorg. Med. Chem. Lett.,(2004)14:20:5193; J. Med. Chem.,(2005)48:16:5232
				Staphylococcus aureus	DNA topoisomerase 4 subunit A	1.58E-06	0.36																	
Lomefloxacin	DNA gyrase inhibitor	0.424	0.001	*Escherichia coli*	DNA gyrase subunit A	7.84E-14	0.40	*Bacillus subtilis (strain 168)*	DNA gyrase subunit A		Unknown	*Escherichia coli K-12*	DNA gyrase	0	Unknown		Unknown				Unknown	180327134208	Unknown	
	DNA topoisomerase IV inhibitor	0.143	0.003		DNA gyrase subunit B	2.31E-08	0.40		DNA gyrase subunit B			*Staphylococcus aureus subsp. aureus Mu50*	DNA gyrase subunit B	0										
				Mycobacterium tuberculosis	DNA gyrase subunit A	8.50E-73	0.62		DNA topoisomerase IV subunit A															
				Staphylococcus aureus	DNA topoisomerase 4 subunit A	3.39E-08	0.46		DNA topoisomerase IV subunit B															
								Escherichia coli	DNA gyrase subunit A															
									DNA gyrase subunit B															
								Escherichia coli K-12	DNA gyrase subunit A															
									DNA gyrase subunit B															
									DNA topoisomerase IV subunit A															
								Pseudomonas aeruginosa (strain ATCC 15692 / PAO1 / 1C / PRS 101 / LMG12228)	DNA gyrase subunit A															
									DNA gyrase subunit B															
								Mycobacterium tuberculosis	DNA gyrase subunit B															
								Staphylococcus aureus subsp. aureus Mu50	DNA gyrase subunit A															
									DNA gyrase subunit B															
								Staphylococcus aureus subsp. aureus N315	DNA gyrase subunit B															
Marbofloxacin	DNA gyrase inhibitor	0.040	0.008	*Escherichia coli*	DNA gyrase subunit A	2.92E-25	0.33	*Escherichia coli*	DNA gyrase subunit A		Unknown	*Escherichia coli K-12*	DNA gyrase	0	Unknown		Unknown				Unknown	18048103948; 180426161627	DNA gyrase	Bioorg. Med. Chem. Lett.,(2006)16:5:1272; J. Med. Chem.,(1988)31:5:991; J. Med. Chem.,(1986)29:4:445; J. Med. Chem.,(2003)46:17:3655; J. Med. Chem.,(2006)49:22:6435
	DNA topoisomerase IV inhibitor	0.042	0.004		DNA gyrase subunit B	4.22E-15	0.33		DNA gyrase subunit B			*Staphylococcus aureus subsp. aureus Mu50*	DNA gyrase subunit B	0									DNA gyrase subunit A	Antimicrob. Agents Chemother.,(2010)54:8:3478
				Mycobacterium tuberculosis	DNA gyrase subunit A	3.16E-39	0.38	*Escherichia coli K-12*	DNA gyrase subunit A														DNA gyrase subunit B	Bioorg. Med. Chem.,(2014)22:17:4924; Bioorg. Med. Chem.,(2015)23:9:2062; J. Med. Chem.,(1992)35:25:4745
				Staphylococcus aureus	DNA topoisomerase 4 subunit A	1.27E-06	0.35		DNA gyrase subunit B														Topoisomerase IV subunit A	Bioorg. Med. Chem. Lett.,(2006)16:5:1272; Bioorg. Med. Chem. Lett.,(2006)16:5:1277
								Mycobacterium tuberculosis	DNA gyrase subunit A															
								Staphylococcus aureus subsp. aureus Mu50	DNA gyrase subunit B															
Merafloxacin	DNA gyrase inhibitor	0.372	0.001	*Escherichia coli*	DNA gyrase subunit A	2.11E-09	0.34	*Escherichia coli*	DNA gyrase subunit A		Unknown	*Escherichia coli K-12*	DNA gyrase	0	Unknown		Unknown			*Staphylococcus aureus (strain MSSA476)*	DNA topoisomerase 4 subunit A	18048124147; 180426161748	Unknown	
	DNA topoisomerase IV inhibitor	0.151	0.002		DNA gyrase subunit B	9.48E-06	0.34		DNA gyrase subunit B			*Staphylococcus aureus subsp. aureus Mu50*	DNA gyrase subunit B	0										
				Mycobacterium tuberculosis	DNA gyrase subunit A	4.75E-60	0.43	*Escherichia coli K-12*	DNA gyrase subunit A															
				Staphylococcus aureus	DNA topoisomerase 4 subunit A	3.82E-07	0.40		DNA gyrase subunit B															
								Pseudomonas aeruginosa (strain ATCC 15692 / PAO1 / 1C / PRS 101 / LMG12228)	DNA gyrase subunit A															
									DNA gyrase subunit B															
								Mycobacterium tuberculosis	DNA gyrase subunit A															
									DNA gyrase subunit B															
								Staphylococcus aureus subsp. aureus Mu50	DNA gyrase subunit A															
									DNA gyrase subunit B															
									DNA topoisomerase IV subunit A															
								Staphylococcus aureus subsp. aureus N315	DNA gyrase subunit B															
Metioxate	Unknown				Unknown				Unknown		Unknown		Unknown		Unknown		Unknown				Unknown	18048132900; 180426161816	Unknown	
MF 5101	DNA gyrase inhibitor	0.280	0.002	*Escherichia coli*	DNA gyrase subunit A	7.41E-53	0.59	*Escherichia coli K-12*	DNA gyrase subunit A		Unknown	*Escherichia coli K-12*	DNA gyrase	0	Unknown		Unknown				Unknown	18048161118; 180426161852	Unknown	
	DNA topoisomerase IV inhibitor	0.073	0.003		DNA gyrase subunit B	2.24E-31	0.59		DNA gyrase subunit B			*Staphylococcus aureus subsp. aureus Mu50*	DNA gyrase	0										
				Mycobacterium tuberculosis	DNA gyrase subunit A	2.21E-39	0.39						DNA gyrase subunit B	0										
				Staphylococcus aureus	DNA topoisomerase 4 subunit A	6.47E-09	0.59																	
					DNA topoisomerase 4 subunit B	1.18E-07	0.59																	
MF 5103	DNA gyrase inhibitor	0.335	0.002	*Escherichia coli*	DNA gyrase subunit A	1.11E-35	0.44	*Bacillus subtilis (strain 168)*	DNA gyrase subunit A		Unknown	*Escherichia coli K-12*	DNA gyrase	0	Unknown		Unknown				Unknown	18048174024; 180426161933	Unknown	
	DNA topoisomerase IV inhibitor	0.131	0.003		DNA gyrase subunit B	3.02E-21	0.44		DNA gyrase subunit B			*Staphylococcus aureus subsp. aureus Mu50*	DNA gyrase	0										
				Mycobacterium tuberculosis	DNA gyrase subunit A	5.67E-50	0.49		DNA topoisomerase IV subunit A				DNA gyrase subunit B	0										
				Staphylococcus aureus	DNA topoisomerase 4 subunit A	1.96E-08	0.45		DNA topoisomerase IV subunit B															
					DNA topoisomerase 4 subunit B	7.36E-06	0.44	*Escherichia coli*	DNA gyrase subunit A															
									DNA gyrase subunit B															
								Escherichia coli K-12	DNA gyrase subunit A															
									DNA gyrase subunit B															
									DNA topoisomerase IV subunit A															
								Pseudomonas aeruginosa (strain ATCC 15692 / PAO1 / 1C / PRS 101 / LMG12228)	DNA gyrase subunit A															
									DNA gyrase subunit B															
								Mycobacterium tuberculosis	DNA gyrase subunit B															
								Staphylococcus aureus subsp. aureus Mu50	DNA gyrase subunit A															
									DNA gyrase subunit B															
									DNA topoisomerase IV subunit A															
								Staphylococcus aureus subsp. aureus N315	DNA gyrase subunit B															
MF 5112 free base	DNA gyrase inhibitor	0.136	0.003	*Escherichia coli*	DNA gyrase subunit A	2.96E-32	0.39		Unknown		Unknown	*Escherichia coli K-12*	DNA gyrase	0	Unknown		Unknown				Unknown	18048174100; 180426162021	Unknown	
	DNA topoisomerase IV inhibitor	0.045	0.004		DNA gyrase subunit B	3.16E-19	0.39					*Staphylococcus aureus subsp. aureus Mu50*	DNA gyrase	0										
				Mycobacterium tuberculosis	DNA gyrase subunit A	9.69E-40	0.39						DNA gyrase subunit B	0										
				Staphylococcus aureus	DNA topoisomerase 4 subunit A	3.09E-07	0.39																	
MF 5126	DNA gyrase inhibitor	0.427	0.001	*Escherichia coli*	DNA gyrase subunit A	2.06E-33	0.43	*Bacillus subtilis (strain 168)*	DNA gyrase subunit A		Unknown	*Escherichia coli K-12*	DNA gyrase	0	Unknown		Unknown			*Staphylococcus aureus (strain MSSA476)*	DNA topoisomerase 4 subunit A	18048174123; 180426162131	Unknown	
	DNA topoisomerase IV inhibitor	0.098	0.003		DNA gyrase subunit B	6.56E-20	0.43		DNA gyrase subunit B			*Staphylococcus aureus subsp. aureus Mu50*	DNA gyrase subunit B	0										
				Mycobacterium tuberculosis	DNA gyrase subunit A	2.81E-49	0.49		DNA topoisomerase IV subunit A															
				Staphylococcus aureus	DNA topoisomerase 4 subunit A	2.54E-08	0.45	*Escherichia coli K-12*	DNA gyrase subunit A															
					DNA topoisomerase 4 subunit B	8.84E-06	0.43		DNA gyrase subunit B															
									DNA topoisomerase IV subunit A															
								Pseudomonas aeruginosa (strain ATCC 15692 / PAO1 / 1C / PRS 101 / LMG12228)	DNA gyrase subunit A															
									DNA gyrase subunit B															
								Staphylococcus aureus subsp. aureus Mu50	DNA gyrase subunit A															
									DNA gyrase subunit B															
									DNA topoisomerase IV subunit A															
								Staphylococcus aureus subsp. aureus N315	DNA gyrase subunit B															

Bioactive molecule	PASS online[4] Activity	Pa	Pi
MF 5137	DNA gyrase inhibitor	0.410	0.001
	DNA topoisomerase IV inhibitor	0.078	0.003
MF 5143	DNA gyrase inhibitor	0.427	0.001
	DNA topoisomerase IV inhibitor	0.144	0.003
MF 5168	DNA gyrase inhibitor	0.158	0.003
	DNA topoisomerase IV inhibitor	0.064	0.003
Miloxacin	DNA topoisomerase IV inhibitor	0.024	0.012
Moxifloxacin	DNA gyrase inhibitor	0.662	0.001
	DNA topoisomerase IV inhibitor	0.406	0.001
Nadifloxacin	DNA gyrase inhibitor	0.278	0.002
	DNA topoisomerase IV inhibitor	0.063	0.003
Nalidixic Acid	DNA gyrase inhibitor	0.073	0.004
	DNA topoisomerase IV inhibitor	0.040	0.004
Nemonoxacin	DNA gyrase inhibitor	0.416	0.001
	DNA topoisomerase IV inhibitor	0.230	0.002
Norfloxacin	DNA gyrase inhibitor	0.296	0.002
	DNA topoisomerase IV inhibitor	0.098	0.003
NSFQ 104	DNA gyrase inhibitor	0.040	0.008
	DNA topoisomerase IV inhibitor	0.039	0.004
NSFQ 105	DNA gyrase inhibitor	0.159	0.003
	DNA topoisomerase IV inhibitor	0.082	0.003
Ofloxacin	DNA gyrase inhibitor	0.408	0.001
	DNA topoisomerase IV inhibitor	0.155	0.002

Bioactive molecule	Similarity Ensemble Approach (SEA)[2] Organism	Target	P-Value	Max TC
MF 5137	*Escherichia coli*	DNA gyrase subunit A	4.14E-31	0.36
		DNA gyrase subunit B	1.50E-18	0.36
	Mycobacterium tuberculosis	DNA gyrase subunit A	1.01E-41	0.41
	Staphylococcus aureus	DNA topoisomerase 4 subunit A	4.51E-07	0.38
MF 5143	*Escherichia coli*	DNA gyrase subunit A	1.10E-32	0.42
		DNA gyrase subunit B	1.76E-19	0.42
	Mycobacterium tuberculosis	DNA gyrase subunit A	2.36E-47	0.46
	Staphylococcus aureus	DNA topoisomerase 4 subunit A	3.55E-08	0.44
MF 5168	*Escherichia coli*	DNA gyrase subunit A	5.75E-41	0.54
		DNA gyrase subunit B	2.30E-24	0.54
	Mycobacterium tuberculosis	DNA gyrase subunit A	6.65E-37	0.36
	Staphylococcus aureus	DNA topoisomerase 4 subunit A	2.67E-08	0.54
		DNA topoisomerase 4 subunit B	4.39E-07	0.54
Miloxacin	*Escherichia coli*	DNA gyrase subunit A	9.23E-09	0.32
	Mycobacterium tuberculosis	DNA gyrase subunit A	1.11E-32	0.32
	Staphylococcus aureus	DNA topoisomerase 4 subunit A	5.45E-06	0.32
Moxifloxacin	*Escherichia coli*	DNA gyrase subunit A	3.59E-33	0.44
		DNA gyrase subunit B	9.10E-20	0.44
	Mycobacterium tuberculosis	DNA gyrase subunit A	4.77E-110	1.00
	Staphylococcus aureus	DNA topoisomerase 4 subunit A	4.74E-21	1.00
		DNA topoisomerase 4 subunit B	6.09E-06	0.44
Nadifloxacin	*Escherichia coli*	DNA gyrase subunit A	2.69E-23	0.40
		DNA gyrase subunit B	6.05E-14	0.40
	Mycobacterium tuberculosis	DNA gyrase subunit A	8.95E-65	0.50
	Staphylococcus aureus	DNA topoisomerase 4 subunit A	5.02E-08	0.44
Nalidixic Acid		Unknown		
Nemonoxacin	*Escherichia coli*	DNA gyrase subunit A	3.55E-22	0.36
		DNA gyrase subunit B	2.78E-13	0.36
	Mycobacterium tuberculosis	DNA gyrase subunit A	3.73E-49	0.49
	Staphylococcus aureus	DNA topoisomerase 4 subunit A	1.27E-07	0.44
Norfloxacin	*Escherichia coli*	DNA gyrase subunit A	9.66E-50	0.67
		DNA gyrase subunit B	1.54E-29	0.67
	Mycobacterium tuberculosis	DNA gyrase subunit A	7.37E-39	0.38
	Staphylococcus aureus	DNA topoisomerase 4 subunit A	1.49E-09	0.67
		DNA topoisomerase 4 subunit B	1.43E-08	0.67
NSFQ 104	*Escherichia coli*	DNA gyrase subunit A	1.66E-44	0.42
		DNA gyrase subunit B	1.88E-26	0.42
	Mycobacterium tuberculosis	DNA gyrase subunit A	1.74E-31	0.31
	Staphylococcus aureus	DNA topoisomerase 4 subunit A	4.98E-07	0.42
NSFQ 105	*Escherichia coli*	DNA gyrase subunit A	1.48E-56	0.63
		DNA gyrase subunit B	1.47E-33	0.63
	Mycobacterium tuberculosis	DNA gyrase subunit A	1.20E-40	0.39
	Staphylococcus aureus	DNA topoisomerase 4 subunit A	1.57E-09	0.63
		DNA topoisomerase 4 subunit B	3.99E-08	0.63
Ofloxacin	*Escherichia coli*	DNA gyrase subunit A	4.29E-35	0.38
		DNA gyrase subunit B	6.68E-21	0.38
	Mycobacterium tuberculosis	DNA gyrase subunit A	5.90E-45	0.45
	Staphylococcus aureus	DNA topoisomerase 4 subunit A	2.01E-07	0.40

Bioactive molecule	ChemProt[3] Organism	Target
MF 5137	*Bacillus subtilis (strain 168)*	DNA gyrase subunit A
		DNA gyrase subunit B
		DNA topoisomerase IV subunit A
		DNA topoisomerase IV subunit B
	Escherichia coli K-12	DNA gyrase subunit A
		DNA gyrase subunit B
		DNA topoisomerase IV subunit A
	Pseudomonas aeruginosa (strain ATCC 15692 / PAO1 / 1C / PRS 101 / LMG12228)	DNA gyrase subunit A
		DNA gyrase subunit B
	Staphylococcus aureus subsp. aureus Mu50	DNA gyrase subunit A
		DNA gyrase subunit B
		DNA topoisomerase IV subunit A
	Staphylococcus aureus subsp. aureus N315	DNA gyrase subunit B
MF 5143	*Bacillus subtilis (strain 168)*	DNA gyrase subunit A
		DNA gyrase subunit B
		DNA topoisomerase IV subunit A
		DNA topoisomerase IV subunit B
	Escherichia coli K-12	DNA gyrase subunit A
		DNA gyrase subunit B
		DNA topoisomerase IV subunit A
	Pseudomonas aeruginosa (strain ATCC 15692 / PAO1 / 1C / PRS 101 / LMG12228)	DNA gyrase subunit A
		DNA gyrase subunit B
	Staphylococcus aureus subsp. aureus Mu50	DNA gyrase subunit A
		DNA gyrase subunit B
		DNA topoisomerase IV subunit A
	Staphylococcus aureus subsp. aureus N315	DNA gyrase subunit B
MF 5168	*Mycobacterium smegmatis*	DNA topoisomerase (ATP-hydrolysing) (DNA gyrase)
	Mycobacterium tuberculosis	DNA gyrase subunit B
Miloxacin	*Escherichia coli K-12*	DNA gyrase subunit A
		DNA gyrase subunit B
Moxifloxacin	*Bacillus subtilis (strain 168)*	DNA gyrase subunit A
		DNA gyrase subunit B
		DNA topoisomerase IV subunit A
		DNA topoisomerase IV subunit B
	Escherichia coli	DNA gyrase subunit A
		DNA gyrase subunit B
	Escherichia coli K-12	DNA gyrase subunit A
		DNA gyrase subunit B
		DNA topoisomerase IV subunit A
	Pseudomonas aeruginosa (strain ATCC 15692 / PAO1 / 1C / PRS 101 / LMG12228)	DNA gyrase subunit A
		DNA gyrase subunit B
	Mycobacterium tuberculosis	DNA gyrase subunit B
	Staphylococcus aureus subsp. aureus Mu50	DNA gyrase subunit A
		DNA gyrase subunit B
		DNA topoisomerase IV subunit A
	Staphylococcus aureus subsp. aureus N315	DNA gyrase subunit B
Nadifloxacin	*Escherichia coli*	DNA gyrase subunit A
		DNA gyrase subunit B
	Escherichia coli K-12	DNA gyrase subunit A
		DNA gyrase subunit B
	Mycobacterium tuberculosis	DNA gyrase subunit A
		DNA gyrase subunit B
	Staphylococcus aureus	DNA topoisomerase IV subunit A
	Staphylococcus aureus subsp. aureus Mu50	DNA gyrase subunit A
	Staphylococcus aureus subsp. aureus N315	DNA gyrase subunit B
Nalidixic Acid	*Bacillus subtilis (strain 168)*	DNA gyrase subunit A
		DNA gyrase subunit B
		DNA topoisomerase IV subunit A
	Escherichia coli	DNA gyrase subunit A
		DNA gyrase subunit B
	Escherichia coli K-12	DNA gyrase subunit A
		DNA gyrase subunit B
	Mycobacterium tuberculosis	DNA gyrase subunit B
Nemonoxacin	*Bacillus subtilis (strain 168)*	DNA gyrase subunit A
		DNA gyrase subunit B
		DNA topoisomerase IV subunit A
		DNA topoisomerase IV subunit B
	Escherichia coli K-12	DNA gyrase subunit A
		DNA gyrase subunit B
	Mycobacterium tuberculosis	DNA gyrase subunit B
	Staphylococcus aureus subsp. aureus Mu50	DNA gyrase subunit B
Norfloxacin	*Bacillus subtilis (strain 168)*	DNA gyrase subunit A
		DNA gyrase subunit B
		DNA topoisomerase IV subunit A
	Escherichia coli K-12	DNA gyrase subunit A
		DNA gyrase subunit B
		DNA topoisomerase IV subunit A
	Pseudomonas aeruginosa (strain ATCC 15692 / PAO1 / 1C / PRS 101 / LMG12228)	DNA gyrase subunit A
		DNA gyrase subunit B
	Mycobacterium tuberculosis	DNA gyrase subunit A
		DNA gyrase subunit B
	Staphylococcus aureus subsp. aureus Mu50	DNA gyrase subunit A
		DNA gyrase subunit B
		DNA topoisomerase IV subunit A
NSFQ 104		Unknown
NSFQ 105		Unknown
Ofloxacin	*Escherichia coli*	DNA gyrase subunit A
		DNA gyrase subunit B
	Escherichia coli K-12	DNA gyrase subunit A
		DNA gyrase subunit B
	Mycobacterium smegmatis	DNA topoisomerase (ATP-hydrolysing) (DNA gyrase)
	Mycobacterium tuberculosis	DNA gyrase subunit B
	Staphylococcus aureus subsp. aureus Mu50	DNA gyrase subunit A
		DNA gyrase subunit B
	Staphylococcus aureus subsp. aureus N315	DNA gyrase subunit B
	Staphylococcus aureus	DNA topoisomerase IV subunit A

Bioactive molecule	SuperPred[6] Organism	SuperPred[6] Target	Polypharmacology Browser (PPB)[1] Organism	PPB Target	PPB P-Value	SPiDER[7] Target	SPiDER Confidence level	HitPick[5] Target	HitPick Precision (%)	HitPick Tc similarity	PharmMapper[9] Organism	PharmMapper Target	PharmMapper Job Id	TargetHunter[8] Target	TargetHunter Reference
MF 5137		Unknown	*Escherichia coli K-12*	DNA gyrase	0	Unknown		Unknown				Unknown	18048161154; 180426162204	DNA gyrase	Bioorg. Med. Chem. Lett.,(1992)2:7:643; J. Med. Chem.,(1986)29:3:394; J. Med. Chem.,(1991)34:2:656; J. Med. Chem.,(1991)34:3:1155; J. Med. Chem.,(1996)39:25:4952
			Staphylococcus aureus subsp. aureus Mu50	DNA gyrase subunit B	0									DNA gyrase subunit B	J. Med. Chem.,(1992)35:25:4745
														Topoisomerase IV subunit A	Bioorg. Med. Chem. Lett.,(2004)14:20:5193; J. Med. Chem.,(2005)48:16:5232; J. Med. Chem.,(2005)48:16:5232; J. Med. Chem.,(2006)49:1:39
MF 5143		Unknown	*Escherichia coli K-12*	DNA gyrase	0	Unknown		Unknown			*Staphylococcus aureus (strain MSSA476)*	DNA topoisomerase 4 subunit A	18048174149; 180426162235	Unknown	
			Staphylococcus aureus subsp. aureus Mu50	DNA gyrase subunit B	0										
MF 5168		Unknown	*Escherichia coli K-12*	DNA gyrase	0	Unknown		Unknown				Unknown	18048161223; 180426162314	Unknown	
			Staphylococcus aureus subsp. aureus Mu50	DNA gyrase subunit B	0										
Miloxacin		Unknown	*Escherichia coli K-12*	DNA gyrase	0	Unknown		Unknown				Unknown	18048111343; 180426162446	DNA gyrase	J. Med. Chem.,(1986)29:3:394; J. Med. Chem.,(1986)29:4:445
Moxifloxacin		Unknown		Unknown		Unknown		Unknown				Unknown	18048100211; 180427223914	Unknown	
Nadifloxacin		Unknown	*Escherichia coli K-12*	DNA gyrase	0	Unknown		Unknown				Unknown	18048101549; 180427223944	Unknown	
			Staphylococcus aureus subsp. aureus Mu50	DNA gyrase subunit B	0										
Nalidixic Acid		Unknown	*Escherichia coli K-12*	DNA gyrase	0	Unknown		Unknown				Unknown	18048111426; 180427224044	DNA gyrase	Bioorg. Med. Chem. Lett.,(1992)2:7:643; Bioorg. Med. Chem. Lett.,(1993)3:2:225; Bioorg. Med. Chem. Lett.,(1998)8:1:97; J. Med. Chem.,(1986)29:3:394; J. Med. Chem.,(1986)29:4:445
Nemonoxacin		Unknown	*Escherichia coli K-12*	DNA gyrase	0	Unknown		Unknown				Unknown	18048104017; 180427224119	Unknown	
			Staphylococcus aureus subsp. aureus Mu50	DNA gyrase	0										
				DNA gyrase subunit B	0										
Norfloxacin		Unknown		Unknown		Unknown		Unknown				Unknown	180327134239	Unknown	
NSFQ 104		Unknown	*Escherichia coli K-12*	DNA gyrase	0	Unknown		Unknown				Unknown	18048161246; 180427224200	Unknown	
			Staphylococcus aureus subsp. aureus Mu50	DNA gyrase subunit B	0										
NSFQ 105		Unknown	*Staphylococcus aureus subsp. aureus Mu50*	DNA gyrase subunit B	0	Unknown		Unknown				Unknown	18048161317; 180427224222	DNA gyrase	J. Med. Chem.,(1988)31:3:503; J. Med. Chem.,(1991)34:3:1155; J. Med. Chem.,(1993)36:14:1964; J. Med. Chem.,(1993)36:22:3444; J. Med. Chem.,(1996)39:25:4952;
														DNA gyrase subunit A	Antimicrob. Agents Chemother.,(2010)54:8:3478
														DNA gyrase subunit B	J. Med. Chem.,(1992)35:25:4745
														Topoisomerase IV subunit A	Bioorg. Med. Chem. Lett.,(2006)16:5:1272; Bioorg. Med. Chem. Lett.,(2006)16:5:1277; J. Med. Chem.,(2005)48:16:5232; J. Med. Chem.,(2006)49:1:39
Ofloxacin		Unknown		Unknown		Unknown		Unknown				Unknown	18048100252; 180427224313	Unknown	

Bioactive molecule	PASS online[4]			Similarity Ensemble Approach (SEA)[2]				ChemProt[3]		SuperPred[6]		Polypharmacology Browser (PPB)[1]			SPiDER[7]		HitPick[5]			PharmMapper[9]			TargetHunter[8]	
	Activity	Pa	Pi	Organism	Target	P-Value	Max TC	Organism	Target	Organism	Target	Organism	Target	P-Value	Target	Confidence level	Target	Precision (%)	Tc similarity	Organism	Target	Job Id	Target	Reference
Olamufloxacin	DNA gyrase inhibitor	0.779	0.000	*Escherichia coli*	DNA gyrase subunit A	5.48E-21	0.33	*Bacillus subtilis (strain 168)*	DNA gyrase subunit A		Unknown	*Escherichia coli K-12*	DNA gyrase	0	Unknown		Unknown			*Staphylococcus aureus (strain MSSA476)*	DNA topoisomerase 4 subunit A	18048111456; 180427224338	Unknown	
	DNA topoisomerase IV inhibitor	0.262	0.002		DNA gyrase subunit B	1.39E-12	0.33		DNA gyrase subunit B			*Staphylococcus aureus subsp. aureus Mu50*	DNA gyrase	0.01 to 0										
				Mycobacterium tuberculosis	DNA gyrase subunit A	5.97E-39	0.38		DNA topoisomerase IV subunit A				DNA gyrase subunit B	0										
				Staphylococcus aureus	DNA topoisomerase 4 subunit A	1.30E-06	0.35		DNA topoisomerase IV subunit B															
								Escherichia coli	DNA gyrase subunit A															
									DNA gyrase subunit B															
								Escherichia coli K-12	DNA gyrase subunit A															
									DNA gyrase subunit B															
									DNA topoisomerase IV subunit A															
								Pseudomonas aeruginosa (strain ATCC 15692 / PAO1 / 1C / PRS 101 / LMG12228)	DNA gyrase subunit A															
									DNA gyrase subunit B															
								Mycobacterium tuberculosis	DNA gyrase subunit A															
									DNA gyrase subunit B															
								Staphylococcus aureus	DNA topoisomerase IV subunit A															
								Staphylococcus aureus subsp. aureus Mu50	DNA gyrase subunit A															
									DNA gyrase subunit B															
									DNA topoisomerase IV subunit A															
								Staphylococcus aureus subsp. aureus N315	DNA gyrase subunit B															
Orbifloxacin	DNA gyrase inhibitor	0.480	0.001	*Escherichia coli*	DNA gyrase subunit A	1.55E-30	0.41	*Bacillus subtilis (strain 168)*	DNA gyrase subunit A		Unknown	*Escherichia coli K-12*	DNA gyrase	0	Unknown		Unknown				Unknown	18048104107; 180427224407	Unknown	
	DNA topoisomerase IV inhibitor	0.224	0.002		DNA gyrase subunit B	3.26E-18	0.41		DNA gyrase subunit B			*Staphylococcus aureus subsp. aureus Mu50*	DNA gyrase subunit B	0										
				Mycobacterium tuberculosis	DNA gyrase subunit A	4.20E-51	0.52		DNA topoisomerase IV subunit A															
				Staphylococcus aureus	DNA topoisomerase 4 subunit A	3.33E-08	0.45		DNA topoisomerase IV subunit B															
								Escherichia coli K-12	DNA gyrase subunit A															
									DNA gyrase subunit B															
									DNA topoisomerase IV subunit A															
								Pseudomonas aeruginosa (strain ATCC 15692 / PAO1 / 1C / PRS 101 / LMG12228)	DNA gyrase subunit A															
									DNA gyrase subunit B															
								Mycobacterium tuberculosis	DNA gyrase subunit B															
								Staphylococcus aureus subsp. aureus Mu50	DNA gyrase subunit A															
									DNA gyrase subunit B															
									DNA topoisomerase IV subunit A															
								Staphylococcus aureus subsp. aureus N315	DNA gyrase subunit B															
Oxolinic acid	DNA topoisomerase IV inhibitor	0.029	0.006	*Escherichia coli*	DNA gyrase subunit A	9.23E-09	0.32	*Escherichia coli K-12*	DNA gyrase subunit A		Unknown	*Escherichia coli K-12*	DNA gyrase	0	Unknown		Unknown				Unknown	180327134329	DNA gyrase	J. Med. Chem.,(1986)29:3:394; J. Med. Chem.,(1986)29:4:445
								Escherichia coli K-12	DNA gyrase subunit B															
Ozenoxacin	DNA gyrase inhibitor	0.161	0.003	*Escherichia coli*	DNA gyrase subunit A	2.90E-25	0.34		Unknown		Unknown	*Escherichia coli K-12*	DNA gyrase	0	Unknown		Unknown				Unknown	18048111523; 180428100922	DNA gyrase	J. Med. Chem.,(1986)29:3:394; J. Med. Chem.,(1996)39:25:4952
	DNA topoisomerase IV inhibitor	0.069	0.003		DNA gyrase subunit B	4.22E-15	0.34					*Staphylococcus aureus subsp. aureus Mu50*	DNA gyrase subunit B	0.01 to 0										
				Mycobacterium tuberculosis	DNA gyrase subunit A	1.33E-38	0.38					*Staphylococcus aureus*	Topoisomerase IV subunit A	0										
				Staphylococcus aureus	DNA topoisomerase 4 subunit A	1.06E-06	0.35																	
Pazufloxacin	DNA gyrase inhibitor	0.311	0.002	*Escherichia coli*	DNA gyrase subunit A	2.56E-21	0.36		Unknown		Unknown	*Escherichia coli K-12*	DNA gyrase	0	Unknown		Unknown				Unknown	18048101631; 180428100947	DNA gyrase	Bioorg. Med. Chem. Lett.,(1998)8:1:97; Bioorg. Med. Chem. Lett.,(2008)18:3:1229; J. Med. Chem.,(1993)36:14:1964; J. Med. Chem.,(1991)34:3:1142; J. Med. Chem.,(1988)31:3:503
	DNA topoisomerase IV inhibitor	0.058	0.003		DNA gyrase subunit B	8.90E-13	0.36					*Staphylococcus aureus subsp. aureus Mu50*	DNA gyrase	0.01 to 0									DNA gyrase subunit A	Antimicrob. Agents Chemother.,(2010)54:8:3478
				Mycobacterium tuberculosis	DNA gyrase subunit A	3.33E-39	0.39						DNA gyrase subunit B	0									DNA gyrase subunit B	J. Med. Chem.,(1992)35:25:4745
												Staphylococcus aureus	Topoisomerase IV subunit A	0.03										
PD 111834	DNA gyrase inhibitor	0.070	0.004	*Escherichia coli*	DNA gyrase subunit A	4.62E-09	0.33	*Escherichia coli K-12*	DNA gyrase subunit A		Unknown	*Escherichia coli K-12*	DNA gyrase	0	Unknown		Unknown				Unknown	18049133214	Unknown	
	DNA topoisomerase IV inhibitor	0.051	0.003	*Mycobacterium tuberculosis*	DNA gyrase subunit A	1.99E-36	0.35		DNA gyrase subunit B			*Staphylococcus aureus subsp. aureus Mu50*	DNA gyrase subunit B	0										
				Staphylococcus aureus	DNA topoisomerase 4 subunit A	1.78E-06	0.34	*Staphylococcus aureus subsp. aureus Mu50*	DNA gyrase subunit B															
PD 112388	DNA gyrase inhibitor	0.034	0.013	*Escherichia coli*	DNA gyrase subunit A	2.03E-25	0.38	*Escherichia coli K-12*	DNA gyrase subunit A		Unknown	*Escherichia coli K-12*	DNA gyrase	0	Unknown		Unknown				Unknown	18049133249	DNA gyrase	J. Med. Chem.,(1986)29:3:394; J. Med. Chem.,(1991)34:2:656; J. Med. Chem.,(1993)36:14:1964; J. Med. Chem.,(1993)36:7:871; J. Med. Chem.,(2003)46:17:3655
	DNA topoisomerase IV inhibitor	0.029	0.006		DNA gyrase subunit B	3.44E-15	0.38		DNA gyrase subunit B			*Staphylococcus aureus subsp. aureus Mu50*	DNA gyrase subunit B	0									DNA gyrase subunit B	J. Med. Chem.,(1992)35:25:4745
				Mycobacterium tuberculosis	DNA gyrase subunit A	1.90E-31	0.30																Topoisomerase IV subunit A	Bioorg. Med. Chem. Lett.,(2006)16:5:1272
				Staphylococcus aureus	DNA topoisomerase 4 subunit A	1.78E-06	0.38																	
PD 114111	DNA gyrase inhibitor	0.324	0.002	*Escherichia coli*	DNA gyrase subunit A	8.39E-48	0.50	*Escherichia coli K-12*	DNA gyrase subunit A		Unknown	*Escherichia coli K-12*	DNA gyrase	0	Unknown		Unknown			*Staphylococcus aureus (strain MSSA476)*	DNA topoisomerase 4 subunit A	18049133317	Unknown	
	DNA topoisomerase IV inhibitor	0.059	0.003		DNA gyrase subunit B	7.99E-28	0.50		DNA gyrase subunit B			*Staphylococcus aureus subsp. aureus Mu50*	DNA gyrase subunit B	0										
				Mycobacterium tuberculosis	DNA gyrase subunit A	3.60E-34	0.32	*Mycobacterium smegmatis*	DNA topoisomerase (ATP-hydrolysing) (DNA gyrase)															
				Staphylococcus aureus	DNA topoisomerase 4 subunit A	8.58E-08	0.50	*Staphylococcus aureus subsp. aureus Mu50*	DNA gyrase subunit A															
					DNA topoisomerase 4 subunit B	1.34E-06	0.50		DNA gyrase subunit B															
								Staphylococcus aureus subsp. aureus N315	DNA gyrase subunit B															
PD 115311	DNA gyrase inhibitor	0.148	0.003	*Escherichia coli*	DNA gyrase subunit A	5.51E-09	0.33	*Escherichia coli K-12*	DNA gyrase subunit A		Unknown	*Escherichia coli K-12*	DNA gyrase	0	Unknown		Unknown				Unknown	18048204640; 180428101016	DNA gyrase	J. Med. Chem.,(1986)29:3:394; J. Med. Chem.,(1988)31:3:503; J. Med. Chem.,(1986)29:4:445; J. Med. Chem.,(1991)34:3:1142; J. Med. Chem.,(1991)34:3:1155
	DNA topoisomerase IV inhibitor	0.059	0.003	*Mycobacterium tuberculosis*	DNA gyrase subunit A	1.17E-40	0.39		DNA gyrase subunit B			*Staphylococcus aureus subsp. aureus Mu50*	DNA gyrase subunit B	0									DNA gyrase subunit A	Antimicrob. Agents Chemother.,(2010)54:8:3478
				Staphylococcus aureus	DNA topoisomerase 4 subunit A	7.36E-07	0.38																DNA gyrase subunit B	J. Med. Chem.,(1992)35:25:4745
PD 116507	DNA gyrase inhibitor	0.257	0.002	*Escherichia coli*	DNA gyrase subunit A	5.87E-21	0.35	*Escherichia coli*	DNA gyrase subunit A		Unknown	*Escherichia coli K-12*	DNA gyrase	0	Unknown		Unknown				Unknown	18049133344	Unknown	
	DNA topoisomerase IV inhibitor	0.149	0.002		DNA gyrase subunit B	1.45E-12	0.35		DNA gyrase subunit B			*Staphylococcus aureus subsp. aureus Mu50*	DNA gyrase subunit B	0										
				Mycobacterium tuberculosis	DNA gyrase subunit A	3.95E-40	0.38	*Escherichia coli K-12*	DNA gyrase subunit A															
				Staphylococcus aureus	DNA topoisomerase 4 subunit A	6.06E-07	0.37		DNA gyrase subunit B															
								Mycobacterium tuberculosis	DNA gyrase subunit A															
									DNA gyrase subunit B															
								Staphylococcus aureus subsp. aureus Mu50	DNA gyrase subunit B															
PD 117596	DNA gyrase inhibitor	0.730	0.000	*Escherichia coli*	DNA gyrase subunit A	2.88E-36	0.48	*Bacillus subtilis (strain 168)*	DNA gyrase subunit A		Unknown	*Escherichia coli K-12*	DNA gyrase	0	Unknown		Unknown				Unknown	18048180616; 180428101055	Unknown	
	DNA topoisomerase IV inhibitor	0.402	0.001		DNA gyrase subunit B	1.36E-21	0.48		DNA gyrase subunit B			*Staphylococcus aureus subsp. aureus Mu50*	DNA gyrase subunit B	0										
				Mycobacterium tuberculosis	DNA gyrase subunit A	2.68E-79	0.60		DNA topoisomerase IV subunit A															
				Staphylococcus aureus	DNA topoisomerase 4 subunit A	1.92E-09	0.53	*Escherichia coli*	DNA gyrase subunit A															
					DNA topoisomerase 4 subunit B	2.57E-06	0.48		DNA gyrase subunit B															
								Escherichia coli K-12	DNA gyrase subunit A															
									DNA gyrase subunit B															
									DNA topoisomerase IV subunit A															
								Pseudomonas aeruginosa (strain ATCC 15692 / PAO1 / 1C / PRS 101 / LMG12228)	DNA gyrase subunit A															
									DNA gyrase subunit B															
								Mycobacterium tuberculosis	DNA gyrase subunit A															
									DNA gyrase subunit B															
								Staphylococcus aureus	DNA topoisomerase IV subunit A															
								Staphylococcus aureus subsp. aureus Mu50	DNA gyrase subunit A															
									DNA gyrase subunit B															
									DNA topoisomerase IV subunit A															
								Staphylococcus aureus subsp. aureus N315	DNA gyrase subunit B															
PD 118362	DNA gyrase inhibitor	0.559	0.001	*Escherichia coli*	DNA gyrase subunit A	3.30E-33	0.40	*Escherichia coli*	DNA gyrase subunit A		Unknown	*Escherichia coli K-12*	DNA gyrase	0	Unknown		Unknown			*Staphylococcus aureus (strain MSSA476)*	DNA topoisomerase 4 subunit A	18049133410	Unknown	
	DNA topoisomerase IV inhibitor	0.269	0.002		DNA gyrase subunit B	8.66E-20	0.40		DNA gyrase subunit B			*Staphylococcus aureus subsp. aureus Mu50*	DNA gyrase subunit B	0										
				Mycobacterium tuberculosis	DNA gyrase subunit A	3.53E-69	0.51	*Escherichia coli K-12*	DNA gyrase subunit A															
				Staphylococcus aureus	DNA topoisomerase 4 subunit A	2.52E-08	0.47		DNA gyrase subunit B															
								Pseudomonas aeruginosa (strain ATCC 15692 / PAO1 / 1C / PRS 101 / LMG12228)	DNA gyrase subunit A															
									DNA gyrase subunit B															
								Mycobacterium tuberculosis	DNA gyrase subunit A															
									DNA gyrase subunit B															
								Staphylococcus aureus subsp. aureus Mu50	DNA gyrase subunit A															
									DNA gyrase subunit B															
									DNA topoisomerase IV subunit A															
								Staphylococcus aureus subsp. aureus N315	DNA gyrase subunit B															
PD 119344	DNA gyrase inhibitor	0.238	0.002	*Escherichia coli*	DNA gyrase subunit A	3.37E-09	0.33	*Bacillus subtilis (strain 168)*	DNA gyrase subunit A		Unknown	*Escherichia coli K-12*	DNA gyrase	0	Unknown		Unknown			*Staphylococcus aureus (strain MSSA476)*	DNA topoisomerase 4 subunit A	18049133433	Unknown	
	DNA topoisomerase IV inhibitor	0.095	0.003	*Mycobacterium tuberculosis*	DNA gyrase subunit A	1.39E-58	0.42		DNA gyrase subunit B			*Staphylococcus aureus subsp. aureus Mu50*	DNA gyrase subunit B	0										
				Staphylococcus aureus	DNA topoisomerase 4 subunit A	5.59E-07	0.39		DNA topoisomerase IV subunit A															
									DNA topoisomerase IV subunit B															
								Escherichia coli	DNA gyrase subunit A															
									DNA gyrase subunit B															
								Escherichia coli K-12	DNA gyrase subunit A															
									DNA gyrase subunit B															
									DNA topoisomerase IV subunit A															
								Pseudomonas aeruginosa (strain ATCC 15692 / PAO1 / 1C / PRS 101 / LMG12228)	DNA gyrase subunit A															
									DNA gyrase subunit B															
								Mycobacterium tuberculosis	DNA gyrase subunit A															
									DNA gyrase subunit B															
								Staphylococcus aureus	DNA topoisomerase IV subunit A															
								Staphylococcus aureus subsp. aureus Mu50	DNA gyrase subunit A															
									DNA gyrase subunit B															
								Staphylococcus aureus subsp. aureus N315	DNA gyrase subunit B															
PD 129626	DNA gyrase inhibitor	0.337	0.002	*Escherichia coli*	DNA gyrase subunit A	6.05E-32	0.39	*Escherichia coli K-12*	DNA gyrase subunit A		Unknown	*Escherichia coli K-12*	DNA gyrase	0	Unknown		Unknown				Unknown	18049133459	Unknown	
	DNA topoisomerase IV inhibitor	0.124	0.003		DNA gyrase subunit B	4.81E-19	0.39		DNA gyrase subunit B			*Staphylococcus aureus subsp. aureus Mu50*	DNA gyrase subunit B	0										
				Mycobacterium tuberculosis	DNA gyrase subunit A	4.86E-66	0.50	*Staphylococcus aureus subsp. aureus Mu50*	DNA gyrase subunit B			*Staphylococcus aureus*	Topoisomerase IV subunit A	0.01 to 0										
				Staphylococcus aureus	DNA topoisomerase 4 subunit A	5.28E-08	0.45																	

Bioactive molecule	PASS online[4]			Similarity Ensemble Approach (SEA)[2]				ChemProt[3]		SuperPred[6]		Polypharmacology Browser (PPB)[1]			SPiDER[7]		HitPick[5]			PharmMapper[9]			TargetHunter[8]	
	Activity	Pa	Pi	Organism	Target	P-Value	Max TC	Organism	Target	Organism	Target	Organism	Target	P-Value	Target	Confidence level	Target	Precision (%)	Tc similarity	Organism	Target	Job Id	Target	Reference
PD 131628	DNA gyrase inhibitor	0.644	0.001	*Escherichia coli*	DNA gyrase subunit A	7.17E-35	0.47	*Bacillus subtilis (strain 168)*	DNA gyrase subunit A		Unknown	*Escherichia coli K-12*	DNA gyrase	0	Unknown		Unknown				Unknown	18048142802	Unknown	
	DNA topoisomerase IV inhibitor	0.323	0.002		DNA gyrase subunit B	9.05E-21	0.47		DNA gyrase subunit B			*Staphylococcus aureus subsp. aureus Mu50*	DNA gyrase	0										
				Mycobacterium tuberculosis	DNA gyrase subunit A	1.33E-48	0.48		DNA topoisomerase IV subunit A				DNA gyrase subunit B	0.01 to 0										
				Staphylococcus aureus	DNA topoisomerase 4 subunit A	1.33E-08	0.47		DNA topoisomerase IV subunit B															
					DNA topoisomerase 4 subunit B	3.14E-06	0.47	*Escherichia coli*	DNA gyrase subunit A															
									DNA gyrase subunit B															
								Escherichia coli K-12	DNA gyrase subunit A															
									DNA gyrase subunit B															
									DNA topoisomerase IV subunit A															
								Pseudomonas aeruginosa (strain ATCC 15692 / PAO1 / 1C / PRS 101 / LMG12228)	DNA gyrase subunit A															
									DNA gyrase subunit B															
								Mycobacterium tuberculosis	DNA gyrase subunit A															
									DNA gyrase subunit B															
								Staphylococcus aureus subsp. aureus Mu50	DNA gyrase subunit A															
									DNA gyrase subunit B															
									DNA topoisomerase IV subunit A															
								Staphylococcus aureus subsp. aureus N315	DNA gyrase subunit B															
PD 135042 (AM 1147)	DNA gyrase inhibitor	0.575	0.001	*Escherichia coli*	DNA gyrase subunit A	4.44E-37	0.61	*Bacillus subtilis (strain 168)*	DNA gyrase subunit A		Unknown	*Staphylococcus aureus subsp. aureus Mu50*	DNA gyrase	0	Unknown		Unknown				Unknown	18048174223; 180428101130	Unknown	
	DNA topoisomerase IV inhibitor	0.393	0.001		DNA gyrase subunit B	4.51E-22	0.61		DNA gyrase subunit B			*Staphylococcus aureus subsp. aureus Mu50*	DNA gyrase subunit B	0										
				Mycobacterium tuberculosis	DNA gyrase subunit A	2.48E-100	0.81		DNA topoisomerase IV subunit A															
				Staphylococcus aureus	DNA topoisomerase 4 subunit A	2.41E-17	0.70	*Escherichia coli*	DNA gyrase subunit A															
					DNA topoisomerase 4 subunit B	6.68E-08	0.61		DNA gyrase subunit B															
								Escherichia coli K-12	DNA gyrase subunit A															
									DNA gyrase subunit B															
									DNA topoisomerase IV subunit A															
								Pseudomonas aeruginosa (strain ATCC 15692 / PAO1 / 1C / PRS 101 / LMG12228)	DNA gyrase subunit A															
									DNA gyrase subunit B															
								Mycobacterium tuberculosis	DNA gyrase subunit B															
								Staphylococcus aureus subsp. aureus Mu50	DNA gyrase subunit A															
									DNA gyrase subunit B															
								Staphylococcus aureus subsp. aureus N315	DNA gyrase subunit B															
PD 135144 (BMY 33315)	DNA gyrase inhibitor	0.152	0.003	*Escherichia coli*	DNA gyrase subunit A	6.60E-07	0.46	*Bacillus subtilis (strain 168)*	DNA gyrase subunit A		Unknown	*Escherichia coli K-12*	DNA gyrase	0	Unknown		Unknown				Unknown	18048174301; 180428101153	Unknown	
	DNA topoisomerase IV inhibitor	0.093	0.003	*Mycobacterium tuberculosis*	DNA gyrase subunit A	9.31E-41	0.41		DNA gyrase subunit B			*Staphylococcus aureus subsp. aureus Mu50*	DNA gyrase subunit B	0										
				Staphylococcus aureus	DNA topoisomerase 4 subunit A	8.10E-08	0.46		DNA topoisomerase IV subunit A															
					DNA topoisomerase 4 subunit B	3.95E-06	0.46		DNA topoisomerase IV subunit B															
								Escherichia coli	DNA gyrase subunit A															
									DNA gyrase subunit B															
								Escherichia coli K-12	DNA gyrase subunit A															
									DNA gyrase subunit B															
									DNA topoisomerase IV subunit A															
								Pseudomonas aeruginosa (strain ATCC 15692 / PAO1 / 1C / PRS 101 / LMG12228)	DNA gyrase subunit A															
									DNA gyrase subunit B															
								Mycobacterium tuberculosis	DNA gyrase subunit A															
									DNA gyrase subunit B															
								Staphylococcus aureus subsp. aureus Mu50	DNA gyrase subunit A															
									DNA gyrase subunit B															
								Staphylococcus aureus subsp. aureus N315	DNA gyrase subunit B															
PD 137156	DNA gyrase inhibitor	0.559	0.001	*Escherichia coli*	DNA gyrase subunit A	4.24E-36	0.50	*Bacillus subtilis (strain 168)*	DNA gyrase subunit A		Unknown	*Escherichia coli K-12*	DNA gyrase	0	Unknown		Unknown				Unknown	18049133521	Unknown	
	DNA topoisomerase IV inhibitor	0.112	0.003		DNA gyrase subunit B	1.71E-21	0.50		DNA gyrase subunit B			*Staphylococcus aureus subsp. aureus Mu50*	DNA gyrase subunit B	0										
				Mycobacterium tuberculosis	DNA gyrase subunit A	7.06E-47	0.51		DNA topoisomerase IV subunit A															
				Staphylococcus aureus	DNA topoisomerase 4 subunit A	2.48E-08	0.50		DNA topoisomerase IV subunit B															
					DNA topoisomerase 4 subunit B	1.34E-06	0.50	*Escherichia coli*	DNA gyrase subunit A															
									DNA gyrase subunit B															
								Escherichia coli K-12	DNA gyrase subunit A															
									DNA gyrase subunit B															
									DNA topoisomerase IV subunit A															
								Pseudomonas aeruginosa (strain ATCC 15692 / PAO1 / 1C / PRS 101 / LMG12228)	DNA gyrase subunit A															
									DNA gyrase subunit B															
								Mycobacterium tuberculosis	DNA gyrase subunit A															
									DNA gyrase subunit B															
								Staphylococcus aureus subsp. aureus Mu50	DNA gyrase subunit A															
									DNA gyrase subunit B															
								Staphylococcus aureus subsp. aureus N315	DNA gyrase subunit B															
PD 138312	DNA gyrase inhibitor	0.628	0.001	*Escherichia coli*	DNA gyrase subunit A	1.54E-32	0.43	*Escherichia coli K-12*	DNA gyrase subunit A		Unknown	*Escherichia coli K-12*	DNA gyrase	0	Unknown		Unknown				Unknown	18048142832	Unknown	
	DNA topoisomerase IV inhibitor	0.159	0.002		DNA gyrase subunit B	2.15E-19	0.43		DNA gyrase subunit B			*Staphylococcus aureus subsp. aureus Mu50*	DNA gyrase	0										
				Mycobacterium tuberculosis	DNA gyrase subunit A	3.42E-71	0.49	*Pseudomonas aeruginosa (strain ATCC 15692 / PAO1 / 1C / PRS 101 / LMG12228)*	DNA gyrase subunit A				DNA gyrase subunit B	0										
				Staphylococcus aureus	DNA topoisomerase 4 subunit A	2.22E-08	0.45		DNA gyrase subunit B															
					DNA topoisomerase 4 subunit B	7.92E-06	0.43	*Staphylococcus aureus subsp. aureus Mu50*	DNA gyrase subunit A															
									DNA gyrase subunit B															
									DNA topoisomerase IV subunit A															
								Staphylococcus aureus subsp. aureus N315	DNA gyrase subunit B															
PD 140248	DNA gyrase inhibitor	0.437	0.001	*Escherichia coli*	DNA gyrase subunit A	1.95E-38	0.36	*Escherichia coli K-12*	DNA gyrase subunit A		Unknown	*Staphylococcus aureus subsp. aureus Mu50*	DNA gyrase	0	Unknown		Unknown				Unknown	18048142911	DNA gyrase	J. Med. Chem.,(1993)36:7:871; J. Med. Chem.,(1993)36:14:1964; J. Med. Chem.,(1996)39:16:3070; J. Med. Chem.,(1991)34:2:656; J. Med. Chem.,(2003)46:17:3655
	DNA topoisomerase IV inhibitor	0.441	0.001		DNA gyrase subunit B	7.15E-23	0.36		DNA gyrase subunit B				DNA gyrase subunit B	0									DNA gyrase subunit B	J. Med. Chem.,(1992)35:25:4745
				Mycobacterium tuberculosis	DNA gyrase subunit A	1.32E-37	0.36	*Pseudomonas aeruginosa (strain ATCC 15692 / PAO1 / 1C / PRS 101 / LMG12228)*	DNA gyrase subunit A															
				Staphylococcus aureus	DNA topoisomerase 4 subunit A	7.53E-07	0.36		DNA gyrase subunit B															
								Staphylococcus aureus subsp. aureus Mu50	DNA gyrase subunit A															
									DNA gyrase subunit B															
									DNA topoisomerase IV subunit A															
								Staphylococcus aureus subsp. aureus N315	DNA gyrase subunit B															
PD 163449	DNA gyrase inhibitor	0.322	0.002	*Escherichia coli*	DNA gyrase subunit A	2.04E-37	0.64	*Bacillus subtilis (strain 168)*	DNA gyrase subunit A		Unknown	*Escherichia coli K-12*	DNA gyrase	0	Unknown		Unknown				Unknown	18048200235; 180428101238	Unknown	
	DNA topoisomerase IV inhibitor	0.114	0.003		DNA gyrase subunit B	2.85E-22	0.64		DNA gyrase subunit B			*Staphylococcus aureus subsp. aureus Mu50*	DNA gyrase	0										
				Mycobacterium tuberculosis	DNA gyrase subunit A	5.04E-76	0.61		DNA topoisomerase IV subunit A				DNA gyrase subunit B	0										
				Staphylococcus aureus	DNA topoisomerase 4 subunit A	5.69E-11	0.64		DNA topoisomerase IV subunit B															
					DNA topoisomerase 4 subunit B	2.74E-08	0.64	*Escherichia coli*	DNA gyrase subunit A															
									DNA gyrase subunit B															
								Escherichia coli K-12	DNA gyrase subunit A															
									DNA gyrase subunit B															
									DNA topoisomerase IV subunit A															
								Pseudomonas aeruginosa (strain ATCC 15692 / PAO1 / 1C / PRS 101 / LMG12228)	DNA gyrase subunit A															
									DNA gyrase subunit B															
								Mycobacterium tuberculosis	DNA gyrase subunit A															
									DNA gyrase subunit B															
								Staphylococcus aureus	DNA topoisomerase IV subunit A															
								Staphylococcus aureus subsp. aureus Mu50	DNA gyrase subunit A															
									DNA gyrase subunit B															
									DNA topoisomerase IV subunit A															
								Staphylococcus aureus subsp. aureus N315	DNA gyrase subunit B															
PD 164488	DNA gyrase inhibitor	0.404	0.001	*Escherichia coli*	DNA gyrase subunit A	1.25E-45	0.58	*Bacillus subtilis (strain 168)*	DNA gyrase subunit A		Unknown	*Escherichia coli K-12*	DNA gyrase	0	Unknown		Unknown			*Staphylococcus aureus (strain MSSA476)*	DNA topoisomerase 4 subunit A	18048200320; 180428101303	Unknown	
	DNA topoisomerase IV inhibitor	0.334	0.002		DNA gyrase subunit B	4.10E-27	0.58		DNA gyrase subunit B			*Staphylococcus aureus subsp. aureus Mu50*	DNA gyrase subunit B	0										
				Mycobacterium tuberculosis	DNA gyrase subunit A	9.90E-82	0.66		DNA topoisomerase IV subunit A															
				Staphylococcus aureus	DNA topoisomerase 4 subunit A	8.69E-11	0.58		DNA topoisomerase IV subunit B															
					DNA topoisomerase 4 subunit B	1.49E-07	0.58	*Escherichia coli*	DNA gyrase subunit A															
									DNA gyrase subunit B															
								Escherichia coli K-12	DNA gyrase subunit A															
									DNA gyrase subunit B															
									DNA topoisomerase IV subunit A															
								Pseudomonas aeruginosa (strain ATCC 15692 / PAO1 / 1C / PRS 101 / LMG12228)	DNA gyrase subunit A															
									DNA gyrase subunit B															
								Mycobacterium tuberculosis	DNA gyrase subunit A															
									DNA gyrase subunit B															
								Staphylococcus aureus subsp. aureus Mu50	DNA gyrase subunit A															
									DNA gyrase subunit B															
								Staphylococcus aureus subsp. aureus N315	DNA gyrase subunit B															
Pefloxacin	DNA gyrase inhibitor	0.163	0.003	*Escherichia coli*	DNA gyrase subunit A	2.38E-50	0.51	*Escherichia coli*	DNA gyrase subunit A		Unknown	*Escherichia coli K-12*	DNA gyrase	0	Unknown		Unknown			*Staphylococcus aureus (strain MSSA476)*	DNA topoisomerase 4 subunit A	180327134414	Unknown	
	DNA topoisomerase IV inhibitor	0.074	0.003		DNA gyrase subunit B	6.73E-30	0.51		DNA gyrase subunit B			*Staphylococcus aureus subsp. aureus Mu50*	DNA gyrase subunit B	0										
				Mycobacterium tuberculosis	DNA gyrase subunit A	1.30E-34	0.33	*Mycobacterium tuberculosis*	DNA gyrase subunit A															
				Staphylococcus aureus	DNA topoisomerase 4 subunit A	6.66E-08	0.51		DNA gyrase subunit B															
					DNA topoisomerase 4 subunit B	1.07E-06	0.51	*Pseudomonas aeruginosa (strain ATCC 15692 / PAO1 / 1C / PRS 101 / LMG12228)*	DNA gyrase subunit A															
									DNA gyrase subunit B															
								Staphylococcus aureus subsp. aureus Mu50	DNA gyrase subunit A															
									DNA gyrase subunit B															

Bioactive molecule	PASS online[4]			Similarity Ensemble Approach (SEA)[2]				ChemProt[3]		SuperPred[6]		Polypharmacology Browser (PPB)[1]			SPiDER[7]		HitPick[5]			PharmMapper[9]			TargetHunter[8]	
	Activity	Pa	Pi	Organism	Target	P-Value	Max TC	Organism	Target	Organism	Target	Organism	Target	P-Value	Target	Confidence level	Target	Precision (%)	Tc similarity	Organism	Target	Job Id	Target	Reference
Pipemidic acid	DNA gyrase inhibitor	0.045	0.006	*Mycobacterium tuberculosis*	DNA gyrase subunit A	2.58E-33	0.32	*Escherichia coli*	DNA gyrase subunit A		Unknown	*Escherichia coli K-12*	DNA gyrase	0	Unknown		Unknown				Unknown	18048100347; 180428101342	DNA gyrase	J. Med. Chem.,(1986)29:3:394; J. Med. Chem.,(1991)34:2:656; J. Med. Chem.,(1993)36:14:1964; J. Med. Chem.,(1993)36:7:871; J. Med. Chem.,(2003)46:17:3655
	DNA topoisomerase IV inhibitor	0.034	0.005	*Staphylococcus aureus*	DNA topoisomerase 4 subunit A	1.87E-06	0.36		DNA gyrase subunit B			*Staphylococcus aureus subsp. aureus Mu50*	DNA gyrase	0									DNA gyrase subunit B	J. Med. Chem.,(1992)35:25:4745
								Escherichia coli K-12	DNA gyrase subunit A				DNA gyrase subunit B	0										
									DNA gyrase subunit B															
								Mycobacterium tuberculosis	DNA gyrase subunit A															
									DNA gyrase subunit B															
								Staphylococcus aureus subsp. aureus Mu50	DNA gyrase subunit B															
Piromidic acid	DNA gyrase inhibitor	0.044	0.006	*Mycobacterium tuberculosis*	DNA gyrase subunit A	6.14E-31	0.29	*Escherichia coli K-12*	DNA gyrase subunit A		Unknown	*Escherichia coli K-12*	DNA gyrase	0	Unknown		Unknown				Unknown	18048100545; 180428101406	DNA gyrase	J. Med. Chem.,(1986)29:3:394; J. Med. Chem.,(1992)35:25:4745; J. Med. Chem.,(1992)35:10:1764; J. Med. Chem.,(1993)36:7:871; J. Med. Chem.,(2003)46:17:3655
	DNA topoisomerase IV inhibitor	0.034	0.005						DNA gyrase subunit B			*Staphylococcus aureus subsp. aureus Mu50*	DNA gyrase subunit B	0									DNA gyrase subunit B	J. Med. Chem.,(1992)35:25:4745
Piroxacin	DNA gyrase inhibitor	0.075	0.004		Unknown			*Escherichia coli K-12*	DNA gyrase subunit A		Unknown	*Escherichia coli K-12*	DNA gyrase	0	Unknown		Unknown			*Staphylococcus aureus (strain MSSA476)*	DNA topoisomerase 4 subunit A	18048132928; 180428101432	Unknown	
	DNA topoisomerase IV inhibitor	0.043	0.004						DNA gyrase subunit B			*Staphylococcus aureus subsp. aureus Mu50*	DNA gyrase subunit B	0										
Pradofloxacin	DNA gyrase inhibitor	0.428	0.001	*Escherichia coli*	DNA gyrase subunit A	6.46E-32	0.44	*Bacillus subtilis (strain 168)*	DNA gyrase subunit A		Unknown	*Escherichia coli K-12*	DNA gyrase	0	Unknown		Unknown			*Staphylococcus aureus (strain MSSA476)*	DNA topoisomerase 4 subunit A	18048104156; 180428101457	Unknown	
	DNA topoisomerase IV inhibitor	0.234	0.002		DNA gyrase subunit B	5.00E-19	0.44		DNA gyrase subunit B			*Staphylococcus aureus subsp. aureus Mu50*	DNA gyrase	0										
				Mycobacterium tuberculosis	DNA gyrase subunit A	5.78E-82	0.75		DNA topoisomerase IV subunit A				DNA gyrase subunit B	0										
				Staphylococcus aureus	DNA topoisomerase 4 subunit A	1.35E-13	0.75		DNA topoisomerase IV subunit B															
					DNA topoisomerase 4 subunit B	7.19E-06	0.44	*Escherichia coli*	DNA gyrase subunit A															
									DNA gyrase subunit B															
								Escherichia coli K-12	DNA gyrase subunit A															
									DNA gyrase subunit B															
									DNA topoisomerase IV subunit A															
								Pseudomonas aeruginosa (strain ATCC 15692 / PAO1 / 1C / PRS 101 / LMG12228)	DNA gyrase subunit A															
									DNA gyrase subunit B															
								Mycobacterium tuberculosis	DNA gyrase subunit A															
									DNA gyrase subunit B															
								Staphylococcus aureus subsp. aureus Mu50	DNA gyrase subunit A															
									DNA gyrase subunit B															
									DNA topoisomerase IV subunit A															
								Staphylococcus aureus subsp. aureus N315	DNA gyrase subunit B															
Premafloxacin	DNA gyrase inhibitor	0.701	0.000	*Escherichia coli*	DNA gyrase subunit A	5.79E-33	0.41	*Escherichia coli*	DNA gyrase subunit A		Unknown	*Escherichia coli K-12*	DNA gyrase	0	Unknown		Unknown				Unknown	18048104232; 180428111357	Unknown	
	DNA topoisomerase IV inhibitor	0.416	0.001		DNA gyrase subunit B	1.21E-19	0.41		DNA gyrase subunit B			*Staphylococcus aureus subsp. aureus Mu50*	DNA gyrase	0										
				Mycobacterium tuberculosis	DNA gyrase subunit A	2.45E-88	0.68	*Escherichia coli K-12*	DNA gyrase subunit A				DNA gyrase subunit B	0										
				Staphylococcus aureus	DNA topoisomerase 4 subunit A	1.60E-09	0.60		DNA gyrase subunit B															
								Pseudomonas aeruginosa (strain ATCC 15692 / PAO1 / 1C / PRS 101 / LMG12228)	DNA gyrase subunit A															
									DNA gyrase subunit B															
								Mycobacterium tuberculosis	DNA gyrase subunit B															
								Staphylococcus aureus subsp. aureus Mu50	DNA gyrase subunit A															
									DNA gyrase subunit B															
									DNA topoisomerase IV subunit A															
								Staphylococcus aureus subsp. aureus N315	DNA gyrase subunit B															
Prulifloxacin	DNA topoisomerase IV inhibitor	0.027	0.008	*Escherichia coli*	DNA gyrase subunit A	2.63E-38	0.33		Unknown		Unknown		Unknown		Unknown		Unknown			*Staphylococcus aureus (strain MSSA476)*	DNA topoisomerase 4 subunit A	18048101710; 180428111426	Unknown	
					DNA gyrase subunit B	8.52E-23	0.33																	
PubChem CID-11531032	DNA gyrase inhibitor	0.614	0.001	*Mycobacterium tuberculosis*	DNA gyrase subunit A	4.14E-24	0.45	*Escherichia coli K-12*	DNA gyrase subunit A		Unknown	*Escherichia coli K-12*	DNA gyrase	0	Unknown		Unknown				Unknown	18049154946	DNA gyrase	Bioorg. Med. Chem. Lett.,(2006)16:5:1272
	DNA topoisomerase IV inhibitor	0.920	0.000	*Staphylococcus aureus*	DNA topoisomerase 4 subunit A	5.46E-110	0.78		DNA gyrase subunit B			*Staphylococcus aureus*	Topoisomerase IV subunit A	0									DNA gyrase subunit A	Antimicrob. Agents Chemother.,(2010)54:8:3478
																							Topoisomerase IV subunit A	Bioorg. Med. Chem. Lett.,(2006)16:5:1272; Bioorg. Med. Chem. Lett.,(2006)16:5:1277
PubChem CID-11566845	DNA gyrase inhibitor	0.596	0.001	*Escherichia coli*	DNA gyrase subunit A	1.09E-09	0.34	*Escherichia coli K-12*	DNA gyrase subunit A		Unknown	*Escherichia coli K-12*	DNA gyrase	0	Unknown		Unknown				Unknown	18049155016	DNA gyrase	Bioorg. Med. Chem. Lett.,(2006)16:5:1272
	DNA topoisomerase IV inhibitor	0.904	0.000		DNA gyrase subunit B	6.43E-06	0.34		DNA gyrase subunit B			*Staphylococcus aureus*	Topoisomerase IV subunit A	0									DNA gyrase subunit A	Antimicrob. Agents Chemother.,(2010)54:8:3478
				Mycobacterium tuberculosis	DNA gyrase subunit A	4.14E-24	0.45																Topoisomerase IV subunit A	Bioorg. Med. Chem. Lett.,(2006)16:5:1272; Bioorg. Med. Chem. Lett.,(2006)16:5:1277
				Staphylococcus aureus	DNA topoisomerase 4 subunit A	1.18E-110	0.80																	
PubChem CID-11610627	DNA gyrase inhibitor	0.587	0.001	*Mycobacterium tuberculosis*	DNA gyrase subunit A	3.17E-23	0.43	*Escherichia coli K-12*	DNA gyrase subunit A		Unknown	*Escherichia coli K-12*	DNA gyrase	0	Unknown		Unknown				Unknown	18049155052	DNA gyrase	Bioorg. Med. Chem. Lett.,(2006)16:5:1272
	DNA topoisomerase IV inhibitor	0.895	0.000	*Staphylococcus aureus*	DNA topoisomerase 4 subunit A	4.01E-106	0.76		DNA gyrase subunit B			*Staphylococcus aureus*	Topoisomerase IV subunit A	0									DNA gyrase subunit A	Antimicrob. Agents Chemother.,(2010)54:8:3478
																							Topoisomerase IV subunit A	Bioorg. Med. Chem. Lett.,(2006)16:5:1272; Bioorg. Med. Chem. Lett.,(2006)16:5:1277
PubChem CID-11696318	DNA gyrase inhibitor	0.545	0.001	*Mycobacterium tuberculosis*	DNA gyrase subunit A	4.14E-24	0.45	*Escherichia coli K-12*	DNA gyrase subunit A		Unknown	*Escherichia coli K-12*	DNA gyrase	0	Unknown		Unknown				Unknown	18049155149	DNA gyrase	Bioorg. Med. Chem. Lett.,(2006)16:5:1272
	DNA topoisomerase IV inhibitor	0.918	0.000	*Staphylococcus aureus*	DNA topoisomerase 4 subunit A	4.14E-24	0.45		DNA gyrase subunit B			*Staphylococcus aureus*	Topoisomerase IV subunit A	0									DNA gyrase subunit A	Antimicrob. Agents Chemother.,(2010)54:8:3478
																							Topoisomerase IV subunit A	Bioorg. Med. Chem. Lett.,(2006)16:5:1272; Bioorg. Med. Chem. Lett.,(2006)16:5:1277
PubChem CID-11844920	DNA gyrase inhibitor	0.697	0.000	*Mycobacterium tuberculosis*	DNA gyrase subunit A	4.78E-60	0.55	*Escherichia coli K-12*	DNA gyrase subunit A		Unknown	*Escherichia coli K-12*	DNA gyrase	0	Unknown		Unknown				Unknown	18049155216	DNA gyrase	Bioorg. Med. Chem. Lett.,(2006)16:5:1272
	DNA topoisomerase IV inhibitor	0.857	0.000	*Staphylococcus aureus*	DNA topoisomerase 4 subunit A	1.14E-83	0.54		DNA gyrase subunit B			*Staphylococcus aureus subsp. aureus Mu50*	DNA gyrase	0.01 to 0									DNA gyrase subunit A	Antimicrob. Agents Chemother.,(2010)54:8:3478
												Staphylococcus aureus	Topoisomerase IV subunit A	0									Topoisomerase IV subunit A	Bioorg. Med. Chem. Lett.,(2006)16:5:1272; Bioorg. Med. Chem. Lett.,(2006)16:5:1277
PubChem CID-11996799	DNA gyrase inhibitor	0.586	0.001	*Mycobacterium tuberculosis*	DNA gyrase subunit A	3.01E-61	0.55		Unknown		Unknown	*Escherichia coli K-12*	DNA gyrase	0	Unknown		Unknown				Unknown	18049155248	DNA gyrase	Bioorg. Med. Chem. Lett.,(2006)16:5:1272
	DNA topoisomerase IV inhibitor	0.822	0.000	*Staphylococcus aureus*	DNA topoisomerase 4 subunit A	1.03E-75	0.48					*Staphylococcus aureus subsp. aureus Mu50*	DNA gyrase	0.01 to 0									DNA gyrase subunit A	Antimicrob. Agents Chemother.,(2010)54:8:3478
												Staphylococcus aureus	Topoisomerase IV subunit A	0									Topoisomerase IV subunit A	Bioorg. Med. Chem. Lett.,(2006)16:5:1272; Bioorg. Med. Chem. Lett.,(2006)16:5:1277
PubChem CID-11996800	DNA gyrase inhibitor	0.619	0.001	*Mycobacterium tuberculosis*	DNA gyrase subunit A	3.91E-61	0.57	*Escherichia coli K-12*	DNA gyrase subunit A		Unknown	*Escherichia coli K-12*	DNA gyrase	0	Unknown		Unknown				Unknown	18049155315	DNA gyrase	Bioorg. Med. Chem. Lett.,(2006)16:5:1272
	DNA topoisomerase IV inhibitor	0.862	0.000	*Staphylococcus aureus*	DNA topoisomerase 4 subunit A	3.29E-78	0.51		DNA gyrase subunit B			*Staphylococcus aureus*	Topoisomerase IV subunit A	0									DNA gyrase subunit A	Antimicrob. Agents Chemother.,(2010)54:8:3478
																							Topoisomerase IV subunit A	Bioorg. Med. Chem. Lett.,(2006)16:5:1272; Bioorg. Med. Chem. Lett.,(2006)16:5:1277
PubChem CID-11997263	DNA gyrase inhibitor	0.723	0.000	*Mycobacterium tuberculosis*	DNA gyrase subunit A	4.06E-63	0.58	*Escherichia coli K-12*	DNA gyrase subunit A		Unknown	*Escherichia coli K-12*	DNA gyrase	0	Unknown		Unknown			*Staphylococcus aureus (strain MSSA476)*	DNA topoisomerase 4 subunit A	18049155350	DNA gyrase	Bioorg. Med. Chem. Lett.,(2006)16:5:1272
	DNA topoisomerase IV inhibitor	0.899	0.000	*Staphylococcus aureus*	DNA topoisomerase 4 subunit A	7.37E-84	0.55		DNA gyrase subunit B			*Staphylococcus aureus subsp. aureus Mu50*	DNA gyrase	0									DNA gyrase subunit A	Antimicrob. Agents Chemother.,(2010)54:8:3478
												Staphylococcus aureus	Topoisomerase IV subunit A	0									Topoisomerase IV subunit A	Bioorg. Med. Chem. Lett.,(2006)16:5:1272; Bioorg. Med. Chem. Lett.,(2006)16:5:1277
PubChem CID-25022869	DNA gyrase inhibitor	0.899	0.000	*Mycobacterium tuberculosis*	DNA gyrase subunit A	7.54E-82	0.76	*Escherichia coli K-12*	DNA gyrase subunit A		Unknown	*Escherichia coli K-12*	DNA gyrase	0	Unknown		Unknown				Unknown	18049155424	DNA gyrase	Bioorg. Med. Chem. Lett.,(2006)16:5:1272
	DNA topoisomerase IV inhibitor	0.936	0.000	*Staphylococcus aureus*	DNA topoisomerase 4 subunit A	1.68E-77	0.51		DNA gyrase subunit B			*Staphylococcus aureus subsp. aureus Mu50*	DNA gyrase	0.01 to 0									DNA gyrase subunit A	Antimicrob. Agents Chemother.,(2010)54:8:3478
													DNA gyrase subunit B	0									Topoisomerase IV subunit A	Bioorg. Med. Chem. Lett.,(2006)16:5:1272; Bioorg. Med. Chem. Lett.,(2006)16:5:1277
												Staphylococcus aureus	Topoisomerase IV subunit A	0									DNA gyrase	Bioorg. Med. Chem. Lett.,(2006)16:5:1272
PubChem CID-44408626	DNA gyrase inhibitor	0.632	0.001	*Mycobacterium tuberculosis*	DNA gyrase subunit A	4.58E-25	0.47	*Escherichia coli K-12*	DNA gyrase subunit A		Unknown	*Escherichia coli K-12*	DNA gyrase	0	Unknown		Unknown			*Staphylococcus aureus (strain MSSA476)*	DNA topoisomerase 4 subunit A	18049155450	Topoisomerase IV subunit A	Bioorg. Med. Chem. Lett.,(2006)16:5:1272; Bioorg. Med. Chem. Lett.,(2006)16:5:1277
	DNA topoisomerase IV inhibitor	0.927	0.000	*Staphylococcus aureus*	DNA topoisomerase 4 subunit A	2.16E-117	0.81		DNA gyrase subunit B			*Staphylococcus aureus*	Topoisomerase IV subunit A	0										
PubChem CID-44408894	DNA gyrase inhibitor	0.584	0.001	*Mycobacterium tuberculosis*	DNA gyrase subunit A	2.70E-22	0.41	*Escherichia coli K-12*	DNA gyrase subunit A		Unknown	*Escherichia coli K-12*	DNA gyrase	0	Unknown		Unknown				Unknown	18049155518	DNA gyrase	Bioorg. Med. Chem. Lett.,(2006)16:5:1272
	DNA topoisomerase IV inhibitor	0.868	0.000	*Staphylococcus aureus*	DNA topoisomerase 4 subunit A	4.43E-107	0.79		DNA gyrase subunit B			*Staphylococcus aureus*	Topoisomerase IV subunit A	0									DNA gyrase subunit A	Antimicrob. Agents Chemother.,(2010)54:8:3478
																							Topoisomerase IV subunit A	Bioorg. Med. Chem. Lett.,(2006)16:5:1272; Bioorg. Med. Chem. Lett.,(2006)16:5:1277
PubChem CID-44408896	DNA gyrase inhibitor	0.612	0.001	*Mycobacterium tuberculosis*	DNA gyrase subunit A	2.00E-23	0.43	*Escherichia coli K-12*	DNA gyrase subunit A		Unknown	*Escherichia coli K-12*	DNA gyrase	0	Unknown		Unknown				Unknown	18049164135	DNA gyrase	Bioorg. Med. Chem. Lett.,(2006)16:5:1272
	DNA topoisomerase IV inhibitor	0.895	0.000	*Staphylococcus aureus*	DNA topoisomerase 4 subunit A	6.01E-112	0.78		DNA gyrase subunit B			*Staphylococcus aureus*	Topoisomerase IV subunit A	0									DNA gyrase subunit A	Antimicrob. Agents Chemother.,(2010)54:8:3478
																							Topoisomerase IV subunit A	Bioorg. Med. Chem. Lett.,(2006)16:5:1272; Bioorg. Med. Chem. Lett.,(2006)16:5:1277
PubChem CID-44408994	DNA gyrase inhibitor	0.502	0.001	*Mycobacterium tuberculosis*	DNA gyrase subunit A	2.03E-24	0.45	*Escherichia coli K-12*	DNA gyrase subunit A		Unknown	*Escherichia coli K-12*	DNA gyrase	0	Unknown		Unknown			*Staphylococcus aureus (strain MSSA476)*	DNA topoisomerase 4 subunit A	18049164205	DNA gyrase	Bioorg. Med. Chem. Lett.,(2006)16:5:1272
	DNA topoisomerase IV inhibitor	0.951	0.000	*Staphylococcus aureus*	DNA topoisomerase 4 subunit A	1.19E-110	0.75		DNA gyrase subunit B			*Staphylococcus aureus*	Topoisomerase IV subunit A	0									DNA gyrase subunit A	Antimicrob. Agents Chemother.,(2010)54:8:3478
																							Topoisomerase IV subunit A	Bioorg. Med. Chem. Lett.,(2006)16:5:1272; Bioorg. Med. Chem. Lett.,(2006)16:5:1277
PubChem CID-44409001	DNA gyrase inhibitor	0.650	0.001	*Mycobacterium tuberculosis*	DNA gyrase subunit A	2.03E-24	0.45	*Escherichia coli K-12*	DNA gyrase subunit A		Unknown	*Escherichia coli K-12*	DNA gyrase	0	Unknown		Unknown			*Staphylococcus aureus (strain MSSA476)*	DNA topoisomerase 4 subunit A	18049164230	DNA gyrase	Bioorg. Med. Chem. Lett.,(2006)16:5:1272
	DNA topoisomerase IV inhibitor	0.920	0.000	*Staphylococcus aureus*	DNA topoisomerase 4 subunit A	1.19E-110	0.75		DNA gyrase subunit B			*Staphylococcus aureus*	Topoisomerase IV subunit A	0									DNA gyrase subunit A	Antimicrob. Agents Chemother.,(2010)54:8:3478
																							Topoisomerase IV subunit A	Bioorg. Med. Chem. Lett.,(2006)16:5:1272; Bioorg. Med. Chem. Lett.,(2006)16:5:1277
PubChem CID-44409010	DNA gyrase inhibitor	0.599	0.001	*Mycobacterium tuberculosis*	DNA gyrase subunit A	4.58E-25	0.47	*Escherichia coli K-12*	DNA gyrase subunit A		Unknown	*Escherichia coli K-12*	DNA gyrase	0	Unknown		Unknown			*Staphylococcus aureus (strain MSSA476)*	DNA topoisomerase 4 subunit A	18049164253	DNA gyrase	Bioorg. Med. Chem. Lett.,(2006)16:5:1272
	DNA topoisomerase IV inhibitor	0.937	0.000	*Staphylococcus aureus*	DNA topoisomerase 4 subunit A	1.52E-114	0.81		DNA gyrase subunit B			*Staphylococcus aureus*	Topoisomerase IV subunit A	0									DNA gyrase subunit A	Antimicrob. Agents Chemother.,(2010)54:8:3478
																							Topoisomerase IV subunit A	Bioorg. Med. Chem. Lett.,(2006)16:5:1272; Bioorg. Med. Chem. Lett.,(2006)16:5:1277
PubChem CID-53236573	DNA gyrase inhibitor	0.839	0.000	*Mycobacterium tuberculosis*	DNA gyrase subunit A	1.25E-77	0.71		Unknown		Unknown	*Escherichia coli K-12*	DNA gyrase	0	Unknown		Unknown				Unknown	18049164314	DNA gyrase	Bioorg. Med. Chem. Lett.,(2006)16:5:1272
	DNA topoisomerase IV inhibitor	0.869	0.000	*Staphylococcus aureus*	DNA topoisomerase 4 subunit A	2.05E-72	0.47					*Staphylococcus aureus subsp. aureus Mu50*	DNA gyrase	0									DNA gyrase subunit A	Antimicrob. Agents Chemother.,(2010)54:8:3478
												Staphylococcus aureus	Topoisomerase IV subunit A	0									Topoisomerase IV subunit A	Bioorg. Med. Chem. Lett.,(2006)16:5:1272; Bioorg. Med. Chem. Lett.,(2006)16:5:1277
PubChem CID-53236796	DNA gyrase inhibitor	0.625	0.001	*Mycobacterium tuberculosis*	DNA gyrase subunit A	5.92E-73	0.67	*Escherichia coli K-12*	DNA gyrase subunit A		Unknown	*Escherichia coli K-12*	DNA gyrase	0	Unknown		Unknown				Unknown	18049164338	DNA gyrase	Bioorg. Med. Chem. Lett.,(2006)16:5:1272
	DNA topoisomerase IV inhibitor	0.842	0.000	*Staphylococcus aureus*	DNA topoisomerase 4 subunit A	7.43E-69	0.45		DNA gyrase subunit B			*Staphylococcus aureus subsp. aureus Mu50*	DNA gyrase	0									DNA gyrase subunit A	Antimicrob. Agents Chemother.,(2010)54:8:3478
												Staphylococcus aureus	Topoisomerase IV subunit A	0									Topoisomerase IV subunit A	Bioorg. Med. Chem. Lett.,(2006)16:5:1272; Bioorg. Med. Chem. Lett.,(2006)16:5:1277
PubChem CID-122195336	DNA gyrase inhibitor	0.350	0.001	*Mycobacterium tuberculosis*	DNA gyrase subunit A	5.682e-38	0.39	*Escherichia coli*	DNA gyrase subunit A		Unknown	*Staphylococcus aureus subsp. aureus Mu50*	DNA gyrase	0	Unknown		Unknown				Unknown	18048142730	Unknown	
	DNA topoisomerase IV inhibitor	0.361	0.002	*Staphylococcus aureus*	DNA gyrase subunit A	2.514e-07	0.42		DNA gyrase subunit B				DNA gyrase	0										
					DNA topoisomerase 4 subunit A	4.933e-13	0.39	*Escherichia coli K-12*	DNA gyrase subunit A				DNA gyrase subunit B	0.01 to 0										
									DNA gyrase subunit B															
								Mycobacterium tuberculosis	DNA gyrase subunit A															
									DNA gyrase subunit B															
								Staphylococcus aureus subsp. aureus Mu50	DNA gyrase subunit A															
PubChem CID-122195337	DNA gyrase inhibitor	0.217	0.002	*Mycobacterium tuberculosis*	DNA gyrase subunit A	4.108e-37	0.38	*Bacillus subtilis (strain 168)*	DNA gyrase subunit A		Unknown	*Escherichia coli K-12*	DNA gyrase	0	Unknown		Unknown				Unknown	18048142730	DNA gyrase	J. Med. Chem.,(1996)39:16:3070
	DNA topoisomerase IV inhibitor	0.287	0.002	*Staphylococcus aureus*	DNA gyrase subunit A	9.56e-13	0.38		DNA gyrase subunit B			*Staphylococcus aureus subsp. aureus Mu50*	DNA gyrase	0.01 to 0										
					DNA topoisomerase 4 subunit A	4.185e-06	0.41		DNA topoisomerase IV subunit A				DNA gyrase subunit B	0										
									DNA topoisomerase IV subunit B															
								Escherichia coli	DNA gyrase subunit A															
									DNA gyrase subunit B															
								Escherichia coli K-12	DNA gyrase subunit A															
									DNA gyrase subunit B															
								Mycobacterium tuberculosis	DNA gyrase subunit A															
									DNA gyrase subunit B															

Bioactive molecule	PASS online[4]			Similarity Ensemble Approach (SEA)[2]				ChemProt[3]		SuperPred[6]		Polypharmacology Browser (PPB)[1]			SPiDER[7]		HitPick[5]			PharmMapper[9]			TargetHunter[8]	
	Activity	Pa	Pi	Organism	Target	P-Value	Max TC	Organism	Target	Organism	Target	Organism	Target	P-Value	Target	Confidence level	Target	Precision (%)	Tc similarity	Organism	Target	Job Id	Target	Reference
QA 241 free base	DNA gyrase inhibitor<br>DNA topoisomerase IV inhibitor	0.131<br>0.062	0.003<br>0.003	*Escherichia coli*<br><br>*Mycobacterium tuberculosis*<br>*Staphylococcus aureus*	DNA gyrase subunit A<br>DNA gyrase subunit B<br>DNA gyrase subunit A<br>DNA topoisomerase 4 subunit A	9.96E-32<br>6.46E-19<br>1.47E-45<br>6.96E-08	0.42<br>0.42<br>0.46<br>0.42	*Escherichia coli*<br><br>*Escherichia coli K-12*<br><br>*Pseudomonas aeruginosa (strain ATCC 15692 / PAO1 / 1C / PRS 101 / LMG12228)*<br><br>*Mycobacterium tuberculosis*<br>*Staphylococcus aureus*<br>*Staphylococcus aureus subsp. aureus Mu50*<br><br>*Staphylococcus aureus subsp. aureus N315*	DNA gyrase subunit A<br>DNA gyrase subunit B<br>DNA gyrase subunit A<br>DNA gyrase subunit B<br>DNA gyrase subunit A<br>DNA gyrase subunit B<br>DNA gyrase subunit B<br>DNA topoisomerase IV subunit A<br>DNA gyrase subunit A<br>DNA gyrase subunit B<br>DNA gyrase subunit B		Unknown	*Escherichia coli K-12*<br>*Staphylococcus aureus subsp. aureus Mu50*	DNA gyrase<br>DNA gyrase subunit B	0<br>0	Unknown		Unknown				Unknown	18048152919; 180428111527	Unknown	
RO 13-5478	DNA topoisomerase IV inhibitor	0.025	0.010		Unknown				Unknown		Unknown	*Escherichia coli K-12*<br>*Staphylococcus aureus*	DNA gyrase<br>DNA gyrase subunit B	0<br>0.01 to 0	Unknown		Unknown				Unknown	18049164403	DNA gyrase	J. Med. Chem.,(1986)29:3:394; J. Med. Chem.,(2005)48:4:1229
RO 14-9578	DNA gyrase inhibitor<br>DNA topoisomerase IV inhibitor	0.029<br>0.028	0.023<br>0.007		Unknown			*Escherichia coli K-12*	DNA gyrase subunit A<br>DNA gyrase subunit B		Unknown	*Escherichia coli K-12*	DNA gyrase	0	Unknown		Unknown				Unknown	18049164426	DNA gyrase	J. Med. Chem.,(2005)48:4:1229
Rosoxacin	DNA gyrase inhibitor<br>DNA topoisomerase IV inhibitor	0.094<br>0.051	0.003<br>0.003		Unknown			*Bacillus subtilis*<br><br><br>*Escherichia coli*<br><br>*Mycobacterium tuberculosis*	DNA gyrase subunit A<br>DNA gyrase subunit B<br>DNA topoisomerase IV subunit A<br>DNA gyrase subunit A<br>DNA gyrase subunit B<br>DNA gyrase subunit B	*Escherichia coli*	DNA gyrase subunit B<br>DNA topoisomerase IV subunit A	*Escherichia coli K-12*<br>*Staphylococcus aureus*	DNA gyrase<br>Topoisomerase IV subunit A	0<br>0	Unknown		Unknown				Unknown	180327134458	DNA gyrase<br>DNA gyrase subunit B	Bioorg. Med. Chem. Lett.,(1992)2:7:643; Bioorg. Med. Chem. Lett.,(1993)3:2:225; Bioorg. Med. Chem. Lett.,(1998)8:1:97; J. Med. Chem.,(1986)29:3:394; J. Med. Chem.,(1986)29:4:445<br>J. Med. Chem.,(1992)35:25:4745
Rufloxacin	DNA gyrase inhibitor<br>DNA topoisomerase IV inhibitor	0.097<br>0.036	0.003<br>0.004	*Escherichia coli*<br><br>*Mycobacterium tuberculosis*<br>*Staphylococcus aureus*	DNA gyrase subunit A<br>DNA gyrase subunit B<br>DNA gyrase subunit A<br>DNA topoisomerase 4 subunit A	1.97E-17<br>1.75E-10<br>3.09E-42<br>4.03E-07	0.36<br>0.36<br>0.41<br>0.38	*Escherichia coli*<br><br>*Escherichia coli K-12*<br><br>*Mycobacterium smegmatis*<br>*Mycobacterium tuberculosis*<br><br>*Staphylococcus aureus subsp. aureus Mu50*<br><br>*Staphylococcus aureus subsp. aureus N315*	DNA gyrase subunit A<br>DNA gyrase subunit B<br>DNA gyrase subunit A<br>DNA gyrase subunit B<br>DNA topoisomerase (ATP-hydrolysing) (DNA gyrase)<br>DNA gyrase subunit A<br>DNA gyrase subunit B<br>DNA gyrase subunit A<br>DNA gyrase subunit B<br>DNA gyrase subunit B		Unknown	*Escherichia coli K-12*<br>*Staphylococcus aureus subsp. aureus Mu50*	DNA gyrase<br>DNA gyrase subunit B	0<br>0	Unknown		Unknown				Unknown	18048101754; 180428111617	DNA gyrase<br>DNA gyrase subunit B<br>Topoisomerase IV<br>Topoisomerase IV subunit A	Bioorg. Med. Chem. Lett.,(1992)2:7:643; J. Med. Chem.,(1986)29:4:445; J. Med. Chem.,(1986)29:3:394; J. Med. Chem.,(1988)31:5:991; J. Med. Chem.,(1991)34:3:1155<br>J. Med. Chem.,(1992)35:25:4745<br>J. Med. Chem.,(2013)56:18:7396<br>Bioorg. Med. Chem. Lett.,(2006)16:5:1272; Bioorg. Med. Chem. Lett.,(2006)16:5:1277; J. Med. Chem.,(2005)48:16:5232; J. Med. Chem.,(2006)49:1:39
S 25932	DNA gyrase inhibitor<br>DNA topoisomerase IV inhibitor	0.136<br>0.054	0.003<br>0.003	*Escherichia coli*<br><br>*Mycobacterium tuberculosis*<br>*Staphylococcus aureus*	DNA gyrase subunit A<br>DNA gyrase subunit B<br>DNA gyrase subunit A<br>DNA topoisomerase 4 subunit A	3.17E-21<br>1.01E-12<br>4.42E-46<br>1.83E-07	0.36<br>0.36<br>0.45<br>0.42		Unknown		Unknown	*Escherichia coli K-12*<br>*Staphylococcus aureus subsp. aureus Mu50*<br><br>*Staphylococcus aureus*	DNA gyrase<br>DNA gyrase<br>DNA gyrase subunit B<br>Topoisomerase IV subunit A	0<br>0<br>0<br>0	Unknown		Unknown				Unknown	18048152951; 180428111640	DNA gyrase<br>Topoisomerase IV subunit A	J. Med. Chem.,(1986)29:3:394; J. Med. Chem.,(1996)39:16:3070; J. Med. Chem.,(2005)48:16:5232<br>J. Med. Chem.,(2005)48:16:5232
S 31076	DNA gyrase inhibitor<br>DNA topoisomerase IV inhibitor	0.552<br>0.323	0.001<br>0.002	*Escherichia coli*<br><br>*Mycobacterium tuberculosis*<br>*Staphylococcus aureus* 	DNA gyrase subunit A<br>DNA gyrase subunit B<br>DNA gyrase subunit A<br>DNA topoisomerase 4 subunit A<br>DNA topoisomerase 4 subunit B	3.01E-34<br>2.11E-20<br>5.06E-88<br>5.76E-10<br>6.66E-06	0.44<br>0.44<br>0.68<br>0.62<br>0.44		Unknown		Unknown	*Escherichia coli K-12*<br>*Staphylococcus aureus subsp. aureus Mu50*	DNA gyrase<br>DNA gyrase subunit B	0<br>0	Unknown		Unknown				Unknown	18048161357; 180428111705	Unknown	
Sarafloxacin	DNA gyrase inhibitor<br>DNA topoisomerase IV inhibitor	0.323<br>0.140	0.002<br>0.003	*Escherichia coli*<br><br>*Mycobacterium tuberculosis*<br>*Staphylococcus aureus* 	DNA gyrase subunit A<br>DNA gyrase subunit B<br>DNA gyrase subunit A<br>DNA topoisomerase 4 subunit A<br>DNA topoisomerase 4 subunit B	3.65E-54<br>3.79E-32<br>1.28E-35<br>4.11E-09<br>1.99E-08	0.65<br>0.65<br>0.36<br>0.65<br>0.65	*Bacillus subtilis (strain 168)*<br><br><br><br>*Escherichia coli*<br><br>*Escherichia coli K-12*<br><br><br>*Pseudomonas aeruginosa (strain ATCC 15692 / PAO1 / 1C / PRS 101 / LMG12228)*<br><br>*Mycobacterium tuberculosis*<br>*Staphylococcus aureus subsp. aureus Mu50*<br><br><br>*Staphylococcus aureus subsp. aureus N315*	DNA gyrase subunit A<br>DNA gyrase subunit B<br>DNA topoisomerase IV subunit A<br>DNA topoisomerase IV subunit B<br>DNA gyrase subunit A<br>DNA gyrase subunit B<br>DNA gyrase subunit A<br>DNA gyrase subunit B<br>DNA topoisomerase IV subunit A<br>DNA gyrase subunit A<br>DNA gyrase subunit B<br>DNA gyrase subunit B<br>DNA gyrase subunit A<br>DNA gyrase subunit B<br>DNA topoisomerase IV subunit A<br>DNA gyrase subunit B		Unknown	*Escherichia coli K-12*<br>*Staphylococcus aureus subsp. aureus Mu50*	DNA gyrase<br>DNA gyrase subunit B	0<br>0	Unknown		Unknown				Unknown	180327134539	Unknown	
Sitafloxacin	DNA gyrase inhibitor<br>DNA topoisomerase IV inhibitor	0.644<br>0.222	0.001<br>0.002	*Escherichia coli*<br>*Mycobacterium tuberculosis*<br>*Staphylococcus aureus*	DNA gyrase subunit A<br>DNA gyrase subunit A<br>DNA topoisomerase 4 subunit A	2.81E-09<br>3.20E-40<br>7.84E-07	0.34<br>0.39<br>0.36	*Bacillus subtilis (strain 168)*<br><br><br><br>*Escherichia coli K-12*<br><br><br>*Pseudomonas aeruginosa (strain ATCC 15692 / PAO1 / 1C / PRS 101 / LMG12228)*<br><br>*Staphylococcus aureus subsp. aureus Mu50*<br><br><br>*Staphylococcus aureus subsp. aureus N315*	DNA gyrase subunit A<br>DNA gyrase subunit B<br>DNA topoisomerase IV subunit A<br>DNA topoisomerase IV subunit B<br>DNA gyrase subunit A<br>DNA gyrase subunit B<br>DNA topoisomerase IV subunit A<br>DNA gyrase subunit A<br>DNA gyrase subunit B<br>DNA gyrase subunit A<br>DNA gyrase subunit B<br>DNA topoisomerase IV subunit A<br>DNA gyrase subunit B		Unknown	*Escherichia coli K-12*<br>*Staphylococcus aureus subsp. aureus Mu50* 	DNA gyrase<br>DNA gyrase<br>DNA gyrase subunit B	0<br>0<br>0	Unknown		Unknown				Unknown	18048101827; 180428114736	Unknown	
Sparfloxacin	DNA gyrase inhibitor<br>DNA topoisomerase IV inhibitor	0.704<br>0.313	0.000<br>0.002	*Escherichia coli*<br><br>*Mycobacterium tuberculosis*<br>*Staphylococcus aureus*	DNA gyrase subunit A<br>DNA gyrase subunit B<br>DNA gyrase subunit A<br>DNA topoisomerase 4 subunit A	9.30E-22<br>4.90E-13<br>2.82E-48<br>8.89E-08	0.39<br>0.39<br>0.49<br>0.43	*Bacillus subtilis (strain 168)*<br><br><br><br>*Escherichia coli K-12*<br><br><br>*Pseudomonas aeruginosa (strain ATCC 15692 / PAO1 / 1C / PRS 101 / LMG12228)*<br><br>*Mycobacterium tuberculosis*<br>*Staphylococcus aureus subsp. aureus Mu50*<br><br><br>*Staphylococcus aureus subsp. aureus N315*	DNA gyrase subunit A<br>DNA gyrase subunit B<br>DNA topoisomerase IV subunit A<br>DNA topoisomerase IV subunit B<br>DNA gyrase subunit A<br>DNA gyrase subunit B<br>DNA topoisomerase IV subunit A<br>DNA gyrase subunit A<br>DNA gyrase subunit B<br>DNA gyrase subunit B<br>DNA gyrase subunit A<br>DNA gyrase subunit B<br>DNA topoisomerase IV subunit A<br>DNA gyrase subunit B		Unknown	*Escherichia coli K-12*<br>*Staphylococcus aureus subsp. aureus Mu50*	DNA gyrase<br>DNA gyrase subunit B	0<br>0	Unknown		Unknown				Unknown	180327134629	Unknown	
T 14097	DNA topoisomerase IV inhibitor	0.026	0.009		Unknown				Unknown		Unknown	*Escherichia coli K-12*	DNA gyrase	0	Unknown		Unknown			*Staphylococcus aureus (strain MSSA476)*	DNA topoisomerase 4 subunit A	18048161425; 180428114804	Unknown	
Temafloxacin	DNA gyrase inhibitor<br>DNA topoisomerase IV inhibitor	0.400<br>0.131	0.001<br>0.003	*Escherichia coli*<br><br>*Mycobacterium tuberculosis*<br>*Staphylococcus aureus* 	DNA gyrase subunit A<br>DNA gyrase subunit B<br>DNA gyrase subunit A<br>DNA topoisomerase 4 subunit A<br>DNA topoisomerase 4 subunit B	3.37E-48<br>1.25E-28<br>3.82E-44<br>2.96E-08<br>8.77E-07	0.52<br>0.52<br>0.48<br>0.52<br>0.52	*Bacillus subtilis (strain 168)*<br><br><br><br>*Escherichia coli*<br><br>*Escherichia coli K-12*<br><br><br>*Pseudomonas aeruginosa (strain ATCC 15692 / PAO1 / 1C / PRS 101 / LMG12228)*<br><br>*Mycobacterium tuberculosis*<br>*Staphylococcus aureus*<br>*Staphylococcus aureus subsp. aureus Mu50*<br><br><br>*Staphylococcus aureus subsp. aureus N315*	DNA gyrase subunit A<br>DNA gyrase subunit B<br>DNA topoisomerase IV subunit A<br>DNA topoisomerase IV subunit B<br>DNA gyrase subunit A<br>DNA gyrase subunit B<br>DNA gyrase subunit A<br>DNA gyrase subunit B<br>DNA topoisomerase IV subunit A<br>DNA gyrase subunit A<br>DNA gyrase subunit B<br>DNA gyrase subunit B<br>DNA topoisomerase IV subunit A<br>DNA gyrase subunit A<br>DNA gyrase subunit B<br>DNA topoisomerase IV subunit A<br>DNA gyrase subunit B		Unknown	*Escherichia coli K-12*<br>*Staphylococcus aureus subsp. aureus Mu50*	DNA gyrase<br>DNA gyrase subunit B	0<br>0	Unknown		Unknown				Unknown	18048100614; 180428114826	Unknown	
Tioxacin	DNA topoisomerase IV inhibitor	0.022	0.014		Unknown				Unknown		Unknown	*Escherichia coli K-12*	DNA gyrase	0	Unknown		Unknown				Unknown	18048111548; 180428114852	Unknown	
Tosufloxacin	DNA gyrase inhibitor<br>DNA topoisomerase IV inhibitor	0.438<br>0.303	0.001<br>0.002	*Escherichia coli*<br><br>*Mycobacterium tuberculosis*<br>*Staphylococcus aureus*	DNA gyrase subunit A<br>DNA gyrase subunit B<br>DNA gyrase subunit A<br>DNA topoisomerase 4 subunit A	3.77E-40<br>6.97E-24<br>7.52E-37<br>6.84E-07	0.38<br>0.38<br>0.36<br>0.38	*Bacillus subtilis (strain 168)*<br><br><br><br>*Escherichia coli*<br><br>*Escherichia coli K-12*<br><br><br>*Pseudomonas aeruginosa (strain ATCC 15692 / PAO1 / 1C / PRS 101 / LMG12228)*<br><br>*Mycobacterium tuberculosis*<br><br>*Staphylococcus aureus subsp. aureus Mu50*<br><br><br>*Staphylococcus aureus subsp. aureus N315*	DNA gyrase subunit A<br>DNA gyrase subunit B<br>DNA topoisomerase IV subunit A<br>DNA topoisomerase IV subunit B<br>DNA gyrase subunit A<br>DNA gyrase subunit B<br>DNA gyrase subunit A<br>DNA gyrase subunit B<br>DNA topoisomerase IV subunit A<br>DNA gyrase subunit A<br>DNA gyrase subunit B<br>DNA gyrase subunit A<br>DNA gyrase subunit B<br>DNA gyrase subunit A<br>DNA gyrase subunit B<br>DNA topoisomerase IV subunit A<br>DNA gyrase subunit B		Unknown	*Staphylococcus aureus subsp. aureus Mu50*	DNA gyrase	0	Unknown		Unknown				Unknown	18048124216; 180428114915	Unknown	

Bioactive molecule	PASS online[4]			Similarity Ensemble Approach (SEA)[2]				ChemProt[3]		SuperPred[6]		Polypharmacology Browser (PPB)[1]			SPiDER[7]		HitPick[5]			PharmMapper[9]			TargetHunter[8]	
	Activity	Pa	Pi	Organism	Target	P-Value	Max TC	Organism	Target	Organism	Target	Organism	Target	P-Value	Target	Confidence level	Target	Precision (%)	Tc similarity	Organism	Target	Job Id	Target	Reference
Trovafloxacin	DNA gyrase inhibitor<br>DNA topoisomerase IV inhibitor	0.389<br>0.319	0.001<br>0.002	*Escherichia coli*<br>*Mycobacterium tuberculosis*<br>*Staphylococcus aureus*	DNA gyrase subunit A<br>DNA gyrase subunit B<br>DNA gyrase subunit A<br>DNA topoisomerase 4 subunit A	3.41E-41<br>1.69E-24<br>1.45E-35<br>1.16E-06	0.37<br>0.37<br>0.35<br>0.37	*Escherichia coli K-12*<br>*Pseudomonas aeruginosa (strain ATCC 15692 / PAO1 / 1C / PRS 101 / LMG12228)*<br>*Staphylococcus aureus subsp. aureus Mu50*<br>*Staphylococcus aureus subsp. aureus N315*	DNA gyrase subunit A<br>DNA gyrase subunit B<br>DNA gyrase subunit A<br>DNA gyrase subunit B<br>DNA gyrase subunit A<br>DNA gyrase subunit B<br>DNA topoisomerase IV subunit A<br>DNA gyrase subunit B		Unknown		Unknown		Unknown		Unknown				Unknown	18048111623; 180428114959	DNA gyrase<br>DNA gyrase subunit B<br>Topoisomerase IV subunit A	J. Med. Chem.,(1993)36:7:871; J. Med. Chem.,(1991)34:2:656; J. Med. Chem.,(1993)36:14:1964; J. Med. Chem.,(1996)39:16:3070; J. Med. Chem.,(2003)46:17:3655<br>J. Med. Chem.,(1992)35:25:4745<br>Bioorg. Med. Chem. Lett.,(2004)14:20:5193; Bioorg. Med. Chem. Lett.,(2006)16:5:1272
Ulifloxacin	DNA gyrase inhibitor<br>DNA topoisomerase IV inhibitor	0.095<br>0.041	0.003<br>0.004	*Escherichia coli*<br>*Mycobacterium tuberculosis*<br>*Staphylococcus aureus*	DNA gyrase subunit A<br>DNA gyrase subunit B<br>DNA gyrase subunit A<br>DNA topoisomerase 4 subunit A<br>DNA topoisomerase 4 subunit B	2.07E-23<br>5.20E-14<br>4.72E-17<br>1.01E-07<br>6.87E-07	0.52<br>0.52<br>0.31<br>0.52<br>0.52	*Bacillus subtilis (strain 168)*<br>*Escherichia coli K-12*<br>*Pseudomonas aeruginosa (strain ATCC 15692 / PAO1 / 1C / PRS 101 / LMG12228)*<br>*Staphylococcus aureus subsp. aureus Mu50*<br>*Staphylococcus aureus subsp. aureus N315*	DNA gyrase subunit A<br>DNA gyrase subunit B<br>DNA topoisomerase IV subunit A<br>DNA topoisomerase IV subunit B<br>DNA gyrase subunit A<br>DNA gyrase subunit B<br>DNA topoisomerase IV subunit A<br>DNA gyrase subunit A<br>DNA gyrase subunit B<br>DNA gyrase subunit A<br>DNA gyrase subunit B<br>DNA gyrase subunit B		Unknown	*Staphylococcus aureus subsp. aureus Mu50*	DNA gyrase	0	Unknown		Unknown				Unknown	18048133000; 180428115022	Unknown	
Vebufloxacin (benofloxacin)	DNA gyrase inhibitor<br>DNA topoisomerase IV inhibitor	0.148<br>0.062	0.003<br>0.003	*Escherichia coli*<br>*Mycobacterium tuberculosis*<br>*Staphylococcus aureus*	DNA gyrase subunit A<br>DNA gyrase subunit B<br>DNA gyrase subunit A<br>DNA topoisomerase 4 subunit A	2.50E-40<br>5.48E-24<br>1.28E-47<br>7.36E-08	0.40<br>0.40<br>0.48<br>0.42	*Escherichia coli*<br>*Escherichia coli K-12*<br>*Pseudomonas aeruginosa (strain ATCC 15692 / PAO1 / 1C / PRS 101 / LMG12228)*<br>*Mycobacterium tuberculosis*<br>*Staphylococcus aureus*<br>*Staphylococcus aureus subsp. aureus Mu50*<br>*Staphylococcus aureus subsp. aureus N315*	DNA gyrase subunit A<br>DNA gyrase subunit B<br>DNA gyrase subunit A<br>DNA gyrase subunit B<br>DNA gyrase subunit A<br>DNA gyrase subunit B<br>DNA gyrase subunit B<br>DNA topoisomerase IV subunit A<br>DNA gyrase subunit A<br>DNA gyrase subunit B<br>DNA gyrase subunit B		Unknown	*Escherichia coli K-12*<br>*Staphylococcus aureus subsp. aureus Mu50*	DNA gyrase<br>DNA gyrase subunit B	0<br>0	Unknown		Unknown				Unknown	18048131547; 180428115050	Unknown	
VG 6/1	DNA gyrase inhibitor<br>DNA topoisomerase IV inhibitor	0.345<br>0.068	0.001<br>0.003	*Escherichia coli*<br>*Mycobacterium tuberculosis*<br>*Staphylococcus aureus*	DNA gyrase subunit A<br>DNA gyrase subunit B<br>DNA gyrase subunit A<br>DNA topoisomerase 4 subunit A	3.80E-29<br>2.15E-17<br>9.58E-40<br>9.35E-07	0.34<br>0.34<br>0.39<br>0.36		Unknown		Unknown	*Escherichia coli K-12*<br>*Staphylococcus aureus subsp. aureus Mu50*	DNA gyrase<br>DNA gyrase subunit B	0<br>0	Unknown		Unknown				Unknown	18048200352; 180428135506	DNA gyrase<br>DNA gyrase subunit B	J. Med. Chem.,(1996)39:25:4952<br>J. Med. Chem.,(1992)35:25:4745
WCK 1152 free base	DNA gyrase inhibitor<br>DNA topoisomerase IV inhibitor	0.619<br>0.325	0.001<br>0.002	*Escherichia coli*<br>*Mycobacterium tuberculosis*<br>*Staphylococcus aureus*	DNA gyrase subunit A<br>DNA gyrase subunit B<br>DNA gyrase subunit A<br>DNA topoisomerase 4 subunit A<br>DNA topoisomerase 4 subunit B	1.48E-33<br>5.40E-20<br>3.30E-88<br>1.06E-09<br>9.38E-06	0.43<br>0.43<br>0.66<br>0.61<br>0.43	*Escherichia coli K-12*<br>*Mycobacterium tuberculosis*<br>*Staphylococcus aureus subsp. aureus Mu50*	DNA gyrase subunit A<br>DNA gyrase subunit B<br>DNA gyrase subunit B<br>DNA gyrase subunit B		Unknown	*Escherichia coli K-12*<br>*Staphylococcus aureus subsp. aureus Mu50*	DNA gyrase<br>DNA gyrase<br>DNA gyrase subunit B	0<br>0.01 to 0<br>0	Unknown		Unknown			*Staphylococcus aureus (strain MSSA476)*	DNA topoisomerase 4 subunit A	18048153037; 180428135531	Unknown	
WIN 57273	DNA gyrase inhibitor<br>DNA topoisomerase IV inhibitor	0.484<br>0.247	0.001<br>0.002	*Escherichia coli*<br>*Mycobacterium tuberculosis*<br>*Staphylococcus aureus*	DNA gyrase subunit A<br>DNA gyrase subunit B<br>DNA gyrase subunit A<br>DNA topoisomerase 4 subunit A<br>DNA topoisomerase 4 subunit B	2.58E-42<br>3.69E-25<br>7.86E-49<br>3.28E-65<br>2.67E-07	0.56<br>0.56<br>0.47<br>0.56<br>0.56		Unknown		Unknown	*Escherichia coli K-12*<br>*Staphylococcus aureus subsp. aureus Mu50*<br>*Staphylococcus aureus*	DNA gyrase<br>DNA gyrase<br>DNA gyrase subunit B<br>Topoisomerase IV subunit A	0<br>0.01 to 0<br>0<br>0	Unknown		Unknown			*Staphylococcus aureus (strain MSSA476)*	DNA topoisomerase 4 subunit A	18048142940	DNA gyrase<br>DNA gyrase subunit B<br>Topoisomerase IV subunit A	J. Med. Chem.,(1986)29:3:394; J. Med. Chem.,(1991)34:3:1155; J. Med. Chem.,(1993)36:14:1964; J. Med. Chem.,(1996)39:25:4952; J. Med. Chem.,(1993)36:7:871<br>J. Med. Chem.,(1992)35:25:4745<br>Bioorg. Med. Chem. Lett.,(2004)14:20:5193; Bioorg. Med. Chem. Lett.,(2006)16:5:1272; J. Med. Chem.,(2005)48:16:5232; J. Med. Chem.,(2006)49:1:39
WIN 57294	DNA gyrase inhibitor<br>DNA topoisomerase IV inhibitor	0.378<br>0.194	0.001<br>0.002	*Escherichia coli*<br>*Mycobacterium tuberculosis*<br>*Staphylococcus aureus*	DNA gyrase subunit A<br>DNA gyrase subunit B<br>DNA gyrase subunit A<br>DNA topoisomerase 4 subunit A<br>DNA topoisomerase 4 subunit B	9.53E-36<br>2.75E-21<br>5.67E-50<br>7.75E-14<br>3.95E-06	0.46<br>0.46<br>0.49<br>0.46<br>0.46		Unknown		Unknown	*Escherichia coli K-12*<br>*Staphylococcus aureus subsp. aureus Mu50*<br>*Staphylococcus aureus*	DNA gyrase<br>DNA gyrase<br>DNA gyrase subunit B<br>Topoisomerase IV subunit A	0<br>0<br>0<br>0	Unknown		Unknown			*Staphylococcus aureus (strain MSSA476)*	DNA topoisomerase 4 subunit A	18048153104; 180428135554	DNA gyrase subunit B<br>Topoisomerase IV subunit A	J. Med. Chem.,(1992)35:25:4745<br>Bioorg. Med. Chem. Lett.,(2004)14:20:5193; J. Med. Chem.,(2005)48:16:5232; J. Med. Chem.,(2006)49:1:39
WIN 58161	DNA gyrase inhibitor<br>DNA topoisomerase IV inhibitor	0.287<br>0.048	0.002<br>0.004	*Escherichia coli*<br>*Mycobacterium tuberculosis*<br>*Staphylococcus aureus*	DNA gyrase subunit A<br>DNA gyrase subunit B<br>DNA gyrase subunit A<br>DNA topoisomerase 4 subunit A	2.30E-30<br>4.12E-18<br>6.62E-43<br>2.90E-07	0.39<br>0.39<br>0.44<br>0.39		Unknown		Unknown	*Escherichia coli K-12*<br>*Staphylococcus aureus subsp. aureus Mu50*<br>*Staphylococcus aureus*	DNA gyrase<br>DNA gyrase<br>DNA gyrase subunit B<br>Topoisomerase IV subunit A	0<br>0.01 to 0<br>0.01 to 0<br>0	Unknown		Unknown			*Staphylococcus aureus (strain MSSA476)*	DNA topoisomerase 4 subunit A	18048133055	DNA gyrase	J. Med. Chem.,(1986)29:3:394
WQ 2743	DNA gyrase inhibitor<br>DNA topoisomerase IV inhibitor	0.114<br>0.181	0.003<br>0.002	*Mycobacterium tuberculosis*<br>*Staphylococcus aureus*	DNA gyrase subunit A<br>DNA topoisomerase 4 subunit A	5.22E-46<br>2.45E-07	0.44<br>0.43		Unknown		Unknown	*Escherichia coli K-12*<br>*Staphylococcus aureus subsp. aureus Mu50*	DNA gyrase<br>DNA gyrase<br>DNA gyrase subunit B	0<br>0.01 to 0<br>0	Unknown		Unknown				Unknown	18048200432; 180428135620	DNA gyrase<br>DNA gyrase subunit B<br>Topoisomerase IV subunit A	J. Med. Chem.,(1986)29:3:394; J. Med. Chem.,(1988)31:3:503; J. Med. Chem.,(1988)31:5:991; J. Med. Chem.,(1991)34:3:1155; J. Med. Chem.,(1991)34:3:1142<br>J. Med. Chem.,(1992)35:25:4745<br>Bioorg. Med. Chem. Lett.,(2004)14:20:5193; Bioorg. Med. Chem. Lett.,(2006)16:5:1272; J. Med. Chem.,(2005)48:16:5232; J. Med. Chem.,(2006)49:1:39
WQ 2756	DNA gyrase inhibitor<br>DNA topoisomerase IV inhibitor	0.409<br>0.309	0.001<br>0.002	*Escherichia coli*<br>*Mycobacterium tuberculosis*<br>*Staphylococcus aureus*	DNA gyrase subunit A<br>DNA gyrase subunit B<br>DNA gyrase subunit A<br>DNA topoisomerase 4 subunit A	2.57E-29<br>1.71E-17<br>1.85E-39<br>5.22E-07	0.36<br>0.36<br>0.38<br>0.37	*Escherichia coli K-12*<br>*Pseudomonas aeruginosa (strain ATCC 15692 / PAO1 / 1C / PRS 101 / LMG12228)*<br>*Staphylococcus aureus subsp. aureus Mu50*<br>*Staphylococcus aureus subsp. aureus N315*	DNA gyrase subunit A<br>DNA gyrase subunit B<br>DNA gyrase subunit A<br>DNA gyrase subunit B<br>DNA gyrase subunit A<br>DNA gyrase subunit B<br>DNA gyrase subunit B		Unknown	*Escherichia coli K-12*<br>*Staphylococcus aureus subsp. aureus Mu50*	DNA gyrase<br>DNA gyrase subunit B	0<br>0	Unknown		Unknown				Unknown	18048202026; 180428135642	Unknown	
WQ 2908	DNA gyrase inhibitor<br>DNA topoisomerase IV inhibitor	0.432<br>0.210	0.001<br>0.002	*Escherichia coli*<br>*Mycobacterium tuberculosis*<br>*Staphylococcus aureus*	DNA gyrase subunit A<br>DNA gyrase subunit B<br>DNA gyrase subunit A<br>DNA topoisomerase 4 subunit A	6.30E-26<br>1.67E-15<br>2.80E-41<br>3.72E-07	0.36<br>0.36<br>0.39<br>0.38	*Bacillus subtilis (strain 168)*<br>*Escherichia coli K-12*<br>*Pseudomonas aeruginosa (strain ATCC 15692 / PAO1 / 1C / PRS 101 / LMG12228)*<br>*Staphylococcus aureus subsp. aureus Mu50*<br>*Staphylococcus aureus subsp. aureus N315*	DNA gyrase subunit A<br>DNA gyrase subunit B<br>DNA topoisomerase IV subunit A<br>DNA topoisomerase IV subunit B<br>DNA gyrase subunit A<br>DNA gyrase subunit B<br>DNA gyrase subunit A<br>DNA gyrase subunit B<br>DNA gyrase subunit A<br>DNA gyrase subunit B<br>DNA topoisomerase IV subunit A<br>DNA gyrase subunit B		Unknown	*Escherichia coli K-12*<br>*Staphylococcus aureus subsp. aureus Mu50*	DNA gyrase<br>DNA gyrase subunit B	0<br>0	Unknown		Unknown				Unknown	18048202101; 180428135707	Unknown	
WQ 2942	DNA gyrase inhibitor<br>DNA topoisomerase IV inhibitor	0.256<br>0.075	0.002<br>0.003	*Escherichia coli*<br>*Mycobacterium tuberculosis*<br>*Staphylococcus aureus*	DNA gyrase subunit A<br>DNA gyrase subunit A<br>DNA topoisomerase 4 subunit A	3.48E-09<br>9.63E-34<br>2.36E-06	0.35<br>0.33<br>0.35		Unknown		Unknown	*Escherichia coli K-12*<br>*Staphylococcus aureus subsp. aureus Mu50*	DNA gyrase<br>DNA gyrase subunit B	0<br>0	Unknown		Unknown				Unknown	18048200503; 180428135726	Unknown	
WQ 3330	DNA gyrase inhibitor<br>DNA topoisomerase IV inhibitor	0.427<br>0.200	0.001<br>0.002	*Escherichia coli*<br>*Mycobacterium tuberculosis*<br>*Staphylococcus aureus*	DNA gyrase subunit A<br>DNA gyrase subunit B<br>DNA gyrase subunit A<br>DNA topoisomerase 4 subunit A	1.24E-20<br>2.25E-12<br>2.10E-38<br>1.11E-06	0.33<br>0.33<br>0.37<br>0.36	*Bacillus subtilis (strain 168)*<br>*Escherichia coli*<br>*Escherichia coli K-12*<br>*Mycobacterium tuberculosis*<br>*Staphylococcus aureus subsp. aureus Mu50*	DNA gyrase subunit A<br>DNA gyrase subunit B<br>DNA topoisomerase IV subunit A<br>DNA topoisomerase IV subunit B<br>DNA gyrase subunit A<br>DNA gyrase subunit B<br>DNA gyrase subunit A<br>DNA gyrase subunit B<br>DNA gyrase subunit A<br>DNA gyrase subunit B<br>DNA gyrase subunit B		Unknown	*Escherichia coli K-12*<br>*Staphylococcus aureus subsp. aureus Mu50*	DNA gyrase<br>DNA gyrase subunit B	0<br>0	Unknown		Unknown				Unknown	18048202130; 180428135753	Unknown	
Y 688	DNA gyrase inhibitor<br>DNA topoisomerase IV inhibitor	0.434<br>0.333	0.001<br>0.002	*Escherichia coli*<br>*Mycobacterium tuberculosis*<br>*Staphylococcus aureus*	DNA gyrase subunit A<br>DNA gyrase subunit B<br>DNA gyrase subunit A<br>DNA topoisomerase 4 subunit A<br>DNA topoisomerase 4 subunit B	2.20E-34<br>1.75E-20<br>6.30E-83<br>1.67E-09<br>8.89E-06	0.43<br>0.43<br>0.63<br>0.58<br>0.43	*Escherichia coli*<br>*Escherichia coli K-12*<br>*Pseudomonas aeruginosa (strain ATCC 15692 / PAO1 / 1C / PRS 101 / LMG12228)*<br>*Mycobacterium tuberculosis*<br>*Staphylococcus aureus subsp. aureus Mu50*<br>*Staphylococcus aureus subsp. aureus N315*	DNA gyrase subunit A<br>DNA gyrase subunit B<br>DNA gyrase subunit A<br>DNA gyrase subunit B<br>DNA gyrase subunit A<br>DNA gyrase subunit B<br>DNA gyrase subunit B<br>DNA gyrase subunit A<br>DNA gyrase subunit B<br>DNA topoisomerase IV subunit A<br>DNA gyrase subunit B		Unknown	*Escherichia coli K-12*<br>*Staphylococcus aureus subsp. aureus Mu50*	DNA gyrase<br>DNA gyrase subunit B	0<br>0	Unknown		Unknown			*Staphylococcus aureus (strain MSSA476)*	DNA topoisomerase 4 subunit A	18048202200; 180428135815	Unknown	
Zabofloxacin	DNA gyrase inhibitor<br>DNA topoisomerase IV inhibitor	0.419<br>0.157	0.001<br>0.002	*Escherichia coli*<br>*Mycobacterium tuberculosis*<br>*Staphylococcus aureus*	DNA gyrase subunit A<br>DNA gyrase subunit B<br>DNA gyrase subunit A<br>DNA topoisomerase 4 subunit A<br>DNA topoisomerase 4 subunit B	1.14E-31<br>7.00E-19<br>9.14E-45<br>6.07E-08<br>9.88E-06	0.43<br>0.43<br>0.44<br>0.43<br>0.43	*Escherichia coli K-12*	DNA gyrase subunit A<br>DNA gyrase subunit B		Unknown	*Escherichia coli K-12*<br>*Staphylococcus aureus subsp. aureus Mu50*<br>*Staphylococcus aureus*	DNA gyrase<br>DNA gyrase subunit B<br>Topoisomerase IV subunit A	0<br>0<br>0	Unknown		Unknown				Unknown	18048131626; 180428135834	DNA gyrase<br>DNA gyrase subunit B<br>Topoisomerase IV subunit A	Bioorg. Med. Chem. Lett.,(2006)16:5:1272; J. Med. Chem.,(1993)36:7:871; J. Med. Chem.,(1991)34:2:656; J. Med. Chem.,(1992)35:10:1764; J. Med. Chem.,(2003)46:17:3655<br>J. Med. Chem.,(1992)35:25:4745<br>Bioorg. Med. Chem. Lett.,(2004)14:20:5193; Bioorg. Med. Chem. Lett.,(2006)16:5:1272

Bioactive molecule	PASS online[4]			Similarity Ensemble Approach (SEA)[2]				ChemProt[3]		SuperPred[6]		Polypharmacology Browser (PPB)[1]			SPiDER[7]		HitPick[5]		
	Activity	Pa	Pi	Organism	Target	P-Value	Max TC	Organism	Target	Organism	Target	Organism	Target	P-Value	Target	Confidence level	Target	Precision (%)	Tc similarity
Azidamfenicol	Peptidyltransferase inhibitor	0.668	0.002		Unknown				Unknown		Unknown		Unknown		Unknown		Unknown		
Bromamphenicol (bromoamphenicol)	Peptidyltransferase inhibitor	0.688	0.002		Unknown				Unknown		Unknown		Unknown		Unknown		Unknown		
	Protein 50S ribosomal subunit inhibitor	0.041	0.040																
Cetofenicol (cetophenicol)	Peptidyltransferase inhibitor	0.943	0.000		Unknown				Unknown		Unknown		Unknown		Unknown		Unknown		
	Protein 50S ribosomal subunit inhibitor	0.175	0.006																
Chloramphenicol	Peptidyltransferase inhibitor	0.947	0.000		Unknown				Unknown		Unknown		Unknown		Unknown		Unknown		
	Protein 50S ribosomal subunit inhibitor	0.179	0.006																
Florfenicol	Peptidyltransferase inhibitor	0.852	0.001		Unknown				Unknown		Unknown		Unknown		Unknown		Unknown		
	Protein 50S ribosomal subunit inhibitor	0.259	0.003																
Monoiodoamphenicol	Peptidyltransferase inhibitor	0.688	0.002		Unknown				Unknown		Unknown		Unknown		Unknown		Unknown		
	Protein 50S ribosomal subunit inhibitor	0.041	0.040																
Racefenicol (racephenicol)	Peptidyltransferase inhibitor	0.940	0.000		Unknown				Unknown		Unknown		Unknown		Unknown		Unknown		
	Protein 50S ribosomal subunit inhibitor	0.169	0.007																
Tevenel	Peptidyltransferase inhibitor	0.939	0.000		Unknown				Unknown		Unknown		Unknown		Unknown		Unknown		
	Protein 50S ribosomal subunit inhibitor	0.167	0.007																
Thiamphenicol	Peptidyltransferase inhibitor	0.940	0.000		Unknown				Unknown		Unknown		Unknown		Unknown		Unknown		
	Protein 50S ribosomal subunit inhibitor	0.169	0.007																
WIN 5094-2	Peptidyltransferase inhibitor	0.943	0.000		Unknown				Unknown		Unknown		Unknown		Unknown		Unknown		
	Protein 50S ribosomal subunit inhibitor	0.176	0.006																

Bioactive molecule	PharmMapper[9]			TargetHunter[8]	
	Organism	Target	Job Id	Target	Reference
Azidamfenicol	*Haloarcula marismortui (strain ATCC 43049 / DSM 3752 / JCM 8966 / VKM B-1809) (Halobacterium marismortui)*	50S ribosomal protein L39e	180328120536	Unknown	
Bromamphenicol (bromoamphenicol)	*Haloarcula marismortui (strain ATCC 43049 / DSM 3752 / JCM 8966 / VKM B-1809) (Halobacterium marismortui)*	50S ribosomal protein L37e	180328120618	Unknown	
	Thermotoga maritima (strain ATCC 43589 / MSB8 / DSM 3109 / JCM 10099)	50S ribosomal protein L28			
		Ribosomal RNA large subunit methyltransferase H			
Cetofenicol (cetophenicol)	*Haloarcula marismortui (strain ATCC 43049 / DSM 3752 / JCM 8966 / VKM B-1809) (Halobacterium marismortui)*	50S ribosomal protein L39e	180328120712	Unknown	
	Thermotoga maritima (strain ATCC 43589 / MSB8 / DSM 3109 / JCM 10099)	50S ribosomal protein L28			
Chloramphenicol	*Haloarcula marismortui (strain ATCC 43049 / DSM 3752 / JCM 8966 / VKM B-1809) (Halobacterium marismortui)*	50S ribosomal protein L37e	180328120820	Unknown	
	Thermotoga maritima (strain ATCC 43589 / MSB8 / DSM 3109 / JCM 10099)	50S ribosomal protein L28			
Florfenicol	*Haloarcula marismortui (strain ATCC 43049 / DSM 3752 / JCM 8966 / VKM B-1809) (Halobacterium marismortui)*	50S ribosomal protein L37e	180328120916	Unknown	
Monoiodoamphenicol	*Haloarcula marismortui (strain ATCC 43049 / DSM 3752 / JCM 8966 / VKM B-1809) (Halobacterium marismortui)*	50S ribosomal protein L37e	180328121032	Unknown	
		50S ribosomal protein L39e			
	Methanothermococcus thermolithotrophicus (Methanococcus thermolithotrophicus)	50S ribosomal protein L1			
	Thermotoga maritima (strain ATCC 43589 / MSB8 / DSM 3109 / JCM 10099)	50S ribosomal protein L28			
Racefenicol (racephenicol)	*Haloarcula marismortui (strain ATCC 43049 / DSM 3752 / JCM 8966 / VKM B-1809) (Halobacterium marismortui)*	50S ribosomal protein L39e	180328121101	Unknown	
		50S ribosomal protein L37e			
	Thermotoga maritima (strain ATCC 43589 / MSB8 / DSM 3109 / JCM 10099)	50S ribosomal protein L28			
Tevenel	*Haloarcula marismortui (strain ATCC 43049 / DSM 3752 / JCM 8966 / VKM B-1809) (Halobacterium marismortui)*	50S ribosomal protein L37e	180328121129	Unknown	
	Thermotoga maritima (strain ATCC 43589 / MSB8 / DSM 3109 / JCM 10099)	50S ribosomal protein L28			
Thiamphenicol	*Haloarcula marismortui (strain ATCC 43049 / DSM 3752 / JCM 8966 / VKM B-1809) (Halobacterium marismortui)*	50S ribosomal protein L39e	180328121204	Unknown	
		50S ribosomal protein L37e			
	Thermotoga maritima (strain ATCC 43589 / MSB8 / DSM 3109 / JCM 10099)	50S ribosomal protein L28			
WIN 5094-2		Unknown	180328121231	Unknown	

Bioactive molecule	PASS online[4]			Similarity Ensemble Approach (SEA)[2]			
	Activity	Pa	Pi	Organism	Target	P-Value	Max TC
9-Methylstreptimidone (S 632A$_2$)	Protein 50S ribosomal subunit inhibitor	0.056	0.031	*Saccharomyces cerevisiae*	60S ribosomal protein L19-A	3.78E-06	0.36
Acetoxycycloheximide (streptovitacin E-73)	Peptidyltransferase inhibitor<br>Protein 50S ribosomal subunit inhibitor	0.414<br>0.189	0.008<br>0.005	*Saccharomyces cerevisiae*	60S ribosomal protein L19-A	2.72E-09	0.56
Actiketal	Unknown				Unknown		
Actiphenol (actinophenol)	Unknown				Unknown		
Cycloheximide	Peptidyltransferase inhibitor<br>Protein 50S ribosomal subunit inhibitor	0.381<br>0.158	0.010<br>0.007	*Saccharomyces cerevisiae*	60S ribosomal protein L19-A	4.44E-16	1.00
Epiderstatin	Unknown			*Saccharomyces cerevisiae*	60S ribosomal protein L19-A	1.09E-06	0.39
Inactone	Peptidyltransferase inhibitor<br>Protein 50S ribosomal subunit inhibitor	0.288<br>0.121	0.037<br>0.012	*Saccharomyces cerevisiae*	60S ribosomal protein L19-A	6.90E-08	0.47
Isocycloheximide	Peptidyltransferase inhibitor<br>Protein 50S ribosomal subunit inhibitor	0.381<br>0.158	0.010<br>0.007	*Saccharomyces cerevisiae*	60S ribosomal protein L19-A	4.44E-16	1.00
Isomigrastatin	Peptidyltransferase inhibitor<br>Protein 50S ribosomal subunit inhibitor	0.209<br>0.179	0.119<br>0.006		Unknown		
Lactimidomycin	Protein 50S ribosomal subunit inhibitor	0.160	0.007		Unknown		
Naramycin B	Peptidyltransferase inhibitor<br>Protein 50S ribosomal subunit inhibitor	0.381<br>0.158	0.010<br>0.007	*Saccharomyces cerevisiae*	60S ribosomal protein L19-A	4.44E-16	1.00
Neoisocycloheximide	Peptidyltransferase inhibitor<br>Protein 50S ribosomal subunit inhibitor	0.381<br>0.158	0.010<br>0.007	*Saccharomyces cerevisiae*	60S ribosomal protein L19-A	4.44E-16	1.00
S 632A$_3$	Protein 50S ribosomal subunit inhibitor	0.056	0.031	*Saccharomyces cerevisiae*	60S ribosomal protein L19-A	3.78E-06	0.36
Streptimidone (S 632A$_1$)	Peptidyltransferase inhibitor	0.184	0.172	*Saccharomyces cerevisiae*	60S ribosomal protein L19-A	3.00E-06	0.36
Streptovitacin A	Peptidyltransferase inhibitor<br>Protein 50S ribosomal subunit inhibitor	0.460<br>0.200	0.005<br>0.005	*Saccharomyces cerevisiae*	60S ribosomal protein L19-A	1.79E-10	0.64
Streptovitacin B	Peptidyltransferase inhibitor<br>Protein 50S ribosomal subunit inhibitor	0.392<br>0.194	0.009<br>0.005	*Saccharomyces cerevisiae*	60S ribosomal protein L19-A	1.79E-10	0.64
Streptovitacin C$_2$	Peptidyltransferase inhibitor<br>Protein 50S ribosomal subunit inhibitor	0.342<br>0.216	0.017<br>0.004	*Saccharomyces cerevisiae*	60S ribosomal protein L19-A	2.96E-10	0.62

Bioactive molecule	ChemProt[3]		SuperPred[6]		Polypharmacology Browser (PPB)[1]			SPiDER[7]	
	Organism	Target	Organism	Target	Organism	Target	P-Value	Target	Confidence level
9-Methylstreptimidone (S 632A$_2$)		Unknown		Unknown	*Saccharomyces cerevisiae S288c*	60S ribosomal protein L19-A	0	Unknown	
Acetoxycycloheximide (streptovitacin E-73)	*Saccharomyces cerevisiae S288c*	60S ribosomal protein L19-A		Unknown	*Saccharomyces cerevisiae S288c*	60S ribosomal protein L19-A	0	Unknown	
Actiketal		Unknown		Unknown		Unknown		Unknown	
Actiphenol (actinophenol)		Unknown		Unknown	*Saccharomyces cerevisiae S288c*	60S ribosomal protein L19-A	0	Unknown	
Cycloheximide	*Saccharomyces cerevisiae S288c*	60S ribosomal protein L19-A		Unknown	*Saccharomyces cerevisiae S288c*	60S ribosomal protein L19-A	0	Unknown	
Epiderstatin		Unknown		Unknown	*Saccharomyces cerevisiae S288c*	60S ribosomal protein L19-A	0.01 to 0	Unknown	
Inactone	*Saccharomyces cerevisiae S288c*	60S ribosomal protein L19-A		Unknown	*Saccharomyces cerevisiae S288c*	60S ribosomal protein L19-A	0	Unknown	
Isocycloheximide	*Saccharomyces cerevisiae S288c*	60S ribosomal protein L19-A		Unknown	*Saccharomyces cerevisiae S288c*	60S ribosomal protein L19-A	0	Unknown	
Isomigrastatin		Unknown		Unknown		Unknown		Bacterial Ribosome Binding On 50S Subunit	0.043
Lactimidomycin		Unknown		Unknown		Unknown		Bacterial Ribosome Binding On 50S Subunit	0.049
Naramycin B	*Saccharomyces cerevisiae S288c*	60S ribosomal protein L19-A		Unknown	*Saccharomyces cerevisiae S288c*	60S ribosomal protein L19-A	0	Unknown	
Neoisocycloheximide	*Saccharomyces cerevisiae S288c*	60S ribosomal protein L19-A		Unknown	*Saccharomyces cerevisiae S288c*	60S ribosomal protein L19-A	0	Unknown	
S 632A$_3$		Unknown		Unknown	*Saccharomyces cerevisiae S288c*	60S ribosomal protein L19-A	0	Unknown	
Streptimidone (S 632A$_1$)		Unknown		Unknown	*Saccharomyces cerevisiae S288c*	60S ribosomal protein L19-A	0	Unknown	
Streptovitacin A		Unknown		Unknown		Unknown		Unknown	
Streptovitacin B	*Saccharomyces cerevisiae S288c*	60S ribosomal protein L19-A		Unknown	*Saccharomyces cerevisiae S288c*	60S ribosomal protein L19-A	0	Unknown	
Streptovitacin C$_2$		Unknown		Unknown		Unknown		Unknown	

Bioactive molecule	HitPick[5]			PharmMapper[9]			TargetHunter[8]	
	Target	Precision (%)	Tc similarity	Organism	Target	Job Id	Target	Reference
9-Methylstreptimidone (S 632A$_2$)	Unknown				Unknown	18044170316	60S ribosomal protein L19-A	PubChem AID:
Acetoxycycloheximide (streptovitacin E-73)	Unknown			*Haloarcula marismortui (strain ATCC 43049 / DSM 3752 / JCM 8966 / VKM B-1809) (Halobacterium marismortui)*	50S ribosomal protein L37e	180329140202	60S ribosomal protein L19-A	PubChem AID:
Actiketal	Unknown				Unknown	180329140235	Unknown	
Actiphenol (actinophenol)	Unknown				Unknown	180329140342	Unknown	
Cycloheximide	Unknown				Unknown	180329140424	60S ribosomal protein L19-A	PubChem AID:
Epiderstatin	Unknown			*Haloarcula marismortui (strain ATCC 43049 / DSM 3752 / JCM 8966 / VKM B-1809) (Halobacterium marismortui)*<br>*Thermotoga maritima (strain ATCC 43589 / MSB8 / DSM 3109 / JCM 10099)*	50S ribosomal protein L39e<br>Ribosomal RNA large subunit methyltransferase H	180329140500	Unknown	
Inactone	Unknown			*Thermotoga maritima (strain ATCC 43589 / MSB8 / DSM 3109 / JCM 10099)*	Ribosomal RNA large subunit methyltransferase H	180329140529	60S ribosomal protein L19-A	PubChem AID:
Isocycloheximide	Unknown			*Thermotoga maritima (strain ATCC 43589 / MSB8 / DSM 3109 / JCM 10099)*	Ribosomal RNA large subunit methyltransferase H	18044170435	60S ribosomal protein L19-A	PubChem AID:
Isomigrastatin	Unknown			*Methanocaldococcus jannaschii (strain ATCC 43067 / DSM 2661 / JAL-1 / JCM 10045 / NBRC 100440) (Methanococcus jannaschii)*	50S ribosomal protein L1P	18044170529	Unknown	
Lactimidomycin	Unknown			*Deinococcus radiodurans (strain ATCC 13939 / DSM 20539 / JCM 16871 / LMG 4051 / NBRC 15346 / NCIMB 9279 / R1 / VKM B-1422)*<br>*Methanocaldococcus jannaschii (strain ATCC 43067 / DSM 2661 / JAL-1 / JCM 10045 / NBRC 100440) (Methanococcus jannaschii)*	50S ribosomal protein L33<br>50S ribosomal protein L1P	180329140602	Unknown	
Naramycin B	Unknown				Unknown	18044170608	60S ribosomal protein L19-A	PubChem AID:
Neoisocycloheximide	Unknown			*Thermotoga maritima (strain ATCC 43589 / MSB8 / DSM 3109 / JCM 10099)*	Ribosomal RNA large subunit methyltransferase H	18044170653	60S ribosomal protein L19-A	PubChem AID:
S 632A$_3$	Unknown				Unknown	18044170734	60S ribosomal protein L19-A	PubChem AID:
Streptimidone (S 632A$_1$)	Unknown			*Thermotoga maritima (strain ATCC 43589 / MSB8 / DSM 3109 / JCM 10099)*	Ribosomal RNA large subunit methyltransferase H	18044170820	60S ribosomal protein L19-A	PubChem AID:
Streptovitacin A	Unknown				Unknown	180329140654	60S ribosomal protein L19-A	PubChem AID:
Streptovitacin B	Unknown			*Haloarcula marismortui (strain ATCC 43049 / DSM 3752 / JCM 8966 / VKM B-1809) (Halobacterium marismortui)*<br>*Thermotoga maritima (strain ATCC 43589 / MSB8 / DSM 3109 / JCM 10099)*	50S ribosomal protein L39e<br>Ribosomal RNA large subunit methyltransferase H	180329140731	60S ribosomal protein L19-A	PubChem AID:
Streptovitacin C$_2$	Unknown			*Haloarcula marismortui (strain ATCC 43049 / DSM 3752 / JCM 8966 / VKM B-1809) (Halobacterium marismortui)*	50S ribosomal protein L39e	180329140817	60S ribosomal protein L19-A	PubChem AID:

Bioactive molecule	PASS online[4]			Similarity Ensemble Approach (SEA)[2]				ChemProt[3]		SuperPred[6]		Polypharmacology Browser (PPB)[1]			SPiDER[7]		HitPick[5]			PharmMapper[9]			TargetHunter[8]	
	Activity	Pa	Pi	Organism	Target	P-Value	Max TC	Organism	Target	Organism	Target	Organism	Target	P-Value	Target	Confidence level	Target	Precision (%)	Tc similarity	Organism	Target	Job Id	Target	Reference
Amicetin (allomycin)	Peptidyltransferase inhibitor	0.893	0.001		Unknown				Unknown		Unknown		Unknown		Unknown		Unknown			*Haloarcula marismortui (strain ATCC 43049 / DSM 3752 / JCM 8966 / VKM B-1809) (Halobacterium marismortui)*	50S ribosomal protein L37e	180329155000	Unknown	
	Protein 50S ribosomal subunit inhibitor	0.123	0.012																	*Methanocaldococcus jannaschii (strain ATCC 43067 / DSM 2661 / JAL-1 / JCM 10045 / NBRC 100440) (Methanococcus jannaschii)*	50S ribosomal protein L1P			
Antelmycin (anthelmycin)	Peptidyltransferase inhibitor	0.552	0.003		Unknown				Unknown		Unknown		Unknown		Unknown		Unknown				Unknown	18044172404	Unknown	
	Protein 50S ribosomal subunit inhibitor	0.096	0.018																					
Arginomycin	Peptidyltransferase inhibitor	0.788	0.001		Unknown				Unknown		Unknown		Unknown		Unknown		Unknown				Unknown	18044172459	Unknown	
Bagougeramine A	Peptidyltransferase inhibitor	0.689	0.002		Unknown				Unknown		Unknown		Unknown		Unknown		Unknown				Unknown	18044172629	Unknown	
Bagougeramine B	Peptidyltransferase inhibitor	0.667	0.002		Unknown				Unknown		Unknown		Unknown		Unknown		Unknown				Unknown	18044172731	Unknown	
Bamicetin	Peptidyltransferase inhibitor	0.828	0.001		Unknown				Unknown		Unknown		Unknown		Unknown		Unknown			*Methanocaldococcus jannaschii (strain ATCC 43067 / DSM 2661 / JAL-1 / JCM 10045 / NBRC 100440) (Methanococcus jannaschii)*	50S ribosomal protein L1P	180329155040	Unknown	
	Protein 50S ribosomal subunit inhibitor	0.102	0.016																					
Blasticidin H	Peptidyltransferase inhibitor	0.712	0.002		Unknown				Unknown		Unknown		Unknown		Unknown		Unknown			*Haloarcula marismortui (strain ATCC 43049 / DSM 3752 / JCM 8966 / VKM B-1809) (Halobacterium marismortui)*	50S ribosomal protein L39e	180329155130	Unknown	
																				Homo sapiens	60S ribosomal protein L9			
Blasticidin S	Peptidyltransferase inhibitor	0.846	0.001		Unknown				Unknown		Unknown		Unknown		Unknown		Unknown			*Haloarcula marismortui (strain ATCC 43049 / DSM 3752 / JCM 8966 / VKM B-1809) (Halobacterium marismortui)*	50S ribosomal protein L37e	180329155203	Unknown	
Cytimidine	Peptidyltransferase inhibitor	0.710	0.002		Unknown				Unknown		Unknown		Unknown		Unknown		Unknown			*Escherichia coli (strain K12)*	Ribosomal large subunit pseudouridine synthase F	18044173329	Unknown	
Cytomycin (saitomycin)	Peptidyltransferase inhibitor	0.743	0.002		Unknown				Unknown		Unknown		Unknown		Unknown		Unknown				Unknown	180412111434	Unknown	
Cytosamine	Peptidyltransferase inhibitor	0.911	0.001		Unknown				Unknown		Unknown		Unknown		Unknown		Unknown				Unknown	180412111512	Unknown	
	Protein 50S ribosomal subunit inhibitor	0.194	0.005																					
Cytosaminomycin A	Peptidyltransferase inhibitor	0.793	0.001		Unknown				Unknown		Unknown		Unknown		Unknown		Unknown			*Thermotoga maritima (strain ATCC 43589 / MSB8 / DSM 3109 / JCM 10099)*	Ribosomal RNA large subunit methyltransferase H	18044173652	Unknown	
	Protein 50S ribosomal subunit inhibitor	0.141	0.009																					
Cytosaminomycin B	Peptidyltransferase inhibitor	0.814	0.001		Unknown				Unknown		Unknown		Unknown		Unknown		Unknown			*Methanocaldococcus jannaschii (strain ATCC 43067 / DSM 2661 / JAL-1 / JCM 10045 / NBRC 100440) (Methanococcus jannaschii)*	50S ribosomal protein L1P	18044173727	Unknown	
	Protein 50S ribosomal subunit inhibitor	0.142	0.009																					
Cytosaminomycin C	Peptidyltransferase inhibitor	0.814	0.001		Unknown				Unknown		Unknown		Unknown		Unknown		Unknown				Unknown	18044173755	Unknown	
	Protein 50S ribosomal subunit inhibitor	0.155	0.008																					
Cytosaminomycin D	Peptidyltransferase inhibitor	0.820	0.001		Unknown				Unknown		Unknown		Unknown		Unknown		Unknown				Unknown	18044173829	Unknown	
	Protein 50S ribosomal subunit inhibitor	0.155	0.008																					
Gougerotin	Peptidyltransferase inhibitor	0.731	0.002		Unknown				Unknown		Unknown		Unknown		Unknown		Unknown				Unknown	180329155237	Unknown	
Mildiomycin	Peptidyltransferase inhibitor	0.660	0.002		Unknown				Unknown		Unknown		Unknown		Unknown		Unknown			*Haloarcula marismortui (strain ATCC 43049 / DSM 3752 / JCM 8966 / VKM B-1809) (Halobacterium marismortui)*	50S ribosomal protein L39e	180329155726	Unknown	
																				Thermotoga maritima (strain ATCC 43589 / MSB8 / DSM 3109 / JCM 10099)	Ribosomal RNA large subunit methyltransferase H			
Mildiomycin B	Peptidyltransferase inhibitor	0.665	0.002		Unknown				Unknown		Unknown		Unknown		Unknown		Unknown				Unknown	180329155337	Unknown	
Mildiomycin C	Peptidyltransferase inhibitor	0.711	0.002		Unknown				Unknown		Unknown		Unknown		Unknown		Unknown				Unknown	180329155428	Unknown	
Mildiomycin D	Peptidyltransferase inhibitor	0.681	0.002		Unknown				Unknown		Unknown		Unknown		Unknown		Unknown				Unknown	180329155529	Unknown	
Mildiomycin M	Peptidyltransferase inhibitor	0.665	0.002		Unknown				Unknown		Unknown		Unknown		Unknown		Unknown				Unknown	180329155655	Unknown	
Norplicacetin	Peptidyltransferase inhibitor	0.772	0.001		Unknown				Unknown		Unknown		Unknown		Unknown		Unknown			*Methanocaldococcus jannaschii (strain ATCC 43067 / DSM 2661 / JAL-1 / JCM 10045 / NBRC 100440) (Methanococcus jannaschii)*	50S ribosomal protein L1P	18044194440	Unknown	
	Protein 50S ribosomal subunit inhibitor	0.121	0.012																					
Oxamicetin	Peptidyltransferase inhibitor	0.857	0.001		Unknown				Unknown		Unknown		Unknown		Unknown		Unknown				Unknown	18044194524	Unknown	
	Protein 50S ribosomal subunit inhibitor	0.117	0.013																					
Oxyplicacetin (cytosaminomycin E)	Peptidyltransferase inhibitor	0.808	0.001		Unknown				Unknown		Unknown		Unknown		Unknown		Unknown			*Thermotoga maritima (strain ATCC 43589 / MSB8 / DSM 3109 / JCM 10099)*	Ribosomal RNA large subunit methyltransferase H	18044194555	Unknown	
	Protein 50S ribosomal subunit inhibitor	0.14	0.009																					
Plicacetin (amicetin B)	Peptidyltransferase inhibitor	0.854	0.001		Unknown				Unknown		Unknown		Unknown		Unknown		Unknown			*Haloarcula marismortui (strain ATCC 43049 / DSM 3752 / JCM 8966 / VKM B-1809) (Halobacterium marismortui)*	50S ribosomal protein L37e	18044194648	Unknown	
	Protein 50S ribosomal subunit inhibitor	0.147	0.009																					
Rodaplutin	Peptidyltransferase inhibitor	0.739	0.002		Unknown				Unknown		Unknown		Unknown		Unknown		Unknown				Unknown	18044194732	Unknown	
SCH 36605	Peptidyltransferase inhibitor	0.739	0.002		Unknown				Unknown		Unknown		Unknown		Unknown		Unknown				Unknown	18044194833	Unknown	
SF 2457	Peptidyltransferase inhibitor	0.703	0.002		Unknown				Unknown		Unknown		Unknown		Unknown		Unknown				Unknown	18044194907	Unknown	
	Protein 50S ribosomal subunit inhibitor	0.105	0.016																					

Bioactive molecule	PASS online[4]			Similarity Ensemble Approach (SEA)[2]				ChemProt[3]		SuperPred[6]		Polypharmacology Browser (PPB)[1]			SPiDER[7]		HitPick[5]			PharmMapper[9]			TargetHunter[8]	
	Activity	Pa	Pi	Organism	Target	P-Value	Max TC	Organism	Target	Organism	Target	Organism	Target	P-Value	Target	Confidence level	Target	Precision (%)	Tc similarity	Organism	Target	Job Id	Target	Reference
1-Epidactimicin	Protein 30S ribosomal subunit inhibitor	0.732	0.000		Unknown				Unknown		Unknown		Unknown		Unknown		Unknown			*Escherichia coli (strain K12)*<br>*Methanosarcina mazei (strain ATCC BAA-159 / DSM 3647 / Goe1 / Go1 / JCM 11833 / OCM 88) (Methanosarcina frisia)*	Ribosomal small subunit pseudouridine synthase A<br>30S ribosomal protein S24e	180330123657	Ribosomal RNA A-site	Bioorg. Med. Chem. Lett.,(2001)11:2:119; J. Med. Chem.,(2006)49:18:5478; J. Med. Chem.,(2009)52:9:2836
A 396I (SS 56D)	Protein 30S ribosomal subunit inhibitor	0.351	0.001		Unknown			*Streptomyces coelicolor (strain ATCC BAA-471 / A3(2) / M145)*	30S ribosomal protein S12		Unknown		Unknown		Unknown		Unknown			*Escherichia coli (strain K12)*	Ribosomal small subunit pseudouridine synthase A	18046155058	Unknown	
Ambistrin (streptoduocin)	Protein 30S ribosomal subunit inhibitor	0.426	0.001		Unknown			*Homo sapiens*	40S ribosomal protein S2; 40S ribosomal protein S3; 40S ribosomal protein S5, N-terminally processed; 40S ribosomal protein S14; 40S ribosomal protein S15; 40S ribosomal protein S16; 40S ribosomal protein S20		Unknown		Unknown		Unknown		Unknown			*Escherichia coli (strain K12)*<br>*Methanococcus maripaludis (strain S2 / LL)*	Ribosomal small subunit pseudouridine synthase A<br>30S ribosomal protein S24e	18045182631	30S ribosomal protein S12<br>Ribosomal RNA A-site	Antimicrob. Agents Chemother.,(2009)53:3:1019<br>Bioorg. Med. Chem. Lett.,(2001)11:2:119; Bioorg. Med. Chem. Lett.,(2004)14:17:4389; Bioorg. Med. Chem. Lett.,(2005)15:22:4919; J. Med. Chem.,(2006)49:18:5478; J. Med. Chem.,(2009)52:9:2836
Amikacin	Protein 30S ribosomal subunit inhibitor	0.489	0.001		Unknown				Unknown		Unknown		Unknown		Unknown		Unknown				Unknown	18045081648	30S ribosomal protein S12<br>Ribosomal RNA A-site	Antimicrob. Agents Chemother.,(2009)53:3:1019<br>Bioorg. Med. Chem. Lett.,(2001)11:2:119; Bioorg. Med. Chem. Lett.,(2004)14:17:4389; Bioorg. Med. Chem. Lett.,(2005)15:22:4919; J. Med. Chem.,(2006)49:18:5478; J. Med. Chem.,(2009)52:9:2836
Apramycin (nebramycin II)	Protein 30S ribosomal subunit inhibitor	0.813	0.000		Unknown				Unknown		Unknown		Unknown		Unknown		Unknown			*Escherichia coli (strain K12)*	Ribosomal small subunit pseudouridine synthase A	18045143620	Unknown	
Aprosamine	Protein 30S ribosomal subunit inhibitor	0.853	0.000		Unknown				Unknown		Unknown		Unknown		Unknown		Unknown			*Escherichia coli (strain K12)*	Ribosomal RNA small subunit methyltransferase F	18046155132	Unknown	
Arbekacin	Protein 30S ribosomal subunit inhibitor	0.626	0.000		Unknown				Unknown		Unknown		Unknown		Unknown		Unknown			*Escherichia coli (strain K12)*	Ribosomal RNA small subunit methyltransferase F<br>Ribosomal small subunit pseudouridine synthase A	180330123731	30S ribosomal protein S12<br>Ribosomal RNA A-site	Antimicrob. Agents Chemother.,(2009)53:3:1019<br>Bioorg. Med. Chem. Lett.,(2001)11:2:119; Bioorg. Med. Chem. Lett.,(2004)14:17:4389; Bioorg. Med. Chem. Lett.,(2005)15:22:4919; J. Med. Chem.,(2006)49:18:5478; J. Med. Chem.,(2009)52:9:2836
Astromicin (fortimicin A)	Protein 30S ribosomal subunit inhibitor	0.706	0.000		Unknown				Unknown		Unknown		Unknown		Unknown		Unknown			*Escherichia coli (strain K12)*	Ribosomal small subunit pseudouridine synthase A	18045194242	30S ribosomal protein S12<br>Ribosomal RNA A-site	Antimicrob. Agents Chemother.,(2009)53:3:1019<br>Bioorg. Med. Chem. Lett.,(2001)11:2:119; Bioorg. Med. Chem. Lett.,(2004)14:17:4389; Bioorg. Med. Chem. Lett.,(2005)15:22:4919; J. Med. Chem.,(2006)49:18:5478; J. Med. Chem.,(2009)52:9:2836
Astromicin B (fortimicin B)	Protein 30S ribosomal subunit inhibitor	0.821	0.000		Unknown				Unknown		Unknown		Unknown		Unknown		Unknown				Unknown	18045201157	Unknown	
Bekanamycin (kanamycin B; nebramycin V)	Protein 30S ribosomal subunit inhibitor	0.707	0.000		Unknown			*Streptomyces coelicolor (strain ATCC BAA-471 / A3(2) / M145)*	30S ribosomal protein S12		Unknown		Unknown		Unknown		Unknown				Unknown	180330123808	30S ribosomal protein S12<br>Ribosomal RNA A-site	Antimicrob. Agents Chemother.,(2009)53:3:1019<br>Bioorg. Med. Chem. Lett.,(2001)11:2:119; Bioorg. Med. Chem. Lett.,(2004)14:17:4389; Bioorg. Med. Chem. Lett.,(2005)15:22:4919; J. Med. Chem.,(2006)49:18:5478; J. Med. Chem.,(2009)52:9:2836
Betamicin (gentamicin B)	Protein 30S ribosomal subunit inhibitor	0.818	0.000		Unknown				Unknown		Unknown		Unknown		Unknown		Unknown			*Escherichia coli (strain K12)*	Ribosomal small subunit pseudouridine synthase A	18045073359	Unknown	
Butikacin	Protein 30S ribosomal subunit inhibitor	0.487	0.001		Unknown			*Streptomyces coelicolor (strain ATCC BAA-471 / A3(2) / M145)*	30S ribosomal protein S12		Unknown		Unknown		Unknown		Unknown			*Escherichia coli (strain K12)*	Ribosomal small subunit pseudouridine synthase A	18045081743	Unknown	
Butirosin A	Protein 30S ribosomal subunit inhibitor	0.527	0.000		Unknown				Unknown		Unknown		Unknown		Unknown		Unknown			*Escherichia coli (strain K12)*	Ribosomal RNA small subunit methyltransferase F	18045081817	30S ribosomal protein S12<br>Ribosomal RNA A-site	Antimicrob. Agents Chemother.,(2009)53:3:1019<br>Bioorg. Med. Chem. Lett.,(2001)11:2:119; Bioorg. Med. Chem. Lett.,(2004)14:17:4389; Bioorg. Med. Chem. Lett.,(2005)15:22:4919; J. Med. Chem.,(2006)49:18:5478; J. Med. Chem.,(2009)52:9:2836
Butirosin B	Protein 30S ribosomal subunit inhibitor	0.527	0.000		Unknown				Unknown		Unknown		Unknown		Unknown		Unknown			*Escherichia coli (strain K12)*	Ribosomal small subunit pseudouridine synthase A	18045081846	30S ribosomal protein S12<br>Ribosomal RNA A-site	Antimicrob. Agents Chemother.,(2009)53:3:1019<br>Bioorg. Med. Chem. Lett.,(2001)11:2:119; Bioorg. Med. Chem. Lett.,(2004)14:17:4389; Bioorg. Med. Chem. Lett.,(2005)15:22:4919; J. Med. Chem.,(2006)49:18:5478; J. Med. Chem.,(2009)52:9:2836
Dactimicin	Protein 30S ribosomal subunit inhibitor	0.732	0.000		Unknown				Unknown		Unknown		Unknown		Unknown		Unknown				Unknown	180330123846	Ribosomal RNA A-site	Bioorg. Med. Chem. Lett.,(2001)11:2:119; J. Med. Chem.,(2006)49:18:5478; J. Med. Chem.,(2009)52:9:2836
Destomycin A	Protein 30S ribosomal subunit inhibitor	0.363	0.001		Unknown				Unknown		Unknown		Unknown		Unknown		Unknown				Unknown	18046144232	Unknown	
Destomycin B	Protein 30S ribosomal subunit inhibitor	0.337	0.001		Unknown				Unknown		Unknown		Unknown		Unknown		Unknown			*Escherichia coli (strain K12)*	Ribosomal small subunit pseudouridine synthase A	18046155211	Unknown	
Dibekacin	Protein 30S ribosomal subunit inhibitor	0.758	0.000		Unknown			*Streptomyces coelicolor (strain ATCC BAA-471 / A3(2) / M145)*	30S ribosomal protein S12		Unknown		Unknown		Unknown		Unknown			*Escherichia coli (strain K12)*	Ribosomal RNA small subunit methyltransferase F	18045073453	30S ribosomal protein S12<br>Ribosomal RNA A-site	Antimicrob. Agents Chemother.,(2009)53:3:1019<br>Bioorg. Med. Chem. Lett.,(2001)11:2:119; Bioorg. Med. Chem. Lett.,(2004)14:17:4389; Bioorg. Med. Chem. Lett.,(2005)15:22:4919; J. Med. Chem.,(2006)49:18:5478; J. Med. Chem.,(2009)52:9:2836
Dihydrostreptomycin	Protein 30S ribosomal subunit inhibitor	0.426	0.001		Unknown				Unknown		Unknown		Unknown		Unknown		Unknown			*Escherichia coli (strain K12)*<br>*Geobacillus stearothermophilus (Bacillus stearothermophilus)*	Ribosomal RNA small subunit methyltransferase F<br>30S ribosomal protein S17	180330123924	30S ribosomal protein S12<br>Ribosomal RNA A-site	Antimicrob. Agents Chemother.,(2009)53:3:1019<br>Bioorg. Med. Chem. Lett.,(2001)11:2:119; Bioorg. Med. Chem. Lett.,(2004)14:17:4389; Bioorg. Med. Chem. Lett.,(2005)15:22:4919; J. Med. Chem.,(2006)49:18:5478; J. Med. Chem.,(2009)52:9:2836
Etimicin	Protein 30S ribosomal subunit inhibitor	0.872	0.000		Unknown				Unknown		Unknown		Unknown		Unknown		Unknown			*Escherichia coli (strain K12)*<br>*Methanococcus maripaludis (strain S2 / LL)*	Ribosomal RNA small subunit methyltransferase F<br>30S ribosomal protein S24e	180419170520	Unknown	
Fortimicin AE	Protein 30S ribosomal subunit inhibitor	0.821	0.000		Unknown				Unknown		Unknown		Unknown		Unknown		Unknown			*Methanococcus maripaludis (strain S2 / LL)*	30S ribosomal protein S24e	18045194348	Unknown	
Fortimicin AH	Protein 30S ribosomal subunit inhibitor	0.396	0.001		Unknown				Unknown		Unknown		Unknown		Unknown		Unknown			*Escherichia coli (strain K12)*	Ribosomal RNA small subunit methyltransferase F	18045194434	Ribosomal RNA A-site	J. Med. Chem.,(2006)49:18:5478
Fortimicin AI	Protein 30S ribosomal subunit inhibitor	0.396	0.001		Unknown				Unknown		Unknown		Unknown		Unknown		Unknown			*Escherichia coli (strain K12)*	Ribosomal RNA small subunit methyltransferase F	18045194510	Ribosomal RNA A-site	J. Med. Chem.,(2006)49:18:5478
Fortimicin AK	Protein 30S ribosomal subunit inhibitor	0.609	0.000		Unknown				Unknown		Unknown		Unknown		Unknown		Unknown			*Escherichia coli (strain K12)*<br>*Methanosarcina mazei (strain ATCC BAA-159 / DSM 3647 / Goe1 / Go1 / JCM 11833 / OCM 88) (Methanosarcina frisia)*	Ribosomal RNA small subunit methyltransferase F<br>30S ribosomal protein S24e	18045194548	Unknown	
Fortimicin AL	Protein 30S ribosomal subunit inhibitor	0.847	0.000		Unknown				Unknown		Unknown		Unknown		Unknown		Unknown			*Escherichia coli (strain K12)*<br>*Methanosarcina mazei (strain ATCC BAA-159 / DSM 3647 / Goe1 / Go1 / JCM 11833 / OCM 88) (Methanosarcina frisia)*	Ribosomal RNA small subunit methyltransferase F<br>30S ribosomal protein S24e	18045194614	Unknown	
Fortimicin AM	Protein 30S ribosomal subunit inhibitor	0.876	0.000		Unknown				Unknown		Unknown		Unknown		Unknown		Unknown				Unknown	18045194651	Unknown	
Fortimicin AN	Protein 30S ribosomal subunit inhibitor	0.692	0.000		Unknown				Unknown		Unknown		Unknown		Unknown		Unknown				Unknown	18045194724	30S ribosomal protein S12<br>Ribosomal RNA A-site	Antimicrob. Agents Chemother.,(2009)53:3:1019<br>Bioorg. Med. Chem. Lett.,(2001)11:2:119; Bioorg. Med. Chem. Lett.,(2004)14:17:4389; Bioorg. Med. Chem. Lett.,(2005)15:22:4919; J. Med. Chem.,(2006)49:18:5478; J. Med. Chem.,(2009)52:9:2836
Fortimicin AO	Protein 30S ribosomal subunit inhibitor	0.615	0.000		Unknown				Unknown		Unknown		Unknown		Unknown		Unknown				Unknown	18045194752	Unknown	
Fortimicin AP	Protein 30S ribosomal subunit inhibitor	0.876	0.000		Unknown				Unknown		Unknown		Unknown		Unknown		Unknown			*Methanococcus maripaludis (strain S2 / LL)*	30S ribosomal protein S24e	18045194817	Unknown	
Fortimicin AQ	Protein 30S ribosomal subunit inhibitor	0.785	0.000		Unknown				Unknown		Unknown		Unknown		Unknown		Unknown				Unknown	18045201300	Unknown	
Fortimicin AS	Protein 30S ribosomal subunit inhibitor	0.623	0.000		Unknown				Unknown		Unknown		Unknown		Unknown		Unknown				Unknown	18045201410	30S ribosomal protein S12<br>Ribosomal RNA A-site	Antimicrob. Agents Chemother.,(2009)53:3:1019<br>Bioorg. Med. Chem. Lett.,(2001)11:2:119; Bioorg. Med. Chem. Lett.,(2004)14:17:4389; Bioorg. Med. Chem. Lett.,(2005)15:22:4919; J. Med. Chem.,(2006)49:18:5478; J. Med. Chem.,(2009)52:9:2836
Fortimicin C	Protein 30S ribosomal subunit inhibitor	0.579	0.000		Unknown				Unknown		Unknown		Unknown		Unknown		Unknown			*Escherichia coli (strain K12)*	Ribosomal small subunit pseudouridine synthase A	18045201444	Ribosomal RNA A-site	Bioorg. Med. Chem. Lett.,(2001)11:2:119; J. Med. Chem.,(2006)49:18:5478; J. Med. Chem.,(2009)52:9:2836
Fortimicin D	Protein 30S ribosomal subunit inhibitor	0.591	0.000		Unknown				Unknown		Unknown		Unknown		Unknown		Unknown			*Escherichia coli (strain K12)*<br>*Thermus thermophilus (strain HB8 / ATCC 27634 / DSM 579)*<br>*Methanosarcina mazei (strain ATCC BAA-159 / DSM 3647 / Goe1 / Go1 / JCM 11833 / OCM 88) (Methanosarcina frisia)*	Ribosomal RNA small subunit methyltransferase F<br>Ribosomal RNA small subunit methyltransferase G<br>30S ribosomal protein S24e	18045201517	30S ribosomal protein S12<br>Ribosomal RNA A-site	Antimicrob. Agents Chemother.,(2009)53:3:1019<br>Bioorg. Med. Chem. Lett.,(2001)11:2:119; Bioorg. Med. Chem. Lett.,(2004)14:17:4389; Bioorg. Med. Chem. Lett.,(2005)15:22:4919; J. Med. Chem.,(2006)49:18:5478; J. Med. Chem.,(2009)52:9:2836
Fortimicin E (fortimicin KH)	Protein 30S ribosomal subunit inhibitor	0.821	0.000		Unknown				Unknown		Unknown		Unknown		Unknown		Unknown			*Escherichia coli (strain K12)*	Ribosomal RNA small subunit methyltransferase F	18045201548	Unknown	
Fortimicin KE	Protein 30S ribosomal subunit inhibitor	0.678	0.000		Unknown				Unknown		Unknown		Unknown		Unknown		Unknown			*Escherichia coli (strain K12)*<br>*Methanosarcina mazei (strain ATCC BAA-159 / DSM 3647 / Goe1 / Go1 / JCM 11833 / OCM 88) (Methanosarcina frisia)*	Ribosomal RNA small subunit methyltransferase F<br>30S ribosomal protein S24e	18045201631	Unknown	
Fortimicin KF	Protein 30S ribosomal subunit inhibitor	0.769	0.000		Unknown				Unknown		Unknown		Unknown		Unknown		Unknown			*Escherichia coli (strain K12)*	Ribosomal RNA small subunit methyltransferase F	18045201703	Ribosomal RNA A-site	Bioorg. Med. Chem. Lett.,(2001)11:2:119; J. Med. Chem.,(2006)49:18:5478
Fortimicin KG	Protein 30S ribosomal subunit inhibitor	0.788	0.000		Unknown				Unknown		Unknown		Unknown		Unknown		Unknown			*Escherichia coli (strain K12)*<br>*Methanosarcina mazei (strain ATCC BAA-159 / DSM 3647 / Goe1 / Go1 / JCM 11833 / OCM 88) (Methanosarcina frisia)*	Ribosomal RNA small subunit methyltransferase F<br>30S ribosomal protein S24e	18045201728	Unknown	
Fortimicin KL_1	Protein 30S ribosomal subunit inhibitor	0.695	0.000		Unknown				Unknown		Unknown		Unknown		Unknown		Unknown			*Escherichia coli (strain K12)*<br>*Methanococcus maripaludis (strain S2 / LL)*	Ribosomal small subunit pseudouridine synthase A<br>30S ribosomal protein S24e	18045201753	Unknown	
Fortimicin KR	Protein 30S ribosomal subunit inhibitor	0.821	0.000		Unknown				Unknown		Unknown		Unknown		Unknown		Unknown				Unknown	18045222421	Unknown	
Framycetin (neomycin B)	Protein 30S ribosomal subunit inhibitor	0.606	0.000		Unknown			*Streptomyces coelicolor (strain ATCC BAA-471 / A3(2) / M145)*	30S ribosomal protein S12		Unknown		Unknown		Unknown		Unknown			*Escherichia coli (strain K12)*	Ribosomal small subunit pseudouridine synthase A	18045143713	30S ribosomal protein S12<br>Ribosomal RNA A-site	Antimicrob. Agents Chemother.,(2009)53:3:1019<br>Bioorg. Med. Chem. Lett.,(2001)11:2:119; Bioorg. Med. Chem. Lett.,(2004)14:17:4389; Bioorg. Med. Chem. Lett.,(2005)15:22:4919; J. Med. Chem.,(2006)49:18:5478; J. Med. Chem.,(2009)52:9:2836
Geneticin (Gentamicin G-418)	Protein 30S ribosomal subunit inhibitor	0.863	0.000		Unknown				Unknown		Unknown		Unknown		Unknown		Unknown			*Escherichia coli (strain K12)*	Ribosomal RNA small subunit methyltransferase F<br>Ribosomal small subunit pseudouridine synthase A	180422222657	Unknown	
Gentamicin A	Protein 30S ribosomal subunit inhibitor	0.626	0.000		Unknown				Unknown		Unknown		Unknown		Unknown		Unknown			*Escherichia coli (strain K12)*	Ribosomal small subunit pseudouridine synthase A	18045073543	Unknown	
Gentamicin A_1	Protein 30S ribosomal subunit inhibitor	0.626	0.000		Unknown				Unknown		Unknown		Unknown		Unknown		Unknown			*Escherichia coli (strain K12)*	Ribosomal small subunit pseudouridine synthase A	18045073618	Unknown	
Gentamicin A_2	Protein 30S ribosomal subunit inhibitor	0.588	0.000		Unknown			*Streptomyces coelicolor (strain ATCC BAA-471 / A3(2) / M145)*	30S ribosomal protein S12		Unknown		Unknown		Unknown		Unknown			*Escherichia coli (strain K12)*	Ribosomal small subunit pseudouridine synthase A	18045073651	30S ribosomal protein S12<br>Ribosomal RNA A-site	Antimicrob. Agents Chemother.,(2009)53:3:1019<br>Bioorg. Med. Chem. Lett.,(2001)11:2:119; Bioorg. Med. Chem. Lett.,(2004)14:17:4389; Bioorg. Med. Chem. Lett.,(2004)14:3:695; Bioorg. Med. Chem. Lett.,(2005)15:22:4919; J. Med. Chem.,(2006)49:18:5478; J. Med. Chem.,(2009)52:9:2836
Gentamicin A_3	Protein 30S ribosomal subunit inhibitor	0.535	0.000		Unknown			*Streptomyces coelicolor (strain ATCC BAA-471 / A3(2) / M145)*	30S ribosomal protein S12		Unknown		Unknown		Unknown		Unknown			*Escherichia coli (strain K12)*	Ribosomal RNA small subunit methyltransferase F<br>Ribosomal small subunit pseudouridine synthase A	18045073722	Unknown	
Gentamicin A_4	Protein 30S ribosomal subunit inhibitor	0.508	0.000		Unknown				Unknown		Unknown		Unknown		Unknown		Unknown			*Escherichia coli (strain K12)*	Ribosomal small subunit pseudouridine synthase A	18045073815	Unknown	
Gentamicin B_1	Protein 30S ribosomal subunit inhibitor	0.879	0.000		Unknown				Unknown		Unknown		Unknown		Unknown		Unknown			*Escherichia coli (strain K12)*	Ribosomal small subunit pseudouridine synthase A	18045073847	Unknown	
Gentamicin C_1	Protein 30S ribosomal subunit inhibitor	0.960	0.000		Unknown				Unknown		Unknown		Unknown		Unknown		Unknown			*Escherichia coli (strain K12)*	Ribosomal RNA small subunit methyltransferase F	18045073927	Unknown	
Gentamicin C_{1a}	Protein 30S ribosomal subunit inhibitor	0.926	0.000		Unknown				Unknown		Unknown		Unknown		Unknown		Unknown				Unknown	18045073954	Unknown	
Gentamicin C_2	Protein 30S ribosomal subunit inhibitor	0.964	0.000		Unknown				Unknown		Unknown		Unknown		Unknown		Unknown				Unknown	180422222842	Unknown	
Gentamicin C_{2a}	Protein 30S ribosomal subunit inhibitor	0.964	0.000		Unknown				Unknown		Unknown		Unknown		Unknown		Unknown			*Escherichia coli (strain K12)*	Ribosomal small subunit pseudouridine synthase A	180422222937	Unknown	
Gentamicin X_2	Protein 30S ribosomal subunit inhibitor	0.877	0.000		Unknown				Unknown		Unknown		Unknown		Unknown		Unknown			*Escherichia coli (strain K12)*<br>*Escherichia coli (strain K12)*	Ribosomal RNA large subunit methyltransferase H<br>Ribosomal small subunit pseudouridine synthase A	180422223029	Unknown	
Hybrimycin A_1	Protein 30S ribosomal subunit inhibitor	0.673	0.000		Unknown				Unknown		Unknown		Unknown		Unknown		Unknown			*Escherichia coli (strain K12)*	Ribosomal RNA small subunit methyltransferase F	18045105358	30S ribosomal protein S12<br>Ribosomal RNA A-site	Antimicrob. Agents Chemother.,(2009)53:3:1019<br>Bioorg. Med. Chem. Lett.,(2001)11:2:119; Bioorg. Med. Chem. Lett.,(2004)14:17:4389; Bioorg. Med. Chem. Lett.,(2005)15:22:4919; J. Med. Chem.,(2006)49:18:5478; J. Med. Chem.,(2009)52:9:2836
Hybrimycin A_2	Protein 30S ribosomal subunit inhibitor	0.673	0.000		Unknown				Unknown		Unknown		Unknown		Unknown		Unknown			*Escherichia coli (strain K12)*	Ribosomal RNA small subunit methyltransferase F	18045105431	30S ribosomal protein S12<br>Ribosomal RNA A-site	Antimicrob. Agents Chemother.,(2009)53:3:1019<br>Bioorg. Med. Chem. Lett.,(2001)11:2:119; Bioorg. Med. Chem. Lett.,(2004)14:17:4389; Bioorg. Med. Chem. Lett.,(2005)15:22:4919; J. Med. Chem.,(2006)49:18:5478; J. Med. Chem.,(2009)52:9:2836
Hybrimycin B_1	Protein 30S ribosomal subunit inhibitor	0.673	0.000		Unknown				Unknown		Unknown		Unknown		Unknown		Unknown			*Escherichia coli (strain K12)*	Ribosomal small subunit pseudouridine synthase A	18045105503	30S ribosomal protein S12<br>Ribosomal RNA A-site	Antimicrob. Agents Chemother.,(2009)53:3:1019<br>Bioorg. Med. Chem. Lett.,(2001)11:2:119; Bioorg. Med. Chem. Lett.,(2004)14:17:4389; Bioorg. Med. Chem. Lett.,(2005)15:22:4919; J. Med. Chem.,(2006)49:18:5478; J. Med. Chem.,(2009)52:9:2836
Hybrimycin B_2	Protein 30S ribosomal subunit inhibitor	0.673	0.000		Unknown				Unknown		Unknown		Unknown		Unknown		Unknown			*Escherichia coli (strain K12)*	Ribosomal RNA small subunit methyltransferase F	18045105528	30S ribosomal protein S12<br>Ribosomal RNA A-site	Antimicrob. Agents Chemother.,(2009)53:3:1019<br>Bioorg. Med. Chem. Lett.,(2001)11:2:119; Bioorg. Med. Chem. Lett.,(2004)14:17:4389; Bioorg. Med. Chem. Lett.,(2005)15:22:4919; J. Med. Chem.,(2006)49:18:5478; J. Med. Chem.,(2009)52:9:2836
Hybrimycin C_1	Protein 30S ribosomal subunit inhibitor	0.673	0.000		Unknown				Unknown		Unknown		Unknown		Unknown		Unknown			*Escherichia coli (strain K12)*	Ribosomal small subunit pseudouridine synthase A	18045105559	30S ribosomal protein S12<br>Ribosomal RNA A-site	Antimicrob. Agents Chemother.,(2009)53:3:1019<br>Bioorg. Med. Chem. Lett.,(2001)11:2:119; Bioorg. Med. Chem. Lett.,(2004)14:17:4389; Bioorg. Med. Chem. Lett.,(2005)15:22:4919; J. Med. Chem.,(2006)49:18:5478; J. Med. Chem.,(2009)52:9:2836
Hybrimycin C_2	Protein 30S ribosomal subunit inhibitor	0.673	0.000		Unknown				Unknown		Unknown		Unknown		Unknown		Unknown			*Escherichia coli (strain K12)*	Ribosomal small subunit pseudouridine synthase A	18045105644	30S ribosomal protein S12<br>Ribosomal RNA A-site	Antimicrob. Agents Chemother.,(2009)53:3:1019<br>Bioorg. Med. Chem. Lett.,(2001)11:2:119; Bioorg. Med. Chem. Lett.,(2004)14:17:4389; Bioorg. Med. Chem. Lett.,(2005)15:22:4919; J. Med. Chem.,(2006)49:18:5478; J. Med. Chem.,(2009)52:9:2836
Hybrimycin D	Protein 30S ribosomal subunit inhibitor	0.571	0.000		Unknown				Unknown		Unknown		Unknown		Unknown		Unknown			*Escherichia coli (strain K12)*	Ribosomal small subunit pseudouridine synthase A	18045105723	30S ribosomal protein S12<br>Ribosomal RNA A-site	Antimicrob. Agents Chemother.,(2009)53:3:1019<br>Bioorg. Med. Chem. Lett.,(2001)11:2:119; Bioorg. Med. Chem. Lett.,(2004)14:17:4389; Bioorg. Med. Chem. Lett.,(2005)15:22:4919; J. Med. Chem.,(2006)49:18:5478; J. Med. Chem.,(2009)52:9:2836
Hygromycin B (A 396II)	Protein 30S ribosomal subunit inhibitor	0.363	0.001		Unknown				Unknown		Unknown		Unknown		Unknown		Unknown			*Escherichia coli (strain K12)*<br>*Methanococcus maripaludis (strain S2 / LL)*	Ribosomal small subunit pseudouridine synthase A<br>30S ribosomal protein S24e	18046155244	Unknown	

Bioactive molecule	PASS online[4]			Similarity Ensemble Approach (SEA)[2]				ChemProt[3]		SuperPred[6]		Polypharmacology Browser (PPB)[1]			SPiDER[7]		HitPick[5]			PharmMapper[9]			TargetHunter[8]	
	Activity	Pa	Pi	Organism	Target	P-Value	Max TC	Organism	Target	Organism	Target	Organism	Target	P-Value	Target	Confidence level	Target	Precision (%)	Tc similarity	Organism	Target	Job Id	Target	Reference
Isepamicin	Protein 30S ribosomal subunit inhibitor	0.714	0.000		Unknown				Unknown		Unknown		Unknown		Unknown		Unknown			*Escherichia coli (strain K12)*	Ribosomal small subunit pseudouridine synthase A	18045105816	30S ribosomal protein S12<br>Ribosomal RNA A-site	Antimicrob. Agents Chemother.,(2009)53:3:1019<br>Bioorg. Med. Chem. Lett.,(2001)11:2:119; Bioorg. Med. Chem. Lett.,(2004)14:17:4389; Bioorg. Med. Chem. Lett.,(2005)15:22:4919; J. Med. Chem.,(2006)49:18:5478; J. Med. Chem.,(2009)52:9:2836
Istamycin A (sannamycin A)	Protein 30S ribosomal subunit inhibitor	0.561	0.000		Unknown				Unknown		Unknown		Unknown		Unknown		Unknown			*Escherichia coli (strain K12)*<br>*Haemophilus influenzae (strain ATCC 51907 / DSM 11121 / KW20 / Rd)*	Ribosomal RNA small subunit methyltransferase F<br>Ribosomal RNA small subunit methyltransferase D	18045222458	30S ribosomal protein S12<br>Ribosomal RNA A-site	Antimicrob. Agents Chemother.,(2009)53:3:1019<br>Bioorg. Med. Chem. Lett.,(2001)11:2:119; Bioorg. Med. Chem. Lett.,(2004)14:17:4389; Bioorg. Med. Chem. Lett.,(2005)15:22:4919; J. Med. Chem.,(2006)49:18:5478; J. Med. Chem.,(2009)52:9:2836
Istamycin A_0 (sannamycin B)	Protein 30S ribosomal subunit inhibitor	0.671	0.000		Unknown				Unknown		Unknown		Unknown		Unknown		Unknown			*Escherichia coli (strain K12)*	Ribosomal RNA small subunit methyltransferase F	18045222530	30S ribosomal protein S12<br>Ribosomal RNA A-site	Antimicrob. Agents Chemother.,(2009)53:3:1019<br>Bioorg. Med. Chem. Lett.,(2001)11:2:119; Bioorg. Med. Chem. Lett.,(2004)14:17:4389; Bioorg. Med. Chem. Lett.,(2004)14:3:695; Bioorg. Med. Chem. Lett.,(2005)15:22:4919; J. Med. Chem.,(2006)49:18:5478; J. Med. Chem.,(2009)52:9:2836
Istamycin A_1	Protein 30S ribosomal subunit inhibitor	0.507	0.000		Unknown				Unknown		Unknown		Unknown		Unknown		Unknown				Unknown	18045222556	Ribosomal RNA A-site	Bioorg. Med. Chem. Lett.,(2001)11:2:119; J. Med. Chem.,(2006)49:18:5478; J. Med. Chem.,(2009)52:9:2836
Istamycin A_2	Protein 30S ribosomal subunit inhibitor	0.487	0.001		Unknown				Unknown		Unknown		Unknown		Unknown		Unknown			*Escherichia coli (strain K12)*	Ribosomal RNA small subunit methyltransferase F	18045222629	Ribosomal RNA A-site	Bioorg. Med. Chem. Lett.,(2001)11:2:119; J. Med. Chem.,(2006)49:18:5478; J. Med. Chem.,(2009)52:9:2836
Istamycin A_3	Protein 30S ribosomal subunit inhibitor	0.645	0.000		Unknown				Unknown		Unknown		Unknown		Unknown		Unknown			*Escherichia coli (strain K12)*	Ribosomal RNA small subunit methyltransferase F	18045222700	Ribosomal RNA A-site	Bioorg. Med. Chem. Lett.,(2001)11:2:119; J. Med. Chem.,(2006)49:18:5478; J. Med. Chem.,(2009)52:9:2836
Istamycin AO	Protein 30S ribosomal subunit inhibitor	0.595	0.000		Unknown				Unknown		Unknown		Unknown		Unknown		Unknown			*Methanococcus maripaludis (strain S2 / LL)*	30S ribosomal protein S24e	18045222731	Unknown	
Istamycin AP (sannamycin E)	Protein 30S ribosomal subunit inhibitor	0.748	0.000		Unknown				Unknown		Unknown		Unknown		Unknown		Unknown			*Escherichia coli (strain K12)*	Ribosomal RNA small subunit methyltransferase F	18045222813	30S ribosomal protein S12<br>Ribosomal RNA A-site	Antimicrob. Agents Chemother.,(2009)53:3:1019<br>Bioorg. Med. Chem. Lett.,(2001)11:2:119; Bioorg. Med. Chem. Lett.,(2004)14:17:4389; Bioorg. Med. Chem. Lett.,(2004)14:3:695; Bioorg. Med. Chem. Lett.,(2005)15:22:4919; J. Med. Chem.,(2006)49:18:5478; J. Med. Chem.,(2009)52:9:2836
Istamycin B	Protein 30S ribosomal subunit inhibitor	0.561	0.000		Unknown				Unknown		Unknown		Unknown		Unknown		Unknown			*Escherichia coli (strain K12)*	Ribosomal RNA small subunit methyltransferase F	18045222840	30S ribosomal protein S12<br>Ribosomal RNA A-site	Antimicrob. Agents Chemother.,(2009)53:3:1019<br>Bioorg. Med. Chem. Lett.,(2001)11:2:119; Bioorg. Med. Chem. Lett.,(2004)14:17:4389; Bioorg. Med. Chem. Lett.,(2005)15:22:4919; J. Med. Chem.,(2006)49:18:5478; J. Med. Chem.,(2009)52:9:2836
Istamycin B_0	Protein 30S ribosomal subunit inhibitor	0.671	0.000		Unknown				Unknown		Unknown		Unknown		Unknown		Unknown			*Escherichia coli (strain K12)*	Ribosomal RNA small subunit methyltransferase F	18045222932	30S ribosomal protein S12<br>Ribosomal RNA A-site	Antimicrob. Agents Chemother.,(2009)53:3:1019<br>Bioorg. Med. Chem. Lett.,(2001)11:2:119; Bioorg. Med. Chem. Lett.,(2004)14:17:4389; Bioorg. Med. Chem. Lett.,(2004)14:3:695; Bioorg. Med. Chem. Lett.,(2005)15:22:4919; J. Med. Chem.,(2006)49:18:5478; J. Med. Chem.,(2009)52:9:2836
Istamycin B_1	Protein 30S ribosomal subunit inhibitor	0.507	0.000		Unknown				Unknown		Unknown		Unknown		Unknown		Unknown			*Bacillus subtilis (strain 168)*<br>*Escherichia coli (strain K12)*	Ribosomal RNA small subunit methyltransferase E<br>Ribosomal RNA small subunit methyltransferase F	18046130821	Ribosomal RNA A-site	Bioorg. Med. Chem. Lett.,(2001)11:2:119; J. Med. Chem.,(2006)49:18:5478; J. Med. Chem.,(2009)52:9:2836
Istamycin B_3	Protein 30S ribosomal subunit inhibitor	0.645	0.000		Unknown				Unknown		Unknown		Unknown		Unknown		Unknown			*Escherichia coli (strain K12)*	Ribosomal RNA small subunit methyltransferase F	18046130855	Ribosomal RNA A-site	Bioorg. Med. Chem. Lett.,(2001)11:2:119; J. Med. Chem.,(2006)49:18:5478; J. Med. Chem.,(2009)52:9:2836
Istamycin C	Protein 30S ribosomal subunit inhibitor	0.503	0.001		Unknown				Unknown		Unknown		Unknown		Unknown		Unknown			*Escherichia coli (strain K12)*	Ribosomal RNA small subunit methyltransferase F	18046130921	30S ribosomal protein S12<br>Ribosomal RNA A-site	Antimicrob. Agents Chemother.,(2009)53:3:1019<br>Bioorg. Med. Chem. Lett.,(2001)11:2:119; Bioorg. Med. Chem. Lett.,(2004)14:17:4389; Bioorg. Med. Chem. Lett.,(2005)15:22:4919; J. Med. Chem.,(2006)49:18:5478; J. Med. Chem.,(2009)52:9:2836
Istamycin C_0	Protein 30S ribosomal subunit inhibitor	0.570	0.000		Unknown				Unknown		Unknown		Unknown		Unknown		Unknown			*Escherichia coli (strain K12)*	Ribosomal RNA small subunit methyltransferase F	18046131013	30S ribosomal protein S12<br>Ribosomal RNA A-site	Antimicrob. Agents Chemother.,(2009)53:3:1019<br>Bioorg. Med. Chem. Lett.,(2001)11:2:119; Bioorg. Med. Chem. Lett.,(2004)14:17:4389; Bioorg. Med. Chem. Lett.,(2005)15:22:4919; J. Med. Chem.,(2006)49:18:5478; J. Med. Chem.,(2009)52:9:2836
Istamycin C_1	Protein 30S ribosomal subunit inhibitor	0.466	0.001		Unknown				Unknown		Unknown		Unknown		Unknown		Unknown			*Haemophilus influenzae (strain ATCC 51907 / DSM 11121 / KW20 / Rd)*	Ribosomal RNA small subunit methyltransferase D	18046131059	Ribosomal RNA A-site	Bioorg. Med. Chem. Lett.,(2001)11:2:119; J. Med. Chem.,(2006)49:18:5478; J. Med. Chem.,(2009)52:9:2836
Istamycin KL_1	Protein 30S ribosomal subunit inhibitor	0.670	0.000		Unknown				Unknown		Unknown		Unknown		Unknown		Unknown				Unknown	18046131125	Unknown	
Istamycin X_0 (sannamycin G)	Protein 30S ribosomal subunit inhibitor	0.698	0.000		Unknown				Unknown		Unknown		Unknown		Unknown		Unknown			*Escherichia coli (strain K12)*	Ribosomal RNA small subunit methyltransferase F	18046131152	Unknown	
Istamycin Y_0 (sannamycin H)	Protein 30S ribosomal subunit inhibitor	0.698	0.000		Unknown				Unknown		Unknown		Unknown		Unknown		Unknown			*Escherichia coli (strain K12)*	Ribosomal RNA small subunit methyltransferase F	18046131230	Unknown	
Kanamycin (Kanamycin A)	Protein 30S ribosomal subunit inhibitor	0.560	0.000		Unknown			*Streptomyces coelicolor (strain ATCC BAA-471 / A3(2) / M145)*	30S ribosomal protein S12		Unknown		Unknown		Unknown		Unknown			*Escherichia coli (strain K12)*	Ribosomal RNA small subunit methyltransferase F<br>Ribosomal small subunit pseudouridine synthase A	180330124148	30S ribosomal protein S12<br>Ribosomal RNA A-site	Antimicrob. Agents Chemother.,(2009)53:3:1019<br>Bioorg. Med. Chem. Lett.,(2001)11:2:119; Bioorg. Med. Chem. Lett.,(2004)14:17:4389; Bioorg. Med. Chem. Lett.,(2005)15:22:4919; J. Med. Chem.,(2006)49:18:5478; J. Med. Chem.,(2009)52:9:2836
Kanamycin C	Protein 30S ribosomal subunit inhibitor	0.737	0.000		Unknown			*Streptomyces coelicolor (strain ATCC BAA-471 / A3(2) / M145)*	30S ribosomal protein S12		Unknown		Unknown		Unknown		Unknown				Unknown	180330123958	30S ribosomal protein S12<br>Ribosomal RNA A-site	Antimicrob. Agents Chemother.,(2009)53:3:1019<br>Bioorg. Med. Chem. Lett.,(2001)11:2:119; Bioorg. Med. Chem. Lett.,(2004)14:17:4389; Bioorg. Med. Chem. Lett.,(2005)15:22:4919; J. Med. Chem.,(2006)49:18:5478; J. Med. Chem.,(2009)52:9:2836
Kanamycin D	Protein 30S ribosomal subunit inhibitor	0.475	0.001		Unknown			*Streptomyces coelicolor (strain ATCC BAA-471 / A3(2) / M145)*	30S ribosomal protein S12		Unknown		Unknown		Unknown		Unknown				Unknown	180330124038	30S ribosomal protein S12<br>Ribosomal RNA A-site	Antimicrob. Agents Chemother.,(2009)53:3:1019<br>Bioorg. Med. Chem. Lett.,(2001)11:2:119; Bioorg. Med. Chem. Lett.,(2004)14:17:4389; Bioorg. Med. Chem. Lett.,(2004)14:3:695; Bioorg. Med. Chem. Lett.,(2005)15:22:4919; J. Med. Chem.,(2006)49:18:5478; J. Med. Chem.,(2009)52:9:2836
Kanamycin X	Protein 30S ribosomal subunit inhibitor	0.567	0.000		Unknown			*Streptomyces coelicolor (strain ATCC BAA-471 / A3(2) / M145)*	30S ribosomal protein S12		Unknown		Unknown		Unknown		Unknown				Unknown	180330124110	30S ribosomal protein S12<br>Ribosomal RNA A-site	Antimicrob. Agents Chemother.,(2009)53:3:1019<br>Bioorg. Med. Chem. Lett.,(2001)11:2:119; Bioorg. Med. Chem. Lett.,(2004)14:17:4389; Bioorg. Med. Chem. Lett.,(2005)15:22:4919; J. Med. Chem.,(2006)49:18:5478; J. Med. Chem.,(2009)52:9:2836
Lividamine (nebramycin IX)	Protein 30S ribosomal subunit inhibitor	0.785	0.000		Unknown				Unknown		Unknown		Unknown		Unknown		Unknown				Unknown	18045154312	30S ribosomal protein S12<br>Ribosomal RNA A-site	Antimicrob. Agents Chemother.,(2009)53:3:1019<br>Bioorg. Med. Chem. Lett.,(2001)11:2:119; Bioorg. Med. Chem. Lett.,(2004)14:17:4389; Bioorg. Med. Chem. Lett.,(2004)14:3:695; Bioorg. Med. Chem. Lett.,(2005)15:22:4919; J. Med. Chem.,(2006)49:18:5478; J. Med. Chem.,(2009)52:9:2836
Lividomycin	Protein 30S ribosomal subunit inhibitor	0.751	0.000		Unknown				Unknown		Unknown		Unknown		Unknown		Unknown				Unknown	18045110018	30S ribosomal protein S12<br>Ribosomal RNA A-site	Antimicrob. Agents Chemother.,(2009)53:3:1019<br>Bioorg. Med. Chem. Lett.,(2001)11:2:119; Bioorg. Med. Chem. Lett.,(2004)14:17:4389; Bioorg. Med. Chem. Lett.,(2005)15:22:4919; J. Med. Chem.,(2006)49:18:5478; J. Med. Chem.,(2009)52:9:2836
Lividomycin B (3'-deoxyparomomycin I)	Protein 30S ribosomal subunit inhibitor	0.755	0.000		Unknown				Unknown		Unknown		Unknown		Unknown		Unknown				Unknown	18045105923	30S ribosomal protein S12<br>Ribosomal RNA A-site	Antimicrob. Agents Chemother.,(2009)53:3:1019<br>Bioorg. Med. Chem. Lett.,(2001)11:2:119; Bioorg. Med. Chem. Lett.,(2004)14:17:4389; Bioorg. Med. Chem. Lett.,(2005)15:22:4919; J. Med. Chem.,(2006)49:18:5478; J. Med. Chem.,(2009)52:9:2836
Mannosylparomomycin	Protein 30S ribosomal subunit inhibitor	0.606	0.000		Unknown			*Streptomyces coelicolor (strain ATCC BAA-471 / A3(2) / M145)*	30S ribosomal protein S12		Unknown		Unknown		Unknown		Unknown			*Escherichia coli (strain K12)*	Ribosomal small subunit pseudouridine synthase A	18045143808	30S ribosomal protein S12<br>Ribosomal RNA A-site	Antimicrob. Agents Chemother.,(2009)53:3:1019<br>Bioorg. Med. Chem. Lett.,(2001)11:2:119; Bioorg. Med. Chem. Lett.,(2004)14:17:4389; Bioorg. Med. Chem. Lett.,(2005)15:22:4919; J. Med. Chem.,(2006)49:18:5478; J. Med. Chem.,(2009)52:9:2836
Micronomicin (gentamicin C_{2b})	Protein 30S ribosomal subunit inhibitor	0.909	0.000		Unknown				Unknown		Unknown		Unknown		Unknown		Unknown			*Thermus thermophilus (strain HB8 / ATCC 27634 / DSM 579)*	Ribosomal RNA small subunit methyltransferase G	180422223208	Unknown	
Neamine (neomycin A; nebramycin X)	Protein 30S ribosomal subunit inhibitor	0.719	0.000		Unknown			*Streptomyces coelicolor (strain ATCC BAA-471 / A3(2) / M145)*	30S ribosomal protein S12		Unknown		Unknown		Unknown		Unknown				Unknown	18045143918	30S ribosomal protein S12<br>Ribosomal RNA A-site	Antimicrob. Agents Chemother.,(2009)53:3:1019<br>Bioorg. Med. Chem. Lett.,(2001)11:2:119; Bioorg. Med. Chem. Lett.,(2004)14:17:4389; Bioorg. Med. Chem. Lett.,(2004)14:3:695; Bioorg. Med. Chem. Lett.,(2005)15:22:4919; J. Med. Chem.,(2006)49:18:5478; J. Med. Chem.,(2009)52:9:2836
Nebramine (nebramycin VIII)	Protein 30S ribosomal subunit inhibitor	0.860	0.000		Unknown				Unknown		Unknown		Unknown		Unknown		Unknown				Unknown	18045154434	30S ribosomal protein S12<br>Ribosomal RNA A-site	Antimicrob. Agents Chemother.,(2009)53:3:1019<br>Bioorg. Med. Chem. Lett.,(2001)11:2:119; Bioorg. Med. Chem. Lett.,(2004)14:17:4389; Bioorg. Med. Chem. Lett.,(2004)14:3:695; Bioorg. Med. Chem. Lett.,(2005)15:22:4919; J. Med. Chem.,(2006)49:18:5478; J. Med. Chem.,(2009)52:9:2836
Nebramycin III	Protein 30S ribosomal subunit inhibitor	0.614	0.000		Unknown			*Streptomyces coelicolor (strain ATCC BAA-471 / A3(2) / M145)*	30S ribosomal protein S12		Unknown		Unknown		Unknown		Unknown			*Escherichia coli (strain K12)*	Ribosomal small subunit pseudouridine synthase A	18045143954	30S ribosomal protein S12<br>Ribosomal RNA A-site	Antimicrob. Agents Chemother.,(2009)53:3:1019<br>Bioorg. Med. Chem. Lett.,(2001)11:2:119; Bioorg. Med. Chem. Lett.,(2004)14:17:4389; Bioorg. Med. Chem. Lett.,(2004)14:3:695; Bioorg. Med. Chem. Lett.,(2005)15:22:4919; J. Med. Chem.,(2006)49:18:5478; J. Med. Chem.,(2009)52:9:2836
Nebramycin IV	Protein 30S ribosomal subunit inhibitor	0.612	0.000		Unknown				Unknown		Unknown		Unknown		Unknown		Unknown			*Methanococcus maripaludis (strain S2 / LL)*	30S ribosomal protein S24e	18045154537	30S ribosomal protein S12<br>Ribosomal RNA A-site	Antimicrob. Agents Chemother.,(2009)53:3:1019<br>Bioorg. Med. Chem. Lett.,(2001)11:2:119; Bioorg. Med. Chem. Lett.,(2004)14:17:4389; Bioorg. Med. Chem. Lett.,(2005)15:22:4919; J. Med. Chem.,(2006)49:18:5478; J. Med. Chem.,(2009)52:9:2836
Nebramycin V'	Protein 30S ribosomal subunit inhibitor	0.700	0.000		Unknown				Unknown		Unknown		Unknown		Unknown		Unknown				Unknown	180422223255; 180428200536	30S ribosomal protein S12<br>Ribosomal RNA A-site	Antimicrob. Agents Chemother.,(2009)53:3:1019<br>Bioorg. Med. Chem. Lett.,(2001)11:2:119; Bioorg. Med. Chem. Lett.,(2004)14:17:4389; Bioorg. Med. Chem. Lett.,(2005)15:22:4919; J. Med. Chem.,(2006)49:18:5478; J. Med. Chem.,(2009)52:9:2836
Nebramycin XI	Protein 30S ribosomal subunit inhibitor	0.530	0.000		Unknown				Unknown		Unknown		Unknown		Unknown		Unknown				Unknown	18045154650	30S ribosomal protein S12<br>Ribosomal RNA A-site	Antimicrob. Agents Chemother.,(2009)53:3:1019<br>Bioorg. Med. Chem. Lett.,(2001)11:2:119; Bioorg. Med. Chem. Lett.,(2004)14:17:4389; Bioorg. Med. Chem. Lett.,(2005)15:22:4919; J. Med. Chem.,(2006)49:18:5478; J. Med. Chem.,(2009)52:9:2836
Nebramycin XII	Protein 30S ribosomal subunit inhibitor	0.701	0.000		Unknown			*Streptomyces coelicolor (strain ATCC BAA-471 / A3(2) / M145)*	30S ribosomal protein S12		Unknown		Unknown		Unknown		Unknown			*Escherichia coli (strain K12)*<br>*Methanococcus maripaludis (strain S2 / LL)*	Ribosomal small subunit pseudouridine synthase A<br>30S ribosomal protein S24e	18045154717	30S ribosomal protein S12<br>Ribosomal RNA A-site	Antimicrob. Agents Chemother.,(2009)53:3:1019<br>Bioorg. Med. Chem. Lett.,(2001)11:2:119; Bioorg. Med. Chem. Lett.,(2004)14:17:4389; Bioorg. Med. Chem. Lett.,(2004)14:3:695; Bioorg. Med. Chem. Lett.,(2005)15:22:4919; J. Med. Chem.,(2006)49:18:5478; J. Med. Chem.,(2009)52:9:2836
Nebramycin XIII	Protein 30S ribosomal subunit inhibitor	0.625	0.000		Unknown				Unknown		Unknown		Unknown		Unknown		Unknown			*Escherichia coli (strain K12)*	Ribosomal small subunit pseudouridine synthase A	18045154801	30S ribosomal protein S12<br>Ribosomal RNA A-site	Antimicrob. Agents Chemother.,(2009)53:3:1019<br>Bioorg. Med. Chem. Lett.,(2001)11:2:119; Bioorg. Med. Chem. Lett.,(2004)14:17:4389; Bioorg. Med. Chem. Lett.,(2005)15:22:4919; J. Med. Chem.,(2006)49:18:5478; J. Med. Chem.,(2009)52:9:2836
Neomycin C	Protein 30S ribosomal subunit inhibitor	0.606	0.000		Unknown			*Streptomyces coelicolor (strain ATCC BAA-471 / A3(2) / M145)*	30S ribosomal protein S12		Unknown		Unknown		Unknown		Unknown			*Escherichia coli (strain K12)*	Ribosomal RNA small subunit methyltransferase F<br>Ribosomal small subunit pseudouridine synthase A	18045144020	30S ribosomal protein S12<br>Ribosomal RNA A-site	Antimicrob. Agents Chemother.,(2009)53:3:1019<br>Bioorg. Med. Chem. Lett.,(2001)11:2:119; Bioorg. Med. Chem. Lett.,(2004)14:17:4389; Bioorg. Med. Chem. Lett.,(2005)15:22:4919; J. Med. Chem.,(2006)49:18:5478; J. Med. Chem.,(2009)52:9:2836
Neomycin F (paromomycin II)	Protein 30S ribosomal subunit inhibitor	0.606	0.000		Unknown				Unknown		Unknown		Unknown		Unknown		Unknown			*Escherichia coli (strain K12)*	Ribosomal RNA small subunit methyltransferase F<br>Ribosomal small subunit pseudouridine synthase A	18045144117	30S ribosomal protein S12<br>Ribosomal RNA A-site	Antimicrob. Agents Chemother.,(2009)53:3:1019<br>Bioorg. Med. Chem. Lett.,(2001)11:2:119; Bioorg. Med. Chem. Lett.,(2004)14:17:4389; Bioorg. Med. Chem. Lett.,(2005)15:22:4919; J. Med. Chem.,(2006)49:18:5478; J. Med. Chem.,(2009)52:9:2836
Netilmicin	Protein 30S ribosomal subunit inhibitor	0.913	0.000		Unknown				Unknown		Unknown		Unknown		Unknown		Unknown			*Escherichia coli (strain K12)*	Ribosomal RNA small subunit methyltransferase F	18045154833	Ribosomal RNA A-site	Bioorg. Med. Chem. Lett.,(2001)11:2:119; J. Med. Chem.,(2006)49:18:5478
NK 1001	Protein 30S ribosomal subunit inhibitor	0.475	0.001		Unknown			*Streptomyces coelicolor (strain ATCC BAA-471 / A3(2) / M145)*	30S ribosomal protein S12		Unknown		Unknown		Unknown		Unknown			*Escherichia coli (strain K12)*	Ribosomal small subunit pseudouridine synthase A	18045171648	30S ribosomal protein S12<br>Ribosomal RNA A-site	Antimicrob. Agents Chemother.,(2009)53:3:1019<br>Bioorg. Med. Chem. Lett.,(2001)11:2:119; Bioorg. Med. Chem. Lett.,(2004)14:17:4389; Bioorg. Med. Chem. Lett.,(2004)14:3:695; Bioorg. Med. Chem. Lett.,(2005)15:22:4919; J. Med. Chem.,(2006)49:18:5478; J. Med. Chem.,(2009)52:9:2836
Oxyapramycin (nebramycin VII)	Protein 30S ribosomal subunit inhibitor	0.710	0.000		Unknown				Unknown		Unknown		Unknown		Unknown		Unknown			*Escherichia coli (strain K12)*	Ribosomal small subunit pseudouridine synthase A	18045154902	Unknown	
Paromamine (neomycin D)	Protein 30S ribosomal subunit inhibitor	0.633	0.000		Unknown				Unknown		Unknown		Unknown		Unknown		Unknown			*Escherichia coli (strain K12)*	Ribosomal small subunit pseudouridine synthase A	18045144208	30S ribosomal protein S12<br>Ribosomal RNA A-site	Antimicrob. Agents Chemother.,(2009)53:3:1019<br>Bioorg. Med. Chem. Lett.,(2001)11:2:119; Bioorg. Med. Chem. Lett.,(2004)14:17:4389; Bioorg. Med. Chem. Lett.,(2005)15:22:4919; J. Med. Chem.,(2006)49:18:5478; J. Med. Chem.,(2009)52:9:2836
Paromomycin (paromomycin I; neomycin E)	Protein 30S ribosomal subunit inhibitor	0.606	0.000		Unknown			*Streptomyces coelicolor (strain ATCC BAA-471 / A3(2) / M145)*	30S ribosomal protein S12		Unknown		Unknown		Unknown		Unknown			*Escherichia coli (strain K12)*<br>*Methanosarcina mazei (strain ATCC BAA-159 / DSM 3647 / Goe1 / Go1 / JCM 11833 / OCM 88) (Methanosarcina frisia)*	Ribosomal small subunit pseudouridine synthase A<br>30S ribosomal protein S24e	18045144242	30S ribosomal protein S12<br>Ribosomal RNA A-site	Antimicrob. Agents Chemother.,(2009)53:3:1019<br>Bioorg. Med. Chem. Lett.,(2001)11:2:119; Bioorg. Med. Chem. Lett.,(2004)14:17:4389; Bioorg. Med. Chem. Lett.,(2005)15:22:4919; J. Med. Chem.,(2006)49:18:5478; J. Med. Chem.,(2009)52:9:2836
Pentisomicin	Protein 30S ribosomal subunit inhibitor	0.952	0.000		Unknown				Unknown		Unknown		Unknown		Unknown		Unknown			*Escherichia coli (strain K12)*<br>*Methanosarcina mazei (strain ATCC BAA-159 / DSM 3647 / Goe1 / Go1 / JCM 11833 / OCM 88) (Methanosarcina frisia)*	Ribosomal RNA small subunit methyltransferase F<br>30S ribosomal protein S24e	18045171714	30S ribosomal protein S12<br>Ribosomal RNA A-site	Antimicrob. Agents Chemother.,(2009)53:3:1019<br>Bioorg. Med. Chem. Lett.,(2001)11:2:119; Bioorg. Med. Chem. Lett.,(2004)14:17:4389; Bioorg. Med. Chem. Lett.,(2005)15:22:4919; J. Med. Chem.,(2006)49:18:5478; J. Med. Chem.,(2009)52:9:2836
Plazomicin	Protein 30S ribosomal subunit inhibitor	0.768	0.000		Unknown				Unknown		Unknown		Unknown		Unknown		Unknown			*Bacillus subtilis (strain 168)*<br>*Escherichia coli (strain K12)*	Ribosomal RNA small subunit methyltransferase E<br>Ribosomal small subunit pseudouridine synthase A	18045171742	Ribosomal RNA A-site	Bioorg. Med. Chem. Lett.,(2001)11:2:119; J. Med. Chem.,(2009)52:9:2836
Propikacin	Protein 30S ribosomal subunit inhibitor	0.630	0.000		Unknown				Unknown		Unknown		Unknown		Unknown		Unknown			*Escherichia coli (strain K12)*	Ribosomal RNA small subunit methyltransferase F	18045171821	Unknown	
Pyrankacin	Protein 30S ribosomal subunit inhibitor	0.598	0.000		Unknown				Unknown		Unknown		Unknown		Unknown		Unknown			*Geobacillus stearothermophilus (Bacillus stearothermophilus)*	30S ribosomal protein S16	18045171851	30S ribosomal protein S12<br>Ribosomal RNA A-site	Antimicrob. Agents Chemother.,(2009)53:3:1019<br>Bioorg. Med. Chem. Lett.,(2001)11:2:119; Bioorg. Med. Chem. Lett.,(2004)14:17:4389; Bioorg. Med. Chem. Lett.,(2005)15:22:4919; J. Med. Chem.,(2006)49:18:5478; J. Med. Chem.,(2009)52:9:2836
Ribostamycin	Protein 30S ribosomal subunit inhibitor	0.630	0.000		Unknown			*Streptomyces coelicolor (strain ATCC BAA-471 / A3(2) / M145)*	30S ribosomal protein S12		Unknown		Unknown		Unknown		Unknown				Unknown	18045144309	30S ribosomal protein S12<br>Ribosomal RNA A-site	Antimicrob. Agents Chemother.,(2009)53:3:1019<br>Bioorg. Med. Chem. Lett.,(2001)11:2:119; Bioorg. Med. Chem. Lett.,(2004)14:17:4389; Bioorg. Med. Chem. Lett.,(2005)15:22:4919; J. Med. Chem.,(2006)49:18:5478; J. Med. Chem.,(2009)52:9:2836
Saccharocin (KA 5685)	Protein 30S ribosomal subunit inhibitor	0.781	0.000		Unknown				Unknown		Unknown		Unknown		Unknown		Unknown			*Escherichia coli (strain K12)*	Ribosomal small subunit pseudouridine synthase A	18046155320	Unknown	
Sannamycin C	Protein 30S ribosomal subunit inhibitor	0.671	0.000		Unknown				Unknown		Unknown		Unknown		Unknown		Unknown				Unknown	18046131253	30S ribosomal protein S12<br>Ribosomal RNA A-site	Antimicrob. Agents Chemother.,(2009)53:3:1019<br>Bioorg. Med. Chem. Lett.,(2001)11:2:119; Bioorg. Med. Chem. Lett.,(2004)14:17:4389; Bioorg. Med. Chem. Lett.,(2004)14:3:695; Bioorg. Med. Chem. Lett.,(2005)15:22:4919; J. Med. Chem.,(2006)49:18:5478; J. Med. Chem.,(2009)52:9:2836

Bioactive molecule	PASS online[4]			Similarity Ensemble Approach (SEA)[2]				ChemProt[3]		SuperPred[6]		Polypharmacology Browser (PPB)[1]			SPiDER[7]		HitPick[5]		
	Activity	Pa	Pi	Organism	Target	P-Value	Max TC	Organism	Target	Organism	Target	Organism	Target	P-Value	Target	Confidence level	Target	Precision (%)	Tc similarity
Sannamycin F	Protein 30S ribosomal subunit inhibitor	0.448	0.001		Unknown				Unknown		Unknown		Unknown		Unknown		Unknown		
Sannamycin J	Protein 30S ribosomal subunit inhibitor	0.698	0.000		Unknown				Unknown		Unknown		Unknown		Unknown		Unknown		
Sannamycin K	Protein 30S ribosomal subunit inhibitor	0.840	0.000		Unknown				Unknown		Unknown		Unknown		Unknown		Unknown		
Sannamycin KR	Protein 30S ribosomal subunit inhibitor	0.671	0.000		Unknown				Unknown		Unknown		Unknown		Unknown		Unknown		
Sannamycin L	Protein 30S ribosomal subunit inhibitor	0.827	0.000		Unknown				Unknown		Unknown		Unknown		Unknown		Unknown		
Seldomycin	Protein 30S ribosomal subunit inhibitor	0.532	0.000		Unknown				Unknown		Unknown		Unknown		Unknown		Unknown		
Seldomycin 1 (seldomycin factor 1)	Protein 30S ribosomal subunit inhibitor	0.588	0.000		Unknown			*Streptomyces coelicolor (strain ATCC BAA-471 / A3(2) / M145)*	30S ribosomal protein S12		Unknown		Unknown		Unknown		Unknown		
Seldomycin 2 (seldomycin factor 2)	Protein 30S ribosomal subunit inhibitor	0.694	0.000		Unknown			*Streptomyces coelicolor (strain ATCC BAA-471 / A3(2) / M145)*	30S ribosomal protein S12		Unknown		Unknown		Unknown		Unknown		
Seldomycin 3 (seldomycin factor 3)	Protein 30S ribosomal subunit inhibitor	0.647	0.000		Unknown				Unknown		Unknown		Unknown		Unknown		Unknown		
Seldomycin 5 (seldomycin factor 5)	Protein 30S ribosomal subunit inhibitor	0.532	0.000		Unknown				Unknown		Unknown		Unknown		Unknown		Unknown		
Sisomicin	Protein 30S ribosomal subunit inhibitor	0.952	0.000		Unknown				Unknown		Unknown		Unknown		Unknown		Unknown		
Sisomicin B	Protein 30S ribosomal subunit inhibitor	0.803	0.000		Unknown				Unknown		Unknown		Unknown		Unknown		Unknown		
Sisomicin D	Protein 30S ribosomal subunit inhibitor	0.803	0.000		Unknown				Unknown		Unknown		Unknown		Unknown		Unknown		
Spectinomycin	Protein 30S ribosomal subunit inhibitor	0.965	0.000		Unknown				Unknown		Unknown		Unknown		Unknown		Unknown		
Sporaricin A	Protein 30S ribosomal subunit inhibitor	0.723	0.000		Unknown				Unknown		Unknown		Unknown		Unknown		Unknown		
Sporaricin B	Protein 30S ribosomal subunit inhibitor	0.836	0.000		Unknown				Unknown		Unknown		Unknown		Unknown		Unknown		
Sporaricin C	Protein 30S ribosomal subunit inhibitor	0.597	0.000		Unknown				Unknown		Unknown		Unknown		Unknown		Unknown		
Sporaricin D	Protein 30S ribosomal subunit inhibitor	0.615	0.000		Unknown				Unknown		Unknown		Unknown		Unknown		Unknown		
Sporaricin E	Protein 30S ribosomal subunit inhibitor	0.836	0.000		Unknown				Unknown		Unknown		Unknown		Unknown		Unknown		
SS 56A	Protein 30S ribosomal subunit inhibitor	0.430	0.001		Unknown			*Streptomyces coelicolor (strain ATCC BAA-471 / A3(2) / M145)*	30S ribosomal protein S12		Unknown		Unknown		Unknown		Unknown		
SS 56B	Protein 30S ribosomal subunit inhibitor	0.430	0.001		Unknown			*Streptomyces coelicolor (strain ATCC BAA-471 / A3(2) / M145)*	30S ribosomal protein S12		Unknown		Unknown		Unknown		Unknown		
SS 56C	Protein 30S ribosomal subunit inhibitor	0.369	0.001		Unknown				Unknown		Unknown		Unknown		Unknown		Unknown		
Streptomycin	Protein 30S ribosomal subunit inhibitor	0.408	0.001		Unknown				Unknown		Unknown		Unknown		Unknown		Unknown		
Streptoniazid (streptonicozid)	Protein 30S ribosomal subunit inhibitor	0.347	0.001		Unknown				Unknown		Unknown		Unknown		Unknown		Unknown		
Tobramycin (nebramycin VI)	Protein 30S ribosomal subunit inhibitor	0.804	0.000		Unknown			*Streptomyces coelicolor (strain ATCC BAA-471 / A3(2) / M145)*	30S ribosomal protein S12		Unknown		Unknown		Unknown		Unknown		
Trospectomycin	Protein 30S ribosomal subunit inhibitor	0.929	0.000		Unknown			*Homo sapiens*	40S ribosomal protein S2; 40S ribosomal protein S3; 40S ribosomal protein S5, N-terminally processed; 40S ribosomal protein S14; 40S ribosomal protein S15; 40S ribosomal protein S16; 40S ribosomal protein S20		Unknown		Unknown		Unknown		Unknown		
Verdamicin	Protein 30S ribosomal subunit inhibitor	0.958	0.000		Unknown				Unknown		Unknown		Unknown		Unknown		Unknown		
Verdamicin C_2	Protein 30S ribosomal subunit inhibitor	0.958	0.000		Unknown				Unknown		Unknown		Unknown		Unknown		Unknown		
Vertilmicin	Protein 30S ribosomal subunit inhibitor	0.928	0.000		Unknown				Unknown		Unknown		Unknown		Unknown		Unknown		

Bioactive molecule	PharmMapper[9]			TargetHunter[8]	
	Organism	Target	Job Id	Target	Reference
Sannamycin F	*Escherichia coli (strain K12)*<br>*Escherichia coli (strain K12)*	Ribosomal RNA small subunit methyltransferase F<br>Ribosomal small subunit pseudouridine synthase A	18046131325	Ribosomal RNA A-site	Bioorg. Med. Chem. Lett.,(2001)11:2:119; J. Med. Chem.,(2006)49:18:5478; J. Med. Chem.,(2009)52:9:2836
Sannamycin J		Unknown	18046144320	Unknown	
Sannamycin K	*Escherichia coli (strain K12)*<br>*Methanosarcina mazei (strain ATCC BAA-159 / DSM 3647 / Goe1 / Go1 / JCM 11833 / OCM 88) (Methanosarcina frisia)*	Ribosomal RNA small subunit methyltransferase F<br>Ribosomal small subunit pseudouridine synthase A<br>30S ribosomal protein S24e	18046144354	Unknown	
Sannamycin KR		Unknown	18046144422	30S ribosomal protein S12<br>Ribosomal RNA A-site	Antimicrob. Agents Chemother.,(2009)53:3:1019<br>Bioorg. Med. Chem. Lett.,(2001)11:2:119; Bioorg. Med. Chem. Lett.,(2004)14:17:4389; Bioorg. Med. Chem. Lett.,(2005)15:22:4919; J. Med. Chem.,(2006)49:18:5478; J. Med. Chem.,(2009)52:9:2836
Sannamycin L	*Escherichia coli (strain K12)*<br>*Methanococcus maripaludis (strain S2 / LL)*	Ribosomal small subunit pseudouridine synthase A<br>30S ribosomal protein S24e	18046144501	30S ribosomal protein S12<br>Ribosomal RNA A-site	Antimicrob. Agents Chemother.,(2009)53:3:1019<br>Bioorg. Med. Chem. Lett.,(2001)11:2:119; Bioorg. Med. Chem. Lett.,(2004)14:17:4389; Bioorg. Med. Chem. Lett.,(2004)14:3:695; Bioorg. Med. Chem. Lett.,(2005)15:22:4919; J. Med. Chem.,(2006)49:18:5478; J. Med. Chem.,(2009)52:9:2836
Seldomycin		Unknown	18045172010	30S ribosomal protein S12<br>Ribosomal RNA A-site	Antimicrob. Agents Chemother.,(2009)53:3:1019<br>Bioorg. Med. Chem. Lett.,(2001)11:2:119; Bioorg. Med. Chem. Lett.,(2004)14:17:4389; Bioorg. Med. Chem. Lett.,(2004)14:3:695; Bioorg. Med. Chem. Lett.,(2005)15:22:4919; J. Med. Chem.,(2006)49:18:5478; J. Med. Chem.,(2009)52:9:2836
Seldomycin 1 (seldomycin factor 1)		Unknown	18045171937	30S ribosomal protein S12<br>Ribosomal RNA A-site	Antimicrob. Agents Chemother.,(2009)53:3:1019<br>Bioorg. Med. Chem. Lett.,(2001)11:2:119; Bioorg. Med. Chem. Lett.,(2004)14:17:4389; Bioorg. Med. Chem. Lett.,(2004)14:3:695; Bioorg. Med. Chem. Lett.,(2005)15:22:4919; J. Med. Chem.,(2006)49:18:5478; J. Med. Chem.,(2009)52:9:2836
Seldomycin 2 (seldomycin factor 2)	*Methanococcus maripaludis (strain S2 / LL)*	30S ribosomal protein S24e	18045182701	30S ribosomal protein S12<br>Ribosomal RNA A-site	Antimicrob. Agents Chemother.,(2009)53:3:1019<br>Bioorg. Med. Chem. Lett.,(2001)11:2:119; Bioorg. Med. Chem. Lett.,(2004)14:17:4389; Bioorg. Med. Chem. Lett.,(2004)14:3:695; Bioorg. Med. Chem. Lett.,(2005)15:22:4919; J. Med. Chem.,(2006)49:18:5478; J. Med. Chem.,(2009)52:9:2836
Seldomycin 3 (seldomycin factor 3)	*Methanococcus maripaludis (strain S2 / LL)*	30S ribosomal protein S24e	18045182734	30S ribosomal protein S12<br>Ribosomal RNA A-site	Antimicrob. Agents Chemother.,(2009)53:3:1019<br>Bioorg. Med. Chem. Lett.,(2001)11:2:119; Bioorg. Med. Chem. Lett.,(2004)14:17:4389; Bioorg. Med. Chem. Lett.,(2004)14:3:695; Bioorg. Med. Chem. Lett.,(2005)15:22:4919; J. Med. Chem.,(2006)49:18:5478; J. Med. Chem.,(2009)52:9:2836
Seldomycin 5 (seldomycin factor 5)		Unknown	18045182820	30S ribosomal protein S12<br>Ribosomal RNA A-site	Antimicrob. Agents Chemother.,(2009)53:3:1019<br>Bioorg. Med. Chem. Lett.,(2001)11:2:119; Bioorg. Med. Chem. Lett.,(2004)14:17:4389; Bioorg. Med. Chem. Lett.,(2004)14:3:695; Bioorg. Med. Chem. Lett.,(2005)15:22:4919; J. Med. Chem.,(2006)49:18:5478; J. Med. Chem.,(2009)52:9:2836
Sisomicin	*Escherichia coli (strain K12)*<br>*Methanosarcina mazei (strain ATCC BAA-159 / DSM 3647 / Goe1 / Go1 / JCM 11833 / OCM 88) (Methanosarcina frisia)*	Ribosomal small subunit pseudouridine synthase A<br>30S ribosomal protein S24e	18045172149	30S ribosomal protein S12<br>Ribosomal RNA A-site	Antimicrob. Agents Chemother.,(2009)53:3:1019<br>Bioorg. Med. Chem. Lett.,(2001)11:2:119; Bioorg. Med. Chem. Lett.,(2004)14:17:4389; Bioorg. Med. Chem. Lett.,(2005)15:22:4919; J. Med. Chem.,(2006)49:18:5478; J. Med. Chem.,(2009)52:9:2836
Sisomicin B	*Escherichia coli (strain K12)*<br>*Methanococcus maripaludis (strain S2 / LL)*<br>*Thermus thermophilus (strain HB8 / ATCC 27634 / DSM 579)*	Ribosomal small subunit pseudouridine synthase A<br>30S ribosomal protein S24e<br>Ribosomal RNA small subunit methyltransferase G	18045172045	30S ribosomal protein S12<br>Ribosomal RNA A-site	Antimicrob. Agents Chemother.,(2009)53:3:1019<br>Bioorg. Med. Chem. Lett.,(2001)11:2:119; Bioorg. Med. Chem. Lett.,(2004)14:17:4389; Bioorg. Med. Chem. Lett.,(2005)15:22:4919; J. Med. Chem.,(2006)49:18:5478; J. Med. Chem.,(2009)52:9:2836
Sisomicin D	*Escherichia coli (strain K12)*<br>*Methanosarcina mazei (strain ATCC BAA-159 / DSM 3647 / Goe1 / Go1 / JCM 11833 / OCM 88) (Methanosarcina frisia)*	Ribosomal small subunit pseudouridine synthase A<br>30S ribosomal protein S24e	18045172124	30S ribosomal protein S12<br>Ribosomal RNA A-site	Antimicrob. Agents Chemother.,(2009)53:3:1019<br>Bioorg. Med. Chem. Lett.,(2001)11:2:119; Bioorg. Med. Chem. Lett.,(2004)14:17:4389; Bioorg. Med. Chem. Lett.,(2005)15:22:4919; J. Med. Chem.,(2006)49:18:5478; J. Med. Chem.,(2009)52:9:2836
Spectinomycin	*Escherichia coli (strain K12)*	Ribosomal RNA small subunit methyltransferase F	18045182844	Unknown	
Sporaricin A		Unknown	18046144541	30S ribosomal protein S12<br>Ribosomal RNA A-site	Antimicrob. Agents Chemother.,(2009)53:3:1019<br>Bioorg. Med. Chem. Lett.,(2001)11:2:119; Bioorg. Med. Chem. Lett.,(2004)14:17:4389; Bioorg. Med. Chem. Lett.,(2005)15:22:4919; J. Med. Chem.,(2006)49:18:5478; J. Med. Chem.,(2009)52:9:2836
Sporaricin B		Unknown	18046144610	Unknown	
Sporaricin C	*Escherichia coli (strain K12)*<br>*Haemophilus influenzae (strain ATCC 51907 / DSM 11121 / KW20 / Rd)*	Ribosomal RNA small subunit methyltransferase F<br>Ribosomal RNA small subunit methyltransferase D	18046144715	Ribosomal RNA A-site	Bioorg. Med. Chem. Lett.,(2001)11:2:119; J. Med. Chem.,(2006)49:18:5478; J. Med. Chem.,(2009)52:9:2836
Sporaricin D		Unknown	18046144745	Ribosomal RNA A-site	Bioorg. Med. Chem. Lett.,(2001)11:2:119; J. Med. Chem.,(2006)49:18:5478; J. Med. Chem.,(2009)52:9:2836
Sporaricin E		Unknown	18046144846	Unknown	
SS 56A		Unknown	18046155350	Unknown	
SS 56B		Unknown	18046155425	Unknown	
SS 56C	*Escherichia coli (strain K12)*<br>*Methanococcus maripaludis (strain S2 / LL)*	Ribosomal small subunit pseudouridine synthase A<br>30S ribosomal protein S24e	18046155453	Unknown	
Streptomycin	*Escherichia coli (strain K12)*	Ribosomal RNA small subunit methyltransferase F	180330124233	30S ribosomal protein S12<br>Ribosomal RNA A-site	Antimicrob. Agents Chemother.,(2009)53:3:1019<br>Bioorg. Med. Chem. Lett.,(2001)11:2:119; Bioorg. Med. Chem. Lett.,(2005)15:22:4919; J. Med. Chem.,(2009)52:9:2836; J. Med. Chem.,(2006)49:18:5478
Streptoniazid (streptonicozid)	*Escherichia coli (strain K12)*	Ribosomal RNA small subunit methyltransferase F	18045182924	Unknown	
Tobramycin (nebramycin VI)	*Escherichia coli (strain K12)*	Ribosomal RNA small subunit methyltransferase F	18045154942	30S ribosomal protein S12<br>Ribosomal RNA A-site	Antimicrob. Agents Chemother.,(2009)53:3:1019<br>Bioorg. Med. Chem. Lett.,(2001)11:2:119; Bioorg. Med. Chem. Lett.,(2004)14:17:4389; Bioorg. Med. Chem. Lett.,(2005)15:22:4919; J. Med. Chem.,(2006)49:18:5478; J. Med. Chem.,(2009)52:9:2836
Trospectomycin	*Escherichia coli (strain K12)*	Ribosomal RNA small subunit methyltransferase F	18045183001	Unknown	
Verdamicin	*Escherichia coli (strain K12)*	Ribosomal RNA small subunit methyltransferase F<br>Ribosomal small subunit pseudouridine synthase A	18045183107	Ribosomal RNA A-site	Bioorg. Med. Chem. Lett.,(2001)11:2:119; J. Med. Chem.,(2006)49:18:5478
Verdamicin C_2	*Methanococcus maripaludis (strain S2 / LL)*	30S ribosomal protein S24e	18045183032	Ribosomal RNA A-site	Bioorg. Med. Chem. Lett.,(2001)11:2:119; J. Med. Chem.,(2006)49:18:5478
Vertilmicin	*Escherichia coli (strain K12)*<br>*Methanococcus maripaludis (strain S2 / LL)*	Ribosomal RNA small subunit methyltransferase F<br>30S ribosomal protein S24e	18045183144	Ribosomal RNA A-site	Bioorg. Med. Chem. Lett.,(2001)11:2:119

Bioactive molecule	PASS online[4]			Similarity Ensemble Approach (SEA)[2]				ChemProt[3]		SuperPred[6]		Polypharmacology Browser (PPB)[1]			SPiDER[7]		HitPick[5]			PharmMapper[9]			TargetHunter[8]	
	Activity	Pa	Pi	Organism	Target	P-Value	Max TC	Organism	Target	Organism	Target	Organism	Target	P-Value	Target	Confidence level	Target	Precision (%)	Tc similarity	Organism	Target	Job Id	Target	Reference
7-Iodosancycline	Protein 30S ribosomal subunit inhibitor	0.333	0.001		Unknown				Unknown		Unknown		Unknown		Unknown		Unknown			*Escherichia coli (strain K12)*	Ribosomal RNA small subunit methyltransferase F	18046181546	Unknown	
Amicycline	Protein 30S ribosomal subunit inhibitor	0.340	0.001		Unknown				Unknown		Unknown		Unknown		Unknown		Unknown			*Escherichia coli (strain K12)*	Ribosomal RNA small subunit methyltransferase F	18046181712	Unknown	
Apicycline	Protein 30S ribosomal subunit inhibitor	0.306	0.002		Unknown				Unknown		Unknown		Unknown		Unknown		Unknown			*Escherichia coli (strain K12)*	Ribosomal RNA small subunit methyltransferase F	18046181746	Unknown	
Bromotetracycline (bromtetracycline)	Protein 30S ribosomal subunit inhibitor	0.368	0.001		Unknown				Unknown		Unknown		Unknown		Unknown		Unknown			*Escherichia coli (strain K12)*	Ribosomal RNA small subunit methyltransferase F	18046181812	Unknown	
Chlortetracycline (chlorotetracycline)	Protein 30S ribosomal subunit inhibitor	0.401	0.001		Unknown				Unknown		Unknown		Unknown		Unknown		Unknown			*Escherichia coli (strain K12)*	Ribosomal RNA small subunit methyltransferase F	180330142018	Unknown	
Clomocycline	Protein 30S ribosomal subunit inhibitor	0.383	0.001		Unknown				Unknown		Unknown		Unknown		Unknown		Unknown				Unknown	180330142050	Unknown	
Demeclocycline	Protein 30S ribosomal subunit inhibitor	0.488	0.001		Unknown				Unknown		Unknown		Unknown		Unknown		Unknown			*Escherichia coli (strain K12)*	Ribosomal RNA small subunit methyltransferase F	180330142127	Unknown	
Demecycline	Protein 30S ribosomal subunit inhibitor	0.360	0.001		Unknown				Unknown		Unknown		Unknown		Unknown		Unknown				Unknown	18046181850	Unknown	
DMG-DMDOT (DMG-DM DOT)	Protein 30S ribosomal subunit inhibitor	0.314	0.002		Unknown				Unknown		Unknown		Unknown		Unknown		Unknown			*Escherichia coli (strain K12)*	Ribosomal RNA small subunit methyltransferase F	18046181955	Unknown	
DMG-MINO	Protein 30S ribosomal subunit inhibitor	0.322	0.001		Unknown				Unknown		Unknown		Unknown		Unknown		Unknown			*Escherichia coli (strain K12)*	Ribosomal RNA small subunit methyltransferase F	18046182023	Unknown	
Doxycycline	Protein 30S ribosomal subunit inhibitor	0.298	0.002		Unknown				Unknown		Unknown		Unknown		Unknown		Unknown			*Pyrococcus horikoshii (strain ATCC 700860 / DSM 12428 / JCM 9974 / NBRC 100139 / OT-3)*	30S ribosomal protein S7P	180330142208	Unknown	
Eravacycline	Protein 30S ribosomal subunit inhibitor	0.294	0.002		Unknown				Unknown		Unknown		Unknown		Unknown		Unknown			*Escherichia coli (strain K12)*	Ribosomal RNA small subunit methyltransferase F	18046182048	Unknown	
Etamocycline	Protein 30S ribosomal subunit inhibitor	0.340	0.001		Unknown				Unknown		Unknown		Unknown		Unknown		Unknown				Unknown	18046182114	Unknown	
Glycocycline	Protein 30S ribosomal subunit inhibitor	0.327	0.001		Unknown				Unknown		Unknown		Unknown		Unknown		Unknown			*Escherichia coli (strain K12)*<br>*Methanosarcina mazei (strain ATCC BAA-159 / DSM 3647 / Goe1 / Go1 / JCM 11833 / OCM 88) (Methanosarcina frisia)*	Ribosomal RNA small subunit methyltransferase F<br>30S ribosomal protein S24e	18046182149	Unknown	
Guamecycline	Protein 30S ribosomal subunit inhibitor	0.309	0.002		Unknown				Unknown		Unknown		Unknown		Unknown		Unknown			*Escherichia coli (strain K12)*	Ribosomal RNA small subunit methyltransferase F	18046194101	Unknown	
Lymecycline	Protein 30S ribosomal subunit inhibitor	0.304	0.002		Unknown				Unknown		Unknown		Unknown		Unknown		Unknown			*Methanosarcina mazei (strain ATCC BAA-159 / DSM 3647 / Goe1 / Go1 / JCM 11833 / OCM 88) (Methanosarcina frisia)*	30S ribosomal protein S24e	18046194125	Unknown	
Meclocycline	Protein 30S ribosomal subunit inhibitor	0.287	0.002		Unknown				Unknown		Unknown		Unknown		Unknown		Unknown			*Escherichia coli (strain K12)*	Ribosomal RNA small subunit methyltransferase F	18046194147	Unknown	
Meglucycline	Protein 30S ribosomal subunit inhibitor	0.350	0.001		Unknown				Unknown		Unknown		Unknown		Unknown		Unknown			*Haemophilus influenzae (strain ATCC 51907 / DSM 11121 / KW20 / Rd)*<br>*Methanosarcina mazei (strain ATCC BAA-159 / DSM 3647 / Goe1 / Go1 / JCM 11833 / OCM 88) (Methanosarcina frisia)*	Ribosomal RNA small subunit methyltransferase D<br>30S ribosomal protein S24e	18046194209	Unknown	
Metacycline (methacycline)	Protein 30S ribosomal subunit inhibitor	0.298	0.002		Unknown				Unknown		Unknown		Unknown		Unknown		Unknown				Unknown	180330142248	Unknown	
Minocycline	Protein 30S ribosomal subunit inhibitor	0.351	0.001		Unknown				Unknown		Unknown		Unknown		Unknown		Unknown				Unknown	180330142335	Unknown	
Morphocycline	Protein 30S ribosomal subunit inhibitor	0.328	0.001		Unknown				Unknown		Unknown		Unknown		Unknown		Unknown				Unknown	18046194237	Unknown	
Nitrocycline	Protein 30S ribosomal subunit inhibitor	0.330	0.001		Unknown				Unknown		Unknown		Unknown		Unknown		Unknown			*Escherichia coli (strain K12)*	Ribosomal RNA small subunit methyltransferase F	18046194308	Unknown	
Omadacycline	Protein 30S ribosomal subunit inhibitor	0.303	0.002		Unknown				Unknown		Unknown		Unknown		Unknown		Unknown			*Escherichia coli (strain K12)*	Ribosomal RNA small subunit methyltransferase F	18046194331	Unknown	
Oxytetracycline	Protein 30S ribosomal subunit inhibitor	0.335	0.001		Unknown				Unknown		Unknown		Unknown		Unknown		Unknown				Unknown	180330142441	Unknown	
Pecocycline	Protein 30S ribosomal subunit inhibitor	0.309	0.002		Unknown				Unknown		Unknown		Unknown		Unknown		Unknown			*Escherichia coli (strain K12)*	Ribosomal RNA small subunit methyltransferase F	18046194538	Unknown	
Penimepicycline	Protein 30S ribosomal subunit inhibitor	0,319	0,001		Unknown				Unknown		Unknown		Unknown		Unknown		Unknown				Unknown	18046194603	Unknown	
Penimocycline	Protein 30S ribosomal subunit inhibitor	0.277	0.002		Unknown				Unknown		Unknown		Unknown		Unknown		Unknown				Unknown	18046194652	Unknown	
Pipacycline	Protein 30S ribosomal subunit inhibitor	0.319	0.001		Unknown				Unknown		Unknown		Unknown		Unknown		Unknown			*Escherichia coli (strain K12)*	Ribosomal RNA small subunit methyltransferase F	18046194717	Unknown	
Rolitetracycline	Protein 30S ribosomal subunit inhibitor	0.340	0.001		Unknown				Unknown		Unknown		Unknown		Unknown		Unknown				Unknown	180330142519	Unknown	
Sancycline	Protein 30S ribosomal subunit inhibitor	0.350	0.001		Unknown				Unknown		Unknown		Unknown		Unknown		Unknown			*Escherichia coli (strain K12)*	Ribosomal RNA small subunit methyltransferase F	18046194744	Unknown	
Sarecycline	Protein 30S ribosomal subunit inhibitor	0.308	0.002		Unknown				Unknown		Unknown		Unknown		Unknown		Unknown			*Escherichia coli (strain K12)*	Ribosomal RNA small subunit methyltransferase F	18046194808	Unknown	
Tetracycline	Protein 30S ribosomal subunit inhibitor	0.382	0.001		Unknown				Unknown		Unknown		Unknown		Unknown		Unknown				Unknown	180330142550	Unknown	
Tigecycline	Protein 30S ribosomal subunit inhibitor	0.308	0.002		Unknown				Unknown		Unknown		Unknown		Unknown		Unknown			*Escherichia coli (strain K12)*	Ribosomal RNA small subunit methyltransferase F	180330142628	Unknown	
TP 271	Protein 30S ribosomal subunit inhibitor	0.286	0.002		Unknown				Unknown		Unknown		Unknown		Unknown		Unknown			*Escherichia coli (strain K12)*	Ribosomal RNA small subunit methyltransferase F	18046194835	Unknown	

Bioactive molecule	PASS online[4]			Similarity Ensemble Approach (SEA)[2]				ChemProt[3]		SuperPred[6]			Polypharmacology Browser (PPB)[1]			SPiDER[7]		HitPick[5]			PharmMapper[9]			TargetHunter[8]	
	Activity	Pa	Pi	Organism	Target	P-Value	Max TC	Organism	Target	Organism	Target	E-value	Organism	Target	P-Value	Target	Confidence level	Target	Precision (%)	Tc similarity	Organism	Target	Job Id	Target	Reference
Albaconazole	CYP51 inhibitor	0.577	0.002	*Mycobacterium tuberculosis*	Lanosterol 14-alpha demethylase	2.67E-11	0.51		Unknown	*Homo sapiens*	Lanosterol 14-alpha demethylase	4.39E-02	*Mycobacterium tuberculosis*	Lanosterol 14-alpha demethylase	0.01 to 0	Unknown		Unknown				Unknown	18047162916	Unknown	
	Lanosterol 14 alpha demethylase inhibitor	0.926	0.000	*Neosartorya fumigata (Aspergillus fumigatus)*	14-alpha sterol demethylase	4.56E-40	0.55																		
					14-alpha sterol demethylase Cyp51A	2.43E-38	0.55																		
				Penicillium digitatum	Cytochrome P450 14alpha-demethylase	8.34E-39	0.35																		
Alteconazole	CYP51 inhibitor	0.612	0.001	*Mycobacterium tuberculosis*	Lanosterol 14-alpha demethylase	7.74E-07	0.29	*Homo sapiens*	Lanosterol 14-alpha demethylase		Unknown		*Homo sapiens*	Cytochrome P450 51	0	Unknown		Unknown				Unknown	180515165534	Cytochrome P450 51	Drug Metab. Dispos.,(2007)35:3:493
	Lanosterol 14 alpha demethylase inhibitor	0.913	0.000	*Neosartorya fumigata (Aspergillus fumigatus)*	14-alpha sterol demethylase	9.74E-25	0.35						*Mycobacterium tuberculosis*	Lanosterol 14-alpha demethylase	0.01 to 0									Lanosterol 14-alpha demethylase	Antimicrob. Agents Chemother.,(2009)53:3:1157
				Penicillium digitatum	Cytochrome P450 14alpha-demethylase	3.59E-24	0.36						*Penicillium digitatum*	Cytochrome P450 14alpha-demethylase	0.01 to 0										
Azaconazole	CYP51 inhibitor	0.626	0.001	*Homo sapiens*	Lanosterol 14-alpha demethylase	7.05E-06	0.33	*Homo sapiens*	Lanosterol 14-alpha demethylase	*Homo sapiens*	Lanosterol 14-alpha demethylase	3.62E-02	*Homo sapiens*	Cytochrome P450 51	0	Unknown		Unknown				Unknown	180331124910	Cytochrome P450 51	Drug Metab. Dispos.,(2007)35:3:493
	Lanosterol 14 alpha demethylase inhibitor	0.869	0.001	*Mycobacterium tuberculosis*	Lanosterol 14-alpha demethylase	4.69E-07	0.30						*Mycobacterium tuberculosis*	Lanosterol 14-alpha demethylase	0.025									Lanosterol 14-alpha demethylase	Antimicrob. Agents Chemother.,(2009)53:3:1157; J Agric Food Chem,(2000)48:6:2547
				Neosartorya fumigata (Aspergillus fumigatus)	14-alpha sterol demethylase	6.87E-28	0.43						*Penicillium digitatum*	Cytochrome P450 14alpha-demethylase	0.01 to 0										
				Penicillium digitatum	Cytochrome P450 14alpha-demethylase	3.70E-24	0.35																		
				Rattus norvegicus	Lanosterol 14-alpha demethylase	2.96E-25	0.33																		
BAS 110	CYP51 inhibitor	0.519	0.002	*Mycobacterium tuberculosis*	Lanosterol 14-alpha demethylase	2.62E-07	0.32		Unknown		Unknown		*Homo sapiens*	Cytochrome P450 51	0	Unknown		Unknown				Unknown	180515165825	Cytochrome P450 51	Drug Metab. Dispos.,(2007)35:3:493
	Lanosterol 14 alpha demethylase inhibitor	0.611	0.001	*Neosartorya fumigata (Aspergillus fumigatus)*	14-alpha sterol demethylase	4.12E-23	0.32						*Mycobacterium tuberculosis*	Lanosterol 14-alpha demethylase	0.079									Lanosterol 14-alpha demethylase	J Agric Food Chem,(2000)48:6:2547
					14-alpha sterol demethylase Cyp51A	2.65E-20	0.29						*Penicillium digitatum*	Cytochrome P450 14alpha-demethylase	0										
				Penicillium digitatum	Cytochrome P450 14alpha-demethylase	4.93E-26	0.44																		
BAS 111	CYP51 inhibitor	0.971	0.000	*Penicillium digitatum*	Cytochrome P450 14alpha-demethylase	6.44E-36	0.49		Unknown		Unknown		*Penicillium digitatum*	Cytochrome P450 14alpha-demethylase	0.01 to 0	Unknown		Unknown				Unknown	180515165922	Cytochrome P450 51	Bioorg. Med. Chem. Lett.,(1998)8:14:1819
	Lanosterol 14 alpha demethylase inhibitor	0.630	0.001																						
BAS 45406F	CYP51 inhibitor	0.591	0.002	*Mycobacterium tuberculosis*	Lanosterol 14-alpha demethylase	1.07E-12	0.29		Unknown		Unknown			Unknown		Unknown		Unknown				Unknown	180515165958	Unknown	
	Lanosterol 14 alpha demethylase inhibitor	0.907	0.000	*Neosartorya fumigata (Aspergillus fumigatus)*	14-alpha sterol demethylase	2.10E-11	0.29																		
				Penicillium digitatum	Cytochrome P450 14alpha-demethylase	4.97E-35	0.33																		
Bitertanol	CYP51 inhibitor	0.551	0.002	*Penicillium digitatum*	Cytochrome P450 14alpha-demethylase	2.75E-45	0.73	*Homo sapiens*	Lanosterol 14-alpha demethylase		Unknown		*Penicillium digitatum*	Cytochrome P450 14alpha-demethylase	0	Unknown		Unknown				Unknown	180331124954	Cytochrome P450 14alpha-demethylase	J Agric Food Chem,(2009)57:15:6914; Pest Manag Sci,(2010)66:12:1344
	Lanosterol 14 alpha demethylase inhibitor	0.564	0.002																					P450 sterol 14-alpha-demethylase	J Agric Food Chem,(2013)61:7:1419
Bromuconazole	CYP51 inhibitor	0.534	0.002	*Neosartorya fumigata (Aspergillus fumigatus)*	14-alpha sterol demethylase	9.91E-28	0.38	*Homo sapiens*	Lanosterol 14-alpha demethylase		Unknown		*Homo sapiens*	Cytochrome P450 51	0	Unknown		Unknown				Unknown	180331125027	Cytochrome P450 51	Drug Metab. Dispos.,(2007)35:3:493
	Lanosterol 14 alpha demethylase inhibitor	0.642	0.001		14-alpha sterol demethylase Cyp51A	2.82E-24	0.34						*Penicillium digitatum*	Cytochrome P450 14alpha-demethylase	0.01 to 0										
				Penicillium digitatum	Cytochrome P450 14alpha-demethylase	6.34E-22	0.32																		
				Rattus norvegicus	Lanosterol 14-alpha demethylase	1.51E-23	0.30																		
Cyproconazole	CYP51 inhibitor	0.602	0.002	*Mycobacterium tuberculosis*	Lanosterol 14-alpha demethylase	1.12E-08	0.38	*Homo sapiens*	Lanosterol 14-alpha demethylase		Unknown		*Homo sapiens*	Cytochrome P450 51	0.01 to 0	Unknown		Unknown				Unknown	180331125103	Cytochrome P450 51	Drug Metab. Dispos.,(2007)35:3:493; J. Med. Chem.,(2002)45:13:2720
	Lanosterol 14 alpha demethylase inhibitor	0.758	0.001	*Neosartorya fumigata (Aspergillus fumigatus)*	14-alpha sterol demethylase	1.32E-30	0.42						*Mycobacterium tuberculosis*	Lanosterol 14-alpha demethylase	0.025									Cytochrome P450 14alpha-demethylase	J Agric Food Chem,(2009)57:15:6914
					14-alpha sterol demethylase Cyp51A	1.43E-29	0.42						*Penicillium digitatum*	Cytochrome P450 14alpha-demethylase	0										
				Penicillium digitatum	Cytochrome P450 14alpha-demethylase	5.87E-87	0.48																		
D 0870	CYP51 inhibitor	0.481	0.002	*Mycobacterium tuberculosis*	Lanosterol 14-alpha demethylase	2.55E-12	0.56		Unknown	*Homo sapiens*	Lanosterol 14-alpha demethylase	9.57E-03	*Mycobacterium tuberculosis*	Lanosterol 14-alpha demethylase	0.01 to 0	Unknown		Unknown				Unknown	18047162956	Unknown	
	Lanosterol 14 alpha demethylase inhibitor	0.970	0.000	*Neosartorya fumigata (Aspergillus fumigatus)*	14-alpha sterol demethylase	8.26E-48	0.56																		
					14-alpha sterol demethylase Cyp51A	3.31E-49	0.43																		
Diclobutrazol	CYP51 inhibitor	0.987	0.000	*Homo sapiens*	Lanosterol 14-alpha demethylase	4.28E-06	0.34		Unknown		Unknown		*Homo sapiens*	Cytochrome P450 51	0.01 to 0	Unknown		Unknown				Unknown	180331125210	Cytochrome P450 51	Drug Metab. Dispos.,(2007)35:3:493; J. Med. Chem.,(2002)45:13:2720
	Lanosterol 14 alpha demethylase inhibitor	0.626	0.001	*Neosartorya fumigata (Aspergillus fumigatus)*	14-alpha sterol demethylase	1.12E-11	0.29						*Penicillium digitatum*	Cytochrome P450 14alpha-demethylase	0									Cytochrome P450 14alpha-demethylase	J Agric Food Chem,(2009)57:15:6914
				Mycobacterium tuberculosis	Lanosterol 14-alpha demethylase	7.28E-14	0.34																	Lanosterol 14-alpha demethylase	J Agric Food Chem,(2007)55:20:8187
				Penicillium digitatum	Cytochrome P450 14alpha-demethylase	5.81E-35	0.55																		
Difenoconazole	CYP51 inhibitor	0.945	0.000	*Homo sapiens*	Lanosterol 14-alpha demethylase	8.27E-07	0.38	*Homo sapiens*	Lanosterol 14-alpha demethylase	*Homo sapiens*	Lanosterol 14-alpha demethylase	1.08E-06	*Homo sapiens*	Cytochrome P450 51	0	Unknown		Cytochrome P450 Family 51 Subfamily A Member 1	53.3	0.45		Unknown	180331125246	Cytochrome P450 51	Drug Metab. Dispos.,(2007)35:3:493
	Lanosterol 14 alpha demethylase inhibitor	0.807	0.001	*Neosartorya fumigata (Aspergillus fumigatus)*	14-alpha sterol demethylase	6.96E-30	0.48																		
					14-alpha sterol demethylase Cyp51A	9.53E-22	0.31																		
				Penicillium digitatum	Cytochrome P450 14alpha-demethylase	5.73E-33	0.34																		
				Rattus norvegicus	Lanosterol 14-alpha demethylase	1.12E-29	0.38																		
Diniconazole	CYP51 inhibitor	0.626	0.001	*Penicillium digitatum*	Cytochrome P450 14alpha-demethylase	7.24E-31	1.00		Unknown		Unknown		*Penicillium digitatum*	Cytochrome P450 14alpha-demethylase	0	Unknown		Unknown				Unknown	180331125320	Cytochrome P450 14alpha-demethylase	J Agric Food Chem,(2009)57:15:6914; Pest Manag Sci,(2010)66:12:1344
	Lanosterol 14 alpha demethylase inhibitor	0.700	0.001																						
Efinaconazole	CYP51 inhibitor	0.602	0.002	*Mycobacterium tuberculosis*	Lanosterol 14-alpha demethylase	1.15E-12	0.57		Unknown	*Homo sapiens*	Lanosterol 14-alpha demethylase	1.25E-02	*Mycobacterium tuberculosis*	Lanosterol 14-alpha demethylase	0.01 to 0	Unknown		Unknown				Unknown	180515170158	14-alpha sterol demethylase	Antimicrob. Agents Chemother.,(2010)54:10:4225
	Lanosterol 14 alpha demethylase inhibitor	0.800	0.001	*Neosartorya fumigata (Aspergillus fumigatus)*	14-alpha sterol demethylase	7.08E-55	0.59						*Penicillium digitatum*	Cytochrome P450 14alpha-demethylase	0.027									14-alpha sterol demethylase Cyp51A	Antimicrob. Agents Chemother.,(2010)54:10:4225
					14-alpha sterol demethylase Cyp51A	4.79E-61	0.59																	Cytochrome P450 51	Antimicrob. Agents Chemother.,(2009)53:3:1157; Bioorg. Med. Chem. Lett.,(1994)4:5:733; Bioorg. Med. Chem. Lett.,(1998)8:14:1819; Bioorg. Med. Chem.,(2008)16:1:209; Drug Metab. Dispos.,(2007)35:3:493; J. Med. Chem.,(1992)35:15:2818
				Penicillium digitatum	Cytochrome P450 14alpha-demethylase	1.57E-44	0.36																	Lanosterol 14-alpha demethylase	Antimicrob. Agents Chemother.,(2007)51:11:3915; Antimicrob. Agents Chemother.,(2009)53:3:1157
Embeconazole	CYP51 inhibitor	0.589	0.002	*Mycobacterium tuberculosis*	Lanosterol 14-alpha demethylase	1.10E-31	0.43		Unknown		Unknown		*Mycobacterium tuberculosis*	Lanosterol 14-alpha demethylase	0.208	Unknown		Unknown				Unknown	180515170415	Unknown	
	Lanosterol 14 alpha demethylase inhibitor	0.979	0.000	*Neosartorya fumigata (Aspergillus fumigatus)*	14-alpha sterol demethylase	1.10E-31	0.43																		
					14-alpha sterol demethylase Cyp51A	3.86E-30	0.43																		
Epoxiconazole	CYP51 inhibitor	0.554	0.002	*Mycobacterium tuberculosis*	Lanosterol 14-alpha demethylase	1.49E-07	0.33	*Homo sapiens*	Lanosterol 14-alpha demethylase		Unknown		*Homo sapiens*	Cytochrome P450 51	0.01 to 0	Unknown		Unknown				Unknown	180331125349	Cytochrome P450 51	Drug Metab. Dispos.,(2007)35:3:493
	Lanosterol 14 alpha demethylase inhibitor	0.911	0.000	*Neosartorya fumigata (Aspergillus fumigatus)*	14-alpha sterol demethylase	5.88E-13	0.33						*Mycobacterium tuberculosis*	Lanosterol 14-alpha demethylase	0.118										
				Penicillium digitatum	Cytochrome P450 14alpha-demethylase	3.44E-50	0.34						*Penicillium digitatum*	Cytochrome P450 14alpha-demethylase	0.012										
Etaconazole	CYP51 inhibitor	0.950	0.000	*Homo sapiens*	Lanosterol 14-alpha demethylase	2.63E-12	0.44	*Homo sapiens*	Lanosterol 14-alpha demethylase	*Homo sapiens*	Lanosterol 14-alpha demethylase	4.38E-07	*Homo sapiens*	Cytochrome P450 51	0	Unknown		Cytochrome P450 Family 51 Subfamily A Member 1	53.3	0.45		Unknown	180331125423	14-alpha sterol demethylase	Antimicrob. Agents Chemother.,(2010)54:10:4225
	Lanosterol 14 alpha demethylase inhibitor	0.872	0.001	*Neosartorya fumigata (Aspergillus fumigatus)*	14-alpha sterol demethylase	1.23E-32	0.56						*Penicillium digitatum*	Cytochrome P450 14alpha-demethylase	0.01 to 0									14-alpha sterol demethylase Cyp51A	Antimicrob. Agents Chemother.,(2010)54:10:4225
					14-alpha sterol demethylase Cyp51A	5.35E-21	0.30																	Cytochrome P450 51	Bioorg. Med. Chem. Lett.,(1994)4:5:733; Drug Metab. Dispos.,(2007)35:3:493
				Penicillium digitatum	Cytochrome P450 14alpha-demethylase	6.42E-25	0.37																	Lanosterol 14-alpha demethylase	Antimicrob. Agents Chemother.,(2009)53:3:1157
				Rattus norvegicus	Lanosterol 14-alpha demethylase	8.51E-56	0.44																		
Fenbuconazole	CYP51 inhibitor	0.540	0.002	*Mycobacterium tuberculosis*	Lanosterol 14-alpha demethylase	1.94E-07	0.32	*Homo sapiens*	Lanosterol 14-alpha demethylase		Unknown		*Homo sapiens*	Cytochrome P450 51	0	Unknown		Unknown				Unknown	180331125454	Cytochrome P450 14alpha-demethylase	J Agric Food Chem,(2009)57:15:6914
	Lanosterol 14 alpha demethylase inhibitor	0.454	0.002	*Neosartorya fumigata (Aspergillus fumigatus)*	14-alpha sterol demethylase	9.44E-13	0.32						*Mycobacterium tuberculosis*	Lanosterol 14-alpha demethylase	0									Cytochrome P450 51	J. Med. Chem.,(2002)45:13:2720
				Penicillium digitatum	Cytochrome P450 14alpha-demethylase	3.84E-45	0.55						*Penicillium digitatum*	Cytochrome P450 14alpha-demethylase	0										
Fluconazole	CYP51 inhibitor	0.719	0.001	*Mycobacterium tuberculosis*	Lanosterol 14-alpha demethylase	1.68E-21	1.00	*Homo sapiens*	Lanosterol 14-alpha demethylase	*Homo sapiens*	Lanosterol 14-alpha demethylase	6.97E-07	*Mycobacterium tuberculosis*	Lanosterol 14-alpha demethylase	0	Unknown		Unknown				Unknown	18047163031	14-alpha sterol demethylase	Antimicrob. Agents Chemother.,(2010)54:10:4225
	Lanosterol 14 alpha demethylase inhibitor	0.858	0.001	*Neosartorya fumigata (Aspergillus fumigatus)*	14-alpha sterol demethylase	5.54E-60	1.00	*Mycobacterium tuberculosis*	Lanosterol 14-alpha demethylase															14-alpha sterol demethylase Cyp51A	Antimicrob. Agents Chemother.,(2010)54:10:4225
					14-alpha sterol demethylase Cyp51A	1.97E-41	0.60	*Neosartorya fumigata*	14-alpha demethylase															Cytochrome P450 51	Antimicrob. Agents Chemother.,(2009)53:3:1157; Bioorg. Med. Chem. Lett.,(1994)4:5:733; Bioorg. Med. Chem. Lett.,(1998)8:14:1819; Bioorg. Med. Chem.,(2008)16:1:209; Drug Metab. Dispos.,(2007)35:3:493
				Penicillium digitatum	Cytochrome P450 14alpha-demethylase	4.45E-69	0.41	*Neosartorya fumigata (strain ATCC MYA-4609 / Af293 / CBS 101355 / FGSCA1100) (Aspergillus fumigatus)*	14-alpha sterol demethylase Cyp51A															Lanosterol 14-alpha demethylase	Antimicrob. Agents Chemother.,(2007)51:11:3915; Antimicrob. Agents Chemother.,(2009)53:3:1157
Fluotrimazole	CYP51 inhibitor	0.743	0.001	*Mycobacterium tuberculosis*	Lanosterol 14-alpha demethylase	5.78E-13	0.30	*Homo sapiens*	Lanosterol 14-alpha demethylase		Unknown		*Homo sapiens*	Cytochrome P450 51	0	Unknown		Unknown				Unknown	18047163101	Unknown	
	Lanosterol 14 alpha demethylase inhibitor	0.372	0.003	*Neosartorya fumigata (Aspergillus fumigatus)*	14-alpha sterol demethylase	1.26E-22	0.30						*Mycobacterium tuberculosis*	Lanosterol 14-alpha demethylase	0										
Fluquinconazole	CYP51 inhibitor	0.169	0.005	*Mycobacterium tuberculosis*	Lanosterol 14-alpha demethylase	1.36E-06	0.28		Unknown		Unknown			Unknown		Unknown		Unknown				Unknown	18047163126	Unknown	
	Lanosterol 14 alpha demethylase inhibitor	0.205	0.008	*Neosartorya fumigata (Aspergillus fumigatus)*	14-alpha sterol demethylase	3.07E-11	0.28																		
Flusilazole	CYP51 inhibitor	0.463	0.003	*Mycobacterium tuberculosis*	Lanosterol 14-alpha demethylase	1.62E-09	0.42	*Homo sapiens*	Lanosterol 14-alpha demethylase		Unknown		*Mycobacterium tuberculosis*	Lanosterol 14-alpha demethylase	0.01 to 0	Unknown		Unknown				Unknown	18047163200	Unknown	
	Lanosterol 14 alpha demethylase inhibitor	0.175	0.015	*Neosartorya fumigata (Aspergillus fumigatus)*	14-alpha sterol demethylase	4.95E-29	0.42						*Penicillium digitatum*	Cytochrome P450 14alpha-demethylase	0										
					14-alpha sterol demethylase Cyp51A	6.28E-24	0.34																		
				Penicillium digitatum	Cytochrome P450 14alpha-demethylase	8.15E-90	0.48																		
Flutriafol	CYP51 inhibitor	0.558	0.002	*Mycobacterium tuberculosis*	Lanosterol 14-alpha demethylase	3.11E-12	0.55	*Homo sapiens*	Lanosterol 14-alpha demethylase	*Homo sapiens*	Lanosterol 14-alpha demethylase	1.36E-02	*Homo sapiens*	Cytochrome P450 51	0.01 to 0	Unknown		Unknown				Unknown	180515170549	14-alpha sterol demethylase	Antimicrob. Agents Chemother.,(2010)54:10:4225
	Lanosterol 14 alpha demethylase inhibitor	0.457	0.002	*Neosartorya fumigata (Aspergillus fumigatus)*	14-alpha sterol demethylase	9.95E-38	0.55	*Mycobacterium tuberculosis*	Lanosterol 14-alpha demethylase				*Mycobacterium tuberculosis*	Lanosterol 14-alpha demethylase	0									14-alpha sterol demethylase Cyp51A	Antimicrob. Agents Chemother.,(2010)54:10:4225
					14-alpha sterol demethylase Cyp51A	5.54E-31	0.44	*Neosartorya fumigata*	14-alpha demethylase															Cytochrome P450 51	Antimicrob. Agents Chemother.,(2009)53:3:1157; Bioorg. Med. Chem. Lett.,(1994)4:5:733; Bioorg. Med. Chem. Lett.,(1998)8:14:1819; Bioorg. Med. Chem.,(2008)16:1:209; Drug Metab. Dispos.,(2007)35:3:493; J. Med. Chem.,(1992)35:15:2818; J. Med. Chem.,(1993)36:15:2235
				Penicillium digitatum	Cytochrome P450 14alpha-demethylase	7.62E-72	0.42	*Neosartorya fumigata (strain ATCC MYA-4609 / Af293 / CBS 101355 / FGSCA1100) (Aspergillus fumigatus)*	14-alpha sterol demethylase Cyp51A															Lanosterol 14-alpha demethylase	Antimicrob. Agents Chemother.,(2007)51:11:3915; Antimicrob. Agents Chemother.,(2009)53:3:1157
Fosfluconazole	CYP51 inhibitor	0.623	0.001	*Mycobacterium tuberculosis*	Lanosterol 14-alpha demethylase	5.34E-14	0.64		Unknown	*Homo sapiens*	Lanosterol 14-alpha demethylase	2.25E-03	*Mycobacterium tuberculosis*	Lanosterol 14-alpha demethylase	0	Unknown		Unknown				Unknown	180515170629	14-alpha sterol demethylase	Antimicrob. Agents Chemother.,(2010)54:10:4225
	Lanosterol 14 alpha demethylase inhibitor	0.381	0.003	*Neosartorya fumigata (Aspergillus fumigatus)*	14-alpha sterol demethylase	2.06E-42	0.64																	14-alpha sterol demethylase Cyp51A	Antimicrob. Agents Chemother.,(2010)54:10:4225
					14-alpha sterol demethylase Cyp51A	8.64E-34	0.48																	Cytochrome P450 51	Antimicrob. Agents Chemother.,(2009)53:3:1157; Bioorg. Med. Chem. Lett.,(1994)4:5:733; Bioorg. Med. Chem. Lett.,(1998)8:14:1819; Bioorg. Med. Chem.,(2008)16:1:209; Drug Metab. Dispos.,(2007)35:3:493
				Penicillium digitatum	Cytochrome P450 14alpha-demethylase	2.09E-23	0.34																	Lanosterol 14-alpha demethylase	Antimicrob. Agents Chemother.,(2007)51:11:3915; Antimicrob. Agents Chemother.,(2009)53:3:1157
Furconazole	CYP51 inhibitor	0.475	0.002	*Neosartorya fumigata (Aspergillus fumigatus)*	14-alpha sterol demethylase	1.13E-13	0.35	*Homo sapiens*	Lanosterol 14-alpha demethylase		Unknown		*Homo sapiens*	Cytochrome P450 51	0.01 to 0	Unknown		Unknown				Unknown	180515170715	Cytochrome P450 51	Drug Metab. Dispos.,(2007)35:3:493
	Lanosterol 14 alpha demethylase inhibitor	0.458	0.002	*Penicillium digitatum*	Cytochrome P450 14alpha-demethylase	8.48E-11	0.30						*Penicillium digitatum*	Cytochrome P450 14alpha-demethylase	0.016										

Bioactive molecule	PASS online[4]			Similarity Ensemble Approach (SEA)[2]				ChemProt[3]	
	Activity	Pa	Pi	Organism	Target	P-Value	Max TC	Organism	Target
Genaconazole (SCH 39304)	CYP51 inhibitor	0.973	0.000	*Mycobacterium tuberculosis*	Lanosterol 14-alpha demethylase	1.38E-14	0.67		Unknown
	Lanosterol 14 alpha demethylase inhibitor	0.948	0.000	*Neosartorya fumigata (Aspergillus fumigatus)*	14-alpha sterol demethylase	2.53E-61	0.67		
					14-alpha sterol demethylase Cyp51A	1.40E-66	0.67		
				Penicillium digitatum	Cytochrome P450 14alpha-demethylase	1.88E-56	0.37		
Hexaconazole	CYP51 inhibitor	0.629	0.001	*Mycobacterium tuberculosis*	Lanosterol 14-alpha demethylase	1.52E-11	0.52	*Homo sapiens*	Lanosterol 14-alpha demethylase
	Lanosterol 14 alpha demethylase inhibitor	0.796	0.001	*Neosartorya fumigata (Aspergillus fumigatus)*	14-alpha sterol demethylase	3.93E-34	0.52		
					14-alpha sterol demethylase Cyp51A	1.23E-26	0.38		
				Penicillium digitatum	Cytochrome P450 14alpha-demethylase	1.69E-90	0.51		
ICI 153066	CYP51 inhibitor	0.580	0.002	*Mycobacterium tuberculosis*	Lanosterol 14-alpha demethylase	1.30E-12	0.57	*Homo sapiens*	Lanosterol 14-alpha demethylase
	Lanosterol 14 alpha demethylase inhibitor	0.630	0.001	*Neosartorya fumigata (Aspergillus fumigatus)*	14-alpha sterol demethylase	4.64E-39	0.57	*Mycobacterium tuberculosis*	Lanosterol 14-alpha demethylase
					14-alpha sterol demethylase Cyp51A	3.46E-32	0.46	*Neosartorya fumigata*	14-alpha demethylase
				Penicillium digitatum	Cytochrome P450 14alpha-demethylase	6.74E-57	0.42	*Neosartorya fumigata (strain ATCC MYA-4609 / Af293 / CBS 101355 / FGSCA1100) (Aspergillus fumigatus)*	14-alpha sterol demethylase Cyp51A
ICI 195739	CYP51 inhibitor	0.481	0.002	*Mycobacterium tuberculosis*	Lanosterol 14-alpha demethylase	2.55E-12	0.56		Unknown
	Lanosterol 14 alpha demethylase inhibitor	0.970	0.000	*Neosartorya fumigata (Aspergillus fumigatus)*	14-alpha sterol demethylase	8.26E-48	0.56		
					14-alpha sterol demethylase Cyp51A	3.31E-49	0.43		
Imibenconazole	CYP51 inhibitor	0.560	0.002	*Mycobacterium tuberculosis*	Lanosterol 14-alpha demethylase	2.51E-07	0.32		Unknown
	Lanosterol 14 alpha demethylase inhibitor	0.609	0.001	*Neosartorya fumigata (Aspergillus fumigatus)*	14-alpha sterol demethylase	1.49E-12	0.32		
				Penicillium digitatum	Cytochrome P450 14alpha-demethylase	1.18E-23	0.38		
Ipconazole	CYP51 inhibitor	0.500	0.002	*Mycobacterium tuberculosis*	Lanosterol 14-alpha demethylase	1.10E-06	0.29		Unknown
	Lanosterol 14 alpha demethylase inhibitor	0.356	0.003	*Neosartorya fumigata (Aspergillus fumigatus)*	14-alpha sterol demethylase	1.33E-22	0.30		
				Penicillium digitatum	Cytochrome P450 14alpha-demethylase	7.89E-29	0.39		
Ipfentrifluconazole	CYP51 inhibitor	0.526	0.002	*Mycobacterium tuberculosis*	Lanosterol 14-alpha demethylase	1.51E-09	0.42		Unknown
	Lanosterol 14 alpha demethylase inhibitor	0.404	0.003	*Neosartorya fumigata (Aspergillus fumigatus)*	14-alpha sterol demethylase	1.69E-43	0.43		
					14-alpha sterol demethylase Cyp51A	1.89E-30	0.43		
				Penicillium digitatum	Cytochrome P450 14alpha-demethylase	1.72E-54	0.44		
Isavuconazole	CYP51 inhibitor	0.493	0.002	*Mycobacterium tuberculosis*	Lanosterol 14-alpha demethylase	2.67E-09	0.41	*Neosartorya fumigata*	14-alpha sterol demethylase
	Lanosterol 14 alpha demethylase inhibitor	0.903	0.001	*Neosartorya fumigata (Aspergillus fumigatus)*	14-alpha sterol demethylase	7.16E-34	0.48	*Neosartorya fumigata (strain ATCC MYA-4609 / Af293 / CBS 101355 / FGSCA1100) (Aspergillus fumigatus)*	14-alpha sterol demethylase Cyp51A
					14-alpha sterol demethylase Cyp51A	1.58E-33	0.48		
				Penicillium digitatum	Cytochrome P450 14alpha-demethylase	5.36E-14	0.30		
Itraconazole	CYP51 inhibitor	0.944	0.000	*Homo sapiens*	Lanosterol 14-alpha demethylase	1.37E-09	0.56	*Homo sapiens*	Lanosterol 14-alpha demethylase
	Lanosterol 14 alpha demethylase inhibitor	0.983	0.000	*Neosartorya fumigata (Aspergillus fumigatus)*	14-alpha sterol demethylase	3.10E-58	1.00	*Neosartorya fumigata*	14-alpha sterol demethylase
					14-alpha sterol demethylase Cyp51A	3.18E-38	0.55	*Neosartorya fumigata (strain ATCC MYA-4609 / Af293 / CBS 101355 / FGSCA1100) (Aspergillus fumigatus)*	14-alpha sterol demethylase Cyp51A
				Rattus norvegicus	Lanosterol 14-alpha demethylase	6.89E-43	0.56	*Trypanosoma cruzi (strain CL Brener)*	Sterol 14-alpha demethylase
LAB 158241F	CYP51 inhibitor	0.571	0.002	*Penicillium digitatum*	Cytochrome P450 14alpha-demethylase	4.41E-18	0.50		Unknown
	Lanosterol 14 alpha demethylase inhibitor	0.439	0.002						
LAB 170250F	CYP51 inhibitor	0.591	0.002	*Mycobacterium tuberculosis*	Lanosterol 14-alpha demethylase	1.40E-06	0.28	*Homo sapiens*	Lanosterol 14-alpha demethylase
	Lanosterol 14 alpha demethylase inhibitor	0.822	0.001	*Neosartorya fumigata (Aspergillus fumigatus)*	14-alpha sterol demethylase	2.43E-22	0.30		
				Penicillium digitatum	Cytochrome P450 14alpha-demethylase	1.36E-43	0.37		
Mefentrifluconazole	CYP51 inhibitor	0.544	0.002	*Mycobacterium tuberculosis*	Lanosterol 14-alpha demethylase	5.10E-10	0.45		Unknown
	Lanosterol 14 alpha demethylase inhibitor	0.440	0.002	*Neosartorya fumigata (Aspergillus fumigatus)*	14-alpha sterol demethylase	3.93E-31	0.45		
					14-alpha sterol demethylase Cyp51A	3.89E-26	0.37		
				Penicillium digitatum	Cytochrome P450 14alpha-demethylase	1.85E-51	0.47		
Metconazole	CYP51 inhibitor	0.496	0.002	*Mycobacterium tuberculosis*	Lanosterol 14-alpha demethylase	7.04E-07	0.30		Unknown
	Lanosterol 14 alpha demethylase inhibitor	0.381	0.003	*Neosartorya fumigata (Aspergillus fumigatus)*	14-alpha sterol demethylase	9.42E-12	0.30		
				Penicillium digitatum	Cytochrome P450 14alpha-demethylase	3.76E-26	0.41		
Myclobutanil	CYP51 inhibitor	0.517	0.002	*Mycobacterium tuberculosis*	Lanosterol 14-alpha demethylase	8.52E-08	0.34	*Homo sapiens*	Lanosterol 14-alpha demethylase
	Lanosterol 14 alpha demethylase inhibitor	0.448	0.002	*Neosartorya fumigata (Aspergillus fumigatus)*	14-alpha sterol demethylase	1.13E-24	0.34		
					14-alpha sterol demethylase Cyp51A	1.43E-21	0.30		
				Penicillium digitatum	Cytochrome P450 14alpha-demethylase	1.04E-91	0.49		
Penconazole	CYP51 inhibitor	0.620	0.001	*Homo sapiens*	Lanosterol 14-alpha demethylase	8.00E-12	0.35		Unknown
	Lanosterol 14 alpha demethylase inhibitor	0.638	0.001	*Mycobacterium tuberculosis*	Lanosterol 14-alpha demethylase	4.56E-22	0.35		
				Neosartorya fumigata (Aspergillus fumigatus)	14-alpha sterol demethylase	1.93E-34	0.33		
					14-alpha sterol demethylase Cyp51A	7.10E-21	0.29		
				Penicillium digitatum	Cytochrome P450 14alpha-demethylase	2.665E-115	0.48		
Posaconazole	CYP51 inhibitor	0.568	0.002	*Homo sapiens*	Lanosterol 14-alpha demethylase	7.23E-06	0.32	*Homo sapiens*	Lanosterol 14-alpha demethylase
	Lanosterol 14 alpha demethylase inhibitor	0.972	0.000	*Neosartorya fumigata (Aspergillus fumigatus)*	14-alpha sterol demethylase	5.50E-69	1.00	*Neosartorya fumigata*	14-alpha sterol demethylase
					14-alpha sterol demethylase Cyp51A	1.18E-88	1.00	*Neosartorya fumigata (strain ATCC MYA-4609 / Af293 / CBS 101355 / FGSCA1100) (Aspergillus fumigatus)*	14-alpha sterol demethylase Cyp51A
				Rattus norvegicus	Lanosterol 14-alpha demethylase	3.33E-25	0.32	*Trypanosoma cruzi (strain CL Brener)*	Sterol 14-alpha demethylase
PP 969	CYP51 inhibitor	0.980	0.000	*Penicillium digitatum*	Cytochrome P450 14alpha-demethylase	3.98E-10	0.44		Unknown
	Lanosterol 14 alpha demethylase inhibitor	0.478	0.002						
Pramiconazole	CYP51 inhibitor	0.390	0.003	*Homo sapiens*	Lanosterol 14-alpha demethylase	4.63E-06	0.34	*Homo sapiens*	Lanosterol 14-alpha demethylase
	Lanosterol 14 alpha demethylase inhibitor	0.583	0.002	*Neosartorya fumigata (Aspergillus fumigatus)*	14-alpha sterol demethylase	1.76E-49	0.58	*Neosartorya fumigata*	14-alpha sterol demethylase
					14-alpha sterol demethylase Cyp51A	4.14E-60	0.58	*Neosartorya fumigata (strain ATCC MYA-4609 / Af293 / CBS 101355 / FGSCA1100) (Aspergillus fumigatus)*	14-alpha sterol demethylase Cyp51A
				Rattus norvegicus	Lanosterol 14-alpha demethylase	4.01E-26	0.34	*Trypanosoma cruzi (strain CL Brener)*	Sterol 14-alpha demethylase
Propiconazole	CYP51 inhibitor	0.945	0.000	*Homo sapiens*	Lanosterol 14-alpha demethylase	1.71E-07	0.43		Unknown
	Lanosterol 14 alpha demethylase inhibitor	0.869	0.001	*Neosartorya fumigata (Aspergillus fumigatus)*	14-alpha sterol demethylase	4.94E-32	0.54		
					14-alpha sterol demethylase Cyp51A	2.82E-21	0.30		
				Penicillium digitatum	Cytochrome P450 14alpha-demethylase	8.40E-28	0.38		
				Rattus norvegicus	Lanosterol 14-alpha demethylase	6.35E-33	0.43		
Quinconazole	CYP51 inhibitor	0.192	0.005		Unknown				Unknown
	Lanosterol 14 alpha demethylase inhibitor	0.166	0.019						
Ravuconazole	CYP51 inhibitor	0.506	0.002	*Mycobacterium tuberculosis*	Lanosterol 14-alpha demethylase	1.19E-10	0.48	*Neosartorya fumigata*	14-alpha sterol demethylase
	Lanosterol 14 alpha demethylase inhibitor	0.918	0.000	*Neosartorya fumigata (Aspergillus fumigatus)*	14-alpha sterol demethylase	2.23E-39	0.56	*Neosartorya fumigata (strain ATCC MYA-4609 / Af293 / CBS 101355 / FGSCA1100) (Aspergillus fumigatus)*	14-alpha sterol demethylase Cyp51A
					14-alpha sterol demethylase Cyp51A	3.35E-39	0.56		
				Penicillium digitatum	Cytochrome P450 14alpha-demethylase	8.36E-14	0.29		
Saperconazole	CYP51 inhibitor	0.918	0.000	*Homo sapiens*	Lanosterol 14-alpha demethylase	1.24E-07	0.44	*Homo sapiens*	Lanosterol 14-alpha demethylase
	Lanosterol 14 alpha demethylase inhibitor	0.977	0.000	*Neosartorya fumigata (Aspergillus fumigatus)*	14-alpha sterol demethylase	1.43E-55	0.81	*Neosartorya fumigata*	14-alpha sterol demethylase
					14-alpha sterol demethylase Cyp51A	5.42E-46	0.66	*Neosartorya fumigata (strain ATCC MYA-4609 / Af293 / CBS 101355 / FGSCA1100) (Aspergillus fumigatus)*	14-alpha sterol demethylase Cyp51A
				Rattus norvegicus	Lanosterol 14-alpha demethylase	1.38E-33	0.44	*Trypanosoma cruzi (strain CL Brener)*	Sterol 14-alpha demethylase

Bioactive molecule	SuperPred[6]			Polypharmacology Browser (PPB)[1]			SPiDER[7]		HitPick[5]		
	Organism	Target	E-value	Organism	Target	P-Value	Target	Confidence level	Target	Precision (%)	Tc similarity
Genaconazole (SCH 39304)	*Homo sapiens*	Lanosterol 14-alpha demethylase	4.13E-03	*Mycobacterium tuberculosis*	Lanosterol 14-alpha demethylase	0	Unknown		Unknown		
				Penicillium digitatum	Cytochrome P450 14alpha-demethylase	0.081					
Hexaconazole	*Homo sapiens*	Lanosterol 14-alpha demethylase	5.98E-02	*Homo sapiens*	Cytochrome P450 51	0	Unknown		Unknown		
				Mycobacterium tuberculosis	Lanosterol 14-alpha demethylase	0.01 to 0					
				Penicillium digitatum	Cytochrome P450 14alpha-demethylase	0					
ICI 153066	*Homo sapiens*	Lanosterol 14-alpha demethylase	1.74E-02	*Homo sapiens*	Cytochrome P450 51	0	Unknown		Unknown		
				Mycobacterium tuberculosis	Lanosterol 14-alpha demethylase	0.01 to 0					
ICI 195739	*Homo sapiens*	Lanosterol 14-alpha demethylase	9.57E-03	*Mycobacterium tuberculosis*	Lanosterol 14-alpha demethylase	0.01 to 0	Unknown		Unknown		
Imibenconazole		Unknown		*Homo sapiens*	Cytochrome P450 51	0.01 to 0	Unknown		Unknown		
				Penicillium digitatum	Cytochrome P450 14alpha-demethylase	0.01 to 0					
Ipconazole		Unknown		*Homo sapiens*	Cytochrome P450 51	0.01 to 0	Unknown		Unknown		
				Penicillium digitatum	Cytochrome P450 14alpha-demethylase	0					
Ipfentrifluconazole		Unknown		*Mycobacterium tuberculosis*	Lanosterol 14-alpha demethylase	0.016	Unknown		Unknown		
				Penicillium digitatum	Cytochrome P450 14alpha-demethylase	0.01 to 0					
Isavuconazole		Unknown					Unknown		Cytochrome P450 Family 51 Subfamily A Member 1	53.3	0.42
					Unknown						
Itraconazole	*Homo sapiens*	Lanosterol 14-alpha demethylase	6.32E-15	*Homo sapiens*	Cytochrome P450 51	0	Unknown		Cytochrome P450 Family 51 Subfamily A Member 1	100	1
LAB 158241F		Unknown		*Penicillium digitatum*	Cytochrome P450 14alpha-demethylase	0	Unknown		Unknown		
LAB 170250F		Unknown		*Homo sapiens*	Cytochrome P450 51	0.01 to 0	Unknown		Unknown		
				Penicillium digitatum	Cytochrome P450 14alpha-demethylase	0.01 to 0					
Mefentrifluconazole		Unknown		*Mycobacterium tuberculosis*	Lanosterol 14-alpha demethylase	0.01 to 0	Unknown		Unknown		
				Penicillium digitatum	Cytochrome P450 14alpha-demethylase	0.031					
Metconazole		Unknown		*Homo sapiens*	Cytochrome P450 51	0.01 to 0	Unknown		Unknown		
				Penicillium digitatum	Cytochrome P450 14alpha-demethylase	0					
Myclobutanil		Unknown		*Penicillium digitatum*	Cytochrome P450 14alpha-demethylase	0	Unknown		Unknown		
Penconazole		Unknown		*Homo sapiens*	Cytochrome P450 51	0	Unknown		Unknown		
				Penicillium digitatum	Cytochrome P450 14alpha-demethylase	0					
Posaconazole	*Homo sapiens*	Lanosterol 14-alpha demethylase	2.17E-02		Unknown		Unknown		Cytochrome P450 Family 51 Subfamily A Member 1	100	1
PP 969		Unknown		*Penicillium digitatum*	Cytochrome P450 14alpha-demethylase	0.01 to 0	Unknown		Unknown		
Pramiconazole		Unknown		*Homo sapiens*	Cytochrome P450 51	0	Unknown		Cytochrome P450 Family 51 Subfamily A Member 1	53.3	0.42
Propiconazole	*Homo sapiens*	Lanosterol 14-alpha demethylase	7.55E-07	*Homo sapiens*	Cytochrome P450 51	0	Unknown		Cytochrome P450 Family 51 Subfamily A Member 1	77	0.54
				Penicillium digitatum	Cytochrome P450 14alpha-demethylase	0.01 to 0					
Quinconazole		Unknown			Unknown		Unknown		Unknown		
Ravuconazole	*Homo sapiens*	Lanosterol 14-alpha demethylase	4.39E-02		Unknown		Unknown		Cytochrome P450 Family 51 Subfamily A Member 1	77	0.53
Saperconazole	*Homo sapiens*	Lanosterol 14-alpha demethylase	6.27E-08	*Homo sapiens*	Cytochrome P450 51	0	Unknown		Cytochrome P450 Family 51 Subfamily A Member 1	97.3	1

Bioactive molecule	PharmMapper[9]			TargetHunter[8]	
	Organism	Target	Job Id	Target	Reference
Genaconazole (SCH 39304)		Unknown	180515170836	14-alpha sterol demethylase	Antimicrob. Agents Chemother.,(2010)54:10:4225
				14-alpha sterol demethylase Cyp51A	Antimicrob. Agents Chemother.,(2010)54:10:4225
				Cytochrome P450 51	Antimicrob. Agents Chemother.,(2009)53:3:1157; Bioorg. Med. Chem. Lett.,(1994)4:5:733; Bioorg. Med. Chem. Lett.,(1998)8:14:1819; Bioorg. Med. Chem.,(2008)16:1:209; Drug Metab. Dispos.,(2007)35:3:493
				Lanosterol 14-alpha demethylase	Antimicrob. Agents Chemother.,(2007)51:11:3915; Antimicrob. Agents Chemother.,(2009)53:3:1157
Hexaconazole		Unknown	180515170915	Cytochrome P450 14alpha-demethylase	J Agric Food Chem,(2009)57:15:6914
				Cytochrome P450 51	Drug Metab. Dispos.,(2007)35:3:493; J. Med. Chem.,(2002)45:13:2720
ICI 153066		Unknown	180515174754	14-alpha sterol demethylase	Antimicrob. Agents Chemother.,(2010)54:10:4225
				14-alpha sterol demethylase Cyp51A	Antimicrob. Agents Chemother.,(2010)54:10:4225
				Cytochrome P450 51	Antimicrob. Agents Chemother.,(2009)53:3:1157; Bioorg. Med. Chem. Lett.,(1994)4:5:733; Bioorg. Med. Chem. Lett.,(1998)8:14:1819; Bioorg. Med. Chem.,(2008)16:1:209; Drug Metab. Dispos.,(2007)35:3:493
				Lanosterol 14-alpha demethylase	Antimicrob. Agents Chemother.,(2007)51:11:3915; Antimicrob. Agents Chemother.,(2009)53:3:1157; J Agric Food Chem,(2007)55:20:8187
ICI 195739		Unknown	180515174945	Unknown	
Imibenconazole		Unknown	18047163229	Unknown	
Ipconazole		Unknown	180515175021	Cytochrome P450 14alpha-demethylase	J Agric Food Chem,(2009)57:15:6914
				Cytochrome P450 51	Bioorg. Med. Chem. Lett.,(1994)4:5:733; Bioorg. Med. Chem. Lett.,(1998)8:14:1819
Ipfentrifluconazole		Unknown	180515175054	Unknown	
Isavuconazole		Unknown	180515175128	Unknown	
Itraconazole		Unknown	180515175841	14-alpha sterol demethylase	Antimicrob. Agents Chemother.,(2010)54:10:4225
				14-alpha sterol demethylase Cyp51A	Antimicrob. Agents Chemother.,(2010)54:10:4225
				Cytochrome P450 51	Antimicrob. Agents Chemother.,(2009)53:3:1157; Bioorg. Med. Chem. Lett.,(1994)4:5:733; Bioorg. Med. Chem.,(2008)16:1:209; Drug Metab. Dispos.,(2007)35:3:493; J. Med. Chem.,(1992)35:15:2818; J. Med. Chem.,(1993)36:15:2235; J. Med. Chem.,(2009)52:6:1639
				Sterol 14-alpha demethylase	Antimicrob. Agents Chemother.,(2010)54:6:2480
LAB 158241F		Unknown	180515175751	Unknown	
LAB 170250F		Unknown	180623164237	Cytochrome P450 14alpha-demethylase	J Agric Food Chem,(2009)57:15:6914
				Cytochrome P450 51	Drug Metab. Dispos.,(2007)35:3:493
				Lanosterol 14-alpha demethylase	Antimicrob. Agents Chemother.,(2009)53:3:1157
Mefentrifluconazole		Unknown	18047163301	Unknown	
Metconazole		Unknown	180515180130	Cytochrome P450 14alpha-demethylase	J Agric Food Chem,(2009)57:15:6914
				Cytochrome P450 51	Bioorg. Med. Chem. Lett.,(1994)4:5:733; Bioorg. Med. Chem. Lett.,(1998)8:14:1819
Myclobutanil		Unknown	180515180305	Cytochrome P450 14alpha-demethylase	J Agric Food Chem,(2009)57:15:6914
				Cytochrome P450 51	J. Med. Chem.,(2002)45:13:2720
Penconazole		Unknown	180515180422	Cytochrome P450 14alpha-demethylase	J Agric Food Chem,(2009)57:15:6914
				Cytochrome P450 51	J. Med. Chem.,(2002)45:13:2720
				Lanosterol 14-alpha demethylase	J Agric Food Chem,(2000)48:6:2547; J Agric Food Chem,(2007)55:20:8187
Posaconazole		Unknown	18047163324	14-alpha sterol demethylase	Antimicrob. Agents Chemother.,(2010)54:10:4225
				14-alpha sterol demethylase Cyp51A	Antimicrob. Agents Chemother.,(2010)54:10:4225
				Cytochrome P450 51	Bioorg. Med. Chem. Lett.,(1994)4:5:733; Drug Metab. Dispos.,(2007)35:3:493
				Sterol 14-alpha demethylase	Antimicrob. Agents Chemother.,(2010)54:6:2480
PP 969		Unknown	180515180553	Unknown	
Pramiconazole		Unknown	180515180929	14-alpha sterol demethylase	Antimicrob. Agents Chemother.,(2010)54:10:4225
				14-alpha sterol demethylase Cyp51A	Antimicrob. Agents Chemother.,(2010)54:10:4225
				Sterol 14-alpha demethylase	Antimicrob. Agents Chemother.,(2010)54:6:2480
Propiconazole		Unknown	180515181009	14-alpha sterol demethylase	Antimicrob. Agents Chemother.,(2010)54:10:4225
				14-alpha sterol demethylase Cyp51A	Antimicrob. Agents Chemother.,(2010)54:10:4225
				Cytochrome P450 51	Antimicrob. Agents Chemother.,(2009)53:3:1157; Bioorg. Med. Chem. Lett.,(1994)4:5:733
				Lanosterol 14-alpha demethylase	Antimicrob. Agents Chemother.,(2009)53:3:1157
Quinconazole		Unknown	180515183150	Unknown	
Ravuconazole		Unknown	180515183227	Unknown	
Saperconazole		Unknown	180515183256	14-alpha sterol demethylase	Antimicrob. Agents Chemother.,(2010)54:10:4225
				14-alpha sterol demethylase Cyp51A	Antimicrob. Agents Chemother.,(2010)54:10:4225
				Cytochrome P450 51	Bioorg. Med. Chem. Lett.,(1994)4:5:733; Drug Metab. Dispos.,(2007)35:3:493
				Sterol 14-alpha demethylase	Antimicrob. Agents Chemother.,(2010)54:6:2480

Bioactive molecule	PASS online[4]			Similarity Ensemble Approach (SEA)[2]				ChemProt[3]		SuperPred[6]			Polypharmacology Browser (PPB)[1]			SPiDER[7]		HitPick[5]			PharmMapper[9]			TargetHunter[8]	
	Activity	Pa	Pi	Organism	Target	P-Value	Max TC	Organism	Target	Organism	Target	E-value	Organism	Target	P-Value	Target	Confidence level	Target	Precision (%)	Tc similarity	Organism	Target	Job Id	Target	Reference
SCH 42427	CYP51 inhibitor<br>Lanosterol 14 alpha demethylase inhibitor	0.973<br>0.948	0.000<br>0.000	*Mycobacterium tuberculosis*<br>*Neosartorya fumigata (Aspergillus fumigatus)*<br><br>*Penicillium digitatum*	Lanosterol 14-alpha demethylase<br>14-alpha sterol demethylase<br>14-alpha sterol demethylase Cyp51A<br>Cytochrome P450 14alpha-demethylase	1.38E-14<br>2.53E-61<br>1.40E-66<br>1.88E-56	0.67<br>0.67<br>0.67<br>0.37		Unknown	*Homo sapiens*	Lanosterol 14-alpha demethylase	4.13E-03	*Mycobacterium tuberculosis*<br>*Penicillium digitatum*	Lanosterol 14-alpha demethylase<br>Cytochrome P450 14alpha-demethylase	0<br>0.081	Unknown		Unknown				Unknown	180515183325	14-alpha sterol demethylase<br>14-alpha sterol demethylase Cyp51A<br>Cytochrome P450 51<br>Lanosterol 14-alpha demethylase	Antimicrob. Agents Chemother.,(2010)54:10:4225<br>Antimicrob. Agents Chemother.,(2010)54:10:4225<br>Antimicrob. Agents Chemother.,(2009)53:3:1157; Bioorg. Med. Chem. Lett.,(1994)4:5:733; Bioorg. Med. Chem. Lett.,(1998)8:14:1819; Bioorg. Med. Chem.,(2008)16:1:209; Drug Metab. Dispos.,(2007)35:3:493<br>Antimicrob. Agents Chemother.,(2007)51:11:3915; Antimicrob. Agents Chemother.,(2009)53:3:1157
SCH 51048	CYP51 inhibitor<br>Lanosterol 14 alpha demethylase inhibitor	0.602<br>0.953	0.002<br>0.000	*Homo sapiens*<br>*Neosartorya fumigata (Aspergillus fumigatus)*<br><br>*Rattus norvegicus*	Lanosterol 14-alpha demethylase<br>14-alpha sterol demethylase<br>14-alpha sterol demethylase Cyp51A<br>Lanosterol 14-alpha demethylase	6.53E-06<br>5.96E-53<br>2.19E-58<br>2.06E-25	0.33<br>0.85<br>0.85<br>0.33	*Homo sapiens*<br>*Neosartorya fumigata*<br>*Neosartorya fumigata (strain ATCC MYA-4609 / Af293 / CBS 101355 / FGSCA1100) (Aspergillus fumigatus)*<br>*Trypanosoma cruzi (strain CL Brener)*	Lanosterol 14-alpha demethylase<br>14-alpha sterol demethylase<br>14-alpha sterol demethylase Cyp51A<br>Sterol 14-alpha demethylase	*Homo sapiens*	Lanosterol 14-alpha demethylase	1.56E-02	*Homo sapiens*	Cytochrome P450 51	0	Unknown		Cytochrome P450 Family 51 Subfamily A Member 1	96.7	0.89		Unknown	180515183435	14-alpha sterol demethylase<br>14-alpha sterol demethylase Cyp51A<br>Cytochrome P450 51<br>Sterol 14-alpha demethylase	Antimicrob. Agents Chemother.,(2010)54:10:4225<br>Antimicrob. Agents Chemother.,(2010)54:10:4225<br>Bioorg. Med. Chem. Lett.,(1994)4:5:733; Drug Metab. Dispos.,(2007)35:3:493<br>Antimicrob. Agents Chemother.,(2010)54:6:2480
SDZ 89-485	CYP51 inhibitor<br>Lanosterol 14 alpha demethylase inhibitor	0.591<br>0.873	0.002<br>0.001	*Mycobacterium tuberculosis*<br>*Neosartorya fumigata (Aspergillus fumigatus)*<br><br>*Penicillium digitatum*	Lanosterol 14-alpha demethylase<br>14-alpha sterol demethylase<br>14-alpha sterol demethylase Cyp51A<br>Cytochrome P450 14alpha-demethylase	7.97E-09<br>5.90E-28<br>3.20E-24<br>8.94E-89	0.39<br>0.39<br>0.34<br>0.52	*Homo sapiens*	Lanosterol 14-alpha demethylase		Unknown		*Homo sapiens*<br>*Mycobacterium tuberculosis*<br>*Penicillium digitatum*	Cytochrome P450 51<br>Lanosterol 14-alpha demethylase<br>Cytochrome P450 14alpha-demethylase	0.01 to 0<br>0.025<br>0	Unknown		Unknown				Unknown	180515183511	Cytochrome P450 14alpha-demethylase<br>Cytochrome P450 51	J Agric Food Chem,(2009)57:15:6914<br>Drug Metab. Dispos.,(2007)35:3:493; J. Med. Chem.,(2002)45:13:2720
Simeconazole	CYP51 inhibitor<br>Lanosterol 14 alpha demethylase inhibitor	0.593<br>0.467	0.002<br>0.002	*Mycobacterium tuberculosis*<br>*Neosartorya fumigata (Aspergillus fumigatus)*<br><br>*Penicillium digitatum*	Lanosterol 14-alpha demethylase<br>14-alpha sterol demethylase<br>14-alpha sterol demethylase Cyp51A<br>Cytochrome P450 14alpha-demethylase	9.62E-13<br>2.70E-38<br>2.38E-30<br>4.19E-77	0.58<br>0.58<br>0.43<br>0.43		Unknown	*Homo sapiens*	Lanosterol 14-alpha demethylase	6.30E-03	*Mycobacterium tuberculosis*<br>*Penicillium digitatum*	Lanosterol 14-alpha demethylase<br>Cytochrome P450 14alpha-demethylase	0<br>0.01 to 0	Unknown		Unknown				Unknown	180515183534	14-alpha sterol demethylase<br>14-alpha sterol demethylase Cyp51A<br>Cytochrome P450 51<br>Lanosterol 14-alpha demethylase	Antimicrob. Agents Chemother.,(2010)54:10:4225<br>Antimicrob. Agents Chemother.,(2010)54:10:4225<br>Antimicrob. Agents Chemother.,(2009)53:3:1157; Bioorg. Med. Chem. Lett.,(1994)4:5:733; Bioorg. Med. Chem. Lett.,(1998)8:14:1819; Bioorg. Med. Chem.,(2008)16:1:209; Drug Metab. Dispos.,(2007)35:3:493; J. Med. Chem.,(1992)35:15:2818<br>Antimicrob. Agents Chemother.,(2007)51:11:3915; Antimicrob. Agents Chemother.,(2009)53:3:1157
SSF 109 (huanjunzuo)	CYP51 inhibitor<br>Lanosterol 14 alpha demethylase inhibitor	0.216<br>0.206	0.005<br>0.008	*Penicillium digitatum*	Cytochrome P450 14alpha-demethylase	7.77E-11	0.31		Unknown		Unknown		*Homo sapiens*<br>*Mycobacterium tuberculosis*<br>*Penicillium digitatum*	Cytochrome P450 51<br>Lanosterol 14-alpha demethylase<br>Cytochrome P450 14alpha-demethylase	0.01 to 0<br>0.042<br>0.01 to 0	Unknown		Unknown				Unknown	180515183607	Cytochrome P450 14alpha-demethylase<br>Cytochrome P450 51	J Agric Food Chem,(2009)57:15:6914<br>Drug Metab. Dispos.,(2007)35:3:493; J. Med. Chem.,(2002)45:13:2720
SSY 726	CYP51 inhibitor<br>Lanosterol 14 alpha demethylase inhibitor	0.586<br>0.880	0.002<br>0.001	*Mycobacterium tuberculosis*<br>*Neosartorya fumigata (Aspergillus fumigatus)*<br><br>*Penicillium digitatum*	Lanosterol 14-alpha demethylase<br>14-alpha sterol demethylase<br>14-alpha sterol demethylase Cyp51A<br>Cytochrome P450 14alpha-demethylase	2.28E-09<br>6.31E-29<br>3.20E-24<br>3.41E-32	0.42<br>0.42<br>0.34<br>0.39		Unknown		Unknown		*Mycobacterium tuberculosis*<br>*Penicillium digitatum*	Lanosterol 14-alpha demethylase<br>Cytochrome P450 14alpha-demethylase	0.01 to 0<br>0.108	Unknown		Unknown				Unknown	18047163356	Unknown	
SYN 2506	CYP51 inhibitor<br>Lanosterol 14 alpha demethylase inhibitor	0.720<br>0.945	0.001<br>0.000	*Mycobacterium tuberculosis*<br>*Neosartorya fumigata (Aspergillus fumigatus)*<br><br>*Penicillium digitatum*	Lanosterol 14-alpha demethylase<br>14-alpha sterol demethylase<br>14-alpha sterol demethylase Cyp51A<br>Cytochrome P450 14alpha-demethylase	4.46E-10<br>6.57E-67<br>1.87E-65<br>2.89E-21	0.45<br>0.47<br>0.47<br>0.31	*Homo sapiens*<br>*Neosartorya fumigata*<br>*Neosartorya fumigata (strain ATCC MYA-4609 / Af293 / CBS 101355 / FGSCA1100) (Aspergillus fumigatus)*<br>*Trypanosoma cruzi (strain CL Brener)*	Lanosterol 14-alpha demethylase<br>14-alpha sterol demethylase<br>14-alpha sterol demethylase Cyp51A<br>Sterol 14-alpha demethylase		Unknown		*Mycobacterium tuberculosis*	Lanosterol 14-alpha demethylase	0.061	Unknown		Cytochrome P450 Family 51 Subfamily A Member 1	53.3	0.49		Unknown	180515183708	14-alpha sterol demethylase<br>14-alpha sterol demethylase Cyp51A<br>Sterol 14-alpha demethylase	Antimicrob. Agents Chemother.,(2010)54:10:4225<br>Antimicrob. Agents Chemother.,(2010)54:10:4225<br>Antimicrob. Agents Chemother.,(2010)54:6:2480
SYN 2836	CYP51 inhibitor<br>Lanosterol 14 alpha demethylase inhibitor	0.640<br>0.945	0.001<br>0.000	*Mycobacterium tuberculosis*<br>*Neosartorya fumigata (Aspergillus fumigatus)*	Lanosterol 14-alpha demethylase<br>14-alpha sterol demethylase<br>14-alpha sterol demethylase Cyp51A	2.48E-09<br>7.19E-60<br>7.22E-59	0.41<br>0.46<br>0.46		Unknown		Unknown		*Mycobacterium tuberculosis*	Lanosterol 14-alpha demethylase	0.169	Unknown		Unknown				Unknown	180515183800	14-alpha sterol demethylase<br>14-alpha sterol demethylase Cyp51A<br>Sterol 14-alpha demethylase	Antimicrob. Agents Chemother.,(2010)54:10:4225<br>Antimicrob. Agents Chemother.,(2010)54:10:4225<br>Antimicrob. Agents Chemother.,(2010)54:6:2480
SYN 2869	CYP51 inhibitor<br>Lanosterol 14 alpha demethylase inhibitor	0.620<br>0.966	0.001<br>0.000	*Mycobacterium tuberculosis*<br>*Neosartorya fumigata (Aspergillus fumigatus)*	Lanosterol 14-alpha demethylase<br>14-alpha sterol demethylase<br>14-alpha sterol demethylase Cyp51A	5.30E-09<br>4.45E-60<br>1.59E-58	0.40<br>0.43<br>0.43	*Homo sapiens*<br>*Neosartorya fumigata*<br>*Neosartorya fumigata (strain ATCC MYA-4609 / Af293 / CBS 101355 / FGSCA1100) (Aspergillus fumigatus)*<br>*Trypanosoma cruzi (strain CL Brener)*	Lanosterol 14-alpha demethylase<br>14-alpha sterol demethylase<br>14-alpha sterol demethylase Cyp51A<br>Sterol 14-alpha demethylase		Unknown		*Mycobacterium tuberculosis*	Lanosterol 14-alpha demethylase	0.299	Unknown		Cytochrome P450 Family 51 Subfamily A Member 1	53.3	0.41		Unknown	180515183834	14-alpha sterol demethylase<br>14-alpha sterol demethylase Cyp51A<br>Sterol 14-alpha demethylase	Antimicrob. Agents Chemother.,(2010)54:10:4225<br>Antimicrob. Agents Chemother.,(2010)54:10:4225<br>Antimicrob. Agents Chemother.,(2010)54:6:2480
SYN 2903	CYP51 inhibitor<br>Lanosterol 14 alpha demethylase inhibitor	0.619<br>0.914	0.001<br>0.000	*Mycobacterium tuberculosis*<br>*Neosartorya fumigata (Aspergillus fumigatus)*	Lanosterol 14-alpha demethylase<br>14-alpha sterol demethylase<br>14-alpha sterol demethylase Cyp51A	8.52E-09<br>6.13E-56<br>4.19E-55	0.39<br>0.43<br>0.43		Unknown		Unknown		*Mycobacterium tuberculosis*	Lanosterol 14-alpha demethylase	0.299	Unknown		Unknown				Unknown	180515183908	14-alpha sterol demethylase<br>14-alpha sterol demethylase Cyp51A<br>Sterol 14-alpha demethylase	Antimicrob. Agents Chemother.,(2010)54:10:4225<br>Antimicrob. Agents Chemother.,(2010)54:10:4225<br>Antimicrob. Agents Chemother.,(2010)54:6:2480
SYN 2921	CYP51 inhibitor<br>Lanosterol 14 alpha demethylase inhibitor	0.600<br>0.946	0.002<br>0.000	*Mycobacterium tuberculosis*<br>*Neosartorya fumigata (Aspergillus fumigatus)*	Lanosterol 14-alpha demethylase<br>14-alpha sterol demethylase<br>14-alpha sterol demethylase Cyp51A	1.34E-08<br>6.39E-56<br>1.43E-54	0.38<br>0.41<br>0.41	*Homo sapiens*<br>*Neosartorya fumigata*<br>*Neosartorya fumigata (strain ATCC MYA-4609 / Af293 / CBS 101355 / FGSCA1100) (Aspergillus fumigatus)*<br>*Trypanosoma cruzi (strain CL Brener)*	Lanosterol 14-alpha demethylase<br>14-alpha sterol demethylase<br>14-alpha sterol demethylase Cyp51A<br>Sterol 14-alpha demethylase		Unknown		*Mycobacterium tuberculosis*	Lanosterol 14-alpha demethylase	0.458	Unknown		Unknown				Unknown	180515190551	14-alpha sterol demethylase<br>14-alpha sterol demethylase Cyp51A<br>Sterol 14-alpha demethylase	Antimicrob. Agents Chemother.,(2010)54:10:4225<br>Antimicrob. Agents Chemother.,(2010)54:10:4225<br>Antimicrob. Agents Chemother.,(2010)54:6:2480
T 8581	CYP51 inhibitor<br>Lanosterol 14 alpha demethylase inhibitor	0.600<br>0.601	0.002<br>0.001	*Mycobacterium tuberculosis*<br>*Neosartorya fumigata (Aspergillus fumigatus)*<br><br>*Penicillium digitatum*	Lanosterol 14-alpha demethylase<br>14-alpha sterol demethylase<br>14-alpha sterol demethylase Cyp51A<br>Cytochrome P450 14alpha-demethylase	1.93E-14<br>8.46E-44<br>6.90E-35<br>2.76E-23	0.66<br>0.66<br>0.50<br>0.34		Unknown	*Homo sapiens*	Lanosterol 14-alpha demethylase	1.44E-03	*Homo sapiens*<br>*Mycobacterium tuberculosis*	Cytochrome P450 51<br>Lanosterol 14-alpha demethylase	0.01 to 0<br>0	Unknown		Unknown				Unknown	180515190626	14-alpha sterol demethylase<br>14-alpha sterol demethylase Cyp51A<br>Cytochrome P450 51<br>Lanosterol 14-alpha demethylase	Antimicrob. Agents Chemother.,(2010)54:10:4225<br>Antimicrob. Agents Chemother.,(2010)54:10:4225<br>Antimicrob. Agents Chemother.,(2009)53:3:1157; Bioorg. Med. Chem. Lett.,(1994)4:5:733; Bioorg. Med. Chem. Lett.,(1998)8:14:1819; Bioorg. Med. Chem.,(2008)16:1:209; Drug Metab. Dispos.,(2007)35:3:493<br>Antimicrob. Agents Chemother.,(2007)51:11:3915; Antimicrob. Agents Chemother.,(2009)53:3:1157
TAK 187	CYP51 inhibitor<br>Lanosterol 14 alpha demethylase inhibitor	0.653<br>0.973	0.001<br>0.000	*Mycobacterium tuberculosis*<br>*Neosartorya fumigata (Aspergillus fumigatus)*<br><br>*Penicillium digitatum*	Lanosterol 14-alpha demethylase<br>14-alpha sterol demethylase<br>14-alpha sterol demethylase Cyp51A<br>Cytochrome P450 14alpha-demethylase	3.25E-10<br>2.45E-65<br>1.54E-64<br>4.69E-14	0.46<br>0.50<br>0.50<br>0.29		Unknown		Unknown		*Mycobacterium tuberculosis*	Lanosterol 14-alpha demethylase	0.061	Unknown		Cytochrome P450 Family 51 Subfamily A Member 1	53.3	0.48		Unknown	180515190718	14-alpha sterol demethylase<br>14-alpha sterol demethylase Cyp51A<br>Sterol 14-alpha demethylase	Antimicrob. Agents Chemother.,(2010)54:10:4225<br>Antimicrob. Agents Chemother.,(2010)54:10:4225<br>Antimicrob. Agents Chemother.,(2010)54:6:2480
TAK 456	CYP51 inhibitor<br>Lanosterol 14 alpha demethylase inhibitor	0.526<br>0.520	0.002<br>0.002	*Mycobacterium tuberculosis*<br>*Neosartorya fumigata (Aspergillus fumigatus)*	Lanosterol 14-alpha demethylase<br>14-alpha sterol demethylase<br>14-alpha sterol demethylase Cyp51A	2.35E-10<br>1.80E-47<br>4.98E-55	0.46<br>0.49<br>0.49		Unknown		Unknown		*Mycobacterium tuberculosis*	Lanosterol 14-alpha demethylase	0.032	Unknown		Unknown				Unknown	180515190750	Unknown	
Tebuconazole	CYP51 inhibitor<br>Lanosterol 14 alpha demethylase inhibitor	0.527<br>0.700	0.002<br>0.001	*Homo sapiens*<br>*Mycobacterium tuberculosis*<br>*Neosartorya fumigata (Aspergillus fumigatus)*<br><br>*Penicillium digitatum*	Lanosterol 14-alpha demethylase<br>Lanosterol 14-alpha demethylase<br>14-alpha sterol demethylase<br>14-alpha sterol demethylase Cyp51A<br>Cytochrome P450 14alpha-demethylase	6.42E-06<br>2.67E-09<br>1.26E-28<br>6.81E-24<br>5.69E-96	0.33<br>0.41<br>0.41<br>0.34<br>1.00	*Homo sapiens*	Lanosterol 14-alpha demethylase		Unknown		*Mycobacterium tuberculosis*<br>*Penicillium digitatum*	Lanosterol 14-alpha demethylase<br>Cytochrome P450 14alpha-demethylase	0.01 to 0<br>0	Unknown		Unknown				Unknown	180515190825	Cytochrome P450 14alpha-demethylase<br>Cytochrome P450 51	J Agric Food Chem,(2009)57:15:6914<br>Bioorg. Med. Chem. Lett.,(1994)4:5:733; Bioorg. Med. Chem. Lett.,(1998)8:14:1819
Terconazole (triaconazole)	CYP51 inhibitor<br>Lanosterol 14 alpha demethylase inhibitor	0.931<br>0.968	0.000<br>0.000	*Homo sapiens*<br>*Neosartorya fumigata (Aspergillus fumigatus)*<br><br>*Penicillium digitatum*<br>*Rattus norvegicus*	Lanosterol 14-alpha demethylase<br>14-alpha sterol demethylase<br>14-alpha sterol demethylase Cyp51A<br>Cytochrome P450 14alpha-demethylase<br>Lanosterol 14-alpha demethylase	1.82E-10<br>2.43E-44<br>6.23E-30<br>1.05E-13<br>4.70E-47	0.61<br>0.75<br>0.43<br>0.29<br>0.61	*Homo sapiens*	Lanosterol 14-alpha demethylase		Unknown		*Homo sapiens*	Cytochrome P450 51	0	Unknown		Cytochrome P450 Family 51 Subfamily A Member 1	100	1		Unknown	180515190857	14-alpha sterol demethylase<br>14-alpha sterol demethylase Cyp51A<br>Cytochrome P450 51	Antimicrob. Agents Chemother.,(2010)54:10:4225<br>Antimicrob. Agents Chemother.,(2010)54:10:4225<br>Antimicrob. Agents Chemother.,(2009)53:3:1157; Bioorg. Med. Chem. Lett.,(1994)4:5:733; Bioorg. Med. Chem.,(2008)16:1:209; Drug Metab. Dispos.,(2007)35:3:493; J. Med. Chem.,(1992)35:15:2818; J. Med. Chem.,(1993)36:15:2235; J. Med. Chem.,(2009)52:6:1639
Tetraconazole	CYP51 inhibitor<br>Lanosterol 14 alpha demethylase inhibitor	0.507<br>0.484	0.002<br>0.002	*Homo sapiens*<br>*Mycobacterium tuberculosis*<br>*Neosartorya fumigata (Aspergillus fumigatus)*<br><br>*Penicillium digitatum*	Lanosterol 14-alpha demethylase<br>Lanosterol 14-alpha demethylase<br>14-alpha sterol demethylase<br>14-alpha sterol demethylase Cyp51A<br>Cytochrome P450 14alpha-demethylase	2.70E-11<br>1.68E-21<br>1.09E-23<br>3.59E-20<br>2.56E-40	0.33<br>0.33<br>0.33<br>0.28<br>0.35		Unknown		Unknown		*Homo sapiens*<br>*Mycobacterium tuberculosis*<br>*Penicillium digitatum*	Cytochrome P450 51<br>Lanosterol 14-alpha demethylase<br>Cytochrome P450 14alpha-demethylase	0.01 to 0<br>0.236<br>0.01 to 0	Unknown		Unknown				Unknown	180515190926	Lanosterol 14-alpha demethylase	J Agric Food Chem,(2000)48:6:2547
Triadimefon	CYP51 inhibitor<br>Lanosterol 14 alpha demethylase inhibitor	0.497<br>0.508	0.002<br>0.002	*Penicillium digitatum*	Cytochrome P450 14alpha-demethylase	7.06E-45	1.00	*Homo sapiens*	Lanosterol 14-alpha demethylase		Unknown		*Penicillium digitatum*	Cytochrome P450 14alpha-demethylase	0	Unknown		Unknown				Unknown	180515190953	Cytochrome P450 14alpha-demethylase<br>P450 sterol 14-alpha-demethylase	J Agric Food Chem,(2009)57:15:6914; Pest Manag Sci,(2010)66:12:1344<br>J Agric Food Chem,(2013)61:7:1419
Triadimenol	CYP51 inhibitor<br>Lanosterol 14 alpha demethylase inhibitor	0.575<br>0.692	0.002<br>0.001	*Penicillium digitatum*	Cytochrome P450 14alpha-demethylase	2.76E-54	1.00	*Homo sapiens*	Lanosterol 14-alpha demethylase		Unknown		*Penicillium digitatum*	Cytochrome P450 14alpha-demethylase	0	Unknown		Unknown				Unknown	180515191030	Cytochrome P450 14alpha-demethylase<br>P450 sterol 14-alpha-demethylase	J Agric Food Chem,(2009)57:15:6914; Pest Manag Sci,(2010)66:12:1344<br>J Agric Food Chem,(2013)61:7:1419
Triticonazole	CYP51 inhibitor<br>Lanosterol 14 alpha demethylase inhibitor	0.532<br>0.373	0.002<br>0.003	*Penicillium digitatum*	Cytochrome P450 14alpha-demethylase	8.83E-33	0.38		Unknown		Unknown		*Homo sapiens*<br>*Penicillium digitatum*	Cytochrome P450 51<br>Cytochrome P450 14alpha-demethylase	0.01 to 0<br>0.01 to 0	Unknown		Unknown				Unknown	180515194944	Cytochrome P450 14alpha-demethylase	J Agric Food Chem,(2009)57:15:6914; Pest Manag Sci,(2010)66:12:1344
UK 47265	CYP51 inhibitor<br>Lanosterol 14 alpha demethylase inhibitor	0.704<br>0.889	0.001<br>0.001	*Mycobacterium tuberculosis*<br>*Neosartorya fumigata (Aspergillus fumigatus)*<br><br>*Penicillium digitatum*	Lanosterol 14-alpha demethylase<br>14-alpha sterol demethylase<br>14-alpha sterol demethylase Cyp51A<br>Cytochrome P450 14alpha-demethylase	1.34E-13<br>7.68E-39<br>1.53E-28<br>3.09E-73	0.62<br>0.62<br>0.41<br>0.50	*Homo sapiens*<br>*Mycobacterium tuberculosis*<br>*Neosartorya fumigata*<br>*Neosartorya fumigata (strain ATCC MYA-4609 / Af293 / CBS 101355 / FGSCA1100) (Aspergillus fumigatus)*	Lanosterol 14-alpha demethylase<br>Lanosterol 14-alpha demethylase<br>14-alpha demethylase<br>14-alpha sterol demethylase Cyp51A	*Homo sapiens*	Lanosterol 14-alpha demethylase		*Mycobacterium tuberculosis*	Lanosterol 14-alpha demethylase	0	Unknown		Unknown				Unknown	180515195013	14-alpha sterol demethylase<br>14-alpha sterol demethylase Cyp51A<br>Cytochrome P450 14alpha-demethylase<br>Cytochrome P450 51<br>Lanosterol 14-alpha demethylase	Antimicrob. Agents Chemother.,(2010)54:10:4225<br>Antimicrob. Agents Chemother.,(2010)54:10:4225<br>J Agric Food Chem,(2009)57:15:6914<br>Antimicrob. Agents Chemother.,(2009)53:3:1157; Bioorg. Med. Chem. Lett.,(1994)4:5:733; Bioorg. Med. Chem. Lett.,(1998)8:14:1819; Bioorg. Med. Chem.,(2008)16:1:209; Drug Metab. Dispos.,(2007)35:3:493<br>Antimicrob. Agents Chemother.,(2007)51:11:3915; Antimicrob. Agents Chemother.,(2009)53:3:1157; J Agric Food Chem,(2000)48:6:2547; J Agric Food Chem,(2007)55:20:8187
UK 51486	CYP51 inhibitor<br>Lanosterol 14 alpha demethylase inhibitor	0.601<br>0.631	0.002<br>0.001	*Mycobacterium tuberculosis*<br>*Neosartorya fumigata (Aspergillus fumigatus)*<br><br>*Penicillium digitatum*	Lanosterol 14-alpha demethylase<br>14-alpha sterol demethylase<br>14-alpha sterol demethylase Cyp51A<br>Cytochrome P450 14alpha-demethylase	2.47E-11<br>8.53E-33<br>7.04E-25<br>3.32E-31	0.51<br>0.51<br>0.35<br>0.42		Unknown	*Homo sapiens*	Lanosterol 14-alpha demethylase	3.27E-02	*Homo sapiens*<br>*Mycobacterium tuberculosis*<br>*Penicillium digitatum*	Cytochrome P450 51<br>Lanosterol 14-alpha demethylase<br>Cytochrome P450 14alpha-demethylase	0.01 to 0<br>0.01 to 0<br>0.014	Unknown		Unknown				Unknown	180515195044	Cytochrome P450 14alpha-demethylase<br>Cytochrome P450 51	J Agric Food Chem,(2009)57:15:6914<br>Drug Metab. Dispos.,(2007)35:3:493

Bioactive molecule	PASS online[4]			Similarity Ensemble Approach (SEA)[2]				ChemProt[3]		SuperPred[6]		
	Activity	Pa	Pi	Organism	Target	P-Value	Max TC	Organism	Target	Organism	Target	E-value
Uniconazole	CYP51 inhibitor	0.612	0.001	*Penicillium digitatum*	Cytochrome P450 14alpha-demethylase	1.90E-37	0.70		Unknown		Unknown	
	Lanosterol 14 alpha demethylase inhibitor	0.582	0.002									
UR 9746	CYP51 inhibitor	0.277	0.004	*Mycobacterium tuberculosis*	Lanosterol 14-alpha demethylase	7.32E-08	0.34		Unknown		Unknown	
	Lanosterol 14 alpha demethylase inhibitor	0.440	0.002	*Neosartorya fumigata (Aspergillus fumigatus)*	14-alpha sterol demethylase	1.06E-49	0.37					
					14-alpha sterol demethylase Cyp51A	1.66E-47	0.37					
UR 9751	CYP51 inhibitor	0.332	0.004	*Mycobacterium tuberculosis*	Lanosterol 14-alpha demethylase	5.83E-08	0.35		Unknown		Unknown	
	Lanosterol 14 alpha demethylase inhibitor	0.642	0.001	*Neosartorya fumigata (Aspergillus fumigatus)*	14-alpha sterol demethylase	9.71E-51	0.39					
					14-alpha sterol demethylase Cyp51A	6.58E-49	0.39					
Vibunazole (BAY N-7133)	CYP51 inhibitor	0.517	0.002	*Mycobacterium tuberculosis*	Lanosterol 14-alpha demethylase	3.90E-09	0.40		Unknown		Unknown	
	Lanosterol 14 alpha demethylase inhibitor	0.868	0.001	*Neosartorya fumigata (Aspergillus fumigatus)*	14-alpha sterol demethylase	1.71E-39	0.40					
					14-alpha sterol demethylase Cyp51A	6.11E-25	0.35					
				Penicillium digitatum	Cytochrome P450 14alpha-demethylase	3.89E-96	0.67					
Voriconazole	CYP51 inhibitor	0.609	0.001	*Mycobacterium tuberculosis*	Lanosterol 14-alpha demethylase	4.00E-13	0.60	*Homo sapiens*	Lanosterol 14-alpha demethylase	*Homo sapiens*	Lanosterol 14-alpha demethylase	8.23E-03
	Lanosterol 14 alpha demethylase inhibitor	0.941	0.000	*Neosartorya fumigata (Aspergillus fumigatus)*	14-alpha sterol demethylase	9.85E-71	1.00	*Mycobacterium tuberculosis*	Lanosterol 14-alpha demethylase			
					14-alpha sterol demethylase Cyp51A	1.18E-88	1.00	*Neosartorya fumigata*	14-alpha demethylase			
				Penicillium digitatum	Cytochrome P450 14alpha-demethylase	3.74E-24	0.35	*Neosartorya fumigata (strain ATCC MYA-4609 / Af293 / CBS 101355 / FGSCA1100) (Aspergillus fumigatus)*	14-alpha sterol demethylase Cyp51A			
YH 1715R	CYP51 inhibitor	0.652	0.001	*Mycobacterium tuberculosis*	Lanosterol 14-alpha demethylase	2.67E-11	0.51		Unknown	*Homo sapiens*	Lanosterol 14-alpha demethylase	4.39E-02
	Lanosterol 14 alpha demethylase inhibitor	0.568	0.002	*Neosartorya fumigata (Aspergillus fumigatus)*	14-alpha sterol demethylase	3.02E-39	0.53					
					14-alpha sterol demethylase Cyp51A	7.83E-37	0.53					
				Penicillium digitatum	Cytochrome P450 14alpha-demethylase	6.08E-38	0.31					

Bioactive molecule	Polypharmacology Browser (PPB)[1]			SPiDER[7]		HitPick[5]			PharmMapper[9]			TargetHunter[8]	
	Organism	Target	P-Value	Target	Confidence level	Target	Precision (%)	Tc similarity	Organism	Target	Job Id	Target	Reference
Uniconazole	*Penicillium digitatum*	Cytochrome P450 14alpha-demethylase	0	Unknown		Unknown				Unknown	180515195106	Cytochrome P450 14alpha-demethylase	J Agric Food Chem,(2009)57:15:6914; Pest Manag Sci,(2010)66:12:1344
UR 9746	*Mycobacterium tuberculosis*	Lanosterol 14-alpha demethylase	0.446	Unknown		Unknown				Unknown	180515195130	Unknown	
UR 9751	*Homo sapiens*	Cytochrome P450 51	0.102	Unknown		Unknown				Unknown	180515195159	Unknown	
	Mycobacterium tuberculosis	Lanosterol 14-alpha demethylase	0.281										
Vibunazole (BAY N-7133)	*Penicillium digitatum*	Cytochrome P450 14alpha-demethylase	0	Unknown		Unknown				Unknown	180515195223	Cytochrome P450 14alpha-demethylase	J Agric Food Chem,(2009)57:15:6914
												Cytochrome P450 51	Bioorg. Med. Chem. Lett.,(1998)8:14:1819
Voriconazole		Unknown		Unknown		Cytochrome P450 Family 51 Subfamily A Member 1	100	1		Unknown	180515195244	14-alpha sterol demethylase	Antimicrob. Agents Chemother.,(2010)54:10:4225
												14-alpha sterol demethylase Cyp51A	Antimicrob. Agents Chemother.,(2010)54:10:4225
YH 1715R	*Mycobacterium tuberculosis*	Lanosterol 14-alpha demethylase	0.01 to 0	Unknown		Unknown				Unknown	180515195333	Unknown	

Bioactive molecule	PASS online[4]			Similarity Ensemble Approach (SEA)[2]				ChemProt[3]		SuperPred[6]			Polypharmacology Browser (PPB)[1]			SPiDER[7]		HitPick[5]			PharmMapper[9]			TargetHunter[8]	
	Activity	Pa	Pi	Organism	Target	P-Value	Max TC	Organism	Target	Organism	Target	E-value	Organism	Target	P-Value	Target	Confidence level	Target	Precision (%)	Tc similarity	Organism	Target	Job Id	Target	Reference
1-Dodecylimidazole (N-dodecylimidazole)	CYP51 inhibitor<br>Lanosterol 14 alpha demethylase inhibitor	0.526<br>0.493	0.002<br>0.002		Unknown				Unknown		Unknown			Unknown		Unknown		Unknown				Unknown	180516112805	Unknown	
AFK 108	CYP51 inhibitor<br>Lanosterol 14 alpha demethylase inhibitor	0.683<br>0.707	0.001<br>0.001	*Homo sapiens*<br>*Mycobacterium tuberculosis*<br>*Rattus norvegicus*	Lanosterol 14-alpha demethylase<br>Lanosterol 14-alpha demethylase<br>Lanosterol 14-alpha demethylase	2.24E-21<br>2.93E-24<br>8.90E-23	0.59<br>0.59<br>0.31		Unknown	*Homo sapiens*	Lanosterol 14-alpha demethylase	8.20E-07	*Homo sapiens*<br>*Mycobacterium tuberculosis*	Cytochrome P450 51<br>Lanosterol 14-alpha demethylase	0<br>0	Unknown		Unknown				Unknown	180516113356	Unknown	
Aliconazole	CYP51 inhibitor<br>Lanosterol 14 alpha demethylase inhibitor	0.524<br>0.882	0.002<br>0.001	*Homo sapiens*<br>*Mycobacterium tuberculosis*<br>*Rattus norvegicus*	Lanosterol 14-alpha demethylase<br>Lanosterol 14-alpha demethylase<br>Lanosterol 14-alpha demethylase	1.11E-16<br>1.90E-17<br>5.17E-24	0.42<br>0.42<br>0.33		Unknown		Unknown			Unknown		Unknown		Cytochrome P450 Family 51 Subfamily A Member 1	53.3	0.46		Unknown	180331151609	Unknown	
Arasertaconazole	CYP51 inhibitor<br>Lanosterol 14 alpha demethylase inhibitor	0.774<br>0.951	0.001<br>0.000	*Homo sapiens*<br>*Mycobacterium tuberculosis*	Lanosterol 14-alpha demethylase<br>Lanosterol 14-alpha demethylase	7.49E-19<br>9.92E-27	0.68<br>0.68	*Homo sapiens*<br>*Mycobacterium tuberculosis*	Lanosterol 14-alpha demethylase<br>Lanosterol 14-alpha demethylase	*Homo sapiens*	Lanosterol 14-alpha demethylase	6.86E-07	*Homo sapiens*<br>*Mycobacterium tuberculosis*	Cytochrome P450 51<br>Lanosterol 14-alpha demethylase	0<br>0	Unknown		Cytochrome P450 Family 51 Subfamily A Member 1	100	1		Unknown	180516113435	Cytochrome P450 51<br>Lanosterol 14-alpha demethylase	Drug Metab. Dispos.,(2007)35:3:493<br>Antimicrob. Agents Chemother.,(2009)53:3:1157
Azalanstat	CYP51 inhibitor<br>Lanosterol 14 alpha demethylase inhibitor	0.945<br>0.927	0.000<br>0.000	*Homo sapiens*<br>*Mycobacterium tuberculosis*<br>*Rattus norvegicus*	Lanosterol 14-alpha demethylase<br>Lanosterol 14-alpha demethylase<br>Lanosterol 14-alpha demethylase	2.10E-23<br>1.62E-06<br>2.60E-97	1.00<br>0.29<br>1.00	*Homo sapiens*<br>*Rattus norvegicus*	Lanosterol 14-alpha demethylase<br>Lanosterol 14-alpha demethylase		Unknown		*Homo sapiens*<br>*Penicillium digitatum*	Cytochrome P450 51<br>Cytochrome P450 14alpha-demethylase	0<br>0.35	Unknown		Cytochrome P450 Family 51 Subfamily A Member 1	100	1		Unknown	180516113605	Cytochrome P450 51	J. Med. Chem.,(1993)36:15:2235
BAY C-9263	CYP51 inhibitor<br>Lanosterol 14 alpha demethylase inhibitor	0.252<br>0.203	0.004<br>0.008	*Homo sapiens*<br>*Mycobacterium tuberculosis*<br>*Neosartorya fumigate (Aspergillus fumigatus)*<br>*Sorghum bicolor (Sorghum vulgare)*	Lanosterol 14-alpha demethylase<br>Lanosterol 14-alpha demethylase<br>14-alpha sterol demethylase<br>14-alpha sterol demethylase Cyp51A<br>Obtusifoliol 14-alpha demethylase	7.53E-11<br>3.30E-09<br>1.33E-15<br>6.61E-18<br>3.23E-12	0.43<br>0.43<br>0.43<br>0.43<br>0.28		Unknown		Unknown		*Homo sapiens*<br>*Mycobacterium tuberculosis*	Cytochrome P450 51<br>Lanosterol 14-alpha demethylase	0<br>0.01 to 0	Unknown		Unknown				Unknown	180516112923	Cytochrome P450 51	Drug Metab. Dispos.,(2007)35:3:493; J. Med. Chem.,(2002)45:13:2720
BAY D-9603	CYP51 inhibitor<br>Lanosterol 14 alpha demethylase inhibitor	0.344<br>0.211	0.003<br>0.007	*Homo sapiens*<br>*Mycobacterium tuberculosis*<br>*Neosartorya fumigate (Aspergillus fumigatus)*	Lanosterol 14-alpha demethylase<br>Lanosterol 14-alpha demethylase<br>14-alpha sterol demethylase<br>14-alpha sterol demethylase Cyp51A	6.27E-13<br>5.99E-12<br>1.98E-20<br>1.85E-23	0.56<br>0.56<br>0.56<br>0.56		Unknown		Unknown		*Homo sapiens*<br>*Mycobacterium tuberculosis*	Cytochrome P450 51<br>Lanosterol 14-alpha demethylase	0<br>0	Unknown		Unknown				Unknown	180516113025	14-alpha sterol demethylase<br>14-alpha sterol demethylase Cyp51A<br>Cytochrome P450 51<br>Lanosterol 14-alpha demethylase	Antimicrob. Agents Chemother.,(2010)54:10:4225<br>Antimicrob. Agents Chemother.,(2010)54:10:4225<br>Drug Metab. Dispos.,(2007)35:3:493; J. Med. Chem.,(2002)45:13:2720<br>Antimicrob. Agents Chemother.,(2009)53:3:1157
Becliconazole	CYP51 inhibitor<br>Lanosterol 14 alpha demethylase inhibitor	0.568<br>0.816	0.002<br>0.001	*Homo sapiens*<br>*Mycobacterium tuberculosis*<br>*Neosartorya fumigate (Aspergillus fumigatus)*	Lanosterol 14-alpha demethylase<br>Lanosterol 14-alpha demethylase<br>14-alpha sterol demethylase<br>14-alpha sterol demethylase Cyp51A	3.07E-18<br>9.22E-19<br>5.10E-11<br>1.34E-12	0.36<br>0.31<br>0.29<br>0.29		Unknown		Unknown		*Homo sapiens*<br>*Mycobacterium tuberculosis*	Cytochrome P450 51<br>Lanosterol 14-alpha demethylase	0.01 to 0<br>0.141	Unknown		Unknown				Unknown	18047170728	14-alpha sterol demethylase<br>14-alpha sterol demethylase Cyp51A<br>Cytochrome P450 51<br>Lanosterol 14-alpha demethylase	Antimicrob. Agents Chemother.,(2010)54:10:4225<br>Antimicrob. Agents Chemother.,(2010)54:10:4225<br>Drug Metab. Dispos.,(2007)35:3:493<br>Antimicrob. Agents Chemother.,(2009)53:3:1157
Bifonazole	CYP51 inhibitor<br>Lanosterol 14 alpha demethylase inhibitor	0.751<br>0.585	0.001<br>0.002	*Homo sapiens*<br>*Mycobacterium tuberculosis*<br>*Neosartorya fumigate (Aspergillus fumigatus)*	Lanosterol 14-alpha demethylase<br>Lanosterol 14-alpha demethylase<br>14-alpha sterol demethylase<br>14-alpha sterol demethylase Cyp51A	1.15E-18<br>1.11E-06<br>3.48E-11<br>8.64E-13	1.00<br>0.30<br>0.30<br>0.30	*Homo sapiens*	Lanosterol 14-alpha demethylase		Unknown		*Homo sapiens*<br>*Mycobacterium tuberculosis*	Cytochrome P450 51<br>Lanosterol 14-alpha demethylase	0<br>0.01 to 0	Unknown		Unknown				Unknown	180516113129	14-alpha sterol demethylase<br>14-alpha sterol demethylase Cyp51A<br>Cytochrome P450 51<br>Lanosterol 14-alpha demethylase	Antimicrob. Agents Chemother.,(2010)54:10:4225<br>Antimicrob. Agents Chemother.,(2010)54:10:4225<br>Drug Metab. Dispos.,(2007)35:3:493; J. Med. Chem.,(2002)45:13:2720<br>Antimicrob. Agents Chemother.,(2009)53:3:1157
Brolaconazole	CYP51 inhibitor<br>Lanosterol 14 alpha demethylase inhibitor	0.378<br>0.605	0.003<br>0.001	*Homo sapiens*<br>*Mycobacterium tuberculosis*	Lanosterol 14-alpha demethylase<br>Lanosterol 14-alpha demethylase	4.89E-21<br>1.66E-13	0.42<br>0.32		Unknown		Unknown		*Homo sapiens*	Cytochrome P450 51	0	Unknown		Cytochrome P450 Family 51 Subfamily A Member 1	53.3	0.42		Unknown	180516113256	Cytochrome P450 51	Drug Metab. Dispos.,(2007)35:3:493; J. Med. Chem.,(2002)45:13:2720
Butoconazole	CYP51 inhibitor<br>Lanosterol 14 alpha demethylase inhibitor	0.398<br>0.872	0.003<br>0.001	*Homo sapiens*<br>*Mycobacterium tuberculosis*<br>*Rattus norvegicus*	Lanosterol 14-alpha demethylase<br>Lanosterol 14-alpha demethylase<br>Lanosterol 14-alpha demethylase	1.65E-24<br>1.11E-16<br>6.27E-25	0.44<br>0.44<br>0.34		Unknown		Unknown			Unknown		Unknown		Unknown				Unknown	18047170759	Unknown	
Cisconazole	CYP51 inhibitor<br>Lanosterol 14 alpha demethylase inhibitor	0.268<br>0.321	0.004<br>0.003		Unknown				Unknown		Unknown			Unknown		Unknown		Unknown				Unknown	180516113703	Unknown	
Climbazole	CYP51 inhibitor<br>Lanosterol 14 alpha demethylase inhibitor	0.314<br>0.575	0.004<br>0.002	*Homo sapiens*<br>*Mycobacterium tuberculosis*<br>*Penicillium digitatum*<br>*Rattus norvegicus*	Lanosterol 14-alpha demethylase<br>Lanosterol 14-alpha demethylase<br>Cytochrome P450 14alpha-demethylase<br>Lanosterol 14-alpha demethylase	1.22E-12<br>1.92E-06<br>3.21E-12<br>1.05E-21	0.29<br>0.29<br>0.62<br>0.29	*Homo sapiens*	Lanosterol 14-alpha demethylase		Unknown		*Penicillium digitatum*	Cytochrome P450 14alpha-demethylase	0	Unknown		Unknown				Unknown	180331151642	Cytochrome P450 14alpha-demethylase<br>Cytochrome P450 14alpha-demethylase<br>P450 sterol 14-alpha-demethylase	J Agric Food Chem,(2009)57:15:6914<br>Pest Manag Sci,(2010)66:12:1344<br>J Agric Food Chem,(2013)61:7:1419
Clotrimazole	CYP51 inhibitor<br>Lanosterol 14 alpha demethylase inhibitor	0.979<br>0.751	0.000<br>0.001	*Homo sapiens*<br>*Mycobacterium tuberculosis*<br>*Neosartorya fumigate (Aspergillus fumigatus)*	Lanosterol 14-alpha demethylase<br>Lanosterol 14-alpha demethylase<br>14-alpha sterol demethylase<br>14-alpha sterol demethylase Cyp51A	1.15E-18<br>1.43E-20<br>1.45E-35<br>6.24E-41	1.00<br>1.00<br>1.00<br>1.00	*Homo sapiens*<br>*Mycobacterium tuberculosis*<br>*Neosartorya fumigata*<br>*Neosartorya fumigata (strain ATCC MYA-4609 / Af293 / CBS 101355 / FGSCA1100) (Aspergillus fumigatus)*	Lanosterol 14-alpha demethylase<br>Lanosterol 14-alpha demethylase<br>14-alpha demethylase<br>14-alpha sterol demethylase Cyp51A	*Homo sapiens*	Lanosterol 14-alpha demethylase	6.97E-07	*Homo sapiens*<br>*Mycobacterium tuberculosis*	Cytochrome P450 51<br>Lanosterol 14-alpha demethylase	0<br>0	Unknown		Cytochrome P450 Family 51 Subfamily A Member 1	100	1		Unknown	180331151709	14-alpha sterol demethylase<br>14-alpha sterol demethylase Cyp51A<br>Lanosterol 14-alpha demethylase	Antimicrob. Agents Chemother.,(2010)54:10:4225<br>Antimicrob. Agents Chemother.,(2010)54:10:4225<br>Antimicrob. Agents Chemother.,(2009)53:3:1157
Croconazole	CYP51 inhibitor<br>Lanosterol 14 alpha demethylase inhibitor	0.295<br>0.802	0.004<br>0.001	*Mycobacterium tuberculosis*	Lanosterol 14-alpha demethylase				Unknown		Unknown			Unknown		Unknown		Unknown				Unknown	18047170829	Unknown	
Democonazole	CYP51 inhibitor<br>Lanosterol 14 alpha demethylase inhibitor	0.369<br>0.652	0.003<br>0.001	*Homo sapiens*<br>*Mycobacterium tuberculosis*<br>*Rattus norvegicus*	Lanosterol 14-alpha demethylase<br>Lanosterol 14-alpha demethylase<br>Lanosterol 14-alpha demethylase	5.19E-14<br>1.39E-13<br>3.18E-23	0.33<br>0.33<br>0.31		Unknown		Unknown		*Homo sapiens*<br>*Mycobacterium tuberculosis*	Cytochrome P450 51<br>Lanosterol 14-alpha demethylase	0.01 to 0<br>0.01 to 0	Unknown		Unknown				Unknown	18047170932	Unknown	
Dichlorophenyl imidazoldioxolan (elubiol)	CYP51 inhibitor<br>Lanosterol 14 alpha demethylase inhibitor	0.871<br>0.945	0.000<br>0.000	*Homo sapiens*<br>*Mycobacterium tuberculosis*<br>*Neosartorya fumigate (Aspergillus fumigatus)*<br>*Rattus norvegicus*	Lanosterol 14-alpha demethylase<br>Lanosterol 14-alpha demethylase<br>14-alpha sterol demethylase<br>14-alpha sterol demethylase Cyp51A<br>Lanosterol 14-alpha demethylase	4.32E-37<br>2.39E-06<br>5.50E-31<br>1.20E-35<br>2.54E-88	0.88<br>0.28<br>0.55<br>0.55<br>0.88	*Homo sapiens*<br>*Rattus norvegicus*<br>*Neosartorya fumigata*<br>*Neosartorya fumigata (strain ATCC MYA-4609 / Af293 / CBS 101355 / FGSCA1100) (Aspergillus fumigatus)*	Lanosterol 14-alpha demethylase<br>Lanosterol 14-alpha demethylase<br>14-alpha demethylase<br>14-alpha sterol demethylase Cyp51A	*Homo sapiens*	Lanosterol 14-alpha demethylase	2.10E-08	*Homo sapiens*	Cytochrome P450 51	0	Unknown		Cytochrome P450 Family 51 Subfamily A Member 1	96.7	0.83		Unknown	180516120106	Cytochrome P450 51<br>Lanosterol 14-alpha demethylase	Antimicrob. Agents Chemother.,(2009)53:3:1157; Bioorg. Med. Chem.,(2008)16:1:209; Drug Metab. Dispos.,(2007)35:3:493; J. Med. Chem.,(1992)35:15:2818; J. Med. Chem.,(1993)36:15:2235; J. Med. Chem.,(2009)52:6:1639<br>Antimicrob. Agents Chemother.,(2009)53:3:1157
Doconazole	CYP51 inhibitor<br>Lanosterol 14 alpha demethylase inhibitor	0.940<br>0.946	0.000<br>0.000	*Homo sapiens*<br>*Mycobacterium tuberculosis*<br>*Neosartorya fumigate (Aspergillus fumigatus)*<br>*Rattus norvegicus*	Lanosterol 14-alpha demethylase<br>Lanosterol 14-alpha demethylase<br>14-alpha sterol demethylase<br>14-alpha sterol demethylase Cyp51A<br>Lanosterol 14-alpha demethylase	7.44E-38<br>1.83E-13<br>3.47E-19<br>3.47E-19<br>2.51E-77	0.70<br>0.33<br>0.46<br>0.46<br>0.70		Unknown	*Homo sapiens*	Lanosterol 14-alpha demethylase	7.11E-03	*Homo sapiens*	Cytochrome P450 51	0	Unknown		Cytochrome P450 Family 51 Subfamily A Member 1	89.8	0.66		Unknown	180516120140	Cytochrome P450 51<br>Lanosterol 14-alpha demethylase	Antimicrob. Agents Chemother.,(2009)53:3:1157; Bioorg. Med. Chem.,(2008)16:1:209; Drug Metab. Dispos.,(2007)35:3:493; J. Med. Chem.,(1992)35:15:2818; J. Med. Chem.,(1993)36:15:2235; J. Med. Chem.,(2009)52:6:1639<br>Antimicrob. Agents Chemother.,(2009)53:3:1157
Eberconazole	CYP51 inhibitor<br>Lanosterol 14 alpha demethylase inhibitor	0.203<br>0.669	0.005<br>0.001	*Mycobacterium tuberculosis*<br>*Neosartorya fumigate (Aspergillus fumigatus)*	Lanosterol 14-alpha demethylase<br>14-alpha sterol demethylase<br>14-alpha sterol demethylase Cyp51A	2.42E-06<br>4.21E-12<br>4.21E-12	0.28<br>0.28<br>0.28		Unknown		Unknown		*Homo sapiens*<br>*Mycobacterium tuberculosis*	Cytochrome P450 51<br>Lanosterol 14-alpha demethylase	0.01 to 0<br>0.01 to 0	Unknown		Unknown				Unknown	18047171013	14-alpha sterol demethylase<br>14-alpha sterol demethylase Cyp51A<br>Cytochrome P450 51<br>Lanosterol 14-alpha demethylase	Antimicrob. Agents Chemother.,(2010)54:10:4225<br>Antimicrob. Agents Chemother.,(2010)54:10:4225<br>Drug Metab. Dispos.,(2007)35:3:493<br>Antimicrob. Agents Chemother.,(2009)53:3:1157
Econazole	CYP51 inhibitor<br>Lanosterol 14 alpha demethylase inhibitor	0.896<br>0.914	0.000<br>0.000	*Homo sapiens*<br>*Mycobacterium tuberculosis*<br>*Rattus norvegicus*	Lanosterol 14-alpha demethylase<br>Lanosterol 14-alpha demethylase<br>Lanosterol 14-alpha demethylase	1.34E-37<br>3.83E-36<br>1.00E-42	1.00<br>1.00<br>0.30	*Homo sapiens*<br>*Mycobacterium tuberculosis*	Lanosterol 14-alpha demethylase<br>Lanosterol 14-alpha demethylase	*Homo sapiens*	Lanosterol 14-alpha demethylase	5.96E-10	*Homo sapiens*<br>*Mycobacterium tuberculosis*	Cytochrome P450 51<br>Lanosterol 14-alpha demethylase	0<br>0	Unknown		Cytochrome P450 Family 51 Subfamily A Member 1	100	1		Unknown	180516120214	Cytochrome P450 51<br>Lanosterol 14-alpha demethylase<br>Sterol 14-alpha demethylase	Drug Metab. Dispos.,(2007)35:3:493<br>Antimicrob. Agents Chemother.,(2009)53:3:1157<br>J. Med. Chem.,(2014)57:15:6704
Fenapanil	CYP51 inhibitor<br>Lanosterol 14 alpha demethylase inhibitor	0.316<br>0.314	0.004<br>0.003	*Mycobacterium tuberculosis*<br>*Neosartorya fumigate (Aspergillus fumigatus)*	Lanosterol 14-alpha demethylase<br>14-alpha sterol demethylase<br>14-alpha sterol demethylase Cyp51A	6.63E-07<br>1.42E-11<br>3.07E-13	0.31<br>0.31<br>0.31		Unknown		Unknown		*Penicillium digitatum*	Cytochrome P450 14alpha-demethylase	0.01 to 0	Unknown		Unknown				Unknown	180331151746	Cytochrome P450 51	Bioorg. Med. Chem. Lett.,(1994)4:5:733
Fenticonazole	CYP51 inhibitor<br>Lanosterol 14 alpha demethylase inhibitor	0.837<br>0.918	0.001<br>0.000	*Homo sapiens*<br>*Mycobacterium tuberculosis*<br>*Rattus norvegicus*	Lanosterol 14-alpha demethylase<br>Lanosterol 14-alpha demethylase<br>Lanosterol 14-alpha demethylase	3.56E-25<br>5.33E-30<br>1.05E-21	0.80<br>0.80<br>0.29	*Homo sapiens*<br>*Mycobacterium tuberculosis*	Lanosterol 14-alpha demethylase<br>Lanosterol 14-alpha demethylase	*Homo sapiens*	Lanosterol 14-alpha demethylase	7.86E-08	*Homo sapiens*<br>*Mycobacterium tuberculosis*	Cytochrome P450 51<br>Lanosterol 14-alpha demethylase	0<br>0	Unknown		Cytochrome P450 Family 51 Subfamily A Member 1	94.8	0.75		Unknown	180516120253	Cytochrome P450 51<br>Lanosterol 14-alpha demethylase	Drug Metab. Dispos.,(2007)35:3:493<br>Antimicrob. Agents Chemother.,(2009)53:3:1157
Flutrimazole	CYP51 inhibitor<br>Lanosterol 14 alpha demethylase inhibitor	0.937<br>0.726	0.000<br>0.001	*Homo sapiens*<br>*Mycobacterium tuberculosis*<br>*Neosartorya fumigate (Aspergillus fumigatus)*	Lanosterol 14-alpha demethylase<br>Lanosterol 14-alpha demethylase<br>14-alpha sterol demethylase<br>14-alpha sterol demethylase Cyp51A	7.69E-10<br>8.12E-14<br>1.04E-23<br>3.03E-27	0.66<br>0.66<br>0.66<br>0.66	*Homo sapiens*<br>*Mycobacterium tuberculosis*<br>*Neosartorya fumigata*<br>*Neosartorya fumigata (strain ATCC MYA-4609 / Af293 / CBS 101355 / FGSCA1100) (Aspergillus fumigatus)*	Lanosterol 14-alpha demethylase<br>Lanosterol 14-alpha demethylase<br>14-alpha demethylase<br>14-alpha sterol demethylase Cyp51A	*Homo sapiens*	Lanosterol 14-alpha demethylase	2.62E-03	*Homo sapiens*<br>*Mycobacterium tuberculosis*	Cytochrome P450 51<br>Lanosterol 14-alpha demethylase	0<br>0	Unknown		Cytochrome P450 Family 51 Subfamily A Member 1	97.7	0.9		Unknown	18047171059	14-alpha sterol demethylase<br>14-alpha sterol demethylase Cyp51A<br>Cytochrome P450 51<br>Lanosterol 14-alpha demethylase	Antimicrob. Agents Chemother.,(2010)54:10:4225<br>Antimicrob. Agents Chemother.,(2010)54:10:4225<br>Drug Metab. Dispos.,(2007)35:3:493; J. Med. Chem.,(2002)45:13:2720<br>Antimicrob. Agents Chemother.,(2009)53:3:1157
Imazalil (enilconazole)	CYP51 inhibitor<br>Lanosterol 14 alpha demethylase inhibitor	0.744<br>0.926	0.001<br>0.000	*Homo sapiens*<br>*Mycobacterium tuberculosis*<br>*Rattus norvegicus*	Lanosterol 14-alpha demethylase<br>Lanosterol 14-alpha demethylase<br>Lanosterol 14-alpha demethylase	5.75E-29<br>1.23E-29<br>3.30E-21	0.73<br>0.73<br>0.29	*Homo sapiens*<br>*Mycobacterium tuberculosis*	Lanosterol 14-alpha demethylase<br>Lanosterol 14-alpha demethylase	*Homo sapiens*	Lanosterol 14-alpha demethylase	1.02E-07	*Homo sapiens*<br>*Mycobacterium tuberculosis*	Cytochrome P450 51<br>Lanosterol 14-alpha demethylase	0<br>0	Unknown		Unknown				Unknown	180331151820	Cytochrome P450 51<br>Lanosterol 14-alpha demethylase<br>Sterol 14-alpha demethylase	Drug Metab. Dispos.,(2007)35:3:493<br>Antimicrob. Agents Chemother.,(2009)53:3:1157<br>J. Med. Chem.,(2014)57:15:6704
Isoconazole	CYP51 inhibitor<br>Lanosterol 14 alpha demethylase inhibitor	0.885<br>0.919	0.000<br>0.000	*Homo sapiens*<br>*Mycobacterium tuberculosis*	Lanosterol 14-alpha demethylase<br>Lanosterol 14-alpha demethylase	1.53E-25<br>1.16E-30	0.76<br>0.76	*Homo sapiens*<br>*Mycobacterium tuberculosis*	Lanosterol 14-alpha demethylase<br>Lanosterol 14-alpha demethylase	*Homo sapiens*	Lanosterol 14-alpha demethylase	4.76E-08	*Homo sapiens*<br>*Mycobacterium tuberculosis*	Cytochrome P450 51<br>Lanosterol 14-alpha demethylase	0<br>0	Unknown		Unknown				Unknown	180516120324	Cytochrome P450 51<br>Lanosterol 14-alpha demethylase<br>Sterol 14-alpha demethylase	Drug Metab. Dispos.,(2007)35:3:493<br>Antimicrob. Agents Chemother.,(2009)53:3:1157<br>J. Med. Chem.,(2014)57:15:6704
Ketaminazole	CYP51 inhibitor<br>Lanosterol 14 alpha demethylase inhibitor	0.855<br>0.851	0.001<br>0.001	*Homo sapiens*<br>*Mycobacterium tuberculosis*<br>*Neosartorya fumigate (Aspergillus fumigatus)*<br>*Rattus norvegicus*	Lanosterol 14-alpha demethylase<br>Lanosterol 14-alpha demethylase<br>14-alpha sterol demethylase<br>14-alpha sterol demethylase Cyp51A<br>Lanosterol 14-alpha demethylase	1.59E-32<br>1.92E-06<br>1.22E-15<br>6.48E-18<br>2.09E-81	0.77<br>0.29<br>0.43<br>0.43<br>0.77	*Homo sapiens*<br>*Rattus norvegicus*	Lanosterol 14-alpha demethylase<br>Lanosterol 14-alpha demethylase	*Homo sapiens*	Lanosterol 14-alpha demethylase	5.53E-04	*Homo sapiens*	Cytochrome P450 51	0	Unknown		Cytochrome P450 Family 51 Subfamily A Member 1	89.8	0.68		Unknown	180516120351	Cytochrome P450 51	Antimicrob. Agents Chemother.,(2009)53:3:1157; Bioorg. Med. Chem.,(2008)16:1:209; Drug Metab. Dispos.,(2007)35:3:493; J. Med. Chem.,(1992)35:15:2818; J. Med. Chem.,(1993)36:15:2235; J. Med. Chem.,(2009)52:6:1639
Ketoconazole	CYP51 inhibitor<br>Lanosterol 14 alpha demethylase inhibitor	0.903<br>0.942	0.000<br>0.000	*Homo sapiens*<br>*Mycobacterium tuberculosis*<br>*Neosartorya fumigate (Aspergillus fumigatus)*<br>*Rattus norvegicus*	Lanosterol 14-alpha demethylase<br>Lanosterol 14-alpha demethylase<br>14-alpha sterol demethylase<br>14-alpha sterol demethylase Cyp51A<br>Lanosterol 14-alpha demethylase	1.29E-43<br>2.91E-12<br>1.61E-31<br>2.93E-36<br>2.60E-97	1.00<br>0.3<br>0.56<br>0.56<br>1.00	*Homo sapiens*<br>*Rattus norvegicus*	Lanosterol 14-alpha demethylase<br>Lanosterol 14-alpha demethylase	*Homo sapiens*	Lanosterol 14-alpha demethylase	8.37E-10	*Homo sapiens*	Cytochrome P450 51	0	Unknown		Cytochrome P450 Family 51 Subfamily A Member 1	100	1		Unknown	18047171129	14-alpha sterol demethylase<br>14-alpha sterol demethylase Cyp51A<br>Cytochrome P450 51	Antimicrob. Agents Chemother.,(2010)54:10:4225<br>Antimicrob. Agents Chemother.,(2010)54:10:4225<br>Antimicrob. Agents Chemother.,(2009)53:3:1157; Bioorg. Med. Chem. Lett.,(1994)4:5:733; Bioorg. Med. Chem.,(2008)16:1:209; Drug Metab. Dispos.,(2007)35:3:493; J. Med. Chem.,(1992)35:15:2818; J. Med. Chem.,(1993)36:15:2235; J. Med. Chem.,(2009)52:6:1639
Lanoconazole	CYP51 inhibitor<br>Lanosterol 14 alpha demethylase inhibitor	0.181<br>0.918	0.005<br>0.000	*Mycobacterium tuberculosis*<br>*Neosartorya fumigate (Aspergillus fumigatus)*	Lanosterol 14-alpha demethylase<br>14-alpha sterol demethylase<br>14-alpha sterol demethylase Cyp51A	3.68E-07<br>5.05E-12<br>9.29E-14	0.32<br>0.32<br>0.32		Unknown		Unknown		*Penicillium digitatum*	Cytochrome P450 14alpha-demethylase	0.03	Unknown		Unknown				Unknown	180331151851	Unknown	
Lombazole	CYP51 inhibitor<br>Lanosterol 14 alpha demethylase inhibitor	0.707<br>0.747	0.001<br>0.001	*Homo sapiens*<br>*Mycobacterium tuberculosis*<br>*Neosartorya fumigate (Aspergillus fumigatus)*	Lanosterol 14-alpha demethylase<br>Lanosterol 14-alpha demethylase<br>14-alpha sterol demethylase<br>14-alpha sterol demethylase Cyp51A	1.55E-15<br>6.52E-08<br>2.43E-13<br>2.78E-15	0.71<br>0.36<br>0.36<br>0.36	*Homo sapiens*<br>*Mycobacterium tuberculosis*<br>*Neosartorya fumigata*<br>*Neosartorya fumigata (strain ATCC MYA-4609 / Af293 / CBS 101355 / FGSCA1100) (Aspergillus fumigatus)*	Lanosterol 14-alpha demethylase<br>Lanosterol 14-alpha demethylase<br>14-alpha demethylase<br>14-alpha sterol demethylase Cyp51A	*Homo sapiens*	Lanosterol 14-alpha demethylase	5.46E-03	*Homo sapiens*<br>*Mycobacterium tuberculosis*	Cytochrome P450 51<br>Lanosterol 14-alpha demethylase	0<br>0	Unknown		Cytochrome P450 Family 51 Subfamily A Member 1	94.8	0.71		Unknown	180516142646	14-alpha sterol demethylase<br>14-alpha sterol demethylase Cyp51A<br>Cytochrome P450 51<br>Lanosterol 14-alpha demethylase	Antimicrob. Agents Chemother.,(2010)54:10:4225<br>Antimicrob. Agents Chemother.,(2010)54:10:4225<br>Drug Metab. Dispos.,(2007)35:3:493; J. Med. Chem.,(2002)45:13:2720<br>Antimicrob. Agents Chemother.,(2009)53:3:1157
Luliconazole	CYP51 inhibitor<br>Lanosterol 14 alpha demethylase inhibitor	0.245<br>0.938	0.004<br>0.000		Unknown				Unknown		Unknown			Unknown		Unknown		Unknown				Unknown	180516142737	Unknown	
MH 0685	CYP51 inhibitor<br>Lanosterol 14 alpha demethylase inhibitor	0.129<br>0.781	0.007<br>0.001		Unknown				Unknown		Unknown			Unknown		Unknown		Unknown				Unknown	180516142806	Unknown	
Miconazole	CYP51 inhibitor<br>Lanosterol 14 alpha demethylase inhibitor	0.913<br>0.923	0.000<br>0.000	*Homo sapiens*<br>*Mycobacterium tuberculosis*<br>*Rattus norvegicus*	Lanosterol 14-alpha demethylase<br>Lanosterol 14-alpha demethylase<br>Lanosterol 14-alpha demethylase	1.09E-33<br>3.83E-36<br>5.74E-21	1.00<br>1.00<br>0.28	*Homo sapiens*<br>*Mycobacterium tuberculosis*	Lanosterol 14-alpha demethylase<br>Lanosterol 14-alpha demethylase		Unknown		*Homo sapiens*<br>*Mycobacterium tuberculosis*	Cytochrome P450 51<br>Lanosterol 14-alpha demethylase	0<br>0	Unknown		Unknown				Unknown	180516142832	Cytochrome P450 51<br>Lanosterol 14-alpha demethylase<br>Sterol 14-alpha demethylase	Drug Metab. Dispos.,(2007)35:3:493<br>Antimicrob. Agents Chemother.,(2009)53:3:1157<br>J. Med. Chem.,(2014)57:15:6704
Neticonazole	CYP51 inhibitor<br>Lanosterol 14 alpha demethylase inhibitor	0.111<br>0.715	0.008<br>0.001		Unknown				Unknown		Unknown		*Penicillium digitatum*	Cytochrome P450 14alpha-demethylase	0.01 to 0	Unknown		Unknown				Unknown	180516142902	Unknown	
OK 8705	CYP51 inhibitor<br>Lanosterol 14 alpha demethylase inhibitor	0.360<br>0.310	0.003<br>0.004	*Sorghum bicolor (Sorghum vulgare)*	Obtusifoliol 14-alpha demethylase	3.01E-17	0.41		Unknown		Unknown			Unknown		Unknown		Unknown				Unknown	180516142935	Unknown	
OK 8801	CYP51 inhibitor<br>Lanosterol 14 alpha demethylase inhibitor	0.373<br>0.252	0.003<br>0.004	*Sorghum bicolor (Sorghum vulgare)*	Obtusifoliol 14-alpha demethylase	5.09E-14	0.33		Unknown		Unknown			Unknown		Unknown		Unknown				Unknown	18047171321	Unknown	

Bioactive molecule	PASS online[4]			Similarity Ensemble Approach (SEA)[2]				ChemProt[3]		SuperPred[6]			Polypharmacology Browser (PPB)[1]			SPiDER[7]		HitPick[5]			PharmMapper[9]			TargetHunter[8]	
	Activity	Pa	Pi	Organism	Target	P-Value	Max TC	Organism	Target	Organism	Target	E-value	Organism	Target	P-Value	Target	Confidence level	Target	Precision (%)	Tc similarity	Organism	Target	Job Id	Target	Reference
Omoconazole	CYP51 inhibitor<br>Lanosterol 14 alpha demethylase inhibitor	0.317<br>0.892	0.004<br>0.001	*Homo sapiens*<br>*Mycobacterium tuberculosis*<br>*Rattus norvegicus*	Lanosterol 14-alpha demethylase<br>Lanosterol 14-alpha demethylase<br>Lanosterol 14-alpha demethylase	6.35E-19<br>2.20E-14<br>1.86E-25	0.35<br>0.35<br>0.35		Unknown		Unknown			Unknown		Unknown		Unknown				Unknown	180516143015	Unknown	
Orconazole	CYP51 inhibitor<br>Lanosterol 14 alpha demethylase inhibitor	0.814<br>0.867	0.001<br>0.001	*Homo sapiens*<br>*Mycobacterium tuberculosis*<br>*Neosartorya fumigate (Aspergillus fumigatus)* 	Lanosterol 14-alpha demethylase<br>Lanosterol 14-alpha demethylase<br>14-alpha sterol demethylase<br>14-alpha sterol demethylase Cyp51A	1.30E-24<br>3.66E-28<br>4.21E-12<br>1.38E-10	0.57<br>0.57<br>0.28<br>0.28	*Homo sapiens*<br>*Mycobacterium tuberculosis*	Lanosterol 14-alpha demethylase<br>Lanosterol 14-alpha demethylase		Unknown		*Homo sapiens*<br>*Mycobacterium tuberculosis*	Cytochrome P450 51<br>Lanosterol 14-alpha demethylase	0<br>0	Unknown		Cytochrome P450 Family 51 Subfamily A Member 1	89.8	0.62		Unknown	180516143056	Cytochrome P450 51<br>Lanosterol 14-alpha demethylase<br>Sterol 14-alpha demethylase	Drug Metab. Dispos.,(2007)35:3:493<br>Antimicrob. Agents Chemother.,(2009)53:3:1157<br>J. Med. Chem.,(2014)57:15:6704
Oxiconazole	CYP51 inhibitor<br>Lanosterol 14 alpha demethylase inhibitor	0.542<br>0.925	0.002<br>0.000	*Homo sapiens*<br>*Mycobacterium tuberculosis*<br>*Rattus norvegicus*	Lanosterol 14-alpha demethylase<br>Lanosterol 14-alpha demethylase<br>Lanosterol 14-alpha demethylase	1.02E-21<br>2.82E-19<br>8.29E-22	0.52<br>0.52<br>0.29		Unknown	*Homo sapiens*	Lanosterol 14-alpha demethylase	2.19E-02		Unknown		Unknown		Unknown				Unknown<br>Unknown	180516143138	Unknown	
Oxpoconazole	CYP51 inhibitor<br>Lanosterol 14 alpha demethylase inhibitor	0.194<br>0.187	0.005<br>0.012	*Homo sapiens*<br>*Mycobacterium tuberculosis*<br>*Rattus norvegicus*	Lanosterol 14-alpha demethylase<br>Lanosterol 14-alpha demethylase<br>Lanosterol 14-alpha demethylase	3.60E-13<br>2.31E-06<br>6.23E-45	0.33<br>0.28<br>0.33		Unknown		Unknown			Unknown		Unknown		Unknown				Unknown	180331151926	Unknown	
Parconazole	CYP51 inhibitor<br>Lanosterol 14 alpha demethylase inhibitor	0.921<br>0.745	0.000<br>0.001	*Homo sapiens*<br>*Mycobacterium tuberculosis*<br>*Neosartorya fumigate (Aspergillus fumigatus)*<br><br>*Rattus norvegicus*	Lanosterol 14-alpha demethylase<br>Lanosterol 14-alpha demethylase<br>14-alpha sterol demethylase<br>14-alpha sterol demethylase Cyp51A<br>Lanosterol 14-alpha demethylase	4.08E-35<br>3.66E-14<br>3.18E-14<br>2.22E-16<br>1.09E-71	0.61<br>0.35<br>0.39<br>0.39<br>0.61		Unknown	*Homo sapiens*	Lanosterol 14-alpha demethylase	3.44E-02	*Homo sapiens*<br>*Mycobacterium tuberculosis*	Cytochrome P450 51<br>Lanosterol 14-alpha demethylase	0.01 to 0<br>0.03	Unknown		Cytochrome P450 Family 51 Subfamily A Member 1	77	0.55		Unknown	180423175320	Cytochrome P450 51<br>Lanosterol 14-alpha demethylase	Antimicrob. Agents Chemother.,(2009)53:3:1157; Bioorg. Med. Chem.,(2008)16:1:209; Drug Metab. Dispos.,(2007)35:3:493; J. Med. Chem.,(1992)35:15:2818; J. Med. Chem.,(1993)36:15:2235; J. Med. Chem.,(2009)52:6:1639<br>Antimicrob. Agents Chemother.,(2009)53:3:1157
Pefurazoate	CYP51 inhibitor	0.052	0.024		Unknown				Unknown		Unknown			Unknown		Unknown		Unknown				Unknown	180331152005	Unknown	
PR 967-234	CYP51 inhibitor<br>Lanosterol 14 alpha demethylase inhibitor	0.296<br>0.563	0.004<br>0.002	*Homo sapiens*<br>*Mycobacterium tuberculosis*<br>*Rattus norvegicus*	Lanosterol 14-alpha demethylase<br>Lanosterol 14-alpha demethylase<br>Lanosterol 14-alpha demethylase	9.10E-15<br>1.07E-06<br>5.42E-52	0.38<br>0.30<br>0.38		Unknown		Unknown		*Homo sapiens*<br>*Mycobacterium tuberculosis*	Cytochrome P450 51<br>Lanosterol 14-alpha demethylase	0.01 to 0<br>0.01 to 0	Unknown		Unknown						Unknown	
Prochloraz	CYP51 inhibitor<br>Lanosterol 14 alpha demethylase inhibitor	0.195<br>0.199	0.005<br>0.009	*Mycobacterium tuberculosis*	Lanosterol 14-alpha demethylase	1.07E-06	0.30	*Homo sapiens*	Lanosterol 14-alpha demethylase		Unknown			Unknown		Unknown		Unknown				Unknown	180331152030	Unknown	
R 31000	CYP51 inhibitor<br>Lanosterol 14 alpha demethylase inhibitor	0.928<br>0.944	0.000<br>0.000	*Homo sapiens*<br>*Mycobacterium tuberculosis*<br>*Neosartorya fumigate (Aspergillus fumigatus)*<br><br>*Rattus norvegicus*	Lanosterol 14-alpha demethylase<br>Lanosterol 14-alpha demethylase<br>14-alpha sterol demethylase<br>14-alpha sterol demethylase Cyp51A<br>Lanosterol 14-alpha demethylase	8.44E-39<br>8.37E-14<br>4.53E-17<br>1.39E-19<br>2.31E-79	0.72<br>0.34<br>0.47<br>0.47<br>0.72		Unknown	*Homo sapiens*	Lanosterol 14-alpha demethylase	2.88E-03	*Homo sapiens*	Cytochrome P450 51	0	Unknown		Cytochrome P450 Family 51 Subfamily A Member 1	94.8	0.72		Unknown	180516143225	14-alpha sterol demethylase<br>14-alpha sterol demethylase Cyp51A<br>Cytochrome P450 51<br>Lanosterol 14-alpha demethylase	Antimicrob. Agents Chemother.,(2010)54:10:4225<br>Antimicrob. Agents Chemother.,(2010)54:10:4225<br>Antimicrob. Agents Chemother.,(2009)53:3:1157; Bioorg. Med. Chem. Lett.,(1994)4:5:733; Drug Metab. Dispos.,(2007)35:3:493; J. Med. Chem.,(1992)35:15:2818; J. Med. Chem.,(1993)36:15:2235; J. Med. Chem.,(2009)52:6:1639<br>Antimicrob. Agents Chemother.,(2009)53:3:1157
Sertaconazole	CYP51 inhibitor<br>Lanosterol 14 alpha demethylase inhibitor	0.774<br>0.951	0.001<br>0.000	*Homo sapiens*<br>*Mycobacterium tuberculosis*	Lanosterol 14-alpha demethylase<br>Lanosterol 14-alpha demethylase	7.49E-19<br>9.92E-27	0.68<br>0.68	*Homo sapiens*<br>*Mycobacterium tuberculosis*	Lanosterol 14-alpha demethylase<br>Lanosterol 14-alpha demethylase	*Homo sapiens*	Lanosterol 14-alpha demethylase	6.86E-07	*Homo sapiens*<br>*Mycobacterium tuberculosis*	Cytochrome P450 51<br>Lanosterol 14-alpha demethylase	0<br>0	Unknown		Cytochrome P450 Family 51 Subfamily A Member 1	100	1		Unknown	180516150014	Cytochrome P450 51<br>Lanosterol 14-alpha demethylase	Drug Metab. Dispos.,(2007)35:3:493<br>Antimicrob. Agents Chemother.,(2009)53:3:1157
SM 4470	CYP51 inhibitor<br>Lanosterol 14 alpha demethylase inhibitor	0.378<br>0.922	0.003<br>0.000	*Homo sapiens*<br>*Mycobacterium tuberculosis*	Lanosterol 14-alpha demethylase<br>Lanosterol 14-alpha demethylase	4.35E-10<br>3.59E-14	0.34<br>0.34		Unknown		Unknown		*Homo sapiens*<br>*Mycobacterium tuberculosis*	Cytochrome P450 51<br>Lanosterol 14-alpha demethylase	0.01 to 0<br>0.015	Unknown		Unknown				Unknown	180516150045	Cytochrome P450 51<br>Lanosterol 14-alpha demethylase	Drug Metab. Dispos.,(2007)35:3:493<br>Antimicrob. Agents Chemother.,(2009)53:3:1157
SSF 105	CYP51 inhibitor<br>Lanosterol 14 alpha demethylase inhibitor	0.478<br>0.875	0.002<br>0.001	*Homo sapiens*<br>*Mycobacterium tuberculosis*	Lanosterol 14-alpha demethylase<br>Lanosterol 14-alpha demethylase	3.86E-11<br>1.11E-15	0.42<br>0.42		Unknown		Unknown		*Homo sapiens*<br>*Mycobacterium tuberculosis*	Cytochrome P450 51<br>Lanosterol 14-alpha demethylase	0<br>0.01 to 0	Unknown		Unknown				Unknown	180516150125	Unknown	
Sulconazole	CYP51 inhibitor<br>Lanosterol 14 alpha demethylase inhibitor	0.479<br>0.924	0.002<br>0.000	*Homo sapiens*<br>*Mycobacterium tuberculosis*<br>*Rattus norvegicus*	Lanosterol 14-alpha demethylase<br>Lanosterol 14-alpha demethylase<br>Lanosterol 14-alpha demethylase	6.13E-29<br>2.15E-23<br>5.94E-44	0.62<br>0.62<br>0.31		Unknown	*Homo sapiens*	Lanosterol 14-alpha demethylase	1.16E-07		Unknown		Unknown		Cytochrome P450 Family 51 Subfamily A Member 1	97.3	1		Unknown	180516150150	Unknown	
Tioconazole	CYP51 inhibitor<br>Lanosterol 14 alpha demethylase inhibitor	0.791<br>0.960	0.001<br>0.000	*Homo sapiens*<br>*Mycobacterium tuberculosis*<br>*Rattus norvegicus*	Lanosterol 14-alpha demethylase<br>Lanosterol 14-alpha demethylase<br>Lanosterol 14-alpha demethylase	3.23E-25<br>2.70E-30<br>7.28E-21	0.78<br>0.78<br>0.28	*Homo sapiens*<br>*Mycobacterium tuberculosis*	Lanosterol 14-alpha demethylase<br>Lanosterol 14-alpha demethylase	*Homo sapiens*	Lanosterol 14-alpha demethylase	2.90E-08	*Homo sapiens*<br>*Mycobacterium tuberculosis*	Cytochrome P450 51<br>Lanosterol 14-alpha demethylase	0<br>0	Unknown		Cytochrome P450 Family 51 Subfamily A Member 1	100	1		Unknown	180516150239	Cytochrome P450 51<br>Lanosterol 14-alpha demethylase	Drug Metab. Dispos.,(2007)35:3:493<br>Antimicrob. Agents Chemother.,(2009)53:3:1157
Triflumizole	CYP51 inhibitor<br>Lanosterol 14 alpha demethylase inhibitor	0.185<br>0.335	0.005<br>0.003	*Sorghum bicolor (Sorghum vulgare)*	Obtusifoliol 14-alpha demethylase	2.41E-13	0.31		Unknown		Unknown			Unknown		Unknown		Unknown				Unknown	180331152052	Unknown	
UK 38667	CYP51 inhibitor<br>Lanosterol 14 alpha demethylase inhibitor	0.462<br>0.772	0.003<br>0.001	*Homo sapiens*<br>*Mycobacterium tuberculosis*<br>*Neosartorya fumigate (Aspergillus fumigatus)*<br><br>*Rattus norvegicus*	Lanosterol 14-alpha demethylase<br>Lanosterol 14-alpha demethylase<br>14-alpha sterol demethylase<br>14-alpha sterol demethylase Cyp51A<br>Lanosterol 14-alpha demethylase	2.29E-19<br>9.36E-28<br>1.49E-21<br>4.21E-12<br>2.59E-22	0.38<br>0.38<br>0.32<br>0.28<br>0.30		Unknown		Unknown		*Homo sapiens*<br>*Mycobacterium tuberculosis*	Cytochrome P450 51<br>Lanosterol 14-alpha demethylase	0.01 to 0<br>0.01 to 0	Unknown		Unknown				Unknown	180516150311	Cytochrome P450 51<br>Lanosterol 14-alpha demethylase	Drug Metab. Dispos.,(2007)35:3:493; J. Med. Chem.,(2002)45:13:2720<br>Antimicrob. Agents Chemother.,(2009)53:3:1157
Valconazole	CYP51 inhibitor<br>Lanosterol 14 alpha demethylase inhibitor	0.405<br>0.385	0.003<br>0.003	*Homo sapiens*<br>*Mycobacterium tuberculosis*<br>*Penicillium digitatum*<br>*Rattus norvegicus*	Lanosterol 14-alpha demethylase<br>Lanosterol 14-alpha demethylase<br>Cytochrome P450 14alpha-demethylase<br>Lanosterol 14-alpha demethylase	1.29E-21<br>2.66E-18<br>1.64E-11<br>2.51E-24	0.44<br>0.44<br>0.37<br>0.33		Unknown		Unknown		*Penicillium digitatum*	Cytochrome P450 14alpha-demethylase	0.01 to 0	Unknown		Unknown				Unknown	180516150334	Unknown	
Zinoconazole	CYP51 inhibitor<br>Lanosterol 14 alpha demethylase inhibitor	0.228<br>0.928	0.005<br>0.000	*Homo sapiens*<br>*Mycobacterium tuberculosis*<br>*Neosartorya fumigate (Aspergillus fumigatus)* 	Lanosterol 14-alpha demethylase<br>Lanosterol 14-alpha demethylase<br>14-alpha sterol demethylase<br>14-alpha sterol demethylase Cyp51A	1.52E-08<br>2.14E-06<br>1.11E-10<br>3.31E-12	0.28<br>0.28<br>0.28<br>0.28		Unknown		Unknown			Unknown		Unknown		Unknown				Unknown	18047171430	Unknown	
Zoficonazole	CYP51 inhibitor<br>DELTA14-sterol reductase inhibitor<br>Lanosterol 14 alpha demethylase inhibitor	0.723<br>0.869<br>0.871	0.001<br>0.002<br>0.001	*Homo sapiens*<br>*Mycobacterium tuberculosis*<br>*Rattus norvegicus*	Lanosterol 14-alpha demethylase<br>Lanosterol 14-alpha demethylase<br>Lanosterol 14-alpha demethylase	2.75E-28<br>1.51E-27<br>1.86E-25	0.69<br>0.69<br>0.35		Unknown	*Homo sapiens*	Lanosterol 14-alpha demethylase	2.82E-07	*Homo sapiens*<br>*Mycobacterium tuberculosis*	Cytochrome P450 51<br>Lanosterol 14-alpha demethylase	0<br>0	Unknown		Cytochrome P450 Family 51 Subfamily A Member 1	77	0.57		Unknown	180516150359	Cytochrome P450 51<br>Lanosterol 14-alpha demethylase	Antimicrob. Agents Chemother.,(2009)53:3:1157; Bioorg. Med. Chem.,(2008)16:1:209; Drug Metab. Dispos.,(2007)35:3:493; J. Med. Chem.,(1992)35:15:2818; J. Med. Chem.,(1993)36:15:2235; J. Med. Chem.,(2009)52:6:1639<br>Antimicrob. Agents Chemother.,(2009)53:3:1157

References

1. Awale, M. & Reymond, J. L. The polypharmacology browser: a web-based multi-fingerprint target prediction tool using ChEMBL bioactivity data. *J. cheminform.* **9**, 11 (2017).
2. Keiser, M. J. *et al.* Relating protein pharmacology by ligand chemistry. *Nat. Biotechnol.* **25**, 197–206 (2007).
3. Kringelum, J. *et al.* ChemProt-3.0: a global chemical biology diseases mapping. *Database* **2016**, 1–7 (2016).
4. Lagunin, A., Stepanchikova, A., Filimonov, D. & Poroikov, V. PASS: prediction of activity spectra for biologically active substances. *Bioinformatics* **16**, 747–748 (2000).
5. Liu, X., Vogt, I., Haque, T. & Campillos, M. HitPick: a web server for hit identification and target prediction of chemical screenings. *Bioinformatics* **29**, 1910–1912 (2013).
6. Nickel, J. *et al.* SuperPred: update on drug classification and target prediction. *Nucleic Acids Res.* **42**, W26–W31 (2014).
7. Reker, D., Rodrigues, T., Schneider, P. & Schneider, G. Identifying the macromolecular targets of de novo-designed chemical entities through self-organizing map consensus. *Proc. Natl. Acad. Sci. USA* **111**, 4067–4072 (2014).
8. Wang, L. *et al.* TargetHunter: an *in silico* target identification tool for predicting therapeutic potential of small organic molecules based on chemogenomic database. *AAPS J.* **15**, 395–406 (2013).
9. Wang, X. *et al.* PharmMapper 2017 update: a web server for potential drug target identification with a comprehensive target pharmacophore database. *Nucleic Acids Res.* **45**, W356–W360 (2017).
